# The Velvet Family of Fungal Regulators Contains a DNA-Binding Domain Structurally Similar to NF-κB

**DOI:** 10.1371/journal.pbio.1001750

**Published:** 2013-12-31

**Authors:** Yasar Luqman Ahmed, Jennifer Gerke, Hee-Soo Park, Özgür Bayram, Piotr Neumann, Min Ni, Achim Dickmanns, Sun Chang Kim, Jae-Hyuk Yu, Gerhard H. Braus, Ralf Ficner

**Affiliations:** 1 Department of Molecular Structural Biology, Institute of Microbiology and Genetics, Georg-August University, Göttingen, Germany; 2 Department of Molecular Microbiology and Genetics, Institute of Microbiology and Genetics, Georg-August University, Göttingen, Germany; 3 Departments of Bacteriology and Genetics, University of Wisconsin–Madison, Madison, Wisconsin, United States of America; 4 Department of Biological Sciences, Korea Advanced Institute of Science and Technology, Dae-Jon, Republic of Korea; Rutgers University, United States of America

## Abstract

Morphological development of fungi and their combined production of secondary metabolites are both acting in defence and protection. These processes are mainly coordinated by *velvet* regulators, which contain a yet functionally and structurally uncharacterized *velvet* domain. Here we demonstrate that the *velvet* domain of VosA is a novel DNA-binding motif that specifically recognizes an 11-nucleotide consensus sequence consisting of two motifs in the promoters of key developmental regulatory genes. The crystal structure analysis of the VosA *velvet* domain revealed an unforeseen structural similarity with the Rel homology domain (RHD) of the mammalian transcription factor NF-κB. Based on this structural similarity several conserved amino acid residues present in all *velvet* domains have been identified and shown to be essential for the DNA binding ability of VosA. The *velvet* domain is also involved in dimer formation as seen in the solved crystal structures of the VosA homodimer and the VosA-VelB heterodimer. These findings suggest that defence mechanisms of both fungi and animals might be governed by structurally related DNA-binding transcription factors.

## Abstract 2

This study reveals an important family of fungal regulatory proteins to be transcription factors that contain a DNA-binding “velvet” domain structurally related to that of mammalian NFkB.

## Author Summary

In many fungi, developmental processes and the synthesis of nonessential chemicals (secondary metabolites) are regulated by various external stimuli, such as light. Although fungi employ them for defensive purposes, secondary metabolites range from useful antibiotics to powerful toxins, so understanding the molecular processes that regulate their synthesis is of particular interest to us. In the mold *Aspergillus nidulans* the main regulators of these processes are the so-called “*velvet*” proteins VeA, VelB, and VosA, which share a 150-amino acid region known as the *velvet* domain. *Velvet* proteins interact with each other, alone (“homodimers”), in various combinations (“heterodimers”), and also with other proteins, but the molecular mechanism by which these proteins exert their regulatory function has been unclear. In this work we show that *velvet* proteins form a family of fungus-specific transcription factors that directly bind to target DNA, even though analysis of their amino acid sequence does not reveal any known DNA-binding domains or motifs. We determined the three-dimensional structure of the VosA-VosA homodimer and the VosA-VelB heterodimer and found that the structure of the *velvet* domain is strongly reminiscent of the N-terminal immunoglobulin-like domain found in the mammalian transcription factor NFκB-p50, despite the very low sequence similarity. We propose that, like NFκB, various homo- or heterodimers of *velvet* proteins modulate gene expression to drive development and defensive pathways in fungi.

## Introduction

The fungal and the animal kingdom are related as they both belong to the ophistokonts with a common ancestor existing about 1 billion years ago [1],[2]. Animals have evolved with an elaborate inflammation and immune system for self-defence. Inflammation, the immune system, and animal development are controlled by various mono- and multiprotein assemblies of RHD-containing proteins. Among others, one family, named NF-κB, consists of five members, which respond to external stimuli [3],[4]. In contrast to animals, fungi are normally secured by a thick cell wall and had been misclassified as plants for centuries due to their loss of motility and the establishment of a cell wall. In addition, in response to various abiotic or biotic signals, filamentous fungi produce small signalling and/or defensive bioactive molecules [5],[6]. These secondary metabolites range from antibiotics such as penicillins to mycotoxins such as aflatoxins, affecting everyday life of animals and human beings [7]. Regulation of the secondary metabolism as well as the control of growth and differentiation of the model mold *Aspergillus nidulans* are coupled by a family of fungal regulators, the *velvet* proteins (Figure S1) [6],[8]. These *velvet* regulators are present in most parts of the fungal kingdom from chytrids to basidiomycetes. The *velvet* proteins share a homologous region comprising about 150 amino acids, which lack significant sequence homology to any other known proteins (Figure S2).

In *A. nidulans* the four *velvet* proteins VeA, VelB, VelC, and VosA have been identified and characterized. They can interact with each other and also with non-*velvet* proteins resulting in complexes, which link morphological and chemical development of fungi [9]. The regulation of sexual development and secondary metabolism has been shown to be a light-regulated process coordinated by the heterotrimeric complex, consisting of the *velvet* proteins VeA, the VeA-like protein B (VelB), and the putative methyltransferase LaeA. The heterotrimeric VelB/VeA/LaeA-complex activates secondary metabolism and sexual development. The Δ*laeA* and Δ*veA* mutant strains are unable to produce hardly any sterigmatocystin, the penultimate precursor of aflatoxins. Similarly Δ*veA* and Δ*velB* strains do not form any sexual fruiting body [9]. Notably, in the dark VeA is predominantly found in the nucleus, whereas it is mostly in the cytoplasm in the light. VeA contains an N-terminally located nuclear localisation signal (NLS) recognized by the nuclear import factor KapA mediating the transport from the cytoplasm into the nucleus [10], once it becomes accessible by a yet unknown factor or mechanism.

VosA contains an N-terminally located *velvet* domain and is required for the transcription of several genes essential for spore viability [11]. Deletion of *vosA* results in a severe down-regulation of genes associated with trehalose biosynthesis (*tpsA*, *tpsC*, and *orlA*) and the lack of trehalose biogenesis in spores. As a consequence, spores of Δ*vosA* strains are much less resistant to heat, UV, and other stress conditions and exhibit a strongly reduced survival rate after 10 d. Studies of spore viability of the Δ*velB* mutant revealed that the interaction of VosA with VelB is required for proper expression of the trehalose biosynthesis genes in fungal spores [12],[13]. Similar to the Δ*vosA* strain, the Δ*velB* strain produces spores that contain virtually no trehalose, rendering them much more susceptible to desiccation and other stresses.

The role of *velvet* proteins in other fungi has been extensively studied in the past few years. While external stimuli can be different, their regulatory function on secondary metabolism and development seems to be conserved. In the human pathogen *Histoplasma capsulatum*, the switch from filamentous growth to the pathogenic yeast form is triggered by a temperature increase and requires the VosA and VelB orthologues Ryp2 and Ryp3, respectively [14],[15]. In *Fusarium fujikuroi*, the deletion of the *veA* and *velB* homologues *Ffvel1* and *Ffvel2* affects the secondary metabolism and virulence on rice [16]. In several cases the *veA* null mutation could be rescued by complementation of cross-genus *veA* from other fungi [16]–[18]. Numerous recent studies support a role of *velvet* proteins in fungal virulence [19]–[22].

Here we report the molecular basis of the *velvet*-mediated gene regulation. Genome-wide and targeted DNA binding studies of VosA reveal that its *velvet* domain recognizes an 11-nucleotide sequence present in the promoter regions of many regulatory and structural genes. The crystal structure analysis of the *velvet* domains of VosA and the heterodimeric VosA-VelB complex demonstrate that the *velvet* domain is an RHD-like domain related to NF-κB. Besides the *velvet* domain, VosA contains a predicted C-terminal transcriptional activation domain, implying that it is likely a transcription factor [11]. Taken together, the existence of novel fungus-specific transcription factors possessing a mammalian NF-κB–like DNA-binding domain suggests a common functional origin for the coordination of fungal development with secondary metabolism and the immuno-inflammatory response control in humans.

## Results

### VosA Is a DNA-Binding Protein

**Figure 1:**
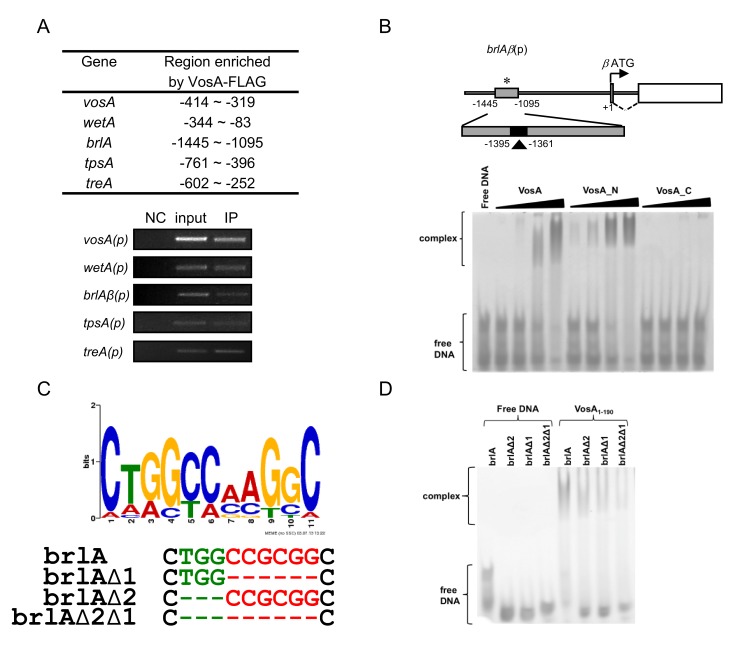
VosA binds DNA specifically. (A) Selected regions enriched by VosA-FLAG ChIP on chip. The promoter regions of *brlA*, *wetA*, *vosA*, *tpsA*, and *treA* enriched by VosA-FLAG ChIP are shown (the start codon ATG is +1). Results of VosA-ChIP-PCR, the PCR amplicons separated on a 2% agarose gel, are shown in the bottom panel. The input DNA before immuno-precipitation (IP) was used as a positive control (input). The chromatin extract being incubated with bead only (without anti-FLAG antibody) was used as a negative control (NC). (B) Schematic presentation of the promoter region of *brlA*. The gray box (−1,095∼1,445 region of *brlA*(p), marked by *) represents the ChIP-PCR amplified region shown in (A), and the filled box (marked by arrowhead) represents a 35 bp fragment used in EMSA. EMSA using serially diluted VosA proteins and the 35 bp DNA probe of the *brlA* promoter (OHS301/302). DNA and protein were used in the molar ratios 1∶0.3, 1∶1, 1∶3, and 1∶9. VosA, full-length VosA; VosA_N, truncated VosA (residues 1–216); VosA_C, truncated VosA (residues 217–430). (C) The consensus DNA sequence predicted to be recognized by VosA is shown. Two cores of this motif were deleted as shown (green, red) and these probes were used for additional EMSA. (D) EMSA using the crystallized VosA_1–190_ (VosA_cryst) with wild-type and mutated DNA probes of the *brlA* promoter. In the mutated versions of the DNA, the predicted VosA binding motifs 1 and 2 were deleted (brlA, wild-type DNA (OHS301/302); brlAΔ2, DNA with deleted core 2 (JG636/637); brlAΔ1, DNA with deleted core 1 (JG638/639); brlAΔ2Δ1, DNA with deleted cores 2 and 1 (JG640/641)).

Due to their regulatory roles and nuclear localization, a function as transcription factor was proposed for *velvet* proteins. However, based on their amino acid sequences, no known DNA-binding domain could be identified. To test for a potential DNA binding activity of *velvet* proteins, *in vivo* chromatin immuno-precipitation (ChIP) employing the VosA protein tagged with FLAG followed by *A. nidulans* tiling microarray analysis (ChIP-chip) was carried out. The results revealed that more than 1,500 genes' promoters were enriched by the VosA-FLAG-ChIP (Table S1). For verification of these results, we further carried out ChIP-PCR and demonstrated that VosA-FLAG-ChIP indeed specifically enriched the promoter regions of the genes associated with asexual development (*brlA*, *wetA*, and *vosA*) and trehalose biosynthesis (*tpsA* and *treA*) (Figure 1A; Figure S3). Using VosA-ChIP-chip result, we then performed the consensus motif analysis, which led to several motifs (Table S2). Independent of this computer-based analysis, we carried out a series of electrophoretic mobility shift assays (EMSAs) with full-length or truncated VosA proteins (VosA, VosA_N, VosA_C) using various regions of the *brlA* promoter as probes. Such a promoter-walking EMSA revealed that full-length and the N-terminal half of VosA (VosA_N, residues 1–216) containing the entire *velvet* homology region binds to a 35 bp fragment of the *brlA*β promoter (−1,395 to −1,361, marked by the arrowhead in Figure 1B). To develop a preliminary motif recognized by VosA, we used 14 sequences from ChIP-chip and Northern blot results (Table S3) and one 35 bp *brlA*β fragment from EMSA. These 15 sequences were subject to MEME (Multiple Em for Motif Elicitation) analysis [23] and an 11-nucleotide consensus sequence was found (Figure 1C). This motif (positions 5 through 10) is similar to motif 1 from ChIP-chip results (Table S2) and has a very good palindrome structure (CCGCGG). In addition, the TGG sequence (positions 2 to 4 in a preliminary motif sequence) is included in three motifs from ChIP-chip results. To test whether these sequences (CCGCGG and TGG) in the 35 bp *brlA*β fragment are core sequences that are needed for DNA-binding, we designed three probes that specifically deleted these regions (Figure 1C). VosA-DNA binding was decreased when the mutated probes were used (Figure 1D). These results suggested that the VosA *velvet* domain represents a novel type of DNA-binding domain that recognizes an 11-nucleotide DNA sequence.

**Figure 2:**
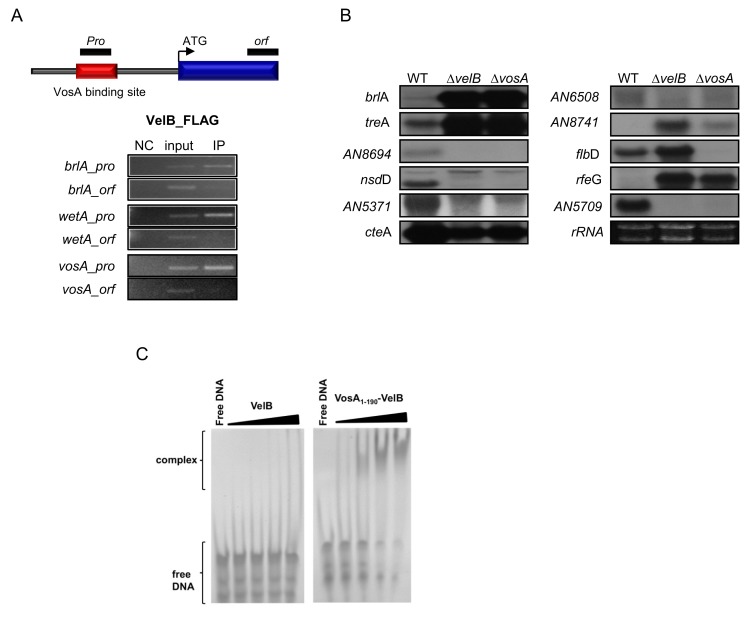
Regulatory roles of VelB and VosA_1–190_-VelB complex. (A) Schematic presentation of the promoter region of a typical *vosA* target gene. The red box represents the region, which was enriched by VosA-ChIP (upper). Black boxes represent two ChIP-PCR target regions shown at the bottom. Results of VelB-ChIP-PCR, the PCR amplicons separated on a 2% agarose gel, are shown (bottom). The input DNA before immunoprecipitation (IP) was used as a positive control (input). The chromatin extract being incubated with bead only (without anti-FLAG antibody) was used as a negative control (NC). (B) mRNA levels of the genes that are predicted to be under the direct regulatory control of VosA in the conidia of wt (wild-type; FGSC4), Δ*velB* (THS16.1), and Δ*vosA* (THS15.1) strains. Equal loading of total RNA was confirmed by ethidium bromide staining of rRNA. (C) EMSA using the VosA_1–190_-VelB heterodimer and VelB with the 35 bp DNA probe of the *brlA* promoter (OHS301/302). DNA and protein were used in the molar ratios 1∶0.3, 1∶1, 1∶3, and 1∶9.

Previous studies proposed that the VelB-VosA heterodimer is a functional unit of trehalose biosynthesis in spores and of spore maturation [12],[13]. To test whether VelB-ChIP also enriches the promoter regions of the VosA target genes, a VelB-ChIP-PCR analysis was performed. VelB-FLAG-ChIP enriched the same promoter, but not ORF-regions of VosA target genes (Figures 2A and S3). To test the molecular consequences of the lack of VosA or VelB in spores, we then examined the mRNA levels of the high score genes in wild-type, Δ*vosA*, and Δ*velB* strain conidia (Table S3). Deletion of *velB*, similar to deletion of *vosA*, caused reduced accumulation of AN8694, *nsdD*, AN5371, *cteA*, AN6508, and AN5709 mRNAs, but increased transcript levels of *brlA*, *treA* AN8741, and *rfeG* (Figure 2B). These results suggest that these two *velvet* regulators play a dual role in activating genes associated with spore maturation and repressing certain development-associated genes. As mentioned above, the *velvet* domain of VosA is a DNA-binding domain. To test the DNA binding ability of the *velvet* domains of other *velvet* proteins, we carried out further EMSAs using VeA, VelB, or a heterodimer composed of VelB and the minimal *velvet* domain of VosA encompassing amino acid residues 1–190 (VosA_1–190_), which was designed for crystallization (see below). The VelB protein alone failed to bind to the *brlA* probe, whereas VosA_1–190_-VelB and VeA bind to this probe readily (Figures 2C and S4). In addition, VosA_1–190_-VelB and VeA binding to DNA was decreased when the mutated probes of the *brlA*-promoter region were used (Figure S5). Overall, these results imply that the homodimers of VosA and VeA may recognize the same promoter regions, while VelB alone at least does not bind the *brlA* promoter. However, binding sequences and affinities might be different for heterodimers.

### Crystal Structure of the Velvet Domain Reveals a NF-κB–Like Fold

In order to gain insights into the structural basis of the specific DNA recognition and to increase the chances for crystallization, a minimal *velvet* domain of VosA encompassing residues 1–190 (VosA_1–190_) was cloned, expressed, and purified. The DNA binding properties—albeit weaker than for the VosA_N—indicated a properly folded and active entity (Figure 1B), which crystallized readily forming well-diffracting tetragonal crystals. The crystal structure of VosA_1–190_ was determined *de novo* by means of single-wavelength anomalous dispersion (SAD). The structure was refined at a resolution of 1.79 Å (Table 1) and comprises residues 8–185 belonging to one VosA monomer occupying the asymmetric unit. Crystal packing analysis revealed the existence of a homodimer defined by a crystallographic 2-fold symmetry axis, which is consistent with the results from size exclusion chromatography and multi-angle-light-scattering (MALS), demonstrating that VosA_1–190_ exists in solution as a homodimer (Figures S6 and S7).

**Table 1:** X-ray diffraction data and crystal structure refinement statistics.

Data/Statistics	VosA_1190_	VelB-VosA_1–190_ Complex
*Data collection*		
Beamline	Rigaku MicroMax 007	ESRF ID 23-2
Wavelength, Å	1.5418	0.87260
Space group	P4_1_22	P2_1_2_1_2_1_
Resolution range, Å	44.15–1.79 (1.90–1.79)	36.95–2.18 (2.30–2.20)
*Cell dimensions*		
a, b, c Å	45.35, 45.35, 189.32	52.03, 56.75, 138.17
α, β, γ, °	90.0, 90.0, 90.0	90.0, 90.0, 90.0
R_merge_, %	3.6 (24.7)	12.6 (48.3)
I/σ	22.17 (5.01)	8.1 (2.9)
Reflections total/unique	142,606/35,879 (19,836/5,553)	99,691/22,156 (14,610/3,252)
Completeness %	99.1 (95.4)	97.0 (98.3)
Redundancy	4.01 (3.57)	4.5 (4.5)
*Phasing*		
No. of heavy atoms	3	
FOM (initial)	0.7	
*Refinement*		
Resolution (Å)	44.15–1.79	36.95–2.20
R_work_ %	20.02 (26.62)	19.00 (22.02)
R_free_ %	24.04 (32.89)	24.56 (28.44)
*No. of atoms*		
Protein	1,412	2,782
Water	193	251
Ions	11	5
*B-factors, Å^2^*		
Protein	25.964	25.362
Water	33.99	30.93
Ions	31.58	20.30
*RMS deviations*		
Bonds (Å)	0.007	0.003
Angles (°)	1.133	0.737
*Ramachandran plot*		
Most favoured, %	93.5	96.2
Disallowed, %	0.0	0.0

Values in parentheses refer to the highest resolution shell. Ramachandran statistics were calculated with MOLPROBITY [56].

**Figure 3:**
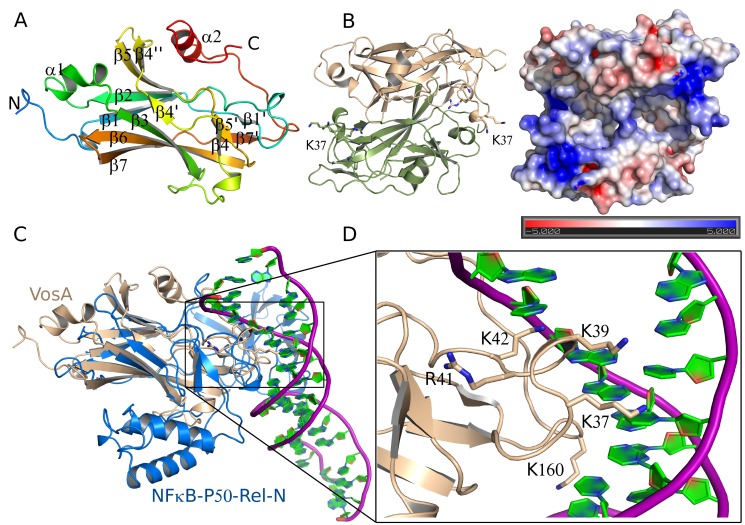
Crystal structure of VosA_1–190_. (A) Structure of the *velvet* domain of VosA. Amino acid residues 1–185 of VosA_1–190_ from *A. nidulans* fold into a seven-stranded β-sandwich and two α-helices. (B) Structure of the VosA homodimer. The two monomers are related by a crystallographic 2-fold symmetry axis. Loop A is indicated by the labelling of K37. The electrostatic surface potential of the VosA_1–190_ homodimer indicates the binding surface for DNA. Surface representation coloured according to the electrostatic potential ranging from red (−5 kBT/e) through white (0 kBT/e) to blue (+5 kBT/e). (C) Superposition of the VosA *velvet* domain and the Rel-N domain of NF-κB bound to DNA [25]. (D) Lys and Arg residues of VosA_1–190_ that are predicted to be involved in DNA binding as deduced by the superposition VosA_1–190_ onto the NF-κB-DNA complex structure (loop B is indicated by the labelling of K160).

The VosA *velvet*-domain folds into a highly twisted β-sandwich containing seven antiparallel β-strands. One side of the β-sandwich is involved in dimer formation, whereas the other one is flanked by several loops of which two fold into an α-helix. These α-helical fragments are located between β-strands 2 and 3 and at the C-terminus (Figure 3A). The 2-fold symmetry of a homodimer results in an antiparallel orientation of β-strands 2, 3, 5, and 7 to the same strands of the other subunit (Figure 3B). The surface area covered by the interaction of the subunits is 1,078 Å^2^ corresponding to 12.7% of the total molecule surface sufficient for a stable interaction required for dimer formation. Both subunits contribute to a positively charged patch on the homodimer's surface likely to be involved in binding and recognition of DNA (Figure 3B).

A search for proteins structurally homologous to VosA using DALI [24] identified the mammalian transcription factor NF-κB-p50 [25],[26] as the most similar protein structure with a root mean square deviation (r.m.s.d.) of 2.8 Å for 113 common C_α_-atoms (Figure 3C). Given the low amino acid sequence identity of 13.7%, this structural similarity was quite unexpected (Figure S8). NF-κB itself belongs to the family of Rel-proteins containing the conserved Rel-homology-region, which encompasses 300 residues that fold into two immunoglobulin-like domains [25],. Both of these domains are involved in DNA-binding, but dimerization of Rel-proteins is exclusively mediated by the C-terminally localized, shorter domain (Rel-C). Opposing, the fold of the VosA *velvet* domain resembles that of the longer N-terminal domain of NF-κB-p50 (Rel-N). The major difference between p50-Rel-N and the *velvet* domain is an insertion of three additional α-helices between β-strands 7 and 8 of p50-Rel-N (Figures 3C and S8). A C-terminal helix that covers the β-sheet in the region formed by β1, β2, β5, and β4 of VosA is missing in p50-Rel-N. Instead p50-Rel-C interacts with a long loop connecting β1 and β2, extending the protein on that side.

The crystallized VosA fragment comprises the 190 N-terminal residues corresponding to the *velvet* domain. An amino acid sequence analysis of the missing C-terminal part of VosA performed with HHpred [27] revealed a homology to the Sec24 transport protein for a fragment comprising residues 240 to 434 of VosA (sequence identity and similarity of 23% and 35%, respectively). This C-terminally located domain is connected via a 50-residue-long region without any detectable structural homology, which nevertheless could correspond to the C-terminally located Rel-C domain of NF-κB-p50. Superposition of VosA and NF-κB-p50 structures revealed that the flexible loop connecting two domains in NF-κB-p50 could be structurally equivalent to a loop preceding the C-terminal helix (K160-M165) in VosA. The interpretation of the experimental electron density map of that loop was not unambiguous, as it is located close to crystallographic 2-fold axis. Hence the two possible conformations of that loop affect the positioning of the remaining C-terminal fragment that can either cover the β-sheet of the same protein molecule or the β-sheet of the adjacent protein molecule related by the 2-fold symmetry by employing a domain swapping (Figure S9).

### VosA Residues Involved in DNA-Binding

**Figure 4:**
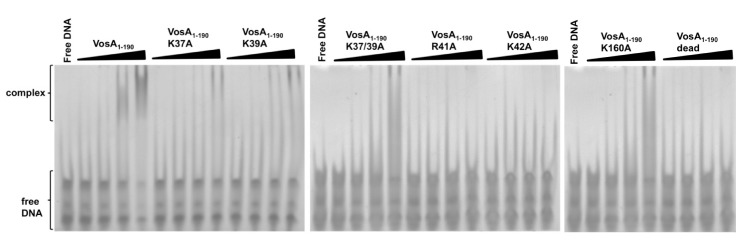
EMSA of the wild-type and mutated VosA_1–190_ proteins. EMSA data using wild-type and mutated VosA_1–190_ with the 35 bp DNA probe of the *brlA* promoter (OHS301/302) containing the predicted VosA-binding sequence are shown. The “dead” mutant contains four substitutions (K37A, K39A, R41A, and K42A). DNA and protein were used in the molar ratios 1∶0.3, 1∶1, 1∶3, and 1∶9. Free DNA without protein was used as negative control.

Utilizing the structural similarity to NF-κB, the mode of DNA binding of the *velvet* domain may be deduced from the superposition of the VosA monomer to crystal structures of NF-κB-DNA complexes [25],[26]—for example, the NF-κB p50 homodimer bound to DNA (PDB ID code 1SVC). The superposition reveals that the loop connecting the first and second β-strand (loop A) as well as the loop located before the C-terminal helix of VosA (loop B) could be involved in DNA binding by interactions with the major groove (Figure 3C). Indeed, several positively charged residues (Lys, Arg) prone for DNA-binding activity are located within these loops (Figure 3D). To test whether these residues are critical for DNA-binding, K37, K39, R41, and K42 located in loop A and K160 in loop B were individually substituted with alanine. The DNA binding activity of the mutated VosA_1–190_ proteins was tested by an EMSA with the 35 bp fragment of the *brlA*β promoter containing the VosA binding motifs (Figure 4) and additionally with the same *brlA*β promoter fragment with deleted VosA binding motifs 1 and 2 (Figure S10), verifying the importance of both motifs in VosA-DNA interaction. Remarkably, severely reduced DNA-binding activity was observed for all mutants located in loop A (Figure 4). In contrast, the loop B K160A mutant retained its DNA-binding capability. This observation suggests a minor, if any, involvement of the second loop in DNA binding and supports its assignment as a flexible loop joining two individual VosA domains. Substituting all four positively charged residues in this loop—namely K37, K39, R41 and K42—simultaneously with alanine completely abolished DNA binding activity. Similar to this quadruple mutant, already the double mutant K37A/K39A cannot bind to DNA anymore (Figure 4). This strongly indicates that this loop of VosA is involved in protein–DNA interactions, which is consistent with structural superposition with the NF-κB-DNA complex (PDB ID code 1SVC) (Figure 3C,D). The function of the double mutated protein K37/39A was also analyzed *in vivo* in *A. nidulans*. The introduction of the mutated version of the *vosA* gene in a *vosA* deletion strain only partially complemented the *vosA* deletion phenotype, suggesting that the mutated protein is not working properly due to its reduced DNA binding activity (Figure S11).

Adding the second molecule of the VosA homodimer to the model of a VosA-DNA complex reveals that a significant bending of the DNA has to occur if both subunits bind DNA simultaneously. The interacting loop A of the second VosA molecule would be positioned about 10 bases distant from the binding site of the first VosA monomer (Figure S12A,B).

### Structure of the VosA_1–190_-VelB Heterodimer

**Figure 5:**
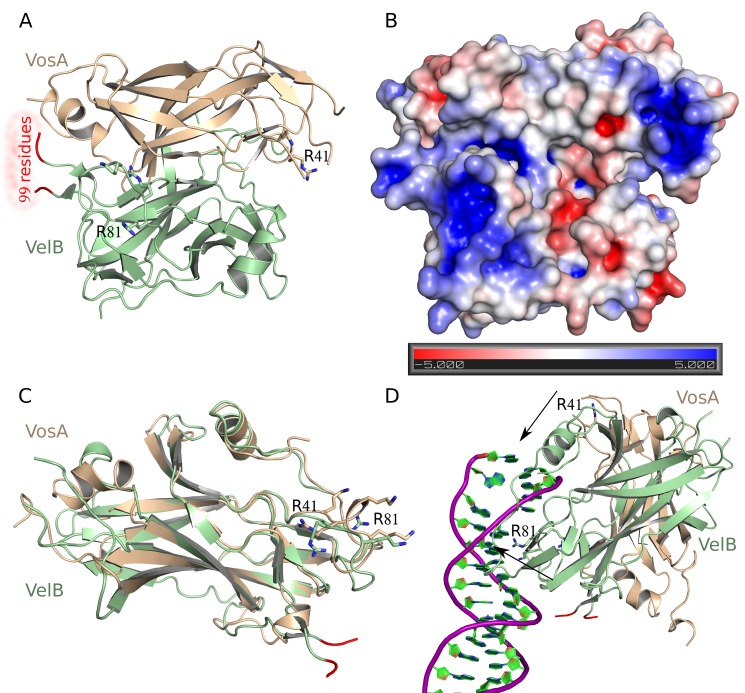
Crystal structure of the VosA_1–190_-VelB complex. (A) Structure of the VosA_1–190_-VelB heterodimer formed by the *velvet* domains of both proteins. Residues 1–7, 37–39, and 161–190 of VosA are not defined in the electron density map, as well as 99 residues inserted into the *velvet* domain of VelB. These 99 residues are predicted to be an intrinsically disordered region that is inserted into VelB in the gap highlighted by the two orange coloured disconnected ends of the VelB polypeptide chain. (B) The electrostatic surface potential of the VosA_1–190_-VelB heterodimer indicates a binding surface for DNA similar to that of the VosA homodimer. Surface representation coloured according to the electrostatic potential ranging from red (−5 kBT/e) through white (0 kBT/e) to blue (+5 kBT/e). (C) Superposition of the *velvet* domains of VosA (in blue) and VelB (in green). The position of the missing 99-residue insertion of the VelB *velvet* domain is highlighted in orange. (D) Proposed model for DNA-binding of the VosA_1–190_-VelB heterodimer based on the NF-κB-DNA complex structure [25]. The arrows show the location of basic residues involved in DNA-binding. Contacts have been optimized for the VelB-subunit leading to a nonoptimal position of the VosA binding site. Hence, some bending of the dsDNA might be introduced upon binding of the heterodimer.

Known physiological functions of VosA depend on the concerted action with the *velvet* protein VelB, which was shown to form a heterodimer with VosA [12],[13]. In order to unveil the molecular basis of the VosA-VelB interaction, we also determined the crystal structure of the VosA_1–190_-VelB heterodimer at a resolution of 2.2 Å (Table 1, Figure 5).

In contrast to the other *velvet*-proteins in *A. nidulans* (VosA, VeA, and VelC), the *velvet*-domain of VelB is not continuous but is interrupted by an insertion of 99 amino acids (residues 132–231; Figure S2). This insertion is rich in proline, glutamine, glycine, tyrosine, and serine residues, and it is predicted to form an intrinsically disordered region. Even though full-length VelB (369 residues) was used for complex formation and crystallization, the insertion is not present in the crystal structure most likely due to a proteolytic removal (Figure S13). This proteolytic activity might also be the reason why residues 161–190 of VosA in this heterodimer structure are not defined in the electron density map, even though residues 161–185 are defined in the structure of the VosA homodimer. Overall VosA exhibits an almost identical fold as in the homodimer structure with an r.m.s.d. of 0.80 Å. The VelB *velvet* domain adopts a fold similar to that of VosA (r.m.s.d. of 1.01 Å for 136 Cα positions), however it contains one additional N-terminal β-strand.

In the heterodimer, VosA and VelB share the same interaction surface with respect to the secondary structure elements involved in the interaction as the monomers of the VosA homodimer (Figure 5). The surface covered by the interaction of both molecules (1,453 Å^2^) is slightly larger than that in the VosA homodimer (1,078 Å^2^). A major difference to the VosA homodimer is that the VelB protein in the VosA-VelB heterodimer is oriented differently. With respect to the second VosA-molecule, in the homodimer it is rotated by around 30° and shifted about 3 Å closer toward VosA (Figure S14). Importantly, the lack of the 99-residue-long insertion in the VelB *velvet* domain does not compromise the binding of VelB to VosA (Figure S5).

Superposition of VelB with VosA reveals that DNA-binding loops A and B in VelB differ in arrangement and/or sequence from VosA (Figure S12). Loop B, which is highly similar in the overall structure to VosA, contains two lysines instead of one, both pointing away from the protein molecule. In both VosA and VelB, loop A is made up of eight residues. While loop A of VelB has an irregular conformation, the corresponding loop A of VosA contains a short helical element resulting in an elongated loop. The lysines in loop A of VosA play an important role in DNA binding, however there is only one lysine in loop A of VelB present and K42 is exchanged into R81. Interestingly, R80 (corresponding to R41 in VosA) is not pointing inward as in the VosA homodimer, but is oriented outward and interacts with a sulfate ion and the neighbouring D77 side chain and D79 carbonyl group. Flexibility of the loop regions comprising the positively charged residues might be a prerequisite for these interactions to contribute to both DNA binding and subsequent stabilization of the loop conformation of VelB (Figure S12).

All together the differences in the DNA-binding loops are indicative for a different interaction surface, which in turn could cause different DNA sequence specificity of VosA and VelB, respectively.

## Discussion

Members of the *velvet* protein family have been defined by a conserved sequence comprising some 150 amino acids, denoted as *velvet* domain. Only in VelB the *velvet* domain contains an insertion of 99 residues of yet unknown function. The crystal structure analysis of VosA and VelB revealed that the *velvet* domain represents a structural entity. The *velvet* domain is involved in specific DNA binding as well as in the dimerization of the different *velvet* proteins, resulting in formation of homo- and heterodimers.

The common fold of the VosA and VelB *velvet*-domain comprises a highly twisted β-sandwich composed of seven antiparallel β-strands, an α-helix inserted in the loop connecting β-strands 2 and 3, and a second, C-terminally located α-helix (Figures 3 and 5). VosA forms a homodimer, as indicated by an interaction surface encompassing 12.7% of the total surface. This is supported by results from gel-filtration and MALS experiments. Head-to-head homodimer formation buries equivalent outside surfaces formed by strands β4, β3, β6, β7 of each VosA monomer resulting in formation of an intradimer eight-stranded β-sandwich. The newly formed β-sandwich buries several hydrophobic residues located on β3, β6, and β7, in particular three Phe (F72, F136, and F145), positioned in a row perpendicular to β-sandwich axis. Thus a cluster of six Phe forming a large hydrophobic patch could be the major driving force for VosA oligomerization. Interestingly, although using the identical region for interaction in the VosA-VelB heterodimer, the complexes significantly differ in relative orientation of individual monomers. This is mostly due to different length and curvature of the β6 and β7 and interconnecting loop found in VelB β-sandwich, which in order to pack against a β-sandwich of VosA needs to be rotated by 32° and shifted by 3 Å. This results in a better fit of the two subunits and increase of the interaction surface, which similarly to the VosA homodimer buries several hydrophobic side chains in the core of newly formed intradimer eight-stranded β-sandwich (Figure 5).

Both proteins reveal a structural similarity to members of the NF-κB-p50-family, especially to a conserved region of about 150 amino acids that resembles an RHD-like fold. In contrast to NF-κB, where the C-terminal domain is responsible for the dimerization and both domains are capable of binding to DNA, the *velvet* domain harbours both functions. Based on the obtained structural information of VosA and VelB and their specific DNA binding properties (discussed below), we propose that the fungal *velvet* proteins represent a new class of direct DNA-binding transcription factors sharing a common ancestor(s) with the NF-κB-p50 family.

Comparison of VosA and VelB overall structure with NF-κB allowed identification of critical residues for DNA-binding activity located in a loop region within a patch of positively charged surface. According to the superposition, binding would occur to the major groove of the DNA. Substitution of these residues with alanine in VosA clearly abolishes DNA-binding activity. The strongest effect on DNA-binding is observed by replacing K37/K39, suggesting that these two residues together with K42 provide the major interactions with the DNA. The introduction of a mutated *vosA* K37/39A in the genome of *A. nidulans* proves that these two residues are important for proper function of the protein. The deletion of *vosA* results in an up-regulation of the *brlA* gene and to a loss of viability after 10 d of growth due to the lack of trehalose. Additionally, the deletion influences the production of sexual spores and of pigments released to the agar (Figure S11B). The introduction of the mutated *vosA* K37/39A allele in a Δ*vosA* strain fails to fully complement these defects. Only the production of sexual spores was restored, whereas the spore viability was partially restored, but the defected control of pigmentation (Figure S11B) and *brlA* expression (Figure S11C) was not rescued.

This patch of positively charged residues defined by K37, K39, and K42 is well conserved among all *velvet* proteins, suggesting a common mode of protein-DNA interaction for VosA and VelB. Furthermore, the structural comparison with NF-κB suggests that the VosA homodimer recognizes about 11 base-pairs, which is in agreement with the predicted 11 nucleotides consensus sequence recognized by VosA in our ChIP-chip analysis.

However, the arrangement of the VosA homodimer and the superposition and modelling of the DNA reveals that the putative DNA-binding loops of the second molecule is at least ∼13 Å distant to the backbone of a dsDNA with ideal B-form conformation. A simultaneous binding to both VosA molecules would require a kink in the DNA. In contrast to the VosA homodimer, in the putative VosA-VelB-DNA complex model with VosA in close proximity to the DNA, the distance of the *velvet* domain of VelB to the DNA backbone is reduced and would require less bending of an ideal B-form DNA for tight interaction. Both DNA sequence motifs 1 and 2 play an important role in DNA binding of VosA, however with differing importance. Deletion of motif 1 has a higher influence on binding efficiency than deletion of motif 2. A recent study demonstrated that the DNA-binding sequence of the *velvet* proteins Ryp2 and Ryp3 in *H. capsulatum* is highly similar to the motif 1 derived from our ChIP-chip result (Table S2) [28].

The electron density obtained from the VosA_1–190_ crystals allows an interpretation of two alternate conformations of its C-terminal helix. The missing C-terminal part of VosA might lead to an overall conformation of VosA similar to the one observed for NF-κB and could increase the effect of loop B on DNA binding. The additional residues present in VosA-N might therefore explain its increased DNA binding in comparison to VosA_1–190_.

In summary, we suggest that the modulation of gene transcription is achieved by the use of varying homo- and heterodimers as seen for VosA and VelB, potentially allowing the *velvet* proteins to specifically recognize different DNA sequences causing differential regulatory outcomes. In *A. nidulans* there are four *velvet* proteins, three of which have been studied. VosA-VelB is essential for the regulation of asexual development and spore viability [11]–[13] and VelB-VeA for sexual development [11]. The trimeric VelB-VeA-LaeA complex coordinates differentiation and secondary metabolism in response to external signals [9]. Little is known about the function of the *velvet* proteins in other clades of fungi outside of the ascomycetes, where much remains to be discovered. Interestingly, the unicellular eukaryote *Capsaspora owczarzaki*, which is a symbiont in the haemolymph of the tropical freshwater snail *Biomphalaria glabrata*, carries a gene for both an NF-κB and a VosA-like protein, suggesting the coexistence of both in this organism [29].

Several studies indicate that the *velvet* proteins are global regulators controlling a diverse set of processes ranging from toxin production and cell wall formation to the development of resting or sexual fruiting structures [6]. It will be interesting to determine what additional common themes and features exist between fungal growth and developmental control by the *velvet* protein family and the immune, inflammation, and differentiation response of animals by the NF-κB protein family. One candidate for a common denominator of RHD and *velvets*' functions is the COP9 signalosome, a conserved multiprotein complex controlling the life span of proteins. The COP9 signalosome is required for the control of NF-κB activation [30] as well as the control of fungal development and secondary metabolism [31]. Recently it has been shown that the physical interactions between the COP9 signalosome and an additional developmental regulator protein are conserved between humans and fungi [32].

Similarly to the NF-κB transcription factors, the founding member of the *velvet* protein family, VeA, resides in the cytoplasm but is prevented from nuclear import in the light by a yet unknown factor or mechanism. It is tempting to speculate that the light signal leads to a posttranscriptional modification—e.g. phosphorylation—of VeA, hindering the interaction with the nuclear import factor KapA. The control of nucleocytoplasmic transport by phosphorylation is known for various proteins like Hxk2, LASP-1, IPMK, and hTERT [33]–[36]. Notably, the regulation on the level of nucleocytoplasmic transport applies also for NF-κB, as the NF-κB inhibitor Iκ-Bα binds to NF-κB, thereby masking the NLS of NF-κB [37]–[39]. However, the similarity of the *velvet* proteins to NF-κB does not extend to this regulatory mechanism, since no Iκ-B homolog or functional homolog could be identified in fungi yet. Hence, the precise understanding of differences and similarities in the molecular mechanisms of the *velvet*/Rel family might help to control fungi, which not only cause increasing problems for human health and crop yield/quality, but also play a crucial role as environmental recyclers, fermenters, industrial producers, and agricultural aids.

## Materials and Methods

### Cloning of VosA_1–190_ and Full-Length VelB

In order to express the truncated VosA (residues 1–190), here denoted as VosA_1–190_, *vosA* cDNA was amplified using the oligos OZG479/480 containing the *Nco*I site. The amplicon was digested with *Nco*I and inserted into the *Nco*I site of pETM13 (EMBL, Heidelberg), yielding the plasmid pETM13-VosA190 with a 3′ coding sequence for a Strep-tag. For construction of plasmid pME3815, *velB* was amplified from *A. nidulans* cDNA with primers JG45/46 and cloned into plasmid pETM-13 digested with *Nco*I and *Xho*I.

The VosA_1–190_ mutations K37A, K39A, K37/39A, R41A, K42A, K160A, and the dead mutation (K37A, K39A, R41A, K42A) were inserted by PCR with mutated primers. For the mutation K37A, the N- and C-terminal VosA fragments were amplified from pETM13-VosA190 with primers OZG479/JG365 and JG366/367. Then, the fragments were fused by PCR with primers OZG479/JG367. The mutations K39A, K37/39A, R41A, K42A, K160A, and the dead mutation were designed in the same way as K37A with the following primers: K39A (OZG479/JG368, JG366/367, OZG479/JG367), K37/39A (OZG479/JG642, JG366/367, OZG479/JG367), R41A (OZG479/JG369, JG366/367, OZG479/JG367), K42A (OZG479/JG370, JG366/367, OZG479/JG367), K160A (OZG479/JG372, JG373/367, OZG479/JG367), and dead (OZG479/JG371, JG366/367, OZG479/JG367). The fused fragments were cloned in *Nco*I-digested pETM13, resulting in plasmids pME3845 (K37A), pME3846 (K39A), pME3845 (K37/39A), pME3847 (R41A), pME3848 (K42A), pME3850 (K160A), and pME3849 (dead).

### Expression and Purification of the VosA_1–190_ Protein

The plasmid pETM13-VosA190 and the mutant forms were transformed into *E. coli* Rosetta 2 (DE3). Expression was carried out in ZYM5052 media [40] at 16°C. Cells were harvested by centrifugation for 20 min at 5,300×g and resuspended in lysis buffer (30 mM HEPES pH 7.4, 400 mM NaCl, 30 mM Imidazol). Cell lysis was performed using a Fluidizer (Microfluidics) at 0.55 MPa. The resulting lysate was cleared by centrifugation at 30,000×g for 30 min at 4°C. The supernatant was applied to a 5 ml StrepTactinHP column (GE Healthcare) equilibrated with lysis buffer. After extensive washing, the protein was eluted with elution buffer S (lysis-buffer +2.5 mM des-thiobiotin). The eluate was applied to a Superdex 200 16/60 column (GE Healthcare) equilibrated in gel-filtration buffer (10 mM HEPES pH 7.4, 400 mM NaCl). The fractions containing VosA_1–190_ were pooled, concentrated to 10.4 mg/ml in centrifugal concentrators (Vivascience), and used for crystallization. VosA mutant forms were expressed and purified as the wild-type, and the gel-filtration step was omitted since the purity was more than 95% as judged by SDS-PAGE.

As a final test for integrity, MALS was performed for selected complexes (Figure S7) and the mutant forms of VosA_1–190_ were compared to the wild-type by means of CD spectroscopy (Figure S15).

### Expression and Purification of the VosA_1–190_-VelB Complex

His-tagged VelB was expressed as described above. Cells expressing VosA_1–190_ with a C-terminal Strep-tag and full-length VelB-His_6_ were harvested, mixed, and lysed in lysis-buffer as described before. The cleared supernatant was applied to a 10 ml NiNTA (GE Healthcare), washed, and eluted with elution buffer N (lysis-buffer +400 mM Imidazol). The resulting eluate was directly applied to a 5 ml StrepTactinHP, washed, and eluted with elution buffer S. The complex was further purified using a Superdex 200 16/600 column (GE Healthcare) equilibrated with gel filtration buffer. The fractions containing the monomeric VelB and VosA_1–190_ proteins were pooled, concentrated to 11.6 mg/ml, and used for crystallization.

### Cloning, Expression, and Purification of GST Tagged VosA

To express the GST tagged VosA proteins used for EMSA and the GST-pull-down experiments, cDNA of the full-length *vosA*, *vosA*-N (N-terminal region, 1–216 aa), or *vosA*-C (C-terminal region, 217–430 aa) was cloned between *Eco*RI and *Sal*I sites (for full-length *vosA*) in pGEX-5X-1 (Amersham) or cloned between *Bam*HI and *Sal*I sites (for *vosA*-N and *vosA*-C) in pGEX-4T-3 (Amersham) to make pNI47, 49, and 50, respectively. These plasmids were introduced into *E*. *coli* BL21 (DE3). The GST fusion protein expression and purification was performed following the manufacturer's (GE Healthcare) instructions. To concentrate and buffer exchange, Amicon Ultra Centrifilter Units (Milllipore) were used. BCA Protein Assay Kit (Pierce) was used to estimate the protein concentration.

### Expression and Purification of the VeA Protein

To express the GST tagged VeA protein used for EMSA experiments, cDNA of the full-length *veA* was amplified with primers OHS723/724 and cloned between *Sal*I and *Not*I sites in pGEX-5X-1 (Amersham). This plasmid (pHS51) was introduced into *E. coli* BL21 (DE3). The GST fusion protein was expressed as described for VosA_1–190_, and purification was performed following the manufacturer's (GE Healthcare) instructions. VeA_1–224_ was cloned into the *Nco*I site of pETM13 (EMBL, Heidelberg), yielding the plasmid pETM13-VeA_1–224_ with a 3′ coding sequence for a Strep-tag. The protein was expressed and purified as described for VosA_1–190_.

### Crystallization and Data Collection

VosA_1–190_ and VosA_1–190_-VelB were crystallized by the sitting-drop vapour diffusion method. X-shaped crystals of VosA_1–190_ grew after 1 d in a condition containing 100 mM MES pH 6.5, 30% PEG 4000 at 20°C. Further optimization led to crystals with the same morphology in a condition containing 100 mM MES pH 6.5, 32% PEG 2000 MME, 150 mM KI, which were used for structure determination. Prior to data collection, crystals were cryo-protected by soaking in reservoir solution supplemented with 12% (v/v) 1,4-butane-diol. X-ray diffraction data were collected at 110 K on a Rigaku MicroMax 007 rotating Cu-anode equipped with a MAR345dtb image-plate detector (Mar Research). The VosA crystals belong to the space group P4_1_22 and have cell dimensions of a = b = 45.40 Å and c = 189.43 Å.

The VosA_1–190_-VelB complex was crystallized in 100 mM MES pH 5.5, 150 mM ammonium sulfate, and 25% (w/v) PEG 4000. The plate-like crystals were cryo-protected in crystallization solution +10% (v/v) 1,4-butanediol. Diffraction data were collected at 100 K at the ESRF microfocus beamline ID23-2. The crystals belong to the space group P2_1_2_1_2_1_ and have cell dimensions of a = 52.03, b = 56.75 Å, and c = 138.17 Å.

### Data Processing and Structure Determination

X-ray diffraction data from the VosA_1–190_ crystal were integrated and scaled with the XDS package [41]. Phases were obtained by SAD using SHELXC/D/E [42] navigated through HKL2MAP [43], which found 10 iodine ions. However, for further processing only iodine ions with occupancy >0.2 were used, which resulted in three sites with occupancies of 1.0, 0.36, and 0.24. The initial electron density map was readily interpretable and the majority of residues were built automatically using ARP/wARP [44]. Manual model building of missing residues was done with COOT [45]. Refinement was carried out in PHENIX [46] and Refmac5 [47]. The final model contains one monomer in the asymmetric unit. Diffraction data obtained from the VosA_1–190_-VelB crystals were integrated and scaled with MOSFLM [48] and SCALA from the CCP4-package [49], respectively.

The calculated Matthews coefficient of 1.59 Å^3^ Da^−1^ (corresponding to a solvent content of 22.9%) excluded the presence of both proteins, VosA and VelB, used for crystallization trials in the asymmetric unit. Assuming that the asymmetric unit comprises two protein molecules with the size of VosA_1–190_, the calculated Matthews coefficient value is 2.27 Å^3^ Da^−1^ (45.6% solvent content). At this step of structure solution, the assumption was made to look for two molecules using the available model of VosA_1–190_ comprising 143 amino acids (Ser 17 to Asp 79 and Ala 86 to Met 165). As the two proteins (VosA and VelB) share about 42% amino acid identity, the VosA_1–190_ model could also be used to search for the fragments of VelB in case a proteolytic digest had occurred prior to crystal formation. The molecular replacement search was carried out with PHASER [50] using data between 30 and 2.2 Å resolution. The search yielded two prominent solutions with an overall likelihood gain (LLG) score of 587 (the first molecule RFZ = 10.7, TFZ = 2 0.4, LLG = 236; the second molecule RFZ = 4.4, TFZ = 16.5, LLG = 501) and R factor of 53.4%. The quality of initial electron density maps (overall mean FOM of 0.41) was not good enough to distinguish whether two VosA_1–190_ or VosA_1–190_ and truncated VelB molecules were occupying the asymmetric unit. The structure was manually rebuilt in COOT and verified against simulated annealing (SA) omit maps calculated with CNS [51], which was also used during initial refinement steps. Refinement was based on slow-cooling SA (both torsion angle dynamics and Cartesian dynamics) combined with standard minimization and individually restrained B-factor refinement. Careful inspection of the electron density maps indicated differences between the two molecules in the asymmetric unit that were modelled and finally let us identify the second molecule as a proteolytically truncated VelB. The final model contains two molecules in the asymmetric unit.

### EMSA

Probes for EMSA were generated by annealing two single-stranded reverse-complementary oligonucleotides. Binding reactions were performed in a 10 µl reaction volume containing 10 mM HEPES/NaOH (pH 7.4), 150 mM NaCl, ∼53 pmol DNA probe, and appropriate amount of each purified VosA protein. The reactions were incubated at RT for 15 min. The complexes were resolved on a 6% polyacrylamide gel (37.5∶1 crosslinking) with 0.5% TBE running buffer at 200 V at RT for 20 min. The gel was stained with ethidium bromide.

### ChIP-on-Chip

For sample preparation, 2-d-old conidia (∼5×10^9^ conidia for three ChIP experiments) from the strain TNI10.34.1 (Δ*vosA*; *vosA*::FLAG) were crosslinked with fresh 1% formaldehyde at RT for 30 min. Then, 1/20 volume of 2.5 M glycine solution was added to stop the cross-linking reaction. The conidia were collected by centrifugation and the conidia were washed with cold PBS for three times. About 1.5 ml FA lysis buffer (50 mM HEPES-KOH [pH 7.5], 150 mM NaCl, 1 mM EDTA, 1% Triton X-100, 0.1% Na deoxycholate, 0.1% SDS, 1 protease inhibitor cocktail tablet (Roche) per 50 ml) was added before use. Silica beads (∼300 µl) were added into cross-linked cell lysates and the lysates were broken by a mini-bead beater for three cycles (1.5 min homogenization with 1.5 min sitting on ice). Subsequently the samples were sonicated for seven cycles (30 s on, 60 s off) with a sonifier equipped with a microtip at 70% amplitude and level 5 of output control. All steps were carried out on ice. The sonicated cell lysates were cleared of cellular debris by a 2,000×g spin for 3 min five times. The supernatant was collected, and its DNA concentration was checked using a biophotometer (Eppendorf). About 25 µl supernatant was kept as input chromatin control (glycerol was added to 10% final concentration if samples had to be frozen). The rest of supernatant was adjusted to 10 ml with FA lysis buffer, and 50 µl of Anti-Flag M2-agarose from mouse (SigmaAldrich) was added for ChIP with constant mixing overnight at 4°C. The agarose beads were collected by centrifugation and washed with FA lysis buffer three times. About 50 µl elution buffer (10 mM Tris, pH 8.0, 1 mM EDTA, 1% SDS) was added to the beads, and the sample incubated at 65°C for 10 min to elute chromatin samples from the beads. Both the recovered supernatant from the elution and the input chromatin supernatant (saved before ChIP) were adjusted to 170 µl by elution buffer, and incubated at 65°C overnight to reverse the crosslinking reaction. Then, the samples were treated by protease K for 2 h at 37°C, and protein extracted twice with phenol (equilibrated with TE, pH 8.0) and once with chloroform∶isoamyl alcohol (24∶1). DNA was precipitated by ethanol and resuspended in 30 µl RNase TE (to digest RNA). Finally, the DNA was purified by QIAquick PCR purification kit (Qiagen) and eluted in 30 µl of the elution buffer provided with the kit. To obtain sufficient amounts of DNA for further labelling and hybridization, DNA was amplified using the WGA kit (Sigma, WGA2). DNA labelling and hybridization were performed by Roche Nimblegen. The DNA bound by the VosA-FLAG protein was labelled with Cy5, while the control DNA was labelled with Cy3. We used the *A. nidulans* whole-genome tiling-oligonucleotide array containing 65,536 oligonucleotides (50∼65 nucleotides at a 75-bp interval, Roche Nimblegen). The array data from hybridization of two independent immune-precipitation experiments for the VosA∶FLAG strain were used for analysis. The data were processed using NimbleScan (Roche Nimblegen). Briefly, NimbleScan detects peaks by searching four or more probes whose signals are above the specified cutoff values using a 500 bp sliding window. The ratio data were then randomized 20 times to calculate the probability for being “false positive.” Every peak is assigned with a false discovery rate (FDR) score based on the randomization. The lower the FDR score, the higher the possibility that the peak corresponds to a real binding site. When finalizing the candidates, we used the cutoff value “1” (log 2 ratio of experiment to control) as peak score and cutoff value “0.05” for FDR score. Then we found the overlapping candidates from two chips.

### VosA Binding Motif Analysis

To define the DNA motif recognized by VosA, we employed two separate approaches. First, using the VosA-FLAG ChIP-on-chip results, sequences of 400 bp on the midpoint of about 1,500 enriched peaks from the two VosA-ChIP biological replicates were used as target sequences. As a background, 6 kbp sequences on about 6,000 promoter regions of genes without the VosA peaks were used (BIOINFORX Inc., Madison, WI, USA). The data were then analysed using HOMER (Hypergeometric Optimization of Motif EnRichment; http://biowhat.ucsd.edu/homer/). The predicted VosA binding motifs are presented in Table S2. Second, the ChIP-on-chip data were imported into RINGO [52], an R/Bioconductor package, for the analysis of ChIP-chip readouts, including the quality assessment, normalization, smoothing, and peak calling. We normalized the probe intensities by variance-stabilizing normalization (VSN) [53]. We only included the peaks that were consistent in two independent VosA-ChIP biological replicates. Then several sequences were selected according to maxLevel, the highest smoothed probe level in the enriched region (Table S3). We then examined mRNA levels of these genes and found that 10 genes' mRNA levels are affected by VosA and/or VelB (Figure 2B). Finally, VosA-ChIP enriched sequences from these 10 genes, four sequences from the known target genes *wetA*, *tpsA*, *orlA*, and *treA*, and the 35 bp *brlAβ* promoter fragment shown to be bound by VosA in EMSA were subject to MEME (Multiple Em for Motif Elecitation) analysis, which led to the predicted VosA binding motif CTGGCCaaGGC (Figure 1C).

### ChIP-PCR

ChIP-PCR analysis was performed according to the manufacturer's instructions with a minor modification using MAGnify Chromatin Immunoprecipitation System (Invitrogen). Two-day-old conidia (1×10^9^) of WT (FGSC4), Δ*velB* (THS16.1), and Δ*vosA* (THS15.1) strains [13] were cross-linked with fresh 1% formaldehyde at RT for 15 min. Then, 1/20 volume of 2.5 M glycine solution was added to stop the cross-linking reaction. The conidia were washed and broken by a mini-bead beater for two cycles (1 min homogenization with 1 min sitting on ice). The cell lysates were sonicated for four cycles (30 s on, 60 s off) with a sonifier. The sonicated cell lysates were cleared of cellular debris by centrifugation at 13,000×g for 10 min. The diluted chromatin extracts were incubated with 2 µg of anti-FLAG antibody-Dynabeads complex for 2 h at 4°C and then washed three times with the IP buffer. The input control and chromatin sample were eluted from the beads at 55°C for 15 min with reverse crosslinking buffer with proteinase K. DNA was purified by DNA purification Magnetic Beads (Invitrogen). For amplification of precipitated DNA by PCR, the GO Taq DNA polymerase (Promega) was used. The primer sets used for PCR are shown in Table S4. As negative controls, the chromatin extract being incubated with bead only (without anti-FLAG antibody) and the samples of FGSC 4 lacking FLAG-tagged VosA or VelB (Figure S3) were used. Individual input DNA samples before immune-precipitation (IP) were used as positive controls. Two biological replicates have provided essentially identical ChIP-PCR results. Signal intensities of PCR results obtained from ChIP assays were analyzed by the ImageJ software available online (National Institutes of Health; http://rsbweb.nih.gov/ij/).

### Nucleic Acid Manipulation

Total RNA isolation and Northern blot analyses were carried out as previously described [54],[55]. The DNA probes were prepared by PCR-amplification of the coding regions of individual genes with appropriate oligonucleotide pairs using FGSC4 genomic DNA as a template (Table S4).

### Accession Numbers

Coordinates and structure factors have been deposited in the PDB, namely VosA as PDB ID code 4N6Q and VosA-VelB as PDB ID code 4N6R.

## Supporting Information

Figure S1 **Schematic drawing of the roles of the** ***velvet*** **regulators in** ***Aspergillus nidulans*** **development.** The VelB protein is part of at least two *velvet* protein complexes. In darkness VosA-VelB inhibits asexual development and simultaneously VelB-VeA favours sexual development. The nuclear heterotrimeric complex consisting of the *velvet* proteins VeA-VelB and the non-*velvet* protein LaeA coordinates sexual development with production of the mycotoxin Sterigmatocystin. Light inhibits this process and favours the asexual program, as the nuclear bridging of VelB to LaeA by VeA is interrupted. VosA contains a NLS, whereas VeA supports the transport of VelB into the nucleus. VosA is also found in the nucleus of spores and the VosA-VelB heterodimer plays an additional important role for spore maturation and long-term viability by coupling spore formation and trehalose biogenesis in spores.
(TIF)

Figure S2 **Comparison of domains of the** ***velvet*** **family proteins (VD) and RHD proteins.** (A) The *velvet* family proteins VeA, VelC, and VosA from *A. nidulans* contain a homologous *velvet* domain (VD) encompassing a 150–200 amino acid region. The *velvet* domain of VelB is disrupted by an insertion of 99 amino acids. NLS, nuclear localization sequence; NES, nuclear export sequence; PEST, proline (P), glutamate (E), serine (S), and threonine (T) rich region presumably involved in stability of the protein; TAD, transcription activation domain. (B) Conserved domains of the Rel family protein. All members of the Rel family contain a RHD covering 150–200 amino acids as well as NLS. RelB has an additional leucine zipper domain (LZD) at the N-terminus. RelA, RelB, and c-Rel possess a transcription activation domain (TAD), whereas NF-κB2 and B1 have multiple ankyrin repeats (ARs) in the C-terminal region of the proteins, which play an important role in dimer formation and nuclear transport.
(TIF)

Figure S3 **Results of the VosA-ChIP-PCR and VelB-ChIP-PCR.** (A) Photos of the PCR amplicons separated on a 2% agarose gel are shown. The input DNA before immuno-precipitation (IP) was used as a positive control (input). The chromatin extract being incubated with bead only (without anti-FLAG antibody) was used as a negative control (NC). The samples of FGSC 4 lacking FLAG-tagged VosA or VelB were used as negative controls. (B) Densitometric analysis of ChIP data analyzed using the ImageJ software. Representative results are shown. Fold enrichment = IP/input. Error bars represent standard deviation (differences between VelB-FLAG/VosA-FLAG and FGSC 4 strains. *** *p*<0.001; ** *p*<0.01; * *p*<0.05).
(TIF)

Figure S4 **VeA binds the** ***brlA*** **promoter.** (A) EMSA using VosA_1–190_, VelB, VeA_1–224_, and GST-VeA with the 35 bp DNA probe of the *brlA* promoter (OHS301/302). DNA and protein were used in the molar ratio 1∶3. GST was used as negative control. (B) VosA_1–190_ and VeA_1–224_ bind equally well to the *brlA* promoter. EMSA using VosA_1–190_ and VeA_1–224_ with the 35 bp DNA probe of the *brlA* promoter (OHS301/302). DNA and protein were used in the molar ratios 1∶0.3, 1∶1, 1∶3, and 1∶9.
(TIF)

Figure S5 **EMSA using VosA_1–190_, the VosA_1–190_-VelB heterodimer, VelB, and VeA_1–224_ with the wild-type and mutated DNA probes of the** ***brlA*** **promoter.** In the mutated versions of the probe, the predicted VosA binding motifs 1 and 2 were deleted (brlA, wild-type DNA (OHS301/302); brlAΔ2, DNA with deleted motif 2 (JG636/637); brlAΔ1, DNA with deleted motif 1 (JG638/639); brlAΔ2Δ1, DNA with deleted motifs 2 and 1(JG640/641)). DNA and protein were used in the molar ratio 1∶3. Free DNA without protein was used as negative control.
(TIF)

Figure S6 **Size exclusion chromatography of VosA_1–190_ and VosA_1–190_-VelB_ΔIL complex.** VelB_ΔIL is a VelB construct lacking the amino acid insertion within the *velvet* domain (for cloning, expression, and purification, see Text S1). (A) Superposition of the elution profiles of VosA_1–190_ and of the VosA_1–190_-VelB_ΔIL complex on a Superdex 200 (16/60) gelfiltration chromatography column. The elution volumes indicate the presence of a homodimeric VosA_1–190_ and heterodimeric VosA_1–190_-VelB_ΔIL, respectively. (B) The SDS PAGE of the peak fraction of VosA_1–190_ and (C) of all peak fractions of VosA_1–190_-VelB_ΔIL complex.
(TIF)

Figure S7 **VosA_1–190_ forms dimers with itself and the VelB_ΔIL mutant as shown by size exclusion chromatography and subsequent mass determination using MALS.** Dimerization is independent of mutations in the loop region required for DNA binding. VosA_1–190_ is shown in green; VosA_1–190_ dead, quadruple mutant of VosA with the mutations K37A, K39A, R41A, and K42A is shown in black. VosA_1–190_ and VelB_ΔIL is shown in red. VelB_ΔIL, residues 44–126 and 240–343 connected by a short linker (see also Text S1).
(TIF)

Figure S8 **Structure-based sequence alignment of the crystallized VosA fragment with NFκB (PDB ID code 1SVC) and VelB reveals a high structural similarity in the canonical fold region but differences in loop regions and the C-terminal region.** (A) Overall structure of VosA_1–190_ in rainbow colouring (from N-terminus (blue) to C-terminus (red)) with the individual secondary structure motifs depicted in cartoon mode and labelled. (B) Superposition of VosA (orange colours) and NF-κB (blue colours) structures. (C) Structure-based sequence alignment of VosA (top) and NF-κB (PDB ID code 1SVC; bottom). (D) Structure-based sequence alignment of VosA (top) and VelB (bottom).
(TIF)

Figure S9 **Arrangement of VosA_1–190_ molecules around 2-fold crystallographic axis in the crystal lattice.** (A, B, C, D) Two possible conformations of a loop resulting in intermolecular (A, B) or swapped (C, D) placement of the C-terminal α-helix are shown. (E) The Simulated Annealing omit mFo-DFc electron density map contoured at 3σ level is consistent with two alternate conformations of the C-terminal helix of VosA_1–190_. The C-terminally located helix of VosA_1–190_ is found in two conformations influencing the arrangement of LoopB, and thus its DNA binding capabilities. The missing part of VosA might lead to an overall conformation of VosA similar to the one observed for NFκB increasing the effect of LoopB on DNA binding. The additional residues present in VosA-N might therefore explain its increased DNA binding.
(TIF)

Figure S10 **EMSA using the mutated VosA proteins with the wild-type and mutated DNA probes of the** ***brlA*** **promoter.** In the mutated versions of the DNA, the predicted VosA binding motifs 1 and 2 were deleted (brlA, wild-type DNA (OHS301/302); brlAΔ2, DNA with deleted motif 2 (JG636/637); brlAΔ1, DNA with deleted motif 1 (JG638/639); brlAΔ2Δ1, DNA with deleted motifs 2 and 1(JG640/641)). DNA and protein were used in the molar ratio 1∶9.
(TIF)

Figure S11 ***In vivo*** **analysis of the VosA K37/39A protein mutations.** (A) Southern hybridization to verify the integration of the transformation cassette. The cassette was integrated twice, at the *vosA* and unknown loci. (B) Phenotypic analysis of the *A. nidulans vosA*K37/39A mutant compared to the wild-type (wt) and the *vosA* deletion (Δ*vosA*) mutant. For asexual development, strains were grown 3 d on minimal medium in light at 37°C. For sexual development, strains were cultivated 7 d in darkness at 37°C. The fruiting bodies were squeezed in water to liberate the red ascospores. Additionally, a viability test was performed. Each strain was cultivated 10 d at 37°C and 250 of these old spores were subsequently grown 2 d at 37°C to compare the amount of surviving colonies. (C) Relative mRNA levels of wild-type, Δ*vosA*, and *vosA*K37/39A measured by quantitative real-time PCR with the primers specific for *brlA* and *vosA*. Strains were cultivated vegetative and asexually. Note that the *vosA*K37/39A mutant allele fails to down-regulate *brlA* properly.
(TIF)

Figure S12 **Superposition-based modelling of DNA (from the NFκB complex structure PDB ID code 1SVC) on the VosA_1–190_ homodimer (A, B) and VosA_1–190_-VelB heterodimer (C, D) and its spatial arrangement.** The overall arrangement of the protein dimers suggests differences in DNA conformation for binding. Whereas the VosA_1–190_ homodimer could bind on the more or less straight superhelical DNA (A and 90° rotation B), the arrangement of the DNA has to be more kinked for a good fit.
(TIF)

Figure S13 **Proteolytic degradation of VelB.** Analysis of VosA_1–190_-VelB crystals by silver stained SDS_PAGE (17.5%) gel reveals proteolytic digest of the proteins present in the crystals.
(TIF)

Figure S14 **Comparison of the VosA_1–190_ homodimer and the VosA_1–190_-VelB heterodimer.** (A) The dimers have been superimposed with the VosA subunit shown as a white surface representation. The other subunit of the dimer is presented as the ribbon plot. The VelB subunit binds to VosA_1–190_ via the same surface, but the VelB subunit is rotated by 30° and shifted by ∼3 Å with respect to the second VosA_1–190_ molecule of the homodimer. (B) Dimerization results in formation of an intermolecular eight-stranded β-sandwitch (orientation similar to A, the right panel). (C) Electrostatic surface representation (orientation as on A, middle panel). Due to missing residues of VosA_1–190_ (37–39, 161–190) in VosA_1–190_-VelB complex, complete VosA_1–190_ monomer has been superimposed and used for figure generation. Surface representation coloured according to the electrostatic potential ranging from red (−5 kBT/e) through white (0 kBT/e) to blue (+5 kBT/e).
(TIF)

Figure S15 **VosA_1–190_ exhibits identical curves in CD spectroscopy independent of the individual or multiple point mutations in the putative DNA binding loops.** The secondary structure content deduced from these curves as described in Materials and Methods indicates no significant differences in secondary structure content of the individual mutant forms of VosA_1–190_. The last column shows the values for the secondary structure elements deduced from the crystal structure as determined by DSSP.
(TIF)

Table S1 **Raw data of the ChIP-on-chip and ChIP-PCR experiments.**
(PDF)

Table S2 **Proposed VosA-binding motifs from VosA-ChIP-chip data.**
(PDF)

Table S3 **Selected VosA target genes used for MEME analysis.**
(PDF)

Table S4 **DNA oligos used in this study.**
(PDF)

Text S1 **Supplementary material and methods.**
(PDF)

## Abbreviations

EMSA: electrophoretic mobility shift assays
IP: immuno-precipitation
MEME: Multiple Em for Motif Elicitation
NC: negative control
NF-κB: Nuclear Factor-κB
VeA: velvet A
VelB: Velvet like B
VelC: Velvet like C
VosA: viability of spores A
