# Correction: The Velvet Family of Fungal Regulators Contains a DNA-Binding Domain Structurally Similar to NF-κB

**DOI:** 10.1371/journal.pbio.1001849

**Published:** 2014-04-10

**Authors:** The PLOS Biology Staff

Some panels of Figure 1 also appear in more complete form in Supplementary Figures S3 and S5. To clarify this, the authors have supplied a corrected version of the legend to Figure 1.

**Figure 1:**
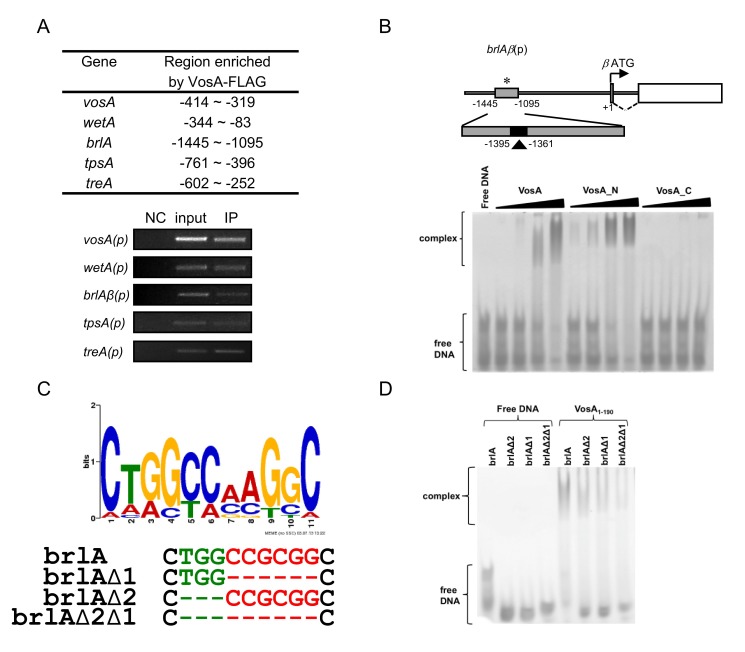
VosA binds DNA specifically. (A) Selected regions enriched by VosA-FLAG ChIP on chip. The promoter regions of brlA, wetA, vosA, tpsA, and treA enriched by VosA-FLAG ChIP are shown (the start codon ATG is +1). Results of VosA-ChIP-PCR, the PCR amplicons separated on a 2% agarose gel, are shown in the bottom panel. The input DNA before immuno-precipitation (IP) was used as a positive control (input). The chromatin extract being incubated with bead only (without anti-FLAG antibody) was used as a negative control (NC). (B) Schematic presentation of the promoter region of brlA. The gray box (−1,095∼1,445 region of brlA(p), marked by *) represents the ChIP-PCR amplified region shown in (A), and the filled box (marked by arrowhead) represents a 35 bp fragment used in EMSA. EMSA using serially diluted VosA proteins and the 35 bp DNA probe of the brlA promoter (OHS301/302). DNA and protein were used in the molar ratios 1:0.3, 1:1, 1:3, and 1:9. VosA, full-length VosA; VosA_N, truncated VosA (residues 1–216); VosA_C, truncated VosA (residues 217–430). (C) The consensus DNA sequence predicted to be recognized by VosA is shown. Two cores of this motif were deleted as shown (green, red) and these probes were used for additional EMSA. (D) EMSA using the crystallized VosA1–190 (VosA_cryst) with wild-type and mutated DNA probes of the brlA promoter. In the mutated versions of the DNA, the predicted VosA binding motifs 1 and 2 were deleted (brlA, wild-type DNA (OHS301/302); brlAΔ2, DNA with deleted core 2 (JG636/637); brlAΔ1, DNA with deleted core 1 (JG638/639); brlAΔ2Δ1, DNA with deleted cores 2 and 1 (JG640/641)). The gels from which cropped panels in (A) were taken are shown in full in Figure S3. The complete gel from which (B) is taken is shown in Figure S5.

The authors inadvertently used the wrong EMSA gel in the central panel of Figure 4, resulting in a duplication. The right-hand panel (VosA_1-190_ K160A and VosA_1-190_ dead) was correct, but the central panel (VosA_1-190_ K37/39A, VosA_1-190_ R41A and VosA_1-190_ K42A) incorrectly showed the same gel. The authors have confirmed that this error does not affect the interpretation of the results or conclusions of the paper. The authors apologize for any confusion caused by this mistake. The authors have provided a corrected version here.

**Figure 4:**
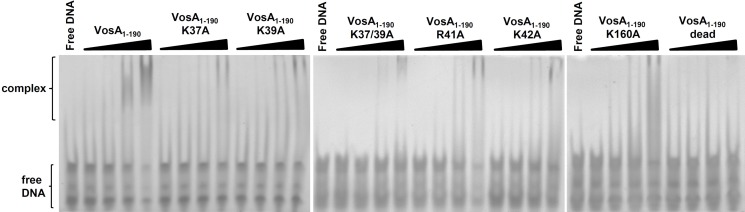
EMSA of the wild-type and mutated VosA1–190 proteins. EMSA data using wild-type and mutated VosA1–190 with the 35 bp DNA probe of the brlA promoter (OHS301/302) containing the predicted VosA-binding sequence are shown. The “dead” mutant contains four substitutions (K37A, K39A, R41A, and K42A). DNA and protein were used in the molar ratios 1:0.3, 1:1, 1:3, and 1:9. Free DNA without protein was used as negative control.
