# Supplementary figures and images for: The Velvet Family of Fungal Regulators Contains a DNA-Binding Domain Structurally Similar to NF-κB
Source: PLoS Biol. 2013 Dec 31;11(12):e1001750. doi: 10.1371/journal.pbio.1001750 (PMC3876986; doi:10.1371/journal.pbio.1001750)

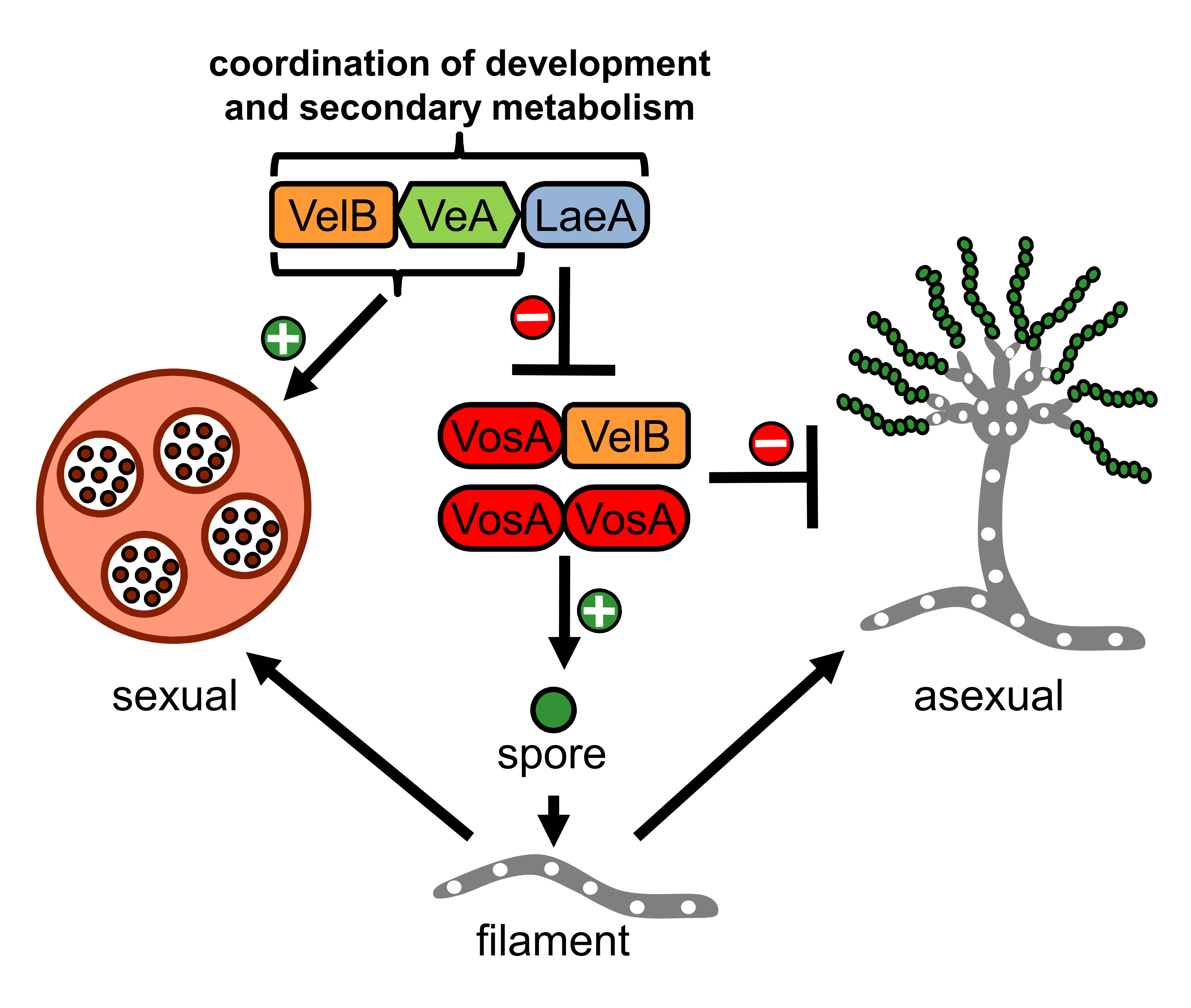

Supplement: Figure S1 — Schematic drawing of the roles of the velvet regulators in Aspergillus nidulans development. The VelB protein is part of at least two velvet protein complexes. In darkness VosA-VelB inhibits asexual development and simultaneously VelB-VeA favours sexual development. The nuclear heterotrimeric complex consisting of the velvet proteins VeA-VelB and the non-velvet protein LaeA coordinates sexual development with production of the mycotoxin Sterigmatocystin. Light inhibits this process and favours the asexual program, as the nuclear bridging of VelB to LaeA by VeA is interrupted. VosA contains a NLS, whereas VeA supports the transport of VelB into the nucleus. VosA is also found in the nucleus of spores and the VosA-VelB heterodimer plays an additional important role for spore maturation and long-term viability by coupling spore formation and trehalose biogenesis in spores. (TIF) [file pbio.1001750.s001.tif]

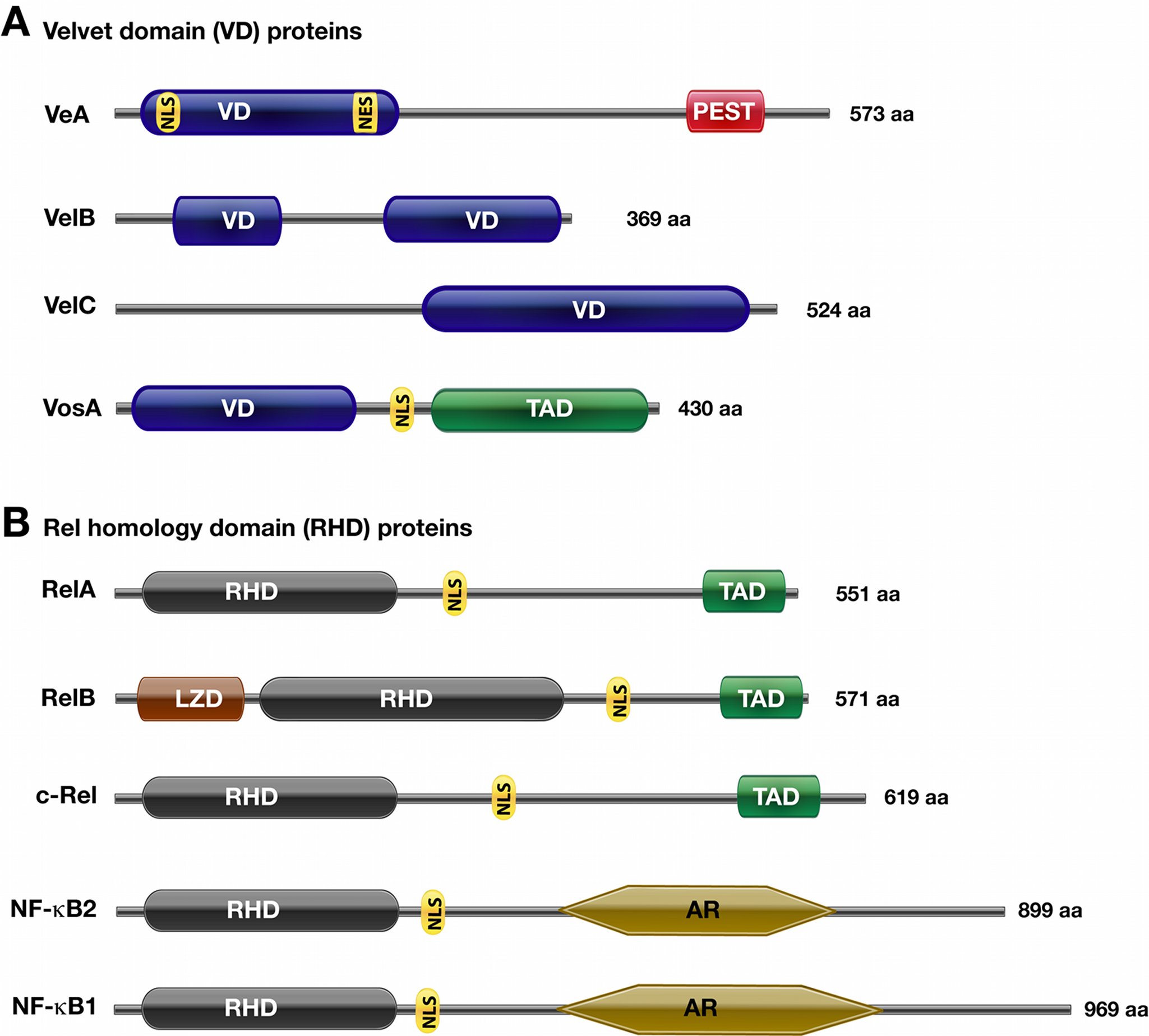

Supplement: Figure S2 — Comparison of domains of the velvet family proteins (VD) and RHD proteins. (A) The velvet family proteins VeA, VelC, and VosA from A. nidulans contain a homologous velvet domain (VD) encompassing a 150–200 amino acid region. The velvet domain of VelB is disrupted by an insertion of 99 amino acids. NLS, nuclear localization sequence; NES, nuclear export sequence; PEST, proline (P), glutamate (E), serine (S), and threonine (T) rich region presumably involved in stability of the protein; TAD, transcription activation domain. (B) Conserved domains of the Rel family protein. All members of the Rel family contain a RHD covering 150–200 amino acids as well as NLS. RelB has an additional leucine zipper domain (LZD) at the N-terminus. RelA, RelB, and c-Rel possess a transcription activation domain (TAD), whereas NF-κB2 and B1 have multiple ankyrin repeats (ARs) in the C-terminal region of the proteins, which play an important role in dimer formation and nuclear transport. (TIF) [file pbio.1001750.s002.tif]

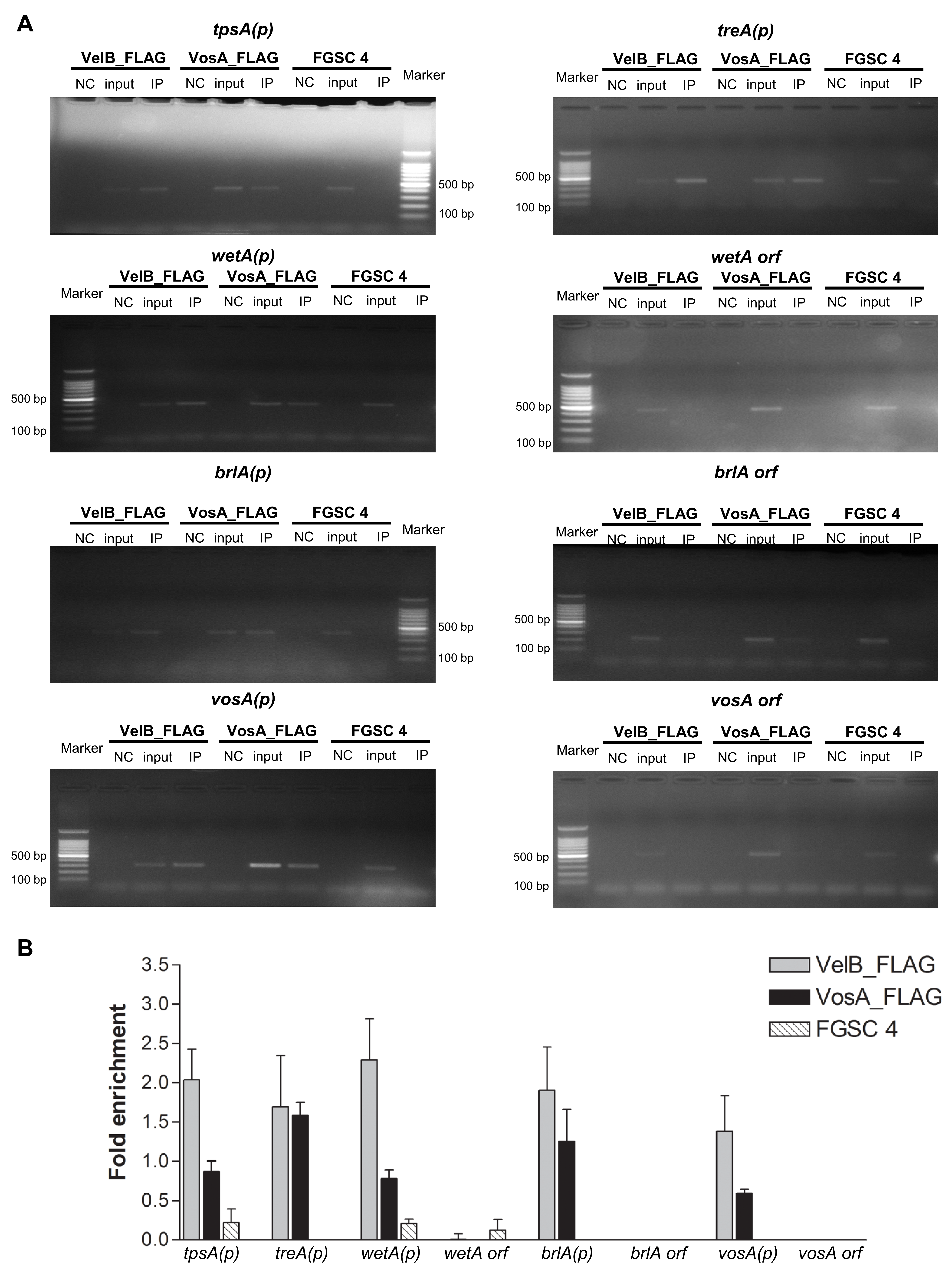

Supplement: Figure S3 — Results of the VosA-ChIP-PCR and VelB-ChIP-PCR. (A) Photos of the PCR amplicons separated on a 2% agarose gel are shown. The input DNA before immuno-precipitation (IP) was used as a positive control (input). The chromatin extract being incubated with bead only (without anti-FLAG antibody) was used as a negative control (NC). The samples of FGSC 4 lacking FLAG-tagged VosA or VelB were used as negative controls. (B) Densitometric analysis of ChIP data analyzed using the ImageJ software. Representative results are shown. Fold enrichment = IP/input. Error bars represent standard deviation (differences between VelB-FLAG/VosA-FLAG and FGSC 4 strains. *** p<0.001; ** p<0.01; * p<0.05). (TIF) [file pbio.1001750.s003.tif]

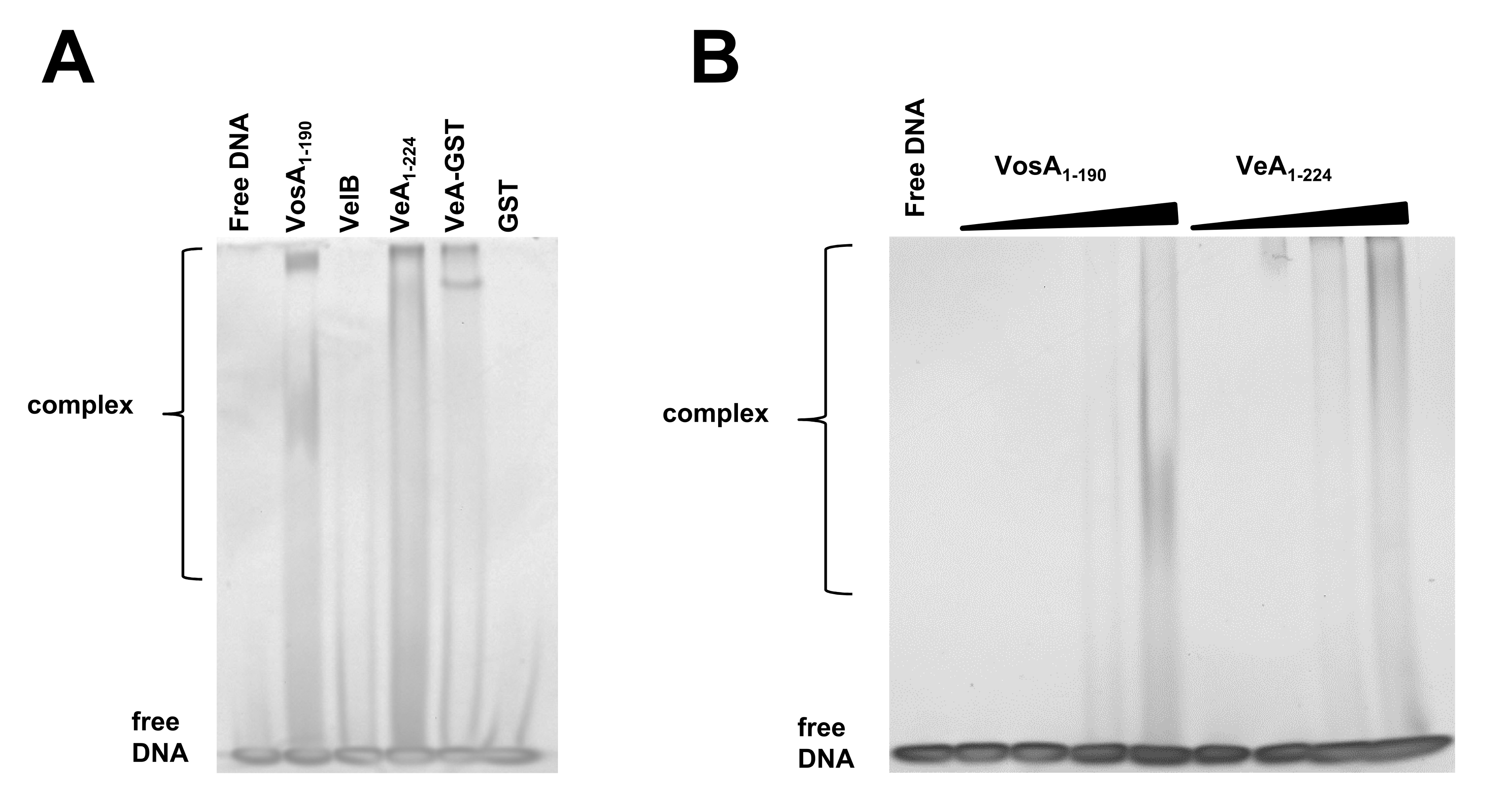

Supplement: Figure S4 — VeA binds the brlA promoter. (A) EMSA using VosA1–190, VelB, VeA1–224, and GST-VeA with the 35 bp DNA probe of the brlA promoter (OHS301/302). DNA and protein were used in the molar ratio 1∶3. GST was used as negative control. (B) VosA1–190 and VeA1–224 bind equally well to the brlA promoter. EMSA using VosA1–190 and VeA1–224 with the 35 bp DNA probe of the brlA promoter (OHS301/302). DNA and protein were used in the molar ratios 1∶0.3, 1∶1, 1∶3, and 1∶9. (TIF) [file pbio.1001750.s004.tif]

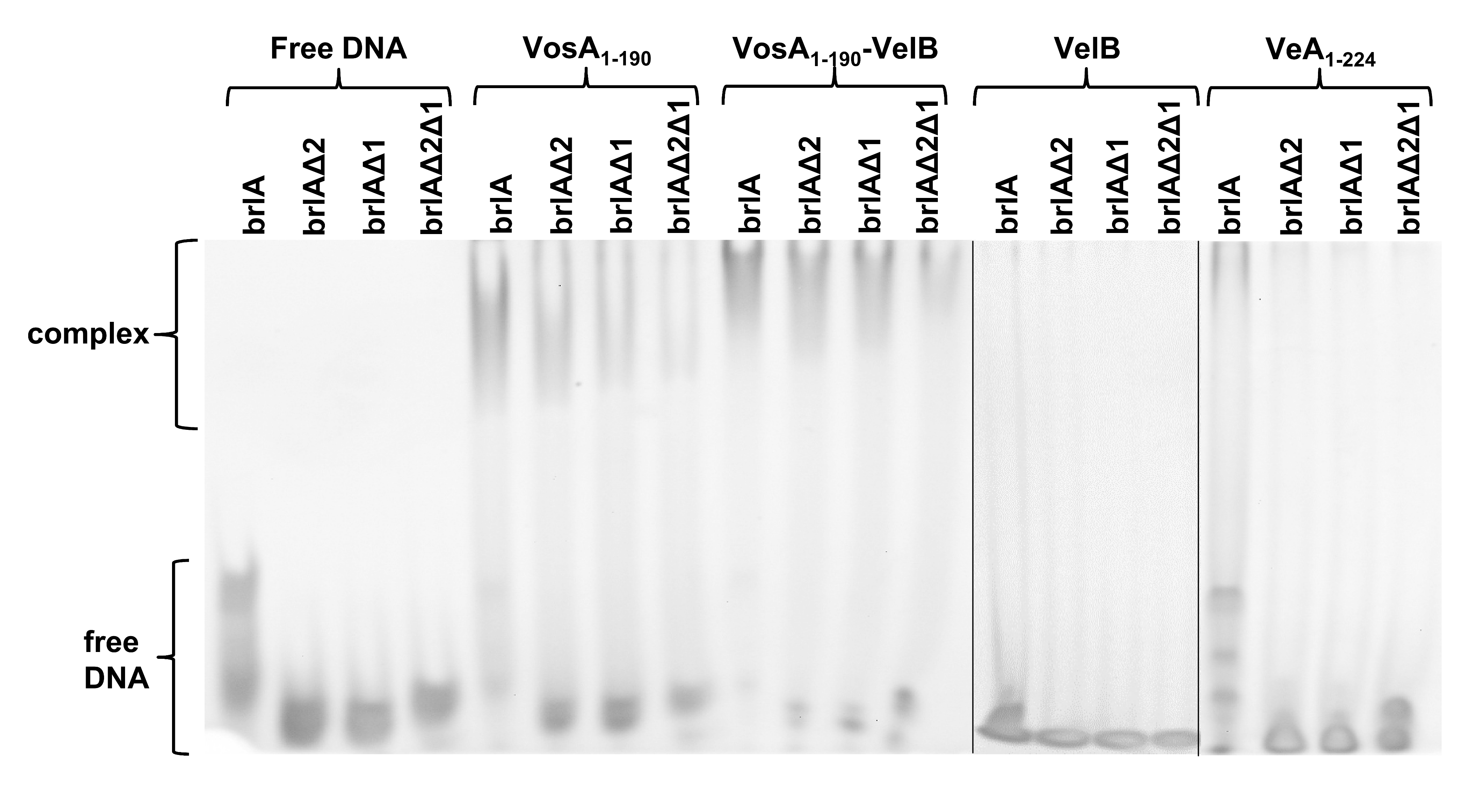

Supplement: Figure S5 — EMSA using VosA1–190, the VosA1–190-VelB heterodimer, VelB, and VeA1–224 with the wild-type and mutated DNA probes of the brlA promoter. In the mutated versions of the probe, the predicted VosA binding motifs 1 and 2 were deleted (brlA, wild-type DNA (OHS301/302); brlAΔ2, DNA with deleted motif 2 (JG636/637); brlAΔ1, DNA with deleted motif 1 (JG638/639); brlAΔ2Δ1, DNA with deleted motifs 2 and 1(JG640/641)). DNA and protein were used in the molar ratio 1∶3. Free DNA without protein was used as negative control. (TIF) [file pbio.1001750.s005.tif]

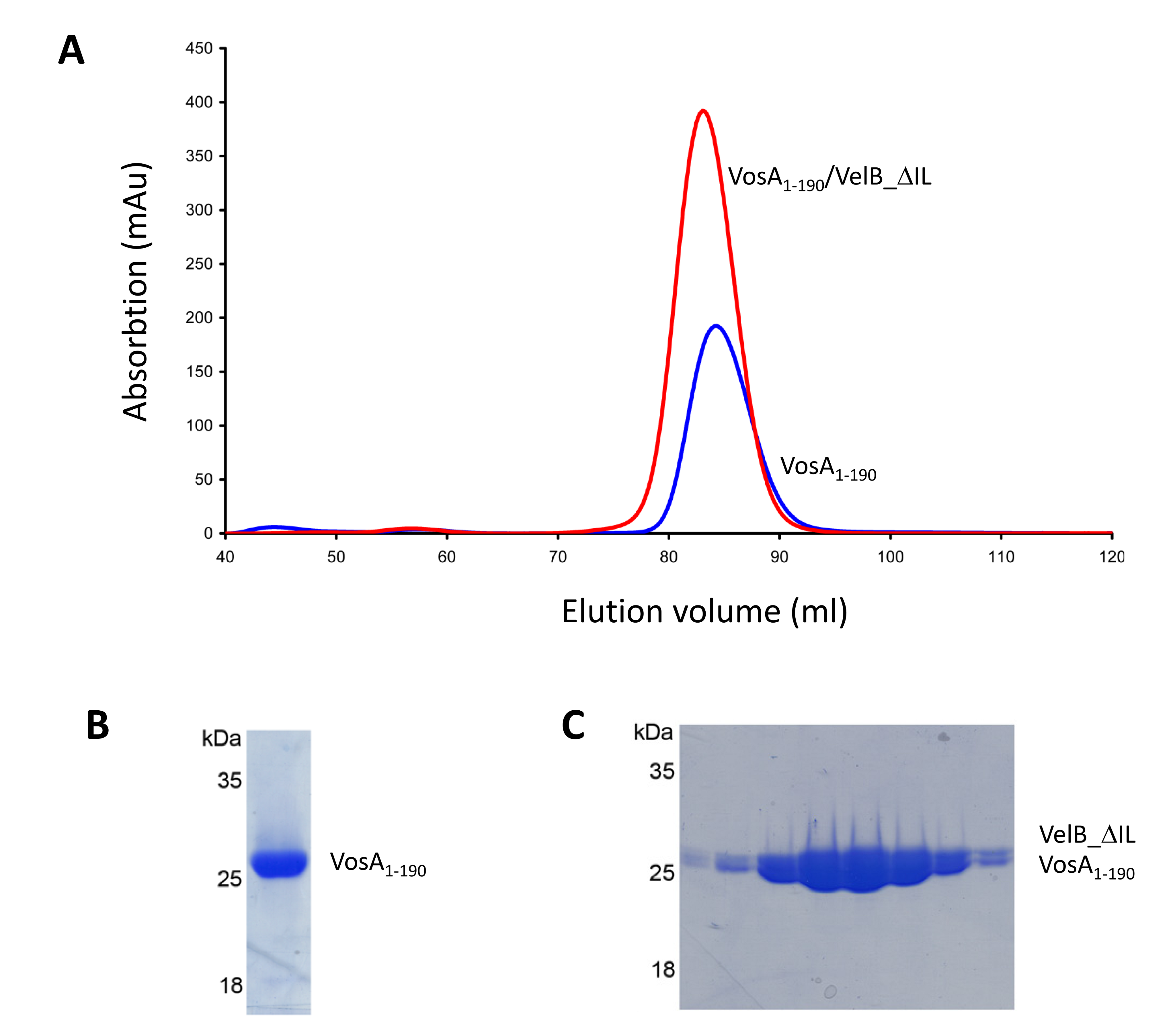

Supplement: Figure S6 — Size exclusion chromatography of VosA1–190 and VosA1–190-VelB_ΔIL complex. VelB_ΔIL is a VelB construct lacking the amino acid insertion within the velvet domain (for cloning, expression, and purification, see Text S1). (A) Superposition of the elution profiles of VosA1–190 and of the VosA1–190-VelB_ΔIL complex on a Superdex 200 (16/60) gelfiltration chromatography column. The elution volumes indicate the presence of a homodimeric VosA1–190 and heterodimeric VosA1–190-VelB_ΔIL, respectively. (B) The SDS PAGE of the peak fraction of VosA1–190 and (C) of all peak fractions of VosA1–190-VelB_ΔIL complex. (TIF) [file pbio.1001750.s006.tif]

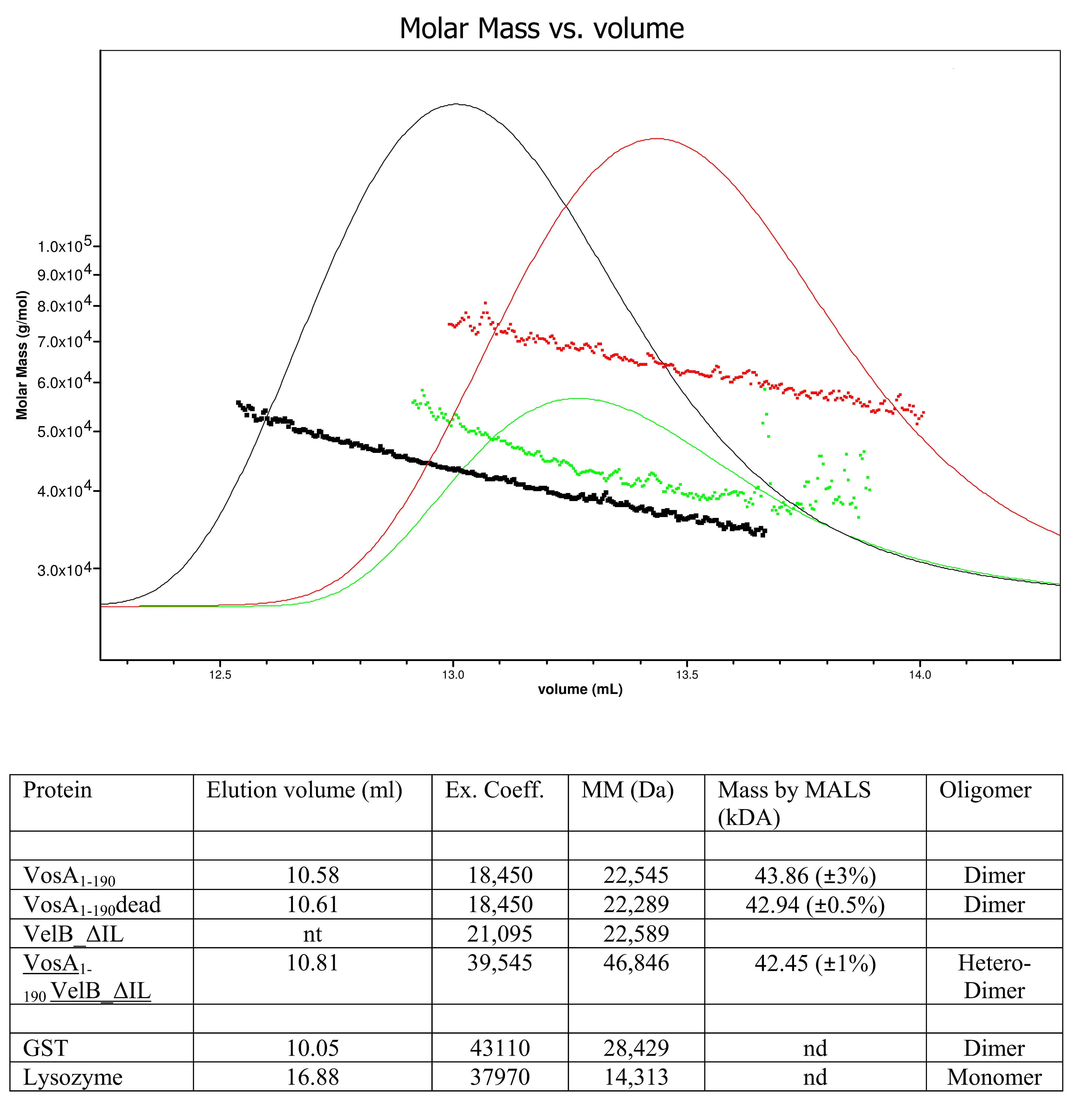

Supplement: Figure S7 — VosA1–190 forms dimers with itself and the VelB_ΔIL mutant as shown by size exclusion chromatography and subsequent mass determination using MALS. Dimerization is independent of mutations in the loop region required for DNA binding. VosA1–190 is shown in green; VosA1–190 dead, quadruple mutant of VosA with the mutations K37A, K39A, R41A, and K42A is shown in black. VosA1–190 and VelB_ΔIL is shown in red. VelB_ΔIL, residues 44–126 and 240–343 connected by a short linker (see also Text S1). (TIF) [file pbio.1001750.s007.tif]

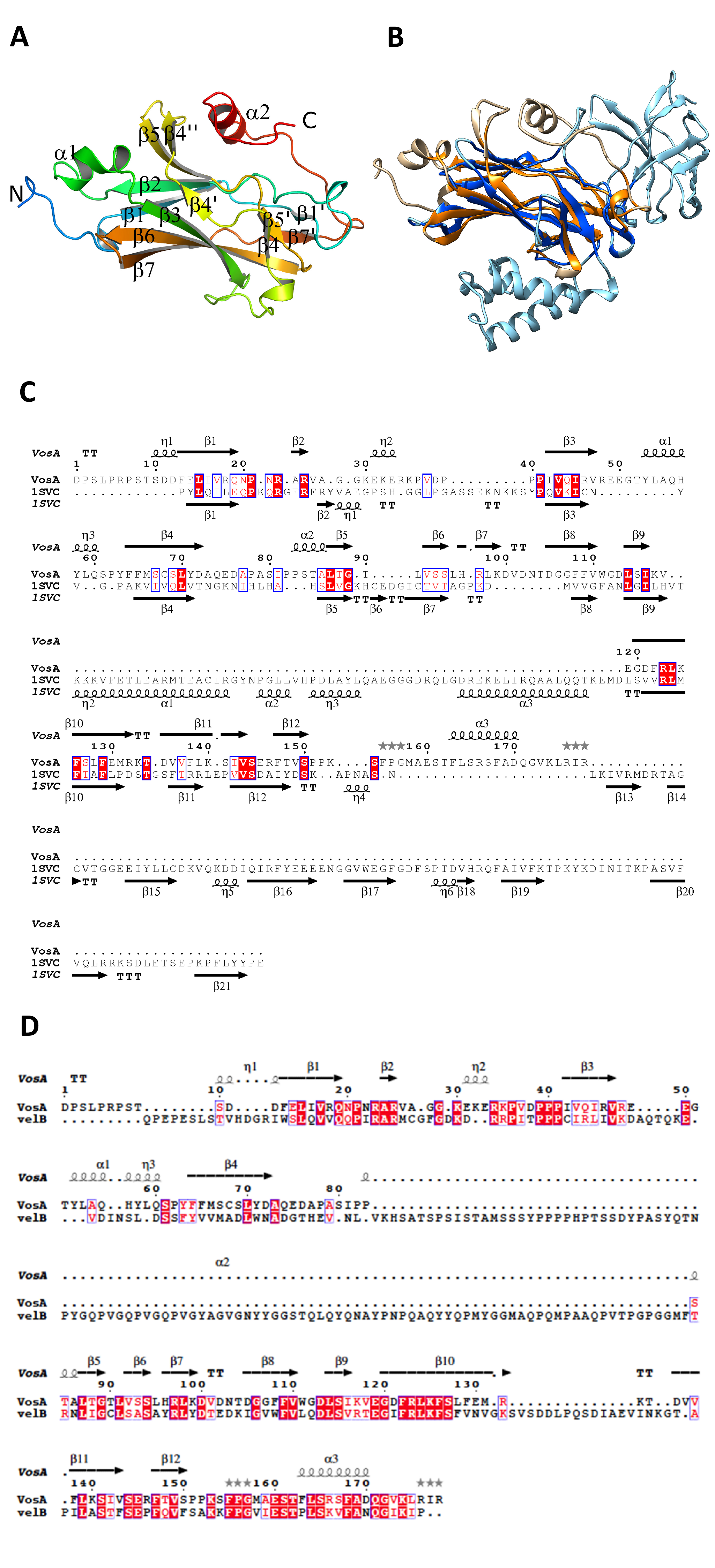

Supplement: Figure S8 — Structure-based sequence alignment of the crystallized VosA fragment with NFκB (PDB ID code 1SVC) and VelB reveals a high structural similarity in the canonical fold region but differences in loop regions and the C-terminal region. (A) Overall structure of VosA1–190 in rainbow colouring (from N-terminus (blue) to C-terminus (red)) with the individual secondary structure motifs depicted in cartoon mode and labelled. (B) Superposition of VosA (orange colours) and NF-κB (blue colours) structures. (C) Structure-based sequence alignment of VosA (top) and NF-κB (PDB ID code 1SVC; bottom). (D) Structure-based sequence alignment of VosA (top) and VelB (bottom). (TIF) [file pbio.1001750.s008.tif]

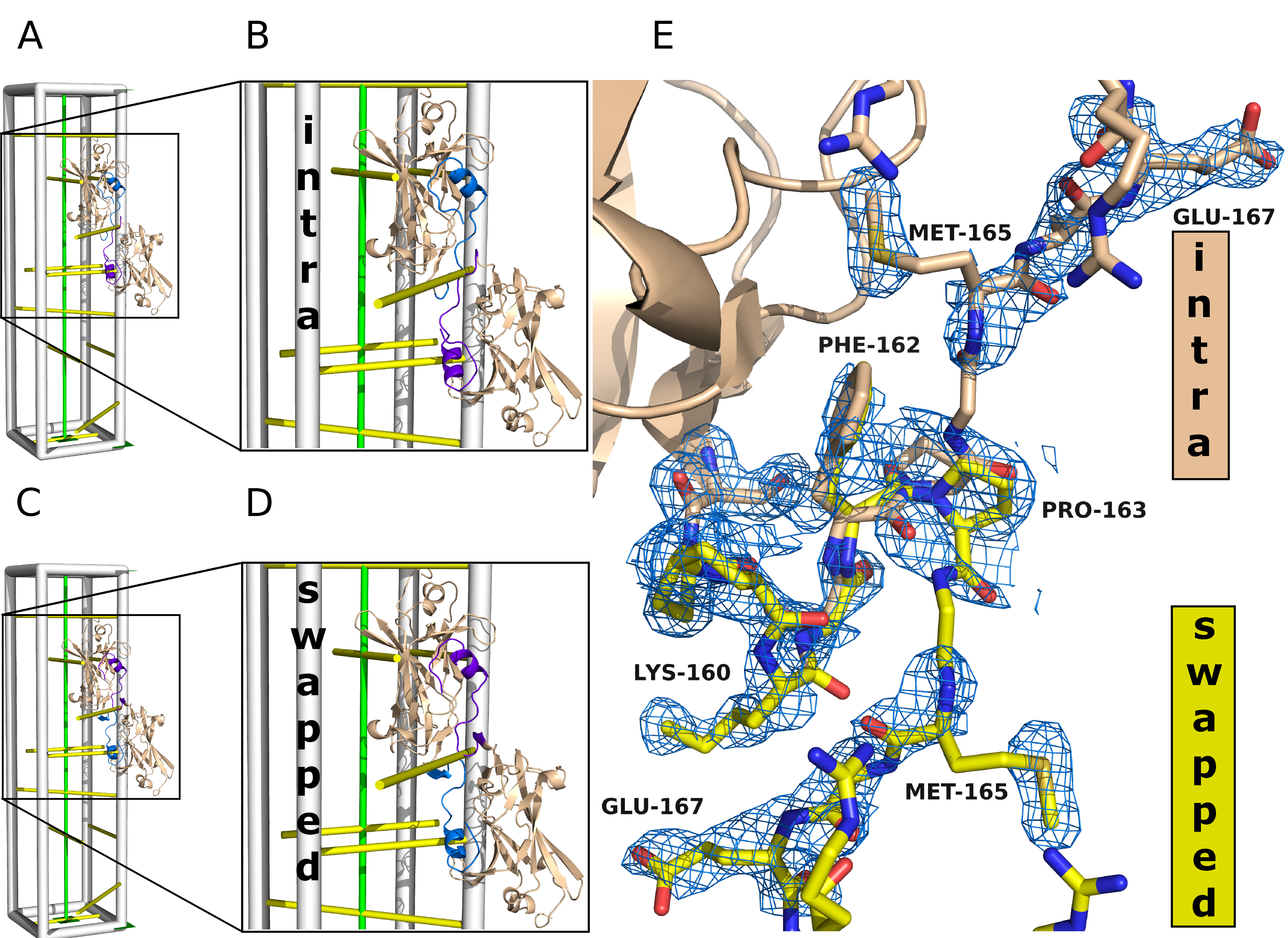

Supplement: Figure S9 — Arrangement of VosA1–190 molecules around 2-fold crystallographic axis in the crystal lattice. (A, B, C, D) Two possible conformations of a loop resulting in intermolecular (A, B) or swapped (C, D) placement of the C-terminal α-helix are shown. (E) The Simulated Annealing omit mFo-DFc electron density map contoured at 3σ level is consistent with two alternate conformations of the C-terminal helix of VosA1–190. The C-terminally located helix of VosA1–190 is found in two conformations influencing the arrangement of LoopB, and thus its DNA binding capabilities. The missing part of VosA might lead to an overall conformation of VosA similar to the one observed for NFκB increasing the effect of LoopB on DNA binding. The additional residues present in VosA-N might therefore explain its increased DNA binding. (TIF) [file pbio.1001750.s009.tif]

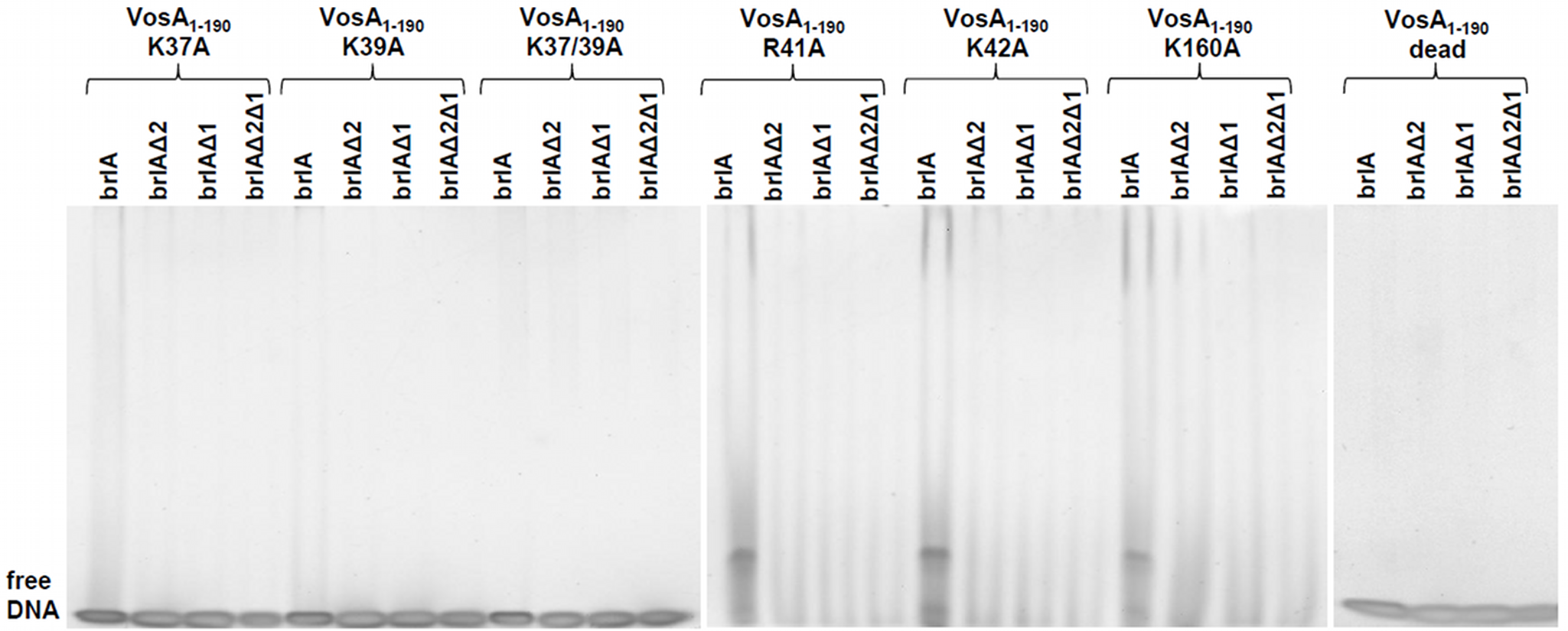

Supplement: Figure S10 — EMSA using the mutated VosA proteins with the wild-type and mutated DNA probes of the brlA promoter. In the mutated versions of the DNA, the predicted VosA binding motifs 1 and 2 were deleted (brlA, wild-type DNA (OHS301/302); brlAΔ2, DNA with deleted motif 2 (JG636/637); brlAΔ1, DNA with deleted motif 1 (JG638/639); brlAΔ2Δ1, DNA with deleted motifs 2 and 1(JG640/641)). DNA and protein were used in the molar ratio 1∶9. (TIF) [file pbio.1001750.s010.tif]

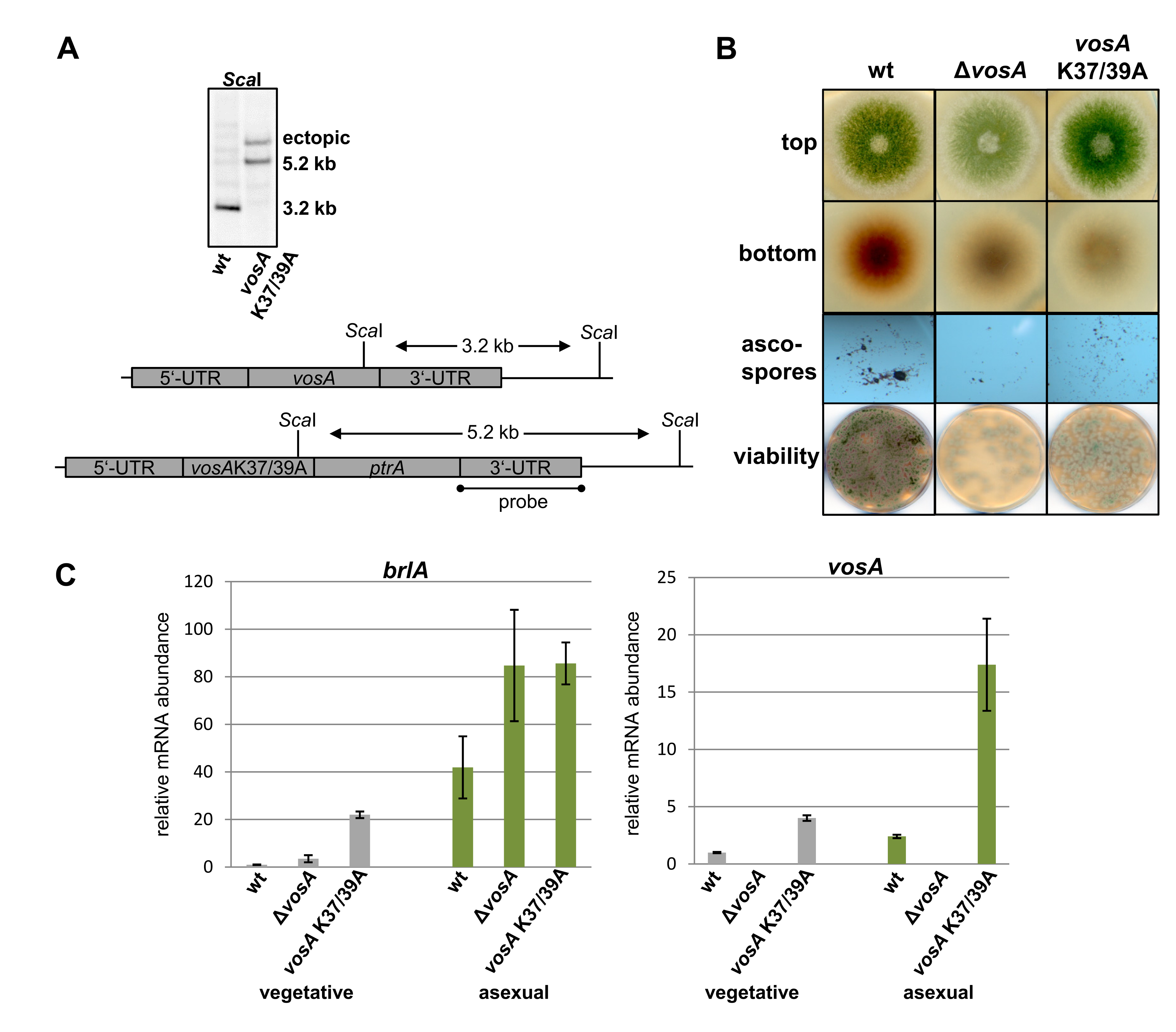

Supplement: Figure S11 — In vivo analysis of the VosA K37/39A protein mutations. (A) Southern hybridization to verify the integration of the transformation cassette. The cassette was integrated twice, at the vosA and unknown loci. (B) Phenotypic analysis of the A. nidulans vosAK37/39A mutant compared to the wild-type (wt) and the vosA deletion (ΔvosA) mutant. For asexual development, strains were grown 3 d on minimal medium in light at 37°C. For sexual development, strains were cultivated 7 d in darkness at 37°C. The fruiting bodies were squeezed in water to liberate the red ascospores. Additionally, a viability test was performed. Each strain was cultivated 10 d at 37°C and 250 of these old spores were subsequently grown 2 d at 37°C to compare the amount of surviving colonies. (C) Relative mRNA levels of wild-type, ΔvosA, and vosAK37/39A measured by quantitative real-time PCR with the primers specific for brlA and vosA. Strains were cultivated vegetative and asexually. Note that the vosAK37/39A mutant allele fails to down-regulate brlA properly. (TIF) [file pbio.1001750.s011.tif]

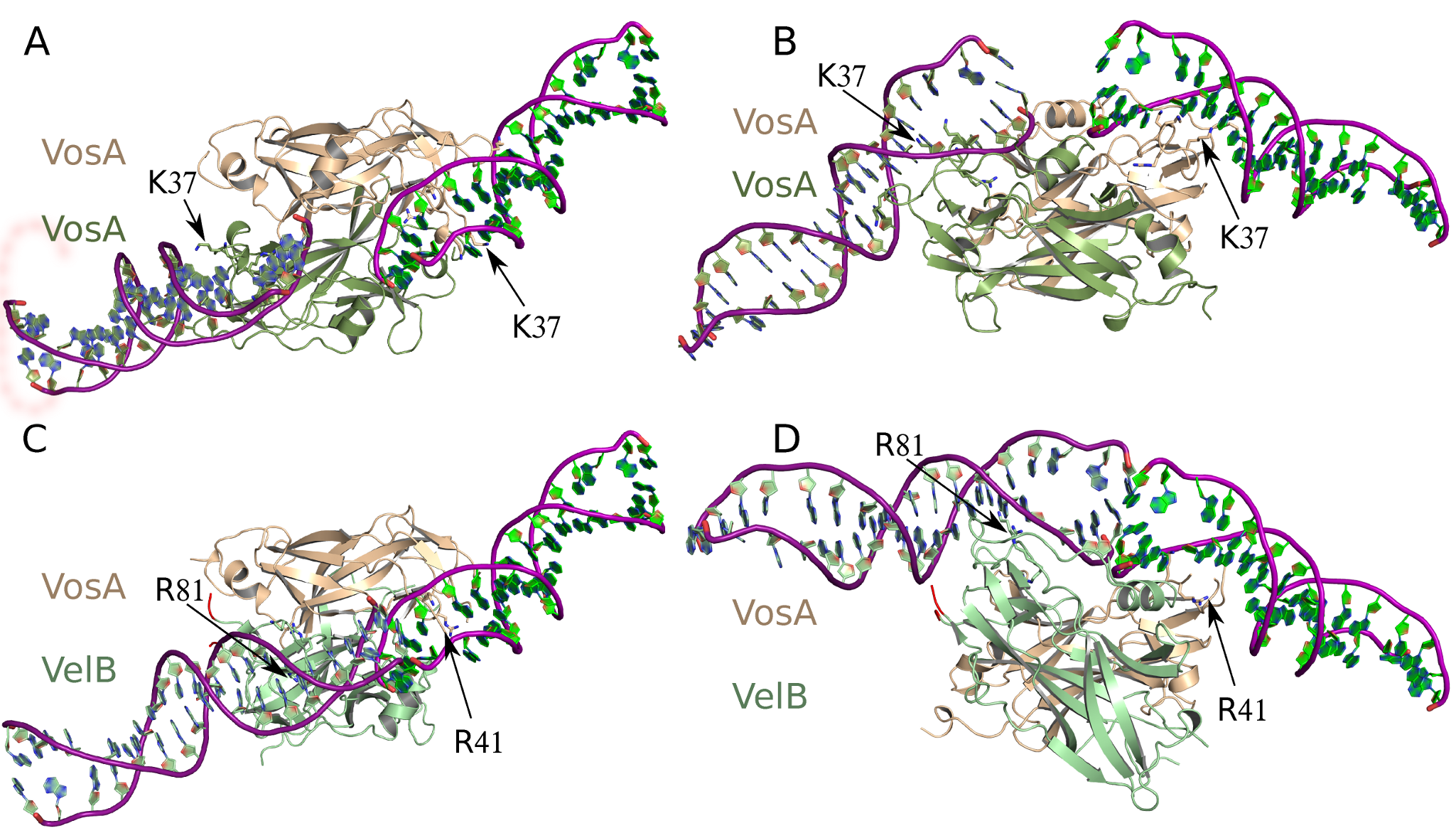

Supplement: Figure S12 — Superposition-based modelling of DNA (from the NFκB complex structure PDB ID code 1SVC) on the VosA1–190 homodimer (A, B) and VosA1–190-VelB heterodimer (C, D) and its spatial arrangement. The overall arrangement of the protein dimers suggests differences in DNA conformation for binding. Whereas the VosA1–190 homodimer could bind on the more or less straight superhelical DNA (A and 90° rotation B), the arrangement of the DNA has to be more kinked for a good fit. (TIF) [file pbio.1001750.s012.tif]

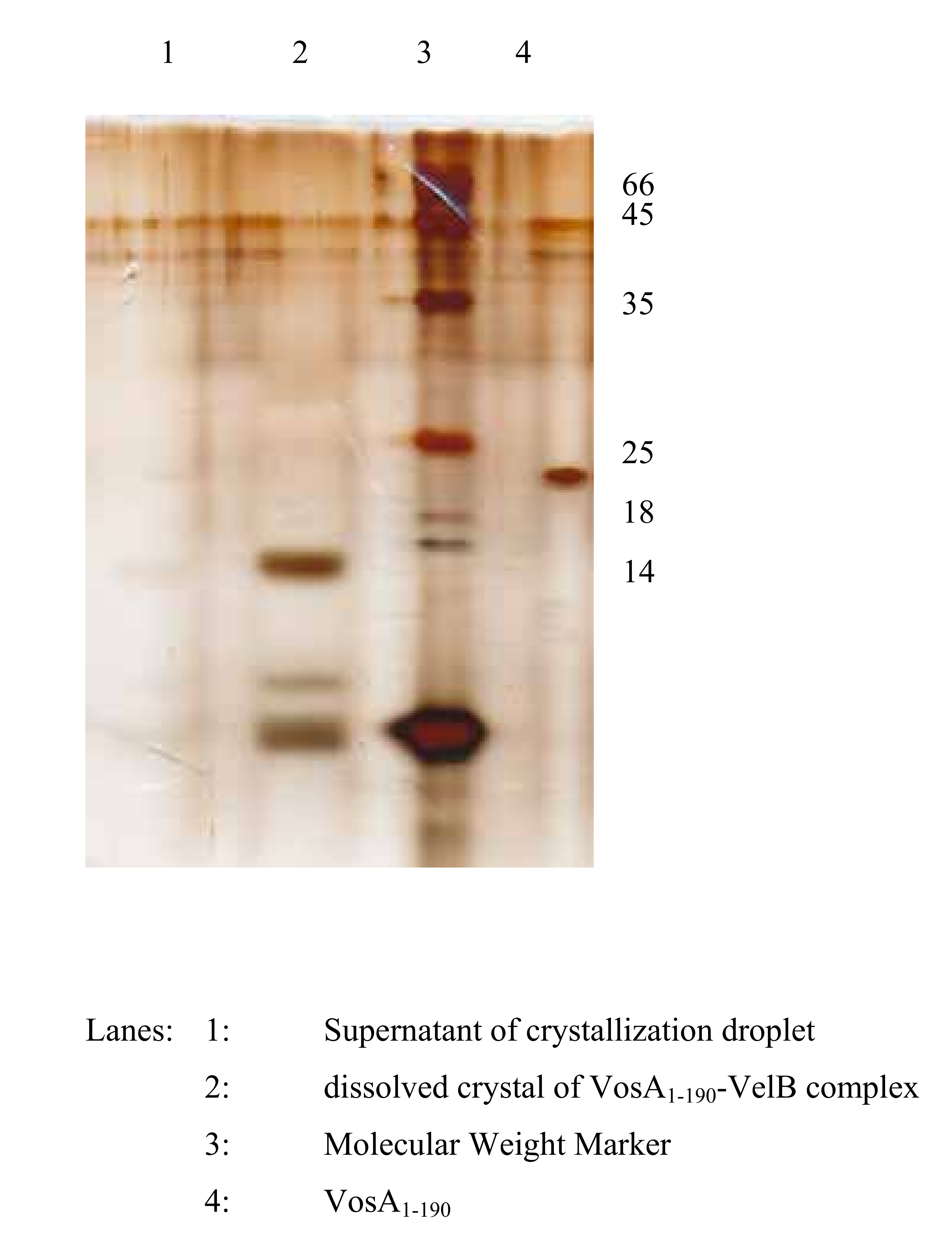

Supplement: Figure S13 — Proteolytic degradation of VelB. Analysis of VosA1–190-VelB crystals by silver stained SDS_PAGE (17.5%) gel reveals proteolytic digest of the proteins present in the crystals. (TIF) [file pbio.1001750.s013.tif]

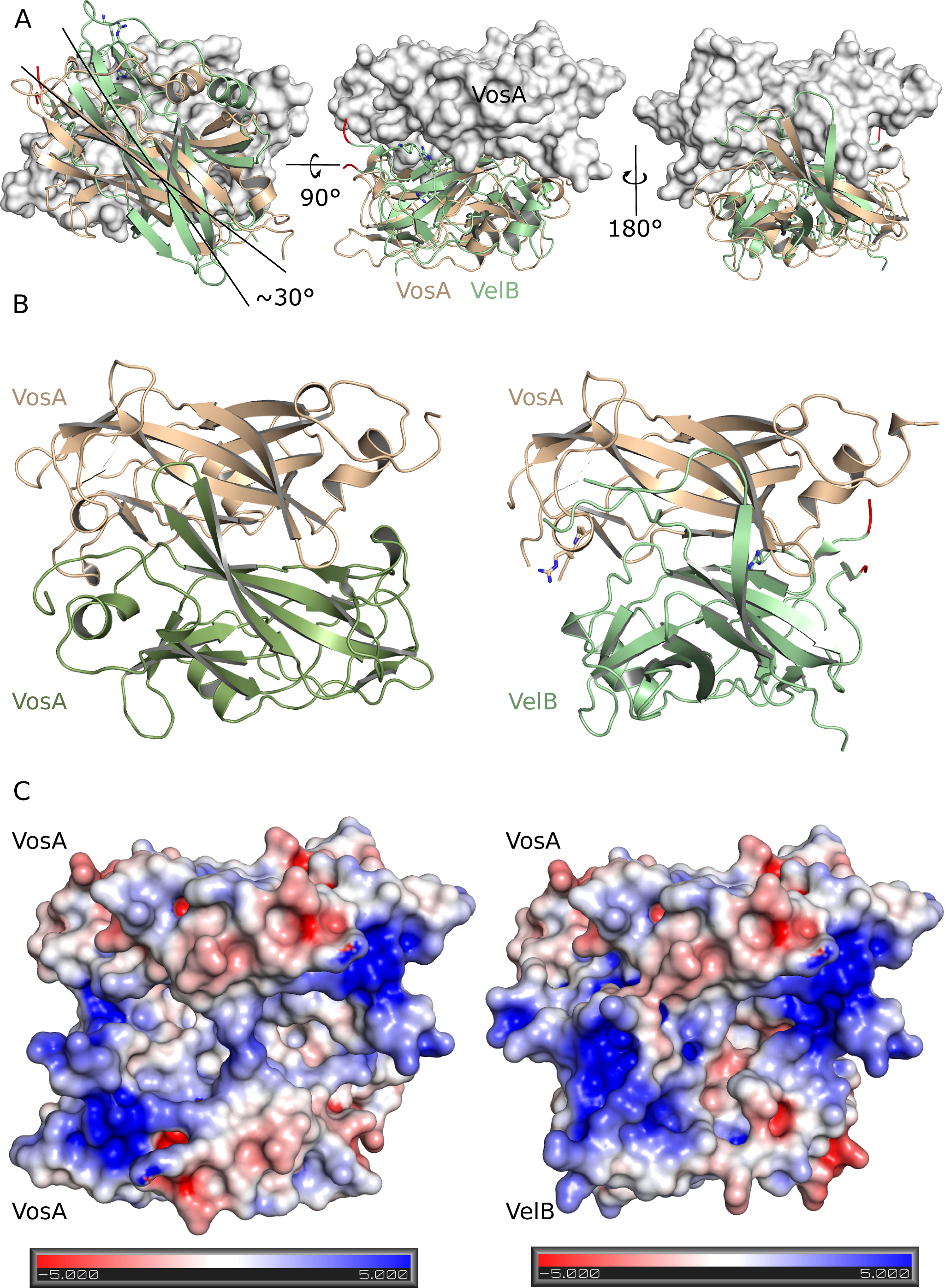

Supplement: Figure S14 — Comparison of the VosA1–190 homodimer and the VosA1–190-VelB heterodimer. (A) The dimers have been superimposed with the VosA subunit shown as a white surface representation. The other subunit of the dimer is presented as the ribbon plot. The VelB subunit binds to VosA1–190 via the same surface, but the VelB subunit is rotated by 30° and shifted by ∼3 Å with respect to the second VosA1–190 molecule of the homodimer. (B) Dimerization results in formation of an intermolecular eight-stranded β-sandwitch (orientation similar to A, the right panel). (C) Electrostatic surface representation (orientation as on A, middle panel). Due to missing residues of VosA1–190 (37–39, 161–190) in VosA1–190-VelB complex, complete VosA1–190 monomer has been superimposed and used for figure generation. Surface representation coloured according to the electrostatic potential ranging from red (−5 kBT/e) through white (0 kBT/e) to blue (+5 kBT/e). (TIF) [file pbio.1001750.s014.tif]

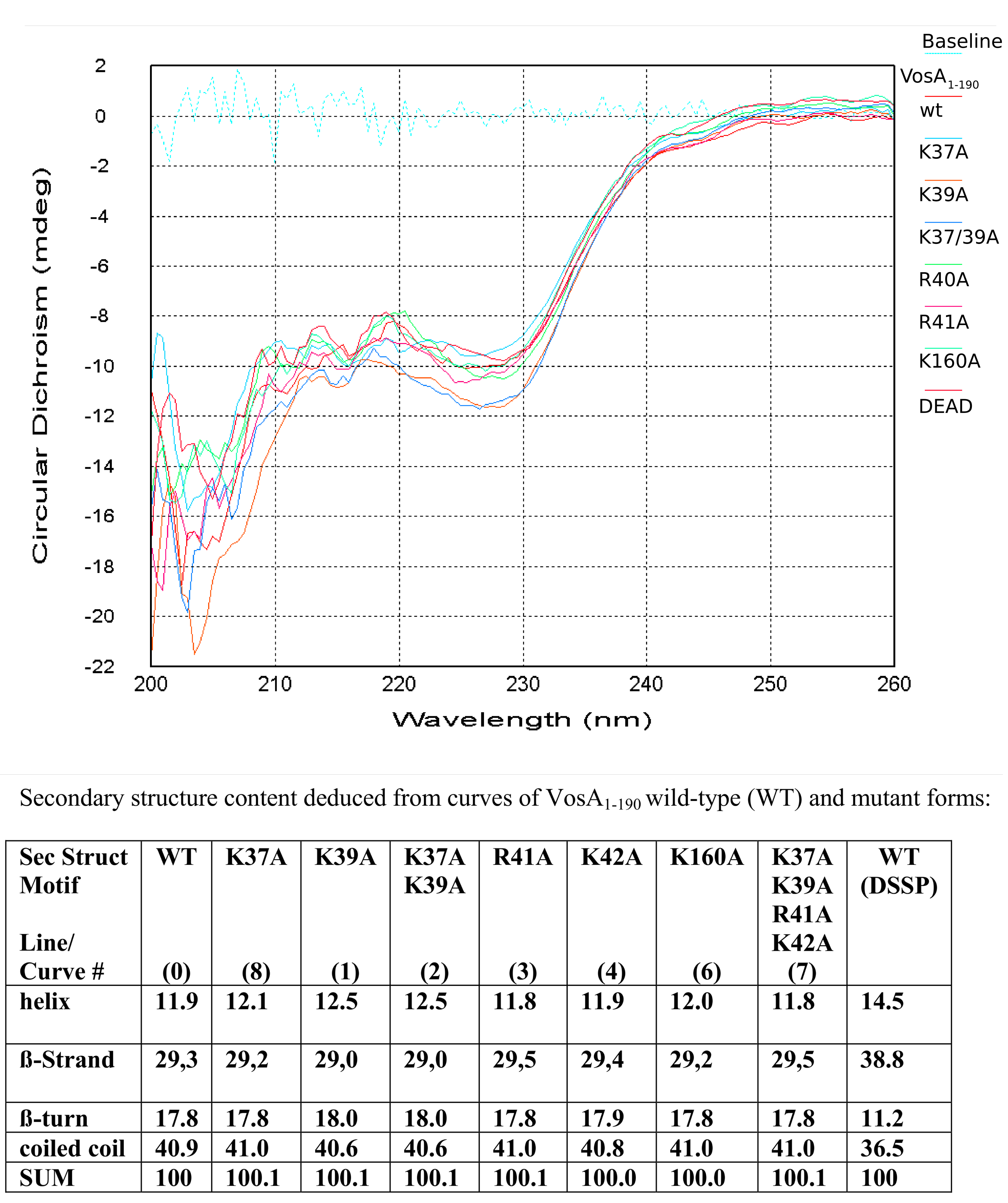

Supplement: Figure S15 — VosA1–190 exhibits identical curves in CD spectroscopy independent of the individual or multiple point mutations in the putative DNA binding loops. The secondary structure content deduced from these curves as described in Materials and Methods indicates no significant differences in secondary structure content of the individual mutant forms of VosA1–190. The last column shows the values for the secondary structure elements deduced from the crystal structure as determined by DSSP. (TIF) [file pbio.1001750.s015.tif]
