# Supplementary material for: The Velvet Family of Fungal Regulators Contains a DNA-Binding Domain Structurally Similar to NF-κB
Source: PLoS Biol. 2013 Dec 31;11(12):e1001750. doi: 10.1371/journal.pbio.1001750 (PMC3876986; doi:10.1371/journal.pbio.1001750)
Supplement: Table S1 — Raw data of the ChIP-on-chip and ChIP-PCR experiments. (PDF) [file pbio.1001750.s016.pdf]

PEAK_ID	CHROMOSOME	PEAK_START	PEAK_END	PEAK_SCORE	PEAK_FDR	FEATURE_TRACK	FEATURE_STRAND	FEATURE_START	FEATURE_END	FEATURE_TO_PEAK_DISTANCE	Parent	accession
2277	CONTIG2	25126	25551	1.27	4.36E-02	transcription_start_site	-	22026	22026	-3312	14	AN0009
2277	CONTIG2	25126	25551	1.27	4.36E-02	transcription_start_site	+	24566	24566	772	18	AN0010
2277	CONTIG2	25126	25551	1.27	4.36E-02	transcription_start_site	+	24138	24138	1200	17	AN0010
2277	CONTIG2	25126	25551	1.27	4.36E-02	transcription_start_site	+	26209	26209	-870	19	AN0011
2277	CONTIG2	25126	25551	1.27	4.36E-02	transcription_start_site	+	26620	26620	-1281	20	AN0011
2277	CONTIG2	25126	25551	1.27	4.36E-02	transcription_start_site	+	26757	26757	-1418	21	AN0011
2277	CONTIG2	25126	25551	1.27	4.36E-02	transcription_start_site	+	27319	27319	-1980	22	AN0011
590	CONTIG2	74042	74303	2.32	0.00E+00	transcription_start_site	-	73218	73218	-954	50	AN0017
590	CONTIG2	74042	74303	2.32	0.00E+00	transcription_start_site	-	73024	73024	-1148	51	AN0017
1286	CONTIG2	75677	76037	1.7	5.20E-03	transcription_start_site	-	73218	73218	-2639	50	AN0017
1286	CONTIG2	75677	76037	1.7	5.20E-03	transcription_start_site	-	73024	73024	-2833	51	AN0017
2068	CONTIG2	74717	75291	1.34	3.08E-02	transcription_start_site	-	73218	73218	-1786	50	AN0017
2068	CONTIG2	74717	75291	1.34	3.08E-02	transcription_start_site	-	73024	73024	-1980	51	AN0017
590	CONTIG2	74042	74303	2.32	0.00E+00	transcription_start_site	+	73927	73927	245	52	AN0018
590	CONTIG2	74042	74303	2.32	0.00E+00	transcription_start_site	+	74648	74648	-475	53	AN0018
590	CONTIG2	74042	74303	2.32	0.00E+00	transcription_start_site	+	75130	75130	-957	54	AN0018
1286	CONTIG2	75677	76037	1.7	5.20E-03	transcription_start_site	+	75130	75130	727	54	AN0018
2068	CONTIG2	74717	75291	1.34	3.08E-02	transcription_start_site	+	75130	75130	-126	54	AN0018
2068	CONTIG2	74717	75291	1.34	3.08E-02	transcription_start_site	+	74648	74648	356	53	AN0018
2068	CONTIG2	74717	75291	1.34	3.08E-02	transcription_start_site	+	73927	73927	1077	52	AN0018
590	CONTIG2	74042	74303	2.32	0.00E+00	transcription_start_site	+	76862	76862	-2689	55	AN0019
590	CONTIG2	74042	74303	2.32	0.00E+00	transcription_start_site	+	77018	77018	-2845	56	AN0019
590	CONTIG2	74042	74303	2.32	0.00E+00	transcription_start_site	+	77350	77350	-3177	57	AN0019
1286	CONTIG2	75677	76037	1.7	5.20E-03	transcription_start_site	+	76862	76862	-1005	55	AN0019
1286	CONTIG2	75677	76037	1.7	5.20E-03	transcription_start_site	+	77018	77018	-1161	56	AN0019
1286	CONTIG2	75677	76037	1.7	5.20E-03	transcription_start_site	+	77350	77350	-1493	57	AN0019
2068	CONTIG2	74717	75291	1.34	3.08E-02	transcription_start_site	+	76862	76862	-1858	55	AN0019
2068	CONTIG2	74717	75291	1.34	3.08E-02	transcription_start_site	+	77018	77018	-2014	56	AN0019
2068	CONTIG2	74717	75291	1.34	3.08E-02	transcription_start_site	+	77350	77350	-2346	57	AN0019
1969	CONTIG2	93984	94418	1.38	2.62E-02	transcription_start_site	-	90373	90373	-3828	73	AN0024
1969	CONTIG2	93984	94418	1.38	2.62E-02	transcription_start_site	-	90259	90259	-3942	74	AN0024
1969	CONTIG2	93984	94418	1.38	2.62E-02	transcription_start_site	-	90119	90119	-4082	75	AN0024
1969	CONTIG2	93984	94418	1.38	2.62E-02	transcription_start_site	-	89998	89998	-4203	76	AN0024
1969	CONTIG2	93984	94418	1.38	2.62E-02	transcription_start_site	-	89867	89867	-4334	77	AN0024
1969	CONTIG2	93984	94418	1.38	2.62E-02	transcription_start_site	-	89566	89566	-4635	78	AN0024
2975	CONTIG2	92719	93653	0.94	1.50E-01	transcription_start_site	-	90373	90373	-2813	73	AN0024
2975	CONTIG2	92719	93653	0.94	1.50E-01	transcription_start_site	-	90259	90259	-2927	74	AN0024
2975	CONTIG2	92719	93653	0.94	1.50E-01	transcription_start_site	-	90119	90119	-3067	75	AN0024
2975	CONTIG2	92719	93653	0.94	1.50E-01	transcription_start_site	-	89998	89998	-3188	76	AN0024
2975	CONTIG2	92719	93653	0.94	1.50E-01	transcription_start_site	-	89867	89867	-3319	77	AN0024
2975	CONTIG2	92719	93653	0.94	1.50E-01	transcription_start_site	-	89566	89566	-3620	78	AN0024
1969	CONTIG2	93984	94418	1.38	2.62E-02	transcription_start_site	-	92486	92486	-1715	79	AN0025
1969	CONTIG2	93984	94418	1.38	2.62E-02	transcription_start_site	-	92063	92063	-2138	80	AN0025
1969	CONTIG2	93984	94418	1.38	2.62E-02	transcription_start_site	-	91376	91376	-2825	81	AN0025
2975	CONTIG2	92719	93653	0.94	1.50E-01	transcription_start_site	-	92486	92486	-700	79	AN0025
2975	CONTIG2	92719	93653	0.94	1.50E-01	transcription_start_site	-	92063	92063	-1123	80	AN0025
2975	CONTIG2	92719	93653	0.94	1.50E-01	transcription_start_site	-	91376	91376	-1810	81	AN0025
1969	CONTIG2	93984	94418	1.38	2.62E-02	transcription_start_site	+	93155	93155	1046	83	AN0026
1969	CONTIG2	93984	94418	1.38	2.62E-02	transcription_start_site	+	93000	93000	1201	82	AN0026
2975	CONTIG2	92719	93653	0.94	1.50E-01	transcription_start_site	+	93155	93155	31	83	AN0026
2975	CONTIG2	92719	93653	0.94	1.50E-01	transcription_start_site	+	93000	93000	186	82	AN0026
1969	CONTIG2	93984	94418	1.38	2.62E-02	transcription_start_site	+	99025	99025	-4824	89	AN0029
1969	CONTIG2	93984	94418	1.38	2.62E-02	transcription_start_site	+	99354	99354	-5153	90	AN0029
1120	CONTIG2	115512	115781	1.81	3.03E-03	transcription_start_site	-	110851	110851	-4795	97	AN0032
1120	CONTIG2	115512	115781	1.81	3.03E-03	transcription_start_site	-	110657	110657	-4989	98	AN0032
2702	CONTIG2	111453	111892	1.12	8.01E-02	transcription_start_site	-	110851	110851	-821	97	AN0032

2702	CONTIG2	111453	111892	1.12	8.01E-02	transcription_start_site	-	110657	110657	-1015	98	AN0032
2702	CONTIG2	111453	111892	1.12	8.01E-02	transcription_start_site	-	110462	110462	-1210	99	AN0032
2702	CONTIG2	111453	111892	1.12	8.01E-02	transcription_start_site	+	111443	111443	229	101	AN0033
2702	CONTIG2	111453	111892	1.12	8.01E-02	transcription_start_site	+	111270	111270	402	100	AN0033
911	CONTIG2	116782	117141	1.99	1.01E-03	transcription_start_site	-	115420	115420	-1541	102	AN0034
911	CONTIG2	116782	117141	1.99	1.01E-03	transcription_start_site	-	115263	115263	-1698	103	AN0034
911	CONTIG2	116782	117141	1.99	1.01E-03	transcription_start_site	-	114707	114707	-2254	104	AN0034
911	CONTIG2	116782	117141	1.99	1.01E-03	transcription_start_site	-	114035	114035	-2926	105	AN0034
1120	CONTIG2	115512	115781	1.81	3.03E-03	transcription_start_site	-	115420	115420	-226	102	AN0034
1120	CONTIG2	115512	115781	1.81	3.03E-03	transcription_start_site	-	115263	115263	-383	103	AN0034
1120	CONTIG2	115512	115781	1.81	3.03E-03	transcription_start_site	-	114707	114707	-939	104	AN0034
1120	CONTIG2	115512	115781	1.81	3.03E-03	transcription_start_site	-	114035	114035	-1611	105	AN0034
1226	CONTIG2	118292	118851	1.74	1.22E-03	transcription_start_site	-	115420	115420	-3151	102	AN0034
1226	CONTIG2	118292	118851	1.74	1.22E-03	transcription_start_site	-	115263	115263	-3308	103	AN0034
1226	CONTIG2	118292	118851	1.74	1.22E-03	transcription_start_site	-	114707	114707	-3864	104	AN0034
1226	CONTIG2	118292	118851	1.74	1.22E-03	transcription_start_site	-	114035	114035	-4536	105	AN0034
1970	CONTIG2	117232	117816	1.38	2.62E-02	transcription_start_site	-	115420	115420	-2104	102	AN0034
1970	CONTIG2	117232	117816	1.38	2.62E-02	transcription_start_site	-	115263	115263	-2261	103	AN0034
1970	CONTIG2	117232	117816	1.38	2.62E-02	transcription_start_site	-	114707	114707	-2817	104	AN0034
1970	CONTIG2	117232	117816	1.38	2.62E-02	transcription_start_site	-	114035	114035	-3489	105	AN0034
911	CONTIG2	116782	117141	1.99	1.01E-03	transcription_start_site	+	118643	118643	-1681	106	AN0035
911	CONTIG2	116782	117141	1.99	1.01E-03	transcription_start_site	+	119220	119220	-2258	107	AN0035
911	CONTIG2	116782	117141	1.99	1.01E-03	transcription_start_site	+	119381	119381	-2419	108	AN0035
911	CONTIG2	116782	117141	1.99	1.01E-03	transcription_start_site	+	119861	119861	-2899	109	AN0035
911	CONTIG2	116782	117141	1.99	1.01E-03	transcription_start_site	+	120281	120281	-3319	110	AN0035
1120	CONTIG2	115512	115781	1.81	3.03E-03	transcription_start_site	+	118643	118643	-2996	106	AN0035
1120	CONTIG2	115512	115781	1.81	3.03E-03	transcription_start_site	+	119220	119220	-3573	107	AN0035
1120	CONTIG2	115512	115781	1.81	3.03E-03	transcription_start_site	+	119381	119381	-3734	108	AN0035
1120	CONTIG2	115512	115781	1.81	3.03E-03	transcription_start_site	+	119861	119861	-4214	109	AN0035
1120	CONTIG2	115512	115781	1.81	3.03E-03	transcription_start_site	+	120281	120281	-4634	110	AN0035
1226	CONTIG2	118292	118851	1.74	1.22E-03	transcription_start_site	+	118643	118643	-71	106	AN0035
1226	CONTIG2	118292	118851	1.74	1.22E-03	transcription_start_site	+	119220	119220	-648	107	AN0035
1226	CONTIG2	118292	118851	1.74	1.22E-03	transcription_start_site	+	119381	119381	-809	108	AN0035
1226	CONTIG2	118292	118851	1.74	1.22E-03	transcription_start_site	+	119861	119861	-1289	109	AN0035
1226	CONTIG2	118292	118851	1.74	1.22E-03	transcription_start_site	+	120281	120281	-1709	110	AN0035
1970	CONTIG2	117232	117816	1.38	2.62E-02	transcription_start_site	+	118643	118643	-1119	106	AN0035
1970	CONTIG2	117232	117816	1.38	2.62E-02	transcription_start_site	+	119220	119220	-1696	107	AN0035
1970	CONTIG2	117232	117816	1.38	2.62E-02	transcription_start_site	+	119381	119381	-1857	108	AN0035
1970	CONTIG2	117232	117816	1.38	2.62E-02	transcription_start_site	+	119861	119861	-2337	109	AN0035
1970	CONTIG2	117232	117816	1.38	2.62E-02	transcription_start_site	+	120281	120281	-2757	110	AN0035
911	CONTIG2	116782	117141	1.99	1.01E-03	transcription_start_site	+	121024	121024	-4062	111	AN0036
911	CONTIG2	116782	117141	1.99	1.01E-03	transcription_start_site	+	121123	121123	-4161	112	AN0036
1226	CONTIG2	118292	118851	1.74	1.22E-03	transcription_start_site	+	121024	121024	-2452	111	AN0036
1226	CONTIG2	118292	118851	1.74	1.22E-03	transcription_start_site	+	121123	121123	-2551	112	AN0036
1970	CONTIG2	117232	117816	1.38	2.62E-02	transcription_start_site	+	121024	121024	-3500	111	AN0036
1970	CONTIG2	117232	117816	1.38	2.62E-02	transcription_start_site	+	121123	121123	-3599	112	AN0036
2870	CONTIG2	156545	157104	1.02	1.07E-01	transcription_start_site	-	151883	151883	-4941	133	AN0043
2870	CONTIG2	156545	157104	1.02	1.07E-01	transcription_start_site	-	151627	151627	-5197	134	AN0043
2871	CONTIG2	155030	155389	1.02	1.25E-01	transcription_start_site	-	151883	151883	-3326	133	AN0043
2871	CONTIG2	155030	155389	1.02	1.25E-01	transcription_start_site	-	151627	151627	-3582	134	AN0043
2870	CONTIG2	156545	157104	1.02	1.07E-01	transcription_start_site	-	157153	157153	328	137	AN0045
2870	CONTIG2	156545	157104	1.02	1.07E-01	transcription_start_site	-	157406	157406	581	136	AN0045
3008	CONTIG2	157370	158004	0.91	1.79E-01	transcription_start_site	-	157406	157406	-281	136	AN0045
3008	CONTIG2	157370	158004	0.91	1.79E-01	transcription_start_site	-	157153	157153	-534	137	AN0045
3008	CONTIG2	157370	158004	0.91	1.79E-01	transcription_start_site	-	158769	158769	1082	142	AN0046
2906	CONTIG3	15463	15812	1	1.93E-01	transcription_start_site	-	12438	12438	-3199	153	AN0054
2906	CONTIG3	15463	15812	1	1.93E-01	transcription_start_site	-	12002	12002	-3635	154	AN0054

2906	CONTIG3	15463	15812	1	1.93E-01	transcription_start_site	-	10845	10845	-4792	155	AN0054
2906	CONTIG3	15463	15812	1	1.93E-01	transcription_start_site	+	14722	14722	915	156	AN0055
405	CONTIG3	20038	20617	2.59	0.00E+00	transcription_start_site	-	18585	18585	-1742	157	AN0056
656	CONTIG3	18908	19257	2.23	9.51E-04	transcription_start_site	-	18585	18585	-497	157	AN0056
405	CONTIG3	20038	20617	2.59	0.00E+00	transcription_start_site	+	22996	22996	-2668	158	AN0057
405	CONTIG3	20038	20617	2.59	0.00E+00	transcription_start_site	+	23657	23657	-3329	159	AN0057
405	CONTIG3	20038	20617	2.59	0.00E+00	transcription_start_site	+	23775	23775	-3447	160	AN0057
656	CONTIG3	18908	19257	2.23	9.51E-04	transcription_start_site	+	22996	22996	-3913	158	AN0057
656	CONTIG3	18908	19257	2.23	9.51E-04	transcription_start_site	+	23657	23657	-4574	159	AN0057
656	CONTIG3	18908	19257	2.23	9.51E-04	transcription_start_site	+	23775	23775	-4692	160	AN0057
2847	CONTIG3	27168	27427	1.04	1.66E-01	transcription_start_site	-	26067	26067	-1230	161	AN0058
2847	CONTIG3	27168	27427	1.04	1.66E-01	transcription_start_site	+	26778	26778	519	162	AN0059
2848	CONTIG3	43364	43644	1.04	1.66E-01	transcription_start_site	-	40765	40765	-2739	175	AN0065
2848	CONTIG3	43364	43644	1.04	1.66E-01	transcription_start_site	-	40611	40611	-2893	176	AN0065
2848	CONTIG3	43364	43644	1.04	1.66E-01	transcription_start_site	-	40344	40344	-3160	177	AN0065
2848	CONTIG3	43364	43644	1.04	1.66E-01	transcription_start_site	+	42386	42386	1118	183	AN0066
2848	CONTIG3	43364	43644	1.04	1.66E-01	transcription_start_site	+	43688	43688	-184	184	AN0067
2848	CONTIG3	43364	43644	1.04	1.66E-01	transcription_start_site	+	43793	43793	-289	185	AN0067
2848	CONTIG3	43364	43644	1.04	1.66E-01	transcription_start_site	+	44140	44140	-636	186	AN0067
2848	CONTIG3	43364	43644	1.04	1.66E-01	transcription_start_site	+	44521	44521	-1017	187	AN0067
2507	CONTIG3	49434	49943	1.2	9.24E-02	transcription_start_site	-	46098	46098	-3590	188	AN0068
2507	CONTIG3	49434	49943	1.2	9.24E-02	transcription_start_site	-	45950	45950	-3738	189	AN0068
1933	CONTIG3	51619	51878	1.39	4.36E-02	transcription_start_site	-	49927	49927	-1821	190	AN0069
1933	CONTIG3	51619	51878	1.39	4.36E-02	transcription_start_site	-	49586	49586	-2162	191	AN0069
1933	CONTIG3	51619	51878	1.39	4.36E-02	transcription_start_site	-	49302	49302	-2446	192	AN0069
1933	CONTIG3	51619	51878	1.39	4.36E-02	transcription_start_site	-	48972	48972	-2776	193	AN0069
1933	CONTIG3	51619	51878	1.39	4.36E-02	transcription_start_site	-	48381	48381	-3367	194	AN0069
2507	CONTIG3	49434	49943	1.2	9.24E-02	transcription_start_site	-	49586	49586	-102	191	AN0069
2507	CONTIG3	49434	49943	1.2	9.24E-02	transcription_start_site	-	49927	49927	238	190	AN0069
2507	CONTIG3	49434	49943	1.2	9.24E-02	transcription_start_site	-	49302	49302	-386	192	AN0069
2507	CONTIG3	49434	49943	1.2	9.24E-02	transcription_start_site	-	48972	48972	-716	193	AN0069
2507	CONTIG3	49434	49943	1.2	9.24E-02	transcription_start_site	-	48381	48381	-1307	194	AN0069
2907	CONTIG3	52884	53233	1	1.93E-01	transcription_start_site	-	49927	49927	-3131	190	AN0069
2907	CONTIG3	52884	53233	1	1.93E-01	transcription_start_site	-	49586	49586	-3472	191	AN0069
2907	CONTIG3	52884	53233	1	1.93E-01	transcription_start_site	-	49302	49302	-3756	192	AN0069
2907	CONTIG3	52884	53233	1	1.93E-01	transcription_start_site	-	48972	48972	-4086	193	AN0069
2907	CONTIG3	52884	53233	1	1.93E-01	transcription_start_site	-	48381	48381	-4677	194	AN0069
1933	CONTIG3	51619	51878	1.39	4.36E-02	transcription_start_site	-	51105	51105	-643	195	AN0070
1933	CONTIG3	51619	51878	1.39	4.36E-02	transcription_start_site	-	50649	50649	-1099	196	AN0070
1933	CONTIG3	51619	51878	1.39	4.36E-02	transcription_start_site	-	50416	50416	-1332	197	AN0070
2507	CONTIG3	49434	49943	1.2	9.24E-02	transcription_start_site	-	50416	50416	727	197	AN0070
2507	CONTIG3	49434	49943	1.2	9.24E-02	transcription_start_site	-	50649	50649	960	196	AN0070
2907	CONTIG3	52884	53233	1	1.93E-01	transcription_start_site	-	51105	51105	-1953	195	AN0070
2907	CONTIG3	52884	53233	1	1.93E-01	transcription_start_site	-	50649	50649	-2409	196	AN0070
2907	CONTIG3	52884	53233	1	1.93E-01	transcription_start_site	-	50416	50416	-2642	197	AN0070
1933	CONTIG3	51619	51878	1.39	4.36E-02	transcription_start_site	+	52164	52164	-415	198	AN0071
1933	CONTIG3	51619	51878	1.39	4.36E-02	transcription_start_site	+	52296	52296	-547	199	AN0071
1933	CONTIG3	51619	51878	1.39	4.36E-02	transcription_start_site	+	52548	52548	-799	200	AN0071
1933	CONTIG3	51619	51878	1.39	4.36E-02	transcription_start_site	+	55650	55650	-3901	201	AN0071
2507	CONTIG3	49434	49943	1.2	9.24E-02	transcription_start_site	+	52164	52164	-2475	198	AN0071
2507	CONTIG3	49434	49943	1.2	9.24E-02	transcription_start_site	+	52296	52296	-2607	199	AN0071
2507	CONTIG3	49434	49943	1.2	9.24E-02	transcription_start_site	+	52548	52548	-2859	200	AN0071
2907	CONTIG3	52884	53233	1	1.93E-01	transcription_start_site	+	52548	52548	510	200	AN0071
2907	CONTIG3	52884	53233	1	1.93E-01	transcription_start_site	+	52296	52296	762	199	AN0071
2907	CONTIG3	52884	53233	1	1.93E-01	transcription_start_site	+	52164	52164	894	198	AN0071
2907	CONTIG3	52884	53233	1	1.93E-01	transcription_start_site	+	55650	55650	-2591	201	AN0071
1934	CONTIG3	60080	60354	1.39	4.36E-02	transcription_start_site	-	57290	57290	-2927	202	AN0072

1934	CONTIG3	60080	60354	1.39	4.36E-02	transcription_start_site	-	57207	57207	-3010	203 AN0072
2907	CONTIG3	52884	53233	1	1.93E-01	transcription_start_site	+	57641	57641	-4582	204 AN0073
2907	CONTIG3	52884	53233	1	1.93E-01	transcription_start_site	+	57914	57914	-4855	205 AN0073
1934	CONTIG3	60080	60354	1.39	4.36E-02	transcription_start_site	-	60474	60474	257	209 AN0074
1934	CONTIG3	60080	60354	1.39	4.36E-02	transcription_start_site	-	61222	61222	1005	208 AN0074
1396	CONTIG3	80721	81065	1.63	1.57E-02	transcription_start_site	+	81101	81101	-208	231 AN0081
1396	CONTIG3	80721	81065	1.63	1.57E-02	transcription_start_site	+	81327	81327	-434	232 AN0081
1396	CONTIG3	80721	81065	1.63	1.57E-02	transcription_start_site	+	82095	82095	-1202	233 AN0081
1396	CONTIG3	80721	81065	1.63	1.57E-02	transcription_start_site	+	82241	82241	-1348	234 AN0081
1396	CONTIG3	80721	81065	1.63	1.57E-02	transcription_start_site	+	82423	82423	-1530	235 AN0081
1396	CONTIG3	80721	81065	1.63	1.57E-02	transcription_start_site	+	83818	83818	-2925	236 AN0082
1396	CONTIG3	80721	81065	1.63	1.57E-02	transcription_start_site	+	84491	84491	-3598	237 AN0082
1396	CONTIG3	80721	81065	1.63	1.57E-02	transcription_start_site	+	84835	84835	-3942	238 AN0082
1853	CONTIG3	83864	84133	1.43	3.70E-02	transcription_start_site	+	83818	83818	180	236 AN0082
1853	CONTIG3	83864	84133	1.43	3.70E-02	transcription_start_site	+	84491	84491	-492	237 AN0082
1853	CONTIG3	83864	84133	1.43	3.70E-02	transcription_start_site	+	84835	84835	-836	238 AN0082
1326	CONTIG3	96693	96952	1.67	1.30E-02	transcription_start_site	-	92299	92299	-4523	250 AN0085
1326	CONTIG3	96693	96952	1.67	1.30E-02	transcription_start_site	-	95477	95477	-1345	254 AN0086
1326	CONTIG3	96693	96952	1.67	1.30E-02	transcription_start_site	-	95140	95140	-1682	255 AN0086
1326	CONTIG3	96693	96952	1.67	1.30E-02	transcription_start_site	-	94692	94692	-2130	256 AN0086
1326	CONTIG3	96693	96952	1.67	1.30E-02	transcription_start_site	-	94556	94556	-2266	257 AN0086
1326	CONTIG3	96693	96952	1.67	1.30E-02	transcription_start_site	-	94105	94105	-2717	258 AN0086
1326	CONTIG3	96693	96952	1.67	1.30E-02	transcription_start_site	+	98130	98130	-1307	259 AN0087
1326	CONTIG3	96693	96952	1.67	1.30E-02	transcription_start_site	+	98435	98435	-1612	260 AN0087
2779	CONTIG3	106430	106721	1.08	1.44E-01	transcription_start_site	-	101732	101732	-4843	261 AN0088
2779	CONTIG3	106430	106721	1.08	1.44E-01	transcription_start_site	-	101550	101550	-5025	262 AN0088
2779	CONTIG3	106430	106721	1.08	1.44E-01	transcription_start_site	-	103238	103238	-3337	266 AN0089
2779	CONTIG3	106430	106721	1.08	1.44E-01	transcription_start_site	-	103150	103150	-3425	267 AN0089
2779	CONTIG3	106430	106721	1.08	1.44E-01	transcription_start_site	-	103034	103034	-3541	268 AN0089
2779	CONTIG3	106430	106721	1.08	1.44E-01	transcription_start_site	-	102885	102885	-3690	269 AN0089
2779	CONTIG3	106430	106721	1.08	1.44E-01	transcription_start_site	-	102656	102656	-3919	270 AN0089
2779	CONTIG3	106430	106721	1.08	1.44E-01	transcription_start_site	-	102457	102457	-4118	271 AN0089
2779	CONTIG3	106430	106721	1.08	1.44E-01	transcription_start_site	-	106291	106291	-284	275 AN0091
2779	CONTIG3	106430	106721	1.08	1.44E-01	transcription_start_site	-	105692	105692	-883	276 AN0091
2779	CONTIG3	106430	106721	1.08	1.44E-01	transcription_start_site	-	107255	107255	679	280 AN0092
2779	CONTIG3	106430	106721	1.08	1.44E-01	transcription_start_site	+	109671	109671	-3095	281 AN0093
2779	CONTIG3	106430	106721	1.08	1.44E-01	transcription_start_site	+	109843	109843	-3267	282 AN0093
2779	CONTIG3	106430	106721	1.08	1.44E-01	transcription_start_site	+	111715	111715	-5139	283 AN0093
211	CONTIG3	119328	119752	2.91	0.00E+00	transcription_start_site	-	115492	115492	-4048	287 AN0094
211	CONTIG3	119328	119752	2.91	0.00E+00	transcription_start_site	-	115248	115248	-4292	288 AN0094
211	CONTIG3	119328	119752	2.91	0.00E+00	transcription_start_site	-	115151	115151	-4389	289 AN0094
211	CONTIG3	119328	119752	2.91	0.00E+00	transcription_start_site	-	114765	114765	-4775	290 AN0094
2849	CONTIG3	115953	116992	1.04	1.66E-01	transcription_start_site	-	115492	115492	-980	287 AN0094
2849	CONTIG3	115953	116992	1.04	1.66E-01	transcription_start_site	-	115248	115248	-1224	288 AN0094
2849	CONTIG3	115953	116992	1.04	1.66E-01	transcription_start_site	-	115151	115151	-1321	289 AN0094
2849	CONTIG3	115953	116992	1.04	1.66E-01	transcription_start_site	-	114765	114765	-1707	290 AN0094
2849	CONTIG3	115953	116992	1.04	1.66E-01	transcription_start_site	-	113975	113975	-2497	291 AN0094
2849	CONTIG3	115953	116992	1.04	1.66E-01	transcription_start_site	-	113873	113873	-2599	292 AN0094
2849	CONTIG3	115953	116992	1.04	1.66E-01	transcription_start_site	-	113750	113750	-2722	293 AN0094
2849	CONTIG3	115953	116992	1.04	1.66E-01	transcription_start_site	-	113434	113434	-3038	294 AN0094
2849	CONTIG3	115953	116992	1.04	1.66E-01	transcription_start_site	-	112691	112691	-3781	295 AN0094
211	CONTIG3	119328	119752	2.91	0.00E+00	transcription_start_site	-	119588	119588	48	296 AN0095
211	CONTIG3	119328	119752	2.91	0.00E+00	transcription_start_site	+	120336	120336	-796	297 AN0096
211	CONTIG3	119328	119752	2.91	0.00E+00	transcription_start_site	+	120455	120455	-915	298 AN0096
211	CONTIG3	119328	119752	2.91	0.00E+00	transcription_start_site	+	120748	120748	-1208	299 AN0096
211	CONTIG3	119328	119752	2.91	0.00E+00	transcription_start_site	+	122202	122202	-2662	300 AN0096
211	CONTIG3	119328	119752	2.91	0.00E+00	transcription_start_site	+	122369	122369	-2829	301 AN0096

211	CONTIG3	119328	119752	2.91	0.00E+00	transcription_start_site	+	122688	122688	-3148	302 AN0096
211	CONTIG3	119328	119752	2.91	0.00E+00	transcription_start_site	+	122885	122885	-3345	303 AN0096
211	CONTIG3	119328	119752	2.91	0.00E+00	transcription_start_site	+	123900	123900	-4360	304 AN0096
2849	CONTIG3	115953	116992	1.04	1.66E-01	transcription_start_site	+	120336	120336	-3863	297 AN0096
2849	CONTIG3	115953	116992	1.04	1.66E-01	transcription_start_site	+	120455	120455	-3982	298 AN0096
2849	CONTIG3	115953	116992	1.04	1.66E-01	transcription_start_site	+	120748	120748	-4275	299 AN0096
211	CONTIG3	119328	119752	2.91	0.00E+00	transcription_start_site	+	124162	124162	-4622	305 AN0097
211	CONTIG3	119328	119752	2.91	0.00E+00	transcription_start_site	+	124296	124296	-4756	306 AN0097
1995	CONTIG4	17106	17390	1.37	4.36E-02	transcription_start_site	-	16612	16612	-636	335 AN0103
1995	CONTIG4	17106	17390	1.37	4.36E-02	transcription_start_site	-	16534	16534	-714	336 AN0103
1995	CONTIG4	17106	17390	1.37	4.36E-02	transcription_start_site	-	15826	15826	-1422	337 AN0103
1995	CONTIG4	17106	17390	1.37	4.36E-02	transcription_start_site	-	15349	15349	-1899	338 AN0103
1996	CONTIG4	21396	21895	1.37	4.36E-02	transcription_start_site	-	16612	16612	-5033	335 AN0103
1996	CONTIG4	21396	21895	1.37	4.36E-02	transcription_start_site	-	16534	16534	-5111	336 AN0103
1995	CONTIG4	17106	17390	1.37	4.36E-02	transcription_start_site	+	18189	18189	-941	339 AN0104
1995	CONTIG4	17106	17390	1.37	4.36E-02	transcription_start_site	+	18374	18374	-1126	340 AN0104
1995	CONTIG4	17106	17390	1.37	4.36E-02	transcription_start_site	+	18953	18953	-1705	341 AN0104
1996	CONTIG4	21396	21895	1.37	4.36E-02	transcription_start_site	-	20148	20148	-1497	342 AN0105
1996	CONTIG4	21396	21895	1.37	4.36E-02	transcription_start_site	-	19784	19784	-1861	343 AN0105
1995	CONTIG4	17106	17390	1.37	4.36E-02	transcription_start_site	+	21586	21586	-4338	344 AN0106
1995	CONTIG4	17106	17390	1.37	4.36E-02	transcription_start_site	+	22074	22074	-4826	345 AN0106
1996	CONTIG4	21396	21895	1.37	4.36E-02	transcription_start_site	+	21586	21586	59	344 AN0106
1996	CONTIG4	21396	21895	1.37	4.36E-02	transcription_start_site	+	22074	22074	-428	345 AN0106
1996	CONTIG4	21396	21895	1.37	4.36E-02	transcription_start_site	+	22399	22399	-753	346 AN0106
1996	CONTIG4	21396	21895	1.37	4.36E-02	transcription_start_site	+	23018	23018	-1372	347 AN0106
1996	CONTIG4	21396	21895	1.37	4.36E-02	transcription_start_site	+	23469	23469	-1823	348 AN0106
1996	CONTIG4	21396	21895	1.37	4.36E-02	transcription_start_site	+	23908	23908	-2262	349 AN0106
1996	CONTIG4	21396	21895	1.37	4.36E-02	transcription_start_site	+	24530	24530	-2884	350 AN0106
1996	CONTIG4	21396	21895	1.37	4.36E-02	transcription_start_site	+	24781	24781	-3135	351 AN0106
1996	CONTIG4	21396	21895	1.37	4.36E-02	transcription_start_site	+	25242	25242	-3596	352 AN0106
1996	CONTIG4	21396	21895	1.37	4.36E-02	transcription_start_site	+	26719	26719	-5073	353 AN0107
2561	CONTIG4	69454	69803	1.18	9.24E-02	transcription_start_site	-	65111	65111	-4517	387 AN0116
2561	CONTIG4	69454	69803	1.18	9.24E-02	transcription_start_site	-	64924	64924	-4704	388 AN0116
3011	CONTIG4	64444	65903	0.9	1.58E-01	transcription_start_site	-	65111	65111	-62	387 AN0116
3011	CONTIG4	64444	65903	0.9	1.58E-01	transcription_start_site	-	64924	64924	-249	388 AN0116
1303	CONTIG4	63079	63353	1.69	1.11E-02	transcription_start_site	+	65613	65613	-2397	389 AN0117
1303	CONTIG4	63079	63353	1.69	1.11E-02	transcription_start_site	+	65714	65714	-2498	390 AN0117
1303	CONTIG4	63079	63353	1.69	1.11E-02	transcription_start_site	+	66008	66008	-2792	391 AN0117
1303	CONTIG4	63079	63353	1.69	1.11E-02	transcription_start_site	+	66488	66488	-3272	392 AN0117
1303	CONTIG4	63079	63353	1.69	1.11E-02	transcription_start_site	+	66913	66913	-3697	393 AN0117
2560	CONTIG4	61964	62233	1.18	9.24E-02	transcription_start_site	+	65613	65613	-3514	389 AN0117
2560	CONTIG4	61964	62233	1.18	9.24E-02	transcription_start_site	+	65714	65714	-3615	390 AN0117
2560	CONTIG4	61964	62233	1.18	9.24E-02	transcription_start_site	+	66008	66008	-3909	391 AN0117
2560	CONTIG4	61964	62233	1.18	9.24E-02	transcription_start_site	+	66488	66488	-4389	392 AN0117
2560	CONTIG4	61964	62233	1.18	9.24E-02	transcription_start_site	+	66913	66913	-4814	393 AN0117
3011	CONTIG4	64444	65903	0.9	1.58E-01	transcription_start_site	+	65613	65613	-439	389 AN0117
3011	CONTIG4	64444	65903	0.9	1.58E-01	transcription_start_site	+	65714	65714	-540	390 AN0117
3011	CONTIG4	64444	65903	0.9	1.58E-01	transcription_start_site	+	66008	66008	-834	391 AN0117
3011	CONTIG4	64444	65903	0.9	1.58E-01	transcription_start_site	+	66488	66488	-1314	392 AN0117
3011	CONTIG4	64444	65903	0.9	1.58E-01	transcription_start_site	+	66913	66913	-1739	393 AN0117
2472	CONTIG4	82204	82478	1.21	8.01E-02	transcription_start_site	-	81977	81977	-364	394 AN0118
2472	CONTIG4	82204	82478	1.21	8.01E-02	transcription_start_site	-	81630	81630	-711	395 AN0118
2472	CONTIG4	82204	82478	1.21	8.01E-02	transcription_start_site	+	86128	86128	-3787	404 AN0120
2472	CONTIG4	82204	82478	1.21	8.01E-02	transcription_start_site	+	86399	86399	-4058	405 AN0120
2472	CONTIG4	82204	82478	1.21	8.01E-02	transcription_start_site	+	86732	86732	-4391	406 AN0120
92	CONTIG4	110630	111219	3.25	0.00E+00	transcription_start_site	-	105826	105826	-5098	422 AN0127
92	CONTIG4	110630	111219	3.25	0.00E+00	transcription_start_site	-	105717	105717	-5207	423 AN0127

92	CONTIG4	110630	111219	3.25	0.00E+00	transcription_start_site	+	112277	112277	-1352	432 AN0129
92	CONTIG4	110630	111219	3.25	0.00E+00	transcription_start_site	+	113083	113083	-2158	433 AN0129
92	CONTIG4	110630	111219	3.25	0.00E+00	transcription_start_site	+	114154	114154	-3229	434 AN0129
92	CONTIG4	110630	111219	3.25	0.00E+00	transcription_start_site	+	114322	114322	-3397	435 AN0129
92	CONTIG4	110630	111219	3.25	0.00E+00	transcription_start_site	+	114433	114433	-3508	436 AN0129
1613	CONTIG4	111826	112475	1.53	1.06E-02	transcription_start_site	+	112277	112277	-126	432 AN0129
1613	CONTIG4	111826	112475	1.53	1.06E-02	transcription_start_site	+	113083	113083	-932	433 AN0129
1613	CONTIG4	111826	112475	1.53	1.06E-02	transcription_start_site	+	114154	114154	-2003	434 AN0129
1613	CONTIG4	111826	112475	1.53	1.06E-02	transcription_start_site	+	114322	114322	-2171	435 AN0129
1613	CONTIG4	111826	112475	1.53	1.06E-02	transcription_start_site	+	114433	114433	-2282	436 AN0129
1613	CONTIG4	111826	112475	1.53	1.06E-02	transcription_start_site	+	117259	117259	-5108	437 AN0130
2813	CONTIG4	129394	129670	1.06	1.44E-01	transcription_start_site	-	124818	124818	-4714	442 AN0133
2813	CONTIG4	129394	129670	1.06	1.44E-01	transcription_start_site	-	124447	124447	-5085	443 AN0133
2813	CONTIG4	129394	129670	1.06	1.44E-01	transcription_start_site	+	129728	129728	-196	449 AN0135
2813	CONTIG4	129394	129670	1.06	1.44E-01	transcription_start_site	+	129923	129923	-391	450 AN0135
216	CONTIG4	134789	135053	2.9	0.00E+00	transcription_start_site	-	134821	134821	-100	451 AN0136
216	CONTIG4	134789	135053	2.9	0.00E+00	transcription_start_site	-	134686	134686	-235	452 AN0136
216	CONTIG4	134789	135053	2.9	0.00E+00	transcription_start_site	-	134458	134458	-463	453 AN0136
216	CONTIG4	134789	135053	2.9	0.00E+00	transcription_start_site	+	135556	135556	-635	454 AN0137
216	CONTIG4	134789	135053	2.9	0.00E+00	transcription_start_site	+	135754	135754	-833	455 AN0137
216	CONTIG4	134789	135053	2.9	0.00E+00	transcription_start_site	+	138649	138649	-3728	456 AN0137
216	CONTIG4	134789	135053	2.9	0.00E+00	transcription_start_site	+	139101	139101	-4180	457 AN0137
216	CONTIG4	134789	135053	2.9	0.00E+00	transcription_start_site	+	139928	139928	-5007	458 AN0138
216	CONTIG4	134789	135053	2.9	0.00E+00	transcription_start_site	+	140037	140037	-5116	459 AN0138
2562	CONTIG4	149794	150143	1.18	9.24E-02	transcription_start_site	-	148068	148068	-1900	469 AN0141
2562	CONTIG4	149794	150143	1.18	9.24E-02	transcription_start_site	-	150224	150224	255	472 AN0143
2562	CONTIG4	149794	150143	1.18	9.24E-02	transcription_start_site	-	150476	150476	507	471 AN0143
1815	CONTIG5	46958	47458	1.45	1.66E-01	transcription_start_site	-	43001	43001	-4207	500 AN0158
1815	CONTIG5	46958	47458	1.45	1.66E-01	transcription_start_site	-	42173	42173	-5035	501 AN0158
1815	CONTIG5	46958	47458	1.45	1.66E-01	transcription_start_site	+	47834	47834	-626	503 AN0159
289	CONTIG5	54909	55193	2.78	3.03E-03	transcription_start_site	-	51690	51690	-3361	505 AN0161
289	CONTIG5	54909	55193	2.78	3.03E-03	transcription_start_site	-	51445	51445	-3606	506 AN0161
289	CONTIG5	54909	55193	2.78	3.03E-03	transcription_start_site	-	50643	50643	-4408	507 AN0161
289	CONTIG5	54909	55193	2.78	3.03E-03	transcription_start_site	-	54027	54027	-1024	508 AN0162
289	CONTIG5	54909	55193	2.78	3.03E-03	transcription_start_site	-	53855	53855	-1196	509 AN0162
289	CONTIG5	54909	55193	2.78	3.03E-03	transcription_start_site	-	53576	53576	-1475	510 AN0162
289	CONTIG5	54909	55193	2.78	3.03E-03	transcription_start_site	-	53348	53348	-1703	511 AN0162
289	CONTIG5	54909	55193	2.78	3.03E-03	transcription_start_site	-	52473	52473	-2578	512 AN0162
289	CONTIG5	54909	55193	2.78	3.03E-03	transcription_start_site	+	56587	56587	-1536	513 AN0163
289	CONTIG5	54909	55193	2.78	3.03E-03	transcription_start_site	+	56883	56883	-1832	514 AN0163
289	CONTIG5	54909	55193	2.78	3.03E-03	transcription_start_site	+	57272	57272	-2221	515 AN0163
289	CONTIG5	54909	55193	2.78	3.03E-03	transcription_start_site	+	57378	57378	-2327	516 AN0163
1967	CONTIG5	67507	68076	1.39	1.93E-01	transcription_start_site	-	64652	64652	-3139	522 AN0166
1967	CONTIG5	67507	68076	1.39	1.93E-01	transcription_start_site	-	64584	64584	-3207	523 AN0166
1967	CONTIG5	67507	68076	1.39	1.93E-01	transcription_start_site	-	64253	64253	-3538	524 AN0166
1967	CONTIG5	67507	68076	1.39	1.93E-01	transcription_start_site	-	63281	63281	-4510	525 AN0166
1967	CONTIG5	67507	68076	1.39	1.93E-01	transcription_start_site	+	71382	71382	-3590	528 AN0168
1179	CONTIG5	74626	74920	1.78	6.84E-02	transcription_start_site	-	74403	74403	-370	529 AN0169
1179	CONTIG5	74626	74920	1.78	6.84E-02	transcription_start_site	+	74725	74725	48	531 AN0170
1179	CONTIG5	74626	74920	1.78	6.84E-02	transcription_start_site	+	75007	75007	-234	532 AN0170
1179	CONTIG5	74626	74920	1.78	6.84E-02	transcription_start_site	+	74428	74428	345	530 AN0170
908	CONTIG5	79426	79945	2	3.70E-02	transcription_start_site	-	78653	78653	-1032	533 AN0171
908	CONTIG5	79426	79945	2	3.70E-02	transcription_start_site	-	78439	78439	-1246	534 AN0171
908	CONTIG5	79426	79945	2	3.70E-02	transcription_start_site	-	78066	78066	-1619	535 AN0171
908	CONTIG5	79426	79945	2	3.70E-02	transcription_start_site	-	77735	77735	-1950	536 AN0171
908	CONTIG5	79426	79945	2	3.70E-02	transcription_start_site	+	84692	84692	-5006	537 AN0172
843	CONTIG5	95479	96368	2.06	1.81E-02	transcription_start_site	+	97554	97554	-1630	549 AN0176

843	CONTIG5	95479	96368	2.06	1.81E-02	transcription_start_site	+	97929	97929	-2005	550 AN0176
843	CONTIG5	95479	96368	2.06	1.81E-02	transcription_start_site	+	98495	98495	-2571	551 AN0176
581	CONTIG5	102237	102741	2.34	1.30E-02	transcription_start_site	-	100141	100141	-2348	552 AN0177
581	CONTIG5	102237	102741	2.34	1.30E-02	transcription_start_site	-	99939	99939	-2550	553 AN0177
581	CONTIG5	102237	102741	2.34	1.30E-02	transcription_start_site	-	101453	101453	-1036	554 AN0178
581	CONTIG5	102237	102741	2.34	1.30E-02	transcription_start_site	-	101034	101034	-1455	555 AN0178
581	CONTIG5	102237	102741	2.34	1.30E-02	transcription_start_site	-	100899	100899	-1590	556 AN0178
581	CONTIG5	102237	102741	2.34	1.30E-02	transcription_start_site	+	105189	105189	-2700	557 AN0179
581	CONTIG5	102237	102741	2.34	1.30E-02	transcription_start_site	+	105671	105671	-3182	558 AN0179
581	CONTIG5	102237	102741	2.34	1.30E-02	transcription_start_site	+	106029	106029	-3540	559 AN0179
581	CONTIG5	102237	102741	2.34	1.30E-02	transcription_start_site	+	106135	106135	-3646	560 AN0179
581	CONTIG5	102237	102741	2.34	1.30E-02	transcription_start_site	+	106296	106296	-3807	561 AN0179
849	CONTIG5	106512	106781	2.06	3.08E-02	transcription_start_site	+	106296	106296	350	561 AN0179
849	CONTIG5	106512	106781	2.06	3.08E-02	transcription_start_site	+	106135	106135	511	560 AN0179
849	CONTIG5	106512	106781	2.06	3.08E-02	transcription_start_site	+	106029	106029	617	559 AN0179
849	CONTIG5	106512	106781	2.06	3.08E-02	transcription_start_site	+	105671	105671	975	558 AN0179
849	CONTIG5	106512	106781	2.06	3.08E-02	transcription_start_site	-	107468	107468	821	562 AN0180
1711	CONTIG5	111393	111737	1.5	1.44E-01	transcription_start_site	-	107468	107468	-4097	562 AN0180
849	CONTIG5	106512	106781	2.06	3.08E-02	transcription_start_site	+	108260	108260	-1613	563 AN0181
849	CONTIG5	106512	106781	2.06	3.08E-02	transcription_start_site	+	109233	109233	-2586	564 AN0181
1711	CONTIG5	111393	111737	1.5	1.44E-01	transcription_start_site	+	111931	111931	-366	565 AN0182
1711	CONTIG5	111393	111737	1.5	1.44E-01	transcription_start_site	+	112073	112073	-508	566 AN0182
1711	CONTIG5	111393	111737	1.5	1.44E-01	transcription_start_site	+	112247	112247	-682	567 AN0182
1711	CONTIG5	111393	111737	1.5	1.44E-01	transcription_start_site	+	112585	112585	-1020	568 AN0182
290	CONTIG5	117995	118481	2.78	3.03E-03	transcription_start_site	-	114512	114512	-3726	569 AN0183
290	CONTIG5	117995	118481	2.78	3.03E-03	transcription_start_site	-	114456	114456	-3782	570 AN0183
290	CONTIG5	117995	118481	2.78	3.03E-03	transcription_start_site	-	114211	114211	-4027	571 AN0183
290	CONTIG5	117995	118481	2.78	3.03E-03	transcription_start_site	-	113542	113542	-4696	572 AN0183
1711	CONTIG5	111393	111737	1.5	1.44E-01	transcription_start_site	+	114973	114973	-3408	573 AN0184
1711	CONTIG5	111393	111737	1.5	1.44E-01	transcription_start_site	+	115059	115059	-3494	574 AN0184
1711	CONTIG5	111393	111737	1.5	1.44E-01	transcription_start_site	+	115247	115247	-3682	575 AN0184
1711	CONTIG5	111393	111737	1.5	1.44E-01	transcription_start_site	+	115451	115451	-3886	576 AN0184
290	CONTIG5	117995	118481	2.78	3.03E-03	transcription_start_site	-	117928	117928	-310	577 AN0185
290	CONTIG5	117995	118481	2.78	3.03E-03	transcription_start_site	-	117831	117831	-407	578 AN0185
844	CONTIG5	121430	121859	2.06	1.81E-02	transcription_start_site	-	117928	117928	-3716	577 AN0185
844	CONTIG5	121430	121859	2.06	1.81E-02	transcription_start_site	-	117831	117831	-3813	578 AN0185
290	CONTIG5	117995	118481	2.78	3.03E-03	transcription_start_site	+	118494	118494	-256	579 AN0186
290	CONTIG5	117995	118481	2.78	3.03E-03	transcription_start_site	+	118696	118696	-458	580 AN0186
290	CONTIG5	117995	118481	2.78	3.03E-03	transcription_start_site	+	119295	119295	-1057	581 AN0186
290	CONTIG5	117995	118481	2.78	3.03E-03	transcription_start_site	+	119542	119542	-1304	582 AN0186
844	CONTIG5	121430	121859	2.06	1.81E-02	transcription_start_site	-	121783	121783	138	584 AN0187
844	CONTIG5	121430	121859	2.06	1.81E-02	transcription_start_site	-	122051	122051	406	583 AN0187
582	CONTIG5	151962	152241	2.34	1.30E-02	transcription_start_site	-	147330	147330	-4771	615 AN0198
582	CONTIG5	151962	152241	2.34	1.30E-02	transcription_start_site	-	151943	151943	-158	617 AN0200
582	CONTIG5	151962	152241	2.34	1.30E-02	transcription_start_site	-	151606	151606	-495	618 AN0200
582	CONTIG5	151962	152241	2.34	1.30E-02	transcription_start_site	-	151319	151319	-782	619 AN0200
582	CONTIG5	151962	152241	2.34	1.30E-02	transcription_start_site	-	152861	152861	759	623 AN0201
582	CONTIG5	151962	152241	2.34	1.30E-02	transcription_start_site	+	155411	155411	-3309	624 AN0202
582	CONTIG5	151962	152241	2.34	1.30E-02	transcription_start_site	+	156044	156044	-3942	625 AN0202
683	CONTIG5	175738	176002	2.22	1.84E-02	transcription_start_site	-	171316	171316	-4554	641 AN0208
683	CONTIG5	175738	176002	2.22	1.84E-02	transcription_start_site	-	171076	171076	-4794	642 AN0208
683	CONTIG5	175738	176002	2.22	1.84E-02	transcription_start_site	-	170763	170763	-5107	643 AN0208
683	CONTIG5	175738	176002	2.22	1.84E-02	transcription_start_site	+	176349	176349	-479	652 AN0210
683	CONTIG5	175738	176002	2.22	1.84E-02	transcription_start_site	+	176420	176420	-550	653 AN0210
683	CONTIG5	175738	176002	2.22	1.84E-02	transcription_start_site	+	176504	176504	-634	654 AN0210
683	CONTIG5	175738	176002	2.22	1.84E-02	transcription_start_site	+	176689	176689	-819	655 AN0210
683	CONTIG5	175738	176002	2.22	1.84E-02	transcription_start_site	+	179705	179705	-3835	656 AN0211

683	CONTIG5	175738	176002	2.22	1.84E-02	transcription_start_site	+	180018	180018	-4148	657 AN0211
683	CONTIG5	175738	176002	2.22	1.84E-02	transcription_start_site	+	180340	180340	-4470	658 AN0211
45	CONTIG5	185794	186355	3.45	0.00E+00	transcription_start_site	+	186301	186301	-226	661 AN0213
907	CONTIG5	187071	187625	2	2.18E-02	transcription_start_site	+	186301	186301	1047	661 AN0213
45	CONTIG5	185794	186355	3.45	0.00E+00	transcription_start_site	+	189660	189660	-3585	662 AN0214
45	CONTIG5	185794	186355	3.45	0.00E+00	transcription_start_site	+	189853	189853	-3778	663 AN0214
45	CONTIG5	185794	186355	3.45	0.00E+00	transcription_start_site	+	191069	191069	-4994	664 AN0214
907	CONTIG5	187071	187625	2	2.18E-02	transcription_start_site	+	189660	189660	-2312	662 AN0214
907	CONTIG5	187071	187625	2	2.18E-02	transcription_start_site	+	189853	189853	-2505	663 AN0214
907	CONTIG5	187071	187625	2	2.18E-02	transcription_start_site	+	191069	191069	-3721	664 AN0214
1817	CONTIG5	218711	219070	1.45	1.66E-01	transcription_start_site	-	219726	219726	835	714 AN0223
1817	CONTIG5	218711	219070	1.45	1.66E-01	transcription_start_site	-	219909	219909	1018	713 AN0223
548	CONTIG5	245490	245776	2.39	1.11E-02	transcription_start_site	-	245121	245121	-512	728 AN0229
548	CONTIG5	245490	245776	2.39	1.11E-02	transcription_start_site	-	245055	245055	-578	729 AN0229
548	CONTIG5	245490	245776	2.39	1.11E-02	transcription_start_site	-	243598	243598	-2035	730 AN0229
548	CONTIG5	245490	245776	2.39	1.11E-02	transcription_start_site	-	243151	243151	-2482	731 AN0229
324	CONTIG5	260180	260539	2.73	3.61E-03	transcription_start_site	-	255745	255745	-4614	740 AN0232
324	CONTIG5	260180	260539	2.73	3.61E-03	transcription_start_site	-	255527	255527	-4832	741 AN0232
324	CONTIG5	260180	260539	2.73	3.61E-03	transcription_start_site	-	255354	255354	-5005	742 AN0232
324	CONTIG5	260180	260539	2.73	3.61E-03	transcription_start_site	+	259763	259763	596	752 AN0234
324	CONTIG5	260180	260539	2.73	3.61E-03	transcription_start_site	+	259496	259496	863	751 AN0234
324	CONTIG5	260180	260539	2.73	3.61E-03	transcription_start_site	+	260478	260478	-118	753 AN0235
324	CONTIG5	260180	260539	2.73	3.61E-03	transcription_start_site	+	262157	262157	-1797	754 AN0235
324	CONTIG5	260180	260539	2.73	3.61E-03	transcription_start_site	+	263534	263534	-3174	755 AN0235
100	CONTIG5	275713	276064	3.23	5.64E-04	transcription_start_site	-	273403	273403	-2485	756 AN0237
100	CONTIG5	275713	276064	3.23	5.64E-04	transcription_start_site	-	273196	273196	-2692	757 AN0237
100	CONTIG5	275713	276064	3.23	5.64E-04	transcription_start_site	-	271367	271367	-4521	758 AN0237
734	CONTIG5	276678	277107	2.17	1.06E-02	transcription_start_site	-	273403	273403	-3489	756 AN0237
734	CONTIG5	276678	277107	2.17	1.06E-02	transcription_start_site	-	273196	273196	-3696	757 AN0237
1180	CONTIG5	277963	278324	1.78	6.84E-02	transcription_start_site	-	273403	273403	-4740	756 AN0237
1180	CONTIG5	277963	278324	1.78	6.84E-02	transcription_start_site	-	273196	273196	-4947	757 AN0237
100	CONTIG5	275713	276064	3.23	5.64E-04	transcription_start_site	+	279748	279748	-3859	761 AN0239
734	CONTIG5	276678	277107	2.17	1.06E-02	transcription_start_site	+	279748	279748	-2855	761 AN0239
1029	CONTIG5	288154	288448	1.89	5.22E-02	transcription_start_site	+	287523	287523	778	768 AN0239
1029	CONTIG5	288154	288448	1.89	5.22E-02	transcription_start_site	+	287295	287295	1006	767 AN0239
1180	CONTIG5	277963	278324	1.78	6.84E-02	transcription_start_site	+	279748	279748	-1604	761 AN0239
1180	CONTIG5	277963	278324	1.78	6.84E-02	transcription_start_site	+	282274	282274	-4130	762 AN0239
1180	CONTIG5	277963	278324	1.78	6.84E-02	transcription_start_site	+	282683	282683	-4539	763 AN0239
1029	CONTIG5	288154	288448	1.89	5.22E-02	transcription_start_site	+	288539	288539	-238	769 AN0240
1029	CONTIG5	288154	288448	1.89	5.22E-02	transcription_start_site	+	288656	288656	-355	770 AN0240
1029	CONTIG5	288154	288448	1.89	5.22E-02	transcription_start_site	+	289020	289020	-719	771 AN0240
1029	CONTIG5	288154	288448	1.89	5.22E-02	transcription_start_site	+	289460	289460	-1159	772 AN0240
1029	CONTIG5	288154	288448	1.89	5.22E-02	transcription_start_site	+	290636	290636	-2335	773 AN0241
1029	CONTIG5	288154	288448	1.89	5.22E-02	transcription_start_site	+	291032	291032	-2731	774 AN0241
1029	CONTIG5	288154	288448	1.89	5.22E-02	transcription_start_site	+	291418	291418	-3117	775 AN0241
1029	CONTIG5	288154	288448	1.89	5.22E-02	transcription_start_site	+	291573	291573	-3272	776 AN0241
1029	CONTIG5	288154	288448	1.89	5.22E-02	transcription_start_site	+	292100	292100	-3799	777 AN0242
1029	CONTIG5	288154	288448	1.89	5.22E-02	transcription_start_site	+	292410	292410	-4109	778 AN0242
1712	CONTIG5	297390	297734	1.5	1.44E-01	transcription_start_site	+	296469	296469	1093	782 AN0243
1712	CONTIG5	297390	297734	1.5	1.44E-01	transcription_start_site	+	297869	297869	-307	783 AN0244
1712	CONTIG5	297390	297734	1.5	1.44E-01	transcription_start_site	+	298273	298273	-711	784 AN0244
1712	CONTIG5	297390	297734	1.5	1.44E-01	transcription_start_site	+	298771	298771	-1209	785 AN0244
850	CONTIG5	310131	310410	2.06	3.08E-02	transcription_start_site	-	309920	309920	-350	788 AN0246
850	CONTIG5	310131	310410	2.06	3.08E-02	transcription_start_site	-	309628	309628	-642	789 AN0246
850	CONTIG5	310131	310410	2.06	3.08E-02	transcription_start_site	-	309305	309305	-965	790 AN0246
850	CONTIG5	310131	310410	2.06	3.08E-02	transcription_start_site	-	308843	308843	-1427	791 AN0246
850	CONTIG5	310131	310410	2.06	3.08E-02	transcription_start_site	-	308649	308649	-1621	792 AN0246

850	CONTIG5	310131	310410	2.06	3.08E-02	transcription_start_site	-	308185	308185	-2085	793 AN0246
850	CONTIG5	310131	310410	2.06	3.08E-02	transcription_start_site	-	307975	307975	-2295	794 AN0246
850	CONTIG5	310131	310410	2.06	3.08E-02	transcription_start_site	+	310850	310850	-579	795 AN0247
850	CONTIG5	310131	310410	2.06	3.08E-02	transcription_start_site	+	313929	313929	-3658	796 AN0247
850	CONTIG5	310131	310410	2.06	3.08E-02	transcription_start_site	+	314221	314221	-3950	797 AN0247
12	CONTIG5	354757	355101	3.67	0.00E+00	transcription_start_site	-	355412	355412	483	871 AN0264
12	CONTIG5	354757	355101	3.67	0.00E+00	transcription_start_site	-	355499	355499	570	870 AN0264
12	CONTIG5	354757	355101	3.67	0.00E+00	transcription_start_site	-	355888	355888	959	869 AN0264
12	CONTIG5	354757	355101	3.67	0.00E+00	transcription_start_site	+	356551	356551	-1622	872 AN0265
12	CONTIG5	354757	355101	3.67	0.00E+00	transcription_start_site	+	358134	358134	-3205	873 AN0265
12	CONTIG5	354757	355101	3.67	0.00E+00	transcription_start_site	+	358368	358368	-3439	874 AN0265
12	CONTIG5	354757	355101	3.67	0.00E+00	transcription_start_site	+	359530	359530	-4601	875 AN0265
909	CONTIG5	364952	365226	2	3.70E-02	transcription_start_site	-	364987	364987	-102	879 AN0267
909	CONTIG5	364952	365226	2	3.70E-02	transcription_start_site	-	364591	364591	-498	880 AN0267
909	CONTIG5	364952	365226	2	3.70E-02	transcription_start_site	+	365832	365832	-743	881 AN0268
909	CONTIG5	364952	365226	2	3.70E-02	transcription_start_site	+	366528	366528	-1439	882 AN0268
909	CONTIG5	364952	365226	2	3.70E-02	transcription_start_site	+	366629	366629	-1540	883 AN0268
739	CONTIG5	372977	373331	2.17	2.12E-02	transcription_start_site	+	372248	372248	906	889 AN0270
291	CONTIG5	377402	377686	2.78	3.03E-03	transcription_start_site	-	373231	373231	-4313	890 AN0271
291	CONTIG5	377402	377686	2.78	3.03E-03	transcription_start_site	-	372793	372793	-4751	891 AN0271
291	CONTIG5	377402	377686	2.78	3.03E-03	transcription_start_site	-	372570	372570	-4974	892 AN0271
739	CONTIG5	372977	373331	2.17	2.12E-02	transcription_start_site	-	373231	373231	77	890 AN0271
739	CONTIG5	372977	373331	2.17	2.12E-02	transcription_start_site	-	372793	372793	-361	891 AN0271
739	CONTIG5	372977	373331	2.17	2.12E-02	transcription_start_site	-	372570	372570	-584	892 AN0271
739	CONTIG5	372977	373331	2.17	2.12E-02	transcription_start_site	+	373735	373735	-581	893 AN0272
739	CONTIG5	372977	373331	2.17	2.12E-02	transcription_start_site	+	373901	373901	-747	894 AN0272
739	CONTIG5	372977	373331	2.17	2.12E-02	transcription_start_site	+	374244	374244	-1090	895 AN0272
291	CONTIG5	377402	377686	2.78	3.03E-03	transcription_start_site	+	379900	379900	-2356	896 AN0273
291	CONTIG5	377402	377686	2.78	3.03E-03	transcription_start_site	+	381111	381111	-3567	897 AN0273
291	CONTIG5	377402	377686	2.78	3.03E-03	transcription_start_site	+	381358	381358	-3814	898 AN0273
1472	CONTIG5	380477	380752	1.61	1.08E-01	transcription_start_site	+	381111	381111	-496	897 AN0273
1472	CONTIG5	380477	380752	1.61	1.08E-01	transcription_start_site	+	379900	379900	714	896 AN0273
1472	CONTIG5	380477	380752	1.61	1.08E-01	transcription_start_site	+	381358	381358	-743	898 AN0273
252	CONTIG5	384244	384599	2.84	2.22E-03	transcription_start_site	-	383466	383466	-955	899 AN0274
252	CONTIG5	384244	384599	2.84	2.22E-03	transcription_start_site	-	383302	383302	-1119	900 AN0274
252	CONTIG5	384244	384599	2.84	2.22E-03	transcription_start_site	-	381944	381944	-2477	901 AN0274
252	CONTIG5	384244	384599	2.84	2.22E-03	transcription_start_site	+	387154	387154	-2732	906 AN0276
252	CONTIG5	384244	384599	2.84	2.22E-03	transcription_start_site	+	387358	387358	-2936	907 AN0276
252	CONTIG5	384244	384599	2.84	2.22E-03	transcription_start_site	+	387774	387774	-3352	908 AN0276
46	CONTIG5	402001	402508	3.45	2.67E-04	transcription_start_site	-	402316	402316	61	913 AN0279
292	CONTIG5	405751	406035	2.78	3.03E-03	transcription_start_site	-	402316	402316	-3577	913 AN0279
359	CONTIG5	403731	404006	2.67	4.61E-03	transcription_start_site	-	402316	402316	-1552	913 AN0279
851	CONTIG5	407121	407755	2.06	3.08E-02	transcription_start_site	-	402316	402316	-5122	913 AN0279
1473	CONTIG5	406296	406940	1.61	1.08E-01	transcription_start_site	-	402316	402316	-4302	913 AN0279
1568	CONTIG5	404786	405425	1.56	1.25E-01	transcription_start_site	-	402316	402316	-2789	913 AN0279
1569	CONTIG5	420167	420426	1.56	1.25E-01	transcription_start_site	-	418871	418871	-1425	919 AN0282
1569	CONTIG5	420167	420426	1.56	1.25E-01	transcription_start_site	-	418738	418738	-1558	920 AN0282
1569	CONTIG5	420167	420426	1.56	1.25E-01	transcription_start_site	-	418527	418527	-1769	921 AN0282
1569	CONTIG5	420167	420426	1.56	1.25E-01	transcription_start_site	-	418281	418281	-2015	922 AN0282
1569	CONTIG5	420167	420426	1.56	1.25E-01	transcription_start_site	-	417045	417045	-3251	923 AN0282
1343	CONTIG5	428480	428906	1.67	9.24E-02	transcription_start_site	-	428778	428778	85	924 AN0283
1343	CONTIG5	428480	428906	1.67	9.24E-02	transcription_start_site	-	428454	428454	-239	925 AN0283
1343	CONTIG5	428480	428906	1.67	9.24E-02	transcription_start_site	-	428265	428265	-428	926 AN0283
1343	CONTIG5	428480	428906	1.67	9.24E-02	transcription_start_site	-	428174	428174	-519	927 AN0283
1343	CONTIG5	428480	428906	1.67	9.24E-02	transcription_start_site	-	427915	427915	-778	928 AN0283
1343	CONTIG5	428480	428906	1.67	9.24E-02	transcription_start_site	+	432041	432041	-3348	929 AN0284
1343	CONTIG5	428480	428906	1.67	9.24E-02	transcription_start_site	+	432553	432553	-3860	930 AN0284

1343	CONTIG5	428480	428906	1.67	9.24E-02	transcription_start_site	+	432972	432972	-4279	931	AN0284
1343	CONTIG5	428480	428906	1.67	9.24E-02	transcription_start_site	+	433095	433095	-4402	932	AN0284
1343	CONTIG5	428480	428906	1.67	9.24E-02	transcription_start_site	+	433320	433320	-4627	933	AN0284
1343	CONTIG5	428480	428906	1.67	9.24E-02	transcription_start_site	+	433608	433608	-4915	934	AN0284
910	CONTIG5	440266	440840	2	3.70E-02	transcription_start_site	-	437181	437181	-3372	937	AN0285
910	CONTIG5	440266	440840	2	3.70E-02	transcription_start_site	+	441254	441254	-701	940	AN0287
910	CONTIG5	440266	440840	2	3.70E-02	transcription_start_site	+	441506	441506	-953	941	AN0287
910	CONTIG5	440266	440840	2	3.70E-02	transcription_start_site	+	444319	444319	-3766	942	AN0287
910	CONTIG5	440266	440840	2	3.70E-02	transcription_start_site	+	444642	444642	-4089	943	AN0287
910	CONTIG5	440266	440840	2	3.70E-02	transcription_start_site	+	444840	444840	-4287	944	AN0287
1814	CONTIG5	447905	448719	1.45	1.50E-01	transcription_start_site	+	448077	448077	235	946	AN0288
2257	CONTIG6	3761	4035	1.28	9.24E-02	transcription_start_site	+	3961	3961	-63	949	AN0290
2257	CONTIG6	3761	4035	1.28	9.24E-02	transcription_start_site	+	3809	3809	89	948	AN0290
2257	CONTIG6	3761	4035	1.28	9.24E-02	transcription_start_site	+	4403	4403	-505	950	AN0290
2257	CONTIG6	3761	4035	1.28	9.24E-02	transcription_start_site	+	4731	4731	-833	951	AN0290
2257	CONTIG6	3761	4035	1.28	9.24E-02	transcription_start_site	+	4831	4831	-933	952	AN0290
2257	CONTIG6	3761	4035	1.28	9.24E-02	transcription_start_site	+	6869	6869	-2971	955	AN0292
2257	CONTIG6	3761	4035	1.28	9.24E-02	transcription_start_site	+	7682	7682	-3784	956	AN0292
1593	CONTIG6	17782	18066	1.54	3.70E-02	transcription_start_site	-	15232	15232	-2692	964	AN0295
1593	CONTIG6	17782	18066	1.54	3.70E-02	transcription_start_site	-	15061	15061	-2863	965	AN0295
1593	CONTIG6	17782	18066	1.54	3.70E-02	transcription_start_site	-	14879	14879	-3045	966	AN0295
1593	CONTIG6	17782	18066	1.54	3.70E-02	transcription_start_site	-	13345	13345	-4579	967	AN0295
1593	CONTIG6	17782	18066	1.54	3.70E-02	transcription_start_site	-	17537	17537	-387	969	AN0297
1593	CONTIG6	17782	18066	1.54	3.70E-02	transcription_start_site	-	17440	17440	-484	970	AN0297
1593	CONTIG6	17782	18066	1.54	3.70E-02	transcription_start_site	-	17090	17090	-834	971	AN0297
1593	CONTIG6	17782	18066	1.54	3.70E-02	transcription_start_site	-	16846	16846	-1078	972	AN0297
1809	CONTIG6	24019	24278	1.45	5.22E-02	transcription_start_site	-	19680	19680	-4468	973	AN0298
1593	CONTIG6	17782	18066	1.54	3.70E-02	transcription_start_site	+	21358	21358	-3434	974	AN0299
1593	CONTIG6	17782	18066	1.54	3.70E-02	transcription_start_site	+	21627	21627	-3703	975	AN0299
1593	CONTIG6	17782	18066	1.54	3.70E-02	transcription_start_site	+	21720	21720	-3796	976	AN0299
1593	CONTIG6	17782	18066	1.54	3.70E-02	transcription_start_site	+	22003	22003	-4079	977	AN0299
374	CONTIG6	27544	27818	2.65	2.67E-04	transcription_start_site	-	24598	24598	-3083	978	AN0300
374	CONTIG6	27544	27818	2.65	2.67E-04	transcription_start_site	-	24340	24340	-3341	979	AN0300
1809	CONTIG6	24019	24278	1.45	5.22E-02	transcription_start_site	-	24340	24340	191	979	AN0300
1809	CONTIG6	24019	24278	1.45	5.22E-02	transcription_start_site	-	24598	24598	449	978	AN0300
374	CONTIG6	27544	27818	2.65	2.67E-04	transcription_start_site	-	26380	26380	-1301	980	AN0301
374	CONTIG6	27544	27818	2.65	2.67E-04	transcription_start_site	-	26175	26175	-1506	981	AN0301
374	CONTIG6	27544	27818	2.65	2.67E-04	transcription_start_site	-	25706	25706	-1975	982	AN0301
374	CONTIG6	27544	27818	2.65	2.67E-04	transcription_start_site	-	25527	25527	-2154	983	AN0301
986	CONTIG6	32254	32528	1.92	7.85E-03	transcription_start_site	-	31222	31222	-1169	984	AN0302
986	CONTIG6	32254	32528	1.92	7.85E-03	transcription_start_site	-	29119	29119	-3272	985	AN0302
2732	CONTIG6	38109	38378	1.11	1.66E-01	transcription_start_site	-	34649	34649	-3594	986	AN0303
2732	CONTIG6	38109	38378	1.11	1.66E-01	transcription_start_site	-	34553	34553	-3690	987	AN0303
2732	CONTIG6	38109	38378	1.11	1.66E-01	transcription_start_site	-	34151	34151	-4092	988	AN0303
2732	CONTIG6	38109	38378	1.11	1.66E-01	transcription_start_site	-	33934	33934	-4309	989	AN0303
986	CONTIG6	32254	32528	1.92	7.85E-03	transcription_start_site	+	35457	35457	-3066	990	AN0304
986	CONTIG6	32254	32528	1.92	7.85E-03	transcription_start_site	+	35793	35793	-3402	991	AN0304
986	CONTIG6	32254	32528	1.92	7.85E-03	transcription_start_site	+	36396	36396	-4005	992	AN0304
2732	CONTIG6	38109	38378	1.11	1.66E-01	transcription_start_site	+	41011	41011	-2767	996	AN0306
2732	CONTIG6	38109	38378	1.11	1.66E-01	transcription_start_site	+	41146	41146	-2902	997	AN0306
2732	CONTIG6	38109	38378	1.11	1.66E-01	transcription_start_site	+	41868	41868	-3624	998	AN0306
2732	CONTIG6	38109	38378	1.11	1.66E-01	transcription_start_site	+	42020	42020	-3776	999	AN0306
2732	CONTIG6	38109	38378	1.11	1.66E-01	transcription_start_site	+	42120	42120	-3876	1000	AN0306
343	CONTIG6	55513	55867	2.69	0.00E+00	transcription_start_site	-	51985	51985	-3705	1003	AN0309
343	CONTIG6	55513	55867	2.69	0.00E+00	transcription_start_site	-	51888	51888	-3802	1004	AN0309
2258	CONTIG6	53268	53607	1.28	9.24E-02	transcription_start_site	-	51985	51985	-1452	1003	AN0309
2258	CONTIG6	53268	53607	1.28	9.24E-02	transcription_start_site	-	51888	51888	-1549	1004	AN0309

2258	CONTIG6	53268	53607	1.28	9.24E-02	transcription_start_site	+	52639	52639	798	1006	AN0310
2258	CONTIG6	53268	53607	1.28	9.24E-02	transcription_start_site	+	52334	52334	1103	1005	AN0310
343	CONTIG6	55513	55867	2.69	0.00E+00	transcription_start_site	+	56422	56422	-732	1007	AN0311
2258	CONTIG6	53268	53607	1.28	9.24E-02	transcription_start_site	+	56422	56422	-2984	1007	AN0311
343	CONTIG6	55513	55867	2.69	0.00E+00	transcription_start_site	+	58110	58110	-2420	1008	AN0312
2258	CONTIG6	53268	53607	1.28	9.24E-02	transcription_start_site	+	58110	58110	-4672	1008	AN0312
1153	CONTIG6	61804	62394	1.79	4.13E-03	transcription_start_site	-	61540	61540	-559	1009	AN0313
1153	CONTIG6	61804	62394	1.79	4.13E-03	transcription_start_site	+	62425	62425	-326	1010	AN0314
2635	CONTIG6	59333	59612	1.15	1.44E-01	transcription_start_site	+	62425	62425	-2952	1010	AN0314
2135	CONTIG6	66920	67259	1.32	8.01E-02	transcription_start_site	-	66575	66575	-514	1011	AN0315
2135	CONTIG6	66920	67259	1.32	8.01E-02	transcription_start_site	-	66223	66223	-866	1012	AN0315
2376	CONTIG6	71180	71469	1.24	1.08E-01	transcription_start_site	-	66575	66575	-4749	1011	AN0315
2376	CONTIG6	71180	71469	1.24	1.08E-01	transcription_start_site	-	66223	66223	-5101	1012	AN0315
1153	CONTIG6	61804	62394	1.79	4.13E-03	transcription_start_site	+	67386	67386	-5287	1013	AN0316
2135	CONTIG6	66920	67259	1.32	8.01E-02	transcription_start_site	+	67386	67386	-296	1013	AN0316
2135	CONTIG6	66920	67259	1.32	8.01E-02	transcription_start_site	+	67474	67474	-384	1014	AN0316
2135	CONTIG6	66920	67259	1.32	8.01E-02	transcription_start_site	+	67622	67622	-532	1015	AN0316
2135	CONTIG6	66920	67259	1.32	8.01E-02	transcription_start_site	+	67755	67755	-665	1016	AN0316
2135	CONTIG6	66920	67259	1.32	8.01E-02	transcription_start_site	+	67900	67900	-810	1017	AN0316
2135	CONTIG6	66920	67259	1.32	8.01E-02	transcription_start_site	+	68911	68911	-1821	1018	AN0316
2135	CONTIG6	66920	67259	1.32	8.01E-02	transcription_start_site	+	69872	69872	-2782	1019	AN0317
2135	CONTIG6	66920	67259	1.32	8.01E-02	transcription_start_site	+	70003	70003	-2913	1020	AN0317
2135	CONTIG6	66920	67259	1.32	8.01E-02	transcription_start_site	+	70233	70233	-3143	1021	AN0317
2135	CONTIG6	66920	67259	1.32	8.01E-02	transcription_start_site	+	70477	70477	-3387	1022	AN0317
2135	CONTIG6	66920	67259	1.32	8.01E-02	transcription_start_site	+	70694	70694	-3604	1023	AN0317
2376	CONTIG6	71180	71469	1.24	1.08E-01	transcription_start_site	+	70694	70694	630	1023	AN0317
2376	CONTIG6	71180	71469	1.24	1.08E-01	transcription_start_site	+	70477	70477	847	1022	AN0317
2376	CONTIG6	71180	71469	1.24	1.08E-01	transcription_start_site	+	70233	70233	1091	1021	AN0317
2376	CONTIG6	71180	71469	1.24	1.08E-01	transcription_start_site	+	74054	74054	-2729	1024	AN0317
1717	CONTIG6	106880	107304	1.49	4.36E-02	transcription_start_site	-	104606	104606	-2486	1042	AN0323
1717	CONTIG6	106880	107304	1.49	4.36E-02	transcription_start_site	+	107815	107815	-723	1043	AN0324
1717	CONTIG6	106880	107304	1.49	4.36E-02	transcription_start_site	+	107930	107930	-838	1044	AN0324
1717	CONTIG6	106880	107304	1.49	4.36E-02	transcription_start_site	+	109711	109711	-2619	1045	AN0325
1717	CONTIG6	106880	107304	1.49	4.36E-02	transcription_start_site	+	109951	109951	-2859	1046	AN0325
1717	CONTIG6	106880	107304	1.49	4.36E-02	transcription_start_site	+	110081	110081	-2989	1047	AN0325
1717	CONTIG6	106880	107304	1.49	4.36E-02	transcription_start_site	+	110317	110317	-3225	1048	AN0325
1717	CONTIG6	106880	107304	1.49	4.36E-02	transcription_start_site	+	110580	110580	-3488	1049	AN0325
1717	CONTIG6	106880	107304	1.49	4.36E-02	transcription_start_site	+	110969	110969	-3877	1050	AN0325
2259	CONTIG6	120467	121556	1.28	9.24E-02	transcription_start_site	-	116981	116981	-4030	1055	AN0328
1352	CONTIG6	126467	126816	1.66	2.12E-02	transcription_start_site	-	121609	121609	-5032	1061	AN0330
2259	CONTIG6	120467	121556	1.28	9.24E-02	transcription_start_site	-	121609	121609	597	1061	AN0330
1352	CONTIG6	126467	126816	1.66	2.12E-02	transcription_start_site	-	122692	122692	-3949	1062	AN0331
1352	CONTIG6	126467	126816	1.66	2.12E-02	transcription_start_site	-	122503	122503	-4138	1063	AN0331
1352	CONTIG6	126467	126816	1.66	2.12E-02	transcription_start_site	-	122349	122349	-4292	1064	AN0331
2259	CONTIG6	120467	121556	1.28	9.24E-02	transcription_start_site	-	122349	122349	1337	1064	AN0331
2259	CONTIG6	120467	121556	1.28	9.24E-02	transcription_start_site	-	122503	122503	1491	1063	AN0331
1352	CONTIG6	126467	126816	1.66	2.12E-02	transcription_start_site	-	125370	125370	-1271	1065	AN0332
1352	CONTIG6	126467	126816	1.66	2.12E-02	transcription_start_site	-	125046	125046	-1595	1066	AN0332
1352	CONTIG6	126467	126816	1.66	2.12E-02	transcription_start_site	-	124665	124665	-1976	1067	AN0332
1352	CONTIG6	126467	126816	1.66	2.12E-02	transcription_start_site	-	124449	124449	-2192	1068	AN0332
1352	CONTIG6	126467	126816	1.66	2.12E-02	transcription_start_site	-	123721	123721	-2920	1069	AN0332
1352	CONTIG6	126467	126816	1.66	2.12E-02	transcription_start_site	+	127333	127333	-691	1070	AN0333
1352	CONTIG6	126467	126816	1.66	2.12E-02	transcription_start_site	+	127688	127688	-1046	1071	AN0333
1352	CONTIG6	126467	126816	1.66	2.12E-02	transcription_start_site	+	128045	128045	-1403	1072	AN0333
1352	CONTIG6	126467	126816	1.66	2.12E-02	transcription_start_site	+	128199	128199	-1557	1073	AN0333
1106	CONTIG6	146493	146832	1.83	1.11E-02	transcription_start_site	-	142068	142068	-4594	1085	AN0338
1106	CONTIG6	146493	146832	1.83	1.11E-02	transcription_start_site	-	141761	141761	-4901	1086	AN0338

1106	CONTIG6	146493	146832	1.83	1.11E-02	transcription_start_site	-	146405	146405	-257	1094	AN0339
1106	CONTIG6	146493	146832	1.83	1.11E-02	transcription_start_site	-	144008	144008	-2654	1095	AN0339
1106	CONTIG6	146493	146832	1.83	1.11E-02	transcription_start_site	-	143720	143720	-2942	1096	AN0339
1106	CONTIG6	146493	146832	1.83	1.11E-02	transcription_start_site	+	147198	147198	-535	1097	AN0340
1106	CONTIG6	146493	146832	1.83	1.11E-02	transcription_start_site	+	147896	147896	-1233	1098	AN0340
2799	CONTIG6	175746	176170	1.07	1.93E-01	transcription_start_site	-	172233	172233	-3725	1119	AN0349
2799	CONTIG6	175746	176170	1.07	1.93E-01	transcription_start_site	-	171681	171681	-4277	1120	AN0349
2799	CONTIG6	175746	176170	1.07	1.93E-01	transcription_start_site	-	173521	173521	-2437	1121	AN0350
2799	CONTIG6	175746	176170	1.07	1.93E-01	transcription_start_site	-	173471	173471	-2487	1122	AN0350
2799	CONTIG6	175746	176170	1.07	1.93E-01	transcription_start_site	+	175507	175507	451	1125	AN0351
238	CONTIG6	184804	185093	2.86	0.00E+00	transcription_start_site	+	185614	185614	-665	1126	AN0353
238	CONTIG6	184804	185093	2.86	0.00E+00	transcription_start_site	+	186068	186068	-1119	1127	AN0353
2260	CONTIG6	185114	185753	1.28	9.24E-02	transcription_start_site	+	185614	185614	-180	1126	AN0353
2260	CONTIG6	185114	185753	1.28	9.24E-02	transcription_start_site	+	186068	186068	-634	1127	AN0353
2730	CONTIG6	194946	195357	1.11	1.50E-01	transcription_start_site	-	190006	190006	-5145	1128	AN0354
2730	CONTIG6	194946	195357	1.11	1.50E-01	transcription_start_site	-	189947	189947	-5204	1129	AN0354
1810	CONTIG6	198021	198275	1.45	5.22E-02	transcription_start_site	-	193155	193155	-4993	1132	AN0355
2730	CONTIG6	194946	195357	1.11	1.50E-01	transcription_start_site	-	193155	193155	-1996	1132	AN0355
2730	CONTIG6	194946	195357	1.11	1.50E-01	transcription_start_site	-	190709	190709	-4442	1133	AN0355
2730	CONTIG6	194946	195357	1.11	1.50E-01	transcription_start_site	+	195290	195290	-138	1134	AN0356
1810	CONTIG6	198021	198275	1.45	5.22E-02	transcription_start_site	-	198199	198199	51	1135	AN0357
1810	CONTIG6	198021	198275	1.45	5.22E-02	transcription_start_site	-	198014	198014	-134	1136	AN0357
1810	CONTIG6	198021	198275	1.45	5.22E-02	transcription_start_site	-	197753	197753	-395	1137	AN0357
1810	CONTIG6	198021	198275	1.45	5.22E-02	transcription_start_site	-	197625	197625	-523	1138	AN0357
1810	CONTIG6	198021	198275	1.45	5.22E-02	transcription_start_site	-	197173	197173	-975	1139	AN0357
987	CONTIG6	208818	209157	1.92	7.85E-03	transcription_start_site	-	206309	206309	-2678	1140	AN0359
987	CONTIG6	208818	209157	1.92	7.85E-03	transcription_start_site	-	206164	206164	-2823	1141	AN0359
987	CONTIG6	208818	209157	1.92	7.85E-03	transcription_start_site	-	205900	205900	-3087	1142	AN0359
987	CONTIG6	208818	209157	1.92	7.85E-03	transcription_start_site	-	204322	204322	-4665	1143	AN0359
2377	CONTIG6	210528	211332	1.24	1.08E-01	transcription_start_site	-	206309	206309	-4621	1140	AN0359
2377	CONTIG6	210528	211332	1.24	1.08E-01	transcription_start_site	-	206164	206164	-4766	1141	AN0359
2377	CONTIG6	210528	211332	1.24	1.08E-01	transcription_start_site	-	205900	205900	-5030	1142	AN0359
987	CONTIG6	208818	209157	1.92	7.85E-03	transcription_start_site	+	208130	208130	857	1148	AN0360
987	CONTIG6	208818	209157	1.92	7.85E-03	transcription_start_site	+	208016	208016	971	1147	AN0360
370	CONTIG6	217968	218612	2.65	0.00E+00	transcription_start_site	-	214975	214975	-3315	1149	AN0362
370	CONTIG6	217968	218612	2.65	0.00E+00	transcription_start_site	-	214657	214657	-3633	1150	AN0362
1154	CONTIG6	218720	219519	1.79	4.13E-03	transcription_start_site	-	214975	214975	-4144	1149	AN0362
1154	CONTIG6	218720	219519	1.79	4.13E-03	transcription_start_site	-	214657	214657	-4462	1150	AN0362
370	CONTIG6	217968	218612	2.65	0.00E+00	transcription_start_site	-	217321	217321	-969	1151	AN0363
370	CONTIG6	217968	218612	2.65	0.00E+00	transcription_start_site	-	217035	217035	-1255	1152	AN0363
370	CONTIG6	217968	218612	2.65	0.00E+00	transcription_start_site	-	216902	216902	-1388	1153	AN0363
370	CONTIG6	217968	218612	2.65	0.00E+00	transcription_start_site	-	216340	216340	-1950	1154	AN0363
1154	CONTIG6	218720	219519	1.79	4.13E-03	transcription_start_site	-	217321	217321	-1798	1151	AN0363
1154	CONTIG6	218720	219519	1.79	4.13E-03	transcription_start_site	-	217035	217035	-2084	1152	AN0363
1154	CONTIG6	218720	219519	1.79	4.13E-03	transcription_start_site	-	216902	216902	-2217	1153	AN0363
1154	CONTIG6	218720	219519	1.79	4.13E-03	transcription_start_site	-	216340	216340	-2779	1154	AN0363
1902	CONTIG6	221263	221537	1.41	5.88E-02	transcription_start_site	-	217321	217321	-4079	1151	AN0363
1902	CONTIG6	221263	221537	1.41	5.88E-02	transcription_start_site	-	217035	217035	-4365	1152	AN0363
1902	CONTIG6	221263	221537	1.41	5.88E-02	transcription_start_site	-	216902	216902	-4498	1153	AN0363
1902	CONTIG6	221263	221537	1.41	5.88E-02	transcription_start_site	-	216340	216340	-5060	1154	AN0363
370	CONTIG6	217968	218612	2.65	0.00E+00	transcription_start_site	+	222001	222001	-3711	1155	AN0364
370	CONTIG6	217968	218612	2.65	0.00E+00	transcription_start_site	+	222225	222225	-3935	1156	AN0364
1154	CONTIG6	218720	219519	1.79	4.13E-03	transcription_start_site	+	222001	222001	-2881	1155	AN0364
1154	CONTIG6	218720	219519	1.79	4.13E-03	transcription_start_site	+	222225	222225	-3105	1156	AN0364
1902	CONTIG6	221263	221537	1.41	5.88E-02	transcription_start_site	+	222001	222001	-601	1155	AN0364
1902	CONTIG6	221263	221537	1.41	5.88E-02	transcription_start_site	+	222225	222225	-825	1156	AN0364
1594	CONTIG6	230478	231057	1.54	3.70E-02	transcription_start_site	-	228027	228027	-2740	1160	AN0366

1594	CONTIG6	230478	231057	1.54	3.70E-02	transcription_start_site	-	227844	227844	-2923	1161	AN0366
1594	CONTIG6	230478	231057	1.54	3.70E-02	transcription_start_site	-	227578	227578	-3189	1162	AN0366
1594	CONTIG6	230478	231057	1.54	3.70E-02	transcription_start_site	-	230262	230262	-505	1163	AN0367
1594	CONTIG6	230478	231057	1.54	3.70E-02	transcription_start_site	+	232725	232725	-1957	1164	AN0368
1594	CONTIG6	230478	231057	1.54	3.70E-02	transcription_start_site	+	233145	233145	-2377	1165	AN0368
1594	CONTIG6	230478	231057	1.54	3.70E-02	transcription_start_site	+	235450	235450	-4682	1166	AN0368
2531	CONTIG6	244884	245228	1.19	1.25E-01	transcription_start_site	-	244851	244851	-205	1178	AN0372
2531	CONTIG6	244884	245228	1.19	1.25E-01	transcription_start_site	-	244723	244723	-333	1179	AN0372
2531	CONTIG6	244884	245228	1.19	1.25E-01	transcription_start_site	-	244622	244622	-434	1180	AN0372
2531	CONTIG6	244884	245228	1.19	1.25E-01	transcription_start_site	-	244540	244540	-516	1181	AN0372
2531	CONTIG6	244884	245228	1.19	1.25E-01	transcription_start_site	-	244295	244295	-761	1182	AN0372
2531	CONTIG6	244884	245228	1.19	1.25E-01	transcription_start_site	-	244190	244190	-866	1183	AN0372
2531	CONTIG6	244884	245228	1.19	1.25E-01	transcription_start_site	-	243982	243982	-1074	1184	AN0372
540	CONTIG7	3226	3595	2.39	1.01E-03	transcription_start_site	-	2045	2045	-1365	1197	AN0377
540	CONTIG7	3226	3595	2.39	1.01E-03	transcription_start_site	-	1460	1460	-1950	1198	AN0377
1521	CONTIG7	6921	7260	1.57	3.70E-02	transcription_start_site	-	2045	2045	-5045	1197	AN0377
540	CONTIG7	3226	3595	2.39	1.01E-03	transcription_start_site	+	2537	2537	873	1200	AN0378
540	CONTIG7	3226	3595	2.39	1.01E-03	transcription_start_site	+	2372	2372	1038	1199	AN0378
540	CONTIG7	3226	3595	2.39	1.01E-03	transcription_start_site	+	4189	4189	-778	1201	AN0379
540	CONTIG7	3226	3595	2.39	1.01E-03	transcription_start_site	+	4806	4806	-1395	1202	AN0379
1521	CONTIG7	6921	7260	1.57	3.70E-02	transcription_start_site	-	7042	7042	-48	1205	AN0380
1521	CONTIG7	6921	7260	1.57	3.70E-02	transcription_start_site	-	7463	7463	372	1204	AN0380
1521	CONTIG7	6921	7260	1.57	3.70E-02	transcription_start_site	-	7587	7587	496	1203	AN0380
540	CONTIG7	3226	3595	2.39	1.01E-03	transcription_start_site	+	7815	7815	-4404	1206	AN0381
540	CONTIG7	3226	3595	2.39	1.01E-03	transcription_start_site	+	7872	7872	-4461	1207	AN0381
540	CONTIG7	3226	3595	2.39	1.01E-03	transcription_start_site	+	8080	8080	-4669	1208	AN0381
1521	CONTIG7	6921	7260	1.57	3.70E-02	transcription_start_site	+	7815	7815	-724	1206	AN0381
1521	CONTIG7	6921	7260	1.57	3.70E-02	transcription_start_site	+	7872	7872	-781	1207	AN0381
1521	CONTIG7	6921	7260	1.57	3.70E-02	transcription_start_site	+	8080	8080	-989	1208	AN0381
1521	CONTIG7	6921	7260	1.57	3.70E-02	transcription_start_site	+	9505	9505	-2414	1209	AN0381
2437	CONTIG7	24021	24280	1.22	1.25E-01	transcription_start_site	-	19333	19333	-4817	1221	AN0385
2437	CONTIG7	24021	24280	1.22	1.25E-01	transcription_start_site	-	19186	19186	-4964	1222	AN0385
2437	CONTIG7	24021	24280	1.22	1.25E-01	transcription_start_site	-	20898	20898	-3252	1223	AN0386
2437	CONTIG7	24021	24280	1.22	1.25E-01	transcription_start_site	+	23984	23984	166	1226	AN0388
2437	CONTIG7	24021	24280	1.22	1.25E-01	transcription_start_site	+	24809	24809	-658	1227	AN0388
1001	CONTIG7	26486	26920	1.91	8.81E-03	transcription_start_site	+	27656	27656	-953	1228	AN0389
1288	CONTIG7	27990	28269	1.7	2.12E-02	transcription_start_site	+	27656	27656	473	1228	AN0389
2437	CONTIG7	24021	24280	1.22	1.25E-01	transcription_start_site	+	27656	27656	-3505	1228	AN0389
811	CONTIG7	30827	31201	2.09	4.61E-03	transcription_start_site	+	30528	30528	486	1231	AN0390
811	CONTIG7	30827	31201	2.09	4.61E-03	transcription_start_site	+	30180	30180	834	1230	AN0390
1001	CONTIG7	26486	26920	1.91	8.81E-03	transcription_start_site	+	29574	29574	-2871	1229	AN0390
1001	CONTIG7	26486	26920	1.91	8.81E-03	transcription_start_site	+	30180	30180	-3477	1230	AN0390
1001	CONTIG7	26486	26920	1.91	8.81E-03	transcription_start_site	+	30528	30528	-3825	1231	AN0390
1288	CONTIG7	27990	28269	1.7	2.12E-02	transcription_start_site	+	29574	29574	-1444	1229	AN0390
1288	CONTIG7	27990	28269	1.7	2.12E-02	transcription_start_site	+	30180	30180	-2050	1230	AN0390
1288	CONTIG7	27990	28269	1.7	2.12E-02	transcription_start_site	+	30528	30528	-2398	1231	AN0390
811	CONTIG7	30827	31201	2.09	4.61E-03	transcription_start_site	+	31227	31227	-213	1232	AN0391
811	CONTIG7	30827	31201	2.09	4.61E-03	transcription_start_site	+	31643	31643	-629	1233	AN0391
1001	CONTIG7	26486	26920	1.91	8.81E-03	transcription_start_site	+	31227	31227	-4524	1232	AN0391
1001	CONTIG7	26486	26920	1.91	8.81E-03	transcription_start_site	+	31643	31643	-4940	1233	AN0391
1288	CONTIG7	27990	28269	1.7	2.12E-02	transcription_start_site	+	31227	31227	-3097	1232	AN0391
1288	CONTIG7	27990	28269	1.7	2.12E-02	transcription_start_site	+	31643	31643	-3513	1233	AN0391
2055	CONTIG7	39902	40327	1.35	8.01E-02	transcription_start_site	+	39124	39124	990	1240	AN0392
1108	CONTIG7	41327	41676	1.83	1.30E-02	transcription_start_site	-	41261	41261	-240	1241	AN0393
1108	CONTIG7	41327	41676	1.83	1.30E-02	transcription_start_site	-	41098	41098	-403	1242	AN0393
1108	CONTIG7	41327	41676	1.83	1.30E-02	transcription_start_site	-	40758	40758	-743	1243	AN0393
2055	CONTIG7	39902	40327	1.35	8.01E-02	transcription_start_site	-	40758	40758	643	1243	AN0393

2055	CONTIG7	39902	40327	1.35	8.01E-02	transcription_start_site	-	41098	41098	983	1242 AN0393
2055	CONTIG7	39902	40327	1.35	8.01E-02	transcription_start_site	-	41261	41261	1146	1241 AN0393
2762	CONTIG7	40442	41226	1.09	1.93E-01	transcription_start_site	-	40758	40758	-76	1243 AN0393
2762	CONTIG7	40442	41226	1.09	1.93E-01	transcription_start_site	-	41098	41098	264	1242 AN0393
2762	CONTIG7	40442	41226	1.09	1.93E-01	transcription_start_site	-	41261	41261	427	1241 AN0393
1108	CONTIG7	41327	41676	1.83	1.30E-02	transcription_start_site	+	44009	44009	-2507	1244 AN0394
1108	CONTIG7	41327	41676	1.83	1.30E-02	transcription_start_site	+	44203	44203	-2701	1245 AN0394
1108	CONTIG7	41327	41676	1.83	1.30E-02	transcription_start_site	+	44727	44727	-3225	1246 AN0394
1108	CONTIG7	41327	41676	1.83	1.30E-02	transcription_start_site	+	45205	45205	-3703	1247 AN0394
1108	CONTIG7	41327	41676	1.83	1.30E-02	transcription_start_site	+	45336	45336	-3834	1248 AN0394
2055	CONTIG7	39902	40327	1.35	8.01E-02	transcription_start_site	+	44009	44009	-3894	1244 AN0394
2055	CONTIG7	39902	40327	1.35	8.01E-02	transcription_start_site	+	44203	44203	-4088	1245 AN0394
2055	CONTIG7	39902	40327	1.35	8.01E-02	transcription_start_site	+	44727	44727	-4612	1246 AN0394
2055	CONTIG7	39902	40327	1.35	8.01E-02	transcription_start_site	+	45205	45205	-5090	1247 AN0394
2762	CONTIG7	40442	41226	1.09	1.93E-01	transcription_start_site	+	44009	44009	-3175	1244 AN0394
2762	CONTIG7	40442	41226	1.09	1.93E-01	transcription_start_site	+	44203	44203	-3369	1245 AN0394
2762	CONTIG7	40442	41226	1.09	1.93E-01	transcription_start_site	+	44727	44727	-3893	1246 AN0394
2762	CONTIG7	40442	41226	1.09	1.93E-01	transcription_start_site	+	45205	45205	-4371	1247 AN0394
2762	CONTIG7	40442	41226	1.09	1.93E-01	transcription_start_site	+	45336	45336	-4502	1248 AN0394
171	CONTIG7	49652	50081	3	0.00E+00	transcription_start_site	-	47101	47101	-2765	1249 AN0395
171	CONTIG7	49652	50081	3	0.00E+00	transcription_start_site	-	46884	46884	-2982	1250 AN0395
171	CONTIG7	49652	50081	3	0.00E+00	transcription_start_site	-	46339	46339	-3527	1251 AN0395
186	CONTIG7	51080	51594	2.96	0.00E+00	transcription_start_site	-	47101	47101	-4236	1249 AN0395
186	CONTIG7	51080	51594	2.96	0.00E+00	transcription_start_site	-	46884	46884	-4453	1250 AN0395
186	CONTIG7	51080	51594	2.96	0.00E+00	transcription_start_site	-	46339	46339	-4998	1251 AN0395
171	CONTIG7	49652	50081	3	0.00E+00	transcription_start_site	-	49329	49329	-537	1252 AN0396
171	CONTIG7	49652	50081	3	0.00E+00	transcription_start_site	-	48981	48981	-885	1253 AN0396
186	CONTIG7	51080	51594	2.96	0.00E+00	transcription_start_site	-	49329	49329	-2008	1252 AN0396
186	CONTIG7	51080	51594	2.96	0.00E+00	transcription_start_site	-	48981	48981	-2356	1253 AN0396
171	CONTIG7	49652	50081	3	0.00E+00	transcription_start_site	+	50073	50073	-206	1254 AN0397
171	CONTIG7	49652	50081	3	0.00E+00	transcription_start_site	+	50424	50424	-557	1255 AN0397
186	CONTIG7	51080	51594	2.96	0.00E+00	transcription_start_site	+	50424	50424	913	1255 AN0397
186	CONTIG7	51080	51594	2.96	0.00E+00	transcription_start_site	+	56344	56344	-5007	1260 AN0399
186	CONTIG7	51080	51594	2.96	0.00E+00	transcription_start_site	+	56566	56566	-5229	1261 AN0399
2330	CONTIG7	59420	59699	1.26	1.08E-01	transcription_start_site	+	59066	59066	493	1265 AN0400
2330	CONTIG7	59420	59699	1.26	1.08E-01	transcription_start_site	+	60050	60050	-490	1266 AN0401
2330	CONTIG7	59420	59699	1.26	1.08E-01	transcription_start_site	+	60162	60162	-602	1267 AN0401
2330	CONTIG7	59420	59699	1.26	1.08E-01	transcription_start_site	+	60298	60298	-738	1268 AN0401
2330	CONTIG7	59420	59699	1.26	1.08E-01	transcription_start_site	+	60620	60620	-1060	1269 AN0401
2330	CONTIG7	59420	59699	1.26	1.08E-01	transcription_start_site	+	60778	60778	-1218	1270 AN0401
2330	CONTIG7	59420	59699	1.26	1.08E-01	transcription_start_site	+	61661	61661	-2101	1271 AN0402
2330	CONTIG7	59420	59699	1.26	1.08E-01	transcription_start_site	+	61974	61974	-2414	1272 AN0402
2330	CONTIG7	59420	59699	1.26	1.08E-01	transcription_start_site	+	62157	62157	-2597	1273 AN0402
2330	CONTIG7	59420	59699	1.26	1.08E-01	transcription_start_site	+	62386	62386	-2826	1274 AN0402
1938	CONTIG7	64579	64858	1.39	6.84E-02	transcription_start_site	+	64507	64507	211	1275 AN0403
1938	CONTIG7	64579	64858	1.39	6.84E-02	transcription_start_site	+	65028	65028	-309	1276 AN0403
1938	CONTIG7	64579	64858	1.39	6.84E-02	transcription_start_site	+	65268	65268	-549	1277 AN0403
1938	CONTIG7	64579	64858	1.39	6.84E-02	transcription_start_site	+	65669	65669	-950	1278 AN0403
1938	CONTIG7	64579	64858	1.39	6.84E-02	transcription_start_site	+	66444	66444	-1725	1279 AN0403
2330	CONTIG7	59420	59699	1.26	1.08E-01	transcription_start_site	+	64507	64507	-4947	1275 AN0403
1938	CONTIG7	64579	64858	1.39	6.84E-02	transcription_start_site	+	67414	67414	-2695	1280 AN0404
1938	CONTIG7	64579	64858	1.39	6.84E-02	transcription_start_site	+	67565	67565	-2846	1281 AN0404
2186	CONTIG7	68265	68679	1.3	9.24E-02	transcription_start_site	+	67565	67565	907	1281 AN0404
2186	CONTIG7	68265	68679	1.3	9.24E-02	transcription_start_site	+	67414	67414	1058	1280 AN0404
1939	CONTIG7	70370	70854	1.39	6.84E-02	transcription_start_site	+	75789	75789	-5177	1287 AN0407
1940	CONTIG7	82218	82477	1.39	6.84E-02	transcription_start_site	-	82342	82342	-5	1292 AN0408
1940	CONTIG7	82218	82477	1.39	6.84E-02	transcription_start_site	-	80843	80843	-1504	1293 AN0408

1232	CONTIG7	91891	92245	1.74	1.84E-02	transcription_start_site	+	92593	92593	-525	1296 AN0410
1232	CONTIG7	91891	92245	1.74	1.84E-02	transcription_start_site	+	92844	92844	-776	1297 AN0410
1232	CONTIG7	91891	92245	1.74	1.84E-02	transcription_start_site	+	93409	93409	-1341	1298 AN0410
1232	CONTIG7	91891	92245	1.74	1.84E-02	transcription_start_site	+	93788	93788	-1720	1299 AN0410
315	CONTIG7	101344	101618	2.74	0.00E+00	transcription_start_site	-	97915	97915	-3566	1300 AN0411
315	CONTIG7	101344	101618	2.74	0.00E+00	transcription_start_site	-	97729	97729	-3752	1301 AN0411
315	CONTIG7	101344	101618	2.74	0.00E+00	transcription_start_site	-	97509	97509	-3972	1302 AN0411
315	CONTIG7	101344	101618	2.74	0.00E+00	transcription_start_site	-	97381	97381	-4100	1303 AN0411
315	CONTIG7	101344	101618	2.74	0.00E+00	transcription_start_site	-	97245	97245	-4236	1304 AN0411
315	CONTIG7	101344	101618	2.74	0.00E+00	transcription_start_site	-	96870	96870	-4611	1305 AN0411
536	CONTIG7	102154	102963	2.39	0.00E+00	transcription_start_site	-	97915	97915	-4643	1300 AN0411
536	CONTIG7	102154	102963	2.39	0.00E+00	transcription_start_site	-	97729	97729	-4829	1301 AN0411
536	CONTIG7	102154	102963	2.39	0.00E+00	transcription_start_site	-	97509	97509	-5049	1302 AN0411
536	CONTIG7	102154	102963	2.39	0.00E+00	transcription_start_site	-	97381	97381	-5177	1303 AN0411
536	CONTIG7	102154	102963	2.39	0.00E+00	transcription_start_site	-	97245	97245	-5313	1304 AN0411
1453	CONTIG7	99978	100267	1.61	3.08E-02	transcription_start_site	-	97915	97915	-2207	1300 AN0411
1453	CONTIG7	99978	100267	1.61	3.08E-02	transcription_start_site	-	97729	97729	-2393	1301 AN0411
1453	CONTIG7	99978	100267	1.61	3.08E-02	transcription_start_site	-	97509	97509	-2613	1302 AN0411
1453	CONTIG7	99978	100267	1.61	3.08E-02	transcription_start_site	-	97381	97381	-2741	1303 AN0411
1453	CONTIG7	99978	100267	1.61	3.08E-02	transcription_start_site	-	97245	97245	-2877	1304 AN0411
1453	CONTIG7	99978	100267	1.61	3.08E-02	transcription_start_site	-	96870	96870	-3252	1305 AN0411
315	CONTIG7	101344	101618	2.74	0.00E+00	transcription_start_site	-	100781	100781	-700	1306 AN0412
536	CONTIG7	102154	102963	2.39	0.00E+00	transcription_start_site	-	100781	100781	-1777	1306 AN0412
1289	CONTIG7	104569	104903	1.7	2.12E-02	transcription_start_site	-	100781	100781	-3955	1306 AN0412
1453	CONTIG7	99978	100267	1.61	3.08E-02	transcription_start_site	-	100781	100781	658	1306 AN0412
1289	CONTIG7	104569	104903	1.7	2.12E-02	transcription_start_site	-	104920	104920	184	1312 AN0413
1289	CONTIG7	104569	104903	1.7	2.12E-02	transcription_start_site	-	105185	105185	449	1311 AN0413
1289	CONTIG7	104569	104903	1.7	2.12E-02	transcription_start_site	-	105447	105447	711	1310 AN0413
1289	CONTIG7	104569	104903	1.7	2.12E-02	transcription_start_site	-	105736	105736	1000	1309 AN0413
541	CONTIG7	120846	121180	2.39	1.01E-03	transcription_start_site	+	121813	121813	-800	1315 AN0416
541	CONTIG7	120846	121180	2.39	1.01E-03	transcription_start_site	+	121951	121951	-938	1316 AN0416
541	CONTIG7	120846	121180	2.39	1.01E-03	transcription_start_site	+	122445	122445	-1432	1317 AN0416
541	CONTIG7	120846	121180	2.39	1.01E-03	transcription_start_site	+	122688	122688	-1675	1318 AN0416
541	CONTIG7	120846	121180	2.39	1.01E-03	transcription_start_site	+	123691	123691	-2678	1319 AN0417
541	CONTIG7	120846	121180	2.39	1.01E-03	transcription_start_site	+	123808	123808	-2795	1320 AN0417
541	CONTIG7	120846	121180	2.39	1.01E-03	transcription_start_site	+	124219	124219	-3206	1321 AN0417
2760	CONTIG7	159760	160335	1.09	1.79E-01	transcription_start_site	-	156041	156041	-4006	1353 AN0428
2760	CONTIG7	159760	160335	1.09	1.79E-01	transcription_start_site	-	155697	155697	-4350	1354 AN0428
2760	CONTIG7	159760	160335	1.09	1.79E-01	transcription_start_site	-	155620	155620	-4427	1355 AN0428
2760	CONTIG7	159760	160335	1.09	1.79E-01	transcription_start_site	-	155491	155491	-4556	1356 AN0428
2760	CONTIG7	159760	160335	1.09	1.79E-01	transcription_start_site	-	154986	154986	-5061	1357 AN0428
2760	CONTIG7	159760	160335	1.09	1.79E-01	transcription_start_site	-	159831	159831	-216	1360 AN0430
1290	CONTIG7	171920	172184	1.7	2.12E-02	transcription_start_site	-	169177	169177	-2875	1361 AN0432
1290	CONTIG7	171920	172184	1.7	2.12E-02	transcription_start_site	-	169054	169054	-2998	1362 AN0432
1290	CONTIG7	171920	172184	1.7	2.12E-02	transcription_start_site	-	168723	168723	-3329	1363 AN0432
1290	CONTIG7	171920	172184	1.7	2.12E-02	transcription_start_site	-	168585	168585	-3467	1364 AN0432
1290	CONTIG7	171920	172184	1.7	2.12E-02	transcription_start_site	-	171236	171236	-816	1365 AN0433
1290	CONTIG7	171920	172184	1.7	2.12E-02	transcription_start_site	-	171071	171071	-981	1366 AN0433
1290	CONTIG7	171920	172184	1.7	2.12E-02	transcription_start_site	-	170835	170835	-1217	1367 AN0433
632	CONTIG7	166821	167236	2.26	1.80E-03	transcription_start_site	+	172118	172118	-5089	1368 AN0434
1290	CONTIG7	171920	172184	1.7	2.12E-02	transcription_start_site	+	172118	172118	-66	1368 AN0434
1290	CONTIG7	171920	172184	1.7	2.12E-02	transcription_start_site	+	172733	172733	-681	1369 AN0434
1290	CONTIG7	171920	172184	1.7	2.12E-02	transcription_start_site	+	172863	172863	-811	1370 AN0434
1290	CONTIG7	171920	172184	1.7	2.12E-02	transcription_start_site	+	173066	173066	-1014	1371 AN0434
1290	CONTIG7	171920	172184	1.7	2.12E-02	transcription_start_site	+	173179	173179	-1127	1372 AN0434
1290	CONTIG7	171920	172184	1.7	2.12E-02	transcription_start_site	+	173654	173654	-1602	1373 AN0434
2763	CONTIG7	182795	183059	1.09	1.93E-01	transcription_start_site	-	182788	182788	-139	1382 AN0437

2763	CONTIG7	182795	183059	1.09	1.93E-01	transcription_start_site	-	182253	182253	-674	1383	AN0437
2763	CONTIG7	182795	183059	1.09	1.93E-01	transcription_start_site	-	182068	182068	-859	1384	AN0437
2763	CONTIG7	182795	183059	1.09	1.93E-01	transcription_start_site	-	181839	181839	-1088	1385	AN0437
2763	CONTIG7	182795	183059	1.09	1.93E-01	transcription_start_site	-	181738	181738	-1189	1386	AN0437
2763	CONTIG7	182795	183059	1.09	1.93E-01	transcription_start_site	-	181447	181447	-1480	1387	AN0437
2763	CONTIG7	182795	183059	1.09	1.93E-01	transcription_start_site	-	181188	181188	-1739	1388	AN0437
2763	CONTIG7	182795	183059	1.09	1.93E-01	transcription_start_site	-	180667	180667	-2260	1389	AN0437
2763	CONTIG7	182795	183059	1.09	1.93E-01	transcription_start_site	-	180576	180576	-2351	1390	AN0437
2763	CONTIG7	182795	183059	1.09	1.93E-01	transcription_start_site	+	183861	183861	-934	1391	AN0438
2763	CONTIG7	182795	183059	1.09	1.93E-01	transcription_start_site	+	185431	185431	-2504	1392	AN0439
2763	CONTIG7	182795	183059	1.09	1.93E-01	transcription_start_site	+	185535	185535	-2608	1393	AN0439
2763	CONTIG7	182795	183059	1.09	1.93E-01	transcription_start_site	+	186012	186012	-3085	1394	AN0439
2763	CONTIG7	182795	183059	1.09	1.93E-01	transcription_start_site	+	186209	186209	-3282	1395	AN0439
467	CONTIG7	208667	209011	2.48	8.69E-04	transcription_start_site	-	206763	206763	-2076	1424	AN0446
467	CONTIG7	208667	209011	2.48	8.69E-04	transcription_start_site	-	204624	204624	-4215	1425	AN0446
672	CONTIG7	209552	210216	2.22	2.22E-03	transcription_start_site	-	206763	206763	-3121	1424	AN0446
672	CONTIG7	209552	210216	2.22	2.22E-03	transcription_start_site	-	204624	204624	-5260	1425	AN0446
672	CONTIG7	209552	210216	2.22	2.22E-03	transcription_start_site	+	214579	214579	-4695	1429	AN0448
672	CONTIG7	209552	210216	2.22	2.22E-03	transcription_start_site	+	214698	214698	-4814	1430	AN0448
2764	CONTIG7	217889	218166	1.09	1.93E-01	transcription_start_site	+	218138	218138	-110	1434	AN0449
2764	CONTIG7	217889	218166	1.09	1.93E-01	transcription_start_site	+	217560	217560	467	1433	AN0449
2764	CONTIG7	217889	218166	1.09	1.93E-01	transcription_start_site	+	217163	217163	864	1432	AN0449
2764	CONTIG7	217889	218166	1.09	1.93E-01	transcription_start_site	+	219663	219663	-1635	1435	AN0449
2764	CONTIG7	217889	218166	1.09	1.93E-01	transcription_start_site	+	220525	220525	-2497	1436	AN0449
2764	CONTIG7	217889	218166	1.09	1.93E-01	transcription_start_site	+	220687	220687	-2659	1437	AN0449
2761	CONTIG7	221780	222132	1.09	1.79E-01	transcription_start_site	-	222770	222770	814	1438	AN0450
1442	CONTIG7	228546	230375	1.61	0.00E+00	transcription_start_site	-	223819	223819	-5641	1439	AN0451
2761	CONTIG7	221780	222132	1.09	1.79E-01	transcription_start_site	+	225514	225514	-3558	1442	AN0452
2761	CONTIG7	221780	222132	1.09	1.79E-01	transcription_start_site	+	225889	225889	-3933	1443	AN0452
1442	CONTIG7	228546	230375	1.61	0.00E+00	transcription_start_site	+	228238	228238	1222	1444	AN0453
1442	CONTIG7	228546	230375	1.61	0.00E+00	transcription_start_site	+	230649	230649	-1188	1445	AN0454
2438	CONTIG7	231231	231650	1.22	1.25E-01	transcription_start_site	+	230649	230649	791	1445	AN0454
2187	CONTIG7	240996	241270	1.3	9.24E-02	transcription_start_site	-	238468	238468	-2665	1446	AN0456
2688	CONTIG7	239041	239320	1.13	1.66E-01	transcription_start_site	-	238468	238468	-712	1446	AN0456
419	CONTIG7	236346	236620	2.57	6.10E-04	transcription_start_site	+	239098	239098	-2615	1447	AN0457
419	CONTIG7	236346	236620	2.57	6.10E-04	transcription_start_site	+	239291	239291	-2808	1448	AN0457
419	CONTIG7	236346	236620	2.57	6.10E-04	transcription_start_site	+	239400	239400	-2917	1449	AN0457
2688	CONTIG7	239041	239320	1.13	1.66E-01	transcription_start_site	+	239098	239098	82	1447	AN0457
2688	CONTIG7	239041	239320	1.13	1.66E-01	transcription_start_site	+	239291	239291	-110	1448	AN0457
2688	CONTIG7	239041	239320	1.13	1.66E-01	transcription_start_site	+	239400	239400	-219	1449	AN0457
2187	CONTIG7	240996	241270	1.3	9.24E-02	transcription_start_site	-	241256	241256	123	1451	AN0458
2187	CONTIG7	240996	241270	1.3	9.24E-02	transcription_start_site	-	241688	241688	555	1450	AN0458
2187	CONTIG7	240996	241270	1.3	9.24E-02	transcription_start_site	+	245428	245428	-4295	1454	AN0460
728	CONTIG7	268505	268869	2.17	3.03E-03	transcription_start_site	-	263810	263810	-4877	1463	AN0464
1742	CONTIG7	263495	264744	1.48	2.48E-02	transcription_start_site	-	263810	263810	-309	1463	AN0464
1742	CONTIG7	263495	264744	1.48	2.48E-02	transcription_start_site	-	263155	263155	-964	1464	AN0464
1742	CONTIG7	263495	264744	1.48	2.48E-02	transcription_start_site	-	262576	262576	-1543	1465	AN0464
2188	CONTIG7	265295	265578	1.3	9.24E-02	transcription_start_site	-	263810	263810	-1626	1463	AN0464
2188	CONTIG7	265295	265578	1.3	9.24E-02	transcription_start_site	-	263155	263155	-2281	1464	AN0464
2188	CONTIG7	265295	265578	1.3	9.24E-02	transcription_start_site	-	262576	262576	-2860	1465	AN0464
728	CONTIG7	268505	268869	2.17	3.03E-03	transcription_start_site	-	264836	264836	-3851	1466	AN0465
728	CONTIG7	268505	268869	2.17	3.03E-03	transcription_start_site	-	264704	264704	-3983	1467	AN0465
1742	CONTIG7	263495	264744	1.48	2.48E-02	transcription_start_site	-	264704	264704	584	1467	AN0465
1742	CONTIG7	263495	264744	1.48	2.48E-02	transcription_start_site	-	264836	264836	716	1466	AN0465
2188	CONTIG7	265295	265578	1.3	9.24E-02	transcription_start_site	-	264836	264836	-600	1466	AN0465
2188	CONTIG7	265295	265578	1.3	9.24E-02	transcription_start_site	-	264704	264704	-732	1467	AN0465
728	CONTIG7	268505	268869	2.17	3.03E-03	transcription_start_site	-	266867	266867	-1820	1468	AN0466

728	CONTIG7	268505	268869	2.17	3.03E-03	transcription_start_site	-	268067	268067	-620	1469	AN0467
728	CONTIG7	268505	268869	2.17	3.03E-03	transcription_start_site	-	267401	267401	-1286	1470	AN0467
728	CONTIG7	268505	268869	2.17	3.03E-03	transcription_start_site	+	269264	269264	-577	1471	AN0468
728	CONTIG7	268505	268869	2.17	3.03E-03	transcription_start_site	+	269857	269857	-1170	1472	AN0468
1742	CONTIG7	263495	264744	1.48	2.48E-02	transcription_start_site	+	269264	269264	-5144	1471	AN0468
2188	CONTIG7	265295	265578	1.3	9.24E-02	transcription_start_site	+	269264	269264	-3827	1471	AN0468
2188	CONTIG7	265295	265578	1.3	9.24E-02	transcription_start_site	+	269857	269857	-4420	1472	AN0468
2689	CONTIG7	278476	279220	1.13	1.66E-01	transcription_start_site	-	274556	274556	-4292	1473	AN0469
2689	CONTIG7	278476	279220	1.13	1.66E-01	transcription_start_site	-	274433	274433	-4415	1474	AN0469
2689	CONTIG7	278476	279220	1.13	1.66E-01	transcription_start_site	-	273866	273866	-4982	1475	AN0469
2689	CONTIG7	278476	279220	1.13	1.66E-01	transcription_start_site	-	273510	273510	-5338	1476	AN0469
134	CONTIG7	281941	282285	3.09	0.00E+00	transcription_start_site	-	279452	279452	-2661	1481	AN0470
134	CONTIG7	281941	282285	3.09	0.00E+00	transcription_start_site	-	279063	279063	-3050	1482	AN0470
134	CONTIG7	281941	282285	3.09	0.00E+00	transcription_start_site	-	278615	278615	-3498	1483	AN0470
2689	CONTIG7	278476	279220	1.13	1.66E-01	transcription_start_site	-	279063	279063	215	1482	AN0470
2689	CONTIG7	278476	279220	1.13	1.66E-01	transcription_start_site	-	278615	278615	-233	1483	AN0470
2689	CONTIG7	278476	279220	1.13	1.66E-01	transcription_start_site	-	279452	279452	604	1481	AN0470
134	CONTIG7	281941	282285	3.09	0.00E+00	transcription_start_site	-	281421	281421	-692	1484	AN0471
134	CONTIG7	281941	282285	3.09	0.00E+00	transcription_start_site	-	280981	280981	-1132	1485	AN0471
134	CONTIG7	281941	282285	3.09	0.00E+00	transcription_start_site	-	280085	280085	-2028	1486	AN0471
2689	CONTIG7	278476	279220	1.13	1.66E-01	transcription_start_site	-	280085	280085	1237	1486	AN0471
134	CONTIG7	281941	282285	3.09	0.00E+00	transcription_start_site	-	282961	282961	848	1493	AN0472
134	CONTIG7	281941	282285	3.09	0.00E+00	transcription_start_site	-	283269	283269	1156	1492	AN0472
670	CONTIG7	287786	288291	2.22	0.00E+00	transcription_start_site	-	285950	285950	-2088	1487	AN0472
670	CONTIG7	287786	288291	2.22	0.00E+00	transcription_start_site	-	285737	285737	-2301	1488	AN0472
670	CONTIG7	287786	288291	2.22	0.00E+00	transcription_start_site	-	285313	285313	-2725	1489	AN0472
670	CONTIG7	287786	288291	2.22	0.00E+00	transcription_start_site	-	285160	285160	-2878	1490	AN0472
670	CONTIG7	287786	288291	2.22	0.00E+00	transcription_start_site	-	283384	283384	-4654	1491	AN0472
670	CONTIG7	287786	288291	2.22	0.00E+00	transcription_start_site	-	283269	283269	-4769	1492	AN0472
670	CONTIG7	287786	288291	2.22	0.00E+00	transcription_start_site	-	282961	282961	-5077	1493	AN0472
670	CONTIG7	287786	288291	2.22	0.00E+00	transcription_start_site	+	288386	288386	-347	1494	AN0473
670	CONTIG7	287786	288291	2.22	0.00E+00	transcription_start_site	+	289096	289096	-1057	1495	AN0473
670	CONTIG7	287786	288291	2.22	0.00E+00	transcription_start_site	+	289410	289410	-1371	1496	AN0473
670	CONTIG7	287786	288291	2.22	0.00E+00	transcription_start_site	+	289590	289590	-1551	1497	AN0473
670	CONTIG7	287786	288291	2.22	0.00E+00	transcription_start_site	+	289762	289762	-1723	1498	AN0473
670	CONTIG7	287786	288291	2.22	0.00E+00	transcription_start_site	+	290681	290681	-2642	1499	AN0474
670	CONTIG7	287786	288291	2.22	0.00E+00	transcription_start_site	+	291206	291206	-3167	1500	AN0474
670	CONTIG7	287786	288291	2.22	0.00E+00	transcription_start_site	+	291364	291364	-3325	1501	AN0474
670	CONTIG7	287786	288291	2.22	0.00E+00	transcription_start_site	+	291637	291637	-3598	1502	AN0474
997	CONTIG7	321152	322044	1.91	4.17E-03	transcription_start_site	-	317332	317332	-4266	1530	AN0482
997	CONTIG7	321152	322044	1.91	4.17E-03	transcription_start_site	-	317253	317253	-4345	1531	AN0482
997	CONTIG7	321152	322044	1.91	4.17E-03	transcription_start_site	-	317119	317119	-4479	1532	AN0482
2316	CONTIG7	320117	321056	1.26	5.82E-02	transcription_start_site	-	317332	317332	-3254	1530	AN0482
2316	CONTIG7	320117	321056	1.26	5.82E-02	transcription_start_site	-	317253	317253	-3333	1531	AN0482
2316	CONTIG7	320117	321056	1.26	5.82E-02	transcription_start_site	-	317119	317119	-3467	1532	AN0482
568	CONTIG7	323782	324511	2.35	0.00E+00	transcription_start_site	-	319919	319919	-4227	1533	AN0483
568	CONTIG7	323782	324511	2.35	0.00E+00	transcription_start_site	-	319552	319552	-4594	1534	AN0483
997	CONTIG7	321152	322044	1.91	4.17E-03	transcription_start_site	-	319919	319919	-1679	1533	AN0483
997	CONTIG7	321152	322044	1.91	4.17E-03	transcription_start_site	-	319552	319552	-2046	1534	AN0483
2189	CONTIG7	322362	323376	1.3	9.24E-02	transcription_start_site	-	319919	319919	-2950	1533	AN0483
2189	CONTIG7	322362	323376	1.3	9.24E-02	transcription_start_site	-	319552	319552	-3317	1534	AN0483
2316	CONTIG7	320117	321056	1.26	5.82E-02	transcription_start_site	-	319919	319919	-667	1533	AN0483
2316	CONTIG7	320117	321056	1.26	5.82E-02	transcription_start_site	-	319552	319552	-1034	1534	AN0483
568	CONTIG7	323782	324511	2.35	0.00E+00	transcription_start_site	-	321486	321486	-2660	1535	AN0484
568	CONTIG7	323782	324511	2.35	0.00E+00	transcription_start_site	-	321383	321383	-2763	1536	AN0484
997	CONTIG7	321152	322044	1.91	4.17E-03	transcription_start_site	-	321486	321486	-112	1535	AN0484
997	CONTIG7	321152	322044	1.91	4.17E-03	transcription_start_site	-	321383	321383	-215	1536	AN0484

2189	CONTIG7	322362	323376	1.3	9.24E-02	transcription_start_site	-	321486	321486	-1383	1535	AN0484
2189	CONTIG7	322362	323376	1.3	9.24E-02	transcription_start_site	-	321383	321383	-1486	1536	AN0484
2316	CONTIG7	320117	321056	1.26	5.82E-02	transcription_start_site	-	321383	321383	796	1536	AN0484
2316	CONTIG7	320117	321056	1.26	5.82E-02	transcription_start_site	-	321486	321486	899	1535	AN0484
2435	CONTIG7	325652	326076	1.22	1.07E-01	transcription_start_site	-	321486	321486	-4378	1535	AN0484
2435	CONTIG7	325652	326076	1.22	1.07E-01	transcription_start_site	-	321383	321383	-4481	1536	AN0484
568	CONTIG7	323782	324511	2.35	0.00E+00	transcription_start_site	-	323981	323981	-165	1537	AN0485
568	CONTIG7	323782	324511	2.35	0.00E+00	transcription_start_site	-	323186	323186	-960	1538	AN0485
568	CONTIG7	323782	324511	2.35	0.00E+00	transcription_start_site	-	323096	323096	-1050	1539	AN0485
568	CONTIG7	323782	324511	2.35	0.00E+00	transcription_start_site	-	322947	322947	-1199	1540	AN0485
997	CONTIG7	321152	322044	1.91	4.17E-03	transcription_start_site	-	322947	322947	1349	1540	AN0485
2189	CONTIG7	322362	323376	1.3	9.24E-02	transcription_start_site	-	322947	322947	78	1540	AN0485
2189	CONTIG7	322362	323376	1.3	9.24E-02	transcription_start_site	-	323096	323096	227	1539	AN0485
2189	CONTIG7	322362	323376	1.3	9.24E-02	transcription_start_site	-	323186	323186	317	1538	AN0485
2189	CONTIG7	322362	323376	1.3	9.24E-02	transcription_start_site	-	323981	323981	1112	1537	AN0485
2435	CONTIG7	325652	326076	1.22	1.07E-01	transcription_start_site	-	323981	323981	-1883	1537	AN0485
2435	CONTIG7	325652	326076	1.22	1.07E-01	transcription_start_site	-	323186	323186	-2678	1538	AN0485
2435	CONTIG7	325652	326076	1.22	1.07E-01	transcription_start_site	-	323096	323096	-2768	1539	AN0485
2435	CONTIG7	325652	326076	1.22	1.07E-01	transcription_start_site	-	322947	322947	-2917	1540	AN0485
568	CONTIG7	323782	324511	2.35	0.00E+00	transcription_start_site	+	324896	324896	-749	1541	AN0486
568	CONTIG7	323782	324511	2.35	0.00E+00	transcription_start_site	+	325153	325153	-1006	1542	AN0486
568	CONTIG7	323782	324511	2.35	0.00E+00	transcription_start_site	+	325465	325465	-1318	1543	AN0486
568	CONTIG7	323782	324511	2.35	0.00E+00	transcription_start_site	+	327062	327062	-2915	1544	AN0486
997	CONTIG7	321152	322044	1.91	4.17E-03	transcription_start_site	+	324896	324896	-3298	1541	AN0486
997	CONTIG7	321152	322044	1.91	4.17E-03	transcription_start_site	+	325153	325153	-3555	1542	AN0486
997	CONTIG7	321152	322044	1.91	4.17E-03	transcription_start_site	+	325465	325465	-3867	1543	AN0486
2189	CONTIG7	322362	323376	1.3	9.24E-02	transcription_start_site	+	324896	324896	-2027	1541	AN0486
2189	CONTIG7	322362	323376	1.3	9.24E-02	transcription_start_site	+	325153	325153	-2284	1542	AN0486
2189	CONTIG7	322362	323376	1.3	9.24E-02	transcription_start_site	+	325465	325465	-2596	1543	AN0486
2189	CONTIG7	322362	323376	1.3	9.24E-02	transcription_start_site	+	327062	327062	-4193	1544	AN0486
2316	CONTIG7	320117	321056	1.26	5.82E-02	transcription_start_site	+	324896	324896	-4309	1541	AN0486
2316	CONTIG7	320117	321056	1.26	5.82E-02	transcription_start_site	+	325153	325153	-4566	1542	AN0486
2316	CONTIG7	320117	321056	1.26	5.82E-02	transcription_start_site	+	325465	325465	-4878	1543	AN0486
2435	CONTIG7	325652	326076	1.22	1.07E-01	transcription_start_site	+	325465	325465	399	1543	AN0486
2435	CONTIG7	325652	326076	1.22	1.07E-01	transcription_start_site	+	325153	325153	711	1542	AN0486
2435	CONTIG7	325652	326076	1.22	1.07E-01	transcription_start_site	+	324896	324896	968	1541	AN0486
2435	CONTIG7	325652	326076	1.22	1.07E-01	transcription_start_site	+	327062	327062	-1198	1544	AN0486
568	CONTIG7	323782	324511	2.35	0.00E+00	transcription_start_site	+	328327	328327	-4180	1545	AN0487
568	CONTIG7	323782	324511	2.35	0.00E+00	transcription_start_site	+	328430	328430	-4283	1546	AN0487
568	CONTIG7	323782	324511	2.35	0.00E+00	transcription_start_site	+	329181	329181	-5034	1547	AN0487
568	CONTIG7	323782	324511	2.35	0.00E+00	transcription_start_site	+	329385	329385	-5238	1548	AN0487
1747	CONTIG7	331662	332166	1.48	5.22E-02	transcription_start_site	+	330899	330899	1015	1551	AN0487
2189	CONTIG7	322362	323376	1.3	9.24E-02	transcription_start_site	+	328327	328327	-5458	1545	AN0487
2435	CONTIG7	325652	326076	1.22	1.07E-01	transcription_start_site	+	328327	328327	-2463	1545	AN0487
2435	CONTIG7	325652	326076	1.22	1.07E-01	transcription_start_site	+	328430	328430	-2566	1546	AN0487
2435	CONTIG7	325652	326076	1.22	1.07E-01	transcription_start_site	+	329181	329181	-3317	1547	AN0487
2435	CONTIG7	325652	326076	1.22	1.07E-01	transcription_start_site	+	329385	329385	-3521	1548	AN0487
2435	CONTIG7	325652	326076	1.22	1.07E-01	transcription_start_site	+	329806	329806	-3942	1549	AN0487
2435	CONTIG7	325652	326076	1.22	1.07E-01	transcription_start_site	+	330211	330211	-4347	1550	AN0487
2435	CONTIG7	325652	326076	1.22	1.07E-01	transcription_start_site	+	330899	330899	-5035	1551	AN0487
2765	CONTIG7	338792	339126	1.09	1.93E-01	transcription_start_site	-	338849	338849	-110	1553	AN0489
2765	CONTIG7	338792	339126	1.09	1.93E-01	transcription_start_site	-	339085	339085	126	1552	AN0489
2765	CONTIG7	338792	339126	1.09	1.93E-01	transcription_start_site	-	338286	338286	-673	1554	AN0489
2765	CONTIG7	338792	339126	1.09	1.93E-01	transcription_start_site	-	337234	337234	-1725	1555	AN0489
542	CONTIG7	351607	351966	2.39	1.01E-03	transcription_start_site	+	352429	352429	-642	1568	AN0493
946	CONTIG7	357842	358186	1.96	7.85E-03	transcription_start_site	-	357154	357154	-860	1569	AN0494
2056	CONTIG7	356117	356541	1.35	8.01E-02	transcription_start_site	-	357154	357154	825	1569	AN0494

946	CONTIG7	357842	358186	1.96	7.85E-03	transcription_start_site	+	358065	358065	-51	1570 AN0495
2056	CONTIG7	356117	356541	1.35	8.01E-02	transcription_start_site	+	358065	358065	-1736	1570 AN0495
2845	CONTIG7	390686	391791	1.04	1.64E-01	transcription_start_site	-	388663	388663	-2575	1591 AN0504
2845	CONTIG7	390686	391791	1.04	1.64E-01	transcription_start_site	-	388447	388447	-2791	1592 AN0504
2845	CONTIG7	390686	391791	1.04	1.64E-01	transcription_start_site	-	387993	387993	-3245	1593 AN0504
2845	CONTIG7	390686	391791	1.04	1.64E-01	transcription_start_site	-	387612	387612	-3626	1594 AN0504
2845	CONTIG7	390686	391791	1.04	1.64E-01	transcription_start_site	+	391079	391079	159	1597 AN0506
2845	CONTIG7	390686	391791	1.04	1.64E-01	transcription_start_site	+	393832	393832	-2593	1598 AN0507
2845	CONTIG7	390686	391791	1.04	1.64E-01	transcription_start_site	+	394728	394728	-3489	1599 AN0507
2845	CONTIG7	390686	391791	1.04	1.64E-01	transcription_start_site	+	395167	395167	-3928	1600 AN0507
2845	CONTIG7	390686	391791	1.04	1.64E-01	transcription_start_site	+	395817	395817	-4578	1601 AN0507
1941	CONTIG7	434701	435800	1.39	6.84E-02	transcription_start_site	+	435397	435397	-146	1635 AN0517
1941	CONTIG7	434701	435800	1.39	6.84E-02	transcription_start_site	+	434959	434959	291	1634 AN0517
1941	CONTIG7	434701	435800	1.39	6.84E-02	transcription_start_site	+	435564	435564	-313	1636 AN0517
1941	CONTIG7	434701	435800	1.39	6.84E-02	transcription_start_site	+	434810	434810	440	1633 AN0517
1941	CONTIG7	434701	435800	1.39	6.84E-02	transcription_start_site	+	435719	435719	-468	1637 AN0517
1941	CONTIG7	434701	435800	1.39	6.84E-02	transcription_start_site	+	436136	436136	-885	1638 AN0517
1941	CONTIG7	434701	435800	1.39	6.84E-02	transcription_start_site	+	434090	434090	1160	1632 AN0517
2190	CONTIG7	436591	436850	1.3	9.24E-02	transcription_start_site	+	436136	436136	584	1638 AN0517
2190	CONTIG7	436591	436850	1.3	9.24E-02	transcription_start_site	+	435719	435719	1001	1637 AN0517
2586	CONTIG7	427354	427638	1.17	1.44E-01	transcription_start_site	+	430154	430154	-2658	1626 AN0517
2586	CONTIG7	427354	427638	1.17	1.44E-01	transcription_start_site	+	430509	430509	-3013	1627 AN0517
2586	CONTIG7	427354	427638	1.17	1.44E-01	transcription_start_site	+	430646	430646	-3150	1628 AN0517
2586	CONTIG7	427354	427638	1.17	1.44E-01	transcription_start_site	+	431013	431013	-3517	1629 AN0517
2586	CONTIG7	427354	427638	1.17	1.44E-01	transcription_start_site	+	431203	431203	-3707	1630 AN0517
2766	CONTIG7	432164	432513	1.09	1.93E-01	transcription_start_site	+	431203	431203	1135	1630 AN0517
2766	CONTIG7	432164	432513	1.09	1.93E-01	transcription_start_site	+	433629	433629	-1290	1631 AN0517
2766	CONTIG7	432164	432513	1.09	1.93E-01	transcription_start_site	+	434090	434090	-1751	1632 AN0517
2766	CONTIG7	432164	432513	1.09	1.93E-01	transcription_start_site	+	434810	434810	-2471	1633 AN0517
2766	CONTIG7	432164	432513	1.09	1.93E-01	transcription_start_site	+	434959	434959	-2620	1634 AN0517
2766	CONTIG7	432164	432513	1.09	1.93E-01	transcription_start_site	+	435397	435397	-3058	1635 AN0517
2766	CONTIG7	432164	432513	1.09	1.93E-01	transcription_start_site	+	435564	435564	-3225	1636 AN0517
2766	CONTIG7	432164	432513	1.09	1.93E-01	transcription_start_site	+	435719	435719	-3380	1637 AN0517
2766	CONTIG7	432164	432513	1.09	1.93E-01	transcription_start_site	+	436136	436136	-3797	1638 AN0517
1941	CONTIG7	434701	435800	1.39	6.84E-02	transcription_start_site	+	436618	436618	-1367	1639 AN0518
2190	CONTIG7	436591	436850	1.3	9.24E-02	transcription_start_site	+	436618	436618	102	1639 AN0518
2766	CONTIG7	432164	432513	1.09	1.93E-01	transcription_start_site	+	436618	436618	-4279	1639 AN0518
1941	CONTIG7	434701	435800	1.39	6.84E-02	transcription_start_site	+	439478	439478	-4227	1640 AN0519
1941	CONTIG7	434701	435800	1.39	6.84E-02	transcription_start_site	+	439582	439582	-4331	1641 AN0519
1941	CONTIG7	434701	435800	1.39	6.84E-02	transcription_start_site	+	440042	440042	-4791	1642 AN0519
1941	CONTIG7	434701	435800	1.39	6.84E-02	transcription_start_site	+	440302	440302	-5051	1643 AN0519
2190	CONTIG7	436591	436850	1.3	9.24E-02	transcription_start_site	+	439478	439478	-2757	1640 AN0519
2190	CONTIG7	436591	436850	1.3	9.24E-02	transcription_start_site	+	439582	439582	-2861	1641 AN0519
2190	CONTIG7	436591	436850	1.3	9.24E-02	transcription_start_site	+	440042	440042	-3321	1642 AN0519
2190	CONTIG7	436591	436850	1.3	9.24E-02	transcription_start_site	+	440302	440302	-3581	1643 AN0519
947	CONTIG7	508741	509232	1.96	7.85E-03	transcription_start_site	-	507869	507869	-1117	1713 AN0544
947	CONTIG7	508741	509232	1.96	7.85E-03	transcription_start_site	-	507680	507680	-1306	1714 AN0544
947	CONTIG7	508741	509232	1.96	7.85E-03	transcription_start_site	-	507580	507580	-1406	1715 AN0544
947	CONTIG7	508741	509232	1.96	7.85E-03	transcription_start_site	-	507418	507418	-1568	1716 AN0544
947	CONTIG7	508741	509232	1.96	7.85E-03	transcription_start_site	+	510718	510718	-1731	1717 AN0545
947	CONTIG7	508741	509232	1.96	7.85E-03	transcription_start_site	+	510788	510788	-1801	1718 AN0545
947	CONTIG7	508741	509232	1.96	7.85E-03	transcription_start_site	+	511100	511100	-2113	1719 AN0545
947	CONTIG7	508741	509232	1.96	7.85E-03	transcription_start_site	+	511277	511277	-2290	1720 AN0545
766	CONTIG7	547596	547935	2.13	3.61E-03	transcription_start_site	-	547187	547187	-578	1755 AN0554
766	CONTIG7	547596	547935	2.13	3.61E-03	transcription_start_site	-	547060	547060	-705	1756 AN0554
766	CONTIG7	547596	547935	2.13	3.61E-03	transcription_start_site	-	545804	545804	-1961	1757 AN0554
2436	CONTIG7	551046	551600	1.22	1.07E-01	transcription_start_site	-	547187	547187	-4136	1755 AN0554

2436	CONTIG7	551046	551600	1.22	1.07E-01	transcription_start_site	-	547060	547060	-4263	1756	AN0554
2436	CONTIG7	551046	551600	1.22	1.07E-01	transcription_start_site	-	552370	552370	1047	1761	AN0556
2436	CONTIG7	551046	551600	1.22	1.07E-01	transcription_start_site	+	555440	555440	-4117	1762	AN0557
2436	CONTIG7	551046	551600	1.22	1.07E-01	transcription_start_site	+	555805	555805	-4482	1763	AN0557
2331	CONTIG7	566338	566762	1.26	1.08E-01	transcription_start_site	-	562812	562812	-3738	1768	AN0559
2331	CONTIG7	566338	566762	1.26	1.08E-01	transcription_start_site	-	562688	562688	-3862	1769	AN0559
2331	CONTIG7	566338	566762	1.26	1.08E-01	transcription_start_site	-	562383	562383	-4167	1770	AN0559
2331	CONTIG7	566338	566762	1.26	1.08E-01	transcription_start_site	+	565491	565491	1059	1774	AN0560
813	CONTIG7	569263	569602	2.09	4.61E-03	transcription_start_site	-	567984	567984	-1448	1775	AN0561
813	CONTIG7	569263	569602	2.09	4.61E-03	transcription_start_site	-	567200	567200	-2232	1776	AN0561
2331	CONTIG7	566338	566762	1.26	1.08E-01	transcription_start_site	-	567200	567200	650	1776	AN0561
813	CONTIG7	569263	569602	2.09	4.61E-03	transcription_start_site	+	569024	569024	408	1778	AN0562
813	CONTIG7	569263	569602	2.09	4.61E-03	transcription_start_site	+	568668	568668	764	1777	AN0562
813	CONTIG7	569263	569602	2.09	4.61E-03	transcription_start_site	+	570255	570255	-822	1779	AN0562
2331	CONTIG7	566338	566762	1.26	1.08E-01	transcription_start_site	+	568668	568668	-2118	1777	AN0562
2331	CONTIG7	566338	566762	1.26	1.08E-01	transcription_start_site	+	569024	569024	-2474	1778	AN0562
2331	CONTIG7	566338	566762	1.26	1.08E-01	transcription_start_site	+	570255	570255	-3705	1779	AN0562
861	CONTIG7	582546	583115	2.04	2.32E-03	transcription_start_site	-	582210	582210	-620	1789	AN0565
861	CONTIG7	582546	583115	2.04	2.32E-03	transcription_start_site	-	581439	581439	-1391	1790	AN0565
861	CONTIG7	582546	583115	2.04	2.32E-03	transcription_start_site	+	583866	583866	-1035	1791	AN0566
861	CONTIG7	582546	583115	2.04	2.32E-03	transcription_start_site	+	584172	584172	-1341	1792	AN0566
2767	CONTIG7	596643	596992	1.09	1.93E-01	transcription_start_site	-	593702	593702	-3115	1800	AN0568
2767	CONTIG7	596643	596992	1.09	1.93E-01	transcription_start_site	-	597778	597778	960	1801	AN0569
2767	CONTIG7	596643	596992	1.09	1.93E-01	transcription_start_site	-	595358	595358	-1459	1802	AN0569
2767	CONTIG7	596643	596992	1.09	1.93E-01	transcription_start_site	+	598313	598313	-1495	1803	AN0570
2767	CONTIG7	596643	596992	1.09	1.93E-01	transcription_start_site	+	598492	598492	-1674	1804	AN0570
2767	CONTIG7	596643	596992	1.09	1.93E-01	transcription_start_site	+	598757	598757	-1939	1805	AN0570
2439	CONTIG7	606683	607102	1.22	1.25E-01	transcription_start_site	-	603171	603171	-3721	1807	AN0572
2439	CONTIG7	606683	607102	1.22	1.25E-01	transcription_start_site	-	607162	607162	269	1808	AN0573
2439	CONTIG7	606683	607102	1.22	1.25E-01	transcription_start_site	+	608031	608031	-1138	1809	AN0574
2439	CONTIG7	606683	607102	1.22	1.25E-01	transcription_start_site	+	608398	608398	-1505	1810	AN0574
2439	CONTIG7	606683	607102	1.22	1.25E-01	transcription_start_site	+	608585	608585	-1692	1811	AN0574
897	CONTIG7	613213	613712	2	2.49E-03	transcription_start_site	-	612750	612750	-712	1812	AN0575
897	CONTIG7	613213	613712	2	2.49E-03	transcription_start_site	-	612319	612319	-1143	1813	AN0575
897	CONTIG7	613213	613712	2	2.49E-03	transcription_start_site	-	611252	611252	-2210	1814	AN0575
2332	CONTIG7	612613	613187	1.26	1.08E-01	transcription_start_site	-	612750	612750	-150	1812	AN0575
2332	CONTIG7	612613	613187	1.26	1.08E-01	transcription_start_site	-	612319	612319	-581	1813	AN0575
2332	CONTIG7	612613	613187	1.26	1.08E-01	transcription_start_site	-	611252	611252	-1648	1814	AN0575
1936	CONTIG7	647345	648144	1.39	5.05E-02	transcription_start_site	-	645103	645103	-2641	1847	AN0584
1936	CONTIG7	647345	648144	1.39	5.05E-02	transcription_start_site	-	644508	644508	-3236	1848	AN0584
1936	CONTIG7	647345	648144	1.39	5.05E-02	transcription_start_site	+	647433	647433	311	1850	AN0585
1936	CONTIG7	647345	648144	1.39	5.05E-02	transcription_start_site	+	646744	646744	1000	1849	AN0585
1233	CONTIG7	690918	691252	1.74	1.84E-02	transcription_start_site	-	688881	688881	-2204	1880	AN0594
1233	CONTIG7	690918	691252	1.74	1.84E-02	transcription_start_site	-	688116	688116	-2969	1881	AN0594
1233	CONTIG7	690918	691252	1.74	1.84E-02	transcription_start_site	-	687829	687829	-3256	1882	AN0594
1942	CONTIG7	688578	688852	1.39	6.84E-02	transcription_start_site	-	688881	688881	166	1880	AN0594
1942	CONTIG7	688578	688852	1.39	6.84E-02	transcription_start_site	-	688116	688116	-599	1881	AN0594
1942	CONTIG7	688578	688852	1.39	6.84E-02	transcription_start_site	-	687829	687829	-886	1882	AN0594
1942	CONTIG7	688578	688852	1.39	6.84E-02	transcription_start_site	-	684481	684481	-4234	1883	AN0594
1233	CONTIG7	690918	691252	1.74	1.84E-02	transcription_start_site	+	691645	691645	-560	1884	AN0595
1233	CONTIG7	690918	691252	1.74	1.84E-02	transcription_start_site	+	693499	693499	-2414	1885	AN0595
1942	CONTIG7	688578	688852	1.39	6.84E-02	transcription_start_site	+	691645	691645	-2930	1884	AN0595
1942	CONTIG7	688578	688852	1.39	6.84E-02	transcription_start_site	+	693499	693499	-4784	1885	AN0595
1233	CONTIG7	690918	691252	1.74	1.84E-02	transcription_start_site	+	694485	694485	-3400	1886	AN0596
1233	CONTIG7	690918	691252	1.74	1.84E-02	transcription_start_site	+	694659	694659	-3574	1887	AN0596
1233	CONTIG7	690918	691252	1.74	1.84E-02	transcription_start_site	+	695362	695362	-4277	1888	AN0596
236	CONTIG7	703369	703786	2.87	0.00E+00	transcription_start_site	-	699227	699227	-4350	1891	AN0597

236	CONTIG7	703369	703786	2.87	0.00E+00	transcription_start_site	+	702507	702507	1070	1898 AN0599
236	CONTIG7	703369	703786	2.87	0.00E+00	transcription_start_site	+	703846	703846	-268	1899 AN0600
236	CONTIG7	703369	703786	2.87	0.00E+00	transcription_start_site	+	704343	704343	-765	1900 AN0600
236	CONTIG7	703369	703786	2.87	0.00E+00	transcription_start_site	+	705730	705730	-2152	1901 AN0601
236	CONTIG7	703369	703786	2.87	0.00E+00	transcription_start_site	+	705924	705924	-2346	1902 AN0601
673	CONTIG7	711762	712041	2.22	2.22E-03	transcription_start_site	-	710655	710655	-1246	1903 AN0602
2049	CONTIG7	709892	710466	1.35	6.16E-02	transcription_start_site	-	710655	710655	476	1903 AN0602
673	CONTIG7	711762	712041	2.22	2.22E-03	transcription_start_site	+	712138	712138	-236	1904 AN0604
673	CONTIG7	711762	712041	2.22	2.22E-03	transcription_start_site	+	712433	712433	-531	1905 AN0604
2049	CONTIG7	709892	710466	1.35	6.16E-02	transcription_start_site	+	712138	712138	-1959	1904 AN0604
2049	CONTIG7	709892	710466	1.35	6.16E-02	transcription_start_site	+	712433	712433	-2254	1905 AN0604
673	CONTIG7	711762	712041	2.22	2.22E-03	transcription_start_site	+	715029	715029	-3127	1906 AN0605
673	CONTIG7	711762	712041	2.22	2.22E-03	transcription_start_site	+	715102	715102	-3200	1907 AN0605
673	CONTIG7	711762	712041	2.22	2.22E-03	transcription_start_site	+	715429	715429	-3527	1908 AN0605
2049	CONTIG7	709892	710466	1.35	6.16E-02	transcription_start_site	+	715029	715029	-4850	1906 AN0605
2049	CONTIG7	709892	710466	1.35	6.16E-02	transcription_start_site	+	715102	715102	-4923	1907 AN0605
2049	CONTIG7	709892	710466	1.35	6.16E-02	transcription_start_site	+	715429	715429	-5250	1908 AN0605
111	CONTIG7	736744	737168	3.17	0.00E+00	transcription_start_site	-	736405	736405	-551	1914 AN0607
111	CONTIG7	736744	737168	3.17	0.00E+00	transcription_start_site	-	735772	735772	-1184	1915 AN0607
111	CONTIG7	736744	737168	3.17	0.00E+00	transcription_start_site	-	733047	733047	-3909	1916 AN0607
1173	CONTIG7	738304	738663	1.78	1.57E-02	transcription_start_site	-	736405	736405	-2078	1914 AN0607
1173	CONTIG7	738304	738663	1.78	1.57E-02	transcription_start_site	-	735772	735772	-2711	1915 AN0607
1227	CONTIG7	739144	740393	1.74	3.45E-03	transcription_start_site	-	736405	736405	-3363	1914 AN0607
1227	CONTIG7	739144	740393	1.74	3.45E-03	transcription_start_site	-	735772	735772	-3996	1915 AN0607
2324	CONTIG7	740854	741743	1.26	9.26E-02	transcription_start_site	-	736405	736405	-4893	1914 AN0607
111	CONTIG7	736744	737168	3.17	0.00E+00	transcription_start_site	+	737167	737167	-211	1917 AN0608
111	CONTIG7	736744	737168	3.17	0.00E+00	transcription_start_site	+	738185	738185	-1229	1918 AN0608
1173	CONTIG7	738304	738663	1.78	1.57E-02	transcription_start_site	+	738185	738185	298	1918 AN0608
1227	CONTIG7	739144	740393	1.74	3.45E-03	transcription_start_site	+	738185	738185	1583	1918 AN0608
1173	CONTIG7	738304	738663	1.78	1.57E-02	transcription_start_site	-	739549	739549	1065	1923 AN0609
1227	CONTIG7	739144	740393	1.74	3.45E-03	transcription_start_site	-	739963	739963	194	1922 AN0609
1227	CONTIG7	739144	740393	1.74	3.45E-03	transcription_start_site	-	739549	739549	-219	1923 AN0609
1227	CONTIG7	739144	740393	1.74	3.45E-03	transcription_start_site	-	740397	740397	628	1921 AN0609
1227	CONTIG7	739144	740393	1.74	3.45E-03	transcription_start_site	-	740726	740726	957	1920 AN0609
1227	CONTIG7	739144	740393	1.74	3.45E-03	transcription_start_site	-	741236	741236	1467	1919 AN0609
2324	CONTIG7	740854	741743	1.26	9.26E-02	transcription_start_site	-	741236	741236	-62	1919 AN0609
2324	CONTIG7	740854	741743	1.26	9.26E-02	transcription_start_site	-	740726	740726	-572	1920 AN0609
2324	CONTIG7	740854	741743	1.26	9.26E-02	transcription_start_site	-	740397	740397	-901	1921 AN0609
2324	CONTIG7	740854	741743	1.26	9.26E-02	transcription_start_site	-	739963	739963	-1335	1922 AN0609
2324	CONTIG7	740854	741743	1.26	9.26E-02	transcription_start_site	-	739549	739549	-1749	1923 AN0609
1227	CONTIG7	739144	740393	1.74	3.45E-03	transcription_start_site	+	745209	745209	-5440	1924 AN0610
1227	CONTIG7	739144	740393	1.74	3.45E-03	transcription_start_site	+	745344	745344	-5575	1925 AN0610
1827	CONTIG7	746569	746988	1.44	5.88E-02	transcription_start_site	+	746320	746320	458	1929 AN0610
1827	CONTIG7	746569	746988	1.44	5.88E-02	transcription_start_site	+	745715	745715	1063	1928 AN0610
2324	CONTIG7	740854	741743	1.26	9.26E-02	transcription_start_site	+	745209	745209	-3910	1924 AN0610
2324	CONTIG7	740854	741743	1.26	9.26E-02	transcription_start_site	+	745344	745344	-4045	1925 AN0610
2324	CONTIG7	740854	741743	1.26	9.26E-02	transcription_start_site	+	745438	745438	-4139	1926 AN0610
2324	CONTIG7	740854	741743	1.26	9.26E-02	transcription_start_site	+	745554	745554	-4255	1927 AN0610
2324	CONTIG7	740854	741743	1.26	9.26E-02	transcription_start_site	+	745715	745715	-4416	1928 AN0610
2324	CONTIG7	740854	741743	1.26	9.26E-02	transcription_start_site	+	746320	746320	-5021	1929 AN0610
2768	CONTIG7	767419	768291	1.09	1.93E-01	transcription_start_site	-	762838	762838	-5017	1946 AN0618
2768	CONTIG7	767419	768291	1.09	1.93E-01	transcription_start_site	-	762460	762460	-5395	1947 AN0618
2768	CONTIG7	767419	768291	1.09	1.93E-01	transcription_start_site	-	767467	767467	-388	1953 AN0620
2768	CONTIG7	767419	768291	1.09	1.93E-01	transcription_start_site	-	767155	767155	-700	1954 AN0620
2768	CONTIG7	767419	768291	1.09	1.93E-01	transcription_start_site	-	766685	766685	-1170	1955 AN0620
2768	CONTIG7	767419	768291	1.09	1.93E-01	transcription_start_site	-	769075	769075	1220	1957 AN0621
1570	CONTIG8	12245	12654	1.56	1.93E-01	transcription_start_site	-	7375	7375	-5074	1958 AN0623

11	CONTIG8	16060	16559	3.69	6.10E-04	transcription_start_site	-	12132	12132	-4177	1964	AN0625
11	CONTIG8	16060	16559	3.69	6.10E-04	transcription_start_site	-	11836	11836	-4473	1965	AN0625
268	CONTIG8	16745	17379	2.81	7.85E-03	transcription_start_site	-	12132	12132	-4930	1964	AN0625
268	CONTIG8	16745	17379	2.81	7.85E-03	transcription_start_site	-	11836	11836	-5226	1965	AN0625
1135	CONTIG8	13885	14234	1.81	1.08E-01	transcription_start_site	-	12132	12132	-1927	1964	AN0625
1135	CONTIG8	13885	14234	1.81	1.08E-01	transcription_start_site	-	11836	11836	-2223	1965	AN0625
1570	CONTIG8	12245	12654	1.56	1.93E-01	transcription_start_site	-	12132	12132	-317	1964	AN0625
1570	CONTIG8	12245	12654	1.56	1.93E-01	transcription_start_site	-	11836	11836	-613	1965	AN0625
1570	CONTIG8	12245	12654	1.56	1.93E-01	transcription_start_site	+	12379	12379	70	1966	AN0626
11	CONTIG8	16060	16559	3.69	6.10E-04	transcription_start_site	-	14631	14631	-1678	1967	AN0627
11	CONTIG8	16060	16559	3.69	6.10E-04	transcription_start_site	-	14016	14016	-2293	1968	AN0627
268	CONTIG8	16745	17379	2.81	7.85E-03	transcription_start_site	-	14631	14631	-2431	1967	AN0627
268	CONTIG8	16745	17379	2.81	7.85E-03	transcription_start_site	-	14016	14016	-3046	1968	AN0627
1135	CONTIG8	13885	14234	1.81	1.08E-01	transcription_start_site	-	14016	14016	-43	1968	AN0627
1135	CONTIG8	13885	14234	1.81	1.08E-01	transcription_start_site	-	14631	14631	571	1967	AN0627
11	CONTIG8	16060	16559	3.69	6.10E-04	transcription_start_site	+	19076	19076	-2766	1969	AN0628
268	CONTIG8	16745	17379	2.81	7.85E-03	transcription_start_site	+	19076	19076	-2014	1969	AN0628
1135	CONTIG8	13885	14234	1.81	1.08E-01	transcription_start_site	+	19076	19076	-5016	1969	AN0628
785	CONTIG8	22578	22852	2.12	5.22E-02	transcription_start_site	-	21159	21159	-1556	1970	AN0629
785	CONTIG8	22578	22852	2.12	5.22E-02	transcription_start_site	-	21069	21069	-1646	1971	AN0629
785	CONTIG8	22578	22852	2.12	5.22E-02	transcription_start_site	-	20634	20634	-2081	1972	AN0629
268	CONTIG8	16745	17379	2.81	7.85E-03	transcription_start_site	+	21824	21824	-4762	1973	AN0630
268	CONTIG8	16745	17379	2.81	7.85E-03	transcription_start_site	+	21896	21896	-4834	1974	AN0630
268	CONTIG8	16745	17379	2.81	7.85E-03	transcription_start_site	+	22052	22052	-4990	1975	AN0630
268	CONTIG8	16745	17379	2.81	7.85E-03	transcription_start_site	+	22255	22255	-5193	1976	AN0630
785	CONTIG8	22578	22852	2.12	5.22E-02	transcription_start_site	+	22255	22255	460	1976	AN0630
785	CONTIG8	22578	22852	2.12	5.22E-02	transcription_start_site	+	22052	22052	663	1975	AN0630
785	CONTIG8	22578	22852	2.12	5.22E-02	transcription_start_site	+	21896	21896	819	1974	AN0630
785	CONTIG8	22578	22852	2.12	5.22E-02	transcription_start_site	+	21824	21824	891	1973	AN0630
642	CONTIG8	27008	27352	2.25	3.70E-02	transcription_start_site	-	23394	23394	-3786	1977	AN0631
642	CONTIG8	27008	27352	2.25	3.70E-02	transcription_start_site	-	22528	22528	-4652	1978	AN0631
785	CONTIG8	22578	22852	2.12	5.22E-02	transcription_start_site	-	22528	22528	-187	1978	AN0631
785	CONTIG8	22578	22852	2.12	5.22E-02	transcription_start_site	-	23394	23394	679	1977	AN0631
785	CONTIG8	22578	22852	2.12	5.22E-02	transcription_start_site	+	23720	23720	-1005	1979	AN0632
785	CONTIG8	22578	22852	2.12	5.22E-02	transcription_start_site	+	24696	24696	-1981	1980	AN0632
642	CONTIG8	27008	27352	2.25	3.70E-02	transcription_start_site	-	26763	26763	-417	1981	AN0633
642	CONTIG8	27008	27352	2.25	3.70E-02	transcription_start_site	-	26640	26640	-540	1982	AN0633
642	CONTIG8	27008	27352	2.25	3.70E-02	transcription_start_site	+	27680	27680	-500	1983	AN0634
642	CONTIG8	27008	27352	2.25	3.70E-02	transcription_start_site	+	27786	27786	-606	1984	AN0634
785	CONTIG8	22578	22852	2.12	5.22E-02	transcription_start_site	+	27680	27680	-4965	1983	AN0634
785	CONTIG8	22578	22852	2.12	5.22E-02	transcription_start_site	+	27786	27786	-5071	1984	AN0634
642	CONTIG8	27008	27352	2.25	3.70E-02	transcription_start_site	+	29125	29125	-1945	1985	AN0635
642	CONTIG8	27008	27352	2.25	3.70E-02	transcription_start_site	+	29307	29307	-2127	1986	AN0635
642	CONTIG8	27008	27352	2.25	3.70E-02	transcription_start_site	+	31866	31866	-4686	1992	AN0637
1224	CONTIG8	40433	40781	1.75	1.25E-01	transcription_start_site	-	36316	36316	-4291	1993	AN0638
1224	CONTIG8	40433	40781	1.75	1.25E-01	transcription_start_site	-	35810	35810	-4797	1994	AN0638
1224	CONTIG8	40433	40781	1.75	1.25E-01	transcription_start_site	-	35672	35672	-4935	1995	AN0638
1224	CONTIG8	40433	40781	1.75	1.25E-01	transcription_start_site	-	40240	40240	-367	1998	AN0640
1224	CONTIG8	40433	40781	1.75	1.25E-01	transcription_start_site	-	39497	39497	-1110	1999	AN0640
1224	CONTIG8	40433	40781	1.75	1.25E-01	transcription_start_site	+	41291	41291	-684	2000	AN0641
1224	CONTIG8	40433	40781	1.75	1.25E-01	transcription_start_site	+	41452	41452	-845	2001	AN0641
1224	CONTIG8	40433	40781	1.75	1.25E-01	transcription_start_site	+	41800	41800	-1193	2002	AN0641
1224	CONTIG8	40433	40781	1.75	1.25E-01	transcription_start_site	+	42015	42015	-1408	2003	AN0641
2026	CONTIG9	4351	4927	1.36	2.24E-03	transcription_start_site	-	1091	1091	-3548	2004	AN0643
2026	CONTIG9	4351	4927	1.36	2.24E-03	transcription_start_site	-	586	586	-4053	2005	AN0643
2026	CONTIG9	4351	4927	1.36	2.24E-03	transcription_start_site	-	4147	4147	-492	2006	AN0644
2026	CONTIG9	4351	4927	1.36	2.24E-03	transcription_start_site	-	3815	3815	-824	2007	AN0644

2026	CONTIG9	4351	4927	1.36	2.24E-03	transcription_start_site	-	5102	5102	463	2010 AN0645
2026	CONTIG9	4351	4927	1.36	2.24E-03	transcription_start_site	-	5397	5397	758	2009 AN0645
2026	CONTIG9	4351	4927	1.36	2.24E-03	transcription_start_site	-	5672	5672	1033	2008 AN0645
1116	CONTIG10	6976	7252	1.82	2.12E-02	transcription_start_site	+	6556	6556	558	2015 AN0649
1116	CONTIG10	6976	7252	1.82	2.12E-02	transcription_start_site	+	7690	7690	-576	2016 AN0649
1116	CONTIG10	6976	7252	1.82	2.12E-02	transcription_start_site	+	8050	8050	-936	2017 AN0649
1116	CONTIG10	6976	7252	1.82	2.12E-02	transcription_start_site	+	8206	8206	-1092	2018 AN0649
1116	CONTIG10	6976	7252	1.82	2.12E-02	transcription_start_site	+	11257	11257	-4143	2019 AN0650
575	CONTIG10	16504	16870	2.34	0.00E+00	transcription_start_site	-	15818	15818	-869	2025 AN0651
575	CONTIG10	16504	16870	2.34	0.00E+00	transcription_start_site	-	15648	15648	-1039	2026 AN0651
575	CONTIG10	16504	16870	2.34	0.00E+00	transcription_start_site	-	15252	15252	-1435	2027 AN0651
575	CONTIG10	16504	16870	2.34	0.00E+00	transcription_start_site	-	14643	14643	-2044	2028 AN0651
575	CONTIG10	16504	16870	2.34	0.00E+00	transcription_start_site	-	17599	17599	912	2031 AN0652
575	CONTIG10	16504	16870	2.34	0.00E+00	transcription_start_site	+	18662	18662	-1975	2032 AN0653
575	CONTIG10	16504	16870	2.34	0.00E+00	transcription_start_site	+	18761	18761	-2074	2033 AN0653
575	CONTIG10	16504	16870	2.34	0.00E+00	transcription_start_site	+	18946	18946	-2259	2034 AN0653
575	CONTIG10	16504	16870	2.34	0.00E+00	transcription_start_site	+	19279	19279	-2592	2035 AN0653
985	CONTIG10	34730	35079	1.92	7.16E-03	transcription_start_site	+	35483	35483	-578	2051 AN0660
985	CONTIG10	34730	35079	1.92	7.16E-03	transcription_start_site	+	35640	35640	-735	2052 AN0660
985	CONTIG10	34730	35079	1.92	7.16E-03	transcription_start_site	+	36015	36015	-1110	2053 AN0660
985	CONTIG10	34730	35079	1.92	7.16E-03	transcription_start_site	+	36080	36080	-1175	2054 AN0660
985	CONTIG10	34730	35079	1.92	7.16E-03	transcription_start_site	+	36860	36860	-1955	2055 AN0660
985	CONTIG10	34730	35079	1.92	7.16E-03	transcription_start_site	+	38544	38544	-3639	2056 AN0661
1922	CONTIG10	46295	46776	1.4	7.51E-02	transcription_start_site	-	42654	42654	-3881	2057 AN0662
1922	CONTIG10	46295	46776	1.4	7.51E-02	transcription_start_site	-	42537	42537	-3998	2058 AN0662
1922	CONTIG10	46295	46776	1.4	7.51E-02	transcription_start_site	-	42274	42274	-4261	2059 AN0662
1922	CONTIG10	46295	46776	1.4	7.51E-02	transcription_start_site	-	41551	41551	-4984	2060 AN0662
1922	CONTIG10	46295	46776	1.4	7.51E-02	transcription_start_site	+	46909	46909	-373	2061 AN0663
1922	CONTIG10	46295	46776	1.4	7.51E-02	transcription_start_site	+	47302	47302	-766	2062 AN0663
1922	CONTIG10	46295	46776	1.4	7.51E-02	transcription_start_site	+	47598	47598	-1062	2063 AN0663
1493	CONTIG10	51082	51362	1.59	5.22E-02	transcription_start_site	+	52714	52714	-1492	2064 AN0664
950	CONTIG10	62102	62386	1.96	1.30E-02	transcription_start_site	-	59773	59773	-2471	2069 AN0666
950	CONTIG10	62102	62386	1.96	1.30E-02	transcription_start_site	-	59471	59471	-2773	2070 AN0666
950	CONTIG10	62102	62386	1.96	1.30E-02	transcription_start_site	-	59283	59283	-2961	2071 AN0666
950	CONTIG10	62102	62386	1.96	1.30E-02	transcription_start_site	-	58595	58595	-3649	2072 AN0666
950	CONTIG10	62102	62386	1.96	1.30E-02	transcription_start_site	-	61763	61763	-481	2073 AN0667
950	CONTIG10	62102	62386	1.96	1.30E-02	transcription_start_site	-	61674	61674	-570	2074 AN0667
950	CONTIG10	62102	62386	1.96	1.30E-02	transcription_start_site	-	61594	61594	-650	2075 AN0667
950	CONTIG10	62102	62386	1.96	1.30E-02	transcription_start_site	-	61444	61444	-800	2076 AN0667
950	CONTIG10	62102	62386	1.96	1.30E-02	transcription_start_site	-	61155	61155	-1089	2077 AN0667
950	CONTIG10	62102	62386	1.96	1.30E-02	transcription_start_site	+	62098	62098	146	2078 AN0668
950	CONTIG10	62102	62386	1.96	1.30E-02	transcription_start_site	+	62767	62767	-523	2079 AN0668
950	CONTIG10	62102	62386	1.96	1.30E-02	transcription_start_site	+	63358	63358	-1114	2080 AN0668
2172	CONTIG10	68043	68302	1.31	1.25E-01	transcription_start_site	-	65616	65616	-2556	2081 AN0669
2172	CONTIG10	68043	68302	1.31	1.25E-01	transcription_start_site	-	64632	64632	-3540	2082 AN0669
950	CONTIG10	62102	62386	1.96	1.30E-02	transcription_start_site	+	65976	65976	-3732	2083 AN0670
950	CONTIG10	62102	62386	1.96	1.30E-02	transcription_start_site	+	67256	67256	-5012	2084 AN0670
2172	CONTIG10	68043	68302	1.31	1.25E-01	transcription_start_site	+	67256	67256	916	2084 AN0670
2172	CONTIG10	68043	68302	1.31	1.25E-01	transcription_start_site	+	68110	68110	62	2085 AN0671
2172	CONTIG10	68043	68302	1.31	1.25E-01	transcription_start_site	+	68742	68742	-569	2086 AN0671
2172	CONTIG10	68043	68302	1.31	1.25E-01	transcription_start_site	+	71884	71884	-3711	2092 AN0673
2172	CONTIG10	68043	68302	1.31	1.25E-01	transcription_start_site	+	71971	71971	-3798	2093 AN0673
2172	CONTIG10	68043	68302	1.31	1.25E-01	transcription_start_site	+	72090	72090	-3917	2094 AN0673
2172	CONTIG10	68043	68302	1.31	1.25E-01	transcription_start_site	+	72353	72353	-4180	2095 AN0673
2172	CONTIG10	68043	68302	1.31	1.25E-01	transcription_start_site	+	72632	72632	-4459	2096 AN0673
2172	CONTIG10	68043	68302	1.31	1.25E-01	transcription_start_site	+	73259	73259	-5086	2097 AN0673
990	CONTIG10	82820	83604	1.92	1.57E-02	transcription_start_site	+	83665	83665	-453	2112 AN0677

1117	CONTIG10	94737	95316	1.82	2.12E-02	transcription_start_site	+	93915	93915	1111	2122	AN0682
578	CONTIG10	99163	99732	2.34	3.03E-03	transcription_start_site	+	98451	98451	996	2124	AN0683
614	CONTIG10	96542	97104	2.29	4.46E-04	transcription_start_site	+	97424	97424	-601	2123	AN0683
614	CONTIG10	96542	97104	2.29	4.46E-04	transcription_start_site	+	98451	98451	-1628	2124	AN0683
1117	CONTIG10	94737	95316	1.82	2.12E-02	transcription_start_site	+	97424	97424	-2397	2123	AN0683
1117	CONTIG10	94737	95316	1.82	2.12E-02	transcription_start_site	+	98451	98451	-3424	2124	AN0683
578	CONTIG10	99163	99732	2.34	3.03E-03	transcription_start_site	+	100437	100437	-989	2125	AN0684
614	CONTIG10	96542	97104	2.29	4.46E-04	transcription_start_site	+	100437	100437	-3614	2125	AN0684
578	CONTIG10	99163	99732	2.34	3.03E-03	transcription_start_site	+	104151	104151	-4703	2126	AN0685
220	CONTIG10	117977	118930	2.9	0.00E+00	transcription_start_site	-	113039	113039	-5414	2134	AN0688
2495	CONTIG10	114017	114277	1.21	1.66E-01	transcription_start_site	-	113039	113039	-1108	2134	AN0688
2495	CONTIG10	114017	114277	1.21	1.66E-01	transcription_start_site	-	112722	112722	-1425	2135	AN0688
2495	CONTIG10	114017	114277	1.21	1.66E-01	transcription_start_site	-	112525	112525	-1622	2136	AN0688
2495	CONTIG10	114017	114277	1.21	1.66E-01	transcription_start_site	-	112371	112371	-1776	2137	AN0688
2495	CONTIG10	114017	114277	1.21	1.66E-01	transcription_start_site	-	112020	112020	-2127	2138	AN0688
86	CONTIG10	119189	119688	3.27	0.00E+00	transcription_start_site	-	117577	117577	-1861	2139	AN0689
86	CONTIG10	119189	119688	3.27	0.00E+00	transcription_start_site	-	117034	117034	-2404	2140	AN0689
86	CONTIG10	119189	119688	3.27	0.00E+00	transcription_start_site	-	116666	116666	-2772	2141	AN0689
86	CONTIG10	119189	119688	3.27	0.00E+00	transcription_start_site	-	116333	116333	-3105	2142	AN0689
86	CONTIG10	119189	119688	3.27	0.00E+00	transcription_start_site	-	115925	115925	-3513	2143	AN0689
220	CONTIG10	117977	118930	2.9	0.00E+00	transcription_start_site	-	117577	117577	-876	2139	AN0689
220	CONTIG10	117977	118930	2.9	0.00E+00	transcription_start_site	-	117034	117034	-1419	2140	AN0689
220	CONTIG10	117977	118930	2.9	0.00E+00	transcription_start_site	-	116666	116666	-1787	2141	AN0689
220	CONTIG10	117977	118930	2.9	0.00E+00	transcription_start_site	-	116333	116333	-2120	2142	AN0689
220	CONTIG10	117977	118930	2.9	0.00E+00	transcription_start_site	-	115925	115925	-2528	2143	AN0689
1699	CONTIG10	121809	122078	1.5	6.84E-02	transcription_start_site	-	117577	117577	-4366	2139	AN0689
1699	CONTIG10	121809	122078	1.5	6.84E-02	transcription_start_site	-	117034	117034	-4909	2140	AN0689
86	CONTIG10	119189	119688	3.27	0.00E+00	transcription_start_site	-	120027	120027	588	2147	AN0690
86	CONTIG10	119189	119688	3.27	0.00E+00	transcription_start_site	-	120677	120677	1238	2146	AN0690
803	CONTIG10	126389	126745	2.1	7.85E-03	transcription_start_site	-	121599	121599	-4968	2144	AN0690
1174	CONTIG10	124959	125326	1.78	2.62E-02	transcription_start_site	-	121599	121599	-3543	2144	AN0690
1174	CONTIG10	124959	125326	1.78	2.62E-02	transcription_start_site	-	121214	121214	-3928	2145	AN0690
1174	CONTIG10	124959	125326	1.78	2.62E-02	transcription_start_site	-	120677	120677	-4465	2146	AN0690
1174	CONTIG10	124959	125326	1.78	2.62E-02	transcription_start_site	-	120027	120027	-5115	2147	AN0690
1699	CONTIG10	121809	122078	1.5	6.84E-02	transcription_start_site	-	121599	121599	-344	2144	AN0690
1699	CONTIG10	121809	122078	1.5	6.84E-02	transcription_start_site	-	121214	121214	-729	2145	AN0690
1699	CONTIG10	121809	122078	1.5	6.84E-02	transcription_start_site	-	120677	120677	-1266	2146	AN0690
1699	CONTIG10	121809	122078	1.5	6.84E-02	transcription_start_site	-	120027	120027	-1916	2147	AN0690
86	CONTIG10	119189	119688	3.27	0.00E+00	transcription_start_site	+	121991	121991	-2552	2148	AN0691
86	CONTIG10	119189	119688	3.27	0.00E+00	transcription_start_site	+	122450	122450	-3011	2149	AN0691
220	CONTIG10	117977	118930	2.9	0.00E+00	transcription_start_site	+	121991	121991	-3537	2148	AN0691
220	CONTIG10	117977	118930	2.9	0.00E+00	transcription_start_site	+	122450	122450	-3996	2149	AN0691
1699	CONTIG10	121809	122078	1.5	6.84E-02	transcription_start_site	+	121991	121991	-47	2148	AN0691
1699	CONTIG10	121809	122078	1.5	6.84E-02	transcription_start_site	+	122450	122450	-506	2149	AN0691
803	CONTIG10	126389	126745	2.1	7.85E-03	transcription_start_site	-	124498	124498	-2069	2150	AN0692
803	CONTIG10	126389	126745	2.1	7.85E-03	transcription_start_site	-	124407	124407	-2160	2151	AN0692
803	CONTIG10	126389	126745	2.1	7.85E-03	transcription_start_site	-	124158	124158	-2409	2152	AN0692
803	CONTIG10	126389	126745	2.1	7.85E-03	transcription_start_site	-	123833	123833	-2734	2153	AN0692
803	CONTIG10	126389	126745	2.1	7.85E-03	transcription_start_site	-	123686	123686	-2881	2154	AN0692
803	CONTIG10	126389	126745	2.1	7.85E-03	transcription_start_site	-	123599	123599	-2968	2155	AN0692
803	CONTIG10	126389	126745	2.1	7.85E-03	transcription_start_site	-	123338	123338	-3229	2156	AN0692
803	CONTIG10	126389	126745	2.1	7.85E-03	transcription_start_site	-	123084	123084	-3483	2157	AN0692
803	CONTIG10	126389	126745	2.1	7.85E-03	transcription_start_site	-	122852	122852	-3715	2158	AN0692
803	CONTIG10	126389	126745	2.1	7.85E-03	transcription_start_site	-	122676	122676	-3891	2159	AN0692
1174	CONTIG10	124959	125326	1.78	2.62E-02	transcription_start_site	-	124498	124498	-644	2150	AN0692
1174	CONTIG10	124959	125326	1.78	2.62E-02	transcription_start_site	-	124407	124407	-735	2151	AN0692
1174	CONTIG10	124959	125326	1.78	2.62E-02	transcription_start_site	-	124158	124158	-984	2152	AN0692

1174	CONTIG10	124959	125326	1.78	2.62E-02	transcription_start_site	-	123833	123833	-1309	2153	AN0692
1174	CONTIG10	124959	125326	1.78	2.62E-02	transcription_start_site	-	123686	123686	-1456	2154	AN0692
1174	CONTIG10	124959	125326	1.78	2.62E-02	transcription_start_site	-	123599	123599	-1543	2155	AN0692
1174	CONTIG10	124959	125326	1.78	2.62E-02	transcription_start_site	-	123338	123338	-1804	2156	AN0692
1174	CONTIG10	124959	125326	1.78	2.62E-02	transcription_start_site	-	123084	123084	-2058	2157	AN0692
1174	CONTIG10	124959	125326	1.78	2.62E-02	transcription_start_site	-	122852	122852	-2290	2158	AN0692
1174	CONTIG10	124959	125326	1.78	2.62E-02	transcription_start_site	-	122676	122676	-2466	2159	AN0692
1699	CONTIG10	121809	122078	1.5	6.84E-02	transcription_start_site	-	122676	122676	732	2159	AN0692
1699	CONTIG10	121809	122078	1.5	6.84E-02	transcription_start_site	-	122852	122852	908	2158	AN0692
803	CONTIG10	126389	126745	2.1	7.85E-03	transcription_start_site	+	125554	125554	1013	2161	AN0693
803	CONTIG10	126389	126745	2.1	7.85E-03	transcription_start_site	+	125390	125390	1177	2160	AN0693
1174	CONTIG10	124959	125326	1.78	2.62E-02	transcription_start_site	+	125390	125390	-247	2160	AN0693
1174	CONTIG10	124959	125326	1.78	2.62E-02	transcription_start_site	+	125554	125554	-411	2161	AN0693
1699	CONTIG10	121809	122078	1.5	6.84E-02	transcription_start_site	+	125390	125390	-3446	2160	AN0693
1699	CONTIG10	121809	122078	1.5	6.84E-02	transcription_start_site	+	125554	125554	-3610	2161	AN0693
803	CONTIG10	126389	126745	2.1	7.85E-03	transcription_start_site	+	126723	126723	-156	2162	AN0694
1174	CONTIG10	124959	125326	1.78	2.62E-02	transcription_start_site	+	126723	126723	-1580	2162	AN0694
1699	CONTIG10	121809	122078	1.5	6.84E-02	transcription_start_site	+	126723	126723	-4779	2162	AN0694
803	CONTIG10	126389	126745	2.1	7.85E-03	transcription_start_site	+	129189	129189	-2622	2165	AN0696
803	CONTIG10	126389	126745	2.1	7.85E-03	transcription_start_site	+	129440	129440	-2873	2166	AN0696
803	CONTIG10	126389	126745	2.1	7.85E-03	transcription_start_site	+	129622	129622	-3055	2167	AN0696
1174	CONTIG10	124959	125326	1.78	2.62E-02	transcription_start_site	+	129189	129189	-4046	2165	AN0696
1174	CONTIG10	124959	125326	1.78	2.62E-02	transcription_start_site	+	129440	129440	-4297	2166	AN0696
1174	CONTIG10	124959	125326	1.78	2.62E-02	transcription_start_site	+	129622	129622	-4479	2167	AN0696
1700	CONTIG10	137646	138125	1.5	6.84E-02	transcription_start_site	-	135221	135221	-2664	2168	AN0697
1700	CONTIG10	137646	138125	1.5	6.84E-02	transcription_start_site	-	135095	135095	-2790	2169	AN0697
1700	CONTIG10	137646	138125	1.5	6.84E-02	transcription_start_site	-	134934	134934	-2951	2170	AN0697
1700	CONTIG10	137646	138125	1.5	6.84E-02	transcription_start_site	-	134746	134746	-3139	2171	AN0697
1700	CONTIG10	137646	138125	1.5	6.84E-02	transcription_start_site	-	134670	134670	-3215	2172	AN0697
1700	CONTIG10	137646	138125	1.5	6.84E-02	transcription_start_site	-	134025	134025	-3860	2173	AN0697
1700	CONTIG10	137646	138125	1.5	6.84E-02	transcription_start_site	-	133602	133602	-4283	2174	AN0697
1700	CONTIG10	137646	138125	1.5	6.84E-02	transcription_start_site	-	133467	133467	-4418	2175	AN0697
1700	CONTIG10	137646	138125	1.5	6.84E-02	transcription_start_site	-	133314	133314	-4571	2176	AN0697
2597	CONTIG10	136896	137225	1.17	1.79E-01	transcription_start_site	-	135221	135221	-1839	2168	AN0697
2597	CONTIG10	136896	137225	1.17	1.79E-01	transcription_start_site	-	135095	135095	-1965	2169	AN0697
2597	CONTIG10	136896	137225	1.17	1.79E-01	transcription_start_site	-	134934	134934	-2126	2170	AN0697
2597	CONTIG10	136896	137225	1.17	1.79E-01	transcription_start_site	-	134746	134746	-2314	2171	AN0697
2597	CONTIG10	136896	137225	1.17	1.79E-01	transcription_start_site	-	134670	134670	-2390	2172	AN0697
2597	CONTIG10	136896	137225	1.17	1.79E-01	transcription_start_site	-	134025	134025	-3035	2173	AN0697
2597	CONTIG10	136896	137225	1.17	1.79E-01	transcription_start_site	-	133602	133602	-3458	2174	AN0697
2597	CONTIG10	136896	137225	1.17	1.79E-01	transcription_start_site	-	133467	133467	-3593	2175	AN0697
2597	CONTIG10	136896	137225	1.17	1.79E-01	transcription_start_site	-	133314	133314	-3746	2176	AN0697
1700	CONTIG10	137646	138125	1.5	6.84E-02	transcription_start_site	-	138994	138994	1108	2177	AN0698
1813	CONTIG10	151351	151635	1.45	8.01E-02	transcription_start_site	-	147684	147684	-3809	2183	AN0700
1813	CONTIG10	151351	151635	1.45	8.01E-02	transcription_start_site	-	146922	146922	-4571	2184	AN0700
227	CONTIG11	7651	8087	2.89	8.69E-04	transcription_start_site	-	7662	7662	-207	2212	AN0709
722	CONTIG11	8564	8978	2.18	1.11E-02	transcription_start_site	-	7662	7662	-1109	2212	AN0709
917	CONTIG11	10429	12428	1.98	0.00E+00	transcription_start_site	-	7662	7662	-3766	2212	AN0709
723	CONTIG11	26120	26394	2.18	1.11E-02	transcription_start_site	-	22386	22386	-3871	2215	AN0711
1437	CONTIG11	27020	27279	1.62	6.84E-02	transcription_start_site	-	22386	22386	-4763	2215	AN0711
723	CONTIG11	26120	26394	2.18	1.11E-02	transcription_start_site	+	26567	26567	-310	2222	AN0713
1437	CONTIG11	27020	27279	1.62	6.84E-02	transcription_start_site	+	26567	26567	582	2222	AN0713
723	CONTIG11	26120	26394	2.18	1.11E-02	transcription_start_site	+	30019	30019	-3762	2223	AN0714
1437	CONTIG11	27020	27279	1.62	6.84E-02	transcription_start_site	+	30019	30019	-2869	2223	AN0714
1658	CONTIG11	60456	60739	1.52	9.24E-02	transcription_start_site	-	58016	58016	-2581	2253	AN0725
1658	CONTIG11	60456	60739	1.52	9.24E-02	transcription_start_site	-	57822	57822	-2775	2254	AN0725
1658	CONTIG11	60456	60739	1.52	9.24E-02	transcription_start_site	-	60037	60037	-560	2255	AN0726

1658	CONTIG11	60456	60739	1.52	9.24E-02	transcription_start_site	-	59673	59673	-924	2256 AN0726
1658	CONTIG11	60456	60739	1.52	9.24E-02	transcription_start_site	-	59390	59390	-1207	2257 AN0726
301	CONTIG12	20251	20768	2.76	2.16E-04	transcription_start_site	-	15828	15828	-4681	2260 AN0730
301	CONTIG12	20251	20768	2.76	2.16E-04	transcription_start_site	-	15349	15349	-5160	2261 AN0730
301	CONTIG12	20251	20768	2.76	2.16E-04	transcription_start_site	-	17449	17449	-3060	2262 AN0731
301	CONTIG12	20251	20768	2.76	2.16E-04	transcription_start_site	-	16850	16850	-3659	2263 AN0731
301	CONTIG12	20251	20768	2.76	2.16E-04	transcription_start_site	-	20029	20029	-480	2264 AN0732
301	CONTIG12	20251	20768	2.76	2.16E-04	transcription_start_site	-	19325	19325	-1184	2265 AN0732
301	CONTIG12	20251	20768	2.76	2.16E-04	transcription_start_site	-	18251	18251	-2258	2266 AN0732
778	CONTIG12	23705	24214	2.12	6.61E-03	transcription_start_site	-	20029	20029	-3930	2264 AN0732
778	CONTIG12	23705	24214	2.12	6.61E-03	transcription_start_site	-	19325	19325	-4634	2265 AN0732
778	CONTIG12	23705	24214	2.12	6.61E-03	transcription_start_site	-	23637	23637	-322	2267 AN0733
778	CONTIG12	23705	24214	2.12	6.61E-03	transcription_start_site	-	23518	23518	-441	2268 AN0733
778	CONTIG12	23705	24214	2.12	6.61E-03	transcription_start_site	-	23358	23358	-601	2269 AN0733
301	CONTIG12	20251	20768	2.76	2.16E-04	transcription_start_site	+	24437	24437	-3927	2270 AN0734
301	CONTIG12	20251	20768	2.76	2.16E-04	transcription_start_site	+	24505	24505	-3995	2271 AN0734
301	CONTIG12	20251	20768	2.76	2.16E-04	transcription_start_site	+	24836	24836	-4326	2272 AN0734
778	CONTIG12	23705	24214	2.12	6.61E-03	transcription_start_site	+	24437	24437	-477	2270 AN0734
778	CONTIG12	23705	24214	2.12	6.61E-03	transcription_start_site	+	24505	24505	-545	2271 AN0734
778	CONTIG12	23705	24214	2.12	6.61E-03	transcription_start_site	+	24836	24836	-876	2272 AN0734
778	CONTIG12	23705	24214	2.12	6.61E-03	transcription_start_site	+	26496	26496	-2536	2273 AN0735
778	CONTIG12	23705	24214	2.12	6.61E-03	transcription_start_site	+	26849	26849	-2889	2274 AN0735
779	CONTIG12	40285	40779	2.12	6.61E-03	transcription_start_site	-	35414	35414	-5118	2281 AN0738
779	CONTIG12	40285	40779	2.12	6.61E-03	transcription_start_site	-	40367	40367	-165	2287 AN0740
779	CONTIG12	40285	40779	2.12	6.61E-03	transcription_start_site	-	40292	40292	-240	2288 AN0740
779	CONTIG12	40285	40779	2.12	6.61E-03	transcription_start_site	-	39259	39259	-1273	2289 AN0740
779	CONTIG12	40285	40779	2.12	6.61E-03	transcription_start_site	+	42760	42760	-2228	2290 AN0741
779	CONTIG12	40285	40779	2.12	6.61E-03	transcription_start_site	+	43135	43135	-2603	2291 AN0741
779	CONTIG12	40285	40779	2.12	6.61E-03	transcription_start_site	+	43429	43429	-2897	2292 AN0741
779	CONTIG12	40285	40779	2.12	6.61E-03	transcription_start_site	+	45767	45767	-5235	2293 AN0742
1464	CONTIG12	45608	45892	1.61	4.36E-02	transcription_start_site	+	45767	45767	-17	2293 AN0742
1464	CONTIG12	45608	45892	1.61	4.36E-02	transcription_start_site	+	46127	46127	-377	2294 AN0742
1464	CONTIG12	45608	45892	1.61	4.36E-02	transcription_start_site	+	46204	46204	-454	2295 AN0742
1464	CONTIG12	45608	45892	1.61	4.36E-02	transcription_start_site	+	46374	46374	-624	2296 AN0742
1464	CONTIG12	45608	45892	1.61	4.36E-02	transcription_start_site	+	46800	46800	-1050	2297 AN0742
1464	CONTIG12	45608	45892	1.61	4.36E-02	transcription_start_site	+	47108	47108	-1358	2298 AN0742
2229	CONTIG12	59938	60442	1.29	1.25E-01	transcription_start_site	+	60354	60354	-164	2316 AN0747
2229	CONTIG12	59938	60442	1.29	1.25E-01	transcription_start_site	+	59613	59613	577	2315 AN0747
2229	CONTIG12	59938	60442	1.29	1.25E-01	transcription_start_site	+	59472	59472	718	2314 AN0747
2229	CONTIG12	59938	60442	1.29	1.25E-01	transcription_start_site	+	59298	59298	892	2313 AN0747
2229	CONTIG12	59938	60442	1.29	1.25E-01	transcription_start_site	+	59093	59093	1097	2312 AN0747
2229	CONTIG12	59938	60442	1.29	1.25E-01	transcription_start_site	+	62385	62385	-2195	2317 AN0748
2229	CONTIG12	59938	60442	1.29	1.25E-01	transcription_start_site	+	62953	62953	-2763	2318 AN0748
2229	CONTIG12	59938	60442	1.29	1.25E-01	transcription_start_site	+	63072	63072	-2882	2319 AN0748
2229	CONTIG12	59938	60442	1.29	1.25E-01	transcription_start_site	+	63535	63535	-3345	2320 AN0748
2229	CONTIG12	59938	60442	1.29	1.25E-01	transcription_start_site	+	63987	63987	-3797	2321 AN0748
2229	CONTIG12	59938	60442	1.29	1.25E-01	transcription_start_site	+	64744	64744	-4554	2322 AN0748
2517	CONTIG12	68630	68919	1.2	1.66E-01	transcription_start_site	-	67608	67608	-1166	2323 AN0749
1981	CONTIG12	74040	74309	1.38	9.24E-02	transcription_start_site	+	74199	74199	-24	2333 AN0750
1981	CONTIG12	74040	74309	1.38	9.24E-02	transcription_start_site	+	73891	73891	283	2332 AN0750
1981	CONTIG12	74040	74309	1.38	9.24E-02	transcription_start_site	+	74627	74627	-452	2334 AN0750
1981	CONTIG12	74040	74309	1.38	9.24E-02	transcription_start_site	+	73425	73425	749	2331 AN0750
2517	CONTIG12	68630	68919	1.2	1.66E-01	transcription_start_site	+	68765	68765	9	2327 AN0750
2517	CONTIG12	68630	68919	1.2	1.66E-01	transcription_start_site	+	68570	68570	204	2326 AN0750
2517	CONTIG12	68630	68919	1.2	1.66E-01	transcription_start_site	+	68281	68281	493	2325 AN0750
2517	CONTIG12	68630	68919	1.2	1.66E-01	transcription_start_site	+	68102	68102	672	2324 AN0750
2517	CONTIG12	68630	68919	1.2	1.66E-01	transcription_start_site	+	71620	71620	-2845	2328 AN0750

2517	CONTIG12	68630	68919	1.2	1.66E-01	transcription_start_site	+	71859	71859	-3084	2329	AN0750
2517	CONTIG12	68630	68919	1.2	1.66E-01	transcription_start_site	+	72842	72842	-4067	2330	AN0750
2517	CONTIG12	68630	68919	1.2	1.66E-01	transcription_start_site	+	73425	73425	-4650	2331	AN0750
2517	CONTIG12	68630	68919	1.2	1.66E-01	transcription_start_site	+	73891	73891	-5116	2332	AN0750
2221	CONTIG12	81377	81881	1.29	1.07E-01	transcription_start_site	-	80479	80479	-1150	2335	AN0751
2221	CONTIG12	81377	81881	1.29	1.07E-01	transcription_start_site	-	80075	80075	-1554	2336	AN0751
1644	CONTIG12	87752	88186	1.52	5.88E-02	transcription_start_site	-	84817	84817	-3152	2337	AN0752
1644	CONTIG12	87752	88186	1.52	5.88E-02	transcription_start_site	-	84662	84662	-3307	2338	AN0752
1644	CONTIG12	87752	88186	1.52	5.88E-02	transcription_start_site	-	84510	84510	-3459	2339	AN0752
2221	CONTIG12	81377	81881	1.29	1.07E-01	transcription_start_site	+	85214	85214	-3585	2340	AN0753
2221	CONTIG12	81377	81881	1.29	1.07E-01	transcription_start_site	+	85450	85450	-3821	2341	AN0753
2221	CONTIG12	81377	81881	1.29	1.07E-01	transcription_start_site	+	86678	86678	-5049	2342	AN0753
1644	CONTIG12	87752	88186	1.52	5.88E-02	transcription_start_site	+	87834	87834	135	2344	AN0754
1644	CONTIG12	87752	88186	1.52	5.88E-02	transcription_start_site	+	87749	87749	220	2343	AN0754
569	CONTIG12	121366	121790	2.35	0.00E+00	transcription_start_site	-	118157	118157	-3421	2385	AN0765
569	CONTIG12	121366	121790	2.35	0.00E+00	transcription_start_site	-	117847	117847	-3731	2386	AN0765
1557	CONTIG12	122196	122685	1.56	5.22E-02	transcription_start_site	-	118157	118157	-4283	2385	AN0765
1557	CONTIG12	122196	122685	1.56	5.22E-02	transcription_start_site	-	117847	117847	-4593	2386	AN0765
569	CONTIG12	121366	121790	2.35	0.00E+00	transcription_start_site	-	120946	120946	-632	2387	AN0766
569	CONTIG12	121366	121790	2.35	0.00E+00	transcription_start_site	-	120277	120277	-1301	2388	AN0766
1557	CONTIG12	122196	122685	1.56	5.22E-02	transcription_start_site	-	120946	120946	-1494	2387	AN0766
1557	CONTIG12	122196	122685	1.56	5.22E-02	transcription_start_site	-	120277	120277	-2163	2388	AN0766
569	CONTIG12	121366	121790	2.35	0.00E+00	transcription_start_site	+	122566	122566	-988	2389	AN0767
569	CONTIG12	121366	121790	2.35	0.00E+00	transcription_start_site	+	122838	122838	-1260	2390	AN0767
569	CONTIG12	121366	121790	2.35	0.00E+00	transcription_start_site	+	122962	122962	-1384	2391	AN0767
1557	CONTIG12	122196	122685	1.56	5.22E-02	transcription_start_site	+	122566	122566	-125	2389	AN0767
1557	CONTIG12	122196	122685	1.56	5.22E-02	transcription_start_site	+	122838	122838	-397	2390	AN0767
1557	CONTIG12	122196	122685	1.56	5.22E-02	transcription_start_site	+	122962	122962	-521	2391	AN0767
569	CONTIG12	121366	121790	2.35	0.00E+00	transcription_start_site	+	123693	123693	-2115	2392	AN0768
569	CONTIG12	121366	121790	2.35	0.00E+00	transcription_start_site	+	124583	124583	-3005	2393	AN0768
1557	CONTIG12	122196	122685	1.56	5.22E-02	transcription_start_site	+	123693	123693	-1252	2392	AN0768
1557	CONTIG12	122196	122685	1.56	5.22E-02	transcription_start_site	+	124583	124583	-2142	2393	AN0768
465	CONTIG12	132087	132426	2.49	1.22E-03	transcription_start_site	+	133388	133388	-1131	2396	AN0771
465	CONTIG12	132087	132426	2.49	1.22E-03	transcription_start_site	+	134080	134080	-1823	2397	AN0771
465	CONTIG12	132087	132426	2.49	1.22E-03	transcription_start_site	+	134577	134577	-2320	2398	AN0771
465	CONTIG12	132087	132426	2.49	1.22E-03	transcription_start_site	+	134719	134719	-2462	2399	AN0771
465	CONTIG12	132087	132426	2.49	1.22E-03	transcription_start_site	+	135113	135113	-2856	2400	AN0771
465	CONTIG12	132087	132426	2.49	1.22E-03	transcription_start_site	+	135405	135405	-3148	2401	AN0771
465	CONTIG12	132087	132426	2.49	1.22E-03	transcription_start_site	+	136373	136373	-4116	2402	AN0771
465	CONTIG12	132087	132426	2.49	1.22E-03	transcription_start_site	+	136733	136733	-4476	2403	AN0771
1548	CONTIG12	146851	147215	1.56	3.33E-02	transcription_start_site	+	146071	146071	962	2404	AN0773
826	CONTIG12	148971	149375	2.07	7.85E-03	transcription_start_site	-	149605	149605	432	2406	AN0774
826	CONTIG12	148971	149375	2.07	7.85E-03	transcription_start_site	-	149718	149718	545	2405	AN0774
2109	CONTIG12	152261	152695	1.33	9.26E-02	transcription_start_site	-	149718	149718	-2760	2405	AN0774
2109	CONTIG12	152261	152695	1.33	9.26E-02	transcription_start_site	-	149605	149605	-2873	2406	AN0774
826	CONTIG12	148971	149375	2.07	7.85E-03	transcription_start_site	+	150297	150297	-1124	2407	AN0775
826	CONTIG12	148971	149375	2.07	7.85E-03	transcription_start_site	+	150436	150436	-1263	2408	AN0775
826	CONTIG12	148971	149375	2.07	7.85E-03	transcription_start_site	+	150584	150584	-1411	2409	AN0775
826	CONTIG12	148971	149375	2.07	7.85E-03	transcription_start_site	+	150749	150749	-1576	2410	AN0775
826	CONTIG12	148971	149375	2.07	7.85E-03	transcription_start_site	+	150914	150914	-1741	2411	AN0775
826	CONTIG12	148971	149375	2.07	7.85E-03	transcription_start_site	+	151049	151049	-1876	2412	AN0775
826	CONTIG12	148971	149375	2.07	7.85E-03	transcription_start_site	+	151375	151375	-2202	2413	AN0775
1548	CONTIG12	146851	147215	1.56	3.33E-02	transcription_start_site	+	150297	150297	-3264	2407	AN0775
1548	CONTIG12	146851	147215	1.56	3.33E-02	transcription_start_site	+	150436	150436	-3403	2408	AN0775
1548	CONTIG12	146851	147215	1.56	3.33E-02	transcription_start_site	+	150584	150584	-3551	2409	AN0775
1548	CONTIG12	146851	147215	1.56	3.33E-02	transcription_start_site	+	150749	150749	-3716	2410	AN0775
1548	CONTIG12	146851	147215	1.56	3.33E-02	transcription_start_site	+	150914	150914	-3881	2411	AN0775

1548	CONTIG12	146851	147215	1.56	3.33E-02	transcription_start_site	+	151049	151049	-4016	2412	AN0775
1548	CONTIG12	146851	147215	1.56	3.33E-02	transcription_start_site	+	151375	151375	-4342	2413	AN0775
2109	CONTIG12	152261	152695	1.33	9.26E-02	transcription_start_site	+	151375	151375	1103	2413	AN0775
826	CONTIG12	148971	149375	2.07	7.85E-03	transcription_start_site	+	151873	151873	-2700	2414	AN0776
826	CONTIG12	148971	149375	2.07	7.85E-03	transcription_start_site	+	152122	152122	-2949	2415	AN0776
826	CONTIG12	148971	149375	2.07	7.85E-03	transcription_start_site	+	152496	152496	-3323	2416	AN0776
826	CONTIG12	148971	149375	2.07	7.85E-03	transcription_start_site	+	152739	152739	-3566	2417	AN0776
1548	CONTIG12	146851	147215	1.56	3.33E-02	transcription_start_site	+	151873	151873	-4840	2414	AN0776
1548	CONTIG12	146851	147215	1.56	3.33E-02	transcription_start_site	+	152122	152122	-5089	2415	AN0776
2109	CONTIG12	152261	152695	1.33	9.26E-02	transcription_start_site	+	152496	152496	-18	2416	AN0776
2109	CONTIG12	152261	152695	1.33	9.26E-02	transcription_start_site	+	152739	152739	-261	2417	AN0776
2109	CONTIG12	152261	152695	1.33	9.26E-02	transcription_start_site	+	152122	152122	356	2415	AN0776
2109	CONTIG12	152261	152695	1.33	9.26E-02	transcription_start_site	+	151873	151873	605	2414	AN0776
826	CONTIG12	148971	149375	2.07	7.85E-03	transcription_start_site	+	153840	153840	-4667	2418	AN0777
826	CONTIG12	148971	149375	2.07	7.85E-03	transcription_start_site	+	153931	153931	-4758	2419	AN0777
826	CONTIG12	148971	149375	2.07	7.85E-03	transcription_start_site	+	154046	154046	-4873	2420	AN0777
826	CONTIG12	148971	149375	2.07	7.85E-03	transcription_start_site	+	154134	154134	-4961	2421	AN0777
2109	CONTIG12	152261	152695	1.33	9.26E-02	transcription_start_site	+	153840	153840	-1362	2418	AN0777
2109	CONTIG12	152261	152695	1.33	9.26E-02	transcription_start_site	+	153931	153931	-1453	2419	AN0777
2109	CONTIG12	152261	152695	1.33	9.26E-02	transcription_start_site	+	154046	154046	-1568	2420	AN0777
2109	CONTIG12	152261	152695	1.33	9.26E-02	transcription_start_site	+	154134	154134	-1656	2421	AN0777
2109	CONTIG12	152261	152695	1.33	9.26E-02	transcription_start_site	+	154568	154568	-2090	2422	AN0777
2109	CONTIG12	152261	152695	1.33	9.26E-02	transcription_start_site	+	156376	156376	-3898	2423	AN0778
2109	CONTIG12	152261	152695	1.33	9.26E-02	transcription_start_site	+	157354	157354	-4876	2424	AN0778
2109	CONTIG12	152261	152695	1.33	9.26E-02	transcription_start_site	+	157429	157429	-4951	2425	AN0778
2230	CONTIG12	156840	157189	1.29	1.25E-01	transcription_start_site	+	157354	157354	-339	2424	AN0778
2230	CONTIG12	156840	157189	1.29	1.25E-01	transcription_start_site	+	157429	157429	-414	2425	AN0778
2230	CONTIG12	156840	157189	1.29	1.25E-01	transcription_start_site	+	156376	156376	638	2423	AN0778
607	CONTIG12	164942	165201	2.3	3.03E-03	transcription_start_site	-	161377	161377	-3694	2426	AN0779
607	CONTIG12	164942	165201	2.3	3.03E-03	transcription_start_site	-	161115	161115	-3956	2427	AN0779
1160	CONTIG12	159230	159659	1.79	2.12E-02	transcription_start_site	-	159652	159652	207	2428	AN0779
1549	CONTIG12	163746	164455	1.56	3.33E-02	transcription_start_site	-	161377	161377	-2723	2426	AN0779
1549	CONTIG12	163746	164455	1.56	3.33E-02	transcription_start_site	-	161115	161115	-2985	2427	AN0779
1549	CONTIG12	163746	164455	1.56	3.33E-02	transcription_start_site	-	159652	159652	-4448	2428	AN0779
607	CONTIG12	164942	165201	2.3	3.03E-03	transcription_start_site	-	164440	164440	-631	2429	AN0780
607	CONTIG12	164942	165201	2.3	3.03E-03	transcription_start_site	-	163919	163919	-1152	2430	AN0780
607	CONTIG12	164942	165201	2.3	3.03E-03	transcription_start_site	-	163489	163489	-1582	2431	AN0780
1452	CONTIG12	166592	167243	1.61	2.64E-02	transcription_start_site	-	164440	164440	-2477	2429	AN0780
1452	CONTIG12	166592	167243	1.61	2.64E-02	transcription_start_site	-	163919	163919	-2998	2430	AN0780
1452	CONTIG12	166592	167243	1.61	2.64E-02	transcription_start_site	-	163489	163489	-3428	2431	AN0780
1549	CONTIG12	163746	164455	1.56	3.33E-02	transcription_start_site	-	163919	163919	-181	2430	AN0780
1549	CONTIG12	163746	164455	1.56	3.33E-02	transcription_start_site	-	164440	164440	339	2429	AN0780
1549	CONTIG12	163746	164455	1.56	3.33E-02	transcription_start_site	-	163489	163489	-611	2431	AN0780
607	CONTIG12	164942	165201	2.3	3.03E-03	transcription_start_site	+	166445	166445	-1373	2432	AN0781
607	CONTIG12	164942	165201	2.3	3.03E-03	transcription_start_site	+	167116	167116	-2044	2433	AN0781
1452	CONTIG12	166592	167243	1.61	2.64E-02	transcription_start_site	+	167116	167116	-198	2433	AN0781
1452	CONTIG12	166592	167243	1.61	2.64E-02	transcription_start_site	+	166445	166445	472	2432	AN0781
1549	CONTIG12	163746	164455	1.56	3.33E-02	transcription_start_site	+	166445	166445	-2344	2432	AN0781
1549	CONTIG12	163746	164455	1.56	3.33E-02	transcription_start_site	+	167116	167116	-3015	2433	AN0781
607	CONTIG12	164942	165201	2.3	3.03E-03	transcription_start_site	+	170038	170038	-4966	2434	AN0782
1157	CONTIG12	170192	170916	1.79	1.06E-02	transcription_start_site	+	170038	170038	516	2434	AN0782
1452	CONTIG12	166592	167243	1.61	2.64E-02	transcription_start_site	+	170038	170038	-3120	2434	AN0782
808	CONTIG13	11107	11376	2.1	1.84E-02	transcription_start_site	-	6337	6337	-4904	2441	AN0785
808	CONTIG13	11107	11376	2.1	1.84E-02	transcription_start_site	-	6219	6219	-5022	2442	AN0785
969	CONTIG13	10437	10936	1.94	3.08E-02	transcription_start_site	-	6337	6337	-4349	2441	AN0785
969	CONTIG13	10437	10936	1.94	3.08E-02	transcription_start_site	-	6219	6219	-4467	2442	AN0785
1092	CONTIG13	6677	7851	1.84	2.64E-02	transcription_start_site	-	6337	6337	-927	2441	AN0785

1092	CONTIG13	6677	7851	1.84	2.64E-02	transcription_start_site	-	6219	6219	-1045	2442	AN0785
1657	CONTIG13	8407	9356	1.52	8.36E-02	transcription_start_site	-	6337	6337	-2544	2441	AN0785
1657	CONTIG13	8407	9356	1.52	8.36E-02	transcription_start_site	-	6219	6219	-2662	2442	AN0785
808	CONTIG13	11107	11376	2.1	1.84E-02	transcription_start_site	-	7344	7344	-3897	2443	AN0786
808	CONTIG13	11107	11376	2.1	1.84E-02	transcription_start_site	-	6933	6933	-4308	2444	AN0786
808	CONTIG13	11107	11376	2.1	1.84E-02	transcription_start_site	-	6641	6641	-4600	2445	AN0786
969	CONTIG13	10437	10936	1.94	3.08E-02	transcription_start_site	-	7344	7344	-3342	2443	AN0786
969	CONTIG13	10437	10936	1.94	3.08E-02	transcription_start_site	-	6933	6933	-3753	2444	AN0786
969	CONTIG13	10437	10936	1.94	3.08E-02	transcription_start_site	-	6641	6641	-4045	2445	AN0786
1092	CONTIG13	6677	7851	1.84	2.64E-02	transcription_start_site	-	7344	7344	80	2443	AN0786
1092	CONTIG13	6677	7851	1.84	2.64E-02	transcription_start_site	-	6933	6933	-331	2444	AN0786
1092	CONTIG13	6677	7851	1.84	2.64E-02	transcription_start_site	-	6641	6641	-623	2445	AN0786
1657	CONTIG13	8407	9356	1.52	8.36E-02	transcription_start_site	-	7344	7344	-1537	2443	AN0786
1657	CONTIG13	8407	9356	1.52	8.36E-02	transcription_start_site	-	6933	6933	-1948	2444	AN0786
1657	CONTIG13	8407	9356	1.52	8.36E-02	transcription_start_site	-	6641	6641	-2240	2445	AN0786
808	CONTIG13	11107	11376	2.1	1.84E-02	transcription_start_site	-	9626	9626	-1615	2446	AN0787
808	CONTIG13	11107	11376	2.1	1.84E-02	transcription_start_site	-	8321	8321	-2920	2447	AN0787
808	CONTIG13	11107	11376	2.1	1.84E-02	transcription_start_site	-	8093	8093	-3148	2448	AN0787
969	CONTIG13	10437	10936	1.94	3.08E-02	transcription_start_site	-	9626	9626	-1060	2446	AN0787
969	CONTIG13	10437	10936	1.94	3.08E-02	transcription_start_site	-	8321	8321	-2365	2447	AN0787
969	CONTIG13	10437	10936	1.94	3.08E-02	transcription_start_site	-	8093	8093	-2593	2448	AN0787
1092	CONTIG13	6677	7851	1.84	2.64E-02	transcription_start_site	-	8093	8093	829	2448	AN0787
1092	CONTIG13	6677	7851	1.84	2.64E-02	transcription_start_site	-	8321	8321	1057	2447	AN0787
1657	CONTIG13	8407	9356	1.52	8.36E-02	transcription_start_site	-	8321	8321	-560	2447	AN0787
1657	CONTIG13	8407	9356	1.52	8.36E-02	transcription_start_site	-	9626	9626	744	2446	AN0787
1657	CONTIG13	8407	9356	1.52	8.36E-02	transcription_start_site	-	8093	8093	-788	2448	AN0787
808	CONTIG13	11107	11376	2.1	1.84E-02	transcription_start_site	+	10595	10595	646	2449	AN0788
969	CONTIG13	10437	10936	1.94	3.08E-02	transcription_start_site	+	10595	10595	91	2449	AN0788
1092	CONTIG13	6677	7851	1.84	2.64E-02	transcription_start_site	+	10595	10595	-3331	2449	AN0788
1657	CONTIG13	8407	9356	1.52	8.36E-02	transcription_start_site	+	10595	10595	-1713	2449	AN0788
9	CONTIG13	57545	58182	3.73	0.00E+00	transcription_start_site	-	53115	53115	-4748	2500	AN0805
1509	CONTIG13	55355	56619	1.58	5.02E-02	transcription_start_site	-	53115	53115	-2872	2500	AN0805
9	CONTIG13	57545	58182	3.73	0.00E+00	transcription_start_site	-	55324	55324	-2539	2501	AN0806
9	CONTIG13	57545	58182	3.73	0.00E+00	transcription_start_site	-	54888	54888	-2975	2502	AN0806
9	CONTIG13	57545	58182	3.73	0.00E+00	transcription_start_site	-	54678	54678	-3185	2503	AN0806
9	CONTIG13	57545	58182	3.73	0.00E+00	transcription_start_site	-	54550	54550	-3313	2504	AN0806
9	CONTIG13	57545	58182	3.73	0.00E+00	transcription_start_site	-	54141	54141	-3722	2505	AN0806
9	CONTIG13	57545	58182	3.73	0.00E+00	transcription_start_site	-	53964	53964	-3899	2506	AN0806
1509	CONTIG13	55355	56619	1.58	5.02E-02	transcription_start_site	-	55324	55324	-663	2501	AN0806
1509	CONTIG13	55355	56619	1.58	5.02E-02	transcription_start_site	-	54888	54888	-1099	2502	AN0806
1509	CONTIG13	55355	56619	1.58	5.02E-02	transcription_start_site	-	54678	54678	-1309	2503	AN0806
1509	CONTIG13	55355	56619	1.58	5.02E-02	transcription_start_site	-	54550	54550	-1437	2504	AN0806
1509	CONTIG13	55355	56619	1.58	5.02E-02	transcription_start_site	-	54141	54141	-1846	2505	AN0806
1509	CONTIG13	55355	56619	1.58	5.02E-02	transcription_start_site	-	53964	53964	-2023	2506	AN0806
9	CONTIG13	57545	58182	3.73	0.00E+00	transcription_start_site	+	59230	59230	-1366	2507	AN0807
9	CONTIG13	57545	58182	3.73	0.00E+00	transcription_start_site	+	59557	59557	-1693	2508	AN0807
1509	CONTIG13	55355	56619	1.58	5.02E-02	transcription_start_site	+	59230	59230	-3243	2507	AN0807
1509	CONTIG13	55355	56619	1.58	5.02E-02	transcription_start_site	+	59557	59557	-3570	2508	AN0807
1659	CONTIG13	63302	63591	1.52	1.08E-01	transcription_start_site	-	63000	63000	-446	2509	AN0808
1659	CONTIG13	63302	63591	1.52	1.08E-01	transcription_start_site	-	62875	62875	-571	2510	AN0808
1659	CONTIG13	63302	63591	1.52	1.08E-01	transcription_start_site	-	62756	62756	-690	2511	AN0808
1659	CONTIG13	63302	63591	1.52	1.08E-01	transcription_start_site	-	62611	62611	-835	2512	AN0808
1659	CONTIG13	63302	63591	1.52	1.08E-01	transcription_start_site	-	62402	62402	-1044	2513	AN0808
1411	CONTIG13	68552	68836	1.63	8.01E-02	transcription_start_site	+	69206	69206	-512	2518	AN0809
1659	CONTIG13	63302	63591	1.52	1.08E-01	transcription_start_site	+	63737	63737	-290	2514	AN0809
1659	CONTIG13	63302	63591	1.52	1.08E-01	transcription_start_site	+	63939	63939	-492	2515	AN0809
1659	CONTIG13	63302	63591	1.52	1.08E-01	transcription_start_site	+	64150	64150	-703	2516	AN0809

1659	CONTIG13	63302	63591	1.52	1.08E-01	transcription_start_site	+	64281	64281	-834	2517	AN0809
1411	CONTIG13	68552	68836	1.63	8.01E-02	transcription_start_site	+	70057	70057	-1363	2519	AN0810
1411	CONTIG13	68552	68836	1.63	8.01E-02	transcription_start_site	+	70174	70174	-1480	2520	AN0810
1411	CONTIG13	68552	68836	1.63	8.01E-02	transcription_start_site	+	70353	70353	-1659	2521	AN0810
1411	CONTIG13	68552	68836	1.63	8.01E-02	transcription_start_site	+	72345	72345	-3651	2522	AN0810
1411	CONTIG13	68552	68836	1.63	8.01E-02	transcription_start_site	+	72951	72951	-4257	2523	AN0810
1411	CONTIG13	68552	68836	1.63	8.01E-02	transcription_start_site	+	73065	73065	-4371	2524	AN0810
1660	CONTIG13	79067	79495	1.52	1.08E-01	transcription_start_site	+	84028	84028	-4747	2533	AN0814
1028	CONTIG13	93690	94044	1.89	3.70E-02	transcription_start_site	-	92803	92803	-1064	2541	AN0817
1028	CONTIG13	93690	94044	1.89	3.70E-02	transcription_start_site	-	92403	92403	-1464	2542	AN0817
1028	CONTIG13	93690	94044	1.89	3.70E-02	transcription_start_site	-	91304	91304	-2563	2543	AN0817
1028	CONTIG13	93690	94044	1.89	3.70E-02	transcription_start_site	+	95241	95241	-1374	2544	AN0818
1028	CONTIG13	93690	94044	1.89	3.70E-02	transcription_start_site	+	95477	95477	-1610	2545	AN0818
1028	CONTIG13	93690	94044	1.89	3.70E-02	transcription_start_site	+	97419	97419	-3552	2546	AN0818
1028	CONTIG13	93690	94044	1.89	3.70E-02	transcription_start_site	+	97959	97959	-4092	2547	AN0819
1028	CONTIG13	93690	94044	1.89	3.70E-02	transcription_start_site	+	98098	98098	-4231	2548	AN0819
1028	CONTIG13	93690	94044	1.89	3.70E-02	transcription_start_site	+	98283	98283	-4416	2549	AN0819
1028	CONTIG13	93690	94044	1.89	3.70E-02	transcription_start_site	+	98448	98448	-4581	2550	AN0819
967	CONTIG13	114528	115626	1.94	1.81E-02	transcription_start_site	-	113712	113712	-1365	2553	AN0822
967	CONTIG13	114528	115626	1.94	1.81E-02	transcription_start_site	-	113623	113623	-1454	2554	AN0822
967	CONTIG13	114528	115626	1.94	1.81E-02	transcription_start_site	-	112222	112222	-2855	2555	AN0822
967	CONTIG13	114528	115626	1.94	1.81E-02	transcription_start_site	+	119563	119563	-4486	2558	AN0824
967	CONTIG13	114528	115626	1.94	1.81E-02	transcription_start_site	+	119763	119763	-4686	2559	AN0824
485	CONTIG13	126602	126956	2.47	5.20E-03	transcription_start_site	-	123015	123015	-3764	2561	AN0825
485	CONTIG13	126602	126956	2.47	5.20E-03	transcription_start_site	-	122634	122634	-4145	2562	AN0825
485	CONTIG13	126602	126956	2.47	5.20E-03	transcription_start_site	-	122021	122021	-4758	2563	AN0825
485	CONTIG13	126602	126956	2.47	5.20E-03	transcription_start_site	-	121833	121833	-4946	2564	AN0825
1412	CONTIG13	124367	124626	1.63	8.01E-02	transcription_start_site	-	123015	123015	-1481	2561	AN0825
1412	CONTIG13	124367	124626	1.63	8.01E-02	transcription_start_site	-	122634	122634	-1862	2562	AN0825
1412	CONTIG13	124367	124626	1.63	8.01E-02	transcription_start_site	-	122021	122021	-2475	2563	AN0825
1412	CONTIG13	124367	124626	1.63	8.01E-02	transcription_start_site	-	121833	121833	-2663	2564	AN0825
485	CONTIG13	126602	126956	2.47	5.20E-03	transcription_start_site	-	126449	126449	-330	2565	AN0826
485	CONTIG13	126602	126956	2.47	5.20E-03	transcription_start_site	+	128594	128594	-1815	2566	AN0827
485	CONTIG13	126602	126956	2.47	5.20E-03	transcription_start_site	+	128762	128762	-1983	2567	AN0827
485	CONTIG13	126602	126956	2.47	5.20E-03	transcription_start_site	+	129768	129768	-2989	2568	AN0827
1412	CONTIG13	124367	124626	1.63	8.01E-02	transcription_start_site	+	128594	128594	-4097	2566	AN0827
1412	CONTIG13	124367	124626	1.63	8.01E-02	transcription_start_site	+	128762	128762	-4265	2567	AN0827
485	CONTIG13	126602	126956	2.47	5.20E-03	transcription_start_site	+	130677	130677	-3898	2569	AN0828
485	CONTIG13	126602	126956	2.47	5.20E-03	transcription_start_site	+	130888	130888	-4109	2570	AN0828
485	CONTIG13	126602	126956	2.47	5.20E-03	transcription_start_site	+	131529	131529	-4750	2571	AN0828
1165	CONTIG13	139363	139632	1.79	5.22E-02	transcription_start_site	-	134919	134919	-4578	2572	AN0829
1165	CONTIG13	139363	139632	1.79	5.22E-02	transcription_start_site	-	134458	134458	-5039	2573	AN0829
1165	CONTIG13	139363	139632	1.79	5.22E-02	transcription_start_site	-	136908	136908	-2589	2576	AN0830
1165	CONTIG13	139363	139632	1.79	5.22E-02	transcription_start_site	-	136211	136211	-3286	2577	AN0830
1165	CONTIG13	139363	139632	1.79	5.22E-02	transcription_start_site	-	139189	139189	-308	2578	AN0831
1165	CONTIG13	139363	139632	1.79	5.22E-02	transcription_start_site	-	138676	138676	-821	2579	AN0831
1165	CONTIG13	139363	139632	1.79	5.22E-02	transcription_start_site	-	138319	138319	-1178	2580	AN0831
1321	CONTIG13	142068	142333	1.68	6.84E-02	transcription_start_site	-	139189	139189	-3011	2578	AN0831
1321	CONTIG13	142068	142333	1.68	6.84E-02	transcription_start_site	-	138676	138676	-3524	2579	AN0831
1321	CONTIG13	142068	142333	1.68	6.84E-02	transcription_start_site	-	138319	138319	-3881	2580	AN0831
1165	CONTIG13	139363	139632	1.79	5.22E-02	transcription_start_site	+	139812	139812	-314	2581	AN0832
1165	CONTIG13	139363	139632	1.79	5.22E-02	transcription_start_site	+	140006	140006	-508	2582	AN0832
1165	CONTIG13	139363	139632	1.79	5.22E-02	transcription_start_site	+	140458	140458	-960	2583	AN0832
1165	CONTIG13	139363	139632	1.79	5.22E-02	transcription_start_site	+	142968	142968	-3470	2584	AN0833
1165	CONTIG13	139363	139632	1.79	5.22E-02	transcription_start_site	+	143352	143352	-3854	2585	AN0833
1321	CONTIG13	142068	142333	1.68	6.84E-02	transcription_start_site	+	142968	142968	-767	2584	AN0833
1321	CONTIG13	142068	142333	1.68	6.84E-02	transcription_start_site	+	143352	143352	-1151	2585	AN0833

1165	CONTIG13	139363	139632	1.79	5.22E-02	transcription_start_site	+	144482	144482	-4984	2586	AN0834
1165	CONTIG13	139363	139632	1.79	5.22E-02	transcription_start_site	+	144576	144576	-5078	2587	AN0834
1321	CONTIG13	142068	142333	1.68	6.84E-02	transcription_start_site	+	144482	144482	-2281	2586	AN0834
1321	CONTIG13	142068	142333	1.68	6.84E-02	transcription_start_site	+	144576	144576	-2375	2587	AN0834
1321	CONTIG13	142068	142333	1.68	6.84E-02	transcription_start_site	+	144725	144725	-2524	2588	AN0834
1321	CONTIG13	142068	142333	1.68	6.84E-02	transcription_start_site	+	145016	145016	-2815	2589	AN0834
1321	CONTIG13	142068	142333	1.68	6.84E-02	transcription_start_site	+	145724	145724	-3523	2590	AN0834
1321	CONTIG13	142068	142333	1.68	6.84E-02	transcription_start_site	+	146715	146715	-4514	2591	AN0834
2174	CONTIG13	184358	184792	1.31	1.93E-01	transcription_start_site	-	182989	182989	-1586	2609	AN0842
2174	CONTIG13	184358	184792	1.31	1.93E-01	transcription_start_site	-	182859	182859	-1716	2610	AN0842
2174	CONTIG13	184358	184792	1.31	1.93E-01	transcription_start_site	-	182690	182690	-1885	2611	AN0842
2174	CONTIG13	184358	184792	1.31	1.93E-01	transcription_start_site	+	184632	184632	-57	2617	AN0843
2174	CONTIG13	184358	184792	1.31	1.93E-01	transcription_start_site	+	184377	184377	198	2616	AN0843
2174	CONTIG13	184358	184792	1.31	1.93E-01	transcription_start_site	+	184043	184043	532	2615	AN0843
2174	CONTIG13	184358	184792	1.31	1.93E-01	transcription_start_site	+	185482	185482	-907	2618	AN0844
2174	CONTIG13	184358	184792	1.31	1.93E-01	transcription_start_site	+	185618	185618	-1043	2619	AN0844
2174	CONTIG13	184358	184792	1.31	1.93E-01	transcription_start_site	+	185720	185720	-1145	2620	AN0844
2174	CONTIG13	184358	184792	1.31	1.93E-01	transcription_start_site	+	186076	186076	-1501	2621	AN0844
2024	CONTIG13	189458	190027	1.37	1.50E-01	transcription_start_site	-	189591	189591	-151	2622	AN0845
2024	CONTIG13	189458	190027	1.37	1.50E-01	transcription_start_site	-	189045	189045	-697	2623	AN0845
2024	CONTIG13	189458	190027	1.37	1.50E-01	transcription_start_site	+	189967	189967	-224	2624	AN0846
133	CONTIG13	221180	221604	3.1	6.10E-04	transcription_start_site	-	216962	216962	-4430	2649	AN0855
133	CONTIG13	221180	221604	3.1	6.10E-04	transcription_start_site	-	216580	216580	-4812	2650	AN0855
204	CONTIG13	219155	219517	2.94	9.51E-04	transcription_start_site	-	216962	216962	-2374	2649	AN0855
204	CONTIG13	219155	219517	2.94	9.51E-04	transcription_start_site	-	216580	216580	-2756	2650	AN0855
1322	CONTIG13	220145	220715	1.68	6.84E-02	transcription_start_site	-	216962	216962	-3468	2649	AN0855
1322	CONTIG13	220145	220715	1.68	6.84E-02	transcription_start_site	-	216580	216580	-3850	2650	AN0855
2025	CONTIG13	217805	218459	1.37	1.50E-01	transcription_start_site	-	216962	216962	-1170	2649	AN0855
2025	CONTIG13	217805	218459	1.37	1.50E-01	transcription_start_site	-	216580	216580	-1552	2650	AN0855
133	CONTIG13	221180	221604	3.1	6.10E-04	transcription_start_site	-	218925	218925	-2467	2651	AN0856
133	CONTIG13	221180	221604	3.1	6.10E-04	transcription_start_site	-	218558	218558	-2834	2652	AN0856
204	CONTIG13	219155	219517	2.94	9.51E-04	transcription_start_site	-	218925	218925	-411	2651	AN0856
204	CONTIG13	219155	219517	2.94	9.51E-04	transcription_start_site	-	218558	218558	-778	2652	AN0856
1026	CONTIG13	223595	224680	1.89	1.86E-02	transcription_start_site	-	218925	218925	-5212	2651	AN0856
1322	CONTIG13	220145	220715	1.68	6.84E-02	transcription_start_site	-	218925	218925	-1505	2651	AN0856
1322	CONTIG13	220145	220715	1.68	6.84E-02	transcription_start_site	-	218558	218558	-1872	2652	AN0856
2025	CONTIG13	217805	218459	1.37	1.50E-01	transcription_start_site	-	218558	218558	426	2652	AN0856
2025	CONTIG13	217805	218459	1.37	1.50E-01	transcription_start_site	-	218925	218925	793	2651	AN0856
133	CONTIG13	221180	221604	3.1	6.10E-04	transcription_start_site	+	222603	222603	-1211	2653	AN0857
133	CONTIG13	221180	221604	3.1	6.10E-04	transcription_start_site	+	222703	222703	-1311	2654	AN0857
133	CONTIG13	221180	221604	3.1	6.10E-04	transcription_start_site	+	222872	222872	-1480	2655	AN0857
133	CONTIG13	221180	221604	3.1	6.10E-04	transcription_start_site	+	223213	223213	-1821	2656	AN0857
133	CONTIG13	221180	221604	3.1	6.10E-04	transcription_start_site	+	223397	223397	-2005	2657	AN0857
204	CONTIG13	219155	219517	2.94	9.51E-04	transcription_start_site	+	222603	222603	-3267	2653	AN0857
204	CONTIG13	219155	219517	2.94	9.51E-04	transcription_start_site	+	222703	222703	-3367	2654	AN0857
204	CONTIG13	219155	219517	2.94	9.51E-04	transcription_start_site	+	222872	222872	-3536	2655	AN0857
204	CONTIG13	219155	219517	2.94	9.51E-04	transcription_start_site	+	223213	223213	-3877	2656	AN0857
204	CONTIG13	219155	219517	2.94	9.51E-04	transcription_start_site	+	223397	223397	-4061	2657	AN0857
1026	CONTIG13	223595	224680	1.89	1.86E-02	transcription_start_site	+	223397	223397	740	2657	AN0857
1026	CONTIG13	223595	224680	1.89	1.86E-02	transcription_start_site	+	223213	223213	924	2656	AN0857
1026	CONTIG13	223595	224680	1.89	1.86E-02	transcription_start_site	+	222872	222872	1265	2655	AN0857
1026	CONTIG13	223595	224680	1.89	1.86E-02	transcription_start_site	+	222703	222703	1434	2654	AN0857
1026	CONTIG13	223595	224680	1.89	1.86E-02	transcription_start_site	+	222603	222603	1534	2653	AN0857
1322	CONTIG13	220145	220715	1.68	6.84E-02	transcription_start_site	+	222603	222603	-2173	2653	AN0857
1322	CONTIG13	220145	220715	1.68	6.84E-02	transcription_start_site	+	222703	222703	-2273	2654	AN0857
1322	CONTIG13	220145	220715	1.68	6.84E-02	transcription_start_site	+	222872	222872	-2442	2655	AN0857
1322	CONTIG13	220145	220715	1.68	6.84E-02	transcription_start_site	+	223213	223213	-2783	2656	AN0857

1322	CONTIG13	220145	220715	1.68	6.84E-02	transcription_start_site	+	223397	223397	-2967	2657	AN0857
2025	CONTIG13	217805	218459	1.37	1.50E-01	transcription_start_site	+	222603	222603	-4471	2653	AN0857
2025	CONTIG13	217805	218459	1.37	1.50E-01	transcription_start_site	+	222703	222703	-4571	2654	AN0857
2025	CONTIG13	217805	218459	1.37	1.50E-01	transcription_start_site	+	222872	222872	-4740	2655	AN0857
2025	CONTIG13	217805	218459	1.37	1.50E-01	transcription_start_site	+	223213	223213	-5081	2656	AN0857
2025	CONTIG13	217805	218459	1.37	1.50E-01	transcription_start_site	+	223397	223397	-5265	2657	AN0857
74	CONTIG13	231685	232259	3.31	0.00E+00	transcription_start_site	-	229869	229869	-2103	2658	AN0858
74	CONTIG13	231685	232259	3.31	0.00E+00	transcription_start_site	-	232511	232511	539	2659	AN0859
74	CONTIG13	231685	232259	3.31	0.00E+00	transcription_start_site	-	231281	231281	-691	2660	AN0859
74	CONTIG13	231685	232259	3.31	0.00E+00	transcription_start_site	+	233277	233277	-1305	2661	AN0860
74	CONTIG13	231685	232259	3.31	0.00E+00	transcription_start_site	+	233427	233427	-1455	2662	AN0860
74	CONTIG13	231685	232259	3.31	0.00E+00	transcription_start_site	+	233673	233673	-1701	2663	AN0860
74	CONTIG13	231685	232259	3.31	0.00E+00	transcription_start_site	+	234657	234657	-2685	2664	AN0860
323	CONTIG13	243392	243662	2.73	1.80E-03	transcription_start_site	-	240869	240869	-2658	2672	AN0863
323	CONTIG13	243392	243662	2.73	1.80E-03	transcription_start_site	-	240382	240382	-3145	2673	AN0863
323	CONTIG13	243392	243662	2.73	1.80E-03	transcription_start_site	-	240082	240082	-3445	2674	AN0863
323	CONTIG13	243392	243662	2.73	1.80E-03	transcription_start_site	-	239405	239405	-4122	2675	AN0863
567	CONTIG13	244808	245387	2.36	7.85E-03	transcription_start_site	-	240869	240869	-4228	2672	AN0863
567	CONTIG13	244808	245387	2.36	7.85E-03	transcription_start_site	-	240382	240382	-4715	2673	AN0863
567	CONTIG13	244808	245387	2.36	7.85E-03	transcription_start_site	-	240082	240082	-5015	2674	AN0863
567	CONTIG13	244808	245387	2.36	7.85E-03	transcription_start_site	-	245340	245340	242	2681	AN0864
567	CONTIG13	244808	245387	2.36	7.85E-03	transcription_start_site	-	245589	245589	491	2680	AN0864
567	CONTIG13	244808	245387	2.36	7.85E-03	transcription_start_site	-	245683	245683	585	2679	AN0864
567	CONTIG13	244808	245387	2.36	7.85E-03	transcription_start_site	-	246049	246049	951	2678	AN0864
323	CONTIG13	243392	243662	2.73	1.80E-03	transcription_start_site	+	247571	247571	-4044	2682	AN0865
323	CONTIG13	243392	243662	2.73	1.80E-03	transcription_start_site	+	247831	247831	-4304	2683	AN0865
567	CONTIG13	244808	245387	2.36	7.85E-03	transcription_start_site	+	247571	247571	-2473	2682	AN0865
567	CONTIG13	244808	245387	2.36	7.85E-03	transcription_start_site	+	247831	247831	-2733	2683	AN0865
424	CONTIG14	1576	1850	2.56	8.69E-04	transcription_start_site	+	2708	2708	-995	2684	AN0866
424	CONTIG14	1576	1850	2.56	8.69E-04	transcription_start_site	+	3222	3222	-1509	2685	AN0866
424	CONTIG14	1576	1850	2.56	8.69E-04	transcription_start_site	+	4205	4205	-2492	2686	AN0866
424	CONTIG14	1576	1850	2.56	8.69E-04	transcription_start_site	+	4708	4708	-2995	2687	AN0866
695	CONTIG14	2326	2760	2.2	3.61E-03	transcription_start_site	+	2708	2708	-165	2684	AN0866
695	CONTIG14	2326	2760	2.2	3.61E-03	transcription_start_site	+	3222	3222	-679	2685	AN0866
695	CONTIG14	2326	2760	2.2	3.61E-03	transcription_start_site	+	4205	4205	-1662	2686	AN0866
695	CONTIG14	2326	2760	2.2	3.61E-03	transcription_start_site	+	4708	4708	-2165	2687	AN0866
423	CONTIG14	17636	18275	2.56	0.00E+00	transcription_start_site	-	13956	13956	-3999	2694	AN0869
423	CONTIG14	17636	18275	2.56	0.00E+00	transcription_start_site	-	13774	13774	-4181	2695	AN0869
423	CONTIG14	17636	18275	2.56	0.00E+00	transcription_start_site	-	13526	13526	-4429	2696	AN0869
423	CONTIG14	17636	18275	2.56	0.00E+00	transcription_start_site	-	17411	17411	-544	2697	AN0870
423	CONTIG14	17636	18275	2.56	0.00E+00	transcription_start_site	-	17163	17163	-792	2698	AN0870
423	CONTIG14	17636	18275	2.56	0.00E+00	transcription_start_site	-	16686	16686	-1269	2699	AN0870
423	CONTIG14	17636	18275	2.56	0.00E+00	transcription_start_site	-	16339	16339	-1616	2700	AN0870
2333	CONTIG14	21901	22405	1.26	1.25E-01	transcription_start_site	-	17411	17411	-4742	2697	AN0870
2333	CONTIG14	21901	22405	1.26	1.25E-01	transcription_start_site	-	17163	17163	-4990	2698	AN0870
423	CONTIG14	17636	18275	2.56	0.00E+00	transcription_start_site	+	20114	20114	-2158	2701	AN0871
423	CONTIG14	17636	18275	2.56	0.00E+00	transcription_start_site	+	21910	21910	-3954	2702	AN0872
423	CONTIG14	17636	18275	2.56	0.00E+00	transcription_start_site	+	22711	22711	-4755	2703	AN0872
2333	CONTIG14	21901	22405	1.26	1.25E-01	transcription_start_site	+	21910	21910	243	2702	AN0872
2333	CONTIG14	21901	22405	1.26	1.25E-01	transcription_start_site	+	22711	22711	-558	2703	AN0872
2333	CONTIG14	21901	22405	1.26	1.25E-01	transcription_start_site	+	25032	25032	-2879	2704	AN0873
2333	CONTIG14	21901	22405	1.26	1.25E-01	transcription_start_site	+	25913	25913	-3760	2705	AN0873
2333	CONTIG14	21901	22405	1.26	1.25E-01	transcription_start_site	+	26428	26428	-4275	2706	AN0873
2053	CONTIG14	32402	32986	1.35	7.25E-02	transcription_start_site	-	29752	29752	-2942	2707	AN0874
2053	CONTIG14	32402	32986	1.35	7.25E-02	transcription_start_site	-	29537	29537	-3157	2708	AN0874
2053	CONTIG14	32402	32986	1.35	7.25E-02	transcription_start_site	-	29325	29325	-3369	2709	AN0874
2053	CONTIG14	32402	32986	1.35	7.25E-02	transcription_start_site	-	28898	28898	-3796	2710	AN0874

2053	CONTIG14	32402	32986	1.35	7.25E-02	transcription_start_site	-	28636	28636	-4058	2711	AN0874
2053	CONTIG14	32402	32986	1.35	7.25E-02	transcription_start_site	-	27981	27981	-4713	2712	AN0874
2053	CONTIG14	32402	32986	1.35	7.25E-02	transcription_start_site	-	27813	27813	-4881	2713	AN0874
2053	CONTIG14	32402	32986	1.35	7.25E-02	transcription_start_site	-	27558	27558	-5136	2714	AN0874
1457	CONTIG14	34818	35302	1.61	3.70E-02	transcription_start_site	-	31604	31604	-3456	2715	AN0875
1457	CONTIG14	34818	35302	1.61	3.70E-02	transcription_start_site	-	31395	31395	-3665	2716	AN0875
1457	CONTIG14	34818	35302	1.61	3.70E-02	transcription_start_site	-	30819	30819	-4241	2717	AN0875
1457	CONTIG14	34818	35302	1.61	3.70E-02	transcription_start_site	-	30674	30674	-4386	2718	AN0875
1457	CONTIG14	34818	35302	1.61	3.70E-02	transcription_start_site	-	30439	30439	-4621	2719	AN0875
1457	CONTIG14	34818	35302	1.61	3.70E-02	transcription_start_site	-	30349	30349	-4711	2720	AN0875
2053	CONTIG14	32402	32986	1.35	7.25E-02	transcription_start_site	-	31604	31604	-1090	2715	AN0875
2053	CONTIG14	32402	32986	1.35	7.25E-02	transcription_start_site	-	31395	31395	-1299	2716	AN0875
2053	CONTIG14	32402	32986	1.35	7.25E-02	transcription_start_site	-	30819	30819	-1875	2717	AN0875
2053	CONTIG14	32402	32986	1.35	7.25E-02	transcription_start_site	-	30674	30674	-2020	2718	AN0875
2053	CONTIG14	32402	32986	1.35	7.25E-02	transcription_start_site	-	30439	30439	-2255	2719	AN0875
2053	CONTIG14	32402	32986	1.35	7.25E-02	transcription_start_site	-	30349	30349	-2345	2720	AN0875
2053	CONTIG14	32402	32986	1.35	7.25E-02	transcription_start_site	+	32812	32812	-118	2721	AN0876
2053	CONTIG14	32402	32986	1.35	7.25E-02	transcription_start_site	+	32926	32926	-232	2722	AN0876
2486	CONTIG14	48078	48369	1.21	1.44E-01	transcription_start_site	-	48502	48502	278	2730	AN0880
2486	CONTIG14	48078	48369	1.21	1.44E-01	transcription_start_site	-	48725	48725	501	2729	AN0880
2486	CONTIG14	48078	48369	1.21	1.44E-01	transcription_start_site	+	49117	49117	-893	2731	AN0881
187	CONTIG14	58958	59377	2.96	0.00E+00	transcription_start_site	-	55541	55541	-3626	2737	AN0884
187	CONTIG14	58958	59377	2.96	0.00E+00	transcription_start_site	-	55125	55125	-4042	2738	AN0884
187	CONTIG14	58958	59377	2.96	0.00E+00	transcription_start_site	-	58771	58771	-396	2739	AN0885
187	CONTIG14	58958	59377	2.96	0.00E+00	transcription_start_site	-	56190	56190	-2977	2740	AN0885
1212	CONTIG14	61053	61332	1.75	2.12E-02	transcription_start_site	-	58771	58771	-2421	2739	AN0885
1212	CONTIG14	61053	61332	1.75	2.12E-02	transcription_start_site	-	56190	56190	-5002	2740	AN0885
1212	CONTIG14	61053	61332	1.75	2.12E-02	transcription_start_site	-	60954	60954	-238	2741	AN0886
1212	CONTIG14	61053	61332	1.75	2.12E-02	transcription_start_site	-	60813	60813	-379	2742	AN0886
187	CONTIG14	58958	59377	2.96	0.00E+00	transcription_start_site	+	61373	61373	-2205	2743	AN0887
187	CONTIG14	58958	59377	2.96	0.00E+00	transcription_start_site	+	61490	61490	-2322	2744	AN0887
187	CONTIG14	58958	59377	2.96	0.00E+00	transcription_start_site	+	61662	61662	-2494	2745	AN0887
187	CONTIG14	58958	59377	2.96	0.00E+00	transcription_start_site	+	61986	61986	-2818	2746	AN0887
187	CONTIG14	58958	59377	2.96	0.00E+00	transcription_start_site	+	62759	62759	-3591	2747	AN0887
187	CONTIG14	58958	59377	2.96	0.00E+00	transcription_start_site	+	64110	64110	-4942	2748	AN0887
187	CONTIG14	58958	59377	2.96	0.00E+00	transcription_start_site	+	64306	64306	-5138	2749	AN0887
1212	CONTIG14	61053	61332	1.75	2.12E-02	transcription_start_site	+	61373	61373	-180	2743	AN0887
1212	CONTIG14	61053	61332	1.75	2.12E-02	transcription_start_site	+	61490	61490	-297	2744	AN0887
1212	CONTIG14	61053	61332	1.75	2.12E-02	transcription_start_site	+	61662	61662	-469	2745	AN0887
1212	CONTIG14	61053	61332	1.75	2.12E-02	transcription_start_site	+	61986	61986	-793	2746	AN0887
1212	CONTIG14	61053	61332	1.75	2.12E-02	transcription_start_site	+	62759	62759	-1566	2747	AN0887
1212	CONTIG14	61053	61332	1.75	2.12E-02	transcription_start_site	+	64110	64110	-2917	2748	AN0887
1212	CONTIG14	61053	61332	1.75	2.12E-02	transcription_start_site	+	64306	64306	-3113	2749	AN0887
1212	CONTIG14	61053	61332	1.75	2.12E-02	transcription_start_site	+	65215	65215	-4022	2750	AN0887
481	CONTIG14	72690	73119	2.47	1.01E-03	transcription_start_site	-	69320	69320	-3584	2752	AN0889
2054	CONTIG14	76595	77689	1.35	7.51E-02	transcription_start_site	+	79063	79063	-1921	2753	AN0890
2054	CONTIG14	76595	77689	1.35	7.51E-02	transcription_start_site	+	79307	79307	-2165	2754	AN0890
2054	CONTIG14	76595	77689	1.35	7.51E-02	transcription_start_site	+	80302	80302	-3160	2755	AN0890
1213	CONTIG14	96007	96285	1.75	2.12E-02	transcription_start_site	-	95146	95146	-1000	2765	AN0894
1213	CONTIG14	96007	96285	1.75	2.12E-02	transcription_start_site	-	94719	94719	-1427	2766	AN0894
1213	CONTIG14	96007	96285	1.75	2.12E-02	transcription_start_site	-	93884	93884	-2262	2767	AN0894
1828	CONTIG14	99007	99291	1.44	6.84E-02	transcription_start_site	-	95146	95146	-4003	2765	AN0894
1828	CONTIG14	99007	99291	1.44	6.84E-02	transcription_start_site	-	94719	94719	-4430	2766	AN0894
1213	CONTIG14	96007	96285	1.75	2.12E-02	transcription_start_site	+	96281	96281	-135	2768	AN0895
1213	CONTIG14	96007	96285	1.75	2.12E-02	transcription_start_site	+	97761	97761	-1615	2769	AN0896
1213	CONTIG14	96007	96285	1.75	2.12E-02	transcription_start_site	+	97999	97999	-1853	2770	AN0896
1213	CONTIG14	96007	96285	1.75	2.12E-02	transcription_start_site	+	98099	98099	-1953	2771	AN0896

1213	CONTIG14	96007	96285	1.75	2.12E-02	transcription_start_site	+	98223	98223	-2077	2772	AN0896
1213	CONTIG14	96007	96285	1.75	2.12E-02	transcription_start_site	+	98396	98396	-2250	2773	AN0896
1828	CONTIG14	99007	99291	1.44	6.84E-02	transcription_start_site	+	98396	98396	753	2773	AN0896
1828	CONTIG14	99007	99291	1.44	6.84E-02	transcription_start_site	+	98223	98223	926	2772	AN0896
1828	CONTIG14	99007	99291	1.44	6.84E-02	transcription_start_site	+	98099	98099	1050	2771	AN0896
1828	CONTIG14	99007	99291	1.44	6.84E-02	transcription_start_site	-	99744	99744	595	2774	AN0897
1828	CONTIG14	99007	99291	1.44	6.84E-02	transcription_start_site	+	101725	101725	-2576	2775	AN0898
1828	CONTIG14	99007	99291	1.44	6.84E-02	transcription_start_site	+	101902	101902	-2753	2776	AN0898
1828	CONTIG14	99007	99291	1.44	6.84E-02	transcription_start_site	+	102717	102717	-3568	2777	AN0898
1458	CONTIG14	112883	113527	1.61	3.70E-02	transcription_start_site	+	113272	113272	-67	2789	AN0902
1458	CONTIG14	112883	113527	1.61	3.70E-02	transcription_start_site	+	114132	114132	-927	2790	AN0902
1458	CONTIG14	112883	113527	1.61	3.70E-02	transcription_start_site	+	114323	114323	-1118	2791	AN0902
1458	CONTIG14	112883	113527	1.61	3.70E-02	transcription_start_site	+	115983	115983	-2778	2792	AN0903
1750	CONTIG14	116183	116452	1.48	5.88E-02	transcription_start_site	+	115983	115983	334	2792	AN0903
1750	CONTIG14	116183	116452	1.48	5.88E-02	transcription_start_site	+	118559	118559	-2241	2793	AN0904
1750	CONTIG14	116183	116452	1.48	5.88E-02	transcription_start_site	+	118642	118642	-2324	2794	AN0904
1750	CONTIG14	116183	116452	1.48	5.88E-02	transcription_start_site	+	118904	118904	-2586	2795	AN0904
1750	CONTIG14	116183	116452	1.48	5.88E-02	transcription_start_site	+	120987	120987	-4669	2796	AN0905
1750	CONTIG14	116183	116452	1.48	5.88E-02	transcription_start_site	+	121309	121309	-4991	2797	AN0905
2058	CONTIG14	171234	171513	1.35	9.24E-02	transcription_start_site	-	166923	166923	-4450	2848	AN0922
2058	CONTIG14	171234	171513	1.35	9.24E-02	transcription_start_site	-	166860	166860	-4513	2849	AN0922
2058	CONTIG14	171234	171513	1.35	9.24E-02	transcription_start_site	-	166367	166367	-5006	2850	AN0922
2058	CONTIG14	171234	171513	1.35	9.24E-02	transcription_start_site	+	171753	171753	-379	2853	AN0924
2058	CONTIG14	171234	171513	1.35	9.24E-02	transcription_start_site	+	171935	171935	-561	2854	AN0924
2058	CONTIG14	171234	171513	1.35	9.24E-02	transcription_start_site	+	172050	172050	-676	2855	AN0924
2058	CONTIG14	171234	171513	1.35	9.24E-02	transcription_start_site	+	172350	172350	-976	2856	AN0924
2058	CONTIG14	171234	171513	1.35	9.24E-02	transcription_start_site	+	172573	172573	-1199	2857	AN0924
2058	CONTIG14	171234	171513	1.35	9.24E-02	transcription_start_site	+	172910	172910	-1536	2858	AN0924
2058	CONTIG14	171234	171513	1.35	9.24E-02	transcription_start_site	+	174246	174246	-2872	2859	AN0925
2058	CONTIG14	171234	171513	1.35	9.24E-02	transcription_start_site	+	174528	174528	-3154	2860	AN0925
2058	CONTIG14	171234	171513	1.35	9.24E-02	transcription_start_site	+	175340	175340	-3966	2861	AN0925
36	CONTIG14	187142	187491	3.5	0.00E+00	transcription_start_site	-	183035	183035	-4281	2865	AN0927
36	CONTIG14	187142	187491	3.5	0.00E+00	transcription_start_site	-	182889	182889	-4427	2866	AN0927
36	CONTIG14	187142	187491	3.5	0.00E+00	transcription_start_site	-	182743	182743	-4573	2867	AN0927
36	CONTIG14	187142	187491	3.5	0.00E+00	transcription_start_site	-	182590	182590	-4726	2868	AN0927
371	CONTIG14	183167	183743	2.65	0.00E+00	transcription_start_site	-	183035	183035	-420	2865	AN0927
371	CONTIG14	183167	183743	2.65	0.00E+00	transcription_start_site	-	182889	182889	-566	2866	AN0927
371	CONTIG14	183167	183743	2.65	0.00E+00	transcription_start_site	-	182743	182743	-712	2867	AN0927
371	CONTIG14	183167	183743	2.65	0.00E+00	transcription_start_site	-	182590	182590	-865	2868	AN0927
1526	CONTIG14	184287	184631	1.57	4.36E-02	transcription_start_site	-	183035	183035	-1424	2865	AN0927
1526	CONTIG14	184287	184631	1.57	4.36E-02	transcription_start_site	-	182889	182889	-1570	2866	AN0927
1526	CONTIG14	184287	184631	1.57	4.36E-02	transcription_start_site	-	182743	182743	-1716	2867	AN0927
1526	CONTIG14	184287	184631	1.57	4.36E-02	transcription_start_site	-	182590	182590	-1869	2868	AN0927
36	CONTIG14	187142	187491	3.5	0.00E+00	transcription_start_site	-	185947	185947	-1369	2869	AN0928
36	CONTIG14	187142	187491	3.5	0.00E+00	transcription_start_site	-	185788	185788	-1528	2870	AN0928
36	CONTIG14	187142	187491	3.5	0.00E+00	transcription_start_site	-	185436	185436	-1880	2871	AN0928
1526	CONTIG14	184287	184631	1.57	4.36E-02	transcription_start_site	-	185436	185436	977	2871	AN0928
645	CONTIG14	199506	200450	2.24	0.00E+00	transcription_start_site	-	195323	195323	-4655	2872	AN0929
645	CONTIG14	199506	200450	2.24	0.00E+00	transcription_start_site	-	195048	195048	-4930	2873	AN0929
287	CONTIG14	200646	200983	2.78	2.67E-04	transcription_start_site	-	198848	198848	-1966	2877	AN0930
287	CONTIG14	200646	200983	2.78	2.67E-04	transcription_start_site	-	198497	198497	-2317	2878	AN0930
287	CONTIG14	200646	200983	2.78	2.67E-04	transcription_start_site	-	198273	198273	-2541	2879	AN0930
287	CONTIG14	200646	200983	2.78	2.67E-04	transcription_start_site	-	196988	196988	-3826	2880	AN0930
645	CONTIG14	199506	200450	2.24	0.00E+00	transcription_start_site	-	198848	198848	-1130	2877	AN0930
645	CONTIG14	199506	200450	2.24	0.00E+00	transcription_start_site	-	198497	198497	-1481	2878	AN0930
645	CONTIG14	199506	200450	2.24	0.00E+00	transcription_start_site	-	198273	198273	-1705	2879	AN0930
645	CONTIG14	199506	200450	2.24	0.00E+00	transcription_start_site	-	196988	196988	-2990	2880	AN0930

287	CONTIG14	200646	200983	2.78	2.67E-04	transcription_start_site	+	201478	201478	-663	2881 AN0931
287	CONTIG14	200646	200983	2.78	2.67E-04	transcription_start_site	+	203074	203074	-2259	2882 AN0931
645	CONTIG14	199506	200450	2.24	0.00E+00	transcription_start_site	+	201478	201478	-1500	2881 AN0931
645	CONTIG14	199506	200450	2.24	0.00E+00	transcription_start_site	+	203074	203074	-3096	2882 AN0931
287	CONTIG14	200646	200983	2.78	2.67E-04	transcription_start_site	+	204241	204241	-3426	2883 AN0932
287	CONTIG14	200646	200983	2.78	2.67E-04	transcription_start_site	+	204704	204704	-3889	2884 AN0932
287	CONTIG14	200646	200983	2.78	2.67E-04	transcription_start_site	+	205601	205601	-4786	2885 AN0932
645	CONTIG14	199506	200450	2.24	0.00E+00	transcription_start_site	+	204241	204241	-4263	2883 AN0932
645	CONTIG14	199506	200450	2.24	0.00E+00	transcription_start_site	+	204704	204704	-4726	2884 AN0932
188	CONTIG14	208282	208701	2.96	0.00E+00	transcription_start_site	+	209299	209299	-807	2886 AN0933
188	CONTIG14	208282	208701	2.96	0.00E+00	transcription_start_site	+	209447	209447	-955	2887 AN0933
188	CONTIG14	208282	208701	2.96	0.00E+00	transcription_start_site	+	209846	209846	-1354	2888 AN0933
2195	CONTIG14	206712	206991	1.3	1.08E-01	transcription_start_site	+	209299	209299	-2447	2886 AN0933
2195	CONTIG14	206712	206991	1.3	1.08E-01	transcription_start_site	+	209447	209447	-2595	2887 AN0933
2195	CONTIG14	206712	206991	1.3	1.08E-01	transcription_start_site	+	209846	209846	-2994	2888 AN0933
2591	CONTIG14	210092	210591	1.17	1.66E-01	transcription_start_site	+	209846	209846	495	2888 AN0933
2591	CONTIG14	210092	210591	1.17	1.66E-01	transcription_start_site	+	209447	209447	894	2887 AN0933
2591	CONTIG14	210092	210591	1.17	1.66E-01	transcription_start_site	+	209299	209299	1042	2886 AN0933
2591	CONTIG14	210092	210591	1.17	1.66E-01	transcription_start_site	+	215529	215529	-5187	2894 AN0936
1355	CONTIG14	245852	246206	1.66	3.08E-02	transcription_start_site	-	246682	246682	653	2924 AN0947
935	CONTIG14	255003	255357	1.97	8.81E-03	transcription_start_site	-	252327	252327	-2853	2925 AN0948
935	CONTIG14	255003	255357	1.97	8.81E-03	transcription_start_site	-	252208	252208	-2972	2926 AN0948
935	CONTIG14	255003	255357	1.97	8.81E-03	transcription_start_site	-	251967	251967	-3213	2927 AN0948
935	CONTIG14	255003	255357	1.97	8.81E-03	transcription_start_site	-	251709	251709	-3471	2928 AN0948
935	CONTIG14	255003	255357	1.97	8.81E-03	transcription_start_site	-	251468	251468	-3712	2929 AN0948
935	CONTIG14	255003	255357	1.97	8.81E-03	transcription_start_site	-	251317	251317	-3863	2930 AN0948
935	CONTIG14	255003	255357	1.97	8.81E-03	transcription_start_site	-	250168	250168	-5012	2931 AN0948
935	CONTIG14	255003	255357	1.97	8.81E-03	transcription_start_site	+	255330	255330	-150	2933 AN0950
935	CONTIG14	255003	255357	1.97	8.81E-03	transcription_start_site	+	255733	255733	-553	2934 AN0950
973	CONTIG14	261244	261878	1.93	4.81E-03	transcription_start_site	+	263249	263249	-1688	2935 AN0951
973	CONTIG14	261244	261878	1.93	4.81E-03	transcription_start_site	+	264320	264320	-2759	2936 AN0951
973	CONTIG14	261244	261878	1.93	4.81E-03	transcription_start_site	+	266005	266005	-4444	2937 AN0951
1214	CONTIG14	262354	262943	1.75	2.12E-02	transcription_start_site	+	263249	263249	-600	2935 AN0951
1214	CONTIG14	262354	262943	1.75	2.12E-02	transcription_start_site	+	264320	264320	-1671	2936 AN0951
1214	CONTIG14	262354	262943	1.75	2.12E-02	transcription_start_site	+	266005	266005	-3356	2937 AN0951
1449	CONTIG14	312016	312745	1.61	2.18E-02	transcription_start_site	-	309781	309781	-2599	2978 AN0973
1449	CONTIG14	312016	312745	1.61	2.18E-02	transcription_start_site	-	310920	310920	-1460	2979 AN0974
1639	CONTIG14	325505	325944	1.52	5.22E-02	transcription_start_site	-	322049	322049	-3675	2983 AN0976
1639	CONTIG14	325505	325944	1.52	5.22E-02	transcription_start_site	-	325686	325686	-38	2984 AN0977
1639	CONTIG14	325505	325944	1.52	5.22E-02	transcription_start_site	-	324960	324960	-764	2985 AN0977
1639	CONTIG14	325505	325944	1.52	5.22E-02	transcription_start_site	+	326261	326261	-536	2986 AN0978
1639	CONTIG14	325505	325944	1.52	5.22E-02	transcription_start_site	+	326497	326497	-772	2987 AN0978
372	CONTIG14	330835	331484	2.65	0.00E+00	transcription_start_site	+	333510	333510	-2350	2988 AN0979
372	CONTIG14	330835	331484	2.65	0.00E+00	transcription_start_site	+	334531	334531	-3371	2989 AN0979
372	CONTIG14	330835	331484	2.65	0.00E+00	transcription_start_site	+	335423	335423	-4263	2990 AN0979
372	CONTIG14	330835	331484	2.65	0.00E+00	transcription_start_site	+	335837	335837	-4677	2991 AN0979
1450	CONTIG14	332115	332914	1.61	2.18E-02	transcription_start_site	+	333510	333510	-995	2988 AN0979
1450	CONTIG14	332115	332914	1.61	2.18E-02	transcription_start_site	+	334531	334531	-2016	2989 AN0979
1450	CONTIG14	332115	332914	1.61	2.18E-02	transcription_start_site	+	335423	335423	-2908	2990 AN0979
1450	CONTIG14	332115	332914	1.61	2.18E-02	transcription_start_site	+	335837	335837	-3322	2991 AN0979
616	CONTIG14	344115	344397	2.29	2.22E-03	transcription_start_site	-	340928	340928	-3328	2992 AN0980
616	CONTIG14	344115	344397	2.29	2.22E-03	transcription_start_site	-	339995	339995	-4261	2993 AN0980
616	CONTIG14	344115	344397	2.29	2.22E-03	transcription_start_site	-	343707	343707	-549	2999 AN0981
616	CONTIG14	344115	344397	2.29	2.22E-03	transcription_start_site	-	342865	342865	-1391	3000 AN0981
2487	CONTIG14	347790	348127	1.21	1.44E-01	transcription_start_site	-	343707	343707	-4251	2999 AN0981
2487	CONTIG14	347790	348127	1.21	1.44E-01	transcription_start_site	-	342865	342865	-5093	3000 AN0981
2487	CONTIG14	347790	348127	1.21	1.44E-01	transcription_start_site	-	345884	345884	-2074	3001 AN0982

2487	CONTIG14	347790	348127	1.21	1.44E-01	transcription_start_site	-	345731	345731	-2227	3002 AN0982
616	CONTIG14	344115	344397	2.29	2.22E-03	transcription_start_site	+	347524	347524	-3268	3003 AN0983
2487	CONTIG14	347790	348127	1.21	1.44E-01	transcription_start_site	+	347524	347524	434	3003 AN0983
2184	CONTIG14	354833	355642	1.3	8.36E-02	transcription_start_site	-	350997	350997	-4240	3004 AN0984
2184	CONTIG14	354833	355642	1.3	8.36E-02	transcription_start_site	-	350736	350736	-4501	3005 AN0984
2184	CONTIG14	354833	355642	1.3	8.36E-02	transcription_start_site	-	353039	353039	-2198	3006 AN0985
2184	CONTIG14	354833	355642	1.3	8.36E-02	transcription_start_site	-	352518	352518	-2719	3007 AN0985
2184	CONTIG14	354833	355642	1.3	8.36E-02	transcription_start_site	-	352345	352345	-2892	3008 AN0985
2184	CONTIG14	354833	355642	1.3	8.36E-02	transcription_start_site	-	351835	351835	-3402	3009 AN0985
2184	CONTIG14	354833	355642	1.3	8.36E-02	transcription_start_site	+	355879	355879	-641	3010 AN0986
2184	CONTIG14	354833	355642	1.3	8.36E-02	transcription_start_site	+	356090	356090	-852	3011 AN0986
2184	CONTIG14	354833	355642	1.3	8.36E-02	transcription_start_site	+	358172	358172	-2934	3012 AN0986
2184	CONTIG14	354833	355642	1.3	8.36E-02	transcription_start_site	+	359006	359006	-3768	3013 AN0987
2196	CONTIG14	362263	362606	1.3	1.08E-01	transcription_start_site	-	362673	362673	238	3014 AN0988
2196	CONTIG14	362263	362606	1.3	1.08E-01	transcription_start_site	-	361802	361802	-632	3015 AN0988
2196	CONTIG14	362263	362606	1.3	1.08E-01	transcription_start_site	-	361670	361670	-764	3016 AN0988
2196	CONTIG14	362263	362606	1.3	1.08E-01	transcription_start_site	-	361272	361272	-1162	3017 AN0988
2196	CONTIG14	362263	362606	1.3	1.08E-01	transcription_start_site	-	360764	360764	-1670	3018 AN0988
2196	CONTIG14	362263	362606	1.3	1.08E-01	transcription_start_site	-	360624	360624	-1810	3019 AN0988
2352	CONTIG15	3471	4028	1.25	8.01E-02	transcription_start_site	-	3956	3956	206	3020 AN0989
2352	CONTIG15	3471	4028	1.25	8.01E-02	transcription_start_site	-	2834	2834	-915	3021 AN0989
2352	CONTIG15	3471	4028	1.25	8.01E-02	transcription_start_site	+	4609	4609	-859	3022 AN0990
2352	CONTIG15	3471	4028	1.25	8.01E-02	transcription_start_site	+	4811	4811	-1061	3023 AN0990
2352	CONTIG15	3471	4028	1.25	8.01E-02	transcription_start_site	+	5210	5210	-1460	3024 AN0990
2352	CONTIG15	3471	4028	1.25	8.01E-02	transcription_start_site	+	6409	6409	-2659	3025 AN0990
2352	CONTIG15	3471	4028	1.25	8.01E-02	transcription_start_site	+	7139	7139	-3389	3026 AN0990
2473	CONTIG15	9546	9820	1.21	9.24E-02	transcription_start_site	-	9483	9483	-200	3027 AN0991
2473	CONTIG15	9546	9820	1.21	9.24E-02	transcription_start_site	-	9123	9123	-560	3028 AN0991
2473	CONTIG15	9546	9820	1.21	9.24E-02	transcription_start_site	-	8702	8702	-981	3029 AN0991
2473	CONTIG15	9546	9820	1.21	9.24E-02	transcription_start_site	-	8173	8173	-1510	3030 AN0991
2473	CONTIG15	9546	9820	1.21	9.24E-02	transcription_start_site	+	10522	10522	-839	3031 AN0992
2473	CONTIG15	9546	9820	1.21	9.24E-02	transcription_start_site	+	10995	10995	-1312	3032 AN0992
2473	CONTIG15	9546	9820	1.21	9.24E-02	transcription_start_site	+	12213	12213	-2530	3033 AN0993
2473	CONTIG15	9546	9820	1.21	9.24E-02	transcription_start_site	+	12469	12469	-2786	3034 AN0993
2473	CONTIG15	9546	9820	1.21	9.24E-02	transcription_start_site	+	12569	12569	-2886	3035 AN0993
2473	CONTIG15	9546	9820	1.21	9.24E-02	transcription_start_site	+	12816	12816	-3133	3036 AN0993
2473	CONTIG15	9546	9820	1.21	9.24E-02	transcription_start_site	+	13016	13016	-3333	3037 AN0993
2473	CONTIG15	9546	9820	1.21	9.24E-02	transcription_start_site	+	13129	13129	-3446	3038 AN0993
2473	CONTIG15	9546	9820	1.21	9.24E-02	transcription_start_site	+	13439	13439	-3756	3039 AN0993
1396	CONTIG3	80721	81065	1.63	1.57E-02	transcription_start_site	-	76110	76110	-4783	3090 AN10013
405	CONTIG3	20038	20617	2.59	0.00E+00	transcription_start_site	+	21825	21825	-1497	3091 AN10014
656	CONTIG3	18908	19257	2.23	9.51E-04	transcription_start_site	+	21825	21825	-2742	3091 AN10014
1396	CONTIG3	80721	81065	1.63	1.57E-02	transcription_start_site	+	82782	82782	-1889	3092 AN10015
2560	CONTIG4	61964	62233	1.18	9.24E-02	transcription_start_site	-	57523	57523	-4575	3096 AN10017
2560	CONTIG4	61964	62233	1.18	9.24E-02	transcription_start_site	-	57355	57355	-4743	3097 AN10017
2560	CONTIG4	61964	62233	1.18	9.24E-02	transcription_start_site	-	57177	57177	-4921	3098 AN10017
1303	CONTIG4	63079	63353	1.69	1.11E-02	transcription_start_site	-	59182	59182	-4034	3104 AN10018
1303	CONTIG4	63079	63353	1.69	1.11E-02	transcription_start_site	-	59052	59052	-4164	3105 AN10018
2560	CONTIG4	61964	62233	1.18	9.24E-02	transcription_start_site	-	59182	59182	-2916	3104 AN10018
2560	CONTIG4	61964	62233	1.18	9.24E-02	transcription_start_site	-	59052	59052	-3046	3105 AN10018
1303	CONTIG4	63079	63353	1.69	1.11E-02	transcription_start_site	-	62720	62720	-496	3106 AN10019
1303	CONTIG4	63079	63353	1.69	1.11E-02	transcription_start_site	-	62585	62585	-631	3107 AN10019
1303	CONTIG4	63079	63353	1.69	1.11E-02	transcription_start_site	-	62225	62225	-991	3108 AN10019
1303	CONTIG4	63079	63353	1.69	1.11E-02	transcription_start_site	-	60800	60800	-2416	3109 AN10019
2560	CONTIG4	61964	62233	1.18	9.24E-02	transcription_start_site	-	62225	62225	126	3108 AN10019
2560	CONTIG4	61964	62233	1.18	9.24E-02	transcription_start_site	-	62585	62585	486	3107 AN10019
2560	CONTIG4	61964	62233	1.18	9.24E-02	transcription_start_site	-	62720	62720	621	3106 AN10019

2560	CONTIG4	61964	62233	1.18	9.24E-02	transcription_start_site	-	60800	60800	-1298	3109	AN10019
3011	CONTIG4	64444	65903	0.9	1.58E-01	transcription_start_site	-	62720	62720	-2453	3106	AN10019
3011	CONTIG4	64444	65903	0.9	1.58E-01	transcription_start_site	-	62585	62585	-2588	3107	AN10019
3011	CONTIG4	64444	65903	0.9	1.58E-01	transcription_start_site	-	62225	62225	-2948	3108	AN10019
3011	CONTIG4	64444	65903	0.9	1.58E-01	transcription_start_site	-	60800	60800	-4373	3109	AN10019
1997	CONTIG15	39242	39506	1.37	5.22E-02	transcription_start_site	-	39152	39152	-222	3110	AN1002
1997	CONTIG15	39242	39506	1.37	5.22E-02	transcription_start_site	-	36733	36733	-2641	3111	AN1002
501	CONTIG5	3471	3725	2.45	8.81E-03	transcription_start_site	-	3747	3747	149	3118	AN10021
501	CONTIG5	3471	3725	2.45	8.81E-03	transcription_start_site	-	4570	4570	972	3117	AN10021
1816	CONTIG5	217821	218095	1.45	1.66E-01	transcription_start_site	-	217284	217284	-674	3137	AN10028
1816	CONTIG5	217821	218095	1.45	1.66E-01	transcription_start_site	-	217145	217145	-813	3138	AN10028
1816	CONTIG5	217821	218095	1.45	1.66E-01	transcription_start_site	-	216954	216954	-1004	3139	AN10028
1816	CONTIG5	217821	218095	1.45	1.66E-01	transcription_start_site	-	215840	215840	-2118	3140	AN10028
1816	CONTIG5	217821	218095	1.45	1.66E-01	transcription_start_site	-	215656	215656	-2302	3141	AN10028
1817	CONTIG5	218711	219070	1.45	1.66E-01	transcription_start_site	-	217284	217284	-1606	3137	AN10028
1817	CONTIG5	218711	219070	1.45	1.66E-01	transcription_start_site	-	217145	217145	-1745	3138	AN10028
1817	CONTIG5	218711	219070	1.45	1.66E-01	transcription_start_site	-	216954	216954	-1936	3139	AN10028
1817	CONTIG5	218711	219070	1.45	1.66E-01	transcription_start_site	-	215840	215840	-3050	3140	AN10028
1817	CONTIG5	218711	219070	1.45	1.66E-01	transcription_start_site	-	215656	215656	-3234	3141	AN10028
1997	CONTIG15	39242	39506	1.37	5.22E-02	transcription_start_site	+	39578	39578	-204	3145	AN1003
1997	CONTIG15	39242	39506	1.37	5.22E-02	transcription_start_site	+	39733	39733	-359	3146	AN1003
1997	CONTIG15	39242	39506	1.37	5.22E-02	transcription_start_site	+	39956	39956	-582	3147	AN1003
1997	CONTIG15	39242	39506	1.37	5.22E-02	transcription_start_site	+	40101	40101	-727	3148	AN1003
1997	CONTIG15	39242	39506	1.37	5.22E-02	transcription_start_site	+	40273	40273	-899	3149	AN1003
1997	CONTIG15	39242	39506	1.37	5.22E-02	transcription_start_site	+	41049	41049	-1675	3150	AN1003
100	CONTIG5	275713	276064	3.23	5.64E-04	transcription_start_site	-	276362	276362	473	3153	AN10030
734	CONTIG5	276678	277107	2.17	1.06E-02	transcription_start_site	-	277336	277336	443	3152	AN10030
734	CONTIG5	276678	277107	2.17	1.06E-02	transcription_start_site	-	276362	276362	-530	3153	AN10030
734	CONTIG5	276678	277107	2.17	1.06E-02	transcription_start_site	-	277772	277772	879	3151	AN10030
1180	CONTIG5	277963	278324	1.78	6.84E-02	transcription_start_site	-	277772	277772	-371	3151	AN10030
1180	CONTIG5	277963	278324	1.78	6.84E-02	transcription_start_site	-	277336	277336	-807	3152	AN10030
1180	CONTIG5	277963	278324	1.78	6.84E-02	transcription_start_site	-	276362	276362	-1781	3153	AN10030
739	CONTIG5	372977	373331	2.17	2.12E-02	transcription_start_site	-	369979	369979	-3175	3154	AN10031
13	CONTIG5	397516	398017	3.67	0.00E+00	transcription_start_site	-	392771	392771	-4995	3155	AN10032
13	CONTIG5	397516	398017	3.67	0.00E+00	transcription_start_site	-	392569	392569	-5197	3156	AN10032
843	CONTIG5	95479	96368	2.06	1.81E-02	transcription_start_site	+	94594	94594	1329	3164	AN10033
501	CONTIG5	3471	3725	2.45	8.81E-03	transcription_start_site	-	2407	2407	-1191	3176	AN10039
100	CONTIG5	275713	276064	3.23	5.64E-04	transcription_start_site	-	275377	275377	-511	3180	AN10040
734	CONTIG5	276678	277107	2.17	1.06E-02	transcription_start_site	-	275377	275377	-1515	3180	AN10040
1180	CONTIG5	277963	278324	1.78	6.84E-02	transcription_start_site	-	275377	275377	-2766	3180	AN10040
1816	CONTIG5	217821	218095	1.45	1.66E-01	transcription_start_site	-	218047	218047	89	3183	AN10041
1816	CONTIG5	217821	218095	1.45	1.66E-01	transcription_start_site	-	218378	218378	420	3182	AN10041
1816	CONTIG5	217821	218095	1.45	1.66E-01	transcription_start_site	-	218843	218843	885	3181	AN10041
1817	CONTIG5	218711	219070	1.45	1.66E-01	transcription_start_site	-	218843	218843	-47	3181	AN10041
1817	CONTIG5	218711	219070	1.45	1.66E-01	transcription_start_site	-	218378	218378	-512	3182	AN10041
1817	CONTIG5	218711	219070	1.45	1.66E-01	transcription_start_site	-	218047	218047	-843	3183	AN10041
548	CONTIG5	245490	245776	2.39	1.11E-02	transcription_start_site	+	247523	247523	-1890	3192	AN10045
739	CONTIG5	372977	373331	2.17	2.12E-02	transcription_start_site	-	368821	368821	-4333	3193	AN10046
1997	CONTIG15	39242	39506	1.37	5.22E-02	transcription_start_site	+	42684	42684	-3310	3200	AN1005
1997	CONTIG15	39242	39506	1.37	5.22E-02	transcription_start_site	+	42857	42857	-3483	3201	AN1005
29	CONTIG5	424967	425601	3.56	0.00E+00	transcription_start_site	-	424938	424938	-346	3202	AN10050
29	CONTIG5	424967	425601	3.56	0.00E+00	transcription_start_site	-	424773	424773	-511	3203	AN10050
245	CONTIG5	426090	426814	2.84	0.00E+00	transcription_start_site	-	424938	424938	-1514	3202	AN10050
245	CONTIG5	426090	426814	2.84	0.00E+00	transcription_start_site	-	424773	424773	-1679	3203	AN10050
1343	CONTIG5	428480	428906	1.67	9.24E-02	transcription_start_site	-	424938	424938	-3755	3202	AN10050
1343	CONTIG5	428480	428906	1.67	9.24E-02	transcription_start_site	-	424773	424773	-3920	3203	AN10050
29	CONTIG5	424967	425601	3.56	0.00E+00	transcription_start_site	-	425666	425666	382	3204	AN10051

245	CONTIG5	426090	426814	2.84	0.00E+00	transcription_start_site	-	425666	425666	-786	3204 AN10051
1343	CONTIG5	428480	428906	1.67	9.24E-02	transcription_start_site	-	425666	425666	-3027	3204 AN10051
176	CONTIG6	87761	88408	2.99	0.00E+00	transcription_start_site	-	87170	87170	-914	3208 AN10053
176	CONTIG6	87761	88408	2.99	0.00E+00	transcription_start_site	-	86552	86552	-1532	3209 AN10053
1810	CONTIG6	198021	198275	1.45	5.22E-02	transcription_start_site	+	202565	202565	-4417	3222 AN10057
1810	CONTIG6	198021	198275	1.45	5.22E-02	transcription_start_site	+	203086	203086	-4938	3223 AN10057
987	CONTIG6	208818	209157	1.92	7.85E-03	transcription_start_site	+	212242	212242	-3254	3225 AN10058
987	CONTIG6	208818	209157	1.92	7.85E-03	transcription_start_site	+	212529	212529	-3541	3226 AN10058
987	CONTIG6	208818	209157	1.92	7.85E-03	transcription_start_site	+	213676	213676	-4688	3227 AN10058
2377	CONTIG6	210528	211332	1.24	1.08E-01	transcription_start_site	+	212242	212242	-1312	3225 AN10058
2377	CONTIG6	210528	211332	1.24	1.08E-01	transcription_start_site	+	212529	212529	-1599	3226 AN10058
2377	CONTIG6	210528	211332	1.24	1.08E-01	transcription_start_site	+	213676	213676	-2746	3227 AN10058
2531	CONTIG6	244884	245228	1.19	1.25E-01	transcription_start_site	+	246337	246337	-1281	3228 AN10059
2531	CONTIG6	244884	245228	1.19	1.25E-01	transcription_start_site	+	247520	247520	-2464	3229 AN10059
2531	CONTIG6	244884	245228	1.19	1.25E-01	transcription_start_site	+	248526	248526	-3470	3230 AN10059
2799	CONTIG6	175746	176170	1.07	1.93E-01	transcription_start_site	+	176173	176173	-215	3238 AN10060
2799	CONTIG6	175746	176170	1.07	1.93E-01	transcription_start_site	+	176440	176440	-482	3239 AN10060
2799	CONTIG6	175746	176170	1.07	1.93E-01	transcription_start_site	+	180841	180841	-4883	3240 AN10060
1810	CONTIG6	198021	198275	1.45	5.22E-02	transcription_start_site	+	199263	199263	-1115	3241 AN10061
1810	CONTIG6	198021	198275	1.45	5.22E-02	transcription_start_site	+	199427	199427	-1279	3242 AN10061
1810	CONTIG6	198021	198275	1.45	5.22E-02	transcription_start_site	+	201734	201734	-3586	3243 AN10061
2730	CONTIG6	194946	195357	1.11	1.50E-01	transcription_start_site	+	199263	199263	-4111	3241 AN10061
2730	CONTIG6	194946	195357	1.11	1.50E-01	transcription_start_site	+	199427	199427	-4275	3242 AN10061
987	CONTIG6	208818	209157	1.92	7.85E-03	transcription_start_site	+	209144	209144	-156	3258 AN10068
987	CONTIG6	208818	209157	1.92	7.85E-03	transcription_start_site	+	209639	209639	-651	3259 AN10068
987	CONTIG6	208818	209157	1.92	7.85E-03	transcription_start_site	+	209938	209938	-950	3260 AN10068
2377	CONTIG6	210528	211332	1.24	1.08E-01	transcription_start_site	+	209938	209938	992	3260 AN10068
2377	CONTIG6	210528	211332	1.24	1.08E-01	transcription_start_site	+	209639	209639	1291	3259 AN10068
2531	CONTIG6	244884	245228	1.19	1.25E-01	transcription_start_site	+	249053	249053	-3997	3261 AN10069
2531	CONTIG6	244884	245228	1.19	1.25E-01	transcription_start_site	+	249560	249560	-4504	3262 AN10069
176	CONTIG6	87761	88408	2.99	0.00E+00	transcription_start_site	-	85532	85532	-2552	3273 AN10071
176	CONTIG6	87761	88408	2.99	0.00E+00	transcription_start_site	-	84609	84609	-3475	3274 AN10071
812	CONTIG7	145354	145628	2.09	4.61E-03	transcription_start_site	-	145192	145192	-299	3294 AN10077
812	CONTIG7	145354	145628	2.09	4.61E-03	transcription_start_site	-	145012	145012	-479	3295 AN10077
812	CONTIG7	145354	145628	2.09	4.61E-03	transcription_start_site	-	143846	143846	-1645	3296 AN10077
812	CONTIG7	145354	145628	2.09	4.61E-03	transcription_start_site	-	143637	143637	-1854	3297 AN10077
812	CONTIG7	145354	145628	2.09	4.61E-03	transcription_start_site	-	143473	143473	-2018	3298 AN10077
812	CONTIG7	145354	145628	2.09	4.61E-03	transcription_start_site	+	146985	146985	-1494	3299 AN10078
632	CONTIG7	166821	167236	2.26	1.80E-03	transcription_start_site	-	164667	164667	-2361	3300 AN10079
632	CONTIG7	166821	167236	2.26	1.80E-03	transcription_start_site	-	164555	164555	-2473	3301 AN10079
632	CONTIG7	166821	167236	2.26	1.80E-03	transcription_start_site	-	164380	164380	-2648	3302 AN10079
632	CONTIG7	166821	167236	2.26	1.80E-03	transcription_start_site	-	164302	164302	-2726	3303 AN10079
632	CONTIG7	166821	167236	2.26	1.80E-03	transcription_start_site	-	162180	162180	-4848	3304 AN10079
632	CONTIG7	166821	167236	2.26	1.80E-03	transcription_start_site	-	162063	162063	-4965	3305 AN10079
1442	CONTIG7	228546	230375	1.61	0.00E+00	transcription_start_site	+	232619	232619	-3158	3310 AN10080
1442	CONTIG7	228546	230375	1.61	0.00E+00	transcription_start_site	+	234062	234062	-4601	3311 AN10080
2438	CONTIG7	231231	231650	1.22	1.25E-01	transcription_start_site	+	232619	232619	-1178	3310 AN10080
2438	CONTIG7	231231	231650	1.22	1.25E-01	transcription_start_site	+	234062	234062	-2621	3311 AN10080
2765	CONTIG7	338792	339126	1.09	1.93E-01	transcription_start_site	-	334390	334390	-4569	3312 AN10081
2765	CONTIG7	338792	339126	1.09	1.93E-01	transcription_start_site	-	334184	334184	-4775	3313 AN10081
2845	CONTIG7	390686	391791	1.04	1.64E-01	transcription_start_site	-	386809	386809	-4429	3324 AN10084
2845	CONTIG7	390686	391791	1.04	1.64E-01	transcription_start_site	-	386222	386222	-5016	3325 AN10084
766	CONTIG7	547596	547935	2.13	3.61E-03	transcription_start_site	+	550054	550054	-2288	3332 AN10086
766	CONTIG7	547596	547935	2.13	3.61E-03	transcription_start_site	+	550361	550361	-2595	3333 AN10086
766	CONTIG7	547596	547935	2.13	3.61E-03	transcription_start_site	+	551582	551582	-3816	3334 AN10086
2436	CONTIG7	551046	551600	1.22	1.07E-01	transcription_start_site	+	551582	551582	-259	3334 AN10086
2436	CONTIG7	551046	551600	1.22	1.07E-01	transcription_start_site	+	550361	550361	962	3333 AN10086

2436	CONTIG7	551046	551600	1.22	1.07E-01	transcription_start_site	+	550054	550054	1269	3332	AN10086
812	CONTIG7	145354	145628	2.09	4.61E-03	transcription_start_site	-	142160	142160	-3331	3340	AN10088
812	CONTIG7	145354	145628	2.09	4.61E-03	transcription_start_site	-	141943	141943	-3548	3341	AN10088
812	CONTIG7	145354	145628	2.09	4.61E-03	transcription_start_site	-	141855	141855	-3636	3342	AN10088
812	CONTIG7	145354	145628	2.09	4.61E-03	transcription_start_site	-	141721	141721	-3770	3343	AN10088
812	CONTIG7	145354	145628	2.09	4.61E-03	transcription_start_site	-	141372	141372	-4119	3344	AN10088
812	CONTIG7	145354	145628	2.09	4.61E-03	transcription_start_site	-	140831	140831	-4660	3345	AN10088
812	CONTIG7	145354	145628	2.09	4.61E-03	transcription_start_site	+	149820	149820	-4329	3346	AN10089
812	CONTIG7	145354	145628	2.09	4.61E-03	transcription_start_site	+	150185	150185	-4694	3347	AN10089
812	CONTIG7	145354	145628	2.09	4.61E-03	transcription_start_site	+	150520	150520	-5029	3348	AN10089
419	CONTIG7	236346	236620	2.57	6.10E-04	transcription_start_site	+	235590	235590	893	3352	AN10090
2438	CONTIG7	231231	231650	1.22	1.25E-01	transcription_start_site	+	235590	235590	-4149	3352	AN10090
2187	CONTIG7	240996	241270	1.3	9.24E-02	transcription_start_site	+	241969	241969	-836	3353	AN10091
2187	CONTIG7	240996	241270	1.3	9.24E-02	transcription_start_site	+	242302	242302	-1169	3354	AN10091
2187	CONTIG7	240996	241270	1.3	9.24E-02	transcription_start_site	+	242502	242502	-1369	3355	AN10091
2688	CONTIG7	239041	239320	1.13	1.66E-01	transcription_start_site	+	241969	241969	-2788	3353	AN10091
2688	CONTIG7	239041	239320	1.13	1.66E-01	transcription_start_site	+	242302	242302	-3121	3354	AN10091
2688	CONTIG7	239041	239320	1.13	1.66E-01	transcription_start_site	+	242502	242502	-3321	3355	AN10091
1747	CONTIG7	331662	332166	1.48	5.22E-02	transcription_start_site	+	336103	336103	-4189	3359	AN10094
1747	CONTIG7	331662	332166	1.48	5.22E-02	transcription_start_site	+	336571	336571	-4657	3360	AN10094
2765	CONTIG7	338792	339126	1.09	1.93E-01	transcription_start_site	+	343981	343981	-5022	3361	AN10095
946	CONTIG7	357842	358186	1.96	7.85E-03	transcription_start_site	-	359111	359111	1097	3366	AN10097
1747	CONTIG7	331662	332166	1.48	5.22E-02	transcription_start_site	-	332324	332324	410	3367	AN10098
766	CONTIG7	547596	547935	2.13	3.61E-03	transcription_start_site	+	548723	548723	-957	3368	AN10099
766	CONTIG7	547596	547935	2.13	3.61E-03	transcription_start_site	+	549179	549179	-1413	3369	AN10099
2903	CONTIG15	62930	63294	1	1.79E-01	transcription_start_site	-	59555	59555	-3557	3370	AN1010
2903	CONTIG15	62930	63294	1	1.79E-01	transcription_start_site	-	58690	58690	-4422	3371	AN1010
2903	CONTIG15	62930	63294	1	1.79E-01	transcription_start_site	-	58581	58581	-4531	3372	AN1010
632	CONTIG7	166821	167236	2.26	1.80E-03	transcription_start_site	-	166660	166660	-368	3380	AN10103
632	CONTIG7	166821	167236	2.26	1.80E-03	transcription_start_site	-	166186	166186	-842	3381	AN10103
632	CONTIG7	166821	167236	2.26	1.80E-03	transcription_start_site	-	165748	165748	-1280	3382	AN10103
2586	CONTIG7	427354	427638	1.17	1.44E-01	transcription_start_site	+	427439	427439	57	3383	AN10104
2586	CONTIG7	427354	427638	1.17	1.44E-01	transcription_start_site	+	427933	427933	-437	3384	AN10104
1813	CONTIG10	151351	151635	1.45	8.01E-02	transcription_start_site	+	150801	150801	692	3398	AN10109
2106	CONTIG15	65195	65459	1.33	5.88E-02	transcription_start_site	-	61066	61066	-4261	3399	AN1011
2106	CONTIG15	65195	65459	1.33	5.88E-02	transcription_start_site	-	60915	60915	-4412	3400	AN1011
2106	CONTIG15	65195	65459	1.33	5.88E-02	transcription_start_site	-	60608	60608	-4719	3401	AN1011
2106	CONTIG15	65195	65459	1.33	5.88E-02	transcription_start_site	-	60387	60387	-4940	3402	AN1011
2106	CONTIG15	65195	65459	1.33	5.88E-02	transcription_start_site	-	60271	60271	-5056	3403	AN1011
2903	CONTIG15	62930	63294	1	1.79E-01	transcription_start_site	-	61066	61066	-2046	3399	AN1011
2903	CONTIG15	62930	63294	1	1.79E-01	transcription_start_site	-	60915	60915	-2197	3400	AN1011
2903	CONTIG15	62930	63294	1	1.79E-01	transcription_start_site	-	60608	60608	-2504	3401	AN1011
2903	CONTIG15	62930	63294	1	1.79E-01	transcription_start_site	-	60387	60387	-2725	3402	AN1011
2903	CONTIG15	62930	63294	1	1.79E-01	transcription_start_site	-	60271	60271	-2841	3403	AN1011
1813	CONTIG10	151351	151635	1.45	8.01E-02	transcription_start_site	+	151459	151459	34	3410	AN10113
1813	CONTIG10	151351	151635	1.45	8.01E-02	transcription_start_site	+	151389	151389	104	3409	AN10113
2152	CONTIG11	49954	50544	1.32	1.66E-01	transcription_start_site	+	54152	54152	-3903	3412	AN10115
2152	CONTIG11	49954	50544	1.32	1.66E-01	transcription_start_site	+	55064	55064	-4815	3413	AN10115
2152	CONTIG11	49954	50544	1.32	1.66E-01	transcription_start_site	+	55294	55294	-5045	3414	AN10115
2152	CONTIG11	49954	50544	1.32	1.66E-01	transcription_start_site	+	51163	51163	-914	3416	AN10116
2152	CONTIG11	49954	50544	1.32	1.66E-01	transcription_start_site	+	51357	51357	-1108	3417	AN10116
2152	CONTIG11	49954	50544	1.32	1.66E-01	transcription_start_site	+	51535	51535	-1286	3418	AN10116
465	CONTIG12	132087	132426	2.49	1.22E-03	transcription_start_site	-	127292	127292	-4964	3426	AN10119
1548	CONTIG12	146851	147215	1.56	3.33E-02	transcription_start_site	-	142697	142697	-4336	3432	AN10120
1548	CONTIG12	146851	147215	1.56	3.33E-02	transcription_start_site	-	142493	142493	-4540	3433	AN10120
465	CONTIG12	132087	132426	2.49	1.22E-03	transcription_start_site	-	128213	128213	-4043	3436	AN10121
465	CONTIG12	132087	132426	2.49	1.22E-03	transcription_start_site	-	128036	128036	-4220	3437	AN10121

465	CONTIG12	132087	132426	2.49	1.22E-03	transcription_start_site	-	127903	127903	-4353	3438 AN10121
465	CONTIG12	132087	132426	2.49	1.22E-03	transcription_start_site	-	127785	127785	-4471	3439 AN10121
1660	CONTIG13	79067	79495	1.52	1.08E-01	transcription_start_site	-	78980	78980	-301	3448 AN10124
1660	CONTIG13	79067	79495	1.52	1.08E-01	transcription_start_site	-	78855	78855	-426	3449 AN10124
1660	CONTIG13	79067	79495	1.52	1.08E-01	transcription_start_site	-	78599	78599	-682	3450 AN10124
1660	CONTIG13	79067	79495	1.52	1.08E-01	transcription_start_site	-	78073	78073	-1208	3451 AN10124
1660	CONTIG13	79067	79495	1.52	1.08E-01	transcription_start_site	-	77415	77415	-1866	3452 AN10124
1457	CONTIG14	34818	35302	1.61	3.70E-02	transcription_start_site	+	35477	35477	-417	3457 AN10128
1457	CONTIG14	34818	35302	1.61	3.70E-02	transcription_start_site	+	35678	35678	-618	3458 AN10128
1457	CONTIG14	34818	35302	1.61	3.70E-02	transcription_start_site	+	35797	35797	-737	3459 AN10128
1457	CONTIG14	34818	35302	1.61	3.70E-02	transcription_start_site	+	36371	36371	-1311	3460 AN10128
1457	CONTIG14	34818	35302	1.61	3.70E-02	transcription_start_site	+	36526	36526	-1466	3461 AN10128
1457	CONTIG14	34818	35302	1.61	3.70E-02	transcription_start_site	+	37574	37574	-2514	3462 AN10128
1457	CONTIG14	34818	35302	1.61	3.70E-02	transcription_start_site	+	37730	37730	-2670	3463 AN10128
1457	CONTIG14	34818	35302	1.61	3.70E-02	transcription_start_site	+	38450	38450	-3390	3464 AN10128
2053	CONTIG14	32402	32986	1.35	7.25E-02	transcription_start_site	+	35477	35477	-2783	3457 AN10128
2053	CONTIG14	32402	32986	1.35	7.25E-02	transcription_start_site	+	35678	35678	-2984	3458 AN10128
2053	CONTIG14	32402	32986	1.35	7.25E-02	transcription_start_site	+	35797	35797	-3103	3459 AN10128
2053	CONTIG14	32402	32986	1.35	7.25E-02	transcription_start_site	+	36371	36371	-3677	3460 AN10128
2053	CONTIG14	32402	32986	1.35	7.25E-02	transcription_start_site	+	36526	36526	-3832	3461 AN10128
2053	CONTIG14	32402	32986	1.35	7.25E-02	transcription_start_site	+	37574	37574	-4880	3462 AN10128
2053	CONTIG14	32402	32986	1.35	7.25E-02	transcription_start_site	+	37730	37730	-5036	3463 AN10128
2325	CONTIG14	241742	242151	1.26	1.07E-01	transcription_start_site	-	240159	240159	-1787	3465 AN10129
2325	CONTIG14	241742	242151	1.26	1.07E-01	transcription_start_site	-	240016	240016	-1930	3466 AN10129
2325	CONTIG14	241742	242151	1.26	1.07E-01	transcription_start_site	-	239724	239724	-2222	3467 AN10129
2903	CONTIG15	62930	63294	1	1.79E-01	transcription_start_site	+	62703	62703	409	3470 AN1013
2903	CONTIG15	62930	63294	1	1.79E-01	transcription_start_site	+	62611	62611	501	3469 AN1013
2903	CONTIG15	62930	63294	1	1.79E-01	transcription_start_site	+	62443	62443	669	3468 AN1013
1457	CONTIG14	34818	35302	1.61	3.70E-02	transcription_start_site	+	39892	39892	-4832	3481 AN10133
2325	CONTIG14	241742	242151	1.26	1.07E-01	transcription_start_site	-	238955	238955	-2991	3482 AN10134
2325	CONTIG14	241742	242151	1.26	1.07E-01	transcription_start_site	-	238569	238569	-3377	3483 AN10134
2325	CONTIG14	241742	242151	1.26	1.07E-01	transcription_start_site	-	237604	237604	-4342	3484 AN10135
2325	CONTIG14	241742	242151	1.26	1.07E-01	transcription_start_site	-	237434	237434	-4512	3485 AN10135
2325	CONTIG14	241742	242151	1.26	1.07E-01	transcription_start_site	-	237030	237030	-4916	3486 AN10135
1355	CONTIG14	245852	246206	1.66	3.08E-02	transcription_start_site	-	245397	245397	-632	3487 AN10136
1355	CONTIG14	245852	246206	1.66	3.08E-02	transcription_start_site	-	245076	245076	-953	3488 AN10136
1355	CONTIG14	245852	246206	1.66	3.08E-02	transcription_start_site	-	244285	244285	-1744	3489 AN10136
1355	CONTIG14	245852	246206	1.66	3.08E-02	transcription_start_site	-	244068	244068	-1961	3490 AN10136
1355	CONTIG14	245852	246206	1.66	3.08E-02	transcription_start_site	-	243848	243848	-2181	3491 AN10136
1355	CONTIG14	245852	246206	1.66	3.08E-02	transcription_start_site	-	243634	243634	-2395	3492 AN10136
2473	CONTIG15	9546	9820	1.21	9.24E-02	transcription_start_site	+	14455	14455	-4772	3496 AN10138
2106	CONTIG15	65195	65459	1.33	5.88E-02	transcription_start_site	+	70159	70159	-4832	3500 AN1014
2106	CONTIG15	65195	65459	1.33	5.88E-02	transcription_start_site	+	65805	65805	-478	3504 AN10140
2106	CONTIG15	65195	65459	1.33	5.88E-02	transcription_start_site	+	65981	65981	-654	3505 AN10140
2106	CONTIG15	65195	65459	1.33	5.88E-02	transcription_start_site	+	68850	68850	-3523	3506 AN10140
2903	CONTIG15	62930	63294	1	1.79E-01	transcription_start_site	+	65805	65805	-2693	3504 AN10140
2903	CONTIG15	62930	63294	1	1.79E-01	transcription_start_site	+	65981	65981	-2869	3505 AN10140
1362	CONTIG15	171379	172038	1.65	7.16E-03	transcription_start_site	+	175795	175795	-4086	3516 AN10144
379	CONTIG16	22068	22477	2.63	0.00E+00	transcription_start_site	-	19021	19021	-3251	3517 AN10145
379	CONTIG16	22068	22477	2.63	0.00E+00	transcription_start_site	-	18571	18571	-3701	3518 AN10145
2090	CONTIG16	226217	226486	1.34	1.44E-01	transcription_start_site	-	221453	221453	-4898	3521 AN10147
466	CONTIG16	307064	307493	2.48	0.00E+00	transcription_start_site	-	306697	306697	-581	3526 AN10149
466	CONTIG16	307064	307493	2.48	0.00E+00	transcription_start_site	-	306428	306428	-850	3527 AN10149
147	CONTIG15	79656	80097	3.05	0.00E+00	transcription_start_site	-	75982	75982	-3894	3528 AN1015
147	CONTIG15	79656	80097	3.05	0.00E+00	transcription_start_site	-	75739	75739	-4137	3529 AN1015
147	CONTIG15	79656	80097	3.05	0.00E+00	transcription_start_site	-	75655	75655	-4221	3530 AN1015
147	CONTIG15	79656	80097	3.05	0.00E+00	transcription_start_site	-	75566	75566	-4310	3531 AN1015

147	CONTIG15	79656	80097	3.05	0.00E+00	transcription_start_site	-	75452	75452	-4424	3532	AN1015
147	CONTIG15	79656	80097	3.05	0.00E+00	transcription_start_site	-	75298	75298	-4578	3533	AN1015
147	CONTIG15	79656	80097	3.05	0.00E+00	transcription_start_site	-	75170	75170	-4706	3534	AN1015
181	CONTIG16	331659	332005	2.98	2.16E-04	transcription_start_site	+	331357	331357	475	3539	AN10150
181	CONTIG16	331659	332005	2.98	2.16E-04	transcription_start_site	+	331150	331150	682	3538	AN10150
181	CONTIG16	331659	332005	2.98	2.16E-04	transcription_start_site	+	330831	330831	1001	3537	AN10150
1605	CONTIG16	414088	414368	1.54	8.01E-02	transcription_start_site	-	415141	415141	913	3547	AN10152
1605	CONTIG16	414088	414368	1.54	8.01E-02	transcription_start_site	-	414165	414165	-63	3549	AN10153
1605	CONTIG16	414088	414368	1.54	8.01E-02	transcription_start_site	-	414526	414526	298	3548	AN10153
1605	CONTIG16	414088	414368	1.54	8.01E-02	transcription_start_site	-	412560	412560	-1668	3550	AN10153
1605	CONTIG16	414088	414368	1.54	8.01E-02	transcription_start_site	-	410722	410722	-3506	3551	AN10153
466	CONTIG16	307064	307493	2.48	0.00E+00	transcription_start_site	-	304949	304949	-2329	3554	AN10155
466	CONTIG16	307064	307493	2.48	0.00E+00	transcription_start_site	-	304463	304463	-2815	3555	AN10155
466	CONTIG16	307064	307493	2.48	0.00E+00	transcription_start_site	-	303766	303766	-3512	3556	AN10155
864	CONTIG16	288844	289108	2.04	1.57E-02	transcription_start_site	-	288286	288286	-690	3557	AN10156
864	CONTIG16	288844	289108	2.04	1.57E-02	transcription_start_site	-	288066	288066	-910	3558	AN10156
864	CONTIG16	288844	289108	2.04	1.57E-02	transcription_start_site	-	287883	287883	-1093	3559	AN10156
864	CONTIG16	288844	289108	2.04	1.57E-02	transcription_start_site	-	287374	287374	-1602	3560	AN10156
864	CONTIG16	288844	289108	2.04	1.57E-02	transcription_start_site	-	286989	286989	-1987	3561	AN10156
1494	CONTIG16	144616	144885	1.59	6.84E-02	transcription_start_site	+	145762	145762	-1011	3562	AN10157
1834	CONTIG16	66685	67264	1.44	9.26E-02	transcription_start_site	-	64659	64659	-2315	3565	AN10159
1834	CONTIG16	66685	67264	1.44	9.26E-02	transcription_start_site	-	64584	64584	-2390	3566	AN10159
1834	CONTIG16	66685	67264	1.44	9.26E-02	transcription_start_site	-	64475	64475	-2499	3567	AN10159
1834	CONTIG16	66685	67264	1.44	9.26E-02	transcription_start_site	-	64345	64345	-2629	3568	AN10159
147	CONTIG15	79656	80097	3.05	0.00E+00	transcription_start_site	-	78761	78761	-1115	3569	AN1016
147	CONTIG15	79656	80097	3.05	0.00E+00	transcription_start_site	-	78546	78546	-1330	3570	AN1016
147	CONTIG15	79656	80097	3.05	0.00E+00	transcription_start_site	-	78149	78149	-1727	3571	AN1016
147	CONTIG15	79656	80097	3.05	0.00E+00	transcription_start_site	-	77967	77967	-1909	3572	AN1016
147	CONTIG15	79656	80097	3.05	0.00E+00	transcription_start_site	-	77788	77788	-2088	3573	AN1016
147	CONTIG15	79656	80097	3.05	0.00E+00	transcription_start_site	-	77490	77490	-2386	3574	AN1016
1304	CONTIG15	82201	82700	1.69	1.30E-02	transcription_start_site	-	78761	78761	-3689	3569	AN1016
1304	CONTIG15	82201	82700	1.69	1.30E-02	transcription_start_site	-	78546	78546	-3904	3570	AN1016
1304	CONTIG15	82201	82700	1.69	1.30E-02	transcription_start_site	-	78149	78149	-4301	3571	AN1016
1304	CONTIG15	82201	82700	1.69	1.30E-02	transcription_start_site	-	77967	77967	-4483	3572	AN1016
1304	CONTIG15	82201	82700	1.69	1.30E-02	transcription_start_site	-	77788	77788	-4662	3573	AN1016
1304	CONTIG15	82201	82700	1.69	1.30E-02	transcription_start_site	-	77490	77490	-4960	3574	AN1016
32	CONTIG16	24243	24657	3.53	0.00E+00	transcription_start_site	-	20149	20149	-4301	3575	AN10160
379	CONTIG16	22068	22477	2.63	0.00E+00	transcription_start_site	-	20149	20149	-2123	3575	AN10160
1094	CONTIG16	122268	122614	1.84	3.08E-02	transcription_start_site	-	117400	117400	-5041	3579	AN10162
380	CONTIG16	139521	139942	2.63	0.00E+00	transcription_start_site	+	143150	143150	-3418	3582	AN10164
380	CONTIG16	139521	139942	2.63	0.00E+00	transcription_start_site	+	143600	143600	-3868	3583	AN10164
380	CONTIG16	139521	139942	2.63	0.00E+00	transcription_start_site	+	143760	143760	-4028	3584	AN10164
380	CONTIG16	139521	139942	2.63	0.00E+00	transcription_start_site	+	144459	144459	-4727	3585	AN10164
380	CONTIG16	139521	139942	2.63	0.00E+00	transcription_start_site	+	144659	144659	-4927	3586	AN10164
1494	CONTIG16	144616	144885	1.59	6.84E-02	transcription_start_site	+	144659	144659	91	3586	AN10164
1494	CONTIG16	144616	144885	1.59	6.84E-02	transcription_start_site	+	144459	144459	291	3585	AN10164
1494	CONTIG16	144616	144885	1.59	6.84E-02	transcription_start_site	+	143760	143760	990	3584	AN10164
2588	CONTIG17	231078	231530	1.17	1.50E-01	transcription_start_site	+	230476	230476	828	3599	AN10169
2588	CONTIG17	231078	231530	1.17	1.50E-01	transcription_start_site	+	230371	230371	933	3598	AN10169
147	CONTIG15	79656	80097	3.05	0.00E+00	transcription_start_site	+	82917	82917	-3040	3600	AN1017
147	CONTIG15	79656	80097	3.05	0.00E+00	transcription_start_site	+	83034	83034	-3157	3601	AN1017
147	CONTIG15	79656	80097	3.05	0.00E+00	transcription_start_site	+	83194	83194	-3317	3602	AN1017
147	CONTIG15	79656	80097	3.05	0.00E+00	transcription_start_site	+	83386	83386	-3509	3603	AN1017
147	CONTIG15	79656	80097	3.05	0.00E+00	transcription_start_site	+	83494	83494	-3617	3604	AN1017
147	CONTIG15	79656	80097	3.05	0.00E+00	transcription_start_site	+	83647	83647	-3770	3605	AN1017
147	CONTIG15	79656	80097	3.05	0.00E+00	transcription_start_site	+	84070	84070	-4193	3606	AN1017
147	CONTIG15	79656	80097	3.05	0.00E+00	transcription_start_site	+	84249	84249	-4372	3607	AN1017

147	CONTIG15	79656	80097	3.05	0.00E+00	transcription_start_site	+	84492	84492	-4615	3608	AN1017
1304	CONTIG15	82201	82700	1.69	1.30E-02	transcription_start_site	+	82917	82917	-466	3600	AN1017
1304	CONTIG15	82201	82700	1.69	1.30E-02	transcription_start_site	+	83034	83034	-583	3601	AN1017
1304	CONTIG15	82201	82700	1.69	1.30E-02	transcription_start_site	+	83194	83194	-743	3602	AN1017
1304	CONTIG15	82201	82700	1.69	1.30E-02	transcription_start_site	+	83386	83386	-935	3603	AN1017
1304	CONTIG15	82201	82700	1.69	1.30E-02	transcription_start_site	+	83494	83494	-1043	3604	AN1017
1304	CONTIG15	82201	82700	1.69	1.30E-02	transcription_start_site	+	83647	83647	-1196	3605	AN1017
1304	CONTIG15	82201	82700	1.69	1.30E-02	transcription_start_site	+	84070	84070	-1619	3606	AN1017
1304	CONTIG15	82201	82700	1.69	1.30E-02	transcription_start_site	+	84249	84249	-1798	3607	AN1017
1304	CONTIG15	82201	82700	1.69	1.30E-02	transcription_start_site	+	84492	84492	-2041	3608	AN1017
2326	CONTIG17	309314	309888	1.26	1.07E-01	transcription_start_site	-	309405	309405	-196	3612	AN10171
2326	CONTIG17	309314	309888	1.26	1.07E-01	transcription_start_site	-	308287	308287	-1314	3613	AN10171
1621	CONTIG17	337358	337715	1.53	5.22E-02	transcription_start_site	-	337378	337378	-158	3614	AN10172
1621	CONTIG17	337358	337715	1.53	5.22E-02	transcription_start_site	-	337241	337241	-295	3615	AN10172
1621	CONTIG17	337358	337715	1.53	5.22E-02	transcription_start_site	-	337144	337144	-392	3616	AN10172
1621	CONTIG17	337358	337715	1.53	5.22E-02	transcription_start_site	-	337053	337053	-483	3617	AN10172
1621	CONTIG17	337358	337715	1.53	5.22E-02	transcription_start_site	-	336876	336876	-660	3618	AN10172
2588	CONTIG17	231078	231530	1.17	1.50E-01	transcription_start_site	+	231581	231581	-277	3621	AN10174
2588	CONTIG17	231078	231530	1.17	1.50E-01	transcription_start_site	+	231925	231925	-621	3622	AN10174
1829	CONTIG17	306319	306901	1.44	6.84E-02	transcription_start_site	-	306514	306514	-96	3625	AN10176
1829	CONTIG17	306319	306901	1.44	6.84E-02	transcription_start_site	-	306375	306375	-235	3626	AN10176
2326	CONTIG17	309314	309888	1.26	1.07E-01	transcription_start_site	-	306514	306514	-3087	3625	AN10176
2326	CONTIG17	309314	309888	1.26	1.07E-01	transcription_start_site	-	306375	306375	-3226	3626	AN10176
752	CONTIG17	318916	319190	2.15	4.61E-03	transcription_start_site	-	314234	314234	-4819	3628	AN10178
752	CONTIG17	318916	319190	2.15	4.61E-03	transcription_start_site	-	314081	314081	-4972	3629	AN10178
1621	CONTIG17	337358	337715	1.53	5.22E-02	transcription_start_site	-	338357	338357	820	3633	AN10179
1304	CONTIG15	82201	82700	1.69	1.30E-02	transcription_start_site	+	85232	85232	-2781	3634	AN1018
1304	CONTIG15	82201	82700	1.69	1.30E-02	transcription_start_site	+	85487	85487	-3036	3635	AN1018
1304	CONTIG15	82201	82700	1.69	1.30E-02	transcription_start_site	+	85653	85653	-3202	3636	AN1018
1304	CONTIG15	82201	82700	1.69	1.30E-02	transcription_start_site	+	86024	86024	-3573	3637	AN1018
1304	CONTIG15	82201	82700	1.69	1.30E-02	transcription_start_site	+	86107	86107	-3656	3638	AN1018
1304	CONTIG15	82201	82700	1.69	1.30E-02	transcription_start_site	+	86223	86223	-3772	3639	AN1018
1304	CONTIG15	82201	82700	1.69	1.30E-02	transcription_start_site	+	86360	86360	-3909	3640	AN1018
1304	CONTIG15	82201	82700	1.69	1.30E-02	transcription_start_site	+	86586	86586	-4135	3641	AN1018
1304	CONTIG15	82201	82700	1.69	1.30E-02	transcription_start_site	+	86681	86681	-4230	3642	AN1018
1304	CONTIG15	82201	82700	1.69	1.30E-02	transcription_start_site	+	86874	86874	-4423	3643	AN1018
222	CONTIG18	99388	99892	2.89	0.00E+00	transcription_start_site	-	98436	98436	-1204	3648	AN10181
222	CONTIG18	99388	99892	2.89	0.00E+00	transcription_start_site	-	95902	95902	-3738	3652	AN10183
222	CONTIG18	99388	99892	2.89	0.00E+00	transcription_start_site	-	95434	95434	-4206	3653	AN10183
3039	CONTIG19	29484	29762	0.8	1.25E-01	transcription_start_site	+	31617	31617	-1994	3654	AN10184
3039	CONTIG19	29484	29762	0.8	1.25E-01	transcription_start_site	+	31864	31864	-2241	3655	AN10184
3039	CONTIG19	29484	29762	0.8	1.25E-01	transcription_start_site	+	32288	32288	-2665	3656	AN10184
1391	CONTIG19	60241	60515	1.63	8.69E-04	transcription_start_site	-	60083	60083	-295	3660	AN10186
1391	CONTIG19	60241	60515	1.63	8.69E-04	transcription_start_site	-	59996	59996	-382	3661	AN10186
1391	CONTIG19	60241	60515	1.63	8.69E-04	transcription_start_site	-	59847	59847	-531	3662	AN10186
1391	CONTIG19	60241	60515	1.63	8.69E-04	transcription_start_site	-	59409	59409	-969	3663	AN10186
1391	CONTIG19	60241	60515	1.63	8.69E-04	transcription_start_site	-	58928	58928	-1450	3664	AN10186
1391	CONTIG19	60241	60515	1.63	8.69E-04	transcription_start_site	-	58579	58579	-1799	3665	AN10186
1391	CONTIG19	60241	60515	1.63	8.69E-04	transcription_start_site	-	58425	58425	-1953	3666	AN10186
2651	CONTIG19	61516	61939	1.14	1.84E-02	transcription_start_site	-	60083	60083	-1644	3660	AN10186
2651	CONTIG19	61516	61939	1.14	1.84E-02	transcription_start_site	-	59996	59996	-1731	3661	AN10186
2651	CONTIG19	61516	61939	1.14	1.84E-02	transcription_start_site	-	59847	59847	-1880	3662	AN10186
2651	CONTIG19	61516	61939	1.14	1.84E-02	transcription_start_site	-	59409	59409	-2318	3663	AN10186
2651	CONTIG19	61516	61939	1.14	1.84E-02	transcription_start_site	-	58928	58928	-2799	3664	AN10186
2651	CONTIG19	61516	61939	1.14	1.84E-02	transcription_start_site	-	58579	58579	-3148	3665	AN10186
2651	CONTIG19	61516	61939	1.14	1.84E-02	transcription_start_site	-	58425	58425	-3302	3666	AN10186
1391	CONTIG19	60241	60515	1.63	8.69E-04	transcription_start_site	-	57982	57982	-2396	3669	AN10188

1391	CONTIG19	60241	60515	1.63	8.69E-04	transcription_start_site	-	57811	57811	-2567	3670	AN10188
1391	CONTIG19	60241	60515	1.63	8.69E-04	transcription_start_site	-	57631	57631	-2747	3671	AN10188
1391	CONTIG19	60241	60515	1.63	8.69E-04	transcription_start_site	-	57343	57343	-3035	3672	AN10188
2651	CONTIG19	61516	61939	1.14	1.84E-02	transcription_start_site	-	57982	57982	-3745	3669	AN10188
2651	CONTIG19	61516	61939	1.14	1.84E-02	transcription_start_site	-	57811	57811	-3916	3670	AN10188
2651	CONTIG19	61516	61939	1.14	1.84E-02	transcription_start_site	-	57631	57631	-4096	3671	AN10188
2651	CONTIG19	61516	61939	1.14	1.84E-02	transcription_start_site	-	57343	57343	-4384	3672	AN10188
3039	CONTIG19	29484	29762	0.8	1.25E-01	transcription_start_site	+	30240	30240	-617	3673	AN10189
3039	CONTIG19	29484	29762	0.8	1.25E-01	transcription_start_site	+	30693	30693	-1070	3674	AN10189
3039	CONTIG19	29484	29762	0.8	1.25E-01	transcription_start_site	+	30864	30864	-1241	3675	AN10189
3039	CONTIG19	29484	29762	0.8	1.25E-01	transcription_start_site	+	31038	31038	-1415	3676	AN10189
2216	CONTIG15	94727	95226	1.29	6.84E-02	transcription_start_site	-	91674	91674	-3302	3677	AN1019
2216	CONTIG15	94727	95226	1.29	6.84E-02	transcription_start_site	-	91543	91543	-3433	3678	AN1019
2216	CONTIG15	94727	95226	1.29	6.84E-02	transcription_start_site	-	91401	91401	-3575	3679	AN1019
2216	CONTIG15	94727	95226	1.29	6.84E-02	transcription_start_site	-	91319	91319	-3657	3680	AN1019
2216	CONTIG15	94727	95226	1.29	6.84E-02	transcription_start_site	-	91171	91171	-3805	3681	AN1019
2451	CONTIG22	103284	103568	1.22	1.93E-01	transcription_start_site	+	104342	104342	-916	3684	AN10191
2451	CONTIG22	103284	103568	1.22	1.93E-01	transcription_start_site	+	106423	106423	-2997	3685	AN10191
316	CONTIG22	183468	183805	2.74	9.51E-04	transcription_start_site	-	181212	181212	-2424	3686	AN10192
1532	CONTIG22	212483	213502	1.57	6.84E-02	transcription_start_site	+	214174	214174	-1181	3687	AN10193
1532	CONTIG22	212483	213502	1.57	6.84E-02	transcription_start_site	+	214347	214347	-1354	3688	AN10193
1532	CONTIG22	212483	213502	1.57	6.84E-02	transcription_start_site	+	215003	215003	-2010	3689	AN10193
316	CONTIG22	183468	183805	2.74	9.51E-04	transcription_start_site	-	182890	182890	-746	3698	AN10197
316	CONTIG22	183468	183805	2.74	9.51E-04	transcription_start_site	-	182583	182583	-1053	3699	AN10197
316	CONTIG22	183468	183805	2.74	9.51E-04	transcription_start_site	-	182491	182491	-1145	3700	AN10197
316	CONTIG22	183468	183805	2.74	9.51E-04	transcription_start_site	-	182321	182321	-1315	3701	AN10197
1886	CONTIG22	108529	108878	1.42	1.08E-01	transcription_start_site	+	109613	109613	-909	3715	AN10204
1886	CONTIG22	108529	108878	1.42	1.08E-01	transcription_start_site	+	110029	110029	-1325	3716	AN10204
1532	CONTIG22	212483	213502	1.57	6.84E-02	transcription_start_site	+	216071	216071	-3078	3720	AN10207
1532	CONTIG22	212483	213502	1.57	6.84E-02	transcription_start_site	+	216325	216325	-3332	3721	AN10207
1532	CONTIG22	212483	213502	1.57	6.84E-02	transcription_start_site	+	216585	216585	-3592	3722	AN10207
1532	CONTIG22	212483	213502	1.57	6.84E-02	transcription_start_site	+	216794	216794	-3801	3723	AN10207
1532	CONTIG22	212483	213502	1.57	6.84E-02	transcription_start_site	+	217277	217277	-4284	3724	AN10207
1532	CONTIG22	212483	213502	1.57	6.84E-02	transcription_start_site	+	217430	217430	-4437	3725	AN10207
1532	CONTIG22	212483	213502	1.57	6.84E-02	transcription_start_site	+	217538	217538	-4545	3726	AN10207
2300	CONTIG22	217808	218087	1.27	1.66E-01	transcription_start_site	+	217538	217538	409	3726	AN10207
2300	CONTIG22	217808	218087	1.27	1.66E-01	transcription_start_site	+	217430	217430	517	3725	AN10207
2300	CONTIG22	217808	218087	1.27	1.66E-01	transcription_start_site	+	217277	217277	670	3724	AN10207
1471	CONTIG23	5251	5525	1.61	8.01E-02	transcription_start_site	+	8579	8579	-3191	3731	AN10209
1471	CONTIG23	5251	5525	1.61	8.01E-02	transcription_start_site	+	8726	8726	-3338	3732	AN10209
1471	CONTIG23	5251	5525	1.61	8.01E-02	transcription_start_site	+	8937	8937	-3549	3733	AN10209
1471	CONTIG23	5251	5525	1.61	8.01E-02	transcription_start_site	+	9347	9347	-3959	3734	AN10209
1471	CONTIG23	5251	5525	1.61	8.01E-02	transcription_start_site	+	9690	9690	-4302	3735	AN10209
2216	CONTIG15	94727	95226	1.29	6.84E-02	transcription_start_site	-	94823	94823	-153	3737	AN1021
2216	CONTIG15	94727	95226	1.29	6.84E-02	transcription_start_site	-	94481	94481	-495	3738	AN1021
1471	CONTIG23	5251	5525	1.61	8.01E-02	transcription_start_site	+	7084	7084	-1696	3746	AN10211
1471	CONTIG23	5251	5525	1.61	8.01E-02	transcription_start_site	+	7231	7231	-1843	3747	AN10211
2607	CONTIG25	86495	86979	1.16	9.24E-02	transcription_start_site	+	86077	86077	660	3761	AN10219
2607	CONTIG25	86495	86979	1.16	9.24E-02	transcription_start_site	+	85586	85586	1151	3760	AN10219
2894	CONTIG25	85205	85494	1.01	1.66E-01	transcription_start_site	+	85586	85586	-236	3760	AN10219
2894	CONTIG25	85205	85494	1.01	1.66E-01	transcription_start_site	+	86077	86077	-727	3761	AN10219
2216	CONTIG15	94727	95226	1.29	6.84E-02	transcription_start_site	+	95400	95400	-423	3762	AN1022
2216	CONTIG15	94727	95226	1.29	6.84E-02	transcription_start_site	+	95911	95911	-934	3763	AN1022
2216	CONTIG15	94727	95226	1.29	6.84E-02	transcription_start_site	+	96020	96020	-1043	3764	AN1022
806	CONTIG26	99535	100044	2.1	1.11E-02	transcription_start_site	+	100033	100033	-243	3765	AN10220
806	CONTIG26	99535	100044	2.1	1.11E-02	transcription_start_site	+	100433	100433	-643	3766	AN10220
2145	CONTIG26	95629	95978	1.32	1.44E-01	transcription_start_site	+	100033	100033	-4229	3765	AN10220

2145	CONTIG26	95629	95978	1.32	1.44E-01	transcription_start_site	+	100433	100433	-4629	3766 AN10220
1888	CONTIG26	193519	193868	1.42	1.08E-01	transcription_start_site	-	194231	194231	537	3768 AN10221
1783	CONTIG26	317117	317391	1.47	9.24E-02	transcription_start_site	+	316138	316138	1116	3776 AN10223
806	CONTIG26	99535	100044	2.1	1.11E-02	transcription_start_site	+	101736	101736	-1946	3777 AN10224
1783	CONTIG26	317117	317391	1.47	9.24E-02	transcription_start_site	+	317984	317984	-730	3782 AN10227
2216	CONTIG15	94727	95226	1.29	6.84E-02	transcription_start_site	+	96718	96718	-1741	3791 AN1023
2216	CONTIG15	94727	95226	1.29	6.84E-02	transcription_start_site	+	97032	97032	-2055	3792 AN1023
2216	CONTIG15	94727	95226	1.29	6.84E-02	transcription_start_site	+	97331	97331	-2354	3793 AN1023
2318	CONTIG27	40587	40944	1.26	6.84E-02	transcription_start_site	+	42956	42956	-2190	3794 AN10230
2318	CONTIG27	40587	40944	1.26	6.84E-02	transcription_start_site	+	43112	43112	-2346	3795 AN10230
2318	CONTIG27	40587	40944	1.26	6.84E-02	transcription_start_site	+	43668	43668	-2902	3796 AN10230
2432	CONTIG27	42842	44089	1.22	5.52E-02	transcription_start_site	+	43668	43668	-202	3796 AN10230
2432	CONTIG27	42842	44089	1.22	5.52E-02	transcription_start_site	+	43112	43112	353	3795 AN10230
2432	CONTIG27	42842	44089	1.22	5.52E-02	transcription_start_site	+	42956	42956	509	3794 AN10230
2926	CONTIG27	38787	39436	0.99	1.93E-01	transcription_start_site	+	42956	42956	-3844	3794 AN10230
2926	CONTIG27	38787	39436	0.99	1.93E-01	transcription_start_site	+	43112	43112	-4000	3795 AN10230
2926	CONTIG27	38787	39436	0.99	1.93E-01	transcription_start_site	+	43668	43668	-4556	3796 AN10230
2318	CONTIG27	40587	40944	1.26	6.84E-02	transcription_start_site	+	41069	41069	-303	3800 AN10233
2924	CONTIG27	37141	37491	0.99	1.79E-01	transcription_start_site	+	41069	41069	-3753	3800 AN10233
2926	CONTIG27	38787	39436	0.99	1.93E-01	transcription_start_site	+	41069	41069	-1957	3800 AN10233
767	CONTIG29	106140	106404	2.13	1.11E-02	transcription_start_site	+	106374	106374	-102	3801 AN10234
767	CONTIG29	106140	106404	2.13	1.11E-02	transcription_start_site	+	107166	107166	-894	3802 AN10234
1240	CONTIG29	107118	107452	1.74	4.36E-02	transcription_start_site	+	107166	107166	119	3802 AN10234
1240	CONTIG29	107118	107452	1.74	4.36E-02	transcription_start_site	+	106374	106374	911	3801 AN10234
2092	CONTIG29	101101	101470	1.34	1.44E-01	transcription_start_site	+	106374	106374	-5088	3801 AN10234
2236	CONTIG29	101791	102290	1.29	1.50E-01	transcription_start_site	+	106374	106374	-4333	3801 AN10234
2236	CONTIG29	101791	102290	1.29	1.50E-01	transcription_start_site	+	107166	107166	-5125	3802 AN10234
2242	CONTIG29	104865	105744	1.29	1.66E-01	transcription_start_site	+	106374	106374	-1069	3801 AN10234
2242	CONTIG29	104865	105744	1.29	1.66E-01	transcription_start_site	+	107166	107166	-1861	3802 AN10234
767	CONTIG29	106140	106404	2.13	1.11E-02	transcription_start_site	+	107828	107828	-1556	3810 AN10238
767	CONTIG29	106140	106404	2.13	1.11E-02	transcription_start_site	+	108063	108063	-1791	3811 AN10238
1240	CONTIG29	107118	107452	1.74	4.36E-02	transcription_start_site	+	107828	107828	-543	3810 AN10238
1240	CONTIG29	107118	107452	1.74	4.36E-02	transcription_start_site	+	108063	108063	-778	3811 AN10238
2242	CONTIG29	104865	105744	1.29	1.66E-01	transcription_start_site	+	107828	107828	-2523	3810 AN10238
2242	CONTIG29	104865	105744	1.29	1.66E-01	transcription_start_site	+	108063	108063	-2758	3811 AN10238
2582	CONTIG15	105302	105656	1.17	1.08E-01	transcription_start_site	-	101200	101200	-4279	3815 AN1024
2582	CONTIG15	105302	105656	1.17	1.08E-01	transcription_start_site	-	100973	100973	-4506	3816 AN1024
1600	CONTIG29	52509	53753	1.54	5.52E-02	transcription_start_site	-	51155	51155	-1976	3822 AN10242
1600	CONTIG29	52509	53753	1.54	5.52E-02	transcription_start_site	-	50721	50721	-2410	3823 AN10242
507	CONTIG29	276226	276595	2.43	4.46E-04	transcription_start_site	+	275269	275269	1141	3829 AN10245
87	CONTIG29	279311	279741	3.27	0.00E+00	transcription_start_site	+	283987	283987	-4461	3830 AN10246
160	CONTIG29	282976	283269	3.03	2.39E-04	transcription_start_site	+	283987	283987	-864	3830 AN10246
1734	CONTIG29	280361	281090	1.49	9.24E-02	transcription_start_site	+	283987	283987	-3261	3830 AN10246
2582	CONTIG15	105302	105656	1.17	1.08E-01	transcription_start_site	+	105946	105946	-467	3836 AN1025
2582	CONTIG15	105302	105656	1.17	1.08E-01	transcription_start_site	+	106086	106086	-607	3837 AN1025
2582	CONTIG15	105302	105656	1.17	1.08E-01	transcription_start_site	+	106175	106175	-696	3838 AN1025
2582	CONTIG15	105302	105656	1.17	1.08E-01	transcription_start_site	+	106759	106759	-1280	3839 AN1025
2582	CONTIG15	105302	105656	1.17	1.08E-01	transcription_start_site	+	108785	108785	-3306	3840 AN1025
2582	CONTIG15	105302	105656	1.17	1.08E-01	transcription_start_site	+	108937	108937	-3458	3841 AN1025
708	CONTIG30	69387	69651	2.19	5.20E-03	transcription_start_site	-	66934	66934	-2585	3846 AN10251
708	CONTIG30	69387	69651	2.19	5.20E-03	transcription_start_site	-	66860	66860	-2659	3847 AN10251
708	CONTIG30	69387	69651	2.19	5.20E-03	transcription_start_site	-	66569	66569	-2950	3848 AN10251
2199	CONTIG30	65707	65991	1.3	1.25E-01	transcription_start_site	-	66569	66569	720	3848 AN10251
2199	CONTIG30	65707	65991	1.3	1.25E-01	transcription_start_site	-	66860	66860	1011	3847 AN10251
2199	CONTIG30	65707	65991	1.3	1.25E-01	transcription_start_site	-	66934	66934	1085	3846 AN10251
1003	CONTIG30	14868	15355	1.91	1.57E-02	transcription_start_site	+	19108	19108	-3996	3852 AN10254
1003	CONTIG30	14868	15355	1.91	1.57E-02	transcription_start_site	+	19323	19323	-4211	3853 AN10254

1003	CONTIG30	14868	15355	1.91	1.57E-02	transcription_start_site	+	19497	19497	-4385	3854	AN10254
1003	CONTIG30	14868	15355	1.91	1.57E-02	transcription_start_site	+	20100	20100	-4988	3855	AN10254
1244	CONTIG34	86177	86461	1.73	1.57E-02	transcription_start_site	+	86622	86622	-303	3884	AN10265
1244	CONTIG34	86177	86461	1.73	1.57E-02	transcription_start_site	+	86762	86762	-443	3885	AN10265
1244	CONTIG34	86177	86461	1.73	1.57E-02	transcription_start_site	+	87490	87490	-1171	3886	AN10265
1244	CONTIG34	86177	86461	1.73	1.57E-02	transcription_start_site	+	87859	87859	-1540	3887	AN10265
2292	CONTIG34	137045	137394	1.27	9.24E-02	transcription_start_site	-	137858	137858	638	3896	AN10267
2292	CONTIG34	137045	137394	1.27	9.24E-02	transcription_start_site	-	137980	137980	760	3895	AN10267
1244	CONTIG34	86177	86461	1.73	1.57E-02	transcription_start_site	+	85623	85623	696	3900	AN10269
2051	CONTIG34	154276	154561	1.35	6.84E-02	transcription_start_site	-	149367	149367	-5051	3907	AN10272
798	CONTIG35	49357	49725	2.1	1.54E-03	transcription_start_site	-	45540	45540	-4001	3913	AN10274
798	CONTIG35	49357	49725	2.1	1.54E-03	transcription_start_site	-	45211	45211	-4330	3914	AN10274
798	CONTIG35	49357	49725	2.1	1.54E-03	transcription_start_site	-	44890	44890	-4651	3915	AN10274
798	CONTIG35	49357	49725	2.1	1.54E-03	transcription_start_site	-	44764	44764	-4777	3916	AN10274
798	CONTIG35	49357	49725	2.1	1.54E-03	transcription_start_site	-	44469	44469	-5072	3917	AN10274
2931	CONTIG35	47717	47988	0.99	1.93E-01	transcription_start_site	-	45540	45540	-2312	3913	AN10274
2931	CONTIG35	47717	47988	0.99	1.93E-01	transcription_start_site	-	45211	45211	-2641	3914	AN10274
2931	CONTIG35	47717	47988	0.99	1.93E-01	transcription_start_site	-	44890	44890	-2962	3915	AN10274
2931	CONTIG35	47717	47988	0.99	1.93E-01	transcription_start_site	-	44764	44764	-3088	3916	AN10274
2931	CONTIG35	47717	47988	0.99	1.93E-01	transcription_start_site	-	44469	44469	-3383	3917	AN10274
2931	CONTIG35	47717	47988	0.99	1.93E-01	transcription_start_site	-	44340	44340	-3512	3918	AN10274
2932	CONTIG35	48377	48956	0.99	1.93E-01	transcription_start_site	-	45540	45540	-3126	3913	AN10274
2932	CONTIG35	48377	48956	0.99	1.93E-01	transcription_start_site	-	45211	45211	-3455	3914	AN10274
2932	CONTIG35	48377	48956	0.99	1.93E-01	transcription_start_site	-	44890	44890	-3776	3915	AN10274
2932	CONTIG35	48377	48956	0.99	1.93E-01	transcription_start_site	-	44764	44764	-3902	3916	AN10274
2932	CONTIG35	48377	48956	0.99	1.93E-01	transcription_start_site	-	44469	44469	-4197	3917	AN10274
2932	CONTIG35	48377	48956	0.99	1.93E-01	transcription_start_site	-	44340	44340	-4326	3918	AN10274
2930	CONTIG35	41187	41986	0.99	1.93E-01	transcription_start_site	-	42161	42161	574	3921	AN10277
2930	CONTIG35	41187	41986	0.99	1.93E-01	transcription_start_site	-	42419	42419	832	3920	AN10277
2931	CONTIG35	47717	47988	0.99	1.93E-01	transcription_start_site	-	43452	43452	-4400	3919	AN10277
2932	CONTIG35	48377	48956	0.99	1.93E-01	transcription_start_site	-	43452	43452	-5214	3919	AN10277
2997	CONTIG15	119046	120220	0.92	1.47E-01	transcription_start_site	-	116987	116987	-2646	3927	AN1028
2997	CONTIG15	119046	120220	0.92	1.47E-01	transcription_start_site	-	116182	116182	-3451	3928	AN1028
2997	CONTIG15	119046	120220	0.92	1.47E-01	transcription_start_site	-	115844	115844	-3789	3929	AN1028
2997	CONTIG15	119046	120220	0.92	1.47E-01	transcription_start_site	-	114946	114946	-4687	3930	AN1028
2997	CONTIG15	119046	120220	0.92	1.47E-01	transcription_start_site	-	114765	114765	-4868	3931	AN1028
2293	CONTIG36	78093	78667	1.27	1.07E-01	transcription_start_site	+	78547	78547	-167	3932	AN10280
2293	CONTIG36	78093	78667	1.27	1.07E-01	transcription_start_site	+	78692	78692	-312	3933	AN10280
2293	CONTIG36	78093	78667	1.27	1.07E-01	transcription_start_site	-	78055	78055	-325	3934	AN10281
2293	CONTIG36	78093	78667	1.27	1.07E-01	transcription_start_site	-	77822	77822	-558	3935	AN10281
2293	CONTIG36	78093	78667	1.27	1.07E-01	transcription_start_site	-	77231	77231	-1149	3936	AN10281
2443	CONTIG36	80043	80387	1.22	1.44E-01	transcription_start_site	-	78055	78055	-2160	3934	AN10281
2443	CONTIG36	80043	80387	1.22	1.44E-01	transcription_start_site	-	77822	77822	-2393	3935	AN10281
2443	CONTIG36	80043	80387	1.22	1.44E-01	transcription_start_site	-	77231	77231	-2984	3936	AN10281
2293	CONTIG36	78093	78667	1.27	1.07E-01	transcription_start_site	-	75763	75763	-2617	3937	AN10282
2293	CONTIG36	78093	78667	1.27	1.07E-01	transcription_start_site	-	75686	75686	-2694	3938	AN10282
2293	CONTIG36	78093	78667	1.27	1.07E-01	transcription_start_site	-	75559	75559	-2821	3939	AN10282
2293	CONTIG36	78093	78667	1.27	1.07E-01	transcription_start_site	-	75358	75358	-3022	3940	AN10282
2293	CONTIG36	78093	78667	1.27	1.07E-01	transcription_start_site	-	75187	75187	-3193	3941	AN10282
2293	CONTIG36	78093	78667	1.27	1.07E-01	transcription_start_site	-	75067	75067	-3313	3942	AN10282
2293	CONTIG36	78093	78667	1.27	1.07E-01	transcription_start_site	-	74269	74269	-4111	3943	AN10282
2443	CONTIG36	80043	80387	1.22	1.44E-01	transcription_start_site	-	75763	75763	-4452	3937	AN10282
2443	CONTIG36	80043	80387	1.22	1.44E-01	transcription_start_site	-	75686	75686	-4529	3938	AN10282
2443	CONTIG36	80043	80387	1.22	1.44E-01	transcription_start_site	-	75559	75559	-4656	3939	AN10282
2443	CONTIG36	80043	80387	1.22	1.44E-01	transcription_start_site	-	75358	75358	-4857	3940	AN10282
2443	CONTIG36	80043	80387	1.22	1.44E-01	transcription_start_site	-	75187	75187	-5028	3941	AN10282
2443	CONTIG36	80043	80387	1.22	1.44E-01	transcription_start_site	-	75067	75067	-5148	3942	AN10282

428	CONTIG38	102527	102806	2.55	0.00E+00	transcription_start_site	-	101902	101902	-764	3947	AN10285
2103	CONTIG38	101417	101676	1.33	4.36E-02	transcription_start_site	-	101902	101902	355	3947	AN10285
2923	CONTIG38	157365	157709	0.99	1.66E-01	transcription_start_site	+	162308	162308	-4771	3948	AN10286
2923	CONTIG38	157365	157709	0.99	1.66E-01	transcription_start_site	+	162614	162614	-5077	3949	AN10286
1324	CONTIG38	194491	194755	1.67	8.81E-03	transcription_start_site	-	194502	194502	-121	3951	AN10287
1324	CONTIG38	194491	194755	1.67	8.81E-03	transcription_start_site	-	193910	193910	-713	3952	AN10287
1324	CONTIG38	194491	194755	1.67	8.81E-03	transcription_start_site	-	193579	193579	-1044	3953	AN10287
1324	CONTIG38	194491	194755	1.67	8.81E-03	transcription_start_site	+	197679	197679	-3056	3954	AN10288
1324	CONTIG38	194491	194755	1.67	8.81E-03	transcription_start_site	+	197918	197918	-3295	3955	AN10288
2211	CONTIG15	123840	124869	1.29	4.42E-02	transcription_start_site	-	120200	120200	-4154	3958	AN1029
2211	CONTIG15	123840	124869	1.29	4.42E-02	transcription_start_site	-	119772	119772	-4582	3959	AN1029
2211	CONTIG15	123840	124869	1.29	4.42E-02	transcription_start_site	-	119251	119251	-5103	3960	AN1029
2997	CONTIG15	119046	120220	0.92	1.47E-01	transcription_start_site	-	119772	119772	139	3959	AN1029
2997	CONTIG15	119046	120220	0.92	1.47E-01	transcription_start_site	-	119251	119251	-382	3960	AN1029
2997	CONTIG15	119046	120220	0.92	1.47E-01	transcription_start_site	-	120200	120200	567	3958	AN1029
2997	CONTIG15	119046	120220	0.92	1.47E-01	transcription_start_site	-	118617	118617	-1016	3961	AN1029
428	CONTIG38	102527	102806	2.55	0.00E+00	transcription_start_site	-	97550	97550	-5116	3962	AN10290
1805	CONTIG38	97132	97420	1.45	2.62E-02	transcription_start_site	-	97346	97346	70	3963	AN10290
1805	CONTIG38	97132	97420	1.45	2.62E-02	transcription_start_site	-	97550	97550	274	3962	AN10290
2103	CONTIG38	101417	101676	1.33	4.36E-02	transcription_start_site	-	97550	97550	-3996	3962	AN10290
2103	CONTIG38	101417	101676	1.33	4.36E-02	transcription_start_site	-	97346	97346	-4200	3963	AN10290
1052	CONTIG38	1511	1851	1.86	3.61E-03	transcription_start_site	+	571	571	1110	3965	AN10291
2923	CONTIG38	157365	157709	0.99	1.66E-01	transcription_start_site	+	158310	158310	-773	3966	AN10292
2923	CONTIG38	157365	157709	0.99	1.66E-01	transcription_start_site	+	158507	158507	-970	3967	AN10292
2923	CONTIG38	157365	157709	0.99	1.66E-01	transcription_start_site	+	158590	158590	-1053	3968	AN10292
2923	CONTIG38	157365	157709	0.99	1.66E-01	transcription_start_site	+	159965	159965	-2428	3969	AN10292
2923	CONTIG38	157365	157709	0.99	1.66E-01	transcription_start_site	+	161109	161109	-3572	3970	AN10292
1324	CONTIG38	194491	194755	1.67	8.81E-03	transcription_start_site	-	195180	195180	557	3973	AN10293
1324	CONTIG38	194491	194755	1.67	8.81E-03	transcription_start_site	+	199372	199372	-4749	3974	AN10294
1324	CONTIG38	194491	194755	1.67	8.81E-03	transcription_start_site	+	199555	199555	-4932	3975	AN10294
2213	CONTIG38	213096	213520	1.29	5.22E-02	transcription_start_site	+	213393	213393	-85	3977	AN10295
2213	CONTIG38	213096	213520	1.29	5.22E-02	transcription_start_site	+	212877	212877	431	3976	AN10295
2213	CONTIG38	213096	213520	1.29	5.22E-02	transcription_start_site	+	214298	214298	-990	3978	AN10295
2970	CONTIG38	213976	214262	0.95	1.93E-01	transcription_start_site	+	214298	214298	-179	3978	AN10295
2970	CONTIG38	213976	214262	0.95	1.93E-01	transcription_start_site	+	213393	213393	726	3977	AN10295
1406	CONTIG39	167103	167472	1.63	5.88E-02	transcription_start_site	-	164415	164415	-2872	3980	AN10297
1406	CONTIG39	167103	167472	1.63	5.88E-02	transcription_start_site	-	163305	163305	-3982	3981	AN10297
1406	CONTIG39	167103	167472	1.63	5.88E-02	transcription_start_site	-	162249	162249	-5038	3982	AN10297
2272	CONTIG39	21694	22033	1.28	1.66E-01	transcription_start_site	+	21824	21824	39	3991	AN10299
2272	CONTIG39	21694	22033	1.28	1.66E-01	transcription_start_site	+	21750	21750	113	3990	AN10299
2272	CONTIG39	21694	22033	1.28	1.66E-01	transcription_start_site	+	22832	22832	-968	3992	AN10299
2997	CONTIG15	119046	120220	0.92	1.47E-01	transcription_start_site	+	121571	121571	-1938	3993	AN1030
1869	CONTIG39	160284	160628	1.43	1.08E-01	transcription_start_site	-	160503	160503	47	3996	AN10300
1869	CONTIG39	160284	160628	1.43	1.08E-01	transcription_start_site	-	160600	160600	144	3995	AN10300
1869	CONTIG39	160284	160628	1.43	1.08E-01	transcription_start_site	-	160737	160737	281	3994	AN10300
1869	CONTIG39	160284	160628	1.43	1.08E-01	transcription_start_site	-	159856	159856	-600	3997	AN10300
1869	CONTIG39	160284	160628	1.43	1.08E-01	transcription_start_site	-	158974	158974	-1482	3998	AN10300
1498	CONTIG40	27161	27597	1.59	7.51E-02	transcription_start_site	-	27253	27253	-126	4018	AN10307
1498	CONTIG40	27161	27597	1.59	7.51E-02	transcription_start_site	-	26997	26997	-382	4019	AN10307
2211	CONTIG15	123840	124869	1.29	4.42E-02	transcription_start_site	-	124640	124640	285	4028	AN1031
2211	CONTIG15	123840	124869	1.29	4.42E-02	transcription_start_site	-	124022	124022	-332	4029	AN1031
2211	CONTIG15	123840	124869	1.29	4.42E-02	transcription_start_site	-	123817	123817	-537	4030	AN1031
2211	CONTIG15	123840	124869	1.29	4.42E-02	transcription_start_site	-	124967	124967	612	4027	AN1031
2211	CONTIG15	123840	124869	1.29	4.42E-02	transcription_start_site	-	125100	125100	745	4026	AN1031
2211	CONTIG15	123840	124869	1.29	4.42E-02	transcription_start_site	-	125412	125412	1057	4025	AN1031
2583	CONTIG15	125945	126279	1.17	1.08E-01	transcription_start_site	-	125412	125412	-700	4025	AN1031
2583	CONTIG15	125945	126279	1.17	1.08E-01	transcription_start_site	-	125100	125100	-1012	4026	AN1031

2583	CONTIG15	125945	126279	1.17	1.08E-01	transcription_start_site	-	124967	124967	-1145	4027	AN1031
2583	CONTIG15	125945	126279	1.17	1.08E-01	transcription_start_site	-	124640	124640	-1472	4028	AN1031
2583	CONTIG15	125945	126279	1.17	1.08E-01	transcription_start_site	-	124022	124022	-2090	4029	AN1031
2583	CONTIG15	125945	126279	1.17	1.08E-01	transcription_start_site	-	123817	123817	-2295	4030	AN1031
2996	CONTIG15	130285	131759	0.92	1.05E-01	transcription_start_site	-	125412	125412	-5610	4025	AN1031
1388	CONTIG40	142741	143020	1.64	8.01E-02	transcription_start_site	+	146168	146168	-3287	4031	AN10310
331	CONTIG41	391	730	2.72	4.61E-03	transcription_start_site	+	43	43	517	4037	AN10314
659	CONTIG43	49067	49333	2.23	1.80E-03	transcription_start_site	+	50588	50588	-1388	4044	AN10318
659	CONTIG43	49067	49333	2.23	1.80E-03	transcription_start_site	+	50962	50962	-1762	4045	AN10318
837	CONTIG43	49512	50016	2.06	4.61E-03	transcription_start_site	+	50588	50588	-824	4044	AN10318
837	CONTIG43	49512	50016	2.06	4.61E-03	transcription_start_site	+	50962	50962	-1198	4045	AN10318
2583	CONTIG15	125945	126279	1.17	1.08E-01	transcription_start_site	-	126690	126690	578	4051	AN1032
2996	CONTIG15	130285	131759	0.92	1.05E-01	transcription_start_site	-	126690	126690	-4332	4051	AN1032
1871	CONTIG43	209795	211194	1.42	2.86E-02	transcription_start_site	-	209109	209109	-1385	4053	AN10321
1871	CONTIG43	209795	211194	1.42	2.86E-02	transcription_start_site	-	209006	209006	-1488	4054	AN10321
1871	CONTIG43	209795	211194	1.42	2.86E-02	transcription_start_site	-	208426	208426	-2068	4055	AN10321
2617	CONTIG43	208294	208853	1.16	1.44E-01	transcription_start_site	-	208426	208426	-147	4055	AN10321
2617	CONTIG43	208294	208853	1.16	1.44E-01	transcription_start_site	-	209006	209006	432	4054	AN10321
2617	CONTIG43	208294	208853	1.16	1.44E-01	transcription_start_site	-	209109	209109	535	4053	AN10321
659	CONTIG43	49067	49333	2.23	1.80E-03	transcription_start_site	+	51373	51373	-2173	4058	AN10323
659	CONTIG43	49067	49333	2.23	1.80E-03	transcription_start_site	+	51758	51758	-2558	4059	AN10323
659	CONTIG43	49067	49333	2.23	1.80E-03	transcription_start_site	+	51880	51880	-2680	4060	AN10323
837	CONTIG43	49512	50016	2.06	4.61E-03	transcription_start_site	+	51373	51373	-1609	4058	AN10323
837	CONTIG43	49512	50016	2.06	4.61E-03	transcription_start_site	+	51758	51758	-1994	4059	AN10323
837	CONTIG43	49512	50016	2.06	4.61E-03	transcription_start_site	+	51880	51880	-2116	4060	AN10323
1876	CONTIG43	11416	11685	1.42	5.88E-02	transcription_start_site	+	16015	16015	-4464	4061	AN10324
1876	CONTIG43	11416	11685	1.42	5.88E-02	transcription_start_site	+	16118	16118	-4567	4062	AN10324
1876	CONTIG43	11416	11685	1.42	5.88E-02	transcription_start_site	+	16286	16286	-4735	4063	AN10324
1876	CONTIG43	11416	11685	1.42	5.88E-02	transcription_start_site	+	16677	16677	-5126	4064	AN10324
655	CONTIG43	115519	115853	2.23	0.00E+00	transcription_start_site	+	120141	120141	-4455	4065	AN10325
1871	CONTIG43	209795	211194	1.42	2.86E-02	transcription_start_site	-	210497	210497	2	4069	AN10327
1871	CONTIG43	209795	211194	1.42	2.86E-02	transcription_start_site	-	211265	211265	770	4068	AN10327
1150	CONTIG45	43293	43642	1.79	2.16E-04	transcription_start_site	+	44020	44020	-552	4070	AN10329
2428	CONTIG45	40055	40344	1.22	1.57E-02	transcription_start_site	+	44020	44020	-3820	4070	AN10329
2739	CONTIG45	41180	41614	1.1	3.08E-02	transcription_start_site	+	44020	44020	-2623	4070	AN10329
2211	CONTIG15	123840	124869	1.29	4.42E-02	transcription_start_site	+	127296	127296	-2941	4071	AN1033
2211	CONTIG15	123840	124869	1.29	4.42E-02	transcription_start_site	+	127868	127868	-3513	4072	AN1033
2211	CONTIG15	123840	124869	1.29	4.42E-02	transcription_start_site	+	128139	128139	-3784	4073	AN1033
2583	CONTIG15	125945	126279	1.17	1.08E-01	transcription_start_site	+	127296	127296	-1184	4071	AN1033
2583	CONTIG15	125945	126279	1.17	1.08E-01	transcription_start_site	+	127868	127868	-1756	4072	AN1033
2583	CONTIG15	125945	126279	1.17	1.08E-01	transcription_start_site	+	128139	128139	-2027	4073	AN1033
1150	CONTIG45	43293	43642	1.79	2.16E-04	transcription_start_site	+	45428	45428	-1960	4074	AN10330
1150	CONTIG45	43293	43642	1.79	2.16E-04	transcription_start_site	+	45578	45578	-2110	4075	AN10330
1150	CONTIG45	43293	43642	1.79	2.16E-04	transcription_start_site	+	45700	45700	-2232	4076	AN10330
1150	CONTIG45	43293	43642	1.79	2.16E-04	transcription_start_site	+	45924	45924	-2456	4077	AN10330
1150	CONTIG45	43293	43642	1.79	2.16E-04	transcription_start_site	+	46085	46085	-2617	4078	AN10330
2739	CONTIG45	41180	41614	1.1	3.08E-02	transcription_start_site	+	45428	45428	-4031	4074	AN10330
2739	CONTIG45	41180	41614	1.1	3.08E-02	transcription_start_site	+	45578	45578	-4181	4075	AN10330
2739	CONTIG45	41180	41614	1.1	3.08E-02	transcription_start_site	+	45700	45700	-4303	4076	AN10330
2739	CONTIG45	41180	41614	1.1	3.08E-02	transcription_start_site	+	45924	45924	-4527	4077	AN10330
2739	CONTIG45	41180	41614	1.1	3.08E-02	transcription_start_site	+	46085	46085	-4688	4078	AN10330
624	CONTIG46	13652	14011	2.28	3.61E-03	transcription_start_site	+	15572	15572	-1740	4079	AN10331
624	CONTIG46	13652	14011	2.28	3.61E-03	transcription_start_site	+	15818	15818	-1986	4080	AN10331
624	CONTIG46	13652	14011	2.28	3.61E-03	transcription_start_site	+	16646	16646	-2814	4081	AN10331
624	CONTIG46	13652	14011	2.28	3.61E-03	transcription_start_site	+	16759	16759	-2927	4082	AN10331
624	CONTIG46	13652	14011	2.28	3.61E-03	transcription_start_site	+	17007	17007	-3175	4083	AN10331
624	CONTIG46	13652	14011	2.28	3.61E-03	transcription_start_site	+	17228	17228	-3396	4084	AN10331

624	CONTIG46	13652	14011	2.28	3.61E-03	transcription_start_site	+	17425	17425	-3593	4085	AN10331
624	CONTIG46	13652	14011	2.28	3.61E-03	transcription_start_site	+	13784	13784	47	4086	AN10332
624	CONTIG46	13652	14011	2.28	3.61E-03	transcription_start_site	+	14296	14296	-464	4087	AN10332
624	CONTIG46	13652	14011	2.28	3.61E-03	transcription_start_site	+	15103	15103	-1271	4088	AN10332
703	CONTIG46	8701	9050	2.19	2.32E-03	transcription_start_site	+	13784	13784	-4908	4086	AN10332
1727	CONTIG46	9696	9970	1.49	6.84E-02	transcription_start_site	+	13784	13784	-3951	4086	AN10332
1727	CONTIG46	9696	9970	1.49	6.84E-02	transcription_start_site	+	14296	14296	-4463	4087	AN10332
2498	CONTIG47	70353	71152	1.2	4.42E-02	transcription_start_site	+	70139	70139	613	4090	AN10334
2253	CONTIG49	50188	50677	1.28	6.84E-02	transcription_start_site	-	50725	50725	292	4093	AN10335
2253	CONTIG49	50188	50677	1.28	6.84E-02	transcription_start_site	-	51024	51024	591	4092	AN10335
2253	CONTIG49	50188	50677	1.28	6.84E-02	transcription_start_site	-	51138	51138	705	4091	AN10335
2780	CONTIG49	70518	70927	1.08	1.44E-01	transcription_start_site	-	70566	70566	-156	4094	AN10336
2780	CONTIG49	70518	70927	1.08	1.44E-01	transcription_start_site	-	70472	70472	-250	4095	AN10336
2253	CONTIG49	50188	50677	1.28	6.84E-02	transcription_start_site	-	49330	49330	-1102	4099	AN10338
2253	CONTIG49	50188	50677	1.28	6.84E-02	transcription_start_site	-	48953	48953	-1479	4100	AN10338
2253	CONTIG49	50188	50677	1.28	6.84E-02	transcription_start_site	-	48677	48677	-1755	4101	AN10338
2780	CONTIG49	70518	70927	1.08	1.44E-01	transcription_start_site	-	68742	68742	-1980	4102	AN10339
2780	CONTIG49	70518	70927	1.08	1.44E-01	transcription_start_site	-	68622	68622	-2100	4103	AN10339
2211	CONTIG15	123840	124869	1.29	4.42E-02	transcription_start_site	+	129597	129597	-5242	4104	AN1034
2471	CONTIG15	135310	136179	1.21	7.51E-02	transcription_start_site	+	138281	138281	-2536	4105	AN1034
2471	CONTIG15	135310	136179	1.21	7.51E-02	transcription_start_site	+	138463	138463	-2718	4106	AN1034
2579	CONTIG15	132910	133639	1.17	9.26E-02	transcription_start_site	+	138281	138281	-5006	4105	AN1034
2579	CONTIG15	132910	133639	1.17	9.26E-02	transcription_start_site	+	138463	138463	-5188	4106	AN1034
2583	CONTIG15	125945	126279	1.17	1.08E-01	transcription_start_site	+	129597	129597	-3485	4104	AN1034
2846	CONTIG15	136895	137990	1.04	1.66E-01	transcription_start_site	+	138281	138281	-838	4105	AN1034
2846	CONTIG15	136895	137990	1.04	1.66E-01	transcription_start_site	+	138463	138463	-1020	4106	AN1034
2996	CONTIG15	130285	131759	0.92	1.05E-01	transcription_start_site	+	129597	129597	1425	4104	AN1034
2369	CONTIG49	92719	93283	1.24	8.01E-02	transcription_start_site	+	96152	96152	-3151	4111	AN10342
1481	CONTIG50	18152	18431	1.6	8.01E-02	transcription_start_site	+	18787	18787	-495	4114	AN10343
1481	CONTIG50	18152	18431	1.6	8.01E-02	transcription_start_site	+	18938	18938	-646	4115	AN10343
1481	CONTIG50	18152	18431	1.6	8.01E-02	transcription_start_site	+	17613	17613	678	4113	AN10343
1481	CONTIG50	18152	18431	1.6	8.01E-02	transcription_start_site	+	17382	17382	909	4112	AN10343
1481	CONTIG50	18152	18431	1.6	8.01E-02	transcription_start_site	+	20288	20288	-1996	4116	AN10344
1481	CONTIG50	18152	18431	1.6	8.01E-02	transcription_start_site	+	20435	20435	-2143	4117	AN10344
1481	CONTIG50	18152	18431	1.6	8.01E-02	transcription_start_site	+	20597	20597	-2305	4118	AN10344
1481	CONTIG50	18152	18431	1.6	8.01E-02	transcription_start_site	+	20993	20993	-2701	4119	AN10344
1481	CONTIG50	18152	18431	1.6	8.01E-02	transcription_start_site	+	21505	21505	-3213	4120	AN10344
1481	CONTIG50	18152	18431	1.6	8.01E-02	transcription_start_site	+	21642	21642	-3350	4121	AN10344
1654	CONTIG51	23344	23611	1.52	8.01E-02	transcription_start_site	+	22834	22834	643	4125	AN10345
1654	CONTIG51	23344	23611	1.52	8.01E-02	transcription_start_site	+	22513	22513	964	4124	AN10345
2471	CONTIG15	135310	136179	1.21	7.51E-02	transcription_start_site	+	139055	139055	-3310	4136	AN1035
2471	CONTIG15	135310	136179	1.21	7.51E-02	transcription_start_site	+	139374	139374	-3629	4137	AN1035
2471	CONTIG15	135310	136179	1.21	7.51E-02	transcription_start_site	+	139638	139638	-3893	4138	AN1035
2846	CONTIG15	136895	137990	1.04	1.66E-01	transcription_start_site	+	139055	139055	-1612	4136	AN1035
2846	CONTIG15	136895	137990	1.04	1.66E-01	transcription_start_site	+	139374	139374	-1931	4137	AN1035
2846	CONTIG15	136895	137990	1.04	1.66E-01	transcription_start_site	+	139638	139638	-2195	4138	AN1035
1436	CONTIG51	399902	400181	1.62	5.88E-02	transcription_start_site	-	399365	399365	-676	4139	AN10350
1436	CONTIG51	399902	400181	1.62	5.88E-02	transcription_start_site	-	399221	399221	-820	4140	AN10350
1436	CONTIG51	399902	400181	1.62	5.88E-02	transcription_start_site	-	398931	398931	-1110	4141	AN10350
1436	CONTIG51	399902	400181	1.62	5.88E-02	transcription_start_site	-	398574	398574	-1467	4142	AN10350
1436	CONTIG51	399902	400181	1.62	5.88E-02	transcription_start_site	-	397882	397882	-2159	4143	AN10350
1436	CONTIG51	399902	400181	1.62	5.88E-02	transcription_start_site	-	397738	397738	-2303	4144	AN10350
1436	CONTIG51	399902	400181	1.62	5.88E-02	transcription_start_site	-	396550	396550	-3491	4145	AN10350
2020	CONTIG51	753760	754114	1.37	1.25E-01	transcription_start_site	-	754879	754879	942	4154	AN10352
108	CONTIG51	954903	955192	3.19	0.00E+00	transcription_start_site	+	958477	958477	-3429	4157	AN10354
108	CONTIG51	954903	955192	3.19	0.00E+00	transcription_start_site	+	958675	958675	-3627	4158	AN10354
108	CONTIG51	954903	955192	3.19	0.00E+00	transcription_start_site	+	958937	958937	-3889	4159	AN10354

154	CONTIG51	967728	968092	3.04	2.67E-04	transcription_start_site	-	967007	967007	-903	4160	AN10355
531	CONTIG51	966678	967112	2.4	4.46E-04	transcription_start_site	-	967007	967007	112	4160	AN10355
574	CONTIG51	1032303	1032577	2.35	4.61E-03	transcription_start_site	-	1030316	1030316	-2124	4165	AN10357
574	CONTIG51	1032303	1032577	2.35	4.61E-03	transcription_start_site	-	1030096	1030096	-2344	4166	AN10357
574	CONTIG51	1032303	1032577	2.35	4.61E-03	transcription_start_site	-	1029910	1029910	-2530	4167	AN10357
574	CONTIG51	1032303	1032577	2.35	4.61E-03	transcription_start_site	-	1029466	1029466	-2974	4168	AN10357
574	CONTIG51	1032303	1032577	2.35	4.61E-03	transcription_start_site	-	1029338	1029338	-3102	4169	AN10357
1528	CONTIG51	1030888	1031977	1.57	5.05E-02	transcription_start_site	-	1030316	1030316	-1116	4165	AN10357
1528	CONTIG51	1030888	1031977	1.57	5.05E-02	transcription_start_site	-	1030096	1030096	-1336	4166	AN10357
1528	CONTIG51	1030888	1031977	1.57	5.05E-02	transcription_start_site	-	1029910	1029910	-1522	4167	AN10357
1528	CONTIG51	1030888	1031977	1.57	5.05E-02	transcription_start_site	-	1029466	1029466	-1966	4168	AN10357
1528	CONTIG51	1030888	1031977	1.57	5.05E-02	transcription_start_site	-	1029338	1029338	-2094	4169	AN10357
1654	CONTIG51	23344	23611	1.52	8.01E-02	transcription_start_site	+	23683	23683	-205	4170	AN10358
1654	CONTIG51	23344	23611	1.52	8.01E-02	transcription_start_site	+	23868	23868	-390	4171	AN10358
1654	CONTIG51	23344	23611	1.52	8.01E-02	transcription_start_site	+	24220	24220	-742	4172	AN10358
1654	CONTIG51	23344	23611	1.52	8.01E-02	transcription_start_site	+	24444	24444	-966	4173	AN10358
522	CONTIG15	154220	154494	2.41	2.16E-04	transcription_start_site	-	150464	150464	-3893	4176	AN1036
522	CONTIG15	154220	154494	2.41	2.16E-04	transcription_start_site	-	150023	150023	-4334	4177	AN1036
522	CONTIG15	154220	154494	2.41	2.16E-04	transcription_start_site	-	149923	149923	-4434	4178	AN1036
1366	CONTIG15	152195	152529	1.65	1.57E-02	transcription_start_site	-	150464	150464	-1898	4176	AN1036
1366	CONTIG15	152195	152529	1.65	1.57E-02	transcription_start_site	-	150023	150023	-2339	4177	AN1036
1366	CONTIG15	152195	152529	1.65	1.57E-02	transcription_start_site	-	149923	149923	-2439	4178	AN1036
1895	CONTIG15	146866	147200	1.41	4.36E-02	transcription_start_site	-	144501	144501	-2532	4179	AN1036
1895	CONTIG15	146866	147200	1.41	4.36E-02	transcription_start_site	-	143927	143927	-3106	4180	AN1036
2776	CONTIG15	145051	146765	1.08	1.01E-01	transcription_start_site	-	144501	144501	-1407	4179	AN1036
2776	CONTIG15	145051	146765	1.08	1.01E-01	transcription_start_site	-	143927	143927	-1981	4180	AN1036
2844	CONTIG15	147606	148705	1.04	1.59E-01	transcription_start_site	-	144501	144501	-3654	4179	AN1036
2844	CONTIG15	147606	148705	1.04	1.59E-01	transcription_start_site	-	143927	143927	-4228	4180	AN1036
1436	CONTIG51	399902	400181	1.62	5.88E-02	transcription_start_site	-	395646	395646	-4395	4181	AN10360
1436	CONTIG51	399902	400181	1.62	5.88E-02	transcription_start_site	-	395449	395449	-4592	4182	AN10360
2146	CONTIG51	392337	392623	1.32	1.44E-01	transcription_start_site	-	392334	392334	-146	4187	AN10362
2146	CONTIG51	392337	392623	1.32	1.44E-01	transcription_start_site	-	392040	392040	-440	4188	AN10362
2146	CONTIG51	392337	392623	1.32	1.44E-01	transcription_start_site	-	391818	391818	-662	4189	AN10362
2020	CONTIG51	753760	754114	1.37	1.25E-01	transcription_start_site	-	754153	754153	216	4192	AN10364
108	CONTIG51	954903	955192	3.19	0.00E+00	transcription_start_site	+	959435	959435	-4387	4193	AN10365
108	CONTIG51	954903	955192	3.19	0.00E+00	transcription_start_site	+	959720	959720	-4672	4194	AN10365
574	CONTIG51	1032303	1032577	2.35	4.61E-03	transcription_start_site	-	1028178	1028178	-4262	4196	AN10367
1528	CONTIG51	1030888	1031977	1.57	5.05E-02	transcription_start_site	-	1028178	1028178	-3254	4196	AN10367
338	CONTIG51	566044	566313	2.7	1.01E-03	transcription_start_site	+	566466	566466	-287	4201	AN10369
338	CONTIG51	566044	566313	2.7	1.01E-03	transcription_start_site	+	566714	566714	-535	4202	AN10369
338	CONTIG51	566044	566313	2.7	1.01E-03	transcription_start_site	+	566956	566956	-777	4203	AN10369
338	CONTIG51	566044	566313	2.7	1.01E-03	transcription_start_site	+	567309	567309	-1130	4204	AN10369
338	CONTIG51	566044	566313	2.7	1.01E-03	transcription_start_site	+	567438	567438	-1259	4205	AN10369
496	CONTIG51	563029	563468	2.45	0.00E+00	transcription_start_site	+	566466	566466	-3217	4201	AN10369
496	CONTIG51	563029	563468	2.45	0.00E+00	transcription_start_site	+	566714	566714	-3465	4202	AN10369
496	CONTIG51	563029	563468	2.45	0.00E+00	transcription_start_site	+	566956	566956	-3707	4203	AN10369
496	CONTIG51	563029	563468	2.45	0.00E+00	transcription_start_site	+	567309	567309	-4060	4204	AN10369
496	CONTIG51	563029	563468	2.45	0.00E+00	transcription_start_site	+	567438	567438	-4189	4205	AN10369
522	CONTIG15	154220	154494	2.41	2.16E-04	transcription_start_site	-	153367	153367	-990	4206	AN1037
1242	CONTIG15	155570	156054	1.73	1.11E-02	transcription_start_site	-	153367	153367	-2445	4206	AN1037
1366	CONTIG15	152195	152529	1.65	1.57E-02	transcription_start_site	-	153367	153367	1005	4206	AN1037
2425	CONTIG51	466443	466779	1.23	1.93E-01	transcription_start_site	-	463167	463167	-3444	4207	AN10370
2425	CONTIG51	466443	466779	1.23	1.93E-01	transcription_start_site	-	462775	462775	-3836	4208	AN10370
2425	CONTIG51	466443	466779	1.23	1.93E-01	transcription_start_site	-	462598	462598	-4013	4209	AN10370
154	CONTIG51	967728	968092	3.04	2.67E-04	transcription_start_site	-	964030	964030	-3880	4222	AN10377
154	CONTIG51	967728	968092	3.04	2.67E-04	transcription_start_site	-	963711	963711	-4199	4223	AN10377
531	CONTIG51	966678	967112	2.4	4.46E-04	transcription_start_site	-	964030	964030	-2865	4222	AN10377

531	CONTIG51	966678	967112	2.4	4.46E-04	transcription_start_site	-	963711	963711	-3184	4223	AN10377
684	CONTIG51	964063	964627	2.21	3.31E-03	transcription_start_site	-	964030	964030	-315	4222	AN10377
684	CONTIG51	964063	964627	2.21	3.31E-03	transcription_start_site	-	963711	963711	-634	4223	AN10377
1065	CONTIG52	49128	49492	1.86	6.84E-02	transcription_start_site	+	49797	49797	-487	4224	AN10378
1065	CONTIG52	49128	49492	1.86	6.84E-02	transcription_start_site	+	50101	50101	-791	4225	AN10378
1065	CONTIG52	49128	49492	1.86	6.84E-02	transcription_start_site	+	50257	50257	-947	4226	AN10378
1065	CONTIG52	49128	49492	1.86	6.84E-02	transcription_start_site	+	50587	50587	-1277	4227	AN10378
392	CONTIG52	65412	65686	2.62	7.85E-03	transcription_start_site	-	62947	62947	-2602	4228	AN10379
392	CONTIG52	65412	65686	2.62	7.85E-03	transcription_start_site	-	62479	62479	-3070	4229	AN10379
392	CONTIG52	65412	65686	2.62	7.85E-03	transcription_start_site	-	61945	61945	-3604	4230	AN10379
1310	CONTIG52	62857	63141	1.69	1.08E-01	transcription_start_site	-	62947	62947	-52	4228	AN10379
1310	CONTIG52	62857	63141	1.69	1.08E-01	transcription_start_site	-	62479	62479	-520	4229	AN10379
1310	CONTIG52	62857	63141	1.69	1.08E-01	transcription_start_site	-	61945	61945	-1054	4230	AN10379
1242	CONTIG15	155570	156054	1.73	1.11E-02	transcription_start_site	-	156330	156330	518	4234	AN1038
1065	CONTIG52	49128	49492	1.86	6.84E-02	transcription_start_site	+	52124	52124	-2814	4235	AN10380
1065	CONTIG52	49128	49492	1.86	6.84E-02	transcription_start_site	+	52290	52290	-2980	4236	AN10380
1065	CONTIG52	49128	49492	1.86	6.84E-02	transcription_start_site	+	52407	52407	-3097	4237	AN10380
1065	CONTIG52	49128	49492	1.86	6.84E-02	transcription_start_site	+	52599	52599	-3289	4238	AN10380
1065	CONTIG52	49128	49492	1.86	6.84E-02	transcription_start_site	+	53497	53497	-4187	4239	AN10380
392	CONTIG52	65412	65686	2.62	7.85E-03	transcription_start_site	-	61583	61583	-3966	4240	AN10381
1310	CONTIG52	62857	63141	1.69	1.08E-01	transcription_start_site	-	61583	61583	-1416	4240	AN10381
375	CONTIG53	41496	41920	2.65	3.03E-03	transcription_start_site	-	37331	37331	-4377	4251	AN10386
375	CONTIG53	41496	41920	2.65	3.03E-03	transcription_start_site	-	36919	36919	-4789	4252	AN10386
375	CONTIG53	41496	41920	2.65	3.03E-03	transcription_start_site	-	36703	36703	-5005	4253	AN10386
175	CONTIG55	8106	8395	2.99	0.00E+00	transcription_start_site	-	8089	8089	-161	4263	AN10389
175	CONTIG55	8106	8395	2.99	0.00E+00	transcription_start_site	-	7807	7807	-443	4264	AN10389
175	CONTIG55	8106	8395	2.99	0.00E+00	transcription_start_site	-	7528	7528	-722	4265	AN10389
175	CONTIG55	8106	8395	2.99	0.00E+00	transcription_start_site	-	7399	7399	-851	4266	AN10389
175	CONTIG55	8106	8395	2.99	0.00E+00	transcription_start_site	-	6719	6719	-1531	4267	AN10389
1910	CONTIG55	8862	9506	1.4	3.08E-02	transcription_start_site	-	8089	8089	-1095	4263	AN10389
1910	CONTIG55	8862	9506	1.4	3.08E-02	transcription_start_site	-	7807	7807	-1377	4264	AN10389
1910	CONTIG55	8862	9506	1.4	3.08E-02	transcription_start_site	-	7528	7528	-1656	4265	AN10389
1910	CONTIG55	8862	9506	1.4	3.08E-02	transcription_start_site	-	7399	7399	-1785	4266	AN10389
1910	CONTIG55	8862	9506	1.4	3.08E-02	transcription_start_site	-	6719	6719	-2465	4267	AN10389
2104	CONTIG55	7201	7775	1.33	4.36E-02	transcription_start_site	-	7528	7528	40	4265	AN10389
2104	CONTIG55	7201	7775	1.33	4.36E-02	transcription_start_site	-	7399	7399	-89	4266	AN10389
2104	CONTIG55	7201	7775	1.33	4.36E-02	transcription_start_site	-	7807	7807	319	4264	AN10389
2104	CONTIG55	7201	7775	1.33	4.36E-02	transcription_start_site	-	8089	8089	601	4263	AN10389
2104	CONTIG55	7201	7775	1.33	4.36E-02	transcription_start_site	-	6719	6719	-769	4267	AN10389
2941	CONTIG55	137327	137616	0.98	1.66E-01	transcription_start_site	+	141707	141707	-4235	4275	AN10391
2941	CONTIG55	137327	137616	0.98	1.66E-01	transcription_start_site	+	142405	142405	-4933	4276	AN10391
433	CONTIG55	264692	264957	2.54	0.00E+00	transcription_start_site	+	264241	264241	583	4279	AN10392
433	CONTIG55	264692	264957	2.54	0.00E+00	transcription_start_site	+	265119	265119	-294	4296	AN10399
433	CONTIG55	264692	264957	2.54	0.00E+00	transcription_start_site	+	265656	265656	-831	4297	AN10399
433	CONTIG55	264692	264957	2.54	0.00E+00	transcription_start_site	+	266071	266071	-1246	4298	AN10399
175	CONTIG55	8106	8395	2.99	0.00E+00	transcription_start_site	-	8455	8455	204	4311	AN10402
175	CONTIG55	8106	8395	2.99	0.00E+00	transcription_start_site	-	8736	8736	485	4310	AN10402
175	CONTIG55	8106	8395	2.99	0.00E+00	transcription_start_site	-	9004	9004	753	4309	AN10402
1910	CONTIG55	8862	9506	1.4	3.08E-02	transcription_start_site	-	9004	9004	-180	4309	AN10402
1910	CONTIG55	8862	9506	1.4	3.08E-02	transcription_start_site	-	8736	8736	-448	4310	AN10402
1910	CONTIG55	8862	9506	1.4	3.08E-02	transcription_start_site	-	8455	8455	-729	4311	AN10402
1910	CONTIG55	8862	9506	1.4	3.08E-02	transcription_start_site	-	10094	10094	910	4308	AN10402
2104	CONTIG55	7201	7775	1.33	4.36E-02	transcription_start_site	-	8455	8455	967	4311	AN10402
2104	CONTIG55	7201	7775	1.33	4.36E-02	transcription_start_site	-	8736	8736	1248	4310	AN10402
2972	CONTIG55	54093	54437	0.95	1.93E-01	transcription_start_site	-	49776	49776	-4489	4312	AN10403
1361	CONTIG56	22735	23011	1.66	9.24E-02	transcription_start_site	+	23141	23141	-268	4318	AN10407
1361	CONTIG56	22735	23011	1.66	9.24E-02	transcription_start_site	+	23466	23466	-593	4319	AN10407

1361	CONTIG56	22735	23011	1.66	9.24E-02	transcription_start_site	+	24302	24302	-1429	4320	AN10407
1361	CONTIG56	22735	23011	1.66	9.24E-02	transcription_start_site	+	24567	24567	-1694	4321	AN10407
1362	CONTIG15	171379	172038	1.65	7.16E-03	transcription_start_site	+	171244	171244	464	4328	AN1041
1362	CONTIG15	171379	172038	1.65	7.16E-03	transcription_start_site	+	170873	170873	835	4327	AN1041
477	CONTIG57	24620	25042	2.48	1.11E-02	transcription_start_site	-	20517	20517	-4314	4329	AN10410
477	CONTIG57	24620	25042	2.48	1.11E-02	transcription_start_site	-	20153	20153	-4678	4330	AN10410
3048	CONTIG58	6376	6823	0.77	1.66E-01	transcription_start_site	-	6447	6447	-152	4336	AN10413
1737	CONTIG58	15996	16400	1.48	3.03E-03	transcription_start_site	-	12926	12926	-3272	4337	AN10414
1737	CONTIG58	15996	16400	1.48	3.03E-03	transcription_start_site	-	12521	12521	-3677	4338	AN10414
1737	CONTIG58	15996	16400	1.48	3.03E-03	transcription_start_site	-	12299	12299	-3899	4339	AN10414
1737	CONTIG58	15996	16400	1.48	3.03E-03	transcription_start_site	-	11576	11576	-4622	4340	AN10414
3048	CONTIG58	6376	6823	0.77	1.66E-01	transcription_start_site	-	7745	7745	1145	4366	AN10418
2128	CONTIG59	127295	127714	1.32	2.62E-02	transcription_start_site	+	127031	127031	473	4367	AN10419
1362	CONTIG15	171379	172038	1.65	7.16E-03	transcription_start_site	-	172522	172522	813	4369	AN1042
1477	CONTIG59	141757	142266	1.6	6.61E-03	transcription_start_site	+	142710	142710	-698	4370	AN10420
1477	CONTIG59	141757	142266	1.6	6.61E-03	transcription_start_site	+	142826	142826	-814	4371	AN10420
1477	CONTIG59	141757	142266	1.6	6.61E-03	transcription_start_site	+	143293	143293	-1281	4372	AN10420
1611	CONTIG59	129530	130344	1.53	1.17E-03	transcription_start_site	+	129103	129103	834	4377	AN10421
1611	CONTIG59	129530	130344	1.53	1.17E-03	transcription_start_site	+	128706	128706	1231	4376	AN10421
2128	CONTIG59	127295	127714	1.32	2.62E-02	transcription_start_site	+	128226	128226	-721	4373	AN10421
2128	CONTIG59	127295	127714	1.32	2.62E-02	transcription_start_site	+	128446	128446	-941	4374	AN10421
2128	CONTIG59	127295	127714	1.32	2.62E-02	transcription_start_site	+	128525	128525	-1020	4375	AN10421
2128	CONTIG59	127295	127714	1.32	2.62E-02	transcription_start_site	+	128706	128706	-1201	4376	AN10421
2128	CONTIG59	127295	127714	1.32	2.62E-02	transcription_start_site	+	129103	129103	-1598	4377	AN10421
2946	CONTIG59	128345	128829	0.97	1.07E-01	transcription_start_site	+	128525	128525	62	4375	AN10421
2946	CONTIG59	128345	128829	0.97	1.07E-01	transcription_start_site	+	128706	128706	-119	4376	AN10421
2946	CONTIG59	128345	128829	0.97	1.07E-01	transcription_start_site	+	128446	128446	141	4374	AN10421
2946	CONTIG59	128345	128829	0.97	1.07E-01	transcription_start_site	+	128226	128226	361	4373	AN10421
2946	CONTIG59	128345	128829	0.97	1.07E-01	transcription_start_site	+	129103	129103	-516	4377	AN10421
996	CONTIG59	130970	131229	1.91	1.01E-03	transcription_start_site	+	130052	130052	1047	4378	AN10422
1611	CONTIG59	129530	130344	1.53	1.17E-03	transcription_start_site	+	130052	130052	-115	4378	AN10422
2128	CONTIG59	127295	127714	1.32	2.62E-02	transcription_start_site	+	130052	130052	-2547	4378	AN10422
2946	CONTIG59	128345	128829	0.97	1.07E-01	transcription_start_site	+	130052	130052	-1465	4378	AN10422
1477	CONTIG59	141757	142266	1.6	6.61E-03	transcription_start_site	+	144996	144996	-2984	4379	AN10423
1477	CONTIG59	141757	142266	1.6	6.61E-03	transcription_start_site	+	145788	145788	-3776	4380	AN10423
1477	CONTIG59	141757	142266	1.6	6.61E-03	transcription_start_site	+	146291	146291	-4279	4381	AN10423
2994	CONTIG60	56626	57070	0.92	6.84E-02	transcription_start_site	+	58382	58382	-1534	4396	AN10427
2994	CONTIG60	56626	57070	0.92	6.84E-02	transcription_start_site	+	58858	58858	-2010	4397	AN10427
1023	CONTIG61	174688	175252	1.89	1.30E-02	transcription_start_site	+	179813	179813	-4843	4404	AN10429
1023	CONTIG61	174688	175252	1.89	1.30E-02	transcription_start_site	+	180001	180001	-5031	4405	AN10429
1023	CONTIG61	174688	175252	1.89	1.30E-02	transcription_start_site	+	180158	180158	-5188	4406	AN10429
1362	CONTIG15	171379	172038	1.65	7.16E-03	transcription_start_site	+	173975	173975	-2266	4409	AN1043
1362	CONTIG15	171379	172038	1.65	7.16E-03	transcription_start_site	+	174329	174329	-2620	4410	AN1043
1362	CONTIG15	171379	172038	1.65	7.16E-03	transcription_start_site	+	174846	174846	-3137	4411	AN1043
1362	CONTIG15	171379	172038	1.65	7.16E-03	transcription_start_site	+	175185	175185	-3476	4412	AN1043
1362	CONTIG15	171379	172038	1.65	7.16E-03	transcription_start_site	+	175425	175425	-3716	4413	AN1043
1724	CONTIG61	429989	430259	1.49	5.88E-02	transcription_start_site	-	430117	430117	-7	4434	AN10436
1724	CONTIG61	429989	430259	1.49	5.88E-02	transcription_start_site	-	429368	429368	-756	4435	AN10436
1724	CONTIG61	429989	430259	1.49	5.88E-02	transcription_start_site	-	428680	428680	-1444	4436	AN10436
2693	CONTIG61	427519	427870	1.13	1.93E-01	transcription_start_site	-	428680	428680	985	4436	AN10436
2440	CONTIG61	496952	497601	1.22	1.28E-01	transcription_start_site	-	496674	496674	-602	4441	AN10438
2440	CONTIG61	496952	497601	1.22	1.28E-01	transcription_start_site	-	496201	496201	-1075	4442	AN10438
1139	CONTIG61	549456	549810	1.8	1.84E-02	transcription_start_site	-	547675	547675	-1958	4457	AN10441
1139	CONTIG61	549456	549810	1.8	1.84E-02	transcription_start_site	-	547299	547299	-2334	4458	AN10441
1200	CONTIG61	651680	651954	1.76	2.12E-02	transcription_start_site	+	652367	652367	-550	4459	AN10442
1725	CONTIG61	650045	650389	1.49	5.88E-02	transcription_start_site	+	652367	652367	-2150	4459	AN10442
1274	CONTIG61	718432	718711	1.71	2.62E-02	transcription_start_site	+	720776	720776	-2204	4469	AN10445

1274	CONTIG61	718432	718711	1.71	2.62E-02	transcription_start_site	+	720927	720927	-2355	4470	AN10445
1274	CONTIG61	718432	718711	1.71	2.62E-02	transcription_start_site	+	721128	721128	-2556	4471	AN10445
1274	CONTIG61	718432	718711	1.71	2.62E-02	transcription_start_site	+	721272	721272	-2700	4472	AN10445
1925	CONTIG61	722179	722603	1.4	8.01E-02	transcription_start_site	+	721272	721272	1119	4472	AN10445
1087	CONTIG16	7893	8307	1.84	1.81E-02	transcription_start_site	-	3909	3909	-4191	4485	AN1045
1330	CONTIG61	438232	438594	1.67	3.08E-02	transcription_start_site	-	439004	439004	591	4499	AN10453
1330	CONTIG61	438232	438594	1.67	3.08E-02	transcription_start_site	-	439154	439154	741	4498	AN10453
2440	CONTIG61	496952	497601	1.22	1.28E-01	transcription_start_site	-	497920	497920	643	4500	AN10454
223	CONTIG61	699092	699366	2.89	0.00E+00	transcription_start_site	-	699027	699027	-202	4509	AN10457
631	CONTIG61	698267	698906	2.26	0.00E+00	transcription_start_site	-	699027	699027	440	4509	AN10457
277	CONTIG61	407346	407755	2.8	2.67E-04	transcription_start_site	+	407083	407083	467	4510	AN10458
1331	CONTIG61	450991	451340	1.67	3.08E-02	transcription_start_site	+	451413	451413	-247	4511	AN10459
1331	CONTIG61	450991	451340	1.67	3.08E-02	transcription_start_site	+	451550	451550	-384	4512	AN10459
1331	CONTIG61	450991	451340	1.67	3.08E-02	transcription_start_site	+	451665	451665	-499	4513	AN10459
1331	CONTIG61	450991	451340	1.67	3.08E-02	transcription_start_site	+	451856	451856	-690	4514	AN10459
1331	CONTIG61	450991	451340	1.67	3.08E-02	transcription_start_site	+	453096	453096	-1930	4515	AN10459
2694	CONTIG61	449855	450369	1.13	1.93E-01	transcription_start_site	+	451413	451413	-1301	4511	AN10459
2694	CONTIG61	449855	450369	1.13	1.93E-01	transcription_start_site	+	451550	451550	-1438	4512	AN10459
2694	CONTIG61	449855	450369	1.13	1.93E-01	transcription_start_site	+	451665	451665	-1553	4513	AN10459
2694	CONTIG61	449855	450369	1.13	1.93E-01	transcription_start_site	+	451856	451856	-1744	4514	AN10459
2694	CONTIG61	449855	450369	1.13	1.93E-01	transcription_start_site	+	453096	453096	-2984	4515	AN10459
1724	CONTIG61	429989	430259	1.49	5.88E-02	transcription_start_site	-	427889	427889	-2235	4518	AN10460
2693	CONTIG61	427519	427870	1.13	1.93E-01	transcription_start_site	-	427889	427889	194	4518	AN10460
361	CONTIG61	816234	817328	2.66	0.00E+00	transcription_start_site	+	815758	815758	1023	4519	AN10461
537	CONTIG61	812104	812913	2.39	0.00E+00	transcription_start_site	+	815758	815758	-3249	4519	AN10461
1275	CONTIG61	815119	815912	1.71	2.62E-02	transcription_start_site	+	815758	815758	-242	4519	AN10461
730	CONTIG61	515864	516138	2.17	4.61E-03	transcription_start_site	+	520527	520527	-4526	4531	AN10465
730	CONTIG61	515864	516138	2.17	4.61E-03	transcription_start_site	+	520791	520791	-4790	4532	AN10465
730	CONTIG61	515864	516138	2.17	4.61E-03	transcription_start_site	+	521066	521066	-5065	4533	AN10465
1139	CONTIG61	549456	549810	1.8	1.84E-02	transcription_start_site	-	545079	545079	-4554	4536	AN10466
1139	CONTIG61	549456	549810	1.8	1.84E-02	transcription_start_site	-	544822	544822	-4811	4537	AN10466
1139	CONTIG61	549456	549810	1.8	1.84E-02	transcription_start_site	-	544648	544648	-4985	4538	AN10466
1139	CONTIG61	549456	549810	1.8	1.84E-02	transcription_start_site	-	544518	544518	-5115	4539	AN10466
1274	CONTIG61	718432	718711	1.71	2.62E-02	transcription_start_site	+	718683	718683	-111	4545	AN10468
1274	CONTIG61	718432	718711	1.71	2.62E-02	transcription_start_site	+	719067	719067	-495	4546	AN10468
1274	CONTIG61	718432	718711	1.71	2.62E-02	transcription_start_site	+	720063	720063	-1491	4547	AN10468
1274	CONTIG61	718432	718711	1.71	2.62E-02	transcription_start_site	+	720208	720208	-1636	4548	AN10468
1087	CONTIG16	7893	8307	1.84	1.81E-02	transcription_start_site	+	8438	8438	-338	4552	AN1047
1087	CONTIG16	7893	8307	1.84	1.81E-02	transcription_start_site	+	8584	8584	-484	4553	AN1047
1087	CONTIG16	7893	8307	1.84	1.81E-02	transcription_start_site	+	8677	8677	-577	4554	AN1047
1087	CONTIG16	7893	8307	1.84	1.81E-02	transcription_start_site	+	10180	10180	-2080	4555	AN1047
1087	CONTIG16	7893	8307	1.84	1.81E-02	transcription_start_site	+	10539	10539	-2439	4556	AN1047
2350	CONTIG62	82576	82850	1.25	5.88E-02	transcription_start_site	+	85831	85831	-3118	4557	AN10470
2350	CONTIG62	82576	82850	1.25	5.88E-02	transcription_start_site	+	85932	85932	-3219	4558	AN10470
2350	CONTIG62	82576	82850	1.25	5.88E-02	transcription_start_site	+	86182	86182	-3469	4559	AN10470
2350	CONTIG62	82576	82850	1.25	5.88E-02	transcription_start_site	+	86362	86362	-3649	4560	AN10470
2350	CONTIG62	82576	82850	1.25	5.88E-02	transcription_start_site	+	87379	87379	-4666	4561	AN10470
893	CONTIG62	151143	151567	2	0.00E+00	transcription_start_site	-	147611	147611	-3744	4565	AN10472
893	CONTIG62	151143	151567	2	0.00E+00	transcription_start_site	-	147462	147462	-3893	4566	AN10472
893	CONTIG62	151143	151567	2	0.00E+00	transcription_start_site	-	147239	147239	-4116	4567	AN10472
893	CONTIG62	151143	151567	2	0.00E+00	transcription_start_site	-	147059	147059	-4296	4568	AN10472
893	CONTIG62	151143	151567	2	0.00E+00	transcription_start_site	-	146600	146600	-4755	4569	AN10472
893	CONTIG62	151143	151567	2	0.00E+00	transcription_start_site	-	146176	146176	-5179	4570	AN10472
2973	CONTIG62	526	895	0.95	1.93E-01	transcription_start_site	+	128	128	582	4577	AN10476
1087	CONTIG16	7893	8307	1.84	1.81E-02	transcription_start_site	+	12978	12978	-4878	4586	AN1048
1599	CONTIG16	14713	15357	1.54	5.52E-02	transcription_start_site	+	13901	13901	1134	4588	AN1048
2044	CONTIG63	6001	6666	1.36	1.07E-01	transcription_start_site	+	5654	5654	679	4590	AN10481

10	CONTIG63	7291	7550	3.69	0.00E+00	transcription_start_site	+	8017	8017	-596	4594	AN10483
10	CONTIG63	7291	7550	3.69	0.00E+00	transcription_start_site	+	8244	8244	-823	4595	AN10483
2044	CONTIG63	6001	6666	1.36	1.07E-01	transcription_start_site	+	8017	8017	-1683	4594	AN10483
2044	CONTIG63	6001	6666	1.36	1.07E-01	transcription_start_site	+	8244	8244	-1910	4595	AN10483
2986	CONTIG64	22818	23542	0.93	1.59E-01	transcription_start_site	+	26116	26116	-2936	4602	AN10486
2986	CONTIG64	22818	23542	0.93	1.59E-01	transcription_start_site	+	27182	27182	-4002	4603	AN10486
2986	CONTIG64	22818	23542	0.93	1.59E-01	transcription_start_site	+	28230	28230	-5050	4604	AN10486
486	CONTIG64	87241	87650	2.46	0.00E+00	transcription_start_site	-	83119	83119	-4326	4605	AN10487
486	CONTIG64	87241	87650	2.46	0.00E+00	transcription_start_site	-	82789	82789	-4656	4606	AN10487
486	CONTIG64	87241	87650	2.46	0.00E+00	transcription_start_site	-	82704	82704	-4741	4607	AN10487
486	CONTIG64	87241	87650	2.46	0.00E+00	transcription_start_site	-	82481	82481	-4964	4608	AN10487
1788	CONTIG64	84676	84960	1.46	1.57E-02	transcription_start_site	-	83119	83119	-1699	4605	AN10487
1788	CONTIG64	84676	84960	1.46	1.57E-02	transcription_start_site	-	82789	82789	-2029	4606	AN10487
1788	CONTIG64	84676	84960	1.46	1.57E-02	transcription_start_site	-	82704	82704	-2114	4607	AN10487
1788	CONTIG64	84676	84960	1.46	1.57E-02	transcription_start_site	-	82481	82481	-2337	4608	AN10487
1788	CONTIG64	84676	84960	1.46	1.57E-02	transcription_start_site	-	81948	81948	-2870	4609	AN10487
1788	CONTIG64	84676	84960	1.46	1.57E-02	transcription_start_site	-	81566	81566	-3252	4610	AN10487
2654	CONTIG64	13729	14153	1.14	6.84E-02	transcription_start_site	-	10642	10642	-3299	4613	AN10489
2654	CONTIG64	13729	14153	1.14	6.84E-02	transcription_start_site	-	10499	10499	-3442	4614	AN10489
2654	CONTIG64	13729	14153	1.14	6.84E-02	transcription_start_site	-	10012	10012	-3929	4615	AN10489
2654	CONTIG64	13729	14153	1.14	6.84E-02	transcription_start_site	-	9240	9240	-4701	4616	AN10489
1599	CONTIG16	14713	15357	1.54	5.52E-02	transcription_start_site	+	14390	14390	645	4617	AN1049
1151	CONTIG64	18919	19193	1.79	3.03E-03	transcription_start_site	+	23203	23203	-4147	4619	AN10491
1151	CONTIG64	18919	19193	1.79	3.03E-03	transcription_start_site	+	24177	24177	-5121	4620	AN10491
2986	CONTIG64	22818	23542	0.93	1.59E-01	transcription_start_site	+	23203	23203	-23	4619	AN10491
2986	CONTIG64	22818	23542	0.93	1.59E-01	transcription_start_site	+	24177	24177	-997	4620	AN10491
2986	CONTIG64	22818	23542	0.93	1.59E-01	transcription_start_site	+	24663	24663	-1483	4621	AN10491
1788	CONTIG64	84676	84960	1.46	1.57E-02	transcription_start_site	-	80892	80892	-3926	4622	AN10492
1788	CONTIG64	84676	84960	1.46	1.57E-02	transcription_start_site	-	80763	80763	-4055	4623	AN10492
2521	CONTIG65	53637	54056	1.19	5.88E-02	transcription_start_site	+	53886	53886	-39	4626	AN10494
2521	CONTIG65	53637	54056	1.19	5.88E-02	transcription_start_site	+	54787	54787	-940	4627	AN10494
2936	CONTIG65	49365	49634	0.98	1.44E-01	transcription_start_site	+	53886	53886	-4386	4626	AN10494
2522	CONTIG65	201680	201965	1.19	5.88E-02	transcription_start_site	+	202083	202083	-260	4631	AN10496
2522	CONTIG65	201680	201965	1.19	5.88E-02	transcription_start_site	+	202309	202309	-486	4632	AN10496
2522	CONTIG65	201680	201965	1.19	5.88E-02	transcription_start_site	+	202373	202373	-550	4633	AN10496
2522	CONTIG65	201680	201965	1.19	5.88E-02	transcription_start_site	+	202451	202451	-628	4634	AN10496
2522	CONTIG65	201680	201965	1.19	5.88E-02	transcription_start_site	+	202649	202649	-826	4635	AN10496
2522	CONTIG65	201680	201965	1.19	5.88E-02	transcription_start_site	+	203012	203012	-1189	4636	AN10496
2522	CONTIG65	201680	201965	1.19	5.88E-02	transcription_start_site	+	203202	203202	-1379	4637	AN10496
2522	CONTIG65	201680	201965	1.19	5.88E-02	transcription_start_site	+	203319	203319	-1496	4638	AN10496
2399	CONTIG65	249682	249951	1.23	5.22E-02	transcription_start_site	+	250162	250162	-345	4644	AN10497
2820	CONTIG65	29481	30289	1.05	5.82E-02	transcription_start_site	-	30100	30100	215	4646	AN10499
2936	CONTIG65	49365	49634	0.98	1.44E-01	transcription_start_site	+	50385	50385	-885	4658	AN10502
2936	CONTIG65	49365	49634	0.98	1.44E-01	transcription_start_site	+	50560	50560	-1060	4659	AN10502
2936	CONTIG65	49365	49634	0.98	1.44E-01	transcription_start_site	+	50936	50936	-1436	4660	AN10502
2936	CONTIG65	49365	49634	0.98	1.44E-01	transcription_start_site	+	51071	51071	-1571	4661	AN10502
2936	CONTIG65	49365	49634	0.98	1.44E-01	transcription_start_site	+	51390	51390	-1890	4662	AN10502
2936	CONTIG65	49365	49634	0.98	1.44E-01	transcription_start_site	+	51889	51889	-2389	4663	AN10502
2522	CONTIG65	201680	201965	1.19	5.88E-02	transcription_start_site	+	204308	204308	-2485	4670	AN10504
2522	CONTIG65	201680	201965	1.19	5.88E-02	transcription_start_site	+	204939	204939	-3116	4671	AN10504
2820	CONTIG65	29481	30289	1.05	5.82E-02	transcription_start_site	-	28645	28645	-1240	4672	AN10505
2820	CONTIG65	29481	30289	1.05	5.82E-02	transcription_start_site	-	28340	28340	-1545	4673	AN10505
2820	CONTIG65	29481	30289	1.05	5.82E-02	transcription_start_site	-	28183	28183	-1702	4674	AN10505
2820	CONTIG65	29481	30289	1.05	5.82E-02	transcription_start_site	-	27460	27460	-2425	4675	AN10505
1696	CONTIG67	117693	117957	1.5	5.22E-02	transcription_start_site	+	119749	119749	-1924	4676	AN10506
1696	CONTIG67	117693	117957	1.5	5.22E-02	transcription_start_site	+	119842	119842	-2017	4677	AN10506
1696	CONTIG67	117693	117957	1.5	5.22E-02	transcription_start_site	+	120311	120311	-2486	4678	AN10506

1696	CONTIG67	117693	117957	1.5	5.22E-02	transcription_start_site	+	120661	120661	-2836	4679	AN10506
1696	CONTIG67	117693	117957	1.5	5.22E-02	transcription_start_site	+	121185	121185	-3360	4680	AN10506
1696	CONTIG67	117693	117957	1.5	5.22E-02	transcription_start_site	+	121358	121358	-3533	4681	AN10506
1696	CONTIG67	117693	117957	1.5	5.22E-02	transcription_start_site	+	118448	118448	-623	4682	AN10507
2751	CONTIG67	237542	237806	1.1	1.93E-01	transcription_start_site	+	242010	242010	-4336	4683	AN10508
2751	CONTIG67	237542	237806	1.1	1.93E-01	transcription_start_site	+	242127	242127	-4453	4684	AN10508
2751	CONTIG67	237542	237806	1.1	1.93E-01	transcription_start_site	+	242303	242303	-4629	4685	AN10508
745	CONTIG67	2851	3200	2.16	3.61E-03	transcription_start_site	+	5874	5874	-2848	4686	AN10509
478	CONTIG67	212930	213584	2.47	0.00E+00	transcription_start_site	+	217660	217660	-4403	4687	AN10510
478	CONTIG67	212930	213584	2.47	0.00E+00	transcription_start_site	+	217795	217795	-4538	4688	AN10510
478	CONTIG67	212930	213584	2.47	0.00E+00	transcription_start_site	+	218241	218241	-4984	4689	AN10510
478	CONTIG67	212930	213584	2.47	0.00E+00	transcription_start_site	+	218370	218370	-5113	4690	AN10510
478	CONTIG67	212930	213584	2.47	0.00E+00	transcription_start_site	+	218449	218449	-5192	4691	AN10510
2535	CONTIG67	214576	214935	1.19	1.44E-01	transcription_start_site	+	217660	217660	-2904	4687	AN10510
2535	CONTIG67	214576	214935	1.19	1.44E-01	transcription_start_site	+	217795	217795	-3039	4688	AN10510
2535	CONTIG67	214576	214935	1.19	1.44E-01	transcription_start_site	+	218241	218241	-3485	4689	AN10510
2535	CONTIG67	214576	214935	1.19	1.44E-01	transcription_start_site	+	218370	218370	-3614	4690	AN10510
2535	CONTIG67	214576	214935	1.19	1.44E-01	transcription_start_site	+	218449	218449	-3693	4691	AN10510
1694	CONTIG67	51090	51970	1.5	5.22E-02	transcription_start_site	+	51777	51777	-247	4692	AN10511
1694	CONTIG67	51090	51970	1.5	5.22E-02	transcription_start_site	+	52619	52619	-1089	4693	AN10511
2724	CONTIG68	17253	18292	1.11	1.11E-01	transcription_start_site	-	18894	18894	1121	4696	AN10512
2132	CONTIG68	36903	37262	1.32	6.16E-02	transcription_start_site	+	36691	36691	391	4705	AN10514
2132	CONTIG68	36903	37262	1.32	6.16E-02	transcription_start_site	+	36432	36432	650	4704	AN10514
1811	CONTIG68	72757	73027	1.45	5.22E-02	transcription_start_site	+	73062	73062	-170	4706	AN10515
1811	CONTIG68	72757	73027	1.45	5.22E-02	transcription_start_site	+	74015	74015	-1123	4707	AN10515
1811	CONTIG68	72757	73027	1.45	5.22E-02	transcription_start_site	+	74162	74162	-1270	4708	AN10515
2800	CONTIG68	109132	109420	1.07	1.93E-01	transcription_start_site	+	113627	113627	-4351	4712	AN10517
2800	CONTIG68	109132	109420	1.07	1.93E-01	transcription_start_site	+	113830	113830	-4554	4713	AN10517
2800	CONTIG68	109132	109420	1.07	1.93E-01	transcription_start_site	+	113921	113921	-4645	4714	AN10517
32	CONTIG16	24243	24657	3.53	0.00E+00	transcription_start_site	+	25596	25596	-1146	4722	AN1052
32	CONTIG16	24243	24657	3.53	0.00E+00	transcription_start_site	+	25697	25697	-1247	4723	AN1052
379	CONTIG16	22068	22477	2.63	0.00E+00	transcription_start_site	+	25596	25596	-3323	4722	AN1052
379	CONTIG16	22068	22477	2.63	0.00E+00	transcription_start_site	+	25697	25697	-3424	4723	AN1052
710	CONTIG16	26343	26607	2.19	8.81E-03	transcription_start_site	+	25697	25697	778	4723	AN1052
710	CONTIG16	26343	26607	2.19	8.81E-03	transcription_start_site	+	25596	25596	879	4722	AN1052
2724	CONTIG68	17253	18292	1.11	1.11E-01	transcription_start_site	-	18391	18391	618	4724	AN10520
1811	CONTIG68	72757	73027	1.45	5.22E-02	transcription_start_site	+	76723	76723	-3831	4725	AN10521
1811	CONTIG68	72757	73027	1.45	5.22E-02	transcription_start_site	+	76841	76841	-3949	4726	AN10521
857	CONTIG68	54453	54727	2.05	4.61E-03	transcription_start_site	-	52112	52112	-2478	4733	AN10525
857	CONTIG68	54453	54727	2.05	4.61E-03	transcription_start_site	-	51960	51960	-2630	4734	AN10525
857	CONTIG68	54453	54727	2.05	4.61E-03	transcription_start_site	-	51716	51716	-2874	4735	AN10525
2733	CONTIG68	50793	51074	1.11	1.66E-01	transcription_start_site	-	51716	51716	782	4735	AN10525
2733	CONTIG68	50793	51074	1.11	1.66E-01	transcription_start_site	-	51960	51960	1026	4734	AN10525
401	CONTIG68	222980	223329	2.6	2.39E-04	transcription_start_site	+	223691	223691	-536	4754	AN10528
1801	CONTIG68	223965	224902	1.45	5.26E-03	transcription_start_site	+	223691	223691	742	4754	AN10528
2375	CONTIG68	221115	221474	1.24	9.26E-02	transcription_start_site	+	223691	223691	-2396	4754	AN10528
32	CONTIG16	24243	24657	3.53	0.00E+00	transcription_start_site	+	28152	28152	-3702	4756	AN1053
32	CONTIG16	24243	24657	3.53	0.00E+00	transcription_start_site	+	28259	28259	-3809	4757	AN1053
710	CONTIG16	26343	26607	2.19	8.81E-03	transcription_start_site	+	28152	28152	-1677	4756	AN1053
710	CONTIG16	26343	26607	2.19	8.81E-03	transcription_start_site	+	28259	28259	-1784	4757	AN1053
2132	CONTIG68	36903	37262	1.32	6.16E-02	transcription_start_site	+	37734	37734	-651	4758	AN10530
2132	CONTIG68	36903	37262	1.32	6.16E-02	transcription_start_site	+	37970	37970	-887	4759	AN10530
702	CONTIG69	33091	33501	2.19	1.54E-03	transcription_start_site	-	32804	32804	-492	4763	AN10533
702	CONTIG69	33091	33501	2.19	1.54E-03	transcription_start_site	-	32726	32726	-570	4764	AN10533
702	CONTIG69	33091	33501	2.19	1.54E-03	transcription_start_site	-	29964	29964	-3332	4765	AN10533
702	CONTIG69	33091	33501	2.19	1.54E-03	transcription_start_site	-	29656	29656	-3640	4766	AN10534
702	CONTIG69	33091	33501	2.19	1.54E-03	transcription_start_site	-	29474	29474	-3822	4767	AN10534

347	CONTIG71	2102	2455	2.69	1.57E-02	transcription_start_site	+	2304	2304	-25	4770	AN10537
2429	CONTIG74	6610	7329	1.22	3.08E-02	transcription_start_site	-	6529	6529	-440	4771	AN10539
2429	CONTIG74	6610	7329	1.22	3.08E-02	transcription_start_site	-	6229	6229	-740	4772	AN10539
2860	CONTIG74	8570	8836	1.03	8.01E-02	transcription_start_site	-	6529	6529	-2174	4771	AN10539
2860	CONTIG74	8570	8836	1.03	8.01E-02	transcription_start_site	-	6229	6229	-2474	4772	AN10539
1093	CONTIG16	33833	34342	1.84	3.08E-02	transcription_start_site	-	32252	32252	-1835	4773	AN1054
1093	CONTIG16	33833	34342	1.84	3.08E-02	transcription_start_site	-	32010	32010	-2077	4774	AN1054
1093	CONTIG16	33833	34342	1.84	3.08E-02	transcription_start_site	-	31834	31834	-2253	4775	AN1054
1093	CONTIG16	33833	34342	1.84	3.08E-02	transcription_start_site	-	31650	31650	-2437	4776	AN1054
1093	CONTIG16	33833	34342	1.84	3.08E-02	transcription_start_site	-	31531	31531	-2556	4777	AN1054
1093	CONTIG16	33833	34342	1.84	3.08E-02	transcription_start_site	-	30837	30837	-3250	4778	AN1054
1974	CONTIG75	98405	100939	1.38	3.64E-02	transcription_start_site	-	97484	97484	-2188	4790	AN10543
1974	CONTIG75	98405	100939	1.38	3.64E-02	transcription_start_site	-	97260	97260	-2412	4791	AN10543
1974	CONTIG75	98405	100939	1.38	3.64E-02	transcription_start_site	-	96625	96625	-3047	4792	AN10543
1974	CONTIG75	98405	100939	1.38	3.64E-02	transcription_start_site	-	95557	95557	-4115	4793	AN10543
1402	CONTIG75	139580	139929	1.63	3.99E-02	transcription_start_site	+	140128	140128	-373	4794	AN10544
1261	CONTIG75	143865	144129	1.72	4.36E-02	transcription_start_site	+	143769	143769	228	4804	AN10548
1261	CONTIG75	143865	144129	1.72	4.36E-02	transcription_start_site	+	143546	143546	451	4803	AN10548
1261	CONTIG75	143865	144129	1.72	4.36E-02	transcription_start_site	+	144925	144925	-928	4805	AN10548
1261	CONTIG75	143865	144129	1.72	4.36E-02	transcription_start_site	+	143028	143028	969	4802	AN10548
1402	CONTIG75	139580	139929	1.63	3.99E-02	transcription_start_site	+	142724	142724	-2969	4801	AN10548
1402	CONTIG75	139580	139929	1.63	3.99E-02	transcription_start_site	+	143028	143028	-3273	4802	AN10548
1402	CONTIG75	139580	139929	1.63	3.99E-02	transcription_start_site	+	143546	143546	-3791	4803	AN10548
1402	CONTIG75	139580	139929	1.63	3.99E-02	transcription_start_site	+	143769	143769	-4014	4804	AN10548
1402	CONTIG75	139580	139929	1.63	3.99E-02	transcription_start_site	+	144925	144925	-5170	4805	AN10548
1261	CONTIG75	143865	144129	1.72	4.36E-02	transcription_start_site	+	146341	146341	-2344	4806	AN10549
1093	CONTIG16	33833	34342	1.84	3.08E-02	transcription_start_site	-	34426	34426	338	4810	AN1055
1093	CONTIG16	33833	34342	1.84	3.08E-02	transcription_start_site	-	34894	34894	806	4809	AN1055
1093	CONTIG16	33833	34342	1.84	3.08E-02	transcription_start_site	-	35009	35009	921	4808	AN1055
1093	CONTIG16	33833	34342	1.84	3.08E-02	transcription_start_site	-	35086	35086	998	4807	AN1055
1974	CONTIG75	98405	100939	1.38	3.64E-02	transcription_start_site	-	99135	99135	-537	4812	AN10550
1974	CONTIG75	98405	100939	1.38	3.64E-02	transcription_start_site	-	100411	100411	739	4811	AN10550
1746	CONTIG76	150839	151253	1.48	4.42E-02	transcription_start_site	-	146448	146448	-4598	4822	AN10552
1746	CONTIG76	150839	151253	1.48	4.42E-02	transcription_start_site	-	146090	146090	-4956	4823	AN10552
1746	CONTIG76	150839	151253	1.48	4.42E-02	transcription_start_site	-	145975	145975	-5071	4824	AN10552
1943	CONTIG76	147019	147653	1.39	7.51E-02	transcription_start_site	-	146448	146448	-888	4822	AN10552
1943	CONTIG76	147019	147653	1.39	7.51E-02	transcription_start_site	-	146090	146090	-1246	4823	AN10552
1943	CONTIG76	147019	147653	1.39	7.51E-02	transcription_start_site	-	145975	145975	-1361	4824	AN10552
1943	CONTIG76	147019	147653	1.39	7.51E-02	transcription_start_site	-	145627	145627	-1709	4825	AN10552
1943	CONTIG76	147019	147653	1.39	7.51E-02	transcription_start_site	-	145394	145394	-1942	4826	AN10552
1943	CONTIG76	147019	147653	1.39	7.51E-02	transcription_start_site	-	144650	144650	-2686	4831	AN10554
1943	CONTIG76	147019	147653	1.39	7.51E-02	transcription_start_site	-	144463	144463	-2873	4832	AN10554
1943	CONTIG76	147019	147653	1.39	7.51E-02	transcription_start_site	-	144134	144134	-3202	4833	AN10554
1943	CONTIG76	147019	147653	1.39	7.51E-02	transcription_start_site	-	143839	143839	-3497	4834	AN10554
1825	CONTIG77	35402	35704	1.44	4.36E-02	transcription_start_site	+	39260	39260	-3707	4835	AN10555
1305	CONTIG77	44041	44402	1.69	1.57E-02	transcription_start_site	+	48169	48169	-3947	4836	AN10556
1305	CONTIG77	44041	44402	1.69	1.57E-02	transcription_start_site	+	48982	48982	-4760	4837	AN10556
1305	CONTIG77	44041	44402	1.69	1.57E-02	transcription_start_site	+	49219	49219	-4997	4838	AN10556
2727	CONTIG77	46881	47245	1.11	1.44E-01	transcription_start_site	+	48169	48169	-1106	4836	AN10556
2727	CONTIG77	46881	47245	1.11	1.44E-01	transcription_start_site	+	48982	48982	-1919	4837	AN10556
2727	CONTIG77	46881	47245	1.11	1.44E-01	transcription_start_site	+	49219	49219	-2156	4838	AN10556
2727	CONTIG77	46881	47245	1.11	1.44E-01	transcription_start_site	+	49742	49742	-2679	4839	AN10556
2727	CONTIG77	46881	47245	1.11	1.44E-01	transcription_start_site	+	51535	51535	-4472	4840	AN10556
757	CONTIG77	126096	126590	2.14	1.80E-03	transcription_start_site	-	126524	126524	181	4842	AN10557
757	CONTIG77	126096	126590	2.14	1.80E-03	transcription_start_site	-	126794	126794	451	4841	AN10557
2725	CONTIG77	127430	128100	1.11	1.33E-01	transcription_start_site	-	126794	126794	-971	4841	AN10557
2725	CONTIG77	127430	128100	1.11	1.33E-01	transcription_start_site	-	126524	126524	-1241	4842	AN10557

1825	CONTIG77	35402	35704	1.44	4.36E-02	transcription_start_site	+	37026	37026	-1473	4843	AN10558
2727	CONTIG77	46881	47245	1.11	1.44E-01	transcription_start_site	+	52031	52031	-4968	4844	AN10559
2727	CONTIG77	46881	47245	1.11	1.44E-01	transcription_start_site	+	52208	52208	-5145	4845	AN10559
2728	CONTIG77	52955	53304	1.11	1.44E-01	transcription_start_site	+	52208	52208	921	4845	AN10559
2728	CONTIG77	52955	53304	1.11	1.44E-01	transcription_start_site	+	52031	52031	1098	4844	AN10559
1093	CONTIG16	33833	34342	1.84	3.08E-02	transcription_start_site	+	35431	35431	-1343	4846	AN1056
1093	CONTIG16	33833	34342	1.84	3.08E-02	transcription_start_site	+	35521	35521	-1433	4847	AN1056
1093	CONTIG16	33833	34342	1.84	3.08E-02	transcription_start_site	+	35749	35749	-1661	4848	AN1056
1093	CONTIG16	33833	34342	1.84	3.08E-02	transcription_start_site	+	36471	36471	-2383	4849	AN1056
757	CONTIG77	126096	126590	2.14	1.80E-03	transcription_start_site	-	122968	122968	-3375	4850	AN10560
757	CONTIG77	126096	126590	2.14	1.80E-03	transcription_start_site	-	122476	122476	-3867	4851	AN10560
757	CONTIG77	126096	126590	2.14	1.80E-03	transcription_start_site	-	121883	121883	-4460	4852	AN10560
757	CONTIG77	126096	126590	2.14	1.80E-03	transcription_start_site	-	121781	121781	-4562	4853	AN10560
757	CONTIG77	126096	126590	2.14	1.80E-03	transcription_start_site	-	121682	121682	-4661	4854	AN10560
2725	CONTIG77	127430	128100	1.11	1.33E-01	transcription_start_site	-	122968	122968	-4797	4850	AN10560
2725	CONTIG77	127430	128100	1.11	1.33E-01	transcription_start_site	-	122476	122476	-5289	4851	AN10560
73	CONTIG78	11568	12502	3.31	0.00E+00	transcription_start_site	+	15572	15572	-3537	4855	AN10561
73	CONTIG78	11568	12502	3.31	0.00E+00	transcription_start_site	+	15692	15692	-3657	4856	AN10561
73	CONTIG78	11568	12502	3.31	0.00E+00	transcription_start_site	+	16148	16148	-4113	4857	AN10561
73	CONTIG78	11568	12502	3.31	0.00E+00	transcription_start_site	+	16243	16243	-4208	4858	AN10561
885	CONTIG78	10593	10927	2.01	7.85E-03	transcription_start_site	+	15572	15572	-4812	4855	AN10561
885	CONTIG78	10593	10927	2.01	7.85E-03	transcription_start_site	+	15692	15692	-4932	4856	AN10561
1459	CONTIG78	14935	15371	1.61	3.70E-02	transcription_start_site	+	15572	15572	-419	4855	AN10561
1459	CONTIG78	14935	15371	1.61	3.70E-02	transcription_start_site	+	15692	15692	-539	4856	AN10561
1459	CONTIG78	14935	15371	1.61	3.70E-02	transcription_start_site	+	16148	16148	-995	4857	AN10561
1459	CONTIG78	14935	15371	1.61	3.70E-02	transcription_start_site	+	16243	16243	-1090	4858	AN10561
123	CONTIG78	303093	303362	3.13	0.00E+00	transcription_start_site	+	306056	306056	-2828	4868	AN10565
123	CONTIG78	303093	303362	3.13	0.00E+00	transcription_start_site	+	306850	306850	-3622	4869	AN10565
1641	CONTIG78	302258	302907	1.52	5.22E-02	transcription_start_site	+	306056	306056	-3473	4868	AN10565
1641	CONTIG78	302258	302907	1.52	5.22E-02	transcription_start_site	+	306850	306850	-4267	4869	AN10565
73	CONTIG78	11568	12502	3.31	0.00E+00	transcription_start_site	+	16636	16636	-4601	4874	AN10568
73	CONTIG78	11568	12502	3.31	0.00E+00	transcription_start_site	+	17156	17156	-5121	4875	AN10568
73	CONTIG78	11568	12502	3.31	0.00E+00	transcription_start_site	+	17265	17265	-5230	4876	AN10568
1459	CONTIG78	14935	15371	1.61	3.70E-02	transcription_start_site	+	16636	16636	-1483	4874	AN10568
1459	CONTIG78	14935	15371	1.61	3.70E-02	transcription_start_site	+	17156	17156	-2003	4875	AN10568
1459	CONTIG78	14935	15371	1.61	3.70E-02	transcription_start_site	+	17265	17265	-2112	4876	AN10568
1093	CONTIG16	33833	34342	1.84	3.08E-02	transcription_start_site	+	37202	37202	-3114	4879	AN1057
1093	CONTIG16	33833	34342	1.84	3.08E-02	transcription_start_site	+	37360	37360	-3272	4880	AN1057
1093	CONTIG16	33833	34342	1.84	3.08E-02	transcription_start_site	+	37533	37533	-3445	4881	AN1057
1093	CONTIG16	33833	34342	1.84	3.08E-02	transcription_start_site	+	39092	39092	-5004	4882	AN1057
123	CONTIG78	303093	303362	3.13	0.00E+00	transcription_start_site	+	307219	307219	-3991	4883	AN10570
123	CONTIG78	303093	303362	3.13	0.00E+00	transcription_start_site	+	307488	307488	-4260	4884	AN10570
1641	CONTIG78	302258	302907	1.52	5.22E-02	transcription_start_site	+	307219	307219	-4636	4883	AN10570
1641	CONTIG78	302258	302907	1.52	5.22E-02	transcription_start_site	+	307488	307488	-4905	4884	AN10570
1807	CONTIG79	123826	124100	1.45	4.36E-02	transcription_start_site	+	125791	125791	-1828	4887	AN10572
1807	CONTIG79	123826	124100	1.45	4.36E-02	transcription_start_site	+	126524	126524	-2561	4888	AN10572
2513	CONTIG79	192617	193113	1.2	1.08E-01	transcription_start_site	-	188628	188628	-4237	4892	AN10574
2614	CONTIG79	188937	189201	1.16	1.25E-01	transcription_start_site	-	188628	188628	-441	4892	AN10574
2614	CONTIG79	188937	189201	1.16	1.25E-01	transcription_start_site	-	186028	186028	-3041	4893	AN10574
2614	CONTIG79	188937	189201	1.16	1.25E-01	transcription_start_site	-	185751	185751	-3318	4894	AN10574
1807	CONTIG79	123826	124100	1.45	4.36E-02	transcription_start_site	+	124429	124429	-466	4895	AN10575
2358	CONTIG79	246	500	1.25	9.24E-02	transcription_start_site	+	116	116	257	4898	AN10577
125	CONTIG16	45153	45742	3.13	0.00E+00	transcription_start_site	+	44636	44636	811	4908	AN1058
125	CONTIG16	45153	45742	3.13	0.00E+00	transcription_start_site	+	44293	44293	1154	4907	AN1058
471	CONTIG16	41778	42652	2.48	3.03E-03	transcription_start_site	+	43649	43649	-1434	4906	AN1058
471	CONTIG16	41778	42652	2.48	3.03E-03	transcription_start_site	+	44293	44293	-2078	4907	AN1058
471	CONTIG16	41778	42652	2.48	3.03E-03	transcription_start_site	+	44636	44636	-2421	4908	AN1058

513	CONTIG16	40738	41017	2.43	3.61E-03	transcription_start_site	+	43649	43649	-2771	4906	AN1058
513	CONTIG16	40738	41017	2.43	3.61E-03	transcription_start_site	+	44293	44293	-3415	4907	AN1058
513	CONTIG16	40738	41017	2.43	3.61E-03	transcription_start_site	+	44636	44636	-3758	4908	AN1058
1836	CONTIG16	43743	44827	1.44	1.08E-01	transcription_start_site	+	44293	44293	-8	4907	AN1058
1836	CONTIG16	43743	44827	1.44	1.08E-01	transcription_start_site	+	44636	44636	-351	4908	AN1058
1836	CONTIG16	43743	44827	1.44	1.08E-01	transcription_start_site	+	43649	43649	636	4906	AN1058
2057	CONTIG80	295363	296007	1.35	8.36E-02	transcription_start_site	-	292807	292807	-2878	4919	AN10583
2057	CONTIG80	295363	296007	1.35	8.36E-02	transcription_start_site	-	292730	292730	-2955	4920	AN10583
2057	CONTIG80	295363	296007	1.35	8.36E-02	transcription_start_site	-	292145	292145	-3540	4921	AN10583
2057	CONTIG80	295363	296007	1.35	8.36E-02	transcription_start_site	-	291862	291862	-3823	4922	AN10583
2057	CONTIG80	295363	296007	1.35	8.36E-02	transcription_start_site	-	291741	291741	-3944	4923	AN10583
2496	CONTIG80	287927	288292	1.21	1.66E-01	transcription_start_site	+	290034	290034	-1924	4933	AN10586
2496	CONTIG80	287927	288292	1.21	1.66E-01	transcription_start_site	+	290520	290520	-2410	4934	AN10586
47	CONTIG80	46142	46551	3.44	0.00E+00	transcription_start_site	-	46393	46393	46	4936	AN10587
47	CONTIG80	46142	46551	3.44	0.00E+00	transcription_start_site	-	46542	46542	195	4935	AN10587
47	CONTIG80	46142	46551	3.44	0.00E+00	transcription_start_site	-	46080	46080	-266	4937	AN10587
657	CONTIG80	47107	47766	2.23	1.22E-03	transcription_start_site	-	46542	46542	-894	4935	AN10587
657	CONTIG80	47107	47766	2.23	1.22E-03	transcription_start_site	-	46393	46393	-1043	4936	AN10587
657	CONTIG80	47107	47766	2.23	1.22E-03	transcription_start_site	-	46080	46080	-1356	4937	AN10587
2180	CONTIG80	44042	45216	1.3	6.32E-02	transcription_start_site	-	46080	46080	1451	4937	AN10587
1719	CONTIG80	81154	81518	1.49	5.05E-02	transcription_start_site	-	81687	81687	351	4938	AN10588
125	CONTIG16	45153	45742	3.13	0.00E+00	transcription_start_site	+	46429	46429	-981	4941	AN1059
125	CONTIG16	45153	45742	3.13	0.00E+00	transcription_start_site	+	46870	46870	-1422	4942	AN1059
125	CONTIG16	45153	45742	3.13	0.00E+00	transcription_start_site	+	48887	48887	-3439	4943	AN1059
471	CONTIG16	41778	42652	2.48	3.03E-03	transcription_start_site	+	46429	46429	-4214	4941	AN1059
471	CONTIG16	41778	42652	2.48	3.03E-03	transcription_start_site	+	46870	46870	-4655	4942	AN1059
1836	CONTIG16	43743	44827	1.44	1.08E-01	transcription_start_site	+	46429	46429	-2144	4941	AN1059
1836	CONTIG16	43743	44827	1.44	1.08E-01	transcription_start_site	+	46870	46870	-2585	4942	AN1059
1836	CONTIG16	43743	44827	1.44	1.08E-01	transcription_start_site	+	48887	48887	-4602	4943	AN1059
2496	CONTIG80	287927	288292	1.21	1.66E-01	transcription_start_site	-	289280	289280	1170	4946	AN10590
1313	CONTIG80	101628	102067	1.68	2.18E-02	transcription_start_site	+	103430	103430	-1582	4948	AN10592
2201	CONTIG80	98118	98462	1.3	1.25E-01	transcription_start_site	+	103430	103430	-5140	4948	AN10592
764	CONTIG80	334276	334850	2.14	6.61E-03	transcription_start_site	+	334488	334488	75	4949	AN10593
1113	CONTIG81	104938	105301	1.82	6.61E-03	transcription_start_site	+	106647	106647	-1527	4953	AN10595
1113	CONTIG81	104938	105301	1.82	6.61E-03	transcription_start_site	+	107232	107232	-2112	4954	AN10595
1113	CONTIG81	104938	105301	1.82	6.61E-03	transcription_start_site	+	108580	108580	-3460	4955	AN10595
1113	CONTIG81	104938	105301	1.82	6.61E-03	transcription_start_site	+	108836	108836	-3716	4956	AN10595
1225	CONTIG81	109593	110017	1.74	1.17E-03	transcription_start_site	+	108836	108836	969	4956	AN10595
1932	CONTIG81	150318	150957	1.39	2.64E-02	transcription_start_site	-	151562	151562	924	4963	AN10597
1932	CONTIG81	150318	150957	1.39	2.64E-02	transcription_start_site	-	151622	151622	984	4962	AN10597
1932	CONTIG81	150318	150957	1.39	2.64E-02	transcription_start_site	-	151804	151804	1166	4961	AN10597
1113	CONTIG81	104938	105301	1.82	6.61E-03	transcription_start_site	+	109942	109942	-4822	4964	AN10598
1225	CONTIG81	109593	110017	1.74	1.17E-03	transcription_start_site	+	109942	109942	-137	4964	AN10598
1225	CONTIG81	109593	110017	1.74	1.17E-03	transcription_start_site	+	112218	112218	-2413	4965	AN10598
1397	CONTIG81	110413	110912	1.63	1.57E-02	transcription_start_site	+	109942	109942	720	4964	AN10598
1397	CONTIG81	110413	110912	1.63	1.57E-02	transcription_start_site	+	112218	112218	-1555	4965	AN10598
1604	CONTIG16	54004	54353	1.54	8.01E-02	transcription_start_site	+	53635	53635	543	4974	AN1060
1110	CONTIG83	12691	12955	1.83	1.57E-02	transcription_start_site	+	13109	13109	-286	4980	AN10601
1110	CONTIG83	12691	12955	1.83	1.57E-02	transcription_start_site	+	13347	13347	-524	4981	AN10601
1110	CONTIG83	12691	12955	1.83	1.57E-02	transcription_start_site	+	13535	13535	-712	4982	AN10601
1110	CONTIG83	12691	12955	1.83	1.57E-02	transcription_start_site	+	13656	13656	-833	4983	AN10601
1294	CONTIG83	8701	8995	1.7	2.62E-02	transcription_start_site	+	13109	13109	-4261	4980	AN10601
1294	CONTIG83	8701	8995	1.7	2.62E-02	transcription_start_site	+	13347	13347	-4499	4981	AN10601
1294	CONTIG83	8701	8995	1.7	2.62E-02	transcription_start_site	+	13535	13535	-4687	4982	AN10601
1294	CONTIG83	8701	8995	1.7	2.62E-02	transcription_start_site	+	13656	13656	-4808	4983	AN10601
1295	CONTIG83	13066	14300	1.7	2.62E-02	transcription_start_site	+	13656	13656	27	4983	AN10601
1295	CONTIG83	13066	14300	1.7	2.62E-02	transcription_start_site	+	13535	13535	148	4982	AN10601

1295	CONTIG83	13066	14300	1.7	2.62E-02	transcription_start_site	+	13347	13347	336	4981	AN10601
1295	CONTIG83	13066	14300	1.7	2.62E-02	transcription_start_site	+	13109	13109	574	4980	AN10601
802	CONTIG83	16052	16413	2.1	5.20E-03	transcription_start_site	+	16088	16088	144	4991	AN10604
802	CONTIG83	16052	16413	2.1	5.20E-03	transcription_start_site	+	15850	15850	382	4990	AN10604
802	CONTIG83	16052	16413	2.1	5.20E-03	transcription_start_site	+	15177	15177	1055	4989	AN10604
1110	CONTIG83	12691	12955	1.83	1.57E-02	transcription_start_site	+	14921	14921	-2098	4988	AN10604
1110	CONTIG83	12691	12955	1.83	1.57E-02	transcription_start_site	+	15177	15177	-2354	4989	AN10604
1110	CONTIG83	12691	12955	1.83	1.57E-02	transcription_start_site	+	15850	15850	-3027	4990	AN10604
1110	CONTIG83	12691	12955	1.83	1.57E-02	transcription_start_site	+	16088	16088	-3265	4991	AN10604
1295	CONTIG83	13066	14300	1.7	2.62E-02	transcription_start_site	+	14921	14921	-1238	4988	AN10604
1295	CONTIG83	13066	14300	1.7	2.62E-02	transcription_start_site	+	15177	15177	-1494	4989	AN10604
1295	CONTIG83	13066	14300	1.7	2.62E-02	transcription_start_site	+	15850	15850	-2167	4990	AN10604
1295	CONTIG83	13066	14300	1.7	2.62E-02	transcription_start_site	+	16088	16088	-2405	4991	AN10604
802	CONTIG83	16052	16413	2.1	5.20E-03	transcription_start_site	+	21287	21287	-5054	4992	AN10605
802	CONTIG83	16052	16413	2.1	5.20E-03	transcription_start_site	+	21409	21409	-5176	4993	AN10605
886	CONTIG83	34367	34631	2.01	7.85E-03	transcription_start_site	-	34244	34244	-255	5000	AN10606
886	CONTIG83	34367	34631	2.01	7.85E-03	transcription_start_site	-	34110	34110	-389	5001	AN10606
886	CONTIG83	34367	34631	2.01	7.85E-03	transcription_start_site	-	35231	35231	732	4999	AN10606
886	CONTIG83	34367	34631	2.01	7.85E-03	transcription_start_site	-	35423	35423	924	4998	AN10606
1705	CONTIG84	3090	3954	1.5	1.08E-01	transcription_start_site	+	8848	8848	-5326	5003	AN10608
1960	CONTIG84	4070	4629	1.39	1.44E-01	transcription_start_site	+	8848	8848	-4498	5003	AN10608
1960	CONTIG84	4070	4629	1.39	1.44E-01	transcription_start_site	+	9240	9240	-4890	5004	AN10608
258	CONTIG16	56270	56697	2.83	8.69E-04	transcription_start_site	+	57049	57049	-565	5006	AN1061
258	CONTIG16	56270	56697	2.83	8.69E-04	transcription_start_site	+	57231	57231	-747	5007	AN1061
258	CONTIG16	56270	56697	2.83	8.69E-04	transcription_start_site	+	57856	57856	-1372	5008	AN1061
1604	CONTIG16	54004	54353	1.54	8.01E-02	transcription_start_site	+	57049	57049	-2870	5006	AN1061
1604	CONTIG16	54004	54353	1.54	8.01E-02	transcription_start_site	+	57231	57231	-3052	5007	AN1061
1604	CONTIG16	54004	54353	1.54	8.01E-02	transcription_start_site	+	57856	57856	-3677	5008	AN1061
2244	CONTIG84	459826	460255	1.29	1.79E-01	transcription_start_site	+	464790	464790	-4749	5014	AN10611
2244	CONTIG84	459826	460255	1.29	1.79E-01	transcription_start_site	+	464973	464973	-4932	5015	AN10611
322	CONTIG84	149259	149619	2.73	1.54E-03	transcription_start_site	+	153421	153421	-3982	5028	AN10614
322	CONTIG84	149259	149619	2.73	1.54E-03	transcription_start_site	+	153699	153699	-4260	5029	AN10614
322	CONTIG84	149259	149619	2.73	1.54E-03	transcription_start_site	+	153841	153841	-4402	5030	AN10614
322	CONTIG84	149259	149619	2.73	1.54E-03	transcription_start_site	+	154069	154069	-4630	5031	AN10614
322	CONTIG84	149259	149619	2.73	1.54E-03	transcription_start_site	+	154273	154273	-4834	5032	AN10614
1483	CONTIG84	151671	151940	1.6	8.01E-02	transcription_start_site	+	153421	153421	-1615	5028	AN10614
1483	CONTIG84	151671	151940	1.6	8.01E-02	transcription_start_site	+	153699	153699	-1893	5029	AN10614
1483	CONTIG84	151671	151940	1.6	8.01E-02	transcription_start_site	+	153841	153841	-2035	5030	AN10614
1483	CONTIG84	151671	151940	1.6	8.01E-02	transcription_start_site	+	154069	154069	-2263	5031	AN10614
1483	CONTIG84	151671	151940	1.6	8.01E-02	transcription_start_site	+	154273	154273	-2467	5032	AN10614
1484	CONTIG84	152781	153275	1.6	8.01E-02	transcription_start_site	+	153421	153421	-393	5028	AN10614
1484	CONTIG84	152781	153275	1.6	8.01E-02	transcription_start_site	+	153699	153699	-671	5029	AN10614
1484	CONTIG84	152781	153275	1.6	8.01E-02	transcription_start_site	+	153841	153841	-813	5030	AN10614
1484	CONTIG84	152781	153275	1.6	8.01E-02	transcription_start_site	+	154069	154069	-1041	5031	AN10614
1484	CONTIG84	152781	153275	1.6	8.01E-02	transcription_start_site	+	154273	154273	-1245	5032	AN10614
1702	CONTIG84	148144	148718	1.5	9.26E-02	transcription_start_site	+	153421	153421	-4990	5028	AN10614
1702	CONTIG84	148144	148718	1.5	9.26E-02	transcription_start_site	+	153699	153699	-5268	5029	AN10614
2097	CONTIG84	153996	154485	1.34	1.66E-01	transcription_start_site	+	154273	154273	-32	5032	AN10614
2097	CONTIG84	153996	154485	1.34	1.66E-01	transcription_start_site	+	154069	154069	171	5031	AN10614
2097	CONTIG84	153996	154485	1.34	1.66E-01	transcription_start_site	+	153841	153841	399	5030	AN10614
2097	CONTIG84	153996	154485	1.34	1.66E-01	transcription_start_site	+	153699	153699	541	5029	AN10614
2097	CONTIG84	153996	154485	1.34	1.66E-01	transcription_start_site	+	153421	153421	819	5028	AN10614
1961	CONTIG84	172135	172479	1.39	1.44E-01	transcription_start_site	-	167491	167491	-4816	5033	AN10615
1961	CONTIG84	172135	172479	1.39	1.44E-01	transcription_start_site	-	167288	167288	-5019	5034	AN10615
1705	CONTIG84	3090	3954	1.5	1.08E-01	transcription_start_site	+	3578	3578	-56	5040	AN10616
1705	CONTIG84	3090	3954	1.5	1.08E-01	transcription_start_site	+	3711	3711	-189	5041	AN10616
1960	CONTIG84	4070	4629	1.39	1.44E-01	transcription_start_site	+	3711	3711	638	5041	AN10616

1960	CONTIG84	4070	4629	1.39	1.44E-01	transcription_start_site	+	3578	3578	771	5040	AN10616
258	CONTIG16	56270	56697	2.83	8.69E-04	transcription_start_site	+	59434	59434	-2950	5048	AN1062
258	CONTIG16	56270	56697	2.83	8.69E-04	transcription_start_site	+	59623	59623	-3139	5049	AN1062
258	CONTIG16	56270	56697	2.83	8.69E-04	transcription_start_site	+	59752	59752	-3268	5050	AN1062
258	CONTIG16	56270	56697	2.83	8.69E-04	transcription_start_site	+	60460	60460	-3976	5051	AN1062
1708	CONTIG84	455251	456808	1.5	1.08E-01	transcription_start_site	+	460753	460753	-4723	5055	AN10621
1708	CONTIG84	455251	456808	1.5	1.08E-01	transcription_start_site	+	461010	461010	-4980	5056	AN10621
2244	CONTIG84	459826	460255	1.29	1.79E-01	transcription_start_site	+	460753	460753	-712	5055	AN10621
2244	CONTIG84	459826	460255	1.29	1.79E-01	transcription_start_site	+	461010	461010	-969	5056	AN10621
1707	CONTIG84	447762	448101	1.5	1.08E-01	transcription_start_site	-	443667	443667	-4264	5057	AN10622
1707	CONTIG84	447762	448101	1.5	1.08E-01	transcription_start_site	-	443568	443568	-4363	5058	AN10622
1707	CONTIG84	447762	448101	1.5	1.08E-01	transcription_start_site	-	443476	443476	-4455	5059	AN10622
1707	CONTIG84	447762	448101	1.5	1.08E-01	transcription_start_site	-	443324	443324	-4607	5060	AN10622
267	CONTIG86	66230	66890	2.81	2.22E-03	transcription_start_site	-	62640	62640	-3920	5067	AN10625
267	CONTIG86	66230	66890	2.81	2.22E-03	transcription_start_site	-	62270	62270	-4290	5068	AN10625
267	CONTIG86	66230	66890	2.81	2.22E-03	transcription_start_site	-	61380	61380	-5180	5069	AN10625
313	CONTIG86	74490	74851	2.75	3.03E-03	transcription_start_site	+	74783	74783	-112	5070	AN10626
1609	CONTIG86	79067	79558	1.54	1.07E-01	transcription_start_site	+	81288	81288	-1975	5077	AN10628
1609	CONTIG86	79067	79558	1.54	1.07E-01	transcription_start_site	+	81408	81408	-2095	5078	AN10628
313	CONTIG86	74490	74851	2.75	3.03E-03	transcription_start_site	+	79549	79549	-4878	5079	AN10629
313	CONTIG86	74490	74851	2.75	3.03E-03	transcription_start_site	+	79755	79755	-5084	5080	AN10629
1609	CONTIG86	79067	79558	1.54	1.07E-01	transcription_start_site	+	79549	79549	-236	5079	AN10629
1609	CONTIG86	79067	79558	1.54	1.07E-01	transcription_start_site	+	79755	79755	-442	5080	AN10629
1609	CONTIG86	79067	79558	1.54	1.07E-01	transcription_start_site	+	80668	80668	-1355	5081	AN10629
1834	CONTIG16	66685	67264	1.44	9.26E-02	transcription_start_site	-	62090	62090	-4884	5082	AN1063
1834	CONTIG16	66685	67264	1.44	9.26E-02	transcription_start_site	-	61972	61972	-5002	5083	AN1063
756	CONTIG86	96396	96808	2.15	1.06E-02	transcription_start_site	+	95909	95909	693	5088	AN10630
756	CONTIG86	96396	96808	2.15	1.06E-02	transcription_start_site	+	95616	95616	986	5087	AN10630
313	CONTIG86	74490	74851	2.75	3.03E-03	transcription_start_site	+	74090	74090	580	5091	AN10631
313	CONTIG86	74490	74851	2.75	3.03E-03	transcription_start_site	+	73897	73897	773	5090	AN10631
313	CONTIG86	74490	74851	2.75	3.03E-03	transcription_start_site	+	73656	73656	1014	5089	AN10631
2370	CONTIG88	113566	113991	1.24	8.01E-02	transcription_start_site	+	113762	113762	16	5096	AN10634
2370	CONTIG88	113566	113991	1.24	8.01E-02	transcription_start_site	+	114016	114016	-237	5097	AN10634
2371	CONTIG88	123246	124555	1.24	8.01E-02	transcription_start_site	+	123123	123123	777	5105	AN10635
2371	CONTIG88	123246	124555	1.24	8.01E-02	transcription_start_site	+	122781	122781	1119	5104	AN10635
2371	CONTIG88	123246	124555	1.24	8.01E-02	transcription_start_site	+	122603	122603	1297	5103	AN10635
2371	CONTIG88	123246	124555	1.24	8.01E-02	transcription_start_site	+	122424	122424	1476	5102	AN10635
1035	CONTIG88	139581	139935	1.88	2.32E-03	transcription_start_site	+	143713	143713	-3955	5110	AN10638
1035	CONTIG88	139581	139935	1.88	2.32E-03	transcription_start_site	+	143865	143865	-4107	5111	AN10638
1035	CONTIG88	139581	139935	1.88	2.32E-03	transcription_start_site	+	144328	144328	-4570	5112	AN10638
1035	CONTIG88	139581	139935	1.88	2.32E-03	transcription_start_site	+	144587	144587	-4829	5113	AN10638
1035	CONTIG88	139581	139935	1.88	2.32E-03	transcription_start_site	+	144668	144668	-4910	5114	AN10638
2850	CONTIG88	141236	141520	1.04	1.66E-01	transcription_start_site	+	143713	143713	-2335	5110	AN10638
2850	CONTIG88	141236	141520	1.04	1.66E-01	transcription_start_site	+	143865	143865	-2487	5111	AN10638
2850	CONTIG88	141236	141520	1.04	1.66E-01	transcription_start_site	+	144328	144328	-2950	5112	AN10638
2850	CONTIG88	141236	141520	1.04	1.66E-01	transcription_start_site	+	144587	144587	-3209	5113	AN10638
2850	CONTIG88	141236	141520	1.04	1.66E-01	transcription_start_site	+	144668	144668	-3290	5114	AN10638
2850	CONTIG88	141236	141520	1.04	1.66E-01	transcription_start_site	+	146001	146001	-4623	5115	AN10638
2850	CONTIG88	141236	141520	1.04	1.66E-01	transcription_start_site	+	146215	146215	-4837	5116	AN10638
1312	CONTIG88	151818	152092	1.68	1.30E-02	transcription_start_site	-	152865	152865	910	5118	AN10639
1136	CONTIG88	159019	159293	1.8	7.85E-03	transcription_start_site	-	154246	154246	-4910	5135	AN10644
2371	CONTIG88	123246	124555	1.24	8.01E-02	transcription_start_site	+	124037	124037	-136	5138	AN10645
2371	CONTIG88	123246	124555	1.24	8.01E-02	transcription_start_site	+	124375	124375	-474	5139	AN10645
449	CONTIG88	172070	172559	2.52	0.00E+00	transcription_start_site	+	176625	176625	-4310	5140	AN10646
2370	CONTIG88	113566	113991	1.24	8.01E-02	transcription_start_site	+	116653	116653	-2874	5142	AN10647
2370	CONTIG88	113566	113991	1.24	8.01E-02	transcription_start_site	+	116935	116935	-3156	5143	AN10647
463	CONTIG89	99088	99960	2.49	1.01E-03	transcription_start_site	-	95559	95559	-3965	5144	AN10648

463	CONTIG89	99088	99960	2.49	1.01E-03	transcription_start_site	-	95406	95406	-4118	5145	AN10648
463	CONTIG89	99088	99960	2.49	1.01E-03	transcription_start_site	-	94283	94283	-5241	5146	AN10648
1125	CONTIG89	268585	269387	1.81	1.84E-02	transcription_start_site	-	269542	269542	556	5152	AN10649
1125	CONTIG89	268585	269387	1.81	1.84E-02	transcription_start_site	-	269646	269646	660	5151	AN10649
1125	CONTIG89	268585	269387	1.81	1.84E-02	transcription_start_site	-	269820	269820	834	5150	AN10649
1834	CONTIG16	66685	67264	1.44	9.26E-02	transcription_start_site	+	66341	66341	633	5156	AN1065
1834	CONTIG16	66685	67264	1.44	9.26E-02	transcription_start_site	+	65795	65795	1179	5155	AN1065
2171	CONTIG89	162995	163264	1.31	1.08E-01	transcription_start_site	-	162314	162314	-815	5161	AN10651
2171	CONTIG89	162995	163264	1.31	1.08E-01	transcription_start_site	-	162232	162232	-897	5162	AN10651
2171	CONTIG89	162995	163264	1.31	1.08E-01	transcription_start_site	-	162114	162114	-1015	5163	AN10651
2171	CONTIG89	162995	163264	1.31	1.08E-01	transcription_start_site	-	161900	161900	-1229	5164	AN10651
1834	CONTIG16	66685	67264	1.44	9.26E-02	transcription_start_site	+	67043	67043	-68	5184	AN1066
1834	CONTIG16	66685	67264	1.44	9.26E-02	transcription_start_site	+	67163	67163	-188	5185	AN1066
346	CONTIG93	264980	265344	2.69	8.69E-04	transcription_start_site	-	260351	260351	-4811	5192	AN10662
346	CONTIG93	264980	265344	2.69	8.69E-04	transcription_start_site	-	260169	260169	-4993	5193	AN10662
346	CONTIG93	264980	265344	2.69	8.69E-04	transcription_start_site	-	260094	260094	-5068	5194	AN10662
872	CONTIG93	258170	258504	2.03	1.11E-02	transcription_start_site	-	259069	259069	732	5198	AN10662
872	CONTIG93	258170	258504	2.03	1.11E-02	transcription_start_site	-	258033	258033	-304	5201	AN10664
872	CONTIG93	258170	258504	2.03	1.11E-02	transcription_start_site	-	257824	257824	-513	5202	AN10664
829	CONTIG93	25428	25867	2.07	8.81E-03	transcription_start_site	-	21307	21307	-4340	5203	AN10665
829	CONTIG93	25428	25867	2.07	8.81E-03	transcription_start_site	-	21069	21069	-4578	5204	AN10665
829	CONTIG93	25428	25867	2.07	8.81E-03	transcription_start_site	-	20891	20891	-4756	5205	AN10665
829	CONTIG93	25428	25867	2.07	8.81E-03	transcription_start_site	-	20776	20776	-4871	5206	AN10665
829	CONTIG93	25428	25867	2.07	8.81E-03	transcription_start_site	-	20655	20655	-4992	5207	AN10665
498	CONTIG94	14329	14603	2.45	2.39E-04	transcription_start_site	+	13336	13336	1130	5228	AN10671
2851	CONTIG94	30301	30810	1.04	1.66E-01	transcription_start_site	+	29383	29383	1172	5234	AN10672
2610	CONTIG94	171314	171750	1.16	1.08E-01	transcription_start_site	-	169494	169494	-2038	5235	AN10673
2610	CONTIG94	171314	171750	1.16	1.08E-01	transcription_start_site	-	169030	169030	-2502	5236	AN10673
2610	CONTIG94	171314	171750	1.16	1.08E-01	transcription_start_site	-	166684	166684	-4848	5237	AN10673
2610	CONTIG94	171314	171750	1.16	1.08E-01	transcription_start_site	-	166431	166431	-5101	5238	AN10673
933	CONTIG94	442968	443467	1.97	3.61E-03	transcription_start_site	-	440647	440647	-2570	5244	AN10676
933	CONTIG94	442968	443467	1.97	3.61E-03	transcription_start_site	-	440495	440495	-2722	5245	AN10676
933	CONTIG94	442968	443467	1.97	3.61E-03	transcription_start_site	-	440404	440404	-2813	5246	AN10676
933	CONTIG94	442968	443467	1.97	3.61E-03	transcription_start_site	-	439380	439380	-3837	5247	AN10676
1516	CONTIG94	441678	441958	1.57	2.12E-02	transcription_start_site	-	440647	440647	-1171	5244	AN10676
1516	CONTIG94	441678	441958	1.57	2.12E-02	transcription_start_site	-	440495	440495	-1323	5245	AN10676
1516	CONTIG94	441678	441958	1.57	2.12E-02	transcription_start_site	-	440404	440404	-1414	5246	AN10676
1516	CONTIG94	441678	441958	1.57	2.12E-02	transcription_start_site	-	439380	439380	-2438	5247	AN10676
2250	CONTIG94	440788	441657	1.28	5.05E-02	transcription_start_site	-	440647	440647	-575	5244	AN10676
2250	CONTIG94	440788	441657	1.28	5.05E-02	transcription_start_site	-	440495	440495	-727	5245	AN10676
2250	CONTIG94	440788	441657	1.28	5.05E-02	transcription_start_site	-	440404	440404	-818	5246	AN10676
2250	CONTIG94	440788	441657	1.28	5.05E-02	transcription_start_site	-	439380	439380	-1842	5247	AN10676
2598	CONTIG94	443943	445102	1.16	3.83E-02	transcription_start_site	-	440647	440647	-3875	5244	AN10676
2598	CONTIG94	443943	445102	1.16	3.83E-02	transcription_start_site	-	440495	440495	-4027	5245	AN10676
2598	CONTIG94	443943	445102	1.16	3.83E-02	transcription_start_site	-	440404	440404	-4118	5246	AN10676
2598	CONTIG94	443943	445102	1.16	3.83E-02	transcription_start_site	-	439380	439380	-5142	5247	AN10676
2783	CONTIG94	441993	442637	1.08	1.44E-01	transcription_start_site	-	440647	440647	-1668	5244	AN10676
2783	CONTIG94	441993	442637	1.08	1.44E-01	transcription_start_site	-	440495	440495	-1820	5245	AN10676
2783	CONTIG94	441993	442637	1.08	1.44E-01	transcription_start_site	-	440404	440404	-1911	5246	AN10676
2783	CONTIG94	441993	442637	1.08	1.44E-01	transcription_start_site	-	439380	439380	-2935	5247	AN10676
972	CONTIG94	474392	474726	1.93	4.61E-03	transcription_start_site	+	477222	477222	-2663	5248	AN10677
972	CONTIG94	474392	474726	1.93	4.61E-03	transcription_start_site	+	477625	477625	-3066	5249	AN10677
972	CONTIG94	474392	474726	1.93	4.61E-03	transcription_start_site	+	477699	477699	-3140	5250	AN10677
972	CONTIG94	474392	474726	1.93	4.61E-03	transcription_start_site	+	477761	477761	-3202	5251	AN10677
972	CONTIG94	474392	474726	1.93	4.61E-03	transcription_start_site	+	478429	478429	-3870	5252	AN10677
972	CONTIG94	474392	474726	1.93	4.61E-03	transcription_start_site	+	478566	478566	-4007	5253	AN10677
972	CONTIG94	474392	474726	1.93	4.61E-03	transcription_start_site	+	478736	478736	-4177	5254	AN10677

2851	CONTIG94	30301	30810	1.04	1.66E-01	transcription_start_site	+	30446	30446	109	5259	AN10679
2851	CONTIG94	30301	30810	1.04	1.66E-01	transcription_start_site	+	30321	30321	234	5258	AN10679
1834	CONTIG16	66685	67264	1.44	9.26E-02	transcription_start_site	+	69467	69467	-2492	5260	AN1068
1834	CONTIG16	66685	67264	1.44	9.26E-02	transcription_start_site	+	69652	69652	-2677	5261	AN1068
1834	CONTIG16	66685	67264	1.44	9.26E-02	transcription_start_site	+	69857	69857	-2882	5262	AN1068
2610	CONTIG94	171314	171750	1.16	1.08E-01	transcription_start_site	-	171668	171668	136	5263	AN10680
2610	CONTIG94	171314	171750	1.16	1.08E-01	transcription_start_site	-	171277	171277	-255	5264	AN10680
2373	CONTIG94	422102	422766	1.24	8.01E-02	transcription_start_site	+	421912	421912	522	5280	AN10684
498	CONTIG94	14329	14603	2.45	2.39E-04	transcription_start_site	+	13584	13584	882	5294	AN10688
498	CONTIG94	14329	14603	2.45	2.39E-04	transcription_start_site	+	13496	13496	970	5293	AN10688
933	CONTIG94	442968	443467	1.97	3.61E-03	transcription_start_site	-	443779	443779	561	5295	AN10689
933	CONTIG94	442968	443467	1.97	3.61E-03	transcription_start_site	-	441317	441317	-1900	5296	AN10689
1516	CONTIG94	441678	441958	1.57	2.12E-02	transcription_start_site	-	441317	441317	-501	5296	AN10689
2250	CONTIG94	440788	441657	1.28	5.05E-02	transcription_start_site	-	441317	441317	94	5296	AN10689
2598	CONTIG94	443943	445102	1.16	3.83E-02	transcription_start_site	-	443779	443779	-743	5295	AN10689
2598	CONTIG94	443943	445102	1.16	3.83E-02	transcription_start_site	-	441317	441317	-3205	5296	AN10689
2783	CONTIG94	441993	442637	1.08	1.44E-01	transcription_start_site	-	441317	441317	-998	5296	AN10689
456	CONTIG95	18686	19325	2.51	1.80E-03	transcription_start_site	-	20057	20057	1051	5330	AN10697
2173	CONTIG95	40818	41152	1.31	1.44E-01	transcription_start_site	-	42088	42088	1103	5339	AN10699
2013	CONTIG98	297227	298123	1.37	1.07E-01	transcription_start_site	-	297779	297779	104	5351	AN10703
462	CONTIG98	470562	471051	2.49	0.00E+00	transcription_start_site	-	467731	467731	-3075	5352	AN10704
462	CONTIG98	470562	471051	2.49	0.00E+00	transcription_start_site	-	467567	467567	-3239	5353	AN10704
462	CONTIG98	470562	471051	2.49	0.00E+00	transcription_start_site	-	467014	467014	-3792	5354	AN10704
1469	CONTIG98	471527	471886	1.61	5.88E-02	transcription_start_site	-	467731	467731	-3975	5352	AN10704
1469	CONTIG98	471527	471886	1.61	5.88E-02	transcription_start_site	-	467567	467567	-4139	5353	AN10704
1469	CONTIG98	471527	471886	1.61	5.88E-02	transcription_start_site	-	467014	467014	-4692	5354	AN10704
1469	CONTIG98	471527	471886	1.61	5.88E-02	transcription_start_site	-	472315	472315	608	5355	AN10705
2023	CONTIG98	477313	477592	1.37	1.25E-01	transcription_start_site	-	472315	472315	-5137	5355	AN10705
2023	CONTIG98	477313	477592	1.37	1.25E-01	transcription_start_site	+	477718	477718	-265	5356	AN10706
2023	CONTIG98	477313	477592	1.37	1.25E-01	transcription_start_site	+	477778	477778	-325	5357	AN10706
2023	CONTIG98	477313	477592	1.37	1.25E-01	transcription_start_site	+	477841	477841	-388	5358	AN10706
2023	CONTIG98	477313	477592	1.37	1.25E-01	transcription_start_site	+	477954	477954	-501	5359	AN10706
1280	CONTIG98	544202	544491	1.71	4.36E-02	transcription_start_site	-	544022	544022	-324	5366	AN10709
1280	CONTIG98	544202	544491	1.71	4.36E-02	transcription_start_site	-	543959	543959	-387	5367	AN10709
1280	CONTIG98	544202	544491	1.71	4.36E-02	transcription_start_site	-	543752	543752	-594	5368	AN10709
1280	CONTIG98	544202	544491	1.71	4.36E-02	transcription_start_site	-	543420	543420	-926	5369	AN10709
1280	CONTIG98	544202	544491	1.71	4.36E-02	transcription_start_site	-	541971	541971	-2375	5370	AN10709
2023	CONTIG98	477313	477592	1.37	1.25E-01	transcription_start_site	+	479004	479004	-1551	5373	AN10710
2023	CONTIG98	477313	477592	1.37	1.25E-01	transcription_start_site	+	479179	479179	-1726	5374	AN10710
2023	CONTIG98	477313	477592	1.37	1.25E-01	transcription_start_site	+	479297	479297	-1844	5375	AN10710
2023	CONTIG98	477313	477592	1.37	1.25E-01	transcription_start_site	+	479665	479665	-2212	5376	AN10710
2023	CONTIG98	477313	477592	1.37	1.25E-01	transcription_start_site	+	479799	479799	-2346	5377	AN10710
2023	CONTIG98	477313	477592	1.37	1.25E-01	transcription_start_site	+	480462	480462	-3009	5378	AN10711
2023	CONTIG98	477313	477592	1.37	1.25E-01	transcription_start_site	+	481083	481083	-3630	5379	AN10711
2023	CONTIG98	477313	477592	1.37	1.25E-01	transcription_start_site	+	481467	481467	-4014	5380	AN10711
2458	CONTIG98	283140	283419	1.22	1.93E-01	transcription_start_site	-	279846	279846	-3433	5387	AN10713
2458	CONTIG98	283140	283419	1.22	1.93E-01	transcription_start_site	-	279302	279302	-3977	5388	AN10713
2458	CONTIG98	283140	283419	1.22	1.93E-01	transcription_start_site	-	278821	278821	-4458	5389	AN10713
2023	CONTIG98	477313	477592	1.37	1.25E-01	transcription_start_site	+	482302	482302	-4849	5391	AN10715
1280	CONTIG98	544202	544491	1.71	4.36E-02	transcription_start_site	-	541069	541069	-3277	5400	AN10718
1280	CONTIG98	544202	544491	1.71	4.36E-02	transcription_start_site	-	540975	540975	-3371	5401	AN10718
1280	CONTIG98	544202	544491	1.71	4.36E-02	transcription_start_site	-	540712	540712	-3634	5402	AN10718
1015	CONTIG98	553371	553630	1.9	2.12E-02	transcription_start_site	+	554118	554118	-617	5403	AN10719
1015	CONTIG98	553371	553630	1.9	2.12E-02	transcription_start_site	+	554278	554278	-777	5404	AN10719
1015	CONTIG98	553371	553630	1.9	2.12E-02	transcription_start_site	+	554456	554456	-955	5405	AN10719
1015	CONTIG98	553371	553630	1.9	2.12E-02	transcription_start_site	-	552138	552138	-1362	5416	AN10721
1015	CONTIG98	553371	553630	1.9	2.12E-02	transcription_start_site	-	551877	551877	-1623	5417	AN10721

903	CONTIG98	210002	210593	2	1.57E-02	transcription_start_site	-	207212	207212	-3085	5418 AN10722
903	CONTIG98	210002	210593	2	1.57E-02	transcription_start_site	-	206918	206918	-3379	5419 AN10722
903	CONTIG98	210002	210593	2	1.57E-02	transcription_start_site	-	206651	206651	-3646	5420 AN10722
903	CONTIG98	210002	210593	2	1.57E-02	transcription_start_site	-	206552	206552	-3745	5421 AN10722
903	CONTIG98	210002	210593	2	1.57E-02	transcription_start_site	-	206099	206099	-4198	5422 AN10722
903	CONTIG98	210002	210593	2	1.57E-02	transcription_start_site	-	205957	205957	-4340	5423 AN10722
903	CONTIG98	210002	210593	2	1.57E-02	transcription_start_site	-	205361	205361	-4936	5424 AN10722
1084	CONTIG98	210757	211331	1.85	2.62E-02	transcription_start_site	-	207212	207212	-3832	5418 AN10722
1084	CONTIG98	210757	211331	1.85	2.62E-02	transcription_start_site	-	206918	206918	-4126	5419 AN10722
1084	CONTIG98	210757	211331	1.85	2.62E-02	transcription_start_site	-	206651	206651	-4393	5420 AN10722
1084	CONTIG98	210757	211331	1.85	2.62E-02	transcription_start_site	-	206552	206552	-4492	5421 AN10722
1084	CONTIG98	210757	211331	1.85	2.62E-02	transcription_start_site	-	206099	206099	-4945	5422 AN10722
1084	CONTIG98	210757	211331	1.85	2.62E-02	transcription_start_site	-	205957	205957	-5087	5423 AN10722
1469	CONTIG98	471527	471886	1.61	5.88E-02	transcription_start_site	-	472866	472866	1159	5431 AN10724
2023	CONTIG98	477313	477592	1.37	1.25E-01	transcription_start_site	-	472866	472866	-4586	5431 AN10724
539	CONTIG98	427893	428465	2.39	4.46E-04	transcription_start_site	+	431937	431937	-3758	5432 AN10725
2310	CONTIG98	432467	432741	1.27	1.66E-01	transcription_start_site	+	431937	431937	667	5432 AN10725
63	CONTIG98	436515	437082	3.37	0.00E+00	transcription_start_site	+	438739	438739	-1940	5433 AN10726
412	CONTIG98	433953	434251	2.59	1.54E-03	transcription_start_site	+	438739	438739	-4637	5433 AN10726
701	CONTIG98	434343	435442	2.19	1.28E-03	transcription_start_site	+	438739	438739	-3846	5433 AN10726
63	CONTIG98	436515	437082	3.37	0.00E+00	transcription_start_site	+	440867	440867	-4068	5434 AN10727
462	CONTIG98	470562	471051	2.49	0.00E+00	transcription_start_site	-	465885	465885	-4921	5443 AN10730
462	CONTIG98	470562	471051	2.49	0.00E+00	transcription_start_site	-	465761	465761	-5045	5444 AN10730
462	CONTIG98	470562	471051	2.49	0.00E+00	transcription_start_site	-	465622	465622	-5184	5445 AN10730
1468	CONTIG98	196512	196866	1.61	5.88E-02	transcription_start_site	-	194437	194437	-2252	5446 AN10731
1468	CONTIG98	196512	196866	1.61	5.88E-02	transcription_start_site	-	194163	194163	-2526	5447 AN10731
1907	CONTIG98	199292	199865	1.41	9.26E-02	transcription_start_site	-	194437	194437	-5141	5446 AN10731
2021	CONTIG98	197027	197606	1.37	1.25E-01	transcription_start_site	-	194437	194437	-2879	5446 AN10731
2021	CONTIG98	197027	197606	1.37	1.25E-01	transcription_start_site	-	194163	194163	-3153	5447 AN10731
354	CONTIG100	194927	195366	2.67	0.00E+00	transcription_start_site	+	195680	195680	-533	5463 AN10735
142	CONTIG100	218628	218987	3.07	2.67E-04	transcription_start_site	-	216760	216760	-2047	5464 AN10736
142	CONTIG100	218628	218987	3.07	2.67E-04	transcription_start_site	-	216055	216055	-2752	5465 AN10736
142	CONTIG100	218628	218987	3.07	2.67E-04	transcription_start_site	-	215666	215666	-3141	5466 AN10736
975	CONTIG100	219528	220192	1.93	1.06E-02	transcription_start_site	-	216760	216760	-3100	5464 AN10736
975	CONTIG100	219528	220192	1.93	1.06E-02	transcription_start_site	-	216055	216055	-3805	5465 AN10736
975	CONTIG100	219528	220192	1.93	1.06E-02	transcription_start_site	-	215666	215666	-4194	5466 AN10736
2238	CONTIG100	215868	216362	1.29	1.66E-01	transcription_start_site	-	216055	216055	-60	5465 AN10736
2238	CONTIG100	215868	216362	1.29	1.66E-01	transcription_start_site	-	215666	215666	-449	5466 AN10736
2238	CONTIG100	215868	216362	1.29	1.66E-01	transcription_start_site	-	216760	216760	645	5464 AN10736
2391	CONTIG100	216463	217112	1.24	1.79E-01	transcription_start_site	-	216760	216760	-27	5464 AN10736
2391	CONTIG100	216463	217112	1.24	1.79E-01	transcription_start_site	-	216055	216055	-732	5465 AN10736
2391	CONTIG100	216463	217112	1.24	1.79E-01	transcription_start_site	-	215666	215666	-1121	5466 AN10736
1245	CONTIG100	262221	262705	1.73	2.64E-02	transcription_start_site	+	263339	263339	-876	5479 AN10742
263	CONTIG100	250746	251231	2.82	0.00E+00	transcription_start_site	-	249162	249162	-1826	5480 AN10743
263	CONTIG100	250746	251231	2.82	0.00E+00	transcription_start_site	-	249010	249010	-1978	5481 AN10743
263	CONTIG100	250746	251231	2.82	0.00E+00	transcription_start_site	-	248805	248805	-2183	5482 AN10743
263	CONTIG100	250746	251231	2.82	0.00E+00	transcription_start_site	-	248197	248197	-2791	5483 AN10743
263	CONTIG100	250746	251231	2.82	0.00E+00	transcription_start_site	-	248015	248015	-2973	5484 AN10743
1603	CONTIG100	249921	250475	1.54	8.01E-02	transcription_start_site	-	249162	249162	-1036	5480 AN10743
1603	CONTIG100	249921	250475	1.54	8.01E-02	transcription_start_site	-	249010	249010	-1188	5481 AN10743
1603	CONTIG100	249921	250475	1.54	8.01E-02	transcription_start_site	-	248805	248805	-1393	5482 AN10743
1603	CONTIG100	249921	250475	1.54	8.01E-02	transcription_start_site	-	248197	248197	-2001	5483 AN10743
1603	CONTIG100	249921	250475	1.54	8.01E-02	transcription_start_site	-	248015	248015	-2183	5484 AN10743
1107	CONTIG100	106366	106870	1.83	1.20E-02	transcription_start_site	+	107680	107680	-1062	5485 AN10744
2393	CONTIG100	108076	108425	1.24	1.93E-01	transcription_start_site	+	107680	107680	570	5485 AN10744
1107	CONTIG100	106366	106870	1.83	1.20E-02	transcription_start_site	+	107973	107973	-1355	5486 AN10745
1107	CONTIG100	106366	106870	1.83	1.20E-02	transcription_start_site	+	108399	108399	-1781	5487 AN10745

1107	CONTIG100	106366	106870	1.83	1.20E-02	transcription_start_site	+	108509	108509	-1891	5488	AN10745
1107	CONTIG100	106366	106870	1.83	1.20E-02	transcription_start_site	+	108649	108649	-2031	5489	AN10745
1107	CONTIG100	106366	106870	1.83	1.20E-02	transcription_start_site	+	108833	108833	-2215	5490	AN10745
2087	CONTIG100	108921	109405	1.34	1.44E-01	transcription_start_site	+	108833	108833	330	5490	AN10745
2087	CONTIG100	108921	109405	1.34	1.44E-01	transcription_start_site	+	108649	108649	514	5489	AN10745
2087	CONTIG100	108921	109405	1.34	1.44E-01	transcription_start_site	+	108509	108509	654	5488	AN10745
2087	CONTIG100	108921	109405	1.34	1.44E-01	transcription_start_site	+	108399	108399	764	5487	AN10745
2087	CONTIG100	108921	109405	1.34	1.44E-01	transcription_start_site	+	107973	107973	1190	5486	AN10745
2393	CONTIG100	108076	108425	1.24	1.93E-01	transcription_start_site	+	108399	108399	-148	5487	AN10745
2393	CONTIG100	108076	108425	1.24	1.93E-01	transcription_start_site	+	108509	108509	-258	5488	AN10745
2393	CONTIG100	108076	108425	1.24	1.93E-01	transcription_start_site	+	107973	107973	277	5486	AN10745
2393	CONTIG100	108076	108425	1.24	1.93E-01	transcription_start_site	+	108649	108649	-398	5489	AN10745
2393	CONTIG100	108076	108425	1.24	1.93E-01	transcription_start_site	+	108833	108833	-582	5490	AN10745
1107	CONTIG100	106366	106870	1.83	1.20E-02	transcription_start_site	+	110315	110315	-3697	5491	AN10746
1107	CONTIG100	106366	106870	1.83	1.20E-02	transcription_start_site	+	110442	110442	-3824	5492	AN10746
2087	CONTIG100	108921	109405	1.34	1.44E-01	transcription_start_site	+	110315	110315	-1152	5491	AN10746
2087	CONTIG100	108921	109405	1.34	1.44E-01	transcription_start_site	+	110442	110442	-1279	5492	AN10746
2393	CONTIG100	108076	108425	1.24	1.93E-01	transcription_start_site	+	110315	110315	-2064	5491	AN10746
2393	CONTIG100	108076	108425	1.24	1.93E-01	transcription_start_site	+	110442	110442	-2191	5492	AN10746
1177	CONTIG100	111466	111725	1.78	3.70E-02	transcription_start_site	+	111991	111991	-395	5493	AN10747
1177	CONTIG100	111466	111725	1.78	3.70E-02	transcription_start_site	+	112121	112121	-525	5494	AN10747
2087	CONTIG100	108921	109405	1.34	1.44E-01	transcription_start_site	+	111991	111991	-2828	5493	AN10747
2087	CONTIG100	108921	109405	1.34	1.44E-01	transcription_start_site	+	112121	112121	-2958	5494	AN10747
2393	CONTIG100	108076	108425	1.24	1.93E-01	transcription_start_site	+	111991	111991	-3740	5493	AN10747
2393	CONTIG100	108076	108425	1.24	1.93E-01	transcription_start_site	+	112121	112121	-3870	5494	AN10747
1245	CONTIG100	262221	262705	1.73	2.64E-02	transcription_start_site	+	266203	266203	-3740	5495	AN10748
1245	CONTIG100	262221	262705	1.73	2.64E-02	transcription_start_site	+	266336	266336	-3873	5496	AN10748
25	CONTIG100	179402	179691	3.57	0.00E+00	transcription_start_site	+	179809	179809	-262	5499	AN10750
25	CONTIG100	179402	179691	3.57	0.00E+00	transcription_start_site	+	183485	183485	-3938	5500	AN10750
511	CONTIG100	180237	180591	2.43	3.61E-03	transcription_start_site	+	179809	179809	605	5499	AN10750
511	CONTIG100	180237	180591	2.43	3.61E-03	transcription_start_site	+	183485	183485	-3071	5500	AN10750
142	CONTIG100	218628	218987	3.07	2.67E-04	transcription_start_site	-	217841	217841	-966	5501	AN10751
142	CONTIG100	218628	218987	3.07	2.67E-04	transcription_start_site	-	217504	217504	-1303	5502	AN10751
975	CONTIG100	219528	220192	1.93	1.06E-02	transcription_start_site	-	217841	217841	-2019	5501	AN10751
975	CONTIG100	219528	220192	1.93	1.06E-02	transcription_start_site	-	217504	217504	-2356	5502	AN10751
2391	CONTIG100	216463	217112	1.24	1.79E-01	transcription_start_site	-	217504	217504	716	5502	AN10751
2391	CONTIG100	216463	217112	1.24	1.79E-01	transcription_start_site	-	217841	217841	1053	5501	AN10751
263	CONTIG100	250746	251231	2.82	0.00E+00	transcription_start_site	-	250921	250921	-67	5503	AN10752
263	CONTIG100	250746	251231	2.82	0.00E+00	transcription_start_site	-	250293	250293	-695	5504	AN10752
263	CONTIG100	250746	251231	2.82	0.00E+00	transcription_start_site	-	250148	250148	-840	5505	AN10752
263	CONTIG100	250746	251231	2.82	0.00E+00	transcription_start_site	-	249661	249661	-1327	5506	AN10752
1603	CONTIG100	249921	250475	1.54	8.01E-02	transcription_start_site	-	250148	250148	-50	5505	AN10752
1603	CONTIG100	249921	250475	1.54	8.01E-02	transcription_start_site	-	250293	250293	95	5504	AN10752
1603	CONTIG100	249921	250475	1.54	8.01E-02	transcription_start_site	-	249661	249661	-537	5506	AN10752
1603	CONTIG100	249921	250475	1.54	8.01E-02	transcription_start_site	-	250921	250921	723	5503	AN10752
2224	CONTIG101	65928	66277	1.29	1.25E-01	transcription_start_site	+	70442	70442	-4339	5507	AN10753
2224	CONTIG101	65928	66277	1.29	1.25E-01	transcription_start_site	+	70909	70909	-4806	5508	AN10753
1210	CONTIG101	192615	192959	1.75	1.44E-02	transcription_start_site	-	193364	193364	577	5524	AN10758
2226	CONTIG101	179565	179909	1.29	1.25E-01	transcription_start_site	+	181851	181851	-2114	5525	AN10759
2224	CONTIG101	65928	66277	1.29	1.25E-01	transcription_start_site	+	67035	67035	-932	5528	AN10760
2224	CONTIG101	65928	66277	1.29	1.25E-01	transcription_start_site	+	69193	69193	-3090	5529	AN10760
2224	CONTIG101	65928	66277	1.29	1.25E-01	transcription_start_site	+	69524	69524	-3421	5530	AN10760
685	CONTIG101	166503	167242	2.21	4.61E-03	transcription_start_site	+	167370	167370	-497	5531	AN10761
685	CONTIG101	166503	167242	2.21	4.61E-03	transcription_start_site	+	167529	167529	-656	5532	AN10761
1770	CONTIG101	167263	168353	1.47	4.42E-02	transcription_start_site	+	167529	167529	279	5532	AN10761
1770	CONTIG101	167263	168353	1.47	4.42E-02	transcription_start_site	+	167370	167370	438	5531	AN10761
2516	CONTIG101	102154	102441	1.2	1.66E-01	transcription_start_site	-	102640	102640	342	5541	AN10762

2516	CONTIG101	102154	102441	1.2	1.66E-01	transcription_start_site	-	102928	102928	630	5540 AN10762
2516	CONTIG101	102154	102441	1.2	1.66E-01	transcription_start_site	-	103101	103101	803	5539 AN10762
2516	CONTIG101	102154	102441	1.2	1.66E-01	transcription_start_site	-	103226	103226	928	5538 AN10762
2516	CONTIG101	102154	102441	1.2	1.66E-01	transcription_start_site	-	103439	103439	1141	5537 AN10762
599	CONTIG102	29181	29537	2.31	6.61E-03	transcription_start_site	+	29808	29808	-449	5542 AN10763
599	CONTIG102	29181	29537	2.31	6.61E-03	transcription_start_site	+	30003	30003	-644	5543 AN10763
599	CONTIG102	29181	29537	2.31	6.61E-03	transcription_start_site	+	31460	31460	-2101	5544 AN10763
599	CONTIG102	29181	29537	2.31	6.61E-03	transcription_start_site	+	31938	31938	-2579	5545 AN10764
599	CONTIG102	29181	29537	2.31	6.61E-03	transcription_start_site	+	32244	32244	-2885	5546 AN10764
1859	CONTIG103	49596	50162	1.43	5.05E-02	transcription_start_site	-	47064	47064	-2815	5551 AN10766
1859	CONTIG103	49596	50162	1.43	5.05E-02	transcription_start_site	-	46814	46814	-3065	5552 AN10766
1859	CONTIG103	49596	50162	1.43	5.05E-02	transcription_start_site	-	46302	46302	-3577	5553 AN10766
1859	CONTIG103	49596	50162	1.43	5.05E-02	transcription_start_site	-	48637	48637	-1242	5556 AN10768
1859	CONTIG103	49596	50162	1.43	5.05E-02	transcription_start_site	-	48111	48111	-1768	5557 AN10768
1859	CONTIG103	49596	50162	1.43	5.05E-02	transcription_start_site	-	47407	47407	-2472	5558 AN10768
1775	CONTIG104	107927	108276	1.47	6.84E-02	transcription_start_site	+	113223	113223	-5121	5575 AN10772
978	CONTIG104	213828	214112	1.93	1.30E-02	transcription_start_site	+	215161	215161	-1191	5576 AN10773
1874	CONTIG104	210303	211192	1.42	5.52E-02	transcription_start_site	+	215161	215161	-4413	5576 AN10773
1608	CONTIG105	224121	224615	1.54	9.24E-02	transcription_start_site	+	229582	229582	-5214	5592 AN10779
1843	CONTIG105	257639	257930	1.44	1.25E-01	transcription_start_site	-	257175	257175	-609	5601 AN10780
1843	CONTIG105	257639	257930	1.44	1.25E-01	transcription_start_site	-	256801	256801	-983	5602 AN10780
1843	CONTIG105	257639	257930	1.44	1.25E-01	transcription_start_site	-	256574	256574	-1210	5603 AN10780
1843	CONTIG105	257639	257930	1.44	1.25E-01	transcription_start_site	-	256106	256106	-1678	5604 AN10780
856	CONTIG105	213085	214794	2.05	3.45E-03	transcription_start_site	-	210716	210716	-3223	5618 AN10786
390	CONTIG105	185336	185675	2.62	2.22E-03	transcription_start_site	+	184734	184734	771	5625 AN10788
1843	CONTIG105	257639	257930	1.44	1.25E-01	transcription_start_site	-	257881	257881	96	5635 AN10790
1381	CONTIG106	22884	23153	1.64	3.08E-02	transcription_start_site	+	22868	22868	150	5639 AN10792
1381	CONTIG106	22884	23153	1.64	3.08E-02	transcription_start_site	+	22714	22714	304	5638 AN10792
1381	CONTIG106	22884	23153	1.64	3.08E-02	transcription_start_site	+	23505	23505	-486	5640 AN10792
1381	CONTIG106	22884	23153	1.64	3.08E-02	transcription_start_site	+	24066	24066	-1047	5641 AN10792
1791	CONTIG106	18319	20168	1.46	3.99E-02	transcription_start_site	+	22714	22714	-3470	5638 AN10792
1791	CONTIG106	18319	20168	1.46	3.99E-02	transcription_start_site	+	22868	22868	-3624	5639 AN10792
1791	CONTIG106	18319	20168	1.46	3.99E-02	transcription_start_site	+	23505	23505	-4261	5640 AN10792
1791	CONTIG106	18319	20168	1.46	3.99E-02	transcription_start_site	+	24066	24066	-4822	5641 AN10792
1381	CONTIG106	22884	23153	1.64	3.08E-02	transcription_start_site	+	25517	25517	-2498	5646 AN10795
1381	CONTIG106	22884	23153	1.64	3.08E-02	transcription_start_site	+	25769	25769	-2750	5647 AN10795
586	CONTIG107	146342	146916	2.33	1.80E-03	transcription_start_site	-	143236	143236	-3393	5653 AN10797
586	CONTIG107	146342	146916	2.33	1.80E-03	transcription_start_site	-	142903	142903	-3726	5654 AN10797
1094	CONTIG16	122268	122614	1.84	3.08E-02	transcription_start_site	-	118395	118395	-4046	5658 AN1080
1094	CONTIG16	122268	122614	1.84	3.08E-02	transcription_start_site	-	117844	117844	-4597	5659 AN1080
261	CONTIG107	338857	339133	2.82	0.00E+00	transcription_start_site	+	340809	340809	-1814	5662 AN10801
261	CONTIG107	338857	339133	2.82	0.00E+00	transcription_start_site	+	340887	340887	-1892	5663 AN10801
261	CONTIG107	338857	339133	2.82	0.00E+00	transcription_start_site	+	341057	341057	-2062	5664 AN10801
261	CONTIG107	338857	339133	2.82	0.00E+00	transcription_start_site	+	341777	341777	-2782	5665 AN10801
261	CONTIG107	338857	339133	2.82	0.00E+00	transcription_start_site	+	342049	342049	-3054	5666 AN10801
651	CONTIG107	339467	339813	2.24	3.03E-03	transcription_start_site	+	340809	340809	-1169	5662 AN10801
651	CONTIG107	339467	339813	2.24	3.03E-03	transcription_start_site	+	340887	340887	-1247	5663 AN10801
651	CONTIG107	339467	339813	2.24	3.03E-03	transcription_start_site	+	341057	341057	-1417	5664 AN10801
651	CONTIG107	339467	339813	2.24	3.03E-03	transcription_start_site	+	341777	341777	-2137	5665 AN10801
651	CONTIG107	339467	339813	2.24	3.03E-03	transcription_start_site	+	342049	342049	-2409	5666 AN10801
586	CONTIG107	146342	146916	2.33	1.80E-03	transcription_start_site	-	141760	141760	-4869	5675 AN10804
586	CONTIG107	146342	146916	2.33	1.80E-03	transcription_start_site	-	141575	141575	-5054	5676 AN10804
2194	CONTIG107	275562	275906	1.3	1.08E-01	transcription_start_site	+	278025	278025	-2291	5682 AN10807
2194	CONTIG107	275562	275906	1.3	1.08E-01	transcription_start_site	+	278643	278643	-2909	5683 AN10807
2194	CONTIG107	275562	275906	1.3	1.08E-01	transcription_start_site	+	278976	278976	-3242	5684 AN10807
2194	CONTIG107	275562	275906	1.3	1.08E-01	transcription_start_site	+	279486	279486	-3752	5685 AN10807
2194	CONTIG107	275562	275906	1.3	1.08E-01	transcription_start_site	+	279863	279863	-4129	5686 AN10807

2714	CONTIG107	277443	278318	1.12	1.79E-01	transcription_start_site	+	278025	278025	-144	5682 AN10807
2714	CONTIG107	277443	278318	1.12	1.79E-01	transcription_start_site	+	278643	278643	-762	5683 AN10807
2714	CONTIG107	277443	278318	1.12	1.79E-01	transcription_start_site	+	278976	278976	-1095	5684 AN10807
2714	CONTIG107	277443	278318	1.12	1.79E-01	transcription_start_site	+	279486	279486	-1605	5685 AN10807
2714	CONTIG107	277443	278318	1.12	1.79E-01	transcription_start_site	+	279863	279863	-1982	5686 AN10807
2719	CONTIG107	276392	277418	1.12	1.99E-01	transcription_start_site	+	278025	278025	-1120	5682 AN10807
2719	CONTIG107	276392	277418	1.12	1.99E-01	transcription_start_site	+	278643	278643	-1738	5683 AN10807
2719	CONTIG107	276392	277418	1.12	1.99E-01	transcription_start_site	+	278976	278976	-2071	5684 AN10807
2719	CONTIG107	276392	277418	1.12	1.99E-01	transcription_start_site	+	279486	279486	-2581	5685 AN10807
2719	CONTIG107	276392	277418	1.12	1.99E-01	transcription_start_site	+	279863	279863	-2958	5686 AN10807
2611	CONTIG107	314026	315055	1.16	1.11E-01	transcription_start_site	-	315677	315677	1136	5688 AN10808
2611	CONTIG107	314026	315055	1.16	1.11E-01	transcription_start_site	-	315815	315815	1274	5687 AN10808
261	CONTIG107	338857	339133	2.82	0.00E+00	transcription_start_site	+	339550	339550	-555	5689 AN10809
261	CONTIG107	338857	339133	2.82	0.00E+00	transcription_start_site	+	340106	340106	-1111	5690 AN10809
651	CONTIG107	339467	339813	2.24	3.03E-03	transcription_start_site	+	339550	339550	90	5689 AN10809
651	CONTIG107	339467	339813	2.24	3.03E-03	transcription_start_site	+	340106	340106	-466	5690 AN10809
135	CONTIG107	364652	365166	3.09	0.00E+00	transcription_start_site	+	369046	369046	-4137	5693 AN10810
1945	CONTIG107	367127	367567	1.39	8.01E-02	transcription_start_site	+	369046	369046	-1699	5693 AN10810
585	CONTIG108	182720	183054	2.33	8.69E-04	transcription_start_site	+	184174	184174	-1287	5724 AN10817
2158	CONTIG108	180685	181109	1.31	4.42E-02	transcription_start_site	+	184174	184174	-3277	5724 AN10817
772	CONTIG108	123841	124100	2.12	1.80E-03	transcription_start_site	-	120277	120277	-3693	5725 AN10818
2280	CONTIG108	122946	123298	1.27	6.16E-02	transcription_start_site	-	120277	120277	-2845	5725 AN10818
1094	CONTIG16	122268	122614	1.84	3.08E-02	transcription_start_site	-	121758	121758	-683	5728 AN1082
1094	CONTIG16	122268	122614	1.84	3.08E-02	transcription_start_site	-	120431	120431	-2010	5729 AN1082
760	CONTIG109	35778	36133	2.14	4.61E-03	transcription_start_site	+	40940	40940	-4984	5743 AN10823
760	CONTIG109	35778	36133	2.14	4.61E-03	transcription_start_site	+	41122	41122	-5166	5744 AN10823
329	CONTIG109	66314	66593	2.72	2.39E-04	transcription_start_site	+	69668	69668	-3214	5746 AN10824
329	CONTIG109	66314	66593	2.72	2.39E-04	transcription_start_site	+	69785	69785	-3331	5747 AN10824
329	CONTIG109	66314	66593	2.72	2.39E-04	transcription_start_site	+	70028	70028	-3574	5748 AN10824
329	CONTIG109	66314	66593	2.72	2.39E-04	transcription_start_site	+	70123	70123	-3669	5749 AN10824
329	CONTIG109	66314	66593	2.72	2.39E-04	transcription_start_site	+	70428	70428	-3974	5750 AN10824
329	CONTIG109	66314	66593	2.72	2.39E-04	transcription_start_site	+	70647	70647	-4193	5751 AN10824
329	CONTIG109	66314	66593	2.72	2.39E-04	transcription_start_site	+	70941	70941	-4487	5752 AN10824
329	CONTIG109	66314	66593	2.72	2.39E-04	transcription_start_site	+	71117	71117	-4663	5753 AN10824
329	CONTIG109	66314	66593	2.72	2.39E-04	transcription_start_site	+	71539	71539	-5085	5754 AN10824
760	CONTIG109	35778	36133	2.14	4.61E-03	transcription_start_site	+	37560	37560	-1604	5762 AN10827
760	CONTIG109	35778	36133	2.14	4.61E-03	transcription_start_site	+	37656	37656	-1700	5763 AN10827
760	CONTIG109	35778	36133	2.14	4.61E-03	transcription_start_site	+	39994	39994	-4038	5764 AN10828
760	CONTIG109	35778	36133	2.14	4.61E-03	transcription_start_site	+	40079	40079	-4123	5765 AN10828
760	CONTIG109	35778	36133	2.14	4.61E-03	transcription_start_site	+	40187	40187	-4231	5766 AN10828
760	CONTIG109	35778	36133	2.14	4.61E-03	transcription_start_site	+	40276	40276	-4320	5767 AN10828
760	CONTIG109	35778	36133	2.14	4.61E-03	transcription_start_site	+	40366	40366	-4410	5768 AN10828
564	CONTIG109	9691	10123	2.36	1.54E-03	transcription_start_site	-	7091	7091	-2816	5769 AN10829
564	CONTIG109	9691	10123	2.36	1.54E-03	transcription_start_site	-	6707	6707	-3200	5770 AN10829
1094	CONTIG16	122268	122614	1.84	3.08E-02	transcription_start_site	+	122624	122624	-183	5771 AN1083
1109	CONTIG109	157061	157502	1.83	1.57E-02	transcription_start_site	+	157392	157392	-110	5772 AN10830
1109	CONTIG109	157061	157502	1.83	1.57E-02	transcription_start_site	-	154044	154044	-3237	5773 AN10831
520	CONTIG110	77046	77320	2.42	2.22E-03	transcription_start_site	-	74033	74033	-3150	5781 AN10834
520	CONTIG110	77046	77320	2.42	2.22E-03	transcription_start_site	-	73641	73641	-3542	5782 AN10834
520	CONTIG110	77046	77320	2.42	2.22E-03	transcription_start_site	-	72663	72663	-4520	5783 AN10834
2120	CONTIG110	197492	197842	1.33	1.25E-01	transcription_start_site	-	194092	194092	-3575	5792 AN10837
2120	CONTIG110	197492	197842	1.33	1.25E-01	transcription_start_site	-	193934	193934	-3733	5793 AN10837
2120	CONTIG110	197492	197842	1.33	1.25E-01	transcription_start_site	-	193431	193431	-4236	5794 AN10837
2120	CONTIG110	197492	197842	1.33	1.25E-01	transcription_start_site	-	192688	192688	-4979	5795 AN10837
491	CONTIG110	268596	269090	2.46	1.80E-03	transcription_start_site	-	268034	268034	-809	5797 AN10838
491	CONTIG110	268596	269090	2.46	1.80E-03	transcription_start_site	-	267645	267645	-1198	5798 AN10838
491	CONTIG110	268596	269090	2.46	1.80E-03	transcription_start_site	-	270043	270043	1200	5796 AN10838

491	CONTIG110	268596	269090	2.46	1.80E-03	transcription_start_site	-	267468	267468	-1375	5799	AN10838
491	CONTIG110	268596	269090	2.46	1.80E-03	transcription_start_site	-	267080	267080	-1763	5800	AN10838
491	CONTIG110	268596	269090	2.46	1.80E-03	transcription_start_site	-	266201	266201	-2642	5801	AN10838
1094	CONTIG16	122268	122614	1.84	3.08E-02	transcription_start_site	+	123764	123764	-1323	5804	AN1084
1094	CONTIG16	122268	122614	1.84	3.08E-02	transcription_start_site	+	123985	123985	-1544	5805	AN1084
1094	CONTIG16	122268	122614	1.84	3.08E-02	transcription_start_site	+	124070	124070	-1629	5806	AN1084
1094	CONTIG16	122268	122614	1.84	3.08E-02	transcription_start_site	+	124398	124398	-1957	5807	AN1084
1094	CONTIG16	122268	122614	1.84	3.08E-02	transcription_start_site	+	125141	125141	-2700	5808	AN1084
491	CONTIG110	268596	269090	2.46	1.80E-03	transcription_start_site	-	265399	265399	-3444	5817	AN10843
491	CONTIG110	268596	269090	2.46	1.80E-03	transcription_start_site	-	265151	265151	-3692	5818	AN10843
2853	CONTIG112	114396	114875	1.04	1.79E-01	transcription_start_site	-	114256	114256	-379	5819	AN10844
2853	CONTIG112	114396	114875	1.04	1.79E-01	transcription_start_site	-	114057	114057	-578	5820	AN10844
2853	CONTIG112	114396	114875	1.04	1.79E-01	transcription_start_site	-	113936	113936	-699	5821	AN10844
2853	CONTIG112	114396	114875	1.04	1.79E-01	transcription_start_site	-	113300	113300	-1335	5822	AN10844
385	CONTIG112	351246	351580	2.62	0.00E+00	transcription_start_site	-	350869	350869	-544	5829	AN10846
385	CONTIG112	351246	351580	2.62	0.00E+00	transcription_start_site	-	349478	349478	-1935	5830	AN10846
385	CONTIG112	351246	351580	2.62	0.00E+00	transcription_start_site	-	348602	348602	-2811	5831	AN10846
385	CONTIG112	351246	351580	2.62	0.00E+00	transcription_start_site	-	347498	347498	-3915	5832	AN10846
385	CONTIG112	351246	351580	2.62	0.00E+00	transcription_start_site	-	346660	346660	-4753	5833	AN10846
1592	CONTIG112	93239	93510	1.54	3.08E-02	transcription_start_site	+	93493	93493	-118	5834	AN10847
1592	CONTIG112	93239	93510	1.54	3.08E-02	transcription_start_site	+	93676	93676	-301	5835	AN10847
1592	CONTIG112	93239	93510	1.54	3.08E-02	transcription_start_site	+	93785	93785	-410	5836	AN10847
1592	CONTIG112	93239	93510	1.54	3.08E-02	transcription_start_site	+	94145	94145	-770	5837	AN10847
1350	CONTIG112	107191	107755	1.66	1.84E-02	transcription_start_site	-	108507	108507	1034	5847	AN10849
2853	CONTIG112	114396	114875	1.04	1.79E-01	transcription_start_site	-	111957	111957	-2678	5844	AN10849
1094	CONTIG16	122268	122614	1.84	3.08E-02	transcription_start_site	+	126155	126155	-3714	5848	AN1085
1094	CONTIG16	122268	122614	1.84	3.08E-02	transcription_start_site	+	126368	126368	-3927	5849	AN1085
1094	CONTIG16	122268	122614	1.84	3.08E-02	transcription_start_site	+	126862	126862	-4421	5850	AN1085
499	CONTIG112	140265	140555	2.45	6.10E-04	transcription_start_site	+	143928	143928	-3518	5853	AN10851
1999	CONTIG112	140937	141366	1.37	5.88E-02	transcription_start_site	+	143928	143928	-2776	5853	AN10851
2351	CONTIG112	144237	144582	1.25	7.51E-02	transcription_start_site	+	143928	143928	481	5853	AN10851
2711	CONTIG112	141612	142111	1.12	1.44E-01	transcription_start_site	+	143928	143928	-2066	5853	AN10851
1350	CONTIG112	107191	107755	1.66	1.84E-02	transcription_start_site	-	108000	108000	527	5855	AN10853
1350	CONTIG112	107191	107755	1.66	1.84E-02	transcription_start_site	-	105943	105943	-1530	5856	AN10853
1433	CONTIG113	161038	161389	1.62	5.88E-02	transcription_start_site	-	158541	158541	-2672	5857	AN10854
1433	CONTIG113	161038	161389	1.62	5.88E-02	transcription_start_site	-	157935	157935	-3278	5858	AN10854
1433	CONTIG113	161038	161389	1.62	5.88E-02	transcription_start_site	-	157740	157740	-3473	5859	AN10854
1433	CONTIG113	161038	161389	1.62	5.88E-02	transcription_start_site	-	157534	157534	-3679	5860	AN10854
1433	CONTIG113	161038	161389	1.62	5.88E-02	transcription_start_site	-	157126	157126	-4087	5861	AN10854
1433	CONTIG113	161038	161389	1.62	5.88E-02	transcription_start_site	-	156965	156965	-4248	5862	AN10854
1868	CONTIG113	158253	159052	1.43	1.08E-01	transcription_start_site	-	158541	158541	-111	5857	AN10854
1868	CONTIG113	158253	159052	1.43	1.08E-01	transcription_start_site	-	157935	157935	-717	5858	AN10854
1868	CONTIG113	158253	159052	1.43	1.08E-01	transcription_start_site	-	157740	157740	-912	5859	AN10854
1868	CONTIG113	158253	159052	1.43	1.08E-01	transcription_start_site	-	157534	157534	-1118	5860	AN10854
1868	CONTIG113	158253	159052	1.43	1.08E-01	transcription_start_site	-	157126	157126	-1526	5861	AN10854
1868	CONTIG113	158253	159052	1.43	1.08E-01	transcription_start_site	-	156965	156965	-1687	5862	AN10854
732	CONTIG113	174385	174804	2.17	8.81E-03	transcription_start_site	-	169414	169414	-5180	5863	AN10855
732	CONTIG113	174385	174804	2.17	8.81E-03	transcription_start_site	-	170593	170593	-4001	5871	AN10857
77	CONTIG113	73427	73851	3.3	0.00E+00	transcription_start_site	-	69089	69089	-4550	5874	AN10859
336	CONTIG113	73892	74466	2.71	1.01E-03	transcription_start_site	-	69089	69089	-5090	5874	AN10859
1335	CONTIG113	70206	70475	1.67	5.22E-02	transcription_start_site	-	69089	69089	-1251	5874	AN10859
1433	CONTIG113	161038	161389	1.62	5.88E-02	transcription_start_site	-	160180	160180	-1033	5875	AN10860
1433	CONTIG113	161038	161389	1.62	5.88E-02	transcription_start_site	-	159835	159835	-1378	5876	AN10860
1868	CONTIG113	158253	159052	1.43	1.08E-01	transcription_start_site	-	159835	159835	1182	5876	AN10860
2985	CONTIG115	26196	26845	0.93	1.50E-01	transcription_start_site	-	25029	25029	-1491	5877	AN10861
2985	CONTIG115	26196	26845	0.93	1.50E-01	transcription_start_site	-	24578	24578	-1942	5878	AN10861
2985	CONTIG115	26196	26845	0.93	1.50E-01	transcription_start_site	-	24306	24306	-2214	5879	AN10861

2985	CONTIG115	26196	26845	0.93	1.50E-01	transcription_start_site	-	24073	24073	-2447	5880	AN10861
2555	CONTIG115	69164	69593	1.18	5.88E-02	transcription_start_site	+	69306	69306	72	5881	AN10862
2555	CONTIG115	69164	69593	1.18	5.88E-02	transcription_start_site	+	70118	70118	-739	5882	AN10862
2555	CONTIG115	69164	69593	1.18	5.88E-02	transcription_start_site	+	70400	70400	-1021	5883	AN10862
2555	CONTIG115	69164	69593	1.18	5.88E-02	transcription_start_site	+	70703	70703	-1324	5884	AN10862
2555	CONTIG115	69164	69593	1.18	5.88E-02	transcription_start_site	+	74115	74115	-4736	5885	AN10863
2555	CONTIG115	69164	69593	1.18	5.88E-02	transcription_start_site	+	74196	74196	-4817	5886	AN10863
2555	CONTIG115	69164	69593	1.18	5.88E-02	transcription_start_site	+	71548	71548	-2169	5888	AN10864
2555	CONTIG115	69164	69593	1.18	5.88E-02	transcription_start_site	+	72148	72148	-2769	5889	AN10864
2555	CONTIG115	69164	69593	1.18	5.88E-02	transcription_start_site	+	72798	72798	-3419	5890	AN10865
2555	CONTIG115	69164	69593	1.18	5.88E-02	transcription_start_site	+	72889	72889	-3510	5891	AN10865
2555	CONTIG115	69164	69593	1.18	5.88E-02	transcription_start_site	+	73164	73164	-3785	5892	AN10865
2555	CONTIG115	69164	69593	1.18	5.88E-02	transcription_start_site	+	73385	73385	-4006	5893	AN10865
2985	CONTIG115	26196	26845	0.93	1.50E-01	transcription_start_site	-	26875	26875	354	5894	AN10866
2985	CONTIG115	26196	26845	0.93	1.50E-01	transcription_start_site	-	25851	25851	-669	5895	AN10866
380	CONTIG16	139521	139942	2.63	0.00E+00	transcription_start_site	-	134762	134762	-4969	5906	AN1087
1954	CONTIG16	134716	135051	1.39	1.07E-01	transcription_start_site	-	134762	134762	-121	5906	AN1087
1954	CONTIG16	134716	135051	1.39	1.07E-01	transcription_start_site	-	133325	133325	-1558	5907	AN1087
577	CONTIG117	39976	40401	2.34	2.22E-03	transcription_start_site	+	39151	39151	1037	5918	AN10871
1463	CONTIG117	59269	59625	1.61	4.36E-02	transcription_start_site	-	58697	58697	-750	5919	AN10872
1463	CONTIG117	59269	59625	1.61	4.36E-02	transcription_start_site	-	57053	57053	-2394	5920	AN10872
1463	CONTIG117	59269	59625	1.61	4.36E-02	transcription_start_site	-	56908	56908	-2539	5921	AN10872
1463	CONTIG117	59269	59625	1.61	4.36E-02	transcription_start_site	-	56415	56415	-3032	5943	AN10877
1463	CONTIG117	59269	59625	1.61	4.36E-02	transcription_start_site	-	56250	56250	-3197	5944	AN10877
1954	CONTIG16	134716	135051	1.39	1.07E-01	transcription_start_site	+	135191	135191	-307	5950	AN1088
1954	CONTIG16	134716	135051	1.39	1.07E-01	transcription_start_site	+	135403	135403	-519	5951	AN1088
1954	CONTIG16	134716	135051	1.39	1.07E-01	transcription_start_site	+	135655	135655	-771	5952	AN1088
577	CONTIG117	39976	40401	2.34	2.22E-03	transcription_start_site	+	39648	39648	540	5956	AN10881
577	CONTIG117	39976	40401	2.34	2.22E-03	transcription_start_site	+	39514	39514	674	5955	AN10881
577	CONTIG117	39976	40401	2.34	2.22E-03	transcription_start_site	+	39235	39235	953	5954	AN10881
2027	CONTIG118	61371	62169	1.36	1.23E-02	transcription_start_site	+	63563	63563	-1793	5970	AN10885
2027	CONTIG118	61371	62169	1.36	1.23E-02	transcription_start_site	+	65157	65157	-3387	5971	AN10885
1761	CONTIG118	66829	67343	1.47	9.21E-03	transcription_start_site	+	71056	71056	-3970	5972	AN10886
1761	CONTIG118	66829	67343	1.47	9.21E-03	transcription_start_site	+	71286	71286	-4200	5973	AN10886
1761	CONTIG118	66829	67343	1.47	9.21E-03	transcription_start_site	+	71378	71378	-4292	5974	AN10886
1761	CONTIG118	66829	67343	1.47	9.21E-03	transcription_start_site	+	71521	71521	-4435	5975	AN10886
1761	CONTIG118	66829	67343	1.47	9.21E-03	transcription_start_site	+	71991	71991	-4905	5976	AN10886
2863	CONTIG118	71778	72127	1.03	1.25E-01	transcription_start_site	+	71991	71991	-38	5976	AN10886
2863	CONTIG118	71778	72127	1.03	1.25E-01	transcription_start_site	+	71521	71521	431	5975	AN10886
2863	CONTIG118	71778	72127	1.03	1.25E-01	transcription_start_site	+	71378	71378	574	5974	AN10886
2863	CONTIG118	71778	72127	1.03	1.25E-01	transcription_start_site	+	72582	72582	-629	5977	AN10886
2863	CONTIG118	71778	72127	1.03	1.25E-01	transcription_start_site	+	71286	71286	666	5973	AN10886
2863	CONTIG118	71778	72127	1.03	1.25E-01	transcription_start_site	+	71056	71056	896	5972	AN10886
2963	CONTIG118	69153	69967	0.95	1.50E-01	transcription_start_site	+	71056	71056	-1496	5972	AN10886
2963	CONTIG118	69153	69967	0.95	1.50E-01	transcription_start_site	+	71286	71286	-1726	5973	AN10886
2963	CONTIG118	69153	69967	0.95	1.50E-01	transcription_start_site	+	71378	71378	-1818	5974	AN10886
2963	CONTIG118	69153	69967	0.95	1.50E-01	transcription_start_site	+	71521	71521	-1961	5975	AN10886
2963	CONTIG118	69153	69967	0.95	1.50E-01	transcription_start_site	+	71991	71991	-2431	5976	AN10886
2963	CONTIG118	69153	69967	0.95	1.50E-01	transcription_start_site	+	72582	72582	-3022	5977	AN10886
875	CONTIG118	74403	74775	2.02	1.01E-03	transcription_start_site	+	73939	73939	650	5980	AN10887
2863	CONTIG118	71778	72127	1.03	1.25E-01	transcription_start_site	+	73040	73040	-1087	5978	AN10887
2863	CONTIG118	71778	72127	1.03	1.25E-01	transcription_start_site	+	73328	73328	-1375	5979	AN10887
2863	CONTIG118	71778	72127	1.03	1.25E-01	transcription_start_site	+	73939	73939	-1986	5980	AN10887
2963	CONTIG118	69153	69967	0.95	1.50E-01	transcription_start_site	+	73040	73040	-3480	5978	AN10887
2963	CONTIG118	69153	69967	0.95	1.50E-01	transcription_start_site	+	73328	73328	-3768	5979	AN10887
2963	CONTIG118	69153	69967	0.95	1.50E-01	transcription_start_site	+	73939	73939	-4379	5980	AN10887
2965	CONTIG118	19136	19402	0.95	1.66E-01	transcription_start_site	+	18859	18859	410	5984	AN10888

2965	CONTIG118	19136	19402	0.95	1.66E-01	transcription_start_site	+	18555	18555	714	5983	AN10888
380	CONTIG16	139521	139942	2.63	0.00E+00	transcription_start_site	-	138614	138614	-1117	5987	AN1089
380	CONTIG16	139521	139942	2.63	0.00E+00	transcription_start_site	-	137831	137831	-1900	5988	AN1089
380	CONTIG16	139521	139942	2.63	0.00E+00	transcription_start_site	-	136718	136718	-3013	5989	AN1089
2027	CONTIG118	61371	62169	1.36	1.23E-02	transcription_start_site	+	63021	63021	-1251	5990	AN10890
2027	CONTIG118	61371	62169	1.36	1.23E-02	transcription_start_site	+	63201	63201	-1431	5991	AN10890
620	CONTIG119	20027	20316	2.28	9.51E-04	transcription_start_site	+	25077	25077	-4905	5992	AN10891
620	CONTIG119	20027	20316	2.28	9.51E-04	transcription_start_site	+	25257	25257	-5085	5993	AN10891
1670	CONTIG119	34893	35915	1.51	3.08E-02	transcription_start_site	+	38173	38173	-2769	5996	AN10892
1670	CONTIG119	34893	35915	1.51	3.08E-02	transcription_start_site	+	38466	38466	-3062	5997	AN10892
1670	CONTIG119	34893	35915	1.51	3.08E-02	transcription_start_site	+	39167	39167	-3763	5998	AN10892
1764	CONTIG119	36228	37402	1.47	2.18E-02	transcription_start_site	+	38173	38173	-1358	5996	AN10892
1764	CONTIG119	36228	37402	1.47	2.18E-02	transcription_start_site	+	38466	38466	-1651	5997	AN10892
1764	CONTIG119	36228	37402	1.47	2.18E-02	transcription_start_site	+	39167	39167	-2352	5998	AN10892
1857	CONTIG119	33828	34257	1.43	4.36E-02	transcription_start_site	+	38173	38173	-4130	5996	AN10892
1857	CONTIG119	33828	34257	1.43	4.36E-02	transcription_start_site	+	38466	38466	-4423	5997	AN10892
1857	CONTIG119	33828	34257	1.43	4.36E-02	transcription_start_site	+	39167	39167	-5124	5998	AN10892
2319	CONTIG119	33018	33732	1.26	8.01E-02	transcription_start_site	+	38173	38173	-4798	5996	AN10892
2319	CONTIG119	33018	33732	1.26	8.01E-02	transcription_start_site	+	38466	38466	-5091	5997	AN10892
2659	CONTIG119	37578	38302	1.14	1.07E-01	transcription_start_site	+	38173	38173	-233	5996	AN10892
2659	CONTIG119	37578	38302	1.14	1.07E-01	transcription_start_site	+	38466	38466	-526	5997	AN10892
2659	CONTIG119	37578	38302	1.14	1.07E-01	transcription_start_site	+	39167	39167	-1227	5998	AN10892
2815	CONTIG119	132903	133262	1.06	1.66E-01	transcription_start_site	-	133991	133991	908	6008	AN10894
2815	CONTIG119	132903	133262	1.06	1.66E-01	transcription_start_site	-	134236	134236	1153	6007	AN10894
1670	CONTIG119	34893	35915	1.51	3.08E-02	transcription_start_site	+	37034	37034	-1630	6013	AN10896
1670	CONTIG119	34893	35915	1.51	3.08E-02	transcription_start_site	+	37239	37239	-1835	6014	AN10896
1764	CONTIG119	36228	37402	1.47	2.18E-02	transcription_start_site	+	37034	37034	-219	6013	AN10896
1764	CONTIG119	36228	37402	1.47	2.18E-02	transcription_start_site	+	37239	37239	-424	6014	AN10896
1857	CONTIG119	33828	34257	1.43	4.36E-02	transcription_start_site	+	37034	37034	-2991	6013	AN10896
1857	CONTIG119	33828	34257	1.43	4.36E-02	transcription_start_site	+	37239	37239	-3196	6014	AN10896
2319	CONTIG119	33018	33732	1.26	8.01E-02	transcription_start_site	+	37034	37034	-3659	6013	AN10896
2319	CONTIG119	33018	33732	1.26	8.01E-02	transcription_start_site	+	37239	37239	-3864	6014	AN10896
2659	CONTIG119	37578	38302	1.14	1.07E-01	transcription_start_site	+	37239	37239	701	6014	AN10896
2659	CONTIG119	37578	38302	1.14	1.07E-01	transcription_start_site	+	37034	37034	906	6013	AN10896
620	CONTIG119	20027	20316	2.28	9.51E-04	transcription_start_site	+	22427	22427	-2255	6015	AN10897
620	CONTIG119	20027	20316	2.28	9.51E-04	transcription_start_site	+	22827	22827	-2655	6016	AN10897
620	CONTIG119	20027	20316	2.28	9.51E-04	transcription_start_site	+	23210	23210	-3038	6017	AN10897
620	CONTIG119	20027	20316	2.28	9.51E-04	transcription_start_site	+	24452	24452	-4280	6018	AN10897
620	CONTIG119	20027	20316	2.28	9.51E-04	transcription_start_site	+	24641	24641	-4469	6019	AN10897
620	CONTIG119	20027	20316	2.28	9.51E-04	transcription_start_site	+	19901	19901	270	6020	AN10898
620	CONTIG119	20027	20316	2.28	9.51E-04	transcription_start_site	+	20672	20672	-500	6021	AN10898
620	CONTIG119	20027	20316	2.28	9.51E-04	transcription_start_site	+	20982	20982	-810	6022	AN10898
620	CONTIG119	20027	20316	2.28	9.51E-04	transcription_start_site	+	21324	21324	-1152	6023	AN10898
620	CONTIG119	20027	20316	2.28	9.51E-04	transcription_start_site	+	21661	21661	-1489	6024	AN10898
620	CONTIG119	20027	20316	2.28	9.51E-04	transcription_start_site	+	21773	21773	-1601	6025	AN10898
2815	CONTIG119	132903	133262	1.06	1.66E-01	transcription_start_site	-	133313	133313	230	6026	AN10899
2815	CONTIG119	132903	133262	1.06	1.66E-01	transcription_start_site	-	132473	132473	-609	6027	AN10899
1494	CONTIG16	144616	144885	1.59	6.84E-02	transcription_start_site	-	142019	142019	-2731	6028	AN1090
1494	CONTIG16	144616	144885	1.59	6.84E-02	transcription_start_site	-	141873	141873	-2877	6029	AN1090
1494	CONTIG16	144616	144885	1.59	6.84E-02	transcription_start_site	-	141692	141692	-3058	6030	AN1090
436	CONTIG122	6155	6446	2.54	5.20E-03	transcription_start_site	-	6468	6468	167	6032	AN10900
6	CONTIG122	101251	101600	3.84	0.00E+00	transcription_start_site	-	101980	101980	554	6037	AN10902
6	CONTIG122	101251	101600	3.84	0.00E+00	transcription_start_site	-	102336	102336	910	6036	AN10902
34	CONTIG122	102696	103185	3.52	0.00E+00	transcription_start_site	-	102735	102735	-205	6035	AN10902
34	CONTIG122	102696	103185	3.52	0.00E+00	transcription_start_site	-	102336	102336	-604	6036	AN10902
34	CONTIG122	102696	103185	3.52	0.00E+00	transcription_start_site	-	101980	101980	-960	6037	AN10902
121	CONTIG122	101866	102360	3.14	5.64E-04	transcription_start_site	-	101980	101980	-133	6037	AN10902

121	CONTIG122	101866	102360	3.14	5.64E-04	transcription_start_site	-	102336	102336	223	6036	AN10902
121	CONTIG122	101866	102360	3.14	5.64E-04	transcription_start_site	-	102735	102735	622	6035	AN10902
184	CONTIG122	106971	107315	2.98	1.01E-03	transcription_start_site	-	102735	102735	-4408	6035	AN10902
184	CONTIG122	106971	107315	2.98	1.01E-03	transcription_start_site	-	102336	102336	-4807	6036	AN10902
184	CONTIG122	106971	107315	2.98	1.01E-03	transcription_start_site	-	101980	101980	-5163	6037	AN10902
503	CONTIG122	104271	106175	2.44	0.00E+00	transcription_start_site	-	102735	102735	-2488	6035	AN10902
503	CONTIG122	104271	106175	2.44	0.00E+00	transcription_start_site	-	102336	102336	-2887	6036	AN10902
503	CONTIG122	104271	106175	2.44	0.00E+00	transcription_start_site	-	101980	101980	-3243	6037	AN10902
436	CONTIG122	6155	6446	2.54	5.20E-03	transcription_start_site	-	5899	5899	-401	6040	AN10904
436	CONTIG122	6155	6446	2.54	5.20E-03	transcription_start_site	-	5697	5697	-603	6041	AN10904
436	CONTIG122	6155	6446	2.54	5.20E-03	transcription_start_site	-	5574	5574	-726	6042	AN10904
436	CONTIG122	6155	6446	2.54	5.20E-03	transcription_start_site	-	5279	5279	-1021	6043	AN10904
436	CONTIG122	6155	6446	2.54	5.20E-03	transcription_start_site	-	4481	4481	-1819	6044	AN10904
436	CONTIG122	6155	6446	2.54	5.20E-03	transcription_start_site	-	4322	4322	-1978	6045	AN10904
436	CONTIG122	6155	6446	2.54	5.20E-03	transcription_start_site	-	4081	4081	-2219	6046	AN10904
436	CONTIG122	6155	6446	2.54	5.20E-03	transcription_start_site	-	3714	3714	-2586	6047	AN10904
436	CONTIG122	6155	6446	2.54	5.20E-03	transcription_start_site	-	3588	3588	-2712	6048	AN10904
436	CONTIG122	6155	6446	2.54	5.20E-03	transcription_start_site	-	3489	3489	-2811	6049	AN10904
436	CONTIG122	6155	6446	2.54	5.20E-03	transcription_start_site	-	3331	3331	-2969	6050	AN10904
436	CONTIG122	6155	6446	2.54	5.20E-03	transcription_start_site	-	3161	3161	-3139	6051	AN10904
436	CONTIG122	6155	6446	2.54	5.20E-03	transcription_start_site	-	2931	2931	-3369	6052	AN10904
436	CONTIG122	6155	6446	2.54	5.20E-03	transcription_start_site	-	2433	2433	-3867	6053	AN10904
6	CONTIG122	101251	101600	3.84	0.00E+00	transcription_start_site	-	101075	101075	-350	6054	AN10905
6	CONTIG122	101251	101600	3.84	0.00E+00	transcription_start_site	-	100775	100775	-650	6055	AN10905
6	CONTIG122	101251	101600	3.84	0.00E+00	transcription_start_site	-	100491	100491	-934	6056	AN10905
6	CONTIG122	101251	101600	3.84	0.00E+00	transcription_start_site	-	99902	99902	-1523	6057	AN10905
6	CONTIG122	101251	101600	3.84	0.00E+00	transcription_start_site	-	99746	99746	-1679	6058	AN10905
6	CONTIG122	101251	101600	3.84	0.00E+00	transcription_start_site	-	99279	99279	-2146	6059	AN10905
34	CONTIG122	102696	103185	3.52	0.00E+00	transcription_start_site	-	101075	101075	-1865	6054	AN10905
34	CONTIG122	102696	103185	3.52	0.00E+00	transcription_start_site	-	100775	100775	-2165	6055	AN10905
34	CONTIG122	102696	103185	3.52	0.00E+00	transcription_start_site	-	100491	100491	-2449	6056	AN10905
34	CONTIG122	102696	103185	3.52	0.00E+00	transcription_start_site	-	99902	99902	-3038	6057	AN10905
34	CONTIG122	102696	103185	3.52	0.00E+00	transcription_start_site	-	99746	99746	-3194	6058	AN10905
34	CONTIG122	102696	103185	3.52	0.00E+00	transcription_start_site	-	99279	99279	-3661	6059	AN10905
121	CONTIG122	101866	102360	3.14	5.64E-04	transcription_start_site	-	101075	101075	-1038	6054	AN10905
121	CONTIG122	101866	102360	3.14	5.64E-04	transcription_start_site	-	100775	100775	-1338	6055	AN10905
121	CONTIG122	101866	102360	3.14	5.64E-04	transcription_start_site	-	100491	100491	-1622	6056	AN10905
121	CONTIG122	101866	102360	3.14	5.64E-04	transcription_start_site	-	99902	99902	-2211	6057	AN10905
121	CONTIG122	101866	102360	3.14	5.64E-04	transcription_start_site	-	99746	99746	-2367	6058	AN10905
121	CONTIG122	101866	102360	3.14	5.64E-04	transcription_start_site	-	99279	99279	-2834	6059	AN10905
503	CONTIG122	104271	106175	2.44	0.00E+00	transcription_start_site	-	101075	101075	-4148	6054	AN10905
503	CONTIG122	104271	106175	2.44	0.00E+00	transcription_start_site	-	100775	100775	-4448	6055	AN10905
503	CONTIG122	104271	106175	2.44	0.00E+00	transcription_start_site	-	100491	100491	-4732	6056	AN10905
503	CONTIG122	104271	106175	2.44	0.00E+00	transcription_start_site	-	99902	99902	-5321	6057	AN10905
503	CONTIG122	104271	106175	2.44	0.00E+00	transcription_start_site	-	99746	99746	-5477	6058	AN10905
503	CONTIG122	104271	106175	2.44	0.00E+00	transcription_start_site	-	99279	99279	-5944	6059	AN10905
848	CONTIG122	100051	100625	2.06	2.62E-02	transcription_start_site	-	100491	100491	153	6056	AN10905
848	CONTIG122	100051	100625	2.06	2.62E-02	transcription_start_site	-	99902	99902	-436	6057	AN10905
848	CONTIG122	100051	100625	2.06	2.62E-02	transcription_start_site	-	100775	100775	437	6055	AN10905
848	CONTIG122	100051	100625	2.06	2.62E-02	transcription_start_site	-	99746	99746	-592	6058	AN10905
848	CONTIG122	100051	100625	2.06	2.62E-02	transcription_start_site	-	101075	101075	737	6054	AN10905
848	CONTIG122	100051	100625	2.06	2.62E-02	transcription_start_site	-	99279	99279	-1059	6059	AN10905
1357	CONTIG123	7888	8242	1.66	4.36E-02	transcription_start_site	-	7646	7646	-419	6060	AN10906
1357	CONTIG123	7888	8242	1.66	4.36E-02	transcription_start_site	-	6985	6985	-1080	6061	AN10906
1357	CONTIG123	7888	8242	1.66	4.36E-02	transcription_start_site	-	6748	6748	-1317	6062	AN10906
1357	CONTIG123	7888	8242	1.66	4.36E-02	transcription_start_site	-	6181	6181	-1884	6063	AN10906
1127	CONTIG123	22129	22493	1.81	2.62E-02	transcription_start_site	-	23270	23270	959	6074	AN10908

1127	CONTIG123	22129	22493	1.81	2.62E-02	transcription_start_site	-	23426	23426	1115	6073 AN10908
709	CONTIG123	58357	58776	2.19	6.61E-03	transcription_start_site	+	58353	58353	213	6076 AN10909
709	CONTIG123	58357	58776	2.19	6.61E-03	transcription_start_site	+	57501	57501	1065	6075 AN10909
1203	CONTIG123	55142	55476	1.76	3.08E-02	transcription_start_site	+	57501	57501	-2192	6075 AN10909
1203	CONTIG123	55142	55476	1.76	3.08E-02	transcription_start_site	+	58353	58353	-3044	6076 AN10909
1204	CONTIG123	68402	68686	1.76	3.08E-02	transcription_start_site	-	65591	65591	-2953	6077 AN10910
1204	CONTIG123	68402	68686	1.76	3.08E-02	transcription_start_site	-	65336	65336	-3208	6078 AN10910
1204	CONTIG123	68402	68686	1.76	3.08E-02	transcription_start_site	-	65233	65233	-3311	6079 AN10910
1204	CONTIG123	68402	68686	1.76	3.08E-02	transcription_start_site	-	65049	65049	-3495	6080 AN10910
1204	CONTIG123	68402	68686	1.76	3.08E-02	transcription_start_site	-	64422	64422	-4122	6081 AN10910
1204	CONTIG123	68402	68686	1.76	3.08E-02	transcription_start_site	-	63944	63944	-4600	6082 AN10910
1204	CONTIG123	68402	68686	1.76	3.08E-02	transcription_start_site	-	63772	63772	-4772	6083 AN10910
1204	CONTIG123	68402	68686	1.76	3.08E-02	transcription_start_site	-	63593	63593	-4951	6084 AN10910
1357	CONTIG123	7888	8242	1.66	4.36E-02	transcription_start_site	-	5149	5149	-2916	6085 AN10911
1357	CONTIG123	7888	8242	1.66	4.36E-02	transcription_start_site	-	5024	5024	-3041	6086 AN10911
709	CONTIG123	58357	58776	2.19	6.61E-03	transcription_start_site	+	58910	58910	-343	6096 AN10915
709	CONTIG123	58357	58776	2.19	6.61E-03	transcription_start_site	+	59774	59774	-1207	6097 AN10915
1203	CONTIG123	55142	55476	1.76	3.08E-02	transcription_start_site	+	58910	58910	-3601	6096 AN10915
1203	CONTIG123	55142	55476	1.76	3.08E-02	transcription_start_site	+	59774	59774	-4465	6097 AN10915
50	CONTIG123	72012	72731	3.42	0.00E+00	transcription_start_site	-	67061	67061	-5310	6098 AN10916
1204	CONTIG123	68402	68686	1.76	3.08E-02	transcription_start_site	-	67061	67061	-1483	6098 AN10916
1204	CONTIG123	68402	68686	1.76	3.08E-02	transcription_start_site	-	66832	66832	-1712	6099 AN10916
1204	CONTIG123	68402	68686	1.76	3.08E-02	transcription_start_site	-	66632	66632	-1912	6100 AN10916
1204	CONTIG123	68402	68686	1.76	3.08E-02	transcription_start_site	-	66476	66476	-2068	6101 AN10916
1204	CONTIG123	68402	68686	1.76	3.08E-02	transcription_start_site	-	66384	66384	-2160	6102 AN10916
1204	CONTIG123	68402	68686	1.76	3.08E-02	transcription_start_site	-	66158	66158	-2386	6103 AN10916
2386	CONTIG123	71037	71841	1.24	1.59E-01	transcription_start_site	-	67061	67061	-4378	6098 AN10916
2386	CONTIG123	71037	71841	1.24	1.59E-01	transcription_start_site	-	66832	66832	-4607	6099 AN10916
2386	CONTIG123	71037	71841	1.24	1.59E-01	transcription_start_site	-	66632	66632	-4807	6100 AN10916
2386	CONTIG123	71037	71841	1.24	1.59E-01	transcription_start_site	-	66476	66476	-4963	6101 AN10916
2386	CONTIG123	71037	71841	1.24	1.59E-01	transcription_start_site	-	66384	66384	-5055	6102 AN10916
2386	CONTIG123	71037	71841	1.24	1.59E-01	transcription_start_site	-	66158	66158	-5281	6103 AN10916
552	CONTIG124	1513	1782	2.38	1.22E-03	transcription_start_site	+	4118	4118	-2470	6104 AN10917
552	CONTIG124	1513	1782	2.38	1.22E-03	transcription_start_site	+	4219	4219	-2571	6105 AN10917
1344	CONTIG126	19146	19400	1.66	2.67E-04	transcription_start_site	-	18249	18249	-1024	6117 AN10921
1344	CONTIG126	19146	19400	1.66	2.67E-04	transcription_start_site	-	18191	18191	-1082	6118 AN10921
1251	CONTIG127	62557	62838	1.72	1.80E-03	transcription_start_site	+	66374	66374	-3676	6122 AN10923
1714	CONTIG127	69527	70031	1.49	7.85E-03	transcription_start_site	+	68955	68955	824	6126 AN10925
1714	CONTIG127	69527	70031	1.49	7.85E-03	transcription_start_site	+	68839	68839	940	6125 AN10925
3028	CONTIG127	18619	18958	0.86	1.66E-01	transcription_start_site	+	22761	22761	-3972	6127 AN10926
589	CONTIG127	9092	9524	2.32	0.00E+00	transcription_start_site	-	4298	4298	-5010	6132 AN10928
589	CONTIG127	9092	9524	2.32	0.00E+00	transcription_start_site	-	4167	4167	-5141	6133 AN10928
2402	CONTIG128	10894	11458	1.23	5.88E-02	transcription_start_site	+	13760	13760	-2584	6137 AN10929
2402	CONTIG128	10894	11458	1.23	5.88E-02	transcription_start_site	+	14286	14286	-3110	6138 AN10929
2402	CONTIG128	10894	11458	1.23	5.88E-02	transcription_start_site	+	15329	15329	-4153	6139 AN10929
2988	CONTIG128	15921	16645	0.93	1.93E-01	transcription_start_site	+	15329	15329	954	6139 AN10929
1494	CONTIG16	144616	144885	1.59	6.84E-02	transcription_start_site	+	149053	149053	-4302	6140 AN1093
1494	CONTIG16	144616	144885	1.59	6.84E-02	transcription_start_site	+	149342	149342	-4591	6141 AN1093
1494	CONTIG16	144616	144885	1.59	6.84E-02	transcription_start_site	+	149409	149409	-4658	6142 AN1093
1102	CONTIG128	82745	83234	1.83	3.61E-03	transcription_start_site	-	79745	79745	-3244	6144 AN10930
1102	CONTIG128	82745	83234	1.83	3.61E-03	transcription_start_site	-	79441	79441	-3548	6145 AN10930
1102	CONTIG128	82745	83234	1.83	3.61E-03	transcription_start_site	-	79178	79178	-3811	6146 AN10930
1102	CONTIG128	82745	83234	1.83	3.61E-03	transcription_start_site	-	78736	78736	-4253	6147 AN10930
1614	CONTIG128	135232	135506	1.53	1.57E-02	transcription_start_site	-	131477	131477	-3892	6152 AN10932
1614	CONTIG128	135232	135506	1.53	1.57E-02	transcription_start_site	-	131301	131301	-4068	6153 AN10932
1614	CONTIG128	135232	135506	1.53	1.57E-02	transcription_start_site	-	130989	130989	-4380	6154 AN10932
2402	CONTIG128	10894	11458	1.23	5.88E-02	transcription_start_site	+	16043	16043	-4867	6162 AN10934

2402	CONTIG128	10894	11458	1.23	5.88E-02	transcription_start_site	+	16400	16400	-5224	6163	AN10934
2988	CONTIG128	15921	16645	0.93	1.93E-01	transcription_start_site	+	16400	16400	-117	6163	AN10934
2988	CONTIG128	15921	16645	0.93	1.93E-01	transcription_start_site	+	16043	16043	240	6162	AN10934
1102	CONTIG128	82745	83234	1.83	3.61E-03	transcription_start_site	-	77797	77797	-5192	6165	AN10936
2953	CONTIG128	124366	124625	0.97	1.66E-01	transcription_start_site	-	119514	119514	-4981	6167	AN10937
2953	CONTIG128	124366	124625	0.97	1.66E-01	transcription_start_site	-	120924	120924	-3571	6170	AN10938
2953	CONTIG128	124366	124625	0.97	1.66E-01	transcription_start_site	-	120802	120802	-3693	6171	AN10938
2953	CONTIG128	124366	124625	0.97	1.66E-01	transcription_start_site	-	120596	120596	-3899	6172	AN10938
2953	CONTIG128	124366	124625	0.97	1.66E-01	transcription_start_site	-	119997	119997	-4498	6173	AN10938
762	CONTIG16	155188	155752	2.14	4.81E-03	transcription_start_site	-	154989	154989	-481	6178	AN1094
762	CONTIG16	155188	155752	2.14	4.81E-03	transcription_start_site	-	154577	154577	-893	6179	AN1094
762	CONTIG16	155188	155752	2.14	4.81E-03	transcription_start_site	-	154184	154184	-1286	6180	AN1094
762	CONTIG16	155188	155752	2.14	4.81E-03	transcription_start_site	-	153141	153141	-2329	6181	AN1094
968	CONTIG16	156013	156366	1.94	2.12E-02	transcription_start_site	-	154989	154989	-1200	6178	AN1094
968	CONTIG16	156013	156366	1.94	2.12E-02	transcription_start_site	-	154577	154577	-1612	6179	AN1094
968	CONTIG16	156013	156366	1.94	2.12E-02	transcription_start_site	-	154184	154184	-2005	6180	AN1094
968	CONTIG16	156013	156366	1.94	2.12E-02	transcription_start_site	-	153141	153141	-3048	6181	AN1094
1237	CONTIG16	158268	158752	1.74	4.36E-02	transcription_start_site	-	154989	154989	-3521	6178	AN1094
1237	CONTIG16	158268	158752	1.74	4.36E-02	transcription_start_site	-	154577	154577	-3933	6179	AN1094
1237	CONTIG16	158268	158752	1.74	4.36E-02	transcription_start_site	-	154184	154184	-4326	6180	AN1094
2113	CONTIG129	292730	293089	1.33	1.08E-01	transcription_start_site	-	291869	291869	-1040	6203	AN10945
2113	CONTIG129	292730	293089	1.33	1.08E-01	transcription_start_site	-	291773	291773	-1136	6204	AN10945
2113	CONTIG129	292730	293089	1.33	1.08E-01	transcription_start_site	-	290998	290998	-1911	6205	AN10945
2383	CONTIG129	298810	299089	1.24	1.44E-01	transcription_start_site	-	298955	298955	5	6207	AN10946
2383	CONTIG129	298810	299089	1.24	1.44E-01	transcription_start_site	-	299147	299147	197	6206	AN10946
2383	CONTIG129	298810	299089	1.24	1.44E-01	transcription_start_site	-	298708	298708	-241	6208	AN10946
2383	CONTIG129	298810	299089	1.24	1.44E-01	transcription_start_site	-	298497	298497	-452	6209	AN10946
1297	CONTIG129	346364	346722	1.7	3.08E-02	transcription_start_site	-	344687	344687	-1856	6210	AN10947
2222	CONTIG129	347039	347683	1.29	1.07E-01	transcription_start_site	-	344687	344687	-2674	6210	AN10947
1090	CONTIG129	531526	531800	1.84	1.84E-02	transcription_start_site	+	532341	532341	-678	6217	AN10949
1090	CONTIG129	531526	531800	1.84	1.84E-02	transcription_start_site	+	533021	533021	-1358	6218	AN10949
1090	CONTIG129	531526	531800	1.84	1.84E-02	transcription_start_site	+	533512	533512	-1849	6219	AN10949
1090	CONTIG129	531526	531800	1.84	1.84E-02	transcription_start_site	+	534627	534627	-2964	6220	AN10949
1090	CONTIG129	531526	531800	1.84	1.84E-02	transcription_start_site	+	536166	536166	-4503	6221	AN10949
1090	CONTIG129	531526	531800	1.84	1.84E-02	transcription_start_site	+	536672	536672	-5009	6222	AN10949
23	CONTIG16	163732	164451	3.58	0.00E+00	transcription_start_site	-	158931	158931	-5160	6223	AN1095
85	CONTIG16	162752	163034	3.28	0.00E+00	transcription_start_site	-	158931	158931	-3962	6223	AN1095
85	CONTIG16	162752	163034	3.28	0.00E+00	transcription_start_site	-	158558	158558	-4335	6224	AN1095
1237	CONTIG16	158268	158752	1.74	4.36E-02	transcription_start_site	-	158558	158558	48	6224	AN1095
1237	CONTIG16	158268	158752	1.74	4.36E-02	transcription_start_site	-	158931	158931	421	6223	AN1095
1956	CONTIG16	160128	160477	1.39	1.25E-01	transcription_start_site	-	158931	158931	-1371	6223	AN1095
1956	CONTIG16	160128	160477	1.39	1.25E-01	transcription_start_site	-	158558	158558	-1744	6224	AN1095
1957	CONTIG16	161328	161843	1.39	1.25E-01	transcription_start_site	-	158931	158931	-2654	6223	AN1095
1957	CONTIG16	161328	161843	1.39	1.25E-01	transcription_start_site	-	158558	158558	-3027	6224	AN1095
113	CONTIG129	567609	567968	3.17	0.00E+00	transcription_start_site	+	568233	568233	-444	6225	AN10950
113	CONTIG129	567609	567968	3.17	0.00E+00	transcription_start_site	+	568597	568597	-808	6226	AN10950
1880	CONTIG129	569034	569608	1.42	8.01E-02	transcription_start_site	+	568597	568597	724	6226	AN10950
1880	CONTIG129	569034	569608	1.42	8.01E-02	transcription_start_site	+	568233	568233	1088	6225	AN10950
822	CONTIG129	621677	622041	2.07	3.31E-03	transcription_start_site	+	622319	622319	-460	6230	AN10952
822	CONTIG129	621677	622041	2.07	3.31E-03	transcription_start_site	+	623033	623033	-1174	6231	AN10952
822	CONTIG129	621677	622041	2.07	3.31E-03	transcription_start_site	+	623386	623386	-1527	6232	AN10952
2113	CONTIG129	292730	293089	1.33	1.08E-01	transcription_start_site	-	290249	290249	-2660	6250	AN10957
2113	CONTIG129	292730	293089	1.33	1.08E-01	transcription_start_site	-	290132	290132	-2777	6251	AN10957
2113	CONTIG129	292730	293089	1.33	1.08E-01	transcription_start_site	-	289407	289407	-3502	6252	AN10957
2113	CONTIG129	292730	293089	1.33	1.08E-01	transcription_start_site	-	287808	287808	-5101	6253	AN10958
23	CONTIG16	163732	164451	3.58	0.00E+00	transcription_start_site	-	161667	161667	-2424	6261	AN1096
23	CONTIG16	163732	164451	3.58	0.00E+00	transcription_start_site	-	161141	161141	-2950	6262	AN1096

23	CONTIG16	163732	164451	3.58	0.00E+00	transcription_start_site	-	160812	160812	-3279	6263	AN1096
85	CONTIG16	162752	163034	3.28	0.00E+00	transcription_start_site	-	161667	161667	-1226	6261	AN1096
85	CONTIG16	162752	163034	3.28	0.00E+00	transcription_start_site	-	161141	161141	-1752	6262	AN1096
85	CONTIG16	162752	163034	3.28	0.00E+00	transcription_start_site	-	160812	160812	-2081	6263	AN1096
320	CONTIG16	165752	166334	2.73	0.00E+00	transcription_start_site	-	161667	161667	-4376	6261	AN1096
320	CONTIG16	165752	166334	2.73	0.00E+00	transcription_start_site	-	161141	161141	-4902	6262	AN1096
320	CONTIG16	165752	166334	2.73	0.00E+00	transcription_start_site	-	160812	160812	-5231	6263	AN1096
1956	CONTIG16	160128	160477	1.39	1.25E-01	transcription_start_site	-	160812	160812	509	6263	AN1096
1956	CONTIG16	160128	160477	1.39	1.25E-01	transcription_start_site	-	161141	161141	838	6262	AN1096
1957	CONTIG16	161328	161843	1.39	1.25E-01	transcription_start_site	-	161667	161667	81	6261	AN1096
1957	CONTIG16	161328	161843	1.39	1.25E-01	transcription_start_site	-	161141	161141	-444	6262	AN1096
1957	CONTIG16	161328	161843	1.39	1.25E-01	transcription_start_site	-	160812	160812	-773	6263	AN1096
1297	CONTIG129	346364	346722	1.7	3.08E-02	transcription_start_site	-	346421	346421	-122	6264	AN10960
2222	CONTIG129	347039	347683	1.29	1.07E-01	transcription_start_site	-	346421	346421	-940	6264	AN10960
545	CONTIG129	556053	556402	2.39	1.80E-03	transcription_start_site	+	560242	560242	-4014	6275	AN10964
1300	CONTIG129	556888	557237	1.7	3.08E-02	transcription_start_site	+	560242	560242	-3179	6275	AN10964
822	CONTIG129	621677	622041	2.07	3.31E-03	transcription_start_site	+	624676	624676	-2817	6276	AN10965
822	CONTIG129	621677	622041	2.07	3.31E-03	transcription_start_site	+	624844	624844	-2985	6277	AN10965
822	CONTIG129	621677	622041	2.07	3.31E-03	transcription_start_site	+	625037	625037	-3178	6278	AN10965
822	CONTIG129	621677	622041	2.07	3.31E-03	transcription_start_site	+	625170	625170	-3311	6279	AN10965
822	CONTIG129	621677	622041	2.07	3.31E-03	transcription_start_site	+	625352	625352	-3493	6280	AN10965
822	CONTIG129	621677	622041	2.07	3.31E-03	transcription_start_site	+	625911	625911	-4052	6281	AN10966
822	CONTIG129	621677	622041	2.07	3.31E-03	transcription_start_site	+	625989	625989	-4130	6282	AN10966
822	CONTIG129	621677	622041	2.07	3.31E-03	transcription_start_site	+	626100	626100	-4241	6283	AN10966
822	CONTIG129	621677	622041	2.07	3.31E-03	transcription_start_site	+	626324	626324	-4465	6284	AN10966
422	CONTIG129	278940	279369	2.57	9.51E-04	transcription_start_site	+	279659	279659	-504	6287	AN10968
686	CONTIG129	276917	277191	2.21	4.61E-03	transcription_start_site	+	279659	279659	-2605	6287	AN10968
747	CONTIG129	278030	278539	2.16	5.20E-03	transcription_start_site	+	279659	279659	-1374	6287	AN10968
2112	CONTIG129	275101	275600	1.33	1.08E-01	transcription_start_site	+	279659	279659	-4308	6287	AN10968
2639	CONTIG129	275641	276800	1.15	1.79E-01	transcription_start_site	+	279659	279659	-3438	6287	AN10968
1091	CONTIG129	573604	573893	1.84	1.84E-02	transcription_start_site	+	574514	574514	-765	6288	AN10969
1287	CONTIG129	571214	572153	1.7	1.23E-02	transcription_start_site	+	574514	574514	-2830	6288	AN10969
1880	CONTIG129	569034	569608	1.42	8.01E-02	transcription_start_site	+	574514	574514	-5193	6288	AN10969
1835	CONTIG16	173421	174072	1.44	9.26E-02	transcription_start_site	-	173297	173297	-449	6289	AN1097
1090	CONTIG129	531526	531800	1.84	1.84E-02	transcription_start_site	+	531082	531082	581	6301	AN10972
1090	CONTIG129	531526	531800	1.84	1.84E-02	transcription_start_site	+	530795	530795	868	6300	AN10972
1879	CONTIG129	525846	526120	1.42	8.01E-02	transcription_start_site	+	529389	529389	-3406	6297	AN10972
1879	CONTIG129	525846	526120	1.42	8.01E-02	transcription_start_site	+	530057	530057	-4074	6298	AN10972
1879	CONTIG129	525846	526120	1.42	8.01E-02	transcription_start_site	+	530460	530460	-4477	6299	AN10972
1879	CONTIG129	525846	526120	1.42	8.01E-02	transcription_start_site	+	530795	530795	-4812	6300	AN10972
1879	CONTIG129	525846	526120	1.42	8.01E-02	transcription_start_site	+	531082	531082	-5099	6301	AN10972
1205	CONTIG130	37438	37783	1.76	3.08E-02	transcription_start_site	+	38136	38136	-525	6306	AN10974
1205	CONTIG130	37438	37783	1.76	3.08E-02	transcription_start_site	+	38485	38485	-874	6307	AN10974
2549	CONTIG130	34353	35602	1.19	1.93E-01	transcription_start_site	+	38136	38136	-3158	6306	AN10974
2549	CONTIG130	34353	35602	1.19	1.93E-01	transcription_start_site	+	38485	38485	-3507	6307	AN10974
2265	CONTIG130	42312	42892	1.28	1.28E-01	transcription_start_site	+	46325	46325	-3723	6308	AN10975
2265	CONTIG130	42312	42892	1.28	1.28E-01	transcription_start_site	+	46537	46537	-3935	6309	AN10975
2265	CONTIG130	42312	42892	1.28	1.28E-01	transcription_start_site	+	46897	46897	-4295	6310	AN10975
279	CONTIG130	125035	125319	2.8	6.10E-04	transcription_start_site	-	124439	124439	-738	6315	AN10977
863	CONTIG130	153226	153505	2.04	1.11E-02	transcription_start_site	-	149936	149936	-3429	6316	AN10978
863	CONTIG130	153226	153505	2.04	1.11E-02	transcription_start_site	-	149814	149814	-3551	6317	AN10978
863	CONTIG130	153226	153505	2.04	1.11E-02	transcription_start_site	-	148646	148646	-4719	6318	AN10978
2550	CONTIG130	149853	150202	1.19	1.93E-01	transcription_start_site	-	149936	149936	-91	6316	AN10978
2550	CONTIG130	149853	150202	1.19	1.93E-01	transcription_start_site	-	149814	149814	-213	6317	AN10978
2550	CONTIG130	149853	150202	1.19	1.93E-01	transcription_start_site	-	148646	148646	-1381	6318	AN10978
1835	CONTIG16	173421	174072	1.44	9.26E-02	transcription_start_site	+	173767	173767	-20	6323	AN1098
1835	CONTIG16	173421	174072	1.44	9.26E-02	transcription_start_site	+	173609	173609	137	6322	AN1098

2237	CONTIG16	169355	170027	1.29	1.59E-01	transcription_start_site	+	173609	173609	-3918	6322	AN1098
2237	CONTIG16	169355	170027	1.29	1.59E-01	transcription_start_site	+	173767	173767	-4076	6323	AN1098
195	CONTIG130	191257	191526	2.95	2.67E-04	transcription_start_site	-	190738	190738	-653	6332	AN10982
195	CONTIG130	191257	191526	2.95	2.67E-04	transcription_start_site	-	190431	190431	-960	6333	AN10982
195	CONTIG130	191257	191526	2.95	2.67E-04	transcription_start_site	-	190239	190239	-1152	6334	AN10982
195	CONTIG130	191257	191526	2.95	2.67E-04	transcription_start_site	-	190100	190100	-1291	6335	AN10982
195	CONTIG130	191257	191526	2.95	2.67E-04	transcription_start_site	-	188135	188135	-3256	6336	AN10982
195	CONTIG130	191257	191526	2.95	2.67E-04	transcription_start_site	-	188060	188060	-3331	6337	AN10982
1984	CONTIG130	259969	260303	1.38	1.08E-01	transcription_start_site	+	260417	260417	-281	6338	AN10983
2110	CONTIG130	47927	48576	1.33	1.07E-01	transcription_start_site	+	47958	47958	293	6351	AN10987
279	CONTIG130	125035	125319	2.8	6.10E-04	transcription_start_site	-	123623	123623	-1554	6352	AN10988
279	CONTIG130	125035	125319	2.8	6.10E-04	transcription_start_site	-	122447	122447	-2730	6353	AN10988
1078	CONTIG130	122420	123219	1.85	2.12E-02	transcription_start_site	-	122447	122447	-372	6353	AN10988
1078	CONTIG130	122420	123219	1.85	2.12E-02	transcription_start_site	-	123623	123623	803	6352	AN10988
2550	CONTIG130	149853	150202	1.19	1.93E-01	transcription_start_site	-	148107	148107	-1920	6354	AN10989
415	CONTIG16	179121	179455	2.58	0.00E+00	transcription_start_site	+	178571	178571	717	6360	AN1099
1835	CONTIG16	173421	174072	1.44	9.26E-02	transcription_start_site	+	177199	177199	-3452	6355	AN1099
1835	CONTIG16	173421	174072	1.44	9.26E-02	transcription_start_site	+	177293	177293	-3546	6356	AN1099
1835	CONTIG16	173421	174072	1.44	9.26E-02	transcription_start_site	+	177695	177695	-3948	6357	AN1099
1835	CONTIG16	173421	174072	1.44	9.26E-02	transcription_start_site	+	177894	177894	-4147	6358	AN1099
1835	CONTIG16	173421	174072	1.44	9.26E-02	transcription_start_site	+	178013	178013	-4266	6359	AN1099
1835	CONTIG16	173421	174072	1.44	9.26E-02	transcription_start_site	+	178571	178571	-4824	6360	AN1099
1012	CONTIG130	176859	177214	1.9	1.84E-02	transcription_start_site	+	175926	175926	1110	6361	AN10990
1984	CONTIG130	259969	260303	1.38	1.08E-01	transcription_start_site	+	259020	259020	1116	6362	AN10991
195	CONTIG130	191257	191526	2.95	2.67E-04	transcription_start_site	-	186832	186832	-4559	6365	AN10993
195	CONTIG130	191257	191526	2.95	2.67E-04	transcription_start_site	-	186680	186680	-4711	6366	AN10993
2550	CONTIG130	149853	150202	1.19	1.93E-01	transcription_start_site	+	150195	150195	-167	6368	AN10994
2550	CONTIG130	149853	150202	1.19	1.93E-01	transcription_start_site	+	150419	150419	-391	6369	AN10994
625	CONTIG130	205890	206244	2.28	4.61E-03	transcription_start_site	-	205278	205278	-789	6370	AN10995
625	CONTIG130	205890	206244	2.28	4.61E-03	transcription_start_site	-	205163	205163	-904	6371	AN10995
1205	CONTIG130	37438	37783	1.76	3.08E-02	transcription_start_site	+	39204	39204	-1593	6372	AN10996
1205	CONTIG130	37438	37783	1.76	3.08E-02	transcription_start_site	+	39485	39485	-1874	6373	AN10996
1205	CONTIG130	37438	37783	1.76	3.08E-02	transcription_start_site	+	40708	40708	-3097	6374	AN10996
1205	CONTIG130	37438	37783	1.76	3.08E-02	transcription_start_site	+	40841	40841	-3230	6375	AN10996
2549	CONTIG130	34353	35602	1.19	1.93E-01	transcription_start_site	+	39204	39204	-4226	6372	AN10996
2549	CONTIG130	34353	35602	1.19	1.93E-01	transcription_start_site	+	39485	39485	-4507	6373	AN10996
2080	CONTIG132	43282	44091	1.34	8.01E-02	transcription_start_site	-	44196	44196	509	6421	AN11008
2080	CONTIG132	43282	44091	1.34	8.01E-02	transcription_start_site	-	42956	42956	-730	6422	AN11008
1766	CONTIG132	142133	142638	1.47	3.33E-02	transcription_start_site	-	140735	140735	-1650	6429	AN11010
1766	CONTIG132	142133	142638	1.47	3.33E-02	transcription_start_site	-	139879	139879	-2506	6430	AN11010
1766	CONTIG132	142133	142638	1.47	3.33E-02	transcription_start_site	-	139482	139482	-2903	6431	AN11010
2686	CONTIG132	142738	143307	1.13	1.66E-01	transcription_start_site	-	140735	140735	-2287	6429	AN11010
2686	CONTIG132	142738	143307	1.13	1.66E-01	transcription_start_site	-	139879	139879	-3143	6430	AN11010
2686	CONTIG132	142738	143307	1.13	1.66E-01	transcription_start_site	-	139482	139482	-3540	6431	AN11010
1669	CONTIG132	155028	155387	1.51	2.64E-02	transcription_start_site	-	155708	155708	500	6433	AN11011
1669	CONTIG132	155028	155387	1.51	2.64E-02	transcription_start_site	-	155834	155834	626	6432	AN11011
1669	CONTIG132	155028	155387	1.51	2.64E-02	transcription_start_site	-	154413	154413	-794	6434	AN11011
1669	CONTIG132	155028	155387	1.51	2.64E-02	transcription_start_site	-	154173	154173	-1034	6435	AN11011
2074	CONTIG132	153618	154777	1.34	4.45E-02	transcription_start_site	-	154173	154173	-24	6435	AN11011
2074	CONTIG132	153618	154777	1.34	4.45E-02	transcription_start_site	-	154413	154413	215	6434	AN11011
2074	CONTIG132	153618	154777	1.34	4.45E-02	transcription_start_site	-	155708	155708	1510	6433	AN11011
1669	CONTIG132	155028	155387	1.51	2.64E-02	transcription_start_site	-	153249	153249	-1958	6443	AN11016
1669	CONTIG132	155028	155387	1.51	2.64E-02	transcription_start_site	-	152227	152227	-2980	6444	AN11016
2074	CONTIG132	153618	154777	1.34	4.45E-02	transcription_start_site	-	153249	153249	-948	6443	AN11016
2074	CONTIG132	153618	154777	1.34	4.45E-02	transcription_start_site	-	152227	152227	-1970	6444	AN11016
869	CONTIG132	4651	5020	2.03	5.20E-03	transcription_start_site	-	4411	4411	-424	6452	AN11019
2685	CONTIG132	5946	6370	1.13	1.66E-01	transcription_start_site	-	4411	4411	-1747	6452	AN11019

711	CONTIG16	193816	194160	2.19	8.81E-03	transcription_start_site	-	193511	193511	-477	6453	AN1102
2687	CONTIG132	216301	216585	1.13	1.66E-01	transcription_start_site	+	219987	219987	-3544	6455	AN11021
2687	CONTIG132	216301	216585	1.13	1.66E-01	transcription_start_site	+	220059	220059	-3616	6456	AN11021
2687	CONTIG132	216301	216585	1.13	1.66E-01	transcription_start_site	+	220336	220336	-3893	6457	AN11021
2080	CONTIG132	43282	44091	1.34	8.01E-02	transcription_start_site	-	44557	44557	870	6459	AN11022
3049	CONTIG133	12906	13268	0.76	8.01E-02	transcription_start_site	+	12269	12269	818	6463	AN11024
2577	CONTIG135	174451	174725	1.17	6.84E-02	transcription_start_site	+	178417	178417	-3829	6474	AN11029
2577	CONTIG135	174451	174725	1.17	6.84E-02	transcription_start_site	+	178687	178687	-4099	6475	AN11029
2577	CONTIG135	174451	174725	1.17	6.84E-02	transcription_start_site	+	179054	179054	-4466	6476	AN11029
2577	CONTIG135	174451	174725	1.17	6.84E-02	transcription_start_site	+	179456	179456	-4868	6477	AN11029
711	CONTIG16	193816	194160	2.19	8.81E-03	transcription_start_site	+	196880	196880	-2892	6479	AN1103
711	CONTIG16	193816	194160	2.19	8.81E-03	transcription_start_site	+	197157	197157	-3169	6480	AN1103
326	CONTIG139	59480	59834	2.72	0.00E+00	transcription_start_site	+	59677	59677	-20	6490	AN11032
326	CONTIG139	59480	59834	2.72	0.00E+00	transcription_start_site	+	59794	59794	-137	6491	AN11032
326	CONTIG139	59480	59834	2.72	0.00E+00	transcription_start_site	+	59923	59923	-266	6492	AN11032
326	CONTIG139	59480	59834	2.72	0.00E+00	transcription_start_site	+	60194	60194	-537	6493	AN11032
326	CONTIG139	59480	59834	2.72	0.00E+00	transcription_start_site	+	60792	60792	-1135	6494	AN11032
326	CONTIG139	59480	59834	2.72	0.00E+00	transcription_start_site	+	61478	61478	-1821	6495	AN11032
429	CONTIG139	61370	61639	2.55	2.39E-04	transcription_start_site	+	61478	61478	26	6495	AN11032
429	CONTIG139	61370	61639	2.55	2.39E-04	transcription_start_site	+	60792	60792	712	6494	AN11032
2178	CONTIG139	61820	62529	1.3	6.16E-02	transcription_start_site	+	61478	61478	696	6495	AN11032
2681	CONTIG139	196592	197091	1.13	1.44E-01	transcription_start_site	+	198204	198204	-1362	6499	AN11034
2681	CONTIG139	196592	197091	1.13	1.44E-01	transcription_start_site	+	198304	198304	-1462	6500	AN11034
2681	CONTIG139	196592	197091	1.13	1.44E-01	transcription_start_site	+	198641	198641	-1799	6501	AN11034
2681	CONTIG139	196592	197091	1.13	1.44E-01	transcription_start_site	+	198939	198939	-2097	6502	AN11034
2681	CONTIG139	196592	197091	1.13	1.44E-01	transcription_start_site	+	199042	199042	-2200	6503	AN11034
2681	CONTIG139	196592	197091	1.13	1.44E-01	transcription_start_site	+	199203	199203	-2361	6504	AN11034
1265	CONTIG139	108156	110155	1.71	3.83E-03	transcription_start_site	-	108146	108146	-1009	6523	AN11039
1265	CONTIG139	108156	110155	1.71	3.83E-03	transcription_start_site	-	107784	107784	-1371	6524	AN11039
1265	CONTIG139	108156	110155	1.71	3.83E-03	transcription_start_site	-	107467	107467	-1688	6525	AN11039
1270	CONTIG139	115131	115705	1.71	1.57E-02	transcription_start_site	+	116964	116964	-1546	6529	AN11041
1270	CONTIG139	115131	115705	1.71	1.57E-02	transcription_start_site	+	117021	117021	-1603	6530	AN11041
1270	CONTIG139	115131	115705	1.71	1.57E-02	transcription_start_site	+	117132	117132	-1714	6531	AN11041
1270	CONTIG139	115131	115705	1.71	1.57E-02	transcription_start_site	+	117274	117274	-1856	6532	AN11041
1270	CONTIG139	115131	115705	1.71	1.57E-02	transcription_start_site	+	117389	117389	-1971	6533	AN11041
1270	CONTIG139	115131	115705	1.71	1.57E-02	transcription_start_site	+	119464	119464	-4046	6534	AN11042
2681	CONTIG139	196592	197091	1.13	1.44E-01	transcription_start_site	+	196845	196845	-3	6538	AN11046
2681	CONTIG139	196592	197091	1.13	1.44E-01	transcription_start_site	+	197100	197100	-258	6539	AN11046
2681	CONTIG139	196592	197091	1.13	1.44E-01	transcription_start_site	+	197381	197381	-539	6540	AN11046
2681	CONTIG139	196592	197091	1.13	1.44E-01	transcription_start_site	+	197582	197582	-740	6541	AN11046
2681	CONTIG139	196592	197091	1.13	1.44E-01	transcription_start_site	+	200714	200714	-3872	6542	AN11047
2681	CONTIG139	196592	197091	1.13	1.44E-01	transcription_start_site	+	201190	201190	-4348	6543	AN11047
2430	CONTIG141	184427	184851	1.22	3.99E-02	transcription_start_site	-	179802	179802	-4837	6561	AN11052
2434	CONTIG144	40069	40328	1.22	9.24E-02	transcription_start_site	-	41277	41277	1078	6571	AN11055
228	CONTIG144	49510	49961	2.88	0.00E+00	transcription_start_site	-	50541	50541	805	6573	AN11056
786	CONTIG144	50555	50909	2.11	1.80E-03	transcription_start_site	-	50541	50541	-191	6573	AN11056
786	CONTIG144	50555	50909	2.11	1.80E-03	transcription_start_site	-	51127	51127	395	6572	AN11056
2322	CONTIG144	14026	14465	1.26	8.01E-02	transcription_start_site	+	14019	14019	226	6580	AN11058
2322	CONTIG144	14026	14465	1.26	8.01E-02	transcription_start_site	+	13609	13609	636	6579	AN11058
2322	CONTIG144	14026	14465	1.26	8.01E-02	transcription_start_site	+	13482	13482	763	6578	AN11058
2322	CONTIG144	14026	14465	1.26	8.01E-02	transcription_start_site	+	15540	15540	-1294	6581	AN11059
2322	CONTIG144	14026	14465	1.26	8.01E-02	transcription_start_site	+	15807	15807	-1561	6582	AN11059
2322	CONTIG144	14026	14465	1.26	8.01E-02	transcription_start_site	+	16038	16038	-1792	6583	AN11059
2434	CONTIG144	40069	40328	1.22	9.24E-02	transcription_start_site	-	40270	40270	71	6588	AN11060
2434	CONTIG144	40069	40328	1.22	9.24E-02	transcription_start_site	-	40076	40076	-122	6589	AN11060
228	CONTIG144	49510	49961	2.88	0.00E+00	transcription_start_site	-	48824	48824	-911	6590	AN11061
228	CONTIG144	49510	49961	2.88	0.00E+00	transcription_start_site	-	48658	48658	-1077	6591	AN11061

228	CONTIG144	49510	49961	2.88	0.00E+00	transcription_start_site	-	47981	47981	-1754	6592	AN11061
786	CONTIG144	50555	50909	2.11	1.80E-03	transcription_start_site	-	48824	48824	-1908	6590	AN11061
786	CONTIG144	50555	50909	2.11	1.80E-03	transcription_start_site	-	48658	48658	-2074	6591	AN11061
786	CONTIG144	50555	50909	2.11	1.80E-03	transcription_start_site	-	47981	47981	-2751	6592	AN11061
62	CONTIG145	40201	40552	3.37	0.00E+00	transcription_start_site	+	41667	41667	-1290	6597	AN11064
62	CONTIG145	40201	40552	3.37	0.00E+00	transcription_start_site	+	41852	41852	-1475	6598	AN11064
62	CONTIG145	40201	40552	3.37	0.00E+00	transcription_start_site	+	42547	42547	-2170	6599	AN11064
62	CONTIG145	40201	40552	3.37	0.00E+00	transcription_start_site	+	42784	42784	-2407	6600	AN11064
1314	CONTIG145	38496	38900	1.68	3.08E-02	transcription_start_site	+	41667	41667	-2969	6597	AN11064
1314	CONTIG145	38496	38900	1.68	3.08E-02	transcription_start_site	+	41852	41852	-3154	6598	AN11064
1314	CONTIG145	38496	38900	1.68	3.08E-02	transcription_start_site	+	42547	42547	-3849	6599	AN11064
1314	CONTIG145	38496	38900	1.68	3.08E-02	transcription_start_site	+	42784	42784	-4086	6600	AN11064
2414	CONTIG145	71494	71843	1.23	1.44E-01	transcription_start_site	+	72567	72567	-898	6607	AN11067
2668	CONTIG145	22517	23001	1.14	1.79E-01	transcription_start_site	+	21898	21898	861	6612	AN11069
2668	CONTIG145	22517	23001	1.14	1.79E-01	transcription_start_site	+	21785	21785	974	6611	AN11069
62	CONTIG145	40201	40552	3.37	0.00E+00	transcription_start_site	+	43412	43412	-3035	6618	AN11070
1314	CONTIG145	38496	38900	1.68	3.08E-02	transcription_start_site	+	43412	43412	-4714	6618	AN11070
2774	CONTIG153	331728	332012	1.08	9.24E-02	transcription_start_site	-	332584	332584	714	6649	AN11080
2774	CONTIG153	331728	332012	1.08	9.24E-02	transcription_start_site	-	331724	331724	-146	6652	AN11082
2774	CONTIG153	331728	332012	1.08	9.24E-02	transcription_start_site	-	332069	332069	199	6651	AN11082
1818	CONTIG153	197631	197975	1.44	1.84E-02	transcription_start_site	+	201065	201065	-3262	6653	AN11083
1818	CONTIG153	197631	197975	1.44	1.84E-02	transcription_start_site	+	201150	201150	-3347	6654	AN11083
2631	CONTIG153	201246	201875	1.15	6.84E-02	transcription_start_site	+	201150	201150	410	6654	AN11083
2631	CONTIG153	201246	201875	1.15	6.84E-02	transcription_start_site	+	201065	201065	495	6653	AN11083
2883	CONTIG153	178276	178717	1.01	1.25E-01	transcription_start_site	-	174058	174058	-4438	6655	AN11084
2829	CONTIG157	65045	65454	1.05	1.50E-01	transcription_start_site	+	70079	70079	-4829	6662	AN11087
2095	CONTIG158	53412	54507	1.34	1.64E-01	transcription_start_site	-	53409	53409	-550	6674	AN11090
2095	CONTIG158	53412	54507	1.34	1.64E-01	transcription_start_site	-	52987	52987	-972	6675	AN11090
2095	CONTIG158	53412	54507	1.34	1.64E-01	transcription_start_site	-	52495	52495	-1464	6676	AN11090
2095	CONTIG158	53412	54507	1.34	1.64E-01	transcription_start_site	-	52339	52339	-1620	6677	AN11090
783	CONTIG158	102394	102669	2.12	2.62E-02	transcription_start_site	-	102293	102293	-238	6682	AN11093
783	CONTIG158	102394	102669	2.12	2.62E-02	transcription_start_site	-	101273	101273	-1258	6683	AN11093
783	CONTIG158	102394	102669	2.12	2.62E-02	transcription_start_site	-	100860	100860	-1671	6684	AN11093
2095	CONTIG158	53412	54507	1.34	1.64E-01	transcription_start_site	-	51814	51814	-2145	6686	AN11095
2095	CONTIG158	53412	54507	1.34	1.64E-01	transcription_start_site	-	51436	51436	-2523	6687	AN11095
2095	CONTIG158	53412	54507	1.34	1.64E-01	transcription_start_site	-	50719	50719	-3240	6688	AN11095
783	CONTIG158	102394	102669	2.12	2.62E-02	transcription_start_site	-	102474	102474	-57	6714	AN11100
783	CONTIG158	102394	102669	2.12	2.62E-02	transcription_start_site	-	103135	103135	603	6713	AN11100
783	CONTIG158	102394	102669	2.12	2.62E-02	transcription_start_site	-	103536	103536	1004	6712	AN11100
1539	CONTIG160	13351	14311	1.57	1.25E-01	transcription_start_site	+	17545	17545	-3714	6715	AN11101
1539	CONTIG160	13351	14311	1.57	1.25E-01	transcription_start_site	+	18181	18181	-4350	6716	AN11101
1539	CONTIG160	13351	14311	1.57	1.25E-01	transcription_start_site	+	18956	18956	-5125	6717	AN11101
1085	CONTIG160	80267	80601	1.85	5.88E-02	transcription_start_site	+	79908	79908	526	6731	AN11104
1085	CONTIG160	80267	80601	1.85	5.88E-02	transcription_start_site	+	79639	79639	795	6730	AN11104
1085	CONTIG160	80267	80601	1.85	5.88E-02	transcription_start_site	+	81692	81692	-1258	6732	AN11104
214	CONTIG160	125112	125381	2.91	1.80E-03	transcription_start_site	+	127330	127330	-2083	6733	AN11105
214	CONTIG160	125112	125381	2.91	1.80E-03	transcription_start_site	+	128289	128289	-3042	6734	AN11105
1085	CONTIG160	80267	80601	1.85	5.88E-02	transcription_start_site	+	82215	82215	-1781	6737	AN11107
1085	CONTIG160	80267	80601	1.85	5.88E-02	transcription_start_site	+	82807	82807	-2373	6738	AN11107
1085	CONTIG160	80267	80601	1.85	5.88E-02	transcription_start_site	+	83490	83490	-3056	6739	AN11107
214	CONTIG160	125112	125381	2.91	1.80E-03	transcription_start_site	+	125879	125879	-632	6740	AN11108
1539	CONTIG160	13351	14311	1.57	1.25E-01	transcription_start_site	+	15719	15719	-1888	6741	AN11109
1539	CONTIG160	13351	14311	1.57	1.25E-01	transcription_start_site	+	16061	16061	-2230	6742	AN11109
1929	CONTIG160	51617	51956	1.4	1.93E-01	transcription_start_site	+	50995	50995	791	6744	AN11110
306	CONTIG161	69904	70263	2.76	4.61E-03	transcription_start_site	+	70318	70318	-234	6749	AN11112
306	CONTIG161	69904	70263	2.76	4.61E-03	transcription_start_site	+	70486	70486	-402	6750	AN11112
306	CONTIG161	69904	70263	2.76	4.61E-03	transcription_start_site	+	70594	70594	-510	6751	AN11112

306	CONTIG161	69904	70263	2.76	4.61E-03	transcription_start_site	+	70754	70754	-670	6752	AN11112
1474	CONTIG161	66156	66594	1.61	1.25E-01	transcription_start_site	+	70318	70318	-3943	6749	AN11112
1474	CONTIG161	66156	66594	1.61	1.25E-01	transcription_start_site	+	70486	70486	-4111	6750	AN11112
1474	CONTIG161	66156	66594	1.61	1.25E-01	transcription_start_site	+	70594	70594	-4219	6751	AN11112
1474	CONTIG161	66156	66594	1.61	1.25E-01	transcription_start_site	+	70754	70754	-4379	6752	AN11112
1846	CONTIG161	97358	97777	1.44	1.93E-01	transcription_start_site	+	100760	100760	-3192	6753	AN11113
1846	CONTIG161	97358	97777	1.44	1.93E-01	transcription_start_site	+	101182	101182	-3614	6754	AN11113
1846	CONTIG161	97358	97777	1.44	1.93E-01	transcription_start_site	+	101291	101291	-3723	6755	AN11113
1846	CONTIG161	97358	97777	1.44	1.93E-01	transcription_start_site	+	101488	101488	-3920	6756	AN11113
1846	CONTIG161	97358	97777	1.44	1.93E-01	transcription_start_site	+	101680	101680	-4112	6757	AN11113
1846	CONTIG161	97358	97777	1.44	1.93E-01	transcription_start_site	+	102192	102192	-4624	6758	AN11113
1846	CONTIG161	97358	97777	1.44	1.93E-01	transcription_start_site	+	102770	102770	-5202	6759	AN11113
306	CONTIG161	69904	70263	2.76	4.61E-03	transcription_start_site	+	71963	71963	-1879	6773	AN11118
306	CONTIG161	69904	70263	2.76	4.61E-03	transcription_start_site	+	72412	72412	-2328	6774	AN11118
832	CONTIG161	104790	105279	2.07	2.18E-02	transcription_start_site	+	105104	105104	-69	6776	AN11119
832	CONTIG161	104790	105279	2.07	2.18E-02	transcription_start_site	+	104956	104956	78	6775	AN11119
832	CONTIG161	104790	105279	2.07	2.18E-02	transcription_start_site	+	105663	105663	-628	6777	AN11119
832	CONTIG161	104790	105279	2.07	2.18E-02	transcription_start_site	+	106029	106029	-994	6778	AN11119
832	CONTIG161	104790	105279	2.07	2.18E-02	transcription_start_site	+	106383	106383	-1348	6779	AN11119
1475	CONTIG161	103878	104537	1.61	1.25E-01	transcription_start_site	+	104956	104956	-748	6775	AN11119
1475	CONTIG161	103878	104537	1.61	1.25E-01	transcription_start_site	+	105104	105104	-896	6776	AN11119
1475	CONTIG161	103878	104537	1.61	1.25E-01	transcription_start_site	+	105663	105663	-1455	6777	AN11119
1475	CONTIG161	103878	104537	1.61	1.25E-01	transcription_start_site	+	106029	106029	-1821	6778	AN11119
1475	CONTIG161	103878	104537	1.61	1.25E-01	transcription_start_site	+	106383	106383	-2175	6779	AN11119
2090	CONTIG16	226217	226486	1.34	1.44E-01	transcription_start_site	-	223510	223510	-2841	6780	AN1112
832	CONTIG161	104790	105279	2.07	2.18E-02	transcription_start_site	+	107486	107486	-2451	6781	AN11120
832	CONTIG161	104790	105279	2.07	2.18E-02	transcription_start_site	+	107970	107970	-2935	6782	AN11120
832	CONTIG161	104790	105279	2.07	2.18E-02	transcription_start_site	+	108409	108409	-3374	6783	AN11120
1475	CONTIG161	103878	104537	1.61	1.25E-01	transcription_start_site	+	107486	107486	-3278	6781	AN11120
1475	CONTIG161	103878	104537	1.61	1.25E-01	transcription_start_site	+	107970	107970	-3762	6782	AN11120
1475	CONTIG161	103878	104537	1.61	1.25E-01	transcription_start_site	+	108409	108409	-4201	6783	AN11120
832	CONTIG161	104790	105279	2.07	2.18E-02	transcription_start_site	+	110232	110232	-5197	6784	AN11121
88	CONTIG161	121596	122010	3.27	8.69E-04	transcription_start_site	+	120909	120909	894	6787	AN11122
1030	CONTIG161	60161	60510	1.89	5.88E-02	transcription_start_site	-	60071	60071	-264	6790	AN11124
1584	CONTIG161	64746	65016	1.55	1.44E-01	transcription_start_site	-	60071	60071	-4810	6790	AN11124
382	CONTIG162	56033	56387	2.63	2.16E-04	transcription_start_site	+	59846	59846	-3636	6794	AN11126
382	CONTIG162	56033	56387	2.63	2.16E-04	transcription_start_site	+	60749	60749	-4539	6795	AN11126
382	CONTIG162	56033	56387	2.63	2.16E-04	transcription_start_site	+	61190	61190	-4980	6796	AN11126
1812	CONTIG162	54603	55037	1.45	5.88E-02	transcription_start_site	+	59846	59846	-5026	6794	AN11126
2837	CONTIG163	42240	42521	1.04	2.12E-02	transcription_start_site	-	38577	38577	-3803	6800	AN11129
2837	CONTIG163	42240	42521	1.04	2.12E-02	transcription_start_site	-	37758	37758	-4622	6801	AN11129
2090	CONTIG16	226217	226486	1.34	1.44E-01	transcription_start_site	-	225012	225012	-1339	6802	AN1113
2090	CONTIG16	226217	226486	1.34	1.44E-01	transcription_start_site	-	224938	224938	-1413	6803	AN1113
2090	CONTIG16	226217	226486	1.34	1.44E-01	transcription_start_site	-	224577	224577	-1774	6804	AN1113
2752	CONTIG163	88216	88570	1.09	1.57E-02	transcription_start_site	-	85608	85608	-2785	6805	AN11130
2752	CONTIG163	88216	88570	1.09	1.57E-02	transcription_start_site	-	85359	85359	-3034	6806	AN11130
2752	CONTIG163	88216	88570	1.09	1.57E-02	transcription_start_site	-	88129	88129	-264	6807	AN11131
2752	CONTIG163	88216	88570	1.09	1.57E-02	transcription_start_site	-	88040	88040	-353	6808	AN11131
2752	CONTIG163	88216	88570	1.09	1.57E-02	transcription_start_site	-	87947	87947	-446	6809	AN11131
2752	CONTIG163	88216	88570	1.09	1.57E-02	transcription_start_site	-	87753	87753	-640	6810	AN11131
3035	CONTIG163	46655	47319	0.82	8.01E-02	transcription_start_site	-	48216	48216	1229	6818	AN11136
3066	CONTIG163	52666	53080	0.66	1.93E-01	transcription_start_site	-	48412	48412	-4461	6817	AN11136
3066	CONTIG163	52666	53080	0.66	1.93E-01	transcription_start_site	-	48216	48216	-4657	6818	AN11136
2721	CONTIG163	78770	79116	1.11	5.41E-03	transcription_start_site	-	73836	73836	-5107	6819	AN11137
3034	CONTIG163	20195	20454	0.82	8.01E-02	transcription_start_site	-	15511	15511	-4813	6821	AN11138
2090	CONTIG16	226217	226486	1.34	1.44E-01	transcription_start_site	-	226650	226650	298	6828	AN1114
2090	CONTIG16	226217	226486	1.34	1.44E-01	transcription_start_site	-	225987	225987	-364	6829	AN1114

1444	CONTIG164	57980	58259	1.61	8.81E-03	transcription_start_site	+	61565	61565	-3445	6830	AN11141
1444	CONTIG164	57980	58259	1.61	8.81E-03	transcription_start_site	+	61827	61827	-3707	6831	AN11141
1100	CONTIG164	91292	91856	1.83	0.00E+00	transcription_start_site	-	88938	88938	-2636	6832	AN11142
1100	CONTIG164	91292	91856	1.83	0.00E+00	transcription_start_site	-	88692	88692	-2882	6833	AN11142
1100	CONTIG164	91292	91856	1.83	0.00E+00	transcription_start_site	-	88200	88200	-3374	6834	AN11142
1100	CONTIG164	91292	91856	1.83	0.00E+00	transcription_start_site	-	87767	87767	-3807	6835	AN11142
1588	CONTIG164	90007	90281	1.54	1.30E-02	transcription_start_site	-	88938	88938	-1206	6832	AN11142
1588	CONTIG164	90007	90281	1.54	1.30E-02	transcription_start_site	-	88692	88692	-1452	6833	AN11142
1588	CONTIG164	90007	90281	1.54	1.30E-02	transcription_start_site	-	88200	88200	-1944	6834	AN11142
1588	CONTIG164	90007	90281	1.54	1.30E-02	transcription_start_site	-	87767	87767	-2377	6835	AN11142
3070	CONTIG164	91892	95321	0.62	0.00E+00	transcription_start_site	-	88938	88938	-4668	6832	AN11142
3070	CONTIG164	91892	95321	0.62	0.00E+00	transcription_start_site	-	88692	88692	-4914	6833	AN11142
3070	CONTIG164	91892	95321	0.62	0.00E+00	transcription_start_site	-	88200	88200	-5406	6834	AN11142
3070	CONTIG164	91892	95321	0.62	0.00E+00	transcription_start_site	-	87767	87767	-5839	6835	AN11142
1849	CONTIG164	104943	105502	1.43	1.06E-02	transcription_start_site	+	107189	107189	-1966	6836	AN11143
1849	CONTIG164	104943	105502	1.43	1.06E-02	transcription_start_site	+	107489	107489	-2266	6837	AN11143
1849	CONTIG164	104943	105502	1.43	1.06E-02	transcription_start_site	+	107823	107823	-2600	6838	AN11143
1849	CONTIG164	104943	105502	1.43	1.06E-02	transcription_start_site	+	107969	107969	-2746	6839	AN11143
1849	CONTIG164	104943	105502	1.43	1.06E-02	transcription_start_site	+	108664	108664	-3441	6840	AN11143
2867	CONTIG164	105838	106112	1.03	1.25E-01	transcription_start_site	+	107189	107189	-1214	6836	AN11143
2867	CONTIG164	105838	106112	1.03	1.25E-01	transcription_start_site	+	107489	107489	-1514	6837	AN11143
2867	CONTIG164	105838	106112	1.03	1.25E-01	transcription_start_site	+	107823	107823	-1848	6838	AN11143
2867	CONTIG164	105838	106112	1.03	1.25E-01	transcription_start_site	+	107969	107969	-1994	6839	AN11143
2867	CONTIG164	105838	106112	1.03	1.25E-01	transcription_start_site	+	108664	108664	-2689	6840	AN11143
260	CONTIG164	48385	48891	2.82	0.00E+00	transcription_start_site	+	52506	52506	-3868	6841	AN11144
260	CONTIG164	48385	48891	2.82	0.00E+00	transcription_start_site	+	53497	53497	-4859	6842	AN11144
2032	CONTIG164	41779	42128	1.36	3.08E-02	transcription_start_site	+	42157	42157	-203	6843	AN11145
2032	CONTIG164	41779	42128	1.36	3.08E-02	transcription_start_site	+	42295	42295	-341	6844	AN11145
2032	CONTIG164	41779	42128	1.36	3.08E-02	transcription_start_site	+	42511	42511	-557	6845	AN11145
2460	CONTIG164	43205	43635	1.21	3.61E-02	transcription_start_site	+	42511	42511	909	6845	AN11145
2460	CONTIG164	43205	43635	1.21	3.61E-02	transcription_start_site	+	42295	42295	1125	6844	AN11145
3003	CONTIG164	39757	40101	0.92	1.93E-01	transcription_start_site	+	42157	42157	-2228	6843	AN11145
3003	CONTIG164	39757	40101	0.92	1.93E-01	transcription_start_site	+	42295	42295	-2366	6844	AN11145
3003	CONTIG164	39757	40101	0.92	1.93E-01	transcription_start_site	+	42511	42511	-2582	6845	AN11145
2032	CONTIG164	41779	42128	1.36	3.08E-02	transcription_start_site	-	40142	40142	-1811	6846	AN11146
2032	CONTIG164	41779	42128	1.36	3.08E-02	transcription_start_site	-	39991	39991	-1962	6847	AN11146
2032	CONTIG164	41779	42128	1.36	3.08E-02	transcription_start_site	-	39754	39754	-2199	6848	AN11146
2032	CONTIG164	41779	42128	1.36	3.08E-02	transcription_start_site	-	39576	39576	-2377	6849	AN11146
2032	CONTIG164	41779	42128	1.36	3.08E-02	transcription_start_site	-	39402	39402	-2551	6850	AN11146
2460	CONTIG164	43205	43635	1.21	3.61E-02	transcription_start_site	-	40142	40142	-3278	6846	AN11146
2460	CONTIG164	43205	43635	1.21	3.61E-02	transcription_start_site	-	39991	39991	-3429	6847	AN11146
2460	CONTIG164	43205	43635	1.21	3.61E-02	transcription_start_site	-	39754	39754	-3666	6848	AN11146
2460	CONTIG164	43205	43635	1.21	3.61E-02	transcription_start_site	-	39576	39576	-3844	6849	AN11146
2460	CONTIG164	43205	43635	1.21	3.61E-02	transcription_start_site	-	39402	39402	-4018	6850	AN11146
3003	CONTIG164	39757	40101	0.92	1.93E-01	transcription_start_site	-	39991	39991	62	6847	AN11146
3003	CONTIG164	39757	40101	0.92	1.93E-01	transcription_start_site	-	39754	39754	-175	6848	AN11146
3003	CONTIG164	39757	40101	0.92	1.93E-01	transcription_start_site	-	40142	40142	213	6846	AN11146
3003	CONTIG164	39757	40101	0.92	1.93E-01	transcription_start_site	-	39576	39576	-353	6849	AN11146
3003	CONTIG164	39757	40101	0.92	1.93E-01	transcription_start_site	-	39402	39402	-527	6850	AN11146
1849	CONTIG164	104943	105502	1.43	1.06E-02	transcription_start_site	+	109717	109717	-4494	6853	AN11149
1849	CONTIG164	104943	105502	1.43	1.06E-02	transcription_start_site	+	110042	110042	-4819	6854	AN11149
2867	CONTIG164	105838	106112	1.03	1.25E-01	transcription_start_site	+	109717	109717	-3742	6853	AN11149
2867	CONTIG164	105838	106112	1.03	1.25E-01	transcription_start_site	+	110042	110042	-4067	6854	AN11149
2867	CONTIG164	105838	106112	1.03	1.25E-01	transcription_start_site	+	110560	110560	-4585	6855	AN11149
2867	CONTIG164	105838	106112	1.03	1.25E-01	transcription_start_site	+	111087	111087	-5112	6856	AN11149
1100	CONTIG164	91292	91856	1.83	0.00E+00	transcription_start_site	-	86817	86817	-4757	6864	AN11150
1100	CONTIG164	91292	91856	1.83	0.00E+00	transcription_start_site	-	86535	86535	-5039	6865	AN11150

1588	CONTIG164	90007	90281	1.54	1.30E-02	transcription_start_site	-	86817	86817	-3327	6864 AN11150
1588	CONTIG164	90007	90281	1.54	1.30E-02	transcription_start_site	-	86535	86535	-3609	6865 AN11150
981	CONTIG165	50180	50604	1.92	1.35E-03	transcription_start_site	-	49773	49773	-619	6866 AN11151
981	CONTIG165	50180	50604	1.92	1.35E-03	transcription_start_site	-	48975	48975	-1417	6867 AN11151
349	CONTIG165	94355	94719	2.68	0.00E+00	transcription_start_site	+	98030	98030	-3493	6868 AN11152
349	CONTIG165	94355	94719	2.68	0.00E+00	transcription_start_site	+	98155	98155	-3618	6869 AN11152
349	CONTIG165	94355	94719	2.68	0.00E+00	transcription_start_site	+	98239	98239	-3702	6870 AN11152
349	CONTIG165	94355	94719	2.68	0.00E+00	transcription_start_site	+	98308	98308	-3771	6871 AN11152
349	CONTIG165	94355	94719	2.68	0.00E+00	transcription_start_site	+	99142	99142	-4605	6872 AN11152
2467	CONTIG165	96006	97625	1.21	5.82E-02	transcription_start_site	+	98030	98030	-1214	6868 AN11152
2467	CONTIG165	96006	97625	1.21	5.82E-02	transcription_start_site	+	98155	98155	-1339	6869 AN11152
2467	CONTIG165	96006	97625	1.21	5.82E-02	transcription_start_site	+	98239	98239	-1423	6870 AN11152
2467	CONTIG165	96006	97625	1.21	5.82E-02	transcription_start_site	+	98308	98308	-1492	6871 AN11152
2467	CONTIG165	96006	97625	1.21	5.82E-02	transcription_start_site	+	99142	99142	-2326	6872 AN11152
2684	CONTIG165	99171	99650	1.13	1.44E-01	transcription_start_site	+	99142	99142	268	6872 AN11152
2684	CONTIG165	99171	99650	1.13	1.44E-01	transcription_start_site	+	98308	98308	1102	6871 AN11152
2684	CONTIG165	99171	99650	1.13	1.44E-01	transcription_start_site	+	98239	98239	1171	6870 AN11152
981	CONTIG165	50180	50604	1.92	1.35E-03	transcription_start_site	-	47922	47922	-2470	6873 AN11153
981	CONTIG165	50180	50604	1.92	1.35E-03	transcription_start_site	-	47683	47683	-2709	6874 AN11153
981	CONTIG165	50180	50604	1.92	1.35E-03	transcription_start_site	-	47514	47514	-2878	6875 AN11153
981	CONTIG165	50180	50604	1.92	1.35E-03	transcription_start_site	-	47309	47309	-3083	6876 AN11153
981	CONTIG165	50180	50604	1.92	1.35E-03	transcription_start_site	-	46571	46571	-3821	6877 AN11153
981	CONTIG165	50180	50604	1.92	1.35E-03	transcription_start_site	-	46338	46338	-4054	6878 AN11153
981	CONTIG165	50180	50604	1.92	1.35E-03	transcription_start_site	-	45745	45745	-4647	6879 AN11154
981	CONTIG165	50180	50604	1.92	1.35E-03	transcription_start_site	-	45379	45379	-5013	6880 AN11154
349	CONTIG165	94355	94719	2.68	0.00E+00	transcription_start_site	+	97367	97367	-2830	6881 AN11155
349	CONTIG165	94355	94719	2.68	0.00E+00	transcription_start_site	+	97502	97502	-2965	6882 AN11155
2467	CONTIG165	96006	97625	1.21	5.82E-02	transcription_start_site	+	97367	97367	-551	6881 AN11155
2467	CONTIG165	96006	97625	1.21	5.82E-02	transcription_start_site	+	97502	97502	-686	6882 AN11155
472	CONTIG167	12082	12351	2.48	4.61E-03	transcription_start_site	-	10890	10890	-1326	6883 AN11156
472	CONTIG167	12082	12351	2.48	4.61E-03	transcription_start_site	-	10713	10713	-1503	6884 AN11156
472	CONTIG167	12082	12351	2.48	4.61E-03	transcription_start_site	-	9473	9473	-2743	6885 AN11157
472	CONTIG167	12082	12351	2.48	4.61E-03	transcription_start_site	-	9303	9303	-2913	6886 AN11157
217	CONTIG168	121218	121575	2.9	0.00E+00	transcription_start_site	-	117956	117956	-3440	6887 AN11158
217	CONTIG168	121218	121575	2.9	0.00E+00	transcription_start_site	-	117644	117644	-3752	6888 AN11158
217	CONTIG168	121218	121575	2.9	0.00E+00	transcription_start_site	-	117253	117253	-4143	6889 AN11158
2183	CONTIG168	172954	173393	1.3	8.01E-02	transcription_start_site	+	177078	177078	-3904	6890 AN11159
2183	CONTIG168	172954	173393	1.3	8.01E-02	transcription_start_site	+	177285	177285	-4111	6891 AN11159
2183	CONTIG168	172954	173393	1.3	8.01E-02	transcription_start_site	+	177483	177483	-4309	6892 AN11159
2183	CONTIG168	172954	173393	1.3	8.01E-02	transcription_start_site	+	177924	177924	-4750	6893 AN11159
2183	CONTIG168	172954	173393	1.3	8.01E-02	transcription_start_site	+	178035	178035	-4861	6894 AN11159
2759	CONTIG168	171834	172328	1.09	1.66E-01	transcription_start_site	+	177078	177078	-4997	6890 AN11159
2759	CONTIG168	171834	172328	1.09	1.66E-01	transcription_start_site	+	177285	177285	-5204	6891 AN11159
2090	CONTIG16	226217	226486	1.34	1.44E-01	transcription_start_site	+	231425	231425	-5073	6895 AN1116
217	CONTIG168	121218	121575	2.9	0.00E+00	transcription_start_site	-	116254	116254	-5142	6896 AN11160
387	CONTIG169	142438	142927	2.62	2.67E-04	transcription_start_site	-	139572	139572	-3110	6907 AN11164
387	CONTIG169	142438	142927	2.62	2.67E-04	transcription_start_site	-	138748	138748	-3934	6908 AN11164
387	CONTIG169	142438	142927	2.62	2.67E-04	transcription_start_site	-	138470	138470	-4212	6909 AN11164
387	CONTIG169	142438	142927	2.62	2.67E-04	transcription_start_site	-	138283	138283	-4399	6910 AN11164
387	CONTIG169	142438	142927	2.62	2.67E-04	transcription_start_site	-	137984	137984	-4698	6911 AN11164
1451	CONTIG169	169445	169714	1.61	2.62E-02	transcription_start_site	+	170538	170538	-958	6913 AN11165
1451	CONTIG169	169445	169714	1.61	2.62E-02	transcription_start_site	+	170713	170713	-1133	6914 AN11165
2282	CONTIG169	173180	174436	1.27	7.02E-02	transcription_start_site	-	174673	174673	865	6918 AN11166
1057	CONTIG169	232520	232784	1.86	8.81E-03	transcription_start_site	+	233124	233124	-472	6919 AN11167
2816	CONTIG169	255531	255800	1.06	1.93E-01	transcription_start_site	-	256405	256405	739	6921 AN11168
2816	CONTIG169	255531	255800	1.06	1.93E-01	transcription_start_site	-	256597	256597	931	6920 AN11168
1745	CONTIG169	284708	284977	1.48	4.36E-02	transcription_start_site	+	287184	287184	-2341	6922 AN11169

1745	CONTIG169	284708	284977	1.48	4.36E-02	transcription_start_site	+	287474	287474	-2631	6923	AN11169
1745	CONTIG169	284708	284977	1.48	4.36E-02	transcription_start_site	+	288041	288041	-3198	6924	AN11169
1745	CONTIG169	284708	284977	1.48	4.36E-02	transcription_start_site	+	288241	288241	-3398	6925	AN11169
1745	CONTIG169	284708	284977	1.48	4.36E-02	transcription_start_site	+	288464	288464	-3621	6926	AN11169
1451	CONTIG169	169445	169714	1.61	2.62E-02	transcription_start_site	+	169509	169509	70	6946	AN11176
2282	CONTIG169	173180	174436	1.27	7.02E-02	transcription_start_site	-	174246	174246	438	6947	AN11177
912	CONTIG169	235968	236227	1.99	5.20E-03	transcription_start_site	+	235334	235334	763	6949	AN11178
912	CONTIG169	235968	236227	1.99	5.20E-03	transcription_start_site	+	235129	235129	968	6948	AN11178
1057	CONTIG169	232520	232784	1.86	8.81E-03	transcription_start_site	+	235129	235129	-2477	6948	AN11178
1057	CONTIG169	232520	232784	1.86	8.81E-03	transcription_start_site	+	235334	235334	-2682	6949	AN11178
1309	CONTIG16	240239	240803	1.69	5.22E-02	transcription_start_site	-	237451	237451	-3070	6951	AN1118
774	CONTIG169	289503	289777	2.12	3.03E-03	transcription_start_site	+	289688	289688	-48	6968	AN11184
774	CONTIG169	289503	289777	2.12	3.03E-03	transcription_start_site	+	290448	290448	-808	6969	AN11184
1745	CONTIG169	284708	284977	1.48	4.36E-02	transcription_start_site	+	289688	289688	-4845	6968	AN11184
774	CONTIG169	289503	289777	2.12	3.03E-03	transcription_start_site	+	291182	291182	-1542	6970	AN11185
774	CONTIG169	289503	289777	2.12	3.03E-03	transcription_start_site	+	292449	292449	-2809	6971	AN11185
2288	CONTIG169	291768	292037	1.27	9.24E-02	transcription_start_site	+	292449	292449	-546	6971	AN11185
2288	CONTIG169	291768	292037	1.27	9.24E-02	transcription_start_site	+	291182	291182	720	6970	AN11185
2161	CONTIG169	326496	326985	1.31	6.16E-02	transcription_start_site	-	327655	327655	914	6980	AN11187
2830	CONTIG170	24842	25401	1.05	1.66E-01	transcription_start_site	-	20565	20565	-4556	6981	AN11188
2830	CONTIG170	24842	25401	1.05	1.66E-01	transcription_start_site	-	20295	20295	-4826	6982	AN11188
2830	CONTIG170	24842	25401	1.05	1.66E-01	transcription_start_site	-	20064	20064	-5057	6983	AN11188
1309	CONTIG16	240239	240803	1.69	5.22E-02	transcription_start_site	-	239192	239192	-1329	6992	AN1119
1309	CONTIG16	240239	240803	1.69	5.22E-02	transcription_start_site	-	239014	239014	-1507	6993	AN1119
835	CONTIG170	168167	168441	2.06	2.22E-03	transcription_start_site	-	166416	166416	-1888	7001	AN11192
835	CONTIG170	168167	168441	2.06	2.22E-03	transcription_start_site	-	166182	166182	-2122	7002	AN11192
835	CONTIG170	168167	168441	2.06	2.22E-03	transcription_start_site	-	165307	165307	-2997	7003	AN11192
835	CONTIG170	168167	168441	2.06	2.22E-03	transcription_start_site	-	165164	165164	-3140	7004	AN11192
834	CONTIG170	35119	35768	2.06	0.00E+00	transcription_start_site	+	39976	39976	-4532	7010	AN11195
2830	CONTIG170	24842	25401	1.05	1.66E-01	transcription_start_site	+	24003	24003	1118	7018	AN11197
1309	CONTIG16	240239	240803	1.69	5.22E-02	transcription_start_site	-	240985	240985	464	7024	AN1120
1309	CONTIG16	240239	240803	1.69	5.22E-02	transcription_start_site	-	241156	241156	635	7023	AN1120
835	CONTIG170	168167	168441	2.06	2.22E-03	transcription_start_site	-	167440	167440	-864	7025	AN11200
835	CONTIG170	168167	168441	2.06	2.22E-03	transcription_start_site	-	167331	167331	-973	7026	AN11200
1309	CONTIG16	240239	240803	1.69	5.22E-02	transcription_start_site	+	241511	241511	-990	7057	AN1121
534	CONTIG172	427209	427493	2.39	0.00E+00	transcription_start_site	-	425129	425129	-2222	7073	AN11213
534	CONTIG172	427209	427493	2.39	0.00E+00	transcription_start_site	-	424888	424888	-2463	7074	AN11213
2777	CONTIG172	428704	429133	1.08	1.08E-01	transcription_start_site	-	425129	425129	-3789	7073	AN11213
2777	CONTIG172	428704	429133	1.08	1.08E-01	transcription_start_site	-	424888	424888	-4030	7074	AN11213
534	CONTIG172	427209	427493	2.39	0.00E+00	transcription_start_site	-	425775	425775	-1576	7078	AN11216
534	CONTIG172	427209	427493	2.39	0.00E+00	transcription_start_site	-	425599	425599	-1752	7079	AN11216
2777	CONTIG172	428704	429133	1.08	1.08E-01	transcription_start_site	-	425775	425775	-3143	7078	AN11216
2777	CONTIG172	428704	429133	1.08	1.08E-01	transcription_start_site	-	425599	425599	-3319	7079	AN11216
2914	CONTIG174	16801	17080	0.99	2.62E-02	transcription_start_site	-	17017	17017	76	7110	AN11225
2914	CONTIG174	16801	17080	0.99	2.62E-02	transcription_start_site	-	14645	14645	-2295	7111	AN11226
2914	CONTIG174	16801	17080	0.99	2.62E-02	transcription_start_site	-	14584	14584	-2356	7112	AN11226
2914	CONTIG174	16801	17080	0.99	2.62E-02	transcription_start_site	-	14445	14445	-2495	7113	AN11226
2914	CONTIG174	16801	17080	0.99	2.62E-02	transcription_start_site	-	14192	14192	-2748	7114	AN11226
2914	CONTIG174	16801	17080	0.99	2.62E-02	transcription_start_site	-	13890	13890	-3050	7115	AN11226
2980	CONTIG175	15301	15803	0.93	5.22E-02	transcription_start_site	+	18281	18281	-2729	7116	AN11227
2980	CONTIG175	15301	15803	0.93	5.22E-02	transcription_start_site	+	18376	18376	-2824	7117	AN11227
2980	CONTIG175	15301	15803	0.93	5.22E-02	transcription_start_site	+	18479	18479	-2927	7118	AN11227
2980	CONTIG175	15301	15803	0.93	5.22E-02	transcription_start_site	+	18655	18655	-3103	7119	AN11227
2980	CONTIG175	15301	15803	0.93	5.22E-02	transcription_start_site	+	18873	18873	-3321	7120	AN11227
2980	CONTIG175	15301	15803	0.93	5.22E-02	transcription_start_site	+	19169	19169	-3617	7121	AN11227
2980	CONTIG175	15301	15803	0.93	5.22E-02	transcription_start_site	+	19292	19292	-3740	7122	AN11227
3063	CONTIG175	17706	18140	0.68	1.93E-01	transcription_start_site	+	18281	18281	-358	7116	AN11227

3063	CONTIG175	17706	18140	0.68	1.93E-01	transcription_start_site	+	18376	18376	-453	7117 AN11227
3063	CONTIG175	17706	18140	0.68	1.93E-01	transcription_start_site	+	18479	18479	-556	7118 AN11227
3063	CONTIG175	17706	18140	0.68	1.93E-01	transcription_start_site	+	18655	18655	-732	7119 AN11227
3063	CONTIG175	17706	18140	0.68	1.93E-01	transcription_start_site	+	18873	18873	-950	7120 AN11227
3063	CONTIG175	17706	18140	0.68	1.93E-01	transcription_start_site	+	19169	19169	-1246	7121 AN11227
3063	CONTIG175	17706	18140	0.68	1.93E-01	transcription_start_site	+	19292	19292	-1369	7122 AN11227
1027	CONTIG16	249842	250104	1.89	2.62E-02	transcription_start_site	-	246356	246356	-3617	7131 AN1123
1027	CONTIG16	249842	250104	1.89	2.62E-02	transcription_start_site	-	246268	246268	-3705	7132 AN1123
1027	CONTIG16	249842	250104	1.89	2.62E-02	transcription_start_site	-	246099	246099	-3874	7133 AN1123
1027	CONTIG16	249842	250104	1.89	2.62E-02	transcription_start_site	-	245962	245962	-4011	7134 AN1123
1027	CONTIG16	249842	250104	1.89	2.62E-02	transcription_start_site	-	245481	245481	-4492	7135 AN1123
1027	CONTIG16	249842	250104	1.89	2.62E-02	transcription_start_site	-	245304	245304	-4669	7136 AN1123
3069	CONTIG179	3156	3445	0.63	9.24E-02	transcription_start_site	+	4047	4047	-746	7137 AN11230
3069	CONTIG179	3156	3445	0.63	9.24E-02	transcription_start_site	+	4188	4188	-887	7138 AN11230
3069	CONTIG179	3156	3445	0.63	9.24E-02	transcription_start_site	+	2586	2586	714	7143 AN11231
3069	CONTIG179	3156	3445	0.63	9.24E-02	transcription_start_site	+	2343	2343	957	7142 AN11231
3045	CONTIG183	5926	6220	0.77	9.24E-02	transcription_start_site	+	6012	6012	61	7144 AN11232
3045	CONTIG183	5926	6220	0.77	9.24E-02	transcription_start_site	+	6449	6449	-376	7145 AN11232
3041	CONTIG183	9246	9742	0.79	8.01E-02	transcription_start_site	+	9301	9301	193	7148 AN11233
3041	CONTIG183	9246	9742	0.79	8.01E-02	transcription_start_site	+	9793	9793	-299	7149 AN11233
3041	CONTIG183	9246	9742	0.79	8.01E-02	transcription_start_site	+	9925	9925	-431	7150 AN11233
3041	CONTIG183	9246	9742	0.79	8.01E-02	transcription_start_site	+	8353	8353	1141	7147 AN11233
3045	CONTIG183	5926	6220	0.77	9.24E-02	transcription_start_site	+	8213	8213	-2140	7146 AN11233
3045	CONTIG183	5926	6220	0.77	9.24E-02	transcription_start_site	+	8353	8353	-2280	7147 AN11233
3045	CONTIG183	5926	6220	0.77	9.24E-02	transcription_start_site	+	9301	9301	-3228	7148 AN11233
3045	CONTIG183	5926	6220	0.77	9.24E-02	transcription_start_site	+	9793	9793	-3720	7149 AN11233
3045	CONTIG183	5926	6220	0.77	9.24E-02	transcription_start_site	+	9925	9925	-3852	7150 AN11233
146	CONTIG186	2026	2395	3.06	2.49E-03	transcription_start_site	+	1370	1370	840	7153 AN11234
146	CONTIG186	2026	2395	3.06	2.49E-03	transcription_start_site	+	1235	1235	975	7152 AN11234
146	CONTIG186	2026	2395	3.06	2.49E-03	transcription_start_site	+	1167	1167	1043	7151 AN11234
1027	CONTIG16	249842	250104	1.89	2.62E-02	transcription_start_site	+	249363	249363	610	7166 AN1124
1027	CONTIG16	249842	250104	1.89	2.62E-02	transcription_start_site	+	249198	249198	775	7165 AN1124
2343	CONTIG197	466	1125	1.25	3.08E-02	transcription_start_site	+	1083	1083	-287	7167 AN11240
2343	CONTIG197	466	1125	1.25	3.08E-02	transcription_start_site	+	6043	6043	-5247	7169 AN11242
1804	CONTIG199	1436	1795	1.45	1.30E-02	transcription_start_site	+	4536	4536	-2920	7171 AN11243
1804	CONTIG199	1436	1795	1.45	1.30E-02	transcription_start_site	+	4842	4842	-3226	7172 AN11243
3064	CONTIG204	6016	6350	0.66	1.50E-01	transcription_start_site	-	3998	3998	-2185	7173 AN11244
3064	CONTIG204	6016	6350	0.66	1.50E-01	transcription_start_site	-	3865	3865	-2318	7174 AN11244
3064	CONTIG204	6016	6350	0.66	1.50E-01	transcription_start_site	-	3726	3726	-2457	7175 AN11244
3064	CONTIG204	6016	6350	0.66	1.50E-01	transcription_start_site	-	3093	3093	-3090	7176 AN11244
3064	CONTIG204	6016	6350	0.66	1.50E-01	transcription_start_site	-	5757	5757	-426	7177 AN11245
3064	CONTIG204	6016	6350	0.66	1.50E-01	transcription_start_site	-	5315	5315	-868	7178 AN11245
2153	CONTIG209	2121	3125	1.31	1.17E-03	transcription_start_site	+	4689	4689	-2066	7179 AN11246
3026	CONTIG209	4969	5696	0.86	9.26E-02	transcription_start_site	+	4689	4689	643	7179 AN11246
3047	CONTIG209	3619	4558	0.77	1.59E-01	transcription_start_site	+	4689	4689	-600	7179 AN11246
2518	CONTIG221	3166	3516	1.2	1.93E-01	transcription_start_site	+	2842	2842	499	7182 AN11248
2518	CONTIG221	3166	3516	1.2	1.93E-01	transcription_start_site	+	2689	2689	652	7181 AN11248
2518	CONTIG221	3166	3516	1.2	1.93E-01	transcription_start_site	+	2463	2463	878	7180 AN11248
1027	CONTIG16	249842	250104	1.89	2.62E-02	transcription_start_site	-	249896	249896	-77	7184 AN1125
1027	CONTIG16	249842	250104	1.89	2.62E-02	transcription_start_site	-	250113	250113	140	7183 AN1125
1162	CONTIG16	252455	252814	1.79	3.70E-02	transcription_start_site	-	250113	250113	-2521	7183 AN1125
1162	CONTIG16	252455	252814	1.79	3.70E-02	transcription_start_site	-	249896	249896	-2738	7184 AN1125
1580	CONTIG84	112505	112940	1.55	9.24E-02	transcription_start_site	+	114997	114997	-2274	7190 AN11253
1580	CONTIG84	112505	112940	1.55	9.24E-02	transcription_start_site	+	115900	115900	-3177	7191 AN11253
1286	CONTIG2	75677	76037	1.7	5.20E-03	transcription_start_site	-	76104	76104	247	7202 AN11259
1286	CONTIG2	75677	76037	1.7	5.20E-03	transcription_start_site	-	76160	76160	303	7201 AN11259
2068	CONTIG2	74717	75291	1.34	3.08E-02	transcription_start_site	-	76104	76104	1100	7202 AN11259

2068	CONTIG2	74717	75291	1.34	3.08E-02	transcription_start_site	-	76160	76160	1156	7201	AN11259
1027	CONTIG16	249842	250104	1.89	2.62E-02	transcription_start_site	+	250643	250643	-670	7203	AN1126
1027	CONTIG16	249842	250104	1.89	2.62E-02	transcription_start_site	+	250900	250900	-927	7204	AN1126
1027	CONTIG16	249842	250104	1.89	2.62E-02	transcription_start_site	+	251201	251201	-1228	7205	AN1126
1027	CONTIG16	249842	250104	1.89	2.62E-02	transcription_start_site	+	251319	251319	-1346	7206	AN1126
1815	CONTIG5	46958	47458	1.45	1.66E-01	transcription_start_site	-	44475	44475	-2733	7207	AN11260
1815	CONTIG5	46958	47458	1.45	1.66E-01	transcription_start_site	-	44249	44249	-2959	7208	AN11260
1967	CONTIG5	67507	68076	1.39	1.93E-01	transcription_start_site	+	69123	69123	-1331	7209	AN11261
1967	CONTIG5	67507	68076	1.39	1.93E-01	transcription_start_site	+	69287	69287	-1495	7210	AN11261
581	CONTIG5	102237	102741	2.34	1.30E-02	transcription_start_site	+	102547	102547	-58	7212	AN11262
581	CONTIG5	102237	102741	2.34	1.30E-02	transcription_start_site	+	102294	102294	195	7211	AN11262
581	CONTIG5	102237	102741	2.34	1.30E-02	transcription_start_site	+	102792	102792	-303	7213	AN11262
986	CONTIG6	32254	32528	1.92	7.85E-03	transcription_start_site	-	32398	32398	7	7222	AN11266
986	CONTIG6	32254	32528	1.92	7.85E-03	transcription_start_site	-	32758	32758	367	7221	AN11266
176	CONTIG6	87761	88408	2.99	0.00E+00	transcription_start_site	+	92873	92873	-4788	7223	AN11267
176	CONTIG6	87761	88408	2.99	0.00E+00	transcription_start_site	+	93145	93145	-5060	7224	AN11267
1162	CONTIG16	252455	252814	1.79	3.70E-02	transcription_start_site	-	252862	252862	227	7232	AN1127
1162	CONTIG16	252455	252814	1.79	3.70E-02	transcription_start_site	-	252932	252932	297	7231	AN1127
1731	CONTIG16	257719	258133	1.49	9.24E-02	transcription_start_site	-	252932	252932	-4994	7231	AN1127
1731	CONTIG16	257719	258133	1.49	9.24E-02	transcription_start_site	-	252862	252862	-5064	7232	AN1127
947	CONTIG7	508741	509232	1.96	7.85E-03	transcription_start_site	+	513357	513357	-4370	7242	AN11273
947	CONTIG7	508741	509232	1.96	7.85E-03	transcription_start_site	+	513463	513463	-4476	7243	AN11273
947	CONTIG7	508741	509232	1.96	7.85E-03	transcription_start_site	+	513654	513654	-4667	7244	AN11273
947	CONTIG7	508741	509232	1.96	7.85E-03	transcription_start_site	+	514072	514072	-5085	7245	AN11273
614	CONTIG10	96542	97104	2.29	4.46E-04	transcription_start_site	+	96137	96137	686	7262	AN11279
614	CONTIG10	96542	97104	2.29	4.46E-04	transcription_start_site	+	95789	95789	1034	7261	AN11279
1117	CONTIG10	94737	95316	1.82	2.12E-02	transcription_start_site	+	95789	95789	-762	7261	AN11279
1117	CONTIG10	94737	95316	1.82	2.12E-02	transcription_start_site	+	96137	96137	-1110	7262	AN11279
1027	CONTIG16	249842	250104	1.89	2.62E-02	transcription_start_site	+	254619	254619	-4646	7263	AN1128
1162	CONTIG16	252455	252814	1.79	3.70E-02	transcription_start_site	+	254619	254619	-1984	7263	AN1128
1464	CONTIG12	45608	45892	1.61	4.36E-02	transcription_start_site	+	50677	50677	-4927	7264	AN11280
973	CONTIG14	261244	261878	1.93	4.81E-03	transcription_start_site	-	257115	257115	-4446	7274	AN11285
973	CONTIG14	261244	261878	1.93	4.81E-03	transcription_start_site	-	256764	256764	-4797	7275	AN11285
973	CONTIG14	261244	261878	1.93	4.81E-03	transcription_start_site	-	256634	256634	-4927	7276	AN11285
616	CONTIG14	344115	344397	2.29	2.22E-03	transcription_start_site	-	341574	341574	-2682	7277	AN11286
616	CONTIG14	344115	344397	2.29	2.22E-03	transcription_start_site	-	341468	341468	-2788	7278	AN11286
2776	CONTIG15	145051	146765	1.08	1.01E-01	transcription_start_site	-	141498	141498	-4410	7279	AN11287
2776	CONTIG15	145051	146765	1.08	1.01E-01	transcription_start_site	-	141381	141381	-4527	7280	AN11287
2776	CONTIG15	145051	146765	1.08	1.01E-01	transcription_start_site	-	141122	141122	-4786	7281	AN11287
522	CONTIG15	154220	154494	2.41	2.16E-04	transcription_start_site	+	154905	154905	-548	7282	AN11288
522	CONTIG15	154220	154494	2.41	2.16E-04	transcription_start_site	+	155055	155055	-698	7283	AN11288
1242	CONTIG15	155570	156054	1.73	1.11E-02	transcription_start_site	+	155055	155055	757	7283	AN11288
1242	CONTIG15	155570	156054	1.73	1.11E-02	transcription_start_site	+	154905	154905	907	7282	AN11288
1366	CONTIG15	152195	152529	1.65	1.57E-02	transcription_start_site	+	154905	154905	-2543	7282	AN11288
1366	CONTIG15	152195	152529	1.65	1.57E-02	transcription_start_site	+	155055	155055	-2693	7283	AN11288
1362	CONTIG15	171379	172038	1.65	7.16E-03	transcription_start_site	-	169157	169157	-2551	7284	AN11289
1362	CONTIG15	171379	172038	1.65	7.16E-03	transcription_start_site	-	168772	168772	-2936	7285	AN11289
1731	CONTIG16	257719	258133	1.49	9.24E-02	transcription_start_site	+	258104	258104	-178	7286	AN1129
1731	CONTIG16	257719	258133	1.49	9.24E-02	transcription_start_site	+	258766	258766	-840	7287	AN1129
1731	CONTIG16	257719	258133	1.49	9.24E-02	transcription_start_site	+	258919	258919	-993	7288	AN1129
1731	CONTIG16	257719	258133	1.49	9.24E-02	transcription_start_site	+	259477	259477	-1551	7289	AN1129
1731	CONTIG16	257719	258133	1.49	9.24E-02	transcription_start_site	+	260013	260013	-2087	7290	AN1129
32	CONTIG16	24243	24657	3.53	0.00E+00	transcription_start_site	-	20986	20986	-3464	7292	AN11291
32	CONTIG16	24243	24657	3.53	0.00E+00	transcription_start_site	-	20869	20869	-3581	7293	AN11291
379	CONTIG16	22068	22477	2.63	0.00E+00	transcription_start_site	-	20986	20986	-1286	7292	AN11291
379	CONTIG16	22068	22477	2.63	0.00E+00	transcription_start_site	-	20869	20869	-1403	7293	AN11291
415	CONTIG16	179121	179455	2.58	0.00E+00	transcription_start_site	-	175282	175282	-4006	7301	AN11294

415	CONTIG16	179121	179455	2.58	0.00E+00	transcription_start_site	-	175194	175194	-4094	7302 AN11294
711	CONTIG16	193816	194160	2.19	8.81E-03	transcription_start_site	-	194590	194590	602	7304 AN11295
711	CONTIG16	193816	194160	2.19	8.81E-03	transcription_start_site	-	194728	194728	740	7303 AN11295
1731	CONTIG16	257719	258133	1.49	9.24E-02	transcription_start_site	+	262554	262554	-4628	7316 AN1130
1731	CONTIG16	257719	258133	1.49	9.24E-02	transcription_start_site	+	263055	263055	-5129	7317 AN1130
283	CONTIG22	40145	40644	2.79	0.00E+00	transcription_start_site	+	42564	42564	-2169	7323 AN11301
283	CONTIG22	40145	40644	2.79	0.00E+00	transcription_start_site	+	43022	43022	-2627	7324 AN11301
1061	CONTIG22	42835	43123	1.86	2.62E-02	transcription_start_site	+	43022	43022	-43	7324 AN11301
1061	CONTIG22	42835	43123	1.86	2.62E-02	transcription_start_site	+	42564	42564	415	7323 AN11301
1168	CONTIG25	8866	9125	1.78	6.61E-03	transcription_start_site	-	4199	4199	-4796	7335 AN11305
1168	CONTIG25	8866	9125	1.78	6.61E-03	transcription_start_site	-	4100	4100	-4895	7336 AN11305
1168	CONTIG25	8866	9125	1.78	6.61E-03	transcription_start_site	-	3905	3905	-5090	7337 AN11305
2825	CONTIG25	35180	35619	1.05	1.44E-01	transcription_start_site	+	37210	37210	-1810	7338 AN11306
2825	CONTIG25	35180	35619	1.05	1.44E-01	transcription_start_site	+	37289	37289	-1889	7339 AN11306
2827	CONTIG25	106744	107153	1.05	1.44E-01	transcription_start_site	-	106744	106744	-204	7340 AN11307
2827	CONTIG25	106744	107153	1.05	1.44E-01	transcription_start_site	-	106665	106665	-283	7341 AN11307
2601	CONTIG25	137779	139263	1.16	7.51E-02	transcription_start_site	-	135300	135300	-3221	7342 AN11308
2601	CONTIG25	137779	139263	1.16	7.51E-02	transcription_start_site	-	135029	135029	-3492	7343 AN11308
2828	CONTIG25	137043	137393	1.05	1.44E-01	transcription_start_site	-	135300	135300	-1918	7342 AN11308
2828	CONTIG25	137043	137393	1.05	1.44E-01	transcription_start_site	-	135029	135029	-2189	7343 AN11308
547	CONTIG26	181506	182014	2.39	3.61E-03	transcription_start_site	-	178398	178398	-3362	7352 AN11311
547	CONTIG26	181506	182014	2.39	3.61E-03	transcription_start_site	-	178017	178017	-3743	7353 AN11311
2012	CONTIG26	287188	288517	1.37	1.07E-01	transcription_start_site	+	290987	290987	-3134	7354 AN11312
2012	CONTIG26	287188	288517	1.37	1.07E-01	transcription_start_site	+	291216	291216	-3363	7355 AN11312
2012	CONTIG26	287188	288517	1.37	1.07E-01	transcription_start_site	+	291344	291344	-3491	7356 AN11312
2301	CONTIG26	286218	286567	1.27	1.66E-01	transcription_start_site	+	290987	290987	-4594	7354 AN11312
2301	CONTIG26	286218	286567	1.27	1.66E-01	transcription_start_site	+	291216	291216	-4823	7355 AN11312
2301	CONTIG26	286218	286567	1.27	1.66E-01	transcription_start_site	+	291344	291344	-4951	7356 AN11312
2318	CONTIG27	40587	40944	1.26	6.84E-02	transcription_start_site	+	45087	45087	-4321	7357 AN11313
2318	CONTIG27	40587	40944	1.26	6.84E-02	transcription_start_site	+	45525	45525	-4759	7358 AN11313
2432	CONTIG27	42842	44089	1.22	5.52E-02	transcription_start_site	+	45087	45087	-1621	7357 AN11313
2432	CONTIG27	42842	44089	1.22	5.52E-02	transcription_start_site	+	45525	45525	-2059	7358 AN11313
2948	CONTIG28	24613	25252	0.97	1.28E-01	transcription_start_site	+	24786	24786	146	7361 AN11314
2948	CONTIG28	24613	25252	0.97	1.28E-01	transcription_start_site	+	24549	24549	383	7360 AN11314
2948	CONTIG28	24613	25252	0.97	1.28E-01	transcription_start_site	+	24404	24404	528	7359 AN11314
515	CONTIG29	113118	113470	2.43	3.61E-03	transcription_start_site	+	114345	114345	-1051	7365 AN11316
515	CONTIG29	113118	113470	2.43	3.61E-03	transcription_start_site	+	114546	114546	-1252	7366 AN11316
515	CONTIG29	113118	113470	2.43	3.61E-03	transcription_start_site	+	114737	114737	-1443	7367 AN11316
1045	CONTIG29	257637	258064	1.88	1.44E-02	transcription_start_site	-	252834	252834	-5016	7375 AN11319
1045	CONTIG29	257637	258064	1.88	1.44E-02	transcription_start_site	-	252654	252654	-5196	7376 AN11319
444	CONTIG16	274430	274794	2.53	2.22E-03	transcription_start_site	-	271508	271508	-3104	7378 AN1132
205	CONTIG30	60076	60378	2.93	0.00E+00	transcription_start_site	+	60730	60730	-503	7381 AN11321
205	CONTIG30	60076	60378	2.93	0.00E+00	transcription_start_site	+	60836	60836	-609	7382 AN11321
205	CONTIG30	60076	60378	2.93	0.00E+00	transcription_start_site	+	61014	61014	-787	7383 AN11321
205	CONTIG30	60076	60378	2.93	0.00E+00	transcription_start_site	+	61241	61241	-1014	7384 AN11321
510	CONTIG32	122258	122602	2.43	2.22E-03	transcription_start_site	-	121332	121332	-1098	7389 AN11323
510	CONTIG32	122258	122602	2.43	2.22E-03	transcription_start_site	-	121215	121215	-1215	7390 AN11323
484	CONTIG32	209046	210430	2.47	1.80E-03	transcription_start_site	-	210485	210485	747	7393 AN11324
484	CONTIG32	209046	210430	2.47	1.80E-03	transcription_start_site	-	210742	210742	1004	7392 AN11324
484	CONTIG32	209046	210430	2.47	1.80E-03	transcription_start_site	-	211007	211007	1269	7391 AN11324
192	CONTIG32	270096	270650	2.95	0.00E+00	transcription_start_site	-	269346	269346	-1027	7394 AN11325
192	CONTIG32	270096	270650	2.95	0.00E+00	transcription_start_site	-	269261	269261	-1112	7395 AN11325
305	CONTIG32	269045	269320	2.76	5.64E-04	transcription_start_site	-	269261	269261	78	7395 AN11325
305	CONTIG32	269045	269320	2.76	5.64E-04	transcription_start_site	-	269346	269346	163	7394 AN11325
621	CONTIG32	431638	431977	2.28	1.22E-03	transcription_start_site	-	429012	429012	-2795	7400 AN11328
621	CONTIG32	431638	431977	2.28	1.22E-03	transcription_start_site	-	428861	428861	-2946	7401 AN11328
444	CONTIG16	274430	274794	2.53	2.22E-03	transcription_start_site	+	273602	273602	1010	7406 AN1133

444	CONTIG16	274430	274794	2.53	2.22E-03	transcription_start_site	+	273472	273472	1140	7405 AN1133
1324	CONTIG38	194491	194755	1.67	8.81E-03	transcription_start_site	-	190795	190795	-3828	7416 AN11333
1324	CONTIG38	194491	194755	1.67	8.81E-03	transcription_start_site	-	190512	190512	-4111	7417 AN11333
2422	CONTIG39	137556	137830	1.23	1.93E-01	transcription_start_site	-	134394	134394	-3299	7422 AN11336
2422	CONTIG39	137556	137830	1.23	1.93E-01	transcription_start_site	-	134186	134186	-3507	7423 AN11336
1407	CONTIG39	177078	177442	1.63	5.88E-02	transcription_start_site	-	177067	177067	-193	7424 AN11337
1407	CONTIG39	177078	177442	1.63	5.88E-02	transcription_start_site	-	176957	176957	-303	7425 AN11337
444	CONTIG16	274430	274794	2.53	2.22E-03	transcription_start_site	+	275399	275399	-787	7431 AN1134
735	CONTIG40	133741	134005	2.17	1.57E-02	transcription_start_site	+	135754	135754	-1881	7436 AN11342
735	CONTIG40	133741	134005	2.17	1.57E-02	transcription_start_site	+	135791	135791	-1918	7437 AN11342
735	CONTIG40	133741	134005	2.17	1.57E-02	transcription_start_site	+	136158	136158	-2285	7438 AN11342
735	CONTIG40	133741	134005	2.17	1.57E-02	transcription_start_site	+	136244	136244	-2371	7439 AN11342
295	CONTIG42	28803	29092	2.77	1.54E-03	transcription_start_site	-	27553	27553	-1394	7446 AN11345
295	CONTIG42	28803	29092	2.77	1.54E-03	transcription_start_site	-	27510	27510	-1437	7447 AN11345
295	CONTIG42	28803	29092	2.77	1.54E-03	transcription_start_site	-	28808	28808	-139	7448 AN11346
295	CONTIG42	28803	29092	2.77	1.54E-03	transcription_start_site	-	28224	28224	-723	7449 AN11346
2004	CONTIG43	193426	193705	1.37	6.84E-02	transcription_start_site	-	194167	194167	601	7461 AN11350
2004	CONTIG43	193426	193705	1.37	6.84E-02	transcription_start_site	-	194251	194251	685	7460 AN11350
2838	CONTIG44	3096	3425	1.04	4.36E-02	transcription_start_site	+	6024	6024	-2763	7467 AN11353
2838	CONTIG44	3096	3425	1.04	4.36E-02	transcription_start_site	+	6158	6158	-2897	7468 AN11353
3044	CONTIG45	51164	51428	0.78	1.66E-01	transcription_start_site	-	49454	49454	-1842	7471 AN11355
3044	CONTIG45	51164	51428	0.78	1.66E-01	transcription_start_site	-	49374	49374	-1922	7472 AN11355
3044	CONTIG45	51164	51428	0.78	1.66E-01	transcription_start_site	-	49308	49308	-1988	7473 AN11355
3044	CONTIG45	51164	51428	0.78	1.66E-01	transcription_start_site	-	49208	49208	-2088	7474 AN11355
3044	CONTIG45	51164	51428	0.78	1.66E-01	transcription_start_site	-	49094	49094	-2202	7475 AN11355
3044	CONTIG45	51164	51428	0.78	1.66E-01	transcription_start_site	-	48907	48907	-2389	7476 AN11355
444	CONTIG16	274430	274794	2.53	2.22E-03	transcription_start_site	+	279106	279106	-4494	7489 AN1136
444	CONTIG16	274430	274794	2.53	2.22E-03	transcription_start_site	+	279332	279332	-4720	7490 AN1136
444	CONTIG16	274430	274794	2.53	2.22E-03	transcription_start_site	+	279523	279523	-4911	7491 AN1136
2780	CONTIG49	70518	70927	1.08	1.44E-01	transcription_start_site	-	71420	71420	697	7499 AN11362
2780	CONTIG49	70518	70927	1.08	1.44E-01	transcription_start_site	-	71614	71614	891	7498 AN11362
1481	CONTIG50	18152	18431	1.6	8.01E-02	transcription_start_site	-	14790	14790	-3501	7512 AN11367
1481	CONTIG50	18152	18431	1.6	8.01E-02	transcription_start_site	-	14649	14649	-3642	7513 AN11367
107	CONTIG50	34138	34487	3.2	2.67E-04	transcription_start_site	-	30852	30852	-3460	7514 AN11368
107	CONTIG50	34138	34487	3.2	2.67E-04	transcription_start_site	-	30220	30220	-4092	7515 AN11368
1207	CONTIG50	30838	31102	1.76	5.22E-02	transcription_start_site	-	30852	30852	-118	7514 AN11368
1207	CONTIG50	30838	31102	1.76	5.22E-02	transcription_start_site	-	30220	30220	-750	7515 AN11368
179	CONTIG51	275571	275843	2.99	2.39E-04	transcription_start_site	+	280645	280645	-4938	7528 AN11373
179	CONTIG51	275571	275843	2.99	2.39E-04	transcription_start_site	+	280815	280815	-5108	7529 AN11373
790	CONTIG51	276770	277259	2.11	4.81E-03	transcription_start_site	+	280645	280645	-3630	7528 AN11373
790	CONTIG51	276770	277259	2.11	4.81E-03	transcription_start_site	+	280815	280815	-3800	7529 AN11373
790	CONTIG51	276770	277259	2.11	4.81E-03	transcription_start_site	+	280986	280986	-3971	7530 AN11373
1260	CONTIG51	1004630	1005589	1.72	4.36E-02	transcription_start_site	+	1007731	1007731	-2621	7535 AN11376
830	CONTIG53	47851	49045	2.07	1.06E-02	transcription_start_site	-	43874	43874	-4574	7539 AN11378
830	CONTIG53	47851	49045	2.07	1.06E-02	transcription_start_site	-	43751	43751	-4697	7540 AN11378
830	CONTIG53	47851	49045	2.07	1.06E-02	transcription_start_site	-	43543	43543	-4905	7541 AN11378
1497	CONTIG53	44851	45740	1.59	7.25E-02	transcription_start_site	-	43874	43874	-1421	7539 AN11378
1497	CONTIG53	44851	45740	1.59	7.25E-02	transcription_start_site	-	43751	43751	-1544	7540 AN11378
1497	CONTIG53	44851	45740	1.59	7.25E-02	transcription_start_site	-	43543	43543	-1752	7541 AN11378
1499	CONTIG53	44251	44535	1.59	9.24E-02	transcription_start_site	-	43874	43874	-519	7539 AN11378
1499	CONTIG53	44251	44535	1.59	9.24E-02	transcription_start_site	-	43751	43751	-642	7540 AN11378
1499	CONTIG53	44251	44535	1.59	9.24E-02	transcription_start_site	-	43543	43543	-850	7541 AN11378
2941	CONTIG55	137327	137616	0.98	1.66E-01	transcription_start_site	-	138547	138547	1075	7559 AN11384
2941	CONTIG55	137327	137616	0.98	1.66E-01	transcription_start_site	+	140545	140545	-3073	7560 AN11385
2939	CONTIG55	189546	190475	0.98	1.50E-01	transcription_start_site	+	194928	194928	-4917	7568 AN11388
2939	CONTIG55	189546	190475	0.98	1.50E-01	transcription_start_site	+	195118	195118	-5107	7569 AN11388
2939	CONTIG55	189546	190475	0.98	1.50E-01	transcription_start_site	+	195383	195383	-5372	7570 AN11388

2656	CONTIG55	204690	205051	1.14	9.24E-02	transcription_start_site	-	200676	200676	-4194	7571	AN11389
2656	CONTIG55	204690	205051	1.14	9.24E-02	transcription_start_site	-	200479	200479	-4391	7572	AN11389
2656	CONTIG55	204690	205051	1.14	9.24E-02	transcription_start_site	-	199980	199980	-4890	7573	AN11389
864	CONTIG16	288844	289108	2.04	1.57E-02	transcription_start_site	-	284878	284878	-4098	7574	AN1139
535	CONTIG55	291845	292265	2.39	0.00E+00	transcription_start_site	+	296900	296900	-4845	7577	AN11391
535	CONTIG55	291845	292265	2.39	0.00E+00	transcription_start_site	+	296955	296955	-4900	7578	AN11391
1820	CONTIG55	357538	357887	1.44	2.62E-02	transcription_start_site	-	354989	354989	-2723	7579	AN11392
1820	CONTIG55	357538	357887	1.44	2.62E-02	transcription_start_site	-	354725	354725	-2987	7580	AN11392
2772	CONTIG59	170478	170829	1.08	8.01E-02	transcription_start_site	-	171096	171096	442	7592	AN11398
2772	CONTIG59	170478	170829	1.08	8.01E-02	transcription_start_site	-	171442	171442	788	7591	AN11398
603	CONTIG61	98853	99150	2.3	2.22E-03	transcription_start_site	-	94176	94176	-4825	7610	AN11404
603	CONTIG61	98853	99150	2.3	2.22E-03	transcription_start_site	-	94084	94084	-4917	7611	AN11404
603	CONTIG61	98853	99150	2.3	2.22E-03	transcription_start_site	-	93997	93997	-5004	7612	AN11404
1330	CONTIG61	438232	438594	1.67	3.08E-02	transcription_start_site	+	442639	442639	-4226	7623	AN11409
1330	CONTIG61	438232	438594	1.67	3.08E-02	transcription_start_site	+	442906	442906	-4493	7624	AN11409
1330	CONTIG61	438232	438594	1.67	3.08E-02	transcription_start_site	+	443459	443459	-5046	7625	AN11410
2446	CONTIG61	512719	512978	1.22	1.44E-01	transcription_start_site	-	510375	510375	-2473	7629	AN11411
2446	CONTIG61	512719	512978	1.22	1.44E-01	transcription_start_site	-	510246	510246	-2602	7630	AN11411
2446	CONTIG61	512719	512978	1.22	1.44E-01	transcription_start_site	-	510079	510079	-2769	7631	AN11411
2446	CONTIG61	512719	512978	1.22	1.44E-01	transcription_start_site	-	509966	509966	-2882	7632	AN11411
2446	CONTIG61	512719	512978	1.22	1.44E-01	transcription_start_site	-	509772	509772	-3076	7633	AN11411
365	CONTIG61	567152	567450	2.66	6.10E-04	transcription_start_site	-	567638	567638	337	7635	AN11412
365	CONTIG61	567152	567450	2.66	6.10E-04	transcription_start_site	-	567797	567797	496	7634	AN11412
1075	CONTIG61	732079	732353	1.85	1.57E-02	transcription_start_site	-	728856	728856	-3360	7639	AN11414
1075	CONTIG61	732079	732353	1.85	1.57E-02	transcription_start_site	-	728658	728658	-3558	7640	AN11414
1075	CONTIG61	732079	732353	1.85	1.57E-02	transcription_start_site	-	733204	733204	988	7642	AN11415
1069	CONTIG61	758032	758676	1.85	3.83E-03	transcription_start_site	+	757612	757612	742	7645	AN11416
1069	CONTIG61	758032	758676	1.85	3.83E-03	transcription_start_site	+	757416	757416	938	7644	AN11416
1069	CONTIG61	758032	758676	1.85	3.83E-03	transcription_start_site	+	757219	757219	1135	7643	AN11416
2447	CONTIG61	756467	756811	1.22	1.44E-01	transcription_start_site	+	757219	757219	-580	7643	AN11416
2447	CONTIG61	756467	756811	1.22	1.44E-01	transcription_start_site	+	757416	757416	-777	7644	AN11416
2447	CONTIG61	756467	756811	1.22	1.44E-01	transcription_start_site	+	757612	757612	-973	7645	AN11416
2441	CONTIG61	785476	785900	1.22	1.28E-01	transcription_start_site	-	785810	785810	122	7647	AN11417
2441	CONTIG61	785476	785900	1.22	1.28E-01	transcription_start_site	-	785901	785901	213	7646	AN11417
1068	CONTIG62	31958	32532	1.85	3.61E-03	transcription_start_site	+	35466	35466	-3221	7648	AN11418
1068	CONTIG62	31958	32532	1.85	3.61E-03	transcription_start_site	+	35519	35519	-3274	7649	AN11418
2943	CONTIG62	36226	36575	0.98	1.66E-01	transcription_start_site	+	35519	35519	881	7649	AN11418
2943	CONTIG62	36226	36575	0.98	1.66E-01	transcription_start_site	+	35466	35466	934	7648	AN11418
2464	CONTIG62	105376	106100	1.21	4.42E-02	transcription_start_site	-	105336	105336	-402	7650	AN11419
2464	CONTIG62	105376	106100	1.21	4.42E-02	transcription_start_site	-	105123	105123	-615	7651	AN11419
2464	CONTIG62	105376	106100	1.21	4.42E-02	transcription_start_site	-	104860	104860	-878	7652	AN11419
2207	CONTIG64	121213	121712	1.29	1.86E-02	transcription_start_site	+	120989	120989	473	7657	AN11420
2207	CONTIG64	121213	121712	1.29	1.86E-02	transcription_start_site	+	120832	120832	630	7656	AN11420
2958	CONTIG64	117537	117826	0.96	1.44E-01	transcription_start_site	+	120832	120832	-3150	7656	AN11420
2958	CONTIG64	117537	117826	0.96	1.44E-01	transcription_start_site	+	120989	120989	-3307	7657	AN11420
2703	CONTIG65	106139	106403	1.12	8.01E-02	transcription_start_site	-	103412	103412	-2859	7660	AN11422
2703	CONTIG65	106139	106403	1.12	8.01E-02	transcription_start_site	-	103162	103162	-3109	7661	AN11422
1490	CONTIG67	103068	103492	1.59	2.18E-02	transcription_start_site	-	98784	98784	-4496	7674	AN11427
1490	CONTIG67	103068	103492	1.59	2.18E-02	transcription_start_site	-	98515	98515	-4765	7675	AN11427
1490	CONTIG67	103068	103492	1.59	2.18E-02	transcription_start_site	-	98227	98227	-5053	7676	AN11427
1491	CONTIG67	112503	112852	1.59	3.70E-02	transcription_start_site	+	114508	114508	-1830	7680	AN11428
1491	CONTIG67	112503	112852	1.59	3.70E-02	transcription_start_site	+	114702	114702	-2024	7681	AN11428
1491	CONTIG67	112503	112852	1.59	3.70E-02	transcription_start_site	+	115566	115566	-2888	7682	AN11428
1491	CONTIG67	112503	112852	1.59	3.70E-02	transcription_start_site	+	115671	115671	-2993	7683	AN11428
1491	CONTIG67	112503	112852	1.59	3.70E-02	transcription_start_site	+	115910	115910	-3232	7684	AN11428
399	CONTIG68	96905	97267	2.6	2.39E-04	transcription_start_site	-	93780	93780	-3306	7690	AN11431
399	CONTIG68	96905	97267	2.6	2.39E-04	transcription_start_site	-	93525	93525	-3561	7691	AN11431

399	CONTIG68	96905	97267	2.6	2.39E-04	transcription_start_site	-	93430	93430	-3656	7692	AN11431
2735	CONTIG68	92933	93209	1.11	1.66E-01	transcription_start_site	-	93430	93430	359	7692	AN11431
2735	CONTIG68	92933	93209	1.11	1.66E-01	transcription_start_site	-	93525	93525	454	7691	AN11431
2735	CONTIG68	92933	93209	1.11	1.66E-01	transcription_start_site	-	93780	93780	709	7690	AN11431
2129	CONTIG69	23645	24144	1.32	5.05E-02	transcription_start_site	-	23122	23122	-772	7693	AN11432
2129	CONTIG69	23645	24144	1.32	5.05E-02	transcription_start_site	-	22931	22931	-963	7694	AN11432
2510	CONTIG69	25295	26169	1.2	9.26E-02	transcription_start_site	-	23122	23122	-2610	7693	AN11432
2510	CONTIG69	25295	26169	1.2	9.26E-02	transcription_start_site	-	22931	22931	-2801	7694	AN11432
1261	CONTIG75	143865	144129	1.72	4.36E-02	transcription_start_site	-	139797	139797	-4200	7702	AN11435
1261	CONTIG75	143865	144129	1.72	4.36E-02	transcription_start_site	-	139328	139328	-4669	7703	AN11435
1261	CONTIG75	143865	144129	1.72	4.36E-02	transcription_start_site	-	139131	139131	-4866	7704	AN11435
1402	CONTIG75	139580	139929	1.63	3.99E-02	transcription_start_site	-	139797	139797	42	7702	AN11435
1402	CONTIG75	139580	139929	1.63	3.99E-02	transcription_start_site	-	139328	139328	-426	7703	AN11435
1402	CONTIG75	139580	139929	1.63	3.99E-02	transcription_start_site	-	139131	139131	-623	7704	AN11435
1011	CONTIG76	136208	136643	1.9	1.57E-02	transcription_start_site	-	135178	135178	-1247	7706	AN11437
1011	CONTIG76	136208	136643	1.9	1.57E-02	transcription_start_site	-	135122	135122	-1303	7707	AN11437
1011	CONTIG76	136208	136643	1.9	1.57E-02	transcription_start_site	-	135034	135034	-1391	7708	AN11437
1011	CONTIG76	136208	136643	1.9	1.57E-02	transcription_start_site	-	135003	135003	-1422	7709	AN11437
1072	CONTIG76	134185	135442	1.85	1.00E-02	transcription_start_site	-	135003	135003	189	7709	AN11437
1072	CONTIG76	134185	135442	1.85	1.00E-02	transcription_start_site	-	135034	135034	220	7708	AN11437
1072	CONTIG76	134185	135442	1.85	1.00E-02	transcription_start_site	-	135122	135122	308	7707	AN11437
1072	CONTIG76	134185	135442	1.85	1.00E-02	transcription_start_site	-	135178	135178	364	7706	AN11437
1952	CONTIG76	135463	136117	1.39	9.24E-02	transcription_start_site	-	135178	135178	-612	7706	AN11437
1952	CONTIG76	135463	136117	1.39	9.24E-02	transcription_start_site	-	135122	135122	-668	7707	AN11437
1952	CONTIG76	135463	136117	1.39	9.24E-02	transcription_start_site	-	135034	135034	-756	7708	AN11437
1952	CONTIG76	135463	136117	1.39	9.24E-02	transcription_start_site	-	135003	135003	-787	7709	AN11437
2084	CONTIG76	137859	138138	1.34	1.08E-01	transcription_start_site	-	135178	135178	-2820	7706	AN11437
2084	CONTIG76	137859	138138	1.34	1.08E-01	transcription_start_site	-	135122	135122	-2876	7707	AN11437
2084	CONTIG76	137859	138138	1.34	1.08E-01	transcription_start_site	-	135034	135034	-2964	7708	AN11437
2084	CONTIG76	137859	138138	1.34	1.08E-01	transcription_start_site	-	135003	135003	-2995	7709	AN11437
1825	CONTIG77	35402	35704	1.44	4.36E-02	transcription_start_site	+	35391	35391	162	7711	AN11438
1825	CONTIG77	35402	35704	1.44	4.36E-02	transcription_start_site	+	35335	35335	218	7710	AN11438
2718	CONTIG78	91953	92237	1.12	1.93E-01	transcription_start_site	-	88710	88710	-3385	7712	AN11439
2625	CONTIG78	138996	139353	1.16	1.66E-01	transcription_start_site	-	138687	138687	-487	7721	AN11440
2625	CONTIG78	138996	139353	1.16	1.66E-01	transcription_start_site	-	138360	138360	-814	7722	AN11440
1756	CONTIG78	343893	344302	1.48	5.88E-02	transcription_start_site	-	342561	342561	-1536	7727	AN11443
1756	CONTIG78	343893	344302	1.48	5.88E-02	transcription_start_site	-	342386	342386	-1711	7728	AN11443
523	CONTIG79	86626	86920	2.41	5.64E-04	transcription_start_site	-	86758	86758	-15	7733	AN11445
523	CONTIG79	86626	86920	2.41	5.64E-04	transcription_start_site	-	86607	86607	-166	7734	AN11445
1105	CONTIG79	87156	87735	1.83	8.81E-03	transcription_start_site	-	86758	86758	-687	7733	AN11445
1105	CONTIG79	87156	87735	1.83	8.81E-03	transcription_start_site	-	86607	86607	-838	7734	AN11445
226	CONTIG80	32939	33509	2.89	0.00E+00	transcription_start_site	+	35483	35483	-2259	7735	AN11446
226	CONTIG80	32939	33509	2.89	0.00E+00	transcription_start_site	+	35594	35594	-2370	7736	AN11446
226	CONTIG80	32939	33509	2.89	0.00E+00	transcription_start_site	+	35873	35873	-2649	7737	AN11446
1126	CONTIG80	35402	35826	1.81	2.12E-02	transcription_start_site	+	35594	35594	20	7736	AN11446
1126	CONTIG80	35402	35826	1.81	2.12E-02	transcription_start_site	+	35483	35483	131	7735	AN11446
1126	CONTIG80	35402	35826	1.81	2.12E-02	transcription_start_site	+	35873	35873	-259	7737	AN11446
2406	CONTIG81	135976	136330	1.23	6.16E-02	transcription_start_site	-	131300	131300	-4853	7738	AN11447
2406	CONTIG81	135976	136330	1.23	6.16E-02	transcription_start_site	-	131225	131225	-4928	7739	AN11447
3030	CONTIG82	30153	30662	0.85	1.08E-01	transcription_start_site	+	31212	31212	-804	7740	AN11448
3030	CONTIG82	30153	30662	0.85	1.08E-01	transcription_start_site	+	31313	31313	-905	7741	AN11448
466	CONTIG16	307064	307493	2.48	0.00E+00	transcription_start_site	-	302632	302632	-4646	7744	AN1145
466	CONTIG16	307064	307493	2.48	0.00E+00	transcription_start_site	-	302398	302398	-4880	7745	AN1145
1962	CONTIG84	238217	238626	1.39	1.44E-01	transcription_start_site	+	239667	239667	-1245	7751	AN11451
1962	CONTIG84	238217	238626	1.39	1.44E-01	transcription_start_site	+	239927	239927	-1505	7752	AN11451
729	CONTIG84	249231	249880	2.17	4.13E-03	transcription_start_site	-	250159	250159	603	7754	AN11452
729	CONTIG84	249231	249880	2.17	4.13E-03	transcription_start_site	-	250622	250622	1066	7753	AN11452

1707	CONTIG84	447762	448101	1.5	1.08E-01	transcription_start_site	+	448728	448728	-796	7757 AN11454
1707	CONTIG84	447762	448101	1.5	1.08E-01	transcription_start_site	+	448840	448840	-908	7758 AN11454
1076	CONTIG85	10956	11243	1.85	1.84E-02	transcription_start_site	-	10816	10816	-283	7761 AN11456
1076	CONTIG85	10956	11243	1.85	1.84E-02	transcription_start_site	-	10705	10705	-394	7762 AN11456
267	CONTIG86	66230	66890	2.81	2.22E-03	transcription_start_site	-	66505	66505	-55	7778 AN11459
161	CONTIG86	89252	89766	3.03	1.01E-03	transcription_start_site	-	88421	88421	-1088	7779 AN11460
161	CONTIG86	89252	89766	3.03	1.01E-03	transcription_start_site	-	88265	88265	-1244	7780 AN11460
756	CONTIG86	96396	96808	2.15	1.06E-02	transcription_start_site	+	99590	99590	-2988	7781 AN11461
756	CONTIG86	96396	96808	2.15	1.06E-02	transcription_start_site	+	99806	99806	-3204	7782 AN11461
1136	CONTIG88	159019	159293	1.8	7.85E-03	transcription_start_site	+	158481	158481	675	7796 AN11465
1136	CONTIG88	159019	159293	1.8	7.85E-03	transcription_start_site	+	158405	158405	751	7795 AN11465
2043	CONTIG89	135765	136189	1.36	9.24E-02	transcription_start_site	+	137035	137035	-1058	7803 AN11469
2043	CONTIG89	135765	136189	1.36	9.24E-02	transcription_start_site	+	137193	137193	-1216	7804 AN11469
2043	CONTIG89	135765	136189	1.36	9.24E-02	transcription_start_site	+	137292	137292	-1315	7805 AN11469
445	CONTIG16	313742	314076	2.53	2.22E-03	transcription_start_site	-	308783	308783	-5126	7806 AN1147
1598	CONTIG89	200256	200530	1.54	5.22E-02	transcription_start_site	-	196868	196868	-3525	7807 AN11470
1598	CONTIG89	200256	200530	1.54	5.22E-02	transcription_start_site	-	196763	196763	-3630	7808 AN11470
24	CONTIG89	224567	225366	3.57	0.00E+00	transcription_start_site	+	228691	228691	-3724	7809 AN11471
24	CONTIG89	224567	225366	3.57	0.00E+00	transcription_start_site	+	228835	228835	-3868	7810 AN11471
24	CONTIG89	224567	225366	3.57	0.00E+00	transcription_start_site	+	228924	228924	-3957	7811 AN11471
24	CONTIG89	224567	225366	3.57	0.00E+00	transcription_start_site	+	229053	229053	-4086	7812 AN11471
765	CONTIG89	227552	227901	2.13	2.32E-03	transcription_start_site	+	228691	228691	-964	7809 AN11471
765	CONTIG89	227552	227901	2.13	2.32E-03	transcription_start_site	+	228835	228835	-1108	7810 AN11471
765	CONTIG89	227552	227901	2.13	2.32E-03	transcription_start_site	+	228924	228924	-1197	7811 AN11471
765	CONTIG89	227552	227901	2.13	2.32E-03	transcription_start_site	+	229053	229053	-1326	7812 AN11471
1125	CONTIG89	268585	269387	1.81	1.84E-02	transcription_start_site	-	264398	264398	-4588	7813 AN11472
1125	CONTIG89	268585	269387	1.81	1.84E-02	transcription_start_site	-	264128	264128	-4858	7814 AN11472
1291	CONTIG93	140859	141358	1.7	2.18E-02	transcription_start_site	+	143758	143758	-2649	7822 AN11475
1291	CONTIG93	140859	141358	1.7	2.18E-02	transcription_start_site	+	144360	144360	-3251	7823 AN11475
1551	CONTIG93	142964	144063	1.56	3.99E-02	transcription_start_site	+	143758	143758	-244	7822 AN11475
1551	CONTIG93	142964	144063	1.56	3.99E-02	transcription_start_site	+	144360	144360	-846	7823 AN11475
407	CONTIG93	272560	273214	2.59	0.00E+00	transcription_start_site	-	273475	273475	588	7826 AN11476
407	CONTIG93	272560	273214	2.59	0.00E+00	transcription_start_site	-	273594	273594	707	7825 AN11476
407	CONTIG93	272560	273214	2.59	0.00E+00	transcription_start_site	-	273731	273731	844	7824 AN11476
2572	CONTIG93	278410	278981	1.18	1.93E-01	transcription_start_site	-	273731	273731	-4964	7824 AN11476
2572	CONTIG93	278410	278981	1.18	1.93E-01	transcription_start_site	-	273594	273594	-5101	7825 AN11476
2572	CONTIG93	278410	278981	1.18	1.93E-01	transcription_start_site	-	273475	273475	-5220	7826 AN11476
498	CONTIG94	14329	14603	2.45	2.39E-04	transcription_start_site	-	14115	14115	-351	7827 AN11477
498	CONTIG94	14329	14603	2.45	2.39E-04	transcription_start_site	-	13952	13952	-514	7828 AN11477
445	CONTIG16	313742	314076	2.53	2.22E-03	transcription_start_site	-	313503	313503	-406	7833 AN1148
445	CONTIG16	313742	314076	2.53	2.22E-03	transcription_start_site	-	313281	313281	-628	7834 AN1148
1837	CONTIG16	316292	316701	1.44	1.08E-01	transcription_start_site	-	313503	313503	-2993	7833 AN1148
1837	CONTIG16	316292	316701	1.44	1.08E-01	transcription_start_site	-	313281	313281	-3215	7834 AN1148
2239	CONTIG16	314867	315809	1.29	1.66E-01	transcription_start_site	-	313503	313503	-1835	7833 AN1148
2239	CONTIG16	314867	315809	1.29	1.66E-01	transcription_start_site	-	313281	313281	-2057	7834 AN1148
330	CONTIG96	36621	37030	2.72	2.22E-03	transcription_start_site	+	37966	37966	-1140	7842 AN11482
330	CONTIG96	36621	37030	2.72	2.22E-03	transcription_start_site	+	38222	38222	-1396	7843 AN11482
768	CONTIG96	32255	33439	2.13	1.84E-02	transcription_start_site	+	37966	37966	-5119	7842 AN11482
768	CONTIG96	32255	33439	2.13	1.84E-02	transcription_start_site	+	38222	38222	-5375	7843 AN11482
358	CONTIG96	42313	42657	2.67	3.03E-03	transcription_start_site	+	42370	42370	115	7845 AN11483
358	CONTIG96	42313	42657	2.67	3.03E-03	transcription_start_site	+	42129	42129	356	7844 AN11483
820	CONTIG96	39906	40250	2.08	2.12E-02	transcription_start_site	+	42129	42129	-2051	7844 AN11483
820	CONTIG96	39906	40250	2.08	2.12E-02	transcription_start_site	+	42370	42370	-2292	7845 AN11483
1277	CONTIG96	41271	42284	1.71	3.41E-02	transcription_start_site	+	42129	42129	-351	7844 AN11483
1277	CONTIG96	41271	42284	1.71	3.41E-02	transcription_start_site	+	42370	42370	-592	7845 AN11483
1485	CONTIG96	43221	43475	1.6	9.24E-02	transcription_start_site	+	42370	42370	978	7845 AN11483
527	CONTIG97	9691	10495	2.41	1.22E-03	transcription_start_site	-	10596	10596	503	7851 AN11485

527	CONTIG97	9691	10495	2.41	1.22E-03	transcription_start_site	-	10910	10910	817	7850	AN11485
527	CONTIG97	9691	10495	2.41	1.22E-03	transcription_start_site	-	11019	11019	926	7849	AN11485
2151	CONTIG98	104552	104977	1.32	1.44E-01	transcription_start_site	+	109534	109534	-4769	7855	AN11487
2151	CONTIG98	104552	104977	1.32	1.44E-01	transcription_start_site	+	109729	109729	-4964	7856	AN11487
2151	CONTIG98	104552	104977	1.32	1.44E-01	transcription_start_site	+	109923	109923	-5158	7857	AN11487
1276	CONTIG98	167709	168888	1.71	2.64E-02	transcription_start_site	-	169211	169211	912	7863	AN11488
1276	CONTIG98	167709	168888	1.71	2.64E-02	transcription_start_site	-	169365	169365	1066	7862	AN11488
1276	CONTIG98	167709	168888	1.71	2.64E-02	transcription_start_site	-	169482	169482	1183	7861	AN11488
1276	CONTIG98	167709	168888	1.71	2.64E-02	transcription_start_site	-	169590	169590	1291	7860	AN11488
1276	CONTIG98	167709	168888	1.71	2.64E-02	transcription_start_site	-	169781	169781	1482	7859	AN11488
1837	CONTIG16	316292	316701	1.44	1.08E-01	transcription_start_site	-	315373	315373	-1123	7866	AN1149
2239	CONTIG16	314867	315809	1.29	1.66E-01	transcription_start_site	-	315373	315373	35	7866	AN1149
439	CONTIG100	119718	120140	2.53	0.00E+00	transcription_start_site	-	119828	119828	-101	7872	AN11491
439	CONTIG100	119718	120140	2.53	0.00E+00	transcription_start_site	-	120089	120089	160	7871	AN11491
439	CONTIG100	119718	120140	2.53	0.00E+00	transcription_start_site	-	120828	120828	899	7870	AN11491
929	CONTIG100	120168	120802	1.98	1.84E-02	transcription_start_site	-	120828	120828	343	7870	AN11491
929	CONTIG100	120168	120802	1.98	1.84E-02	transcription_start_site	-	120089	120089	-396	7871	AN11491
929	CONTIG100	120168	120802	1.98	1.84E-02	transcription_start_site	-	119828	119828	-657	7872	AN11491
2088	CONTIG100	123243	123577	1.34	1.44E-01	transcription_start_site	-	120828	120828	-2582	7870	AN11491
2088	CONTIG100	123243	123577	1.34	1.44E-01	transcription_start_site	-	120089	120089	-3321	7871	AN11491
2088	CONTIG100	123243	123577	1.34	1.44E-01	transcription_start_site	-	119828	119828	-3582	7872	AN11491
2089	CONTIG100	125493	125977	1.34	1.44E-01	transcription_start_site	-	120828	120828	-4907	7870	AN11491
1318	CONTIG100	291014	291301	1.68	5.22E-02	transcription_start_site	+	290238	290238	919	7875	AN11492
1318	CONTIG100	291014	291301	1.68	5.22E-02	transcription_start_site	+	290190	290190	967	7874	AN11492
294	CONTIG100	325515	326019	2.77	9.51E-04	transcription_start_site	-	325843	325843	76	7876	AN11493
294	CONTIG100	325515	326019	2.77	9.51E-04	transcription_start_site	-	325617	325617	-150	7877	AN11493
356	CONTIG100	328817	329076	2.67	1.22E-03	transcription_start_site	-	325843	325843	-3103	7876	AN11493
356	CONTIG100	328817	329076	2.67	1.22E-03	transcription_start_site	-	325617	325617	-3329	7877	AN11493
2086	CONTIG100	329795	330439	1.34	1.28E-01	transcription_start_site	-	325843	325843	-4274	7876	AN11493
2086	CONTIG100	329795	330439	1.34	1.28E-01	transcription_start_site	-	325617	325617	-4500	7877	AN11493
26	CONTIG102	7955	9281	3.57	0.00E+00	transcription_start_site	-	8054	8054	-564	7882	AN11496
26	CONTIG102	7955	9281	3.57	0.00E+00	transcription_start_site	-	7924	7924	-694	7883	AN11496
26	CONTIG102	7955	9281	3.57	0.00E+00	transcription_start_site	-	7795	7795	-823	7884	AN11496
1156	CONTIG104	149414	149753	1.79	1.06E-02	transcription_start_site	-	145833	145833	-3750	7888	AN11498
1156	CONTIG104	149414	149753	1.79	1.06E-02	transcription_start_site	-	145719	145719	-3864	7889	AN11498
2540	CONTIG104	161794	162203	1.19	1.66E-01	transcription_start_site	+	165932	165932	-3933	7890	AN11499
2540	CONTIG104	161794	162203	1.19	1.66E-01	transcription_start_site	+	166037	166037	-4038	7891	AN11499
2540	CONTIG104	161794	162203	1.19	1.66E-01	transcription_start_site	+	166221	166221	-4222	7892	AN11499
445	CONTIG16	313742	314076	2.53	2.22E-03	transcription_start_site	+	315652	315652	-1743	7893	AN1150
445	CONTIG16	313742	314076	2.53	2.22E-03	transcription_start_site	+	315792	315792	-1883	7894	AN1150
445	CONTIG16	313742	314076	2.53	2.22E-03	transcription_start_site	+	316110	316110	-2201	7895	AN1150
1837	CONTIG16	316292	316701	1.44	1.08E-01	transcription_start_site	+	316110	316110	386	7895	AN1150
1837	CONTIG16	316292	316701	1.44	1.08E-01	transcription_start_site	+	315792	315792	704	7894	AN1150
1837	CONTIG16	316292	316701	1.44	1.08E-01	transcription_start_site	+	315652	315652	844	7893	AN1150
2239	CONTIG16	314867	315809	1.29	1.66E-01	transcription_start_site	+	315652	315652	-314	7893	AN1150
2239	CONTIG16	314867	315809	1.29	1.66E-01	transcription_start_site	+	315792	315792	-454	7894	AN1150
2239	CONTIG16	314867	315809	1.29	1.66E-01	transcription_start_site	+	316110	316110	-772	7895	AN1150
183	CONTIG105	218326	218685	2.98	5.64E-04	transcription_start_site	+	218403	218403	102	7902	AN11502
183	CONTIG105	218326	218685	2.98	5.64E-04	transcription_start_site	+	218250	218250	255	7901	AN11502
183	CONTIG105	218326	218685	2.98	5.64E-04	transcription_start_site	+	218205	218205	300	7900	AN11502
856	CONTIG105	213085	214794	2.05	3.45E-03	transcription_start_site	+	218205	218205	-4265	7900	AN11502
856	CONTIG105	213085	214794	2.05	3.45E-03	transcription_start_site	+	218250	218250	-4310	7901	AN11502
856	CONTIG105	213085	214794	2.05	3.45E-03	transcription_start_site	+	218403	218403	-4463	7902	AN11502
482	CONTIG105	243243	243666	2.47	1.22E-03	transcription_start_site	+	242449	242449	1005	7904	AN11503
482	CONTIG105	243243	243666	2.47	1.22E-03	transcription_start_site	+	242348	242348	1106	7903	AN11503
1384	CONTIG105	241728	242842	1.64	4.42E-02	transcription_start_site	+	242348	242348	-63	7903	AN11503
1384	CONTIG105	241728	242842	1.64	4.42E-02	transcription_start_site	+	242449	242449	-164	7904	AN11503

2096	CONTIG105	238578	239317	1.34	1.66E-01	transcription_start_site	+	242348	242348	-3400	7903 AN11503
2096	CONTIG105	238578	239317	1.34	1.66E-01	transcription_start_site	+	242449	242449	-3501	7904 AN11503
2515	CONTIG106	4446	5390	1.2	1.33E-01	transcription_start_site	-	1270	1270	-3648	7905 AN11504
2515	CONTIG106	4446	5390	1.2	1.33E-01	transcription_start_site	-	953	953	-3965	7906 AN11504
1293	CONTIG107	33320	34644	1.7	2.62E-02	transcription_start_site	-	31203	31203	-2779	7907 AN11505
1293	CONTIG107	33320	34644	1.7	2.62E-02	transcription_start_site	-	31072	31072	-2910	7908 AN11505
369	CONTIG108	168768	169259	2.65	0.00E+00	transcription_start_site	-	170145	170145	1131	7915 AN11508
524	CONTIG108	171621	171967	2.41	6.10E-04	transcription_start_site	-	170641	170641	-1153	7913 AN11508
524	CONTIG108	171621	171967	2.41	6.10E-04	transcription_start_site	-	170606	170606	-1188	7914 AN11508
524	CONTIG108	171621	171967	2.41	6.10E-04	transcription_start_site	-	170145	170145	-1649	7915 AN11508
895	CONTIG108	169733	170302	2	4.46E-04	transcription_start_site	-	170145	170145	127	7915 AN11508
895	CONTIG108	169733	170302	2	4.46E-04	transcription_start_site	-	170606	170606	588	7914 AN11508
895	CONTIG108	169733	170302	2	4.46E-04	transcription_start_site	-	170641	170641	623	7913 AN11508
1837	CONTIG16	316292	316701	1.44	1.08E-01	transcription_start_site	+	319934	319934	-3437	7920 AN1151
1837	CONTIG16	316292	316701	1.44	1.08E-01	transcription_start_site	+	320124	320124	-3627	7921 AN1151
1837	CONTIG16	316292	316701	1.44	1.08E-01	transcription_start_site	+	320293	320293	-3796	7922 AN1151
1837	CONTIG16	316292	316701	1.44	1.08E-01	transcription_start_site	+	320813	320813	-4316	7923 AN1151
2239	CONTIG16	314867	315809	1.29	1.66E-01	transcription_start_site	+	319934	319934	-4596	7920 AN1151
2239	CONTIG16	314867	315809	1.29	1.66E-01	transcription_start_site	+	320124	320124	-4786	7921 AN1151
2239	CONTIG16	314867	315809	1.29	1.66E-01	transcription_start_site	+	320293	320293	-4955	7922 AN1151
564	CONTIG109	9691	10123	2.36	1.54E-03	transcription_start_site	-	5415	5415	-4492	7948 AN11519
564	CONTIG109	9691	10123	2.36	1.54E-03	transcription_start_site	-	4819	4819	-5088	7949 AN11519
564	CONTIG109	9691	10123	2.36	1.54E-03	transcription_start_site	-	4707	4707	-5200	7950 AN11519
329	CONTIG109	66314	66593	2.72	2.39E-04	transcription_start_site	-	64824	64824	-1629	7960 AN11520
329	CONTIG109	66314	66593	2.72	2.39E-04	transcription_start_site	-	64564	64564	-1889	7961 AN11520
1220	CONTIG110	13732	14236	1.75	3.08E-02	transcription_start_site	-	14762	14762	778	7963 AN11521
1220	CONTIG110	13732	14236	1.75	3.08E-02	transcription_start_site	-	14942	14942	958	7962 AN11521
402	CONTIG110	22069	22329	2.6	1.01E-03	transcription_start_site	-	22293	22293	94	7965 AN11522
402	CONTIG110	22069	22329	2.6	1.01E-03	transcription_start_site	-	22442	22442	243	7964 AN11522
360	CONTIG112	24003	24502	2.66	0.00E+00	transcription_start_site	+	25652	25652	-1399	7973 AN11525
360	CONTIG112	24003	24502	2.66	0.00E+00	transcription_start_site	+	25792	25792	-1539	7974 AN11525
360	CONTIG112	24003	24502	2.66	0.00E+00	transcription_start_site	+	26095	26095	-1842	7975 AN11525
773	CONTIG112	193055	193404	2.12	2.22E-03	transcription_start_site	+	193001	193001	228	7977 AN11526
773	CONTIG112	193055	193404	2.12	2.22E-03	transcription_start_site	+	192449	192449	780	7976 AN11526
2712	CONTIG112	193580	194154	1.12	1.44E-01	transcription_start_site	+	193001	193001	866	7977 AN11526
999	CONTIG112	305476	305758	1.91	6.61E-03	transcription_start_site	-	301085	301085	-4532	7978 AN11527
999	CONTIG112	305476	305758	1.91	6.61E-03	transcription_start_site	-	300752	300752	-4865	7979 AN11527
385	CONTIG112	351246	351580	2.62	0.00E+00	transcription_start_site	+	352906	352906	-1493	7987 AN11529
385	CONTIG112	351246	351580	2.62	0.00E+00	transcription_start_site	+	353108	353108	-1695	7988 AN11529
2122	CONTIG113	123527	123876	1.33	1.28E-01	transcription_start_site	-	122198	122198	-1503	7998 AN11531
2122	CONTIG113	123527	123876	1.33	1.28E-01	transcription_start_site	-	121958	121958	-1743	7999 AN11531
2122	CONTIG113	123527	123876	1.33	1.28E-01	transcription_start_site	-	121830	121830	-1871	8000 AN11531
2271	CONTIG113	120992	121341	1.28	1.66E-01	transcription_start_site	-	121830	121830	663	8000 AN11531
2271	CONTIG113	120992	121341	1.28	1.66E-01	transcription_start_site	-	121958	121958	791	7999 AN11531
2271	CONTIG113	120992	121341	1.28	1.66E-01	transcription_start_site	-	122198	122198	1031	7998 AN11531
879	CONTIG117	6393	6667	2.02	8.81E-03	transcription_start_site	-	3732	3732	-2798	8020 AN11539
879	CONTIG117	6393	6667	2.02	8.81E-03	transcription_start_site	-	3506	3506	-3024	8021 AN11539
181	CONTIG16	331659	332005	2.98	2.16E-04	transcription_start_site	-	328441	328441	-3391	8022 AN1154
181	CONTIG16	331659	332005	2.98	2.16E-04	transcription_start_site	-	328143	328143	-3689	8023 AN1154
181	CONTIG16	331659	332005	2.98	2.16E-04	transcription_start_site	-	327861	327861	-3971	8024 AN1154
181	CONTIG16	331659	332005	2.98	2.16E-04	transcription_start_site	-	327608	327608	-4224	8025 AN1154
37	CONTIG117	166963	167377	3.49	0.00E+00	transcription_start_site	+	171586	171586	-4416	8030 AN11541
37	CONTIG117	166963	167377	3.49	0.00E+00	transcription_start_site	+	171770	171770	-4600	8031 AN11541
37	CONTIG117	166963	167377	3.49	0.00E+00	transcription_start_site	+	172105	172105	-4935	8032 AN11541
452	CONTIG117	173195	173459	2.52	1.01E-03	transcription_start_site	-	174072	174072	745	8036 AN11542
452	CONTIG117	173195	173459	2.52	1.01E-03	transcription_start_site	-	174154	174154	827	8035 AN11542
452	CONTIG117	173195	173459	2.52	1.01E-03	transcription_start_site	-	174222	174222	895	8034 AN11542

1671	CONTIG117	228240	228879	1.51	3.99E-02	transcription_start_site	+	228930	228930	-370	8037	AN11543
1671	CONTIG117	228240	228879	1.51	3.99E-02	transcription_start_site	+	229088	229088	-528	8038	AN11543
1671	CONTIG117	228240	228879	1.51	3.99E-02	transcription_start_site	+	229341	229341	-781	8039	AN11543
2227	CONTIG117	230045	230304	1.29	1.25E-01	transcription_start_site	+	229341	229341	833	8039	AN11543
2227	CONTIG117	230045	230304	1.29	1.25E-01	transcription_start_site	+	229088	229088	1086	8038	AN11543
644	CONTIG119	122643	123142	2.24	0.00E+00	transcription_start_site	+	124158	124158	-1265	8042	AN11545
644	CONTIG119	122643	123142	2.24	0.00E+00	transcription_start_site	+	124398	124398	-1505	8043	AN11545
699	CONTIG123	43052	44161	2.19	0.00E+00	transcription_start_site	-	41646	41646	-1960	8055	AN11551
699	CONTIG123	43052	44161	2.19	0.00E+00	transcription_start_site	-	41474	41474	-2132	8056	AN11551
2401	CONTIG128	3841	4105	1.23	5.88E-02	transcription_start_site	+	6650	6650	-2677	8063	AN11554
2401	CONTIG128	3841	4105	1.23	5.88E-02	transcription_start_site	+	6844	6844	-2871	8064	AN11554
487	CONTIG128	107561	108148	2.46	0.00E+00	transcription_start_site	-	103426	103426	-4428	8065	AN11555
487	CONTIG128	107561	108148	2.46	0.00E+00	transcription_start_site	-	103173	103173	-4681	8066	AN11555
487	CONTIG128	107561	108148	2.46	0.00E+00	transcription_start_site	-	103015	103015	-4839	8067	AN11555
2950	CONTIG128	105526	106120	0.97	1.50E-01	transcription_start_site	-	103426	103426	-2397	8065	AN11555
2950	CONTIG128	105526	106120	0.97	1.50E-01	transcription_start_site	-	103173	103173	-2650	8066	AN11555
2950	CONTIG128	105526	106120	0.97	1.50E-01	transcription_start_site	-	103015	103015	-2808	8067	AN11555
181	CONTIG16	331659	332005	2.98	2.16E-04	transcription_start_site	+	332009	332009	-177	8076	AN1156
181	CONTIG16	331659	332005	2.98	2.16E-04	transcription_start_site	+	332197	332197	-365	8077	AN1156
181	CONTIG16	331659	332005	2.98	2.16E-04	transcription_start_site	+	332882	332882	-1050	8078	AN1156
273	CONTIG129	95113	95377	2.8	2.39E-04	transcription_start_site	+	95919	95919	-674	8091	AN11563
1217	CONTIG129	91053	91569	1.75	2.62E-02	transcription_start_site	+	95919	95919	-4608	8091	AN11563
2648	CONTIG129	94058	94421	1.15	1.93E-01	transcription_start_site	+	95919	95919	-1679	8091	AN11563
442	CONTIG129	184971	185245	2.53	1.01E-03	transcription_start_site	+	184835	184835	273	8093	AN11564
442	CONTIG129	184971	185245	2.53	1.01E-03	transcription_start_site	+	184585	184585	523	8092	AN11564
2222	CONTIG129	347039	347683	1.29	1.07E-01	transcription_start_site	+	352169	352169	-4808	8101	AN11567
2222	CONTIG129	347039	347683	1.29	1.07E-01	transcription_start_site	+	352266	352266	-4905	8102	AN11567
2649	CONTIG129	461026	461300	1.15	1.93E-01	transcription_start_site	+	466115	466115	-4952	8105	AN11569
2649	CONTIG129	461026	461300	1.15	1.93E-01	transcription_start_site	+	466281	466281	-5118	8106	AN11569
712	CONTIG16	343129	343564	2.19	8.81E-03	transcription_start_site	-	342583	342583	-763	8107	AN1157
712	CONTIG16	343129	343564	2.19	8.81E-03	transcription_start_site	-	341985	341985	-1361	8108	AN1157
712	CONTIG16	343129	343564	2.19	8.81E-03	transcription_start_site	-	341711	341711	-1635	8109	AN1157
279	CONTIG130	125035	125319	2.8	6.10E-04	transcription_start_site	+	126429	126429	-1252	8110	AN11570
279	CONTIG130	125035	125319	2.8	6.10E-04	transcription_start_site	+	126565	126565	-1388	8111	AN11570
1078	CONTIG130	122420	123219	1.85	2.12E-02	transcription_start_site	+	126429	126429	-3609	8110	AN11570
1078	CONTIG130	122420	123219	1.85	2.12E-02	transcription_start_site	+	126565	126565	-3745	8111	AN11570
769	CONTIG131	99690	100254	2.12	0.00E+00	transcription_start_site	-	95459	95459	-4513	8112	AN11571
769	CONTIG131	99690	100254	2.12	0.00E+00	transcription_start_site	-	95337	95337	-4635	8113	AN11571
712	CONTIG16	343129	343564	2.19	8.81E-03	transcription_start_site	+	345593	345593	-2246	8137	AN1158
712	CONTIG16	343129	343564	2.19	8.81E-03	transcription_start_site	+	345762	345762	-2415	8138	AN1158
712	CONTIG16	343129	343564	2.19	8.81E-03	transcription_start_site	+	348447	348447	-5100	8139	AN1158
1683	CONTIG134	74720	76494	1.5	3.03E-03	transcription_start_site	-	73238	73238	-2369	8144	AN11581
2652	CONTIG134	76970	77310	1.14	2.62E-02	transcription_start_site	-	73238	73238	-3902	8144	AN11581
2807	CONTIG135	3541	3800	1.06	1.08E-01	transcription_start_site	-	1868	1868	-1802	8145	AN11582
2807	CONTIG135	3541	3800	1.06	1.08E-01	transcription_start_site	-	1495	1495	-2175	8146	AN11582
2861	CONTIG135	2551	3135	1.03	1.07E-01	transcription_start_site	-	1868	1868	-975	8145	AN11582
2861	CONTIG135	2551	3135	1.03	1.07E-01	transcription_start_site	-	1495	1495	-1348	8146	AN11582
2344	CONTIG135	96770	97119	1.25	3.33E-02	transcription_start_site	+	95961	95961	983	8150	AN11584
2462	CONTIG135	279768	280412	1.21	3.99E-02	transcription_start_site	+	282978	282978	-2888	8151	AN11585
2462	CONTIG135	279768	280412	1.21	3.99E-02	transcription_start_site	+	283413	283413	-3323	8152	AN11585
2462	CONTIG135	279768	280412	1.21	3.99E-02	transcription_start_site	+	283781	283781	-3691	8153	AN11585
2462	CONTIG135	279768	280412	1.21	3.99E-02	transcription_start_site	+	283960	283960	-3870	8154	AN11585
1354	CONTIG140	19887	20301	1.66	2.64E-02	transcription_start_site	+	21720	21720	-1626	8160	AN11588
1354	CONTIG140	19887	20301	1.66	2.64E-02	transcription_start_site	+	21873	21873	-1779	8161	AN11588
1780	CONTIG140	16952	17241	1.47	8.01E-02	transcription_start_site	+	21720	21720	-4623	8160	AN11588
1780	CONTIG140	16952	17241	1.47	8.01E-02	transcription_start_site	+	21873	21873	-4776	8161	AN11588
2418	CONTIG140	19132	19788	1.23	1.50E-01	transcription_start_site	+	21720	21720	-2260	8160	AN11588

2418	CONTIG140	19132	19788	1.23	1.50E-01	transcription_start_site	+	21873	21873	-2413	8161	AN11588
2959	CONTIG141	57977	58491	0.96	1.50E-01	transcription_start_site	+	58947	58947	-713	8168	AN11590
2959	CONTIG141	57977	58491	0.96	1.50E-01	transcription_start_site	+	59039	59039	-805	8169	AN11590
1236	CONTIG142	19651	20463	1.74	2.62E-02	transcription_start_site	+	24825	24825	-4768	8183	AN11597
1236	CONTIG142	19651	20463	1.74	2.62E-02	transcription_start_site	+	25101	25101	-5044	8184	AN11597
62	CONTIG145	40201	40552	3.37	0.00E+00	transcription_start_site	-	37459	37459	-2917	8185	AN11598
62	CONTIG145	40201	40552	3.37	0.00E+00	transcription_start_site	-	37339	37339	-3037	8186	AN11598
62	CONTIG145	40201	40552	3.37	0.00E+00	transcription_start_site	-	37001	37001	-3375	8187	AN11598
1314	CONTIG145	38496	38900	1.68	3.08E-02	transcription_start_site	-	37459	37459	-1239	8185	AN11598
1314	CONTIG145	38496	38900	1.68	3.08E-02	transcription_start_site	-	37339	37339	-1359	8186	AN11598
1314	CONTIG145	38496	38900	1.68	3.08E-02	transcription_start_site	-	37001	37001	-1697	8187	AN11598
3040	CONTIG146	7506	8090	0.8	1.66E-01	transcription_start_site	-	2934	2934	-4864	8188	AN11599
3040	CONTIG146	7506	8090	0.8	1.66E-01	transcription_start_site	-	2584	2584	-5214	8189	AN11599
2962	CONTIG150	104251	104620	0.95	1.25E-01	transcription_start_site	-	102527	102527	-1908	8196	AN11601
2962	CONTIG150	104251	104620	0.95	1.25E-01	transcription_start_site	-	102240	102240	-2195	8197	AN11601
2769	CONTIG152	26858	27282	1.08	5.41E-03	transcription_start_site	+	31546	31546	-4476	8202	AN11604
2769	CONTIG152	26858	27282	1.08	5.41E-03	transcription_start_site	+	31630	31630	-4560	8203	AN11604
2769	CONTIG152	26858	27282	1.08	5.41E-03	transcription_start_site	+	31890	31890	-4820	8204	AN11605
1893	CONTIG153	82068	82429	1.41	2.12E-02	transcription_start_site	+	82432	82432	-183	8209	AN11607
1893	CONTIG153	82068	82429	1.41	2.12E-02	transcription_start_site	+	82686	82686	-437	8210	AN11607
2882	CONTIG153	79578	80017	1.01	1.25E-01	transcription_start_site	+	82432	82432	-2634	8209	AN11607
2882	CONTIG153	79578	80017	1.01	1.25E-01	transcription_start_site	+	82686	82686	-2888	8210	AN11607
3013	CONTIG153	77788	78202	0.9	1.93E-01	transcription_start_site	+	82432	82432	-4437	8209	AN11607
3013	CONTIG153	77788	78202	0.9	1.93E-01	transcription_start_site	+	82686	82686	-4691	8210	AN11607
2822	CONTIG153	115126	115711	1.05	8.36E-02	transcription_start_site	-	112029	112029	-3389	8211	AN11608
3014	CONTIG153	116182	116761	0.9	1.93E-01	transcription_start_site	-	112029	112029	-4442	8211	AN11608
2628	CONTIG153	157815	158769	1.15	5.05E-02	transcription_start_site	-	155746	155746	-2546	8223	AN11612
2628	CONTIG153	157815	158769	1.15	5.05E-02	transcription_start_site	-	155548	155548	-2744	8224	AN11612
2628	CONTIG153	157815	158769	1.15	5.05E-02	transcription_start_site	-	155448	155448	-2844	8225	AN11612
2628	CONTIG153	157815	158769	1.15	5.05E-02	transcription_start_site	-	155347	155347	-2945	8226	AN11612
2883	CONTIG153	178276	178717	1.01	1.25E-01	transcription_start_site	-	175743	175743	-2753	8227	AN11613
2883	CONTIG153	178276	178717	1.01	1.25E-01	transcription_start_site	-	175520	175520	-2976	8228	AN11613
1414	CONTIG153	342843	343327	1.62	3.31E-03	transcription_start_site	+	344755	344755	-1670	8231	AN11615
1414	CONTIG153	342843	343327	1.62	3.31E-03	transcription_start_site	+	344908	344908	-1823	8232	AN11615
1632	CONTIG153	340058	340632	1.52	1.30E-02	transcription_start_site	+	344755	344755	-4410	8231	AN11615
1632	CONTIG153	340058	340632	1.52	1.30E-02	transcription_start_site	+	344908	344908	-4563	8232	AN11615
1349	CONTIG153	359414	359683	1.66	6.61E-03	transcription_start_site	+	362984	362984	-3435	8233	AN11616
1349	CONTIG153	359414	359683	1.66	6.61E-03	transcription_start_site	+	363753	363753	-4204	8234	AN11616
2175	CONTIG153	359788	360891	1.3	2.18E-02	transcription_start_site	+	362984	362984	-2644	8233	AN11616
2175	CONTIG153	359788	360891	1.3	2.18E-02	transcription_start_site	+	363753	363753	-3413	8234	AN11616
1398	CONTIG154	3471	3743	1.63	3.08E-02	transcription_start_site	-	2063	2063	-1544	8235	AN11617
1398	CONTIG154	3471	3743	1.63	3.08E-02	transcription_start_site	-	1913	1913	-1694	8236	AN11617
506	CONTIG157	38854	39203	2.43	2.16E-04	transcription_start_site	-	37909	37909	-1119	8250	AN11621
506	CONTIG157	38854	39203	2.43	2.16E-04	transcription_start_site	-	37717	37717	-1311	8251	AN11621
506	CONTIG157	38854	39203	2.43	2.16E-04	transcription_start_site	-	37424	37424	-1604	8252	AN11621
352	CONTIG158	86194	87058	2.68	1.22E-03	transcription_start_site	-	86670	86670	44	8261	AN11624
352	CONTIG158	86194	87058	2.68	1.22E-03	transcription_start_site	-	86774	86774	148	8260	AN11624
352	CONTIG158	86194	87058	2.68	1.22E-03	transcription_start_site	-	86439	86439	-187	8262	AN11624
1250	CONTIG158	84454	85783	1.73	8.01E-02	transcription_start_site	-	86439	86439	1320	8262	AN11624
1250	CONTIG158	84454	85783	1.73	8.01E-02	transcription_start_site	-	86670	86670	1551	8261	AN11624
1250	CONTIG158	84454	85783	1.73	8.01E-02	transcription_start_site	-	86774	86774	1655	8260	AN11624
352	CONTIG158	86194	87058	2.68	1.22E-03	transcription_start_site	+	87039	87039	-413	8263	AN11625
352	CONTIG158	86194	87058	2.68	1.22E-03	transcription_start_site	+	87246	87246	-620	8264	AN11625
352	CONTIG158	86194	87058	2.68	1.22E-03	transcription_start_site	+	87527	87527	-901	8265	AN11625
1250	CONTIG158	84454	85783	1.73	8.01E-02	transcription_start_site	+	87039	87039	-1920	8263	AN11625
1250	CONTIG158	84454	85783	1.73	8.01E-02	transcription_start_site	+	87246	87246	-2127	8264	AN11625
1250	CONTIG158	84454	85783	1.73	8.01E-02	transcription_start_site	+	87527	87527	-2408	8265	AN11625

1682	CONTIG160	135382	135661	1.51	1.44E-01	transcription_start_site	-	136387	136387	865	8272	AN11629
1682	CONTIG160	135382	135661	1.51	1.44E-01	transcription_start_site	-	136466	136466	944	8271	AN11629
3057	CONTIG163	55671	56095	0.72	1.44E-01	transcription_start_site	+	57252	57252	-1369	8278	AN11632
3057	CONTIG163	55671	56095	0.72	1.44E-01	transcription_start_site	+	57551	57551	-1668	8279	AN11632
3066	CONTIG163	52666	53080	0.66	1.93E-01	transcription_start_site	+	57252	57252	-4379	8278	AN11632
3066	CONTIG163	52666	53080	0.66	1.93E-01	transcription_start_site	+	57551	57551	-4678	8279	AN11632
260	CONTIG164	48385	48891	2.82	0.00E+00	transcription_start_site	-	47259	47259	-1379	8280	AN11633
260	CONTIG164	48385	48891	2.82	0.00E+00	transcription_start_site	-	46996	46996	-1642	8281	AN11633
260	CONTIG164	48385	48891	2.82	0.00E+00	transcription_start_site	-	46824	46824	-1814	8282	AN11633
260	CONTIG164	48385	48891	2.82	0.00E+00	transcription_start_site	-	46694	46694	-1944	8283	AN11633
2835	CONTIG165	25878	26227	1.05	1.93E-01	transcription_start_site	-	22128	22128	-3924	8284	AN11634
2835	CONTIG165	25878	26227	1.05	1.93E-01	transcription_start_site	-	22013	22013	-4039	8285	AN11634
698	CONTIG168	6451	6875	2.19	0.00E+00	transcription_start_site	-	3517	3517	-3146	8291	AN11638
698	CONTIG168	6451	6875	2.19	0.00E+00	transcription_start_site	-	3319	3319	-3344	8292	AN11638
698	CONTIG168	6451	6875	2.19	0.00E+00	transcription_start_site	-	3037	3037	-3626	8293	AN11638
1478	CONTIG168	65029	65303	1.6	2.62E-02	transcription_start_site	-	61563	61563	-3603	8294	AN11639
1478	CONTIG168	65029	65303	1.6	2.62E-02	transcription_start_site	-	61420	61420	-3746	8295	AN11639
2663	CONTIG168	64054	64558	1.14	1.44E-01	transcription_start_site	-	61563	61563	-2743	8294	AN11639
2663	CONTIG168	64054	64558	1.14	1.44E-01	transcription_start_site	-	61420	61420	-2886	8295	AN11639
21	CONTIG169	238070	238404	3.6	0.00E+00	transcription_start_site	-	238319	238319	82	8303	AN11641
21	CONTIG169	238070	238404	3.6	0.00E+00	transcription_start_site	-	238614	238614	377	8302	AN11641
2409	CONTIG169	320266	320845	1.23	1.08E-01	transcription_start_site	-	316737	316737	-3818	8307	AN11643
2409	CONTIG169	320266	320845	1.23	1.08E-01	transcription_start_site	-	316642	316642	-3913	8308	AN11643
2409	CONTIG169	320266	320845	1.23	1.08E-01	transcription_start_site	-	316501	316501	-4054	8309	AN11643
2409	CONTIG169	320266	320845	1.23	1.08E-01	transcription_start_site	-	316414	316414	-4141	8310	AN11643
2674	CONTIG170	120170	120964	1.13	1.09E-01	transcription_start_site	+	125032	125032	-4465	8313	AN11645
2674	CONTIG170	120170	120964	1.13	1.09E-01	transcription_start_site	+	125102	125102	-4535	8314	AN11645
69	CONTIG172	84095	84440	3.32	0.00E+00	transcription_start_site	-	83534	83534	-733	8322	AN11649
69	CONTIG172	84095	84440	3.32	0.00E+00	transcription_start_site	-	83489	83489	-778	8323	AN11649
69	CONTIG172	84095	84440	3.32	0.00E+00	transcription_start_site	+	84506	84506	-238	8327	AN11650
69	CONTIG172	84095	84440	3.32	0.00E+00	transcription_start_site	+	84625	84625	-357	8328	AN11650
2404	CONTIG172	306159	306653	1.23	5.88E-02	transcription_start_site	-	301476	301476	-4930	8343	AN11656
2404	CONTIG172	306159	306653	1.23	5.88E-02	transcription_start_site	-	301309	301309	-5097	8344	AN11656
2603	CONTIG172	343429	343713	1.16	8.01E-02	transcription_start_site	+	347030	347030	-3459	8345	AN11657
2603	CONTIG172	343429	343713	1.16	8.01E-02	transcription_start_site	+	347197	347197	-3626	8346	AN11657
2914	CONTIG174	16801	17080	0.99	2.62E-02	transcription_start_site	+	19974	19974	-3033	8367	AN11662
2914	CONTIG174	16801	17080	0.99	2.62E-02	transcription_start_site	+	20181	20181	-3240	8368	AN11662
2914	CONTIG174	16801	17080	0.99	2.62E-02	transcription_start_site	+	20298	20298	-3357	8369	AN11662
2914	CONTIG174	16801	17080	0.99	2.62E-02	transcription_start_site	+	20484	20484	-3543	8370	AN11662
2914	CONTIG174	16801	17080	0.99	2.62E-02	transcription_start_site	+	20833	20833	-3892	8371	AN11662
2914	CONTIG174	16801	17080	0.99	2.62E-02	transcription_start_site	+	20901	20901	-3960	8372	AN11662
2914	CONTIG174	16801	17080	0.99	2.62E-02	transcription_start_site	+	21053	21053	-4112	8373	AN11662
2914	CONTIG174	16801	17080	0.99	2.62E-02	transcription_start_site	+	21190	21190	-4249	8374	AN11662
995	CONTIG187	4352	4641	1.92	1.66E-01	transcription_start_site	+	3906	3906	590	8390	AN11667
995	CONTIG187	4352	4641	1.92	1.66E-01	transcription_start_site	+	3824	3824	672	8389	AN11667
995	CONTIG187	4352	4641	1.92	1.66E-01	transcription_start_site	+	3389	3389	1107	8388	AN11667
197	CONTIG187	6992	7266	2.95	1.84E-02	transcription_start_site	-	6561	6561	-568	8391	AN11668
197	CONTIG187	6992	7266	2.95	1.84E-02	transcription_start_site	-	6387	6387	-742	8392	AN11668
833	CONTIG187	7817	8461	2.07	1.07E-01	transcription_start_site	-	6561	6561	-1578	8391	AN11668
833	CONTIG187	7817	8461	2.07	1.07E-01	transcription_start_site	-	6387	6387	-1752	8392	AN11668
958	CONTIG189	5041	5600	1.95	9.21E-03	transcription_start_site	-	3444	3444	-1876	8393	AN11669
958	CONTIG189	5041	5600	1.95	9.21E-03	transcription_start_site	-	3150	3150	-2170	8394	AN11669
2034	CONTIG223	4276	4775	1.36	3.70E-02	transcription_start_site	-	1539	1539	-2986	8408	AN11674
2034	CONTIG223	4276	4775	1.36	3.70E-02	transcription_start_site	-	1309	1309	-3216	8409	AN11674
2091	CONTIG16	386265	386529	1.34	1.44E-01	transcription_start_site	-	381951	381951	-4446	8422	AN1170
2091	CONTIG16	386265	386529	1.34	1.44E-01	transcription_start_site	-	381877	381877	-4520	8423	AN1170
2091	CONTIG16	386265	386529	1.34	1.44E-01	transcription_start_site	-	381776	381776	-4621	8424	AN1170

1944	CONTIG16	389251	390425	1.39	8.00E-02	transcription_start_site	-	385968	385968	-3870	8426 AN1171
1944	CONTIG16	389251	390425	1.39	8.00E-02	transcription_start_site	-	385707	385707	-4131	8427 AN1171
1944	CONTIG16	389251	390425	1.39	8.00E-02	transcription_start_site	-	385021	385021	-4817	8428 AN1171
2091	CONTIG16	386265	386529	1.34	1.44E-01	transcription_start_site	-	385968	385968	-429	8426 AN1171
2091	CONTIG16	386265	386529	1.34	1.44E-01	transcription_start_site	-	385707	385707	-690	8427 AN1171
2091	CONTIG16	386265	386529	1.34	1.44E-01	transcription_start_site	-	385021	385021	-1376	8428 AN1171
2091	CONTIG16	386265	386529	1.34	1.44E-01	transcription_start_site	+	387432	387432	-1035	8429 AN1172
2091	CONTIG16	386265	386529	1.34	1.44E-01	transcription_start_site	+	387697	387697	-1300	8430 AN1172
1944	CONTIG16	389251	390425	1.39	8.00E-02	transcription_start_site	+	390102	390102	-264	8431 AN1173
1944	CONTIG16	389251	390425	1.39	8.00E-02	transcription_start_site	+	390168	390168	-330	8432 AN1173
1944	CONTIG16	389251	390425	1.39	8.00E-02	transcription_start_site	+	391173	391173	-1335	8433 AN1173
2091	CONTIG16	386265	386529	1.34	1.44E-01	transcription_start_site	+	390102	390102	-3705	8431 AN1173
2091	CONTIG16	386265	386529	1.34	1.44E-01	transcription_start_site	+	390168	390168	-3771	8432 AN1173
2091	CONTIG16	386265	386529	1.34	1.44E-01	transcription_start_site	+	391173	391173	-4776	8433 AN1173
1238	CONTIG16	397516	398093	1.74	4.36E-02	transcription_start_site	-	396986	396986	-818	8434 AN1174
1238	CONTIG16	397516	398093	1.74	4.36E-02	transcription_start_site	-	395885	395885	-1919	8435 AN1174
1238	CONTIG16	397516	398093	1.74	4.36E-02	transcription_start_site	-	395742	395742	-2062	8436 AN1174
1238	CONTIG16	397516	398093	1.74	4.36E-02	transcription_start_site	-	395204	395204	-2600	8437 AN1174
1238	CONTIG16	397516	398093	1.74	4.36E-02	transcription_start_site	-	395076	395076	-2728	8438 AN1174
1238	CONTIG16	397516	398093	1.74	4.36E-02	transcription_start_site	-	394969	394969	-2835	8439 AN1174
1238	CONTIG16	397516	398093	1.74	4.36E-02	transcription_start_site	-	394733	394733	-3071	8440 AN1174
1238	CONTIG16	397516	398093	1.74	4.36E-02	transcription_start_site	-	393257	393257	-4547	8441 AN1174
1238	CONTIG16	397516	398093	1.74	4.36E-02	transcription_start_site	-	392802	392802	-5002	8442 AN1174
1238	CONTIG16	397516	398093	1.74	4.36E-02	transcription_start_site	+	398927	398927	-1122	8444 AN1175
1238	CONTIG16	397516	398093	1.74	4.36E-02	transcription_start_site	+	399026	399026	-1221	8445 AN1175
1238	CONTIG16	397516	398093	1.74	4.36E-02	transcription_start_site	+	399195	399195	-1390	8446 AN1175
1838	CONTIG16	407178	407542	1.44	1.08E-01	transcription_start_site	-	405791	405791	-1569	8450 AN1177
1838	CONTIG16	407178	407542	1.44	1.08E-01	transcription_start_site	-	405421	405421	-1939	8451 AN1177
1838	CONTIG16	407178	407542	1.44	1.08E-01	transcription_start_site	-	404951	404951	-2409	8452 AN1177
1838	CONTIG16	407178	407542	1.44	1.08E-01	transcription_start_site	-	404514	404514	-2846	8453 AN1177
1838	CONTIG16	407178	407542	1.44	1.08E-01	transcription_start_site	-	402864	402864	-4496	8454 AN1177
1838	CONTIG16	407178	407542	1.44	1.08E-01	transcription_start_site	+	406983	406983	377	8461 AN1178
1838	CONTIG16	407178	407542	1.44	1.08E-01	transcription_start_site	+	406802	406802	558	8460 AN1178
1838	CONTIG16	407178	407542	1.44	1.08E-01	transcription_start_site	+	406664	406664	696	8459 AN1178
1838	CONTIG16	407178	407542	1.44	1.08E-01	transcription_start_site	+	406548	406548	812	8458 AN1178
1838	CONTIG16	407178	407542	1.44	1.08E-01	transcription_start_site	+	406397	406397	963	8457 AN1178
1838	CONTIG16	407178	407542	1.44	1.08E-01	transcription_start_site	+	406308	406308	1052	8456 AN1178
1838	CONTIG16	407178	407542	1.44	1.08E-01	transcription_start_site	+	406216	406216	1144	8455 AN1178
1838	CONTIG16	407178	407542	1.44	1.08E-01	transcription_start_site	+	407543	407543	-183	8462 AN1179
1838	CONTIG16	407178	407542	1.44	1.08E-01	transcription_start_site	+	407993	407993	-633	8463 AN1179
1838	CONTIG16	407178	407542	1.44	1.08E-01	transcription_start_site	+	408220	408220	-860	8464 AN1179
1838	CONTIG16	407178	407542	1.44	1.08E-01	transcription_start_site	+	408628	408628	-1268	8465 AN1179
1732	CONTIG16	431710	431994	1.49	9.24E-02	transcription_start_site	+	431161	431161	691	8486 AN1183
1732	CONTIG16	431710	431994	1.49	9.24E-02	transcription_start_site	+	430938	430938	914	8485 AN1183
1732	CONTIG16	431710	431994	1.49	9.24E-02	transcription_start_site	+	433878	433878	-2026	8488 AN1185
1732	CONTIG16	431710	431994	1.49	9.24E-02	transcription_start_site	+	434079	434079	-2227	8489 AN1185
1732	CONTIG16	431710	431994	1.49	9.24E-02	transcription_start_site	+	435148	435148	-3296	8490 AN1185
1019	CONTIG16	449476	450219	1.89	0.00E+00	transcription_start_site	-	449162	449162	-685	8504 AN1189
1019	CONTIG16	449476	450219	1.89	0.00E+00	transcription_start_site	-	446094	446094	-3753	8505 AN1189
1019	CONTIG16	449476	450219	1.89	0.00E+00	transcription_start_site	-	445906	445906	-3941	8506 AN1189
1019	CONTIG16	449476	450219	1.89	0.00E+00	transcription_start_site	-	445600	445600	-4247	8507 AN1189
1618	CONTIG17	7811	8240	1.53	3.33E-02	transcription_start_site	-	4608	4608	-3417	8508 AN1190
1618	CONTIG17	7811	8240	1.53	3.33E-02	transcription_start_site	-	4476	4476	-3549	8509 AN1190
1618	CONTIG17	7811	8240	1.53	3.33E-02	transcription_start_site	-	4205	4205	-3820	8510 AN1190
2587	CONTIG17	5251	5691	1.17	1.50E-01	transcription_start_site	-	4608	4608	-863	8508 AN1190
2587	CONTIG17	5251	5691	1.17	1.50E-01	transcription_start_site	-	4476	4476	-995	8509 AN1190
2587	CONTIG17	5251	5691	1.17	1.50E-01	transcription_start_site	-	4205	4205	-1266	8510 AN1190

2587	CONTIG17	5251	5691	1.17	1.50E-01	transcription_start_site	-	2108	2108	-3363	8511	AN1190
2587	CONTIG17	5251	5691	1.17	1.50E-01	transcription_start_site	-	1586	1586	-3885	8512	AN1190
2587	CONTIG17	5251	5691	1.17	1.50E-01	transcription_start_site	+	6062	6062	-591	8513	AN1191
2587	CONTIG17	5251	5691	1.17	1.50E-01	transcription_start_site	+	6382	6382	-911	8514	AN1191
1618	CONTIG17	7811	8240	1.53	3.33E-02	transcription_start_site	-	7757	7757	-268	8515	AN1192
1618	CONTIG17	7811	8240	1.53	3.33E-02	transcription_start_site	-	7560	7560	-465	8516	AN1192
1618	CONTIG17	7811	8240	1.53	3.33E-02	transcription_start_site	+	8657	8657	-631	8517	AN1193
1618	CONTIG17	7811	8240	1.53	3.33E-02	transcription_start_site	+	8929	8929	-903	8518	AN1193
1618	CONTIG17	7811	8240	1.53	3.33E-02	transcription_start_site	+	9092	9092	-1066	8519	AN1193
1618	CONTIG17	7811	8240	1.53	3.33E-02	transcription_start_site	+	9288	9288	-1262	8520	AN1193
1618	CONTIG17	7811	8240	1.53	3.33E-02	transcription_start_site	+	9715	9715	-1689	8521	AN1193
1618	CONTIG17	7811	8240	1.53	3.33E-02	transcription_start_site	+	9959	9959	-1933	8522	AN1193
1618	CONTIG17	7811	8240	1.53	3.33E-02	transcription_start_site	+	10640	10640	-2614	8523	AN1193
1618	CONTIG17	7811	8240	1.53	3.33E-02	transcription_start_site	+	10728	10728	-2702	8524	AN1193
1618	CONTIG17	7811	8240	1.53	3.33E-02	transcription_start_site	+	11105	11105	-3079	8525	AN1193
2587	CONTIG17	5251	5691	1.17	1.50E-01	transcription_start_site	+	8657	8657	-3186	8517	AN1193
2587	CONTIG17	5251	5691	1.17	1.50E-01	transcription_start_site	+	8929	8929	-3458	8518	AN1193
2587	CONTIG17	5251	5691	1.17	1.50E-01	transcription_start_site	+	9092	9092	-3621	8519	AN1193
2587	CONTIG17	5251	5691	1.17	1.50E-01	transcription_start_site	+	9288	9288	-3817	8520	AN1193
2587	CONTIG17	5251	5691	1.17	1.50E-01	transcription_start_site	+	9715	9715	-4244	8521	AN1193
2587	CONTIG17	5251	5691	1.17	1.50E-01	transcription_start_site	+	9959	9959	-4488	8522	AN1193
2587	CONTIG17	5251	5691	1.17	1.50E-01	transcription_start_site	+	10640	10640	-5169	8523	AN1193
1751	CONTIG17	15463	15744	1.48	5.88E-02	transcription_start_site	-	12117	12117	-3486	8526	AN1194
1751	CONTIG17	15463	15744	1.48	5.88E-02	transcription_start_site	-	12050	12050	-3553	8527	AN1194
1751	CONTIG17	15463	15744	1.48	5.88E-02	transcription_start_site	-	13890	13890	-1713	8528	AN1195
1751	CONTIG17	15463	15744	1.48	5.88E-02	transcription_start_site	-	13532	13532	-2071	8529	AN1195
1751	CONTIG17	15463	15744	1.48	5.88E-02	transcription_start_site	-	13391	13391	-2212	8530	AN1195
1751	CONTIG17	15463	15744	1.48	5.88E-02	transcription_start_site	-	13252	13252	-2351	8531	AN1195
1751	CONTIG17	15463	15744	1.48	5.88E-02	transcription_start_site	-	12713	12713	-2890	8532	AN1195
1751	CONTIG17	15463	15744	1.48	5.88E-02	transcription_start_site	-	15316	15316	-287	8533	AN1196
1751	CONTIG17	15463	15744	1.48	5.88E-02	transcription_start_site	-	15126	15126	-477	8534	AN1196
1751	CONTIG17	15463	15744	1.48	5.88E-02	transcription_start_site	+	16525	16525	-921	8535	AN1197
1751	CONTIG17	15463	15744	1.48	5.88E-02	transcription_start_site	+	16704	16704	-1100	8536	AN1197
1751	CONTIG17	15463	15744	1.48	5.88E-02	transcription_start_site	+	17392	17392	-1788	8537	AN1197
1751	CONTIG17	15463	15744	1.48	5.88E-02	transcription_start_site	+	17759	17759	-2155	8538	AN1197
1751	CONTIG17	15463	15744	1.48	5.88E-02	transcription_start_site	+	17953	17953	-2349	8539	AN1197
1751	CONTIG17	15463	15744	1.48	5.88E-02	transcription_start_site	+	18451	18451	-2847	8540	AN1197
1751	CONTIG17	15463	15744	1.48	5.88E-02	transcription_start_site	+	18597	18597	-2993	8541	AN1197
97	CONTIG17	24978	25786	3.23	0.00E+00	transcription_start_site	-	21889	21889	-3493	8542	AN1198
97	CONTIG17	24978	25786	3.23	0.00E+00	transcription_start_site	-	21458	21458	-3924	8543	AN1198
97	CONTIG17	24978	25786	3.23	0.00E+00	transcription_start_site	-	24240	24240	-1142	8544	AN1199
97	CONTIG17	24978	25786	3.23	0.00E+00	transcription_start_site	-	23948	23948	-1434	8545	AN1199
97	CONTIG17	24978	25786	3.23	0.00E+00	transcription_start_site	-	23746	23746	-1636	8546	AN1199
97	CONTIG17	24978	25786	3.23	0.00E+00	transcription_start_site	-	23572	23572	-1810	8547	AN1199
97	CONTIG17	24978	25786	3.23	0.00E+00	transcription_start_site	-	22571	22571	-2811	8548	AN1199
97	CONTIG17	24978	25786	3.23	0.00E+00	transcription_start_site	-	26675	26675	1293	8552	AN1200
97	CONTIG17	24978	25786	3.23	0.00E+00	transcription_start_site	+	30542	30542	-5160	8553	AN1201
1752	CONTIG17	44185	44454	1.48	5.88E-02	transcription_start_site	-	39513	39513	-4806	8556	AN1203
1752	CONTIG17	44185	44454	1.48	5.88E-02	transcription_start_site	-	39257	39257	-5062	8557	AN1203
1752	CONTIG17	44185	44454	1.48	5.88E-02	transcription_start_site	-	42181	42181	-2138	8559	AN1204
1752	CONTIG17	44185	44454	1.48	5.88E-02	transcription_start_site	-	42067	42067	-2252	8560	AN1204
1752	CONTIG17	44185	44454	1.48	5.88E-02	transcription_start_site	-	41893	41893	-2426	8561	AN1204
1752	CONTIG17	44185	44454	1.48	5.88E-02	transcription_start_site	+	44297	44297	22	8562	AN1205
1752	CONTIG17	44185	44454	1.48	5.88E-02	transcription_start_site	+	44427	44427	-107	8563	AN1205
1752	CONTIG17	44185	44454	1.48	5.88E-02	transcription_start_site	+	44774	44774	-454	8564	AN1205
1752	CONTIG17	44185	44454	1.48	5.88E-02	transcription_start_site	+	44948	44948	-628	8565	AN1205
2488	CONTIG17	49880	50154	1.21	1.44E-01	transcription_start_site	-	46851	46851	-3166	8566	AN1206

2488	CONTIG17	49880	50154	1.21	1.44E-01	transcription_start_site	-	46043	46043	-3974	8567 AN1206
2488	CONTIG17	49880	50154	1.21	1.44E-01	transcription_start_site	-	45868	45868	-4149	8568 AN1206
2488	CONTIG17	49880	50154	1.21	1.44E-01	transcription_start_site	-	45476	45476	-4541	8569 AN1206
1752	CONTIG17	44185	44454	1.48	5.88E-02	transcription_start_site	+	48070	48070	-3750	8570 AN1207
1752	CONTIG17	44185	44454	1.48	5.88E-02	transcription_start_site	+	48916	48916	-4596	8571 AN1207
1752	CONTIG17	44185	44454	1.48	5.88E-02	transcription_start_site	+	49095	49095	-4775	8572 AN1207
2488	CONTIG17	49880	50154	1.21	1.44E-01	transcription_start_site	+	49095	49095	922	8572 AN1207
2488	CONTIG17	49880	50154	1.21	1.44E-01	transcription_start_site	+	48916	48916	1101	8571 AN1207
2488	CONTIG17	49880	50154	1.21	1.44E-01	transcription_start_site	+	52597	52597	-2580	8574 AN1209
2488	CONTIG17	49880	50154	1.21	1.44E-01	transcription_start_site	+	52713	52713	-2696	8575 AN1209
2488	CONTIG17	49880	50154	1.21	1.44E-01	transcription_start_site	+	52804	52804	-2787	8576 AN1209
2488	CONTIG17	49880	50154	1.21	1.44E-01	transcription_start_site	+	52997	52997	-2980	8577 AN1209
2488	CONTIG17	49880	50154	1.21	1.44E-01	transcription_start_site	+	53177	53177	-3160	8578 AN1209
2488	CONTIG17	49880	50154	1.21	1.44E-01	transcription_start_site	+	53557	53557	-3540	8579 AN1209
1038	CONTIG17	62629	63128	1.88	5.41E-03	transcription_start_site	+	63126	63126	-247	8591 AN1213
1038	CONTIG17	62629	63128	1.88	5.41E-03	transcription_start_site	+	62468	62468	410	8590 AN1213
1038	CONTIG17	62629	63128	1.88	5.41E-03	transcription_start_site	+	64106	64106	-1227	8592 AN1213
1038	CONTIG17	62629	63128	1.88	5.41E-03	transcription_start_site	+	65691	65691	-2812	8593 AN1213
1038	CONTIG17	62629	63128	1.88	5.41E-03	transcription_start_site	+	66546	66546	-3667	8594 AN1214
1038	CONTIG17	62629	63128	1.88	5.41E-03	transcription_start_site	+	66707	66707	-3828	8595 AN1214
1038	CONTIG17	62629	63128	1.88	5.41E-03	transcription_start_site	+	67379	67379	-4500	8596 AN1214
1038	CONTIG17	62629	63128	1.88	5.41E-03	transcription_start_site	+	67459	67459	-4580	8597 AN1214
652	CONTIG17	75169	75509	2.24	3.03E-03	transcription_start_site	+	76910	76910	-1571	8605 AN1217
652	CONTIG17	75169	75509	2.24	3.03E-03	transcription_start_site	+	77210	77210	-1871	8606 AN1217
652	CONTIG17	75169	75509	2.24	3.03E-03	transcription_start_site	+	78202	78202	-2863	8607 AN1217
1138	CONTIG17	77942	78366	1.8	1.84E-02	transcription_start_site	+	78202	78202	-48	8607 AN1217
1138	CONTIG17	77942	78366	1.8	1.84E-02	transcription_start_site	+	77210	77210	944	8606 AN1217
652	CONTIG17	75169	75509	2.24	3.03E-03	transcription_start_site	+	79310	79310	-3971	8608 AN1218
652	CONTIG17	75169	75509	2.24	3.03E-03	transcription_start_site	+	79537	79537	-4198	8609 AN1218
652	CONTIG17	75169	75509	2.24	3.03E-03	transcription_start_site	+	79872	79872	-4533	8610 AN1218
1138	CONTIG17	77942	78366	1.8	1.84E-02	transcription_start_site	+	79310	79310	-1156	8608 AN1218
1138	CONTIG17	77942	78366	1.8	1.84E-02	transcription_start_site	+	79537	79537	-1383	8609 AN1218
1138	CONTIG17	77942	78366	1.8	1.84E-02	transcription_start_site	+	79872	79872	-1718	8610 AN1218
237	CONTIG17	82958	84427	2.87	0.00E+00	transcription_start_site	-	83703	83703	10	8611 AN1219
237	CONTIG17	82958	84427	2.87	0.00E+00	transcription_start_site	-	83432	83432	-260	8612 AN1219
237	CONTIG17	82958	84427	2.87	0.00E+00	transcription_start_site	-	83168	83168	-524	8613 AN1219
98	CONTIG17	90542	90881	3.23	0.00E+00	transcription_start_site	+	91114	91114	-402	8614 AN1220
167	CONTIG17	89262	89691	3.01	0.00E+00	transcription_start_site	+	91114	91114	-1637	8614 AN1220
2489	CONTIG17	97142	97626	1.21	1.44E-01	transcription_start_site	-	96134	96134	-1250	8615 AN1221
2489	CONTIG17	97142	97626	1.21	1.44E-01	transcription_start_site	-	95935	95935	-1449	8616 AN1221
2489	CONTIG17	97142	97626	1.21	1.44E-01	transcription_start_site	-	95168	95168	-2216	8617 AN1221
2489	CONTIG17	97142	97626	1.21	1.44E-01	transcription_start_site	-	97901	97901	517	8620 AN1222
2489	CONTIG17	97142	97626	1.21	1.44E-01	transcription_start_site	-	98014	98014	630	8619 AN1222
2489	CONTIG17	97142	97626	1.21	1.44E-01	transcription_start_site	-	98178	98178	794	8618 AN1222
2489	CONTIG17	97142	97626	1.21	1.44E-01	transcription_start_site	+	98735	98735	-1351	8621 AN1223
2489	CONTIG17	97142	97626	1.21	1.44E-01	transcription_start_site	+	99479	99479	-2095	8622 AN1223
2592	CONTIG17	93527	93816	1.17	1.66E-01	transcription_start_site	+	98735	98735	-5063	8621 AN1223
2490	CONTIG17	107851	108212	1.21	1.44E-01	transcription_start_site	-	108143	108143	111	8628 AN1226
2490	CONTIG17	107851	108212	1.21	1.44E-01	transcription_start_site	-	107652	107652	-379	8629 AN1226
2490	CONTIG17	107851	108212	1.21	1.44E-01	transcription_start_site	+	109535	109535	-1503	8630 AN1227
2490	CONTIG17	107851	108212	1.21	1.44E-01	transcription_start_site	+	109736	109736	-1704	8631 AN1227
2490	CONTIG17	107851	108212	1.21	1.44E-01	transcription_start_site	+	110252	110252	-2220	8632 AN1227
2490	CONTIG17	107851	108212	1.21	1.44E-01	transcription_start_site	+	110903	110903	-2871	8633 AN1227
2490	CONTIG17	107851	108212	1.21	1.44E-01	transcription_start_site	+	111170	111170	-3138	8634 AN1227
2490	CONTIG17	107851	108212	1.21	1.44E-01	transcription_start_site	+	111341	111341	-3309	8635 AN1227
2490	CONTIG17	107851	108212	1.21	1.44E-01	transcription_start_site	+	111500	111500	-3468	8636 AN1227
2490	CONTIG17	107851	108212	1.21	1.44E-01	transcription_start_site	+	111913	111913	-3881	8637 AN1227

2490	CONTIG17	107851	108212	1.21	1.44E-01	transcription_start_site	+	112008	112008	-3976	8638 AN1227
2334	CONTIG17	114226	114510	1.26	1.25E-01	transcription_start_site	-	114181	114181	-187	8639 AN1228
2334	CONTIG17	114226	114510	1.26	1.25E-01	transcription_start_site	-	114107	114107	-261	8640 AN1228
2334	CONTIG17	114226	114510	1.26	1.25E-01	transcription_start_site	-	113638	113638	-730	8641 AN1228
2334	CONTIG17	114226	114510	1.26	1.25E-01	transcription_start_site	-	113319	113319	-1049	8642 AN1228
2334	CONTIG17	114226	114510	1.26	1.25E-01	transcription_start_site	+	114536	114536	-168	8643 AN1229
2334	CONTIG17	114226	114510	1.26	1.25E-01	transcription_start_site	+	114676	114676	-308	8644 AN1229
2334	CONTIG17	114226	114510	1.26	1.25E-01	transcription_start_site	+	114840	114840	-472	8645 AN1229
2334	CONTIG17	114226	114510	1.26	1.25E-01	transcription_start_site	+	115179	115179	-811	8646 AN1229
2334	CONTIG17	114226	114510	1.26	1.25E-01	transcription_start_site	+	117176	117176	-2808	8649 AN1231
2334	CONTIG17	114226	114510	1.26	1.25E-01	transcription_start_site	+	117395	117395	-3027	8650 AN1231
2334	CONTIG17	114226	114510	1.26	1.25E-01	transcription_start_site	+	117635	117635	-3267	8651 AN1231
2334	CONTIG17	114226	114510	1.26	1.25E-01	transcription_start_site	+	118061	118061	-3693	8652 AN1231
2197	CONTIG17	173564	173918	1.3	1.08E-01	transcription_start_site	-	173576	173576	-165	8705 AN1243
2197	CONTIG17	173564	173918	1.3	1.08E-01	transcription_start_site	-	173123	173123	-618	8706 AN1243
2197	CONTIG17	173564	173918	1.3	1.08E-01	transcription_start_site	-	173040	173040	-701	8707 AN1243
2197	CONTIG17	173564	173918	1.3	1.08E-01	transcription_start_site	-	172884	172884	-857	8708 AN1243
2197	CONTIG17	173564	173918	1.3	1.08E-01	transcription_start_site	-	172492	172492	-1249	8709 AN1243
1044	CONTIG17	180754	181107	1.88	1.30E-02	transcription_start_site	-	176432	176432	-4498	8710 AN1244
1044	CONTIG17	180754	181107	1.88	1.30E-02	transcription_start_site	-	176333	176333	-4597	8711 AN1244
2197	CONTIG17	173564	173918	1.3	1.08E-01	transcription_start_site	+	178059	178059	-4318	8713 AN1245
2197	CONTIG17	173564	173918	1.3	1.08E-01	transcription_start_site	+	178300	178300	-4559	8714 AN1245
2197	CONTIG17	173564	173918	1.3	1.08E-01	transcription_start_site	+	178571	178571	-4830	8715 AN1245
1044	CONTIG17	180754	181107	1.88	1.30E-02	transcription_start_site	-	180603	180603	-327	8716 AN1246
1044	CONTIG17	180754	181107	1.88	1.30E-02	transcription_start_site	-	180480	180480	-450	8717 AN1246
1044	CONTIG17	180754	181107	1.88	1.30E-02	transcription_start_site	-	179996	179996	-934	8718 AN1246
1044	CONTIG17	180754	181107	1.88	1.30E-02	transcription_start_site	-	181532	181532	601	8723 AN1247
1044	CONTIG17	180754	181107	1.88	1.30E-02	transcription_start_site	-	181752	181752	821	8722 AN1247
1044	CONTIG17	180754	181107	1.88	1.30E-02	transcription_start_site	+	185699	185699	-4768	8730 AN1249
1044	CONTIG17	180754	181107	1.88	1.30E-02	transcription_start_site	+	185901	185901	-4970	8731 AN1249
932	CONTIG17	194490	195125	1.97	1.17E-03	transcription_start_site	-	191241	191241	-3566	8733 AN1250
932	CONTIG17	194490	195125	1.97	1.17E-03	transcription_start_site	-	190868	190868	-3939	8734 AN1250
1039	CONTIG17	191044	191995	1.88	5.41E-03	transcription_start_site	-	191241	191241	-278	8733 AN1250
1039	CONTIG17	191044	191995	1.88	5.41E-03	transcription_start_site	-	190868	190868	-651	8734 AN1250
2593	CONTIG17	190444	191013	1.17	1.66E-01	transcription_start_site	-	190868	190868	139	8734 AN1250
2593	CONTIG17	190444	191013	1.17	1.66E-01	transcription_start_site	-	191241	191241	512	8733 AN1250
932	CONTIG17	194490	195125	1.97	1.17E-03	transcription_start_site	-	193333	193333	-1474	8735 AN1251
1215	CONTIG17	197196	197624	1.75	2.12E-02	transcription_start_site	-	193333	193333	-4077	8735 AN1251
1753	CONTIG17	202746	203000	1.48	5.88E-02	transcription_start_site	-	199950	199950	-2923	8736 AN1252
1215	CONTIG17	197196	197624	1.75	2.12E-02	transcription_start_site	+	200955	200955	-3545	8737 AN1253
1215	CONTIG17	197196	197624	1.75	2.12E-02	transcription_start_site	+	201195	201195	-3785	8738 AN1253
1753	CONTIG17	202746	203000	1.48	5.88E-02	transcription_start_site	+	202804	202804	69	8739 AN1254
1753	CONTIG17	202746	203000	1.48	5.88E-02	transcription_start_site	+	203420	203420	-547	8740 AN1254
976	CONTIG17	213992	214342	1.93	1.11E-02	transcription_start_site	-	209538	209538	-4629	8741 AN1255
1620	CONTIG17	210092	210366	1.53	5.22E-02	transcription_start_site	-	209538	209538	-691	8741 AN1255
976	CONTIG17	213992	214342	1.93	1.11E-02	transcription_start_site	+	213013	213013	1154	8748 AN1256
1620	CONTIG17	210092	210366	1.53	5.22E-02	transcription_start_site	+	211151	211151	-922	8743 AN1256
1620	CONTIG17	210092	210366	1.53	5.22E-02	transcription_start_site	+	211366	211366	-1137	8744 AN1256
1620	CONTIG17	210092	210366	1.53	5.22E-02	transcription_start_site	+	211578	211578	-1349	8745 AN1256
1620	CONTIG17	210092	210366	1.53	5.22E-02	transcription_start_site	+	211726	211726	-1497	8746 AN1256
1620	CONTIG17	210092	210366	1.53	5.22E-02	transcription_start_site	+	212859	212859	-2630	8747 AN1256
1620	CONTIG17	210092	210366	1.53	5.22E-02	transcription_start_site	+	213013	213013	-2784	8748 AN1256
976	CONTIG17	213992	214342	1.93	1.11E-02	transcription_start_site	-	214923	214923	756	8751 AN1257
2335	CONTIG17	219392	219738	1.26	1.25E-01	transcription_start_site	-	215541	215541	-4024	8749 AN1257
2335	CONTIG17	219392	219738	1.26	1.25E-01	transcription_start_site	-	215418	215418	-4147	8750 AN1257
2335	CONTIG17	219392	219738	1.26	1.25E-01	transcription_start_site	-	214923	214923	-4642	8751 AN1257
2335	CONTIG17	219392	219738	1.26	1.25E-01	transcription_start_site	-	218625	218625	-940	8752 AN1258

2335	CONTIG17	219392	219738	1.26	1.25E-01	transcription_start_site	-	220401	220401	836	8754 AN1259
2335	CONTIG17	219392	219738	1.26	1.25E-01	transcription_start_site	+	222461	222461	-2896	8755 AN1260
2335	CONTIG17	219392	219738	1.26	1.25E-01	transcription_start_site	+	224717	224717	-5152	8756 AN1261
587	CONTIG17	243153	243510	2.33	1.80E-03	transcription_start_site	-	240463	240463	-2868	8774 AN1266
587	CONTIG17	243153	243510	2.33	1.80E-03	transcription_start_site	-	240257	240257	-3074	8775 AN1266
587	CONTIG17	243153	243510	2.33	1.80E-03	transcription_start_site	-	239882	239882	-3449	8776 AN1266
587	CONTIG17	243153	243510	2.33	1.80E-03	transcription_start_site	-	239410	239410	-3921	8777 AN1266
587	CONTIG17	243153	243510	2.33	1.80E-03	transcription_start_site	+	244338	244338	-1006	8782 AN1268
587	CONTIG17	243153	243510	2.33	1.80E-03	transcription_start_site	+	244632	244632	-1300	8783 AN1268
587	CONTIG17	243153	243510	2.33	1.80E-03	transcription_start_site	+	245409	245409	-2077	8784 AN1268
1754	CONTIG17	247132	247416	1.48	5.88E-02	transcription_start_site	+	248511	248511	-1237	8785 AN1268
1754	CONTIG17	247132	247416	1.48	5.88E-02	transcription_start_site	+	248688	248688	-1414	8786 AN1268
1947	CONTIG17	246232	246506	1.39	8.01E-02	transcription_start_site	+	245409	245409	960	8784 AN1268
1947	CONTIG17	246232	246506	1.39	8.01E-02	transcription_start_site	+	248511	248511	-2142	8785 AN1268
1947	CONTIG17	246232	246506	1.39	8.01E-02	transcription_start_site	+	248688	248688	-2319	8786 AN1268
2491	CONTIG17	245477	245751	1.21	1.44E-01	transcription_start_site	+	245409	245409	205	8784 AN1268
2491	CONTIG17	245477	245751	1.21	1.44E-01	transcription_start_site	+	244632	244632	982	8783 AN1268
2491	CONTIG17	245477	245751	1.21	1.44E-01	transcription_start_site	+	248511	248511	-2897	8785 AN1268
2491	CONTIG17	245477	245751	1.21	1.44E-01	transcription_start_site	+	248688	248688	-3074	8786 AN1268
2589	CONTIG17	249377	249816	1.17	1.50E-01	transcription_start_site	+	248688	248688	908	8786 AN1268
2589	CONTIG17	249377	249816	1.17	1.50E-01	transcription_start_site	+	248511	248511	1085	8785 AN1268
2492	CONTIG17	263567	263841	1.21	1.44E-01	transcription_start_site	-	259248	259248	-4456	8797 AN1272
2492	CONTIG17	263567	263841	1.21	1.44E-01	transcription_start_site	-	258755	258755	-4949	8798 AN1272
2492	CONTIG17	263567	263841	1.21	1.44E-01	transcription_start_site	-	261251	261251	-2453	8800 AN1273
2492	CONTIG17	263567	263841	1.21	1.44E-01	transcription_start_site	-	260955	260955	-2749	8801 AN1273
2492	CONTIG17	263567	263841	1.21	1.44E-01	transcription_start_site	-	260654	260654	-3050	8802 AN1273
2492	CONTIG17	263567	263841	1.21	1.44E-01	transcription_start_site	-	260465	260465	-3239	8803 AN1273
2492	CONTIG17	263567	263841	1.21	1.44E-01	transcription_start_site	-	260157	260157	-3547	8804 AN1273
2492	CONTIG17	263567	263841	1.21	1.44E-01	transcription_start_site	-	259861	259861	-3843	8805 AN1273
2492	CONTIG17	263567	263841	1.21	1.44E-01	transcription_start_site	+	266216	266216	-2512	8810 AN1275
2492	CONTIG17	263567	263841	1.21	1.44E-01	transcription_start_site	+	266471	266471	-2767	8811 AN1275
1829	CONTIG17	306319	306901	1.44	6.84E-02	transcription_start_site	-	303984	303984	-2626	8856 AN1288
1829	CONTIG17	306319	306901	1.44	6.84E-02	transcription_start_site	+	310275	310275	-3665	8857 AN1290
1829	CONTIG17	306319	306901	1.44	6.84E-02	transcription_start_site	+	310442	310442	-3832	8858 AN1290
1829	CONTIG17	306319	306901	1.44	6.84E-02	transcription_start_site	+	310905	310905	-4295	8859 AN1290
1829	CONTIG17	306319	306901	1.44	6.84E-02	transcription_start_site	+	311620	311620	-5010	8860 AN1290
2326	CONTIG17	309314	309888	1.26	1.07E-01	transcription_start_site	+	310275	310275	-674	8857 AN1290
2326	CONTIG17	309314	309888	1.26	1.07E-01	transcription_start_site	+	310442	310442	-841	8858 AN1290
2326	CONTIG17	309314	309888	1.26	1.07E-01	transcription_start_site	+	310905	310905	-1304	8859 AN1290
2326	CONTIG17	309314	309888	1.26	1.07E-01	transcription_start_site	+	311620	311620	-2019	8860 AN1290
2326	CONTIG17	309314	309888	1.26	1.07E-01	transcription_start_site	+	314701	314701	-5100	8861 AN1291
2326	CONTIG17	309314	309888	1.26	1.07E-01	transcription_start_site	+	314845	314845	-5244	8862 AN1291
752	CONTIG17	318916	319190	2.15	4.61E-03	transcription_start_site	+	318304	318304	749	8867 AN1292
752	CONTIG17	318916	319190	2.15	4.61E-03	transcription_start_site	+	317965	317965	1088	8866 AN1292
752	CONTIG17	318916	319190	2.15	4.61E-03	transcription_start_site	-	319464	319464	411	8869 AN1293
752	CONTIG17	318916	319190	2.15	4.61E-03	transcription_start_site	-	319795	319795	742	8868 AN1293
1948	CONTIG17	326701	327050	1.39	8.01E-02	transcription_start_site	-	322841	322841	-4034	8872 AN1295
752	CONTIG17	318916	319190	2.15	4.61E-03	transcription_start_site	+	323379	323379	-4326	8873 AN1296
425	CONTIG17	332719	332988	2.56	8.69E-04	transcription_start_site	-	328013	328013	-4840	8874 AN1297
1616	CONTIG17	328736	329225	1.53	3.08E-02	transcription_start_site	-	328013	328013	-967	8874 AN1297
1616	CONTIG17	328736	329225	1.53	3.08E-02	transcription_start_site	-	327539	327539	-1441	8875 AN1297
1616	CONTIG17	328736	329225	1.53	3.08E-02	transcription_start_site	-	327210	327210	-1770	8876 AN1297
1616	CONTIG17	328736	329225	1.53	3.08E-02	transcription_start_site	-	326612	326612	-2368	8877 AN1297
1948	CONTIG17	326701	327050	1.39	8.01E-02	transcription_start_site	-	326612	326612	-263	8877 AN1297
1948	CONTIG17	326701	327050	1.39	8.01E-02	transcription_start_site	-	327210	327210	334	8876 AN1297
1948	CONTIG17	326701	327050	1.39	8.01E-02	transcription_start_site	-	327539	327539	663	8875 AN1297
1948	CONTIG17	326701	327050	1.39	8.01E-02	transcription_start_site	-	328013	328013	1137	8874 AN1297

425	CONTIG17	332719	332988	2.56	8.69E-04	transcription_start_site	+	333755	333755	-901	8878 AN1298
1616	CONTIG17	328736	329225	1.53	3.08E-02	transcription_start_site	+	333755	333755	-4774	8878 AN1298
2410	CONTIG18	1741	2024	1.23	1.08E-01	transcription_start_site	-	1601	1601	-281	8884 AN1302
2410	CONTIG18	1741	2024	1.23	1.08E-01	transcription_start_site	-	1380	1380	-502	8885 AN1302
2410	CONTIG18	1741	2024	1.23	1.08E-01	transcription_start_site	-	1092	1092	-790	8886 AN1302
2410	CONTIG18	1741	2024	1.23	1.08E-01	transcription_start_site	-	238	238	-1644	8887 AN1302
2731	CONTIG18	5346	5605	1.11	1.66E-01	transcription_start_site	-	1601	1601	-3874	8884 AN1302
2731	CONTIG18	5346	5605	1.11	1.66E-01	transcription_start_site	-	1380	1380	-4095	8885 AN1302
2731	CONTIG18	5346	5605	1.11	1.66E-01	transcription_start_site	-	1092	1092	-4383	8886 AN1302
2039	CONTIG18	6986	7270	1.36	6.84E-02	transcription_start_site	-	4751	4751	-2377	8888 AN1303
2039	CONTIG18	6986	7270	1.36	6.84E-02	transcription_start_site	-	2672	2672	-4456	8889 AN1303
2410	CONTIG18	1741	2024	1.23	1.08E-01	transcription_start_site	-	2672	2672	789	8889 AN1303
2731	CONTIG18	5346	5605	1.11	1.66E-01	transcription_start_site	-	4751	4751	-724	8888 AN1303
2731	CONTIG18	5346	5605	1.11	1.66E-01	transcription_start_site	-	2672	2672	-2803	8889 AN1303
2039	CONTIG18	6986	7270	1.36	6.84E-02	transcription_start_site	-	6814	6814	-314	8890 AN1304
2039	CONTIG18	6986	7270	1.36	6.84E-02	transcription_start_site	-	6338	6338	-790	8891 AN1304
2255	CONTIG18	11106	11375	1.28	9.24E-02	transcription_start_site	-	6814	6814	-4426	8890 AN1304
2255	CONTIG18	11106	11375	1.28	9.24E-02	transcription_start_site	-	6338	6338	-4902	8891 AN1304
2731	CONTIG18	5346	5605	1.11	1.66E-01	transcription_start_site	-	6338	6338	862	8891 AN1304
2039	CONTIG18	6986	7270	1.36	6.84E-02	transcription_start_site	-	7869	7869	741	8893 AN1305
2255	CONTIG18	11106	11375	1.28	9.24E-02	transcription_start_site	-	8735	8735	-2505	8892 AN1305
2255	CONTIG18	11106	11375	1.28	9.24E-02	transcription_start_site	-	7869	7869	-3371	8893 AN1305
2039	CONTIG18	6986	7270	1.36	6.84E-02	transcription_start_site	+	11315	11315	-4187	8894 AN1306
2255	CONTIG18	11106	11375	1.28	9.24E-02	transcription_start_site	+	11315	11315	-74	8894 AN1306
551	CONTIG18	47401	47750	2.38	9.51E-04	transcription_start_site	-	47496	47496	-79	8946 AN1319
551	CONTIG18	47401	47750	2.38	9.51E-04	transcription_start_site	-	47394	47394	-181	8947 AN1319
551	CONTIG18	47401	47750	2.38	9.51E-04	transcription_start_site	-	47229	47229	-346	8948 AN1319
551	CONTIG18	47401	47750	2.38	9.51E-04	transcription_start_site	+	49044	49044	-1468	8949 AN1320
551	CONTIG18	47401	47750	2.38	9.51E-04	transcription_start_site	+	49161	49161	-1585	8950 AN1320
1716	CONTIG18	66460	66812	1.49	4.36E-02	transcription_start_site	-	65141	65141	-1495	8961 AN1324
1716	CONTIG18	66460	66812	1.49	4.36E-02	transcription_start_site	-	65002	65002	-1634	8962 AN1324
2527	CONTIG18	69543	70253	1.19	1.09E-01	transcription_start_site	-	65141	65141	-4757	8961 AN1324
2527	CONTIG18	69543	70253	1.19	1.09E-01	transcription_start_site	-	65002	65002	-4896	8962 AN1324
2529	CONTIG18	64280	64569	1.19	1.25E-01	transcription_start_site	-	65002	65002	577	8962 AN1324
2529	CONTIG18	64280	64569	1.19	1.25E-01	transcription_start_site	-	65141	65141	716	8961 AN1324
1716	CONTIG18	66460	66812	1.49	4.36E-02	transcription_start_site	+	66862	66862	-226	8963 AN1325
1716	CONTIG18	66460	66812	1.49	4.36E-02	transcription_start_site	+	67269	67269	-633	8964 AN1325
1716	CONTIG18	66460	66812	1.49	4.36E-02	transcription_start_site	+	67513	67513	-877	8965 AN1325
1716	CONTIG18	66460	66812	1.49	4.36E-02	transcription_start_site	+	67803	67803	-1167	8966 AN1325
1716	CONTIG18	66460	66812	1.49	4.36E-02	transcription_start_site	+	70239	70239	-3603	8967 AN1325
2527	CONTIG18	69543	70253	1.19	1.09E-01	transcription_start_site	+	70239	70239	-341	8967 AN1325
2529	CONTIG18	64280	64569	1.19	1.25E-01	transcription_start_site	+	66862	66862	-2437	8963 AN1325
2529	CONTIG18	64280	64569	1.19	1.25E-01	transcription_start_site	+	67269	67269	-2844	8964 AN1325
2529	CONTIG18	64280	64569	1.19	1.25E-01	transcription_start_site	+	67513	67513	-3088	8965 AN1325
2529	CONTIG18	64280	64569	1.19	1.25E-01	transcription_start_site	+	67803	67803	-3378	8966 AN1325
2527	CONTIG18	69543	70253	1.19	1.09E-01	transcription_start_site	+	72361	72361	-2463	8968 AN1326
2527	CONTIG18	69543	70253	1.19	1.09E-01	transcription_start_site	+	72551	72551	-2653	8969 AN1326
2527	CONTIG18	69543	70253	1.19	1.09E-01	transcription_start_site	+	72751	72751	-2853	8970 AN1326
2527	CONTIG18	69543	70253	1.19	1.09E-01	transcription_start_site	+	72916	72916	-3018	8971 AN1326
2527	CONTIG18	69543	70253	1.19	1.09E-01	transcription_start_site	+	73111	73111	-3213	8972 AN1326
2527	CONTIG18	69543	70253	1.19	1.09E-01	transcription_start_site	+	73526	73526	-3628	8973 AN1326
2527	CONTIG18	69543	70253	1.19	1.09E-01	transcription_start_site	+	73872	73872	-3974	8974 AN1326
2527	CONTIG18	69543	70253	1.19	1.09E-01	transcription_start_site	+	74123	74123	-4225	8975 AN1326
2527	CONTIG18	69543	70253	1.19	1.09E-01	transcription_start_site	+	74342	74342	-4444	8976 AN1326
2634	CONTIG18	81767	82031	1.15	1.44E-01	transcription_start_site	-	78850	78850	-3049	8978 AN1328
2634	CONTIG18	81767	82031	1.15	1.44E-01	transcription_start_site	-	78438	78438	-3461	8979 AN1328
2634	CONTIG18	81767	82031	1.15	1.44E-01	transcription_start_site	+	82091	82091	-192	8982 AN1329

2634	CONTIG18	81767	82031	1.15	1.44E-01	transcription_start_site	+	81505	81505	394	8981	AN1329
2634	CONTIG18	81767	82031	1.15	1.44E-01	transcription_start_site	+	81331	81331	568	8980	AN1329
2634	CONTIG18	81767	82031	1.15	1.44E-01	transcription_start_site	+	82800	82800	-901	8983	AN1329
1229	CONTIG18	84455	85051	1.74	7.16E-03	transcription_start_site	-	84666	84666	-87	8984	AN1330
1229	CONTIG18	84455	85051	1.74	7.16E-03	transcription_start_site	-	84025	84025	-728	8985	AN1330
1229	CONTIG18	84455	85051	1.74	7.16E-03	transcription_start_site	-	83377	83377	-1376	8986	AN1330
2530	CONTIG18	86871	87352	1.19	1.25E-01	transcription_start_site	-	84666	84666	-2445	8984	AN1330
2530	CONTIG18	86871	87352	1.19	1.25E-01	transcription_start_site	-	84025	84025	-3086	8985	AN1330
2530	CONTIG18	86871	87352	1.19	1.25E-01	transcription_start_site	-	83377	83377	-3734	8986	AN1330
1229	CONTIG18	84455	85051	1.74	7.16E-03	transcription_start_site	+	85664	85664	-911	8987	AN1331
1229	CONTIG18	84455	85051	1.74	7.16E-03	transcription_start_site	+	86120	86120	-1367	8988	AN1331
2530	CONTIG18	86871	87352	1.19	1.25E-01	transcription_start_site	+	86120	86120	991	8988	AN1331
2634	CONTIG18	81767	82031	1.15	1.44E-01	transcription_start_site	+	85664	85664	-3765	8987	AN1331
2634	CONTIG18	81767	82031	1.15	1.44E-01	transcription_start_site	+	86120	86120	-4221	8988	AN1331
1229	CONTIG18	84455	85051	1.74	7.16E-03	transcription_start_site	+	87436	87436	-2683	8989	AN1332
2530	CONTIG18	86871	87352	1.19	1.25E-01	transcription_start_site	+	87436	87436	-324	8989	AN1332
509	CONTIG18	89778	90127	2.43	8.69E-04	transcription_start_site	-	89561	89561	-391	8990	AN1333
509	CONTIG18	89778	90127	2.43	8.69E-04	transcription_start_site	-	89449	89449	-503	8991	AN1333
509	CONTIG18	89778	90127	2.43	8.69E-04	transcription_start_site	-	89317	89317	-635	8992	AN1333
509	CONTIG18	89778	90127	2.43	8.69E-04	transcription_start_site	-	89176	89176	-776	8993	AN1333
509	CONTIG18	89778	90127	2.43	8.69E-04	transcription_start_site	+	90354	90354	-401	8994	AN1334
509	CONTIG18	89778	90127	2.43	8.69E-04	transcription_start_site	+	90450	90450	-497	8995	AN1334
2530	CONTIG18	86871	87352	1.19	1.25E-01	transcription_start_site	+	90354	90354	-3242	8994	AN1334
2530	CONTIG18	86871	87352	1.19	1.25E-01	transcription_start_site	+	90450	90450	-3338	8995	AN1334
509	CONTIG18	89778	90127	2.43	8.69E-04	transcription_start_site	+	93114	93114	-3161	9000	AN1336
222	CONTIG18	99388	99892	2.89	0.00E+00	transcription_start_site	-	100213	100213	573	9003	AN1338
222	CONTIG18	99388	99892	2.89	0.00E+00	transcription_start_site	-	100389	100389	749	9002	AN1338
222	CONTIG18	99388	99892	2.89	0.00E+00	transcription_start_site	-	100572	100572	932	9001	AN1338
1424	CONTIG18	105093	105352	1.62	2.62E-02	transcription_start_site	-	100572	100572	-4650	9001	AN1338
1424	CONTIG18	105093	105352	1.62	2.62E-02	transcription_start_site	-	100389	100389	-4833	9002	AN1338
1424	CONTIG18	105093	105352	1.62	2.62E-02	transcription_start_site	-	100213	100213	-5009	9003	AN1338
602	CONTIG18	109503	109867	2.3	1.22E-03	transcription_start_site	-	104935	104935	-4750	9004	AN1339
602	CONTIG18	109503	109867	2.3	1.22E-03	transcription_start_site	-	104833	104833	-4852	9005	AN1339
602	CONTIG18	109503	109867	2.3	1.22E-03	transcription_start_site	-	104732	104732	-4953	9006	AN1339
602	CONTIG18	109503	109867	2.3	1.22E-03	transcription_start_site	-	104629	104629	-5056	9007	AN1339
1424	CONTIG18	105093	105352	1.62	2.62E-02	transcription_start_site	-	104935	104935	-287	9004	AN1339
1424	CONTIG18	105093	105352	1.62	2.62E-02	transcription_start_site	-	104833	104833	-389	9005	AN1339
1424	CONTIG18	105093	105352	1.62	2.62E-02	transcription_start_site	-	104732	104732	-490	9006	AN1339
1424	CONTIG18	105093	105352	1.62	2.62E-02	transcription_start_site	-	104629	104629	-593	9007	AN1339
1424	CONTIG18	105093	105352	1.62	2.62E-02	transcription_start_site	-	104454	104454	-768	9008	AN1339
1424	CONTIG18	105093	105352	1.62	2.62E-02	transcription_start_site	-	104217	104217	-1005	9009	AN1339
1424	CONTIG18	105093	105352	1.62	2.62E-02	transcription_start_site	-	102952	102952	-2270	9010	AN1339
1424	CONTIG18	105093	105352	1.62	2.62E-02	transcription_start_site	-	102611	102611	-2611	9011	AN1339
1424	CONTIG18	105093	105352	1.62	2.62E-02	transcription_start_site	-	102426	102426	-2796	9012	AN1339
1424	CONTIG18	105093	105352	1.62	2.62E-02	transcription_start_site	-	101980	101980	-3242	9013	AN1339
602	CONTIG18	109503	109867	2.3	1.22E-03	transcription_start_site	-	107244	107244	-2441	9014	AN1340
602	CONTIG18	109503	109867	2.3	1.22E-03	transcription_start_site	-	107059	107059	-2626	9015	AN1340
602	CONTIG18	109503	109867	2.3	1.22E-03	transcription_start_site	-	106716	106716	-2969	9016	AN1340
602	CONTIG18	109503	109867	2.3	1.22E-03	transcription_start_site	-	106570	106570	-3115	9017	AN1340
602	CONTIG18	109503	109867	2.3	1.22E-03	transcription_start_site	-	108727	108727	-958	9018	AN1341
602	CONTIG18	109503	109867	2.3	1.22E-03	transcription_start_site	-	108607	108607	-1078	9019	AN1341
602	CONTIG18	109503	109867	2.3	1.22E-03	transcription_start_site	-	108518	108518	-1167	9020	AN1341
602	CONTIG18	109503	109867	2.3	1.22E-03	transcription_start_site	-	108040	108040	-1645	9021	AN1341
602	CONTIG18	109503	109867	2.3	1.22E-03	transcription_start_site	+	109851	109851	-166	9022	AN1342
602	CONTIG18	109503	109867	2.3	1.22E-03	transcription_start_site	+	109994	109994	-309	9023	AN1342
1424	CONTIG18	105093	105352	1.62	2.62E-02	transcription_start_site	+	109851	109851	-4628	9022	AN1342
1424	CONTIG18	105093	105352	1.62	2.62E-02	transcription_start_site	+	109994	109994	-4771	9023	AN1342

602	CONTIG18	109503	109867	2.3	1.22E-03	transcription_start_site	+	114307	114307	-4622	9026 AN1344
602	CONTIG18	109503	109867	2.3	1.22E-03	transcription_start_site	+	114459	114459	-4774	9027 AN1344
602	CONTIG18	109503	109867	2.3	1.22E-03	transcription_start_site	+	114810	114810	-5125	9028 AN1344
328	CONTIG18	137338	137677	2.72	0.00E+00	transcription_start_site	+	137985	137985	-477	9058 AN1356
2256	CONTIG18	144903	145201	1.28	9.24E-02	transcription_start_site	-	141569	141569	-3483	9059 AN1357
2256	CONTIG18	144903	145201	1.28	9.24E-02	transcription_start_site	-	141005	141005	-4047	9060 AN1357
2256	CONTIG18	144903	145201	1.28	9.24E-02	transcription_start_site	-	140607	140607	-4445	9061 AN1357
2256	CONTIG18	144903	145201	1.28	9.24E-02	transcription_start_site	-	140158	140158	-4894	9062 AN1357
2256	CONTIG18	144903	145201	1.28	9.24E-02	transcription_start_site	-	144843	144843	-209	9063 AN1358
2256	CONTIG18	144903	145201	1.28	9.24E-02	transcription_start_site	-	144408	144408	-644	9064 AN1358
2256	CONTIG18	144903	145201	1.28	9.24E-02	transcription_start_site	-	144017	144017	-1035	9065 AN1358
2256	CONTIG18	144903	145201	1.28	9.24E-02	transcription_start_site	-	143891	143891	-1161	9066 AN1358
2256	CONTIG18	144903	145201	1.28	9.24E-02	transcription_start_site	-	143752	143752	-1300	9067 AN1358
2256	CONTIG18	144903	145201	1.28	9.24E-02	transcription_start_site	-	143493	143493	-1559	9068 AN1358
2256	CONTIG18	144903	145201	1.28	9.24E-02	transcription_start_site	-	143171	143171	-1881	9069 AN1358
2256	CONTIG18	144903	145201	1.28	9.24E-02	transcription_start_site	-	143022	143022	-2030	9070 AN1358
2818	CONTIG18	160371	160945	1.06	1.99E-01	transcription_start_site	-	155833	155833	-4825	9083 AN1364
2818	CONTIG18	160371	160945	1.06	1.99E-01	transcription_start_site	-	155626	155626	-5032	9084 AN1364
2818	CONTIG18	160371	160945	1.06	1.99E-01	transcription_start_site	-	157191	157191	-3467	9086 AN1365
2818	CONTIG18	160371	160945	1.06	1.99E-01	transcription_start_site	-	157014	157014	-3644	9087 AN1365
2818	CONTIG18	160371	160945	1.06	1.99E-01	transcription_start_site	-	156597	156597	-4061	9088 AN1365
1152	CONTIG18	165841	166326	1.79	4.13E-03	transcription_start_site	-	162411	162411	-3672	9092 AN1367
1152	CONTIG18	165841	166326	1.79	4.13E-03	transcription_start_site	-	162352	162352	-3731	9093 AN1367
2818	CONTIG18	160371	160945	1.06	1.99E-01	transcription_start_site	+	162628	162628	-1970	9094 AN1368
2818	CONTIG18	160371	160945	1.06	1.99E-01	transcription_start_site	+	162816	162816	-2158	9095 AN1368
1152	CONTIG18	165841	166326	1.79	4.13E-03	transcription_start_site	-	165829	165829	-254	9096 AN1369
1152	CONTIG18	165841	166326	1.79	4.13E-03	transcription_start_site	-	165580	165580	-503	9097 AN1369
1152	CONTIG18	165841	166326	1.79	4.13E-03	transcription_start_site	-	165343	165343	-740	9098 AN1369
1152	CONTIG18	165841	166326	1.79	4.13E-03	transcription_start_site	+	166670	166670	-586	9099 AN1370
1152	CONTIG18	165841	166326	1.79	4.13E-03	transcription_start_site	+	166965	166965	-881	9100 AN1370
1152	CONTIG18	165841	166326	1.79	4.13E-03	transcription_start_site	+	167136	167136	-1052	9101 AN1370
1152	CONTIG18	165841	166326	1.79	4.13E-03	transcription_start_site	+	169165	169165	-3081	9103 AN1372
1152	CONTIG18	165841	166326	1.79	4.13E-03	transcription_start_site	+	170068	170068	-3984	9104 AN1372
1152	CONTIG18	165841	166326	1.79	4.13E-03	transcription_start_site	+	170222	170222	-4138	9105 AN1372
1152	CONTIG18	165841	166326	1.79	4.13E-03	transcription_start_site	+	170607	170607	-4523	9106 AN1372
1152	CONTIG18	165841	166326	1.79	4.13E-03	transcription_start_site	+	170844	170844	-4760	9107 AN1372
3059	CONTIG19	10146	10415	0.71	1.93E-01	transcription_start_site	+	9516	9516	764	9116 AN1375
3059	CONTIG19	10146	10415	0.71	1.93E-01	transcription_start_site	+	9287	9287	993	9115 AN1375
3059	CONTIG19	10146	10415	0.71	1.93E-01	transcription_start_site	+	12698	12698	-2417	9122 AN1377
3059	CONTIG19	10146	10415	0.71	1.93E-01	transcription_start_site	+	15392	15392	-5111	9123 AN1377
2396	CONTIG19	18454	19108	1.23	4.81E-03	transcription_start_site	-	18067	18067	-714	9124 AN1378
2396	CONTIG19	18454	19108	1.23	4.81E-03	transcription_start_site	-	17821	17821	-960	9125 AN1378
2396	CONTIG19	18454	19108	1.23	4.81E-03	transcription_start_site	-	17723	17723	-1058	9126 AN1378
2396	CONTIG19	18454	19108	1.23	4.81E-03	transcription_start_site	-	17480	17480	-1301	9127 AN1378
2396	CONTIG19	18454	19108	1.23	4.81E-03	transcription_start_site	+	19747	19747	-966	9128 AN1379
2396	CONTIG19	18454	19108	1.23	4.81E-03	transcription_start_site	+	20073	20073	-1292	9129 AN1379
2396	CONTIG19	18454	19108	1.23	4.81E-03	transcription_start_site	+	23627	23627	-4846	9132 AN1381
3039	CONTIG19	29484	29762	0.8	1.25E-01	transcription_start_site	-	29273	29273	-350	9135 AN1383
3039	CONTIG19	29484	29762	0.8	1.25E-01	transcription_start_site	-	29176	29176	-447	9136 AN1383
3039	CONTIG19	29484	29762	0.8	1.25E-01	transcription_start_site	-	29029	29029	-594	9137 AN1383
1031	CONTIG19	66538	66807	1.88	0.00E+00	transcription_start_site	-	66140	66140	-532	9167 AN1396
1031	CONTIG19	66538	66807	1.88	0.00E+00	transcription_start_site	-	65633	65633	-1039	9168 AN1396
1031	CONTIG19	66538	66807	1.88	0.00E+00	transcription_start_site	-	65321	65321	-1351	9169 AN1396
1031	CONTIG19	66538	66807	1.88	0.00E+00	transcription_start_site	-	63939	63939	-2733	9170 AN1396
1031	CONTIG19	66538	66807	1.88	0.00E+00	transcription_start_site	+	68186	68186	-1513	9171 AN1397
1031	CONTIG19	66538	66807	1.88	0.00E+00	transcription_start_site	+	68626	68626	-1953	9172 AN1397
1031	CONTIG19	66538	66807	1.88	0.00E+00	transcription_start_site	+	69450	69450	-2777	9173 AN1397

1031	CONTIG19	66538	66807	1.88	0.00E+00	transcription_start_site	+	69654	69654	-2981	9174	AN1397
1031	CONTIG19	66538	66807	1.88	0.00E+00	transcription_start_site	+	69997	69997	-3324	9175	AN1397
1031	CONTIG19	66538	66807	1.88	0.00E+00	transcription_start_site	+	70207	70207	-3534	9176	AN1397
1032	CONTIG20	696	1030	1.88	5.64E-04	transcription_start_site	+	4057	4057	-3194	9180	AN1399
1032	CONTIG20	696	1030	1.88	5.64E-04	transcription_start_site	+	4165	4165	-3302	9181	AN1399
1032	CONTIG20	696	1030	1.88	5.64E-04	transcription_start_site	+	4660	4660	-3797	9182	AN1399
1032	CONTIG20	696	1030	1.88	5.64E-04	transcription_start_site	+	5287	5287	-4424	9183	AN1399
1032	CONTIG20	696	1030	1.88	5.64E-04	transcription_start_site	+	5725	5725	-4862	9184	AN1399
2099	CONTIG20	12751	13040	1.33	1.57E-02	transcription_start_site	-	11577	11577	-1318	9187	AN1401
2099	CONTIG20	12751	13040	1.33	1.57E-02	transcription_start_site	-	11444	11444	-1451	9188	AN1401
2099	CONTIG20	12751	13040	1.33	1.57E-02	transcription_start_site	-	11352	11352	-1543	9189	AN1401
2099	CONTIG20	12751	13040	1.33	1.57E-02	transcription_start_site	-	11232	11232	-1663	9190	AN1401
2099	CONTIG20	12751	13040	1.33	1.57E-02	transcription_start_site	-	11103	11103	-1792	9191	AN1401
2099	CONTIG20	12751	13040	1.33	1.57E-02	transcription_start_site	-	10332	10332	-2563	9192	AN1401
2099	CONTIG20	12751	13040	1.33	1.57E-02	transcription_start_site	-	9508	9508	-3387	9193	AN1401
2099	CONTIG20	12751	13040	1.33	1.57E-02	transcription_start_site	-	8990	8990	-3905	9194	AN1401
2099	CONTIG20	12751	13040	1.33	1.57E-02	transcription_start_site	-	8683	8683	-4212	9195	AN1401
2099	CONTIG20	12751	13040	1.33	1.57E-02	transcription_start_site	-	8246	8246	-4649	9196	AN1401
2099	CONTIG20	12751	13040	1.33	1.57E-02	transcription_start_site	-	8019	8019	-4876	9197	AN1401
2099	CONTIG20	12751	13040	1.33	1.57E-02	transcription_start_site	+	13966	13966	-1070	9198	AN1402
2099	CONTIG20	12751	13040	1.33	1.57E-02	transcription_start_site	+	14899	14899	-2003	9199	AN1402
804	CONTIG22	9321	9576	2.1	1.11E-02	transcription_start_site	-	7876	7876	-1572	9205	AN1406
804	CONTIG22	9321	9576	2.1	1.11E-02	transcription_start_site	-	7774	7774	-1674	9206	AN1406
804	CONTIG22	9321	9576	2.1	1.11E-02	transcription_start_site	-	7743	7743	-1705	9207	AN1406
804	CONTIG22	9321	9576	2.1	1.11E-02	transcription_start_site	-	7646	7646	-1802	9208	AN1406
804	CONTIG22	9321	9576	2.1	1.11E-02	transcription_start_site	-	7078	7078	-2370	9209	AN1406
804	CONTIG22	9321	9576	2.1	1.11E-02	transcription_start_site	-	6689	6689	-2759	9210	AN1406
804	CONTIG22	9321	9576	2.1	1.11E-02	transcription_start_site	-	6361	6361	-3087	9211	AN1406
804	CONTIG22	9321	9576	2.1	1.11E-02	transcription_start_site	-	5761	5761	-3687	9212	AN1406
804	CONTIG22	9321	9576	2.1	1.11E-02	transcription_start_site	-	5259	5259	-4189	9213	AN1406
804	CONTIG22	9321	9576	2.1	1.11E-02	transcription_start_site	+	9798	9798	-349	9214	AN1407
804	CONTIG22	9321	9576	2.1	1.11E-02	transcription_start_site	+	10078	10078	-629	9215	AN1407
804	CONTIG22	9321	9576	2.1	1.11E-02	transcription_start_site	+	14139	14139	-4690	9220	AN1409
804	CONTIG22	9321	9576	2.1	1.11E-02	transcription_start_site	+	14266	14266	-4817	9221	AN1409
804	CONTIG22	9321	9576	2.1	1.11E-02	transcription_start_site	+	14372	14372	-4923	9222	AN1409
804	CONTIG22	9321	9576	2.1	1.11E-02	transcription_start_site	+	14498	14498	-5049	9223	AN1409
33	CONTIG22	31065	31859	3.52	0.00E+00	transcription_start_site	-	30335	30335	-1127	9240	AN1414
33	CONTIG22	31065	31859	3.52	0.00E+00	transcription_start_site	-	30131	30131	-1331	9241	AN1414
33	CONTIG22	31065	31859	3.52	0.00E+00	transcription_start_site	-	28529	28529	-2933	9242	AN1414
139	CONTIG22	32120	32904	3.08	0.00E+00	transcription_start_site	-	30335	30335	-2177	9240	AN1414
139	CONTIG22	32120	32904	3.08	0.00E+00	transcription_start_site	-	30131	30131	-2381	9241	AN1414
139	CONTIG22	32120	32904	3.08	0.00E+00	transcription_start_site	-	28529	28529	-3983	9242	AN1414
33	CONTIG22	31065	31859	3.52	0.00E+00	transcription_start_site	+	32578	32578	-1116	9243	AN1415
33	CONTIG22	31065	31859	3.52	0.00E+00	transcription_start_site	+	33024	33024	-1562	9244	AN1415
139	CONTIG22	32120	32904	3.08	0.00E+00	transcription_start_site	+	32578	32578	-66	9243	AN1415
139	CONTIG22	32120	32904	3.08	0.00E+00	transcription_start_site	+	33024	33024	-512	9244	AN1415
139	CONTIG22	32120	32904	3.08	0.00E+00	transcription_start_site	-	33851	33851	1339	9248	AN1416
283	CONTIG22	40145	40644	2.79	0.00E+00	transcription_start_site	-	36720	36720	-3674	9245	AN1416
283	CONTIG22	40145	40644	2.79	0.00E+00	transcription_start_site	-	36552	36552	-3842	9246	AN1416
283	CONTIG22	40145	40644	2.79	0.00E+00	transcription_start_site	-	36385	36385	-4009	9247	AN1416
1465	CONTIG22	36990	37344	1.61	5.88E-02	transcription_start_site	-	36720	36720	-447	9245	AN1416
1465	CONTIG22	36990	37344	1.61	5.88E-02	transcription_start_site	-	36552	36552	-615	9246	AN1416
1465	CONTIG22	36990	37344	1.61	5.88E-02	transcription_start_site	-	36385	36385	-782	9247	AN1416
1465	CONTIG22	36990	37344	1.61	5.88E-02	transcription_start_site	-	33851	33851	-3316	9248	AN1416
283	CONTIG22	40145	40644	2.79	0.00E+00	transcription_start_site	-	40615	40615	220	9249	AN1417
283	CONTIG22	40145	40644	2.79	0.00E+00	transcription_start_site	-	40167	40167	-227	9250	AN1417
283	CONTIG22	40145	40644	2.79	0.00E+00	transcription_start_site	-	40043	40043	-351	9251	AN1417

283	CONTIG22	40145	40644	2.79	0.00E+00	transcription_start_site	-	39898	39898	-496	9252 AN1417
283	CONTIG22	40145	40644	2.79	0.00E+00	transcription_start_site	-	39776	39776	-618	9253 AN1417
283	CONTIG22	40145	40644	2.79	0.00E+00	transcription_start_site	-	39566	39566	-828	9254 AN1417
1061	CONTIG22	42835	43123	1.86	2.62E-02	transcription_start_site	-	40615	40615	-2364	9249 AN1417
1061	CONTIG22	42835	43123	1.86	2.62E-02	transcription_start_site	-	40167	40167	-2812	9250 AN1417
1061	CONTIG22	42835	43123	1.86	2.62E-02	transcription_start_site	-	40043	40043	-2936	9251 AN1417
1061	CONTIG22	42835	43123	1.86	2.62E-02	transcription_start_site	-	39898	39898	-3081	9252 AN1417
1061	CONTIG22	42835	43123	1.86	2.62E-02	transcription_start_site	-	39776	39776	-3203	9253 AN1417
1061	CONTIG22	42835	43123	1.86	2.62E-02	transcription_start_site	-	39566	39566	-3413	9254 AN1417
283	CONTIG22	40145	40644	2.79	0.00E+00	transcription_start_site	+	40775	40775	-380	9255 AN1418
283	CONTIG22	40145	40644	2.79	0.00E+00	transcription_start_site	+	40830	40830	-435	9256 AN1418
283	CONTIG22	40145	40644	2.79	0.00E+00	transcription_start_site	+	40957	40957	-562	9257 AN1418
283	CONTIG22	40145	40644	2.79	0.00E+00	transcription_start_site	+	41154	41154	-759	9258 AN1418
1465	CONTIG22	36990	37344	1.61	5.88E-02	transcription_start_site	+	40775	40775	-3608	9255 AN1418
1465	CONTIG22	36990	37344	1.61	5.88E-02	transcription_start_site	+	40830	40830	-3663	9256 AN1418
1465	CONTIG22	36990	37344	1.61	5.88E-02	transcription_start_site	+	40957	40957	-3790	9257 AN1418
1465	CONTIG22	36990	37344	1.61	5.88E-02	transcription_start_site	+	41154	41154	-3987	9258 AN1418
283	CONTIG22	40145	40644	2.79	0.00E+00	transcription_start_site	+	43443	43443	-3048	9259 AN1419
283	CONTIG22	40145	40644	2.79	0.00E+00	transcription_start_site	+	43714	43714	-3319	9260 AN1419
283	CONTIG22	40145	40644	2.79	0.00E+00	transcription_start_site	+	44144	44144	-3749	9261 AN1419
283	CONTIG22	40145	40644	2.79	0.00E+00	transcription_start_site	+	44430	44430	-4035	9262 AN1419
1061	CONTIG22	42835	43123	1.86	2.62E-02	transcription_start_site	+	43443	43443	-464	9259 AN1419
1061	CONTIG22	42835	43123	1.86	2.62E-02	transcription_start_site	+	43714	43714	-735	9260 AN1419
1061	CONTIG22	42835	43123	1.86	2.62E-02	transcription_start_site	+	44144	44144	-1165	9261 AN1419
1061	CONTIG22	42835	43123	1.86	2.62E-02	transcription_start_site	+	44430	44430	-1451	9262 AN1419
1004	CONTIG22	52296	52570	1.91	2.12E-02	transcription_start_site	-	47928	47928	-4505	9263 AN1420
1004	CONTIG22	52296	52570	1.91	2.12E-02	transcription_start_site	-	47474	47474	-4959	9264 AN1420
1004	CONTIG22	52296	52570	1.91	2.12E-02	transcription_start_site	+	51744	51744	689	9269 AN1422
1004	CONTIG22	52296	52570	1.91	2.12E-02	transcription_start_site	+	51577	51577	856	9268 AN1422
1004	CONTIG22	52296	52570	1.91	2.12E-02	transcription_start_site	+	53009	53009	-576	9270 AN1423
1004	CONTIG22	52296	52570	1.91	2.12E-02	transcription_start_site	+	53420	53420	-987	9271 AN1423
1004	CONTIG22	52296	52570	1.91	2.12E-02	transcription_start_site	+	53487	53487	-1054	9272 AN1423
1004	CONTIG22	52296	52570	1.91	2.12E-02	transcription_start_site	+	55246	55246	-2813	9273 AN1424
84	CONTIG22	60919	61343	3.28	0.00E+00	transcription_start_site	+	61870	61870	-739	9274 AN1425
84	CONTIG22	60919	61343	3.28	0.00E+00	transcription_start_site	+	61995	61995	-864	9275 AN1425
84	CONTIG22	60919	61343	3.28	0.00E+00	transcription_start_site	+	62247	62247	-1116	9276 AN1425
410	CONTIG22	58821	59093	2.59	1.54E-03	transcription_start_site	+	61870	61870	-2913	9274 AN1425
410	CONTIG22	58821	59093	2.59	1.54E-03	transcription_start_site	+	61995	61995	-3038	9275 AN1425
410	CONTIG22	58821	59093	2.59	1.54E-03	transcription_start_site	+	62247	62247	-3290	9276 AN1425
1652	CONTIG22	57231	57515	1.52	8.01E-02	transcription_start_site	+	61870	61870	-4497	9274 AN1425
1652	CONTIG22	57231	57515	1.52	8.01E-02	transcription_start_site	+	61995	61995	-4622	9275 AN1425
1652	CONTIG22	57231	57515	1.52	8.01E-02	transcription_start_site	+	62247	62247	-4874	9276 AN1425
84	CONTIG22	60919	61343	3.28	0.00E+00	transcription_start_site	+	65376	65376	-4245	9277 AN1426
84	CONTIG22	60919	61343	3.28	0.00E+00	transcription_start_site	+	65784	65784	-4653	9278 AN1426
84	CONTIG22	60919	61343	3.28	0.00E+00	transcription_start_site	+	65941	65941	-4810	9279 AN1426
84	CONTIG22	60919	61343	3.28	0.00E+00	transcription_start_site	+	66111	66111	-4980	9280 AN1426
51	CONTIG22	70653	71227	3.42	0.00E+00	transcription_start_site	-	69542	69542	-1398	9285 AN1427
51	CONTIG22	70653	71227	3.42	0.00E+00	transcription_start_site	-	68909	68909	-2031	9286 AN1427
51	CONTIG22	70653	71227	3.42	0.00E+00	transcription_start_site	-	68243	68243	-2697	9287 AN1427
51	CONTIG22	70653	71227	3.42	0.00E+00	transcription_start_site	-	68094	68094	-2846	9288 AN1427
1537	CONTIG22	71933	74082	1.57	7.89E-02	transcription_start_site	-	69542	69542	-3465	9285 AN1427
1537	CONTIG22	71933	74082	1.57	7.89E-02	transcription_start_site	-	68909	68909	-4098	9286 AN1427
1537	CONTIG22	71933	74082	1.57	7.89E-02	transcription_start_site	-	68243	68243	-4764	9287 AN1427
1537	CONTIG22	71933	74082	1.57	7.89E-02	transcription_start_site	-	68094	68094	-4913	9288 AN1427
51	CONTIG22	70653	71227	3.42	0.00E+00	transcription_start_site	+	71217	71217	-277	9289 AN1428
1537	CONTIG22	71933	74082	1.57	7.89E-02	transcription_start_site	+	71217	71217	1790	9289 AN1428
694	CONTIG22	74568	75582	2.2	3.31E-03	transcription_start_site	-	74533	74533	-542	9290 AN1429

694	CONTIG22	74568	75582	2.2	3.31E-03	transcription_start_site	-	74234	74234	-841	9291 AN1429
805	CONTIG22	78528	78817	2.1	1.11E-02	transcription_start_site	-	74533	74533	-4139	9290 AN1429
805	CONTIG22	78528	78817	2.1	1.11E-02	transcription_start_site	-	74234	74234	-4438	9291 AN1429
1537	CONTIG22	71933	74082	1.57	7.89E-02	transcription_start_site	-	74234	74234	1226	9291 AN1429
1537	CONTIG22	71933	74082	1.57	7.89E-02	transcription_start_site	-	74533	74533	1525	9290 AN1429
51	CONTIG22	70653	71227	3.42	0.00E+00	transcription_start_site	+	75595	75595	-4655	9292 AN1430
51	CONTIG22	70653	71227	3.42	0.00E+00	transcription_start_site	+	76144	76144	-5204	9293 AN1430
694	CONTIG22	74568	75582	2.2	3.31E-03	transcription_start_site	+	75595	75595	-520	9292 AN1430
694	CONTIG22	74568	75582	2.2	3.31E-03	transcription_start_site	+	76144	76144	-1069	9293 AN1430
1537	CONTIG22	71933	74082	1.57	7.89E-02	transcription_start_site	+	75595	75595	-2587	9292 AN1430
1537	CONTIG22	71933	74082	1.57	7.89E-02	transcription_start_site	+	76144	76144	-3136	9293 AN1430
694	CONTIG22	74568	75582	2.2	3.31E-03	transcription_start_site	+	78413	78413	-3338	9294 AN1431
694	CONTIG22	74568	75582	2.2	3.31E-03	transcription_start_site	+	79295	79295	-4220	9295 AN1431
805	CONTIG22	78528	78817	2.1	1.11E-02	transcription_start_site	+	78413	78413	259	9294 AN1431
805	CONTIG22	78528	78817	2.1	1.11E-02	transcription_start_site	+	79295	79295	-622	9295 AN1431
1537	CONTIG22	71933	74082	1.57	7.89E-02	transcription_start_site	+	78413	78413	-5405	9294 AN1431
805	CONTIG22	78528	78817	2.1	1.11E-02	transcription_start_site	+	81902	81902	-3229	9298 AN1433
805	CONTIG22	78528	78817	2.1	1.11E-02	transcription_start_site	+	82169	82169	-3496	9299 AN1433
805	CONTIG22	78528	78817	2.1	1.11E-02	transcription_start_site	+	82578	82578	-3905	9300 AN1433
2451	CONTIG22	103284	103568	1.22	1.93E-01	transcription_start_site	-	102965	102965	-461	9310 AN1439
2451	CONTIG22	103284	103568	1.22	1.93E-01	transcription_start_site	-	102764	102764	-662	9311 AN1439
2451	CONTIG22	103284	103568	1.22	1.93E-01	transcription_start_site	-	102584	102584	-842	9312 AN1439
1886	CONTIG22	108529	108878	1.42	1.08E-01	transcription_start_site	+	113852	113852	-5148	9313 AN1442
2452	CONTIG22	118144	118488	1.22	1.93E-01	transcription_start_site	-	116421	116421	-1895	9317 AN1443
2452	CONTIG22	118144	118488	1.22	1.93E-01	transcription_start_site	+	121003	121003	-2687	9318 AN1444
2452	CONTIG22	118144	118488	1.22	1.93E-01	transcription_start_site	+	121197	121197	-2881	9319 AN1444
2452	CONTIG22	118144	118488	1.22	1.93E-01	transcription_start_site	+	121500	121500	-3184	9320 AN1444
2298	CONTIG22	127218	127477	1.27	1.66E-01	transcription_start_site	-	123256	123256	-4091	9321 AN1445
2298	CONTIG22	127218	127477	1.27	1.66E-01	transcription_start_site	-	122994	122994	-4353	9322 AN1445
2298	CONTIG22	127218	127477	1.27	1.66E-01	transcription_start_site	-	122562	122562	-4785	9323 AN1445
754	CONTIG22	129393	131087	2.15	8.81E-03	transcription_start_site	-	125192	125192	-5048	9324 AN1446
754	CONTIG22	129393	131087	2.15	8.81E-03	transcription_start_site	-	124991	124991	-5249	9325 AN1446
754	CONTIG22	129393	131087	2.15	8.81E-03	transcription_start_site	-	124853	124853	-5387	9326 AN1446
2298	CONTIG22	127218	127477	1.27	1.66E-01	transcription_start_site	-	125192	125192	-2155	9324 AN1446
2298	CONTIG22	127218	127477	1.27	1.66E-01	transcription_start_site	-	124991	124991	-2356	9325 AN1446
2298	CONTIG22	127218	127477	1.27	1.66E-01	transcription_start_site	-	124853	124853	-2494	9326 AN1446
2298	CONTIG22	127218	127477	1.27	1.66E-01	transcription_start_site	-	124354	124354	-2993	9327 AN1446
754	CONTIG22	129393	131087	2.15	8.81E-03	transcription_start_site	-	126913	126913	-3327	9328 AN1447
754	CONTIG22	129393	131087	2.15	8.81E-03	transcription_start_site	-	126846	126846	-3394	9329 AN1447
754	CONTIG22	129393	131087	2.15	8.81E-03	transcription_start_site	-	126674	126674	-3566	9330 AN1447
754	CONTIG22	129393	131087	2.15	8.81E-03	transcription_start_site	-	126513	126513	-3727	9331 AN1447
754	CONTIG22	129393	131087	2.15	8.81E-03	transcription_start_site	-	126374	126374	-3866	9332 AN1447
754	CONTIG22	129393	131087	2.15	8.81E-03	transcription_start_site	-	126264	126264	-3976	9333 AN1447
754	CONTIG22	129393	131087	2.15	8.81E-03	transcription_start_site	-	125865	125865	-4375	9334 AN1447
754	CONTIG22	129393	131087	2.15	8.81E-03	transcription_start_site	-	125695	125695	-4545	9335 AN1447
2298	CONTIG22	127218	127477	1.27	1.66E-01	transcription_start_site	-	126913	126913	-434	9328 AN1447
2298	CONTIG22	127218	127477	1.27	1.66E-01	transcription_start_site	-	126846	126846	-501	9329 AN1447
2298	CONTIG22	127218	127477	1.27	1.66E-01	transcription_start_site	-	126674	126674	-673	9330 AN1447
2298	CONTIG22	127218	127477	1.27	1.66E-01	transcription_start_site	-	126513	126513	-834	9331 AN1447
2298	CONTIG22	127218	127477	1.27	1.66E-01	transcription_start_site	-	126374	126374	-973	9332 AN1447
2298	CONTIG22	127218	127477	1.27	1.66E-01	transcription_start_site	-	126264	126264	-1083	9333 AN1447
2298	CONTIG22	127218	127477	1.27	1.66E-01	transcription_start_site	-	125865	125865	-1482	9334 AN1447
2298	CONTIG22	127218	127477	1.27	1.66E-01	transcription_start_site	-	125695	125695	-1652	9335 AN1447
754	CONTIG22	129393	131087	2.15	8.81E-03	transcription_start_site	+	129138	129138	1102	9337 AN1448
2298	CONTIG22	127218	127477	1.27	1.66E-01	transcription_start_site	+	127352	127352	-4	9336 AN1448
2298	CONTIG22	127218	127477	1.27	1.66E-01	transcription_start_site	+	129138	129138	-1790	9337 AN1448
754	CONTIG22	129393	131087	2.15	8.81E-03	transcription_start_site	+	131291	131291	-1051	9338 AN1449

754	CONTIG22	129393	131087	2.15	8.81E-03	transcription_start_site	+	131557	131557	-1317	9339	AN1449
2298	CONTIG22	127218	127477	1.27	1.66E-01	transcription_start_site	+	131291	131291	-3943	9338	AN1449
2298	CONTIG22	127218	127477	1.27	1.66E-01	transcription_start_site	+	131557	131557	-4209	9339	AN1449
2299	CONTIG22	135153	135437	1.27	1.66E-01	transcription_start_site	-	134466	134466	-829	9340	AN1450
2299	CONTIG22	135153	135437	1.27	1.66E-01	transcription_start_site	-	134243	134243	-1052	9341	AN1450
2299	CONTIG22	135153	135437	1.27	1.66E-01	transcription_start_site	+	140310	140310	-5015	9346	AN1453
1359	CONTIG22	159464	159738	1.66	5.22E-02	transcription_start_site	-	158275	158275	-1326	9368	AN1457
1359	CONTIG22	159464	159738	1.66	5.22E-02	transcription_start_site	-	157217	157217	-2384	9369	AN1457
1359	CONTIG22	159464	159738	1.66	5.22E-02	transcription_start_site	-	156704	156704	-2897	9370	AN1457
1359	CONTIG22	159464	159738	1.66	5.22E-02	transcription_start_site	-	156563	156563	-3038	9371	AN1457
1359	CONTIG22	159464	159738	1.66	5.22E-02	transcription_start_site	-	156464	156464	-3137	9372	AN1457
1359	CONTIG22	159464	159738	1.66	5.22E-02	transcription_start_site	+	160296	160296	-695	9373	AN1458
1359	CONTIG22	159464	159738	1.66	5.22E-02	transcription_start_site	+	161887	161887	-2286	9374	AN1459
1359	CONTIG22	159464	159738	1.66	5.22E-02	transcription_start_site	+	162171	162171	-2570	9375	AN1459
1359	CONTIG22	159464	159738	1.66	5.22E-02	transcription_start_site	+	163865	163865	-4264	9376	AN1459
1002	CONTIG22	171679	172418	1.91	1.06E-02	transcription_start_site	+	172569	172569	-520	9386	AN1462
1002	CONTIG22	171679	172418	1.91	1.06E-02	transcription_start_site	+	170737	170737	1311	9385	AN1462
1002	CONTIG22	171679	172418	1.91	1.06E-02	transcription_start_site	+	177091	177091	-5042	9387	AN1463
1002	CONTIG22	171679	172418	1.91	1.06E-02	transcription_start_site	+	177201	177201	-5152	9388	AN1463
1466	CONTIG22	179253	179542	1.61	5.88E-02	transcription_start_site	+	178280	178280	1117	9392	AN1463
1887	CONTIG22	174304	174651	1.42	1.08E-01	transcription_start_site	+	177091	177091	-2613	9387	AN1463
1887	CONTIG22	174304	174651	1.42	1.08E-01	transcription_start_site	+	177201	177201	-2723	9388	AN1463
1887	CONTIG22	174304	174651	1.42	1.08E-01	transcription_start_site	+	177634	177634	-3156	9389	AN1463
1887	CONTIG22	174304	174651	1.42	1.08E-01	transcription_start_site	+	177844	177844	-3366	9390	AN1463
1887	CONTIG22	174304	174651	1.42	1.08E-01	transcription_start_site	+	178075	178075	-3597	9391	AN1463
1887	CONTIG22	174304	174651	1.42	1.08E-01	transcription_start_site	+	178280	178280	-3802	9392	AN1463
316	CONTIG22	183468	183805	2.74	9.51E-04	transcription_start_site	+	184604	184604	-967	9393	AN1465
316	CONTIG22	183468	183805	2.74	9.51E-04	transcription_start_site	+	185073	185073	-1436	9394	AN1465
316	CONTIG22	183468	183805	2.74	9.51E-04	transcription_start_site	+	185659	185659	-2022	9395	AN1465
316	CONTIG22	183468	183805	2.74	9.51E-04	transcription_start_site	+	187696	187696	-4059	9396	AN1466
316	CONTIG22	183468	183805	2.74	9.51E-04	transcription_start_site	+	187980	187980	-4343	9397	AN1466
316	CONTIG22	183468	183805	2.74	9.51E-04	transcription_start_site	+	188521	188521	-4884	9398	AN1466
2143	CONTIG22	196221	196712	1.32	1.28E-01	transcription_start_site	-	192839	192839	-3627	9401	AN1467
2143	CONTIG22	196221	196712	1.32	1.28E-01	transcription_start_site	+	195806	195806	660	9405	AN1469
2143	CONTIG22	196221	196712	1.32	1.28E-01	transcription_start_site	+	198096	198096	-1629	9406	AN1470
2143	CONTIG22	196221	196712	1.32	1.28E-01	transcription_start_site	+	198302	198302	-1835	9407	AN1470
530	CONTIG22	201301	201650	2.4	4.46E-04	transcription_start_site	-	201617	201617	141	9408	AN1471
530	CONTIG22	201301	201650	2.4	4.46E-04	transcription_start_site	-	201057	201057	-418	9409	AN1471
530	CONTIG22	201301	201650	2.4	4.46E-04	transcription_start_site	+	203772	203772	-2296	9410	AN1472
1532	CONTIG22	212483	213502	1.57	6.84E-02	transcription_start_site	-	209367	209367	-3625	9414	AN1474
1532	CONTIG22	212483	213502	1.57	6.84E-02	transcription_start_site	-	212846	212846	-146	9415	AN1475
2300	CONTIG22	217808	218087	1.27	1.66E-01	transcription_start_site	-	212846	212846	-5101	9415	AN1475
2016	CONTIG22	220430	220709	1.37	1.25E-01	transcription_start_site	-	220083	220083	-486	9417	AN1477
2016	CONTIG22	220430	220709	1.37	1.25E-01	transcription_start_site	-	221184	221184	614	9416	AN1477
1471	CONTIG23	5251	5525	1.61	8.01E-02	transcription_start_site	+	5252	5252	136	9464	AN1494
1471	CONTIG23	5251	5525	1.61	8.01E-02	transcription_start_site	+	4396	4396	992	9463	AN1494
1471	CONTIG23	5251	5525	1.61	8.01E-02	transcription_start_site	-	6301	6301	913	9469	AN1495
1471	CONTIG23	5251	5525	1.61	8.01E-02	transcription_start_site	-	6427	6427	1039	9468	AN1495
991	CONTIG23	23333	23827	1.92	3.08E-02	transcription_start_site	-	18948	18948	-4632	9476	AN1499
991	CONTIG23	23333	23827	1.92	3.08E-02	transcription_start_site	-	18843	18843	-4737	9477	AN1499
991	CONTIG23	23333	23827	1.92	3.08E-02	transcription_start_site	-	18704	18704	-4876	9478	AN1499
131	CONTIG23	24243	24653	3.11	2.16E-04	transcription_start_site	-	21224	21224	-3224	9480	AN1500
131	CONTIG23	24243	24653	3.11	2.16E-04	transcription_start_site	-	20733	20733	-3715	9481	AN1500
991	CONTIG23	23333	23827	1.92	3.08E-02	transcription_start_site	-	21224	21224	-2356	9480	AN1500
991	CONTIG23	23333	23827	1.92	3.08E-02	transcription_start_site	-	20733	20733	-2847	9481	AN1500
1279	CONTIG23	28671	29020	1.71	3.99E-02	transcription_start_site	-	27895	27895	-950	9482	AN1501
1279	CONTIG23	28671	29020	1.71	3.99E-02	transcription_start_site	-	27385	27385	-1460	9483	AN1501

1279	CONTIG23	28671	29020	1.71	3.99E-02	transcription_start_site	-	27167	27167	-1678	9484 AN1501
1279	CONTIG23	28671	29020	1.71	3.99E-02	transcription_start_site	-	26661	26661	-2184	9485 AN1501
1279	CONTIG23	28671	29020	1.71	3.99E-02	transcription_start_site	-	26097	26097	-2748	9486 AN1501
280	CONTIG23	34446	34720	2.8	1.22E-03	transcription_start_site	-	33842	33842	-741	9487 AN1502
992	CONTIG23	35556	36070	1.92	3.08E-02	transcription_start_site	-	33842	33842	-1971	9487 AN1502
992	CONTIG23	35556	36070	1.92	3.08E-02	transcription_start_site	-	36246	36246	433	9488 AN1503
1565	CONTIG23	38927	39358	1.56	7.51E-02	transcription_start_site	-	36246	36246	-2896	9488 AN1503
280	CONTIG23	34446	34720	2.8	1.22E-03	transcription_start_site	+	37970	37970	-3387	9489 AN1504
280	CONTIG23	34446	34720	2.8	1.22E-03	transcription_start_site	+	38778	38778	-4195	9490 AN1504
992	CONTIG23	35556	36070	1.92	3.08E-02	transcription_start_site	+	37970	37970	-2157	9489 AN1504
992	CONTIG23	35556	36070	1.92	3.08E-02	transcription_start_site	+	38778	38778	-2965	9490 AN1504
992	CONTIG23	35556	36070	1.92	3.08E-02	transcription_start_site	+	39731	39731	-3918	9491 AN1504
1565	CONTIG23	38927	39358	1.56	7.51E-02	transcription_start_site	+	38778	38778	364	9490 AN1504
1565	CONTIG23	38927	39358	1.56	7.51E-02	transcription_start_site	+	39731	39731	-588	9491 AN1504
1565	CONTIG23	38927	39358	1.56	7.51E-02	transcription_start_site	+	37970	37970	1172	9489 AN1504
2067	CONTIG23	42919	43352	1.35	1.66E-01	transcription_start_site	-	42909	42909	-226	9492 AN1505
2067	CONTIG23	42919	43352	1.35	1.66E-01	transcription_start_site	-	42631	42631	-504	9493 AN1505
2067	CONTIG23	42919	43352	1.35	1.66E-01	transcription_start_site	-	42463	42463	-672	9494 AN1505
2067	CONTIG23	42919	43352	1.35	1.66E-01	transcription_start_site	-	42078	42078	-1057	9495 AN1505
2067	CONTIG23	42919	43352	1.35	1.66E-01	transcription_start_site	-	41879	41879	-1256	9496 AN1505
2067	CONTIG23	42919	43352	1.35	1.66E-01	transcription_start_site	-	41609	41609	-1526	9497 AN1505
1565	CONTIG23	38927	39358	1.56	7.51E-02	transcription_start_site	+	44167	44167	-5024	9498 AN1506
2067	CONTIG23	42919	43352	1.35	1.66E-01	transcription_start_site	+	44167	44167	-1031	9498 AN1506
2067	CONTIG23	42919	43352	1.35	1.66E-01	transcription_start_site	+	44396	44396	-1260	9499 AN1506
2067	CONTIG23	42919	43352	1.35	1.66E-01	transcription_start_site	+	45084	45084	-1948	9500 AN1506
2067	CONTIG23	42919	43352	1.35	1.66E-01	transcription_start_site	+	45360	45360	-2224	9501 AN1506
2067	CONTIG23	42919	43352	1.35	1.66E-01	transcription_start_site	+	45507	45507	-2371	9502 AN1506
882	CONTIG23	50327	50676	2.02	2.12E-02	transcription_start_site	-	46778	46778	-3723	9503 AN1507
882	CONTIG23	50327	50676	2.02	2.12E-02	transcription_start_site	-	46697	46697	-3804	9504 AN1507
993	CONTIG23	49667	49934	1.92	3.08E-02	transcription_start_site	-	46778	46778	-3022	9503 AN1507
993	CONTIG23	49667	49934	1.92	3.08E-02	transcription_start_site	-	46697	46697	-3103	9504 AN1507
882	CONTIG23	50327	50676	2.02	2.12E-02	transcription_start_site	-	48214	48214	-2287	9505 AN1508
882	CONTIG23	50327	50676	2.02	2.12E-02	transcription_start_site	-	47911	47911	-2590	9506 AN1508
882	CONTIG23	50327	50676	2.02	2.12E-02	transcription_start_site	-	47751	47751	-2750	9507 AN1508
882	CONTIG23	50327	50676	2.02	2.12E-02	transcription_start_site	-	47632	47632	-2869	9508 AN1508
993	CONTIG23	49667	49934	1.92	3.08E-02	transcription_start_site	-	48214	48214	-1586	9505 AN1508
993	CONTIG23	49667	49934	1.92	3.08E-02	transcription_start_site	-	47911	47911	-1889	9506 AN1508
993	CONTIG23	49667	49934	1.92	3.08E-02	transcription_start_site	-	47751	47751	-2049	9507 AN1508
993	CONTIG23	49667	49934	1.92	3.08E-02	transcription_start_site	-	47632	47632	-2168	9508 AN1508
882	CONTIG23	50327	50676	2.02	2.12E-02	transcription_start_site	+	50799	50799	-297	9509 AN1509
882	CONTIG23	50327	50676	2.02	2.12E-02	transcription_start_site	+	51434	51434	-932	9510 AN1509
993	CONTIG23	49667	49934	1.92	3.08E-02	transcription_start_site	+	50799	50799	-998	9509 AN1509
993	CONTIG23	49667	49934	1.92	3.08E-02	transcription_start_site	+	51434	51434	-1633	9510 AN1509
882	CONTIG23	50327	50676	2.02	2.12E-02	transcription_start_site	+	53235	53235	-2733	9511 AN1510
882	CONTIG23	50327	50676	2.02	2.12E-02	transcription_start_site	+	53399	53399	-2897	9512 AN1510
882	CONTIG23	50327	50676	2.02	2.12E-02	transcription_start_site	+	55028	55028	-4526	9513 AN1510
993	CONTIG23	49667	49934	1.92	3.08E-02	transcription_start_site	+	53235	53235	-3434	9511 AN1510
993	CONTIG23	49667	49934	1.92	3.08E-02	transcription_start_site	+	53399	53399	-3598	9512 AN1510
882	CONTIG23	50327	50676	2.02	2.12E-02	transcription_start_site	+	55568	55568	-5066	9514 AN1511
229	CONTIG24	16967	17226	2.88	0.00E+00	transcription_start_site	-	12431	12431	-4665	9525 AN1517
229	CONTIG24	16967	17226	2.88	0.00E+00	transcription_start_site	-	12367	12367	-4729	9526 AN1517
229	CONTIG24	16967	17226	2.88	0.00E+00	transcription_start_site	-	15908	15908	-1188	9528 AN1518
229	CONTIG24	16967	17226	2.88	0.00E+00	transcription_start_site	-	15731	15731	-1365	9529 AN1518
229	CONTIG24	16967	17226	2.88	0.00E+00	transcription_start_site	-	15265	15265	-1831	9530 AN1518
229	CONTIG24	16967	17226	2.88	0.00E+00	transcription_start_site	-	14987	14987	-2109	9531 AN1518
229	CONTIG24	16967	17226	2.88	0.00E+00	transcription_start_site	-	14902	14902	-2194	9532 AN1518
229	CONTIG24	16967	17226	2.88	0.00E+00	transcription_start_site	-	14079	14079	-3017	9533 AN1518

229	CONTIG24	16967	17226	2.88	0.00E+00	transcription_start_site	-	13315	13315	-3781	9534 AN1518
229	CONTIG24	16967	17226	2.88	0.00E+00	transcription_start_site	+	18263	18263	-1166	9535 AN1519
229	CONTIG24	16967	17226	2.88	0.00E+00	transcription_start_site	+	18574	18574	-1477	9536 AN1519
229	CONTIG24	16967	17226	2.88	0.00E+00	transcription_start_site	+	19727	19727	-2630	9537 AN1519
229	CONTIG24	16967	17226	2.88	0.00E+00	transcription_start_site	+	20027	20027	-2930	9538 AN1519
1685	CONTIG24	23043	24282	1.5	1.70E-02	transcription_start_site	-	23603	23603	-59	9540 AN1520
1685	CONTIG24	23043	24282	1.5	1.70E-02	transcription_start_site	-	23979	23979	316	9539 AN1520
1976	CONTIG24	27618	27967	1.38	5.88E-02	transcription_start_site	-	23979	23979	-3813	9539 AN1520
1976	CONTIG24	27618	27967	1.38	5.88E-02	transcription_start_site	-	23603	23603	-4189	9540 AN1520
1685	CONTIG24	23043	24282	1.5	1.70E-02	transcription_start_site	-	24969	24969	1306	9544 AN1521
1685	CONTIG24	23043	24282	1.5	1.70E-02	transcription_start_site	-	25201	25201	1538	9543 AN1521
1976	CONTIG24	27618	27967	1.38	5.88E-02	transcription_start_site	-	25640	25640	-2152	9541 AN1521
1976	CONTIG24	27618	27967	1.38	5.88E-02	transcription_start_site	-	25342	25342	-2450	9542 AN1521
1976	CONTIG24	27618	27967	1.38	5.88E-02	transcription_start_site	-	25201	25201	-2591	9543 AN1521
1976	CONTIG24	27618	27967	1.38	5.88E-02	transcription_start_site	-	24969	24969	-2823	9544 AN1521
1685	CONTIG24	23043	24282	1.5	1.70E-02	transcription_start_site	+	26411	26411	-2748	9545 AN1522
1685	CONTIG24	23043	24282	1.5	1.70E-02	transcription_start_site	+	26582	26582	-2919	9546 AN1522
1685	CONTIG24	23043	24282	1.5	1.70E-02	transcription_start_site	+	26705	26705	-3042	9547 AN1522
1976	CONTIG24	27618	27967	1.38	5.88E-02	transcription_start_site	+	26705	26705	1087	9547 AN1522
1685	CONTIG24	23043	24282	1.5	1.70E-02	transcription_start_site	+	28102	28102	-4439	9548 AN1523
1685	CONTIG24	23043	24282	1.5	1.70E-02	transcription_start_site	+	28353	28353	-4690	9549 AN1523
1685	CONTIG24	23043	24282	1.5	1.70E-02	transcription_start_site	+	28659	28659	-4996	9550 AN1523
1685	CONTIG24	23043	24282	1.5	1.70E-02	transcription_start_site	+	28772	28772	-5109	9551 AN1523
1685	CONTIG24	23043	24282	1.5	1.70E-02	transcription_start_site	+	28955	28955	-5292	9552 AN1523
1976	CONTIG24	27618	27967	1.38	5.88E-02	transcription_start_site	+	28102	28102	-309	9548 AN1523
1976	CONTIG24	27618	27967	1.38	5.88E-02	transcription_start_site	+	28353	28353	-560	9549 AN1523
1976	CONTIG24	27618	27967	1.38	5.88E-02	transcription_start_site	+	28659	28659	-866	9550 AN1523
1976	CONTIG24	27618	27967	1.38	5.88E-02	transcription_start_site	+	28772	28772	-979	9551 AN1523
1976	CONTIG24	27618	27967	1.38	5.88E-02	transcription_start_site	+	28955	28955	-1162	9552 AN1523
1976	CONTIG24	27618	27967	1.38	5.88E-02	transcription_start_site	+	31081	31081	-3288	9553 AN1524
1976	CONTIG24	27618	27967	1.38	5.88E-02	transcription_start_site	+	31437	31437	-3644	9554 AN1524
1976	CONTIG24	27618	27967	1.38	5.88E-02	transcription_start_site	+	32128	32128	-4335	9555 AN1524
1976	CONTIG24	27618	27967	1.38	5.88E-02	transcription_start_site	+	32272	32272	-4479	9556 AN1524
2505	CONTIG25	1966	2381	1.2	8.01E-02	transcription_start_site	-	1406	1406	-767	9577 AN1531
2505	CONTIG25	1966	2381	1.2	8.01E-02	transcription_start_site	-	1105	1105	-1068	9578 AN1531
2505	CONTIG25	1966	2381	1.2	8.01E-02	transcription_start_site	-	3035	3035	861	9580 AN1532
2505	CONTIG25	1966	2381	1.2	8.01E-02	transcription_start_site	-	3223	3223	1049	9579 AN1532
1168	CONTIG25	8866	9125	1.78	6.61E-03	transcription_start_site	-	7127	7127	-1868	9581 AN1533
1168	CONTIG25	8866	9125	1.78	6.61E-03	transcription_start_site	-	6668	6668	-2327	9582 AN1533
1168	CONTIG25	8866	9125	1.78	6.61E-03	transcription_start_site	-	6398	6398	-2597	9583 AN1533
1168	CONTIG25	8866	9125	1.78	6.61E-03	transcription_start_site	-	5200	5200	-3795	9584 AN1533
1168	CONTIG25	8866	9125	1.78	6.61E-03	transcription_start_site	+	8277	8277	718	9588 AN1534
1168	CONTIG25	8866	9125	1.78	6.61E-03	transcription_start_site	+	7996	7996	999	9587 AN1534
2505	CONTIG25	1966	2381	1.2	8.01E-02	transcription_start_site	+	7370	7370	-5196	9585 AN1534
1168	CONTIG25	8866	9125	1.78	6.61E-03	transcription_start_site	+	9237	9237	-241	9589 AN1535
1168	CONTIG25	8866	9125	1.78	6.61E-03	transcription_start_site	+	9842	9842	-846	9590 AN1535
1168	CONTIG25	8866	9125	1.78	6.61E-03	transcription_start_site	+	9952	9952	-956	9591 AN1535
1168	CONTIG25	8866	9125	1.78	6.61E-03	transcription_start_site	+	10112	10112	-1116	9592 AN1535
1168	CONTIG25	8866	9125	1.78	6.61E-03	transcription_start_site	+	10567	10567	-1571	9593 AN1535
1168	CONTIG25	8866	9125	1.78	6.61E-03	transcription_start_site	+	10978	10978	-1982	9594 AN1535
2825	CONTIG25	35180	35619	1.05	1.44E-01	transcription_start_site	+	38115	38115	-2715	9614 AN1543
2825	CONTIG25	35180	35619	1.05	1.44E-01	transcription_start_site	+	38218	38218	-2818	9615 AN1543
2825	CONTIG25	35180	35619	1.05	1.44E-01	transcription_start_site	+	38401	38401	-3001	9616 AN1543
2825	CONTIG25	35180	35619	1.05	1.44E-01	transcription_start_site	+	38696	38696	-3296	9617 AN1543
2825	CONTIG25	35180	35619	1.05	1.44E-01	transcription_start_site	+	40194	40194	-4794	9618 AN1543
1666	CONTIG25	42080	42429	1.51	1.06E-02	transcription_start_site	-	41714	41714	-540	9619 AN1544
2778	CONTIG25	45380	45654	1.08	1.25E-01	transcription_start_site	-	41714	41714	-3803	9619 AN1544

2826	CONTIG25	46065	46329	1.05	1.44E-01	transcription_start_site	-	41714	41714	-4483	9619	AN1544
1666	CONTIG25	42080	42429	1.51	1.06E-02	transcription_start_site	+	42956	42956	-701	9620	AN1545
1666	CONTIG25	42080	42429	1.51	1.06E-02	transcription_start_site	+	43170	43170	-915	9621	AN1545
1666	CONTIG25	42080	42429	1.51	1.06E-02	transcription_start_site	+	43276	43276	-1021	9622	AN1545
1666	CONTIG25	42080	42429	1.51	1.06E-02	transcription_start_site	+	43433	43433	-1178	9623	AN1545
1666	CONTIG25	42080	42429	1.51	1.06E-02	transcription_start_site	+	44148	44148	-1893	9624	AN1545
1666	CONTIG25	42080	42429	1.51	1.06E-02	transcription_start_site	+	44773	44773	-2518	9625	AN1545
2778	CONTIG25	45380	45654	1.08	1.25E-01	transcription_start_site	+	44773	44773	744	9625	AN1545
1666	CONTIG25	42080	42429	1.51	1.06E-02	transcription_start_site	+	46007	46007	-3752	9626	AN1546
1666	CONTIG25	42080	42429	1.51	1.06E-02	transcription_start_site	+	46178	46178	-3923	9627	AN1546
1666	CONTIG25	42080	42429	1.51	1.06E-02	transcription_start_site	+	46322	46322	-4067	9628	AN1546
1666	CONTIG25	42080	42429	1.51	1.06E-02	transcription_start_site	+	47026	47026	-4771	9629	AN1546
1666	CONTIG25	42080	42429	1.51	1.06E-02	transcription_start_site	+	47192	47192	-4937	9630	AN1546
2778	CONTIG25	45380	45654	1.08	1.25E-01	transcription_start_site	+	46007	46007	-490	9626	AN1546
2778	CONTIG25	45380	45654	1.08	1.25E-01	transcription_start_site	+	46178	46178	-661	9627	AN1546
2778	CONTIG25	45380	45654	1.08	1.25E-01	transcription_start_site	+	46322	46322	-805	9628	AN1546
2778	CONTIG25	45380	45654	1.08	1.25E-01	transcription_start_site	+	47026	47026	-1509	9629	AN1546
2778	CONTIG25	45380	45654	1.08	1.25E-01	transcription_start_site	+	47192	47192	-1675	9630	AN1546
2778	CONTIG25	45380	45654	1.08	1.25E-01	transcription_start_site	+	47946	47946	-2429	9631	AN1546
2778	CONTIG25	45380	45654	1.08	1.25E-01	transcription_start_site	+	48635	48635	-3118	9632	AN1546
2826	CONTIG25	46065	46329	1.05	1.44E-01	transcription_start_site	+	46178	46178	19	9627	AN1546
2826	CONTIG25	46065	46329	1.05	1.44E-01	transcription_start_site	+	46322	46322	-125	9628	AN1546
2826	CONTIG25	46065	46329	1.05	1.44E-01	transcription_start_site	+	46007	46007	190	9626	AN1546
2826	CONTIG25	46065	46329	1.05	1.44E-01	transcription_start_site	+	47026	47026	-829	9629	AN1546
2826	CONTIG25	46065	46329	1.05	1.44E-01	transcription_start_site	+	47192	47192	-995	9630	AN1546
2826	CONTIG25	46065	46329	1.05	1.44E-01	transcription_start_site	+	47946	47946	-1749	9631	AN1546
2826	CONTIG25	46065	46329	1.05	1.44E-01	transcription_start_site	+	48635	48635	-2438	9632	AN1546
2826	CONTIG25	46065	46329	1.05	1.44E-01	transcription_start_site	+	50784	50784	-4587	9633	AN1546
1395	CONTIG25	51977	52416	1.63	1.30E-02	transcription_start_site	+	52665	52665	-468	9634	AN1547
1395	CONTIG25	51977	52416	1.63	1.30E-02	transcription_start_site	+	52858	52858	-661	9635	AN1547
1395	CONTIG25	51977	52416	1.63	1.30E-02	transcription_start_site	+	53147	53147	-950	9636	AN1547
1931	CONTIG25	53417	53916	1.39	2.18E-02	transcription_start_site	+	53147	53147	519	9636	AN1547
1931	CONTIG25	53417	53916	1.39	2.18E-02	transcription_start_site	+	52858	52858	808	9635	AN1547
1931	CONTIG25	53417	53916	1.39	2.18E-02	transcription_start_site	+	52665	52665	1001	9634	AN1547
1395	CONTIG25	51977	52416	1.63	1.30E-02	transcription_start_site	+	55018	55018	-2821	9637	AN1548
1395	CONTIG25	51977	52416	1.63	1.30E-02	transcription_start_site	+	55406	55406	-3209	9638	AN1548
1931	CONTIG25	53417	53916	1.39	2.18E-02	transcription_start_site	+	55018	55018	-1351	9637	AN1548
1931	CONTIG25	53417	53916	1.39	2.18E-02	transcription_start_site	+	55406	55406	-1739	9638	AN1548
447	CONTIG25	63307	63662	2.52	0.00E+00	transcription_start_site	-	61347	61347	-2137	9642	AN1550
447	CONTIG25	63307	63662	2.52	0.00E+00	transcription_start_site	-	61133	61133	-2351	9643	AN1550
447	CONTIG25	63307	63662	2.52	0.00E+00	transcription_start_site	-	59697	59697	-3787	9644	AN1550
1169	CONTIG25	64578	65237	1.78	6.61E-03	transcription_start_site	-	61347	61347	-3560	9642	AN1550
1169	CONTIG25	64578	65237	1.78	6.61E-03	transcription_start_site	-	61133	61133	-3774	9643	AN1550
1169	CONTIG25	64578	65237	1.78	6.61E-03	transcription_start_site	-	59697	59697	-5210	9644	AN1550
447	CONTIG25	63307	63662	2.52	0.00E+00	transcription_start_site	-	63878	63878	393	9646	AN1551
447	CONTIG25	63307	63662	2.52	0.00E+00	transcription_start_site	-	64115	64115	630	9645	AN1551
1169	CONTIG25	64578	65237	1.78	6.61E-03	transcription_start_site	-	64115	64115	-792	9645	AN1551
1169	CONTIG25	64578	65237	1.78	6.61E-03	transcription_start_site	-	63878	63878	-1029	9646	AN1551
447	CONTIG25	63307	63662	2.52	0.00E+00	transcription_start_site	+	67610	67610	-4125	9647	AN1552
447	CONTIG25	63307	63662	2.52	0.00E+00	transcription_start_site	+	67818	67818	-4333	9648	AN1552
447	CONTIG25	63307	63662	2.52	0.00E+00	transcription_start_site	+	67879	67879	-4394	9649	AN1552
1169	CONTIG25	64578	65237	1.78	6.61E-03	transcription_start_site	+	67610	67610	-2702	9647	AN1552
1169	CONTIG25	64578	65237	1.78	6.61E-03	transcription_start_site	+	67818	67818	-2910	9648	AN1552
1169	CONTIG25	64578	65237	1.78	6.61E-03	transcription_start_site	+	67879	67879	-2971	9649	AN1552
448	CONTIG25	70427	70926	2.52	0.00E+00	transcription_start_site	-	70182	70182	-494	9650	AN1553
448	CONTIG25	70427	70926	2.52	0.00E+00	transcription_start_site	-	69733	69733	-943	9651	AN1553
2037	CONTIG25	72682	73411	1.36	4.36E-02	transcription_start_site	-	70182	70182	-2864	9650	AN1553

2037	CONTIG25	72682	73411	1.36	4.36E-02	transcription_start_site	-	69733	69733	-3313	9651	AN1553
448	CONTIG25	70427	70926	2.52	0.00E+00	transcription_start_site	-	71106	71106	429	9654	AN1554
448	CONTIG25	70427	70926	2.52	0.00E+00	transcription_start_site	-	71372	71372	695	9653	AN1554
2037	CONTIG25	72682	73411	1.36	4.36E-02	transcription_start_site	-	73287	73287	240	9652	AN1554
2037	CONTIG25	72682	73411	1.36	4.36E-02	transcription_start_site	-	71372	71372	-1674	9653	AN1554
2037	CONTIG25	72682	73411	1.36	4.36E-02	transcription_start_site	-	71106	71106	-1940	9654	AN1554
2499	CONTIG25	75755	76109	1.2	6.16E-02	transcription_start_site	-	73287	73287	-2645	9652	AN1554
2499	CONTIG25	75755	76109	1.2	6.16E-02	transcription_start_site	-	71372	71372	-4560	9653	AN1554
2499	CONTIG25	75755	76109	1.2	6.16E-02	transcription_start_site	-	71106	71106	-4826	9654	AN1554
2037	CONTIG25	72682	73411	1.36	4.36E-02	transcription_start_site	+	77614	77614	-4567	9655	AN1555
2499	CONTIG25	75755	76109	1.2	6.16E-02	transcription_start_site	+	77614	77614	-1682	9655	AN1555
2499	CONTIG25	75755	76109	1.2	6.16E-02	transcription_start_site	+	80358	80358	-4426	9656	AN1555
2607	CONTIG25	86495	86979	1.16	9.24E-02	transcription_start_site	+	87224	87224	-487	9658	AN1557
2607	CONTIG25	86495	86979	1.16	9.24E-02	transcription_start_site	+	87376	87376	-639	9659	AN1557
2607	CONTIG25	86495	86979	1.16	9.24E-02	transcription_start_site	+	87972	87972	-1235	9660	AN1557
2607	CONTIG25	86495	86979	1.16	9.24E-02	transcription_start_site	+	88034	88034	-1297	9661	AN1557
2607	CONTIG25	86495	86979	1.16	9.24E-02	transcription_start_site	+	88801	88801	-2064	9662	AN1557
2894	CONTIG25	85205	85494	1.01	1.66E-01	transcription_start_site	+	87224	87224	-1874	9658	AN1557
2894	CONTIG25	85205	85494	1.01	1.66E-01	transcription_start_site	+	87376	87376	-2026	9659	AN1557
2894	CONTIG25	85205	85494	1.01	1.66E-01	transcription_start_site	+	87972	87972	-2622	9660	AN1557
2894	CONTIG25	85205	85494	1.01	1.66E-01	transcription_start_site	+	88034	88034	-2684	9661	AN1557
2894	CONTIG25	85205	85494	1.01	1.66E-01	transcription_start_site	+	88801	88801	-3451	9662	AN1557
2031	CONTIG25	90090	90579	1.36	2.64E-02	transcription_start_site	-	90219	90219	-115	9671	AN1558
2031	CONTIG25	90090	90579	1.36	2.64E-02	transcription_start_site	-	90790	90790	455	9670	AN1558
2031	CONTIG25	90090	90579	1.36	2.64E-02	transcription_start_site	-	91084	91084	749	9669	AN1558
2031	CONTIG25	90090	90579	1.36	2.64E-02	transcription_start_site	-	91220	91220	885	9668	AN1558
2955	CONTIG25	96245	96509	0.97	1.93E-01	transcription_start_site	-	93986	93986	-2391	9663	AN1558
2955	CONTIG25	96245	96509	0.97	1.93E-01	transcription_start_site	-	93905	93905	-2472	9664	AN1558
2955	CONTIG25	96245	96509	0.97	1.93E-01	transcription_start_site	-	93603	93603	-2774	9665	AN1558
2955	CONTIG25	96245	96509	0.97	1.93E-01	transcription_start_site	-	93498	93498	-2879	9666	AN1558
2955	CONTIG25	96245	96509	0.97	1.93E-01	transcription_start_site	-	92965	92965	-3412	9667	AN1558
2506	CONTIG25	101345	101619	1.2	8.01E-02	transcription_start_site	-	96597	96597	-4885	9672	AN1559
2506	CONTIG25	101345	101619	1.2	8.01E-02	transcription_start_site	-	96473	96473	-5009	9673	AN1559
2955	CONTIG25	96245	96509	0.97	1.93E-01	transcription_start_site	-	96473	96473	96	9673	AN1559
2955	CONTIG25	96245	96509	0.97	1.93E-01	transcription_start_site	-	96597	96597	220	9672	AN1559
2955	CONTIG25	96245	96509	0.97	1.93E-01	transcription_start_site	+	97362	97362	-985	9674	AN1560
2955	CONTIG25	96245	96509	0.97	1.93E-01	transcription_start_site	+	97719	97719	-1342	9675	AN1560
2955	CONTIG25	96245	96509	0.97	1.93E-01	transcription_start_site	+	98226	98226	-1849	9676	AN1560
2506	CONTIG25	101345	101619	1.2	8.01E-02	transcription_start_site	+	101450	101450	32	9678	AN1561
2506	CONTIG25	101345	101619	1.2	8.01E-02	transcription_start_site	+	101159	101159	323	9677	AN1561
2955	CONTIG25	96245	96509	0.97	1.93E-01	transcription_start_site	+	101159	101159	-4782	9677	AN1561
2955	CONTIG25	96245	96509	0.97	1.93E-01	transcription_start_site	+	101450	101450	-5073	9678	AN1561
2827	CONTIG25	106744	107153	1.05	1.44E-01	transcription_start_site	-	104568	104568	-2380	9679	AN1562
2827	CONTIG25	106744	107153	1.05	1.44E-01	transcription_start_site	+	109276	109276	-2327	9680	AN1563
2827	CONTIG25	106744	107153	1.05	1.44E-01	transcription_start_site	+	109498	109498	-2549	9681	AN1563
2827	CONTIG25	106744	107153	1.05	1.44E-01	transcription_start_site	+	109637	109637	-2688	9682	AN1563
2827	CONTIG25	106744	107153	1.05	1.44E-01	transcription_start_site	+	109990	109990	-3041	9683	AN1563
2601	CONTIG25	137779	139263	1.16	7.51E-02	transcription_start_site	+	137283	137283	1238	9706	AN1572
2828	CONTIG25	137043	137393	1.05	1.44E-01	transcription_start_site	+	137283	137283	-65	9706	AN1572
2828	CONTIG25	137043	137393	1.05	1.44E-01	transcription_start_site	+	136382	136382	836	9705	AN1572
2601	CONTIG25	137779	139263	1.16	7.51E-02	transcription_start_site	-	138995	138995	474	9709	AN1573
2601	CONTIG25	137779	139263	1.16	7.51E-02	transcription_start_site	-	139382	139382	861	9708	AN1573
2601	CONTIG25	137779	139263	1.16	7.51E-02	transcription_start_site	-	139632	139632	1111	9707	AN1573
2895	CONTIG25	141694	142119	1.01	1.66E-01	transcription_start_site	-	139632	139632	-2274	9707	AN1573
2895	CONTIG25	141694	142119	1.01	1.66E-01	transcription_start_site	-	139382	139382	-2524	9708	AN1573
2895	CONTIG25	141694	142119	1.01	1.66E-01	transcription_start_site	-	138995	138995	-2911	9709	AN1573
2956	CONTIG25	140419	141435	0.97	1.99E-01	transcription_start_site	-	139632	139632	-1295	9707	AN1573

2956	CONTIG25	140419	141435	0.97	1.99E-01	transcription_start_site	-	139382	139382	-1545	9708	AN1573
2956	CONTIG25	140419	141435	0.97	1.99E-01	transcription_start_site	-	138995	138995	-1932	9709	AN1573
2895	CONTIG25	141694	142119	1.01	1.66E-01	transcription_start_site	-	141850	141850	-56	9710	AN1574
2895	CONTIG25	141694	142119	1.01	1.66E-01	transcription_start_site	-	141375	141375	-531	9711	AN1574
2956	CONTIG25	140419	141435	0.97	1.99E-01	transcription_start_site	-	141375	141375	448	9711	AN1574
2956	CONTIG25	140419	141435	0.97	1.99E-01	transcription_start_site	-	141850	141850	923	9710	AN1574
2895	CONTIG25	141694	142119	1.01	1.66E-01	transcription_start_site	-	143092	143092	1185	9721	AN1575
398	CONTIG25	159984	160403	2.6	0.00E+00	transcription_start_site	-	160034	160034	-159	9732	AN1578
398	CONTIG25	159984	160403	2.6	0.00E+00	transcription_start_site	-	159821	159821	-372	9733	AN1578
398	CONTIG25	159984	160403	2.6	0.00E+00	transcription_start_site	+	162086	162086	-1892	9734	AN1579
398	CONTIG25	159984	160403	2.6	0.00E+00	transcription_start_site	+	162343	162343	-2149	9735	AN1579
398	CONTIG25	159984	160403	2.6	0.00E+00	transcription_start_site	+	162603	162603	-2409	9736	AN1579
398	CONTIG25	159984	160403	2.6	0.00E+00	transcription_start_site	+	162817	162817	-2623	9737	AN1579
398	CONTIG25	159984	160403	2.6	0.00E+00	transcription_start_site	+	163057	163057	-2863	9738	AN1579
398	CONTIG25	159984	160403	2.6	0.00E+00	transcription_start_site	+	163245	163245	-3051	9739	AN1579
1230	CONTIG25	188026	188320	1.74	7.85E-03	transcription_start_site	-	187620	187620	-553	9755	AN1587
1230	CONTIG25	188026	188320	1.74	7.85E-03	transcription_start_site	-	186552	186552	-1621	9756	AN1587
1230	CONTIG25	188026	188320	1.74	7.85E-03	transcription_start_site	+	188056	188056	117	9757	AN1588
1230	CONTIG25	188026	188320	1.74	7.85E-03	transcription_start_site	+	188644	188644	-471	9758	AN1588
1230	CONTIG25	188026	188320	1.74	7.85E-03	transcription_start_site	+	189065	189065	-892	9759	AN1588
1230	CONTIG25	188026	188320	1.74	7.85E-03	transcription_start_site	+	189166	189166	-993	9760	AN1588
1230	CONTIG25	188026	188320	1.74	7.85E-03	transcription_start_site	+	189282	189282	-1109	9761	AN1588
1230	CONTIG25	188026	188320	1.74	7.85E-03	transcription_start_site	+	189650	189650	-1477	9762	AN1588
1230	CONTIG25	188026	188320	1.74	7.85E-03	transcription_start_site	+	189797	189797	-1624	9763	AN1588
1230	CONTIG25	188026	188320	1.74	7.85E-03	transcription_start_site	+	189945	189945	-1772	9764	AN1588
1230	CONTIG25	188026	188320	1.74	7.85E-03	transcription_start_site	+	190211	190211	-2038	9765	AN1588
1230	CONTIG25	188026	188320	1.74	7.85E-03	transcription_start_site	+	190442	190442	-2269	9766	AN1588
2896	CONTIG25	212861	213125	1.01	1.66E-01	transcription_start_site	-	210413	210413	-2580	9784	AN1594
2896	CONTIG25	212861	213125	1.01	1.66E-01	transcription_start_site	-	209387	209387	-3606	9785	AN1594
2896	CONTIG25	212861	213125	1.01	1.66E-01	transcription_start_site	-	209046	209046	-3947	9786	AN1594
2368	CONTIG25	217051	217335	1.24	6.84E-02	transcription_start_site	-	213476	213476	-3717	9791	AN1596
2368	CONTIG25	217051	217335	1.24	6.84E-02	transcription_start_site	-	213070	213070	-4123	9792	AN1596
2896	CONTIG25	212861	213125	1.01	1.66E-01	transcription_start_site	-	213070	213070	77	9792	AN1596
2896	CONTIG25	212861	213125	1.01	1.66E-01	transcription_start_site	-	213476	213476	483	9791	AN1596
2896	CONTIG25	212861	213125	1.01	1.66E-01	transcription_start_site	+	213746	213746	-753	9793	AN1597
2896	CONTIG25	212861	213125	1.01	1.66E-01	transcription_start_site	+	214093	214093	-1100	9794	AN1597
2896	CONTIG25	212861	213125	1.01	1.66E-01	transcription_start_site	+	214696	214696	-1703	9795	AN1597
2368	CONTIG25	217051	217335	1.24	6.84E-02	transcription_start_site	+	217597	217597	-404	9798	AN1598
2368	CONTIG25	217051	217335	1.24	6.84E-02	transcription_start_site	+	216650	216650	543	9797	AN1598
2368	CONTIG25	217051	217335	1.24	6.84E-02	transcription_start_site	+	216349	216349	844	9796	AN1598
2896	CONTIG25	212861	213125	1.01	1.66E-01	transcription_start_site	+	216349	216349	-3356	9796	AN1598
2896	CONTIG25	212861	213125	1.01	1.66E-01	transcription_start_site	+	216650	216650	-3657	9797	AN1598
2896	CONTIG25	212861	213125	1.01	1.66E-01	transcription_start_site	+	217597	217597	-4604	9798	AN1598
2368	CONTIG25	217051	217335	1.24	6.84E-02	transcription_start_site	+	218964	218964	-1771	9799	AN1599
2368	CONTIG25	217051	217335	1.24	6.84E-02	transcription_start_site	+	219174	219174	-1981	9800	AN1599
2368	CONTIG25	217051	217335	1.24	6.84E-02	transcription_start_site	+	219580	219580	-2387	9801	AN1599
2368	CONTIG25	217051	217335	1.24	6.84E-02	transcription_start_site	+	220216	220216	-3023	9802	AN1599
2368	CONTIG25	217051	217335	1.24	6.84E-02	transcription_start_site	+	220606	220606	-3413	9803	AN1599
124	CONTIG26	6691	6955	3.13	0.00E+00	transcription_start_site	-	3799	3799	-3024	9811	AN1602
1461	CONTIG26	3011	3585	1.61	3.99E-02	transcription_start_site	-	3799	3799	501	9811	AN1602
1555	CONTIG26	5706	6430	1.56	4.42E-02	transcription_start_site	-	3799	3799	-2269	9811	AN1602
2453	CONTIG26	7596	8246	1.22	1.93E-01	transcription_start_site	-	3799	3799	-4122	9811	AN1602
124	CONTIG26	6691	6955	3.13	0.00E+00	transcription_start_site	+	6344	6344	479	9813	AN1603
124	CONTIG26	6691	6955	3.13	0.00E+00	transcription_start_site	+	5965	5965	858	9812	AN1603
1461	CONTIG26	3011	3585	1.61	3.99E-02	transcription_start_site	+	5965	5965	-2667	9812	AN1603
1461	CONTIG26	3011	3585	1.61	3.99E-02	transcription_start_site	+	6344	6344	-3046	9813	AN1603
1555	CONTIG26	5706	6430	1.56	4.42E-02	transcription_start_site	+	5965	5965	103	9812	AN1603

1555	CONTIG26	5706	6430	1.56	4.42E-02	transcription_start_site	+	6344	6344	-276	9813	AN1603
124	CONTIG26	6691	6955	3.13	0.00E+00	transcription_start_site	+	8263	8263	-1440	9814	AN1604
124	CONTIG26	6691	6955	3.13	0.00E+00	transcription_start_site	+	8413	8413	-1590	9815	AN1604
1461	CONTIG26	3011	3585	1.61	3.99E-02	transcription_start_site	+	8263	8263	-4965	9814	AN1604
1461	CONTIG26	3011	3585	1.61	3.99E-02	transcription_start_site	+	8413	8413	-5115	9815	AN1604
1555	CONTIG26	5706	6430	1.56	4.42E-02	transcription_start_site	+	8263	8263	-2195	9814	AN1604
1555	CONTIG26	5706	6430	1.56	4.42E-02	transcription_start_site	+	8413	8413	-2345	9815	AN1604
2453	CONTIG26	7596	8246	1.22	1.93E-01	transcription_start_site	+	8263	8263	-342	9814	AN1604
2453	CONTIG26	7596	8246	1.22	1.93E-01	transcription_start_site	+	8413	8413	-492	9815	AN1604
71	CONTIG26	9696	9950	3.32	0.00E+00	transcription_start_site	+	10567	10567	-744	9816	AN1605
124	CONTIG26	6691	6955	3.13	0.00E+00	transcription_start_site	+	10567	10567	-3744	9816	AN1605
1555	CONTIG26	5706	6430	1.56	4.42E-02	transcription_start_site	+	10567	10567	-4499	9816	AN1605
2453	CONTIG26	7596	8246	1.22	1.93E-01	transcription_start_site	+	10567	10567	-2646	9816	AN1605
1561	CONTIG26	13056	13325	1.56	6.84E-02	transcription_start_site	-	13388	13388	197	9817	AN1606
1356	CONTIG26	35268	35832	1.66	3.33E-02	transcription_start_site	-	33219	33219	-2331	9838	AN1611
1356	CONTIG26	35268	35832	1.66	3.33E-02	transcription_start_site	-	36003	36003	453	9842	AN1612
1356	CONTIG26	35268	35832	1.66	3.33E-02	transcription_start_site	-	36410	36410	860	9841	AN1612
1356	CONTIG26	35268	35832	1.66	3.33E-02	transcription_start_site	-	36579	36579	1029	9840	AN1612
1356	CONTIG26	35268	35832	1.66	3.33E-02	transcription_start_site	-	36716	36716	1166	9839	AN1612
317	CONTIG26	41717	42881	2.74	9.51E-04	transcription_start_site	-	39177	39177	-3122	9843	AN1613
317	CONTIG26	41717	42881	2.74	9.51E-04	transcription_start_site	-	38827	38827	-3472	9844	AN1613
317	CONTIG26	41717	42881	2.74	9.51E-04	transcription_start_site	-	38598	38598	-3701	9845	AN1613
317	CONTIG26	41717	42881	2.74	9.51E-04	transcription_start_site	-	38199	38199	-4100	9846	AN1613
317	CONTIG26	41717	42881	2.74	9.51E-04	transcription_start_site	-	37947	37947	-4352	9847	AN1613
317	CONTIG26	41717	42881	2.74	9.51E-04	transcription_start_site	-	41280	41280	-1019	9848	AN1614
1454	CONTIG26	49963	50527	1.61	3.61E-02	transcription_start_site	-	47555	47555	-2690	9849	AN1615
1454	CONTIG26	49963	50527	1.61	3.61E-02	transcription_start_site	-	46207	46207	-4038	9850	AN1615
1454	CONTIG26	49963	50527	1.61	3.61E-02	transcription_start_site	-	46071	46071	-4174	9851	AN1615
1454	CONTIG26	49963	50527	1.61	3.61E-02	transcription_start_site	-	45709	45709	-4536	9852	AN1615
1454	CONTIG26	49963	50527	1.61	3.61E-02	transcription_start_site	-	45267	45267	-4978	9853	AN1615
1454	CONTIG26	49963	50527	1.61	3.61E-02	transcription_start_site	-	44983	44983	-5262	9854	AN1615
1454	CONTIG26	49963	50527	1.61	3.61E-02	transcription_start_site	+	50497	50497	-252	9857	AN1616
1454	CONTIG26	49963	50527	1.61	3.61E-02	transcription_start_site	+	50699	50699	-454	9858	AN1616
1454	CONTIG26	49963	50527	1.61	3.61E-02	transcription_start_site	+	50842	50842	-597	9859	AN1616
1454	CONTIG26	49963	50527	1.61	3.61E-02	transcription_start_site	+	50988	50988	-743	9860	AN1616
1454	CONTIG26	49963	50527	1.61	3.61E-02	transcription_start_site	+	52744	52744	-2499	9861	AN1617
1454	CONTIG26	49963	50527	1.61	3.61E-02	transcription_start_site	+	53159	53159	-2914	9862	AN1617
949	CONTIG26	73817	75441	1.96	9.21E-03	transcription_start_site	-	69123	69123	-5506	9875	AN1623
52	CONTIG26	75909	76418	3.42	0.00E+00	transcription_start_site	-	75486	75486	-677	9880	AN1624
755	CONTIG26	80029	80311	2.15	8.81E-03	transcription_start_site	-	75486	75486	-4684	9880	AN1624
949	CONTIG26	73817	75441	1.96	9.21E-03	transcription_start_site	-	75486	75486	857	9880	AN1624
52	CONTIG26	75909	76418	3.42	0.00E+00	transcription_start_site	+	80324	80324	-4160	9881	AN1625
52	CONTIG26	75909	76418	3.42	0.00E+00	transcription_start_site	+	80709	80709	-4545	9882	AN1625
52	CONTIG26	75909	76418	3.42	0.00E+00	transcription_start_site	+	80947	80947	-4783	9883	AN1625
755	CONTIG26	80029	80311	2.15	8.81E-03	transcription_start_site	+	80324	80324	-154	9881	AN1625
755	CONTIG26	80029	80311	2.15	8.81E-03	transcription_start_site	+	80709	80709	-539	9882	AN1625
755	CONTIG26	80029	80311	2.15	8.81E-03	transcription_start_site	+	80947	80947	-777	9883	AN1625
755	CONTIG26	80029	80311	2.15	8.81E-03	transcription_start_site	+	81548	81548	-1378	9884	AN1625
755	CONTIG26	80029	80311	2.15	8.81E-03	transcription_start_site	+	81827	81827	-1657	9885	AN1625
949	CONTIG26	73817	75441	1.96	9.21E-03	transcription_start_site	+	80324	80324	-5695	9881	AN1625
232	CONTIG26	94444	94853	2.88	6.10E-04	transcription_start_site	-	94223	94223	-425	9892	AN1628
232	CONTIG26	94444	94853	2.88	6.10E-04	transcription_start_site	-	93896	93896	-752	9893	AN1628
232	CONTIG26	94444	94853	2.88	6.10E-04	transcription_start_site	-	93580	93580	-1068	9894	AN1628
2145	CONTIG26	95629	95978	1.32	1.44E-01	transcription_start_site	-	94223	94223	-1580	9892	AN1628
2145	CONTIG26	95629	95978	1.32	1.44E-01	transcription_start_site	-	93896	93896	-1907	9893	AN1628
2145	CONTIG26	95629	95978	1.32	1.44E-01	transcription_start_site	-	93580	93580	-2223	9894	AN1628
232	CONTIG26	94444	94853	2.88	6.10E-04	transcription_start_site	-	95733	95733	1084	9899	AN1629

806	CONTIG26	99535	100044	2.1	1.11E-02	transcription_start_site	-	97350	97350	-2439	9895	AN1629
806	CONTIG26	99535	100044	2.1	1.11E-02	transcription_start_site	-	97174	97174	-2615	9896	AN1629
806	CONTIG26	99535	100044	2.1	1.11E-02	transcription_start_site	-	97094	97094	-2695	9897	AN1629
806	CONTIG26	99535	100044	2.1	1.11E-02	transcription_start_site	-	96923	96923	-2866	9898	AN1629
806	CONTIG26	99535	100044	2.1	1.11E-02	transcription_start_site	-	95733	95733	-4056	9899	AN1629
2145	CONTIG26	95629	95978	1.32	1.44E-01	transcription_start_site	-	95733	95733	-70	9899	AN1629
2145	CONTIG26	95629	95978	1.32	1.44E-01	transcription_start_site	-	96923	96923	1119	9898	AN1629
2011	CONTIG26	106280	106799	1.37	1.07E-01	transcription_start_site	-	105852	105852	-687	9900	AN1631
2011	CONTIG26	106280	106799	1.37	1.07E-01	transcription_start_site	-	105700	105700	-839	9901	AN1631
2011	CONTIG26	106280	106799	1.37	1.07E-01	transcription_start_site	-	105415	105415	-1124	9902	AN1631
2011	CONTIG26	106280	106799	1.37	1.07E-01	transcription_start_site	-	104916	104916	-1623	9903	AN1631
2011	CONTIG26	106280	106799	1.37	1.07E-01	transcription_start_site	-	104436	104436	-2103	9904	AN1631
2011	CONTIG26	106280	106799	1.37	1.07E-01	transcription_start_site	-	106963	106963	423	9907	AN1632
1653	CONTIG26	118962	119226	1.52	8.01E-02	transcription_start_site	-	115717	115717	-3377	9910	AN1634
1653	CONTIG26	118962	119226	1.52	8.01E-02	transcription_start_site	-	115357	115357	-3737	9911	AN1634
1130	CONTIG26	122642	122998	1.81	3.08E-02	transcription_start_site	-	118634	118634	-4186	9913	AN1635
1130	CONTIG26	122642	122998	1.81	3.08E-02	transcription_start_site	-	118510	118510	-4310	9914	AN1635
1130	CONTIG26	122642	122998	1.81	3.08E-02	transcription_start_site	-	118227	118227	-4593	9915	AN1635
1653	CONTIG26	118962	119226	1.52	8.01E-02	transcription_start_site	-	118634	118634	-460	9913	AN1635
1653	CONTIG26	118962	119226	1.52	8.01E-02	transcription_start_site	-	118510	118510	-584	9914	AN1635
1653	CONTIG26	118962	119226	1.52	8.01E-02	transcription_start_site	-	118227	118227	-867	9915	AN1635
1653	CONTIG26	118962	119226	1.52	8.01E-02	transcription_start_site	-	117640	117640	-1454	9916	AN1635
1653	CONTIG26	118962	119226	1.52	8.01E-02	transcription_start_site	+	119007	119007	87	9917	AN1636
1653	CONTIG26	118962	119226	1.52	8.01E-02	transcription_start_site	+	119654	119654	-560	9918	AN1636
1130	CONTIG26	122642	122998	1.81	3.08E-02	transcription_start_site	-	122633	122633	-187	9919	AN1637
1130	CONTIG26	122642	122998	1.81	3.08E-02	transcription_start_site	-	122476	122476	-344	9920	AN1637
1130	CONTIG26	122642	122998	1.81	3.08E-02	transcription_start_site	-	123401	123401	581	9923	AN1638
1130	CONTIG26	122642	122998	1.81	3.08E-02	transcription_start_site	+	126949	126949	-4129	9924	AN1639
1130	CONTIG26	122642	122998	1.81	3.08E-02	transcription_start_site	+	127142	127142	-4322	9925	AN1639
1130	CONTIG26	122642	122998	1.81	3.08E-02	transcription_start_site	+	127348	127348	-4528	9926	AN1639
546	CONTIG26	173641	174126	2.39	3.61E-03	transcription_start_site	-	169131	169131	-4752	9977	AN1651
546	CONTIG26	173641	174126	2.39	3.61E-03	transcription_start_site	-	168876	168876	-5007	9978	AN1651
547	CONTIG26	181506	182014	2.39	3.61E-03	transcription_start_site	+	182997	182997	-1237	9982	AN1652
547	CONTIG26	181506	182014	2.39	3.61E-03	transcription_start_site	+	183623	183623	-1863	9983	AN1652
547	CONTIG26	181506	182014	2.39	3.61E-03	transcription_start_site	+	185838	185838	-4078	9984	AN1653
807	CONTIG26	186543	186817	2.1	1.11E-02	transcription_start_site	+	185838	185838	842	9984	AN1653
807	CONTIG26	186543	186817	2.1	1.11E-02	transcription_start_site	+	188673	188673	-1993	9985	AN1653
807	CONTIG26	186543	186817	2.1	1.11E-02	transcription_start_site	+	188822	188822	-2142	9986	AN1653
807	CONTIG26	186543	186817	2.1	1.11E-02	transcription_start_site	+	188926	188926	-2246	9987	AN1653
807	CONTIG26	186543	186817	2.1	1.11E-02	transcription_start_site	+	189615	189615	-2935	9988	AN1653
1888	CONTIG26	193519	193868	1.42	1.08E-01	transcription_start_site	+	192522	192522	1171	9990	AN1654
72	CONTIG26	211819	212083	3.32	0.00E+00	transcription_start_site	-	211203	211203	-748	9992	AN1658
606	CONTIG26	214289	214858	2.3	2.32E-03	transcription_start_site	-	211203	211203	-3370	9992	AN1658
1782	CONTIG26	213229	213520	1.47	9.24E-02	transcription_start_site	-	211203	211203	-2171	9992	AN1658
72	CONTIG26	211819	212083	3.32	0.00E+00	transcription_start_site	+	212808	212808	-857	9993	AN1659
72	CONTIG26	211819	212083	3.32	0.00E+00	transcription_start_site	+	212873	212873	-922	9994	AN1659
72	CONTIG26	211819	212083	3.32	0.00E+00	transcription_start_site	+	213500	213500	-1549	9995	AN1659
72	CONTIG26	211819	212083	3.32	0.00E+00	transcription_start_site	+	214105	214105	-2154	9996	AN1659
606	CONTIG26	214289	214858	2.3	2.32E-03	transcription_start_site	+	214105	214105	468	9996	AN1659
606	CONTIG26	214289	214858	2.3	2.32E-03	transcription_start_site	+	213500	213500	1073	9995	AN1659
1782	CONTIG26	213229	213520	1.47	9.24E-02	transcription_start_site	+	213500	213500	-125	9995	AN1659
1782	CONTIG26	213229	213520	1.47	9.24E-02	transcription_start_site	+	212873	212873	501	9994	AN1659
1782	CONTIG26	213229	213520	1.47	9.24E-02	transcription_start_site	+	212808	212808	566	9993	AN1659
1782	CONTIG26	213229	213520	1.47	9.24E-02	transcription_start_site	+	214105	214105	-730	9996	AN1659
435	CONTIG26	263417	263928	2.54	1.80E-03	transcription_start_site	-	260467	260467	-3205	10045	AN1674
435	CONTIG26	263417	263928	2.54	1.80E-03	transcription_start_site	-	263025	263025	-647	10047	AN1675
435	CONTIG26	263417	263928	2.54	1.80E-03	transcription_start_site	-	262920	262920	-752	10048	AN1675

435	CONTIG26	263417	263928	2.54	1.80E-03	transcription_start_site	-	262225	262225	-1447	10049 AN1675
951	CONTIG26	267987	268411	1.96	1.84E-02	transcription_start_site	-	263025	263025	-5174	10047 AN1675
435	CONTIG26	263417	263928	2.54	1.80E-03	transcription_start_site	-	264481	264481	808	10051 AN1676
951	CONTIG26	267987	268411	1.96	1.84E-02	transcription_start_site	-	264983	264983	-3216	10050 AN1676
951	CONTIG26	267987	268411	1.96	1.84E-02	transcription_start_site	-	264481	264481	-3718	10051 AN1676
1005	CONTIG26	269702	271192	1.91	2.12E-02	transcription_start_site	-	264983	264983	-5464	10050 AN1676
2454	CONTIG26	268667	269301	1.22	1.93E-01	transcription_start_site	-	264983	264983	-4001	10050 AN1676
2454	CONTIG26	268667	269301	1.22	1.93E-01	transcription_start_site	-	264481	264481	-4503	10051 AN1676
951	CONTIG26	267987	268411	1.96	1.84E-02	transcription_start_site	+	269175	269175	-976	10052 AN1677
951	CONTIG26	267987	268411	1.96	1.84E-02	transcription_start_site	+	269444	269444	-1245	10053 AN1677
951	CONTIG26	267987	268411	1.96	1.84E-02	transcription_start_site	+	269896	269896	-1697	10054 AN1677
1005	CONTIG26	269702	271192	1.91	2.12E-02	transcription_start_site	+	269896	269896	551	10054 AN1677
1005	CONTIG26	269702	271192	1.91	2.12E-02	transcription_start_site	+	269444	269444	1003	10053 AN1677
1005	CONTIG26	269702	271192	1.91	2.12E-02	transcription_start_site	+	269175	269175	1272	10052 AN1677
2454	CONTIG26	268667	269301	1.22	1.93E-01	transcription_start_site	+	269175	269175	-191	10052 AN1677
2454	CONTIG26	268667	269301	1.22	1.93E-01	transcription_start_site	+	269444	269444	-460	10053 AN1677
2454	CONTIG26	268667	269301	1.22	1.93E-01	transcription_start_site	+	269896	269896	-912	10054 AN1677
951	CONTIG26	267987	268411	1.96	1.84E-02	transcription_start_site	+	273254	273254	-5055	10055 AN1678
1005	CONTIG26	269702	271192	1.91	2.12E-02	transcription_start_site	+	273254	273254	-2807	10055 AN1678
1005	CONTIG26	269702	271192	1.91	2.12E-02	transcription_start_site	+	275515	275515	-5068	10056 AN1678
2454	CONTIG26	268667	269301	1.22	1.93E-01	transcription_start_site	+	273254	273254	-4270	10055 AN1678
1005	CONTIG26	269702	271192	1.91	2.12E-02	transcription_start_site	+	275926	275926	-5479	10057 AN1679
1889	CONTIG26	284478	284912	1.42	1.08E-01	transcription_start_site	-	284543	284543	-152	10068 AN1682
1889	CONTIG26	284478	284912	1.42	1.08E-01	transcription_start_site	-	284887	284887	192	10067 AN1682
2012	CONTIG26	287188	288517	1.37	1.07E-01	transcription_start_site	-	284887	284887	-2965	10067 AN1682
2012	CONTIG26	287188	288517	1.37	1.07E-01	transcription_start_site	-	284543	284543	-3309	10068 AN1682
2301	CONTIG26	286218	286567	1.27	1.66E-01	transcription_start_site	-	284887	284887	-1505	10067 AN1682
2301	CONTIG26	286218	286567	1.27	1.66E-01	transcription_start_site	-	284543	284543	-1849	10068 AN1682
1889	CONTIG26	284478	284912	1.42	1.08E-01	transcription_start_site	-	285430	285430	735	10072 AN1683
1889	CONTIG26	284478	284912	1.42	1.08E-01	transcription_start_site	-	285776	285776	1081	10071 AN1683
2012	CONTIG26	287188	288517	1.37	1.07E-01	transcription_start_site	-	286249	286249	-1603	10069 AN1683
2012	CONTIG26	287188	288517	1.37	1.07E-01	transcription_start_site	-	286024	286024	-1828	10070 AN1683
2012	CONTIG26	287188	288517	1.37	1.07E-01	transcription_start_site	-	285776	285776	-2076	10071 AN1683
2012	CONTIG26	287188	288517	1.37	1.07E-01	transcription_start_site	-	285430	285430	-2422	10072 AN1683
2301	CONTIG26	286218	286567	1.27	1.66E-01	transcription_start_site	-	286249	286249	-143	10069 AN1683
2301	CONTIG26	286218	286567	1.27	1.66E-01	transcription_start_site	-	286024	286024	-368	10070 AN1683
2301	CONTIG26	286218	286567	1.27	1.66E-01	transcription_start_site	-	285776	285776	-616	10071 AN1683
2301	CONTIG26	286218	286567	1.27	1.66E-01	transcription_start_site	-	285430	285430	-962	10072 AN1683
1889	CONTIG26	284478	284912	1.42	1.08E-01	transcription_start_site	+	287089	287089	-2394	10073 AN1684
1889	CONTIG26	284478	284912	1.42	1.08E-01	transcription_start_site	+	287278	287278	-2583	10074 AN1684
2012	CONTIG26	287188	288517	1.37	1.07E-01	transcription_start_site	+	287278	287278	574	10074 AN1684
2012	CONTIG26	287188	288517	1.37	1.07E-01	transcription_start_site	+	287089	287089	763	10073 AN1684
2301	CONTIG26	286218	286567	1.27	1.66E-01	transcription_start_site	+	287089	287089	-696	10073 AN1684
2301	CONTIG26	286218	286567	1.27	1.66E-01	transcription_start_site	+	287278	287278	-885	10074 AN1684
2302	CONTIG26	296718	297062	1.27	1.66E-01	transcription_start_site	-	294837	294837	-2053	10075 AN1685
2302	CONTIG26	296718	297062	1.27	1.66E-01	transcription_start_site	-	294736	294736	-2154	10076 AN1685
2302	CONTIG26	296718	297062	1.27	1.66E-01	transcription_start_site	-	297342	297342	452	10080 AN1686
2302	CONTIG26	296718	297062	1.27	1.66E-01	transcription_start_site	-	297924	297924	1034	10079 AN1686
2302	CONTIG26	296718	297062	1.27	1.66E-01	transcription_start_site	+	300308	300308	-3418	10081 AN1687
2302	CONTIG26	296718	297062	1.27	1.66E-01	transcription_start_site	+	300488	300488	-3598	10082 AN1687
2302	CONTIG26	296718	297062	1.27	1.66E-01	transcription_start_site	+	300635	300635	-3745	10083 AN1687
2302	CONTIG26	296718	297062	1.27	1.66E-01	transcription_start_site	+	301084	301084	-4194	10084 AN1687
2302	CONTIG26	296718	297062	1.27	1.66E-01	transcription_start_site	+	301389	301389	-4499	10085 AN1687
1562	CONTIG26	304596	304937	1.56	6.84E-02	transcription_start_site	-	304864	304864	97	10087 AN1688
1562	CONTIG26	304596	304937	1.56	6.84E-02	transcription_start_site	-	304947	304947	180	10086 AN1688
1562	CONTIG26	304596	304937	1.56	6.84E-02	transcription_start_site	-	304335	304335	-431	10088 AN1688
1562	CONTIG26	304596	304937	1.56	6.84E-02	transcription_start_site	-	303974	303974	-792	10089 AN1688

1562	CONTIG26	304596	304937	1.56	6.84E-02	transcription_start_site	-	303715	303715	-1051	10090 AN1688
1562	CONTIG26	304596	304937	1.56	6.84E-02	transcription_start_site	-	303543	303543	-1223	10091 AN1688
1562	CONTIG26	304596	304937	1.56	6.84E-02	transcription_start_site	-	303344	303344	-1422	10092 AN1688
1563	CONTIG26	332117	332606	1.56	6.84E-02	transcription_start_site	-	330463	330463	-1898	10116 AN1696
1563	CONTIG26	332117	332606	1.56	6.84E-02	transcription_start_site	+	331757	331757	604	10117 AN1697
1563	CONTIG26	332117	332606	1.56	6.84E-02	transcription_start_site	+	333496	333496	-1134	10118 AN1697
1563	CONTIG26	332117	332606	1.56	6.84E-02	transcription_start_site	+	334009	334009	-1647	10119 AN1697
2144	CONTIG26	356265	356609	1.32	1.28E-01	transcription_start_site	-	353154	353154	-3283	10147 AN1704
2144	CONTIG26	356265	356609	1.32	1.28E-01	transcription_start_site	-	353044	353044	-3393	10148 AN1704
2144	CONTIG26	356265	356609	1.32	1.28E-01	transcription_start_site	-	355739	355739	-698	10149 AN1705
2144	CONTIG26	356265	356609	1.32	1.28E-01	transcription_start_site	-	354358	354358	-2079	10150 AN1705
2144	CONTIG26	356265	356609	1.32	1.28E-01	transcription_start_site	-	354134	354134	-2303	10151 AN1705
2144	CONTIG26	356265	356609	1.32	1.28E-01	transcription_start_site	-	353921	353921	-2516	10152 AN1705
2144	CONTIG26	356265	356609	1.32	1.28E-01	transcription_start_site	+	360685	360685	-4248	10156 AN1707
2144	CONTIG26	356265	356609	1.32	1.28E-01	transcription_start_site	+	360890	360890	-4453	10157 AN1707
2144	CONTIG26	356265	356609	1.32	1.28E-01	transcription_start_site	+	361315	361315	-4878	10158 AN1707
1006	CONTIG26	375380	375672	1.91	2.12E-02	transcription_start_site	+	375762	375762	-236	10173 AN1713
1006	CONTIG26	375380	375672	1.91	2.12E-02	transcription_start_site	+	376116	376116	-590	10174 AN1713
1006	CONTIG26	375380	375672	1.91	2.12E-02	transcription_start_site	+	377009	377009	-1483	10175 AN1713
1006	CONTIG26	375380	375672	1.91	2.12E-02	transcription_start_site	+	378323	378323	-2797	10176 AN1713
1006	CONTIG26	375380	375672	1.91	2.12E-02	transcription_start_site	+	379228	379228	-3702	10177 AN1714
1006	CONTIG26	375380	375672	1.91	2.12E-02	transcription_start_site	+	380366	380366	-4840	10178 AN1715
1006	CONTIG26	375380	375672	1.91	2.12E-02	transcription_start_site	+	380464	380464	-4938	10179 AN1715
1006	CONTIG26	375380	375672	1.91	2.12E-02	transcription_start_site	+	380620	380620	-5094	10180 AN1715
1416	CONTIG27	33076	34487	1.62	7.16E-03	transcription_start_site	-	29543	29543	-4238	10210 AN1729
1416	CONTIG27	33076	34487	1.62	7.16E-03	transcription_start_site	-	29381	29381	-4400	10211 AN1729
1416	CONTIG27	33076	34487	1.62	7.16E-03	transcription_start_site	-	29177	29177	-4604	10212 AN1729
1416	CONTIG27	33076	34487	1.62	7.16E-03	transcription_start_site	-	28839	28839	-4942	10213 AN1729
1416	CONTIG27	33076	34487	1.62	7.16E-03	transcription_start_site	-	28697	28697	-5084	10214 AN1729
1416	CONTIG27	33076	34487	1.62	7.16E-03	transcription_start_site	-	31479	31479	-2302	10217 AN1730
1416	CONTIG27	33076	34487	1.62	7.16E-03	transcription_start_site	-	33330	33330	-451	10218 AN1731
1416	CONTIG27	33076	34487	1.62	7.16E-03	transcription_start_site	-	32704	32704	-1077	10219 AN1731
2924	CONTIG27	37141	37491	0.99	1.79E-01	transcription_start_site	-	33330	33330	-3986	10218 AN1731
2924	CONTIG27	37141	37491	0.99	1.79E-01	transcription_start_site	-	32704	32704	-4612	10219 AN1731
1416	CONTIG27	33076	34487	1.62	7.16E-03	transcription_start_site	+	34994	34994	-1212	10220 AN1732
1416	CONTIG27	33076	34487	1.62	7.16E-03	transcription_start_site	+	35463	35463	-1681	10221 AN1732
1416	CONTIG27	33076	34487	1.62	7.16E-03	transcription_start_site	+	35630	35630	-1848	10222 AN1732
1416	CONTIG27	33076	34487	1.62	7.16E-03	transcription_start_site	+	37477	37477	-3695	10223 AN1733
1416	CONTIG27	33076	34487	1.62	7.16E-03	transcription_start_site	+	37653	37653	-3871	10224 AN1733
1416	CONTIG27	33076	34487	1.62	7.16E-03	transcription_start_site	+	37808	37808	-4026	10225 AN1733
1416	CONTIG27	33076	34487	1.62	7.16E-03	transcription_start_site	+	38216	38216	-4434	10226 AN1733
2924	CONTIG27	37141	37491	0.99	1.79E-01	transcription_start_site	+	37477	37477	-161	10223 AN1733
2924	CONTIG27	37141	37491	0.99	1.79E-01	transcription_start_site	+	37653	37653	-337	10224 AN1733
2924	CONTIG27	37141	37491	0.99	1.79E-01	transcription_start_site	+	37808	37808	-492	10225 AN1733
2924	CONTIG27	37141	37491	0.99	1.79E-01	transcription_start_site	+	38216	38216	-900	10226 AN1733
2926	CONTIG27	38787	39436	0.99	1.93E-01	transcription_start_site	+	38216	38216	895	10226 AN1733
2926	CONTIG27	38787	39436	0.99	1.93E-01	transcription_start_site	+	37808	37808	1303	10225 AN1733
2318	CONTIG27	40587	40944	1.26	6.84E-02	transcription_start_site	-	40653	40653	-112	10227 AN1734
2318	CONTIG27	40587	40944	1.26	6.84E-02	transcription_start_site	-	40445	40445	-320	10228 AN1734
2432	CONTIG27	42842	44089	1.22	5.52E-02	transcription_start_site	-	40653	40653	-2812	10227 AN1734
2432	CONTIG27	42842	44089	1.22	5.52E-02	transcription_start_site	-	40445	40445	-3020	10228 AN1734
2432	CONTIG27	42842	44089	1.22	5.52E-02	transcription_start_site	+	45965	45965	-2499	10229 AN1736
2917	CONTIG27	49591	51278	0.99	1.32E-01	transcription_start_site	-	50248	50248	-186	10230 AN1737
2917	CONTIG27	49591	51278	0.99	1.32E-01	transcription_start_site	+	51370	51370	-935	10231 AN1738
2917	CONTIG27	49591	51278	0.99	1.32E-01	transcription_start_site	+	51676	51676	-1241	10232 AN1738
2917	CONTIG27	49591	51278	0.99	1.32E-01	transcription_start_site	+	51799	51799	-1364	10233 AN1738
2917	CONTIG27	49591	51278	0.99	1.32E-01	transcription_start_site	+	56261	56261	-5826	10241 AN1740

2927	CONTIG27	66536	66875	0.99	1.93E-01	transcription_start_site	-	63981	63981	-2724	10254	AN1742
2927	CONTIG27	66536	66875	0.99	1.93E-01	transcription_start_site	-	63753	63753	-2952	10255	AN1742
2927	CONTIG27	66536	66875	0.99	1.93E-01	transcription_start_site	-	63701	63701	-3004	10256	AN1742
2927	CONTIG27	66536	66875	0.99	1.93E-01	transcription_start_site	-	63574	63574	-3131	10257	AN1742
2927	CONTIG27	66536	66875	0.99	1.93E-01	transcription_start_site	+	66584	66584	121	10262	AN1744
2927	CONTIG27	66536	66875	0.99	1.93E-01	transcription_start_site	+	66088	66088	617	10261	AN1744
1872	CONTIG27	71701	72275	1.42	3.70E-02	transcription_start_site	-	69703	69703	-2285	10263	AN1745
1872	CONTIG27	71701	72275	1.42	3.70E-02	transcription_start_site	-	69338	69338	-2650	10264	AN1745
1872	CONTIG27	71701	72275	1.42	3.70E-02	transcription_start_site	-	69014	69014	-2974	10265	AN1745
2177	CONTIG27	69756	70102	1.3	3.99E-02	transcription_start_site	-	69703	69703	-226	10263	AN1745
2177	CONTIG27	69756	70102	1.3	3.99E-02	transcription_start_site	-	69338	69338	-591	10264	AN1745
2177	CONTIG27	69756	70102	1.3	3.99E-02	transcription_start_site	-	69014	69014	-915	10265	AN1745
1872	CONTIG27	71701	72275	1.42	3.70E-02	transcription_start_site	+	72035	72035	-47	10266	AN1746
1872	CONTIG27	71701	72275	1.42	3.70E-02	transcription_start_site	+	72387	72387	-399	10267	AN1746
1872	CONTIG27	71701	72275	1.42	3.70E-02	transcription_start_site	+	72577	72577	-589	10268	AN1746
1872	CONTIG27	71701	72275	1.42	3.70E-02	transcription_start_site	+	73301	73301	-1313	10269	AN1746
1872	CONTIG27	71701	72275	1.42	3.70E-02	transcription_start_site	+	75006	75006	-3018	10270	AN1746
2177	CONTIG27	69756	70102	1.3	3.99E-02	transcription_start_site	+	72035	72035	-2106	10266	AN1746
2177	CONTIG27	69756	70102	1.3	3.99E-02	transcription_start_site	+	72387	72387	-2458	10267	AN1746
2177	CONTIG27	69756	70102	1.3	3.99E-02	transcription_start_site	+	72577	72577	-2648	10268	AN1746
2177	CONTIG27	69756	70102	1.3	3.99E-02	transcription_start_site	+	73301	73301	-3372	10269	AN1746
2177	CONTIG27	69756	70102	1.3	3.99E-02	transcription_start_site	+	75006	75006	-5077	10270	AN1746
2811	CONTIG27	78836	79120	1.06	1.44E-01	transcription_start_site	-	79237	79237	259	10273	AN1747
2811	CONTIG27	78836	79120	1.06	1.44E-01	transcription_start_site	-	78255	78255	-723	10274	AN1747
2811	CONTIG27	78836	79120	1.06	1.44E-01	transcription_start_site	+	81935	81935	-2957	10275	AN1748
2811	CONTIG27	78836	79120	1.06	1.44E-01	transcription_start_site	+	82433	82433	-3455	10276	AN1748
2811	CONTIG27	78836	79120	1.06	1.44E-01	transcription_start_site	+	82530	82530	-3552	10277	AN1748
2812	CONTIG27	91427	91711	1.06	1.44E-01	transcription_start_site	-	89042	89042	-2527	10280	AN1750
2812	CONTIG27	91427	91711	1.06	1.44E-01	transcription_start_site	-	88741	88741	-2828	10281	AN1750
2812	CONTIG27	91427	91711	1.06	1.44E-01	transcription_start_site	-	87484	87484	-4085	10282	AN1750
2928	CONTIG27	93542	93811	0.99	1.93E-01	transcription_start_site	-	89042	89042	-4634	10280	AN1750
2928	CONTIG27	93542	93811	0.99	1.93E-01	transcription_start_site	-	88741	88741	-4935	10281	AN1750
2559	CONTIG27	97060	97414	1.18	9.24E-02	transcription_start_site	-	94090	94090	-3147	10284	AN1752
2928	CONTIG27	93542	93811	0.99	1.93E-01	transcription_start_site	-	94090	94090	413	10284	AN1752
2812	CONTIG27	91427	91711	1.06	1.44E-01	transcription_start_site	+	94568	94568	-2999	10285	AN1753
2812	CONTIG27	91427	91711	1.06	1.44E-01	transcription_start_site	+	94704	94704	-3135	10286	AN1753
2928	CONTIG27	93542	93811	0.99	1.93E-01	transcription_start_site	+	94568	94568	-891	10285	AN1753
2928	CONTIG27	93542	93811	0.99	1.93E-01	transcription_start_site	+	94704	94704	-1027	10286	AN1753
2559	CONTIG27	97060	97414	1.18	9.24E-02	transcription_start_site	-	96060	96060	-1177	10287	AN1754
2559	CONTIG27	97060	97414	1.18	9.24E-02	transcription_start_site	+	97240	97240	-3	10289	AN1755
2559	CONTIG27	97060	97414	1.18	9.24E-02	transcription_start_site	+	97356	97356	-119	10290	AN1755
2559	CONTIG27	97060	97414	1.18	9.24E-02	transcription_start_site	+	97023	97023	214	10288	AN1755
2559	CONTIG27	97060	97414	1.18	9.24E-02	transcription_start_site	+	97582	97582	-345	10291	AN1755
2928	CONTIG27	93542	93811	0.99	1.93E-01	transcription_start_site	+	97023	97023	-3346	10288	AN1755
2928	CONTIG27	93542	93811	0.99	1.93E-01	transcription_start_site	+	97240	97240	-3563	10289	AN1755
2928	CONTIG27	93542	93811	0.99	1.93E-01	transcription_start_site	+	97356	97356	-3679	10290	AN1755
2928	CONTIG27	93542	93811	0.99	1.93E-01	transcription_start_site	+	97582	97582	-3905	10291	AN1755
2559	CONTIG27	97060	97414	1.18	9.24E-02	transcription_start_site	+	99800	99800	-2563	10292	AN1756
208	CONTIG27	108019	108428	2.92	0.00E+00	transcription_start_site	-	105821	105821	-2402	10299	AN1759
208	CONTIG27	108019	108428	2.92	0.00E+00	transcription_start_site	-	104795	104795	-3428	10300	AN1759
208	CONTIG27	108019	108428	2.92	0.00E+00	transcription_start_site	-	107361	107361	-862	10301	AN1760
208	CONTIG27	108019	108428	2.92	0.00E+00	transcription_start_site	-	106960	106960	-1263	10302	AN1760
208	CONTIG27	108019	108428	2.92	0.00E+00	transcription_start_site	-	106687	106687	-1536	10303	AN1760
208	CONTIG27	108019	108428	2.92	0.00E+00	transcription_start_site	+	108574	108574	-350	10304	AN1761
208	CONTIG27	108019	108428	2.92	0.00E+00	transcription_start_site	+	108954	108954	-730	10305	AN1761
208	CONTIG27	108019	108428	2.92	0.00E+00	transcription_start_site	+	109022	109022	-798	10306	AN1761
208	CONTIG27	108019	108428	2.92	0.00E+00	transcription_start_site	+	109118	109118	-894	10307	AN1761

208	CONTIG27	108019	108428	2.92	0.00E+00	transcription_start_site	+	109286	109286	-1062	10308 AN1761
208	CONTIG27	108019	108428	2.92	0.00E+00	transcription_start_site	+	109524	109524	-1300	10309 AN1761
208	CONTIG27	108019	108428	2.92	0.00E+00	transcription_start_site	+	112668	112668	-4444	10313 AN1763
208	CONTIG27	108019	108428	2.92	0.00E+00	transcription_start_site	+	113322	113322	-5098	10314 AN1763
918	CONTIG28	6096	6367	1.98	1.01E-03	transcription_start_site	-	2446	2446	-3785	10319 AN1765
918	CONTIG28	6096	6367	1.98	1.01E-03	transcription_start_site	-	2357	2357	-3874	10320 AN1765
918	CONTIG28	6096	6367	1.98	1.01E-03	transcription_start_site	-	2072	2072	-4159	10321 AN1765
1573	CONTIG28	1896	2150	1.55	1.11E-02	transcription_start_site	-	2072	2072	49	10321 AN1765
1573	CONTIG28	1896	2150	1.55	1.11E-02	transcription_start_site	-	2357	2357	334	10320 AN1765
1573	CONTIG28	1896	2150	1.55	1.11E-02	transcription_start_site	-	2446	2446	423	10319 AN1765
1573	CONTIG28	1896	2150	1.55	1.11E-02	transcription_start_site	+	3343	3343	-1320	10322 AN1766
1573	CONTIG28	1896	2150	1.55	1.11E-02	transcription_start_site	+	3475	3475	-1452	10323 AN1766
1573	CONTIG28	1896	2150	1.55	1.11E-02	transcription_start_site	+	3877	3877	-1854	10324 AN1766
1573	CONTIG28	1896	2150	1.55	1.11E-02	transcription_start_site	+	4189	4189	-2166	10325 AN1766
918	CONTIG28	6096	6367	1.98	1.01E-03	transcription_start_site	+	6850	6850	-618	10326 AN1767
918	CONTIG28	6096	6367	1.98	1.01E-03	transcription_start_site	+	8758	8758	-2526	10327 AN1767
1573	CONTIG28	1896	2150	1.55	1.11E-02	transcription_start_site	+	6850	6850	-4827	10326 AN1767
2948	CONTIG28	24613	25252	0.97	1.28E-01	transcription_start_site	-	22437	22437	-2495	10351 AN1774
2520	CONTIG28	30393	30667	1.19	5.88E-02	transcription_start_site	-	26812	26812	-3718	10352 AN1775
2520	CONTIG28	30393	30667	1.19	5.88E-02	transcription_start_site	-	26408	26408	-4122	10353 AN1775
2520	CONTIG28	30393	30667	1.19	5.88E-02	transcription_start_site	-	25948	25948	-4582	10354 AN1775
2948	CONTIG28	24613	25252	0.97	1.28E-01	transcription_start_site	-	25948	25948	1015	10354 AN1775
2520	CONTIG28	30393	30667	1.19	5.88E-02	transcription_start_site	+	29663	29663	867	10358 AN1776
2948	CONTIG28	24613	25252	0.97	1.28E-01	transcription_start_site	+	28534	28534	-3601	10355 AN1776
2948	CONTIG28	24613	25252	0.97	1.28E-01	transcription_start_site	+	28639	28639	-3706	10356 AN1776
2948	CONTIG28	24613	25252	0.97	1.28E-01	transcription_start_site	+	29045	29045	-4112	10357 AN1776
2948	CONTIG28	24613	25252	0.97	1.28E-01	transcription_start_site	+	29663	29663	-4730	10358 AN1776
2314	CONTIG28	90236	90585	1.26	4.36E-02	transcription_start_site	-	87318	87318	-3092	10426 AN1794
2314	CONTIG28	90236	90585	1.26	4.36E-02	transcription_start_site	-	86978	86978	-3432	10427 AN1794
2314	CONTIG28	90236	90585	1.26	4.36E-02	transcription_start_site	-	86035	86035	-4375	10428 AN1794
2314	CONTIG28	90236	90585	1.26	4.36E-02	transcription_start_site	-	85904	85904	-4506	10429 AN1794
2314	CONTIG28	90236	90585	1.26	4.36E-02	transcription_start_site	-	85687	85687	-4723	10430 AN1794
2314	CONTIG28	90236	90585	1.26	4.36E-02	transcription_start_site	+	90457	90457	-46	10435 AN1795
2314	CONTIG28	90236	90585	1.26	4.36E-02	transcription_start_site	+	90087	90087	323	10434 AN1795
2314	CONTIG28	90236	90585	1.26	4.36E-02	transcription_start_site	+	89941	89941	469	10433 AN1795
2314	CONTIG28	90236	90585	1.26	4.36E-02	transcription_start_site	+	89848	89848	562	10432 AN1795
2314	CONTIG28	90236	90585	1.26	4.36E-02	transcription_start_site	+	89717	89717	693	10431 AN1795
2314	CONTIG28	90236	90585	1.26	4.36E-02	transcription_start_site	+	92144	92144	-1733	10436 AN1796
2314	CONTIG28	90236	90585	1.26	4.36E-02	transcription_start_site	+	92536	92536	-2125	10437 AN1796
180	CONTIG28	95259	95538	2.98	0.00E+00	transcription_start_site	+	96084	96084	-685	10438 AN1797
180	CONTIG28	95259	95538	2.98	0.00E+00	transcription_start_site	+	96237	96237	-838	10439 AN1797
180	CONTIG28	95259	95538	2.98	0.00E+00	transcription_start_site	+	97075	97075	-1676	10440 AN1797
964	CONTIG28	93621	93951	1.94	1.22E-03	transcription_start_site	+	96084	96084	-2298	10438 AN1797
964	CONTIG28	93621	93951	1.94	1.22E-03	transcription_start_site	+	96237	96237	-2451	10439 AN1797
964	CONTIG28	93621	93951	1.94	1.22E-03	transcription_start_site	+	97075	97075	-3289	10440 AN1797
1733	CONTIG29	5477	5836	1.49	9.24E-02	transcription_start_site	+	5354	5354	302	10442 AN1799
2240	CONTIG29	6082	6666	1.29	1.66E-01	transcription_start_site	+	5354	5354	1020	10442 AN1799
514	CONTIG29	7217	7556	2.43	3.61E-03	transcription_start_site	+	7741	7741	-354	10443 AN1800
514	CONTIG29	7217	7556	2.43	3.61E-03	transcription_start_site	+	7877	7877	-490	10444 AN1800
514	CONTIG29	7217	7556	2.43	3.61E-03	transcription_start_site	+	7964	7964	-577	10445 AN1800
514	CONTIG29	7217	7556	2.43	3.61E-03	transcription_start_site	+	8571	8571	-1184	10446 AN1800
514	CONTIG29	7217	7556	2.43	3.61E-03	transcription_start_site	+	10782	10782	-3395	10447 AN1800
514	CONTIG29	7217	7556	2.43	3.61E-03	transcription_start_site	+	10953	10953	-3566	10448 AN1800
514	CONTIG29	7217	7556	2.43	3.61E-03	transcription_start_site	+	11144	11144	-3757	10449 AN1800
1733	CONTIG29	5477	5836	1.49	9.24E-02	transcription_start_site	+	7741	7741	-2084	10443 AN1800
1733	CONTIG29	5477	5836	1.49	9.24E-02	transcription_start_site	+	7877	7877	-2220	10444 AN1800
1733	CONTIG29	5477	5836	1.49	9.24E-02	transcription_start_site	+	7964	7964	-2307	10445 AN1800

1733	CONTIG29	5477	5836	1.49	9.24E-02	transcription_start_site	+	8571	8571	-2914	10446	AN1800
1733	CONTIG29	5477	5836	1.49	9.24E-02	transcription_start_site	+	10782	10782	-5125	10447	AN1800
2240	CONTIG29	6082	6666	1.29	1.66E-01	transcription_start_site	+	7741	7741	-1367	10443	AN1800
2240	CONTIG29	6082	6666	1.29	1.66E-01	transcription_start_site	+	7877	7877	-1503	10444	AN1800
2240	CONTIG29	6082	6666	1.29	1.66E-01	transcription_start_site	+	7964	7964	-1590	10445	AN1800
2240	CONTIG29	6082	6666	1.29	1.66E-01	transcription_start_site	+	8571	8571	-2197	10446	AN1800
2240	CONTIG29	6082	6666	1.29	1.66E-01	transcription_start_site	+	10782	10782	-4408	10447	AN1800
2240	CONTIG29	6082	6666	1.29	1.66E-01	transcription_start_site	+	10953	10953	-4579	10448	AN1800
2240	CONTIG29	6082	6666	1.29	1.66E-01	transcription_start_site	+	11144	11144	-4770	10449	AN1800
2394	CONTIG29	12242	12581	1.24	1.93E-01	transcription_start_site	-	12274	12274	-137	10450	AN1801
2394	CONTIG29	12242	12581	1.24	1.93E-01	transcription_start_site	-	11535	11535	-876	10451	AN1801
2394	CONTIG29	12242	12581	1.24	1.93E-01	transcription_start_site	+	14355	14355	-1943	10452	AN1802
2394	CONTIG29	12242	12581	1.24	1.93E-01	transcription_start_site	+	14423	14423	-2011	10453	AN1802
2394	CONTIG29	12242	12581	1.24	1.93E-01	transcription_start_site	+	14514	14514	-2102	10454	AN1802
2394	CONTIG29	12242	12581	1.24	1.93E-01	transcription_start_site	+	14658	14658	-2246	10455	AN1802
2394	CONTIG29	12242	12581	1.24	1.93E-01	transcription_start_site	+	14821	14821	-2409	10456	AN1802
2394	CONTIG29	12242	12581	1.24	1.93E-01	transcription_start_site	+	14922	14922	-2510	10457	AN1802
2394	CONTIG29	12242	12581	1.24	1.93E-01	transcription_start_site	+	15004	15004	-2592	10458	AN1802
669	CONTIG29	26718	26977	2.23	7.85E-03	transcription_start_site	-	27823	27823	975	10462	AN1805
1606	CONTIG29	39844	40188	1.54	8.01E-02	transcription_start_site	-	37458	37458	-2558	10469	AN1809
1606	CONTIG29	39844	40188	1.54	8.01E-02	transcription_start_site	-	37233	37233	-2783	10470	AN1809
1606	CONTIG29	39844	40188	1.54	8.01E-02	transcription_start_site	-	36937	36937	-3079	10471	AN1809
1606	CONTIG29	39844	40188	1.54	8.01E-02	transcription_start_site	-	36359	36359	-3657	10472	AN1809
1606	CONTIG29	39844	40188	1.54	8.01E-02	transcription_start_site	-	36145	36145	-3871	10473	AN1809
1606	CONTIG29	39844	40188	1.54	8.01E-02	transcription_start_site	-	39614	39614	-402	10474	AN1810
1606	CONTIG29	39844	40188	1.54	8.01E-02	transcription_start_site	-	39079	39079	-937	10475	AN1810
1606	CONTIG29	39844	40188	1.54	8.01E-02	transcription_start_site	-	38330	38330	-1686	10476	AN1810
1606	CONTIG29	39844	40188	1.54	8.01E-02	transcription_start_site	+	39954	39954	62	10477	AN1811
1606	CONTIG29	39844	40188	1.54	8.01E-02	transcription_start_site	+	40315	40315	-299	10478	AN1811
1606	CONTIG29	39844	40188	1.54	8.01E-02	transcription_start_site	+	40883	40883	-867	10479	AN1811
1606	CONTIG29	39844	40188	1.54	8.01E-02	transcription_start_site	+	41004	41004	-988	10480	AN1811
1600	CONTIG29	52509	53753	1.54	5.52E-02	transcription_start_site	-	54383	54383	1252	10489	AN1814
1600	CONTIG29	52509	53753	1.54	5.52E-02	transcription_start_site	-	54677	54677	1546	10488	AN1814
2235	CONTIG29	68644	69521	1.29	1.50E-01	transcription_start_site	-	63894	63894	-5188	10493	AN1817
1239	CONTIG29	69544	69803	1.74	4.36E-02	transcription_start_site	-	66525	66525	-3148	10495	AN1818
1239	CONTIG29	69544	69803	1.74	4.36E-02	transcription_start_site	-	66231	66231	-3442	10496	AN1818
1239	CONTIG29	69544	69803	1.74	4.36E-02	transcription_start_site	-	66130	66130	-3543	10497	AN1818
1239	CONTIG29	69544	69803	1.74	4.36E-02	transcription_start_site	-	66032	66032	-3641	10498	AN1818
1239	CONTIG29	69544	69803	1.74	4.36E-02	transcription_start_site	-	65828	65828	-3845	10499	AN1818
1239	CONTIG29	69544	69803	1.74	4.36E-02	transcription_start_site	-	65635	65635	-4038	10500	AN1818
1239	CONTIG29	69544	69803	1.74	4.36E-02	transcription_start_site	-	65342	65342	-4331	10501	AN1818
1239	CONTIG29	69544	69803	1.74	4.36E-02	transcription_start_site	-	65200	65200	-4473	10502	AN1818
2235	CONTIG29	68644	69521	1.29	1.50E-01	transcription_start_site	-	66525	66525	-2557	10495	AN1818
2235	CONTIG29	68644	69521	1.29	1.50E-01	transcription_start_site	-	66231	66231	-2851	10496	AN1818
2235	CONTIG29	68644	69521	1.29	1.50E-01	transcription_start_site	-	66130	66130	-2952	10497	AN1818
2235	CONTIG29	68644	69521	1.29	1.50E-01	transcription_start_site	-	66032	66032	-3050	10498	AN1818
2235	CONTIG29	68644	69521	1.29	1.50E-01	transcription_start_site	-	65828	65828	-3254	10499	AN1818
2235	CONTIG29	68644	69521	1.29	1.50E-01	transcription_start_site	-	65635	65635	-3447	10500	AN1818
2235	CONTIG29	68644	69521	1.29	1.50E-01	transcription_start_site	-	65342	65342	-3740	10501	AN1818
2235	CONTIG29	68644	69521	1.29	1.50E-01	transcription_start_site	-	65200	65200	-3882	10502	AN1818
1239	CONTIG29	69544	69803	1.74	4.36E-02	transcription_start_site	-	68200	68200	-1473	10503	AN1819
1239	CONTIG29	69544	69803	1.74	4.36E-02	transcription_start_site	-	67134	67134	-2539	10504	AN1819
2235	CONTIG29	68644	69521	1.29	1.50E-01	transcription_start_site	-	68200	68200	-882	10503	AN1819
2235	CONTIG29	68644	69521	1.29	1.50E-01	transcription_start_site	-	67134	67134	-1948	10504	AN1819
1239	CONTIG29	69544	69803	1.74	4.36E-02	transcription_start_site	+	69522	69522	151	10506	AN1820
1239	CONTIG29	69544	69803	1.74	4.36E-02	transcription_start_site	+	70287	70287	-613	10507	AN1820
1239	CONTIG29	69544	69803	1.74	4.36E-02	transcription_start_site	+	68785	68785	888	10505	AN1820

1239	CONTIG29	69544	69803	1.74	4.36E-02	transcription_start_site	+	70901	70901	-1227	10508	AN1820
2235	CONTIG29	68644	69521	1.29	1.50E-01	transcription_start_site	+	68785	68785	297	10505	AN1820
2235	CONTIG29	68644	69521	1.29	1.50E-01	transcription_start_site	+	69522	69522	-439	10506	AN1820
2235	CONTIG29	68644	69521	1.29	1.50E-01	transcription_start_site	+	70287	70287	-1204	10507	AN1820
2235	CONTIG29	68644	69521	1.29	1.50E-01	transcription_start_site	+	70901	70901	-1818	10508	AN1820
719	CONTIG29	73204	73478	2.18	8.81E-03	transcription_start_site	-	73831	73831	490	10509	AN1821
719	CONTIG29	73204	73478	2.18	8.81E-03	transcription_start_site	+	74166	74166	-825	10510	AN1822
719	CONTIG29	73204	73478	2.18	8.81E-03	transcription_start_site	+	74769	74769	-1428	10511	AN1822
1239	CONTIG29	69544	69803	1.74	4.36E-02	transcription_start_site	+	74166	74166	-4492	10510	AN1822
1239	CONTIG29	69544	69803	1.74	4.36E-02	transcription_start_site	+	74769	74769	-5095	10511	AN1822
2235	CONTIG29	68644	69521	1.29	1.50E-01	transcription_start_site	+	74166	74166	-5083	10510	AN1822
719	CONTIG29	73204	73478	2.18	8.81E-03	transcription_start_site	+	76728	76728	-3387	10512	AN1823
719	CONTIG29	73204	73478	2.18	8.81E-03	transcription_start_site	+	76943	76943	-3602	10513	AN1823
720	CONTIG29	79879	80908	2.18	8.81E-03	transcription_start_site	-	80730	80730	336	10514	AN1824
720	CONTIG29	79879	80908	2.18	8.81E-03	transcription_start_site	-	78853	78853	-1540	10515	AN1824
2241	CONTIG29	84986	85255	1.29	1.66E-01	transcription_start_site	-	80730	80730	-4390	10514	AN1824
2241	CONTIG29	84986	85255	1.29	1.66E-01	transcription_start_site	-	82491	82491	-2629	10516	AN1825
720	CONTIG29	79879	80908	2.18	8.81E-03	transcription_start_site	+	83121	83121	-2727	10517	AN1826
720	CONTIG29	79879	80908	2.18	8.81E-03	transcription_start_site	+	84013	84013	-3619	10518	AN1826
2241	CONTIG29	84986	85255	1.29	1.66E-01	transcription_start_site	+	84013	84013	1107	10518	AN1826
2092	CONTIG29	101101	101470	1.34	1.44E-01	transcription_start_site	-	97878	97878	-3407	10541	AN1832
2092	CONTIG29	101101	101470	1.34	1.44E-01	transcription_start_site	-	97478	97478	-3807	10542	AN1832
2092	CONTIG29	101101	101470	1.34	1.44E-01	transcription_start_site	-	96989	96989	-4296	10543	AN1832
2236	CONTIG29	101791	102290	1.29	1.50E-01	transcription_start_site	-	97878	97878	-4162	10541	AN1832
2236	CONTIG29	101791	102290	1.29	1.50E-01	transcription_start_site	-	97478	97478	-4562	10542	AN1832
2236	CONTIG29	101791	102290	1.29	1.50E-01	transcription_start_site	-	96989	96989	-5051	10543	AN1832
2092	CONTIG29	101101	101470	1.34	1.44E-01	transcription_start_site	+	101382	101382	-96	10548	AN1834
2092	CONTIG29	101101	101470	1.34	1.44E-01	transcription_start_site	+	102672	102672	-1386	10549	AN1834
2092	CONTIG29	101101	101470	1.34	1.44E-01	transcription_start_site	+	103702	103702	-2416	10550	AN1834
2236	CONTIG29	101791	102290	1.29	1.50E-01	transcription_start_site	+	102672	102672	-631	10549	AN1834
2236	CONTIG29	101791	102290	1.29	1.50E-01	transcription_start_site	+	101382	101382	658	10548	AN1834
2236	CONTIG29	101791	102290	1.29	1.50E-01	transcription_start_site	+	103702	103702	-1661	10550	AN1834
767	CONTIG29	106140	106404	2.13	1.11E-02	transcription_start_site	+	105428	105428	844	10554	AN1835
2092	CONTIG29	101101	101470	1.34	1.44E-01	transcription_start_site	+	104312	104312	-3026	10551	AN1835
2092	CONTIG29	101101	101470	1.34	1.44E-01	transcription_start_site	+	104635	104635	-3349	10552	AN1835
2092	CONTIG29	101101	101470	1.34	1.44E-01	transcription_start_site	+	104919	104919	-3633	10553	AN1835
2092	CONTIG29	101101	101470	1.34	1.44E-01	transcription_start_site	+	105428	105428	-4142	10554	AN1835
2236	CONTIG29	101791	102290	1.29	1.50E-01	transcription_start_site	+	104312	104312	-2271	10551	AN1835
2236	CONTIG29	101791	102290	1.29	1.50E-01	transcription_start_site	+	104635	104635	-2594	10552	AN1835
2236	CONTIG29	101791	102290	1.29	1.50E-01	transcription_start_site	+	104919	104919	-2878	10553	AN1835
2236	CONTIG29	101791	102290	1.29	1.50E-01	transcription_start_site	+	105428	105428	-3387	10554	AN1835
2242	CONTIG29	104865	105744	1.29	1.66E-01	transcription_start_site	+	105428	105428	-123	10554	AN1835
2242	CONTIG29	104865	105744	1.29	1.66E-01	transcription_start_site	+	104919	104919	385	10553	AN1835
2242	CONTIG29	104865	105744	1.29	1.66E-01	transcription_start_site	+	104635	104635	669	10552	AN1835
2242	CONTIG29	104865	105744	1.29	1.66E-01	transcription_start_site	+	104312	104312	992	10551	AN1835
515	CONTIG29	113118	113470	2.43	3.61E-03	transcription_start_site	-	109386	109386	-3908	10555	AN1837
515	CONTIG29	113118	113470	2.43	3.61E-03	transcription_start_site	-	109035	109035	-4259	10556	AN1837
515	CONTIG29	113118	113470	2.43	3.61E-03	transcription_start_site	-	108895	108895	-4399	10557	AN1837
515	CONTIG29	113118	113470	2.43	3.61E-03	transcription_start_site	-	110929	110929	-2365	10558	AN1838
515	CONTIG29	113118	113470	2.43	3.61E-03	transcription_start_site	-	110675	110675	-2619	10559	AN1838
515	CONTIG29	113118	113470	2.43	3.61E-03	transcription_start_site	-	112786	112786	-508	10560	AN1839
515	CONTIG29	113118	113470	2.43	3.61E-03	transcription_start_site	-	112367	112367	-927	10561	AN1839
515	CONTIG29	113118	113470	2.43	3.61E-03	transcription_start_site	-	112126	112126	-1168	10562	AN1839
1163	CONTIG29	121803	122092	1.79	3.70E-02	transcription_start_site	-	116884	116884	-5063	10563	AN1840
1163	CONTIG29	121803	122092	1.79	3.70E-02	transcription_start_site	-	118237	118237	-3710	10565	AN1841
1163	CONTIG29	121803	122092	1.79	3.70E-02	transcription_start_site	-	117881	117881	-4066	10566	AN1841
1163	CONTIG29	121803	122092	1.79	3.70E-02	transcription_start_site	-	117734	117734	-4213	10567	AN1841

1163	CONTIG29	121803	122092	1.79	3.70E-02	transcription_start_site	+	120870	120870	1077	10571	AN1843
1163	CONTIG29	121803	122092	1.79	3.70E-02	transcription_start_site	+	120805	120805	1142	10570	AN1843
1163	CONTIG29	121803	122092	1.79	3.70E-02	transcription_start_site	+	122707	122707	-759	10572	AN1844
1163	CONTIG29	121803	122092	1.79	3.70E-02	transcription_start_site	+	123256	123256	-1308	10573	AN1844
1163	CONTIG29	121803	122092	1.79	3.70E-02	transcription_start_site	+	125457	125457	-3509	10574	AN1845
1163	CONTIG29	121803	122092	1.79	3.70E-02	transcription_start_site	+	125612	125612	-3664	10575	AN1845
159	CONTIG29	130594	130853	3.03	2.39E-04	transcription_start_site	-	127557	127557	-3166	10576	AN1846
159	CONTIG29	130594	130853	3.03	2.39E-04	transcription_start_site	-	126754	126754	-3969	10577	AN1846
818	CONTIG29	132234	132729	2.08	1.30E-02	transcription_start_site	-	127557	127557	-4924	10576	AN1846
159	CONTIG29	130594	130853	3.03	2.39E-04	transcription_start_site	-	131630	131630	906	10579	AN1847
818	CONTIG29	132234	132729	2.08	1.30E-02	transcription_start_site	-	132368	132368	-113	10578	AN1847
818	CONTIG29	132234	132729	2.08	1.30E-02	transcription_start_site	-	131630	131630	-851	10579	AN1847
1386	CONTIG29	178953	179307	1.64	5.88E-02	transcription_start_site	-	175603	175603	-3527	10632	AN1864
1386	CONTIG29	178953	179307	1.64	5.88E-02	transcription_start_site	-	174741	174741	-4389	10633	AN1864
1386	CONTIG29	178953	179307	1.64	5.88E-02	transcription_start_site	+	178535	178535	595	10636	AN1865
1386	CONTIG29	178953	179307	1.64	5.88E-02	transcription_start_site	+	179303	179303	-173	10637	AN1866
1386	CONTIG29	178953	179307	1.64	5.88E-02	transcription_start_site	+	179548	179548	-418	10638	AN1866
1386	CONTIG29	178953	179307	1.64	5.88E-02	transcription_start_site	+	179993	179993	-863	10639	AN1866
1386	CONTIG29	178953	179307	1.64	5.88E-02	transcription_start_site	+	180218	180218	-1088	10640	AN1866
1386	CONTIG29	178953	179307	1.64	5.88E-02	transcription_start_site	+	180636	180636	-1506	10641	AN1866
1492	CONTIG29	183833	184177	1.59	5.05E-02	transcription_start_site	-	182071	182071	-1934	10642	AN1867
1492	CONTIG29	183833	184177	1.59	5.05E-02	transcription_start_site	-	181140	181140	-2865	10643	AN1867
1492	CONTIG29	183833	184177	1.59	5.05E-02	transcription_start_site	-	181022	181022	-2983	10644	AN1867
1386	CONTIG29	178953	179307	1.64	5.88E-02	transcription_start_site	+	183007	183007	-3877	10645	AN1868
1386	CONTIG29	178953	179307	1.64	5.88E-02	transcription_start_site	+	183478	183478	-4348	10646	AN1868
1386	CONTIG29	178953	179307	1.64	5.88E-02	transcription_start_site	+	183588	183588	-4458	10647	AN1868
1492	CONTIG29	183833	184177	1.59	5.05E-02	transcription_start_site	+	183588	183588	417	10647	AN1868
1492	CONTIG29	183833	184177	1.59	5.05E-02	transcription_start_site	+	183478	183478	527	10646	AN1868
1492	CONTIG29	183833	184177	1.59	5.05E-02	transcription_start_site	+	183007	183007	998	10645	AN1868
721	CONTIG29	187278	187552	2.18	8.81E-03	transcription_start_site	-	186081	186081	-1334	10648	AN1869
721	CONTIG29	187278	187552	2.18	8.81E-03	transcription_start_site	-	185994	185994	-1421	10649	AN1869
721	CONTIG29	187278	187552	2.18	8.81E-03	transcription_start_site	-	185884	185884	-1531	10650	AN1869
721	CONTIG29	187278	187552	2.18	8.81E-03	transcription_start_site	+	187267	187267	148	10651	AN1870
721	CONTIG29	187278	187552	2.18	8.81E-03	transcription_start_site	+	188055	188055	-640	10652	AN1870
1492	CONTIG29	183833	184177	1.59	5.05E-02	transcription_start_site	+	187267	187267	-3262	10651	AN1870
1492	CONTIG29	183833	184177	1.59	5.05E-02	transcription_start_site	+	188055	188055	-4050	10652	AN1870
721	CONTIG29	187278	187552	2.18	8.81E-03	transcription_start_site	+	192188	192188	-4773	10656	AN1872
721	CONTIG29	187278	187552	2.18	8.81E-03	transcription_start_site	+	192398	192398	-4983	10657	AN1872
2395	CONTIG29	224184	224528	1.24	1.93E-01	transcription_start_site	-	221300	221300	-3056	10683	AN1879
2395	CONTIG29	224184	224528	1.24	1.93E-01	transcription_start_site	-	220989	220989	-3367	10684	AN1879
2395	CONTIG29	224184	224528	1.24	1.93E-01	transcription_start_site	-	220806	220806	-3550	10685	AN1879
2395	CONTIG29	224184	224528	1.24	1.93E-01	transcription_start_site	-	220622	220622	-3734	10686	AN1879
2395	CONTIG29	224184	224528	1.24	1.93E-01	transcription_start_site	-	220027	220027	-4329	10687	AN1879
2395	CONTIG29	224184	224528	1.24	1.93E-01	transcription_start_site	-	219790	219790	-4566	10688	AN1879
2395	CONTIG29	224184	224528	1.24	1.93E-01	transcription_start_site	+	223483	223483	873	10690	AN1880
2395	CONTIG29	224184	224528	1.24	1.93E-01	transcription_start_site	+	223366	223366	990	10689	AN1880
2395	CONTIG29	224184	224528	1.24	1.93E-01	transcription_start_site	+	228548	228548	-4192	10691	AN1881
2395	CONTIG29	224184	224528	1.24	1.93E-01	transcription_start_site	+	229250	229250	-4894	10692	AN1881
1045	CONTIG29	257637	258064	1.88	1.44E-02	transcription_start_site	-	256444	256444	-1406	10733	AN1893
1045	CONTIG29	257637	258064	1.88	1.44E-02	transcription_start_site	-	256167	256167	-1683	10734	AN1893
1045	CONTIG29	257637	258064	1.88	1.44E-02	transcription_start_site	-	255755	255755	-2095	10735	AN1893
1045	CONTIG29	257637	258064	1.88	1.44E-02	transcription_start_site	-	254766	254766	-3084	10736	AN1893
1045	CONTIG29	257637	258064	1.88	1.44E-02	transcription_start_site	+	258483	258483	-632	10737	AN1894
1045	CONTIG29	257637	258064	1.88	1.44E-02	transcription_start_site	+	258722	258722	-871	10738	AN1894
1045	CONTIG29	257637	258064	1.88	1.44E-02	transcription_start_site	+	261357	261357	-3506	10739	AN1895
507	CONTIG29	276226	276595	2.43	4.46E-04	transcription_start_site	-	271445	271445	-4965	10750	AN1900
160	CONTIG29	282976	283269	3.03	2.39E-04	transcription_start_site	-	282330	282330	-792	10762	AN1902

160	CONTIG29	282976	283269	3.03	2.39E-04	transcription_start_site	-	282184	282184	-938	10763	AN1902
231	CONTIG29	286435	286929	2.88	5.64E-04	transcription_start_site	-	282330	282330	-4352	10762	AN1902
231	CONTIG29	286435	286929	2.88	5.64E-04	transcription_start_site	-	282184	282184	-4498	10763	AN1902
930	CONTIG29	329636	330058	1.98	1.84E-02	transcription_start_site	-	329388	329388	-459	10813	AN1917
930	CONTIG29	329636	330058	1.98	1.84E-02	transcription_start_site	-	328419	328419	-1428	10814	AN1917
1387	CONTIG29	334286	334720	1.64	5.88E-02	transcription_start_site	-	329388	329388	-5115	10813	AN1917
54	CONTIG29	336301	337640	3.42	0.00E+00	transcription_start_site	-	336073	336073	-897	10815	AN1918
54	CONTIG29	336301	337640	3.42	0.00E+00	transcription_start_site	-	335829	335829	-1141	10816	AN1918
54	CONTIG29	336301	337640	3.42	0.00E+00	transcription_start_site	-	335692	335692	-1278	10817	AN1918
54	CONTIG29	336301	337640	3.42	0.00E+00	transcription_start_site	-	335218	335218	-1752	10818	AN1918
1387	CONTIG29	334286	334720	1.64	5.88E-02	transcription_start_site	-	335218	335218	715	10818	AN1918
1387	CONTIG29	334286	334720	1.64	5.88E-02	transcription_start_site	-	335692	335692	1189	10817	AN1918
1495	CONTIG29	348527	348961	1.59	6.84E-02	transcription_start_site	-	345454	345454	-3290	10836	AN1921
1495	CONTIG29	348527	348961	1.59	6.84E-02	transcription_start_site	-	345258	345258	-3486	10837	AN1921
1495	CONTIG29	348527	348961	1.59	6.84E-02	transcription_start_site	-	344722	344722	-4022	10838	AN1921
1495	CONTIG29	348527	348961	1.59	6.84E-02	transcription_start_site	+	347863	347863	881	10841	AN1922
1495	CONTIG29	348527	348961	1.59	6.84E-02	transcription_start_site	+	349026	349026	-282	10842	AN1923
1495	CONTIG29	348527	348961	1.59	6.84E-02	transcription_start_site	+	349487	349487	-743	10843	AN1923
1495	CONTIG29	348527	348961	1.59	6.84E-02	transcription_start_site	+	350789	350789	-2045	10844	AN1923
1839	CONTIG29	350937	351738	1.44	1.08E-01	transcription_start_site	+	350789	350789	548	10844	AN1923
259	CONTIG29	355807	356166	2.83	8.69E-04	transcription_start_site	-	354300	354300	-1686	10845	AN1924
259	CONTIG29	355807	356166	2.83	8.69E-04	transcription_start_site	-	354168	354168	-1818	10846	AN1924
259	CONTIG29	355807	356166	2.83	8.69E-04	transcription_start_site	-	353939	353939	-2047	10847	AN1924
259	CONTIG29	355807	356166	2.83	8.69E-04	transcription_start_site	-	356293	356293	306	10850	AN1925
259	CONTIG29	355807	356166	2.83	8.69E-04	transcription_start_site	-	356368	356368	381	10849	AN1925
259	CONTIG29	355807	356166	2.83	8.69E-04	transcription_start_site	-	356559	356559	572	10848	AN1925
182	CONTIG29	371030	371539	2.98	2.16E-04	transcription_start_site	-	366385	366385	-4899	10860	AN1928
182	CONTIG29	371030	371539	2.98	2.16E-04	transcription_start_site	+	372426	372426	-1141	10867	AN1930
182	CONTIG29	371030	371539	2.98	2.16E-04	transcription_start_site	+	372645	372645	-1360	10868	AN1930
182	CONTIG29	371030	371539	2.98	2.16E-04	transcription_start_site	+	372758	372758	-1473	10869	AN1930
182	CONTIG29	371030	371539	2.98	2.16E-04	transcription_start_site	+	372903	372903	-1618	10870	AN1930
182	CONTIG29	371030	371539	2.98	2.16E-04	transcription_start_site	+	373324	373324	-2039	10871	AN1930
182	CONTIG29	371030	371539	2.98	2.16E-04	transcription_start_site	+	373456	373456	-2171	10872	AN1930
979	CONTIG29	378001	378295	1.93	2.12E-02	transcription_start_site	-	376414	376414	-1734	10873	AN1931
979	CONTIG29	378001	378295	1.93	2.12E-02	transcription_start_site	-	376225	376225	-1923	10874	AN1931
979	CONTIG29	378001	378295	1.93	2.12E-02	transcription_start_site	-	376000	376000	-2148	10875	AN1931
979	CONTIG29	378001	378295	1.93	2.12E-02	transcription_start_site	-	375900	375900	-2248	10876	AN1931
979	CONTIG29	378001	378295	1.93	2.12E-02	transcription_start_site	-	375743	375743	-2405	10877	AN1931
1046	CONTIG29	374795	375144	1.88	1.44E-02	transcription_start_site	-	375743	375743	773	10877	AN1931
1046	CONTIG29	374795	375144	1.88	1.44E-02	transcription_start_site	-	375900	375900	930	10876	AN1931
1046	CONTIG29	374795	375144	1.88	1.44E-02	transcription_start_site	-	376000	376000	1030	10875	AN1931
2093	CONTIG29	382506	382933	1.34	1.44E-01	transcription_start_site	-	382667	382667	-52	10878	AN1932
2093	CONTIG29	382506	382933	1.34	1.44E-01	transcription_start_site	-	382462	382462	-257	10879	AN1932
2093	CONTIG29	382506	382933	1.34	1.44E-01	transcription_start_site	+	383942	383942	-1222	10880	AN1933
2093	CONTIG29	382506	382933	1.34	1.44E-01	transcription_start_site	+	384238	384238	-1518	10881	AN1933
2093	CONTIG29	382506	382933	1.34	1.44E-01	transcription_start_site	+	384676	384676	-1956	10882	AN1933
2093	CONTIG29	382506	382933	1.34	1.44E-01	transcription_start_site	+	385635	385635	-2915	10883	AN1934
2093	CONTIG29	382506	382933	1.34	1.44E-01	transcription_start_site	+	385822	385822	-3102	10884	AN1934
143	CONTIG29	393305	393579	3.07	2.67E-04	transcription_start_site	-	389780	389780	-3662	10885	AN1935
143	CONTIG29	393305	393579	3.07	2.67E-04	transcription_start_site	-	389429	389429	-4013	10886	AN1935
2094	CONTIG29	388070	388409	1.34	1.44E-01	transcription_start_site	+	390254	390254	-2014	10887	AN1936
2094	CONTIG29	388070	388409	1.34	1.44E-01	transcription_start_site	+	390394	390394	-2154	10888	AN1936
2094	CONTIG29	388070	388409	1.34	1.44E-01	transcription_start_site	+	390610	390610	-2370	10889	AN1936
143	CONTIG29	393305	393579	3.07	2.67E-04	transcription_start_site	-	393221	393221	-221	10890	AN1937
143	CONTIG29	393305	393579	3.07	2.67E-04	transcription_start_site	+	398251	398251	-4809	10891	AN1938
143	CONTIG29	393305	393579	3.07	2.67E-04	transcription_start_site	+	398358	398358	-4916	10892	AN1938
1840	CONTIG29	401341	401620	1.44	1.08E-01	transcription_start_site	+	402075	402075	-594	10894	AN1939

1958	CONTIG29	405696	406175	1.39	1.25E-01	transcription_start_site	+	406741	406741	-805	10895	AN1940
1958	CONTIG29	405696	406175	1.39	1.25E-01	transcription_start_site	+	409080	409080	-3144	10896	AN1940
1164	CONTIG29	430363	430632	1.79	3.70E-02	transcription_start_site	-	428466	428466	-2031	10916	AN1948
588	CONTIG29	435677	436111	2.33	2.32E-03	transcription_start_site	-	432924	432924	-2970	10919	AN1949
588	CONTIG29	435677	436111	2.33	2.32E-03	transcription_start_site	-	432261	432261	-3633	10920	AN1949
588	CONTIG29	435677	436111	2.33	2.32E-03	transcription_start_site	-	431490	431490	-4404	10921	AN1949
588	CONTIG29	435677	436111	2.33	2.32E-03	transcription_start_site	-	431162	431162	-4732	10922	AN1949
1164	CONTIG29	430363	430632	1.79	3.70E-02	transcription_start_site	-	431162	431162	664	10922	AN1949
1164	CONTIG29	430363	430632	1.79	3.70E-02	transcription_start_site	-	431490	431490	992	10921	AN1949
588	CONTIG29	435677	436111	2.33	2.32E-03	transcription_start_site	+	436765	436765	-871	10923	AN1950
588	CONTIG29	435677	436111	2.33	2.32E-03	transcription_start_site	+	437188	437188	-1294	10924	AN1950
588	CONTIG29	435677	436111	2.33	2.32E-03	transcription_start_site	+	440095	440095	-4201	10925	AN1951
588	CONTIG29	435677	436111	2.33	2.32E-03	transcription_start_site	+	440231	440231	-4337	10926	AN1951
588	CONTIG29	435677	436111	2.33	2.32E-03	transcription_start_site	+	440417	440417	-4523	10927	AN1951
588	CONTIG29	435677	436111	2.33	2.32E-03	transcription_start_site	+	440533	440533	-4639	10928	AN1951
416	CONTIG29	443254	443753	2.58	1.80E-03	transcription_start_site	+	444024	444024	-520	10929	AN1952
1496	CONTIG29	442129	442651	1.59	6.84E-02	transcription_start_site	+	444024	444024	-1634	10929	AN1952
873	CONTIG29	461416	461765	2.03	1.57E-02	transcription_start_site	-	458600	458600	-2990	10937	AN1956
873	CONTIG29	461416	461765	2.03	1.57E-02	transcription_start_site	-	458329	458329	-3261	10938	AN1956
873	CONTIG29	461416	461765	2.03	1.57E-02	transcription_start_site	+	461058	461058	532	10942	AN1957
873	CONTIG29	461416	461765	2.03	1.57E-02	transcription_start_site	+	460621	460621	969	10941	AN1957
18	CONTIG29	464418	464903	3.62	0.00E+00	transcription_start_site	+	464432	464432	228	10944	AN1958
18	CONTIG29	464418	464903	3.62	0.00E+00	transcription_start_site	+	464152	464152	508	10943	AN1958
873	CONTIG29	461416	461765	2.03	1.57E-02	transcription_start_site	+	464152	464152	-2561	10943	AN1958
873	CONTIG29	461416	461765	2.03	1.57E-02	transcription_start_site	+	464432	464432	-2841	10944	AN1958
18	CONTIG29	464418	464903	3.62	0.00E+00	transcription_start_site	+	465956	465956	-1295	10945	AN1959
18	CONTIG29	464418	464903	3.62	0.00E+00	transcription_start_site	+	466089	466089	-1428	10946	AN1959
18	CONTIG29	464418	464903	3.62	0.00E+00	transcription_start_site	+	466198	466198	-1537	10947	AN1959
18	CONTIG29	464418	464903	3.62	0.00E+00	transcription_start_site	+	466419	466419	-1758	10948	AN1959
18	CONTIG29	464418	464903	3.62	0.00E+00	transcription_start_site	+	466578	466578	-1917	10949	AN1959
18	CONTIG29	464418	464903	3.62	0.00E+00	transcription_start_site	+	466710	466710	-2049	10950	AN1959
18	CONTIG29	464418	464903	3.62	0.00E+00	transcription_start_site	+	466843	466843	-2182	10951	AN1959
18	CONTIG29	464418	464903	3.62	0.00E+00	transcription_start_site	+	467009	467009	-2348	10952	AN1959
18	CONTIG29	464418	464903	3.62	0.00E+00	transcription_start_site	+	467449	467449	-2788	10953	AN1959
18	CONTIG29	464418	464903	3.62	0.00E+00	transcription_start_site	+	467636	467636	-2975	10954	AN1959
144	CONTIG29	465542	465816	3.07	2.67E-04	transcription_start_site	+	465956	465956	-277	10945	AN1959
144	CONTIG29	465542	465816	3.07	2.67E-04	transcription_start_site	+	466089	466089	-410	10946	AN1959
144	CONTIG29	465542	465816	3.07	2.67E-04	transcription_start_site	+	466198	466198	-519	10947	AN1959
144	CONTIG29	465542	465816	3.07	2.67E-04	transcription_start_site	+	466419	466419	-740	10948	AN1959
144	CONTIG29	465542	465816	3.07	2.67E-04	transcription_start_site	+	466578	466578	-899	10949	AN1959
144	CONTIG29	465542	465816	3.07	2.67E-04	transcription_start_site	+	466710	466710	-1031	10950	AN1959
144	CONTIG29	465542	465816	3.07	2.67E-04	transcription_start_site	+	466843	466843	-1164	10951	AN1959
144	CONTIG29	465542	465816	3.07	2.67E-04	transcription_start_site	+	467009	467009	-1330	10952	AN1959
144	CONTIG29	465542	465816	3.07	2.67E-04	transcription_start_site	+	467449	467449	-1770	10953	AN1959
144	CONTIG29	465542	465816	3.07	2.67E-04	transcription_start_site	+	467636	467636	-1957	10954	AN1959
873	CONTIG29	461416	461765	2.03	1.57E-02	transcription_start_site	+	465956	465956	-4365	10945	AN1959
873	CONTIG29	461416	461765	2.03	1.57E-02	transcription_start_site	+	466089	466089	-4498	10946	AN1959
873	CONTIG29	461416	461765	2.03	1.57E-02	transcription_start_site	+	466198	466198	-4607	10947	AN1959
873	CONTIG29	461416	461765	2.03	1.57E-02	transcription_start_site	+	466419	466419	-4828	10948	AN1959
873	CONTIG29	461416	461765	2.03	1.57E-02	transcription_start_site	+	466578	466578	-4987	10949	AN1959
873	CONTIG29	461416	461765	2.03	1.57E-02	transcription_start_site	+	466710	466710	-5119	10950	AN1959
18	CONTIG29	464418	464903	3.62	0.00E+00	transcription_start_site	+	468518	468518	-3857	10955	AN1960
18	CONTIG29	464418	464903	3.62	0.00E+00	transcription_start_site	+	468702	468702	-4041	10956	AN1960
144	CONTIG29	465542	465816	3.07	2.67E-04	transcription_start_site	+	468518	468518	-2839	10955	AN1960
144	CONTIG29	465542	465816	3.07	2.67E-04	transcription_start_site	+	468702	468702	-3023	10956	AN1960
1003	CONTIG30	14868	15355	1.91	1.57E-02	transcription_start_site	-	13758	13758	-1353	11033	AN1983
1003	CONTIG30	14868	15355	1.91	1.57E-02	transcription_start_site	-	13153	13153	-1958	11034	AN1983

1003	CONTIG30	14868	15355	1.91	1.57E-02	transcription_start_site	+	15866	15866	-754	11035	AN1984
1003	CONTIG30	14868	15355	1.91	1.57E-02	transcription_start_site	+	16031	16031	-919	11036	AN1984
1003	CONTIG30	14868	15355	1.91	1.57E-02	transcription_start_site	+	18078	18078	-2966	11037	AN1984
1502	CONTIG30	48152	49336	1.58	2.48E-02	transcription_start_site	-	47912	47912	-832	11058	AN1994
1502	CONTIG30	48152	49336	1.58	2.48E-02	transcription_start_site	-	47728	47728	-1016	11059	AN1994
1502	CONTIG30	48152	49336	1.58	2.48E-02	transcription_start_site	-	47485	47485	-1259	11060	AN1994
1502	CONTIG30	48152	49336	1.58	2.48E-02	transcription_start_site	-	46892	46892	-1852	11061	AN1994
1502	CONTIG30	48152	49336	1.58	2.48E-02	transcription_start_site	+	48824	48824	-80	11062	AN1995
1502	CONTIG30	48152	49336	1.58	2.48E-02	transcription_start_site	+	49029	49029	-285	11063	AN1995
1502	CONTIG30	48152	49336	1.58	2.48E-02	transcription_start_site	+	49283	49283	-539	11064	AN1995
1502	CONTIG30	48152	49336	1.58	2.48E-02	transcription_start_site	+	52680	52680	-3936	11065	AN1996
1502	CONTIG30	48152	49336	1.58	2.48E-02	transcription_start_site	+	52805	52805	-4061	11066	AN1996
205	CONTIG30	60076	60378	2.93	0.00E+00	transcription_start_site	-	57449	57449	-2778	11069	AN1997
205	CONTIG30	60076	60378	2.93	0.00E+00	transcription_start_site	-	57028	57028	-3199	11070	AN1997
205	CONTIG30	60076	60378	2.93	0.00E+00	transcription_start_site	-	56829	56829	-3398	11071	AN1997
2199	CONTIG30	65707	65991	1.3	1.25E-01	transcription_start_site	-	63223	63223	-2626	11072	AN1998
2199	CONTIG30	65707	65991	1.3	1.25E-01	transcription_start_site	-	63144	63144	-2705	11073	AN1998
2341	CONTIG30	64146	64415	1.26	1.44E-01	transcription_start_site	-	63223	63223	-1057	11072	AN1998
2341	CONTIG30	64146	64415	1.26	1.44E-01	transcription_start_site	-	63144	63144	-1136	11073	AN1998
205	CONTIG30	60076	60378	2.93	0.00E+00	transcription_start_site	+	64170	64170	-3943	11074	AN1999
2341	CONTIG30	64146	64415	1.26	1.44E-01	transcription_start_site	+	64170	64170	110	11074	AN1999
128	CONTIG30	73811	74380	3.12	0.00E+00	transcription_start_site	-	69040	69040	-5055	11075	AN2000
128	CONTIG30	73811	74380	3.12	0.00E+00	transcription_start_site	-	68957	68957	-5138	11076	AN2000
708	CONTIG30	69387	69651	2.19	5.20E-03	transcription_start_site	-	69040	69040	-479	11075	AN2000
708	CONTIG30	69387	69651	2.19	5.20E-03	transcription_start_site	-	68957	68957	-562	11076	AN2000
708	CONTIG30	69387	69651	2.19	5.20E-03	transcription_start_site	-	68683	68683	-836	11077	AN2000
128	CONTIG30	73811	74380	3.12	0.00E+00	transcription_start_site	-	73418	73418	-677	11078	AN2001
128	CONTIG30	73811	74380	3.12	0.00E+00	transcription_start_site	-	72263	72263	-1832	11079	AN2001
128	CONTIG30	73811	74380	3.12	0.00E+00	transcription_start_site	-	71958	71958	-2137	11080	AN2001
128	CONTIG30	73811	74380	3.12	0.00E+00	transcription_start_site	-	71360	71360	-2735	11081	AN2001
128	CONTIG30	73811	74380	3.12	0.00E+00	transcription_start_site	-	71113	71113	-2982	11082	AN2001
128	CONTIG30	73811	74380	3.12	0.00E+00	transcription_start_site	+	76396	76396	-2300	11083	AN2002
128	CONTIG30	73811	74380	3.12	0.00E+00	transcription_start_site	+	77034	77034	-2938	11084	AN2002
128	CONTIG30	73811	74380	3.12	0.00E+00	transcription_start_site	+	77236	77236	-3140	11085	AN2002
128	CONTIG30	73811	74380	3.12	0.00E+00	transcription_start_site	+	77364	77364	-3268	11086	AN2002
128	CONTIG30	73811	74380	3.12	0.00E+00	transcription_start_site	+	77538	77538	-3442	11087	AN2002
128	CONTIG30	73811	74380	3.12	0.00E+00	transcription_start_site	+	77707	77707	-3611	11088	AN2002
128	CONTIG30	73811	74380	3.12	0.00E+00	transcription_start_site	+	78033	78033	-3937	11089	AN2002
396	CONTIG30	89253	89762	2.61	9.51E-04	transcription_start_site	-	86144	86144	-3363	11090	AN2004
396	CONTIG30	89253	89762	2.61	9.51E-04	transcription_start_site	-	85982	85982	-3525	11091	AN2004
396	CONTIG30	89253	89762	2.61	9.51E-04	transcription_start_site	-	85397	85397	-4110	11092	AN2004
396	CONTIG30	89253	89762	2.61	9.51E-04	transcription_start_site	-	88569	88569	-938	11093	AN2005
396	CONTIG30	89253	89762	2.61	9.51E-04	transcription_start_site	-	88466	88466	-1041	11094	AN2005
396	CONTIG30	89253	89762	2.61	9.51E-04	transcription_start_site	-	88250	88250	-1257	11095	AN2005
265	CONTIG32	7880	8229	2.81	6.10E-04	transcription_start_site	-	6198	6198	-1856	11104	AN2009
265	CONTIG32	7880	8229	2.81	6.10E-04	transcription_start_site	-	5810	5810	-2244	11105	AN2009
265	CONTIG32	7880	8229	2.81	6.10E-04	transcription_start_site	-	5550	5550	-2504	11106	AN2009
1128	CONTIG32	18611	18889	1.81	2.62E-02	transcription_start_site	-	18136	18136	-614	11109	AN2011
2270	CONTIG32	22296	22720	1.28	1.44E-01	transcription_start_site	-	18136	18136	-4372	11109	AN2011
2270	CONTIG32	22296	22720	1.28	1.44E-01	transcription_start_site	-	22337	22337	-171	11113	AN2012
2270	CONTIG32	22296	22720	1.28	1.44E-01	transcription_start_site	-	23061	23061	553	11112	AN2012
2270	CONTIG32	22296	22720	1.28	1.44E-01	transcription_start_site	-	23294	23294	786	11111	AN2012
2270	CONTIG32	22296	22720	1.28	1.44E-01	transcription_start_site	-	23476	23476	968	11110	AN2012
2270	CONTIG32	22296	22720	1.28	1.44E-01	transcription_start_site	-	21400	21400	-1108	11114	AN2012
2270	CONTIG32	22296	22720	1.28	1.44E-01	transcription_start_site	+	24455	24455	-1947	11115	AN2013
304	CONTIG32	29268	29527	2.76	5.64E-04	transcription_start_site	-	27940	27940	-1457	11116	AN2014
304	CONTIG32	29268	29527	2.76	5.64E-04	transcription_start_site	-	27561	27561	-1836	11117	AN2014

304	CONTIG32	29268	29527	2.76	5.64E-04	transcription_start_site	-	27234	27234	-2163	11118	AN2014
304	CONTIG32	29268	29527	2.76	5.64E-04	transcription_start_site	-	27040	27040	-2357	11119	AN2014
1862	CONTIG32	29569	30353	1.43	7.51E-02	transcription_start_site	-	27940	27940	-2021	11116	AN2014
1862	CONTIG32	29569	30353	1.43	7.51E-02	transcription_start_site	-	27561	27561	-2400	11117	AN2014
1862	CONTIG32	29569	30353	1.43	7.51E-02	transcription_start_site	-	27234	27234	-2727	11118	AN2014
1862	CONTIG32	29569	30353	1.43	7.51E-02	transcription_start_site	-	27040	27040	-2921	11119	AN2014
304	CONTIG32	29268	29527	2.76	5.64E-04	transcription_start_site	+	29761	29761	-363	11120	AN2015
1862	CONTIG32	29569	30353	1.43	7.51E-02	transcription_start_site	+	29761	29761	200	11120	AN2015
450	CONTIG32	34669	35476	2.52	0.00E+00	transcription_start_site	-	34223	34223	-849	11121	AN2016
450	CONTIG32	34669	35476	2.52	0.00E+00	transcription_start_site	-	33202	33202	-1870	11122	AN2016
450	CONTIG32	34669	35476	2.52	0.00E+00	transcription_start_site	-	32674	32674	-2398	11123	AN2016
1530	CONTIG32	33694	33963	1.57	5.88E-02	transcription_start_site	-	34223	34223	394	11121	AN2016
1530	CONTIG32	33694	33963	1.57	5.88E-02	transcription_start_site	-	33202	33202	-626	11122	AN2016
1530	CONTIG32	33694	33963	1.57	5.88E-02	transcription_start_site	-	32674	32674	-1154	11123	AN2016
450	CONTIG32	34669	35476	2.52	0.00E+00	transcription_start_site	+	35862	35862	-789	11124	AN2017
450	CONTIG32	34669	35476	2.52	0.00E+00	transcription_start_site	+	36636	36636	-1563	11125	AN2017
450	CONTIG32	34669	35476	2.52	0.00E+00	transcription_start_site	+	37090	37090	-2017	11126	AN2017
450	CONTIG32	34669	35476	2.52	0.00E+00	transcription_start_site	+	37305	37305	-2232	11127	AN2017
1530	CONTIG32	33694	33963	1.57	5.88E-02	transcription_start_site	+	35862	35862	-2033	11124	AN2017
1530	CONTIG32	33694	33963	1.57	5.88E-02	transcription_start_site	+	36636	36636	-2807	11125	AN2017
1530	CONTIG32	33694	33963	1.57	5.88E-02	transcription_start_site	+	37090	37090	-3261	11126	AN2017
1530	CONTIG32	33694	33963	1.57	5.88E-02	transcription_start_site	+	37305	37305	-3476	11127	AN2017
450	CONTIG32	34669	35476	2.52	0.00E+00	transcription_start_site	+	39330	39330	-4257	11128	AN2018
450	CONTIG32	34669	35476	2.52	0.00E+00	transcription_start_site	+	39534	39534	-4461	11129	AN2018
450	CONTIG32	34669	35476	2.52	0.00E+00	transcription_start_site	+	39733	39733	-4660	11130	AN2018
450	CONTIG32	34669	35476	2.52	0.00E+00	transcription_start_site	+	39902	39902	-4829	11131	AN2018
450	CONTIG32	34669	35476	2.52	0.00E+00	transcription_start_site	+	40202	40202	-5129	11132	AN2018
450	CONTIG32	34669	35476	2.52	0.00E+00	transcription_start_site	+	40417	40417	-5344	11133	AN2018
16	CONTIG32	52431	53020	3.66	0.00E+00	transcription_start_site	-	48894	48894	-3831	11138	AN2020
16	CONTIG32	52431	53020	3.66	0.00E+00	transcription_start_site	-	48672	48672	-4053	11139	AN2020
16	CONTIG32	52431	53020	3.66	0.00E+00	transcription_start_site	-	47991	47991	-4734	11140	AN2020
49	CONTIG32	47032	47381	3.43	0.00E+00	transcription_start_site	-	47991	47991	784	11140	AN2020
584	CONTIG32	49202	49701	2.33	0.00E+00	transcription_start_site	-	48894	48894	-557	11138	AN2020
584	CONTIG32	49202	49701	2.33	0.00E+00	transcription_start_site	-	48672	48672	-779	11139	AN2020
584	CONTIG32	49202	49701	2.33	0.00E+00	transcription_start_site	-	47991	47991	-1460	11140	AN2020
510	CONTIG32	122258	122602	2.43	2.22E-03	transcription_start_site	-	120334	120334	-2096	11173	AN2036
510	CONTIG32	122258	122602	2.43	2.22E-03	transcription_start_site	-	120234	120234	-2196	11174	AN2036
510	CONTIG32	122258	122602	2.43	2.22E-03	transcription_start_site	-	119733	119733	-2697	11175	AN2036
510	CONTIG32	122258	122602	2.43	2.22E-03	transcription_start_site	-	119139	119139	-3291	11176	AN2036
510	CONTIG32	122258	122602	2.43	2.22E-03	transcription_start_site	-	118596	118596	-3834	11177	AN2036
510	CONTIG32	122258	122602	2.43	2.22E-03	transcription_start_site	-	123590	123590	1160	11179	AN2037
653	CONTIG32	139584	139862	2.24	5.20E-03	transcription_start_site	-	139171	139171	-552	11193	AN2042
653	CONTIG32	139584	139862	2.24	5.20E-03	transcription_start_site	-	139119	139119	-604	11194	AN2042
653	CONTIG32	139584	139862	2.24	5.20E-03	transcription_start_site	-	137149	137149	-2574	11195	AN2042
758	CONTIG32	168685	169334	2.14	3.31E-03	transcription_start_site	-	167911	167911	-1098	11239	AN2054
758	CONTIG32	168685	169334	2.14	3.31E-03	transcription_start_site	-	166672	166672	-2337	11240	AN2054
758	CONTIG32	168685	169334	2.14	3.31E-03	transcription_start_site	-	166319	166319	-2690	11241	AN2054
758	CONTIG32	168685	169334	2.14	3.31E-03	transcription_start_site	+	169638	169638	-628	11242	AN2055
758	CONTIG32	168685	169334	2.14	3.31E-03	transcription_start_site	+	170030	170030	-1020	11243	AN2055
758	CONTIG32	168685	169334	2.14	3.31E-03	transcription_start_site	+	172164	172164	-3154	11244	AN2056
758	CONTIG32	168685	169334	2.14	3.31E-03	transcription_start_site	+	172308	172308	-3298	11245	AN2056
758	CONTIG32	168685	169334	2.14	3.31E-03	transcription_start_site	+	173229	173229	-4219	11246	AN2056
1985	CONTIG32	173870	174373	1.38	1.08E-01	transcription_start_site	+	173229	173229	892	11246	AN2056
758	CONTIG32	168685	169334	2.14	3.31E-03	transcription_start_site	+	174046	174046	-5036	11247	AN2057
1985	CONTIG32	173870	174373	1.38	1.08E-01	transcription_start_site	+	174046	174046	75	11247	AN2057
1985	CONTIG32	173870	174373	1.38	1.08E-01	transcription_start_site	+	174394	174394	-272	11248	AN2057
1985	CONTIG32	173870	174373	1.38	1.08E-01	transcription_start_site	+	175798	175798	-1676	11249	AN2058

1985	CONTIG32	173870	174373	1.38	1.08E-01	transcription_start_site	+	176398	176398	-2276	11250	AN2058
484	CONTIG32	209046	210430	2.47	1.80E-03	transcription_start_site	-	205135	205135	-4603	11276	AN2066
484	CONTIG32	209046	210430	2.47	1.80E-03	transcription_start_site	-	205013	205013	-4725	11277	AN2066
484	CONTIG32	209046	210430	2.47	1.80E-03	transcription_start_site	-	204877	204877	-4861	11278	AN2066
484	CONTIG32	209046	210430	2.47	1.80E-03	transcription_start_site	-	204771	204771	-4967	11279	AN2066
484	CONTIG32	209046	210430	2.47	1.80E-03	transcription_start_site	-	204646	204646	-5092	11280	AN2066
484	CONTIG32	209046	210430	2.47	1.80E-03	transcription_start_site	-	204495	204495	-5243	11281	AN2066
484	CONTIG32	209046	210430	2.47	1.80E-03	transcription_start_site	-	204323	204323	-5415	11282	AN2066
1531	CONTIG32	207766	208035	1.57	5.88E-02	transcription_start_site	-	205135	205135	-2765	11276	AN2066
1531	CONTIG32	207766	208035	1.57	5.88E-02	transcription_start_site	-	205013	205013	-2887	11277	AN2066
1531	CONTIG32	207766	208035	1.57	5.88E-02	transcription_start_site	-	204877	204877	-3023	11278	AN2066
1531	CONTIG32	207766	208035	1.57	5.88E-02	transcription_start_site	-	204771	204771	-3129	11279	AN2066
1531	CONTIG32	207766	208035	1.57	5.88E-02	transcription_start_site	-	204646	204646	-3254	11280	AN2066
1531	CONTIG32	207766	208035	1.57	5.88E-02	transcription_start_site	-	204495	204495	-3405	11281	AN2066
1531	CONTIG32	207766	208035	1.57	5.88E-02	transcription_start_site	-	204323	204323	-3577	11282	AN2066
1531	CONTIG32	207766	208035	1.57	5.88E-02	transcription_start_site	-	203655	203655	-4245	11283	AN2066
1531	CONTIG32	207766	208035	1.57	5.88E-02	transcription_start_site	-	203394	203394	-4506	11284	AN2066
484	CONTIG32	209046	210430	2.47	1.80E-03	transcription_start_site	-	209022	209022	-716	11285	AN2067
484	CONTIG32	209046	210430	2.47	1.80E-03	transcription_start_site	-	206964	206964	-2774	11286	AN2067
484	CONTIG32	209046	210430	2.47	1.80E-03	transcription_start_site	-	206812	206812	-2926	11287	AN2067
484	CONTIG32	209046	210430	2.47	1.80E-03	transcription_start_site	-	206429	206429	-3309	11288	AN2067
1531	CONTIG32	207766	208035	1.57	5.88E-02	transcription_start_site	-	206964	206964	-936	11286	AN2067
1531	CONTIG32	207766	208035	1.57	5.88E-02	transcription_start_site	-	206812	206812	-1088	11287	AN2067
1531	CONTIG32	207766	208035	1.57	5.88E-02	transcription_start_site	-	209022	209022	1121	11285	AN2067
1531	CONTIG32	207766	208035	1.57	5.88E-02	transcription_start_site	-	206429	206429	-1471	11288	AN2067
484	CONTIG32	209046	210430	2.47	1.80E-03	transcription_start_site	+	212683	212683	-2945	11289	AN2068
484	CONTIG32	209046	210430	2.47	1.80E-03	transcription_start_site	+	213135	213135	-3397	11290	AN2068
484	CONTIG32	209046	210430	2.47	1.80E-03	transcription_start_site	+	213439	213439	-3701	11291	AN2068
1531	CONTIG32	207766	208035	1.57	5.88E-02	transcription_start_site	+	212683	212683	-4782	11289	AN2068
1651	CONTIG32	221569	221833	1.52	6.84E-02	transcription_start_site	-	218475	218475	-3226	11293	AN2069
1651	CONTIG32	221569	221833	1.52	6.84E-02	transcription_start_site	-	218282	218282	-3419	11294	AN2069
1651	CONTIG32	221569	221833	1.52	6.84E-02	transcription_start_site	-	218097	218097	-3604	11295	AN2069
2385	CONTIG32	220579	221078	1.24	1.50E-01	transcription_start_site	-	218475	218475	-2353	11293	AN2069
2385	CONTIG32	220579	221078	1.24	1.50E-01	transcription_start_site	-	218282	218282	-2546	11294	AN2069
2385	CONTIG32	220579	221078	1.24	1.50E-01	transcription_start_site	-	218097	218097	-2731	11295	AN2069
1651	CONTIG32	221569	221833	1.52	6.84E-02	transcription_start_site	-	221907	221907	206	11296	AN2070
2385	CONTIG32	220579	221078	1.24	1.50E-01	transcription_start_site	-	221907	221907	1078	11296	AN2070
1431	CONTIG32	229809	230319	1.62	5.22E-02	transcription_start_site	-	224881	224881	-5183	11297	AN2071
1651	CONTIG32	221569	221833	1.52	6.84E-02	transcription_start_site	+	225550	225550	-3849	11301	AN2072
1651	CONTIG32	221569	221833	1.52	6.84E-02	transcription_start_site	+	226067	226067	-4366	11302	AN2072
2385	CONTIG32	220579	221078	1.24	1.50E-01	transcription_start_site	+	225550	225550	-4721	11301	AN2072
2385	CONTIG32	220579	221078	1.24	1.50E-01	transcription_start_site	+	226067	226067	-5238	11302	AN2072
1431	CONTIG32	229809	230319	1.62	5.22E-02	transcription_start_site	-	231310	231310	1246	11305	AN2073
2387	CONTIG32	262894	263905	1.24	1.59E-01	transcription_start_site	-	258676	258676	-4723	11325	AN2080
2387	CONTIG32	262894	263905	1.24	1.59E-01	transcription_start_site	-	263247	263247	-152	11332	AN2082
2387	CONTIG32	262894	263905	1.24	1.59E-01	transcription_start_site	-	262168	262168	-1231	11333	AN2082
192	CONTIG32	270096	270650	2.95	0.00E+00	transcription_start_site	-	265338	265338	-5035	11334	AN2083
305	CONTIG32	269045	269320	2.76	5.64E-04	transcription_start_site	-	265338	265338	-3844	11334	AN2083
192	CONTIG32	270096	270650	2.95	0.00E+00	transcription_start_site	+	272006	272006	-1633	11335	AN2084
192	CONTIG32	270096	270650	2.95	0.00E+00	transcription_start_site	+	272724	272724	-2351	11336	AN2084
192	CONTIG32	270096	270650	2.95	0.00E+00	transcription_start_site	+	273163	273163	-2790	11337	AN2084
305	CONTIG32	269045	269320	2.76	5.64E-04	transcription_start_site	+	272006	272006	-2823	11335	AN2084
305	CONTIG32	269045	269320	2.76	5.64E-04	transcription_start_site	+	272724	272724	-3541	11336	AN2084
305	CONTIG32	269045	269320	2.76	5.64E-04	transcription_start_site	+	273163	273163	-3980	11337	AN2084
1522	CONTIG32	312769	313188	1.57	3.99E-02	transcription_start_site	-	311288	311288	-1690	11374	AN2096
1522	CONTIG32	312769	313188	1.57	3.99E-02	transcription_start_site	-	310974	310974	-2004	11375	AN2096
1522	CONTIG32	312769	313188	1.57	3.99E-02	transcription_start_site	-	310846	310846	-2132	11376	AN2096

1522	CONTIG32	312769	313188	1.57	3.99E-02	transcription_start_site	-	309129	309129	-3849	11377	AN2096
1522	CONTIG32	312769	313188	1.57	3.99E-02	transcription_start_site	-	308990	308990	-3988	11378	AN2096
1986	CONTIG32	314564	314851	1.38	1.08E-01	transcription_start_site	-	311288	311288	-3419	11374	AN2096
1986	CONTIG32	314564	314851	1.38	1.08E-01	transcription_start_site	-	310974	310974	-3733	11375	AN2096
1986	CONTIG32	314564	314851	1.38	1.08E-01	transcription_start_site	-	310846	310846	-3861	11376	AN2096
1522	CONTIG32	312769	313188	1.57	3.99E-02	transcription_start_site	-	313229	313229	250	11380	AN2097
1522	CONTIG32	312769	313188	1.57	3.99E-02	transcription_start_site	-	312613	312613	-365	11381	AN2097
1522	CONTIG32	312769	313188	1.57	3.99E-02	transcription_start_site	-	313425	313425	446	11379	AN2097
1522	CONTIG32	312769	313188	1.57	3.99E-02	transcription_start_site	-	312384	312384	-594	11382	AN2097
1986	CONTIG32	314564	314851	1.38	1.08E-01	transcription_start_site	-	313425	313425	-1282	11379	AN2097
1986	CONTIG32	314564	314851	1.38	1.08E-01	transcription_start_site	-	313229	313229	-1478	11380	AN2097
1986	CONTIG32	314564	314851	1.38	1.08E-01	transcription_start_site	-	312613	312613	-2094	11381	AN2097
1986	CONTIG32	314564	314851	1.38	1.08E-01	transcription_start_site	-	312384	312384	-2323	11382	AN2097
1522	CONTIG32	312769	313188	1.57	3.99E-02	transcription_start_site	+	313825	313825	-846	11383	AN2098
1522	CONTIG32	312769	313188	1.57	3.99E-02	transcription_start_site	+	313908	313908	-929	11384	AN2098
1522	CONTIG32	312769	313188	1.57	3.99E-02	transcription_start_site	+	313973	313973	-994	11385	AN2098
1522	CONTIG32	312769	313188	1.57	3.99E-02	transcription_start_site	+	314072	314072	-1093	11386	AN2098
1522	CONTIG32	312769	313188	1.57	3.99E-02	transcription_start_site	+	314222	314222	-1243	11387	AN2098
1986	CONTIG32	314564	314851	1.38	1.08E-01	transcription_start_site	+	314222	314222	485	11387	AN2098
1986	CONTIG32	314564	314851	1.38	1.08E-01	transcription_start_site	+	314072	314072	635	11386	AN2098
1986	CONTIG32	314564	314851	1.38	1.08E-01	transcription_start_site	+	313973	313973	734	11385	AN2098
1986	CONTIG32	314564	314851	1.38	1.08E-01	transcription_start_site	+	313908	313908	799	11384	AN2098
1986	CONTIG32	314564	314851	1.38	1.08E-01	transcription_start_site	+	313825	313825	882	11383	AN2098
1522	CONTIG32	312769	313188	1.57	3.99E-02	transcription_start_site	+	315316	315316	-2337	11388	AN2099
1522	CONTIG32	312769	313188	1.57	3.99E-02	transcription_start_site	+	315628	315628	-2649	11389	AN2099
1522	CONTIG32	312769	313188	1.57	3.99E-02	transcription_start_site	+	315979	315979	-3000	11390	AN2099
1986	CONTIG32	314564	314851	1.38	1.08E-01	transcription_start_site	+	315316	315316	-608	11388	AN2099
1986	CONTIG32	314564	314851	1.38	1.08E-01	transcription_start_site	+	315628	315628	-920	11389	AN2099
1986	CONTIG32	314564	314851	1.38	1.08E-01	transcription_start_site	+	315979	315979	-1271	11390	AN2099
1522	CONTIG32	312769	313188	1.57	3.99E-02	transcription_start_site	+	317381	317381	-4402	11391	AN2100
1986	CONTIG32	314564	314851	1.38	1.08E-01	transcription_start_site	+	317381	317381	-2673	11391	AN2100
814	CONTIG32	320264	320528	2.09	8.81E-03	transcription_start_site	+	319888	319888	508	11392	AN2101
1781	CONTIG32	322294	322568	1.47	8.01E-02	transcription_start_site	-	323313	323313	882	11395	AN2102
1781	CONTIG32	322294	322568	1.47	8.01E-02	transcription_start_site	-	323428	323428	997	11394	AN2102
814	CONTIG32	320264	320528	2.09	8.81E-03	transcription_start_site	+	325094	325094	-4698	11399	AN2104
1781	CONTIG32	322294	322568	1.47	8.01E-02	transcription_start_site	+	325094	325094	-2663	11399	AN2104
266	CONTIG32	340436	340795	2.81	6.10E-04	transcription_start_site	+	343418	343418	-2802	11407	AN2108
266	CONTIG32	340436	340795	2.81	6.10E-04	transcription_start_site	+	343644	343644	-3028	11408	AN2108
266	CONTIG32	340436	340795	2.81	6.10E-04	transcription_start_site	+	344125	344125	-3509	11409	AN2108
266	CONTIG32	340436	340795	2.81	6.10E-04	transcription_start_site	+	344351	344351	-3735	11410	AN2108
266	CONTIG32	340436	340795	2.81	6.10E-04	transcription_start_site	+	344503	344503	-3887	11411	AN2108
266	CONTIG32	340436	340795	2.81	6.10E-04	transcription_start_site	+	344600	344600	-3984	11412	AN2108
1334	CONTIG32	339231	339590	1.67	4.36E-02	transcription_start_site	+	343418	343418	-4007	11407	AN2108
1334	CONTIG32	339231	339590	1.67	4.36E-02	transcription_start_site	+	343644	343644	-4233	11408	AN2108
1334	CONTIG32	339231	339590	1.67	4.36E-02	transcription_start_site	+	344125	344125	-4714	11409	AN2108
1334	CONTIG32	339231	339590	1.67	4.36E-02	transcription_start_site	+	344351	344351	-4940	11410	AN2108
1334	CONTIG32	339231	339590	1.67	4.36E-02	transcription_start_site	+	344503	344503	-5092	11411	AN2108
2389	CONTIG32	357676	358100	1.24	1.66E-01	transcription_start_site	-	352942	352942	-4946	11413	AN2110
151	CONTIG32	362721	363055	3.04	0.00E+00	transcription_start_site	-	359115	359115	-3773	11420	AN2113
151	CONTIG32	362721	363055	3.04	0.00E+00	transcription_start_site	-	358839	358839	-4049	11421	AN2113
151	CONTIG32	362721	363055	3.04	0.00E+00	transcription_start_site	-	358630	358630	-4258	11422	AN2113
151	CONTIG32	362721	363055	3.04	0.00E+00	transcription_start_site	-	357973	357973	-4915	11423	AN2113
1432	CONTIG32	359721	360280	1.62	5.22E-02	transcription_start_site	-	359115	359115	-885	11420	AN2113
1432	CONTIG32	359721	360280	1.62	5.22E-02	transcription_start_site	-	358839	358839	-1161	11421	AN2113
1432	CONTIG32	359721	360280	1.62	5.22E-02	transcription_start_site	-	358630	358630	-1370	11422	AN2113
1432	CONTIG32	359721	360280	1.62	5.22E-02	transcription_start_site	-	357973	357973	-2027	11423	AN2113
2389	CONTIG32	357676	358100	1.24	1.66E-01	transcription_start_site	-	357973	357973	85	11423	AN2113

2389	CONTIG32	357676	358100	1.24	1.66E-01	transcription_start_site	-	358630	358630	742	11422	AN2113
2389	CONTIG32	357676	358100	1.24	1.66E-01	transcription_start_site	-	358839	358839	951	11421	AN2113
151	CONTIG32	362721	363055	3.04	0.00E+00	transcription_start_site	+	364723	364723	-1835	11424	AN2114
151	CONTIG32	362721	363055	3.04	0.00E+00	transcription_start_site	+	365487	365487	-2599	11425	AN2114
151	CONTIG32	362721	363055	3.04	0.00E+00	transcription_start_site	+	365766	365766	-2878	11426	AN2114
1432	CONTIG32	359721	360280	1.62	5.22E-02	transcription_start_site	+	364723	364723	-4722	11424	AN2114
1864	CONTIG32	426162	426507	1.43	9.24E-02	transcription_start_site	-	425993	425993	-341	11471	AN2130
1864	CONTIG32	426162	426507	1.43	9.24E-02	transcription_start_site	-	422772	422772	-3562	11472	AN2130
1864	CONTIG32	426162	426507	1.43	9.24E-02	transcription_start_site	-	422477	422477	-3857	11473	AN2130
434	CONTIG34	7351	7722	2.54	2.16E-04	transcription_start_site	-	3195	3195	-4341	11476	AN2132
1743	CONTIG34	2476	3065	1.48	2.64E-02	transcription_start_site	-	3195	3195	424	11476	AN2132
1743	CONTIG34	2476	3065	1.48	2.64E-02	transcription_start_site	-	1794	1794	-976	11477	AN2132
1743	CONTIG34	2476	3065	1.48	2.64E-02	transcription_start_site	-	1755	1755	-1015	11478	AN2132
2746	CONTIG34	4146	4711	1.1	1.50E-01	transcription_start_site	-	3195	3195	-1233	11476	AN2132
2746	CONTIG34	4146	4711	1.1	1.50E-01	transcription_start_site	-	1794	1794	-2634	11477	AN2132
2746	CONTIG34	4146	4711	1.1	1.50E-01	transcription_start_site	-	1755	1755	-2673	11478	AN2132
1743	CONTIG34	2476	3065	1.48	2.64E-02	transcription_start_site	+	3640	3640	-869	11479	AN2133
1743	CONTIG34	2476	3065	1.48	2.64E-02	transcription_start_site	+	3876	3876	-1105	11480	AN2133
1743	CONTIG34	2476	3065	1.48	2.64E-02	transcription_start_site	+	4033	4033	-1262	11481	AN2133
2746	CONTIG34	4146	4711	1.1	1.50E-01	transcription_start_site	+	4033	4033	395	11481	AN2133
2746	CONTIG34	4146	4711	1.1	1.50E-01	transcription_start_site	+	3876	3876	552	11480	AN2133
2746	CONTIG34	4146	4711	1.1	1.50E-01	transcription_start_site	+	3640	3640	788	11479	AN2133
434	CONTIG34	7351	7722	2.54	2.16E-04	transcription_start_site	-	6844	6844	-692	11482	AN2134
434	CONTIG34	7351	7722	2.54	2.16E-04	transcription_start_site	-	6117	6117	-1419	11483	AN2134
434	CONTIG34	7351	7722	2.54	2.16E-04	transcription_start_site	-	6051	6051	-1485	11484	AN2134
434	CONTIG34	7351	7722	2.54	2.16E-04	transcription_start_site	-	5844	5844	-1692	11485	AN2134
434	CONTIG34	7351	7722	2.54	2.16E-04	transcription_start_site	-	5735	5735	-1801	11486	AN2134
434	CONTIG34	7351	7722	2.54	2.16E-04	transcription_start_site	-	5371	5371	-2165	11487	AN2134
434	CONTIG34	7351	7722	2.54	2.16E-04	transcription_start_site	-	4957	4957	-2579	11488	AN2134
1306	CONTIG34	9021	9575	1.69	1.84E-02	transcription_start_site	-	6844	6844	-2454	11482	AN2134
1306	CONTIG34	9021	9575	1.69	1.84E-02	transcription_start_site	-	6117	6117	-3181	11483	AN2134
1306	CONTIG34	9021	9575	1.69	1.84E-02	transcription_start_site	-	6051	6051	-3247	11484	AN2134
1306	CONTIG34	9021	9575	1.69	1.84E-02	transcription_start_site	-	5844	5844	-3454	11485	AN2134
1306	CONTIG34	9021	9575	1.69	1.84E-02	transcription_start_site	-	5735	5735	-3563	11486	AN2134
1306	CONTIG34	9021	9575	1.69	1.84E-02	transcription_start_site	-	5371	5371	-3927	11487	AN2134
1306	CONTIG34	9021	9575	1.69	1.84E-02	transcription_start_site	-	4957	4957	-4341	11488	AN2134
2746	CONTIG34	4146	4711	1.1	1.50E-01	transcription_start_site	-	4957	4957	528	11488	AN2134
2746	CONTIG34	4146	4711	1.1	1.50E-01	transcription_start_site	-	5371	5371	942	11487	AN2134
1306	CONTIG34	9021	9575	1.69	1.84E-02	transcription_start_site	-	9544	9544	246	11489	AN2135
789	CONTIG34	42920	43254	2.11	3.03E-03	transcription_start_site	-	39010	39010	-4077	11510	AN2143
789	CONTIG34	42920	43254	2.11	3.03E-03	transcription_start_site	-	38453	38453	-4634	11511	AN2143
789	CONTIG34	42920	43254	2.11	3.03E-03	transcription_start_site	-	42921	42921	-166	11513	AN2145
789	CONTIG34	42920	43254	2.11	3.03E-03	transcription_start_site	-	42450	42450	-637	11514	AN2145
789	CONTIG34	42920	43254	2.11	3.03E-03	transcription_start_site	+	44646	44646	-1559	11515	AN2146
789	CONTIG34	42920	43254	2.11	3.03E-03	transcription_start_site	+	44723	44723	-1636	11516	AN2146
2748	CONTIG34	62401	62675	1.1	1.66E-01	transcription_start_site	-	61735	61735	-803	11534	AN2151
1244	CONTIG34	86177	86461	1.73	1.57E-02	transcription_start_site	+	88745	88745	-2426	11560	AN2160
1244	CONTIG34	86177	86461	1.73	1.57E-02	transcription_start_site	+	88932	88932	-2613	11561	AN2160
1244	CONTIG34	86177	86461	1.73	1.57E-02	transcription_start_site	+	89797	89797	-3478	11562	AN2160
1244	CONTIG34	86177	86461	1.73	1.57E-02	transcription_start_site	+	90049	90049	-3730	11563	AN2160
2289	CONTIG34	97805	98155	1.27	9.24E-02	transcription_start_site	-	97503	97503	-477	11568	AN2163
2817	CONTIG34	99084	99428	1.06	1.93E-01	transcription_start_site	-	97503	97503	-1753	11568	AN2163
2289	CONTIG34	97805	98155	1.27	9.24E-02	transcription_start_site	+	99581	99581	-1601	11569	AN2164
2817	CONTIG34	99084	99428	1.06	1.93E-01	transcription_start_site	+	99581	99581	-325	11569	AN2164
868	CONTIG34	102994	103478	2.03	4.61E-03	transcription_start_site	+	103453	103453	-217	11570	AN2165
868	CONTIG34	102994	103478	2.03	4.61E-03	transcription_start_site	+	104390	104390	-1154	11571	AN2165
2666	CONTIG34	104784	105058	1.14	1.44E-01	transcription_start_site	+	104390	104390	531	11571	AN2165

2817	CONTIG34	99084	99428	1.06	1.93E-01	transcription_start_site	+	103453	103453	-4197	11570 AN2165
2817	CONTIG34	99084	99428	1.06	1.93E-01	transcription_start_site	+	104390	104390	-5134	11571 AN2165
2050	CONTIG34	107559	108818	1.35	6.84E-02	transcription_start_site	-	105792	105792	-2396	11572 AN2166
2666	CONTIG34	104784	105058	1.14	1.44E-01	transcription_start_site	-	105792	105792	871	11572 AN2166
868	CONTIG34	102994	103478	2.03	4.61E-03	transcription_start_site	+	106334	106334	-3098	11573 AN2167
868	CONTIG34	102994	103478	2.03	4.61E-03	transcription_start_site	+	106496	106496	-3260	11574 AN2167
868	CONTIG34	102994	103478	2.03	4.61E-03	transcription_start_site	+	106710	106710	-3474	11575 AN2167
868	CONTIG34	102994	103478	2.03	4.61E-03	transcription_start_site	+	106921	106921	-3685	11576 AN2167
2050	CONTIG34	107559	108818	1.35	6.84E-02	transcription_start_site	+	106921	106921	1267	11576 AN2167
2050	CONTIG34	107559	108818	1.35	6.84E-02	transcription_start_site	+	106710	106710	1478	11575 AN2167
2666	CONTIG34	104784	105058	1.14	1.44E-01	transcription_start_site	+	106334	106334	-1413	11573 AN2167
2666	CONTIG34	104784	105058	1.14	1.44E-01	transcription_start_site	+	106496	106496	-1575	11574 AN2167
2666	CONTIG34	104784	105058	1.14	1.44E-01	transcription_start_site	+	106710	106710	-1789	11575 AN2167
2666	CONTIG34	104784	105058	1.14	1.44E-01	transcription_start_site	+	106921	106921	-2000	11576 AN2167
1365	CONTIG34	113194	113614	1.65	1.06E-02	transcription_start_site	-	110815	110815	-2589	11577 AN2168
1365	CONTIG34	113194	113614	1.65	1.06E-02	transcription_start_site	-	110175	110175	-3229	11578 AN2168
2290	CONTIG34	112054	113153	1.27	9.24E-02	transcription_start_site	-	110815	110815	-1788	11577 AN2168
2290	CONTIG34	112054	113153	1.27	9.24E-02	transcription_start_site	-	110175	110175	-2428	11578 AN2168
2291	CONTIG34	114908	115345	1.27	9.24E-02	transcription_start_site	-	110815	110815	-4311	11577 AN2168
2291	CONTIG34	114908	115345	1.27	9.24E-02	transcription_start_site	-	110175	110175	-4951	11578 AN2168
2050	CONTIG34	107559	108818	1.35	6.84E-02	transcription_start_site	+	111450	111450	-3261	11579 AN2169
2290	CONTIG34	112054	113153	1.27	9.24E-02	transcription_start_site	+	111450	111450	1153	11579 AN2169
1365	CONTIG34	113194	113614	1.65	1.06E-02	transcription_start_site	+	113323	113323	81	11580 AN2170
1365	CONTIG34	113194	113614	1.65	1.06E-02	transcription_start_site	+	113593	113593	-189	11581 AN2170
2050	CONTIG34	107559	108818	1.35	6.84E-02	transcription_start_site	+	113323	113323	-5134	11580 AN2170
2050	CONTIG34	107559	108818	1.35	6.84E-02	transcription_start_site	+	113593	113593	-5404	11581 AN2170
2290	CONTIG34	112054	113153	1.27	9.24E-02	transcription_start_site	+	113323	113323	-719	11580 AN2170
2290	CONTIG34	112054	113153	1.27	9.24E-02	transcription_start_site	+	113593	113593	-989	11581 AN2170
525	CONTIG34	116488	116841	2.41	8.69E-04	transcription_start_site	-	115658	115658	-1006	11582 AN2171
525	CONTIG34	116488	116841	2.41	8.69E-04	transcription_start_site	-	115291	115291	-1373	11583 AN2171
1919	CONTIG34	119643	119907	1.4	5.88E-02	transcription_start_site	-	115658	115658	-4117	11582 AN2171
1919	CONTIG34	119643	119907	1.4	5.88E-02	transcription_start_site	-	115291	115291	-4484	11583 AN2171
2291	CONTIG34	114908	115345	1.27	9.24E-02	transcription_start_site	-	115291	115291	164	11583 AN2171
2291	CONTIG34	114908	115345	1.27	9.24E-02	transcription_start_site	-	115658	115658	531	11582 AN2171
1919	CONTIG34	119643	119907	1.4	5.88E-02	transcription_start_site	-	119197	119197	-578	11584 AN2172
1919	CONTIG34	119643	119907	1.4	5.88E-02	transcription_start_site	-	118964	118964	-811	11585 AN2172
525	CONTIG34	116488	116841	2.41	8.69E-04	transcription_start_site	+	120005	120005	-3340	11586 AN2173
525	CONTIG34	116488	116841	2.41	8.69E-04	transcription_start_site	+	120225	120225	-3560	11587 AN2173
525	CONTIG34	116488	116841	2.41	8.69E-04	transcription_start_site	+	120396	120396	-3731	11588 AN2173
525	CONTIG34	116488	116841	2.41	8.69E-04	transcription_start_site	+	120663	120663	-3998	11589 AN2173
525	CONTIG34	116488	116841	2.41	8.69E-04	transcription_start_site	+	121314	121314	-4649	11590 AN2173
525	CONTIG34	116488	116841	2.41	8.69E-04	transcription_start_site	+	121437	121437	-4772	11591 AN2173
525	CONTIG34	116488	116841	2.41	8.69E-04	transcription_start_site	+	121594	121594	-4929	11592 AN2173
525	CONTIG34	116488	116841	2.41	8.69E-04	transcription_start_site	+	121677	121677	-5012	11593 AN2173
1919	CONTIG34	119643	119907	1.4	5.88E-02	transcription_start_site	+	120005	120005	-230	11586 AN2173
1919	CONTIG34	119643	119907	1.4	5.88E-02	transcription_start_site	+	120225	120225	-450	11587 AN2173
1919	CONTIG34	119643	119907	1.4	5.88E-02	transcription_start_site	+	120396	120396	-621	11588 AN2173
1919	CONTIG34	119643	119907	1.4	5.88E-02	transcription_start_site	+	120663	120663	-888	11589 AN2173
1919	CONTIG34	119643	119907	1.4	5.88E-02	transcription_start_site	+	121314	121314	-1539	11590 AN2173
1919	CONTIG34	119643	119907	1.4	5.88E-02	transcription_start_site	+	121437	121437	-1662	11591 AN2173
1919	CONTIG34	119643	119907	1.4	5.88E-02	transcription_start_site	+	121594	121594	-1819	11592 AN2173
1919	CONTIG34	119643	119907	1.4	5.88E-02	transcription_start_site	+	121677	121677	-1902	11593 AN2173
2291	CONTIG34	114908	115345	1.27	9.24E-02	transcription_start_site	+	120005	120005	-4878	11586 AN2173
2291	CONTIG34	114908	115345	1.27	9.24E-02	transcription_start_site	+	120225	120225	-5098	11587 AN2173
209	CONTIG34	135018	135292	2.92	0.00E+00	transcription_start_site	+	135894	135894	-739	11602 AN2177
209	CONTIG34	135018	135292	2.92	0.00E+00	transcription_start_site	+	136288	136288	-1133	11603 AN2177
1741	CONTIG34	135468	136329	1.48	2.45E-02	transcription_start_site	+	135894	135894	4	11602 AN2177

1741	CONTIG34	135468	136329	1.48	2.45E-02	transcription_start_site	+	136288	136288	-389	11603	AN2177
2292	CONTIG34	137045	137394	1.27	9.24E-02	transcription_start_site	+	136288	136288	931	11603	AN2177
2051	CONTIG34	154276	154561	1.35	6.84E-02	transcription_start_site	+	154637	154637	-218	11611	AN2183
2051	CONTIG34	154276	154561	1.35	6.84E-02	transcription_start_site	+	156347	156347	-1928	11612	AN2184
2051	CONTIG34	154276	154561	1.35	6.84E-02	transcription_start_site	+	156970	156970	-2551	11613	AN2184
2051	CONTIG34	154276	154561	1.35	6.84E-02	transcription_start_site	+	157267	157267	-2848	11614	AN2184
2052	CONTIG34	156607	156876	1.35	6.84E-02	transcription_start_site	+	156970	156970	-228	11613	AN2184
2052	CONTIG34	156607	156876	1.35	6.84E-02	transcription_start_site	+	156347	156347	394	11612	AN2184
2052	CONTIG34	156607	156876	1.35	6.84E-02	transcription_start_site	+	157267	157267	-525	11614	AN2184
2284	CONTIG34	158027	158687	1.27	7.51E-02	transcription_start_site	+	157267	157267	1090	11614	AN2184
2284	CONTIG34	158027	158687	1.27	7.51E-02	transcription_start_site	-	158754	158754	397	11615	AN2185
2052	CONTIG34	156607	156876	1.35	6.84E-02	transcription_start_site	+	159796	159796	-3054	11616	AN2186
2052	CONTIG34	156607	156876	1.35	6.84E-02	transcription_start_site	+	160517	160517	-3775	11617	AN2186
2284	CONTIG34	158027	158687	1.27	7.51E-02	transcription_start_site	+	159796	159796	-1439	11616	AN2186
2284	CONTIG34	158027	158687	1.27	7.51E-02	transcription_start_site	+	160517	160517	-2160	11617	AN2186
2667	CONTIG34	164933	165202	1.14	1.44E-01	transcription_start_site	-	162515	162515	-2552	11618	AN2187
2667	CONTIG34	164933	165202	1.14	1.44E-01	transcription_start_site	-	161929	161929	-3138	11619	AN2187
2284	CONTIG34	158027	158687	1.27	7.51E-02	transcription_start_site	+	163091	163091	-4734	11620	AN2188
2667	CONTIG34	164933	165202	1.14	1.44E-01	transcription_start_site	-	165500	165500	432	11622	AN2189
2667	CONTIG34	164933	165202	1.14	1.44E-01	transcription_start_site	+	166085	166085	-1017	11623	AN2190
2667	CONTIG34	164933	165202	1.14	1.44E-01	transcription_start_site	+	167230	167230	-2162	11624	AN2190
2667	CONTIG34	164933	165202	1.14	1.44E-01	transcription_start_site	+	170093	170093	-5025	11625	AN2191
2929	CONTIG35	27826	28270	0.99	1.93E-01	transcription_start_site	-	24376	24376	-3672	11670	AN2199
2929	CONTIG35	27826	28270	0.99	1.93E-01	transcription_start_site	-	23538	23538	-4510	11671	AN2199
2929	CONTIG35	27826	28270	0.99	1.93E-01	transcription_start_site	-	23170	23170	-4878	11672	AN2199
2929	CONTIG35	27826	28270	0.99	1.93E-01	transcription_start_site	+	27701	27701	347	11674	AN2200
2929	CONTIG35	27826	28270	0.99	1.93E-01	transcription_start_site	+	28471	28471	-423	11675	AN2200
2929	CONTIG35	27826	28270	0.99	1.93E-01	transcription_start_site	+	27516	27516	532	11673	AN2200
2929	CONTIG35	27826	28270	0.99	1.93E-01	transcription_start_site	+	28691	28691	-643	11676	AN2200
2930	CONTIG35	41187	41986	0.99	1.93E-01	transcription_start_site	-	36798	36798	-4788	11685	AN2204
2930	CONTIG35	41187	41986	0.99	1.93E-01	transcription_start_site	-	36278	36278	-5308	11686	AN2204
2930	CONTIG35	41187	41986	0.99	1.93E-01	transcription_start_site	-	40699	40699	-887	11688	AN2205
2930	CONTIG35	41187	41986	0.99	1.93E-01	transcription_start_site	-	40345	40345	-1241	11689	AN2205
2930	CONTIG35	41187	41986	0.99	1.93E-01	transcription_start_site	+	46843	46843	-5256	11690	AN2207
2930	CONTIG35	41187	41986	0.99	1.93E-01	transcription_start_site	+	46939	46939	-5352	11691	AN2207
2931	CONTIG35	47717	47988	0.99	1.93E-01	transcription_start_site	+	47745	47745	107	11692	AN2207
2931	CONTIG35	47717	47988	0.99	1.93E-01	transcription_start_site	+	46939	46939	913	11691	AN2207
2931	CONTIG35	47717	47988	0.99	1.93E-01	transcription_start_site	+	46843	46843	1009	11690	AN2207
2932	CONTIG35	48377	48956	0.99	1.93E-01	transcription_start_site	+	47745	47745	921	11692	AN2207
798	CONTIG35	49357	49725	2.1	1.54E-03	transcription_start_site	+	49193	49193	348	11697	AN2208
798	CONTIG35	49357	49725	2.1	1.54E-03	transcription_start_site	+	49041	49041	500	11696	AN2208
798	CONTIG35	49357	49725	2.1	1.54E-03	transcription_start_site	+	48578	48578	963	11695	AN2208
798	CONTIG35	49357	49725	2.1	1.54E-03	transcription_start_site	+	48430	48430	1111	11694	AN2208
2931	CONTIG35	47717	47988	0.99	1.93E-01	transcription_start_site	+	48056	48056	-203	11693	AN2208
2931	CONTIG35	47717	47988	0.99	1.93E-01	transcription_start_site	+	48430	48430	-577	11694	AN2208
2931	CONTIG35	47717	47988	0.99	1.93E-01	transcription_start_site	+	48578	48578	-725	11695	AN2208
2931	CONTIG35	47717	47988	0.99	1.93E-01	transcription_start_site	+	49041	49041	-1188	11696	AN2208
2931	CONTIG35	47717	47988	0.99	1.93E-01	transcription_start_site	+	49193	49193	-1340	11697	AN2208
2932	CONTIG35	48377	48956	0.99	1.93E-01	transcription_start_site	+	48578	48578	88	11695	AN2208
2932	CONTIG35	48377	48956	0.99	1.93E-01	transcription_start_site	+	48430	48430	236	11694	AN2208
2932	CONTIG35	48377	48956	0.99	1.93E-01	transcription_start_site	+	49041	49041	-374	11696	AN2208
2932	CONTIG35	48377	48956	0.99	1.93E-01	transcription_start_site	+	49193	49193	-526	11697	AN2208
2932	CONTIG35	48377	48956	0.99	1.93E-01	transcription_start_site	+	48056	48056	610	11693	AN2208
798	CONTIG35	49357	49725	2.1	1.54E-03	transcription_start_site	+	50405	50405	-864	11698	AN2209
798	CONTIG35	49357	49725	2.1	1.54E-03	transcription_start_site	+	50791	50791	-1250	11699	AN2209
965	CONTIG35	51696	51957	1.94	3.61E-03	transcription_start_site	+	50791	50791	1035	11699	AN2209
2931	CONTIG35	47717	47988	0.99	1.93E-01	transcription_start_site	+	50405	50405	-2552	11698	AN2209

2931	CONTIG35	47717	47988	0.99	1.93E-01	transcription_start_site	+	50791	50791	-2938	11699 AN2209
2932	CONTIG35	48377	48956	0.99	1.93E-01	transcription_start_site	+	50405	50405	-1738	11698 AN2209
2932	CONTIG35	48377	48956	0.99	1.93E-01	transcription_start_site	+	50791	50791	-2124	11699 AN2209
798	CONTIG35	49357	49725	2.1	1.54E-03	transcription_start_site	+	52026	52026	-2485	11700 AN2210
798	CONTIG35	49357	49725	2.1	1.54E-03	transcription_start_site	+	52638	52638	-3097	11701 AN2210
965	CONTIG35	51696	51957	1.94	3.61E-03	transcription_start_site	+	52026	52026	-199	11700 AN2210
965	CONTIG35	51696	51957	1.94	3.61E-03	transcription_start_site	+	52638	52638	-811	11701 AN2210
2931	CONTIG35	47717	47988	0.99	1.93E-01	transcription_start_site	+	52026	52026	-4173	11700 AN2210
2931	CONTIG35	47717	47988	0.99	1.93E-01	transcription_start_site	+	52638	52638	-4785	11701 AN2210
2932	CONTIG35	48377	48956	0.99	1.93E-01	transcription_start_site	+	52026	52026	-3359	11700 AN2210
2932	CONTIG35	48377	48956	0.99	1.93E-01	transcription_start_site	+	52638	52638	-3971	11701 AN2210
1170	CONTIG35	54992	55341	1.78	7.85E-03	transcription_start_site	-	55584	55584	417	11703 AN2211
1170	CONTIG35	54992	55341	1.78	7.85E-03	transcription_start_site	-	55996	55996	829	11702 AN2211
1170	CONTIG35	54992	55341	1.78	7.85E-03	transcription_start_site	+	58274	58274	-3107	11707 AN2213
1170	CONTIG35	54992	55341	1.78	7.85E-03	transcription_start_site	+	58336	58336	-3169	11708 AN2213
1170	CONTIG35	54992	55341	1.78	7.85E-03	transcription_start_site	+	58448	58448	-3281	11709 AN2213
1170	CONTIG35	54992	55341	1.78	7.85E-03	transcription_start_site	+	59564	59564	-4397	11710 AN2213
2933	CONTIG35	80040	80304	0.99	1.93E-01	transcription_start_site	-	76874	76874	-3298	11729 AN2218
2933	CONTIG35	80040	80304	0.99	1.93E-01	transcription_start_site	-	75901	75901	-4271	11730 AN2218
1009	CONTIG35	83420	83904	1.9	4.61E-03	transcription_start_site	-	80255	80255	-3407	11731 AN2219
1009	CONTIG35	83420	83904	1.9	4.61E-03	transcription_start_site	-	79532	79532	-4130	11732 AN2219
1009	CONTIG35	83420	83904	1.9	4.61E-03	transcription_start_site	-	79077	79077	-4585	11733 AN2219
1009	CONTIG35	83420	83904	1.9	4.61E-03	transcription_start_site	-	78934	78934	-4728	11734 AN2219
2933	CONTIG35	80040	80304	0.99	1.93E-01	transcription_start_site	-	80255	80255	83	11731 AN2219
2933	CONTIG35	80040	80304	0.99	1.93E-01	transcription_start_site	-	79532	79532	-640	11732 AN2219
2933	CONTIG35	80040	80304	0.99	1.93E-01	transcription_start_site	-	79077	79077	-1095	11733 AN2219
2933	CONTIG35	80040	80304	0.99	1.93E-01	transcription_start_site	-	78934	78934	-1238	11734 AN2219
2933	CONTIG35	80040	80304	0.99	1.93E-01	transcription_start_site	-	77958	77958	-2214	11735 AN2219
2933	CONTIG35	80040	80304	0.99	1.93E-01	transcription_start_site	+	80918	80918	-746	11736 AN2220
2933	CONTIG35	80040	80304	0.99	1.93E-01	transcription_start_site	+	81487	81487	-1315	11737 AN2220
1009	CONTIG35	83420	83904	1.9	4.61E-03	transcription_start_site	+	82453	82453	1209	11739 AN2221
2933	CONTIG35	80040	80304	0.99	1.93E-01	transcription_start_site	+	82124	82124	-1952	11738 AN2221
2933	CONTIG35	80040	80304	0.99	1.93E-01	transcription_start_site	+	82453	82453	-2281	11739 AN2221
1901	CONTIG36	16654	17843	1.41	5.52E-02	transcription_start_site	+	16053	16053	1195	11752 AN2226
1901	CONTIG36	16654	17843	1.41	5.52E-02	transcription_start_site	+	19352	19352	-2103	11753 AN2227
1901	CONTIG36	16654	17843	1.41	5.52E-02	transcription_start_site	+	19480	19480	-2231	11754 AN2227
1901	CONTIG36	16654	17843	1.41	5.52E-02	transcription_start_site	+	20150	20150	-2901	11755 AN2227
2690	CONTIG36	25595	25929	1.13	1.93E-01	transcription_start_site	-	23711	23711	-2051	11756 AN2228
2690	CONTIG36	25595	25929	1.13	1.93E-01	transcription_start_site	-	23595	23595	-2167	11757 AN2228
2690	CONTIG36	25595	25929	1.13	1.93E-01	transcription_start_site	-	26282	26282	520	11760 AN2229
2690	CONTIG36	25595	25929	1.13	1.93E-01	transcription_start_site	-	26723	26723	961	11759 AN2229
2690	CONTIG36	25595	25929	1.13	1.93E-01	transcription_start_site	-	26820	26820	1058	11758 AN2229
2690	CONTIG36	25595	25929	1.13	1.93E-01	transcription_start_site	+	29918	29918	-4156	11765 AN2231
89	CONTIG36	42902	43481	3.26	0.00E+00	transcription_start_site	+	43506	43506	-314	11779 AN2236
1390	CONTIG36	44187	45583	1.63	0.00E+00	transcription_start_site	+	43506	43506	1379	11779 AN2236
89	CONTIG36	42902	43481	3.26	0.00E+00	transcription_start_site	+	46265	46265	-3073	11780 AN2237
89	CONTIG36	42902	43481	3.26	0.00E+00	transcription_start_site	+	46534	46534	-3342	11781 AN2237
303	CONTIG36	45770	46267	2.76	2.39E-04	transcription_start_site	+	46265	46265	-246	11780 AN2237
303	CONTIG36	45770	46267	2.76	2.39E-04	transcription_start_site	+	46534	46534	-515	11781 AN2237
1390	CONTIG36	44187	45583	1.63	0.00E+00	transcription_start_site	+	46265	46265	-1380	11780 AN2237
1390	CONTIG36	44187	45583	1.63	0.00E+00	transcription_start_site	+	46534	46534	-1649	11781 AN2237
303	CONTIG36	45770	46267	2.76	2.39E-04	transcription_start_site	+	48993	48993	-2974	11782 AN2238
303	CONTIG36	45770	46267	2.76	2.39E-04	transcription_start_site	+	49200	49200	-3181	11783 AN2238
303	CONTIG36	45770	46267	2.76	2.39E-04	transcription_start_site	+	49961	49961	-3942	11784 AN2238
1390	CONTIG36	44187	45583	1.63	0.00E+00	transcription_start_site	+	48993	48993	-4108	11782 AN2238
1390	CONTIG36	44187	45583	1.63	0.00E+00	transcription_start_site	+	49200	49200	-4315	11783 AN2238
1390	CONTIG36	44187	45583	1.63	0.00E+00	transcription_start_site	+	49961	49961	-5076	11784 AN2238

1185	CONTIG36	84758	85327	1.77	2.12E-02	transcription_start_site	-	81072	81072	-3970	11815	AN2248
1185	CONTIG36	84758	85327	1.77	2.12E-02	transcription_start_site	-	80760	80760	-4282	11816	AN2248
1185	CONTIG36	84758	85327	1.77	2.12E-02	transcription_start_site	-	80661	80661	-4381	11817	AN2248
2293	CONTIG36	78093	78667	1.27	1.07E-01	transcription_start_site	-	79448	79448	1068	11818	AN2248
2443	CONTIG36	80043	80387	1.22	1.44E-01	transcription_start_site	-	80661	80661	446	11817	AN2248
2443	CONTIG36	80043	80387	1.22	1.44E-01	transcription_start_site	-	80760	80760	545	11816	AN2248
2443	CONTIG36	80043	80387	1.22	1.44E-01	transcription_start_site	-	79448	79448	-767	11818	AN2248
2443	CONTIG36	80043	80387	1.22	1.44E-01	transcription_start_site	-	81072	81072	857	11815	AN2248
1185	CONTIG36	84758	85327	1.77	2.12E-02	transcription_start_site	-	83560	83560	-1482	11819	AN2249
1185	CONTIG36	84758	85327	1.77	2.12E-02	transcription_start_site	-	83306	83306	-1736	11820	AN2249
1185	CONTIG36	84758	85327	1.77	2.12E-02	transcription_start_site	-	82951	82951	-2091	11821	AN2249
1185	CONTIG36	84758	85327	1.77	2.12E-02	transcription_start_site	-	82578	82578	-2464	11822	AN2249
1185	CONTIG36	84758	85327	1.77	2.12E-02	transcription_start_site	-	82014	82014	-3028	11823	AN2249
1905	CONTIG36	88958	89302	1.41	8.01E-02	transcription_start_site	-	87155	87155	-1975	11824	AN2250
1905	CONTIG36	88958	89302	1.41	8.01E-02	transcription_start_site	-	86830	86830	-2300	11825	AN2250
1905	CONTIG36	88958	89302	1.41	8.01E-02	transcription_start_site	-	86588	86588	-2542	11826	AN2250
1185	CONTIG36	84758	85327	1.77	2.12E-02	transcription_start_site	+	89582	89582	-4539	11827	AN2251
1185	CONTIG36	84758	85327	1.77	2.12E-02	transcription_start_site	+	89903	89903	-4860	11828	AN2251
1185	CONTIG36	84758	85327	1.77	2.12E-02	transcription_start_site	+	90100	90100	-5057	11829	AN2251
1185	CONTIG36	84758	85327	1.77	2.12E-02	transcription_start_site	+	90238	90238	-5195	11830	AN2251
1905	CONTIG36	88958	89302	1.41	8.01E-02	transcription_start_site	+	89582	89582	-452	11827	AN2251
1905	CONTIG36	88958	89302	1.41	8.01E-02	transcription_start_site	+	89903	89903	-773	11828	AN2251
1905	CONTIG36	88958	89302	1.41	8.01E-02	transcription_start_site	+	90100	90100	-970	11829	AN2251
1905	CONTIG36	88958	89302	1.41	8.01E-02	transcription_start_site	+	90238	90238	-1108	11830	AN2251
1905	CONTIG36	88958	89302	1.41	8.01E-02	transcription_start_site	+	90413	90413	-1283	11831	AN2251
1905	CONTIG36	88958	89302	1.41	8.01E-02	transcription_start_site	+	90812	90812	-1682	11832	AN2251
1905	CONTIG36	88958	89302	1.41	8.01E-02	transcription_start_site	+	94106	94106	-4976	11838	AN2254
1905	CONTIG36	88958	89302	1.41	8.01E-02	transcription_start_site	+	94174	94174	-5044	11839	AN2254
22	CONTIG37	37817	38686	3.6	0.00E+00	transcription_start_site	-	33205	33205	-5046	11867	AN2264
22	CONTIG37	37817	38686	3.6	0.00E+00	transcription_start_site	-	36317	36317	-1934	11875	AN2265
1060	CONTIG37	53492	53774	1.86	1.11E-02	transcription_start_site	-	52776	52776	-857	11887	AN2269
1060	CONTIG37	53492	53774	1.86	1.11E-02	transcription_start_site	-	52543	52543	-1090	11888	AN2269
1060	CONTIG37	53492	53774	1.86	1.11E-02	transcription_start_site	-	52416	52416	-1217	11889	AN2269
1060	CONTIG37	53492	53774	1.86	1.11E-02	transcription_start_site	-	52215	52215	-1418	11890	AN2269
1060	CONTIG37	53492	53774	1.86	1.11E-02	transcription_start_site	+	55075	55075	-1442	11891	AN2270
1060	CONTIG37	53492	53774	1.86	1.11E-02	transcription_start_site	+	55274	55274	-1641	11892	AN2270
1060	CONTIG37	53492	53774	1.86	1.11E-02	transcription_start_site	+	55398	55398	-1765	11893	AN2270
1000	CONTIG37	73508	73782	1.91	8.81E-03	transcription_start_site	-	68933	68933	-4712	11911	AN2274
1000	CONTIG37	73508	73782	1.91	8.81E-03	transcription_start_site	-	68623	68623	-5022	11912	AN2274
1000	CONTIG37	73508	73782	1.91	8.81E-03	transcription_start_site	+	74426	74426	-781	11921	AN2277
1000	CONTIG37	73508	73782	1.91	8.81E-03	transcription_start_site	+	74616	74616	-971	11922	AN2277
1000	CONTIG37	73508	73782	1.91	8.81E-03	transcription_start_site	+	74840	74840	-1195	11923	AN2277
1000	CONTIG37	73508	73782	1.91	8.81E-03	transcription_start_site	+	75011	75011	-1366	11924	AN2277
1052	CONTIG38	1511	1851	1.86	3.61E-03	transcription_start_site	+	2093	2093	-412	11925	AN2278
1052	CONTIG38	1511	1851	1.86	3.61E-03	transcription_start_site	+	2334	2334	-653	11926	AN2278
1052	CONTIG38	1511	1851	1.86	3.61E-03	transcription_start_site	+	2603	2603	-922	11927	AN2278
1052	CONTIG38	1511	1851	1.86	3.61E-03	transcription_start_site	+	6372	6372	-4691	11928	AN2278
1053	CONTIG38	9687	10029	1.86	3.61E-03	transcription_start_site	-	9640	9640	-218	11935	AN2280
1053	CONTIG38	9687	10029	1.86	3.61E-03	transcription_start_site	-	9483	9483	-375	11936	AN2280
1053	CONTIG38	9687	10029	1.86	3.61E-03	transcription_start_site	-	9091	9091	-767	11937	AN2280
1631	CONTIG38	12832	13716	1.52	3.45E-03	transcription_start_site	-	9640	9640	-3634	11935	AN2280
1631	CONTIG38	12832	13716	1.52	3.45E-03	transcription_start_site	-	9483	9483	-3791	11936	AN2280
1631	CONTIG38	12832	13716	1.52	3.45E-03	transcription_start_site	-	9091	9091	-4183	11937	AN2280
1053	CONTIG38	9687	10029	1.86	3.61E-03	transcription_start_site	+	10587	10587	-729	11938	AN2281
1053	CONTIG38	9687	10029	1.86	3.61E-03	transcription_start_site	+	10822	10822	-964	11939	AN2281
1053	CONTIG38	9687	10029	1.86	3.61E-03	transcription_start_site	+	11301	11301	-1443	11940	AN2281
1053	CONTIG38	9687	10029	1.86	3.61E-03	transcription_start_site	+	11755	11755	-1897	11941	AN2281

1053	CONTIG38	9687	10029	1.86	3.61E-03	transcription_start_site	+	13477	13477	-3619	11942 AN2282
1053	CONTIG38	9687	10029	1.86	3.61E-03	transcription_start_site	+	13539	13539	-3681	11943 AN2282
1053	CONTIG38	9687	10029	1.86	3.61E-03	transcription_start_site	+	13804	13804	-3946	11944 AN2282
1053	CONTIG38	9687	10029	1.86	3.61E-03	transcription_start_site	+	14016	14016	-4158	11945 AN2282
1053	CONTIG38	9687	10029	1.86	3.61E-03	transcription_start_site	+	14161	14161	-4303	11946 AN2282
1053	CONTIG38	9687	10029	1.86	3.61E-03	transcription_start_site	+	14245	14245	-4387	11947 AN2282
1053	CONTIG38	9687	10029	1.86	3.61E-03	transcription_start_site	+	14660	14660	-4802	11948 AN2282
1053	CONTIG38	9687	10029	1.86	3.61E-03	transcription_start_site	+	14772	14772	-4914	11949 AN2282
1053	CONTIG38	9687	10029	1.86	3.61E-03	transcription_start_site	+	14870	14870	-5012	11950 AN2282
1631	CONTIG38	12832	13716	1.52	3.45E-03	transcription_start_site	+	13477	13477	-203	11942 AN2282
1631	CONTIG38	12832	13716	1.52	3.45E-03	transcription_start_site	+	13539	13539	-265	11943 AN2282
1631	CONTIG38	12832	13716	1.52	3.45E-03	transcription_start_site	+	13804	13804	-530	11944 AN2282
1631	CONTIG38	12832	13716	1.52	3.45E-03	transcription_start_site	+	14016	14016	-742	11945 AN2282
1631	CONTIG38	12832	13716	1.52	3.45E-03	transcription_start_site	+	14161	14161	-887	11946 AN2282
1631	CONTIG38	12832	13716	1.52	3.45E-03	transcription_start_site	+	14245	14245	-971	11947 AN2282
1631	CONTIG38	12832	13716	1.52	3.45E-03	transcription_start_site	+	14660	14660	-1386	11948 AN2282
1631	CONTIG38	12832	13716	1.52	3.45E-03	transcription_start_site	+	14772	14772	-1498	11949 AN2282
1631	CONTIG38	12832	13716	1.52	3.45E-03	transcription_start_site	+	14870	14870	-1596	11950 AN2282
1631	CONTIG38	12832	13716	1.52	3.45E-03	transcription_start_site	+	15237	15237	-1963	11951 AN2282
1544	CONTIG38	17562	17911	1.56	1.57E-02	transcription_start_site	+	17399	17399	337	11954 AN2283
1544	CONTIG38	17562	17911	1.56	1.57E-02	transcription_start_site	+	17007	17007	729	11953 AN2283
1544	CONTIG38	17562	17911	1.56	1.57E-02	transcription_start_site	+	16791	16791	945	11952 AN2283
1631	CONTIG38	12832	13716	1.52	3.45E-03	transcription_start_site	+	16791	16791	-3517	11952 AN2283
1631	CONTIG38	12832	13716	1.52	3.45E-03	transcription_start_site	+	17007	17007	-3733	11953 AN2283
1631	CONTIG38	12832	13716	1.52	3.45E-03	transcription_start_site	+	17399	17399	-4125	11954 AN2283
2212	CONTIG38	20777	21217	1.29	5.22E-02	transcription_start_site	-	20852	20852	-145	11955 AN2284
2212	CONTIG38	20777	21217	1.29	5.22E-02	transcription_start_site	-	20433	20433	-564	11956 AN2284
1266	CONTIG38	30396	30660	1.71	7.85E-03	transcription_start_site	-	27019	27019	-3509	11957 AN2285
1266	CONTIG38	30396	30660	1.71	7.85E-03	transcription_start_site	-	26416	26416	-4112	11958 AN2285
1266	CONTIG38	30396	30660	1.71	7.85E-03	transcription_start_site	-	25855	25855	-4673	11959 AN2285
1740	CONTIG38	26871	27205	1.48	2.12E-02	transcription_start_site	-	27019	27019	-19	11957 AN2285
1740	CONTIG38	26871	27205	1.48	2.12E-02	transcription_start_site	-	26416	26416	-622	11958 AN2285
1740	CONTIG38	26871	27205	1.48	2.12E-02	transcription_start_site	-	25855	25855	-1183	11959 AN2285
1740	CONTIG38	26871	27205	1.48	2.12E-02	transcription_start_site	-	23349	23349	-3689	11960 AN2285
1740	CONTIG38	26871	27205	1.48	2.12E-02	transcription_start_site	-	22027	22027	-5011	11961 AN2285
2212	CONTIG38	20777	21217	1.29	5.22E-02	transcription_start_site	-	22027	22027	1030	11961 AN2285
1266	CONTIG38	30396	30660	1.71	7.85E-03	transcription_start_site	-	30070	30070	-458	11962 AN2286
1266	CONTIG38	30396	30660	1.71	7.85E-03	transcription_start_site	-	29340	29340	-1188	11963 AN2286
1266	CONTIG38	30396	30660	1.71	7.85E-03	transcription_start_site	-	28917	28917	-1611	11964 AN2286
1266	CONTIG38	30396	30660	1.71	7.85E-03	transcription_start_site	-	31025	31025	497	11974 AN2287
1266	CONTIG38	30396	30660	1.71	7.85E-03	transcription_start_site	-	31399	31399	871	11973 AN2287
1267	CONTIG38	43205	43491	1.71	7.85E-03	transcription_start_site	-	39015	39015	-4333	11980 AN2289
1267	CONTIG38	43205	43491	1.71	7.85E-03	transcription_start_site	-	38747	38747	-4601	11981 AN2289
1267	CONTIG38	43205	43491	1.71	7.85E-03	transcription_start_site	-	38654	38654	-4694	11982 AN2289
1267	CONTIG38	43205	43491	1.71	7.85E-03	transcription_start_site	-	38283	38283	-5065	11983 AN2289
1267	CONTIG38	43205	43491	1.71	7.85E-03	transcription_start_site	-	42443	42443	-905	11984 AN2290
1267	CONTIG38	43205	43491	1.71	7.85E-03	transcription_start_site	-	42268	42268	-1080	11985 AN2290
1267	CONTIG38	43205	43491	1.71	7.85E-03	transcription_start_site	-	42111	42111	-1237	11986 AN2290
1267	CONTIG38	43205	43491	1.71	7.85E-03	transcription_start_site	-	40485	40485	-2863	11987 AN2290
1267	CONTIG38	43205	43491	1.71	7.85E-03	transcription_start_site	-	40347	40347	-3001	11988 AN2290
1323	CONTIG38	74942	75276	1.67	8.81E-03	transcription_start_site	-	74196	74196	-913	12018 AN2300
1323	CONTIG38	74942	75276	1.67	8.81E-03	transcription_start_site	-	73721	73721	-1388	12019 AN2300
1323	CONTIG38	74942	75276	1.67	8.81E-03	transcription_start_site	-	70399	70399	-4710	12020 AN2300
1323	CONTIG38	74942	75276	1.67	8.81E-03	transcription_start_site	+	77018	77018	-1909	12021 AN2301
1323	CONTIG38	74942	75276	1.67	8.81E-03	transcription_start_site	+	77160	77160	-2051	12022 AN2301
1323	CONTIG38	74942	75276	1.67	8.81E-03	transcription_start_site	+	77529	77529	-2420	12023 AN2301
2740	CONTIG38	83861	84220	1.1	1.08E-01	transcription_start_site	-	79519	79519	-4521	12024 AN2302

2740	CONTIG38	83861	84220	1.1	1.08E-01	transcription_start_site	-	79396	79396	-4644	12025	AN2302
2740	CONTIG38	83861	84220	1.1	1.08E-01	transcription_start_site	-	79250	79250	-4790	12026	AN2302
2740	CONTIG38	83861	84220	1.1	1.08E-01	transcription_start_site	-	79021	79021	-5019	12027	AN2302
2740	CONTIG38	83861	84220	1.1	1.08E-01	transcription_start_site	-	83900	83900	-140	12038	AN2304
2740	CONTIG38	83861	84220	1.1	1.08E-01	transcription_start_site	-	84290	84290	249	12037	AN2304
2740	CONTIG38	83861	84220	1.1	1.08E-01	transcription_start_site	-	84427	84427	386	12036	AN2304
2740	CONTIG38	83861	84220	1.1	1.08E-01	transcription_start_site	-	84564	84564	523	12035	AN2304
2740	CONTIG38	83861	84220	1.1	1.08E-01	transcription_start_site	-	84671	84671	630	12034	AN2304
2740	CONTIG38	83861	84220	1.1	1.08E-01	transcription_start_site	-	83406	83406	-634	12039	AN2304
2740	CONTIG38	83861	84220	1.1	1.08E-01	transcription_start_site	-	84757	84757	716	12033	AN2304
2740	CONTIG38	83861	84220	1.1	1.08E-01	transcription_start_site	+	86679	86679	-2638	12040	AN2305
2740	CONTIG38	83861	84220	1.1	1.08E-01	transcription_start_site	+	86791	86791	-2750	12041	AN2305
2790	CONTIG38	95412	96066	1.07	1.07E-01	transcription_start_site	-	90848	90848	-4891	12042	AN2306
2790	CONTIG38	95412	96066	1.07	1.07E-01	transcription_start_site	-	90446	90446	-5293	12043	AN2306
2969	CONTIG38	90677	90961	0.95	1.93E-01	transcription_start_site	-	90848	90848	29	12042	AN2306
2969	CONTIG38	90677	90961	0.95	1.93E-01	transcription_start_site	-	90446	90446	-373	12043	AN2306
2969	CONTIG38	90677	90961	0.95	1.93E-01	transcription_start_site	-	90283	90283	-536	12044	AN2306
2969	CONTIG38	90677	90961	0.95	1.93E-01	transcription_start_site	-	90075	90075	-744	12045	AN2306
2790	CONTIG38	95412	96066	1.07	1.07E-01	transcription_start_site	-	91851	91851	-3888	12046	AN2307
2790	CONTIG38	95412	96066	1.07	1.07E-01	transcription_start_site	-	91600	91600	-4139	12047	AN2307
2969	CONTIG38	90677	90961	0.95	1.93E-01	transcription_start_site	-	91600	91600	781	12047	AN2307
2969	CONTIG38	90677	90961	0.95	1.93E-01	transcription_start_site	-	91851	91851	1032	12046	AN2307
2790	CONTIG38	95412	96066	1.07	1.07E-01	transcription_start_site	+	94442	94442	1297	12054	AN2308
2969	CONTIG38	90677	90961	0.95	1.93E-01	transcription_start_site	+	92405	92405	-1586	12048	AN2308
2969	CONTIG38	90677	90961	0.95	1.93E-01	transcription_start_site	+	92472	92472	-1653	12049	AN2308
2969	CONTIG38	90677	90961	0.95	1.93E-01	transcription_start_site	+	93160	93160	-2341	12050	AN2308
2969	CONTIG38	90677	90961	0.95	1.93E-01	transcription_start_site	+	93549	93549	-2730	12051	AN2308
2969	CONTIG38	90677	90961	0.95	1.93E-01	transcription_start_site	+	93674	93674	-2855	12052	AN2308
2969	CONTIG38	90677	90961	0.95	1.93E-01	transcription_start_site	+	94043	94043	-3224	12053	AN2308
2969	CONTIG38	90677	90961	0.95	1.93E-01	transcription_start_site	+	94442	94442	-3623	12054	AN2308
1805	CONTIG38	97132	97420	1.45	2.62E-02	transcription_start_site	-	96047	96047	-1229	12055	AN2309
1805	CONTIG38	97132	97420	1.45	2.62E-02	transcription_start_site	-	95878	95878	-1398	12056	AN2309
1805	CONTIG38	97132	97420	1.45	2.62E-02	transcription_start_site	-	95692	95692	-1584	12057	AN2309
2790	CONTIG38	95412	96066	1.07	1.07E-01	transcription_start_site	-	95692	95692	-47	12057	AN2309
2790	CONTIG38	95412	96066	1.07	1.07E-01	transcription_start_site	-	95878	95878	139	12056	AN2309
2790	CONTIG38	95412	96066	1.07	1.07E-01	transcription_start_site	-	96047	96047	308	12055	AN2309
428	CONTIG38	102527	102806	2.55	0.00E+00	transcription_start_site	+	103767	103767	-1100	12058	AN2311
428	CONTIG38	102527	102806	2.55	0.00E+00	transcription_start_site	+	104118	104118	-1451	12059	AN2311
428	CONTIG38	102527	102806	2.55	0.00E+00	transcription_start_site	+	105504	105504	-2837	12060	AN2311
2103	CONTIG38	101417	101676	1.33	4.36E-02	transcription_start_site	+	103767	103767	-2220	12058	AN2311
2103	CONTIG38	101417	101676	1.33	4.36E-02	transcription_start_site	+	104118	104118	-2571	12059	AN2311
2103	CONTIG38	101417	101676	1.33	4.36E-02	transcription_start_site	+	105504	105504	-3957	12060	AN2311
428	CONTIG38	102527	102806	2.55	0.00E+00	transcription_start_site	+	107128	107128	-4461	12061	AN2312
428	CONTIG38	102527	102806	2.55	0.00E+00	transcription_start_site	+	107235	107235	-4568	12062	AN2312
1992	CONTIG38	117155	118254	1.37	2.18E-02	transcription_start_site	-	117681	117681	-23	12073	AN2315
1992	CONTIG38	117155	118254	1.37	2.18E-02	transcription_start_site	-	117599	117599	-105	12074	AN2315
1992	CONTIG38	117155	118254	1.37	2.18E-02	transcription_start_site	-	117290	117290	-414	12075	AN2315
1992	CONTIG38	117155	118254	1.37	2.18E-02	transcription_start_site	-	117140	117140	-564	12076	AN2315
1992	CONTIG38	117155	118254	1.37	2.18E-02	transcription_start_site	-	117018	117018	-686	12077	AN2315
1992	CONTIG38	117155	118254	1.37	2.18E-02	transcription_start_site	-	116651	116651	-1053	12078	AN2315
1992	CONTIG38	117155	118254	1.37	2.18E-02	transcription_start_site	-	115747	115747	-1957	12079	AN2315
1992	CONTIG38	117155	118254	1.37	2.18E-02	transcription_start_site	-	118968	118968	1263	12082	AN2316
1992	CONTIG38	117155	118254	1.37	2.18E-02	transcription_start_site	-	119254	119254	1549	12081	AN2316
2868	CONTIG38	137712	138216	1.03	1.44E-01	transcription_start_site	-	132722	132722	-5242	12093	AN2320
2868	CONTIG38	137712	138216	1.03	1.44E-01	transcription_start_site	-	134823	134823	-3141	12097	AN2321
2868	CONTIG38	137712	138216	1.03	1.44E-01	transcription_start_site	-	134603	134603	-3361	12098	AN2321
2868	CONTIG38	137712	138216	1.03	1.44E-01	transcription_start_site	-	134267	134267	-3697	12099	AN2321

2868	CONTIG38	137712	138216	1.03	1.44E-01	transcription_start_site	-	137707	137707	-257	12101	AN2322
2868	CONTIG38	137712	138216	1.03	1.44E-01	transcription_start_site	-	138427	138427	463	12100	AN2322
2868	CONTIG38	137712	138216	1.03	1.44E-01	transcription_start_site	+	142280	142280	-4316	12104	AN2324
2868	CONTIG38	137712	138216	1.03	1.44E-01	transcription_start_site	+	142413	142413	-4449	12105	AN2324
2868	CONTIG38	137712	138216	1.03	1.44E-01	transcription_start_site	+	142543	142543	-4579	12106	AN2324
2923	CONTIG38	157365	157709	0.99	1.66E-01	transcription_start_site	-	157547	157547	10	12124	AN2328
2317	CONTIG38	169070	169404	1.26	5.88E-02	transcription_start_site	-	167229	167229	-2008	12127	AN2331
2317	CONTIG38	169070	169404	1.26	5.88E-02	transcription_start_site	-	166959	166959	-2278	12128	AN2331
2317	CONTIG38	169070	169404	1.26	5.88E-02	transcription_start_site	-	166618	166618	-2619	12129	AN2331
2317	CONTIG38	169070	169404	1.26	5.88E-02	transcription_start_site	-	166481	166481	-2756	12130	AN2331
2317	CONTIG38	169070	169404	1.26	5.88E-02	transcription_start_site	-	166154	166154	-3083	12131	AN2331
2922	CONTIG38	169505	170154	0.99	1.50E-01	transcription_start_site	-	167229	167229	-2600	12127	AN2331
2922	CONTIG38	169505	170154	0.99	1.50E-01	transcription_start_site	-	166959	166959	-2870	12128	AN2331
2922	CONTIG38	169505	170154	0.99	1.50E-01	transcription_start_site	-	166618	166618	-3211	12129	AN2331
2922	CONTIG38	169505	170154	0.99	1.50E-01	transcription_start_site	-	166481	166481	-3348	12130	AN2331
2922	CONTIG38	169505	170154	0.99	1.50E-01	transcription_start_site	-	166154	166154	-3675	12131	AN2331
2317	CONTIG38	169070	169404	1.26	5.88E-02	transcription_start_site	+	169244	169244	-7	12132	AN2332
2317	CONTIG38	169070	169404	1.26	5.88E-02	transcription_start_site	+	169403	169403	-166	12133	AN2332
2317	CONTIG38	169070	169404	1.26	5.88E-02	transcription_start_site	+	169705	169705	-468	12134	AN2332
2317	CONTIG38	169070	169404	1.26	5.88E-02	transcription_start_site	+	169905	169905	-668	12135	AN2332
2922	CONTIG38	169505	170154	0.99	1.50E-01	transcription_start_site	+	169905	169905	-75	12135	AN2332
2922	CONTIG38	169505	170154	0.99	1.50E-01	transcription_start_site	+	169705	169705	124	12134	AN2332
2922	CONTIG38	169505	170154	0.99	1.50E-01	transcription_start_site	+	169403	169403	426	12133	AN2332
2922	CONTIG38	169505	170154	0.99	1.50E-01	transcription_start_site	+	169244	169244	585	12132	AN2332
2922	CONTIG38	169505	170154	0.99	1.50E-01	transcription_start_site	-	170743	170743	913	12139	AN2333
1104	CONTIG38	179028	179302	1.83	4.61E-03	transcription_start_site	-	176919	176919	-2246	12140	AN2334
1634	CONTIG38	181893	182152	1.52	1.84E-02	transcription_start_site	-	176919	176919	-5103	12140	AN2334
2558	CONTIG38	180528	180952	1.18	8.01E-02	transcription_start_site	-	176919	176919	-3821	12140	AN2334
2967	CONTIG38	177618	178402	0.95	1.79E-01	transcription_start_site	-	176919	176919	-1091	12140	AN2334
1104	CONTIG38	179028	179302	1.83	4.61E-03	transcription_start_site	-	179033	179033	-132	12141	AN2335
1104	CONTIG38	179028	179302	1.83	4.61E-03	transcription_start_site	-	178668	178668	-497	12142	AN2335
1104	CONTIG38	179028	179302	1.83	4.61E-03	transcription_start_site	-	178110	178110	-1055	12143	AN2335
1634	CONTIG38	181893	182152	1.52	1.84E-02	transcription_start_site	-	179033	179033	-2989	12141	AN2335
1634	CONTIG38	181893	182152	1.52	1.84E-02	transcription_start_site	-	178668	178668	-3354	12142	AN2335
1634	CONTIG38	181893	182152	1.52	1.84E-02	transcription_start_site	-	178110	178110	-3912	12143	AN2335
2558	CONTIG38	180528	180952	1.18	8.01E-02	transcription_start_site	-	179033	179033	-1707	12141	AN2335
2558	CONTIG38	180528	180952	1.18	8.01E-02	transcription_start_site	-	178668	178668	-2072	12142	AN2335
2558	CONTIG38	180528	180952	1.18	8.01E-02	transcription_start_site	-	178110	178110	-2630	12143	AN2335
2967	CONTIG38	177618	178402	0.95	1.79E-01	transcription_start_site	-	178110	178110	100	12143	AN2335
2967	CONTIG38	177618	178402	0.95	1.79E-01	transcription_start_site	-	178668	178668	658	12142	AN2335
2967	CONTIG38	177618	178402	0.95	1.79E-01	transcription_start_site	-	179033	179033	1023	12141	AN2335
1634	CONTIG38	181893	182152	1.52	1.84E-02	transcription_start_site	-	181842	181842	-180	12144	AN2336
1634	CONTIG38	181893	182152	1.52	1.84E-02	transcription_start_site	-	181231	181231	-791	12145	AN2336
2558	CONTIG38	180528	180952	1.18	8.01E-02	transcription_start_site	-	181231	181231	491	12145	AN2336
2558	CONTIG38	180528	180952	1.18	8.01E-02	transcription_start_site	-	181842	181842	1102	12144	AN2336
1634	CONTIG38	181893	182152	1.52	1.84E-02	transcription_start_site	+	185784	185784	-3761	12146	AN2337
1634	CONTIG38	181893	182152	1.52	1.84E-02	transcription_start_site	+	185874	185874	-3851	12147	AN2337
1634	CONTIG38	181893	182152	1.52	1.84E-02	transcription_start_site	+	186269	186269	-4246	12148	AN2337
2558	CONTIG38	180528	180952	1.18	8.01E-02	transcription_start_site	+	185784	185784	-5044	12146	AN2337
2558	CONTIG38	180528	180952	1.18	8.01E-02	transcription_start_site	+	185874	185874	-5134	12147	AN2337
1268	CONTIG38	187808	188167	1.71	7.85E-03	transcription_start_site	-	187828	187828	-159	12149	AN2338
1545	CONTIG38	188788	189137	1.56	1.57E-02	transcription_start_site	-	187828	187828	-1134	12149	AN2338
1268	CONTIG38	187808	188167	1.71	7.85E-03	transcription_start_site	+	191482	191482	-3494	12150	AN2339
1268	CONTIG38	187808	188167	1.71	7.85E-03	transcription_start_site	+	191762	191762	-3774	12151	AN2339
1268	CONTIG38	187808	188167	1.71	7.85E-03	transcription_start_site	+	192193	192193	-4205	12152	AN2339
1545	CONTIG38	188788	189137	1.56	1.57E-02	transcription_start_site	+	191482	191482	-2519	12150	AN2339
1545	CONTIG38	188788	189137	1.56	1.57E-02	transcription_start_site	+	191762	191762	-2799	12151	AN2339

1545	CONTIG38	188788	189137	1.56	1.57E-02	transcription_start_site	+	192193	192193	-3230	12152	AN2339
1894	CONTIG38	203101	203530	1.41	3.08E-02	transcription_start_site	+	202156	202156	1159	12155	AN2342
1894	CONTIG38	203101	203530	1.41	3.08E-02	transcription_start_site	-	203096	203096	-219	12156	AN2343
1894	CONTIG38	203101	203530	1.41	3.08E-02	transcription_start_site	-	202710	202710	-605	12157	AN2343
1894	CONTIG38	203101	203530	1.41	3.08E-02	transcription_start_site	-	202534	202534	-781	12158	AN2343
1894	CONTIG38	203101	203530	1.41	3.08E-02	transcription_start_site	+	206029	206029	-2713	12159	AN2344
1894	CONTIG38	203101	203530	1.41	3.08E-02	transcription_start_site	+	206265	206265	-2949	12160	AN2344
1894	CONTIG38	203101	203530	1.41	3.08E-02	transcription_start_site	+	206345	206345	-3029	12161	AN2344
2971	CONTIG38	226580	227001	0.95	1.93E-01	transcription_start_site	+	226459	226459	331	12169	AN2348
2971	CONTIG38	226580	227001	0.95	1.93E-01	transcription_start_site	+	228757	228757	-1966	12170	AN2349
2971	CONTIG38	226580	227001	0.95	1.93E-01	transcription_start_site	+	228942	228942	-2151	12171	AN2349
2971	CONTIG38	226580	227001	0.95	1.93E-01	transcription_start_site	+	229176	229176	-2385	12172	AN2349
2971	CONTIG38	226580	227001	0.95	1.93E-01	transcription_start_site	+	229890	229890	-3099	12173	AN2349
1118	CONTIG39	35929	36278	1.82	3.08E-02	transcription_start_site	-	31233	31233	-4870	12201	AN2360
1118	CONTIG39	35929	36278	1.82	3.08E-02	transcription_start_site	-	31050	31050	-5053	12202	AN2360
1118	CONTIG39	35929	36278	1.82	3.08E-02	transcription_start_site	-	32647	32647	-3456	12203	AN2361
1118	CONTIG39	35929	36278	1.82	3.08E-02	transcription_start_site	-	32428	32428	-3675	12204	AN2361
1118	CONTIG39	35929	36278	1.82	3.08E-02	transcription_start_site	-	34743	34743	-1360	12205	AN2362
1118	CONTIG39	35929	36278	1.82	3.08E-02	transcription_start_site	-	34478	34478	-1625	12206	AN2362
1118	CONTIG39	35929	36278	1.82	3.08E-02	transcription_start_site	-	34033	34033	-2070	12207	AN2362
1118	CONTIG39	35929	36278	1.82	3.08E-02	transcription_start_site	-	33864	33864	-2239	12208	AN2362
1118	CONTIG39	35929	36278	1.82	3.08E-02	transcription_start_site	-	33669	33669	-2434	12209	AN2362
1118	CONTIG39	35929	36278	1.82	3.08E-02	transcription_start_site	+	36466	36466	-362	12210	AN2363
1118	CONTIG39	35929	36278	1.82	3.08E-02	transcription_start_site	+	36604	36604	-500	12211	AN2363
1118	CONTIG39	35929	36278	1.82	3.08E-02	transcription_start_site	+	37815	37815	-1711	12212	AN2363
1118	CONTIG39	35929	36278	1.82	3.08E-02	transcription_start_site	+	38583	38583	-2479	12213	AN2363
1118	CONTIG39	35929	36278	1.82	3.08E-02	transcription_start_site	+	39661	39661	-3557	12214	AN2363
1118	CONTIG39	35929	36278	1.82	3.08E-02	transcription_start_site	+	40006	40006	-3902	12215	AN2363
1118	CONTIG39	35929	36278	1.82	3.08E-02	transcription_start_site	+	40171	40171	-4067	12216	AN2363
78	CONTIG39	49889	50468	3.3	0.00E+00	transcription_start_site	-	45846	45846	-4332	12219	AN2365
78	CONTIG39	49889	50468	3.3	0.00E+00	transcription_start_site	-	45244	45244	-4934	12220	AN2365
78	CONTIG39	49889	50468	3.3	0.00E+00	transcription_start_site	+	50426	50426	-247	12222	AN2366
940	CONTIG39	51089	51588	1.97	1.84E-02	transcription_start_site	+	50426	50426	912	12222	AN2366
940	CONTIG39	51089	51588	1.97	1.84E-02	transcription_start_site	-	52078	52078	739	12225	AN2367
2273	CONTIG39	52204	52493	1.28	1.66E-01	transcription_start_site	-	52078	52078	-270	12225	AN2367
2273	CONTIG39	52204	52493	1.28	1.66E-01	transcription_start_site	-	52626	52626	277	12224	AN2367
2273	CONTIG39	52204	52493	1.28	1.66E-01	transcription_start_site	-	52989	52989	640	12223	AN2367
78	CONTIG39	49889	50468	3.3	0.00E+00	transcription_start_site	+	53717	53717	-3538	12226	AN2368
78	CONTIG39	49889	50468	3.3	0.00E+00	transcription_start_site	+	54018	54018	-3839	12227	AN2368
78	CONTIG39	49889	50468	3.3	0.00E+00	transcription_start_site	+	54463	54463	-4284	12228	AN2368
78	CONTIG39	49889	50468	3.3	0.00E+00	transcription_start_site	+	54541	54541	-4362	12229	AN2368
78	CONTIG39	49889	50468	3.3	0.00E+00	transcription_start_site	+	54881	54881	-4702	12230	AN2368
940	CONTIG39	51089	51588	1.97	1.84E-02	transcription_start_site	+	53717	53717	-2378	12226	AN2368
940	CONTIG39	51089	51588	1.97	1.84E-02	transcription_start_site	+	54018	54018	-2679	12227	AN2368
940	CONTIG39	51089	51588	1.97	1.84E-02	transcription_start_site	+	54463	54463	-3124	12228	AN2368
940	CONTIG39	51089	51588	1.97	1.84E-02	transcription_start_site	+	54541	54541	-3202	12229	AN2368
940	CONTIG39	51089	51588	1.97	1.84E-02	transcription_start_site	+	54881	54881	-3542	12230	AN2368
2273	CONTIG39	52204	52493	1.28	1.66E-01	transcription_start_site	+	53717	53717	-1368	12226	AN2368
2273	CONTIG39	52204	52493	1.28	1.66E-01	transcription_start_site	+	54018	54018	-1669	12227	AN2368
2273	CONTIG39	52204	52493	1.28	1.66E-01	transcription_start_site	+	54463	54463	-2114	12228	AN2368
2273	CONTIG39	52204	52493	1.28	1.66E-01	transcription_start_site	+	54541	54541	-2192	12229	AN2368
2273	CONTIG39	52204	52493	1.28	1.66E-01	transcription_start_site	+	54881	54881	-2532	12230	AN2368
1319	CONTIG39	92704	93503	1.68	5.22E-02	transcription_start_site	-	87960	87960	-5143	12271	AN2380
1319	CONTIG39	92704	93503	1.68	5.22E-02	transcription_start_site	-	89462	89462	-3641	12273	AN2381
1319	CONTIG39	92704	93503	1.68	5.22E-02	transcription_start_site	-	90752	90752	-2351	12274	AN2382
1319	CONTIG39	92704	93503	1.68	5.22E-02	transcription_start_site	+	93356	93356	-252	12277	AN2383
1319	CONTIG39	92704	93503	1.68	5.22E-02	transcription_start_site	+	93544	93544	-440	12278	AN2383

1319	CONTIG39	92704	93503	1.68	5.22E-02	transcription_start_site	+	92643	92643	460	12276	AN2383
1319	CONTIG39	92704	93503	1.68	5.22E-02	transcription_start_site	+	92278	92278	825	12275	AN2383
1319	CONTIG39	92704	93503	1.68	5.22E-02	transcription_start_site	+	94092	94092	-988	12279	AN2383
1319	CONTIG39	92704	93503	1.68	5.22E-02	transcription_start_site	+	95564	95564	-2460	12280	AN2384
1319	CONTIG39	92704	93503	1.68	5.22E-02	transcription_start_site	+	96018	96018	-2914	12281	AN2384
1319	CONTIG39	92704	93503	1.68	5.22E-02	transcription_start_site	+	96387	96387	-3283	12282	AN2384
1319	CONTIG39	92704	93503	1.68	5.22E-02	transcription_start_site	+	97405	97405	-4301	12283	AN2384
362	CONTIG39	102677	103026	2.66	0.00E+00	transcription_start_site	-	101937	101937	-914	12284	AN2385
1405	CONTIG39	100502	101017	1.63	5.88E-02	transcription_start_site	-	101937	101937	1177	12284	AN2385
1315	CONTIG39	120376	120725	1.68	3.33E-02	transcription_start_site	+	120488	120488	62	12307	AN2390
1315	CONTIG39	120376	120725	1.68	3.33E-02	transcription_start_site	+	120696	120696	-145	12308	AN2390
1315	CONTIG39	120376	120725	1.68	3.33E-02	transcription_start_site	+	120265	120265	285	12306	AN2390
189	CONTIG39	125646	126126	2.96	2.16E-04	transcription_start_site	-	125946	125946	60	12309	AN2391
189	CONTIG39	125646	126126	2.96	2.16E-04	transcription_start_site	-	125673	125673	-213	12310	AN2391
189	CONTIG39	125646	126126	2.96	2.16E-04	transcription_start_site	-	125454	125454	-432	12311	AN2391
189	CONTIG39	125646	126126	2.96	2.16E-04	transcription_start_site	-	123601	123601	-2285	12312	AN2391
1247	CONTIG39	128496	128750	1.73	4.36E-02	transcription_start_site	-	125946	125946	-2677	12309	AN2391
1247	CONTIG39	128496	128750	1.73	4.36E-02	transcription_start_site	-	125673	125673	-2950	12310	AN2391
1247	CONTIG39	128496	128750	1.73	4.36E-02	transcription_start_site	-	125454	125454	-3169	12311	AN2391
1247	CONTIG39	128496	128750	1.73	4.36E-02	transcription_start_site	-	123601	123601	-5022	12312	AN2391
189	CONTIG39	125646	126126	2.96	2.16E-04	transcription_start_site	+	130155	130155	-4269	12313	AN2392
189	CONTIG39	125646	126126	2.96	2.16E-04	transcription_start_site	+	130346	130346	-4460	12314	AN2392
1247	CONTIG39	128496	128750	1.73	4.36E-02	transcription_start_site	+	130155	130155	-1532	12313	AN2392
1247	CONTIG39	128496	128750	1.73	4.36E-02	transcription_start_site	+	130346	130346	-1723	12314	AN2392
1247	CONTIG39	128496	128750	1.73	4.36E-02	transcription_start_site	+	131448	131448	-2825	12315	AN2392
1247	CONTIG39	128496	128750	1.73	4.36E-02	transcription_start_site	+	131697	131697	-3074	12316	AN2392
678	CONTIG39	142666	143095	2.22	7.85E-03	transcription_start_site	-	138263	138263	-4617	12319	AN2394
678	CONTIG39	142666	143095	2.22	7.85E-03	transcription_start_site	-	138054	138054	-4826	12320	AN2394
2422	CONTIG39	137556	137830	1.23	1.93E-01	transcription_start_site	-	138054	138054	361	12320	AN2394
2422	CONTIG39	137556	137830	1.23	1.93E-01	transcription_start_site	-	137271	137271	-422	12321	AN2394
2422	CONTIG39	137556	137830	1.23	1.93E-01	transcription_start_site	-	138263	138263	570	12319	AN2394
678	CONTIG39	142666	143095	2.22	7.85E-03	transcription_start_site	-	142224	142224	-656	12322	AN2395
678	CONTIG39	142666	143095	2.22	7.85E-03	transcription_start_site	-	141827	141827	-1053	12323	AN2395
678	CONTIG39	142666	143095	2.22	7.85E-03	transcription_start_site	-	141561	141561	-1319	12324	AN2395
678	CONTIG39	142666	143095	2.22	7.85E-03	transcription_start_site	-	141318	141318	-1562	12325	AN2395
678	CONTIG39	142666	143095	2.22	7.85E-03	transcription_start_site	-	140851	140851	-2029	12326	AN2395
678	CONTIG39	142666	143095	2.22	7.85E-03	transcription_start_site	-	140600	140600	-2280	12327	AN2395
678	CONTIG39	142666	143095	2.22	7.85E-03	transcription_start_site	-	140395	140395	-2485	12328	AN2395
678	CONTIG39	142666	143095	2.22	7.85E-03	transcription_start_site	-	143798	143798	917	12330	AN2396
678	CONTIG39	142666	143095	2.22	7.85E-03	transcription_start_site	+	145043	145043	-2162	12331	AN2397
678	CONTIG39	142666	143095	2.22	7.85E-03	transcription_start_site	+	145236	145236	-2355	12332	AN2397
678	CONTIG39	142666	143095	2.22	7.85E-03	transcription_start_site	+	146510	146510	-3629	12333	AN2397
1866	CONTIG39	153171	153595	1.43	9.26E-02	transcription_start_site	-	149138	149138	-4245	12334	AN2398
1866	CONTIG39	153171	153595	1.43	9.26E-02	transcription_start_site	-	148887	148887	-4496	12335	AN2398
1866	CONTIG39	153171	153595	1.43	9.26E-02	transcription_start_site	-	148687	148687	-4696	12336	AN2398
1866	CONTIG39	153171	153595	1.43	9.26E-02	transcription_start_site	-	148459	148459	-4924	12337	AN2398
1866	CONTIG39	153171	153595	1.43	9.26E-02	transcription_start_site	-	150954	150954	-2429	12338	AN2399
1866	CONTIG39	153171	153595	1.43	9.26E-02	transcription_start_site	-	150836	150836	-2547	12339	AN2399
1866	CONTIG39	153171	153595	1.43	9.26E-02	transcription_start_site	-	150711	150711	-2672	12340	AN2399
1866	CONTIG39	153171	153595	1.43	9.26E-02	transcription_start_site	-	150545	150545	-2838	12341	AN2399
1866	CONTIG39	153171	153595	1.43	9.26E-02	transcription_start_site	-	150466	150466	-2917	12342	AN2399
2423	CONTIG39	154669	154934	1.23	1.93E-01	transcription_start_site	-	150954	150954	-3847	12338	AN2399
2423	CONTIG39	154669	154934	1.23	1.93E-01	transcription_start_site	-	150836	150836	-3965	12339	AN2399
2423	CONTIG39	154669	154934	1.23	1.93E-01	transcription_start_site	-	150711	150711	-4090	12340	AN2399
2423	CONTIG39	154669	154934	1.23	1.93E-01	transcription_start_site	-	150545	150545	-4256	12341	AN2399
2423	CONTIG39	154669	154934	1.23	1.93E-01	transcription_start_site	-	150466	150466	-4335	12342	AN2399
1866	CONTIG39	153171	153595	1.43	9.26E-02	transcription_start_site	-	153565	153565	182	12349	AN2401

1866	CONTIG39	153171	153595	1.43	9.26E-02	transcription_start_site	-	154543	154543	1160	12348	AN2401
1869	CONTIG39	160284	160628	1.43	1.08E-01	transcription_start_site	-	156763	156763	-3693	12344	AN2401
1869	CONTIG39	160284	160628	1.43	1.08E-01	transcription_start_site	-	156544	156544	-3912	12345	AN2401
1869	CONTIG39	160284	160628	1.43	1.08E-01	transcription_start_site	-	156047	156047	-4409	12346	AN2401
1869	CONTIG39	160284	160628	1.43	1.08E-01	transcription_start_site	-	155719	155719	-4737	12347	AN2401
2423	CONTIG39	154669	154934	1.23	1.93E-01	transcription_start_site	-	154543	154543	-258	12348	AN2401
2423	CONTIG39	154669	154934	1.23	1.93E-01	transcription_start_site	-	155719	155719	917	12347	AN2401
2423	CONTIG39	154669	154934	1.23	1.93E-01	transcription_start_site	-	153565	153565	-1236	12349	AN2401
1866	CONTIG39	153171	153595	1.43	9.26E-02	transcription_start_site	+	157427	157427	-4044	12350	AN2402
1866	CONTIG39	153171	153595	1.43	9.26E-02	transcription_start_site	+	157585	157585	-4202	12351	AN2402
2423	CONTIG39	154669	154934	1.23	1.93E-01	transcription_start_site	+	157427	157427	-2625	12350	AN2402
2423	CONTIG39	154669	154934	1.23	1.93E-01	transcription_start_site	+	157585	157585	-2783	12351	AN2402
1406	CONTIG39	167103	167472	1.63	5.88E-02	transcription_start_site	-	167363	167363	75	12352	AN2404
1406	CONTIG39	167103	167472	1.63	5.88E-02	transcription_start_site	-	166447	166447	-840	12353	AN2404
1406	CONTIG39	167103	167472	1.63	5.88E-02	transcription_start_site	+	171417	171417	-4129	12355	AN2406
1406	CONTIG39	167103	167472	1.63	5.88E-02	transcription_start_site	+	171795	171795	-4507	12356	AN2406
1407	CONTIG39	177078	177442	1.63	5.88E-02	transcription_start_site	+	176237	176237	1023	12364	AN2407
1407	CONTIG39	177078	177442	1.63	5.88E-02	transcription_start_site	+	179872	179872	-2612	12365	AN2408
679	CONTIG39	186993	187402	2.22	7.85E-03	transcription_start_site	+	188583	188583	-1385	12366	AN2410
679	CONTIG39	186993	187402	2.22	7.85E-03	transcription_start_site	+	188779	188779	-1581	12367	AN2410
2125	CONTIG39	187888	188464	1.33	1.44E-01	transcription_start_site	+	188583	188583	-407	12366	AN2410
2125	CONTIG39	187888	188464	1.33	1.44E-01	transcription_start_site	+	188779	188779	-603	12367	AN2410
679	CONTIG39	186993	187402	2.22	7.85E-03	transcription_start_site	+	190560	190560	-3362	12368	AN2411
679	CONTIG39	186993	187402	2.22	7.85E-03	transcription_start_site	+	190843	190843	-3645	12369	AN2411
679	CONTIG39	186993	187402	2.22	7.85E-03	transcription_start_site	+	191294	191294	-4096	12370	AN2411
679	CONTIG39	186993	187402	2.22	7.85E-03	transcription_start_site	+	191894	191894	-4696	12371	AN2411
2125	CONTIG39	187888	188464	1.33	1.44E-01	transcription_start_site	+	190560	190560	-2384	12368	AN2411
2125	CONTIG39	187888	188464	1.33	1.44E-01	transcription_start_site	+	190843	190843	-2667	12369	AN2411
2125	CONTIG39	187888	188464	1.33	1.44E-01	transcription_start_site	+	191294	191294	-3118	12370	AN2411
2125	CONTIG39	187888	188464	1.33	1.44E-01	transcription_start_site	+	191894	191894	-3718	12371	AN2411
1867	CONTIG39	201465	201884	1.43	9.26E-02	transcription_start_site	-	201592	201592	-82	12379	AN2414
1867	CONTIG39	201465	201884	1.43	9.26E-02	transcription_start_site	-	200863	200863	-811	12380	AN2414
1867	CONTIG39	201465	201884	1.43	9.26E-02	transcription_start_site	-	200092	200092	-1582	12381	AN2414
1867	CONTIG39	201465	201884	1.43	9.26E-02	transcription_start_site	+	201987	201987	-312	12382	AN2415
1867	CONTIG39	201465	201884	1.43	9.26E-02	transcription_start_site	+	202083	202083	-408	12383	AN2415
1867	CONTIG39	201465	201884	1.43	9.26E-02	transcription_start_site	+	202236	202236	-561	12384	AN2415
1867	CONTIG39	201465	201884	1.43	9.26E-02	transcription_start_site	+	205412	205412	-3737	12390	AN2417
1867	CONTIG39	201465	201884	1.43	9.26E-02	transcription_start_site	+	206323	206323	-4648	12391	AN2417
1867	CONTIG39	201465	201884	1.43	9.26E-02	transcription_start_site	+	206863	206863	-5188	12392	AN2417
1628	CONTIG39	211665	212014	1.53	8.01E-02	transcription_start_site	-	211187	211187	-652	12396	AN2418
1628	CONTIG39	211665	212014	1.53	8.01E-02	transcription_start_site	-	211047	211047	-792	12397	AN2418
1628	CONTIG39	211665	212014	1.53	8.01E-02	transcription_start_site	-	210618	210618	-1221	12398	AN2418
1628	CONTIG39	211665	212014	1.53	8.01E-02	transcription_start_site	-	210357	210357	-1482	12399	AN2418
1628	CONTIG39	211665	212014	1.53	8.01E-02	transcription_start_site	+	211578	211578	261	12400	AN2419
1628	CONTIG39	211665	212014	1.53	8.01E-02	transcription_start_site	+	212859	212859	-1019	12401	AN2419
1628	CONTIG39	211665	212014	1.53	8.01E-02	transcription_start_site	+	214645	214645	-2805	12402	AN2420
79	CONTIG39	224930	225205	3.3	0.00E+00	transcription_start_site	+	228887	228887	-3819	12403	AN2421
79	CONTIG39	224930	225205	3.3	0.00E+00	transcription_start_site	+	229398	229398	-4330	12404	AN2421
79	CONTIG39	224930	225205	3.3	0.00E+00	transcription_start_site	+	229949	229949	-4881	12405	AN2421
130	CONTIG39	223895	224454	3.11	0.00E+00	transcription_start_site	+	228887	228887	-4712	12403	AN2421
130	CONTIG39	223895	224454	3.11	0.00E+00	transcription_start_site	+	229398	229398	-5223	12404	AN2421
518	CONTIG39	226955	227389	2.42	4.46E-04	transcription_start_site	+	228887	228887	-1715	12403	AN2421
518	CONTIG39	226955	227389	2.42	4.46E-04	transcription_start_site	+	229398	229398	-2226	12404	AN2421
518	CONTIG39	226955	227389	2.42	4.46E-04	transcription_start_site	+	229949	229949	-2777	12405	AN2421
680	CONTIG39	228090	228520	2.22	7.85E-03	transcription_start_site	+	228887	228887	-582	12403	AN2421
680	CONTIG39	228090	228520	2.22	7.85E-03	transcription_start_site	+	229398	229398	-1093	12404	AN2421
680	CONTIG39	228090	228520	2.22	7.85E-03	transcription_start_site	+	229949	229949	-1644	12405	AN2421

1119	CONTIG39	228621	229270	1.82	3.08E-02	transcription_start_site	+	228887	228887	58	12403 AN2421
1119	CONTIG39	228621	229270	1.82	3.08E-02	transcription_start_site	+	229398	229398	-452	12404 AN2421
1119	CONTIG39	228621	229270	1.82	3.08E-02	transcription_start_site	+	229949	229949	-1003	12405 AN2421
518	CONTIG39	226955	227389	2.42	4.46E-04	transcription_start_site	+	231469	231469	-4297	12406 AN2422
518	CONTIG39	226955	227389	2.42	4.46E-04	transcription_start_site	+	231714	231714	-4542	12407 AN2422
518	CONTIG39	226955	227389	2.42	4.46E-04	transcription_start_site	+	231827	231827	-4655	12408 AN2422
518	CONTIG39	226955	227389	2.42	4.46E-04	transcription_start_site	+	232260	232260	-5088	12409 AN2422
680	CONTIG39	228090	228520	2.22	7.85E-03	transcription_start_site	+	231469	231469	-3164	12406 AN2422
680	CONTIG39	228090	228520	2.22	7.85E-03	transcription_start_site	+	231714	231714	-3409	12407 AN2422
680	CONTIG39	228090	228520	2.22	7.85E-03	transcription_start_site	+	231827	231827	-3522	12408 AN2422
680	CONTIG39	228090	228520	2.22	7.85E-03	transcription_start_site	+	232260	232260	-3955	12409 AN2422
680	CONTIG39	228090	228520	2.22	7.85E-03	transcription_start_site	+	233243	233243	-4938	12410 AN2422
1119	CONTIG39	228621	229270	1.82	3.08E-02	transcription_start_site	+	231469	231469	-2523	12406 AN2422
1119	CONTIG39	228621	229270	1.82	3.08E-02	transcription_start_site	+	231714	231714	-2768	12407 AN2422
1119	CONTIG39	228621	229270	1.82	3.08E-02	transcription_start_site	+	231827	231827	-2881	12408 AN2422
1119	CONTIG39	228621	229270	1.82	3.08E-02	transcription_start_site	+	232260	232260	-3314	12409 AN2422
1119	CONTIG39	228621	229270	1.82	3.08E-02	transcription_start_site	+	233243	233243	-4297	12410 AN2422
38	CONTIG40	14259	14538	3.49	0.00E+00	transcription_start_site	+	15158	15158	-759	12415 AN2425
38	CONTIG40	14259	14538	3.49	0.00E+00	transcription_start_site	+	15298	15298	-899	12416 AN2425
38	CONTIG40	14259	14538	3.49	0.00E+00	transcription_start_site	+	15590	15590	-1191	12417 AN2425
677	CONTIG40	12242	12886	2.22	5.41E-03	transcription_start_site	+	15158	15158	-2594	12415 AN2425
677	CONTIG40	12242	12886	2.22	5.41E-03	transcription_start_site	+	15298	15298	-2734	12416 AN2425
677	CONTIG40	12242	12886	2.22	5.41E-03	transcription_start_site	+	15590	15590	-3026	12417 AN2425
38	CONTIG40	14259	14538	3.49	0.00E+00	transcription_start_site	+	19059	19059	-4660	12418 AN2426
38	CONTIG40	14259	14538	3.49	0.00E+00	transcription_start_site	+	19235	19235	-4836	12419 AN2426
847	CONTIG40	18604	18878	2.06	2.12E-02	transcription_start_site	+	19059	19059	-318	12418 AN2426
847	CONTIG40	18604	18878	2.06	2.12E-02	transcription_start_site	+	19235	19235	-494	12419 AN2426
556	CONTIG40	21904	22338	2.38	7.85E-03	transcription_start_site	+	22461	22461	-340	12420 AN2427
847	CONTIG40	18604	18878	2.06	2.12E-02	transcription_start_site	+	22461	22461	-3720	12420 AN2427
682	CONTIG40	47193	47677	2.22	1.30E-02	transcription_start_site	-	47314	47314	-121	12440 AN2435
682	CONTIG40	47193	47677	2.22	1.30E-02	transcription_start_site	-	46816	46816	-619	12441 AN2435
1143	CONTIG40	48228	48817	1.8	3.33E-02	transcription_start_site	-	47314	47314	-1208	12440 AN2435
1143	CONTIG40	48228	48817	1.8	3.33E-02	transcription_start_site	-	46816	46816	-1706	12441 AN2435
682	CONTIG40	47193	47677	2.22	1.30E-02	transcription_start_site	+	49235	49235	-1800	12442 AN2436
682	CONTIG40	47193	47677	2.22	1.30E-02	transcription_start_site	+	49426	49426	-1991	12443 AN2436
682	CONTIG40	47193	47677	2.22	1.30E-02	transcription_start_site	+	49600	49600	-2165	12444 AN2436
682	CONTIG40	47193	47677	2.22	1.30E-02	transcription_start_site	+	49846	49846	-2411	12445 AN2436
682	CONTIG40	47193	47677	2.22	1.30E-02	transcription_start_site	+	51001	51001	-3566	12446 AN2436
1143	CONTIG40	48228	48817	1.8	3.33E-02	transcription_start_site	+	49235	49235	-712	12442 AN2436
1143	CONTIG40	48228	48817	1.8	3.33E-02	transcription_start_site	+	49426	49426	-903	12443 AN2436
1143	CONTIG40	48228	48817	1.8	3.33E-02	transcription_start_site	+	49600	49600	-1077	12444 AN2436
1143	CONTIG40	48228	48817	1.8	3.33E-02	transcription_start_site	+	49846	49846	-1323	12445 AN2436
1143	CONTIG40	48228	48817	1.8	3.33E-02	transcription_start_site	+	51001	51001	-2478	12446 AN2436
682	CONTIG40	47193	47677	2.22	1.30E-02	transcription_start_site	+	51971	51971	-4536	12447 AN2437
682	CONTIG40	47193	47677	2.22	1.30E-02	transcription_start_site	+	52310	52310	-4875	12448 AN2437
1143	CONTIG40	48228	48817	1.8	3.33E-02	transcription_start_site	+	51971	51971	-3448	12447 AN2437
1143	CONTIG40	48228	48817	1.8	3.33E-02	transcription_start_site	+	52310	52310	-3787	12448 AN2437
413	CONTIG40	109295	109569	2.59	3.61E-03	transcription_start_site	-	109745	109745	313	12506 AN2459
413	CONTIG40	109295	109569	2.59	3.61E-03	transcription_start_site	-	105487	105487	-3945	12507 AN2459
1759	CONTIG40	111240	111819	1.48	8.00E-02	transcription_start_site	-	109745	109745	-1784	12506 AN2459
413	CONTIG40	109295	109569	2.59	3.61E-03	transcription_start_site	+	111434	111434	-2002	12508 AN2460
1759	CONTIG40	111240	111819	1.48	8.00E-02	transcription_start_site	+	111434	111434	95	12508 AN2460
413	CONTIG40	109295	109569	2.59	3.61E-03	transcription_start_site	+	113743	113743	-4311	12509 AN2461
413	CONTIG40	109295	109569	2.59	3.61E-03	transcription_start_site	+	113818	113818	-4386	12510 AN2461
413	CONTIG40	109295	109569	2.59	3.61E-03	transcription_start_site	+	113947	113947	-4515	12511 AN2461
413	CONTIG40	109295	109569	2.59	3.61E-03	transcription_start_site	+	114299	114299	-4867	12512 AN2461
1759	CONTIG40	111240	111819	1.48	8.00E-02	transcription_start_site	+	113743	113743	-2213	12509 AN2461

1759	CONTIG40	111240	111819	1.48	8.00E-02	transcription_start_site	+	113818	113818	-2288	12510 AN2461
1759	CONTIG40	111240	111819	1.48	8.00E-02	transcription_start_site	+	113947	113947	-2417	12511 AN2461
1759	CONTIG40	111240	111819	1.48	8.00E-02	transcription_start_site	+	114299	114299	-2769	12512 AN2461
1759	CONTIG40	111240	111819	1.48	8.00E-02	transcription_start_site	+	114975	114975	-3445	12513 AN2461
1759	CONTIG40	111240	111819	1.48	8.00E-02	transcription_start_site	+	115259	115259	-3729	12514 AN2461
1759	CONTIG40	111240	111819	1.48	8.00E-02	transcription_start_site	+	115638	115638	-4108	12515 AN2461
1759	CONTIG40	111240	111819	1.48	8.00E-02	transcription_start_site	+	116235	116235	-4705	12516 AN2461
1759	CONTIG40	111240	111819	1.48	8.00E-02	transcription_start_site	+	116687	116687	-5157	12517 AN2461
735	CONTIG40	133741	134005	2.17	1.57E-02	transcription_start_site	-	129221	129221	-4652	12522 AN2464
735	CONTIG40	133741	134005	2.17	1.57E-02	transcription_start_site	-	129037	129037	-4836	12523 AN2464
735	CONTIG40	133741	134005	2.17	1.57E-02	transcription_start_site	-	133549	133549	-324	12525 AN2465
735	CONTIG40	133741	134005	2.17	1.57E-02	transcription_start_site	-	133151	133151	-722	12526 AN2465
735	CONTIG40	133741	134005	2.17	1.57E-02	transcription_start_site	-	131703	131703	-2170	12527 AN2465
735	CONTIG40	133741	134005	2.17	1.57E-02	transcription_start_site	-	131323	131323	-2550	12528 AN2465
735	CONTIG40	133741	134005	2.17	1.57E-02	transcription_start_site	-	131090	131090	-2783	12529 AN2465
735	CONTIG40	133741	134005	2.17	1.57E-02	transcription_start_site	-	130840	130840	-3033	12530 AN2465
1989	CONTIG40	138471	139480	1.38	1.59E-01	transcription_start_site	-	133549	133549	-5426	12525 AN2465
31	CONTIG40	140646	141070	3.54	0.00E+00	transcription_start_site	-	139652	139652	-1206	12531 AN2466
31	CONTIG40	140646	141070	3.54	0.00E+00	transcription_start_site	-	139478	139478	-1380	12532 AN2466
31	CONTIG40	140646	141070	3.54	0.00E+00	transcription_start_site	-	139219	139219	-1639	12533 AN2466
31	CONTIG40	140646	141070	3.54	0.00E+00	transcription_start_site	-	139043	139043	-1815	12534 AN2466
213	CONTIG40	140031	140600	2.91	1.01E-03	transcription_start_site	-	139652	139652	-663	12531 AN2466
213	CONTIG40	140031	140600	2.91	1.01E-03	transcription_start_site	-	139478	139478	-837	12532 AN2466
213	CONTIG40	140031	140600	2.91	1.01E-03	transcription_start_site	-	139219	139219	-1096	12533 AN2466
213	CONTIG40	140031	140600	2.91	1.01E-03	transcription_start_site	-	139043	139043	-1272	12534 AN2466
1388	CONTIG40	142741	143020	1.64	8.01E-02	transcription_start_site	-	139652	139652	-3228	12531 AN2466
1388	CONTIG40	142741	143020	1.64	8.01E-02	transcription_start_site	-	139478	139478	-3402	12532 AN2466
1388	CONTIG40	142741	143020	1.64	8.01E-02	transcription_start_site	-	139219	139219	-3661	12533 AN2466
1388	CONTIG40	142741	143020	1.64	8.01E-02	transcription_start_site	-	139043	139043	-3837	12534 AN2466
1989	CONTIG40	138471	139480	1.38	1.59E-01	transcription_start_site	-	139043	139043	67	12534 AN2466
1989	CONTIG40	138471	139480	1.38	1.59E-01	transcription_start_site	-	139219	139219	243	12533 AN2466
1989	CONTIG40	138471	139480	1.38	1.59E-01	transcription_start_site	-	139478	139478	502	12532 AN2466
1989	CONTIG40	138471	139480	1.38	1.59E-01	transcription_start_site	-	139652	139652	676	12531 AN2466
1388	CONTIG40	142741	143020	1.64	8.01E-02	transcription_start_site	-	143040	143040	159	12535 AN2467
31	CONTIG40	140646	141070	3.54	0.00E+00	transcription_start_site	+	143760	143760	-2902	12536 AN2468
31	CONTIG40	140646	141070	3.54	0.00E+00	transcription_start_site	+	143965	143965	-3107	12537 AN2468
31	CONTIG40	140646	141070	3.54	0.00E+00	transcription_start_site	+	144126	144126	-3268	12538 AN2468
31	CONTIG40	140646	141070	3.54	0.00E+00	transcription_start_site	+	144465	144465	-3607	12539 AN2468
31	CONTIG40	140646	141070	3.54	0.00E+00	transcription_start_site	+	144697	144697	-3839	12540 AN2468
31	CONTIG40	140646	141070	3.54	0.00E+00	transcription_start_site	+	144842	144842	-3984	12541 AN2468
213	CONTIG40	140031	140600	2.91	1.01E-03	transcription_start_site	+	143760	143760	-3444	12536 AN2468
213	CONTIG40	140031	140600	2.91	1.01E-03	transcription_start_site	+	143965	143965	-3649	12537 AN2468
213	CONTIG40	140031	140600	2.91	1.01E-03	transcription_start_site	+	144126	144126	-3810	12538 AN2468
213	CONTIG40	140031	140600	2.91	1.01E-03	transcription_start_site	+	144465	144465	-4149	12539 AN2468
213	CONTIG40	140031	140600	2.91	1.01E-03	transcription_start_site	+	144697	144697	-4381	12540 AN2468
213	CONTIG40	140031	140600	2.91	1.01E-03	transcription_start_site	+	144842	144842	-4526	12541 AN2468
1388	CONTIG40	142741	143020	1.64	8.01E-02	transcription_start_site	+	143760	143760	-879	12536 AN2468
1388	CONTIG40	142741	143020	1.64	8.01E-02	transcription_start_site	+	143965	143965	-1084	12537 AN2468
1388	CONTIG40	142741	143020	1.64	8.01E-02	transcription_start_site	+	144126	144126	-1245	12538 AN2468
1388	CONTIG40	142741	143020	1.64	8.01E-02	transcription_start_site	+	144465	144465	-1584	12539 AN2468
1388	CONTIG40	142741	143020	1.64	8.01E-02	transcription_start_site	+	144697	144697	-1816	12540 AN2468
1388	CONTIG40	142741	143020	1.64	8.01E-02	transcription_start_site	+	144842	144842	-1961	12541 AN2468
1989	CONTIG40	138471	139480	1.38	1.59E-01	transcription_start_site	+	143760	143760	-4784	12536 AN2468
1989	CONTIG40	138471	139480	1.38	1.59E-01	transcription_start_site	+	143965	143965	-4989	12537 AN2468
1989	CONTIG40	138471	139480	1.38	1.59E-01	transcription_start_site	+	144126	144126	-5150	12538 AN2468
1989	CONTIG40	138471	139480	1.38	1.59E-01	transcription_start_site	+	144465	144465	-5489	12539 AN2468
331	CONTIG41	391	730	2.72	4.61E-03	transcription_start_site	+	4066	4066	-3505	12544 AN2470

331	CONTIG41	391	730	2.72	4.61E-03	transcription_start_site	+	4293	4293	-3732	12545	AN2470
331	CONTIG41	391	730	2.72	4.61E-03	transcription_start_site	+	4473	4473	-3912	12546	AN2470
331	CONTIG41	391	730	2.72	4.61E-03	transcription_start_site	+	5335	5335	-4774	12547	AN2470
1389	CONTIG41	9693	10198	1.64	1.08E-01	transcription_start_site	-	6106	6106	-3839	12548	AN2471
1389	CONTIG41	9693	10198	1.64	1.08E-01	transcription_start_site	-	6010	6010	-3935	12549	AN2471
1389	CONTIG41	9693	10198	1.64	1.08E-01	transcription_start_site	-	5851	5851	-4094	12550	AN2471
246	CONTIG41	14792	15426	2.84	0.00E+00	transcription_start_site	-	9879	9879	-5230	12555	AN2473
1389	CONTIG41	9693	10198	1.64	1.08E-01	transcription_start_site	-	9879	9879	-66	12555	AN2473
1389	CONTIG41	9693	10198	1.64	1.08E-01	transcription_start_site	-	9511	9511	-434	12556	AN2473
1389	CONTIG41	9693	10198	1.64	1.08E-01	transcription_start_site	-	8869	8869	-1076	12557	AN2473
246	CONTIG41	14792	15426	2.84	0.00E+00	transcription_start_site	-	11990	11990	-3119	12558	AN2474
246	CONTIG41	14792	15426	2.84	0.00E+00	transcription_start_site	-	11881	11881	-3228	12559	AN2474
504	CONTIG41	16507	17001	2.44	4.81E-03	transcription_start_site	-	11990	11990	-4764	12558	AN2474
504	CONTIG41	16507	17001	2.44	4.81E-03	transcription_start_site	-	11881	11881	-4873	12559	AN2474
246	CONTIG41	14792	15426	2.84	0.00E+00	transcription_start_site	-	14444	14444	-665	12560	AN2475
246	CONTIG41	14792	15426	2.84	0.00E+00	transcription_start_site	-	14094	14094	-1015	12561	AN2475
504	CONTIG41	16507	17001	2.44	4.81E-03	transcription_start_site	-	14444	14444	-2310	12560	AN2475
504	CONTIG41	16507	17001	2.44	4.81E-03	transcription_start_site	-	14094	14094	-2660	12561	AN2475
59	CONTIG41	61070	61404	3.4	2.16E-04	transcription_start_site	+	60263	60263	974	12596	AN2487
59	CONTIG41	61070	61404	3.4	2.16E-04	transcription_start_site	+	61732	61732	-495	12597	AN2488
59	CONTIG41	61070	61404	3.4	2.16E-04	transcription_start_site	+	61926	61926	-689	12598	AN2488
59	CONTIG41	61070	61404	3.4	2.16E-04	transcription_start_site	+	62089	62089	-852	12599	AN2488
59	CONTIG41	61070	61404	3.4	2.16E-04	transcription_start_site	+	62262	62262	-1025	12600	AN2488
295	CONTIG42	28803	29092	2.77	1.54E-03	transcription_start_site	+	30566	30566	-1618	12626	AN2498
295	CONTIG42	28803	29092	2.77	1.54E-03	transcription_start_site	+	31408	31408	-2460	12627	AN2498
295	CONTIG42	28803	29092	2.77	1.54E-03	transcription_start_site	+	31727	31727	-2779	12628	AN2498
916	CONTIG42	36828	37342	1.99	2.62E-02	transcription_start_site	-	34922	34922	-2163	12629	AN2499
916	CONTIG42	36828	37342	1.99	2.62E-02	transcription_start_site	-	34854	34854	-2231	12630	AN2499
916	CONTIG42	36828	37342	1.99	2.62E-02	transcription_start_site	-	34442	34442	-2643	12631	AN2499
916	CONTIG42	36828	37342	1.99	2.62E-02	transcription_start_site	-	33287	33287	-3798	12632	AN2499
1339	CONTIG42	39754	40043	1.67	6.84E-02	transcription_start_site	-	34922	34922	-4976	12629	AN2499
1339	CONTIG42	39754	40043	1.67	6.84E-02	transcription_start_site	-	34854	34854	-5044	12630	AN2499
2047	CONTIG42	33843	34102	1.36	1.66E-01	transcription_start_site	-	34442	34442	469	12631	AN2499
2047	CONTIG42	33843	34102	1.36	1.66E-01	transcription_start_site	-	33287	33287	-685	12632	AN2499
2047	CONTIG42	33843	34102	1.36	1.66E-01	transcription_start_site	-	34854	34854	881	12630	AN2499
2047	CONTIG42	33843	34102	1.36	1.66E-01	transcription_start_site	-	34922	34922	949	12629	AN2499
916	CONTIG42	36828	37342	1.99	2.62E-02	transcription_start_site	-	36108	36108	-977	12633	AN2500
916	CONTIG42	36828	37342	1.99	2.62E-02	transcription_start_site	-	35960	35960	-1125	12634	AN2500
916	CONTIG42	36828	37342	1.99	2.62E-02	transcription_start_site	-	35291	35291	-1794	12635	AN2500
1339	CONTIG42	39754	40043	1.67	6.84E-02	transcription_start_site	-	36108	36108	-3790	12633	AN2500
1339	CONTIG42	39754	40043	1.67	6.84E-02	transcription_start_site	-	35960	35960	-3938	12634	AN2500
1339	CONTIG42	39754	40043	1.67	6.84E-02	transcription_start_site	-	35291	35291	-4607	12635	AN2500
1340	CONTIG42	40954	41458	1.67	6.84E-02	transcription_start_site	-	36108	36108	-5098	12633	AN2500
1340	CONTIG42	40954	41458	1.67	6.84E-02	transcription_start_site	-	35960	35960	-5246	12634	AN2500
916	CONTIG42	36828	37342	1.99	2.62E-02	transcription_start_site	+	37206	37206	-121	12636	AN2501
2047	CONTIG42	33843	34102	1.36	1.66E-01	transcription_start_site	+	37206	37206	-3233	12636	AN2501
20	CONTIG42	43596	44396	3.61	0.00E+00	transcription_start_site	-	40040	40040	-3956	12637	AN2502
81	CONTIG42	42151	42579	3.3	0.00E+00	transcription_start_site	-	40040	40040	-2325	12637	AN2502
612	CONTIG42	42696	43270	2.3	8.81E-03	transcription_start_site	-	40040	40040	-2943	12637	AN2502
1339	CONTIG42	39754	40043	1.67	6.84E-02	transcription_start_site	-	40040	40040	141	12637	AN2502
1340	CONTIG42	40954	41458	1.67	6.84E-02	transcription_start_site	-	40040	40040	-1166	12637	AN2502
20	CONTIG42	43596	44396	3.61	0.00E+00	transcription_start_site	-	43689	43689	-307	12639	AN2503
20	CONTIG42	43596	44396	3.61	0.00E+00	transcription_start_site	-	44351	44351	355	12638	AN2503
20	CONTIG42	43596	44396	3.61	0.00E+00	transcription_start_site	-	43556	43556	-440	12640	AN2503
20	CONTIG42	43596	44396	3.61	0.00E+00	transcription_start_site	-	43430	43430	-566	12641	AN2503
20	CONTIG42	43596	44396	3.61	0.00E+00	transcription_start_site	-	43272	43272	-724	12642	AN2503
20	CONTIG42	43596	44396	3.61	0.00E+00	transcription_start_site	-	42981	42981	-1015	12643	AN2503

81	CONTIG42	42151	42579	3.3	0.00E+00	transcription_start_site	-	42981	42981	616	12643	AN2503
81	CONTIG42	42151	42579	3.3	0.00E+00	transcription_start_site	-	43272	43272	907	12642	AN2503
81	CONTIG42	42151	42579	3.3	0.00E+00	transcription_start_site	-	43430	43430	1065	12641	AN2503
81	CONTIG42	42151	42579	3.3	0.00E+00	transcription_start_site	-	43556	43556	1191	12640	AN2503
612	CONTIG42	42696	43270	2.3	8.81E-03	transcription_start_site	-	42981	42981	-2	12643	AN2503
612	CONTIG42	42696	43270	2.3	8.81E-03	transcription_start_site	-	43272	43272	289	12642	AN2503
612	CONTIG42	42696	43270	2.3	8.81E-03	transcription_start_site	-	43430	43430	447	12641	AN2503
612	CONTIG42	42696	43270	2.3	8.81E-03	transcription_start_site	-	43556	43556	573	12640	AN2503
612	CONTIG42	42696	43270	2.3	8.81E-03	transcription_start_site	-	43689	43689	706	12639	AN2503
235	CONTIG42	71042	71301	2.88	1.01E-03	transcription_start_site	-	67236	67236	-3935	12657	AN2507
235	CONTIG42	71042	71301	2.88	1.01E-03	transcription_start_site	-	67178	67178	-3993	12658	AN2507
1112	CONTIG42	68702	68986	1.83	4.36E-02	transcription_start_site	-	67236	67236	-1608	12657	AN2507
1112	CONTIG42	68702	68986	1.83	4.36E-02	transcription_start_site	-	67178	67178	-1666	12658	AN2507
235	CONTIG42	71042	71301	2.88	1.01E-03	transcription_start_site	+	70246	70246	925	12660	AN2508
1112	CONTIG42	68702	68986	1.83	4.36E-02	transcription_start_site	+	68953	68953	-109	12659	AN2508
1112	CONTIG42	68702	68986	1.83	4.36E-02	transcription_start_site	+	70246	70246	-1402	12660	AN2508
235	CONTIG42	71042	71301	2.88	1.01E-03	transcription_start_site	+	72109	72109	-937	12661	AN2509
235	CONTIG42	71042	71301	2.88	1.01E-03	transcription_start_site	+	72478	72478	-1306	12662	AN2509
235	CONTIG42	71042	71301	2.88	1.01E-03	transcription_start_site	+	73735	73735	-2563	12663	AN2509
235	CONTIG42	71042	71301	2.88	1.01E-03	transcription_start_site	+	73869	73869	-2697	12664	AN2509
1112	CONTIG42	68702	68986	1.83	4.36E-02	transcription_start_site	+	72109	72109	-3265	12661	AN2509
1112	CONTIG42	68702	68986	1.83	4.36E-02	transcription_start_site	+	72478	72478	-3634	12662	AN2509
1112	CONTIG42	68702	68986	1.83	4.36E-02	transcription_start_site	+	73735	73735	-4891	12663	AN2509
1112	CONTIG42	68702	68986	1.83	4.36E-02	transcription_start_site	+	73869	73869	-5025	12664	AN2509
235	CONTIG42	71042	71301	2.88	1.01E-03	transcription_start_site	+	76281	76281	-5109	12665	AN2510
1341	CONTIG42	96606	97028	1.67	6.84E-02	transcription_start_site	-	96163	96163	-654	12686	AN2517
1341	CONTIG42	96606	97028	1.67	6.84E-02	transcription_start_site	-	95774	95774	-1043	12687	AN2517
1341	CONTIG42	96606	97028	1.67	6.84E-02	transcription_start_site	-	95556	95556	-1261	12688	AN2517
1341	CONTIG42	96606	97028	1.67	6.84E-02	transcription_start_site	-	95394	95394	-1423	12689	AN2517
1341	CONTIG42	96606	97028	1.67	6.84E-02	transcription_start_site	+	97655	97655	-838	12690	AN2518
1341	CONTIG42	96606	97028	1.67	6.84E-02	transcription_start_site	+	97735	97735	-918	12691	AN2518
1341	CONTIG42	96606	97028	1.67	6.84E-02	transcription_start_site	+	98021	98021	-1204	12692	AN2518
1341	CONTIG42	96606	97028	1.67	6.84E-02	transcription_start_site	+	98434	98434	-1617	12693	AN2518
971	CONTIG43	4071	4795	1.93	3.31E-03	transcription_start_site	-	2519	2519	-1914	12696	AN2520
971	CONTIG43	4071	4795	1.93	3.31E-03	transcription_start_site	-	2345	2345	-2088	12697	AN2520
971	CONTIG43	4071	4795	1.93	3.31E-03	transcription_start_site	-	1501	1501	-2932	12698	AN2520
1875	CONTIG43	6376	6650	1.42	5.88E-02	transcription_start_site	-	2519	2519	-3994	12696	AN2520
1875	CONTIG43	6376	6650	1.42	5.88E-02	transcription_start_site	-	2345	2345	-4168	12697	AN2520
1875	CONTIG43	6376	6650	1.42	5.88E-02	transcription_start_site	-	1501	1501	-5012	12698	AN2520
971	CONTIG43	4071	4795	1.93	3.31E-03	transcription_start_site	+	4627	4627	-194	12703	AN2521
971	CONTIG43	4071	4795	1.93	3.31E-03	transcription_start_site	+	4067	4067	366	12702	AN2521
971	CONTIG43	4071	4795	1.93	3.31E-03	transcription_start_site	+	3767	3767	666	12701	AN2521
971	CONTIG43	4071	4795	1.93	3.31E-03	transcription_start_site	+	3600	3600	833	12700	AN2521
971	CONTIG43	4071	4795	1.93	3.31E-03	transcription_start_site	+	3497	3497	936	12699	AN2521
971	CONTIG43	4071	4795	1.93	3.31E-03	transcription_start_site	+	5677	5677	-1244	12704	AN2521
971	CONTIG43	4071	4795	1.93	3.31E-03	transcription_start_site	+	5763	5763	-1330	12705	AN2521
1875	CONTIG43	6376	6650	1.42	5.88E-02	transcription_start_site	+	5763	5763	750	12705	AN2521
1875	CONTIG43	6376	6650	1.42	5.88E-02	transcription_start_site	+	5677	5677	836	12704	AN2521
878	CONTIG43	10521	10850	2.02	5.20E-03	transcription_start_site	-	7518	7518	-3167	12706	AN2522
878	CONTIG43	10521	10850	2.02	5.20E-03	transcription_start_site	-	7426	7426	-3259	12707	AN2522
1875	CONTIG43	6376	6650	1.42	5.88E-02	transcription_start_site	-	7426	7426	913	12707	AN2522
1875	CONTIG43	6376	6650	1.42	5.88E-02	transcription_start_site	-	7518	7518	1005	12706	AN2522
1876	CONTIG43	11416	11685	1.42	5.88E-02	transcription_start_site	-	7518	7518	-4032	12706	AN2522
1876	CONTIG43	11416	11685	1.42	5.88E-02	transcription_start_site	-	7426	7426	-4124	12707	AN2522
878	CONTIG43	10521	10850	2.02	5.20E-03	transcription_start_site	+	12355	12355	-1669	12708	AN2523
878	CONTIG43	10521	10850	2.02	5.20E-03	transcription_start_site	+	12509	12509	-1823	12709	AN2523
878	CONTIG43	10521	10850	2.02	5.20E-03	transcription_start_site	+	13049	13049	-2363	12710	AN2523

878	CONTIG43	10521	10850	2.02	5.20E-03	transcription_start_site	+	13466	13466	-2780	12711	AN2523
878	CONTIG43	10521	10850	2.02	5.20E-03	transcription_start_site	+	14367	14367	-3681	12712	AN2523
878	CONTIG43	10521	10850	2.02	5.20E-03	transcription_start_site	+	15254	15254	-4568	12713	AN2523
1876	CONTIG43	11416	11685	1.42	5.88E-02	transcription_start_site	+	12355	12355	-804	12708	AN2523
1876	CONTIG43	11416	11685	1.42	5.88E-02	transcription_start_site	+	12509	12509	-958	12709	AN2523
1876	CONTIG43	11416	11685	1.42	5.88E-02	transcription_start_site	+	13049	13049	-1498	12710	AN2523
1876	CONTIG43	11416	11685	1.42	5.88E-02	transcription_start_site	+	13466	13466	-1915	12711	AN2523
1876	CONTIG43	11416	11685	1.42	5.88E-02	transcription_start_site	+	14367	14367	-2816	12712	AN2523
1876	CONTIG43	11416	11685	1.42	5.88E-02	transcription_start_site	+	15254	15254	-3703	12713	AN2523
1877	CONTIG43	24843	25182	1.42	5.88E-02	transcription_start_site	-	23455	23455	-1557	12714	AN2525
1877	CONTIG43	24843	25182	1.42	5.88E-02	transcription_start_site	-	23052	23052	-1960	12715	AN2525
1877	CONTIG43	24843	25182	1.42	5.88E-02	transcription_start_site	-	24981	24981	-31	12716	AN2526
1877	CONTIG43	24843	25182	1.42	5.88E-02	transcription_start_site	+	25785	25785	-772	12717	AN2527
926	CONTIG43	37967	38241	1.98	6.61E-03	transcription_start_site	-	33742	33742	-4362	12723	AN2530
926	CONTIG43	37967	38241	1.98	6.61E-03	transcription_start_site	-	37808	37808	-296	12724	AN2531
926	CONTIG43	37967	38241	1.98	6.61E-03	transcription_start_site	-	37579	37579	-525	12725	AN2531
926	CONTIG43	37967	38241	1.98	6.61E-03	transcription_start_site	-	37314	37314	-790	12726	AN2531
926	CONTIG43	37967	38241	1.98	6.61E-03	transcription_start_site	-	37054	37054	-1050	12727	AN2531
926	CONTIG43	37967	38241	1.98	6.61E-03	transcription_start_site	-	36841	36841	-1263	12728	AN2531
926	CONTIG43	37967	38241	1.98	6.61E-03	transcription_start_site	-	36375	36375	-1729	12729	AN2531
926	CONTIG43	37967	38241	1.98	6.61E-03	transcription_start_site	+	38873	38873	-769	12730	AN2532
926	CONTIG43	37967	38241	1.98	6.61E-03	transcription_start_site	+	39262	39262	-1158	12731	AN2532
926	CONTIG43	37967	38241	1.98	6.61E-03	transcription_start_site	+	39890	39890	-1786	12732	AN2532
926	CONTIG43	37967	38241	1.98	6.61E-03	transcription_start_site	+	40909	40909	-2805	12733	AN2532
659	CONTIG43	49067	49333	2.23	1.80E-03	transcription_start_site	-	45242	45242	-3958	12734	AN2533
659	CONTIG43	49067	49333	2.23	1.80E-03	transcription_start_site	-	44894	44894	-4306	12735	AN2533
659	CONTIG43	49067	49333	2.23	1.80E-03	transcription_start_site	-	44754	44754	-4446	12736	AN2533
659	CONTIG43	49067	49333	2.23	1.80E-03	transcription_start_site	-	44257	44257	-4943	12737	AN2533
837	CONTIG43	49512	50016	2.06	4.61E-03	transcription_start_site	-	45242	45242	-4522	12734	AN2533
837	CONTIG43	49512	50016	2.06	4.61E-03	transcription_start_site	-	44894	44894	-4870	12735	AN2533
837	CONTIG43	49512	50016	2.06	4.61E-03	transcription_start_site	-	44754	44754	-5010	12736	AN2533
659	CONTIG43	49067	49333	2.23	1.80E-03	transcription_start_site	-	48893	48893	-307	12745	AN2535
659	CONTIG43	49067	49333	2.23	1.80E-03	transcription_start_site	-	48730	48730	-470	12746	AN2535
659	CONTIG43	49067	49333	2.23	1.80E-03	transcription_start_site	-	48565	48565	-635	12747	AN2535
837	CONTIG43	49512	50016	2.06	4.61E-03	transcription_start_site	-	48893	48893	-871	12745	AN2535
837	CONTIG43	49512	50016	2.06	4.61E-03	transcription_start_site	-	48730	48730	-1034	12746	AN2535
837	CONTIG43	49512	50016	2.06	4.61E-03	transcription_start_site	-	48565	48565	-1199	12747	AN2535
659	CONTIG43	49067	49333	2.23	1.80E-03	transcription_start_site	+	52816	52816	-3616	12748	AN2537
659	CONTIG43	49067	49333	2.23	1.80E-03	transcription_start_site	+	53157	53157	-3957	12749	AN2537
659	CONTIG43	49067	49333	2.23	1.80E-03	transcription_start_site	+	53383	53383	-4183	12750	AN2537
659	CONTIG43	49067	49333	2.23	1.80E-03	transcription_start_site	+	53729	53729	-4529	12751	AN2537
837	CONTIG43	49512	50016	2.06	4.61E-03	transcription_start_site	+	52816	52816	-3052	12748	AN2537
837	CONTIG43	49512	50016	2.06	4.61E-03	transcription_start_site	+	53157	53157	-3393	12749	AN2537
837	CONTIG43	49512	50016	2.06	4.61E-03	transcription_start_site	+	53383	53383	-3619	12750	AN2537
837	CONTIG43	49512	50016	2.06	4.61E-03	transcription_start_site	+	53729	53729	-3965	12751	AN2537
2616	CONTIG43	63535	63805	1.16	1.44E-01	transcription_start_site	-	62975	62975	-695	12759	AN2541
2616	CONTIG43	63535	63805	1.16	1.44E-01	transcription_start_site	-	62526	62526	-1144	12760	AN2541
2616	CONTIG43	63535	63805	1.16	1.44E-01	transcription_start_site	-	62276	62276	-1394	12761	AN2541
2616	CONTIG43	63535	63805	1.16	1.44E-01	transcription_start_site	+	66790	66790	-3120	12762	AN2542
2616	CONTIG43	63535	63805	1.16	1.44E-01	transcription_start_site	+	67312	67312	-3642	12763	AN2542
2616	CONTIG43	63535	63805	1.16	1.44E-01	transcription_start_site	+	67645	67645	-3975	12764	AN2542
655	CONTIG43	115519	115853	2.23	0.00E+00	transcription_start_site	-	114202	114202	-1484	12783	AN2549
655	CONTIG43	115519	115853	2.23	0.00E+00	transcription_start_site	-	113861	113861	-1825	12784	AN2549
655	CONTIG43	115519	115853	2.23	0.00E+00	transcription_start_site	-	113430	113430	-2256	12785	AN2549
655	CONTIG43	115519	115853	2.23	0.00E+00	transcription_start_site	-	113138	113138	-2548	12786	AN2549
655	CONTIG43	115519	115853	2.23	0.00E+00	transcription_start_site	-	112925	112925	-2761	12787	AN2549
655	CONTIG43	115519	115853	2.23	0.00E+00	transcription_start_site	-	112534	112534	-3152	12788	AN2549

655	CONTIG43	115519	115853	2.23	0.00E+00	transcription_start_site	+	117496	117496	-1810	12789 AN2550
655	CONTIG43	115519	115853	2.23	0.00E+00	transcription_start_site	+	117742	117742	-2056	12790 AN2550
655	CONTIG43	115519	115853	2.23	0.00E+00	transcription_start_site	+	117994	117994	-2308	12791 AN2550
655	CONTIG43	115519	115853	2.23	0.00E+00	transcription_start_site	+	118441	118441	-2755	12792 AN2550
2615	CONTIG43	146265	148109	1.16	1.28E-01	transcription_start_site	-	142269	142269	-4918	12809 AN2557
2615	CONTIG43	146265	148109	1.16	1.28E-01	transcription_start_site	+	146036	146036	1151	12813 AN2558
2615	CONTIG43	146265	148109	1.16	1.28E-01	transcription_start_site	+	145729	145729	1458	12812 AN2558
2615	CONTIG43	146265	148109	1.16	1.28E-01	transcription_start_site	+	145391	145391	1796	12811 AN2558
1577	CONTIG43	149190	149469	1.55	3.70E-02	transcription_start_site	-	149232	149232	-97	12815 AN2559
1577	CONTIG43	149190	149469	1.55	3.70E-02	transcription_start_site	-	149625	149625	295	12814 AN2559
1577	CONTIG43	149190	149469	1.55	3.70E-02	transcription_start_site	-	148730	148730	-599	12816 AN2559
2615	CONTIG43	146265	148109	1.16	1.28E-01	transcription_start_site	-	148730	148730	1543	12816 AN2559
1577	CONTIG43	149190	149469	1.55	3.70E-02	transcription_start_site	+	150227	150227	-897	12817 AN2560
1577	CONTIG43	149190	149469	1.55	3.70E-02	transcription_start_site	+	150404	150404	-1074	12818 AN2560
1577	CONTIG43	149190	149469	1.55	3.70E-02	transcription_start_site	+	151571	151571	-2241	12819 AN2560
1577	CONTIG43	149190	149469	1.55	3.70E-02	transcription_start_site	+	152043	152043	-2713	12820 AN2560
1577	CONTIG43	149190	149469	1.55	3.70E-02	transcription_start_site	+	152205	152205	-2875	12821 AN2560
2615	CONTIG43	146265	148109	1.16	1.28E-01	transcription_start_site	+	150227	150227	-3040	12817 AN2560
2615	CONTIG43	146265	148109	1.16	1.28E-01	transcription_start_site	+	150404	150404	-3217	12818 AN2560
2615	CONTIG43	146265	148109	1.16	1.28E-01	transcription_start_site	+	151571	151571	-4384	12819 AN2560
2615	CONTIG43	146265	148109	1.16	1.28E-01	transcription_start_site	+	152043	152043	-4856	12820 AN2560
2615	CONTIG43	146265	148109	1.16	1.28E-01	transcription_start_site	+	152205	152205	-5018	12821 AN2560
1577	CONTIG43	149190	149469	1.55	3.70E-02	transcription_start_site	+	152746	152746	-3416	12822 AN2561
1577	CONTIG43	149190	149469	1.55	3.70E-02	transcription_start_site	+	153179	153179	-3849	12823 AN2561
1577	CONTIG43	149190	149469	1.55	3.70E-02	transcription_start_site	+	153808	153808	-4478	12824 AN2561
2615	CONTIG43	146265	148109	1.16	1.28E-01	transcription_start_site	+	152746	152746	-5559	12822 AN2561
1577	CONTIG43	149190	149469	1.55	3.70E-02	transcription_start_site	+	154321	154321	-4991	12825 AN2562
1577	CONTIG43	149190	149469	1.55	3.70E-02	transcription_start_site	+	154433	154433	-5103	12826 AN2562
660	CONTIG43	171605	172029	2.23	1.80E-03	transcription_start_site	-	166658	166658	-5159	12839 AN2567
660	CONTIG43	171605	172029	2.23	1.80E-03	transcription_start_site	-	170575	170575	-1242	12844 AN2569
660	CONTIG43	171605	172029	2.23	1.80E-03	transcription_start_site	-	170320	170320	-1497	12845 AN2569
660	CONTIG43	171605	172029	2.23	1.80E-03	transcription_start_site	-	170115	170115	-1702	12846 AN2569
660	CONTIG43	171605	172029	2.23	1.80E-03	transcription_start_site	-	169935	169935	-1882	12847 AN2569
660	CONTIG43	171605	172029	2.23	1.80E-03	transcription_start_site	-	169780	169780	-2037	12848 AN2569
660	CONTIG43	171605	172029	2.23	1.80E-03	transcription_start_site	-	169500	169500	-2317	12849 AN2569
751	CONTIG43	174460	174739	2.15	3.03E-03	transcription_start_site	-	170575	170575	-4024	12844 AN2569
751	CONTIG43	174460	174739	2.15	3.03E-03	transcription_start_site	-	170320	170320	-4279	12845 AN2569
751	CONTIG43	174460	174739	2.15	3.03E-03	transcription_start_site	-	170115	170115	-4484	12846 AN2569
751	CONTIG43	174460	174739	2.15	3.03E-03	transcription_start_site	-	169935	169935	-4664	12847 AN2569
751	CONTIG43	174460	174739	2.15	3.03E-03	transcription_start_site	-	169780	169780	-4819	12848 AN2569
751	CONTIG43	174460	174739	2.15	3.03E-03	transcription_start_site	-	169500	169500	-5099	12849 AN2569
660	CONTIG43	171605	172029	2.23	1.80E-03	transcription_start_site	+	172014	172014	-197	12850 AN2570
660	CONTIG43	171605	172029	2.23	1.80E-03	transcription_start_site	+	172698	172698	-881	12851 AN2570
660	CONTIG43	171605	172029	2.23	1.80E-03	transcription_start_site	+	174961	174961	-3144	12852 AN2571
751	CONTIG43	174460	174739	2.15	3.03E-03	transcription_start_site	+	174961	174961	-361	12852 AN2571
751	CONTIG43	174460	174739	2.15	3.03E-03	transcription_start_site	+	178374	178374	-3774	12853 AN2572
751	CONTIG43	174460	174739	2.15	3.03E-03	transcription_start_site	+	178508	178508	-3908	12854 AN2572
751	CONTIG43	174460	174739	2.15	3.03E-03	transcription_start_site	+	178643	178643	-4043	12855 AN2572
751	CONTIG43	174460	174739	2.15	3.03E-03	transcription_start_site	+	178925	178925	-4325	12856 AN2572
2004	CONTIG43	193426	193705	1.37	6.84E-02	transcription_start_site	+	192462	192462	1103	12866 AN2574
2004	CONTIG43	193426	193705	1.37	6.84E-02	transcription_start_site	+	194467	194467	-901	12867 AN2575
2004	CONTIG43	193426	193705	1.37	6.84E-02	transcription_start_site	+	194804	194804	-1238	12868 AN2575
2004	CONTIG43	193426	193705	1.37	6.84E-02	transcription_start_site	+	194891	194891	-1325	12869 AN2575
2004	CONTIG43	193426	193705	1.37	6.84E-02	transcription_start_site	+	195048	195048	-1482	12870 AN2575
2004	CONTIG43	193426	193705	1.37	6.84E-02	transcription_start_site	+	195688	195688	-2122	12871 AN2575
2004	CONTIG43	193426	193705	1.37	6.84E-02	transcription_start_site	+	196160	196160	-2594	12872 AN2575
2004	CONTIG43	193426	193705	1.37	6.84E-02	transcription_start_site	+	196603	196603	-3037	12873 AN2576

2004	CONTIG43	193426	193705	1.37	6.84E-02	transcription_start_site	+	198128	198128	-4562	12874	AN2576
1871	CONTIG43	209795	211194	1.42	2.86E-02	transcription_start_site	+	213629	213629	-3134	12884	AN2580
1871	CONTIG43	209795	211194	1.42	2.86E-02	transcription_start_site	+	214096	214096	-3601	12885	AN2580
1871	CONTIG43	209795	211194	1.42	2.86E-02	transcription_start_site	+	214331	214331	-3836	12886	AN2580
1871	CONTIG43	209795	211194	1.42	2.86E-02	transcription_start_site	+	214923	214923	-4428	12887	AN2580
1871	CONTIG43	209795	211194	1.42	2.86E-02	transcription_start_site	+	215065	215065	-4570	12888	AN2580
2617	CONTIG43	208294	208853	1.16	1.44E-01	transcription_start_site	+	213629	213629	-5055	12884	AN2580
148	CONTIG43	217054	217418	3.05	0.00E+00	transcription_start_site	+	217545	217545	-309	12889	AN2581
148	CONTIG43	217054	217418	3.05	0.00E+00	transcription_start_site	+	217675	217675	-439	12890	AN2581
148	CONTIG43	217054	217418	3.05	0.00E+00	transcription_start_site	+	221329	221329	-4093	12891	AN2581
2218	CONTIG43	218419	218838	1.29	9.24E-02	transcription_start_site	+	217675	217675	953	12890	AN2581
2218	CONTIG43	218419	218838	1.29	9.24E-02	transcription_start_site	+	217545	217545	1083	12889	AN2581
2218	CONTIG43	218419	218838	1.29	9.24E-02	transcription_start_site	+	221329	221329	-2700	12891	AN2581
2797	CONTIG43	220439	220933	1.07	1.93E-01	transcription_start_site	+	221329	221329	-643	12891	AN2581
2219	CONTIG43	222464	223478	1.29	9.24E-02	transcription_start_site	-	223307	223307	336	12893	AN2582
2219	CONTIG43	222464	223478	1.29	9.24E-02	transcription_start_site	-	223601	223601	630	12892	AN2582
2618	CONTIG43	225229	225503	1.16	1.44E-01	transcription_start_site	-	223601	223601	-1765	12892	AN2582
2618	CONTIG43	225229	225503	1.16	1.44E-01	transcription_start_site	-	223307	223307	-2059	12893	AN2582
2798	CONTIG43	224114	224603	1.07	1.93E-01	transcription_start_site	-	223601	223601	-757	12892	AN2582
2798	CONTIG43	224114	224603	1.07	1.93E-01	transcription_start_site	-	223307	223307	-1051	12893	AN2582
2618	CONTIG43	225229	225503	1.16	1.44E-01	transcription_start_site	-	225508	225508	142	12895	AN2583
2618	CONTIG43	225229	225503	1.16	1.44E-01	transcription_start_site	-	225635	225635	269	12894	AN2583
2618	CONTIG43	225229	225503	1.16	1.44E-01	transcription_start_site	-	224882	224882	-484	12896	AN2583
2618	CONTIG43	225229	225503	1.16	1.44E-01	transcription_start_site	-	224689	224689	-677	12897	AN2583
2798	CONTIG43	224114	224603	1.07	1.93E-01	transcription_start_site	-	224689	224689	330	12897	AN2583
2798	CONTIG43	224114	224603	1.07	1.93E-01	transcription_start_site	-	224882	224882	523	12896	AN2583
2798	CONTIG43	224114	224603	1.07	1.93E-01	transcription_start_site	-	225508	225508	1149	12895	AN2583
1691	CONTIG43	232821	233155	1.5	4.36E-02	transcription_start_site	-	228332	228332	-4656	12898	AN2584
592	CONTIG43	233946	234200	2.32	1.22E-03	transcription_start_site	-	233431	233431	-642	12899	AN2585
592	CONTIG43	233946	234200	2.32	1.22E-03	transcription_start_site	-	232191	232191	-1882	12900	AN2585
592	CONTIG43	233946	234200	2.32	1.22E-03	transcription_start_site	-	232055	232055	-2018	12901	AN2585
1691	CONTIG43	232821	233155	1.5	4.36E-02	transcription_start_site	-	233431	233431	443	12899	AN2585
1691	CONTIG43	232821	233155	1.5	4.36E-02	transcription_start_site	-	232191	232191	-797	12900	AN2585
1691	CONTIG43	232821	233155	1.5	4.36E-02	transcription_start_site	-	232055	232055	-933	12901	AN2585
2619	CONTIG43	255167	255501	1.16	1.44E-01	transcription_start_site	+	255277	255277	57	12932	AN2593
2619	CONTIG43	255167	255501	1.16	1.44E-01	transcription_start_site	+	254779	254779	555	12931	AN2593
2619	CONTIG43	255167	255501	1.16	1.44E-01	transcription_start_site	+	254646	254646	688	12930	AN2593
2619	CONTIG43	255167	255501	1.16	1.44E-01	transcription_start_site	+	254462	254462	872	12929	AN2593
2619	CONTIG43	255167	255501	1.16	1.44E-01	transcription_start_site	+	254206	254206	1128	12928	AN2593
2619	CONTIG43	255167	255501	1.16	1.44E-01	transcription_start_site	-	256265	256265	931	12934	AN2594
2619	CONTIG43	255167	255501	1.16	1.44E-01	transcription_start_site	-	256490	256490	1156	12933	AN2594
39	CONTIG43	262216	262551	3.48	0.00E+00	transcription_start_site	-	258243	258243	-4140	12935	AN2595
39	CONTIG43	262216	262551	3.48	0.00E+00	transcription_start_site	-	257848	257848	-4535	12936	AN2595
309	CONTIG43	262667	263616	2.75	0.00E+00	transcription_start_site	-	258243	258243	-4898	12935	AN2595
309	CONTIG43	262667	263616	2.75	0.00E+00	transcription_start_site	-	257848	257848	-5293	12936	AN2595
39	CONTIG43	262216	262551	3.48	0.00E+00	transcription_start_site	-	262055	262055	-328	12937	AN2596
39	CONTIG43	262216	262551	3.48	0.00E+00	transcription_start_site	-	261675	261675	-708	12938	AN2596
39	CONTIG43	262216	262551	3.48	0.00E+00	transcription_start_site	-	261279	261279	-1104	12939	AN2596
39	CONTIG43	262216	262551	3.48	0.00E+00	transcription_start_site	-	261126	261126	-1257	12940	AN2596
39	CONTIG43	262216	262551	3.48	0.00E+00	transcription_start_site	-	260929	260929	-1454	12941	AN2596
39	CONTIG43	262216	262551	3.48	0.00E+00	transcription_start_site	-	260548	260548	-1835	12942	AN2596
39	CONTIG43	262216	262551	3.48	0.00E+00	transcription_start_site	-	260167	260167	-2216	12943	AN2596
309	CONTIG43	262667	263616	2.75	0.00E+00	transcription_start_site	-	262055	262055	-1086	12937	AN2596
309	CONTIG43	262667	263616	2.75	0.00E+00	transcription_start_site	-	261675	261675	-1466	12938	AN2596
309	CONTIG43	262667	263616	2.75	0.00E+00	transcription_start_site	-	261279	261279	-1862	12939	AN2596
309	CONTIG43	262667	263616	2.75	0.00E+00	transcription_start_site	-	261126	261126	-2015	12940	AN2596
309	CONTIG43	262667	263616	2.75	0.00E+00	transcription_start_site	-	260929	260929	-2212	12941	AN2596

309	CONTIG43	262667	263616	2.75	0.00E+00	transcription_start_site	-	260548	260548	-2593	12942	AN2596
309	CONTIG43	262667	263616	2.75	0.00E+00	transcription_start_site	-	260167	260167	-2974	12943	AN2596
39	CONTIG43	262216	262551	3.48	0.00E+00	transcription_start_site	+	263523	263523	-1139	12944	AN2597
39	CONTIG43	262216	262551	3.48	0.00E+00	transcription_start_site	+	263678	263678	-1294	12945	AN2597
309	CONTIG43	262667	263616	2.75	0.00E+00	transcription_start_site	+	263523	263523	-381	12944	AN2597
309	CONTIG43	262667	263616	2.75	0.00E+00	transcription_start_site	+	263678	263678	-536	12945	AN2597
2713	CONTIG43	281255	281769	1.12	1.66E-01	transcription_start_site	+	281090	281090	422	12973	AN2602
2713	CONTIG43	281255	281769	1.12	1.66E-01	transcription_start_site	+	280970	280970	542	12972	AN2602
2713	CONTIG43	281255	281769	1.12	1.66E-01	transcription_start_site	+	280421	280421	1091	12971	AN2602
2713	CONTIG43	281255	281769	1.12	1.66E-01	transcription_start_site	+	281777	281777	-265	12974	AN2603
2713	CONTIG43	281255	281769	1.12	1.66E-01	transcription_start_site	+	281999	281999	-487	12975	AN2603
2713	CONTIG43	281255	281769	1.12	1.66E-01	transcription_start_site	+	282971	282971	-1459	12976	AN2603
2713	CONTIG43	281255	281769	1.12	1.66E-01	transcription_start_site	+	283124	283124	-1612	12977	AN2603
2713	CONTIG43	281255	281769	1.12	1.66E-01	transcription_start_site	+	283308	283308	-1796	12978	AN2603
2713	CONTIG43	281255	281769	1.12	1.66E-01	transcription_start_site	+	283671	283671	-2159	12979	AN2603
2514	CONTIG43	310145	310709	1.2	1.25E-01	transcription_start_site	-	305543	305543	-4884	12995	AN2609
2514	CONTIG43	310145	310709	1.2	1.25E-01	transcription_start_site	-	305285	305285	-5142	12996	AN2609
2108	CONTIG43	312010	312519	1.33	8.01E-02	transcription_start_site	-	309412	309412	-2852	12999	AN2610
2108	CONTIG43	312010	312519	1.33	8.01E-02	transcription_start_site	-	309014	309014	-3250	13000	AN2610
2108	CONTIG43	312010	312519	1.33	8.01E-02	transcription_start_site	-	308707	308707	-3557	13001	AN2610
2108	CONTIG43	312010	312519	1.33	8.01E-02	transcription_start_site	-	308520	308520	-3744	13002	AN2610
2514	CONTIG43	310145	310709	1.2	1.25E-01	transcription_start_site	-	309412	309412	-1015	12999	AN2610
2514	CONTIG43	310145	310709	1.2	1.25E-01	transcription_start_site	-	309014	309014	-1413	13000	AN2610
2514	CONTIG43	310145	310709	1.2	1.25E-01	transcription_start_site	-	308707	308707	-1720	13001	AN2610
2514	CONTIG43	310145	310709	1.2	1.25E-01	transcription_start_site	-	308520	308520	-1907	13002	AN2610
1197	CONTIG43	316956	317253	1.76	1.57E-02	transcription_start_site	-	315713	315713	-1391	13003	AN2611
1197	CONTIG43	316956	317253	1.76	1.57E-02	transcription_start_site	-	315342	315342	-1762	13004	AN2611
1197	CONTIG43	316956	317253	1.76	1.57E-02	transcription_start_site	-	314977	314977	-2127	13005	AN2611
1197	CONTIG43	316956	317253	1.76	1.57E-02	transcription_start_site	-	314202	314202	-2902	13006	AN2611
1197	CONTIG43	316956	317253	1.76	1.57E-02	transcription_start_site	-	314031	314031	-3073	13007	AN2611
1197	CONTIG43	316956	317253	1.76	1.57E-02	transcription_start_site	-	313083	313083	-4021	13008	AN2611
1197	CONTIG43	316956	317253	1.76	1.57E-02	transcription_start_site	-	312933	312933	-4171	13009	AN2611
1198	CONTIG43	318993	319787	1.76	1.57E-02	transcription_start_site	-	315713	315713	-3677	13003	AN2611
1198	CONTIG43	318993	319787	1.76	1.57E-02	transcription_start_site	-	315342	315342	-4048	13004	AN2611
1198	CONTIG43	318993	319787	1.76	1.57E-02	transcription_start_site	-	314977	314977	-4413	13005	AN2611
1198	CONTIG43	318993	319787	1.76	1.57E-02	transcription_start_site	-	314202	314202	-5188	13006	AN2611
1198	CONTIG43	318993	319787	1.76	1.57E-02	transcription_start_site	-	314031	314031	-5359	13007	AN2611
2108	CONTIG43	312010	312519	1.33	8.01E-02	transcription_start_site	-	312933	312933	668	13009	AN2611
2108	CONTIG43	312010	312519	1.33	8.01E-02	transcription_start_site	-	313083	313083	818	13008	AN2611
1198	CONTIG43	318993	319787	1.76	1.57E-02	transcription_start_site	-	320118	320118	728	13012	AN2612
1198	CONTIG43	318993	319787	1.76	1.57E-02	transcription_start_site	-	318622	318622	-768	13013	AN2612
1198	CONTIG43	318993	319787	1.76	1.57E-02	transcription_start_site	-	320784	320784	1394	13011	AN2612
1692	CONTIG43	324603	325028	1.5	4.36E-02	transcription_start_site	-	320931	320931	-3884	13010	AN2612
1692	CONTIG43	324603	325028	1.5	4.36E-02	transcription_start_site	-	320784	320784	-4031	13011	AN2612
1692	CONTIG43	324603	325028	1.5	4.36E-02	transcription_start_site	-	320118	320118	-4697	13012	AN2612
1197	CONTIG43	316956	317253	1.76	1.57E-02	transcription_start_site	+	321553	321553	-4448	13014	AN2613
1197	CONTIG43	316956	317253	1.76	1.57E-02	transcription_start_site	+	321896	321896	-4791	13015	AN2613
1198	CONTIG43	318993	319787	1.76	1.57E-02	transcription_start_site	+	321553	321553	-2163	13014	AN2613
1198	CONTIG43	318993	319787	1.76	1.57E-02	transcription_start_site	+	321896	321896	-2506	13015	AN2613
1198	CONTIG43	318993	319787	1.76	1.57E-02	transcription_start_site	+	322457	322457	-3067	13016	AN2613
1692	CONTIG43	324603	325028	1.5	4.36E-02	transcription_start_site	+	325250	325250	-434	13017	AN2614
1692	CONTIG43	324603	325028	1.5	4.36E-02	transcription_start_site	+	325356	325356	-540	13018	AN2614
1692	CONTIG43	324603	325028	1.5	4.36E-02	transcription_start_site	+	325526	325526	-710	13019	AN2614
1692	CONTIG43	324603	325028	1.5	4.36E-02	transcription_start_site	+	325935	325935	-1119	13020	AN2614
1692	CONTIG43	324603	325028	1.5	4.36E-02	transcription_start_site	+	326128	326128	-1312	13021	AN2614
1688	CONTIG43	329570	330214	1.5	2.64E-02	transcription_start_site	+	330048	330048	-156	13023	AN2615
1688	CONTIG43	329570	330214	1.5	2.64E-02	transcription_start_site	+	329674	329674	218	13022	AN2615

1692	CONTIG43	324603	325028	1.5	4.36E-02	transcription_start_site	+	329674	329674	-4858	13022	AN2615
3036	CONTIG45	6171	6575	0.81	1.28E-01	transcription_start_site	+	6277	6277	96	13033	AN2619
3036	CONTIG45	6171	6575	0.81	1.28E-01	transcription_start_site	+	6902	6902	-529	13034	AN2619
3036	CONTIG45	6171	6575	0.81	1.28E-01	transcription_start_site	+	7246	7246	-873	13035	AN2619
2427	CONTIG45	29036	29310	1.22	1.57E-02	transcription_start_site	-	26703	26703	-2470	13050	AN2621
2427	CONTIG45	29036	29310	1.22	1.57E-02	transcription_start_site	+	29384	29384	-211	13052	AN2623
2427	CONTIG45	29036	29310	1.22	1.57E-02	transcription_start_site	+	29473	29473	-300	13053	AN2623
2427	CONTIG45	29036	29310	1.22	1.57E-02	transcription_start_site	+	29703	29703	-530	13054	AN2623
2427	CONTIG45	29036	29310	1.22	1.57E-02	transcription_start_site	+	29914	29914	-741	13055	AN2623
1150	CONTIG45	43293	43642	1.79	2.16E-04	transcription_start_site	-	42339	42339	-1128	13063	AN2626
1150	CONTIG45	43293	43642	1.79	2.16E-04	transcription_start_site	-	42115	42115	-1352	13064	AN2626
1150	CONTIG45	43293	43642	1.79	2.16E-04	transcription_start_site	-	41683	41683	-1784	13065	AN2626
2739	CONTIG45	41180	41614	1.1	3.08E-02	transcription_start_site	-	41683	41683	286	13065	AN2626
2739	CONTIG45	41180	41614	1.1	3.08E-02	transcription_start_site	-	42115	42115	718	13064	AN2626
2739	CONTIG45	41180	41614	1.1	3.08E-02	transcription_start_site	-	42339	42339	942	13063	AN2626
703	CONTIG46	8701	9050	2.19	2.32E-03	transcription_start_site	-	5274	5274	-3601	13092	AN2637
1727	CONTIG46	9696	9970	1.49	6.84E-02	transcription_start_site	-	5274	5274	-4559	13092	AN2637
703	CONTIG46	8701	9050	2.19	2.32E-03	transcription_start_site	-	8360	8360	-515	13093	AN2638
703	CONTIG46	8701	9050	2.19	2.32E-03	transcription_start_site	-	8022	8022	-853	13094	AN2638
1727	CONTIG46	9696	9970	1.49	6.84E-02	transcription_start_site	-	8360	8360	-1473	13093	AN2638
1727	CONTIG46	9696	9970	1.49	6.84E-02	transcription_start_site	-	8022	8022	-1811	13094	AN2638
703	CONTIG46	8701	9050	2.19	2.32E-03	transcription_start_site	+	9779	9779	-903	13095	AN2639
703	CONTIG46	8701	9050	2.19	2.32E-03	transcription_start_site	+	9876	9876	-1000	13096	AN2639
703	CONTIG46	8701	9050	2.19	2.32E-03	transcription_start_site	+	10545	10545	-1669	13097	AN2639
1727	CONTIG46	9696	9970	1.49	6.84E-02	transcription_start_site	+	9876	9876	-43	13096	AN2639
1727	CONTIG46	9696	9970	1.49	6.84E-02	transcription_start_site	+	9779	9779	54	13095	AN2639
1727	CONTIG46	9696	9970	1.49	6.84E-02	transcription_start_site	+	10545	10545	-712	13097	AN2639
703	CONTIG46	8701	9050	2.19	2.32E-03	transcription_start_site	+	11716	11716	-2840	13098	AN2640
1727	CONTIG46	9696	9970	1.49	6.84E-02	transcription_start_site	+	11716	11716	-1883	13098	AN2640
1254	CONTIG46	23942	24426	1.72	1.81E-02	transcription_start_site	-	19972	19972	-4212	13099	AN2642
1254	CONTIG46	23942	24426	1.72	1.81E-02	transcription_start_site	-	19814	19814	-4370	13100	AN2642
1254	CONTIG46	23942	24426	1.72	1.81E-02	transcription_start_site	-	19598	19598	-4586	13101	AN2642
1254	CONTIG46	23942	24426	1.72	1.81E-02	transcription_start_site	-	19332	19332	-4852	13102	AN2642
1254	CONTIG46	23942	24426	1.72	1.81E-02	transcription_start_site	-	19067	19067	-5117	13103	AN2642
1254	CONTIG46	23942	24426	1.72	1.81E-02	transcription_start_site	-	23240	23240	-944	13104	AN2643
1254	CONTIG46	23942	24426	1.72	1.81E-02	transcription_start_site	-	22963	22963	-1221	13105	AN2643
1254	CONTIG46	23942	24426	1.72	1.81E-02	transcription_start_site	-	22385	22385	-1799	13106	AN2643
1254	CONTIG46	23942	24426	1.72	1.81E-02	transcription_start_site	-	22154	22154	-2030	13107	AN2643
1254	CONTIG46	23942	24426	1.72	1.81E-02	transcription_start_site	-	21730	21730	-2454	13108	AN2643
1254	CONTIG46	23942	24426	1.72	1.81E-02	transcription_start_site	+	23759	23759	425	13109	AN2644
1254	CONTIG46	23942	24426	1.72	1.81E-02	transcription_start_site	+	25421	25421	-1237	13110	AN2644
1254	CONTIG46	23942	24426	1.72	1.81E-02	transcription_start_site	+	26758	26758	-2574	13111	AN2645
1254	CONTIG46	23942	24426	1.72	1.81E-02	transcription_start_site	+	26947	26947	-2763	13112	AN2645
1254	CONTIG46	23942	24426	1.72	1.81E-02	transcription_start_site	+	27382	27382	-3198	13113	AN2645
1254	CONTIG46	23942	24426	1.72	1.81E-02	transcription_start_site	+	27616	27616	-3432	13114	AN2645
129	CONTIG46	37145	37704	3.12	0.00E+00	transcription_start_site	-	37101	37101	-323	13130	AN2648
129	CONTIG46	37145	37704	3.12	0.00E+00	transcription_start_site	-	36785	36785	-639	13131	AN2648
129	CONTIG46	37145	37704	3.12	0.00E+00	transcription_start_site	-	36530	36530	-894	13132	AN2648
129	CONTIG46	37145	37704	3.12	0.00E+00	transcription_start_site	+	38445	38445	-1020	13133	AN2649
129	CONTIG46	37145	37704	3.12	0.00E+00	transcription_start_site	+	38652	38652	-1227	13134	AN2649
129	CONTIG46	37145	37704	3.12	0.00E+00	transcription_start_site	+	39101	39101	-1676	13135	AN2649
129	CONTIG46	37145	37704	3.12	0.00E+00	transcription_start_site	+	39889	39889	-2464	13136	AN2649
129	CONTIG46	37145	37704	3.12	0.00E+00	transcription_start_site	+	40077	40077	-2652	13137	AN2649
2062	CONTIG46	63003	64027	1.35	9.26E-02	transcription_start_site	-	59557	59557	-3958	13169	AN2657
2062	CONTIG46	63003	64027	1.35	9.26E-02	transcription_start_site	-	58604	58604	-4911	13170	AN2657
2198	CONTIG46	59643	60592	1.3	1.09E-01	transcription_start_site	-	59557	59557	-560	13169	AN2657
2198	CONTIG46	59643	60592	1.3	1.09E-01	transcription_start_site	-	58604	58604	-1513	13170	AN2657

2627	CONTIG46	61203	61482	1.16	1.93E-01	transcription_start_site	-	59557	59557	-1785	13169	AN2657
2627	CONTIG46	61203	61482	1.16	1.93E-01	transcription_start_site	-	58604	58604	-2738	13170	AN2657
2062	CONTIG46	63003	64027	1.35	9.26E-02	transcription_start_site	-	62290	62290	-1225	13171	AN2658
2062	CONTIG46	63003	64027	1.35	9.26E-02	transcription_start_site	-	62042	62042	-1473	13172	AN2658
2062	CONTIG46	63003	64027	1.35	9.26E-02	transcription_start_site	-	61778	61778	-1737	13173	AN2658
2062	CONTIG46	63003	64027	1.35	9.26E-02	transcription_start_site	-	61182	61182	-2333	13174	AN2658
2062	CONTIG46	63003	64027	1.35	9.26E-02	transcription_start_site	-	60715	60715	-2800	13175	AN2658
2062	CONTIG46	63003	64027	1.35	9.26E-02	transcription_start_site	-	60487	60487	-3028	13176	AN2658
2062	CONTIG46	63003	64027	1.35	9.26E-02	transcription_start_site	-	60178	60178	-3337	13177	AN2658
2198	CONTIG46	59643	60592	1.3	1.09E-01	transcription_start_site	-	60178	60178	60	13177	AN2658
2198	CONTIG46	59643	60592	1.3	1.09E-01	transcription_start_site	-	60487	60487	369	13176	AN2658
2198	CONTIG46	59643	60592	1.3	1.09E-01	transcription_start_site	-	60715	60715	597	13175	AN2658
2198	CONTIG46	59643	60592	1.3	1.09E-01	transcription_start_site	-	61182	61182	1064	13174	AN2658
2627	CONTIG46	61203	61482	1.16	1.93E-01	transcription_start_site	-	61182	61182	-160	13174	AN2658
2627	CONTIG46	61203	61482	1.16	1.93E-01	transcription_start_site	-	61778	61778	435	13173	AN2658
2627	CONTIG46	61203	61482	1.16	1.93E-01	transcription_start_site	-	60715	60715	-627	13175	AN2658
2627	CONTIG46	61203	61482	1.16	1.93E-01	transcription_start_site	-	62042	62042	699	13172	AN2658
2627	CONTIG46	61203	61482	1.16	1.93E-01	transcription_start_site	-	60487	60487	-855	13176	AN2658
2627	CONTIG46	61203	61482	1.16	1.93E-01	transcription_start_site	-	62290	62290	947	13171	AN2658
2627	CONTIG46	61203	61482	1.16	1.93E-01	transcription_start_site	-	60178	60178	-1164	13177	AN2658
2062	CONTIG46	63003	64027	1.35	9.26E-02	transcription_start_site	+	63543	63543	-28	13178	AN2659
2062	CONTIG46	63003	64027	1.35	9.26E-02	transcription_start_site	+	63940	63940	-425	13179	AN2659
2062	CONTIG46	63003	64027	1.35	9.26E-02	transcription_start_site	+	64908	64908	-1393	13180	AN2659
2062	CONTIG46	63003	64027	1.35	9.26E-02	transcription_start_site	+	65020	65020	-1505	13181	AN2659
2198	CONTIG46	59643	60592	1.3	1.09E-01	transcription_start_site	+	63543	63543	-3425	13178	AN2659
2198	CONTIG46	59643	60592	1.3	1.09E-01	transcription_start_site	+	63940	63940	-3822	13179	AN2659
2198	CONTIG46	59643	60592	1.3	1.09E-01	transcription_start_site	+	64908	64908	-4790	13180	AN2659
2198	CONTIG46	59643	60592	1.3	1.09E-01	transcription_start_site	+	65020	65020	-4902	13181	AN2659
2627	CONTIG46	61203	61482	1.16	1.93E-01	transcription_start_site	+	63543	63543	-2200	13178	AN2659
2627	CONTIG46	61203	61482	1.16	1.93E-01	transcription_start_site	+	63940	63940	-2597	13179	AN2659
2627	CONTIG46	61203	61482	1.16	1.93E-01	transcription_start_site	+	64908	64908	-3565	13180	AN2659
2627	CONTIG46	61203	61482	1.16	1.93E-01	transcription_start_site	+	65020	65020	-3677	13181	AN2659
1623	CONTIG46	93534	93909	1.53	5.88E-02	transcription_start_site	-	90495	90495	-3226	13214	AN2667
1623	CONTIG46	93534	93909	1.53	5.88E-02	transcription_start_site	-	90278	90278	-3443	13215	AN2667
1623	CONTIG46	93534	93909	1.53	5.88E-02	transcription_start_site	-	92496	92496	-1225	13217	AN2668
1623	CONTIG46	93534	93909	1.53	5.88E-02	transcription_start_site	-	92311	92311	-1410	13218	AN2668
1623	CONTIG46	93534	93909	1.53	5.88E-02	transcription_start_site	-	92109	92109	-1612	13219	AN2668
1623	CONTIG46	93534	93909	1.53	5.88E-02	transcription_start_site	-	91983	91983	-1738	13220	AN2668
1623	CONTIG46	93534	93909	1.53	5.88E-02	transcription_start_site	-	91524	91524	-2197	13221	AN2668
1623	CONTIG46	93534	93909	1.53	5.88E-02	transcription_start_site	-	91295	91295	-2426	13222	AN2668
1623	CONTIG46	93534	93909	1.53	5.88E-02	transcription_start_site	+	95591	95591	-1869	13223	AN2669
2605	CONTIG47	18545	18884	1.16	8.01E-02	transcription_start_site	-	14958	14958	-3756	13235	AN2673
2605	CONTIG47	18545	18884	1.16	8.01E-02	transcription_start_site	-	14401	14401	-4313	13236	AN2673
2605	CONTIG47	18545	18884	1.16	8.01E-02	transcription_start_site	-	13684	13684	-5030	13237	AN2673
2605	CONTIG47	18545	18884	1.16	8.01E-02	transcription_start_site	-	13580	13580	-5134	13238	AN2673
619	CONTIG47	20630	20909	2.28	2.39E-04	transcription_start_site	-	18605	18605	-2164	13240	AN2674
619	CONTIG47	20630	20909	2.28	2.39E-04	transcription_start_site	-	18396	18396	-2373	13241	AN2674
619	CONTIG47	20630	20909	2.28	2.39E-04	transcription_start_site	-	18152	18152	-2617	13242	AN2674
619	CONTIG47	20630	20909	2.28	2.39E-04	transcription_start_site	-	17620	17620	-3149	13243	AN2674
619	CONTIG47	20630	20909	2.28	2.39E-04	transcription_start_site	-	17106	17106	-3663	13244	AN2674
2605	CONTIG47	18545	18884	1.16	8.01E-02	transcription_start_site	-	18605	18605	-109	13240	AN2674
2605	CONTIG47	18545	18884	1.16	8.01E-02	transcription_start_site	-	18396	18396	-318	13241	AN2674
2605	CONTIG47	18545	18884	1.16	8.01E-02	transcription_start_site	-	18152	18152	-562	13242	AN2674
2605	CONTIG47	18545	18884	1.16	8.01E-02	transcription_start_site	-	17620	17620	-1094	13243	AN2674
2605	CONTIG47	18545	18884	1.16	8.01E-02	transcription_start_site	-	17106	17106	-1608	13244	AN2674
619	CONTIG47	20630	20909	2.28	2.39E-04	transcription_start_site	-	20575	20575	-194	13245	AN2675
619	CONTIG47	20630	20909	2.28	2.39E-04	transcription_start_site	-	20327	20327	-442	13246	AN2675

619	CONTIG47	20630	20909	2.28	2.39E-04	transcription_start_site	-	19786	19786	-983	13247 AN2675
2605	CONTIG47	18545	18884	1.16	8.01E-02	transcription_start_site	-	19786	19786	1071	13247 AN2675
2503	CONTIG47	32480	32914	1.2	6.84E-02	transcription_start_site	-	30548	30548	-2149	13250 AN2677
2503	CONTIG47	32480	32914	1.2	6.84E-02	transcription_start_site	-	30381	30381	-2316	13251 AN2677
2503	CONTIG47	32480	32914	1.2	6.84E-02	transcription_start_site	-	29872	29872	-2825	13252 AN2677
2503	CONTIG47	32480	32914	1.2	6.84E-02	transcription_start_site	-	29053	29053	-3644	13253 AN2677
2503	CONTIG47	32480	32914	1.2	6.84E-02	transcription_start_site	-	28801	28801	-3896	13254 AN2677
2503	CONTIG47	32480	32914	1.2	6.84E-02	transcription_start_site	-	32356	32356	-341	13255 AN2678
2503	CONTIG47	32480	32914	1.2	6.84E-02	transcription_start_site	+	32961	32961	-264	13256 AN2679
2503	CONTIG47	32480	32914	1.2	6.84E-02	transcription_start_site	+	33275	33275	-578	13257 AN2679
2503	CONTIG47	32480	32914	1.2	6.84E-02	transcription_start_site	+	33777	33777	-1080	13258 AN2679
2503	CONTIG47	32480	32914	1.2	6.84E-02	transcription_start_site	+	33948	33948	-1251	13259 AN2679
2503	CONTIG47	32480	32914	1.2	6.84E-02	transcription_start_site	+	36453	36453	-3756	13260 AN2680
2503	CONTIG47	32480	32914	1.2	6.84E-02	transcription_start_site	+	36686	36686	-3989	13261 AN2680
2503	CONTIG47	32480	32914	1.2	6.84E-02	transcription_start_site	+	36820	36820	-4123	13262 AN2680
2503	CONTIG47	32480	32914	1.2	6.84E-02	transcription_start_site	+	37234	37234	-4537	13263 AN2680
2824	CONTIG47	42385	43559	1.05	1.07E-01	transcription_start_site	-	40241	40241	-2731	13264 AN2681
2824	CONTIG47	42385	43559	1.05	1.07E-01	transcription_start_site	-	40192	40192	-2780	13265 AN2681
2824	CONTIG47	42385	43559	1.05	1.07E-01	transcription_start_site	-	40018	40018	-2954	13266 AN2681
2824	CONTIG47	42385	43559	1.05	1.07E-01	transcription_start_site	-	39691	39691	-3281	13267 AN2681
2824	CONTIG47	42385	43559	1.05	1.07E-01	transcription_start_site	-	39346	39346	-3626	13268 AN2681
2824	CONTIG47	42385	43559	1.05	1.07E-01	transcription_start_site	-	38849	38849	-4123	13269 AN2681
2824	CONTIG47	42385	43559	1.05	1.07E-01	transcription_start_site	-	38536	38536	-4436	13270 AN2681
2824	CONTIG47	42385	43559	1.05	1.07E-01	transcription_start_site	-	42921	42921	-51	13272 AN2682
2824	CONTIG47	42385	43559	1.05	1.07E-01	transcription_start_site	-	43224	43224	252	13271 AN2682
2824	CONTIG47	42385	43559	1.05	1.07E-01	transcription_start_site	-	42224	42224	-748	13273 AN2682
2824	CONTIG47	42385	43559	1.05	1.07E-01	transcription_start_site	+	46468	46468	-3496	13274 AN2683
2824	CONTIG47	42385	43559	1.05	1.07E-01	transcription_start_site	+	46599	46599	-3627	13275 AN2683
2824	CONTIG47	42385	43559	1.05	1.07E-01	transcription_start_site	+	46749	46749	-3777	13276 AN2683
2824	CONTIG47	42385	43559	1.05	1.07E-01	transcription_start_site	+	46852	46852	-3880	13277 AN2683
2824	CONTIG47	42385	43559	1.05	1.07E-01	transcription_start_site	+	47496	47496	-4524	13278 AN2683
2892	CONTIG47	50345	50614	1.01	1.44E-01	transcription_start_site	-	50350	50350	-129	13279 AN2684
2892	CONTIG47	50345	50614	1.01	1.44E-01	transcription_start_site	-	50282	50282	-197	13280 AN2684
2892	CONTIG47	50345	50614	1.01	1.44E-01	transcription_start_site	-	49393	49393	-1086	13281 AN2684
2892	CONTIG47	50345	50614	1.01	1.44E-01	transcription_start_site	-	48989	48989	-1490	13282 AN2684
2892	CONTIG47	50345	50614	1.01	1.44E-01	transcription_start_site	+	53171	53171	-2691	13283 AN2685
2892	CONTIG47	50345	50614	1.01	1.44E-01	transcription_start_site	+	55081	55081	-4601	13284 AN2686
2892	CONTIG47	50345	50614	1.01	1.44E-01	transcription_start_site	+	55232	55232	-4752	13285 AN2686
2892	CONTIG47	50345	50614	1.01	1.44E-01	transcription_start_site	+	55379	55379	-4899	13286 AN2686
2498	CONTIG47	70353	71152	1.2	4.42E-02	transcription_start_site	-	66447	66447	-4305	13297 AN2690
395	CONTIG47	72078	72592	2.61	0.00E+00	transcription_start_site	+	72814	72814	-479	13300 AN2692
395	CONTIG47	72078	72592	2.61	0.00E+00	transcription_start_site	+	73608	73608	-1273	13301 AN2692
2498	CONTIG47	70353	71152	1.2	4.42E-02	transcription_start_site	+	72814	72814	-2061	13300 AN2692
2498	CONTIG47	70353	71152	1.2	4.42E-02	transcription_start_site	+	73608	73608	-2855	13301 AN2692
2701	CONTIG47	76593	76927	1.12	7.51E-02	transcription_start_site	-	76585	76585	-175	13304 AN2693
2701	CONTIG47	76593	76927	1.12	7.51E-02	transcription_start_site	-	77151	77151	391	13303 AN2693
2701	CONTIG47	76593	76927	1.12	7.51E-02	transcription_start_site	-	77269	77269	509	13302 AN2693
2701	CONTIG47	76593	76927	1.12	7.51E-02	transcription_start_site	+	77786	77786	-1026	13305 AN2694
2701	CONTIG47	76593	76927	1.12	7.51E-02	transcription_start_site	+	77883	77883	-1123	13306 AN2694
2701	CONTIG47	76593	76927	1.12	7.51E-02	transcription_start_site	+	78252	78252	-1492	13307 AN2694
2701	CONTIG47	76593	76927	1.12	7.51E-02	transcription_start_site	+	78613	78613	-1853	13308 AN2694
2701	CONTIG47	76593	76927	1.12	7.51E-02	transcription_start_site	+	79326	79326	-2566	13309 AN2695
2701	CONTIG47	76593	76927	1.12	7.51E-02	transcription_start_site	+	79522	79522	-2762	13310 AN2695
1973	CONTIG47	85814	86308	1.38	3.08E-02	transcription_start_site	-	82686	82686	-3375	13311 AN2696
1973	CONTIG47	85814	86308	1.38	3.08E-02	transcription_start_site	-	82523	82523	-3538	13312 AN2696
1973	CONTIG47	85814	86308	1.38	3.08E-02	transcription_start_site	-	82283	82283	-3778	13313 AN2696
1973	CONTIG47	85814	86308	1.38	3.08E-02	transcription_start_site	-	81991	81991	-4070	13314 AN2696

1973	CONTIG47	85814	86308	1.38	3.08E-02	transcription_start_site	-	81515	81515	-4546	13315	AN2696
1973	CONTIG47	85814	86308	1.38	3.08E-02	transcription_start_site	-	81271	81271	-4790	13316	AN2696
2952	CONTIG47	85144	85703	0.97	1.50E-01	transcription_start_site	-	82686	82686	-2737	13311	AN2696
2952	CONTIG47	85144	85703	0.97	1.50E-01	transcription_start_site	-	82523	82523	-2900	13312	AN2696
2952	CONTIG47	85144	85703	0.97	1.50E-01	transcription_start_site	-	82283	82283	-3140	13313	AN2696
2952	CONTIG47	85144	85703	0.97	1.50E-01	transcription_start_site	-	81991	81991	-3432	13314	AN2696
2952	CONTIG47	85144	85703	0.97	1.50E-01	transcription_start_site	-	81515	81515	-3908	13315	AN2696
2952	CONTIG47	85144	85703	0.97	1.50E-01	transcription_start_site	-	81271	81271	-4152	13316	AN2696
1973	CONTIG47	85814	86308	1.38	3.08E-02	transcription_start_site	-	86538	86538	477	13317	AN2697
1973	CONTIG47	85814	86308	1.38	3.08E-02	transcription_start_site	-	85186	85186	-875	13318	AN2697
2952	CONTIG47	85144	85703	0.97	1.50E-01	transcription_start_site	-	85186	85186	-237	13318	AN2697
2952	CONTIG47	85144	85703	0.97	1.50E-01	transcription_start_site	-	86538	86538	1114	13317	AN2697
1973	CONTIG47	85814	86308	1.38	3.08E-02	transcription_start_site	+	89280	89280	-3219	13320	AN2699
2952	CONTIG47	85144	85703	0.97	1.50E-01	transcription_start_site	+	89280	89280	-3856	13320	AN2699
1377	CONTIG47	93919	94403	1.64	4.17E-03	transcription_start_site	-	92212	92212	-1949	13321	AN2700
2893	CONTIG47	104269	104608	1.01	1.44E-01	transcription_start_site	+	103831	103831	607	13327	AN2702
2157	CONTIG47	105769	106028	1.31	4.36E-02	transcription_start_site	-	105840	105840	-58	13328	AN2703
2157	CONTIG47	105769	106028	1.31	4.36E-02	transcription_start_site	+	106683	106683	-784	13329	AN2704
2157	CONTIG47	105769	106028	1.31	4.36E-02	transcription_start_site	+	108660	108660	-2761	13330	AN2704
2893	CONTIG47	104269	104608	1.01	1.44E-01	transcription_start_site	+	106683	106683	-2244	13329	AN2704
2893	CONTIG47	104269	104608	1.01	1.44E-01	transcription_start_site	+	108660	108660	-4221	13330	AN2704
1171	CONTIG48	23703	23992	1.78	1.11E-02	transcription_start_site	+	24149	24149	-301	13389	AN2720
1171	CONTIG48	23703	23992	1.78	1.11E-02	transcription_start_site	+	24559	24559	-711	13390	AN2720
1171	CONTIG48	23703	23992	1.78	1.11E-02	transcription_start_site	+	24968	24968	-1120	13391	AN2720
1171	CONTIG48	23703	23992	1.78	1.11E-02	transcription_start_site	+	28945	28945	-5097	13396	AN2722
96	CONTIG48	65041	65310	3.23	0.00E+00	transcription_start_site	-	64232	64232	-943	13426	AN2730
96	CONTIG48	65041	65310	3.23	0.00E+00	transcription_start_site	-	63751	63751	-1424	13427	AN2730
2107	CONTIG48	73654	73939	1.33	6.84E-02	transcription_start_site	-	73350	73350	-446	13428	AN2731
2107	CONTIG48	73654	73939	1.33	6.84E-02	transcription_start_site	-	73251	73251	-545	13429	AN2731
2107	CONTIG48	73654	73939	1.33	6.84E-02	transcription_start_site	-	73119	73119	-677	13430	AN2731
2107	CONTIG48	73654	73939	1.33	6.84E-02	transcription_start_site	-	72232	72232	-1564	13431	AN2731
2511	CONTIG48	88959	89843	1.2	1.08E-01	transcription_start_site	-	85124	85124	-4277	13444	AN2736
2511	CONTIG48	88959	89843	1.2	1.08E-01	transcription_start_site	-	85010	85010	-4391	13445	AN2736
2511	CONTIG48	88959	89843	1.2	1.08E-01	transcription_start_site	-	87878	87878	-1523	13448	AN2737
2511	CONTIG48	88959	89843	1.2	1.08E-01	transcription_start_site	-	87225	87225	-2176	13449	AN2737
2511	CONTIG48	88959	89843	1.2	1.08E-01	transcription_start_site	-	85653	85653	-3748	13450	AN2737
2511	CONTIG48	88959	89843	1.2	1.08E-01	transcription_start_site	+	89179	89179	222	13452	AN2738
2511	CONTIG48	88959	89843	1.2	1.08E-01	transcription_start_site	+	88746	88746	655	13451	AN2738
649	CONTIG48	94204	94478	2.24	1.22E-03	transcription_start_site	-	93855	93855	-486	13453	AN2739
649	CONTIG48	94204	94478	2.24	1.22E-03	transcription_start_site	-	93028	93028	-1313	13454	AN2739
649	CONTIG48	94204	94478	2.24	1.22E-03	transcription_start_site	-	92806	92806	-1535	13455	AN2739
649	CONTIG48	94204	94478	2.24	1.22E-03	transcription_start_site	-	92265	92265	-2076	13456	AN2739
649	CONTIG48	94204	94478	2.24	1.22E-03	transcription_start_site	-	92047	92047	-2294	13457	AN2739
649	CONTIG48	94204	94478	2.24	1.22E-03	transcription_start_site	-	91839	91839	-2502	13458	AN2739
649	CONTIG48	94204	94478	2.24	1.22E-03	transcription_start_site	-	91342	91342	-2999	13459	AN2739
649	CONTIG48	94204	94478	2.24	1.22E-03	transcription_start_site	-	91016	91016	-3325	13460	AN2739
649	CONTIG48	94204	94478	2.24	1.22E-03	transcription_start_site	-	90870	90870	-3471	13461	AN2739
649	CONTIG48	94204	94478	2.24	1.22E-03	transcription_start_site	-	90694	90694	-3647	13462	AN2739
2511	CONTIG48	88959	89843	1.2	1.08E-01	transcription_start_site	-	90694	90694	1293	13462	AN2739
529	CONTIG49	4277	5151	2.4	0.00E+00	transcription_start_site	+	6592	6592	-1878	13463	AN2740
529	CONTIG49	4277	5151	2.4	0.00E+00	transcription_start_site	+	6809	6809	-2095	13464	AN2740
529	CONTIG49	4277	5151	2.4	0.00E+00	transcription_start_site	+	7447	7447	-2733	13465	AN2740
529	CONTIG49	4277	5151	2.4	0.00E+00	transcription_start_site	+	7563	7563	-2849	13466	AN2740
815	CONTIG49	3016	3595	2.08	0.00E+00	transcription_start_site	+	6592	6592	-3286	13463	AN2740
815	CONTIG49	3016	3595	2.08	0.00E+00	transcription_start_site	+	6809	6809	-3503	13464	AN2740
815	CONTIG49	3016	3595	2.08	0.00E+00	transcription_start_site	+	7447	7447	-4141	13465	AN2740
815	CONTIG49	3016	3595	2.08	0.00E+00	transcription_start_site	+	7563	7563	-4257	13466	AN2740

1037	CONTIG49	5329	6058	1.88	5.20E-03	transcription_start_site	+	6592	6592	-898	13463 AN2740
1037	CONTIG49	5329	6058	1.88	5.20E-03	transcription_start_site	+	6809	6809	-1115	13464 AN2740
1037	CONTIG49	5329	6058	1.88	5.20E-03	transcription_start_site	+	7447	7447	-1753	13465 AN2740
1037	CONTIG49	5329	6058	1.88	5.20E-03	transcription_start_site	+	7563	7563	-1869	13466 AN2740
126	CONTIG49	15087	15584	3.12	0.00E+00	transcription_start_site	-	11569	11569	-3766	13467 AN2741
126	CONTIG49	15087	15584	3.12	0.00E+00	transcription_start_site	-	11349	11349	-3986	13468 AN2741
126	CONTIG49	15087	15584	3.12	0.00E+00	transcription_start_site	-	11072	11072	-4263	13469 AN2741
2504	CONTIG49	15832	16711	1.2	7.51E-02	transcription_start_site	-	11569	11569	-4702	13467 AN2741
2504	CONTIG49	15832	16711	1.2	7.51E-02	transcription_start_site	-	11349	11349	-4922	13468 AN2741
2504	CONTIG49	15832	16711	1.2	7.51E-02	transcription_start_site	-	11072	11072	-5199	13469 AN2741
126	CONTIG49	15087	15584	3.12	0.00E+00	transcription_start_site	-	15045	15045	-290	13470 AN2742
126	CONTIG49	15087	15584	3.12	0.00E+00	transcription_start_site	-	14824	14824	-511	13471 AN2742
126	CONTIG49	15087	15584	3.12	0.00E+00	transcription_start_site	-	14717	14717	-618	13472 AN2742
126	CONTIG49	15087	15584	3.12	0.00E+00	transcription_start_site	-	14632	14632	-703	13473 AN2742
2504	CONTIG49	15832	16711	1.2	7.51E-02	transcription_start_site	-	15045	15045	-1226	13470 AN2742
2504	CONTIG49	15832	16711	1.2	7.51E-02	transcription_start_site	-	14824	14824	-1447	13471 AN2742
2504	CONTIG49	15832	16711	1.2	7.51E-02	transcription_start_site	-	14717	14717	-1554	13472 AN2742
2504	CONTIG49	15832	16711	1.2	7.51E-02	transcription_start_site	-	14632	14632	-1639	13473 AN2742
126	CONTIG49	15087	15584	3.12	0.00E+00	transcription_start_site	+	16482	16482	-1146	13474 AN2743
126	CONTIG49	15087	15584	3.12	0.00E+00	transcription_start_site	+	16607	16607	-1271	13475 AN2743
126	CONTIG49	15087	15584	3.12	0.00E+00	transcription_start_site	+	16888	16888	-1552	13476 AN2743
126	CONTIG49	15087	15584	3.12	0.00E+00	transcription_start_site	+	19317	19317	-3981	13477 AN2743
2504	CONTIG49	15832	16711	1.2	7.51E-02	transcription_start_site	+	16482	16482	-210	13474 AN2743
2504	CONTIG49	15832	16711	1.2	7.51E-02	transcription_start_site	+	16607	16607	-335	13475 AN2743
2504	CONTIG49	15832	16711	1.2	7.51E-02	transcription_start_site	+	16888	16888	-616	13476 AN2743
2504	CONTIG49	15832	16711	1.2	7.51E-02	transcription_start_site	+	19317	19317	-3045	13477 AN2743
1543	CONTIG49	35420	36286	1.56	1.05E-02	transcription_start_site	-	34475	34475	-1378	13492 AN2749
1543	CONTIG49	35420	36286	1.56	1.05E-02	transcription_start_site	-	34376	34376	-1477	13493 AN2749
1543	CONTIG49	35420	36286	1.56	1.05E-02	transcription_start_site	-	33999	33999	-1854	13494 AN2749
1543	CONTIG49	35420	36286	1.56	1.05E-02	transcription_start_site	-	33786	33786	-2067	13495 AN2749
1543	CONTIG49	35420	36286	1.56	1.05E-02	transcription_start_site	+	37461	37461	-1608	13496 AN2750
1543	CONTIG49	35420	36286	1.56	1.05E-02	transcription_start_site	+	38107	38107	-2254	13497 AN2750
1543	CONTIG49	35420	36286	1.56	1.05E-02	transcription_start_site	+	38937	38937	-3084	13498 AN2750
2908	CONTIG49	42753	43192	1	1.93E-01	transcription_start_site	-	43568	43568	595	13500 AN2751
2908	CONTIG49	42753	43192	1	1.93E-01	transcription_start_site	-	43801	43801	828	13499 AN2751
2908	CONTIG49	42753	43192	1	1.93E-01	transcription_start_site	-	41322	41322	-1650	13501 AN2751
2908	CONTIG49	42753	43192	1	1.93E-01	transcription_start_site	+	44684	44684	-1711	13502 AN2752
2908	CONTIG49	42753	43192	1	1.93E-01	transcription_start_site	+	44789	44789	-1816	13503 AN2752
2908	CONTIG49	42753	43192	1	1.93E-01	transcription_start_site	+	45628	45628	-2655	13504 AN2752
2908	CONTIG49	42753	43192	1	1.93E-01	transcription_start_site	+	45972	45972	-2999	13505 AN2752
2253	CONTIG49	50188	50677	1.28	6.84E-02	transcription_start_site	-	46851	46851	-3581	13506 AN2753
2253	CONTIG49	50188	50677	1.28	6.84E-02	transcription_start_site	-	46598	46598	-3834	13507 AN2753
2253	CONTIG49	50188	50677	1.28	6.84E-02	transcription_start_site	-	46422	46422	-4010	13508 AN2753
2253	CONTIG49	50188	50677	1.28	6.84E-02	transcription_start_site	+	53697	53697	-3264	13509 AN2755
2253	CONTIG49	50188	50677	1.28	6.84E-02	transcription_start_site	+	54042	54042	-3609	13510 AN2755
2780	CONTIG49	70518	70927	1.08	1.44E-01	transcription_start_site	+	73399	73399	-2676	13520 AN2759
2780	CONTIG49	70518	70927	1.08	1.44E-01	transcription_start_site	+	73758	73758	-3035	13521 AN2759
2780	CONTIG49	70518	70927	1.08	1.44E-01	transcription_start_site	+	74092	74092	-3369	13522 AN2759
2130	CONTIG49	80629	80981	1.32	5.88E-02	transcription_start_site	-	80292	80292	-513	13525 AN2761
2130	CONTIG49	80629	80981	1.32	5.88E-02	transcription_start_site	-	80098	80098	-707	13526 AN2761
2130	CONTIG49	80629	80981	1.32	5.88E-02	transcription_start_site	-	79953	79953	-852	13527 AN2761
2130	CONTIG49	80629	80981	1.32	5.88E-02	transcription_start_site	-	79802	79802	-1003	13528 AN2761
2130	CONTIG49	80629	80981	1.32	5.88E-02	transcription_start_site	-	79560	79560	-1245	13529 AN2761
2130	CONTIG49	80629	80981	1.32	5.88E-02	transcription_start_site	-	81409	81409	604	13533 AN2762
2130	CONTIG49	80629	80981	1.32	5.88E-02	transcription_start_site	-	81569	81569	764	13532 AN2762
1546	CONTIG49	88369	88651	1.56	2.12E-02	transcription_start_site	-	85748	85748	-2762	13534 AN2763
1546	CONTIG49	88369	88651	1.56	2.12E-02	transcription_start_site	-	84885	84885	-3625	13535 AN2763

1546	CONTIG49	88369	88651	1.56	2.12E-02	transcription_start_site	-	84529	84529	-3981	13536	AN2763
1546	CONTIG49	88369	88651	1.56	2.12E-02	transcription_start_site	+	87600	87600	910	13541	AN2764
1546	CONTIG49	88369	88651	1.56	2.12E-02	transcription_start_site	+	90263	90263	-1753	13542	AN2765
1546	CONTIG49	88369	88651	1.56	2.12E-02	transcription_start_site	+	90428	90428	-1918	13543	AN2765
1546	CONTIG49	88369	88651	1.56	2.12E-02	transcription_start_site	+	90613	90613	-2103	13544	AN2765
1546	CONTIG49	88369	88651	1.56	2.12E-02	transcription_start_site	+	90760	90760	-2250	13545	AN2765
1546	CONTIG49	88369	88651	1.56	2.12E-02	transcription_start_site	+	91030	91030	-2520	13546	AN2765
1546	CONTIG49	88369	88651	1.56	2.12E-02	transcription_start_site	+	91141	91141	-2631	13547	AN2765
1546	CONTIG49	88369	88651	1.56	2.12E-02	transcription_start_site	+	91366	91366	-2856	13548	AN2765
1546	CONTIG49	88369	88651	1.56	2.12E-02	transcription_start_site	+	93342	93342	-4832	13549	AN2766
2369	CONTIG49	92719	93283	1.24	8.01E-02	transcription_start_site	+	93342	93342	-341	13549	AN2766
2369	CONTIG49	92719	93283	1.24	8.01E-02	transcription_start_site	+	93780	93780	-779	13550	AN2766
2369	CONTIG49	92719	93283	1.24	8.01E-02	transcription_start_site	+	94533	94533	-1532	13551	AN2766
2369	CONTIG49	92719	93283	1.24	8.01E-02	transcription_start_site	+	95545	95545	-2544	13552	AN2766
894	CONTIG49	111471	111950	2	0.00E+00	transcription_start_site	-	110186	110186	-1524	13558	AN2770
894	CONTIG49	111471	111950	2	0.00E+00	transcription_start_site	-	109685	109685	-2025	13559	AN2770
894	CONTIG49	111471	111950	2	0.00E+00	transcription_start_site	-	108310	108310	-3400	13560	AN2770
984	CONTIG49	109206	109495	1.92	4.61E-03	transcription_start_site	-	109685	109685	334	13559	AN2770
984	CONTIG49	109206	109495	1.92	4.61E-03	transcription_start_site	-	110186	110186	835	13558	AN2770
984	CONTIG49	109206	109495	1.92	4.61E-03	transcription_start_site	-	108310	108310	-1040	13560	AN2770
1311	CONTIG49	109896	110525	1.68	1.30E-02	transcription_start_site	-	110186	110186	-24	13558	AN2770
1311	CONTIG49	109896	110525	1.68	1.30E-02	transcription_start_site	-	109685	109685	-525	13559	AN2770
1311	CONTIG49	109896	110525	1.68	1.30E-02	transcription_start_site	-	108310	108310	-1900	13560	AN2770
894	CONTIG49	111471	111950	2	0.00E+00	transcription_start_site	+	111156	111156	554	13561	AN2771
984	CONTIG49	109206	109495	1.92	4.61E-03	transcription_start_site	+	111156	111156	-1805	13561	AN2771
1311	CONTIG49	109896	110525	1.68	1.30E-02	transcription_start_site	+	111156	111156	-945	13561	AN2771
1909	CONTIG49	126771	127555	1.4	2.47E-02	transcription_start_site	-	125568	125568	-1595	13570	AN2775
1909	CONTIG49	126771	127555	1.4	2.47E-02	transcription_start_site	-	125471	125471	-1692	13571	AN2775
1909	CONTIG49	126771	127555	1.4	2.47E-02	transcription_start_site	-	125219	125219	-1944	13572	AN2775
1909	CONTIG49	126771	127555	1.4	2.47E-02	transcription_start_site	-	124759	124759	-2404	13573	AN2775
1909	CONTIG49	126771	127555	1.4	2.47E-02	transcription_start_site	-	124481	124481	-2682	13574	AN2775
1909	CONTIG49	126771	127555	1.4	2.47E-02	transcription_start_site	-	127285	127285	122	13576	AN2776
1909	CONTIG49	126771	127555	1.4	2.47E-02	transcription_start_site	-	127408	127408	245	13575	AN2776
2909	CONTIG49	133971	134233	1	1.93E-01	transcription_start_site	-	130225	130225	-3877	13577	AN2777
2909	CONTIG49	133971	134233	1	1.93E-01	transcription_start_site	-	131699	131699	-2403	13578	AN2778
2909	CONTIG49	133971	134233	1	1.93E-01	transcription_start_site	-	130969	130969	-3133	13579	AN2778
2909	CONTIG49	133971	134233	1	1.93E-01	transcription_start_site	-	130919	130919	-3183	13580	AN2778
1909	CONTIG49	126771	127555	1.4	2.47E-02	transcription_start_site	+	132155	132155	-4992	13581	AN2779
1909	CONTIG49	126771	127555	1.4	2.47E-02	transcription_start_site	+	132425	132425	-5262	13582	AN2779
2909	CONTIG49	133971	134233	1	1.93E-01	transcription_start_site	+	133573	133573	529	13587	AN2779
2909	CONTIG49	133971	134233	1	1.93E-01	transcription_start_site	+	133302	133302	800	13586	AN2779
2909	CONTIG49	133971	134233	1	1.93E-01	transcription_start_site	+	133072	133072	1030	13585	AN2779
2909	CONTIG49	133971	134233	1	1.93E-01	transcription_start_site	+	134234	134234	-132	13588	AN2780
771	CONTIG49	169059	169333	2.12	1.54E-03	transcription_start_site	-	164758	164758	-4438	13626	AN2792
771	CONTIG49	169059	169333	2.12	1.54E-03	transcription_start_site	-	164504	164504	-4692	13627	AN2792
771	CONTIG49	169059	169333	2.12	1.54E-03	transcription_start_site	-	164191	164191	-5005	13628	AN2792
771	CONTIG49	169059	169333	2.12	1.54E-03	transcription_start_site	-	166681	166681	-2515	13631	AN2793
771	CONTIG49	169059	169333	2.12	1.54E-03	transcription_start_site	-	166149	166149	-3047	13632	AN2793
771	CONTIG49	169059	169333	2.12	1.54E-03	transcription_start_site	-	165668	165668	-3528	13633	AN2793
771	CONTIG49	169059	169333	2.12	1.54E-03	transcription_start_site	+	170235	170235	-1039	13634	AN2794
771	CONTIG49	169059	169333	2.12	1.54E-03	transcription_start_site	+	170538	170538	-1342	13635	AN2794
771	CONTIG49	169059	169333	2.12	1.54E-03	transcription_start_site	+	170825	170825	-1629	13636	AN2794
771	CONTIG49	169059	169333	2.12	1.54E-03	transcription_start_site	+	172625	172625	-3429	13637	AN2795
771	CONTIG49	169059	169333	2.12	1.54E-03	transcription_start_site	+	174312	174312	-5116	13638	AN2795
1129	CONTIG50	6546	6895	1.81	2.64E-02	transcription_start_site	+	6935	6935	-214	13692	AN2809
1129	CONTIG50	6546	6895	1.81	2.64E-02	transcription_start_site	+	7173	7173	-452	13693	AN2809
1959	CONTIG50	8251	8545	1.39	1.44E-01	transcription_start_site	-	9062	9062	664	13696	AN2810

1207	CONTIG50	30838	31102	1.76	5.22E-02	transcription_start_site	-	27697	27697	-3273	13706	AN2814
1207	CONTIG50	30838	31102	1.76	5.22E-02	transcription_start_site	-	27532	27532	-3438	13707	AN2814
1207	CONTIG50	30838	31102	1.76	5.22E-02	transcription_start_site	-	27419	27419	-3551	13708	AN2814
1207	CONTIG50	30838	31102	1.76	5.22E-02	transcription_start_site	-	27318	27318	-3652	13709	AN2814
1207	CONTIG50	30838	31102	1.76	5.22E-02	transcription_start_site	-	27159	27159	-3811	13710	AN2814
1207	CONTIG50	30838	31102	1.76	5.22E-02	transcription_start_site	-	26864	26864	-4106	13711	AN2814
1207	CONTIG50	30838	31102	1.76	5.22E-02	transcription_start_site	-	26496	26496	-4474	13712	AN2814
1207	CONTIG50	30838	31102	1.76	5.22E-02	transcription_start_site	-	26176	26176	-4794	13713	AN2814
107	CONTIG50	34138	34487	3.2	2.67E-04	transcription_start_site	+	34837	34837	-524	13715	AN2815
1207	CONTIG50	30838	31102	1.76	5.22E-02	transcription_start_site	+	34837	34837	-3867	13715	AN2815
839	CONTIG51	12829	13103	2.06	1.30E-02	transcription_start_site	+	13540	13540	-574	13730	AN2822
1256	CONTIG51	13139	13853	1.72	2.45E-02	transcription_start_site	+	13540	13540	-44	13730	AN2822
1654	CONTIG51	23344	23611	1.52	8.01E-02	transcription_start_site	-	21165	21165	-2312	13733	AN2824
1654	CONTIG51	23344	23611	1.52	8.01E-02	transcription_start_site	-	18530	18530	-4947	13734	AN2824
1654	CONTIG51	23344	23611	1.52	8.01E-02	transcription_start_site	-	18398	18398	-5079	13735	AN2824
1654	CONTIG51	23344	23611	1.52	8.01E-02	transcription_start_site	+	28209	28209	-4731	13738	AN2827
1654	CONTIG51	23344	23611	1.52	8.01E-02	transcription_start_site	+	28302	28302	-4824	13739	AN2827
1655	CONTIG51	40745	42356	1.52	8.01E-02	transcription_start_site	-	37684	37684	-3866	13741	AN2828
1655	CONTIG51	40745	42356	1.52	8.01E-02	transcription_start_site	-	37093	37093	-4457	13742	AN2828
1655	CONTIG51	40745	42356	1.52	8.01E-02	transcription_start_site	-	36325	36325	-5225	13743	AN2828
1655	CONTIG51	40745	42356	1.52	8.01E-02	transcription_start_site	-	36112	36112	-5438	13744	AN2828
1655	CONTIG51	40745	42356	1.52	8.01E-02	transcription_start_site	-	40113	40113	-1437	13746	AN2829
1655	CONTIG51	40745	42356	1.52	8.01E-02	transcription_start_site	-	38656	38656	-2894	13747	AN2829
1655	CONTIG51	40745	42356	1.52	8.01E-02	transcription_start_site	-	38523	38523	-3027	13748	AN2829
1655	CONTIG51	40745	42356	1.52	8.01E-02	transcription_start_site	-	41841	41841	290	13749	AN2830
2303	CONTIG51	78542	78808	1.27	1.66E-01	transcription_start_site	-	73921	73921	-4754	13780	AN2841
2303	CONTIG51	78542	78808	1.27	1.66E-01	transcription_start_site	-	76227	76227	-2448	13782	AN2842
2303	CONTIG51	78542	78808	1.27	1.66E-01	transcription_start_site	-	75523	75523	-3152	13783	AN2842
2303	CONTIG51	78542	78808	1.27	1.66E-01	transcription_start_site	+	78140	78140	535	13784	AN2843
2303	CONTIG51	78542	78808	1.27	1.66E-01	transcription_start_site	+	79854	79854	-1179	13785	AN2844
2303	CONTIG51	78542	78808	1.27	1.66E-01	transcription_start_site	+	80267	80267	-1592	13786	AN2844
2303	CONTIG51	78542	78808	1.27	1.66E-01	transcription_start_site	+	80459	80459	-1784	13787	AN2844
247	CONTIG51	101271	101540	2.84	5.64E-04	transcription_start_site	-	100678	100678	-727	13809	AN2852
247	CONTIG51	101271	101540	2.84	5.64E-04	transcription_start_site	-	100338	100338	-1067	13810	AN2852
247	CONTIG51	101271	101540	2.84	5.64E-04	transcription_start_site	-	99482	99482	-1923	13811	AN2852
247	CONTIG51	101271	101540	2.84	5.64E-04	transcription_start_site	-	98823	98823	-2582	13812	AN2852
889	CONTIG51	101716	102220	2.01	1.57E-02	transcription_start_site	-	100678	100678	-1290	13809	AN2852
889	CONTIG51	101716	102220	2.01	1.57E-02	transcription_start_site	-	100338	100338	-1630	13810	AN2852
889	CONTIG51	101716	102220	2.01	1.57E-02	transcription_start_site	-	99482	99482	-2486	13811	AN2852
889	CONTIG51	101716	102220	2.01	1.57E-02	transcription_start_site	-	98823	98823	-3145	13812	AN2852
2304	CONTIG51	104861	105200	1.27	1.66E-01	transcription_start_site	-	100678	100678	-4352	13809	AN2852
2304	CONTIG51	104861	105200	1.27	1.66E-01	transcription_start_site	-	100338	100338	-4692	13810	AN2852
637	CONTIG51	107252	107676	2.26	6.61E-03	transcription_start_site	-	104014	104014	-3450	13813	AN2853
2017	CONTIG51	105762	106116	1.37	1.25E-01	transcription_start_site	-	104014	104014	-1925	13813	AN2853
2304	CONTIG51	104861	105200	1.27	1.66E-01	transcription_start_site	-	104014	104014	-1016	13813	AN2853
248	CONTIG51	110642	111051	2.84	5.64E-04	transcription_start_site	-	106882	106882	-3964	13814	AN2854
248	CONTIG51	110642	111051	2.84	5.64E-04	transcription_start_site	-	106630	106630	-4216	13815	AN2854
637	CONTIG51	107252	107676	2.26	6.61E-03	transcription_start_site	-	106882	106882	-582	13814	AN2854
637	CONTIG51	107252	107676	2.26	6.61E-03	transcription_start_site	-	106630	106630	-834	13815	AN2854
637	CONTIG51	107252	107676	2.26	6.61E-03	transcription_start_site	-	104629	104629	-2835	13816	AN2854
2017	CONTIG51	105762	106116	1.37	1.25E-01	transcription_start_site	-	106630	106630	691	13815	AN2854
2017	CONTIG51	105762	106116	1.37	1.25E-01	transcription_start_site	-	106882	106882	943	13814	AN2854
2017	CONTIG51	105762	106116	1.37	1.25E-01	transcription_start_site	-	104629	104629	-1310	13816	AN2854
2304	CONTIG51	104861	105200	1.27	1.66E-01	transcription_start_site	-	104629	104629	-401	13816	AN2854
248	CONTIG51	110642	111051	2.84	5.64E-04	transcription_start_site	-	110402	110402	-444	13817	AN2855
248	CONTIG51	110642	111051	2.84	5.64E-04	transcription_start_site	-	110035	110035	-811	13818	AN2855
248	CONTIG51	110642	111051	2.84	5.64E-04	transcription_start_site	-	109693	109693	-1153	13819	AN2855

1434	CONTIG51	118805	119244	1.62	5.88E-02	transcription_start_site	-	114176	114176	-4848	13820 AN2856
248	CONTIG51	110642	111051	2.84	5.64E-04	transcription_start_site	+	115254	115254	-4407	13824 AN2857
1434	CONTIG51	118805	119244	1.62	5.88E-02	transcription_start_site	-	118951	118951	-73	13827 AN2858
1434	CONTIG51	118805	119244	1.62	5.88E-02	transcription_start_site	-	119201	119201	176	13826 AN2858
1434	CONTIG51	118805	119244	1.62	5.88E-02	transcription_start_site	-	118190	118190	-834	13828 AN2858
1434	CONTIG51	118805	119244	1.62	5.88E-02	transcription_start_site	-	118046	118046	-978	13829 AN2858
1533	CONTIG51	122855	123129	1.57	6.84E-02	transcription_start_site	-	119201	119201	-3791	13826 AN2858
1533	CONTIG51	122855	123129	1.57	6.84E-02	transcription_start_site	-	118951	118951	-4041	13827 AN2858
1533	CONTIG51	122855	123129	1.57	6.84E-02	transcription_start_site	-	118190	118190	-4802	13828 AN2858
1533	CONTIG51	122855	123129	1.57	6.84E-02	transcription_start_site	-	118046	118046	-4946	13829 AN2858
1434	CONTIG51	118805	119244	1.62	5.88E-02	transcription_start_site	+	119746	119746	-721	13830 AN2859
1434	CONTIG51	118805	119244	1.62	5.88E-02	transcription_start_site	+	121446	121446	-2421	13831 AN2860
1434	CONTIG51	118805	119244	1.62	5.88E-02	transcription_start_site	+	121576	121576	-2551	13832 AN2860
1434	CONTIG51	118805	119244	1.62	5.88E-02	transcription_start_site	+	121656	121656	-2631	13833 AN2860
1434	CONTIG51	118805	119244	1.62	5.88E-02	transcription_start_site	+	121769	121769	-2744	13834 AN2860
1434	CONTIG51	118805	119244	1.62	5.88E-02	transcription_start_site	+	121858	121858	-2833	13835 AN2860
1533	CONTIG51	122855	123129	1.57	6.84E-02	transcription_start_site	+	121858	121858	1134	13835 AN2860
1434	CONTIG51	118805	119244	1.62	5.88E-02	transcription_start_site	+	123619	123619	-4594	13836 AN2861
1533	CONTIG51	122855	123129	1.57	6.84E-02	transcription_start_site	+	123619	123619	-627	13836 AN2861
1533	CONTIG51	122855	123129	1.57	6.84E-02	transcription_start_site	+	124667	124667	-1675	13837 AN2861
202	CONTIG51	127970	128304	2.94	2.16E-04	transcription_start_site	+	127237	127237	900	13843 AN2862
1533	CONTIG51	122855	123129	1.57	6.84E-02	transcription_start_site	+	126199	126199	-3207	13838 AN2862
1533	CONTIG51	122855	123129	1.57	6.84E-02	transcription_start_site	+	126292	126292	-3300	13839 AN2862
1533	CONTIG51	122855	123129	1.57	6.84E-02	transcription_start_site	+	126473	126473	-3481	13840 AN2862
1533	CONTIG51	122855	123129	1.57	6.84E-02	transcription_start_site	+	126570	126570	-3578	13841 AN2862
1533	CONTIG51	122855	123129	1.57	6.84E-02	transcription_start_site	+	126706	126706	-3714	13842 AN2862
1533	CONTIG51	122855	123129	1.57	6.84E-02	transcription_start_site	+	127237	127237	-4245	13843 AN2862
202	CONTIG51	127970	128304	2.94	2.16E-04	transcription_start_site	+	128876	128876	-739	13844 AN2863
202	CONTIG51	127970	128304	2.94	2.16E-04	transcription_start_site	+	131400	131400	-3263	13845 AN2864
202	CONTIG51	127970	128304	2.94	2.16E-04	transcription_start_site	+	131597	131597	-3460	13846 AN2864
202	CONTIG51	127970	128304	2.94	2.16E-04	transcription_start_site	+	132016	132016	-3879	13847 AN2864
2305	CONTIG51	130295	130779	1.27	1.66E-01	transcription_start_site	+	131400	131400	-863	13845 AN2864
2305	CONTIG51	130295	130779	1.27	1.66E-01	transcription_start_site	+	131597	131597	-1060	13846 AN2864
2305	CONTIG51	130295	130779	1.27	1.66E-01	transcription_start_site	+	132016	132016	-1479	13847 AN2864
2305	CONTIG51	130295	130779	1.27	1.66E-01	transcription_start_site	+	134491	134491	-3954	13848 AN2865
1520	CONTIG51	156237	157936	1.57	3.41E-02	transcription_start_site	-	155006	155006	-2080	13861 AN2870
1520	CONTIG51	156237	157936	1.57	3.41E-02	transcription_start_site	-	154217	154217	-2869	13862 AN2870
2018	CONTIG51	159004	159375	1.37	1.25E-01	transcription_start_site	-	155006	155006	-4183	13861 AN2870
2018	CONTIG51	159004	159375	1.37	1.25E-01	transcription_start_site	-	154217	154217	-4972	13862 AN2870
1520	CONTIG51	156237	157936	1.57	3.41E-02	transcription_start_site	-	158287	158287	1200	13864 AN2871
1520	CONTIG51	156237	157936	1.57	3.41E-02	transcription_start_site	-	158651	158651	1564	13863 AN2871
2018	CONTIG51	159004	159375	1.37	1.25E-01	transcription_start_site	-	158651	158651	-538	13863 AN2871
2018	CONTIG51	159004	159375	1.37	1.25E-01	transcription_start_site	-	158287	158287	-902	13864 AN2871
1520	CONTIG51	156237	157936	1.57	3.41E-02	transcription_start_site	+	159635	159635	-2548	13865 AN2872
1520	CONTIG51	156237	157936	1.57	3.41E-02	transcription_start_site	+	161089	161089	-4002	13866 AN2872
1520	CONTIG51	156237	157936	1.57	3.41E-02	transcription_start_site	+	161636	161636	-4549	13867 AN2872
2018	CONTIG51	159004	159375	1.37	1.25E-01	transcription_start_site	+	159635	159635	-445	13865 AN2872
2018	CONTIG51	159004	159375	1.37	1.25E-01	transcription_start_site	+	161089	161089	-1899	13866 AN2872
2018	CONTIG51	159004	159375	1.37	1.25E-01	transcription_start_site	+	161636	161636	-2446	13867 AN2872
1520	CONTIG51	156237	157936	1.57	3.41E-02	transcription_start_site	+	162550	162550	-5463	13868 AN2873
1520	CONTIG51	156237	157936	1.57	3.41E-02	transcription_start_site	+	162662	162662	-5575	13869 AN2873
1520	CONTIG51	156237	157936	1.57	3.41E-02	transcription_start_site	+	162857	162857	-5770	13870 AN2873
1520	CONTIG51	156237	157936	1.57	3.41E-02	transcription_start_site	+	162932	162932	-5845	13871 AN2873
2018	CONTIG51	159004	159375	1.37	1.25E-01	transcription_start_site	+	162550	162550	-3360	13868 AN2873
2018	CONTIG51	159004	159375	1.37	1.25E-01	transcription_start_site	+	162662	162662	-3472	13869 AN2873
2018	CONTIG51	159004	159375	1.37	1.25E-01	transcription_start_site	+	162857	162857	-3667	13870 AN2873
2018	CONTIG51	159004	159375	1.37	1.25E-01	transcription_start_site	+	162932	162932	-3742	13871 AN2873

2018	CONTIG51	159004	159375	1.37	1.25E-01	transcription_start_site	+	163228	163228	-4038	13872 AN2873
2018	CONTIG51	159004	159375	1.37	1.25E-01	transcription_start_site	+	163306	163306	-4116	13873 AN2873
2018	CONTIG51	159004	159375	1.37	1.25E-01	transcription_start_site	+	163531	163531	-4341	13874 AN2873
2018	CONTIG51	159004	159375	1.37	1.25E-01	transcription_start_site	+	163675	163675	-4485	13875 AN2873
2018	CONTIG51	159004	159375	1.37	1.25E-01	transcription_start_site	+	163824	163824	-4634	13876 AN2873
2018	CONTIG51	159004	159375	1.37	1.25E-01	transcription_start_site	+	163946	163946	-4756	13877 AN2873
791	CONTIG51	175507	175784	2.11	1.11E-02	transcription_start_site	+	176018	176018	-372	13885 AN2875
791	CONTIG51	175507	175784	2.11	1.11E-02	transcription_start_site	+	176182	176182	-536	13886 AN2875
791	CONTIG51	175507	175784	2.11	1.11E-02	transcription_start_site	+	176341	176341	-695	13887 AN2875
791	CONTIG51	175507	175784	2.11	1.11E-02	transcription_start_site	+	176696	176696	-1050	13888 AN2875
791	CONTIG51	175507	175784	2.11	1.11E-02	transcription_start_site	+	177923	177923	-2277	13889 AN2876
791	CONTIG51	175507	175784	2.11	1.11E-02	transcription_start_site	+	178139	178139	-2493	13890 AN2876
791	CONTIG51	175507	175784	2.11	1.11E-02	transcription_start_site	+	178224	178224	-2578	13891 AN2876
791	CONTIG51	175507	175784	2.11	1.11E-02	transcription_start_site	+	178758	178758	-3112	13892 AN2876
791	CONTIG51	175507	175784	2.11	1.11E-02	transcription_start_site	+	178928	178928	-3282	13893 AN2876
791	CONTIG51	175507	175784	2.11	1.11E-02	transcription_start_site	+	179187	179187	-3541	13894 AN2876
791	CONTIG51	175507	175784	2.11	1.11E-02	transcription_start_site	+	179473	179473	-3827	13895 AN2876
791	CONTIG51	175507	175784	2.11	1.11E-02	transcription_start_site	+	179590	179590	-3944	13896 AN2876
791	CONTIG51	175507	175784	2.11	1.11E-02	transcription_start_site	+	179748	179748	-4102	13897 AN2876
608	CONTIG51	187361	187630	2.3	5.20E-03	transcription_start_site	+	186641	186641	854	13908 AN2879
608	CONTIG51	187361	187630	2.3	5.20E-03	transcription_start_site	+	186501	186501	994	13907 AN2879
608	CONTIG51	187361	187630	2.3	5.20E-03	transcription_start_site	+	188166	188166	-670	13909 AN2880
608	CONTIG51	187361	187630	2.3	5.20E-03	transcription_start_site	+	188301	188301	-805	13910 AN2880
608	CONTIG51	187361	187630	2.3	5.20E-03	transcription_start_site	+	188890	188890	-1394	13911 AN2880
608	CONTIG51	187361	187630	2.3	5.20E-03	transcription_start_site	+	191260	191260	-3764	13912 AN2880
608	CONTIG51	187361	187630	2.3	5.20E-03	transcription_start_site	+	192528	192528	-5032	13913 AN2881
2306	CONTIG51	194936	195210	1.27	1.66E-01	transcription_start_site	-	195138	195138	65	13917 AN2882
2306	CONTIG51	194936	195210	1.27	1.66E-01	transcription_start_site	-	195277	195277	204	13916 AN2882
2306	CONTIG51	194936	195210	1.27	1.66E-01	transcription_start_site	+	195540	195540	-467	13918 AN2883
1777	CONTIG51	202962	203301	1.47	7.51E-02	transcription_start_site	-	198959	198959	-4172	13919 AN2884
1777	CONTIG51	202962	203301	1.47	7.51E-02	transcription_start_site	-	198487	198487	-4644	13920 AN2884
1777	CONTIG51	202962	203301	1.47	7.51E-02	transcription_start_site	-	198029	198029	-5102	13921 AN2884
1883	CONTIG51	201617	202341	1.42	9.26E-02	transcription_start_site	-	198959	198959	-3020	13919 AN2884
1883	CONTIG51	201617	202341	1.42	9.26E-02	transcription_start_site	-	198487	198487	-3492	13920 AN2884
1883	CONTIG51	201617	202341	1.42	9.26E-02	transcription_start_site	-	198029	198029	-3950	13921 AN2884
1883	CONTIG51	201617	202341	1.42	9.26E-02	transcription_start_site	-	197901	197901	-4078	13922 AN2884
1883	CONTIG51	201617	202341	1.42	9.26E-02	transcription_start_site	-	197638	197638	-4341	13923 AN2884
1883	CONTIG51	201617	202341	1.42	9.26E-02	transcription_start_site	-	197436	197436	-4543	13924 AN2884
1883	CONTIG51	201617	202341	1.42	9.26E-02	transcription_start_site	-	197083	197083	-4896	13925 AN2884
1777	CONTIG51	202962	203301	1.47	7.51E-02	transcription_start_site	-	201379	201379	-1752	13926 AN2885
1777	CONTIG51	202962	203301	1.47	7.51E-02	transcription_start_site	-	201260	201260	-1871	13927 AN2885
1777	CONTIG51	202962	203301	1.47	7.51E-02	transcription_start_site	-	201111	201111	-2020	13928 AN2885
1777	CONTIG51	202962	203301	1.47	7.51E-02	transcription_start_site	-	200926	200926	-2205	13929 AN2885
1883	CONTIG51	201617	202341	1.42	9.26E-02	transcription_start_site	-	201379	201379	-600	13926 AN2885
1883	CONTIG51	201617	202341	1.42	9.26E-02	transcription_start_site	-	201260	201260	-719	13927 AN2885
1883	CONTIG51	201617	202341	1.42	9.26E-02	transcription_start_site	-	201111	201111	-868	13928 AN2885
1883	CONTIG51	201617	202341	1.42	9.26E-02	transcription_start_site	-	200926	200926	-1053	13929 AN2885
1777	CONTIG51	202962	203301	1.47	7.51E-02	transcription_start_site	+	205287	205287	-2155	13933 AN2887
1777	CONTIG51	202962	203301	1.47	7.51E-02	transcription_start_site	+	205374	205374	-2242	13934 AN2887
1883	CONTIG51	201617	202341	1.42	9.26E-02	transcription_start_site	+	205287	205287	-3308	13933 AN2887
1883	CONTIG51	201617	202341	1.42	9.26E-02	transcription_start_site	+	205374	205374	-3395	13934 AN2887
687	CONTIG51	215632	215901	2.21	7.85E-03	transcription_start_site	-	211143	211143	-4623	13936 AN2888
687	CONTIG51	215632	215901	2.21	7.85E-03	transcription_start_site	-	216062	216062	295	13939 AN2890
687	CONTIG51	215632	215901	2.21	7.85E-03	transcription_start_site	-	216607	216607	840	13938 AN2890
1435	CONTIG51	222676	223195	1.62	5.88E-02	transcription_start_site	-	218304	218304	-4631	13940 AN2891
1435	CONTIG51	222676	223195	1.62	5.88E-02	transcription_start_site	-	218026	218026	-4909	13941 AN2891
1435	CONTIG51	222676	223195	1.62	5.88E-02	transcription_start_site	-	220755	220755	-2180	13943 AN2892

1435	CONTIG51	222676	223195	1.62	5.88E-02	transcription_start_site	-	220550	220550	-2385	13944	AN2892
1435	CONTIG51	222676	223195	1.62	5.88E-02	transcription_start_site	-	220122	220122	-2813	13945	AN2892
1435	CONTIG51	222676	223195	1.62	5.88E-02	transcription_start_site	-	219885	219885	-3050	13946	AN2892
1435	CONTIG51	222676	223195	1.62	5.88E-02	transcription_start_site	-	219342	219342	-3593	13947	AN2892
1435	CONTIG51	222676	223195	1.62	5.88E-02	transcription_start_site	+	222661	222661	274	13948	AN2893
1435	CONTIG51	222676	223195	1.62	5.88E-02	transcription_start_site	-	224049	224049	1113	13950	AN2894
1124	CONTIG51	230643	230995	1.81	1.81E-02	transcription_start_site	-	229970	229970	-849	13951	AN2895
1124	CONTIG51	230643	230995	1.81	1.81E-02	transcription_start_site	-	229508	229508	-1311	13952	AN2895
1124	CONTIG51	230643	230995	1.81	1.81E-02	transcription_start_site	-	228491	228491	-2328	13953	AN2895
952	CONTIG51	240317	240731	1.96	1.84E-02	transcription_start_site	-	237723	237723	-2801	13956	AN2897
952	CONTIG51	240317	240731	1.96	1.84E-02	transcription_start_site	-	237499	237499	-3025	13957	AN2897
952	CONTIG51	240317	240731	1.96	1.84E-02	transcription_start_site	+	241347	241347	-823	13960	AN2898
952	CONTIG51	240317	240731	1.96	1.84E-02	transcription_start_site	+	245386	245386	-4862	13964	AN2900
2019	CONTIG51	256214	256493	1.37	1.25E-01	transcription_start_site	-	253838	253838	-2515	13971	AN2902
2019	CONTIG51	256214	256493	1.37	1.25E-01	transcription_start_site	-	253539	253539	-2814	13972	AN2902
2019	CONTIG51	256214	256493	1.37	1.25E-01	transcription_start_site	-	253403	253403	-2950	13973	AN2902
2424	CONTIG51	254339	254617	1.23	1.93E-01	transcription_start_site	-	253838	253838	-640	13971	AN2902
2424	CONTIG51	254339	254617	1.23	1.93E-01	transcription_start_site	-	253539	253539	-939	13972	AN2902
2424	CONTIG51	254339	254617	1.23	1.93E-01	transcription_start_site	-	253403	253403	-1075	13973	AN2902
2019	CONTIG51	256214	256493	1.37	1.25E-01	transcription_start_site	-	256203	256203	-150	13974	AN2903
2019	CONTIG51	256214	256493	1.37	1.25E-01	transcription_start_site	-	256038	256038	-315	13975	AN2903
2019	CONTIG51	256214	256493	1.37	1.25E-01	transcription_start_site	-	255848	255848	-505	13976	AN2903
2019	CONTIG51	256214	256493	1.37	1.25E-01	transcription_start_site	-	255134	255134	-1219	13977	AN2903
2424	CONTIG51	254339	254617	1.23	1.93E-01	transcription_start_site	-	255134	255134	656	13977	AN2903
2019	CONTIG51	256214	256493	1.37	1.25E-01	transcription_start_site	-	257163	257163	809	13983	AN2904
2019	CONTIG51	256214	256493	1.37	1.25E-01	transcription_start_site	+	261418	261418	-5064	13984	AN2905
179	CONTIG51	275571	275843	2.99	2.39E-04	transcription_start_site	-	273162	273162	-2545	13995	AN2909
179	CONTIG51	275571	275843	2.99	2.39E-04	transcription_start_site	-	272727	272727	-2980	13996	AN2909
179	CONTIG51	275571	275843	2.99	2.39E-04	transcription_start_site	-	272083	272083	-3624	13997	AN2909
790	CONTIG51	276770	277259	2.11	4.81E-03	transcription_start_site	-	273162	273162	-3852	13995	AN2909
790	CONTIG51	276770	277259	2.11	4.81E-03	transcription_start_site	-	272727	272727	-4287	13996	AN2909
790	CONTIG51	276770	277259	2.11	4.81E-03	transcription_start_site	-	272083	272083	-4931	13997	AN2909
1429	CONTIG51	273986	274572	1.62	3.99E-02	transcription_start_site	-	273162	273162	-1117	13995	AN2909
1429	CONTIG51	273986	274572	1.62	3.99E-02	transcription_start_site	-	272727	272727	-1552	13996	AN2909
1429	CONTIG51	273986	274572	1.62	3.99E-02	transcription_start_site	-	272083	272083	-2196	13997	AN2909
179	CONTIG51	275571	275843	2.99	2.39E-04	transcription_start_site	+	274832	274832	875	13999	AN2910
1429	CONTIG51	273986	274572	1.62	3.99E-02	transcription_start_site	+	273994	273994	285	13998	AN2910
1429	CONTIG51	273986	274572	1.62	3.99E-02	transcription_start_site	+	274832	274832	-553	13999	AN2910
179	CONTIG51	275571	275843	2.99	2.39E-04	transcription_start_site	+	276855	276855	-1148	14000	AN2911
179	CONTIG51	275571	275843	2.99	2.39E-04	transcription_start_site	+	277205	277205	-1498	14001	AN2911
179	CONTIG51	275571	275843	2.99	2.39E-04	transcription_start_site	+	278390	278390	-2683	14002	AN2911
790	CONTIG51	276770	277259	2.11	4.81E-03	transcription_start_site	+	276855	276855	159	14000	AN2911
790	CONTIG51	276770	277259	2.11	4.81E-03	transcription_start_site	+	277205	277205	-190	14001	AN2911
790	CONTIG51	276770	277259	2.11	4.81E-03	transcription_start_site	+	278390	278390	-1375	14002	AN2911
1429	CONTIG51	273986	274572	1.62	3.99E-02	transcription_start_site	+	276855	276855	-2576	14000	AN2911
1429	CONTIG51	273986	274572	1.62	3.99E-02	transcription_start_site	+	277205	277205	-2926	14001	AN2911
1429	CONTIG51	273986	274572	1.62	3.99E-02	transcription_start_site	+	278390	278390	-4111	14002	AN2911
1191	CONTIG51	286126	286410	1.77	3.70E-02	transcription_start_site	-	285575	285575	-693	14003	AN2912
1191	CONTIG51	286126	286410	1.77	3.70E-02	transcription_start_site	-	285425	285425	-843	14004	AN2912
1191	CONTIG51	286126	286410	1.77	3.70E-02	transcription_start_site	-	285063	285063	-1205	14005	AN2912
1191	CONTIG51	286126	286410	1.77	3.70E-02	transcription_start_site	+	287650	287650	-1382	14006	AN2913
1191	CONTIG51	286126	286410	1.77	3.70E-02	transcription_start_site	+	287797	287797	-1529	14007	AN2913
1191	CONTIG51	286126	286410	1.77	3.70E-02	transcription_start_site	+	291013	291013	-4745	14008	AN2914
1191	CONTIG51	286126	286410	1.77	3.70E-02	transcription_start_site	+	291130	291130	-4862	14009	AN2914
571	CONTIG51	306231	306652	2.35	1.22E-03	transcription_start_site	-	305813	305813	-628	14030	AN2919
571	CONTIG51	306231	306652	2.35	1.22E-03	transcription_start_site	-	305242	305242	-1199	14031	AN2919
571	CONTIG51	306231	306652	2.35	1.22E-03	transcription_start_site	-	304692	304692	-1749	14032	AN2919

571	CONTIG51	306231	306652	2.35	1.22E-03	transcription_start_site	+	306674	306674	-232	14033 AN2920
609	CONTIG51	311110	311394	2.3	5.20E-03	transcription_start_site	-	311858	311858	606	14037 AN2921
609	CONTIG51	311110	311394	2.3	5.20E-03	transcription_start_site	+	316393	316393	-5141	14042 AN2923
638	CONTIG51	331578	331852	2.26	6.61E-03	transcription_start_site	-	331271	331271	-444	14055 AN2926
638	CONTIG51	331578	331852	2.26	6.61E-03	transcription_start_site	-	331205	331205	-510	14056 AN2926
638	CONTIG51	331578	331852	2.26	6.61E-03	transcription_start_site	-	331141	331141	-574	14057 AN2926
638	CONTIG51	331578	331852	2.26	6.61E-03	transcription_start_site	-	330587	330587	-1128	14058 AN2926
638	CONTIG51	331578	331852	2.26	6.61E-03	transcription_start_site	-	330220	330220	-1495	14059 AN2926
890	CONTIG51	363842	364406	2.01	1.57E-02	transcription_start_site	-	359656	359656	-4468	14082 AN2936
890	CONTIG51	363842	364406	2.01	1.57E-02	transcription_start_site	-	359407	359407	-4717	14083 AN2936
890	CONTIG51	363842	364406	2.01	1.57E-02	transcription_start_site	-	358846	358846	-5278	14084 AN2936
1186	CONTIG51	362045	362459	1.77	2.18E-02	transcription_start_site	-	359656	359656	-2596	14082 AN2936
1186	CONTIG51	362045	362459	1.77	2.18E-02	transcription_start_site	-	359407	359407	-2845	14083 AN2936
1186	CONTIG51	362045	362459	1.77	2.18E-02	transcription_start_site	-	358846	358846	-3406	14084 AN2936
890	CONTIG51	363842	364406	2.01	1.57E-02	transcription_start_site	+	363508	363508	616	14086 AN2937
1186	CONTIG51	362045	362459	1.77	2.18E-02	transcription_start_site	+	363508	363508	-1256	14086 AN2937
1187	CONTIG51	370652	371001	1.77	2.18E-02	transcription_start_site	-	368212	368212	-2614	14087 AN2938
1187	CONTIG51	370652	371001	1.77	2.18E-02	transcription_start_site	-	368106	368106	-2720	14088 AN2938
1187	CONTIG51	370652	371001	1.77	2.18E-02	transcription_start_site	-	367810	367810	-3016	14089 AN2938
1187	CONTIG51	370652	371001	1.77	2.18E-02	transcription_start_site	-	367700	367700	-3126	14090 AN2938
1187	CONTIG51	370652	371001	1.77	2.18E-02	transcription_start_site	-	367264	367264	-3562	14091 AN2938
1187	CONTIG51	370652	371001	1.77	2.18E-02	transcription_start_site	-	366756	366756	-4070	14092 AN2938
1187	CONTIG51	370652	371001	1.77	2.18E-02	transcription_start_site	-	366451	366451	-4375	14093 AN2938
1187	CONTIG51	370652	371001	1.77	2.18E-02	transcription_start_site	-	370571	370571	-255	14094 AN2939
1187	CONTIG51	370652	371001	1.77	2.18E-02	transcription_start_site	-	370426	370426	-400	14095 AN2939
1187	CONTIG51	370652	371001	1.77	2.18E-02	transcription_start_site	-	369853	369853	-973	14096 AN2939
1884	CONTIG51	374637	374994	1.42	9.26E-02	transcription_start_site	-	370571	370571	-4244	14094 AN2939
1884	CONTIG51	374637	374994	1.42	9.26E-02	transcription_start_site	-	370426	370426	-4389	14095 AN2939
1884	CONTIG51	374637	374994	1.42	9.26E-02	transcription_start_site	-	369853	369853	-4962	14096 AN2939
1884	CONTIG51	374637	374994	1.42	9.26E-02	transcription_start_site	-	374318	374318	-497	14097 AN2940
1884	CONTIG51	374637	374994	1.42	9.26E-02	transcription_start_site	-	373456	373456	-1359	14098 AN2940
572	CONTIG51	384237	384591	2.35	4.61E-03	transcription_start_site	-	383275	383275	-1139	14105 AN2942
572	CONTIG51	384237	384591	2.35	4.61E-03	transcription_start_site	-	382789	382789	-1625	14106 AN2942
1534	CONTIG51	387077	387356	1.57	6.84E-02	transcription_start_site	-	383275	383275	-3941	14105 AN2942
1534	CONTIG51	387077	387356	1.57	6.84E-02	transcription_start_site	-	382789	382789	-4427	14106 AN2942
572	CONTIG51	384237	384591	2.35	4.61E-03	transcription_start_site	+	384661	384661	-247	14107 AN2943
572	CONTIG51	384237	384591	2.35	4.61E-03	transcription_start_site	+	385918	385918	-1504	14108 AN2943
68	CONTIG51	390087	390426	3.33	0.00E+00	transcription_start_site	-	388927	388927	-1329	14109 AN2944
68	CONTIG51	390087	390426	3.33	0.00E+00	transcription_start_site	-	388346	388346	-1910	14110 AN2944
1534	CONTIG51	387077	387356	1.57	6.84E-02	transcription_start_site	-	388346	388346	1129	14110 AN2944
1656	CONTIG51	388582	388866	1.52	8.01E-02	transcription_start_site	-	388927	388927	203	14109 AN2944
1656	CONTIG51	388582	388866	1.52	8.01E-02	transcription_start_site	-	388346	388346	-378	14110 AN2944
2146	CONTIG51	392337	392623	1.32	1.44E-01	transcription_start_site	-	388927	388927	-3553	14109 AN2944
2146	CONTIG51	392337	392623	1.32	1.44E-01	transcription_start_site	-	388346	388346	-4134	14110 AN2944
1436	CONTIG51	399902	400181	1.62	5.88E-02	transcription_start_site	+	401192	401192	-1150	14111 AN2946
1436	CONTIG51	399902	400181	1.62	5.88E-02	transcription_start_site	+	403589	403589	-3547	14112 AN2946
1436	CONTIG51	399902	400181	1.62	5.88E-02	transcription_start_site	+	403940	403940	-3898	14113 AN2946
119	CONTIG51	421576	421860	3.14	0.00E+00	transcription_start_site	-	416996	416996	-4722	14126 AN2951
1131	CONTIG51	419552	419989	1.81	3.08E-02	transcription_start_site	-	416996	416996	-2774	14126 AN2951
1131	CONTIG51	419552	419989	1.81	3.08E-02	transcription_start_site	+	418828	418828	942	14130 AN2952
610	CONTIG51	424671	424925	2.3	5.20E-03	transcription_start_site	-	423790	423790	-1008	14131 AN2953
1258	CONTIG51	422946	423210	1.72	4.36E-02	transcription_start_site	-	423790	423790	712	14131 AN2953
1885	CONTIG51	426906	428170	1.42	9.26E-02	transcription_start_site	-	423790	423790	-3748	14131 AN2953
945	CONTIG51	429906	430645	1.96	3.45E-03	transcription_start_site	-	429607	429607	-668	14132 AN2954
945	CONTIG51	429906	430645	1.96	3.45E-03	transcription_start_site	-	427032	427032	-3243	14133 AN2954
1188	CONTIG51	428851	429370	1.77	2.18E-02	transcription_start_site	-	429607	429607	496	14132 AN2954
1188	CONTIG51	428851	429370	1.77	2.18E-02	transcription_start_site	-	427032	427032	-2078	14133 AN2954

1885	CONTIG51	426906	428170	1.42	9.26E-02	transcription_start_site	-	427032	427032	-506	14133 AN2954
945	CONTIG51	429906	430645	1.96	3.45E-03	transcription_start_site	+	431649	431649	-1373	14134 AN2955
945	CONTIG51	429906	430645	1.96	3.45E-03	transcription_start_site	+	431919	431919	-1643	14135 AN2955
1188	CONTIG51	428851	429370	1.77	2.18E-02	transcription_start_site	+	431649	431649	-2538	14134 AN2955
1188	CONTIG51	428851	429370	1.77	2.18E-02	transcription_start_site	+	431919	431919	-2808	14135 AN2955
1885	CONTIG51	426906	428170	1.42	9.26E-02	transcription_start_site	+	431649	431649	-4111	14134 AN2955
1885	CONTIG51	426906	428170	1.42	9.26E-02	transcription_start_site	+	431919	431919	-4381	14135 AN2955
639	CONTIG51	439876	440170	2.26	6.61E-03	transcription_start_site	+	441177	441177	-1154	14136 AN2956
639	CONTIG51	439876	440170	2.26	6.61E-03	transcription_start_site	+	441432	441432	-1409	14137 AN2956
639	CONTIG51	439876	440170	2.26	6.61E-03	transcription_start_site	+	441675	441675	-1652	14138 AN2956
639	CONTIG51	439876	440170	2.26	6.61E-03	transcription_start_site	+	442536	442536	-2513	14139 AN2956
639	CONTIG51	439876	440170	2.26	6.61E-03	transcription_start_site	+	443046	443046	-3023	14140 AN2956
639	CONTIG51	439876	440170	2.26	6.61E-03	transcription_start_site	+	443451	443451	-3428	14141 AN2956
639	CONTIG51	439876	440170	2.26	6.61E-03	transcription_start_site	+	443575	443575	-3552	14142 AN2956
431	CONTIG51	445583	446079	2.55	1.80E-03	transcription_start_site	-	445561	445561	-270	14143 AN2957
431	CONTIG51	445583	446079	2.55	1.80E-03	transcription_start_site	-	444998	444998	-833	14144 AN2957
431	CONTIG51	445583	446079	2.55	1.80E-03	transcription_start_site	-	444556	444556	-1275	14145 AN2957
431	CONTIG51	445583	446079	2.55	1.80E-03	transcription_start_site	+	446410	446410	-579	14146 AN2958
431	CONTIG51	445583	446079	2.55	1.80E-03	transcription_start_site	+	446535	446535	-704	14147 AN2958
431	CONTIG51	445583	446079	2.55	1.80E-03	transcription_start_site	+	446788	446788	-957	14148 AN2958
431	CONTIG51	445583	446079	2.55	1.80E-03	transcription_start_site	+	447040	447040	-1209	14149 AN2958
431	CONTIG51	445583	446079	2.55	1.80E-03	transcription_start_site	+	447221	447221	-1390	14150 AN2958
431	CONTIG51	445583	446079	2.55	1.80E-03	transcription_start_site	+	447511	447511	-1680	14151 AN2958
431	CONTIG51	445583	446079	2.55	1.80E-03	transcription_start_site	+	447634	447634	-1803	14152 AN2958
431	CONTIG51	445583	446079	2.55	1.80E-03	transcription_start_site	+	448069	448069	-2238	14153 AN2958
2425	CONTIG51	466443	466779	1.23	1.93E-01	transcription_start_site	-	461513	461513	-5098	14164 AN2961
2425	CONTIG51	466443	466779	1.23	1.93E-01	transcription_start_site	-	466143	466143	-468	14167 AN2962
2425	CONTIG51	466443	466779	1.23	1.93E-01	transcription_start_site	-	465987	465987	-624	14168 AN2962
2425	CONTIG51	466443	466779	1.23	1.93E-01	transcription_start_site	-	465884	465884	-727	14169 AN2962
2425	CONTIG51	466443	466779	1.23	1.93E-01	transcription_start_site	-	465760	465760	-851	14170 AN2962
2425	CONTIG51	466443	466779	1.23	1.93E-01	transcription_start_site	-	465636	465636	-975	14171 AN2962
2425	CONTIG51	466443	466779	1.23	1.93E-01	transcription_start_site	-	465492	465492	-1119	14172 AN2962
2425	CONTIG51	466443	466779	1.23	1.93E-01	transcription_start_site	-	465357	465357	-1254	14173 AN2962
2425	CONTIG51	466443	466779	1.23	1.93E-01	transcription_start_site	-	465232	465232	-1379	14174 AN2962
2425	CONTIG51	466443	466779	1.23	1.93E-01	transcription_start_site	-	465056	465056	-1555	14175 AN2962
2425	CONTIG51	466443	466779	1.23	1.93E-01	transcription_start_site	-	464929	464929	-1682	14176 AN2962
2425	CONTIG51	466443	466779	1.23	1.93E-01	transcription_start_site	-	464844	464844	-1767	14177 AN2962
2425	CONTIG51	466443	466779	1.23	1.93E-01	transcription_start_site	-	464762	464762	-1849	14178 AN2962
2425	CONTIG51	466443	466779	1.23	1.93E-01	transcription_start_site	-	464680	464680	-1931	14179 AN2962
2425	CONTIG51	466443	466779	1.23	1.93E-01	transcription_start_site	-	464600	464600	-2011	14180 AN2962
2425	CONTIG51	466443	466779	1.23	1.93E-01	transcription_start_site	-	464340	464340	-2271	14181 AN2962
2425	CONTIG51	466443	466779	1.23	1.93E-01	transcription_start_site	-	464207	464207	-2404	14182 AN2962
2425	CONTIG51	466443	466779	1.23	1.93E-01	transcription_start_site	-	464032	464032	-2579	14183 AN2962
2425	CONTIG51	466443	466779	1.23	1.93E-01	transcription_start_site	-	463808	463808	-2803	14184 AN2962
2425	CONTIG51	466443	466779	1.23	1.93E-01	transcription_start_site	-	463681	463681	-2930	14185 AN2962
2425	CONTIG51	466443	466779	1.23	1.93E-01	transcription_start_site	-	466812	466812	201	14191 AN2963
2425	CONTIG51	466443	466779	1.23	1.93E-01	transcription_start_site	-	467229	467229	618	14190 AN2963
2425	CONTIG51	466443	466779	1.23	1.93E-01	transcription_start_site	-	467585	467585	974	14189 AN2963
840	CONTIG51	483093	484035	2.06	1.30E-02	transcription_start_site	-	483101	483101	-463	14198 AN2967
840	CONTIG51	483093	484035	2.06	1.30E-02	transcription_start_site	+	484222	484222	-658	14199 AN2968
840	CONTIG51	483093	484035	2.06	1.30E-02	transcription_start_site	+	484476	484476	-912	14200 AN2968
840	CONTIG51	483093	484035	2.06	1.30E-02	transcription_start_site	+	484658	484658	-1094	14201 AN2968
840	CONTIG51	483093	484035	2.06	1.30E-02	transcription_start_site	+	485052	485052	-1488	14202 AN2968
840	CONTIG51	483093	484035	2.06	1.30E-02	transcription_start_site	+	485184	485184	-1620	14203 AN2968
840	CONTIG51	483093	484035	2.06	1.30E-02	transcription_start_site	+	488168	488168	-4604	14204 AN2969
840	CONTIG51	483093	484035	2.06	1.30E-02	transcription_start_site	+	488343	488343	-4779	14205 AN2969
840	CONTIG51	483093	484035	2.06	1.30E-02	transcription_start_site	+	488474	488474	-4910	14206 AN2969

2147	CONTIG51	511070	511569	1.32	1.44E-01	transcription_start_site	-	510340	510340	-979	14239 AN2979
2147	CONTIG51	511070	511569	1.32	1.44E-01	transcription_start_site	-	510080	510080	-1239	14240 AN2979
2147	CONTIG51	511070	511569	1.32	1.44E-01	transcription_start_site	-	509953	509953	-1366	14241 AN2979
2147	CONTIG51	511070	511569	1.32	1.44E-01	transcription_start_site	-	509785	509785	-1534	14242 AN2979
2148	CONTIG51	514595	514930	1.32	1.44E-01	transcription_start_site	-	510340	510340	-4422	14239 AN2979
2148	CONTIG51	514595	514930	1.32	1.44E-01	transcription_start_site	-	510080	510080	-4682	14240 AN2979
2148	CONTIG51	514595	514930	1.32	1.44E-01	transcription_start_site	-	509953	509953	-4809	14241 AN2979
2148	CONTIG51	514595	514930	1.32	1.44E-01	transcription_start_site	-	509785	509785	-4977	14242 AN2979
2148	CONTIG51	514595	514930	1.32	1.44E-01	transcription_start_site	-	514568	514568	-194	14243 AN2980
2148	CONTIG51	514595	514930	1.32	1.44E-01	transcription_start_site	-	514419	514419	-343	14244 AN2980
2148	CONTIG51	514595	514930	1.32	1.44E-01	transcription_start_site	-	514274	514274	-488	14245 AN2980
2148	CONTIG51	514595	514930	1.32	1.44E-01	transcription_start_site	-	514161	514161	-601	14246 AN2980
2148	CONTIG51	514595	514930	1.32	1.44E-01	transcription_start_site	-	514062	514062	-700	14247 AN2980
2148	CONTIG51	514595	514930	1.32	1.44E-01	transcription_start_site	-	513884	513884	-878	14248 AN2980
2148	CONTIG51	514595	514930	1.32	1.44E-01	transcription_start_site	+	516991	516991	-2228	14249 AN2981
2148	CONTIG51	514595	514930	1.32	1.44E-01	transcription_start_site	+	517305	517305	-2542	14250 AN2981
2148	CONTIG51	514595	514930	1.32	1.44E-01	transcription_start_site	+	517440	517440	-2677	14251 AN2981
2148	CONTIG51	514595	514930	1.32	1.44E-01	transcription_start_site	+	517522	517522	-2759	14252 AN2981
2148	CONTIG51	514595	514930	1.32	1.44E-01	transcription_start_site	+	517936	517936	-3173	14253 AN2981
2148	CONTIG51	514595	514930	1.32	1.44E-01	transcription_start_site	+	518195	518195	-3432	14254 AN2981
2148	CONTIG51	514595	514930	1.32	1.44E-01	transcription_start_site	+	518332	518332	-3569	14255 AN2981
2148	CONTIG51	514595	514930	1.32	1.44E-01	transcription_start_site	+	518459	518459	-3696	14256 AN2981
2148	CONTIG51	514595	514930	1.32	1.44E-01	transcription_start_site	+	518719	518719	-3956	14257 AN2981
2148	CONTIG51	514595	514930	1.32	1.44E-01	transcription_start_site	+	519148	519148	-4385	14258 AN2981
688	CONTIG51	529507	529786	2.21	7.85E-03	transcription_start_site	-	526566	526566	-3080	14267 AN2983
688	CONTIG51	529507	529786	2.21	7.85E-03	transcription_start_site	-	526291	526291	-3355	14268 AN2983
688	CONTIG51	529507	529786	2.21	7.85E-03	transcription_start_site	-	526052	526052	-3594	14269 AN2983
688	CONTIG51	529507	529786	2.21	7.85E-03	transcription_start_site	-	525893	525893	-3753	14270 AN2983
688	CONTIG51	529507	529786	2.21	7.85E-03	transcription_start_site	-	525614	525614	-4032	14271 AN2983
1132	CONTIG51	527107	527393	1.81	3.08E-02	transcription_start_site	-	526566	526566	-684	14267 AN2983
1132	CONTIG51	527107	527393	1.81	3.08E-02	transcription_start_site	-	526291	526291	-959	14268 AN2983
1132	CONTIG51	527107	527393	1.81	3.08E-02	transcription_start_site	-	526052	526052	-1198	14269 AN2983
1132	CONTIG51	527107	527393	1.81	3.08E-02	transcription_start_site	-	525893	525893	-1357	14270 AN2983
1132	CONTIG51	527107	527393	1.81	3.08E-02	transcription_start_site	-	525614	525614	-1636	14271 AN2983
1132	CONTIG51	527107	527393	1.81	3.08E-02	transcription_start_site	-	523300	523300	-3950	14272 AN2983
688	CONTIG51	529507	529786	2.21	7.85E-03	transcription_start_site	+	529198	529198	448	14275 AN2984
688	CONTIG51	529507	529786	2.21	7.85E-03	transcription_start_site	+	528983	528983	663	14274 AN2984
688	CONTIG51	529507	529786	2.21	7.85E-03	transcription_start_site	+	528863	528863	783	14273 AN2984
1132	CONTIG51	527107	527393	1.81	3.08E-02	transcription_start_site	+	528863	528863	-1613	14273 AN2984
1132	CONTIG51	527107	527393	1.81	3.08E-02	transcription_start_site	+	528983	528983	-1733	14274 AN2984
1132	CONTIG51	527107	527393	1.81	3.08E-02	transcription_start_site	+	529198	529198	-1948	14275 AN2984
2307	CONTIG51	535052	535341	1.27	1.66E-01	transcription_start_site	-	533683	533683	-1513	14276 AN2985
2307	CONTIG51	535052	535341	1.27	1.66E-01	transcription_start_site	-	533329	533329	-1867	14277 AN2985
2307	CONTIG51	535052	535341	1.27	1.66E-01	transcription_start_site	-	533246	533246	-1950	14278 AN2985
2307	CONTIG51	535052	535341	1.27	1.66E-01	transcription_start_site	-	532881	532881	-2315	14279 AN2985
2307	CONTIG51	535052	535341	1.27	1.66E-01	transcription_start_site	-	532707	532707	-2489	14280 AN2985
244	CONTIG51	539792	540366	2.84	0.00E+00	transcription_start_site	-	535500	535500	-4579	14281 AN2986
244	CONTIG51	539792	540366	2.84	0.00E+00	transcription_start_site	-	535396	535396	-4683	14282 AN2986
244	CONTIG51	539792	540366	2.84	0.00E+00	transcription_start_site	-	535310	535310	-4769	14283 AN2986
2307	CONTIG51	535052	535341	1.27	1.66E-01	transcription_start_site	-	535310	535310	113	14283 AN2986
2307	CONTIG51	535052	535341	1.27	1.66E-01	transcription_start_site	-	535396	535396	199	14282 AN2986
2307	CONTIG51	535052	535341	1.27	1.66E-01	transcription_start_site	-	535500	535500	303	14281 AN2986
2307	CONTIG51	535052	535341	1.27	1.66E-01	transcription_start_site	-	534423	534423	-773	14284 AN2986
244	CONTIG51	539792	540366	2.84	0.00E+00	transcription_start_site	-	537233	537233	-2846	14285 AN2987
244	CONTIG51	539792	540366	2.84	0.00E+00	transcription_start_site	-	536310	536310	-3769	14286 AN2987
2307	CONTIG51	535052	535341	1.27	1.66E-01	transcription_start_site	-	536310	536310	1113	14286 AN2987
2307	CONTIG51	535052	535341	1.27	1.66E-01	transcription_start_site	+	538194	538194	-2997	14287 AN2988

2307	CONTIG51	535052	535341	1.27	1.66E-01	transcription_start_site	+	538339	538339	-3142	14288	AN2988
2307	CONTIG51	535052	535341	1.27	1.66E-01	transcription_start_site	+	538432	538432	-3235	14289	AN2988
2307	CONTIG51	535052	535341	1.27	1.66E-01	transcription_start_site	+	538536	538536	-3339	14290	AN2988
2307	CONTIG51	535052	535341	1.27	1.66E-01	transcription_start_site	+	538611	538611	-3414	14291	AN2988
2307	CONTIG51	535052	535341	1.27	1.66E-01	transcription_start_site	+	538757	538757	-3560	14292	AN2988
244	CONTIG51	539792	540366	2.84	0.00E+00	transcription_start_site	-	540182	540182	103	14296	AN2989
244	CONTIG51	539792	540366	2.84	0.00E+00	transcription_start_site	-	540341	540341	262	14295	AN2989
244	CONTIG51	539792	540366	2.84	0.00E+00	transcription_start_site	-	540484	540484	405	14294	AN2989
244	CONTIG51	539792	540366	2.84	0.00E+00	transcription_start_site	-	540618	540618	539	14293	AN2989
244	CONTIG51	539792	540366	2.84	0.00E+00	transcription_start_site	+	543979	543979	-3900	14297	AN2990
244	CONTIG51	539792	540366	2.84	0.00E+00	transcription_start_site	+	544111	544111	-4032	14298	AN2990
1527	CONTIG51	555379	555815	1.57	5.05E-02	transcription_start_site	-	553526	553526	-2071	14310	AN2994
1527	CONTIG51	555379	555815	1.57	5.05E-02	transcription_start_site	-	553346	553346	-2251	14311	AN2994
1527	CONTIG51	555379	555815	1.57	5.05E-02	transcription_start_site	-	552776	552776	-2821	14312	AN2994
1527	CONTIG51	555379	555815	1.57	5.05E-02	transcription_start_site	-	555201	555201	-396	14313	AN2995
1527	CONTIG51	555379	555815	1.57	5.05E-02	transcription_start_site	-	555065	555065	-532	14314	AN2995
1527	CONTIG51	555379	555815	1.57	5.05E-02	transcription_start_site	-	554578	554578	-1019	14315	AN2995
1527	CONTIG51	555379	555815	1.57	5.05E-02	transcription_start_site	-	554305	554305	-1292	14316	AN2995
1527	CONTIG51	555379	555815	1.57	5.05E-02	transcription_start_site	+	556138	556138	-541	14317	AN2996
1527	CONTIG51	555379	555815	1.57	5.05E-02	transcription_start_site	+	556356	556356	-759	14318	AN2996
496	CONTIG51	563029	563468	2.45	0.00E+00	transcription_start_site	-	558406	558406	-4842	14319	AN2997
496	CONTIG51	563029	563468	2.45	0.00E+00	transcription_start_site	-	558287	558287	-4961	14320	AN2997
496	CONTIG51	563029	563468	2.45	0.00E+00	transcription_start_site	+	562271	562271	977	14327	AN2998
1527	CONTIG51	555379	555815	1.57	5.05E-02	transcription_start_site	+	559037	559037	-3440	14323	AN2998
1527	CONTIG51	555379	555815	1.57	5.05E-02	transcription_start_site	+	559498	559498	-3901	14324	AN2998
1527	CONTIG51	555379	555815	1.57	5.05E-02	transcription_start_site	+	559578	559578	-3981	14325	AN2998
1527	CONTIG51	555379	555815	1.57	5.05E-02	transcription_start_site	+	559688	559688	-4091	14326	AN2998
338	CONTIG51	566044	566313	2.7	1.01E-03	transcription_start_site	+	565248	565248	930	14335	AN2999
496	CONTIG51	563029	563468	2.45	0.00E+00	transcription_start_site	+	563550	563550	-301	14328	AN2999
496	CONTIG51	563029	563468	2.45	0.00E+00	transcription_start_site	+	564142	564142	-893	14329	AN2999
496	CONTIG51	563029	563468	2.45	0.00E+00	transcription_start_site	+	564268	564268	-1019	14330	AN2999
496	CONTIG51	563029	563468	2.45	0.00E+00	transcription_start_site	+	564331	564331	-1082	14331	AN2999
496	CONTIG51	563029	563468	2.45	0.00E+00	transcription_start_site	+	564416	564416	-1167	14332	AN2999
496	CONTIG51	563029	563468	2.45	0.00E+00	transcription_start_site	+	564608	564608	-1359	14333	AN2999
496	CONTIG51	563029	563468	2.45	0.00E+00	transcription_start_site	+	564703	564703	-1454	14334	AN2999
496	CONTIG51	563029	563468	2.45	0.00E+00	transcription_start_site	+	565248	565248	-1999	14335	AN2999
338	CONTIG51	566044	566313	2.7	1.01E-03	transcription_start_site	+	568306	568306	-2127	14336	AN3000
496	CONTIG51	563029	563468	2.45	0.00E+00	transcription_start_site	+	568306	568306	-5057	14336	AN3000
689	CONTIG51	569029	569303	2.21	7.85E-03	transcription_start_site	+	568306	568306	860	14336	AN3000
338	CONTIG51	566044	566313	2.7	1.01E-03	transcription_start_site	+	570560	570560	-4381	14337	AN3001
689	CONTIG51	569029	569303	2.21	7.85E-03	transcription_start_site	+	570560	570560	-1394	14337	AN3001
787	CONTIG51	569704	570808	2.11	2.24E-03	transcription_start_site	+	570560	570560	-304	14337	AN3001
1647	CONTIG51	577811	578245	1.52	6.16E-02	transcription_start_site	-	575163	575163	-2865	14338	AN3002
1647	CONTIG51	577811	578245	1.52	6.16E-02	transcription_start_site	-	574968	574968	-3060	14339	AN3002
787	CONTIG51	569704	570808	2.11	2.24E-03	transcription_start_site	+	575553	575553	-5297	14340	AN3003
787	CONTIG51	569704	570808	2.11	2.24E-03	transcription_start_site	+	575752	575752	-5496	14341	AN3003
1647	CONTIG51	577811	578245	1.52	6.16E-02	transcription_start_site	+	577606	577606	422	14342	AN3004
1647	CONTIG51	577811	578245	1.52	6.16E-02	transcription_start_site	+	578867	578867	-839	14343	AN3004
1890	CONTIG51	582301	582595	1.42	1.08E-01	transcription_start_site	-	582020	582020	-428	14344	AN3005
1890	CONTIG51	582301	582595	1.42	1.08E-01	transcription_start_site	-	581858	581858	-590	14345	AN3005
1890	CONTIG51	582301	582595	1.42	1.08E-01	transcription_start_site	-	580256	580256	-2192	14346	AN3005
1890	CONTIG51	582301	582595	1.42	1.08E-01	transcription_start_site	-	583419	583419	971	14348	AN3006
1890	CONTIG51	582301	582595	1.42	1.08E-01	transcription_start_site	-	583587	583587	1139	14347	AN3006
1890	CONTIG51	582301	582595	1.42	1.08E-01	transcription_start_site	+	587469	587469	-5021	14356	AN3009
460	CONTIG51	615388	615729	2.5	2.22E-03	transcription_start_site	-	613246	613246	-2312	14393	AN3019
460	CONTIG51	615388	615729	2.5	2.22E-03	transcription_start_site	-	612619	612619	-2939	14394	AN3019
460	CONTIG51	615388	615729	2.5	2.22E-03	transcription_start_site	-	615520	615520	-38	14398	AN3020

460	CONTIG51	615388	615729	2.5	2.22E-03	transcription_start_site	-	615130	615130	-428	14399 AN3020
460	CONTIG51	615388	615729	2.5	2.22E-03	transcription_start_site	-	616159	616159	600	14397 AN3020
460	CONTIG51	615388	615729	2.5	2.22E-03	transcription_start_site	+	619952	619952	-4393	14400 AN3021
460	CONTIG51	615388	615729	2.5	2.22E-03	transcription_start_site	+	620646	620646	-5087	14401 AN3021
1784	CONTIG51	623556	623923	1.47	9.24E-02	transcription_start_site	+	622667	622667	1072	14406 AN3021
1784	CONTIG51	623556	623923	1.47	9.24E-02	transcription_start_site	+	623986	623986	-246	14407 AN3022
1784	CONTIG51	623556	623923	1.47	9.24E-02	transcription_start_site	+	624180	624180	-440	14408 AN3022
1784	CONTIG51	623556	623923	1.47	9.24E-02	transcription_start_site	+	624396	624396	-656	14409 AN3022
1784	CONTIG51	623556	623923	1.47	9.24E-02	transcription_start_site	+	625196	625196	-1456	14410 AN3022
2005	CONTIG51	645766	646645	1.37	8.00E-02	transcription_start_site	-	646809	646809	603	14448 AN3032
2005	CONTIG51	645766	646645	1.37	8.00E-02	transcription_start_site	-	647020	647020	814	14447 AN3032
2149	CONTIG51	648306	648575	1.32	1.44E-01	transcription_start_site	-	647020	647020	-1420	14447 AN3032
2149	CONTIG51	648306	648575	1.32	1.44E-01	transcription_start_site	-	646809	646809	-1631	14448 AN3032
2005	CONTIG51	645766	646645	1.37	8.00E-02	transcription_start_site	+	648693	648693	-2487	14449 AN3033
2005	CONTIG51	645766	646645	1.37	8.00E-02	transcription_start_site	+	648980	648980	-2774	14450 AN3033
2005	CONTIG51	645766	646645	1.37	8.00E-02	transcription_start_site	+	649233	649233	-3027	14451 AN3033
2149	CONTIG51	648306	648575	1.32	1.44E-01	transcription_start_site	+	648693	648693	-252	14449 AN3033
2149	CONTIG51	648306	648575	1.32	1.44E-01	transcription_start_site	+	648980	648980	-539	14450 AN3033
2149	CONTIG51	648306	648575	1.32	1.44E-01	transcription_start_site	+	649233	649233	-792	14451 AN3033
2149	CONTIG51	648306	648575	1.32	1.44E-01	transcription_start_site	+	653535	653535	-5094	14457 AN3035
2308	CONTIG51	681083	681443	1.27	1.66E-01	transcription_start_site	-	681295	681295	32	14487 AN3044
2308	CONTIG51	681083	681443	1.27	1.66E-01	transcription_start_site	-	680432	680432	-831	14488 AN3044
2308	CONTIG51	681083	681443	1.27	1.66E-01	transcription_start_site	-	680191	680191	-1072	14489 AN3044
2308	CONTIG51	681083	681443	1.27	1.66E-01	transcription_start_site	-	680084	680084	-1179	14490 AN3044
2308	CONTIG51	681083	681443	1.27	1.66E-01	transcription_start_site	-	679953	679953	-1310	14491 AN3044
2308	CONTIG51	681083	681443	1.27	1.66E-01	transcription_start_site	-	679687	679687	-1576	14492 AN3044
2308	CONTIG51	681083	681443	1.27	1.66E-01	transcription_start_site	+	685861	685861	-4598	14497 AN3046
1785	CONTIG51	693770	694044	1.47	9.24E-02	transcription_start_site	+	693998	693998	-91	14505 AN3049
1785	CONTIG51	693770	694044	1.47	9.24E-02	transcription_start_site	+	694327	694327	-420	14506 AN3049
1785	CONTIG51	693770	694044	1.47	9.24E-02	transcription_start_site	+	694647	694647	-740	14507 AN3049
1785	CONTIG51	693770	694044	1.47	9.24E-02	transcription_start_site	+	695188	695188	-1281	14508 AN3049
1259	CONTIG51	712365	712629	1.72	4.36E-02	transcription_start_site	-	707495	707495	-5002	14521 AN3052
1259	CONTIG51	712365	712629	1.72	4.36E-02	transcription_start_site	-	711998	711998	-499	14522 AN3053
1259	CONTIG51	712365	712629	1.72	4.36E-02	transcription_start_site	-	711150	711150	-1347	14523 AN3053
2309	CONTIG51	717680	717964	1.27	1.66E-01	transcription_start_site	-	717053	717053	-769	14524 AN3054
2309	CONTIG51	717680	717964	1.27	1.66E-01	transcription_start_site	-	716742	716742	-1080	14525 AN3054
2309	CONTIG51	717680	717964	1.27	1.66E-01	transcription_start_site	-	716572	716572	-1250	14526 AN3054
2309	CONTIG51	717680	717964	1.27	1.66E-01	transcription_start_site	-	716378	716378	-1444	14527 AN3054
2309	CONTIG51	717680	717964	1.27	1.66E-01	transcription_start_site	-	716209	716209	-1613	14528 AN3054
2309	CONTIG51	717680	717964	1.27	1.66E-01	transcription_start_site	-	715647	715647	-2175	14529 AN3054
2309	CONTIG51	717680	717964	1.27	1.66E-01	transcription_start_site	-	715507	715507	-2315	14530 AN3054
2309	CONTIG51	717680	717964	1.27	1.66E-01	transcription_start_site	-	714907	714907	-2915	14531 AN3054
2309	CONTIG51	717680	717964	1.27	1.66E-01	transcription_start_site	-	714727	714727	-3095	14532 AN3054
2309	CONTIG51	717680	717964	1.27	1.66E-01	transcription_start_site	-	714603	714603	-3219	14533 AN3054
2309	CONTIG51	717680	717964	1.27	1.66E-01	transcription_start_site	-	714443	714443	-3379	14534 AN3054
1259	CONTIG51	712365	712629	1.72	4.36E-02	transcription_start_site	+	717365	717365	-4868	14535 AN3055
2309	CONTIG51	717680	717964	1.27	1.66E-01	transcription_start_site	+	717758	717758	64	14536 AN3055
2309	CONTIG51	717680	717964	1.27	1.66E-01	transcription_start_site	+	717365	717365	457	14535 AN3055
2309	CONTIG51	717680	717964	1.27	1.66E-01	transcription_start_site	+	720581	720581	-2759	14539 AN3057
2309	CONTIG51	717680	717964	1.27	1.66E-01	transcription_start_site	+	720789	720789	-2967	14540 AN3057
1336	CONTIG51	725790	726054	1.67	5.22E-02	transcription_start_site	-	724414	724414	-1508	14541 AN3058
1336	CONTIG51	725790	726054	1.67	5.22E-02	transcription_start_site	-	724313	724313	-1609	14542 AN3058
1336	CONTIG51	725790	726054	1.67	5.22E-02	transcription_start_site	-	724166	724166	-1756	14543 AN3058
1336	CONTIG51	725790	726054	1.67	5.22E-02	transcription_start_site	-	723745	723745	-2177	14544 AN3058
1336	CONTIG51	725790	726054	1.67	5.22E-02	transcription_start_site	+	726267	726267	-345	14545 AN3059
1336	CONTIG51	725790	726054	1.67	5.22E-02	transcription_start_site	+	726366	726366	-444	14546 AN3059
1336	CONTIG51	725790	726054	1.67	5.22E-02	transcription_start_site	+	727748	727748	-1826	14547 AN3059

1336	CONTIG51	725790	726054	1.67	5.22E-02	transcription_start_site	+	729460	729460	-3538	14552	AN3061
1336	CONTIG51	725790	726054	1.67	5.22E-02	transcription_start_site	+	729832	729832	-3910	14553	AN3061
1336	CONTIG51	725790	726054	1.67	5.22E-02	transcription_start_site	+	730217	730217	-4295	14554	AN3061
1336	CONTIG51	725790	726054	1.67	5.22E-02	transcription_start_site	+	730720	730720	-4798	14555	AN3061
2020	CONTIG51	753760	754114	1.37	1.25E-01	transcription_start_site	-	751996	751996	-1941	14574	AN3067
2020	CONTIG51	753760	754114	1.37	1.25E-01	transcription_start_site	-	751295	751295	-2642	14575	AN3067
2020	CONTIG51	753760	754114	1.37	1.25E-01	transcription_start_site	-	751152	751152	-2785	14576	AN3067
2020	CONTIG51	753760	754114	1.37	1.25E-01	transcription_start_site	+	752813	752813	1124	14578	AN3068
2020	CONTIG51	753760	754114	1.37	1.25E-01	transcription_start_site	+	756649	756649	-2712	14579	AN3070
2020	CONTIG51	753760	754114	1.37	1.25E-01	transcription_start_site	+	756740	756740	-2803	14580	AN3070
2020	CONTIG51	753760	754114	1.37	1.25E-01	transcription_start_site	+	756891	756891	-2954	14581	AN3070
2020	CONTIG51	753760	754114	1.37	1.25E-01	transcription_start_site	+	756968	756968	-3031	14582	AN3070
2020	CONTIG51	753760	754114	1.37	1.25E-01	transcription_start_site	+	757061	757061	-3124	14583	AN3070
2020	CONTIG51	753760	754114	1.37	1.25E-01	transcription_start_site	+	757135	757135	-3198	14584	AN3070
2020	CONTIG51	753760	754114	1.37	1.25E-01	transcription_start_site	+	757359	757359	-3422	14585	AN3070
310	CONTIG51	768230	769426	2.75	0.00E+00	transcription_start_site	+	770027	770027	-1199	14592	AN3074
310	CONTIG51	768230	769426	2.75	0.00E+00	transcription_start_site	+	770405	770405	-1577	14593	AN3074
310	CONTIG51	768230	769426	2.75	0.00E+00	transcription_start_site	+	770742	770742	-1914	14594	AN3074
432	CONTIG51	770110	770394	2.55	1.80E-03	transcription_start_site	+	770405	770405	-153	14593	AN3074
432	CONTIG51	770110	770394	2.55	1.80E-03	transcription_start_site	+	770027	770027	225	14592	AN3074
432	CONTIG51	770110	770394	2.55	1.80E-03	transcription_start_site	+	770742	770742	-490	14594	AN3074
1430	CONTIG51	771020	771669	1.62	3.99E-02	transcription_start_site	+	770742	770742	602	14594	AN3074
1430	CONTIG51	771020	771669	1.62	3.99E-02	transcription_start_site	+	770405	770405	939	14593	AN3074
1430	CONTIG51	771020	771669	1.62	3.99E-02	transcription_start_site	+	770027	770027	1317	14592	AN3074
611	CONTIG51	779490	779829	2.3	5.20E-03	transcription_start_site	-	777501	777501	-2158	14595	AN3075
611	CONTIG51	779490	779829	2.3	5.20E-03	transcription_start_site	-	776835	776835	-2824	14596	AN3075
611	CONTIG51	779490	779829	2.3	5.20E-03	transcription_start_site	-	776702	776702	-2957	14597	AN3075
741	CONTIG51	776707	777586	2.16	0.00E+00	transcription_start_site	-	776835	776835	-311	14596	AN3075
741	CONTIG51	776707	777586	2.16	0.00E+00	transcription_start_site	-	777501	777501	354	14595	AN3075
741	CONTIG51	776707	777586	2.16	0.00E+00	transcription_start_site	-	776702	776702	-444	14597	AN3075
1337	CONTIG51	776117	776676	1.67	5.22E-02	transcription_start_site	-	776702	776702	305	14597	AN3075
1337	CONTIG51	776117	776676	1.67	5.22E-02	transcription_start_site	-	776835	776835	438	14596	AN3075
1337	CONTIG51	776117	776676	1.67	5.22E-02	transcription_start_site	-	777501	777501	1104	14595	AN3075
611	CONTIG51	779490	779829	2.3	5.20E-03	transcription_start_site	+	779937	779937	-277	14598	AN3076
611	CONTIG51	779490	779829	2.3	5.20E-03	transcription_start_site	+	780455	780455	-795	14599	AN3076
611	CONTIG51	779490	779829	2.3	5.20E-03	transcription_start_site	+	780754	780754	-1094	14600	AN3076
741	CONTIG51	776707	777586	2.16	0.00E+00	transcription_start_site	+	779937	779937	-2790	14598	AN3076
741	CONTIG51	776707	777586	2.16	0.00E+00	transcription_start_site	+	780455	780455	-3308	14599	AN3076
741	CONTIG51	776707	777586	2.16	0.00E+00	transcription_start_site	+	780754	780754	-3607	14600	AN3076
1337	CONTIG51	776117	776676	1.67	5.22E-02	transcription_start_site	+	779937	779937	-3540	14598	AN3076
1337	CONTIG51	776117	776676	1.67	5.22E-02	transcription_start_site	+	780455	780455	-4058	14599	AN3076
1337	CONTIG51	776117	776676	1.67	5.22E-02	transcription_start_site	+	780754	780754	-4357	14600	AN3076
1	CONTIG51	792233	792657	4.02	0.00E+00	transcription_start_site	-	787945	787945	-4500	14601	AN3077
1	CONTIG51	792233	792657	4.02	0.00E+00	transcription_start_site	-	787652	787652	-4793	14602	AN3077
311	CONTIG51	791343	791985	2.75	9.51E-04	transcription_start_site	-	787945	787945	-3719	14601	AN3077
311	CONTIG51	791343	791985	2.75	9.51E-04	transcription_start_site	-	787652	787652	-4012	14602	AN3077
339	CONTIG51	789753	790178	2.7	1.01E-03	transcription_start_site	-	787945	787945	-2020	14601	AN3077
339	CONTIG51	789753	790178	2.7	1.01E-03	transcription_start_site	-	787652	787652	-2313	14602	AN3077
749	CONTIG51	790433	791085	2.16	8.81E-03	transcription_start_site	-	787945	787945	-2814	14601	AN3077
749	CONTIG51	790433	791085	2.16	8.81E-03	transcription_start_site	-	787652	787652	-3107	14602	AN3077
339	CONTIG51	789753	790178	2.7	1.01E-03	transcription_start_site	+	789676	789676	289	14604	AN3078
339	CONTIG51	789753	790178	2.7	1.01E-03	transcription_start_site	+	789496	789496	469	14603	AN3078
749	CONTIG51	790433	791085	2.16	8.81E-03	transcription_start_site	+	789676	789676	1083	14604	AN3078
749	CONTIG51	790433	791085	2.16	8.81E-03	transcription_start_site	+	789496	789496	1263	14603	AN3078
1	CONTIG51	792233	792657	4.02	0.00E+00	transcription_start_site	-	791804	791804	-641	14605	AN3079
311	CONTIG51	791343	791985	2.75	9.51E-04	transcription_start_site	-	791804	791804	140	14605	AN3079
749	CONTIG51	790433	791085	2.16	8.81E-03	transcription_start_site	-	791804	791804	1045	14605	AN3079

2426	CONTIG51	795843	796177	1.23	1.93E-01	transcription_start_site	-	791804	791804	-4206	14605 AN3079
1	CONTIG51	792233	792657	4.02	0.00E+00	transcription_start_site	-	793592	793592	1147	14610 AN3080
2426	CONTIG51	795843	796177	1.23	1.93E-01	transcription_start_site	-	796246	796246	236	14606 AN3080
2426	CONTIG51	795843	796177	1.23	1.93E-01	transcription_start_site	-	794850	794850	-1160	14607 AN3080
2426	CONTIG51	795843	796177	1.23	1.93E-01	transcription_start_site	-	794704	794704	-1306	14608 AN3080
2426	CONTIG51	795843	796177	1.23	1.93E-01	transcription_start_site	-	793833	793833	-2177	14609 AN3080
2426	CONTIG51	795843	796177	1.23	1.93E-01	transcription_start_site	-	793592	793592	-2418	14610 AN3080
1	CONTIG51	792233	792657	4.02	0.00E+00	transcription_start_site	+	796662	796662	-4217	14611 AN3081
1	CONTIG51	792233	792657	4.02	0.00E+00	transcription_start_site	+	796769	796769	-4324	14612 AN3081
1	CONTIG51	792233	792657	4.02	0.00E+00	transcription_start_site	+	797218	797218	-4773	14613 AN3081
1	CONTIG51	792233	792657	4.02	0.00E+00	transcription_start_site	+	797430	797430	-4985	14614 AN3081
1	CONTIG51	792233	792657	4.02	0.00E+00	transcription_start_site	+	797614	797614	-5169	14615 AN3081
311	CONTIG51	791343	791985	2.75	9.51E-04	transcription_start_site	+	796662	796662	-4998	14611 AN3081
311	CONTIG51	791343	791985	2.75	9.51E-04	transcription_start_site	+	796769	796769	-5105	14612 AN3081
2426	CONTIG51	795843	796177	1.23	1.93E-01	transcription_start_site	+	796662	796662	-652	14611 AN3081
2426	CONTIG51	795843	796177	1.23	1.93E-01	transcription_start_site	+	796769	796769	-759	14612 AN3081
2426	CONTIG51	795843	796177	1.23	1.93E-01	transcription_start_site	+	797218	797218	-1208	14613 AN3081
2426	CONTIG51	795843	796177	1.23	1.93E-01	transcription_start_site	+	797430	797430	-1420	14614 AN3081
2426	CONTIG51	795843	796177	1.23	1.93E-01	transcription_start_site	+	797614	797614	-1604	14615 AN3081
2426	CONTIG51	795843	796177	1.23	1.93E-01	transcription_start_site	+	797877	797877	-1867	14616 AN3081
2426	CONTIG51	795843	796177	1.23	1.93E-01	transcription_start_site	+	798390	798390	-2380	14617 AN3081
2426	CONTIG51	795843	796177	1.23	1.93E-01	transcription_start_site	+	798528	798528	-2518	14618 AN3081
340	CONTIG51	812493	812842	2.7	1.01E-03	transcription_start_site	-	811986	811986	-681	14626 AN3085
340	CONTIG51	812493	812842	2.7	1.01E-03	transcription_start_site	-	811531	811531	-1136	14627 AN3085
841	CONTIG51	818183	818527	2.06	1.30E-02	transcription_start_site	-	816393	816393	-1962	14628 AN3086
841	CONTIG51	818183	818527	2.06	1.30E-02	transcription_start_site	+	819112	819112	-757	14629 AN3087
841	CONTIG51	818183	818527	2.06	1.30E-02	transcription_start_site	+	819521	819521	-1166	14630 AN3087
841	CONTIG51	818183	818527	2.06	1.30E-02	transcription_start_site	+	821284	821284	-2929	14631 AN3088
841	CONTIG51	818183	818527	2.06	1.30E-02	transcription_start_site	+	821419	821419	-3064	14632 AN3088
841	CONTIG51	818183	818527	2.06	1.30E-02	transcription_start_site	+	821550	821550	-3195	14633 AN3088
841	CONTIG51	818183	818527	2.06	1.30E-02	transcription_start_site	+	821733	821733	-3378	14634 AN3088
841	CONTIG51	818183	818527	2.06	1.30E-02	transcription_start_site	+	821944	821944	-3589	14635 AN3088
841	CONTIG51	818183	818527	2.06	1.30E-02	transcription_start_site	+	822114	822114	-3759	14636 AN3088
120	CONTIG51	876458	876804	3.14	0.00E+00	transcription_start_site	-	874661	874661	-1970	14682 AN3101
120	CONTIG51	876458	876804	3.14	0.00E+00	transcription_start_site	+	877650	877650	-1019	14685 AN3102
120	CONTIG51	876458	876804	3.14	0.00E+00	transcription_start_site	+	878107	878107	-1476	14686 AN3102
120	CONTIG51	876458	876804	3.14	0.00E+00	transcription_start_site	+	878565	878565	-1934	14687 AN3102
120	CONTIG51	876458	876804	3.14	0.00E+00	transcription_start_site	+	881194	881194	-4563	14688 AN3102
1333	CONTIG51	907577	907941	1.67	3.33E-02	transcription_start_site	-	904708	904708	-3051	14703 AN3109
1333	CONTIG51	907577	907941	1.67	3.33E-02	transcription_start_site	-	904662	904662	-3097	14704 AN3109
1333	CONTIG51	907577	907941	1.67	3.33E-02	transcription_start_site	-	908076	908076	317	14705 AN3110
1333	CONTIG51	907577	907941	1.67	3.33E-02	transcription_start_site	-	905921	905921	-1838	14706 AN3110
1333	CONTIG51	907577	907941	1.67	3.33E-02	transcription_start_site	+	909120	909120	-1361	14707 AN3111
1333	CONTIG51	907577	907941	1.67	3.33E-02	transcription_start_site	+	909881	909881	-2122	14708 AN3111
1333	CONTIG51	907577	907941	1.67	3.33E-02	transcription_start_site	+	910269	910269	-2510	14709 AN3111
1333	CONTIG51	907577	907941	1.67	3.33E-02	transcription_start_site	+	910448	910448	-2689	14710 AN3111
1333	CONTIG51	907577	907941	1.67	3.33E-02	transcription_start_site	+	910746	910746	-2987	14711 AN3111
953	CONTIG51	915695	916414	1.96	1.84E-02	transcription_start_site	-	913774	913774	-2280	14712 AN3112
953	CONTIG51	915695	916414	1.96	1.84E-02	transcription_start_site	-	913627	913627	-2427	14713 AN3112
953	CONTIG51	915695	916414	1.96	1.84E-02	transcription_start_site	-	913503	913503	-2551	14714 AN3112
953	CONTIG51	915695	916414	1.96	1.84E-02	transcription_start_site	-	913365	913365	-2689	14715 AN3112
953	CONTIG51	915695	916414	1.96	1.84E-02	transcription_start_site	-	912948	912948	-3106	14716 AN3112
953	CONTIG51	915695	916414	1.96	1.84E-02	transcription_start_site	-	912470	912470	-3584	14717 AN3112
953	CONTIG51	915695	916414	1.96	1.84E-02	transcription_start_site	+	916945	916945	-890	14718 AN3113
953	CONTIG51	915695	916414	1.96	1.84E-02	transcription_start_site	+	917140	917140	-1085	14719 AN3113
953	CONTIG51	915695	916414	1.96	1.84E-02	transcription_start_site	+	917280	917280	-1225	14720 AN3113
953	CONTIG51	915695	916414	1.96	1.84E-02	transcription_start_site	+	917562	917562	-1507	14721 AN3113

953	CONTIG51	915695	916414	1.96	1.84E-02	transcription_start_site	+	918022	918022	-1967	14722	AN3113
403	CONTIG51	919068	919402	2.6	1.54E-03	transcription_start_site	+	919849	919849	-614	14723	AN3114
953	CONTIG51	915695	916414	1.96	1.84E-02	transcription_start_site	+	919849	919849	-3794	14723	AN3114
1535	CONTIG51	919593	920227	1.57	6.84E-02	transcription_start_site	+	919849	919849	61	14723	AN3114
1058	CONTIG51	939544	940268	1.86	1.00E-02	transcription_start_site	-	934928	934928	-4978	14738	AN3118
1058	CONTIG51	939544	940268	1.86	1.00E-02	transcription_start_site	-	934668	934668	-5238	14739	AN3118
1536	CONTIG51	936544	936883	1.57	6.84E-02	transcription_start_site	-	934928	934928	-1785	14738	AN3118
1536	CONTIG51	936544	936883	1.57	6.84E-02	transcription_start_site	-	934668	934668	-2045	14739	AN3118
1536	CONTIG51	936544	936883	1.57	6.84E-02	transcription_start_site	-	934491	934491	-2222	14740	AN3118
1536	CONTIG51	936544	936883	1.57	6.84E-02	transcription_start_site	-	934268	934268	-2445	14741	AN3118
1536	CONTIG51	936544	936883	1.57	6.84E-02	transcription_start_site	-	933814	933814	-2899	14742	AN3118
1058	CONTIG51	939544	940268	1.86	1.00E-02	transcription_start_site	-	936207	936207	-3699	14743	AN3119
1536	CONTIG51	936544	936883	1.57	6.84E-02	transcription_start_site	-	936207	936207	-506	14743	AN3119
1058	CONTIG51	939544	940268	1.86	1.00E-02	transcription_start_site	+	938556	938556	1350	14747	AN3120
1536	CONTIG51	936544	936883	1.57	6.84E-02	transcription_start_site	+	937003	937003	-289	14744	AN3120
1536	CONTIG51	936544	936883	1.57	6.84E-02	transcription_start_site	+	937200	937200	-486	14745	AN3120
1536	CONTIG51	936544	936883	1.57	6.84E-02	transcription_start_site	+	937450	937450	-736	14746	AN3120
1536	CONTIG51	936544	936883	1.57	6.84E-02	transcription_start_site	+	938556	938556	-1842	14747	AN3120
1058	CONTIG51	939544	940268	1.86	1.00E-02	transcription_start_site	+	940604	940604	-698	14748	AN3121
1058	CONTIG51	939544	940268	1.86	1.00E-02	transcription_start_site	+	941011	941011	-1105	14749	AN3121
1058	CONTIG51	939544	940268	1.86	1.00E-02	transcription_start_site	+	941193	941193	-1287	14750	AN3121
1536	CONTIG51	936544	936883	1.57	6.84E-02	transcription_start_site	+	940604	940604	-3890	14748	AN3121
1536	CONTIG51	936544	936883	1.57	6.84E-02	transcription_start_site	+	941011	941011	-4297	14749	AN3121
1536	CONTIG51	936544	936883	1.57	6.84E-02	transcription_start_site	+	941193	941193	-4479	14750	AN3121
108	CONTIG51	954903	955192	3.19	0.00E+00	transcription_start_site	-	954808	954808	-239	14762	AN3124
108	CONTIG51	954903	955192	3.19	0.00E+00	transcription_start_site	-	954405	954405	-642	14763	AN3124
108	CONTIG51	954903	955192	3.19	0.00E+00	transcription_start_site	-	953344	953344	-1703	14764	AN3124
108	CONTIG51	954903	955192	3.19	0.00E+00	transcription_start_site	-	953166	953166	-1881	14765	AN3124
108	CONTIG51	954903	955192	3.19	0.00E+00	transcription_start_site	-	952597	952597	-2450	14766	AN3124
108	CONTIG51	954903	955192	3.19	0.00E+00	transcription_start_site	-	952480	952480	-2567	14767	AN3124
108	CONTIG51	954903	955192	3.19	0.00E+00	transcription_start_site	+	955500	955500	-452	14768	AN3125
108	CONTIG51	954903	955192	3.19	0.00E+00	transcription_start_site	+	955571	955571	-523	14769	AN3125
108	CONTIG51	954903	955192	3.19	0.00E+00	transcription_start_site	+	955717	955717	-669	14770	AN3125
108	CONTIG51	954903	955192	3.19	0.00E+00	transcription_start_site	+	955915	955915	-867	14771	AN3125
154	CONTIG51	967728	968092	3.04	2.67E-04	transcription_start_site	+	969035	969035	-1125	14775	AN3129
154	CONTIG51	967728	968092	3.04	2.67E-04	transcription_start_site	+	969389	969389	-1479	14776	AN3129
154	CONTIG51	967728	968092	3.04	2.67E-04	transcription_start_site	+	969731	969731	-1821	14777	AN3129
154	CONTIG51	967728	968092	3.04	2.67E-04	transcription_start_site	+	970605	970605	-2695	14778	AN3129
154	CONTIG51	967728	968092	3.04	2.67E-04	transcription_start_site	+	970739	970739	-2829	14779	AN3129
531	CONTIG51	966678	967112	2.4	4.46E-04	transcription_start_site	+	969035	969035	-2140	14775	AN3129
531	CONTIG51	966678	967112	2.4	4.46E-04	transcription_start_site	+	969389	969389	-2494	14776	AN3129
531	CONTIG51	966678	967112	2.4	4.46E-04	transcription_start_site	+	969731	969731	-2836	14777	AN3129
531	CONTIG51	966678	967112	2.4	4.46E-04	transcription_start_site	+	970605	970605	-3710	14778	AN3129
531	CONTIG51	966678	967112	2.4	4.46E-04	transcription_start_site	+	970739	970739	-3844	14779	AN3129
684	CONTIG51	964063	964627	2.21	3.31E-03	transcription_start_site	+	969035	969035	-4690	14775	AN3129
684	CONTIG51	964063	964627	2.21	3.31E-03	transcription_start_site	+	969389	969389	-5044	14776	AN3129
792	CONTIG51	982667	983151	2.11	1.11E-02	transcription_start_site	+	982021	982021	888	14794	AN3134
792	CONTIG51	982667	983151	2.11	1.11E-02	transcription_start_site	-	982852	982852	-57	14795	AN3135
792	CONTIG51	982667	983151	2.11	1.11E-02	transcription_start_site	+	983950	983950	-1041	14796	AN3136
792	CONTIG51	982667	983151	2.11	1.11E-02	transcription_start_site	+	984139	984139	-1230	14797	AN3136
792	CONTIG51	982667	983151	2.11	1.11E-02	transcription_start_site	+	988106	988106	-5197	14798	AN3137
1133	CONTIG51	994742	995011	1.81	3.08E-02	transcription_start_site	-	994744	994744	-132	14804	AN3139
1133	CONTIG51	994742	995011	1.81	3.08E-02	transcription_start_site	-	994308	994308	-568	14805	AN3139
1133	CONTIG51	994742	995011	1.81	3.08E-02	transcription_start_site	-	994103	994103	-773	14806	AN3139
1636	CONTIG51	998494	999369	1.52	4.45E-02	transcription_start_site	-	994744	994744	-4187	14804	AN3139
1636	CONTIG51	998494	999369	1.52	4.45E-02	transcription_start_site	-	994308	994308	-4623	14805	AN3139
1636	CONTIG51	998494	999369	1.52	4.45E-02	transcription_start_site	-	994103	994103	-4828	14806	AN3139

1133	CONTIG51	994742	995011	1.81	3.08E-02	transcription_start_site	+	995165	995165	-288	14807	AN3140
1133	CONTIG51	994742	995011	1.81	3.08E-02	transcription_start_site	+	995780	995780	-903	14808	AN3140
1133	CONTIG51	994742	995011	1.81	3.08E-02	transcription_start_site	+	996436	996436	-1559	14809	AN3140
1260	CONTIG51	1004630	1005589	1.72	4.36E-02	transcription_start_site	-	1000937	1000937	-4172	14810	AN3141
1260	CONTIG51	1004630	1005589	1.72	4.36E-02	transcription_start_site	-	1000815	1000815	-4294	14811	AN3141
1260	CONTIG51	1004630	1005589	1.72	4.36E-02	transcription_start_site	-	1000565	1000565	-4544	14812	AN3141
1260	CONTIG51	1004630	1005589	1.72	4.36E-02	transcription_start_site	-	1004091	1004091	-1018	14813	AN3142
1260	CONTIG51	1004630	1005589	1.72	4.36E-02	transcription_start_site	-	1003122	1003122	-1987	14814	AN3142
500	CONTIG51	1013263	1013605	2.45	3.03E-03	transcription_start_site	-	1010983	1010983	-2451	14815	AN3143
573	CONTIG51	1012218	1012705	2.35	4.61E-03	transcription_start_site	-	1010983	1010983	-1478	14815	AN3143
574	CONTIG51	1032303	1032577	2.35	4.61E-03	transcription_start_site	-	1033533	1033533	1093	14826	AN3147
574	CONTIG51	1032303	1032577	2.35	4.61E-03	transcription_start_site	+	1034479	1034479	-2039	14827	AN3148
574	CONTIG51	1032303	1032577	2.35	4.61E-03	transcription_start_site	+	1034610	1034610	-2170	14828	AN3148
574	CONTIG51	1032303	1032577	2.35	4.61E-03	transcription_start_site	+	1037127	1037127	-4687	14829	AN3148
1528	CONTIG51	1030888	1031977	1.57	5.05E-02	transcription_start_site	+	1034479	1034479	-3046	14827	AN3148
1528	CONTIG51	1030888	1031977	1.57	5.05E-02	transcription_start_site	+	1034610	1034610	-3177	14828	AN3148
0	CONTIG51	1054142	1054561	4.07	0.00E+00	transcription_start_site	+	1054842	1054842	-490	14840	AN3152
0	CONTIG51	1054142	1054561	4.07	0.00E+00	transcription_start_site	+	1054948	1054948	-596	14841	AN3152
0	CONTIG51	1054142	1054561	4.07	0.00E+00	transcription_start_site	+	1055811	1055811	-1459	14842	AN3152
0	CONTIG51	1054142	1054561	4.07	0.00E+00	transcription_start_site	+	1056197	1056197	-1845	14843	AN3152
3	CONTIG51	1056452	1058008	3.97	0.00E+00	transcription_start_site	+	1056197	1056197	1033	14843	AN3152
3	CONTIG51	1056452	1058008	3.97	0.00E+00	transcription_start_site	+	1055811	1055811	1419	14842	AN3152
1338	CONTIG51	1052120	1052544	1.67	5.22E-02	transcription_start_site	+	1054842	1054842	-2510	14840	AN3152
1338	CONTIG51	1052120	1052544	1.67	5.22E-02	transcription_start_site	+	1054948	1054948	-2616	14841	AN3152
1338	CONTIG51	1052120	1052544	1.67	5.22E-02	transcription_start_site	+	1055811	1055811	-3479	14842	AN3152
1338	CONTIG51	1052120	1052544	1.67	5.22E-02	transcription_start_site	+	1056197	1056197	-3865	14843	AN3152
3	CONTIG51	1056452	1058008	3.97	0.00E+00	transcription_start_site	+	1062610	1062610	-5380	14844	AN3153
1134	CONTIG51	1084221	1084855	1.81	3.08E-02	transcription_start_site	-	1080859	1080859	-3679	14866	AN3159
1134	CONTIG51	1084221	1084855	1.81	3.08E-02	transcription_start_site	-	1079955	1079955	-4583	14867	AN3159
1134	CONTIG51	1084221	1084855	1.81	3.08E-02	transcription_start_site	-	1079655	1079655	-4883	14868	AN3159
1134	CONTIG51	1084221	1084855	1.81	3.08E-02	transcription_start_site	-	1083325	1083325	-1213	14869	AN3160
1134	CONTIG51	1084221	1084855	1.81	3.08E-02	transcription_start_site	-	1083030	1083030	-1508	14870	AN3160
1134	CONTIG51	1084221	1084855	1.81	3.08E-02	transcription_start_site	-	1082710	1082710	-1828	14871	AN3160
1134	CONTIG51	1084221	1084855	1.81	3.08E-02	transcription_start_site	-	1082426	1082426	-2112	14872	AN3160
1134	CONTIG51	1084221	1084855	1.81	3.08E-02	transcription_start_site	-	1082302	1082302	-2236	14873	AN3160
1134	CONTIG51	1084221	1084855	1.81	3.08E-02	transcription_start_site	+	1084749	1084749	-211	14874	AN3161
1134	CONTIG51	1084221	1084855	1.81	3.08E-02	transcription_start_site	+	1085413	1085413	-875	14875	AN3161
1134	CONTIG51	1084221	1084855	1.81	3.08E-02	transcription_start_site	+	1085562	1085562	-1024	14876	AN3161
1134	CONTIG51	1084221	1084855	1.81	3.08E-02	transcription_start_site	+	1087160	1087160	-2622	14877	AN3162
1134	CONTIG51	1084221	1084855	1.81	3.08E-02	transcription_start_site	+	1087636	1087636	-3098	14878	AN3162
690	CONTIG51	1088778	1089127	2.21	7.85E-03	transcription_start_site	+	1089185	1089185	-232	14879	AN3163
690	CONTIG51	1088778	1089127	2.21	7.85E-03	transcription_start_site	+	1089436	1089436	-483	14880	AN3163
690	CONTIG51	1088778	1089127	2.21	7.85E-03	transcription_start_site	+	1089619	1089619	-666	14881	AN3163
690	CONTIG51	1088778	1089127	2.21	7.85E-03	transcription_start_site	+	1090157	1090157	-1204	14882	AN3163
1134	CONTIG51	1084221	1084855	1.81	3.08E-02	transcription_start_site	+	1089185	1089185	-4647	14879	AN3163
1134	CONTIG51	1084221	1084855	1.81	3.08E-02	transcription_start_site	+	1089436	1089436	-4898	14880	AN3163
1134	CONTIG51	1084221	1084855	1.81	3.08E-02	transcription_start_site	+	1089619	1089619	-5081	14881	AN3163
690	CONTIG51	1088778	1089127	2.21	7.85E-03	transcription_start_site	+	1090824	1090824	-1871	14883	AN3164
2150	CONTIG51	1091553	1091927	1.32	1.44E-01	transcription_start_site	+	1090824	1090824	916	14883	AN3164
690	CONTIG51	1088778	1089127	2.21	7.85E-03	transcription_start_site	+	1092613	1092613	-3660	14884	AN3165
690	CONTIG51	1088778	1089127	2.21	7.85E-03	transcription_start_site	+	1092871	1092871	-3918	14885	AN3165
2150	CONTIG51	1091553	1091927	1.32	1.44E-01	transcription_start_site	+	1092613	1092613	-873	14884	AN3165
2150	CONTIG51	1091553	1091927	1.32	1.44E-01	transcription_start_site	+	1092871	1092871	-1131	14885	AN3165
2	CONTIG52	15526	16105	4.02	0.00E+00	transcription_start_site	-	12302	12302	-3513	14915	AN3178
2	CONTIG52	15526	16105	4.02	0.00E+00	transcription_start_site	-	11800	11800	-4015	14916	AN3178
2	CONTIG52	15526	16105	4.02	0.00E+00	transcription_start_site	-	16056	16056	240	14917	AN3179
2	CONTIG52	15526	16105	4.02	0.00E+00	transcription_start_site	-	15466	15466	-349	14918	AN3179

2	CONTIG52	15526	16105	4.02	0.00E+00	transcription_start_site	-	13977	13977	-1838	14919	AN3179
137	CONTIG52	19956	20320	3.09	1.54E-03	transcription_start_site	-	16056	16056	-4082	14917	AN3179
137	CONTIG52	19956	20320	3.09	1.54E-03	transcription_start_site	-	15466	15466	-4672	14918	AN3179
1800	CONTIG52	17796	18665	1.46	1.79E-01	transcription_start_site	-	16056	16056	-2174	14917	AN3179
1800	CONTIG52	17796	18665	1.46	1.79E-01	transcription_start_site	-	15466	15466	-2764	14918	AN3179
1800	CONTIG52	17796	18665	1.46	1.79E-01	transcription_start_site	-	13977	13977	-4253	14919	AN3179
137	CONTIG52	19956	20320	3.09	1.54E-03	transcription_start_site	-	19805	19805	-333	14920	AN3180
137	CONTIG52	19956	20320	3.09	1.54E-03	transcription_start_site	-	19603	19603	-535	14921	AN3180
137	CONTIG52	19956	20320	3.09	1.54E-03	transcription_start_site	-	18738	18738	-1400	14922	AN3180
1800	CONTIG52	17796	18665	1.46	1.79E-01	transcription_start_site	-	18738	18738	507	14922	AN3180
1800	CONTIG52	17796	18665	1.46	1.79E-01	transcription_start_site	-	19603	19603	1372	14921	AN3180
1065	CONTIG52	49128	49492	1.86	6.84E-02	transcription_start_site	-	48493	48493	-817	14938	AN3188
1065	CONTIG52	49128	49492	1.86	6.84E-02	transcription_start_site	-	48272	48272	-1038	14939	AN3188
1065	CONTIG52	49128	49492	1.86	6.84E-02	transcription_start_site	-	48130	48130	-1180	14940	AN3188
1065	CONTIG52	49128	49492	1.86	6.84E-02	transcription_start_site	-	47861	47861	-1449	14941	AN3188
1065	CONTIG52	49128	49492	1.86	6.84E-02	transcription_start_site	-	47388	47388	-1922	14942	AN3188
1065	CONTIG52	49128	49492	1.86	6.84E-02	transcription_start_site	-	45919	45919	-3391	14943	AN3188
1065	CONTIG52	49128	49492	1.86	6.84E-02	transcription_start_site	-	45104	45104	-4206	14944	AN3188
1310	CONTIG52	62857	63141	1.69	1.08E-01	transcription_start_site	-	58038	58038	-4961	14945	AN3190
1310	CONTIG52	62857	63141	1.69	1.08E-01	transcription_start_site	-	57951	57951	-5048	14946	AN3190
1310	CONTIG52	62857	63141	1.69	1.08E-01	transcription_start_site	-	59525	59525	-3474	14948	AN3191
1310	CONTIG52	62857	63141	1.69	1.08E-01	transcription_start_site	-	59325	59325	-3674	14949	AN3191
1310	CONTIG52	62857	63141	1.69	1.08E-01	transcription_start_site	-	58506	58506	-4493	14950	AN3191
392	CONTIG52	65412	65686	2.62	7.85E-03	transcription_start_site	-	65282	65282	-267	14951	AN3193
1541	CONTIG52	73802	74301	1.57	1.28E-01	transcription_start_site	-	70687	70687	-3364	14955	AN3195
1541	CONTIG52	73802	74301	1.57	1.28E-01	transcription_start_site	-	70246	70246	-3805	14956	AN3195
1541	CONTIG52	73802	74301	1.57	1.28E-01	transcription_start_site	-	69901	69901	-4150	14957	AN3195
1541	CONTIG52	73802	74301	1.57	1.28E-01	transcription_start_site	-	69511	69511	-4540	14958	AN3195
1541	CONTIG52	73802	74301	1.57	1.28E-01	transcription_start_site	-	69230	69230	-4821	14959	AN3195
1541	CONTIG52	73802	74301	1.57	1.28E-01	transcription_start_site	+	73848	73848	203	14961	AN3197
1541	CONTIG52	73802	74301	1.57	1.28E-01	transcription_start_site	+	74649	74649	-597	14962	AN3197
1375	CONTIG53	4820	5309	1.65	8.01E-02	transcription_start_site	+	4985	4985	79	14974	AN3201
1375	CONTIG53	4820	5309	1.65	8.01E-02	transcription_start_site	+	8575	8575	-3510	14975	AN3202
1375	CONTIG53	4820	5309	1.65	8.01E-02	transcription_start_site	+	8750	8750	-3685	14976	AN3202
1375	CONTIG53	4820	5309	1.65	8.01E-02	transcription_start_site	+	9008	9008	-3943	14977	AN3202
1375	CONTIG53	4820	5309	1.65	8.01E-02	transcription_start_site	+	9394	9394	-4329	14978	AN3202
1375	CONTIG53	4820	5309	1.65	8.01E-02	transcription_start_site	+	9765	9765	-4700	14979	AN3202
332	CONTIG53	10955	11614	2.71	0.00E+00	transcription_start_site	+	11363	11363	-78	14980	AN3203
2127	CONTIG53	15235	15514	1.33	1.93E-01	transcription_start_site	+	19806	19806	-4431	14983	AN3205
1990	CONTIG53	24227	24586	1.38	1.66E-01	transcription_start_site	+	24299	24299	107	14987	AN3206
1990	CONTIG53	24227	24586	1.38	1.66E-01	transcription_start_site	+	23505	23505	901	14986	AN3206
1990	CONTIG53	24227	24586	1.38	1.66E-01	transcription_start_site	+	23404	23404	1002	14985	AN3206
1990	CONTIG53	24227	24586	1.38	1.66E-01	transcription_start_site	+	26735	26735	-2328	14988	AN3207
1990	CONTIG53	24227	24586	1.38	1.66E-01	transcription_start_site	+	27103	27103	-2696	14989	AN3207
1990	CONTIG53	24227	24586	1.38	1.66E-01	transcription_start_site	+	27197	27197	-2790	14990	AN3207
1990	CONTIG53	24227	24586	1.38	1.66E-01	transcription_start_site	+	27320	27320	-2913	14991	AN3207
1990	CONTIG53	24227	24586	1.38	1.66E-01	transcription_start_site	+	27726	27726	-3319	14992	AN3207
1990	CONTIG53	24227	24586	1.38	1.66E-01	transcription_start_site	+	27925	27925	-3518	14993	AN3207
1990	CONTIG53	24227	24586	1.38	1.66E-01	transcription_start_site	+	28216	28216	-3809	14994	AN3207
1990	CONTIG53	24227	24586	1.38	1.66E-01	transcription_start_site	+	28477	28477	-4070	14995	AN3207
1990	CONTIG53	24227	24586	1.38	1.66E-01	transcription_start_site	+	28675	28675	-4268	14996	AN3207
1991	CONTIG53	30918	31332	1.38	1.66E-01	transcription_start_site	-	31105	31105	-20	14997	AN3208
1991	CONTIG53	30918	31332	1.38	1.66E-01	transcription_start_site	+	31495	31495	-370	14998	AN3209
1991	CONTIG53	30918	31332	1.38	1.66E-01	transcription_start_site	+	31650	31650	-525	14999	AN3209
375	CONTIG53	41496	41920	2.65	3.03E-03	transcription_start_site	-	41087	41087	-621	15000	AN3210
375	CONTIG53	41496	41920	2.65	3.03E-03	transcription_start_site	-	40546	40546	-1162	15001	AN3210
375	CONTIG53	41496	41920	2.65	3.03E-03	transcription_start_site	-	40189	40189	-1519	15002	AN3210

375	CONTIG53	41496	41920	2.65	3.03E-03	transcription_start_site	-	39993	39993	-1715	15003	AN3210
375	CONTIG53	41496	41920	2.65	3.03E-03	transcription_start_site	-	39846	39846	-1862	15004	AN3210
375	CONTIG53	41496	41920	2.65	3.03E-03	transcription_start_site	-	39518	39518	-2190	15005	AN3210
375	CONTIG53	41496	41920	2.65	3.03E-03	transcription_start_site	-	39123	39123	-2585	15006	AN3210
1497	CONTIG53	44851	45740	1.59	7.25E-02	transcription_start_site	-	41087	41087	-4208	15000	AN3210
1497	CONTIG53	44851	45740	1.59	7.25E-02	transcription_start_site	-	40546	40546	-4749	15001	AN3210
1497	CONTIG53	44851	45740	1.59	7.25E-02	transcription_start_site	-	40189	40189	-5106	15002	AN3210
1497	CONTIG53	44851	45740	1.59	7.25E-02	transcription_start_site	-	39993	39993	-5302	15003	AN3210
1499	CONTIG53	44251	44535	1.59	9.24E-02	transcription_start_site	-	41087	41087	-3306	15000	AN3210
1499	CONTIG53	44251	44535	1.59	9.24E-02	transcription_start_site	-	40546	40546	-3847	15001	AN3210
1499	CONTIG53	44251	44535	1.59	9.24E-02	transcription_start_site	-	40189	40189	-4204	15002	AN3210
1499	CONTIG53	44251	44535	1.59	9.24E-02	transcription_start_site	-	39993	39993	-4400	15003	AN3210
1499	CONTIG53	44251	44535	1.59	9.24E-02	transcription_start_site	-	39846	39846	-4547	15004	AN3210
1499	CONTIG53	44251	44535	1.59	9.24E-02	transcription_start_site	-	39518	39518	-4875	15005	AN3210
830	CONTIG53	47851	49045	2.07	1.06E-02	transcription_start_site	-	45748	45748	-2700	15007	AN3211
830	CONTIG53	47851	49045	2.07	1.06E-02	transcription_start_site	-	45458	45458	-2990	15008	AN3211
1497	CONTIG53	44851	45740	1.59	7.25E-02	transcription_start_site	-	45458	45458	162	15008	AN3211
1497	CONTIG53	44851	45740	1.59	7.25E-02	transcription_start_site	-	45748	45748	452	15007	AN3211
1499	CONTIG53	44251	44535	1.59	9.24E-02	transcription_start_site	-	45458	45458	1065	15008	AN3211
830	CONTIG53	47851	49045	2.07	1.06E-02	transcription_start_site	-	47148	47148	-1300	15009	AN3212
830	CONTIG53	47851	49045	2.07	1.06E-02	transcription_start_site	-	46860	46860	-1588	15010	AN3212
830	CONTIG53	47851	49045	2.07	1.06E-02	transcription_start_site	-	48869	48869	421	15011	AN3213
35	CONTIG54	7806	8095	3.51	0.00E+00	transcription_start_site	-	7670	7670	-280	15012	AN3214
35	CONTIG54	7806	8095	3.51	0.00E+00	transcription_start_site	-	7105	7105	-845	15013	AN3214
35	CONTIG54	7806	8095	3.51	0.00E+00	transcription_start_site	-	6797	6797	-1153	15014	AN3214
35	CONTIG54	7806	8095	3.51	0.00E+00	transcription_start_site	-	6359	6359	-1591	15015	AN3214
35	CONTIG54	7806	8095	3.51	0.00E+00	transcription_start_site	-	5329	5329	-2621	15016	AN3214
35	CONTIG54	7806	8095	3.51	0.00E+00	transcription_start_site	-	5039	5039	-2911	15017	AN3214
35	CONTIG54	7806	8095	3.51	0.00E+00	transcription_start_site	-	4877	4877	-3073	15018	AN3214
94	CONTIG54	12161	12740	3.24	0.00E+00	transcription_start_site	-	7670	7670	-4780	15012	AN3214
1697	CONTIG54	11196	11825	1.5	5.88E-02	transcription_start_site	-	7670	7670	-3840	15012	AN3214
1697	CONTIG54	11196	11825	1.5	5.88E-02	transcription_start_site	-	7105	7105	-4405	15013	AN3214
1697	CONTIG54	11196	11825	1.5	5.88E-02	transcription_start_site	-	6797	6797	-4713	15014	AN3214
1697	CONTIG54	11196	11825	1.5	5.88E-02	transcription_start_site	-	6359	6359	-5151	15015	AN3214
94	CONTIG54	12161	12740	3.24	0.00E+00	transcription_start_site	-	9993	9993	-2457	15019	AN3215
94	CONTIG54	12161	12740	3.24	0.00E+00	transcription_start_site	-	9841	9841	-2609	15020	AN3215
94	CONTIG54	12161	12740	3.24	0.00E+00	transcription_start_site	-	9724	9724	-2726	15021	AN3215
1697	CONTIG54	11196	11825	1.5	5.88E-02	transcription_start_site	-	9993	9993	-1517	15019	AN3215
1697	CONTIG54	11196	11825	1.5	5.88E-02	transcription_start_site	-	9841	9841	-1669	15020	AN3215
1697	CONTIG54	11196	11825	1.5	5.88E-02	transcription_start_site	-	9724	9724	-1786	15021	AN3215
35	CONTIG54	7806	8095	3.51	0.00E+00	transcription_start_site	+	11190	11190	-3239	15022	AN3216
35	CONTIG54	7806	8095	3.51	0.00E+00	transcription_start_site	+	12083	12083	-4132	15023	AN3216
35	CONTIG54	7806	8095	3.51	0.00E+00	transcription_start_site	+	12528	12528	-4577	15024	AN3216
35	CONTIG54	7806	8095	3.51	0.00E+00	transcription_start_site	+	12803	12803	-4852	15025	AN3216
94	CONTIG54	12161	12740	3.24	0.00E+00	transcription_start_site	+	12528	12528	-77	15024	AN3216
94	CONTIG54	12161	12740	3.24	0.00E+00	transcription_start_site	+	12803	12803	-352	15025	AN3216
94	CONTIG54	12161	12740	3.24	0.00E+00	transcription_start_site	+	12083	12083	367	15023	AN3216
94	CONTIG54	12161	12740	3.24	0.00E+00	transcription_start_site	+	13262	13262	-811	15026	AN3216
94	CONTIG54	12161	12740	3.24	0.00E+00	transcription_start_site	+	11190	11190	1260	15022	AN3216
94	CONTIG54	12161	12740	3.24	0.00E+00	transcription_start_site	+	13971	13971	-1520	15027	AN3216
94	CONTIG54	12161	12740	3.24	0.00E+00	transcription_start_site	+	14144	14144	-1693	15028	AN3216
1697	CONTIG54	11196	11825	1.5	5.88E-02	transcription_start_site	+	11190	11190	320	15022	AN3216
1697	CONTIG54	11196	11825	1.5	5.88E-02	transcription_start_site	+	12083	12083	-572	15023	AN3216
1697	CONTIG54	11196	11825	1.5	5.88E-02	transcription_start_site	+	12528	12528	-1017	15024	AN3216
1697	CONTIG54	11196	11825	1.5	5.88E-02	transcription_start_site	+	12803	12803	-1292	15025	AN3216
1697	CONTIG54	11196	11825	1.5	5.88E-02	transcription_start_site	+	13262	13262	-1751	15026	AN3216
1697	CONTIG54	11196	11825	1.5	5.88E-02	transcription_start_site	+	13971	13971	-2460	15027	AN3216

1697	CONTIG54	11196	11825	1.5	5.88E-02	transcription_start_site	+	14144	14144	-2633	15028	AN3216
94	CONTIG54	12161	12740	3.24	0.00E+00	transcription_start_site	+	15427	15427	-2976	15029	AN3217
94	CONTIG54	12161	12740	3.24	0.00E+00	transcription_start_site	+	16342	16342	-3891	15030	AN3217
94	CONTIG54	12161	12740	3.24	0.00E+00	transcription_start_site	+	16558	16558	-4107	15031	AN3217
94	CONTIG54	12161	12740	3.24	0.00E+00	transcription_start_site	+	16904	16904	-4453	15032	AN3217
94	CONTIG54	12161	12740	3.24	0.00E+00	transcription_start_site	+	17166	17166	-4715	15033	AN3217
1697	CONTIG54	11196	11825	1.5	5.88E-02	transcription_start_site	+	15427	15427	-3916	15029	AN3217
1697	CONTIG54	11196	11825	1.5	5.88E-02	transcription_start_site	+	16342	16342	-4831	15030	AN3217
1697	CONTIG54	11196	11825	1.5	5.88E-02	transcription_start_site	+	16558	16558	-5047	15031	AN3217
663	CONTIG54	28127	28482	2.23	3.61E-03	transcription_start_site	-	25029	25029	-3275	15039	AN3220
663	CONTIG54	28127	28482	2.23	3.61E-03	transcription_start_site	-	24486	24486	-3818	15040	AN3220
663	CONTIG54	28127	28482	2.23	3.61E-03	transcription_start_site	-	24287	24287	-4017	15041	AN3220
663	CONTIG54	28127	28482	2.23	3.61E-03	transcription_start_site	-	23895	23895	-4409	15042	AN3220
1698	CONTIG54	25437	25779	1.5	5.88E-02	transcription_start_site	-	25029	25029	-579	15039	AN3220
1698	CONTIG54	25437	25779	1.5	5.88E-02	transcription_start_site	-	24486	24486	-1122	15040	AN3220
1698	CONTIG54	25437	25779	1.5	5.88E-02	transcription_start_site	-	24287	24287	-1321	15041	AN3220
1698	CONTIG54	25437	25779	1.5	5.88E-02	transcription_start_site	-	23895	23895	-1713	15042	AN3220
2007	CONTIG54	26032	26911	1.37	9.24E-02	transcription_start_site	-	25029	25029	-1442	15039	AN3220
2007	CONTIG54	26032	26911	1.37	9.24E-02	transcription_start_site	-	24486	24486	-1985	15040	AN3220
2007	CONTIG54	26032	26911	1.37	9.24E-02	transcription_start_site	-	24287	24287	-2184	15041	AN3220
2007	CONTIG54	26032	26911	1.37	9.24E-02	transcription_start_site	-	23895	23895	-2576	15042	AN3220
2547	CONTIG54	28667	29241	1.19	1.66E-01	transcription_start_site	-	25029	25029	-3925	15039	AN3220
2547	CONTIG54	28667	29241	1.19	1.66E-01	transcription_start_site	-	24486	24486	-4468	15040	AN3220
2547	CONTIG54	28667	29241	1.19	1.66E-01	transcription_start_site	-	24287	24287	-4667	15041	AN3220
2547	CONTIG54	28667	29241	1.19	1.66E-01	transcription_start_site	-	23895	23895	-5059	15042	AN3220
663	CONTIG54	28127	28482	2.23	3.61E-03	transcription_start_site	-	27712	27712	-592	15043	AN3221
663	CONTIG54	28127	28482	2.23	3.61E-03	transcription_start_site	-	27415	27415	-889	15044	AN3221
663	CONTIG54	28127	28482	2.23	3.61E-03	transcription_start_site	-	27163	27163	-1141	15045	AN3221
663	CONTIG54	28127	28482	2.23	3.61E-03	transcription_start_site	-	26720	26720	-1584	15046	AN3221
1578	CONTIG54	30312	30591	1.55	5.22E-02	transcription_start_site	-	27712	27712	-2739	15043	AN3221
1578	CONTIG54	30312	30591	1.55	5.22E-02	transcription_start_site	-	27415	27415	-3036	15044	AN3221
1578	CONTIG54	30312	30591	1.55	5.22E-02	transcription_start_site	-	27163	27163	-3288	15045	AN3221
1578	CONTIG54	30312	30591	1.55	5.22E-02	transcription_start_site	-	26720	26720	-3731	15046	AN3221
1698	CONTIG54	25437	25779	1.5	5.88E-02	transcription_start_site	-	26720	26720	1112	15046	AN3221
2007	CONTIG54	26032	26911	1.37	9.24E-02	transcription_start_site	-	26720	26720	248	15046	AN3221
2007	CONTIG54	26032	26911	1.37	9.24E-02	transcription_start_site	-	27163	27163	691	15045	AN3221
2007	CONTIG54	26032	26911	1.37	9.24E-02	transcription_start_site	-	27415	27415	943	15044	AN3221
2007	CONTIG54	26032	26911	1.37	9.24E-02	transcription_start_site	-	27712	27712	1240	15043	AN3221
2547	CONTIG54	28667	29241	1.19	1.66E-01	transcription_start_site	-	27712	27712	-1242	15043	AN3221
2547	CONTIG54	28667	29241	1.19	1.66E-01	transcription_start_site	-	27415	27415	-1539	15044	AN3221
2547	CONTIG54	28667	29241	1.19	1.66E-01	transcription_start_site	-	27163	27163	-1791	15045	AN3221
2547	CONTIG54	28667	29241	1.19	1.66E-01	transcription_start_site	-	26720	26720	-2234	15046	AN3221
663	CONTIG54	28127	28482	2.23	3.61E-03	transcription_start_site	+	30738	30738	-2433	15047	AN3222
663	CONTIG54	28127	28482	2.23	3.61E-03	transcription_start_site	+	30826	30826	-2521	15048	AN3222
663	CONTIG54	28127	28482	2.23	3.61E-03	transcription_start_site	+	31045	31045	-2740	15049	AN3222
663	CONTIG54	28127	28482	2.23	3.61E-03	transcription_start_site	+	31247	31247	-2942	15050	AN3222
663	CONTIG54	28127	28482	2.23	3.61E-03	transcription_start_site	+	32346	32346	-4041	15051	AN3222
1578	CONTIG54	30312	30591	1.55	5.22E-02	transcription_start_site	+	30738	30738	-286	15047	AN3222
1578	CONTIG54	30312	30591	1.55	5.22E-02	transcription_start_site	+	30826	30826	-374	15048	AN3222
1578	CONTIG54	30312	30591	1.55	5.22E-02	transcription_start_site	+	31045	31045	-593	15049	AN3222
1578	CONTIG54	30312	30591	1.55	5.22E-02	transcription_start_site	+	31247	31247	-795	15050	AN3222
1578	CONTIG54	30312	30591	1.55	5.22E-02	transcription_start_site	+	32346	32346	-1894	15051	AN3222
1698	CONTIG54	25437	25779	1.5	5.88E-02	transcription_start_site	+	30738	30738	-5130	15047	AN3222
2007	CONTIG54	26032	26911	1.37	9.24E-02	transcription_start_site	+	30738	30738	-4266	15047	AN3222
2007	CONTIG54	26032	26911	1.37	9.24E-02	transcription_start_site	+	30826	30826	-4354	15048	AN3222
2007	CONTIG54	26032	26911	1.37	9.24E-02	transcription_start_site	+	31045	31045	-4573	15049	AN3222
2007	CONTIG54	26032	26911	1.37	9.24E-02	transcription_start_site	+	31247	31247	-4775	15050	AN3222

2547	CONTIG54	28667	29241	1.19	1.66E-01	transcription_start_site	+	30738	30738	-1784	15047	AN3222
2547	CONTIG54	28667	29241	1.19	1.66E-01	transcription_start_site	+	30826	30826	-1872	15048	AN3222
2547	CONTIG54	28667	29241	1.19	1.66E-01	transcription_start_site	+	31045	31045	-2091	15049	AN3222
2547	CONTIG54	28667	29241	1.19	1.66E-01	transcription_start_site	+	31247	31247	-2293	15050	AN3222
2547	CONTIG54	28667	29241	1.19	1.66E-01	transcription_start_site	+	32346	32346	-3392	15051	AN3222
2008	CONTIG54	35627	35925	1.37	9.24E-02	transcription_start_site	-	35541	35541	-235	15052	AN3223
2008	CONTIG54	35627	35925	1.37	9.24E-02	transcription_start_site	-	34747	34747	-1029	15053	AN3223
2008	CONTIG54	35627	35925	1.37	9.24E-02	transcription_start_site	-	34236	34236	-1540	15054	AN3223
2263	CONTIG54	33677	33961	1.28	1.25E-01	transcription_start_site	-	34236	34236	417	15054	AN3223
2263	CONTIG54	33677	33961	1.28	1.25E-01	transcription_start_site	-	34747	34747	928	15053	AN3223
2008	CONTIG54	35627	35925	1.37	9.24E-02	transcription_start_site	+	36527	36527	-751	15055	AN3224
2008	CONTIG54	35627	35925	1.37	9.24E-02	transcription_start_site	+	36854	36854	-1078	15056	AN3224
2008	CONTIG54	35627	35925	1.37	9.24E-02	transcription_start_site	+	37284	37284	-1508	15057	AN3224
2263	CONTIG54	33677	33961	1.28	1.25E-01	transcription_start_site	+	36527	36527	-2708	15055	AN3224
2263	CONTIG54	33677	33961	1.28	1.25E-01	transcription_start_site	+	36854	36854	-3035	15056	AN3224
2263	CONTIG54	33677	33961	1.28	1.25E-01	transcription_start_site	+	37284	37284	-3465	15057	AN3224
2537	CONTIG54	44870	45654	1.19	1.59E-01	transcription_start_site	-	41893	41893	-3369	15058	AN3225
2537	CONTIG54	44870	45654	1.19	1.59E-01	transcription_start_site	-	41144	41144	-4118	15059	AN3225
2537	CONTIG54	44870	45654	1.19	1.59E-01	transcription_start_site	-	40856	40856	-4406	15060	AN3225
2537	CONTIG54	44870	45654	1.19	1.59E-01	transcription_start_site	-	40337	40337	-4925	15061	AN3225
2537	CONTIG54	44870	45654	1.19	1.59E-01	transcription_start_site	-	40176	40176	-5086	15062	AN3225
2671	CONTIG54	43445	43794	1.14	1.93E-01	transcription_start_site	-	41893	41893	-1726	15058	AN3225
2671	CONTIG54	43445	43794	1.14	1.93E-01	transcription_start_site	-	41144	41144	-2475	15059	AN3225
2671	CONTIG54	43445	43794	1.14	1.93E-01	transcription_start_site	-	40856	40856	-2763	15060	AN3225
2671	CONTIG54	43445	43794	1.14	1.93E-01	transcription_start_site	-	40337	40337	-3282	15061	AN3225
2671	CONTIG54	43445	43794	1.14	1.93E-01	transcription_start_site	-	40176	40176	-3443	15062	AN3225
2537	CONTIG54	44870	45654	1.19	1.59E-01	transcription_start_site	-	44038	44038	-1224	15063	AN3226
2537	CONTIG54	44870	45654	1.19	1.59E-01	transcription_start_site	-	43891	43891	-1371	15064	AN3226
2671	CONTIG54	43445	43794	1.14	1.93E-01	transcription_start_site	-	43891	43891	271	15064	AN3226
2671	CONTIG54	43445	43794	1.14	1.93E-01	transcription_start_site	-	44038	44038	418	15063	AN3226
2537	CONTIG54	44870	45654	1.19	1.59E-01	transcription_start_site	-	45468	45468	206	15066	AN3227
2537	CONTIG54	44870	45654	1.19	1.59E-01	transcription_start_site	-	46245	46245	983	15065	AN3227
2672	CONTIG54	80108	80382	1.14	1.93E-01	transcription_start_site	-	78107	78107	-2138	15097	AN3238
2672	CONTIG54	80108	80382	1.14	1.93E-01	transcription_start_site	-	77957	77957	-2288	15098	AN3238
2672	CONTIG54	80108	80382	1.14	1.93E-01	transcription_start_site	-	77149	77149	-3096	15099	AN3238
2672	CONTIG54	80108	80382	1.14	1.93E-01	transcription_start_site	+	80343	80343	-98	15101	AN3239
2672	CONTIG54	80108	80382	1.14	1.93E-01	transcription_start_site	+	79685	79685	560	15100	AN3239
2672	CONTIG54	80108	80382	1.14	1.93E-01	transcription_start_site	+	80903	80903	-658	15102	AN3239
256	CONTIG54	99463	99732	2.83	2.67E-04	transcription_start_site	-	96999	96999	-2598	15123	AN3246
256	CONTIG54	99463	99732	2.83	2.67E-04	transcription_start_site	-	96814	96814	-2783	15124	AN3246
256	CONTIG54	99463	99732	2.83	2.67E-04	transcription_start_site	+	100366	100366	-768	15125	AN3247
256	CONTIG54	99463	99732	2.83	2.67E-04	transcription_start_site	+	101243	101243	-1645	15126	AN3247
256	CONTIG54	99463	99732	2.83	2.67E-04	transcription_start_site	+	101592	101592	-1994	15127	AN3247
256	CONTIG54	99463	99732	2.83	2.67E-04	transcription_start_site	+	102334	102334	-2736	15128	AN3247
664	CONTIG54	107268	107532	2.23	3.61E-03	transcription_start_site	-	107192	107192	-208	15131	AN3248
664	CONTIG54	107268	107532	2.23	3.61E-03	transcription_start_site	+	107552	107552	-152	15132	AN3249
664	CONTIG54	107268	107532	2.23	3.61E-03	transcription_start_site	+	107982	107982	-582	15133	AN3249
664	CONTIG54	107268	107532	2.23	3.61E-03	transcription_start_site	+	108410	108410	-1010	15134	AN3249
664	CONTIG54	107268	107532	2.23	3.61E-03	transcription_start_site	+	110421	110421	-3021	15135	AN3250
664	CONTIG54	107268	107532	2.23	3.61E-03	transcription_start_site	+	110668	110668	-3268	15136	AN3250
664	CONTIG54	107268	107532	2.23	3.61E-03	transcription_start_site	+	110754	110754	-3354	15137	AN3250
664	CONTIG54	107268	107532	2.23	3.61E-03	transcription_start_site	+	111168	111168	-3768	15138	AN3250
1579	CONTIG54	113788	114052	1.55	5.22E-02	transcription_start_site	-	113769	113769	-151	15139	AN3251
1579	CONTIG54	113788	114052	1.55	5.22E-02	transcription_start_site	-	113259	113259	-661	15140	AN3251
1579	CONTIG54	113788	114052	1.55	5.22E-02	transcription_start_site	-	115042	115042	1122	15146	AN3252
2416	CONTIG54	141541	141881	1.23	1.44E-01	transcription_start_site	-	141703	141703	-8	15174	AN3260
2416	CONTIG54	141541	141881	1.23	1.44E-01	transcription_start_site	-	142276	142276	565	15173	AN3260

2416	CONTIG54	141541	141881	1.23	1.44E-01	transcription_start_site	+	143385	143385	-1674	15175 AN3261
2416	CONTIG54	141541	141881	1.23	1.44E-01	transcription_start_site	+	143504	143504	-1793	15176 AN3261
2416	CONTIG54	141541	141881	1.23	1.44E-01	transcription_start_site	+	143919	143919	-2208	15177 AN3261
2416	CONTIG54	141541	141881	1.23	1.44E-01	transcription_start_site	+	144233	144233	-2522	15178 AN3261
2416	CONTIG54	141541	141881	1.23	1.44E-01	transcription_start_site	+	144460	144460	-2749	15179 AN3261
2416	CONTIG54	141541	141881	1.23	1.44E-01	transcription_start_site	+	144666	144666	-2955	15180 AN3261
2416	CONTIG54	141541	141881	1.23	1.44E-01	transcription_start_site	+	145170	145170	-3459	15181 AN3261
2416	CONTIG54	141541	141881	1.23	1.44E-01	transcription_start_site	+	145281	145281	-3570	15182 AN3261
2416	CONTIG54	141541	141881	1.23	1.44E-01	transcription_start_site	+	145573	145573	-3862	15183 AN3261
2548	CONTIG54	150471	150740	1.19	1.66E-01	transcription_start_site	-	150211	150211	-394	15184 AN3262
2548	CONTIG54	150471	150740	1.19	1.66E-01	transcription_start_site	-	149899	149899	-706	15185 AN3262
2548	CONTIG54	150471	150740	1.19	1.66E-01	transcription_start_site	-	149778	149778	-827	15186 AN3262
2548	CONTIG54	150471	150740	1.19	1.66E-01	transcription_start_site	-	149646	149646	-959	15187 AN3262
2548	CONTIG54	150471	150740	1.19	1.66E-01	transcription_start_site	-	149568	149568	-1037	15188 AN3262
2548	CONTIG54	150471	150740	1.19	1.66E-01	transcription_start_site	-	149287	149287	-1318	15189 AN3262
2548	CONTIG54	150471	150740	1.19	1.66E-01	transcription_start_site	-	149070	149070	-1535	15190 AN3262
2548	CONTIG54	150471	150740	1.19	1.66E-01	transcription_start_site	-	148547	148547	-2058	15191 AN3262
2548	CONTIG54	150471	150740	1.19	1.66E-01	transcription_start_site	-	151535	151535	929	15192 AN3263
2264	CONTIG54	157516	157775	1.28	1.25E-01	transcription_start_site	-	154794	154794	-2851	15193 AN3264
2264	CONTIG54	157516	157775	1.28	1.25E-01	transcription_start_site	-	154517	154517	-3128	15194 AN3264
2264	CONTIG54	157516	157775	1.28	1.25E-01	transcription_start_site	-	154070	154070	-3575	15195 AN3264
2264	CONTIG54	157516	157775	1.28	1.25E-01	transcription_start_site	-	156719	156719	-926	15196 AN3265
2264	CONTIG54	157516	157775	1.28	1.25E-01	transcription_start_site	-	156602	156602	-1043	15197 AN3265
2264	CONTIG54	157516	157775	1.28	1.25E-01	transcription_start_site	-	156425	156425	-1220	15198 AN3265
2264	CONTIG54	157516	157775	1.28	1.25E-01	transcription_start_site	-	156059	156059	-1586	15199 AN3265
2264	CONTIG54	157516	157775	1.28	1.25E-01	transcription_start_site	-	155599	155599	-2046	15200 AN3265
2264	CONTIG54	157516	157775	1.28	1.25E-01	transcription_start_site	+	160241	160241	-2595	15201 AN3267
2264	CONTIG54	157516	157775	1.28	1.25E-01	transcription_start_site	+	160835	160835	-3189	15202 AN3267
2264	CONTIG54	157516	157775	1.28	1.25E-01	transcription_start_site	+	161509	161509	-3863	15203 AN3267
2264	CONTIG54	157516	157775	1.28	1.25E-01	transcription_start_site	+	161571	161571	-3925	15204 AN3267
887	CONTIG54	171981	172256	2.01	8.81E-03	transcription_start_site	-	171959	171959	-159	15205 AN3269
887	CONTIG54	171981	172256	2.01	8.81E-03	transcription_start_site	-	171797	171797	-321	15206 AN3269
887	CONTIG54	171981	172256	2.01	8.81E-03	transcription_start_site	-	170877	170877	-1241	15207 AN3269
887	CONTIG54	171981	172256	2.01	8.81E-03	transcription_start_site	-	170431	170431	-1687	15208 AN3269
2417	CONTIG54	171301	171880	1.23	1.44E-01	transcription_start_site	-	171797	171797	206	15206 AN3269
2417	CONTIG54	171301	171880	1.23	1.44E-01	transcription_start_site	-	171959	171959	368	15205 AN3269
2417	CONTIG54	171301	171880	1.23	1.44E-01	transcription_start_site	-	170877	170877	-713	15207 AN3269
2417	CONTIG54	171301	171880	1.23	1.44E-01	transcription_start_site	-	170431	170431	-1159	15208 AN3269
887	CONTIG54	171981	172256	2.01	8.81E-03	transcription_start_site	+	172382	172382	-263	15209 AN3270
887	CONTIG54	171981	172256	2.01	8.81E-03	transcription_start_site	+	172789	172789	-670	15210 AN3270
2417	CONTIG54	171301	171880	1.23	1.44E-01	transcription_start_site	+	172382	172382	-791	15209 AN3270
2417	CONTIG54	171301	171880	1.23	1.44E-01	transcription_start_site	+	172789	172789	-1198	15210 AN3270
887	CONTIG54	171981	172256	2.01	8.81E-03	transcription_start_site	+	174919	174919	-2800	15211 AN3271
887	CONTIG54	171981	172256	2.01	8.81E-03	transcription_start_site	+	174992	174992	-2873	15212 AN3271
887	CONTIG54	171981	172256	2.01	8.81E-03	transcription_start_site	+	175168	175168	-3049	15213 AN3271
887	CONTIG54	171981	172256	2.01	8.81E-03	transcription_start_site	+	176403	176403	-4284	15214 AN3271
2417	CONTIG54	171301	171880	1.23	1.44E-01	transcription_start_site	+	174919	174919	-3328	15211 AN3271
2417	CONTIG54	171301	171880	1.23	1.44E-01	transcription_start_site	+	174992	174992	-3401	15212 AN3271
2417	CONTIG54	171301	171880	1.23	1.44E-01	transcription_start_site	+	175168	175168	-3577	15213 AN3271
2417	CONTIG54	171301	171880	1.23	1.44E-01	transcription_start_site	+	176403	176403	-4812	15214 AN3271
175	CONTIG55	8106	8395	2.99	0.00E+00	transcription_start_site	+	10440	10440	-2189	15223 AN3275
175	CONTIG55	8106	8395	2.99	0.00E+00	transcription_start_site	+	10684	10684	-2433	15224 AN3275
175	CONTIG55	8106	8395	2.99	0.00E+00	transcription_start_site	+	10824	10824	-2573	15225 AN3275
175	CONTIG55	8106	8395	2.99	0.00E+00	transcription_start_site	+	11260	11260	-3009	15226 AN3275
175	CONTIG55	8106	8395	2.99	0.00E+00	transcription_start_site	+	11532	11532	-3281	15227 AN3275
175	CONTIG55	8106	8395	2.99	0.00E+00	transcription_start_site	+	11707	11707	-3456	15228 AN3275
175	CONTIG55	8106	8395	2.99	0.00E+00	transcription_start_site	+	12070	12070	-3819	15229 AN3275

175	CONTIG55	8106	8395	2.99	0.00E+00	transcription_start_site	+	12296	12296	-4045	15230 AN3275
1910	CONTIG55	8862	9506	1.4	3.08E-02	transcription_start_site	+	10440	10440	-1256	15223 AN3275
1910	CONTIG55	8862	9506	1.4	3.08E-02	transcription_start_site	+	10684	10684	-1500	15224 AN3275
1910	CONTIG55	8862	9506	1.4	3.08E-02	transcription_start_site	+	10824	10824	-1640	15225 AN3275
1910	CONTIG55	8862	9506	1.4	3.08E-02	transcription_start_site	+	11260	11260	-2076	15226 AN3275
1910	CONTIG55	8862	9506	1.4	3.08E-02	transcription_start_site	+	11532	11532	-2348	15227 AN3275
1910	CONTIG55	8862	9506	1.4	3.08E-02	transcription_start_site	+	11707	11707	-2523	15228 AN3275
1910	CONTIG55	8862	9506	1.4	3.08E-02	transcription_start_site	+	12070	12070	-2886	15229 AN3275
1910	CONTIG55	8862	9506	1.4	3.08E-02	transcription_start_site	+	12296	12296	-3112	15230 AN3275
2104	CONTIG55	7201	7775	1.33	4.36E-02	transcription_start_site	+	10440	10440	-2952	15223 AN3275
2104	CONTIG55	7201	7775	1.33	4.36E-02	transcription_start_site	+	10684	10684	-3196	15224 AN3275
2104	CONTIG55	7201	7775	1.33	4.36E-02	transcription_start_site	+	10824	10824	-3336	15225 AN3275
2104	CONTIG55	7201	7775	1.33	4.36E-02	transcription_start_site	+	11260	11260	-3772	15226 AN3275
2104	CONTIG55	7201	7775	1.33	4.36E-02	transcription_start_site	+	11532	11532	-4044	15227 AN3275
2104	CONTIG55	7201	7775	1.33	4.36E-02	transcription_start_site	+	11707	11707	-4219	15228 AN3275
2104	CONTIG55	7201	7775	1.33	4.36E-02	transcription_start_site	+	12070	12070	-4582	15229 AN3275
2104	CONTIG55	7201	7775	1.33	4.36E-02	transcription_start_site	+	12296	12296	-4808	15230 AN3275
1571	CONTIG55	15978	17077	1.55	3.83E-03	transcription_start_site	-	13970	13970	-2557	15231 AN3276
1571	CONTIG55	15978	17077	1.55	3.83E-03	transcription_start_site	-	13457	13457	-3070	15232 AN3276
1571	CONTIG55	15978	17077	1.55	3.83E-03	transcription_start_site	-	13038	13038	-3489	15233 AN3276
2741	CONTIG55	18094	18353	1.1	1.08E-01	transcription_start_site	-	13970	13970	-4253	15231 AN3276
2741	CONTIG55	18094	18353	1.1	1.08E-01	transcription_start_site	-	13457	13457	-4766	15232 AN3276
1571	CONTIG55	15978	17077	1.55	3.83E-03	transcription_start_site	-	15761	15761	-766	15234 AN3277
1571	CONTIG55	15978	17077	1.55	3.83E-03	transcription_start_site	-	15413	15413	-1114	15235 AN3277
1571	CONTIG55	15978	17077	1.55	3.83E-03	transcription_start_site	-	15287	15287	-1240	15236 AN3277
1571	CONTIG55	15978	17077	1.55	3.83E-03	transcription_start_site	-	15008	15008	-1519	15237 AN3277
2035	CONTIG55	19654	19928	1.36	3.70E-02	transcription_start_site	-	15761	15761	-4030	15234 AN3277
2035	CONTIG55	19654	19928	1.36	3.70E-02	transcription_start_site	-	15413	15413	-4378	15235 AN3277
2035	CONTIG55	19654	19928	1.36	3.70E-02	transcription_start_site	-	15287	15287	-4504	15236 AN3277
2035	CONTIG55	19654	19928	1.36	3.70E-02	transcription_start_site	-	15008	15008	-4783	15237 AN3277
2741	CONTIG55	18094	18353	1.1	1.08E-01	transcription_start_site	-	15761	15761	-2462	15234 AN3277
2741	CONTIG55	18094	18353	1.1	1.08E-01	transcription_start_site	-	15413	15413	-2810	15235 AN3277
2741	CONTIG55	18094	18353	1.1	1.08E-01	transcription_start_site	-	15287	15287	-2936	15236 AN3277
2741	CONTIG55	18094	18353	1.1	1.08E-01	transcription_start_site	-	15008	15008	-3215	15237 AN3277
1571	CONTIG55	15978	17077	1.55	3.83E-03	transcription_start_site	+	16550	16550	-22	15239 AN3278
1571	CONTIG55	15978	17077	1.55	3.83E-03	transcription_start_site	+	16075	16075	452	15238 AN3278
2035	CONTIG55	19654	19928	1.36	3.70E-02	transcription_start_site	-	19265	19265	-526	15240 AN3279
2035	CONTIG55	19654	19928	1.36	3.70E-02	transcription_start_site	-	18580	18580	-1211	15241 AN3279
2741	CONTIG55	18094	18353	1.1	1.08E-01	transcription_start_site	-	18580	18580	356	15241 AN3279
2741	CONTIG55	18094	18353	1.1	1.08E-01	transcription_start_site	-	19265	19265	1041	15240 AN3279
1571	CONTIG55	15978	17077	1.55	3.83E-03	transcription_start_site	+	20165	20165	-3637	15242 AN3280
1571	CONTIG55	15978	17077	1.55	3.83E-03	transcription_start_site	+	20264	20264	-3736	15243 AN3280
1571	CONTIG55	15978	17077	1.55	3.83E-03	transcription_start_site	+	20363	20363	-3835	15244 AN3280
1571	CONTIG55	15978	17077	1.55	3.83E-03	transcription_start_site	+	20903	20903	-4375	15245 AN3280
2035	CONTIG55	19654	19928	1.36	3.70E-02	transcription_start_site	+	20165	20165	-374	15242 AN3280
2035	CONTIG55	19654	19928	1.36	3.70E-02	transcription_start_site	+	20264	20264	-473	15243 AN3280
2035	CONTIG55	19654	19928	1.36	3.70E-02	transcription_start_site	+	20363	20363	-572	15244 AN3280
2035	CONTIG55	19654	19928	1.36	3.70E-02	transcription_start_site	+	20903	20903	-1112	15245 AN3280
2741	CONTIG55	18094	18353	1.1	1.08E-01	transcription_start_site	+	20165	20165	-1941	15242 AN3280
2741	CONTIG55	18094	18353	1.1	1.08E-01	transcription_start_site	+	20264	20264	-2040	15243 AN3280
2741	CONTIG55	18094	18353	1.1	1.08E-01	transcription_start_site	+	20363	20363	-2139	15244 AN3280
2741	CONTIG55	18094	18353	1.1	1.08E-01	transcription_start_site	+	20903	20903	-2679	15245 AN3280
1571	CONTIG55	15978	17077	1.55	3.83E-03	transcription_start_site	+	22054	22054	-5526	15246 AN3281
2035	CONTIG55	19654	19928	1.36	3.70E-02	transcription_start_site	+	22054	22054	-2263	15246 AN3281
2035	CONTIG55	19654	19928	1.36	3.70E-02	transcription_start_site	+	22152	22152	-2361	15247 AN3281
2035	CONTIG55	19654	19928	1.36	3.70E-02	transcription_start_site	+	22302	22302	-2511	15248 AN3281
2035	CONTIG55	19654	19928	1.36	3.70E-02	transcription_start_site	+	22462	22462	-2671	15249 AN3281

2035	CONTIG55	19654	19928	1.36	3.70E-02	transcription_start_site	+	22897	22897	-3106	15250	AN3281
2035	CONTIG55	19654	19928	1.36	3.70E-02	transcription_start_site	+	23054	23054	-3263	15251	AN3281
2035	CONTIG55	19654	19928	1.36	3.70E-02	transcription_start_site	+	23221	23221	-3430	15252	AN3281
2035	CONTIG55	19654	19928	1.36	3.70E-02	transcription_start_site	+	23386	23386	-3595	15253	AN3281
2035	CONTIG55	19654	19928	1.36	3.70E-02	transcription_start_site	+	23711	23711	-3920	15254	AN3281
2035	CONTIG55	19654	19928	1.36	3.70E-02	transcription_start_site	+	23950	23950	-4159	15255	AN3281
2035	CONTIG55	19654	19928	1.36	3.70E-02	transcription_start_site	+	24126	24126	-4335	15256	AN3281
2741	CONTIG55	18094	18353	1.1	1.08E-01	transcription_start_site	+	22054	22054	-3830	15246	AN3281
2741	CONTIG55	18094	18353	1.1	1.08E-01	transcription_start_site	+	22152	22152	-3928	15247	AN3281
2741	CONTIG55	18094	18353	1.1	1.08E-01	transcription_start_site	+	22302	22302	-4078	15248	AN3281
2741	CONTIG55	18094	18353	1.1	1.08E-01	transcription_start_site	+	22462	22462	-4238	15249	AN3281
2741	CONTIG55	18094	18353	1.1	1.08E-01	transcription_start_site	+	22897	22897	-4673	15250	AN3281
2741	CONTIG55	18094	18353	1.1	1.08E-01	transcription_start_site	+	23054	23054	-4830	15251	AN3281
2741	CONTIG55	18094	18353	1.1	1.08E-01	transcription_start_site	+	23221	23221	-4997	15252	AN3281
1325	CONTIG55	40515	40794	1.67	8.81E-03	transcription_start_site	-	40085	40085	-569	15270	AN3287
1325	CONTIG55	40515	40794	1.67	8.81E-03	transcription_start_site	-	39492	39492	-1162	15271	AN3287
2972	CONTIG55	54093	54437	0.95	1.93E-01	transcription_start_site	-	54368	54368	103	15276	AN3290
2972	CONTIG55	54093	54437	0.95	1.93E-01	transcription_start_site	-	54549	54549	284	15275	AN3290
2972	CONTIG55	54093	54437	0.95	1.93E-01	transcription_start_site	-	53935	53935	-330	15277	AN3290
2972	CONTIG55	54093	54437	0.95	1.93E-01	transcription_start_site	-	54665	54665	400	15274	AN3290
2972	CONTIG55	54093	54437	0.95	1.93E-01	transcription_start_site	-	53295	53295	-970	15278	AN3290
2972	CONTIG55	54093	54437	0.95	1.93E-01	transcription_start_site	-	52958	52958	-1307	15279	AN3290
2972	CONTIG55	54093	54437	0.95	1.93E-01	transcription_start_site	-	52701	52701	-1564	15280	AN3290
2972	CONTIG55	54093	54437	0.95	1.93E-01	transcription_start_site	-	52456	52456	-1809	15281	AN3290
2972	CONTIG55	54093	54437	0.95	1.93E-01	transcription_start_site	-	51698	51698	-2567	15282	AN3290
2972	CONTIG55	54093	54437	0.95	1.93E-01	transcription_start_site	+	55311	55311	-1046	15283	AN3291
2972	CONTIG55	54093	54437	0.95	1.93E-01	transcription_start_site	+	55712	55712	-1447	15284	AN3291
2972	CONTIG55	54093	54437	0.95	1.93E-01	transcription_start_site	+	56083	56083	-1818	15285	AN3291
1054	CONTIG55	60453	60742	1.86	3.61E-03	transcription_start_site	-	57789	57789	-2808	15286	AN3292
1054	CONTIG55	60453	60742	1.86	3.61E-03	transcription_start_site	-	57654	57654	-2943	15287	AN3292
1054	CONTIG55	60453	60742	1.86	3.61E-03	transcription_start_site	-	57399	57399	-3198	15288	AN3292
2972	CONTIG55	54093	54437	0.95	1.93E-01	transcription_start_site	+	59257	59257	-4992	15289	AN3293
1054	CONTIG55	60453	60742	1.86	3.61E-03	transcription_start_site	+	60731	60731	-133	15290	AN3294
1054	CONTIG55	60453	60742	1.86	3.61E-03	transcription_start_site	+	61068	61068	-470	15291	AN3294
1054	CONTIG55	60453	60742	1.86	3.61E-03	transcription_start_site	+	61165	61165	-567	15292	AN3294
1054	CONTIG55	60453	60742	1.86	3.61E-03	transcription_start_site	+	61273	61273	-675	15293	AN3294
1054	CONTIG55	60453	60742	1.86	3.61E-03	transcription_start_site	+	61515	61515	-917	15294	AN3294
1054	CONTIG55	60453	60742	1.86	3.61E-03	transcription_start_site	+	61644	61644	-1046	15295	AN3294
1054	CONTIG55	60453	60742	1.86	3.61E-03	transcription_start_site	+	61793	61793	-1195	15296	AN3294
1054	CONTIG55	60453	60742	1.86	3.61E-03	transcription_start_site	+	63160	63160	-2562	15297	AN3295
2578	CONTIG55	63153	63442	1.17	8.01E-02	transcription_start_site	+	63160	63160	137	15297	AN3295
1054	CONTIG55	60453	60742	1.86	3.61E-03	transcription_start_site	+	64417	64417	-3819	15298	AN3296
1054	CONTIG55	60453	60742	1.86	3.61E-03	transcription_start_site	+	64731	64731	-4133	15299	AN3296
2578	CONTIG55	63153	63442	1.17	8.01E-02	transcription_start_site	+	64417	64417	-1119	15298	AN3296
2578	CONTIG55	63153	63442	1.17	8.01E-02	transcription_start_site	+	64731	64731	-1433	15299	AN3296
2578	CONTIG55	63153	63442	1.17	8.01E-02	transcription_start_site	+	67748	67748	-4450	15302	AN3298
2578	CONTIG55	63153	63442	1.17	8.01E-02	transcription_start_site	+	68115	68115	-4817	15303	AN3298
2578	CONTIG55	63153	63442	1.17	8.01E-02	transcription_start_site	+	68279	68279	-4981	15304	AN3298
2742	CONTIG55	73428	73718	1.1	1.08E-01	transcription_start_site	-	69711	69711	-3862	15305	AN3299
2742	CONTIG55	73428	73718	1.1	1.08E-01	transcription_start_site	-	69502	69502	-4071	15306	AN3299
2742	CONTIG55	73428	73718	1.1	1.08E-01	transcription_start_site	+	72752	72752	821	15310	AN3300
2742	CONTIG55	73428	73718	1.1	1.08E-01	transcription_start_site	+	73787	73787	-214	15311	AN3301
2742	CONTIG55	73428	73718	1.1	1.08E-01	transcription_start_site	+	74014	74014	-441	15312	AN3301
2742	CONTIG55	73428	73718	1.1	1.08E-01	transcription_start_site	+	74882	74882	-1309	15313	AN3301
2742	CONTIG55	73428	73718	1.1	1.08E-01	transcription_start_site	+	75504	75504	-1931	15314	AN3301
2742	CONTIG55	73428	73718	1.1	1.08E-01	transcription_start_site	+	76746	76746	-3173	15315	AN3302
2742	CONTIG55	73428	73718	1.1	1.08E-01	transcription_start_site	+	77491	77491	-3918	15316	AN3302

270	CONTIG55	100054	100328	2.8	0.00E+00	transcription_start_site	-	99189	99189	-1002	15338	AN3307
270	CONTIG55	100054	100328	2.8	0.00E+00	transcription_start_site	-	98759	98759	-1432	15339	AN3307
270	CONTIG55	100054	100328	2.8	0.00E+00	transcription_start_site	-	98511	98511	-1680	15340	AN3307
2469	CONTIG55	97369	97628	1.21	6.84E-02	transcription_start_site	-	98511	98511	1012	15340	AN3307
2469	CONTIG55	97369	97628	1.21	6.84E-02	transcription_start_site	-	92421	92421	-5077	15341	AN3307
2743	CONTIG55	93171	94775	1.1	1.08E-01	transcription_start_site	-	92421	92421	-1552	15341	AN3307
2873	CONTIG55	101639	102068	1.02	1.44E-01	transcription_start_site	-	99189	99189	-2664	15338	AN3307
2873	CONTIG55	101639	102068	1.02	1.44E-01	transcription_start_site	-	98759	98759	-3094	15339	AN3307
2873	CONTIG55	101639	102068	1.02	1.44E-01	transcription_start_site	-	98511	98511	-3342	15340	AN3307
270	CONTIG55	100054	100328	2.8	0.00E+00	transcription_start_site	+	102033	102033	-1842	15342	AN3308
270	CONTIG55	100054	100328	2.8	0.00E+00	transcription_start_site	+	103153	103153	-2962	15343	AN3308
2469	CONTIG55	97369	97628	1.21	6.84E-02	transcription_start_site	+	102033	102033	-4534	15342	AN3308
2873	CONTIG55	101639	102068	1.02	1.44E-01	transcription_start_site	+	102033	102033	-179	15342	AN3308
2873	CONTIG55	101639	102068	1.02	1.44E-01	transcription_start_site	+	103153	103153	-1299	15343	AN3308
270	CONTIG55	100054	100328	2.8	0.00E+00	transcription_start_site	+	104572	104572	-4381	15344	AN3309
270	CONTIG55	100054	100328	2.8	0.00E+00	transcription_start_site	+	104690	104690	-4499	15345	AN3309
270	CONTIG55	100054	100328	2.8	0.00E+00	transcription_start_site	+	105157	105157	-4966	15346	AN3309
1021	CONTIG55	104254	104753	1.89	3.03E-03	transcription_start_site	+	104572	104572	-68	15344	AN3309
1021	CONTIG55	104254	104753	1.89	3.03E-03	transcription_start_site	+	104690	104690	-186	15345	AN3309
1021	CONTIG55	104254	104753	1.89	3.03E-03	transcription_start_site	+	105157	105157	-653	15346	AN3309
1021	CONTIG55	104254	104753	1.89	3.03E-03	transcription_start_site	+	105895	105895	-1391	15347	AN3309
1021	CONTIG55	104254	104753	1.89	3.03E-03	transcription_start_site	+	105974	105974	-1470	15348	AN3309
1021	CONTIG55	104254	104753	1.89	3.03E-03	transcription_start_site	+	106254	106254	-1750	15349	AN3309
1021	CONTIG55	104254	104753	1.89	3.03E-03	transcription_start_site	+	106698	106698	-2194	15350	AN3309
2655	CONTIG55	106279	106638	1.14	9.24E-02	transcription_start_site	+	106254	106254	204	15349	AN3309
2655	CONTIG55	106279	106638	1.14	9.24E-02	transcription_start_site	+	106698	106698	-239	15350	AN3309
2655	CONTIG55	106279	106638	1.14	9.24E-02	transcription_start_site	+	105974	105974	484	15348	AN3309
2655	CONTIG55	106279	106638	1.14	9.24E-02	transcription_start_site	+	105895	105895	563	15347	AN3309
2873	CONTIG55	101639	102068	1.02	1.44E-01	transcription_start_site	+	104572	104572	-2718	15344	AN3309
2873	CONTIG55	101639	102068	1.02	1.44E-01	transcription_start_site	+	104690	104690	-2836	15345	AN3309
2873	CONTIG55	101639	102068	1.02	1.44E-01	transcription_start_site	+	105157	105157	-3303	15346	AN3309
2873	CONTIG55	101639	102068	1.02	1.44E-01	transcription_start_site	+	105895	105895	-4041	15347	AN3309
2873	CONTIG55	101639	102068	1.02	1.44E-01	transcription_start_site	+	105974	105974	-4120	15348	AN3309
2873	CONTIG55	101639	102068	1.02	1.44E-01	transcription_start_site	+	106254	106254	-4400	15349	AN3309
2873	CONTIG55	101639	102068	1.02	1.44E-01	transcription_start_site	+	106698	106698	-4844	15350	AN3309
2941	CONTIG55	137327	137616	0.98	1.66E-01	transcription_start_site	-	134744	134744	-2727	15382	AN3318
2941	CONTIG55	137327	137616	0.98	1.66E-01	transcription_start_site	-	134518	134518	-2953	15383	AN3318
2941	CONTIG55	137327	137616	0.98	1.66E-01	transcription_start_site	-	134415	134415	-3056	15384	AN3318
2941	CONTIG55	137327	137616	0.98	1.66E-01	transcription_start_site	-	134282	134282	-3189	15385	AN3318
2941	CONTIG55	137327	137616	0.98	1.66E-01	transcription_start_site	-	133203	133203	-4268	15386	AN3318
2941	CONTIG55	137327	137616	0.98	1.66E-01	transcription_start_site	-	132928	132928	-4543	15387	AN3318
2941	CONTIG55	137327	137616	0.98	1.66E-01	transcription_start_site	-	132410	132410	-5061	15388	AN3318
2941	CONTIG55	137327	137616	0.98	1.66E-01	transcription_start_site	+	137154	137154	317	15396	AN3319
2941	CONTIG55	137327	137616	0.98	1.66E-01	transcription_start_site	+	136921	136921	550	15395	AN3319
2941	CONTIG55	137327	137616	0.98	1.66E-01	transcription_start_site	+	136742	136742	729	15394	AN3319
1055	CONTIG55	180906	181255	1.86	3.61E-03	transcription_start_site	+	183328	183328	-2247	15421	AN3329
2939	CONTIG55	189546	190475	0.98	1.50E-01	transcription_start_site	-	189408	189408	-602	15425	AN3330
2939	CONTIG55	189546	190475	0.98	1.50E-01	transcription_start_site	-	190692	190692	681	15424	AN3330
2939	CONTIG55	189546	190475	0.98	1.50E-01	transcription_start_site	-	191102	191102	1091	15423	AN3330
2656	CONTIG55	204690	205051	1.14	9.24E-02	transcription_start_site	-	204625	204625	-245	15432	AN3334
2656	CONTIG55	204690	205051	1.14	9.24E-02	transcription_start_site	-	204074	204074	-796	15433	AN3334
2656	CONTIG55	204690	205051	1.14	9.24E-02	transcription_start_site	-	203728	203728	-1142	15434	AN3334
2656	CONTIG55	204690	205051	1.14	9.24E-02	transcription_start_site	+	208427	208427	-3556	15435	AN3335
2656	CONTIG55	204690	205051	1.14	9.24E-02	transcription_start_site	+	208880	208880	-4009	15436	AN3335
2348	CONTIG55	236788	237054	1.25	5.88E-02	transcription_start_site	-	236412	236412	-509	15453	AN3342
2348	CONTIG55	236788	237054	1.25	5.88E-02	transcription_start_site	-	236103	236103	-818	15454	AN3342
2348	CONTIG55	236788	237054	1.25	5.88E-02	transcription_start_site	-	233605	233605	-3316	15455	AN3342

2348	CONTIG55	236788	237054	1.25	5.88E-02	transcription_start_site	-	233290	233290	-3631	15456	AN3342
2348	CONTIG55	236788	237054	1.25	5.88E-02	transcription_start_site	-	237995	237995	1074	15463	AN3343
433	CONTIG55	264692	264957	2.54	0.00E+00	transcription_start_site	-	262712	262712	-2112	15490	AN3349
433	CONTIG55	264692	264957	2.54	0.00E+00	transcription_start_site	-	262448	262448	-2376	15491	AN3349
433	CONTIG55	264692	264957	2.54	0.00E+00	transcription_start_site	-	262376	262376	-2448	15492	AN3349
433	CONTIG55	264692	264957	2.54	0.00E+00	transcription_start_site	-	262171	262171	-2653	15493	AN3349
433	CONTIG55	264692	264957	2.54	0.00E+00	transcription_start_site	-	261715	261715	-3109	15494	AN3349
433	CONTIG55	264692	264957	2.54	0.00E+00	transcription_start_site	-	261053	261053	-3771	15495	AN3349
433	CONTIG55	264692	264957	2.54	0.00E+00	transcription_start_site	-	260786	260786	-4038	15496	AN3349
433	CONTIG55	264692	264957	2.54	0.00E+00	transcription_start_site	-	260722	260722	-4102	15497	AN3349
433	CONTIG55	264692	264957	2.54	0.00E+00	transcription_start_site	-	260482	260482	-4342	15498	AN3349
433	CONTIG55	264692	264957	2.54	0.00E+00	transcription_start_site	-	260336	260336	-4488	15499	AN3349
433	CONTIG55	264692	264957	2.54	0.00E+00	transcription_start_site	-	260203	260203	-4621	15500	AN3349
433	CONTIG55	264692	264957	2.54	0.00E+00	transcription_start_site	-	260082	260082	-4742	15501	AN3349
433	CONTIG55	264692	264957	2.54	0.00E+00	transcription_start_site	-	259837	259837	-4987	15502	AN3349
433	CONTIG55	264692	264957	2.54	0.00E+00	transcription_start_site	-	259700	259700	-5124	15503	AN3349
2349	CONTIG55	275628	276127	1.25	5.88E-02	transcription_start_site	-	276262	276262	384	15524	AN3353
2349	CONTIG55	275628	276127	1.25	5.88E-02	transcription_start_site	-	275420	275420	-457	15525	AN3353
2349	CONTIG55	275628	276127	1.25	5.88E-02	transcription_start_site	+	278486	278486	-2608	15530	AN3355
2349	CONTIG55	275628	276127	1.25	5.88E-02	transcription_start_site	+	278720	278720	-2842	15531	AN3355
2349	CONTIG55	275628	276127	1.25	5.88E-02	transcription_start_site	+	279009	279009	-3131	15532	AN3355
2349	CONTIG55	275628	276127	1.25	5.88E-02	transcription_start_site	+	279334	279334	-3456	15533	AN3355
2349	CONTIG55	275628	276127	1.25	5.88E-02	transcription_start_site	+	279486	279486	-3608	15534	AN3355
2349	CONTIG55	275628	276127	1.25	5.88E-02	transcription_start_site	+	280558	280558	-4680	15535	AN3355
2349	CONTIG55	275628	276127	1.25	5.88E-02	transcription_start_site	+	280847	280847	-4969	15536	AN3355
535	CONTIG55	291845	292265	2.39	0.00E+00	transcription_start_site	-	291005	291005	-1050	15543	AN3357
535	CONTIG55	291845	292265	2.39	0.00E+00	transcription_start_site	-	289579	289579	-2476	15544	AN3357
535	CONTIG55	291845	292265	2.39	0.00E+00	transcription_start_site	+	293530	293530	-1475	15545	AN3358
535	CONTIG55	291845	292265	2.39	0.00E+00	transcription_start_site	+	294365	294365	-2310	15546	AN3358
535	CONTIG55	291845	292265	2.39	0.00E+00	transcription_start_site	+	294565	294565	-2510	15547	AN3358
156	CONTIG55	306085	306516	3.03	0.00E+00	transcription_start_site	-	301939	301939	-4361	15549	AN3360
2808	CONTIG55	300985	301489	1.06	1.25E-01	transcription_start_site	-	300675	300675	-562	15550	AN3360
2808	CONTIG55	300985	301489	1.06	1.25E-01	transcription_start_site	-	301939	301939	702	15549	AN3360
2808	CONTIG55	300985	301489	1.06	1.25E-01	transcription_start_site	-	300529	300529	-708	15551	AN3360
156	CONTIG55	306085	306516	3.03	0.00E+00	transcription_start_site	-	306023	306023	-277	15552	AN3361
156	CONTIG55	306085	306516	3.03	0.00E+00	transcription_start_site	-	305686	305686	-614	15553	AN3361
156	CONTIG55	306085	306516	3.03	0.00E+00	transcription_start_site	-	305573	305573	-727	15554	AN3361
156	CONTIG55	306085	306516	3.03	0.00E+00	transcription_start_site	-	305037	305037	-1263	15555	AN3361
156	CONTIG55	306085	306516	3.03	0.00E+00	transcription_start_site	-	304840	304840	-1460	15556	AN3361
488	CONTIG55	328053	328332	2.46	0.00E+00	transcription_start_site	-	324278	324278	-3914	15565	AN3367
488	CONTIG55	328053	328332	2.46	0.00E+00	transcription_start_site	-	323939	323939	-4253	15566	AN3367
488	CONTIG55	328053	328332	2.46	0.00E+00	transcription_start_site	-	327238	327238	-954	15567	AN3368
488	CONTIG55	328053	328332	2.46	0.00E+00	transcription_start_site	-	327046	327046	-1146	15568	AN3368
488	CONTIG55	328053	328332	2.46	0.00E+00	transcription_start_site	-	326041	326041	-2151	15569	AN3368
488	CONTIG55	328053	328332	2.46	0.00E+00	transcription_start_site	-	325558	325558	-2634	15570	AN3368
488	CONTIG55	328053	328332	2.46	0.00E+00	transcription_start_site	-	325251	325251	-2941	15571	AN3368
488	CONTIG55	328053	328332	2.46	0.00E+00	transcription_start_site	+	330682	330682	-2489	15572	AN3369
488	CONTIG55	328053	328332	2.46	0.00E+00	transcription_start_site	+	330967	330967	-2774	15573	AN3369
488	CONTIG55	328053	328332	2.46	0.00E+00	transcription_start_site	+	332115	332115	-3922	15574	AN3369
1820	CONTIG55	357538	357887	1.44	2.62E-02	transcription_start_site	+	356915	356915	797	15590	AN3378
1820	CONTIG55	357538	357887	1.44	2.62E-02	transcription_start_site	+	359571	359571	-1858	15591	AN3379
1820	CONTIG55	357538	357887	1.44	2.62E-02	transcription_start_site	+	360878	360878	-3165	15592	AN3379
2942	CONTIG55	381394	381953	0.98	1.66E-01	transcription_start_site	-	379509	379509	-2164	15608	AN3384
2942	CONTIG55	381394	381953	0.98	1.66E-01	transcription_start_site	+	381785	381785	-111	15610	AN3385
2942	CONTIG55	381394	381953	0.98	1.66E-01	transcription_start_site	+	381254	381254	419	15609	AN3385
2942	CONTIG55	381394	381953	0.98	1.66E-01	transcription_start_site	+	382371	382371	-697	15611	AN3385
2942	CONTIG55	381394	381953	0.98	1.66E-01	transcription_start_site	+	383877	383877	-2203	15612	AN3386

2942	CONTIG55	381394	381953	0.98	1.66E-01	transcription_start_site	+	384583	384583	-2909	15613 AN3386
296	CONTIG55	393234	393751	2.76	0.00E+00	transcription_start_site	-	393119	393119	-373	15615 AN3387
296	CONTIG55	393234	393751	2.76	0.00E+00	transcription_start_site	-	392440	392440	-1052	15616 AN3387
1665	CONTIG55	392264	392918	1.51	9.21E-03	transcription_start_site	-	392440	392440	-151	15616 AN3387
1665	CONTIG55	392264	392918	1.51	9.21E-03	transcription_start_site	-	393119	393119	528	15615 AN3387
1667	CONTIG55	394819	395170	1.51	1.84E-02	transcription_start_site	-	393119	393119	-1875	15615 AN3387
1667	CONTIG55	394819	395170	1.51	1.84E-02	transcription_start_site	-	392440	392440	-2554	15616 AN3387
296	CONTIG55	393234	393751	2.76	0.00E+00	transcription_start_site	+	398500	398500	-5007	15617 AN3388
1667	CONTIG55	394819	395170	1.51	1.84E-02	transcription_start_site	+	398500	398500	-3505	15617 AN3388
1667	CONTIG55	394819	395170	1.51	1.84E-02	transcription_start_site	+	398823	398823	-3828	15618 AN3388
1667	CONTIG55	394819	395170	1.51	1.84E-02	transcription_start_site	+	398996	398996	-4001	15619 AN3388
1668	CONTIG55	423983	424339	1.51	1.84E-02	transcription_start_site	-	422169	422169	-1992	15638 AN3394
1668	CONTIG55	423983	424339	1.51	1.84E-02	transcription_start_site	-	421537	421537	-2624	15639 AN3394
1668	CONTIG55	423983	424339	1.51	1.84E-02	transcription_start_site	+	423636	423636	525	15643 AN3395
1668	CONTIG55	423983	424339	1.51	1.84E-02	transcription_start_site	+	423493	423493	668	15642 AN3395
1668	CONTIG55	423983	424339	1.51	1.84E-02	transcription_start_site	+	423038	423038	1123	15641 AN3395
1668	CONTIG55	423983	424339	1.51	1.84E-02	transcription_start_site	+	425016	425016	-855	15644 AN3396
1668	CONTIG55	423983	424339	1.51	1.84E-02	transcription_start_site	+	425793	425793	-1632	15645 AN3396
1668	CONTIG55	423983	424339	1.51	1.84E-02	transcription_start_site	+	427065	427065	-2904	15646 AN3396
2805	CONTIG55	435828	436867	1.06	1.07E-01	transcription_start_site	+	435030	435030	1317	15652 AN3398
2805	CONTIG55	435828	436867	1.06	1.07E-01	transcription_start_site	+	434866	434866	1481	15651 AN3398
2805	CONTIG55	435828	436867	1.06	1.07E-01	transcription_start_site	+	436259	436259	88	15654 AN3399
2805	CONTIG55	435828	436867	1.06	1.07E-01	transcription_start_site	+	436464	436464	-116	15655 AN3399
2805	CONTIG55	435828	436867	1.06	1.07E-01	transcription_start_site	+	435764	435764	583	15653 AN3399
2805	CONTIG55	435828	436867	1.06	1.07E-01	transcription_start_site	+	437349	437349	-1001	15656 AN3399
2805	CONTIG55	435828	436867	1.06	1.07E-01	transcription_start_site	+	437722	437722	-1374	15657 AN3400
2805	CONTIG55	435828	436867	1.06	1.07E-01	transcription_start_site	+	438301	438301	-1953	15658 AN3400
2805	CONTIG55	435828	436867	1.06	1.07E-01	transcription_start_site	+	438574	438574	-2226	15659 AN3400
2805	CONTIG55	435828	436867	1.06	1.07E-01	transcription_start_site	+	441631	441631	-5283	15664 AN3402
2805	CONTIG55	435828	436867	1.06	1.07E-01	transcription_start_site	+	441842	441842	-5494	15665 AN3402
2346	CONTIG55	449329	450428	1.25	3.61E-02	transcription_start_site	-	446161	446161	-3717	15673 AN3403
2346	CONTIG55	449329	450428	1.25	3.61E-02	transcription_start_site	+	449352	449352	526	15677 AN3405
1821	CONTIG55	455404	455678	1.44	2.62E-02	transcription_start_site	-	452611	452611	-2930	15678 AN3406
1821	CONTIG55	455404	455678	1.44	2.62E-02	transcription_start_site	-	452386	452386	-3155	15679 AN3406
1821	CONTIG55	455404	455678	1.44	2.62E-02	transcription_start_site	-	451862	451862	-3679	15680 AN3406
1821	CONTIG55	455404	455678	1.44	2.62E-02	transcription_start_site	+	455050	455050	491	15683 AN3407
1821	CONTIG55	455404	455678	1.44	2.62E-02	transcription_start_site	+	454502	454502	1039	15682 AN3407
2346	CONTIG55	449329	450428	1.25	3.61E-02	transcription_start_site	+	454238	454238	-4359	15681 AN3407
2346	CONTIG55	449329	450428	1.25	3.61E-02	transcription_start_site	+	454502	454502	-4623	15682 AN3407
2346	CONTIG55	449329	450428	1.25	3.61E-02	transcription_start_site	+	455050	455050	-5171	15683 AN3407
1393	CONTIG55	459980	460269	1.63	1.11E-02	transcription_start_site	-	459826	459826	-298	15684 AN3408
1393	CONTIG55	459980	460269	1.63	1.11E-02	transcription_start_site	-	459554	459554	-570	15685 AN3408
1393	CONTIG55	459980	460269	1.63	1.11E-02	transcription_start_site	-	459157	459157	-967	15686 AN3408
1393	CONTIG55	459980	460269	1.63	1.11E-02	transcription_start_site	-	458888	458888	-1236	15687 AN3408
2463	CONTIG55	460516	461611	1.21	4.42E-02	transcription_start_site	-	459826	459826	-1237	15684 AN3408
2463	CONTIG55	460516	461611	1.21	4.42E-02	transcription_start_site	-	459554	459554	-1509	15685 AN3408
2463	CONTIG55	460516	461611	1.21	4.42E-02	transcription_start_site	-	459157	459157	-1906	15686 AN3408
2463	CONTIG55	460516	461611	1.21	4.42E-02	transcription_start_site	-	458888	458888	-2175	15687 AN3408
1393	CONTIG55	459980	460269	1.63	1.11E-02	transcription_start_site	+	462473	462473	-2348	15688 AN3409
1393	CONTIG55	459980	460269	1.63	1.11E-02	transcription_start_site	+	462541	462541	-2416	15689 AN3409
1393	CONTIG55	459980	460269	1.63	1.11E-02	transcription_start_site	+	462732	462732	-2607	15690 AN3409
1393	CONTIG55	459980	460269	1.63	1.11E-02	transcription_start_site	+	463422	463422	-3297	15691 AN3409
2463	CONTIG55	460516	461611	1.21	4.42E-02	transcription_start_site	+	462473	462473	-1409	15688 AN3409
2463	CONTIG55	460516	461611	1.21	4.42E-02	transcription_start_site	+	462541	462541	-1477	15689 AN3409
2463	CONTIG55	460516	461611	1.21	4.42E-02	transcription_start_site	+	462732	462732	-1668	15690 AN3409
2463	CONTIG55	460516	461611	1.21	4.42E-02	transcription_start_site	+	463422	463422	-2358	15691 AN3409
1166	CONTIG55	469505	469944	1.78	5.20E-03	transcription_start_site	-	465990	465990	-3734	15692 AN3410

1166	CONTIG55	469505	469944	1.78	5.20E-03	transcription_start_site	-	465757	465757	-3967	15693	AN3410
1166	CONTIG55	469505	469944	1.78	5.20E-03	transcription_start_site	-	465635	465635	-4089	15694	AN3410
1166	CONTIG55	469505	469944	1.78	5.20E-03	transcription_start_site	-	465291	465291	-4433	15695	AN3410
2657	CONTIG55	470415	470684	1.14	9.24E-02	transcription_start_site	-	465990	465990	-4559	15692	AN3410
2657	CONTIG55	470415	470684	1.14	9.24E-02	transcription_start_site	-	465757	465757	-4792	15693	AN3410
2657	CONTIG55	470415	470684	1.14	9.24E-02	transcription_start_site	-	465635	465635	-4914	15694	AN3410
1166	CONTIG55	469505	469944	1.78	5.20E-03	transcription_start_site	-	467718	467718	-2006	15697	AN3411
1166	CONTIG55	469505	469944	1.78	5.20E-03	transcription_start_site	-	467465	467465	-2259	15698	AN3411
1166	CONTIG55	469505	469944	1.78	5.20E-03	transcription_start_site	-	467333	467333	-2391	15699	AN3411
2657	CONTIG55	470415	470684	1.14	9.24E-02	transcription_start_site	-	467718	467718	-2831	15697	AN3411
2657	CONTIG55	470415	470684	1.14	9.24E-02	transcription_start_site	-	467465	467465	-3084	15698	AN3411
2657	CONTIG55	470415	470684	1.14	9.24E-02	transcription_start_site	-	467333	467333	-3216	15699	AN3411
1166	CONTIG55	469505	469944	1.78	5.20E-03	transcription_start_site	-	469266	469266	-458	15700	AN3412
1166	CONTIG55	469505	469944	1.78	5.20E-03	transcription_start_site	-	469193	469193	-531	15701	AN3412
1166	CONTIG55	469505	469944	1.78	5.20E-03	transcription_start_site	-	469064	469064	-660	15702	AN3412
1166	CONTIG55	469505	469944	1.78	5.20E-03	transcription_start_site	-	468867	468867	-857	15703	AN3412
2657	CONTIG55	470415	470684	1.14	9.24E-02	transcription_start_site	-	469266	469266	-1283	15700	AN3412
2657	CONTIG55	470415	470684	1.14	9.24E-02	transcription_start_site	-	469193	469193	-1356	15701	AN3412
2657	CONTIG55	470415	470684	1.14	9.24E-02	transcription_start_site	-	469064	469064	-1485	15702	AN3412
2657	CONTIG55	470415	470684	1.14	9.24E-02	transcription_start_site	-	468867	468867	-1682	15703	AN3412
1166	CONTIG55	469505	469944	1.78	5.20E-03	transcription_start_site	+	469790	469790	-65	15704	AN3413
1166	CONTIG55	469505	469944	1.78	5.20E-03	transcription_start_site	+	469987	469987	-262	15705	AN3413
1166	CONTIG55	469505	469944	1.78	5.20E-03	transcription_start_site	+	470256	470256	-531	15706	AN3413
1166	CONTIG55	469505	469944	1.78	5.20E-03	transcription_start_site	+	470563	470563	-838	15707	AN3413
2657	CONTIG55	470415	470684	1.14	9.24E-02	transcription_start_site	+	470563	470563	-13	15707	AN3413
2657	CONTIG55	470415	470684	1.14	9.24E-02	transcription_start_site	+	470256	470256	293	15706	AN3413
2657	CONTIG55	470415	470684	1.14	9.24E-02	transcription_start_site	+	469987	469987	562	15705	AN3413
2657	CONTIG55	470415	470684	1.14	9.24E-02	transcription_start_site	+	469790	469790	759	15704	AN3413
1166	CONTIG55	469505	469944	1.78	5.20E-03	transcription_start_site	+	472116	472116	-2391	15708	AN3414
1166	CONTIG55	469505	469944	1.78	5.20E-03	transcription_start_site	+	472456	472456	-2731	15709	AN3414
1166	CONTIG55	469505	469944	1.78	5.20E-03	transcription_start_site	+	472672	472672	-2947	15710	AN3414
1166	CONTIG55	469505	469944	1.78	5.20E-03	transcription_start_site	+	473110	473110	-3385	15711	AN3414
2657	CONTIG55	470415	470684	1.14	9.24E-02	transcription_start_site	+	472116	472116	-1566	15708	AN3414
2657	CONTIG55	470415	470684	1.14	9.24E-02	transcription_start_site	+	472456	472456	-1906	15709	AN3414
2657	CONTIG55	470415	470684	1.14	9.24E-02	transcription_start_site	+	472672	472672	-2122	15710	AN3414
2657	CONTIG55	470415	470684	1.14	9.24E-02	transcription_start_site	+	473110	473110	-2560	15711	AN3414
152	CONTIG56	35711	36745	3.04	0.00E+00	transcription_start_site	-	31408	31408	-4820	15742	AN3431
152	CONTIG56	35711	36745	3.04	0.00E+00	transcription_start_site	-	31179	31179	-5049	15743	AN3431
152	CONTIG56	35711	36745	3.04	0.00E+00	transcription_start_site	-	31068	31068	-5160	15744	AN3431
152	CONTIG56	35711	36745	3.04	0.00E+00	transcription_start_site	-	34687	34687	-1541	15745	AN3432
152	CONTIG56	35711	36745	3.04	0.00E+00	transcription_start_site	-	34138	34138	-2090	15746	AN3432
337	CONTIG56	39017	39430	2.71	3.61E-03	transcription_start_site	-	34687	34687	-4536	15745	AN3432
337	CONTIG56	39017	39430	2.71	3.61E-03	transcription_start_site	-	34138	34138	-5085	15746	AN3432
915	CONTIG56	37962	38611	1.99	2.18E-02	transcription_start_site	-	34687	34687	-3599	15745	AN3432
915	CONTIG56	37962	38611	1.99	2.18E-02	transcription_start_site	-	34138	34138	-4148	15746	AN3432
337	CONTIG56	39017	39430	2.71	3.61E-03	transcription_start_site	-	38713	38713	-510	15747	AN3433
337	CONTIG56	39017	39430	2.71	3.61E-03	transcription_start_site	-	38544	38544	-679	15748	AN3433
337	CONTIG56	39017	39430	2.71	3.61E-03	transcription_start_site	-	38385	38385	-838	15749	AN3433
915	CONTIG56	37962	38611	1.99	2.18E-02	transcription_start_site	-	38385	38385	98	15749	AN3433
915	CONTIG56	37962	38611	1.99	2.18E-02	transcription_start_site	-	38544	38544	257	15748	AN3433
915	CONTIG56	37962	38611	1.99	2.18E-02	transcription_start_site	-	38713	38713	426	15747	AN3433
337	CONTIG56	39017	39430	2.71	3.61E-03	transcription_start_site	+	43563	43563	-4339	15754	AN3435
337	CONTIG56	39017	39430	2.71	3.61E-03	transcription_start_site	+	44189	44189	-4965	15755	AN3435
337	CONTIG56	39017	39430	2.71	3.61E-03	transcription_start_site	+	44335	44335	-5111	15756	AN3435
915	CONTIG56	37962	38611	1.99	2.18E-02	transcription_start_site	+	43563	43563	-5276	15754	AN3435
892	CONTIG57	9621	9892	2.01	4.36E-02	transcription_start_site	-	7737	7737	-2019	15761	AN3437
892	CONTIG57	9621	9892	2.01	4.36E-02	transcription_start_site	-	7209	7209	-2547	15762	AN3437

104	CONTIG57	14717	15137	3.22	9.51E-04	transcription_start_site	-	14504	14504	-423	15763 AN3438
104	CONTIG57	14717	15137	3.22	9.51E-04	transcription_start_site	-	11929	11929	-2998	15764 AN3438
104	CONTIG57	14717	15137	3.22	9.51E-04	transcription_start_site	+	16135	16135	-1208	15765 AN3439
104	CONTIG57	14717	15137	3.22	9.51E-04	transcription_start_site	+	16299	16299	-1372	15766 AN3439
104	CONTIG57	14717	15137	3.22	9.51E-04	transcription_start_site	+	16456	16456	-1529	15767 AN3439
104	CONTIG57	14717	15137	3.22	9.51E-04	transcription_start_site	+	16517	16517	-1590	15768 AN3439
104	CONTIG57	14717	15137	3.22	9.51E-04	transcription_start_site	+	16925	16925	-1998	15769 AN3439
477	CONTIG57	24620	25042	2.48	1.11E-02	transcription_start_site	-	24408	24408	-423	15770 AN3441
477	CONTIG57	24620	25042	2.48	1.11E-02	transcription_start_site	-	24032	24032	-799	15771 AN3441
477	CONTIG57	24620	25042	2.48	1.11E-02	transcription_start_site	-	21729	21729	-3102	15772 AN3441
477	CONTIG57	24620	25042	2.48	1.11E-02	transcription_start_site	+	25510	25510	-679	15773 AN3442
1737	CONTIG58	15996	16400	1.48	3.03E-03	transcription_start_site	+	16595	16595	-397	15778 AN3445
1737	CONTIG58	15996	16400	1.48	3.03E-03	transcription_start_site	+	15321	15321	877	15777 AN3445
1737	CONTIG58	15996	16400	1.48	3.03E-03	transcription_start_site	+	15177	15177	1021	15776 AN3445
1737	CONTIG58	15996	16400	1.48	3.03E-03	transcription_start_site	+	17007	17007	-809	15779 AN3446
1737	CONTIG58	15996	16400	1.48	3.03E-03	transcription_start_site	+	17104	17104	-906	15780 AN3446
1737	CONTIG58	15996	16400	1.48	3.03E-03	transcription_start_site	+	17403	17403	-1205	15781 AN3446
1737	CONTIG58	15996	16400	1.48	3.03E-03	transcription_start_site	+	17508	17508	-1310	15782 AN3446
1737	CONTIG58	15996	16400	1.48	3.03E-03	transcription_start_site	+	18076	18076	-1878	15783 AN3446
1737	CONTIG58	15996	16400	1.48	3.03E-03	transcription_start_site	+	18273	18273	-2075	15784 AN3446
1737	CONTIG58	15996	16400	1.48	3.03E-03	transcription_start_site	+	18879	18879	-2681	15785 AN3447
1737	CONTIG58	15996	16400	1.48	3.03E-03	transcription_start_site	+	19195	19195	-2997	15786 AN3447
1737	CONTIG58	15996	16400	1.48	3.03E-03	transcription_start_site	+	19392	19392	-3194	15787 AN3447
1512	CONTIG58	29104	29378	1.57	1.54E-03	transcription_start_site	-	24885	24885	-4356	15788 AN3448
1512	CONTIG58	29104	29378	1.57	1.54E-03	transcription_start_site	-	24616	24616	-4625	15789 AN3448
1512	CONTIG58	29104	29378	1.57	1.54E-03	transcription_start_site	-	24380	24380	-4861	15790 AN3448
3015	CONTIG58	25283	25632	0.89	9.24E-02	transcription_start_site	-	24885	24885	-572	15788 AN3448
3015	CONTIG58	25283	25632	0.89	9.24E-02	transcription_start_site	-	24616	24616	-841	15789 AN3448
3015	CONTIG58	25283	25632	0.89	9.24E-02	transcription_start_site	-	24380	24380	-1077	15790 AN3448
3015	CONTIG58	25283	25632	0.89	9.24E-02	transcription_start_site	-	22978	22978	-2479	15791 AN3448
3015	CONTIG58	25283	25632	0.89	9.24E-02	transcription_start_site	+	25956	25956	-498	15792 AN3449
1512	CONTIG58	29104	29378	1.57	1.54E-03	transcription_start_site	-	28704	28704	-537	15793 AN3450
1512	CONTIG58	29104	29378	1.57	1.54E-03	transcription_start_site	-	28353	28353	-888	15794 AN3450
1512	CONTIG58	29104	29378	1.57	1.54E-03	transcription_start_site	+	29431	29431	-190	15795 AN3451
1512	CONTIG58	29104	29378	1.57	1.54E-03	transcription_start_site	+	29742	29742	-501	15796 AN3451
1512	CONTIG58	29104	29378	1.57	1.54E-03	transcription_start_site	+	30304	30304	-1063	15797 AN3451
1512	CONTIG58	29104	29378	1.57	1.54E-03	transcription_start_site	+	30481	30481	-1240	15798 AN3451
1512	CONTIG58	29104	29378	1.57	1.54E-03	transcription_start_site	+	30682	30682	-1441	15799 AN3451
1512	CONTIG58	29104	29378	1.57	1.54E-03	transcription_start_site	+	33140	33140	-3899	15800 AN3451
3015	CONTIG58	25283	25632	0.89	9.24E-02	transcription_start_site	+	29431	29431	-3973	15795 AN3451
3015	CONTIG58	25283	25632	0.89	9.24E-02	transcription_start_site	+	29742	29742	-4284	15796 AN3451
3015	CONTIG58	25283	25632	0.89	9.24E-02	transcription_start_site	+	30304	30304	-4846	15797 AN3451
3015	CONTIG58	25283	25632	0.89	9.24E-02	transcription_start_site	+	30481	30481	-5023	15798 AN3451
944	CONTIG58	74195	74534	1.96	0.00E+00	transcription_start_site	-	72216	72216	-2148	15848 AN3466
944	CONTIG58	74195	74534	1.96	0.00E+00	transcription_start_site	-	71995	71995	-2369	15849 AN3466
944	CONTIG58	74195	74534	1.96	0.00E+00	transcription_start_site	-	71888	71888	-2476	15850 AN3466
944	CONTIG58	74195	74534	1.96	0.00E+00	transcription_start_site	-	71768	71768	-2596	15851 AN3466
944	CONTIG58	74195	74534	1.96	0.00E+00	transcription_start_site	-	71651	71651	-2713	15852 AN3466
944	CONTIG58	74195	74534	1.96	0.00E+00	transcription_start_site	-	71238	71238	-3126	15853 AN3466
944	CONTIG58	74195	74534	1.96	0.00E+00	transcription_start_site	-	70797	70797	-3567	15854 AN3466
944	CONTIG58	74195	74534	1.96	0.00E+00	transcription_start_site	-	70684	70684	-3680	15855 AN3466
944	CONTIG58	74195	74534	1.96	0.00E+00	transcription_start_site	-	70576	70576	-3788	15856 AN3466
944	CONTIG58	74195	74534	1.96	0.00E+00	transcription_start_site	-	70453	70453	-3911	15857 AN3466
944	CONTIG58	74195	74534	1.96	0.00E+00	transcription_start_site	-	70318	70318	-4046	15858 AN3466
944	CONTIG58	74195	74534	1.96	0.00E+00	transcription_start_site	-	74049	74049	-315	15864 AN3468
944	CONTIG58	74195	74534	1.96	0.00E+00	transcription_start_site	-	73945	73945	-419	15865 AN3468
944	CONTIG58	74195	74534	1.96	0.00E+00	transcription_start_site	-	73799	73799	-565	15866 AN3468

944	CONTIG58	74195	74534	1.96	0.00E+00	transcription_start_site	-	73545	73545	-819	15867	AN3468
944	CONTIG58	74195	74534	1.96	0.00E+00	transcription_start_site	+	74691	74691	-326	15868	AN3469
944	CONTIG58	74195	74534	1.96	0.00E+00	transcription_start_site	+	74915	74915	-550	15869	AN3469
944	CONTIG58	74195	74534	1.96	0.00E+00	transcription_start_site	+	75039	75039	-674	15870	AN3469
944	CONTIG58	74195	74534	1.96	0.00E+00	transcription_start_site	+	75173	75173	-808	15871	AN3469
3024	CONTIG59	13054	13418	0.87	1.93E-01	transcription_start_site	-	10492	10492	-2744	15890	AN3473
3024	CONTIG59	13054	13418	0.87	1.93E-01	transcription_start_site	-	12927	12927	-309	15891	AN3474
3024	CONTIG59	13054	13418	0.87	1.93E-01	transcription_start_site	+	17946	17946	-4710	15895	AN3476
2974	CONTIG59	29029	29305	0.94	1.44E-01	transcription_start_site	-	29036	29036	-131	15909	AN3480
2974	CONTIG59	29029	29305	0.94	1.44E-01	transcription_start_site	-	28890	28890	-277	15910	AN3480
2974	CONTIG59	29029	29305	0.94	1.44E-01	transcription_start_site	-	28540	28540	-627	15911	AN3480
2974	CONTIG59	29029	29305	0.94	1.44E-01	transcription_start_site	+	31096	31096	-1929	15912	AN3481
2974	CONTIG59	29029	29305	0.94	1.44E-01	transcription_start_site	+	31312	31312	-2145	15913	AN3481
2878	CONTIG59	34291	34640	1.01	1.08E-01	transcription_start_site	-	33365	33365	-1100	15914	AN3482
2878	CONTIG59	34291	34640	1.01	1.08E-01	transcription_start_site	-	33301	33301	-1164	15915	AN3482
2878	CONTIG59	34291	34640	1.01	1.08E-01	transcription_start_site	-	32393	32393	-2072	15916	AN3482
1033	CONTIG59	60767	61111	1.88	1.22E-03	transcription_start_site	+	60945	60945	-6	15933	AN3489
1033	CONTIG59	60767	61111	1.88	1.22E-03	transcription_start_site	+	61201	61201	-262	15934	AN3489
1033	CONTIG59	60767	61111	1.88	1.22E-03	transcription_start_site	+	61574	61574	-635	15935	AN3489
1033	CONTIG59	60767	61111	1.88	1.22E-03	transcription_start_site	+	63320	63320	-2381	15936	AN3489
1033	CONTIG59	60767	61111	1.88	1.22E-03	transcription_start_site	+	64095	64095	-3156	15937	AN3489
3005	CONTIG59	62702	63881	0.91	1.50E-01	transcription_start_site	+	63320	63320	-28	15936	AN3489
3005	CONTIG59	62702	63881	0.91	1.50E-01	transcription_start_site	+	64095	64095	-803	15937	AN3489
2431	CONTIG59	69307	70419	1.22	4.36E-02	transcription_start_site	-	66389	66389	-3474	15938	AN3490
2431	CONTIG59	69307	70419	1.22	4.36E-02	transcription_start_site	-	66224	66224	-3639	15939	AN3490
2431	CONTIG59	69307	70419	1.22	4.36E-02	transcription_start_site	-	66052	66052	-3811	15940	AN3490
2431	CONTIG59	69307	70419	1.22	4.36E-02	transcription_start_site	-	65639	65639	-4224	15941	AN3490
2431	CONTIG59	69307	70419	1.22	4.36E-02	transcription_start_site	-	65157	65157	-4706	15942	AN3490
2431	CONTIG59	69307	70419	1.22	4.36E-02	transcription_start_site	-	64410	64410	-5453	15943	AN3490
3005	CONTIG59	62702	63881	0.91	1.50E-01	transcription_start_site	-	64231	64231	939	15944	AN3490
3005	CONTIG59	62702	63881	0.91	1.50E-01	transcription_start_site	-	64410	64410	1118	15943	AN3490
3005	CONTIG59	62702	63881	0.91	1.50E-01	transcription_start_site	+	67029	67029	-3737	15945	AN3491
3005	CONTIG59	62702	63881	0.91	1.50E-01	transcription_start_site	+	67290	67290	-3998	15946	AN3491
3005	CONTIG59	62702	63881	0.91	1.50E-01	transcription_start_site	+	67537	67537	-4245	15947	AN3491
3005	CONTIG59	62702	63881	0.91	1.50E-01	transcription_start_site	+	68163	68163	-4871	15948	AN3491
2431	CONTIG59	69307	70419	1.22	4.36E-02	transcription_start_site	+	69198	69198	665	15949	AN3492
2431	CONTIG59	69307	70419	1.22	4.36E-02	transcription_start_site	+	70649	70649	-786	15950	AN3493
2431	CONTIG59	69307	70419	1.22	4.36E-02	transcription_start_site	+	70736	70736	-873	15951	AN3493
2431	CONTIG59	69307	70419	1.22	4.36E-02	transcription_start_site	+	72664	72664	-2801	15952	AN3494
2553	CONTIG59	93171	93520	1.18	5.22E-02	transcription_start_site	-	88430	88430	-4915	15957	AN3497
2879	CONTIG59	91221	91555	1.01	1.08E-01	transcription_start_site	-	88430	88430	-2958	15957	AN3497
2879	CONTIG59	91221	91555	1.01	1.08E-01	transcription_start_site	-	87770	87770	-3618	15958	AN3497
2553	CONTIG59	93171	93520	1.18	5.22E-02	transcription_start_site	-	93116	93116	-229	15959	AN3498
2553	CONTIG59	93171	93520	1.18	5.22E-02	transcription_start_site	-	92664	92664	-681	15960	AN3498
2553	CONTIG59	93171	93520	1.18	5.22E-02	transcription_start_site	-	92516	92516	-829	15961	AN3498
2553	CONTIG59	93171	93520	1.18	5.22E-02	transcription_start_site	-	92059	92059	-1286	15962	AN3498
2553	CONTIG59	93171	93520	1.18	5.22E-02	transcription_start_site	-	91820	91820	-1525	15963	AN3498
2553	CONTIG59	93171	93520	1.18	5.22E-02	transcription_start_site	-	91025	91025	-2320	15964	AN3498
2553	CONTIG59	93171	93520	1.18	5.22E-02	transcription_start_site	-	90445	90445	-2900	15965	AN3498
2553	CONTIG59	93171	93520	1.18	5.22E-02	transcription_start_site	-	90204	90204	-3141	15966	AN3498
2553	CONTIG59	93171	93520	1.18	5.22E-02	transcription_start_site	-	90156	90156	-3189	15967	AN3498
2879	CONTIG59	91221	91555	1.01	1.08E-01	transcription_start_site	-	91025	91025	-363	15964	AN3498
2879	CONTIG59	91221	91555	1.01	1.08E-01	transcription_start_site	-	91820	91820	432	15963	AN3498
2879	CONTIG59	91221	91555	1.01	1.08E-01	transcription_start_site	-	92059	92059	671	15962	AN3498
2879	CONTIG59	91221	91555	1.01	1.08E-01	transcription_start_site	-	90445	90445	-943	15965	AN3498
2879	CONTIG59	91221	91555	1.01	1.08E-01	transcription_start_site	-	92516	92516	1128	15961	AN3498
2879	CONTIG59	91221	91555	1.01	1.08E-01	transcription_start_site	-	90204	90204	-1184	15966	AN3498

2879	CONTIG59	91221	91555	1.01	1.08E-01	transcription_start_site	-	90156	90156	-1232	15967	AN3498
3007	CONTIG59	94366	94640	0.91	1.66E-01	transcription_start_site	-	93116	93116	-1387	15959	AN3498
3007	CONTIG59	94366	94640	0.91	1.66E-01	transcription_start_site	-	92664	92664	-1839	15960	AN3498
3007	CONTIG59	94366	94640	0.91	1.66E-01	transcription_start_site	-	92516	92516	-1987	15961	AN3498
3007	CONTIG59	94366	94640	0.91	1.66E-01	transcription_start_site	-	92059	92059	-2444	15962	AN3498
3007	CONTIG59	94366	94640	0.91	1.66E-01	transcription_start_site	-	91820	91820	-2683	15963	AN3498
3007	CONTIG59	94366	94640	0.91	1.66E-01	transcription_start_site	-	91025	91025	-3478	15964	AN3498
3007	CONTIG59	94366	94640	0.91	1.66E-01	transcription_start_site	-	90445	90445	-4058	15965	AN3498
3007	CONTIG59	94366	94640	0.91	1.66E-01	transcription_start_site	-	90204	90204	-4299	15966	AN3498
3007	CONTIG59	94366	94640	0.91	1.66E-01	transcription_start_site	-	90156	90156	-4347	15967	AN3498
2553	CONTIG59	93171	93520	1.18	5.22E-02	transcription_start_site	+	93548	93548	-202	15968	AN3499
2553	CONTIG59	93171	93520	1.18	5.22E-02	transcription_start_site	+	93671	93671	-325	15969	AN3499
2553	CONTIG59	93171	93520	1.18	5.22E-02	transcription_start_site	+	93796	93796	-450	15970	AN3499
2879	CONTIG59	91221	91555	1.01	1.08E-01	transcription_start_site	+	93548	93548	-2160	15968	AN3499
2879	CONTIG59	91221	91555	1.01	1.08E-01	transcription_start_site	+	93671	93671	-2283	15969	AN3499
2879	CONTIG59	91221	91555	1.01	1.08E-01	transcription_start_site	+	93796	93796	-2408	15970	AN3499
3007	CONTIG59	94366	94640	0.91	1.66E-01	transcription_start_site	+	93796	93796	707	15970	AN3499
3007	CONTIG59	94366	94640	0.91	1.66E-01	transcription_start_site	+	93671	93671	832	15969	AN3499
3007	CONTIG59	94366	94640	0.91	1.66E-01	transcription_start_site	+	93548	93548	955	15968	AN3499
2553	CONTIG59	93171	93520	1.18	5.22E-02	transcription_start_site	+	95438	95438	-2092	15971	AN3500
2553	CONTIG59	93171	93520	1.18	5.22E-02	transcription_start_site	+	95999	95999	-2653	15972	AN3500
2553	CONTIG59	93171	93520	1.18	5.22E-02	transcription_start_site	+	96750	96750	-3404	15973	AN3500
2553	CONTIG59	93171	93520	1.18	5.22E-02	transcription_start_site	+	97067	97067	-3721	15974	AN3500
2553	CONTIG59	93171	93520	1.18	5.22E-02	transcription_start_site	+	97414	97414	-4068	15975	AN3500
2879	CONTIG59	91221	91555	1.01	1.08E-01	transcription_start_site	+	95438	95438	-4050	15971	AN3500
2879	CONTIG59	91221	91555	1.01	1.08E-01	transcription_start_site	+	95999	95999	-4611	15972	AN3500
3007	CONTIG59	94366	94640	0.91	1.66E-01	transcription_start_site	+	95438	95438	-935	15971	AN3500
3007	CONTIG59	94366	94640	0.91	1.66E-01	transcription_start_site	+	95999	95999	-1496	15972	AN3500
3007	CONTIG59	94366	94640	0.91	1.66E-01	transcription_start_site	+	96750	96750	-2247	15973	AN3500
3007	CONTIG59	94366	94640	0.91	1.66E-01	transcription_start_site	+	97067	97067	-2564	15974	AN3500
3007	CONTIG59	94366	94640	0.91	1.66E-01	transcription_start_site	+	97414	97414	-2911	15975	AN3500
2553	CONTIG59	93171	93520	1.18	5.22E-02	transcription_start_site	+	97975	97975	-4629	15976	AN3501
2553	CONTIG59	93171	93520	1.18	5.22E-02	transcription_start_site	+	98335	98335	-4989	15977	AN3501
3007	CONTIG59	94366	94640	0.91	1.66E-01	transcription_start_site	+	97975	97975	-3472	15976	AN3501
3007	CONTIG59	94366	94640	0.91	1.66E-01	transcription_start_site	+	98335	98335	-3832	15977	AN3501
3007	CONTIG59	94366	94640	0.91	1.66E-01	transcription_start_site	+	98970	98970	-4467	15978	AN3501
2347	CONTIG59	109071	109415	1.25	3.70E-02	transcription_start_site	-	105713	105713	-3530	15984	AN3502
2347	CONTIG59	109071	109415	1.25	3.70E-02	transcription_start_site	-	105517	105517	-3726	15985	AN3502
2347	CONTIG59	109071	109415	1.25	3.70E-02	transcription_start_site	-	105357	105357	-3886	15986	AN3502
2347	CONTIG59	109071	109415	1.25	3.70E-02	transcription_start_site	-	104927	104927	-4316	15987	AN3502
2347	CONTIG59	109071	109415	1.25	3.70E-02	transcription_start_site	-	104865	104865	-4378	15988	AN3502
2347	CONTIG59	109071	109415	1.25	3.70E-02	transcription_start_site	-	104708	104708	-4535	15989	AN3502
2347	CONTIG59	109071	109415	1.25	3.70E-02	transcription_start_site	-	104333	104333	-4910	15990	AN3502
2347	CONTIG59	109071	109415	1.25	3.70E-02	transcription_start_site	-	104164	104164	-5079	15991	AN3502
2347	CONTIG59	109071	109415	1.25	3.70E-02	transcription_start_site	-	104078	104078	-5165	15992	AN3502
2347	CONTIG59	109071	109415	1.25	3.70E-02	transcription_start_site	-	108184	108184	-1059	15994	AN3503
2347	CONTIG59	109071	109415	1.25	3.70E-02	transcription_start_site	-	107588	107588	-1655	15995	AN3503
2347	CONTIG59	109071	109415	1.25	3.70E-02	transcription_start_site	-	107431	107431	-1812	15996	AN3503
2347	CONTIG59	109071	109415	1.25	3.70E-02	transcription_start_site	-	107261	107261	-1982	15997	AN3503
2347	CONTIG59	109071	109415	1.25	3.70E-02	transcription_start_site	-	107022	107022	-2221	15998	AN3503
2347	CONTIG59	109071	109415	1.25	3.70E-02	transcription_start_site	-	106708	106708	-2535	15999	AN3503
2347	CONTIG59	109071	109415	1.25	3.70E-02	transcription_start_site	-	106645	106645	-2598	16000	AN3503
2347	CONTIG59	109071	109415	1.25	3.70E-02	transcription_start_site	-	106473	106473	-2770	16001	AN3503
2347	CONTIG59	109071	109415	1.25	3.70E-02	transcription_start_site	-	106360	106360	-2883	16002	AN3503
2347	CONTIG59	109071	109415	1.25	3.70E-02	transcription_start_site	-	106272	106272	-2971	16003	AN3503
2347	CONTIG59	109071	109415	1.25	3.70E-02	transcription_start_site	+	108970	108970	273	16004	AN3504
2347	CONTIG59	109071	109415	1.25	3.70E-02	transcription_start_site	+	110563	110563	-1320	16005	AN3504

2880	CONTIG59	116330	116619	1.01	1.08E-01	transcription_start_site	-	113118	113118	-3356	16006 AN3505
2880	CONTIG59	116330	116619	1.01	1.08E-01	transcription_start_site	-	112917	112917	-3557	16007 AN3505
2880	CONTIG59	116330	116619	1.01	1.08E-01	transcription_start_site	-	112036	112036	-4438	16008 AN3505
2880	CONTIG59	116330	116619	1.01	1.08E-01	transcription_start_site	+	116421	116421	53	16010 AN3506
2880	CONTIG59	116330	116619	1.01	1.08E-01	transcription_start_site	+	116305	116305	169	16009 AN3506
2880	CONTIG59	116330	116619	1.01	1.08E-01	transcription_start_site	+	118053	118053	-1578	16011 AN3507
2880	CONTIG59	116330	116619	1.01	1.08E-01	transcription_start_site	+	118274	118274	-1799	16012 AN3507
2880	CONTIG59	116330	116619	1.01	1.08E-01	transcription_start_site	+	118503	118503	-2028	16013 AN3507
2880	CONTIG59	116330	116619	1.01	1.08E-01	transcription_start_site	+	120356	120356	-3881	16017 AN3509
2880	CONTIG59	116330	116619	1.01	1.08E-01	transcription_start_site	+	120835	120835	-4360	16018 AN3509
2128	CONTIG59	127295	127714	1.32	2.62E-02	transcription_start_site	-	123478	123478	-4026	16019 AN3510
2128	CONTIG59	127295	127714	1.32	2.62E-02	transcription_start_site	-	123270	123270	-4234	16020 AN3510
2946	CONTIG59	128345	128829	0.97	1.07E-01	transcription_start_site	-	123478	123478	-5109	16019 AN3510
996	CONTIG59	130970	131229	1.91	1.01E-03	transcription_start_site	+	131150	131150	-50	16024 AN3512
996	CONTIG59	130970	131229	1.91	1.01E-03	transcription_start_site	+	131032	131032	67	16023 AN3512
1611	CONTIG59	129530	130344	1.53	1.17E-03	transcription_start_site	+	131032	131032	-1095	16023 AN3512
1611	CONTIG59	129530	130344	1.53	1.17E-03	transcription_start_site	+	131150	131150	-1213	16024 AN3512
2128	CONTIG59	127295	127714	1.32	2.62E-02	transcription_start_site	+	131032	131032	-3527	16023 AN3512
2128	CONTIG59	127295	127714	1.32	2.62E-02	transcription_start_site	+	131150	131150	-3645	16024 AN3512
2881	CONTIG59	131485	132069	1.01	1.08E-01	transcription_start_site	+	131150	131150	627	16024 AN3512
2881	CONTIG59	131485	132069	1.01	1.08E-01	transcription_start_site	+	131032	131032	745	16023 AN3512
2946	CONTIG59	128345	128829	0.97	1.07E-01	transcription_start_site	+	131032	131032	-2445	16023 AN3512
2946	CONTIG59	128345	128829	0.97	1.07E-01	transcription_start_site	+	131150	131150	-2563	16024 AN3512
1477	CONTIG59	141757	142266	1.6	6.61E-03	transcription_start_site	-	137040	137040	-4971	16026 AN3514
1477	CONTIG59	141757	142266	1.6	6.61E-03	transcription_start_site	-	142326	142326	314	16029 AN3515
1513	CONTIG59	150232	150523	1.57	7.85E-03	transcription_start_site	+	151148	151148	-770	16030 AN3517
2554	CONTIG59	153317	153581	1.18	5.22E-02	transcription_start_site	-	153766	153766	317	16031 AN3518
2554	CONTIG59	153317	153581	1.18	5.22E-02	transcription_start_site	-	152735	152735	-714	16032 AN3518
1513	CONTIG59	150232	150523	1.57	7.85E-03	transcription_start_site	+	154219	154219	-3841	16033 AN3519
1513	CONTIG59	150232	150523	1.57	7.85E-03	transcription_start_site	+	154344	154344	-3966	16034 AN3519
1513	CONTIG59	150232	150523	1.57	7.85E-03	transcription_start_site	+	154924	154924	-4546	16035 AN3519
1513	CONTIG59	150232	150523	1.57	7.85E-03	transcription_start_site	+	155182	155182	-4804	16036 AN3519
1513	CONTIG59	150232	150523	1.57	7.85E-03	transcription_start_site	+	155459	155459	-5081	16037 AN3519
2554	CONTIG59	153317	153581	1.18	5.22E-02	transcription_start_site	+	154219	154219	-770	16033 AN3519
2554	CONTIG59	153317	153581	1.18	5.22E-02	transcription_start_site	+	154344	154344	-895	16034 AN3519
2554	CONTIG59	153317	153581	1.18	5.22E-02	transcription_start_site	+	154924	154924	-1475	16035 AN3519
2554	CONTIG59	153317	153581	1.18	5.22E-02	transcription_start_site	+	155182	155182	-1733	16036 AN3519
2554	CONTIG59	153317	153581	1.18	5.22E-02	transcription_start_site	+	155459	155459	-2010	16037 AN3519
2554	CONTIG59	153317	153581	1.18	5.22E-02	transcription_start_site	+	155608	155608	-2159	16038 AN3519
2554	CONTIG59	153317	153581	1.18	5.22E-02	transcription_start_site	+	156003	156003	-2554	16039 AN3519
2772	CONTIG59	170478	170829	1.08	8.01E-02	transcription_start_site	-	169369	169369	-1284	16053 AN3524
2772	CONTIG59	170478	170829	1.08	8.01E-02	transcription_start_site	-	169186	169186	-1467	16054 AN3524
2772	CONTIG59	170478	170829	1.08	8.01E-02	transcription_start_site	-	169000	169000	-1653	16055 AN3524
859	CONTIG60	11926	12225	2.04	0.00E+00	transcription_start_site	-	11641	11641	-434	16069 AN3529
859	CONTIG60	11926	12225	2.04	0.00E+00	transcription_start_site	-	11270	11270	-805	16070 AN3529
859	CONTIG60	11926	12225	2.04	0.00E+00	transcription_start_site	+	13401	13401	-1325	16071 AN3530
859	CONTIG60	11926	12225	2.04	0.00E+00	transcription_start_site	+	13656	13656	-1580	16072 AN3530
859	CONTIG60	11926	12225	2.04	0.00E+00	transcription_start_site	+	13977	13977	-1901	16073 AN3530
859	CONTIG60	11926	12225	2.04	0.00E+00	transcription_start_site	+	14293	14293	-2217	16074 AN3530
859	CONTIG60	11926	12225	2.04	0.00E+00	transcription_start_site	+	14473	14473	-2397	16075 AN3530
859	CONTIG60	11926	12225	2.04	0.00E+00	transcription_start_site	+	15993	15993	-3917	16076 AN3531
3042	CONTIG60	40743	41087	0.78	1.44E-01	transcription_start_site	-	37757	37757	-3158	16088 AN3536
3042	CONTIG60	40743	41087	0.78	1.44E-01	transcription_start_site	-	37628	37628	-3287	16089 AN3536
3042	CONTIG60	40743	41087	0.78	1.44E-01	transcription_start_site	-	37380	37380	-3535	16090 AN3536
3042	CONTIG60	40743	41087	0.78	1.44E-01	transcription_start_site	-	36844	36844	-4071	16091 AN3536
3042	CONTIG60	40743	41087	0.78	1.44E-01	transcription_start_site	-	36563	36563	-4352	16092 AN3536
3042	CONTIG60	40743	41087	0.78	1.44E-01	transcription_start_site	+	41098	41098	-183	16096 AN3538

2594	CONTIG61	33093	33357	1.17	1.66E-01	transcription_start_site	-	28349	28349	-4876	16131	AN3549
2594	CONTIG61	33093	33357	1.17	1.66E-01	transcription_start_site	-	28237	28237	-4988	16132	AN3549
1920	CONTIG61	38185	38679	1.4	6.16E-02	transcription_start_site	-	36581	36581	-1851	16149	AN3552
1920	CONTIG61	38185	38679	1.4	6.16E-02	transcription_start_site	-	38836	38836	404	16150	AN3553
1920	CONTIG61	38185	38679	1.4	6.16E-02	transcription_start_site	+	39553	39553	-1121	16151	AN3554
1920	CONTIG61	38185	38679	1.4	6.16E-02	transcription_start_site	+	42257	42257	-3825	16152	AN3554
468	CONTIG61	73355	74154	2.48	1.01E-03	transcription_start_site	-	71987	71987	-1767	16192	AN3564
468	CONTIG61	73355	74154	2.48	1.01E-03	transcription_start_site	-	71884	71884	-1870	16193	AN3564
468	CONTIG61	73355	74154	2.48	1.01E-03	transcription_start_site	-	71495	71495	-2259	16194	AN3564
468	CONTIG61	73355	74154	2.48	1.01E-03	transcription_start_site	-	70896	70896	-2858	16195	AN3564
468	CONTIG61	73355	74154	2.48	1.01E-03	transcription_start_site	+	72892	72892	862	16197	AN3565
468	CONTIG61	73355	74154	2.48	1.01E-03	transcription_start_site	+	73753	73753	1	16198	AN3566
603	CONTIG61	98853	99150	2.3	2.22E-03	transcription_start_site	-	95057	95057	-3944	16219	AN3572
603	CONTIG61	98853	99150	2.3	2.22E-03	transcription_start_site	-	94977	94977	-4024	16220	AN3572
603	CONTIG61	98853	99150	2.3	2.22E-03	transcription_start_site	-	98737	98737	-264	16231	AN3574
603	CONTIG61	98853	99150	2.3	2.22E-03	transcription_start_site	-	99406	99406	404	16230	AN3574
603	CONTIG61	98853	99150	2.3	2.22E-03	transcription_start_site	-	99485	99485	483	16229	AN3574
603	CONTIG61	98853	99150	2.3	2.22E-03	transcription_start_site	-	99772	99772	770	16228	AN3574
603	CONTIG61	98853	99150	2.3	2.22E-03	transcription_start_site	-	100077	100077	1075	16227	AN3574
603	CONTIG61	98853	99150	2.3	2.22E-03	transcription_start_site	+	101626	101626	-2624	16232	AN3575
603	CONTIG61	98853	99150	2.3	2.22E-03	transcription_start_site	+	101779	101779	-2777	16233	AN3575
603	CONTIG61	98853	99150	2.3	2.22E-03	transcription_start_site	+	102179	102179	-3177	16234	AN3575
603	CONTIG61	98853	99150	2.3	2.22E-03	transcription_start_site	+	102968	102968	-3966	16235	AN3575
1507	CONTIG61	118951	119235	1.58	4.36E-02	transcription_start_site	-	118847	118847	-246	16249	AN3581
1507	CONTIG61	118951	119235	1.58	4.36E-02	transcription_start_site	-	118740	118740	-353	16250	AN3581
1273	CONTIG61	135610	135959	1.71	2.62E-02	transcription_start_site	-	131776	131776	-4008	16261	AN3585
1273	CONTIG61	135610	135959	1.71	2.62E-02	transcription_start_site	-	131350	131350	-4434	16262	AN3585
1715	CONTIG61	133360	134079	1.49	3.99E-02	transcription_start_site	-	131776	131776	-1943	16261	AN3585
1715	CONTIG61	133360	134079	1.49	3.99E-02	transcription_start_site	-	131350	131350	-2369	16262	AN3585
1826	CONTIG61	136365	137394	1.44	5.05E-02	transcription_start_site	-	131776	131776	-5103	16261	AN3585
1715	CONTIG61	133360	134079	1.49	3.99E-02	transcription_start_site	+	133134	133134	585	16264	AN3586
1715	CONTIG61	133360	134079	1.49	3.99E-02	transcription_start_site	+	132891	132891	828	16263	AN3586
1273	CONTIG61	135610	135959	1.71	2.62E-02	transcription_start_site	+	137531	137531	-1746	16265	AN3587
1273	CONTIG61	135610	135959	1.71	2.62E-02	transcription_start_site	+	137760	137760	-1975	16266	AN3587
1273	CONTIG61	135610	135959	1.71	2.62E-02	transcription_start_site	+	137967	137967	-2182	16267	AN3587
1273	CONTIG61	135610	135959	1.71	2.62E-02	transcription_start_site	+	138035	138035	-2250	16268	AN3587
1715	CONTIG61	133360	134079	1.49	3.99E-02	transcription_start_site	+	137531	137531	-3811	16265	AN3587
1715	CONTIG61	133360	134079	1.49	3.99E-02	transcription_start_site	+	137760	137760	-4040	16266	AN3587
1715	CONTIG61	133360	134079	1.49	3.99E-02	transcription_start_site	+	137967	137967	-4247	16267	AN3587
1715	CONTIG61	133360	134079	1.49	3.99E-02	transcription_start_site	+	138035	138035	-4315	16268	AN3587
1826	CONTIG61	136365	137394	1.44	5.05E-02	transcription_start_site	+	137531	137531	-651	16265	AN3587
1826	CONTIG61	136365	137394	1.44	5.05E-02	transcription_start_site	+	137760	137760	-880	16266	AN3587
1826	CONTIG61	136365	137394	1.44	5.05E-02	transcription_start_site	+	137967	137967	-1087	16267	AN3587
1826	CONTIG61	136365	137394	1.44	5.05E-02	transcription_start_site	+	138035	138035	-1155	16268	AN3587
1826	CONTIG61	136365	137394	1.44	5.05E-02	transcription_start_site	+	141977	141977	-5097	16269	AN3588
1826	CONTIG61	136365	137394	1.44	5.05E-02	transcription_start_site	+	142372	142372	-5492	16270	AN3588
163	CONTIG61	149335	149679	3.02	0.00E+00	transcription_start_site	-	145513	145513	-3994	16271	AN3589
163	CONTIG61	149335	149679	3.02	0.00E+00	transcription_start_site	-	145421	145421	-4086	16272	AN3589
163	CONTIG61	149335	149679	3.02	0.00E+00	transcription_start_site	-	145311	145311	-4196	16273	AN3589
1720	CONTIG61	144540	144804	1.49	5.88E-02	transcription_start_site	-	145311	145311	639	16273	AN3589
1720	CONTIG61	144540	144804	1.49	5.88E-02	transcription_start_site	-	145421	145421	749	16272	AN3589
1720	CONTIG61	144540	144804	1.49	5.88E-02	transcription_start_site	-	145513	145513	841	16271	AN3589
1720	CONTIG61	144540	144804	1.49	5.88E-02	transcription_start_site	+	146344	146344	-1672	16274	AN3590
1720	CONTIG61	144540	144804	1.49	5.88E-02	transcription_start_site	+	146509	146509	-1837	16275	AN3590
1720	CONTIG61	144540	144804	1.49	5.88E-02	transcription_start_site	+	146619	146619	-1947	16276	AN3590
1720	CONTIG61	144540	144804	1.49	5.88E-02	transcription_start_site	+	147136	147136	-2464	16277	AN3590
163	CONTIG61	149335	149679	3.02	0.00E+00	transcription_start_site	-	149192	149192	-315	16278	AN3591

163	CONTIG61	149335	149679	3.02	0.00E+00	transcription_start_site	-	148587	148587	-920	16279	AN3591
163	CONTIG61	149335	149679	3.02	0.00E+00	transcription_start_site	+	153263	153263	-3756	16285	AN3593
163	CONTIG61	149335	149679	3.02	0.00E+00	transcription_start_site	+	153469	153469	-3962	16286	AN3593
163	CONTIG61	149335	149679	3.02	0.00E+00	transcription_start_site	+	153996	153996	-4489	16287	AN3593
163	CONTIG61	149335	149679	3.02	0.00E+00	transcription_start_site	+	154612	154612	-5105	16288	AN3594
604	CONTIG61	164410	164839	2.3	2.22E-03	transcription_start_site	-	162305	162305	-2319	16297	AN3597
604	CONTIG61	164410	164839	2.3	2.22E-03	transcription_start_site	-	161268	161268	-3356	16298	AN3597
604	CONTIG61	164410	164839	2.3	2.22E-03	transcription_start_site	-	160508	160508	-4116	16299	AN3597
604	CONTIG61	164410	164839	2.3	2.22E-03	transcription_start_site	-	165596	165596	971	16303	AN3598
604	CONTIG61	164410	164839	2.3	2.22E-03	transcription_start_site	-	165688	165688	1063	16302	AN3598
604	CONTIG61	164410	164839	2.3	2.22E-03	transcription_start_site	-	165828	165828	1203	16301	AN3598
1195	CONTIG61	170410	171212	1.76	1.05E-02	transcription_start_site	-	166131	166131	-4680	16300	AN3598
1195	CONTIG61	170410	171212	1.76	1.05E-02	transcription_start_site	-	165828	165828	-4983	16301	AN3598
1195	CONTIG61	170410	171212	1.76	1.05E-02	transcription_start_site	-	165688	165688	-5123	16302	AN3598
1195	CONTIG61	170410	171212	1.76	1.05E-02	transcription_start_site	-	165596	165596	-5215	16303	AN3598
1721	CONTIG61	169735	170019	1.49	5.88E-02	transcription_start_site	-	166131	166131	-3746	16300	AN3598
1721	CONTIG61	169735	170019	1.49	5.88E-02	transcription_start_site	-	165828	165828	-4049	16301	AN3598
1721	CONTIG61	169735	170019	1.49	5.88E-02	transcription_start_site	-	165688	165688	-4189	16302	AN3598
1721	CONTIG61	169735	170019	1.49	5.88E-02	transcription_start_site	-	165596	165596	-4281	16303	AN3598
114	CONTIG61	171243	171593	3.16	0.00E+00	transcription_start_site	-	167717	167717	-3701	16304	AN3599
114	CONTIG61	171243	171593	3.16	0.00E+00	transcription_start_site	-	167273	167273	-4145	16305	AN3599
114	CONTIG61	171243	171593	3.16	0.00E+00	transcription_start_site	-	167156	167156	-4262	16306	AN3599
114	CONTIG61	171243	171593	3.16	0.00E+00	transcription_start_site	-	166919	166919	-4499	16307	AN3599
1195	CONTIG61	170410	171212	1.76	1.05E-02	transcription_start_site	-	167717	167717	-3094	16304	AN3599
1195	CONTIG61	170410	171212	1.76	1.05E-02	transcription_start_site	-	167273	167273	-3538	16305	AN3599
1195	CONTIG61	170410	171212	1.76	1.05E-02	transcription_start_site	-	167156	167156	-3655	16306	AN3599
1195	CONTIG61	170410	171212	1.76	1.05E-02	transcription_start_site	-	166919	166919	-3892	16307	AN3599
1721	CONTIG61	169735	170019	1.49	5.88E-02	transcription_start_site	-	167717	167717	-2160	16304	AN3599
1721	CONTIG61	169735	170019	1.49	5.88E-02	transcription_start_site	-	167273	167273	-2604	16305	AN3599
1721	CONTIG61	169735	170019	1.49	5.88E-02	transcription_start_site	-	167156	167156	-2721	16306	AN3599
1721	CONTIG61	169735	170019	1.49	5.88E-02	transcription_start_site	-	166919	166919	-2958	16307	AN3599
114	CONTIG61	171243	171593	3.16	0.00E+00	transcription_start_site	-	170064	170064	-1354	16308	AN3600
1023	CONTIG61	174688	175252	1.89	1.30E-02	transcription_start_site	-	170064	170064	-4906	16308	AN3600
1195	CONTIG61	170410	171212	1.76	1.05E-02	transcription_start_site	-	170064	170064	-747	16308	AN3600
1721	CONTIG61	169735	170019	1.49	5.88E-02	transcription_start_site	-	170064	170064	187	16308	AN3600
1023	CONTIG61	174688	175252	1.89	1.30E-02	transcription_start_site	-	173220	173220	-1750	16309	AN3601
1023	CONTIG61	174688	175252	1.89	1.30E-02	transcription_start_site	-	172946	172946	-2024	16310	AN3601
114	CONTIG61	171243	171593	3.16	0.00E+00	transcription_start_site	+	175146	175146	-3728	16311	AN3602
114	CONTIG61	171243	171593	3.16	0.00E+00	transcription_start_site	+	176054	176054	-4636	16312	AN3602
1023	CONTIG61	174688	175252	1.89	1.30E-02	transcription_start_site	+	175146	175146	-176	16311	AN3602
1023	CONTIG61	174688	175252	1.89	1.30E-02	transcription_start_site	+	176054	176054	-1084	16312	AN3602
1195	CONTIG61	170410	171212	1.76	1.05E-02	transcription_start_site	+	175146	175146	-4335	16311	AN3602
1195	CONTIG61	170410	171212	1.76	1.05E-02	transcription_start_site	+	176054	176054	-5243	16312	AN3602
1508	CONTIG61	189154	189445	1.58	4.36E-02	transcription_start_site	-	185071	185071	-4228	16315	AN3605
1508	CONTIG61	189154	189445	1.58	4.36E-02	transcription_start_site	-	184821	184821	-4478	16316	AN3605
1508	CONTIG61	189154	189445	1.58	4.36E-02	transcription_start_site	-	184375	184375	-4924	16317	AN3605
1508	CONTIG61	189154	189445	1.58	4.36E-02	transcription_start_site	-	189220	189220	-79	16322	AN3607
1508	CONTIG61	189154	189445	1.58	4.36E-02	transcription_start_site	-	187902	187902	-1397	16323	AN3607
2167	CONTIG61	252459	252878	1.31	1.08E-01	transcription_start_site	-	249995	249995	-2673	16356	AN3620
2167	CONTIG61	252459	252878	1.31	1.08E-01	transcription_start_site	+	251607	251607	1061	16361	AN3621
2167	CONTIG61	252459	252878	1.31	1.08E-01	transcription_start_site	+	253369	253369	-700	16362	AN3622
2167	CONTIG61	252459	252878	1.31	1.08E-01	transcription_start_site	+	253598	253598	-929	16363	AN3622
2167	CONTIG61	252459	252878	1.31	1.08E-01	transcription_start_site	+	254371	254371	-1702	16364	AN3622
2167	CONTIG61	252459	252878	1.31	1.08E-01	transcription_start_site	+	257428	257428	-4759	16366	AN3624
1269	CONTIG61	264468	264802	1.71	1.44E-02	transcription_start_site	-	262925	262925	-1710	16367	AN3625
1269	CONTIG61	264468	264802	1.71	1.44E-02	transcription_start_site	-	262231	262231	-2404	16368	AN3625
1269	CONTIG61	264468	264802	1.71	1.44E-02	transcription_start_site	-	261973	261973	-2662	16369	AN3625

1269	CONTIG61	264468	264802	1.71	1.44E-02	transcription_start_site	+	264300	264300	335	16372 AN3626
1269	CONTIG61	264468	264802	1.71	1.44E-02	transcription_start_site	+	264160	264160	475	16371 AN3626
1269	CONTIG61	264468	264802	1.71	1.44E-02	transcription_start_site	+	265351	265351	-716	16373 AN3626
1269	CONTIG61	264468	264802	1.71	1.44E-02	transcription_start_site	+	263643	263643	992	16370 AN3626
630	CONTIG61	269118	269767	2.26	0.00E+00	transcription_start_site	-	268847	268847	-595	16374 AN3627
630	CONTIG61	269118	269767	2.26	0.00E+00	transcription_start_site	-	268672	268672	-770	16375 AN3627
630	CONTIG61	269118	269767	2.26	0.00E+00	transcription_start_site	-	268294	268294	-1148	16376 AN3627
630	CONTIG61	269118	269767	2.26	0.00E+00	transcription_start_site	-	268047	268047	-1395	16377 AN3627
630	CONTIG61	269118	269767	2.26	0.00E+00	transcription_start_site	-	267725	267725	-1717	16378 AN3627
630	CONTIG61	269118	269767	2.26	0.00E+00	transcription_start_site	-	267535	267535	-1907	16379 AN3627
630	CONTIG61	269118	269767	2.26	0.00E+00	transcription_start_site	-	267348	267348	-2094	16380 AN3627
630	CONTIG61	269118	269767	2.26	0.00E+00	transcription_start_site	-	270516	270516	1073	16383 AN3628
1426	CONTIG61	279758	280027	1.62	3.70E-02	transcription_start_site	-	279875	279875	-17	16389 AN3631
1426	CONTIG61	279758	280027	1.62	3.70E-02	transcription_start_site	-	280614	280614	721	16388 AN3631
1073	CONTIG61	291833	292267	1.85	1.57E-02	transcription_start_site	-	288737	288737	-3313	16395 AN3634
1073	CONTIG61	291833	292267	1.85	1.57E-02	transcription_start_site	-	288353	288353	-3697	16396 AN3634
1073	CONTIG61	291833	292267	1.85	1.57E-02	transcription_start_site	-	291888	291888	-162	16398 AN3636
1073	CONTIG61	291833	292267	1.85	1.57E-02	transcription_start_site	+	292440	292440	-390	16399 AN3637
1073	CONTIG61	291833	292267	1.85	1.57E-02	transcription_start_site	+	292616	292616	-566	16400 AN3637
1073	CONTIG61	291833	292267	1.85	1.57E-02	transcription_start_site	+	292774	292774	-724	16401 AN3637
1073	CONTIG61	291833	292267	1.85	1.57E-02	transcription_start_site	+	294209	294209	-2159	16402 AN3637
1722	CONTIG61	300021	300730	1.49	5.88E-02	transcription_start_site	-	295628	295628	-4747	16403 AN3638
1722	CONTIG61	300021	300730	1.49	5.88E-02	transcription_start_site	-	295341	295341	-5034	16404 AN3638
1073	CONTIG61	291833	292267	1.85	1.57E-02	transcription_start_site	+	296255	296255	-4205	16405 AN3639
1073	CONTIG61	291833	292267	1.85	1.57E-02	transcription_start_site	+	296527	296527	-4477	16406 AN3639
1073	CONTIG61	291833	292267	1.85	1.57E-02	transcription_start_site	+	296684	296684	-4634	16407 AN3639
1073	CONTIG61	291833	292267	1.85	1.57E-02	transcription_start_site	+	296812	296812	-4762	16408 AN3639
1722	CONTIG61	300021	300730	1.49	5.88E-02	transcription_start_site	-	300911	300911	535	16409 AN3640
2168	CONTIG61	302867	303141	1.31	1.08E-01	transcription_start_site	-	300911	300911	-2093	16409 AN3640
2691	CONTIG61	303457	304336	1.13	1.93E-01	transcription_start_site	-	300911	300911	-2985	16409 AN3640
1723	CONTIG61	306827	307191	1.49	5.88E-02	transcription_start_site	-	303271	303271	-3738	16410 AN3641
1723	CONTIG61	306827	307191	1.49	5.88E-02	transcription_start_site	-	302943	302943	-4066	16411 AN3641
2168	CONTIG61	302867	303141	1.31	1.08E-01	transcription_start_site	-	302943	302943	-61	16411 AN3641
2168	CONTIG61	302867	303141	1.31	1.08E-01	transcription_start_site	-	303271	303271	267	16410 AN3641
2691	CONTIG61	303457	304336	1.13	1.93E-01	transcription_start_site	-	303271	303271	-625	16410 AN3641
2691	CONTIG61	303457	304336	1.13	1.93E-01	transcription_start_site	-	302943	302943	-953	16411 AN3641
1722	CONTIG61	300021	300730	1.49	5.88E-02	transcription_start_site	+	305580	305580	-5204	16412 AN3642
1723	CONTIG61	306827	307191	1.49	5.88E-02	transcription_start_site	+	307095	307095	-86	16416 AN3642
1723	CONTIG61	306827	307191	1.49	5.88E-02	transcription_start_site	+	306569	306569	440	16415 AN3642
1723	CONTIG61	306827	307191	1.49	5.88E-02	transcription_start_site	+	306427	306427	582	16414 AN3642
1723	CONTIG61	306827	307191	1.49	5.88E-02	transcription_start_site	+	305899	305899	1110	16413 AN3642
2168	CONTIG61	302867	303141	1.31	1.08E-01	transcription_start_site	+	305580	305580	-2576	16412 AN3642
2168	CONTIG61	302867	303141	1.31	1.08E-01	transcription_start_site	+	305899	305899	-2895	16413 AN3642
2168	CONTIG61	302867	303141	1.31	1.08E-01	transcription_start_site	+	306427	306427	-3423	16414 AN3642
2168	CONTIG61	302867	303141	1.31	1.08E-01	transcription_start_site	+	306569	306569	-3565	16415 AN3642
2168	CONTIG61	302867	303141	1.31	1.08E-01	transcription_start_site	+	307095	307095	-4091	16416 AN3642
2691	CONTIG61	303457	304336	1.13	1.93E-01	transcription_start_site	+	305580	305580	-1683	16412 AN3642
2691	CONTIG61	303457	304336	1.13	1.93E-01	transcription_start_site	+	305899	305899	-2002	16413 AN3642
2691	CONTIG61	303457	304336	1.13	1.93E-01	transcription_start_site	+	306427	306427	-2530	16414 AN3642
2691	CONTIG61	303457	304336	1.13	1.93E-01	transcription_start_site	+	306569	306569	-2672	16415 AN3642
2691	CONTIG61	303457	304336	1.13	1.93E-01	transcription_start_site	+	307095	307095	-3198	16416 AN3642
2692	CONTIG61	333242	333581	1.13	1.93E-01	transcription_start_site	-	333668	333668	256	16446 AN3651
2692	CONTIG61	333242	333581	1.13	1.93E-01	transcription_start_site	-	334036	334036	624	16445 AN3651
2692	CONTIG61	333242	333581	1.13	1.93E-01	transcription_start_site	-	334229	334229	817	16444 AN3651
2692	CONTIG61	333242	333581	1.13	1.93E-01	transcription_start_site	+	334587	334587	-1175	16447 AN3652
2692	CONTIG61	333242	333581	1.13	1.93E-01	transcription_start_site	+	335056	335056	-1644	16448 AN3652
2692	CONTIG61	333242	333581	1.13	1.93E-01	transcription_start_site	+	335170	335170	-1758	16449 AN3652

2692	CONTIG61	333242	333581	1.13	1.93E-01	transcription_start_site	+	335324	335324	-1912	16450	AN3652
2692	CONTIG61	333242	333581	1.13	1.93E-01	transcription_start_site	+	335515	335515	-2103	16451	AN3652
2692	CONTIG61	333242	333581	1.13	1.93E-01	transcription_start_site	+	335927	335927	-2515	16452	AN3652
1427	CONTIG61	346503	346867	1.62	3.70E-02	transcription_start_site	-	341507	341507	-5178	16456	AN3654
1427	CONTIG61	346503	346867	1.62	3.70E-02	transcription_start_site	-	343942	343942	-2743	16459	AN3656
1427	CONTIG61	346503	346867	1.62	3.70E-02	transcription_start_site	-	343862	343862	-2823	16460	AN3656
1427	CONTIG61	346503	346867	1.62	3.70E-02	transcription_start_site	-	343709	343709	-2976	16461	AN3656
1427	CONTIG61	346503	346867	1.62	3.70E-02	transcription_start_site	-	343372	343372	-3313	16462	AN3656
1427	CONTIG61	346503	346867	1.62	3.70E-02	transcription_start_site	-	343226	343226	-3459	16463	AN3656
1427	CONTIG61	346503	346867	1.62	3.70E-02	transcription_start_site	+	347381	347381	-696	16466	AN3657
776	CONTIG61	349508	349852	2.12	5.20E-03	transcription_start_site	-	349121	349121	-559	16467	AN3658
776	CONTIG61	349508	349852	2.12	5.20E-03	transcription_start_site	-	348846	348846	-834	16468	AN3658
776	CONTIG61	349508	349852	2.12	5.20E-03	transcription_start_site	+	350195	350195	-515	16469	AN3659
776	CONTIG61	349508	349852	2.12	5.20E-03	transcription_start_site	+	350427	350427	-747	16470	AN3659
776	CONTIG61	349508	349852	2.12	5.20E-03	transcription_start_site	+	352381	352381	-2701	16471	AN3659
1427	CONTIG61	346503	346867	1.62	3.70E-02	transcription_start_site	+	350195	350195	-3510	16469	AN3659
1427	CONTIG61	346503	346867	1.62	3.70E-02	transcription_start_site	+	350427	350427	-3742	16470	AN3659
1329	CONTIG61	369992	370251	1.67	3.08E-02	transcription_start_site	-	369613	369613	-508	16478	AN3664
1329	CONTIG61	369992	370251	1.67	3.08E-02	transcription_start_site	-	368285	368285	-1836	16479	AN3664
1329	CONTIG61	369992	370251	1.67	3.08E-02	transcription_start_site	-	368150	368150	-1971	16480	AN3664
1830	CONTIG61	374492	374908	1.44	6.84E-02	transcription_start_site	-	369613	369613	-5087	16478	AN3664
1830	CONTIG61	374492	374908	1.44	6.84E-02	transcription_start_site	-	374463	374463	-237	16481	AN3665
1830	CONTIG61	374492	374908	1.44	6.84E-02	transcription_start_site	-	374181	374181	-519	16482	AN3665
1830	CONTIG61	374492	374908	1.44	6.84E-02	transcription_start_site	-	372805	372805	-1895	16483	AN3665
1830	CONTIG61	374492	374908	1.44	6.84E-02	transcription_start_site	-	372557	372557	-2143	16484	AN3665
1830	CONTIG61	374492	374908	1.44	6.84E-02	transcription_start_site	-	372278	372278	-2422	16485	AN3665
1830	CONTIG61	374492	374908	1.44	6.84E-02	transcription_start_site	-	375397	375397	697	16488	AN3666
1918	CONTIG61	380026	381515	1.4	5.52E-02	transcription_start_site	-	376136	376136	-4634	16486	AN3666
1918	CONTIG61	380026	381515	1.4	5.52E-02	transcription_start_site	-	376037	376037	-4733	16487	AN3666
1918	CONTIG61	380026	381515	1.4	5.52E-02	transcription_start_site	-	375397	375397	-5373	16488	AN3666
1918	CONTIG61	380026	381515	1.4	5.52E-02	transcription_start_site	-	378571	378571	-2199	16489	AN3667
1918	CONTIG61	380026	381515	1.4	5.52E-02	transcription_start_site	-	378330	378330	-2440	16490	AN3667
2327	CONTIG61	395720	396073	1.26	1.07E-01	transcription_start_site	-	393335	393335	-2561	16491	AN3668
2327	CONTIG61	395720	396073	1.26	1.07E-01	transcription_start_site	-	393059	393059	-2837	16492	AN3668
2327	CONTIG61	395720	396073	1.26	1.07E-01	transcription_start_site	-	392896	392896	-3000	16493	AN3668
2327	CONTIG61	395720	396073	1.26	1.07E-01	transcription_start_site	-	392623	392623	-3273	16494	AN3668
2327	CONTIG61	395720	396073	1.26	1.07E-01	transcription_start_site	-	391732	391732	-4164	16495	AN3668
2327	CONTIG61	395720	396073	1.26	1.07E-01	transcription_start_site	-	395636	395636	-260	16496	AN3669
2327	CONTIG61	395720	396073	1.26	1.07E-01	transcription_start_site	-	395101	395101	-795	16497	AN3669
2327	CONTIG61	395720	396073	1.26	1.07E-01	transcription_start_site	-	394885	394885	-1011	16498	AN3669
2327	CONTIG61	395720	396073	1.26	1.07E-01	transcription_start_site	-	394461	394461	-1435	16499	AN3669
2327	CONTIG61	395720	396073	1.26	1.07E-01	transcription_start_site	-	396719	396719	822	16501	AN3670
277	CONTIG61	407346	407755	2.8	2.67E-04	transcription_start_site	+	408593	408593	-1042	16509	AN3674
277	CONTIG61	407346	407755	2.8	2.67E-04	transcription_start_site	+	408939	408939	-1388	16510	AN3674
277	CONTIG61	407346	407755	2.8	2.67E-04	transcription_start_site	+	409196	409196	-1645	16511	AN3674
277	CONTIG61	407346	407755	2.8	2.67E-04	transcription_start_site	+	409777	409777	-2226	16512	AN3674
2444	CONTIG61	410042	410391	1.22	1.44E-01	transcription_start_site	+	409777	409777	439	16512	AN3674
2444	CONTIG61	410042	410391	1.22	1.44E-01	transcription_start_site	+	409196	409196	1020	16511	AN3674
440	CONTIG61	411904	412478	2.53	9.51E-04	transcription_start_site	+	413385	413385	-1194	16513	AN3675
440	CONTIG61	411904	412478	2.53	9.51E-04	transcription_start_site	+	413473	413473	-1282	16514	AN3675
2444	CONTIG61	410042	410391	1.22	1.44E-01	transcription_start_site	+	413385	413385	-3168	16513	AN3675
2444	CONTIG61	410042	410391	1.22	1.44E-01	transcription_start_site	+	413473	413473	-3256	16514	AN3675
2445	CONTIG61	412894	413913	1.22	1.44E-01	transcription_start_site	+	413385	413385	18	16513	AN3675
2445	CONTIG61	412894	413913	1.22	1.44E-01	transcription_start_site	+	413473	413473	-69	16514	AN3675
2595	CONTIG61	421518	422077	1.17	1.66E-01	transcription_start_site	-	418035	418035	-3762	16515	AN3677
2595	CONTIG61	421518	422077	1.17	1.66E-01	transcription_start_site	-	417238	417238	-4559	16516	AN3677
2445	CONTIG61	412894	413913	1.22	1.44E-01	transcription_start_site	+	418426	418426	-5022	16517	AN3678

2590	CONTIG61	423228	424115	1.17	1.59E-01	transcription_start_site	+	422764	422764	907	16524	AN3679
2590	CONTIG61	423228	424115	1.17	1.59E-01	transcription_start_site	+	422384	422384	1287	16523	AN3679
2590	CONTIG61	423228	424115	1.17	1.59E-01	transcription_start_site	+	422267	422267	1404	16522	AN3679
2595	CONTIG61	421518	422077	1.17	1.66E-01	transcription_start_site	+	422151	422151	-353	16521	AN3679
2595	CONTIG61	421518	422077	1.17	1.66E-01	transcription_start_site	+	422267	422267	-469	16522	AN3679
2595	CONTIG61	421518	422077	1.17	1.66E-01	transcription_start_site	+	422384	422384	-586	16523	AN3679
2595	CONTIG61	421518	422077	1.17	1.66E-01	transcription_start_site	+	422764	422764	-966	16524	AN3679
2590	CONTIG61	423228	424115	1.17	1.59E-01	transcription_start_site	+	423586	423586	85	16525	AN3680
2595	CONTIG61	421518	422077	1.17	1.66E-01	transcription_start_site	+	423586	423586	-1788	16525	AN3680
2059	CONTIG61	426164	426521	1.35	9.24E-02	transcription_start_site	+	426508	426508	-165	16527	AN3681
2059	CONTIG61	426164	426521	1.35	9.24E-02	transcription_start_site	+	426055	426055	287	16526	AN3681
2590	CONTIG61	423228	424115	1.17	1.59E-01	transcription_start_site	+	426055	426055	-2383	16526	AN3681
2590	CONTIG61	423228	424115	1.17	1.59E-01	transcription_start_site	+	426508	426508	-2836	16527	AN3681
2595	CONTIG61	421518	422077	1.17	1.66E-01	transcription_start_site	+	426055	426055	-4257	16526	AN3681
2595	CONTIG61	421518	422077	1.17	1.66E-01	transcription_start_site	+	426508	426508	-4710	16527	AN3681
1330	CONTIG61	438232	438594	1.67	3.08E-02	transcription_start_site	-	433364	433364	-5049	16528	AN3683
1724	CONTIG61	429989	430259	1.49	5.88E-02	transcription_start_site	+	435201	435201	-5077	16532	AN3684
1331	CONTIG61	450991	451340	1.67	3.08E-02	transcription_start_site	-	446291	446291	-4874	16537	AN3687
1331	CONTIG61	450991	451340	1.67	3.08E-02	transcription_start_site	-	445997	445997	-5168	16538	AN3687
2694	CONTIG61	449855	450369	1.13	1.93E-01	transcription_start_site	-	446291	446291	-3821	16537	AN3687
2694	CONTIG61	449855	450369	1.13	1.93E-01	transcription_start_site	-	445997	445997	-4115	16538	AN3687
2694	CONTIG61	449855	450369	1.13	1.93E-01	transcription_start_site	-	445813	445813	-4299	16539	AN3687
2694	CONTIG61	449855	450369	1.13	1.93E-01	transcription_start_site	-	445714	445714	-4398	16540	AN3687
2694	CONTIG61	449855	450369	1.13	1.93E-01	transcription_start_site	-	445496	445496	-4616	16541	AN3687
1331	CONTIG61	450991	451340	1.67	3.08E-02	transcription_start_site	+	450194	450194	971	16547	AN3688
2694	CONTIG61	449855	450369	1.13	1.93E-01	transcription_start_site	+	450194	450194	-82	16547	AN3688
2694	CONTIG61	449855	450369	1.13	1.93E-01	transcription_start_site	+	449801	449801	311	16546	AN3688
2694	CONTIG61	449855	450369	1.13	1.93E-01	transcription_start_site	+	449557	449557	555	16545	AN3688
1331	CONTIG61	450991	451340	1.67	3.08E-02	transcription_start_site	+	455026	455026	-3860	16548	AN3689
1331	CONTIG61	450991	451340	1.67	3.08E-02	transcription_start_site	+	455180	455180	-4014	16549	AN3689
1331	CONTIG61	450991	451340	1.67	3.08E-02	transcription_start_site	+	455774	455774	-4608	16550	AN3689
2694	CONTIG61	449855	450369	1.13	1.93E-01	transcription_start_site	+	455026	455026	-4914	16548	AN3689
2694	CONTIG61	449855	450369	1.13	1.93E-01	transcription_start_site	+	455180	455180	-5068	16549	AN3689
1924	CONTIG61	465157	465726	1.4	8.01E-02	transcription_start_site	-	464948	464948	-493	16564	AN3692
1924	CONTIG61	465157	465726	1.4	8.01E-02	transcription_start_site	+	466348	466348	-906	16565	AN3693
1924	CONTIG61	465157	465726	1.4	8.01E-02	transcription_start_site	+	466471	466471	-1029	16566	AN3693
1924	CONTIG61	465157	465726	1.4	8.01E-02	transcription_start_site	+	466583	466583	-1141	16567	AN3693
1924	CONTIG61	465157	465726	1.4	8.01E-02	transcription_start_site	+	466802	466802	-1360	16568	AN3693
1924	CONTIG61	465157	465726	1.4	8.01E-02	transcription_start_site	+	468426	468426	-2984	16569	AN3693
388	CONTIG61	491637	491977	2.62	5.64E-04	transcription_start_site	+	492093	492093	-286	16602	AN3703
388	CONTIG61	491637	491977	2.62	5.64E-04	transcription_start_site	-	492810	492810	1003	16609	AN3704
2440	CONTIG61	496952	497601	1.22	1.28E-01	transcription_start_site	-	494173	494173	-3103	16603	AN3704
2440	CONTIG61	496952	497601	1.22	1.28E-01	transcription_start_site	-	493995	493995	-3281	16604	AN3704
2440	CONTIG61	496952	497601	1.22	1.28E-01	transcription_start_site	-	493482	493482	-3794	16605	AN3704
2440	CONTIG61	496952	497601	1.22	1.28E-01	transcription_start_site	-	493280	493280	-3996	16606	AN3704
2440	CONTIG61	496952	497601	1.22	1.28E-01	transcription_start_site	-	493217	493217	-4059	16607	AN3704
2440	CONTIG61	496952	497601	1.22	1.28E-01	transcription_start_site	-	493052	493052	-4224	16608	AN3704
2440	CONTIG61	496952	497601	1.22	1.28E-01	transcription_start_site	-	492810	492810	-4466	16609	AN3704
2440	CONTIG61	496952	497601	1.22	1.28E-01	transcription_start_site	+	498292	498292	-1015	16610	AN3706
2440	CONTIG61	496952	497601	1.22	1.28E-01	transcription_start_site	+	498350	498350	-1073	16611	AN3706
2440	CONTIG61	496952	497601	1.22	1.28E-01	transcription_start_site	+	498507	498507	-1230	16612	AN3706
2440	CONTIG61	496952	497601	1.22	1.28E-01	transcription_start_site	+	498732	498732	-1455	16613	AN3706
2440	CONTIG61	496952	497601	1.22	1.28E-01	transcription_start_site	+	501282	501282	-4005	16617	AN3708
2440	CONTIG61	496952	497601	1.22	1.28E-01	transcription_start_site	+	501389	501389	-4112	16618	AN3708
2440	CONTIG61	496952	497601	1.22	1.28E-01	transcription_start_site	+	501574	501574	-4297	16619	AN3708
2440	CONTIG61	496952	497601	1.22	1.28E-01	transcription_start_site	+	501858	501858	-4581	16620	AN3708
2440	CONTIG61	496952	497601	1.22	1.28E-01	transcription_start_site	+	502022	502022	-4745	16621	AN3708

2440	CONTIG61	496952	497601	1.22	1.28E-01	transcription_start_site	+	502089	502089	-4812	16622	AN3708
2446	CONTIG61	512719	512978	1.22	1.44E-01	transcription_start_site	+	512925	512925	-76	16639	AN3712
2446	CONTIG61	512719	512978	1.22	1.44E-01	transcription_start_site	+	512770	512770	78	16638	AN3712
2446	CONTIG61	512719	512978	1.22	1.44E-01	transcription_start_site	+	513312	513312	-463	16640	AN3712
2446	CONTIG61	512719	512978	1.22	1.44E-01	transcription_start_site	+	513509	513509	-660	16641	AN3712
730	CONTIG61	515864	516138	2.17	4.61E-03	transcription_start_site	-	515995	515995	-6	16642	AN3713
730	CONTIG61	515864	516138	2.17	4.61E-03	transcription_start_site	-	515706	515706	-295	16643	AN3713
730	CONTIG61	515864	516138	2.17	4.61E-03	transcription_start_site	+	517353	517353	-1352	16644	AN3714
730	CONTIG61	515864	516138	2.17	4.61E-03	transcription_start_site	+	517676	517676	-1675	16645	AN3714
730	CONTIG61	515864	516138	2.17	4.61E-03	transcription_start_site	+	518370	518370	-2369	16646	AN3714
730	CONTIG61	515864	516138	2.17	4.61E-03	transcription_start_site	+	519118	519118	-3117	16647	AN3714
2446	CONTIG61	512719	512978	1.22	1.44E-01	transcription_start_site	+	517353	517353	-4504	16644	AN3714
2446	CONTIG61	512719	512978	1.22	1.44E-01	transcription_start_site	+	517676	517676	-4827	16645	AN3714
2060	CONTIG61	536795	537064	1.35	9.24E-02	transcription_start_site	-	532415	532415	-4514	16653	AN3719
2060	CONTIG61	536795	537064	1.35	9.24E-02	transcription_start_site	-	532256	532256	-4673	16654	AN3719
2060	CONTIG61	536795	537064	1.35	9.24E-02	transcription_start_site	-	532003	532003	-4926	16655	AN3719
2060	CONTIG61	536795	537064	1.35	9.24E-02	transcription_start_site	-	537159	537159	229	16658	AN3720
2060	CONTIG61	536795	537064	1.35	9.24E-02	transcription_start_site	-	536598	536598	-331	16659	AN3720
2060	CONTIG61	536795	537064	1.35	9.24E-02	transcription_start_site	-	534895	534895	-2034	16660	AN3720
2060	CONTIG61	536795	537064	1.35	9.24E-02	transcription_start_site	+	538437	538437	-1507	16661	AN3721
2060	CONTIG61	536795	537064	1.35	9.24E-02	transcription_start_site	+	538759	538759	-1829	16662	AN3721
2060	CONTIG61	536795	537064	1.35	9.24E-02	transcription_start_site	+	540681	540681	-3751	16663	AN3721
1139	CONTIG61	549456	549810	1.8	1.84E-02	transcription_start_site	-	549342	549342	-291	16669	AN3725
1139	CONTIG61	549456	549810	1.8	1.84E-02	transcription_start_site	-	549253	549253	-380	16670	AN3725
1139	CONTIG61	549456	549810	1.8	1.84E-02	transcription_start_site	-	549008	549008	-625	16671	AN3725
1139	CONTIG61	549456	549810	1.8	1.84E-02	transcription_start_site	+	550199	550199	-566	16672	AN3726
1139	CONTIG61	549456	549810	1.8	1.84E-02	transcription_start_site	+	551156	551156	-1523	16673	AN3726
1139	CONTIG61	549456	549810	1.8	1.84E-02	transcription_start_site	+	552000	552000	-2367	16674	AN3726
1139	CONTIG61	549456	549810	1.8	1.84E-02	transcription_start_site	+	552241	552241	-2608	16675	AN3726
1139	CONTIG61	549456	549810	1.8	1.84E-02	transcription_start_site	+	553712	553712	-4079	16676	AN3727
1139	CONTIG61	549456	549810	1.8	1.84E-02	transcription_start_site	+	553938	553938	-4305	16677	AN3727
365	CONTIG61	567152	567450	2.66	6.10E-04	transcription_start_site	-	564109	564109	-3192	16682	AN3729
365	CONTIG61	567152	567450	2.66	6.10E-04	transcription_start_site	-	563895	563895	-3406	16683	AN3729
502	CONTIG61	565142	565494	2.44	0.00E+00	transcription_start_site	-	564109	564109	-1209	16682	AN3729
502	CONTIG61	565142	565494	2.44	0.00E+00	transcription_start_site	-	563895	563895	-1423	16683	AN3729
365	CONTIG61	567152	567450	2.66	6.10E-04	transcription_start_site	+	568900	568900	-1599	16685	AN3730
365	CONTIG61	567152	567450	2.66	6.10E-04	transcription_start_site	+	569048	569048	-1747	16686	AN3730
365	CONTIG61	567152	567450	2.66	6.10E-04	transcription_start_site	+	569141	569141	-1840	16687	AN3730
365	CONTIG61	567152	567450	2.66	6.10E-04	transcription_start_site	+	569228	569228	-1927	16688	AN3730
365	CONTIG61	567152	567450	2.66	6.10E-04	transcription_start_site	+	570030	570030	-2729	16689	AN3730
502	CONTIG61	565142	565494	2.44	0.00E+00	transcription_start_site	+	568900	568900	-3582	16685	AN3730
502	CONTIG61	565142	565494	2.44	0.00E+00	transcription_start_site	+	569048	569048	-3730	16686	AN3730
502	CONTIG61	565142	565494	2.44	0.00E+00	transcription_start_site	+	569141	569141	-3823	16687	AN3730
502	CONTIG61	565142	565494	2.44	0.00E+00	transcription_start_site	+	569228	569228	-3910	16688	AN3730
502	CONTIG61	565142	565494	2.44	0.00E+00	transcription_start_site	+	570030	570030	-4712	16689	AN3730
365	CONTIG61	567152	567450	2.66	6.10E-04	transcription_start_site	+	571692	571692	-4391	16690	AN3731
927	CONTIG61	574587	574868	1.98	8.81E-03	transcription_start_site	-	575755	575755	1027	16696	AN3732
634	CONTIG61	585682	585964	2.26	3.03E-03	transcription_start_site	-	583805	583805	-2018	16699	AN3734
634	CONTIG61	585682	585964	2.26	3.03E-03	transcription_start_site	-	583006	583006	-2817	16700	AN3734
634	CONTIG61	585682	585964	2.26	3.03E-03	transcription_start_site	-	582261	582261	-3562	16701	AN3734
634	CONTIG61	585682	585964	2.26	3.03E-03	transcription_start_site	+	586348	586348	-525	16702	AN3735
634	CONTIG61	585682	585964	2.26	3.03E-03	transcription_start_site	+	589217	589217	-3394	16703	AN3735
1140	CONTIG61	596861	597378	1.8	1.84E-02	transcription_start_site	-	592393	592393	-4726	16704	AN3736
1428	CONTIG61	595587	595861	1.62	3.70E-02	transcription_start_site	-	592393	592393	-3331	16704	AN3736
1428	CONTIG61	595587	595861	1.62	3.70E-02	transcription_start_site	-	591709	591709	-4015	16705	AN3736
1140	CONTIG61	596861	597378	1.8	1.84E-02	transcription_start_site	-	595814	595814	-1305	16709	AN3738
1140	CONTIG61	596861	597378	1.8	1.84E-02	transcription_start_site	-	595248	595248	-1871	16710	AN3738

1428	CONTIG61	595587	595861	1.62	3.70E-02	transcription_start_site	-	595814	595814	90	16709	AN3738
1428	CONTIG61	595587	595861	1.62	3.70E-02	transcription_start_site	-	595248	595248	-476	16710	AN3738
1140	CONTIG61	596861	597378	1.8	1.84E-02	transcription_start_site	+	597983	597983	-863	16711	AN3739
1140	CONTIG61	596861	597378	1.8	1.84E-02	transcription_start_site	+	598480	598480	-1360	16712	AN3739
1140	CONTIG61	596861	597378	1.8	1.84E-02	transcription_start_site	+	598668	598668	-1548	16713	AN3739
1140	CONTIG61	596861	597378	1.8	1.84E-02	transcription_start_site	+	598728	598728	-1608	16714	AN3739
1140	CONTIG61	596861	597378	1.8	1.84E-02	transcription_start_site	+	598879	598879	-1759	16715	AN3739
1140	CONTIG61	596861	597378	1.8	1.84E-02	transcription_start_site	+	599082	599082	-1962	16716	AN3739
1140	CONTIG61	596861	597378	1.8	1.84E-02	transcription_start_site	+	599245	599245	-2125	16717	AN3739
1140	CONTIG61	596861	597378	1.8	1.84E-02	transcription_start_site	+	599722	599722	-2602	16718	AN3739
1140	CONTIG61	596861	597378	1.8	1.84E-02	transcription_start_site	+	599926	599926	-2806	16719	AN3739
1428	CONTIG61	595587	595861	1.62	3.70E-02	transcription_start_site	+	597983	597983	-2259	16711	AN3739
1428	CONTIG61	595587	595861	1.62	3.70E-02	transcription_start_site	+	598480	598480	-2756	16712	AN3739
1428	CONTIG61	595587	595861	1.62	3.70E-02	transcription_start_site	+	598668	598668	-2944	16713	AN3739
1428	CONTIG61	595587	595861	1.62	3.70E-02	transcription_start_site	+	598728	598728	-3004	16714	AN3739
1428	CONTIG61	595587	595861	1.62	3.70E-02	transcription_start_site	+	598879	598879	-3155	16715	AN3739
1428	CONTIG61	595587	595861	1.62	3.70E-02	transcription_start_site	+	599082	599082	-3358	16716	AN3739
1428	CONTIG61	595587	595861	1.62	3.70E-02	transcription_start_site	+	599245	599245	-3521	16717	AN3739
1428	CONTIG61	595587	595861	1.62	3.70E-02	transcription_start_site	+	599722	599722	-3998	16718	AN3739
1428	CONTIG61	595587	595861	1.62	3.70E-02	transcription_start_site	+	599926	599926	-4202	16719	AN3739
1504	CONTIG61	609751	610120	1.58	2.64E-02	transcription_start_site	-	605893	605893	-4042	16726	AN3742
1504	CONTIG61	609751	610120	1.58	2.64E-02	transcription_start_site	-	605346	605346	-4589	16727	AN3742
1504	CONTIG61	609751	610120	1.58	2.64E-02	transcription_start_site	-	610096	610096	160	16729	AN3743
1504	CONTIG61	609751	610120	1.58	2.64E-02	transcription_start_site	-	610426	610426	490	16728	AN3743
1504	CONTIG61	609751	610120	1.58	2.64E-02	transcription_start_site	+	613027	613027	-3091	16732	AN3745
1504	CONTIG61	609751	610120	1.58	2.64E-02	transcription_start_site	+	613143	613143	-3207	16733	AN3745
870	CONTIG61	618311	618590	2.03	7.85E-03	transcription_start_site	-	616710	616710	-1740	16734	AN3746
870	CONTIG61	618311	618590	2.03	7.85E-03	transcription_start_site	-	616133	616133	-2317	16735	AN3746
870	CONTIG61	618311	618590	2.03	7.85E-03	transcription_start_site	-	615427	615427	-3023	16736	AN3746
870	CONTIG61	618311	618590	2.03	7.85E-03	transcription_start_site	-	615250	615250	-3200	16737	AN3746
870	CONTIG61	618311	618590	2.03	7.85E-03	transcription_start_site	+	619870	619870	-1419	16738	AN3747
870	CONTIG61	618311	618590	2.03	7.85E-03	transcription_start_site	+	620037	620037	-1586	16739	AN3747
870	CONTIG61	618311	618590	2.03	7.85E-03	transcription_start_site	+	620383	620383	-1932	16740	AN3747
870	CONTIG61	618311	618590	2.03	7.85E-03	transcription_start_site	+	620883	620883	-2432	16741	AN3747
870	CONTIG61	618311	618590	2.03	7.85E-03	transcription_start_site	+	621064	621064	-2613	16742	AN3747
2336	CONTIG61	639695	640264	1.26	1.25E-01	transcription_start_site	-	634997	634997	-4982	16751	AN3750
2596	CONTIG61	638705	639144	1.17	1.66E-01	transcription_start_site	-	634997	634997	-3927	16751	AN3750
2596	CONTIG61	638705	639144	1.17	1.66E-01	transcription_start_site	-	634544	634544	-4380	16752	AN3750
41	CONTIG61	644405	644834	3.47	0.00E+00	transcription_start_site	-	640367	640367	-4252	16753	AN3751
605	CONTIG61	642920	643184	2.3	2.22E-03	transcription_start_site	-	640367	640367	-2685	16753	AN3751
635	CONTIG61	640365	640644	2.26	3.03E-03	transcription_start_site	-	640367	640367	-137	16753	AN3751
1831	CONTIG61	643505	644079	1.44	6.84E-02	transcription_start_site	-	640367	640367	-3425	16753	AN3751
2336	CONTIG61	639695	640264	1.26	1.25E-01	transcription_start_site	-	640367	640367	387	16753	AN3751
41	CONTIG61	644405	644834	3.47	0.00E+00	transcription_start_site	-	643505	643505	-1114	16754	AN3752
41	CONTIG61	644405	644834	3.47	0.00E+00	transcription_start_site	-	641835	641835	-2784	16755	AN3752
605	CONTIG61	642920	643184	2.3	2.22E-03	transcription_start_site	-	643505	643505	453	16754	AN3752
605	CONTIG61	642920	643184	2.3	2.22E-03	transcription_start_site	-	641835	641835	-1217	16755	AN3752
1831	CONTIG61	643505	644079	1.44	6.84E-02	transcription_start_site	-	643505	643505	-287	16754	AN3752
1831	CONTIG61	643505	644079	1.44	6.84E-02	transcription_start_site	-	641835	641835	-1957	16755	AN3752
1200	CONTIG61	651680	651954	1.76	2.12E-02	transcription_start_site	-	648671	648671	-3146	16756	AN3753
1200	CONTIG61	651680	651954	1.76	2.12E-02	transcription_start_site	-	648144	648144	-3673	16757	AN3753
1200	CONTIG61	651680	651954	1.76	2.12E-02	transcription_start_site	-	647791	647791	-4026	16758	AN3753
1200	CONTIG61	651680	651954	1.76	2.12E-02	transcription_start_site	-	647487	647487	-4330	16759	AN3753
1200	CONTIG61	651680	651954	1.76	2.12E-02	transcription_start_site	-	647080	647080	-4737	16760	AN3753
1725	CONTIG61	650045	650389	1.49	5.88E-02	transcription_start_site	-	648671	648671	-1546	16756	AN3753
1725	CONTIG61	650045	650389	1.49	5.88E-02	transcription_start_site	-	648144	648144	-2073	16757	AN3753
1725	CONTIG61	650045	650389	1.49	5.88E-02	transcription_start_site	-	647791	647791	-2426	16758	AN3753

1725	CONTIG61	650045	650389	1.49	5.88E-02	transcription_start_site	-	647487	647487	-2730	16759	AN3753
1725	CONTIG61	650045	650389	1.49	5.88E-02	transcription_start_site	-	647080	647080	-3137	16760	AN3753
1725	CONTIG61	650045	650389	1.49	5.88E-02	transcription_start_site	-	646620	646620	-3597	16761	AN3753
2165	CONTIG61	663469	664408	1.31	9.26E-02	transcription_start_site	-	663903	663903	-35	16762	AN3755
2165	CONTIG61	663469	664408	1.31	9.26E-02	transcription_start_site	-	662661	662661	-1277	16763	AN3755
1024	CONTIG61	669164	669433	1.89	1.30E-02	transcription_start_site	-	666437	666437	-2861	16764	AN3756
1024	CONTIG61	669164	669433	1.89	1.30E-02	transcription_start_site	-	666336	666336	-2962	16765	AN3756
1024	CONTIG61	669164	669433	1.89	1.30E-02	transcription_start_site	-	666231	666231	-3067	16766	AN3756
1024	CONTIG61	669164	669433	1.89	1.30E-02	transcription_start_site	-	666156	666156	-3142	16767	AN3756
1024	CONTIG61	669164	669433	1.89	1.30E-02	transcription_start_site	-	665824	665824	-3474	16768	AN3756
2165	CONTIG61	663469	664408	1.31	9.26E-02	transcription_start_site	+	666982	666982	-3043	16769	AN3757
2165	CONTIG61	663469	664408	1.31	9.26E-02	transcription_start_site	+	667124	667124	-3185	16770	AN3757
2165	CONTIG61	663469	664408	1.31	9.26E-02	transcription_start_site	+	667660	667660	-3721	16771	AN3757
2165	CONTIG61	663469	664408	1.31	9.26E-02	transcription_start_site	+	667887	667887	-3948	16772	AN3757
1024	CONTIG61	669164	669433	1.89	1.30E-02	transcription_start_site	-	669428	669428	129	16774	AN3758
1024	CONTIG61	669164	669433	1.89	1.30E-02	transcription_start_site	-	669663	669663	364	16773	AN3758
1024	CONTIG61	669164	669433	1.89	1.30E-02	transcription_start_site	+	670305	670305	-1006	16775	AN3759
1024	CONTIG61	669164	669433	1.89	1.30E-02	transcription_start_site	+	670563	670563	-1264	16776	AN3759
1024	CONTIG61	669164	669433	1.89	1.30E-02	transcription_start_site	+	670749	670749	-1450	16777	AN3759
1024	CONTIG61	669164	669433	1.89	1.30E-02	transcription_start_site	+	671096	671096	-1797	16778	AN3759
1024	CONTIG61	669164	669433	1.89	1.30E-02	transcription_start_site	+	672631	672631	-3332	16779	AN3760
1024	CONTIG61	669164	669433	1.89	1.30E-02	transcription_start_site	+	672738	672738	-3439	16780	AN3760
1024	CONTIG61	669164	669433	1.89	1.30E-02	transcription_start_site	+	673059	673059	-3760	16781	AN3760
1501	CONTIG61	674707	675436	1.58	2.45E-02	transcription_start_site	+	675037	675037	34	16782	AN3761
420	CONTIG61	681753	682184	2.57	8.69E-04	transcription_start_site	+	681182	681182	786	16785	AN3762
420	CONTIG61	681753	682184	2.57	8.69E-04	transcription_start_site	+	680833	680833	1135	16784	AN3762
420	CONTIG61	681753	682184	2.57	8.69E-04	transcription_start_site	+	682468	682468	-499	16786	AN3763
420	CONTIG61	681753	682184	2.57	8.69E-04	transcription_start_site	+	682781	682781	-812	16787	AN3763
420	CONTIG61	681753	682184	2.57	8.69E-04	transcription_start_site	+	683441	683441	-1472	16788	AN3763
420	CONTIG61	681753	682184	2.57	8.69E-04	transcription_start_site	+	685327	685327	-3358	16789	AN3764
420	CONTIG61	681753	682184	2.57	8.69E-04	transcription_start_site	+	685458	685458	-3489	16790	AN3764
420	CONTIG61	681753	682184	2.57	8.69E-04	transcription_start_site	+	686301	686301	-4332	16791	AN3764
420	CONTIG61	681753	682184	2.57	8.69E-04	transcription_start_site	+	686581	686581	-4612	16792	AN3764
543	CONTIG61	694357	694626	2.39	1.54E-03	transcription_start_site	-	692136	692136	-2355	16794	AN3765
543	CONTIG61	694357	694626	2.39	1.54E-03	transcription_start_site	-	691491	691491	-3000	16795	AN3765
2328	CONTIG61	696917	697416	1.26	1.07E-01	transcription_start_site	-	692136	692136	-5030	16794	AN3765
1274	CONTIG61	718432	718711	1.71	2.62E-02	transcription_start_site	-	717187	717187	-1384	16805	AN3770
1925	CONTIG61	722179	722603	1.4	8.01E-02	transcription_start_site	-	717187	717187	-5204	16805	AN3770
1274	CONTIG61	718432	718711	1.71	2.62E-02	transcription_start_site	+	717903	717903	668	16809	AN3771
1274	CONTIG61	718432	718711	1.71	2.62E-02	transcription_start_site	+	717721	717721	850	16808	AN3771
1274	CONTIG61	718432	718711	1.71	2.62E-02	transcription_start_site	+	717564	717564	1007	16807	AN3771
1274	CONTIG61	718432	718711	1.71	2.62E-02	transcription_start_site	+	717441	717441	1130	16806	AN3771
1074	CONTIG61	725859	726128	1.85	1.57E-02	transcription_start_site	-	723021	723021	-2972	16810	AN3773
1074	CONTIG61	725859	726128	1.85	1.57E-02	transcription_start_site	-	722329	722329	-3664	16811	AN3773
1925	CONTIG61	722179	722603	1.4	8.01E-02	transcription_start_site	-	722329	722329	-62	16811	AN3773
1925	CONTIG61	722179	722603	1.4	8.01E-02	transcription_start_site	-	723021	723021	630	16810	AN3773
1075	CONTIG61	732079	732353	1.85	1.57E-02	transcription_start_site	-	727912	727912	-4304	16812	AN3774
1075	CONTIG61	732079	732353	1.85	1.57E-02	transcription_start_site	-	727765	727765	-4451	16813	AN3774
1075	CONTIG61	732079	732353	1.85	1.57E-02	transcription_start_site	-	727490	727490	-4726	16814	AN3774
1074	CONTIG61	725859	726128	1.85	1.57E-02	transcription_start_site	+	729127	729127	-3133	16815	AN3775
1074	CONTIG61	725859	726128	1.85	1.57E-02	transcription_start_site	+	729693	729693	-3699	16816	AN3775
1075	CONTIG61	732079	732353	1.85	1.57E-02	transcription_start_site	-	732172	732172	-44	16817	AN3776
1075	CONTIG61	732079	732353	1.85	1.57E-02	transcription_start_site	-	731450	731450	-766	16818	AN3776
1075	CONTIG61	732079	732353	1.85	1.57E-02	transcription_start_site	+	734492	734492	-2276	16819	AN3777
1075	CONTIG61	732079	732353	1.85	1.57E-02	transcription_start_site	+	735103	735103	-2887	16820	AN3777
1075	CONTIG61	732079	732353	1.85	1.57E-02	transcription_start_site	+	735359	735359	-3143	16821	AN3777
1075	CONTIG61	732079	732353	1.85	1.57E-02	transcription_start_site	+	735819	735819	-3603	16822	AN3777

278	CONTIG61	750467	750741	2.8	2.67E-04	transcription_start_site	-	746289	746289	-4315	16829	AN3781
278	CONTIG61	750467	750741	2.8	2.67E-04	transcription_start_site	-	746052	746052	-4552	16830	AN3781
777	CONTIG61	749412	749901	2.12	5.20E-03	transcription_start_site	-	746289	746289	-3367	16829	AN3781
777	CONTIG61	749412	749901	2.12	5.20E-03	transcription_start_site	-	746052	746052	-3604	16830	AN3781
777	CONTIG61	749412	749901	2.12	5.20E-03	transcription_start_site	-	744508	744508	-5148	16831	AN3781
867	CONTIG61	745962	746991	2.03	3.31E-03	transcription_start_site	-	746289	746289	-187	16829	AN3781
867	CONTIG61	745962	746991	2.03	3.31E-03	transcription_start_site	-	746052	746052	-424	16830	AN3781
867	CONTIG61	745962	746991	2.03	3.31E-03	transcription_start_site	-	744508	744508	-1968	16831	AN3781
278	CONTIG61	750467	750741	2.8	2.67E-04	transcription_start_site	-	749332	749332	-1272	16832	AN3782
777	CONTIG61	749412	749901	2.12	5.20E-03	transcription_start_site	-	749332	749332	-324	16832	AN3782
278	CONTIG61	750467	750741	2.8	2.67E-04	transcription_start_site	-	750396	750396	-208	16833	AN3783
278	CONTIG61	750467	750741	2.8	2.67E-04	transcription_start_site	-	750292	750292	-312	16834	AN3783
777	CONTIG61	749412	749901	2.12	5.20E-03	transcription_start_site	-	750292	750292	635	16834	AN3783
777	CONTIG61	749412	749901	2.12	5.20E-03	transcription_start_site	-	750396	750396	739	16833	AN3783
278	CONTIG61	750467	750741	2.8	2.67E-04	transcription_start_site	+	752439	752439	-1835	16835	AN3784
278	CONTIG61	750467	750741	2.8	2.67E-04	transcription_start_site	+	752603	752603	-1999	16836	AN3784
278	CONTIG61	750467	750741	2.8	2.67E-04	transcription_start_site	+	752802	752802	-2198	16837	AN3784
278	CONTIG61	750467	750741	2.8	2.67E-04	transcription_start_site	+	753231	753231	-2627	16838	AN3784
777	CONTIG61	749412	749901	2.12	5.20E-03	transcription_start_site	+	752439	752439	-2782	16835	AN3784
777	CONTIG61	749412	749901	2.12	5.20E-03	transcription_start_site	+	752603	752603	-2946	16836	AN3784
777	CONTIG61	749412	749901	2.12	5.20E-03	transcription_start_site	+	752802	752802	-3145	16837	AN3784
777	CONTIG61	749412	749901	2.12	5.20E-03	transcription_start_site	+	753231	753231	-3574	16838	AN3784
1069	CONTIG61	758032	758676	1.85	3.83E-03	transcription_start_site	-	756366	756366	-1988	16839	AN3785
1069	CONTIG61	758032	758676	1.85	3.83E-03	transcription_start_site	-	756201	756201	-2153	16840	AN3785
1069	CONTIG61	758032	758676	1.85	3.83E-03	transcription_start_site	-	756119	756119	-2235	16841	AN3785
1069	CONTIG61	758032	758676	1.85	3.83E-03	transcription_start_site	-	755962	755962	-2392	16842	AN3785
1069	CONTIG61	758032	758676	1.85	3.83E-03	transcription_start_site	-	755667	755667	-2687	16843	AN3785
1069	CONTIG61	758032	758676	1.85	3.83E-03	transcription_start_site	-	754604	754604	-3750	16844	AN3785
2061	CONTIG61	759677	760036	1.35	9.24E-02	transcription_start_site	-	756366	756366	-3490	16839	AN3785
2061	CONTIG61	759677	760036	1.35	9.24E-02	transcription_start_site	-	756201	756201	-3655	16840	AN3785
2061	CONTIG61	759677	760036	1.35	9.24E-02	transcription_start_site	-	756119	756119	-3737	16841	AN3785
2061	CONTIG61	759677	760036	1.35	9.24E-02	transcription_start_site	-	755962	755962	-3894	16842	AN3785
2061	CONTIG61	759677	760036	1.35	9.24E-02	transcription_start_site	-	755667	755667	-4189	16843	AN3785
2447	CONTIG61	756467	756811	1.22	1.44E-01	transcription_start_site	-	756366	756366	-273	16839	AN3785
2447	CONTIG61	756467	756811	1.22	1.44E-01	transcription_start_site	-	756201	756201	-438	16840	AN3785
2447	CONTIG61	756467	756811	1.22	1.44E-01	transcription_start_site	-	756119	756119	-520	16841	AN3785
2447	CONTIG61	756467	756811	1.22	1.44E-01	transcription_start_site	-	755962	755962	-677	16842	AN3785
2447	CONTIG61	756467	756811	1.22	1.44E-01	transcription_start_site	-	755667	755667	-972	16843	AN3785
2447	CONTIG61	756467	756811	1.22	1.44E-01	transcription_start_site	-	754604	754604	-2035	16844	AN3785
1069	CONTIG61	758032	758676	1.85	3.83E-03	transcription_start_site	-	759369	759369	1015	16849	AN3786
1069	CONTIG61	758032	758676	1.85	3.83E-03	transcription_start_site	-	759561	759561	1207	16848	AN3786
1069	CONTIG61	758032	758676	1.85	3.83E-03	transcription_start_site	-	759626	759626	1272	16847	AN3786
2061	CONTIG61	759677	760036	1.35	9.24E-02	transcription_start_site	-	759842	759842	-14	16846	AN3786
2061	CONTIG61	759677	760036	1.35	9.24E-02	transcription_start_site	-	760021	760021	164	16845	AN3786
2061	CONTIG61	759677	760036	1.35	9.24E-02	transcription_start_site	-	759626	759626	-230	16847	AN3786
2061	CONTIG61	759677	760036	1.35	9.24E-02	transcription_start_site	-	759561	759561	-295	16848	AN3786
2061	CONTIG61	759677	760036	1.35	9.24E-02	transcription_start_site	-	759369	759369	-487	16849	AN3786
1069	CONTIG61	758032	758676	1.85	3.83E-03	transcription_start_site	+	760528	760528	-2174	16850	AN3787
1069	CONTIG61	758032	758676	1.85	3.83E-03	transcription_start_site	+	761108	761108	-2754	16851	AN3787
1069	CONTIG61	758032	758676	1.85	3.83E-03	transcription_start_site	+	761560	761560	-3206	16852	AN3787
2061	CONTIG61	759677	760036	1.35	9.24E-02	transcription_start_site	+	760528	760528	-671	16850	AN3787
2061	CONTIG61	759677	760036	1.35	9.24E-02	transcription_start_site	+	761108	761108	-1251	16851	AN3787
2061	CONTIG61	759677	760036	1.35	9.24E-02	transcription_start_site	+	761560	761560	-1703	16852	AN3787
2447	CONTIG61	756467	756811	1.22	1.44E-01	transcription_start_site	+	760528	760528	-3889	16850	AN3787
2447	CONTIG61	756467	756811	1.22	1.44E-01	transcription_start_site	+	761108	761108	-4469	16851	AN3787
2447	CONTIG61	756467	756811	1.22	1.44E-01	transcription_start_site	+	761560	761560	-4921	16852	AN3787
871	CONTIG61	775446	776000	2.03	7.85E-03	transcription_start_site	+	776620	776620	-897	16866	AN3793

871	CONTIG61	775446	776000	2.03	7.85E-03	transcription_start_site	+	778023	778023	-2300	16867	AN3793
871	CONTIG61	775446	776000	2.03	7.85E-03	transcription_start_site	+	778316	778316	-2593	16868	AN3793
2448	CONTIG61	778146	778485	1.22	1.44E-01	transcription_start_site	+	778316	778316	0	16868	AN3793
2448	CONTIG61	778146	778485	1.22	1.44E-01	transcription_start_site	+	778023	778023	292	16867	AN3793
2441	CONTIG61	785476	785900	1.22	1.28E-01	transcription_start_site	-	782738	782738	-2950	16869	AN3794
2441	CONTIG61	785476	785900	1.22	1.28E-01	transcription_start_site	-	781635	781635	-4053	16870	AN3794
2441	CONTIG61	785476	785900	1.22	1.28E-01	transcription_start_site	-	781337	781337	-4351	16871	AN3794
2695	CONTIG61	780751	781100	1.13	1.93E-01	transcription_start_site	-	781337	781337	411	16871	AN3794
2695	CONTIG61	780751	781100	1.13	1.93E-01	transcription_start_site	-	781635	781635	709	16870	AN3794
2337	CONTIG61	802201	802475	1.26	1.25E-01	transcription_start_site	-	801503	801503	-835	16876	AN3797
2337	CONTIG61	802201	802475	1.26	1.25E-01	transcription_start_site	-	801071	801071	-1267	16877	AN3797
2337	CONTIG61	802201	802475	1.26	1.25E-01	transcription_start_site	+	801861	801861	477	16878	AN3798
2337	CONTIG61	802201	802475	1.26	1.25E-01	transcription_start_site	+	805564	805564	-3226	16880	AN3800
2337	CONTIG61	802201	802475	1.26	1.25E-01	transcription_start_site	+	805725	805725	-3387	16881	AN3800
2337	CONTIG61	802201	802475	1.26	1.25E-01	transcription_start_site	+	805835	805835	-3497	16882	AN3800
2337	CONTIG61	802201	802475	1.26	1.25E-01	transcription_start_site	+	806015	806015	-3677	16883	AN3800
2337	CONTIG61	802201	802475	1.26	1.25E-01	transcription_start_site	+	806152	806152	-3814	16884	AN3800
2337	CONTIG61	802201	802475	1.26	1.25E-01	transcription_start_site	+	806291	806291	-3953	16885	AN3800
537	CONTIG61	812104	812913	2.39	0.00E+00	transcription_start_site	-	809767	809767	-2741	16886	AN3801
537	CONTIG61	812104	812913	2.39	0.00E+00	transcription_start_site	-	809393	809393	-3115	16887	AN3801
537	CONTIG61	812104	812913	2.39	0.00E+00	transcription_start_site	-	809164	809164	-3344	16888	AN3801
537	CONTIG61	812104	812913	2.39	0.00E+00	transcription_start_site	-	809003	809003	-3505	16889	AN3801
537	CONTIG61	812104	812913	2.39	0.00E+00	transcription_start_site	-	808616	808616	-3892	16890	AN3801
537	CONTIG61	812104	812913	2.39	0.00E+00	transcription_start_site	-	808493	808493	-4015	16891	AN3801
537	CONTIG61	812104	812913	2.39	0.00E+00	transcription_start_site	-	808323	808323	-4185	16892	AN3801
537	CONTIG61	812104	812913	2.39	0.00E+00	transcription_start_site	-	808081	808081	-4427	16893	AN3801
537	CONTIG61	812104	812913	2.39	0.00E+00	transcription_start_site	-	807852	807852	-4656	16894	AN3801
537	CONTIG61	812104	812913	2.39	0.00E+00	transcription_start_site	-	807699	807699	-4809	16895	AN3801
537	CONTIG61	812104	812913	2.39	0.00E+00	transcription_start_site	-	807624	807624	-4884	16896	AN3801
537	CONTIG61	812104	812913	2.39	0.00E+00	transcription_start_site	-	807502	807502	-5006	16897	AN3801
537	CONTIG61	812104	812913	2.39	0.00E+00	transcription_start_site	-	807347	807347	-5161	16898	AN3801
537	CONTIG61	812104	812913	2.39	0.00E+00	transcription_start_site	-	807161	807161	-5347	16899	AN3801
361	CONTIG61	816234	817328	2.66	0.00E+00	transcription_start_site	+	818986	818986	-2205	16907	AN3803
361	CONTIG61	816234	817328	2.66	0.00E+00	transcription_start_site	+	819215	819215	-2434	16908	AN3803
361	CONTIG61	816234	817328	2.66	0.00E+00	transcription_start_site	+	819417	819417	-2636	16909	AN3803
1275	CONTIG61	815119	815912	1.71	2.62E-02	transcription_start_site	+	818986	818986	-3470	16907	AN3803
1275	CONTIG61	815119	815912	1.71	2.62E-02	transcription_start_site	+	819215	819215	-3699	16908	AN3803
1275	CONTIG61	815119	815912	1.71	2.62E-02	transcription_start_site	+	819417	819417	-3901	16909	AN3803
2696	CONTIG61	819094	819503	1.13	1.93E-01	transcription_start_site	+	819215	819215	83	16908	AN3803
2696	CONTIG61	819094	819503	1.13	1.93E-01	transcription_start_site	+	819417	819417	-118	16909	AN3803
2696	CONTIG61	819094	819503	1.13	1.93E-01	transcription_start_site	+	818986	818986	312	16907	AN3803
42	CONTIG61	824554	824834	3.47	0.00E+00	transcription_start_site	-	822828	822828	-1866	16910	AN3804
1622	CONTIG61	822004	822890	1.53	5.22E-02	transcription_start_site	-	822828	822828	381	16910	AN3804
2338	CONTIG61	826204	826718	1.26	1.25E-01	transcription_start_site	-	822828	822828	-3633	16910	AN3804
2338	CONTIG61	826204	826718	1.26	1.25E-01	transcription_start_site	-	825957	825957	-504	16911	AN3805
42	CONTIG61	824554	824834	3.47	0.00E+00	transcription_start_site	+	826512	826512	-1818	16912	AN3806
1622	CONTIG61	822004	822890	1.53	5.22E-02	transcription_start_site	+	826512	826512	-4065	16912	AN3806
2338	CONTIG61	826204	826718	1.26	1.25E-01	transcription_start_site	+	826512	826512	-51	16912	AN3806
1832	CONTIG61	838804	839168	1.44	6.84E-02	transcription_start_site	-	838171	838171	-815	16927	AN3811
1832	CONTIG61	838804	839168	1.44	6.84E-02	transcription_start_site	-	838010	838010	-976	16928	AN3811
2697	CONTIG61	840009	840733	1.13	1.93E-01	transcription_start_site	-	838171	838171	-2200	16927	AN3811
2697	CONTIG61	840009	840733	1.13	1.93E-01	transcription_start_site	-	838010	838010	-2361	16928	AN3811
1832	CONTIG61	838804	839168	1.44	6.84E-02	transcription_start_site	+	838932	838932	54	16930	AN3812
1832	CONTIG61	838804	839168	1.44	6.84E-02	transcription_start_site	+	838567	838567	419	16929	AN3812
1832	CONTIG61	838804	839168	1.44	6.84E-02	transcription_start_site	+	840297	840297	-1311	16931	AN3813
1832	CONTIG61	838804	839168	1.44	6.84E-02	transcription_start_site	+	840417	840417	-1431	16932	AN3813
1832	CONTIG61	838804	839168	1.44	6.84E-02	transcription_start_site	+	840511	840511	-1525	16933	AN3813

1832	CONTIG61	838804	839168	1.44	6.84E-02	transcription_start_site	+	841163	841163	-2177	16934	AN3813
2697	CONTIG61	840009	840733	1.13	1.93E-01	transcription_start_site	+	840417	840417	-46	16932	AN3813
2697	CONTIG61	840009	840733	1.13	1.93E-01	transcription_start_site	+	840297	840297	74	16931	AN3813
2697	CONTIG61	840009	840733	1.13	1.93E-01	transcription_start_site	+	840511	840511	-140	16933	AN3813
2697	CONTIG61	840009	840733	1.13	1.93E-01	transcription_start_site	+	841163	841163	-792	16934	AN3813
1201	CONTIG61	852839	853108	1.76	2.12E-02	transcription_start_site	-	851966	851966	-1007	16943	AN3817
1201	CONTIG61	852839	853108	1.76	2.12E-02	transcription_start_site	-	851566	851566	-1407	16944	AN3817
1201	CONTIG61	852839	853108	1.76	2.12E-02	transcription_start_site	-	851150	851150	-1823	16945	AN3817
1201	CONTIG61	852839	853108	1.76	2.12E-02	transcription_start_site	-	848789	848789	-4184	16946	AN3817
1201	CONTIG61	852839	853108	1.76	2.12E-02	transcription_start_site	+	855771	855771	-2797	16947	AN3818
1911	CONTIG62	3691	3965	1.4	3.08E-02	transcription_start_site	+	3106	3106	722	16960	AN3822
1911	CONTIG62	3691	3965	1.4	3.08E-02	transcription_start_site	+	2701	2701	1127	16959	AN3822
2973	CONTIG62	526	895	0.95	1.93E-01	transcription_start_site	+	2391	2391	-1680	16958	AN3822
2973	CONTIG62	526	895	0.95	1.93E-01	transcription_start_site	+	2701	2701	-1990	16959	AN3822
2973	CONTIG62	526	895	0.95	1.93E-01	transcription_start_site	+	3106	3106	-2395	16960	AN3822
1911	CONTIG62	3691	3965	1.4	3.08E-02	transcription_start_site	+	3962	3962	-134	16961	AN3823
1911	CONTIG62	3691	3965	1.4	3.08E-02	transcription_start_site	+	4255	4255	-427	16962	AN3823
2973	CONTIG62	526	895	0.95	1.93E-01	transcription_start_site	+	3962	3962	-3251	16961	AN3823
2973	CONTIG62	526	895	0.95	1.93E-01	transcription_start_site	+	4255	4255	-3544	16962	AN3823
1068	CONTIG62	31958	32532	1.85	3.61E-03	transcription_start_site	-	30721	30721	-1524	16990	AN3833
1068	CONTIG62	31958	32532	1.85	3.61E-03	transcription_start_site	-	30535	30535	-1710	16991	AN3833
1068	CONTIG62	31958	32532	1.85	3.61E-03	transcription_start_site	-	30406	30406	-1839	16992	AN3833
1068	CONTIG62	31958	32532	1.85	3.61E-03	transcription_start_site	-	30151	30151	-2094	16993	AN3833
1068	CONTIG62	31958	32532	1.85	3.61E-03	transcription_start_site	-	29289	29289	-2956	16994	AN3833
1068	CONTIG62	31958	32532	1.85	3.61E-03	transcription_start_site	+	31584	31584	661	16997	AN3834
1068	CONTIG62	31958	32532	1.85	3.61E-03	transcription_start_site	+	31219	31219	1026	16996	AN3834
1068	CONTIG62	31958	32532	1.85	3.61E-03	transcription_start_site	+	31156	31156	1089	16995	AN3834
1068	CONTIG62	31958	32532	1.85	3.61E-03	transcription_start_site	+	36671	36671	-4426	16998	AN3835
1068	CONTIG62	31958	32532	1.85	3.61E-03	transcription_start_site	+	36825	36825	-4580	16999	AN3835
1068	CONTIG62	31958	32532	1.85	3.61E-03	transcription_start_site	+	36965	36965	-4720	17000	AN3835
2943	CONTIG62	36226	36575	0.98	1.66E-01	transcription_start_site	+	36671	36671	-270	16998	AN3835
2943	CONTIG62	36226	36575	0.98	1.66E-01	transcription_start_site	+	36825	36825	-424	16999	AN3835
2943	CONTIG62	36226	36575	0.98	1.66E-01	transcription_start_site	+	36965	36965	-564	17000	AN3835
2943	CONTIG62	36226	36575	0.98	1.66E-01	transcription_start_site	+	37938	37938	-1537	17001	AN3835
2943	CONTIG62	36226	36575	0.98	1.66E-01	transcription_start_site	+	38687	38687	-2286	17002	AN3835
2968	CONTIG62	39988	40942	0.95	1.79E-01	transcription_start_site	-	40525	40525	60	17003	AN3836
2943	CONTIG62	36226	36575	0.98	1.66E-01	transcription_start_site	+	40913	40913	-4512	17004	AN3837
2968	CONTIG62	39988	40942	0.95	1.79E-01	transcription_start_site	+	40913	40913	-448	17004	AN3837
2968	CONTIG62	39988	40942	0.95	1.79E-01	transcription_start_site	+	42499	42499	-2034	17005	AN3837
2809	CONTIG62	52731	53094	1.06	1.25E-01	transcription_start_site	-	49860	49860	-3052	17013	AN3840
2809	CONTIG62	52731	53094	1.06	1.25E-01	transcription_start_site	-	49789	49789	-3123	17014	AN3840
2809	CONTIG62	52731	53094	1.06	1.25E-01	transcription_start_site	-	49581	49581	-3331	17015	AN3840
2809	CONTIG62	52731	53094	1.06	1.25E-01	transcription_start_site	-	52629	52629	-283	17016	AN3841
2809	CONTIG62	52731	53094	1.06	1.25E-01	transcription_start_site	-	52354	52354	-558	17017	AN3841
2809	CONTIG62	52731	53094	1.06	1.25E-01	transcription_start_site	-	50563	50563	-2349	17018	AN3841
2809	CONTIG62	52731	53094	1.06	1.25E-01	transcription_start_site	+	53246	53246	-333	17019	AN3842
2809	CONTIG62	52731	53094	1.06	1.25E-01	transcription_start_site	+	53398	53398	-485	17020	AN3842
2809	CONTIG62	52731	53094	1.06	1.25E-01	transcription_start_site	+	53475	53475	-562	17021	AN3842
2809	CONTIG62	52731	53094	1.06	1.25E-01	transcription_start_site	+	53549	53549	-636	17022	AN3842
2809	CONTIG62	52731	53094	1.06	1.25E-01	transcription_start_site	+	53806	53806	-893	17023	AN3842
2809	CONTIG62	52731	53094	1.06	1.25E-01	transcription_start_site	+	55055	55055	-2142	17024	AN3843
2809	CONTIG62	52731	53094	1.06	1.25E-01	transcription_start_site	+	55233	55233	-2320	17025	AN3843
2809	CONTIG62	52731	53094	1.06	1.25E-01	transcription_start_site	+	55392	55392	-2479	17026	AN3843
2809	CONTIG62	52731	53094	1.06	1.25E-01	transcription_start_site	+	57063	57063	-4150	17027	AN3843
2809	CONTIG62	52731	53094	1.06	1.25E-01	transcription_start_site	+	57633	57633	-4720	17028	AN3844
489	CONTIG62	63302	63738	2.46	0.00E+00	transcription_start_site	-	63795	63795	275	17030	AN3845
489	CONTIG62	63302	63738	2.46	0.00E+00	transcription_start_site	-	64111	64111	591	17029	AN3845

2940	CONTIG62	64579	65392	0.98	1.50E-01	transcription_start_site	-	64111	64111	-874	17029	AN3845
2940	CONTIG62	64579	65392	0.98	1.50E-01	transcription_start_site	-	63795	63795	-1190	17030	AN3845
489	CONTIG62	63302	63738	2.46	0.00E+00	transcription_start_site	+	64337	64337	-817	17031	AN3846
489	CONTIG62	63302	63738	2.46	0.00E+00	transcription_start_site	+	64649	64649	-1129	17032	AN3846
2214	CONTIG62	60677	61048	1.29	5.22E-02	transcription_start_site	+	64337	64337	-3474	17031	AN3846
2214	CONTIG62	60677	61048	1.29	5.22E-02	transcription_start_site	+	64649	64649	-3786	17032	AN3846
2940	CONTIG62	64579	65392	0.98	1.50E-01	transcription_start_site	+	64649	64649	336	17032	AN3846
2940	CONTIG62	64579	65392	0.98	1.50E-01	transcription_start_site	+	64337	64337	648	17031	AN3846
2940	CONTIG62	64579	65392	0.98	1.50E-01	transcription_start_site	+	68785	68785	-3799	17033	AN3848
2940	CONTIG62	64579	65392	0.98	1.50E-01	transcription_start_site	+	68966	68966	-3980	17034	AN3848
2350	CONTIG62	82576	82850	1.25	5.88E-02	transcription_start_site	-	79410	79410	-3303	17045	AN3853
2350	CONTIG62	82576	82850	1.25	5.88E-02	transcription_start_site	-	78968	78968	-3745	17046	AN3853
2350	CONTIG62	82576	82850	1.25	5.88E-02	transcription_start_site	-	83242	83242	529	17052	AN3855
1912	CONTIG62	110866	111275	1.4	3.08E-02	transcription_start_site	-	106966	106966	-4104	17068	AN3864
1912	CONTIG62	110866	111275	1.4	3.08E-02	transcription_start_site	-	106869	106869	-4201	17069	AN3864
2464	CONTIG62	105376	106100	1.21	4.42E-02	transcription_start_site	-	106869	106869	1131	17069	AN3864
2464	CONTIG62	105376	106100	1.21	4.42E-02	transcription_start_site	-	106966	106966	1228	17068	AN3864
2464	CONTIG62	105376	106100	1.21	4.42E-02	transcription_start_site	+	107244	107244	-1506	17070	AN3865
2464	CONTIG62	105376	106100	1.21	4.42E-02	transcription_start_site	+	107500	107500	-1762	17071	AN3865
2464	CONTIG62	105376	106100	1.21	4.42E-02	transcription_start_site	+	107667	107667	-1929	17072	AN3865
2464	CONTIG62	105376	106100	1.21	4.42E-02	transcription_start_site	+	107797	107797	-2059	17073	AN3865
2464	CONTIG62	105376	106100	1.21	4.42E-02	transcription_start_site	+	107931	107931	-2193	17074	AN3865
2464	CONTIG62	105376	106100	1.21	4.42E-02	transcription_start_site	+	108107	108107	-2369	17075	AN3865
2464	CONTIG62	105376	106100	1.21	4.42E-02	transcription_start_site	+	108515	108515	-2777	17076	AN3865
2464	CONTIG62	105376	106100	1.21	4.42E-02	transcription_start_site	+	109042	109042	-3304	17077	AN3865
1912	CONTIG62	110866	111275	1.4	3.08E-02	transcription_start_site	+	109986	109986	1084	17078	AN3866
2464	CONTIG62	105376	106100	1.21	4.42E-02	transcription_start_site	+	109986	109986	-4248	17078	AN3866
1912	CONTIG62	110866	111275	1.4	3.08E-02	transcription_start_site	-	111408	111408	337	17083	AN3867
1912	CONTIG62	110866	111275	1.4	3.08E-02	transcription_start_site	-	111821	111821	750	17082	AN3867
1912	CONTIG62	110866	111275	1.4	3.08E-02	transcription_start_site	-	112156	112156	1085	17081	AN3867
2036	CONTIG62	114601	115030	1.36	3.70E-02	transcription_start_site	-	112582	112582	-2233	17079	AN3867
2036	CONTIG62	114601	115030	1.36	3.70E-02	transcription_start_site	-	112311	112311	-2504	17080	AN3867
2036	CONTIG62	114601	115030	1.36	3.70E-02	transcription_start_site	-	112156	112156	-2659	17081	AN3867
2036	CONTIG62	114601	115030	1.36	3.70E-02	transcription_start_site	-	111821	111821	-2994	17082	AN3867
2036	CONTIG62	114601	115030	1.36	3.70E-02	transcription_start_site	-	111408	111408	-3407	17083	AN3867
1912	CONTIG62	110866	111275	1.4	3.08E-02	transcription_start_site	+	113216	113216	-2145	17084	AN3868
1912	CONTIG62	110866	111275	1.4	3.08E-02	transcription_start_site	+	113323	113323	-2252	17085	AN3868
2036	CONTIG62	114601	115030	1.36	3.70E-02	transcription_start_site	+	119640	119640	-4824	17092	AN3870
2036	CONTIG62	114601	115030	1.36	3.70E-02	transcription_start_site	+	120000	120000	-5184	17093	AN3870
437	CONTIG62	133371	133625	2.53	0.00E+00	transcription_start_site	-	130934	130934	-2564	17104	AN3874
437	CONTIG62	133371	133625	2.53	0.00E+00	transcription_start_site	-	130829	130829	-2669	17105	AN3874
437	CONTIG62	133371	133625	2.53	0.00E+00	transcription_start_site	-	132922	132922	-576	17106	AN3875
437	CONTIG62	133371	133625	2.53	0.00E+00	transcription_start_site	-	132407	132407	-1091	17107	AN3875
437	CONTIG62	133371	133625	2.53	0.00E+00	transcription_start_site	-	132130	132130	-1368	17108	AN3875
437	CONTIG62	133371	133625	2.53	0.00E+00	transcription_start_site	+	133911	133911	-413	17109	AN3876
437	CONTIG62	133371	133625	2.53	0.00E+00	transcription_start_site	+	134439	134439	-941	17110	AN3876
893	CONTIG62	151143	151567	2	0.00E+00	transcription_start_site	-	151427	151427	72	17116	AN3880
893	CONTIG62	151143	151567	2	0.00E+00	transcription_start_site	-	151191	151191	-164	17117	AN3880
893	CONTIG62	151143	151567	2	0.00E+00	transcription_start_site	-	150118	150118	-1237	17118	AN3880
893	CONTIG62	151143	151567	2	0.00E+00	transcription_start_site	+	152920	152920	-1565	17119	AN3881
893	CONTIG62	151143	151567	2	0.00E+00	transcription_start_site	+	155815	155815	-4460	17120	AN3881
1167	CONTIG62	160668	161088	1.78	5.20E-03	transcription_start_site	-	158389	158389	-2489	17121	AN3882
1167	CONTIG62	160668	161088	1.78	5.20E-03	transcription_start_site	-	156748	156748	-4130	17122	AN3882
1167	CONTIG62	160668	161088	1.78	5.20E-03	transcription_start_site	+	162554	162554	-1676	17123	AN3883
1167	CONTIG62	160668	161088	1.78	5.20E-03	transcription_start_site	+	162943	162943	-2065	17124	AN3883
1167	CONTIG62	160668	161088	1.78	5.20E-03	transcription_start_site	+	163454	163454	-2576	17125	AN3883
2810	CONTIG62	177532	177811	1.06	1.25E-01	transcription_start_site	-	177659	177659	-12	17148	AN3889

2810	CONTIG62	177532	177811	1.06	1.25E-01	transcription_start_site	-	177476	177476	-195	17149	AN3889
2810	CONTIG62	177532	177811	1.06	1.25E-01	transcription_start_site	-	176698	176698	-973	17150	AN3889
1762	CONTIG62	184142	184493	1.47	1.06E-02	transcription_start_site	-	181288	181288	-3029	17151	AN3890
1762	CONTIG62	184142	184493	1.47	1.06E-02	transcription_start_site	-	180639	180639	-3678	17152	AN3890
1762	CONTIG62	184142	184493	1.47	1.06E-02	transcription_start_site	-	180433	180433	-3884	17153	AN3890
1762	CONTIG62	184142	184493	1.47	1.06E-02	transcription_start_site	-	180041	180041	-4276	17154	AN3890
1762	CONTIG62	184142	184493	1.47	1.06E-02	transcription_start_site	-	184392	184392	74	17156	AN3891
1762	CONTIG62	184142	184493	1.47	1.06E-02	transcription_start_site	-	184534	184534	216	17155	AN3891
1762	CONTIG62	184142	184493	1.47	1.06E-02	transcription_start_site	-	184070	184070	-247	17157	AN3891
1762	CONTIG62	184142	184493	1.47	1.06E-02	transcription_start_site	-	183599	183599	-718	17158	AN3891
1762	CONTIG62	184142	184493	1.47	1.06E-02	transcription_start_site	+	187319	187319	-3001	17159	AN3892
1762	CONTIG62	184142	184493	1.47	1.06E-02	transcription_start_site	+	187419	187419	-3101	17160	AN3892
1762	CONTIG62	184142	184493	1.47	1.06E-02	transcription_start_site	+	187606	187606	-3288	17161	AN3892
549	CONTIG62	195237	195656	2.38	0.00E+00	transcription_start_site	+	197834	197834	-2387	17175	AN3895
549	CONTIG62	195237	195656	2.38	0.00E+00	transcription_start_site	+	198238	198238	-2791	17176	AN3895
549	CONTIG62	195237	195656	2.38	0.00E+00	transcription_start_site	+	198590	198590	-3143	17177	AN3895
549	CONTIG62	195237	195656	2.38	0.00E+00	transcription_start_site	+	199142	199142	-3695	17178	AN3895
10	CONTIG63	7291	7550	3.69	0.00E+00	transcription_start_site	-	2975	2975	-4445	17181	AN3897
10	CONTIG63	7291	7550	3.69	0.00E+00	transcription_start_site	-	2782	2782	-4638	17182	AN3897
2044	CONTIG63	6001	6666	1.36	1.07E-01	transcription_start_site	-	2975	2975	-3358	17181	AN3897
2044	CONTIG63	6001	6666	1.36	1.07E-01	transcription_start_site	-	2782	2782	-3551	17182	AN3897
10	CONTIG63	7291	7550	3.69	0.00E+00	transcription_start_site	-	4857	4857	-2563	17183	AN3898
10	CONTIG63	7291	7550	3.69	0.00E+00	transcription_start_site	-	4560	4560	-2860	17184	AN3898
10	CONTIG63	7291	7550	3.69	0.00E+00	transcription_start_site	-	4090	4090	-3330	17185	AN3898
10	CONTIG63	7291	7550	3.69	0.00E+00	transcription_start_site	-	3924	3924	-3496	17186	AN3898
1080	CONTIG63	9676	9950	1.85	2.62E-02	transcription_start_site	-	4857	4857	-4956	17183	AN3898
2044	CONTIG63	6001	6666	1.36	1.07E-01	transcription_start_site	-	4857	4857	-1476	17183	AN3898
2044	CONTIG63	6001	6666	1.36	1.07E-01	transcription_start_site	-	4560	4560	-1773	17184	AN3898
2044	CONTIG63	6001	6666	1.36	1.07E-01	transcription_start_site	-	4090	4090	-2243	17185	AN3898
2044	CONTIG63	6001	6666	1.36	1.07E-01	transcription_start_site	-	3924	3924	-2409	17186	AN3898
914	CONTIG63	13971	14241	1.99	1.57E-02	transcription_start_site	-	11691	11691	-2415	17187	AN3900
914	CONTIG63	13971	14241	1.99	1.57E-02	transcription_start_site	-	11483	11483	-2623	17188	AN3900
914	CONTIG63	13971	14241	1.99	1.57E-02	transcription_start_site	-	11324	11324	-2782	17189	AN3900
914	CONTIG63	13971	14241	1.99	1.57E-02	transcription_start_site	-	10446	10446	-3660	17190	AN3900
914	CONTIG63	13971	14241	1.99	1.57E-02	transcription_start_site	-	10305	10305	-3801	17191	AN3900
1080	CONTIG63	9676	9950	1.85	2.62E-02	transcription_start_site	-	10305	10305	492	17191	AN3900
1080	CONTIG63	9676	9950	1.85	2.62E-02	transcription_start_site	-	10446	10446	633	17190	AN3900
914	CONTIG63	13971	14241	1.99	1.57E-02	transcription_start_site	-	13896	13896	-210	17192	AN3901
914	CONTIG63	13971	14241	1.99	1.57E-02	transcription_start_site	-	13729	13729	-377	17193	AN3901
914	CONTIG63	13971	14241	1.99	1.57E-02	transcription_start_site	-	13357	13357	-749	17194	AN3901
1081	CONTIG63	33527	33801	1.85	2.62E-02	transcription_start_site	-	30643	30643	-3021	17201	AN3905
1081	CONTIG63	33527	33801	1.85	2.62E-02	transcription_start_site	-	29959	29959	-3705	17202	AN3905
1081	CONTIG63	33527	33801	1.85	2.62E-02	transcription_start_site	-	29545	29545	-4119	17203	AN3905
1081	CONTIG63	33527	33801	1.85	2.62E-02	transcription_start_site	-	29300	29300	-4364	17204	AN3905
1081	CONTIG63	33527	33801	1.85	2.62E-02	transcription_start_site	+	34606	34606	-942	17205	AN3906
1081	CONTIG63	33527	33801	1.85	2.62E-02	transcription_start_site	+	37996	37996	-4332	17206	AN3906
1081	CONTIG63	33527	33801	1.85	2.62E-02	transcription_start_site	+	38115	38115	-4451	17207	AN3906
2654	CONTIG64	13729	14153	1.14	6.84E-02	transcription_start_site	+	15666	15666	-1725	17213	AN3910
2654	CONTIG64	13729	14153	1.14	6.84E-02	transcription_start_site	+	17291	17291	-3350	17214	AN3910
1151	CONTIG64	18919	19193	1.79	3.03E-03	transcription_start_site	+	21241	21241	-2185	17215	AN3911
1151	CONTIG64	18919	19193	1.79	3.03E-03	transcription_start_site	+	21523	21523	-2467	17216	AN3911
2987	CONTIG64	69165	69729	0.93	1.66E-01	transcription_start_site	+	68498	68498	949	17250	AN3924
2987	CONTIG64	69165	69729	0.93	1.66E-01	transcription_start_site	+	68217	68217	1230	17249	AN3924
2987	CONTIG64	69165	69729	0.93	1.66E-01	transcription_start_site	+	72300	72300	-2853	17251	AN3925
2028	CONTIG64	73435	74469	1.36	1.44E-02	transcription_start_site	-	74650	74650	698	17254	AN3926
2028	CONTIG64	73435	74469	1.36	1.44E-02	transcription_start_site	-	74861	74861	909	17253	AN3926
2556	CONTIG64	74870	75129	1.18	5.88E-02	transcription_start_site	-	74861	74861	-138	17253	AN3926

2556	CONTIG64	74870	75129	1.18	5.88E-02	transcription_start_site	-	74650	74650	-349	17254	AN3926
2556	CONTIG64	74870	75129	1.18	5.88E-02	transcription_start_site	-	75489	75489	489	17252	AN3926
2982	CONTIG64	75380	76719	0.93	9.40E-02	transcription_start_site	-	75489	75489	-560	17252	AN3926
2982	CONTIG64	75380	76719	0.93	9.40E-02	transcription_start_site	-	74861	74861	-1188	17253	AN3926
2982	CONTIG64	75380	76719	0.93	9.40E-02	transcription_start_site	-	74650	74650	-1399	17254	AN3926
2028	CONTIG64	73435	74469	1.36	1.44E-02	transcription_start_site	+	75686	75686	-1734	17255	AN3927
2556	CONTIG64	74870	75129	1.18	5.88E-02	transcription_start_site	+	75686	75686	-686	17255	AN3927
2982	CONTIG64	75380	76719	0.93	9.40E-02	transcription_start_site	+	75686	75686	363	17255	AN3927
1788	CONTIG64	84676	84960	1.46	1.57E-02	transcription_start_site	+	84730	84730	88	17258	AN3930
486	CONTIG64	87241	87650	2.46	0.00E+00	transcription_start_site	+	87779	87779	-333	17259	AN3931
486	CONTIG64	87241	87650	2.46	0.00E+00	transcription_start_site	+	87838	87838	-392	17260	AN3931
486	CONTIG64	87241	87650	2.46	0.00E+00	transcription_start_site	+	88402	88402	-956	17261	AN3931
1788	CONTIG64	84676	84960	1.46	1.57E-02	transcription_start_site	+	87779	87779	-2961	17259	AN3931
1788	CONTIG64	84676	84960	1.46	1.57E-02	transcription_start_site	+	87838	87838	-3020	17260	AN3931
1788	CONTIG64	84676	84960	1.46	1.57E-02	transcription_start_site	+	88402	88402	-3584	17261	AN3931
486	CONTIG64	87241	87650	2.46	0.00E+00	transcription_start_site	+	89446	89446	-2000	17262	AN3932
486	CONTIG64	87241	87650	2.46	0.00E+00	transcription_start_site	+	89733	89733	-2287	17263	AN3932
486	CONTIG64	87241	87650	2.46	0.00E+00	transcription_start_site	+	89901	89901	-2455	17264	AN3932
486	CONTIG64	87241	87650	2.46	0.00E+00	transcription_start_site	+	90114	90114	-2668	17265	AN3932
486	CONTIG64	87241	87650	2.46	0.00E+00	transcription_start_site	+	90212	90212	-2766	17266	AN3932
486	CONTIG64	87241	87650	2.46	0.00E+00	transcription_start_site	+	90381	90381	-2935	17267	AN3932
1788	CONTIG64	84676	84960	1.46	1.57E-02	transcription_start_site	+	89446	89446	-4628	17262	AN3932
1788	CONTIG64	84676	84960	1.46	1.57E-02	transcription_start_site	+	89733	89733	-4915	17263	AN3932
1788	CONTIG64	84676	84960	1.46	1.57E-02	transcription_start_site	+	89901	89901	-5083	17264	AN3932
1514	CONTIG64	98716	98990	1.57	8.81E-03	transcription_start_site	-	94879	94879	-3974	17268	AN3933
1514	CONTIG64	98716	98990	1.57	8.81E-03	transcription_start_site	-	94328	94328	-4525	17269	AN3933
1850	CONTIG64	98101	98398	1.43	1.84E-02	transcription_start_site	-	94879	94879	-3370	17268	AN3933
1850	CONTIG64	98101	98398	1.43	1.84E-02	transcription_start_site	-	94328	94328	-3921	17269	AN3933
2466	CONTIG64	95926	96895	1.21	5.22E-02	transcription_start_site	-	94879	94879	-1531	17268	AN3933
2466	CONTIG64	95926	96895	1.21	5.22E-02	transcription_start_site	-	94328	94328	-2082	17269	AN3933
2466	CONTIG64	95926	96895	1.21	5.22E-02	transcription_start_site	-	91823	91823	-4587	17270	AN3933
2466	CONTIG64	95926	96895	1.21	5.22E-02	transcription_start_site	+	96348	96348	62	17272	AN3934
2466	CONTIG64	95926	96895	1.21	5.22E-02	transcription_start_site	+	96565	96565	-154	17273	AN3934
2466	CONTIG64	95926	96895	1.21	5.22E-02	transcription_start_site	+	96197	96197	213	17271	AN3934
2466	CONTIG64	95926	96895	1.21	5.22E-02	transcription_start_site	+	96682	96682	-271	17274	AN3934
2466	CONTIG64	95926	96895	1.21	5.22E-02	transcription_start_site	+	96802	96802	-391	17275	AN3934
1514	CONTIG64	98716	98990	1.57	8.81E-03	transcription_start_site	+	98846	98846	7	17278	AN3935
1514	CONTIG64	98716	98990	1.57	8.81E-03	transcription_start_site	+	98667	98667	186	17277	AN3935
1514	CONTIG64	98716	98990	1.57	8.81E-03	transcription_start_site	+	98529	98529	324	17276	AN3935
1850	CONTIG64	98101	98398	1.43	1.84E-02	transcription_start_site	+	98529	98529	-279	17276	AN3935
1850	CONTIG64	98101	98398	1.43	1.84E-02	transcription_start_site	+	98667	98667	-417	17277	AN3935
1850	CONTIG64	98101	98398	1.43	1.84E-02	transcription_start_site	+	98846	98846	-596	17278	AN3935
2466	CONTIG64	95926	96895	1.21	5.22E-02	transcription_start_site	+	98529	98529	-2118	17276	AN3935
2466	CONTIG64	95926	96895	1.21	5.22E-02	transcription_start_site	+	98667	98667	-2256	17277	AN3935
2466	CONTIG64	95926	96895	1.21	5.22E-02	transcription_start_site	+	98846	98846	-2435	17278	AN3935
1514	CONTIG64	98716	98990	1.57	8.81E-03	transcription_start_site	+	100708	100708	-1855	17279	AN3936
1514	CONTIG64	98716	98990	1.57	8.81E-03	transcription_start_site	+	100874	100874	-2021	17280	AN3936
1850	CONTIG64	98101	98398	1.43	1.84E-02	transcription_start_site	+	100708	100708	-2458	17279	AN3936
1850	CONTIG64	98101	98398	1.43	1.84E-02	transcription_start_site	+	100874	100874	-2624	17280	AN3936
2466	CONTIG64	95926	96895	1.21	5.22E-02	transcription_start_site	+	100708	100708	-4297	17279	AN3936
2466	CONTIG64	95926	96895	1.21	5.22E-02	transcription_start_site	+	100874	100874	-4463	17280	AN3936
896	CONTIG64	103126	103405	2	9.51E-04	transcription_start_site	-	104064	104064	798	17282	AN3937
896	CONTIG64	103126	103405	2	9.51E-04	transcription_start_site	-	104125	104125	859	17281	AN3937
896	CONTIG64	103126	103405	2	9.51E-04	transcription_start_site	+	104544	104544	-1278	17283	AN3938
896	CONTIG64	103126	103405	2	9.51E-04	transcription_start_site	+	105944	105944	-2678	17284	AN3938
2207	CONTIG64	121213	121712	1.29	1.86E-02	transcription_start_site	-	117761	117761	-3701	17289	AN3940
2207	CONTIG64	121213	121712	1.29	1.86E-02	transcription_start_site	-	117519	117519	-3943	17290	AN3940

2958	CONTIG64	117537	117826	0.96	1.44E-01	transcription_start_site	-	117761	117761	79	17289	AN3940
2958	CONTIG64	117537	117826	0.96	1.44E-01	transcription_start_site	-	117519	117519	-162	17290	AN3940
2207	CONTIG64	121213	121712	1.29	1.86E-02	transcription_start_site	-	120620	120620	-842	17291	AN3941
2207	CONTIG64	121213	121712	1.29	1.86E-02	transcription_start_site	-	120286	120286	-1176	17292	AN3941
2207	CONTIG64	121213	121712	1.29	1.86E-02	transcription_start_site	-	120163	120163	-1299	17293	AN3941
2207	CONTIG64	121213	121712	1.29	1.86E-02	transcription_start_site	-	119873	119873	-1589	17294	AN3941
2898	CONTIG64	140944	141213	1	1.25E-01	transcription_start_site	+	140804	140804	274	17304	AN3945
2839	CONTIG64	151514	152018	1.04	8.36E-02	transcription_start_site	-	148460	148460	-3306	17305	AN3946
2839	CONTIG64	151514	152018	1.04	8.36E-02	transcription_start_site	-	147360	147360	-4406	17306	AN3946
2839	CONTIG64	151514	152018	1.04	8.36E-02	transcription_start_site	-	146866	146866	-4900	17307	AN3946
2839	CONTIG64	151514	152018	1.04	8.36E-02	transcription_start_site	+	153729	153729	-1963	17313	AN3948
2839	CONTIG64	151514	152018	1.04	8.36E-02	transcription_start_site	+	154204	154204	-2438	17314	AN3948
2030	CONTIG64	176343	176827	1.36	2.62E-02	transcription_start_site	-	173558	173558	-3027	17333	AN3952
2030	CONTIG64	176343	176827	1.36	2.62E-02	transcription_start_site	-	172862	172862	-3723	17334	AN3952
2030	CONTIG64	176343	176827	1.36	2.62E-02	transcription_start_site	-	172747	172747	-3838	17335	AN3952
2030	CONTIG64	176343	176827	1.36	2.62E-02	transcription_start_site	-	171780	171780	-4805	17336	AN3952
2030	CONTIG64	176343	176827	1.36	2.62E-02	transcription_start_site	-	171623	171623	-4962	17337	AN3952
2030	CONTIG64	176343	176827	1.36	2.62E-02	transcription_start_site	-	171483	171483	-5102	17338	AN3952
2899	CONTIG64	177768	178327	1	1.25E-01	transcription_start_site	-	173558	173558	-4489	17333	AN3952
2899	CONTIG64	177768	178327	1	1.25E-01	transcription_start_site	-	172862	172862	-5185	17334	AN3952
2030	CONTIG64	176343	176827	1.36	2.62E-02	transcription_start_site	-	176865	176865	280	17347	AN3953
2208	CONTIG64	179028	180142	1.29	2.18E-02	transcription_start_site	-	176865	176865	-2720	17347	AN3953
2899	CONTIG64	177768	178327	1	1.25E-01	transcription_start_site	-	176865	176865	-1182	17347	AN3953
2030	CONTIG64	176343	176827	1.36	2.62E-02	transcription_start_site	+	178271	178271	-1686	17348	AN3954
2030	CONTIG64	176343	176827	1.36	2.62E-02	transcription_start_site	+	178621	178621	-2036	17349	AN3954
2030	CONTIG64	176343	176827	1.36	2.62E-02	transcription_start_site	+	178825	178825	-2240	17350	AN3954
2030	CONTIG64	176343	176827	1.36	2.62E-02	transcription_start_site	+	179322	179322	-2737	17351	AN3954
2030	CONTIG64	176343	176827	1.36	2.62E-02	transcription_start_site	+	179802	179802	-3217	17352	AN3954
2208	CONTIG64	179028	180142	1.29	2.18E-02	transcription_start_site	+	179802	179802	-217	17352	AN3954
2208	CONTIG64	179028	180142	1.29	2.18E-02	transcription_start_site	+	179322	179322	263	17351	AN3954
2208	CONTIG64	179028	180142	1.29	2.18E-02	transcription_start_site	+	178825	178825	760	17350	AN3954
2208	CONTIG64	179028	180142	1.29	2.18E-02	transcription_start_site	+	178621	178621	964	17349	AN3954
2208	CONTIG64	179028	180142	1.29	2.18E-02	transcription_start_site	+	178271	178271	1314	17348	AN3954
2899	CONTIG64	177768	178327	1	1.25E-01	transcription_start_site	+	178271	178271	-223	17348	AN3954
2899	CONTIG64	177768	178327	1	1.25E-01	transcription_start_site	+	178621	178621	-573	17349	AN3954
2899	CONTIG64	177768	178327	1	1.25E-01	transcription_start_site	+	178825	178825	-777	17350	AN3954
2899	CONTIG64	177768	178327	1	1.25E-01	transcription_start_site	+	179322	179322	-1274	17351	AN3954
2899	CONTIG64	177768	178327	1	1.25E-01	transcription_start_site	+	179802	179802	-1754	17352	AN3954
2030	CONTIG64	176343	176827	1.36	2.62E-02	transcription_start_site	+	180998	180998	-4413	17353	AN3955
2030	CONTIG64	176343	176827	1.36	2.62E-02	transcription_start_site	+	181248	181248	-4663	17354	AN3955
2030	CONTIG64	176343	176827	1.36	2.62E-02	transcription_start_site	+	181806	181806	-5221	17355	AN3955
2208	CONTIG64	179028	180142	1.29	2.18E-02	transcription_start_site	+	180998	180998	-1413	17353	AN3955
2208	CONTIG64	179028	180142	1.29	2.18E-02	transcription_start_site	+	181248	181248	-1663	17354	AN3955
2208	CONTIG64	179028	180142	1.29	2.18E-02	transcription_start_site	+	181806	181806	-2221	17355	AN3955
2899	CONTIG64	177768	178327	1	1.25E-01	transcription_start_site	+	180998	180998	-2950	17353	AN3955
2899	CONTIG64	177768	178327	1	1.25E-01	transcription_start_site	+	181248	181248	-3200	17354	AN3955
2899	CONTIG64	177768	178327	1	1.25E-01	transcription_start_site	+	181806	181806	-3758	17355	AN3955
2342	CONTIG64	183244	184418	1.25	2.45E-02	transcription_start_site	-	184008	184008	177	17357	AN3956
2342	CONTIG64	183244	184418	1.25	2.45E-02	transcription_start_site	-	184642	184642	811	17356	AN3956
2342	CONTIG64	183244	184418	1.25	2.45E-02	transcription_start_site	-	185210	185210	1379	17362	AN3957
2342	CONTIG64	183244	184418	1.25	2.45E-02	transcription_start_site	+	187671	187671	-3840	17363	AN3958
2342	CONTIG64	183244	184418	1.25	2.45E-02	transcription_start_site	+	188001	188001	-4170	17364	AN3958
2069	CONTIG65	1351	1645	1.34	3.08E-02	transcription_start_site	+	6303	6303	-4805	17375	AN3963
1971	CONTIG65	14868	15142	1.38	2.62E-02	transcription_start_site	+	15268	15268	-263	17391	AN3967
1971	CONTIG65	14868	15142	1.38	2.62E-02	transcription_start_site	+	14529	14529	476	17390	AN3967
1971	CONTIG65	14868	15142	1.38	2.62E-02	transcription_start_site	+	14303	14303	702	17389	AN3967
1228	CONTIG65	18228	18585	1.74	4.61E-03	transcription_start_site	+	18868	18868	-461	17392	AN3968

1971	CONTIG65	14868	15142	1.38	2.62E-02	transcription_start_site	+	18868	18868	-3863	17392	AN3968
1228	CONTIG65	18228	18585	1.74	4.61E-03	transcription_start_site	+	22792	22792	-4385	17393	AN3969
1228	CONTIG65	18228	18585	1.74	4.61E-03	transcription_start_site	+	23075	23075	-4668	17394	AN3969
2978	CONTIG65	20268	20762	0.94	1.66E-01	transcription_start_site	+	22792	22792	-2277	17393	AN3969
2978	CONTIG65	20268	20762	0.94	1.66E-01	transcription_start_site	+	23075	23075	-2560	17394	AN3969
2978	CONTIG65	20268	20762	0.94	1.66E-01	transcription_start_site	+	23617	23617	-3102	17395	AN3969
2820	CONTIG65	29481	30289	1.05	5.82E-02	transcription_start_site	-	25624	25624	-4261	17396	AN3970
2936	CONTIG65	49365	49634	0.98	1.44E-01	transcription_start_site	-	48285	48285	-1214	17406	AN3977
2936	CONTIG65	49365	49634	0.98	1.44E-01	transcription_start_site	-	48068	48068	-1431	17407	AN3977
2936	CONTIG65	49365	49634	0.98	1.44E-01	transcription_start_site	-	47664	47664	-1835	17408	AN3977
2521	CONTIG65	53637	54056	1.19	5.88E-02	transcription_start_site	+	56230	56230	-2383	17409	AN3979
2885	CONTIG65	78085	78354	1.01	1.25E-01	transcription_start_site	-	73886	73886	-4333	17423	AN3985
2885	CONTIG65	78085	78354	1.01	1.25E-01	transcription_start_site	-	73528	73528	-4691	17424	AN3985
2885	CONTIG65	78085	78354	1.01	1.25E-01	transcription_start_site	-	76499	76499	-1720	17426	AN3986
2885	CONTIG65	78085	78354	1.01	1.25E-01	transcription_start_site	-	76400	76400	-1819	17427	AN3986
2885	CONTIG65	78085	78354	1.01	1.25E-01	transcription_start_site	-	76273	76273	-1946	17428	AN3986
2885	CONTIG65	78085	78354	1.01	1.25E-01	transcription_start_site	-	75891	75891	-2328	17429	AN3986
2885	CONTIG65	78085	78354	1.01	1.25E-01	transcription_start_site	-	75304	75304	-2915	17430	AN3986
2885	CONTIG65	78085	78354	1.01	1.25E-01	transcription_start_site	-	75213	75213	-3006	17431	AN3986
2979	CONTIG65	101630	101919	0.94	1.66E-01	transcription_start_site	-	97184	97184	-4590	17443	AN3992
2703	CONTIG65	106139	106403	1.12	8.01E-02	transcription_start_site	-	101164	101164	-5107	17449	AN3994
2979	CONTIG65	101630	101919	0.94	1.66E-01	transcription_start_site	-	101164	101164	-610	17449	AN3994
2979	CONTIG65	101630	101919	0.94	1.66E-01	transcription_start_site	-	101007	101007	-767	17450	AN3994
2979	CONTIG65	101630	101919	0.94	1.66E-01	transcription_start_site	-	100726	100726	-1048	17451	AN3994
2979	CONTIG65	101630	101919	0.94	1.66E-01	transcription_start_site	+	101600	101600	174	17452	AN3995
2979	CONTIG65	101630	101919	0.94	1.66E-01	transcription_start_site	+	102959	102959	-1184	17453	AN3995
2703	CONTIG65	106139	106403	1.12	8.01E-02	transcription_start_site	+	106073	106073	198	17454	AN3996
2979	CONTIG65	101630	101919	0.94	1.66E-01	transcription_start_site	+	106073	106073	-4298	17454	AN3996
2278	CONTIG65	112295	112555	1.27	4.36E-02	transcription_start_site	-	110208	110208	-2217	17455	AN3997
2278	CONTIG65	112295	112555	1.27	4.36E-02	transcription_start_site	-	109954	109954	-2471	17456	AN3997
2754	CONTIG65	111619	112045	1.09	9.24E-02	transcription_start_site	-	110208	110208	-1624	17455	AN3997
2754	CONTIG65	111619	112045	1.09	9.24E-02	transcription_start_site	-	109954	109954	-1878	17456	AN3997
2278	CONTIG65	112295	112555	1.27	4.36E-02	transcription_start_site	-	112131	112131	-294	17457	AN3998
2754	CONTIG65	111619	112045	1.09	9.24E-02	transcription_start_site	-	112131	112131	299	17457	AN3998
2278	CONTIG65	112295	112555	1.27	4.36E-02	transcription_start_site	+	114940	114940	-2515	17458	AN3999
2754	CONTIG65	111619	112045	1.09	9.24E-02	transcription_start_site	+	114940	114940	-3108	17458	AN3999
1392	CONTIG65	121067	121491	1.63	7.85E-03	transcription_start_site	+	121692	121692	-413	17461	AN4000
2398	CONTIG65	119552	119841	1.23	5.22E-02	transcription_start_site	+	119581	119581	115	17460	AN4000
2398	CONTIG65	119552	119841	1.23	5.22E-02	transcription_start_site	+	121692	121692	-1995	17461	AN4000
1972	CONTIG65	123095	123519	1.38	2.62E-02	transcription_start_site	-	124266	124266	959	17462	AN4001
1972	CONTIG65	123095	123519	1.38	2.62E-02	transcription_start_site	+	127254	127254	-3947	17470	AN4003
1972	CONTIG65	123095	123519	1.38	2.62E-02	transcription_start_site	+	127539	127539	-4232	17471	AN4003
1968	CONTIG65	154583	155467	1.38	1.01E-02	transcription_start_site	+	154229	154229	796	17500	AN4012
1968	CONTIG65	154583	155467	1.38	1.01E-02	transcription_start_site	+	154129	154129	896	17499	AN4012
1968	CONTIG65	154583	155467	1.38	1.01E-02	transcription_start_site	+	155065	155065	-40	17501	AN4013
1968	CONTIG65	154583	155467	1.38	1.01E-02	transcription_start_site	+	155189	155189	-164	17502	AN4013
1968	CONTIG65	154583	155467	1.38	1.01E-02	transcription_start_site	+	155296	155296	-271	17503	AN4013
1968	CONTIG65	154583	155467	1.38	1.01E-02	transcription_start_site	+	156453	156453	-1428	17504	AN4013
1968	CONTIG65	154583	155467	1.38	1.01E-02	transcription_start_site	+	156616	156616	-1591	17505	AN4013
1968	CONTIG65	154583	155467	1.38	1.01E-02	transcription_start_site	+	156711	156711	-1686	17506	AN4013
1968	CONTIG65	154583	155467	1.38	1.01E-02	transcription_start_site	+	156887	156887	-1862	17507	AN4013
1739	CONTIG65	178054	178943	1.48	7.16E-03	transcription_start_site	-	177977	177977	-521	17537	AN4021
1739	CONTIG65	178054	178943	1.48	7.16E-03	transcription_start_site	-	177756	177756	-742	17538	AN4021
1739	CONTIG65	178054	178943	1.48	7.16E-03	transcription_start_site	-	177321	177321	-1177	17539	AN4021
2937	CONTIG65	182938	183509	0.98	1.44E-01	transcription_start_site	-	177977	177977	-5246	17537	AN4021
2823	CONTIG65	185043	185677	1.05	9.26E-02	transcription_start_site	-	180827	180827	-4533	17540	AN4022
2937	CONTIG65	182938	183509	0.98	1.44E-01	transcription_start_site	-	180827	180827	-2396	17540	AN4022

2600	CONTIG65	187058	187637	1.16	6.84E-02	transcription_start_site	-	183111	183111	-4236	17541 AN4023
2600	CONTIG65	187058	187637	1.16	6.84E-02	transcription_start_site	-	182494	182494	-4853	17542 AN4023
2823	CONTIG65	185043	185677	1.05	9.26E-02	transcription_start_site	-	183111	183111	-2249	17541 AN4023
2823	CONTIG65	185043	185677	1.05	9.26E-02	transcription_start_site	-	182494	182494	-2866	17542 AN4023
2937	CONTIG65	182938	183509	0.98	1.44E-01	transcription_start_site	-	183111	183111	-112	17541 AN4023
2937	CONTIG65	182938	183509	0.98	1.44E-01	transcription_start_site	-	182494	182494	-729	17542 AN4023
1739	CONTIG65	178054	178943	1.48	7.16E-03	transcription_start_site	+	183710	183710	-5211	17543 AN4024
1739	CONTIG65	178054	178943	1.48	7.16E-03	transcription_start_site	+	183912	183912	-5413	17544 AN4024
2823	CONTIG65	185043	185677	1.05	9.26E-02	transcription_start_site	+	184822	184822	538	17546 AN4024
2823	CONTIG65	185043	185677	1.05	9.26E-02	transcription_start_site	+	184181	184181	1179	17545 AN4024
2937	CONTIG65	182938	183509	0.98	1.44E-01	transcription_start_site	+	183710	183710	-486	17543 AN4024
2937	CONTIG65	182938	183509	0.98	1.44E-01	transcription_start_site	+	183912	183912	-688	17544 AN4024
2937	CONTIG65	182938	183509	0.98	1.44E-01	transcription_start_site	+	184181	184181	-957	17545 AN4024
2937	CONTIG65	182938	183509	0.98	1.44E-01	transcription_start_site	+	184822	184822	-1598	17546 AN4024
282	CONTIG65	188183	188602	2.79	0.00E+00	transcription_start_site	-	187535	187535	-857	17547 AN4025
282	CONTIG65	188183	188602	2.79	0.00E+00	transcription_start_site	-	187332	187332	-1060	17548 AN4025
282	CONTIG65	188183	188602	2.79	0.00E+00	transcription_start_site	-	186327	186327	-2065	17549 AN4025
2600	CONTIG65	187058	187637	1.16	6.84E-02	transcription_start_site	-	187332	187332	-15	17548 AN4025
2600	CONTIG65	187058	187637	1.16	6.84E-02	transcription_start_site	-	187535	187535	187	17547 AN4025
2600	CONTIG65	187058	187637	1.16	6.84E-02	transcription_start_site	-	186327	186327	-1020	17549 AN4025
2823	CONTIG65	185043	185677	1.05	9.26E-02	transcription_start_site	-	186327	186327	967	17549 AN4025
282	CONTIG65	188183	188602	2.79	0.00E+00	transcription_start_site	+	189460	189460	-1067	17550 AN4026
282	CONTIG65	188183	188602	2.79	0.00E+00	transcription_start_site	+	189746	189746	-1353	17551 AN4026
282	CONTIG65	188183	188602	2.79	0.00E+00	transcription_start_site	+	190924	190924	-2531	17552 AN4026
282	CONTIG65	188183	188602	2.79	0.00E+00	transcription_start_site	+	191082	191082	-2689	17553 AN4026
282	CONTIG65	188183	188602	2.79	0.00E+00	transcription_start_site	+	191455	191455	-3062	17554 AN4026
2600	CONTIG65	187058	187637	1.16	6.84E-02	transcription_start_site	+	189460	189460	-2112	17550 AN4026
2600	CONTIG65	187058	187637	1.16	6.84E-02	transcription_start_site	+	189746	189746	-2398	17551 AN4026
2600	CONTIG65	187058	187637	1.16	6.84E-02	transcription_start_site	+	190924	190924	-3576	17552 AN4026
2600	CONTIG65	187058	187637	1.16	6.84E-02	transcription_start_site	+	191082	191082	-3734	17553 AN4026
2600	CONTIG65	187058	187637	1.16	6.84E-02	transcription_start_site	+	191455	191455	-4107	17554 AN4026
2823	CONTIG65	185043	185677	1.05	9.26E-02	transcription_start_site	+	189460	189460	-4100	17550 AN4026
2823	CONTIG65	185043	185677	1.05	9.26E-02	transcription_start_site	+	189746	189746	-4386	17551 AN4026
2522	CONTIG65	201680	201965	1.19	5.88E-02	transcription_start_site	-	199470	199470	-2352	17557 AN4028
2522	CONTIG65	201680	201965	1.19	5.88E-02	transcription_start_site	-	197288	197288	-4534	17558 AN4028
2522	CONTIG65	201680	201965	1.19	5.88E-02	transcription_start_site	-	200721	200721	-1101	17559 AN4029
2522	CONTIG65	201680	201965	1.19	5.88E-02	transcription_start_site	-	200616	200616	-1206	17560 AN4029
2522	CONTIG65	201680	201965	1.19	5.88E-02	transcription_start_site	-	200373	200373	-1449	17561 AN4029
2176	CONTIG65	213994	214403	1.3	2.18E-02	transcription_start_site	-	211703	211703	-2495	17565 AN4032
2176	CONTIG65	213994	214403	1.3	2.18E-02	transcription_start_site	-	209248	209248	-4950	17566 AN4032
2176	CONTIG65	213994	214403	1.3	2.18E-02	transcription_start_site	+	215129	215129	-930	17571 AN4034
2176	CONTIG65	213994	214403	1.3	2.18E-02	transcription_start_site	+	215529	215529	-1330	17572 AN4034
2176	CONTIG65	213994	214403	1.3	2.18E-02	transcription_start_site	+	215692	215692	-1493	17573 AN4034
2176	CONTIG65	213994	214403	1.3	2.18E-02	transcription_start_site	+	215904	215904	-1705	17574 AN4034
1633	CONTIG65	230935	231219	1.52	1.30E-02	transcription_start_site	+	230351	230351	726	17588 AN4038
982	CONTIG65	236570	236920	1.92	1.54E-03	transcription_start_site	-	231746	231746	-4999	17589 AN4039
1633	CONTIG65	230935	231219	1.52	1.30E-02	transcription_start_site	-	231372	231372	295	17590 AN4039
1633	CONTIG65	230935	231219	1.52	1.30E-02	transcription_start_site	-	231746	231746	669	17589 AN4039
982	CONTIG65	236570	236920	1.92	1.54E-03	transcription_start_site	-	233716	233716	-3029	17591 AN4040
982	CONTIG65	236570	236920	1.92	1.54E-03	transcription_start_site	-	232449	232449	-4296	17592 AN4040
982	CONTIG65	236570	236920	1.92	1.54E-03	transcription_start_site	-	235292	235292	-1453	17593 AN4041
982	CONTIG65	236570	236920	1.92	1.54E-03	transcription_start_site	+	237261	237261	-516	17594 AN4042
982	CONTIG65	236570	236920	1.92	1.54E-03	transcription_start_site	+	237471	237471	-726	17595 AN4042
982	CONTIG65	236570	236920	1.92	1.54E-03	transcription_start_site	+	237683	237683	-938	17596 AN4042
982	CONTIG65	236570	236920	1.92	1.54E-03	transcription_start_site	+	238986	238986	-2241	17597 AN4042
1489	CONTIG65	252617	254081	1.59	8.81E-03	transcription_start_site	-	252096	252096	-1253	17601 AN4045
2938	CONTIG65	255607	255876	0.98	1.44E-01	transcription_start_site	-	252096	252096	-3645	17601 AN4045

1489	CONTIG65	252617	254081	1.59	8.81E-03	transcription_start_site	+	253038	253038	311	17603 AN4046
1489	CONTIG65	252617	254081	1.59	8.81E-03	transcription_start_site	+	252944	252944	405	17602 AN4046
2399	CONTIG65	249682	249951	1.23	5.22E-02	transcription_start_site	+	252944	252944	-3127	17602 AN4046
2399	CONTIG65	249682	249951	1.23	5.22E-02	transcription_start_site	+	253038	253038	-3221	17603 AN4046
1489	CONTIG65	252617	254081	1.59	8.81E-03	transcription_start_site	-	255022	255022	1673	17605 AN4047
1738	CONTIG65	258392	259181	1.48	3.83E-03	transcription_start_site	-	256219	256219	-2567	17604 AN4047
1738	CONTIG65	258392	259181	1.48	3.83E-03	transcription_start_site	-	255022	255022	-3764	17605 AN4047
2523	CONTIG65	260637	261051	1.19	5.88E-02	transcription_start_site	-	256219	256219	-4625	17604 AN4047
2876	CONTIG65	259652	260601	1.01	8.00E-02	transcription_start_site	-	256219	256219	-3907	17604 AN4047
2876	CONTIG65	259652	260601	1.01	8.00E-02	transcription_start_site	-	255022	255022	-5104	17605 AN4047
2938	CONTIG65	255607	255876	0.98	1.44E-01	transcription_start_site	-	256219	256219	477	17604 AN4047
2938	CONTIG65	255607	255876	0.98	1.44E-01	transcription_start_site	-	255022	255022	-719	17605 AN4047
1489	CONTIG65	252617	254081	1.59	8.81E-03	transcription_start_site	+	256530	256530	-3181	17606 AN4048
2938	CONTIG65	255607	255876	0.98	1.44E-01	transcription_start_site	+	256530	256530	-788	17606 AN4048
1738	CONTIG65	258392	259181	1.48	3.83E-03	transcription_start_site	-	258825	258825	38	17607 AN4049
2523	CONTIG65	260637	261051	1.19	5.88E-02	transcription_start_site	-	258825	258825	-2019	17607 AN4049
2876	CONTIG65	259652	260601	1.01	8.00E-02	transcription_start_site	-	258825	258825	-1301	17607 AN4049
3009	CONTIG65	261692	261968	0.91	1.93E-01	transcription_start_site	-	258825	258825	-3005	17607 AN4049
3009	CONTIG65	261692	261968	0.91	1.93E-01	transcription_start_site	-	262192	262192	362	17611 AN4050
3009	CONTIG65	261692	261968	0.91	1.93E-01	transcription_start_site	-	262693	262693	863	17610 AN4050
2877	CONTIG65	270907	271266	1.01	1.07E-01	transcription_start_site	-	271294	271294	207	17625 AN4053
2877	CONTIG65	270907	271266	1.01	1.07E-01	transcription_start_site	-	271535	271535	448	17624 AN4053
2877	CONTIG65	270907	271266	1.01	1.07E-01	transcription_start_site	-	272053	272053	966	17623 AN4053
2877	CONTIG65	270907	271266	1.01	1.07E-01	transcription_start_site	+	274503	274503	-3416	17626 AN4054
2877	CONTIG65	270907	271266	1.01	1.07E-01	transcription_start_site	+	274633	274633	-3546	17627 AN4054
2877	CONTIG65	270907	271266	1.01	1.07E-01	transcription_start_site	+	274769	274769	-3682	17628 AN4054
2877	CONTIG65	270907	271266	1.01	1.07E-01	transcription_start_site	+	275252	275252	-4165	17629 AN4054
2872	CONTIG66	11552	12372	1.02	1.28E-01	transcription_start_site	-	8405	8405	-3557	17637 AN4058
2872	CONTIG66	11552	12372	1.02	1.28E-01	transcription_start_site	-	8189	8189	-3773	17638 AN4058
2872	CONTIG66	11552	12372	1.02	1.28E-01	transcription_start_site	-	8010	8010	-3952	17639 AN4058
2872	CONTIG66	11552	12372	1.02	1.28E-01	transcription_start_site	-	7860	7860	-4102	17640 AN4058
2872	CONTIG66	11552	12372	1.02	1.28E-01	transcription_start_site	-	9674	9674	-2288	17641 AN4059
2872	CONTIG66	11552	12372	1.02	1.28E-01	transcription_start_site	-	9202	9202	-2760	17642 AN4059
2872	CONTIG66	11552	12372	1.02	1.28E-01	transcription_start_site	-	8978	8978	-2984	17643 AN4059
2872	CONTIG66	11552	12372	1.02	1.28E-01	transcription_start_site	+	11434	11434	528	17646 AN4060
2872	CONTIG66	11552	12372	1.02	1.28E-01	transcription_start_site	+	11318	11318	644	17645 AN4060
2872	CONTIG66	11552	12372	1.02	1.28E-01	transcription_start_site	+	10926	10926	1036	17644 AN4060
1913	CONTIG66	15617	15951	1.4	3.08E-02	transcription_start_site	-	12795	12795	-2989	17647 AN4061
1913	CONTIG66	15617	15951	1.4	3.08E-02	transcription_start_site	-	12563	12563	-3221	17648 AN4061
1913	CONTIG66	15617	15951	1.4	3.08E-02	transcription_start_site	-	12153	12153	-3631	17649 AN4061
2872	CONTIG66	11552	12372	1.02	1.28E-01	transcription_start_site	-	12153	12153	191	17649 AN4061
2872	CONTIG66	11552	12372	1.02	1.28E-01	transcription_start_site	-	12563	12563	601	17648 AN4061
2872	CONTIG66	11552	12372	1.02	1.28E-01	transcription_start_site	-	12795	12795	833	17647 AN4061
2944	CONTIG66	16577	16866	0.98	1.66E-01	transcription_start_site	-	12795	12795	-3926	17647 AN4061
2944	CONTIG66	16577	16866	0.98	1.66E-01	transcription_start_site	-	12563	12563	-4158	17648 AN4061
2944	CONTIG66	16577	16866	0.98	1.66E-01	transcription_start_site	-	12153	12153	-4568	17649 AN4061
1913	CONTIG66	15617	15951	1.4	3.08E-02	transcription_start_site	+	15812	15812	-28	17650 AN4062
2872	CONTIG66	11552	12372	1.02	1.28E-01	transcription_start_site	+	15812	15812	-3850	17650 AN4062
2944	CONTIG66	16577	16866	0.98	1.66E-01	transcription_start_site	+	15812	15812	909	17650 AN4062
628	CONTIG66	20489	20828	2.27	2.16E-04	transcription_start_site	-	18652	18652	-2006	17651 AN4063
628	CONTIG66	20489	20828	2.27	2.16E-04	transcription_start_site	-	20324	20324	-334	17652 AN4064
628	CONTIG66	20489	20828	2.27	2.16E-04	transcription_start_site	-	20079	20079	-579	17653 AN4064
628	CONTIG66	20489	20828	2.27	2.16E-04	transcription_start_site	-	19936	19936	-722	17654 AN4064
628	CONTIG66	20489	20828	2.27	2.16E-04	transcription_start_site	-	19564	19564	-1094	17655 AN4064
628	CONTIG66	20489	20828	2.27	2.16E-04	transcription_start_site	+	23855	23855	-3196	17658 AN4066
628	CONTIG66	20489	20828	2.27	2.16E-04	transcription_start_site	+	24001	24001	-3342	17659 AN4066
628	CONTIG66	20489	20828	2.27	2.16E-04	transcription_start_site	+	24414	24414	-3755	17660 AN4066

2470	CONTIG66	32339	32753	1.21	6.84E-02	transcription_start_site	-	28981	28981	-3565	17664	AN4067
191	CONTIG66	34739	35078	2.95	0.00E+00	transcription_start_site	-	31547	31547	-3361	17667	AN4069
191	CONTIG66	34739	35078	2.95	0.00E+00	transcription_start_site	-	31139	31139	-3769	17668	AN4069
191	CONTIG66	34739	35078	2.95	0.00E+00	transcription_start_site	-	30948	30948	-3960	17669	AN4069
2470	CONTIG66	32339	32753	1.21	6.84E-02	transcription_start_site	-	31547	31547	-999	17667	AN4069
2470	CONTIG66	32339	32753	1.21	6.84E-02	transcription_start_site	-	31139	31139	-1407	17668	AN4069
2470	CONTIG66	32339	32753	1.21	6.84E-02	transcription_start_site	-	30948	30948	-1598	17669	AN4069
2470	CONTIG66	32339	32753	1.21	6.84E-02	transcription_start_site	+	32511	32511	35	17671	AN4070
2470	CONTIG66	32339	32753	1.21	6.84E-02	transcription_start_site	+	32185	32185	361	17670	AN4070
191	CONTIG66	34739	35078	2.95	0.00E+00	transcription_start_site	-	34421	34421	-487	17672	AN4071
191	CONTIG66	34739	35078	2.95	0.00E+00	transcription_start_site	-	34333	34333	-575	17673	AN4071
191	CONTIG66	34739	35078	2.95	0.00E+00	transcription_start_site	-	34168	34168	-740	17674	AN4071
191	CONTIG66	34739	35078	2.95	0.00E+00	transcription_start_site	-	33615	33615	-1293	17675	AN4071
2470	CONTIG66	32339	32753	1.21	6.84E-02	transcription_start_site	-	33615	33615	1069	17675	AN4071
191	CONTIG66	34739	35078	2.95	0.00E+00	transcription_start_site	+	36068	36068	-1159	17676	AN4072
191	CONTIG66	34739	35078	2.95	0.00E+00	transcription_start_site	+	39610	39610	-4701	17677	AN4072
2470	CONTIG66	32339	32753	1.21	6.84E-02	transcription_start_site	+	36068	36068	-3522	17676	AN4072
2945	CONTIG66	42318	42577	0.98	1.66E-01	transcription_start_site	-	43040	43040	592	17681	AN4074
2945	CONTIG66	42318	42577	0.98	1.66E-01	transcription_start_site	-	43196	43196	748	17680	AN4074
2945	CONTIG66	42318	42577	0.98	1.66E-01	transcription_start_site	-	41480	41480	-967	17682	AN4074
2945	CONTIG66	42318	42577	0.98	1.66E-01	transcription_start_site	+	46127	46127	-3679	17684	AN4076
2945	CONTIG66	42318	42577	0.98	1.66E-01	transcription_start_site	+	46796	46796	-4348	17685	AN4076
2806	CONTIG66	65490	65909	1.06	1.07E-01	transcription_start_site	-	62982	62982	-2717	17699	AN4080
2806	CONTIG66	65490	65909	1.06	1.07E-01	transcription_start_site	-	62903	62903	-2796	17700	AN4080
2806	CONTIG66	65490	65909	1.06	1.07E-01	transcription_start_site	-	62828	62828	-2871	17701	AN4080
2806	CONTIG66	65490	65909	1.06	1.07E-01	transcription_start_site	-	62730	62730	-2969	17702	AN4080
2806	CONTIG66	65490	65909	1.06	1.07E-01	transcription_start_site	-	62599	62599	-3100	17703	AN4080
2806	CONTIG66	65490	65909	1.06	1.07E-01	transcription_start_site	-	62506	62506	-3193	17704	AN4080
2806	CONTIG66	65490	65909	1.06	1.07E-01	transcription_start_site	-	62379	62379	-3320	17705	AN4080
2806	CONTIG66	65490	65909	1.06	1.07E-01	transcription_start_site	-	61972	61972	-3727	17706	AN4080
2806	CONTIG66	65490	65909	1.06	1.07E-01	transcription_start_site	-	61562	61562	-4137	17707	AN4080
2806	CONTIG66	65490	65909	1.06	1.07E-01	transcription_start_site	-	66010	66010	310	17717	AN4082
2806	CONTIG66	65490	65909	1.06	1.07E-01	transcription_start_site	-	65237	65237	-462	17718	AN4082
2806	CONTIG66	65490	65909	1.06	1.07E-01	transcription_start_site	-	66688	66688	988	17716	AN4082
2806	CONTIG66	65490	65909	1.06	1.07E-01	transcription_start_site	-	66842	66842	1142	17715	AN4082
2673	CONTIG66	76886	77225	1.13	9.24E-02	transcription_start_site	-	72557	72557	-4498	17723	AN4084
2673	CONTIG66	76886	77225	1.13	9.24E-02	transcription_start_site	-	76560	76560	-495	17724	AN4085
2673	CONTIG66	76886	77225	1.13	9.24E-02	transcription_start_site	-	76423	76423	-632	17725	AN4085
2673	CONTIG66	76886	77225	1.13	9.24E-02	transcription_start_site	-	76248	76248	-807	17726	AN4085
2673	CONTIG66	76886	77225	1.13	9.24E-02	transcription_start_site	-	76042	76042	-1013	17727	AN4085
2673	CONTIG66	76886	77225	1.13	9.24E-02	transcription_start_site	-	75494	75494	-1561	17728	AN4085
2673	CONTIG66	76886	77225	1.13	9.24E-02	transcription_start_site	-	75321	75321	-1734	17729	AN4085
2673	CONTIG66	76886	77225	1.13	9.24E-02	transcription_start_site	-	75113	75113	-1942	17730	AN4085
2673	CONTIG66	76886	77225	1.13	9.24E-02	transcription_start_site	-	74961	74961	-2094	17731	AN4085
2673	CONTIG66	76886	77225	1.13	9.24E-02	transcription_start_site	-	74570	74570	-2485	17732	AN4085
2673	CONTIG66	76886	77225	1.13	9.24E-02	transcription_start_site	+	77374	77374	-318	17733	AN4086
2673	CONTIG66	76886	77225	1.13	9.24E-02	transcription_start_site	+	77484	77484	-428	17734	AN4086
2673	CONTIG66	76886	77225	1.13	9.24E-02	transcription_start_site	+	77589	77589	-533	17735	AN4086
2673	CONTIG66	76886	77225	1.13	9.24E-02	transcription_start_site	+	77663	77663	-607	17736	AN4086
2673	CONTIG66	76886	77225	1.13	9.24E-02	transcription_start_site	+	77794	77794	-738	17737	AN4086
2673	CONTIG66	76886	77225	1.13	9.24E-02	transcription_start_site	+	77987	77987	-931	17738	AN4086
2673	CONTIG66	76886	77225	1.13	9.24E-02	transcription_start_site	+	78303	78303	-1247	17739	AN4086
2673	CONTIG66	76886	77225	1.13	9.24E-02	transcription_start_site	+	78609	78609	-1553	17740	AN4086
2673	CONTIG66	76886	77225	1.13	9.24E-02	transcription_start_site	+	79478	79478	-2422	17741	AN4086
615	CONTIG67	8944	9203	2.29	1.80E-03	transcription_start_site	+	10748	10748	-1674	17745	AN4088
2411	CONTIG67	7671	7945	1.23	1.25E-01	transcription_start_site	+	10748	10748	-2940	17745	AN4088
615	CONTIG67	8944	9203	2.29	1.80E-03	transcription_start_site	+	13102	13102	-4028	17746	AN4089

1693	CONTIG67	26788	27592	1.5	5.22E-02	transcription_start_site	+	29236	29236	-2046	17765	AN4095
1693	CONTIG67	26788	27592	1.5	5.22E-02	transcription_start_site	+	29645	29645	-2455	17766	AN4095
1693	CONTIG67	26788	27592	1.5	5.22E-02	transcription_start_site	+	29968	29968	-2778	17767	AN4095
1693	CONTIG67	26788	27592	1.5	5.22E-02	transcription_start_site	+	30471	30471	-3281	17768	AN4095
1122	CONTIG67	34442	34713	1.81	1.57E-02	transcription_start_site	-	34497	34497	-80	17769	AN4096
1122	CONTIG67	34442	34713	1.81	1.57E-02	transcription_start_site	-	31381	31381	-3196	17770	AN4096
1122	CONTIG67	34442	34713	1.81	1.57E-02	transcription_start_site	+	36893	36893	-2315	17772	AN4098
1122	CONTIG67	34442	34713	1.81	1.57E-02	transcription_start_site	+	37115	37115	-2537	17773	AN4098
1193	CONTIG67	45009	45668	1.76	9.21E-03	transcription_start_site	-	41655	41655	-3683	17774	AN4099
1193	CONTIG67	45009	45668	1.76	9.21E-03	transcription_start_site	-	41058	41058	-4280	17775	AN4099
1193	CONTIG67	45009	45668	1.76	9.21E-03	transcription_start_site	-	40916	40916	-4422	17776	AN4099
1193	CONTIG67	45009	45668	1.76	9.21E-03	transcription_start_site	-	40727	40727	-4611	17777	AN4099
1193	CONTIG67	45009	45668	1.76	9.21E-03	transcription_start_site	-	40399	40399	-4939	17778	AN4099
1193	CONTIG67	45009	45668	1.76	9.21E-03	transcription_start_site	-	40250	40250	-5088	17779	AN4099
2138	CONTIG67	44339	44994	1.32	9.24E-02	transcription_start_site	-	41655	41655	-3011	17774	AN4099
2138	CONTIG67	44339	44994	1.32	9.24E-02	transcription_start_site	-	41058	41058	-3608	17775	AN4099
2138	CONTIG67	44339	44994	1.32	9.24E-02	transcription_start_site	-	40916	40916	-3750	17776	AN4099
2138	CONTIG67	44339	44994	1.32	9.24E-02	transcription_start_site	-	40727	40727	-3939	17777	AN4099
2138	CONTIG67	44339	44994	1.32	9.24E-02	transcription_start_site	-	40399	40399	-4267	17778	AN4099
2138	CONTIG67	44339	44994	1.32	9.24E-02	transcription_start_site	-	40250	40250	-4416	17779	AN4099
2412	CONTIG67	41189	41678	1.23	1.25E-01	transcription_start_site	-	41655	41655	221	17774	AN4099
2412	CONTIG67	41189	41678	1.23	1.25E-01	transcription_start_site	-	41058	41058	-375	17775	AN4099
2412	CONTIG67	41189	41678	1.23	1.25E-01	transcription_start_site	-	40916	40916	-517	17776	AN4099
2412	CONTIG67	41189	41678	1.23	1.25E-01	transcription_start_site	-	40727	40727	-706	17777	AN4099
2412	CONTIG67	41189	41678	1.23	1.25E-01	transcription_start_site	-	40399	40399	-1034	17778	AN4099
2412	CONTIG67	41189	41678	1.23	1.25E-01	transcription_start_site	-	40250	40250	-1183	17779	AN4099
2412	CONTIG67	41189	41678	1.23	1.25E-01	transcription_start_site	-	38803	38803	-2630	17780	AN4099
2412	CONTIG67	41189	41678	1.23	1.25E-01	transcription_start_site	+	42164	42164	-730	17781	AN4100
2412	CONTIG67	41189	41678	1.23	1.25E-01	transcription_start_site	+	42238	42238	-804	17782	AN4100
2412	CONTIG67	41189	41678	1.23	1.25E-01	transcription_start_site	+	42754	42754	-1320	17783	AN4100
1193	CONTIG67	45009	45668	1.76	9.21E-03	transcription_start_site	-	44257	44257	-1081	17784	AN4101
1193	CONTIG67	45009	45668	1.76	9.21E-03	transcription_start_site	-	43949	43949	-1389	17785	AN4101
1193	CONTIG67	45009	45668	1.76	9.21E-03	transcription_start_site	-	43753	43753	-1585	17786	AN4101
1193	CONTIG67	45009	45668	1.76	9.21E-03	transcription_start_site	-	43543	43543	-1795	17787	AN4101
2138	CONTIG67	44339	44994	1.32	9.24E-02	transcription_start_site	-	44257	44257	-409	17784	AN4101
2138	CONTIG67	44339	44994	1.32	9.24E-02	transcription_start_site	-	43949	43949	-717	17785	AN4101
2138	CONTIG67	44339	44994	1.32	9.24E-02	transcription_start_site	-	43753	43753	-913	17786	AN4101
2138	CONTIG67	44339	44994	1.32	9.24E-02	transcription_start_site	-	43543	43543	-1123	17787	AN4101
1193	CONTIG67	45009	45668	1.76	9.21E-03	transcription_start_site	+	46064	46064	-725	17788	AN4102
1193	CONTIG67	45009	45668	1.76	9.21E-03	transcription_start_site	+	46173	46173	-834	17789	AN4102
1193	CONTIG67	45009	45668	1.76	9.21E-03	transcription_start_site	+	46366	46366	-1027	17790	AN4102
1193	CONTIG67	45009	45668	1.76	9.21E-03	transcription_start_site	+	46457	46457	-1118	17791	AN4102
1193	CONTIG67	45009	45668	1.76	9.21E-03	transcription_start_site	+	46562	46562	-1223	17792	AN4102
1193	CONTIG67	45009	45668	1.76	9.21E-03	transcription_start_site	+	47800	47800	-2461	17793	AN4102
1193	CONTIG67	45009	45668	1.76	9.21E-03	transcription_start_site	+	48717	48717	-3378	17794	AN4102
2138	CONTIG67	44339	44994	1.32	9.24E-02	transcription_start_site	+	46064	46064	-1397	17788	AN4102
2138	CONTIG67	44339	44994	1.32	9.24E-02	transcription_start_site	+	46173	46173	-1506	17789	AN4102
2138	CONTIG67	44339	44994	1.32	9.24E-02	transcription_start_site	+	46366	46366	-1699	17790	AN4102
2138	CONTIG67	44339	44994	1.32	9.24E-02	transcription_start_site	+	46457	46457	-1790	17791	AN4102
2138	CONTIG67	44339	44994	1.32	9.24E-02	transcription_start_site	+	46562	46562	-1895	17792	AN4102
2138	CONTIG67	44339	44994	1.32	9.24E-02	transcription_start_site	+	47800	47800	-3133	17793	AN4102
2138	CONTIG67	44339	44994	1.32	9.24E-02	transcription_start_site	+	48717	48717	-4050	17794	AN4102
2412	CONTIG67	41189	41678	1.23	1.25E-01	transcription_start_site	+	46064	46064	-4630	17788	AN4102
2412	CONTIG67	41189	41678	1.23	1.25E-01	transcription_start_site	+	46173	46173	-4739	17789	AN4102
2412	CONTIG67	41189	41678	1.23	1.25E-01	transcription_start_site	+	46366	46366	-4932	17790	AN4102
2412	CONTIG67	41189	41678	1.23	1.25E-01	transcription_start_site	+	46457	46457	-5023	17791	AN4102
2412	CONTIG67	41189	41678	1.23	1.25E-01	transcription_start_site	+	46562	46562	-5128	17792	AN4102

1193	CONTIG67	45009	45668	1.76	9.21E-03	transcription_start_site	+	49564	49564	-4225	17795	AN4103
1193	CONTIG67	45009	45668	1.76	9.21E-03	transcription_start_site	+	49730	49730	-4391	17796	AN4103
1193	CONTIG67	45009	45668	1.76	9.21E-03	transcription_start_site	+	50041	50041	-4702	17797	AN4103
1193	CONTIG67	45009	45668	1.76	9.21E-03	transcription_start_site	+	50268	50268	-4929	17798	AN4103
1193	CONTIG67	45009	45668	1.76	9.21E-03	transcription_start_site	+	50417	50417	-5078	17799	AN4103
1694	CONTIG67	51090	51970	1.5	5.22E-02	transcription_start_site	+	50417	50417	1113	17799	AN4103
1694	CONTIG67	51090	51970	1.5	5.22E-02	transcription_start_site	+	50268	50268	1262	17798	AN4103
2138	CONTIG67	44339	44994	1.32	9.24E-02	transcription_start_site	+	49564	49564	-4897	17795	AN4103
2138	CONTIG67	44339	44994	1.32	9.24E-02	transcription_start_site	+	49730	49730	-5063	17796	AN4103
1255	CONTIG67	54306	55250	1.72	2.12E-02	transcription_start_site	-	54182	54182	-596	17800	AN4104
1255	CONTIG67	54306	55250	1.72	2.12E-02	transcription_start_site	-	53151	53151	-1627	17801	AN4104
2139	CONTIG67	55276	55630	1.32	9.24E-02	transcription_start_site	-	54182	54182	-1271	17800	AN4104
2139	CONTIG67	55276	55630	1.32	9.24E-02	transcription_start_site	-	53151	53151	-2302	17801	AN4104
1255	CONTIG67	54306	55250	1.72	2.12E-02	transcription_start_site	+	54488	54488	290	17802	AN4105
1694	CONTIG67	51090	51970	1.5	5.22E-02	transcription_start_site	+	54488	54488	-2958	17802	AN4105
2139	CONTIG67	55276	55630	1.32	9.24E-02	transcription_start_site	+	54488	54488	965	17802	AN4105
1255	CONTIG67	54306	55250	1.72	2.12E-02	transcription_start_site	-	55981	55981	1203	17810	AN4106
1255	CONTIG67	54306	55250	1.72	2.12E-02	transcription_start_site	-	56241	56241	1463	17809	AN4106
2139	CONTIG67	55276	55630	1.32	9.24E-02	transcription_start_site	-	55981	55981	528	17810	AN4106
2139	CONTIG67	55276	55630	1.32	9.24E-02	transcription_start_site	-	56241	56241	788	17809	AN4106
2139	CONTIG67	55276	55630	1.32	9.24E-02	transcription_start_site	-	56608	56608	1155	17808	AN4106
1199	CONTIG67	71192	71466	1.76	1.84E-02	transcription_start_site	-	67852	67852	-3477	17837	AN4110
1199	CONTIG67	71192	71466	1.76	1.84E-02	transcription_start_site	-	67674	67674	-3655	17838	AN4110
1199	CONTIG67	71192	71466	1.76	1.84E-02	transcription_start_site	-	67513	67513	-3816	17839	AN4110
1199	CONTIG67	71192	71466	1.76	1.84E-02	transcription_start_site	-	67198	67198	-4131	17840	AN4110
1199	CONTIG67	71192	71466	1.76	1.84E-02	transcription_start_site	-	67006	67006	-4323	17841	AN4110
1597	CONTIG67	72387	72726	1.54	4.36E-02	transcription_start_site	-	67852	67852	-4704	17837	AN4110
1597	CONTIG67	72387	72726	1.54	4.36E-02	transcription_start_site	-	67674	67674	-4882	17838	AN4110
1597	CONTIG67	72387	72726	1.54	4.36E-02	transcription_start_site	-	67513	67513	-5043	17839	AN4110
1199	CONTIG67	71192	71466	1.76	1.84E-02	transcription_start_site	+	70492	70492	837	17846	AN4112
1199	CONTIG67	71192	71466	1.76	1.84E-02	transcription_start_site	+	70372	70372	957	17845	AN4112
1199	CONTIG67	71192	71466	1.76	1.84E-02	transcription_start_site	+	72819	72819	-1490	17847	AN4113
1199	CONTIG67	71192	71466	1.76	1.84E-02	transcription_start_site	+	72943	72943	-1614	17848	AN4113
1597	CONTIG67	72387	72726	1.54	4.36E-02	transcription_start_site	+	72819	72819	-262	17847	AN4113
1597	CONTIG67	72387	72726	1.54	4.36E-02	transcription_start_site	+	72943	72943	-386	17848	AN4113
1903	CONTIG67	86187	86456	1.41	6.84E-02	transcription_start_site	-	83047	83047	-3274	17849	AN4114
1903	CONTIG67	86187	86456	1.41	6.84E-02	transcription_start_site	-	82344	82344	-3977	17850	AN4114
1903	CONTIG67	86187	86456	1.41	6.84E-02	transcription_start_site	-	81341	81341	-4980	17851	AN4114
1903	CONTIG67	86187	86456	1.41	6.84E-02	transcription_start_site	+	85629	85629	692	17855	AN4115
1903	CONTIG67	86187	86456	1.41	6.84E-02	transcription_start_site	+	85414	85414	907	17854	AN4115
1695	CONTIG67	93908	94267	1.5	5.22E-02	transcription_start_site	-	88961	88961	-5126	17856	AN4116
1695	CONTIG67	93908	94267	1.5	5.22E-02	transcription_start_site	-	92350	92350	-1737	17860	AN4117
1695	CONTIG67	93908	94267	1.5	5.22E-02	transcription_start_site	-	92097	92097	-1990	17861	AN4117
1695	CONTIG67	93908	94267	1.5	5.22E-02	transcription_start_site	-	95224	95224	1136	17862	AN4118
1695	CONTIG67	93908	94267	1.5	5.22E-02	transcription_start_site	+	95525	95525	-1437	17863	AN4119
1695	CONTIG67	93908	94267	1.5	5.22E-02	transcription_start_site	+	95747	95747	-1659	17864	AN4119
1695	CONTIG67	93908	94267	1.5	5.22E-02	transcription_start_site	+	96810	96810	-2722	17865	AN4119
1695	CONTIG67	93908	94267	1.5	5.22E-02	transcription_start_site	+	96995	96995	-2907	17866	AN4119
1490	CONTIG67	103068	103492	1.59	2.18E-02	transcription_start_site	-	100963	100963	-2317	17867	AN4120
1490	CONTIG67	103068	103492	1.59	2.18E-02	transcription_start_site	-	103697	103697	417	17869	AN4121
1490	CONTIG67	103068	103492	1.59	2.18E-02	transcription_start_site	-	102654	102654	-626	17870	AN4121
1490	CONTIG67	103068	103492	1.59	2.18E-02	transcription_start_site	-	104038	104038	758	17868	AN4121
2251	CONTIG67	106883	107692	1.28	5.82E-02	transcription_start_site	-	104038	104038	-3249	17868	AN4121
2251	CONTIG67	106883	107692	1.28	5.82E-02	transcription_start_site	-	103697	103697	-3590	17869	AN4121
2251	CONTIG67	106883	107692	1.28	5.82E-02	transcription_start_site	-	102654	102654	-4633	17870	AN4121
2251	CONTIG67	106883	107692	1.28	5.82E-02	transcription_start_site	-	106889	106889	-398	17871	AN4122
2251	CONTIG67	106883	107692	1.28	5.82E-02	transcription_start_site	-	106298	106298	-989	17872	AN4122

2251	CONTIG67	106883	107692	1.28	5.82E-02	transcription_start_site	-	105924	105924	-1363	17873	AN4122
2251	CONTIG67	106883	107692	1.28	5.82E-02	transcription_start_site	-	105802	105802	-1485	17874	AN4122
1490	CONTIG67	103068	103492	1.59	2.18E-02	transcription_start_site	+	107377	107377	-4097	17875	AN4123
2251	CONTIG67	106883	107692	1.28	5.82E-02	transcription_start_site	+	107377	107377	-89	17875	AN4123
1491	CONTIG67	112503	112852	1.59	3.70E-02	transcription_start_site	-	110438	110438	-2239	17876	AN4124
1491	CONTIG67	112503	112852	1.59	3.70E-02	transcription_start_site	-	109062	109062	-3615	17877	AN4124
2251	CONTIG67	106883	107692	1.28	5.82E-02	transcription_start_site	+	111068	111068	-3780	17878	AN4125
1491	CONTIG67	112503	112852	1.59	3.70E-02	transcription_start_site	+	112740	112740	-62	17881	AN4126
1491	CONTIG67	112503	112852	1.59	3.70E-02	transcription_start_site	+	112218	112218	459	17880	AN4126
1491	CONTIG67	112503	112852	1.59	3.70E-02	transcription_start_site	+	113167	113167	-489	17882	AN4126
1491	CONTIG67	112503	112852	1.59	3.70E-02	transcription_start_site	+	112110	112110	567	17879	AN4126
1491	CONTIG67	112503	112852	1.59	3.70E-02	transcription_start_site	+	113704	113704	-1026	17883	AN4126
1491	CONTIG67	112503	112852	1.59	3.70E-02	transcription_start_site	+	113881	113881	-1203	17884	AN4126
2251	CONTIG67	106883	107692	1.28	5.82E-02	transcription_start_site	+	112110	112110	-4822	17879	AN4126
2251	CONTIG67	106883	107692	1.28	5.82E-02	transcription_start_site	+	112218	112218	-4930	17880	AN4126
1491	CONTIG67	112503	112852	1.59	3.70E-02	transcription_start_site	+	116383	116383	-3705	17885	AN4127
2750	CONTIG67	147165	147444	1.1	1.93E-01	transcription_start_site	-	142392	142392	-4912	17913	AN4134
2750	CONTIG67	147165	147444	1.1	1.93E-01	transcription_start_site	-	142311	142311	-4993	17914	AN4134
2750	CONTIG67	147165	147444	1.1	1.93E-01	transcription_start_site	+	146941	146941	363	17921	AN4136
2750	CONTIG67	147165	147444	1.1	1.93E-01	transcription_start_site	+	146599	146599	705	17920	AN4136
2750	CONTIG67	147165	147444	1.1	1.93E-01	transcription_start_site	+	146404	146404	900	17919	AN4136
2140	CONTIG67	167345	167619	1.32	9.24E-02	transcription_start_site	-	164601	164601	-2881	17940	AN4142
2140	CONTIG67	167345	167619	1.32	9.24E-02	transcription_start_site	+	168120	168120	-638	17941	AN4143
2140	CONTIG67	167345	167619	1.32	9.24E-02	transcription_start_site	+	168732	168732	-1250	17942	AN4143
553	CONTIG67	178885	179154	2.38	1.22E-03	transcription_start_site	-	176304	176304	-2715	17945	AN4145
553	CONTIG67	178885	179154	2.38	1.22E-03	transcription_start_site	-	174729	174729	-4290	17946	AN4145
553	CONTIG67	178885	179154	2.38	1.22E-03	transcription_start_site	-	174180	174180	-4839	17947	AN4145
1194	CONTIG67	179195	180279	1.76	1.00E-02	transcription_start_site	-	176304	176304	-3433	17945	AN4145
1194	CONTIG67	179195	180279	1.76	1.00E-02	transcription_start_site	-	174729	174729	-5008	17946	AN4145
553	CONTIG67	178885	179154	2.38	1.22E-03	transcription_start_site	-	177258	177258	-1761	17949	AN4146
1194	CONTIG67	179195	180279	1.76	1.00E-02	transcription_start_site	-	177258	177258	-2479	17949	AN4146
553	CONTIG67	178885	179154	2.38	1.22E-03	transcription_start_site	+	178716	178716	303	17951	AN4147
553	CONTIG67	178885	179154	2.38	1.22E-03	transcription_start_site	+	178061	178061	958	17950	AN4147
1194	CONTIG67	179195	180279	1.76	1.00E-02	transcription_start_site	+	178716	178716	1021	17951	AN4147
553	CONTIG67	178885	179154	2.38	1.22E-03	transcription_start_site	+	180804	180804	-1784	17952	AN4148
553	CONTIG67	178885	179154	2.38	1.22E-03	transcription_start_site	+	181075	181075	-2055	17953	AN4148
553	CONTIG67	178885	179154	2.38	1.22E-03	transcription_start_site	+	181349	181349	-2329	17954	AN4148
553	CONTIG67	178885	179154	2.38	1.22E-03	transcription_start_site	+	182339	182339	-3319	17955	AN4148
1194	CONTIG67	179195	180279	1.76	1.00E-02	transcription_start_site	+	180804	180804	-1067	17952	AN4148
1194	CONTIG67	179195	180279	1.76	1.00E-02	transcription_start_site	+	181075	181075	-1338	17953	AN4148
1194	CONTIG67	179195	180279	1.76	1.00E-02	transcription_start_site	+	181349	181349	-1612	17954	AN4148
1194	CONTIG67	179195	180279	1.76	1.00E-02	transcription_start_site	+	182339	182339	-2602	17955	AN4148
693	CONTIG67	197196	197695	2.2	3.03E-03	transcription_start_site	-	194277	194277	-3168	17963	AN4152
693	CONTIG67	197196	197695	2.2	3.03E-03	transcription_start_site	-	194124	194124	-3321	17964	AN4152
693	CONTIG67	197196	197695	2.2	3.03E-03	transcription_start_site	-	193978	193978	-3467	17965	AN4152
693	CONTIG67	197196	197695	2.2	3.03E-03	transcription_start_site	-	192932	192932	-4513	17966	AN4152
693	CONTIG67	197196	197695	2.2	3.03E-03	transcription_start_site	-	192574	192574	-4871	17967	AN4152
693	CONTIG67	197196	197695	2.2	3.03E-03	transcription_start_site	-	196738	196738	-707	17968	AN4153
693	CONTIG67	197196	197695	2.2	3.03E-03	transcription_start_site	-	196618	196618	-827	17969	AN4153
693	CONTIG67	197196	197695	2.2	3.03E-03	transcription_start_site	-	196444	196444	-1001	17970	AN4153
693	CONTIG67	197196	197695	2.2	3.03E-03	transcription_start_site	-	195930	195930	-1515	17971	AN4153
693	CONTIG67	197196	197695	2.2	3.03E-03	transcription_start_site	+	197711	197711	-265	17972	AN4154
693	CONTIG67	197196	197695	2.2	3.03E-03	transcription_start_site	+	201808	201808	-4362	17975	AN4156
693	CONTIG67	197196	197695	2.2	3.03E-03	transcription_start_site	+	202018	202018	-4572	17976	AN4156
693	CONTIG67	197196	197695	2.2	3.03E-03	transcription_start_site	+	202253	202253	-4807	17977	AN4156
693	CONTIG67	197196	197695	2.2	3.03E-03	transcription_start_site	+	202631	202631	-5185	17978	AN4156
1394	CONTIG67	210386	211564	1.63	1.23E-02	transcription_start_site	-	205963	205963	-5012	17981	AN4157

1394	CONTIG67	210386	211564	1.63	1.23E-02	transcription_start_site	-	205609	205609	-5366	17982	AN4157
1394	CONTIG67	210386	211564	1.63	1.23E-02	transcription_start_site	-	207896	207896	-3079	17987	AN4158
1394	CONTIG67	210386	211564	1.63	1.23E-02	transcription_start_site	-	207684	207684	-3291	17988	AN4158
1394	CONTIG67	210386	211564	1.63	1.23E-02	transcription_start_site	-	207499	207499	-3476	17989	AN4158
478	CONTIG67	212930	213584	2.47	0.00E+00	transcription_start_site	-	211211	211211	-2046	17990	AN4159
478	CONTIG67	212930	213584	2.47	0.00E+00	transcription_start_site	-	211148	211148	-2109	17991	AN4159
478	CONTIG67	212930	213584	2.47	0.00E+00	transcription_start_site	-	211042	211042	-2215	17992	AN4159
478	CONTIG67	212930	213584	2.47	0.00E+00	transcription_start_site	-	210781	210781	-2476	17993	AN4159
478	CONTIG67	212930	213584	2.47	0.00E+00	transcription_start_site	-	209806	209806	-3451	17994	AN4159
1394	CONTIG67	210386	211564	1.63	1.23E-02	transcription_start_site	-	211042	211042	67	17992	AN4159
1394	CONTIG67	210386	211564	1.63	1.23E-02	transcription_start_site	-	211148	211148	173	17991	AN4159
1394	CONTIG67	210386	211564	1.63	1.23E-02	transcription_start_site	-	210781	210781	-194	17993	AN4159
1394	CONTIG67	210386	211564	1.63	1.23E-02	transcription_start_site	-	211211	211211	236	17990	AN4159
1394	CONTIG67	210386	211564	1.63	1.23E-02	transcription_start_site	-	209806	209806	-1169	17994	AN4159
2535	CONTIG67	214576	214935	1.19	1.44E-01	transcription_start_site	-	211211	211211	-3544	17990	AN4159
2535	CONTIG67	214576	214935	1.19	1.44E-01	transcription_start_site	-	211148	211148	-3607	17991	AN4159
2535	CONTIG67	214576	214935	1.19	1.44E-01	transcription_start_site	-	211042	211042	-3713	17992	AN4159
2535	CONTIG67	214576	214935	1.19	1.44E-01	transcription_start_site	-	210781	210781	-3974	17993	AN4159
2535	CONTIG67	214576	214935	1.19	1.44E-01	transcription_start_site	-	209806	209806	-4949	17994	AN4159
478	CONTIG67	212930	213584	2.47	0.00E+00	transcription_start_site	-	214387	214387	1130	17997	AN4160
2535	CONTIG67	214576	214935	1.19	1.44E-01	transcription_start_site	-	214387	214387	-368	17997	AN4160
2535	CONTIG67	214576	214935	1.19	1.44E-01	transcription_start_site	-	215538	215538	782	17996	AN4160
2003	CONTIG67	232056	232490	1.37	6.16E-02	transcription_start_site	-	229289	229289	-2984	18012	AN4166
2003	CONTIG67	232056	232490	1.37	6.16E-02	transcription_start_site	-	229117	229117	-3156	18013	AN4166
2003	CONTIG67	232056	232490	1.37	6.16E-02	transcription_start_site	-	228861	228861	-3412	18014	AN4166
2003	CONTIG67	232056	232490	1.37	6.16E-02	transcription_start_site	-	227874	227874	-4399	18015	AN4166
2003	CONTIG67	232056	232490	1.37	6.16E-02	transcription_start_site	-	227524	227524	-4749	18016	AN4166
2003	CONTIG67	232056	232490	1.37	6.16E-02	transcription_start_site	-	227268	227268	-5005	18017	AN4166
2003	CONTIG67	232056	232490	1.37	6.16E-02	transcription_start_site	-	227138	227138	-5135	18018	AN4166
2003	CONTIG67	232056	232490	1.37	6.16E-02	transcription_start_site	-	232312	232312	39	18019	AN4167
2003	CONTIG67	232056	232490	1.37	6.16E-02	transcription_start_site	-	231955	231955	-318	18020	AN4167
2003	CONTIG67	232056	232490	1.37	6.16E-02	transcription_start_site	+	232740	232740	-467	18021	AN4168
2003	CONTIG67	232056	232490	1.37	6.16E-02	transcription_start_site	+	232876	232876	-603	18022	AN4168
2003	CONTIG67	232056	232490	1.37	6.16E-02	transcription_start_site	+	233041	233041	-768	18023	AN4168
2003	CONTIG67	232056	232490	1.37	6.16E-02	transcription_start_site	+	234159	234159	-1886	18024	AN4168
2751	CONTIG67	237542	237806	1.1	1.93E-01	transcription_start_site	+	238126	238126	-452	18025	AN4169
2751	CONTIG67	237542	237806	1.1	1.93E-01	transcription_start_site	+	238365	238365	-691	18026	AN4169
2751	CONTIG67	237542	237806	1.1	1.93E-01	transcription_start_site	+	240718	240718	-3044	18027	AN4169
519	CONTIG67	244283	244797	2.42	1.01E-03	transcription_start_site	+	247018	247018	-2478	18028	AN4170
519	CONTIG67	244283	244797	2.42	1.01E-03	transcription_start_site	+	247150	247150	-2610	18029	AN4170
519	CONTIG67	244283	244797	2.42	1.01E-03	transcription_start_site	+	247358	247358	-2818	18030	AN4170
519	CONTIG67	244283	244797	2.42	1.01E-03	transcription_start_site	+	247727	247727	-3187	18031	AN4170
519	CONTIG67	244283	244797	2.42	1.01E-03	transcription_start_site	+	248206	248206	-3666	18032	AN4170
1123	CONTIG67	246088	246437	1.81	1.57E-02	transcription_start_site	+	247018	247018	-755	18028	AN4170
1123	CONTIG67	246088	246437	1.81	1.57E-02	transcription_start_site	+	247150	247150	-887	18029	AN4170
1123	CONTIG67	246088	246437	1.81	1.57E-02	transcription_start_site	+	247358	247358	-1095	18030	AN4170
1123	CONTIG67	246088	246437	1.81	1.57E-02	transcription_start_site	+	247727	247727	-1464	18031	AN4170
1123	CONTIG67	246088	246437	1.81	1.57E-02	transcription_start_site	+	248206	248206	-1943	18032	AN4170
1123	CONTIG67	246088	246437	1.81	1.57E-02	transcription_start_site	+	250289	250289	-4026	18033	AN4171
1123	CONTIG67	246088	246437	1.81	1.57E-02	transcription_start_site	+	250491	250491	-4228	18034	AN4171
1123	CONTIG67	246088	246437	1.81	1.57E-02	transcription_start_site	+	250688	250688	-4425	18035	AN4171
1123	CONTIG67	246088	246437	1.81	1.57E-02	transcription_start_site	+	250831	250831	-4568	18036	AN4171
1904	CONTIG67	249768	250032	1.41	6.84E-02	transcription_start_site	+	250289	250289	-389	18033	AN4171
1904	CONTIG67	249768	250032	1.41	6.84E-02	transcription_start_site	+	250491	250491	-591	18034	AN4171
1904	CONTIG67	249768	250032	1.41	6.84E-02	transcription_start_site	+	250688	250688	-788	18035	AN4171
1904	CONTIG67	249768	250032	1.41	6.84E-02	transcription_start_site	+	250831	250831	-931	18036	AN4171
1897	CONTIG67	252620	253109	1.41	5.05E-02	transcription_start_site	-	253258	253258	393	18038	AN4172

1897	CONTIG67	252620	253109	1.41	5.05E-02	transcription_start_site	-	253732	253732	867	18037	AN4172
1718	CONTIG68	15468	16267	1.49	4.36E-02	transcription_start_site	-	13101	13101	-2766	18053	AN4176
1718	CONTIG68	15468	16267	1.49	4.36E-02	transcription_start_site	-	11361	11361	-4506	18054	AN4176
2040	CONTIG68	16353	16720	1.36	6.84E-02	transcription_start_site	-	13101	13101	-3435	18053	AN4176
2040	CONTIG68	16353	16720	1.36	6.84E-02	transcription_start_site	-	11361	11361	-5175	18054	AN4176
2724	CONTIG68	17253	18292	1.11	1.11E-01	transcription_start_site	-	13101	13101	-4671	18053	AN4176
1718	CONTIG68	15468	16267	1.49	4.36E-02	transcription_start_site	+	15634	15634	233	18058	AN4178
1718	CONTIG68	15468	16267	1.49	4.36E-02	transcription_start_site	+	15510	15510	357	18057	AN4178
2040	CONTIG68	16353	16720	1.36	6.84E-02	transcription_start_site	+	15634	15634	902	18058	AN4178
2040	CONTIG68	16353	16720	1.36	6.84E-02	transcription_start_site	+	15510	15510	1026	18057	AN4178
1718	CONTIG68	15468	16267	1.49	4.36E-02	transcription_start_site	+	21071	21071	-5203	18059	AN4180
2040	CONTIG68	16353	16720	1.36	6.84E-02	transcription_start_site	+	21071	21071	-4534	18059	AN4180
2724	CONTIG68	17253	18292	1.11	1.11E-01	transcription_start_site	+	21071	21071	-3298	18059	AN4180
2724	CONTIG68	17253	18292	1.11	1.11E-01	transcription_start_site	+	21961	21961	-4188	18060	AN4180
2724	CONTIG68	17253	18292	1.11	1.11E-01	transcription_start_site	+	22274	22274	-4501	18061	AN4180
857	CONTIG68	54453	54727	2.05	4.61E-03	transcription_start_site	-	50236	50236	-4354	18080	AN4187
857	CONTIG68	54453	54727	2.05	4.61E-03	transcription_start_site	-	50173	50173	-4417	18081	AN4187
857	CONTIG68	54453	54727	2.05	4.61E-03	transcription_start_site	-	50083	50083	-4507	18082	AN4187
2733	CONTIG68	50793	51074	1.11	1.66E-01	transcription_start_site	-	50236	50236	-697	18080	AN4187
2733	CONTIG68	50793	51074	1.11	1.66E-01	transcription_start_site	-	50173	50173	-760	18081	AN4187
2733	CONTIG68	50793	51074	1.11	1.66E-01	transcription_start_site	-	50083	50083	-850	18082	AN4187
857	CONTIG68	54453	54727	2.05	4.61E-03	transcription_start_site	+	54074	54074	516	18086	AN4188
857	CONTIG68	54453	54727	2.05	4.61E-03	transcription_start_site	+	53867	53867	723	18085	AN4188
2733	CONTIG68	50793	51074	1.11	1.66E-01	transcription_start_site	+	52299	52299	-1365	18084	AN4188
2733	CONTIG68	50793	51074	1.11	1.66E-01	transcription_start_site	+	53867	53867	-2933	18085	AN4188
2733	CONTIG68	50793	51074	1.11	1.66E-01	transcription_start_site	+	54074	54074	-3140	18086	AN4188
899	CONTIG68	58134	58403	2	5.20E-03	transcription_start_site	-	57823	57823	-445	18087	AN4189
899	CONTIG68	58134	58403	2	5.20E-03	transcription_start_site	-	57063	57063	-1205	18088	AN4189
899	CONTIG68	58134	58403	2	5.20E-03	transcription_start_site	-	56724	56724	-1544	18089	AN4189
899	CONTIG68	58134	58403	2	5.20E-03	transcription_start_site	-	56312	56312	-1956	18090	AN4189
899	CONTIG68	58134	58403	2	5.20E-03	transcription_start_site	-	56117	56117	-2151	18091	AN4189
1595	CONTIG68	61521	62151	1.54	3.70E-02	transcription_start_site	-	57823	57823	-4013	18087	AN4189
1595	CONTIG68	61521	62151	1.54	3.70E-02	transcription_start_site	-	57063	57063	-4773	18088	AN4189
1595	CONTIG68	61521	62151	1.54	3.70E-02	transcription_start_site	-	56724	56724	-5112	18089	AN4189
899	CONTIG68	58134	58403	2	5.20E-03	transcription_start_site	+	61788	61788	-3519	18092	AN4190
899	CONTIG68	58134	58403	2	5.20E-03	transcription_start_site	+	62273	62273	-4004	18093	AN4190
1595	CONTIG68	61521	62151	1.54	3.70E-02	transcription_start_site	+	61788	61788	48	18092	AN4190
1595	CONTIG68	61521	62151	1.54	3.70E-02	transcription_start_site	+	62273	62273	-437	18093	AN4190
1595	CONTIG68	61521	62151	1.54	3.70E-02	transcription_start_site	+	64116	64116	-2280	18094	AN4191
1595	CONTIG68	61521	62151	1.54	3.70E-02	transcription_start_site	+	64328	64328	-2492	18095	AN4191
1595	CONTIG68	61521	62151	1.54	3.70E-02	transcription_start_site	+	64537	64537	-2701	18096	AN4191
2734	CONTIG68	64662	64941	1.11	1.66E-01	transcription_start_site	+	64537	64537	264	18096	AN4191
2734	CONTIG68	64662	64941	1.11	1.66E-01	transcription_start_site	+	64328	64328	473	18095	AN4191
2734	CONTIG68	64662	64941	1.11	1.66E-01	transcription_start_site	+	64116	64116	685	18094	AN4191
1595	CONTIG68	61521	62151	1.54	3.70E-02	transcription_start_site	+	65956	65956	-4120	18097	AN4192
1595	CONTIG68	61521	62151	1.54	3.70E-02	transcription_start_site	+	66497	66497	-4661	18098	AN4192
2734	CONTIG68	64662	64941	1.11	1.66E-01	transcription_start_site	+	65956	65956	-1154	18097	AN4192
2734	CONTIG68	64662	64941	1.11	1.66E-01	transcription_start_site	+	66497	66497	-1695	18098	AN4192
2734	CONTIG68	64662	64941	1.11	1.66E-01	transcription_start_site	+	67711	67711	-2909	18099	AN4192
1811	CONTIG68	72757	73027	1.45	5.22E-02	transcription_start_site	-	71221	71221	-1671	18100	AN4193
1811	CONTIG68	72757	73027	1.45	5.22E-02	transcription_start_site	-	68771	68771	-4121	18101	AN4193
2254	CONTIG68	86269	86678	1.28	7.25E-02	transcription_start_site	-	83061	83061	-3412	18112	AN4196
2254	CONTIG68	86269	86678	1.28	7.25E-02	transcription_start_site	-	82977	82977	-3496	18113	AN4196
2254	CONTIG68	86269	86678	1.28	7.25E-02	transcription_start_site	+	86252	86252	221	18119	AN4197
2254	CONTIG68	86269	86678	1.28	7.25E-02	transcription_start_site	+	85973	85973	500	18118	AN4197
2254	CONTIG68	86269	86678	1.28	7.25E-02	transcription_start_site	+	85530	85530	943	18117	AN4197
2254	CONTIG68	86269	86678	1.28	7.25E-02	transcription_start_site	-	87248	87248	774	18124	AN4198

2254	CONTIG68	86269	86678	1.28	7.25E-02	transcription_start_site	-	87515	87515	1041	18123	AN4198
2254	CONTIG68	86269	86678	1.28	7.25E-02	transcription_start_site	-	87637	87637	1163	18122	AN4198
2735	CONTIG68	92933	93209	1.11	1.66E-01	transcription_start_site	-	88406	88406	-4665	18120	AN4198
2735	CONTIG68	92933	93209	1.11	1.66E-01	transcription_start_site	-	87933	87933	-5138	18121	AN4198
2735	CONTIG68	92933	93209	1.11	1.66E-01	transcription_start_site	+	94267	94267	-1196	18125	AN4199
2800	CONTIG68	109132	109420	1.07	1.93E-01	transcription_start_site	-	106827	106827	-2449	18126	AN4201
2800	CONTIG68	109132	109420	1.07	1.93E-01	transcription_start_site	-	106625	106625	-2651	18127	AN4201
2800	CONTIG68	109132	109420	1.07	1.93E-01	transcription_start_site	-	106338	106338	-2938	18128	AN4201
2800	CONTIG68	109132	109420	1.07	1.93E-01	transcription_start_site	-	105833	105833	-3443	18129	AN4201
2800	CONTIG68	109132	109420	1.07	1.93E-01	transcription_start_site	-	105145	105145	-4131	18130	AN4201
2800	CONTIG68	109132	109420	1.07	1.93E-01	transcription_start_site	-	109117	109117	-159	18131	AN4202
2800	CONTIG68	109132	109420	1.07	1.93E-01	transcription_start_site	-	108995	108995	-281	18132	AN4202
2800	CONTIG68	109132	109420	1.07	1.93E-01	transcription_start_site	-	108661	108661	-615	18133	AN4202
2800	CONTIG68	109132	109420	1.07	1.93E-01	transcription_start_site	-	108272	108272	-1004	18134	AN4202
239	CONTIG68	119787	120056	2.86	0.00E+00	transcription_start_site	+	121654	121654	-1732	18140	AN4206
344	CONTIG68	122112	122467	2.69	0.00E+00	transcription_start_site	+	121654	121654	635	18140	AN4206
983	CONTIG68	120977	121630	1.92	3.31E-03	transcription_start_site	+	121654	121654	-350	18140	AN4206
671	CONTIG68	138162	138501	2.22	1.80E-03	transcription_start_site	-	137126	137126	-1205	18156	AN4210
671	CONTIG68	138162	138501	2.22	1.80E-03	transcription_start_site	-	136811	136811	-1520	18157	AN4210
671	CONTIG68	138162	138501	2.22	1.80E-03	transcription_start_site	-	136647	136647	-1684	18158	AN4210
671	CONTIG68	138162	138501	2.22	1.80E-03	transcription_start_site	-	133797	133797	-4534	18159	AN4210
671	CONTIG68	138162	138501	2.22	1.80E-03	transcription_start_site	+	139399	139399	-1067	18160	AN4211
671	CONTIG68	138162	138501	2.22	1.80E-03	transcription_start_site	+	139763	139763	-1431	18161	AN4211
671	CONTIG68	138162	138501	2.22	1.80E-03	transcription_start_site	+	141660	141660	-3328	18162	AN4211
671	CONTIG68	138162	138501	2.22	1.80E-03	transcription_start_site	+	142185	142185	-3853	18163	AN4211
715	CONTIG68	142207	142486	2.18	2.22E-03	transcription_start_site	+	142185	142185	161	18163	AN4211
715	CONTIG68	142207	142486	2.18	2.22E-03	transcription_start_site	+	141660	141660	686	18162	AN4211
1271	CONTIG68	149706	150052	1.71	1.84E-02	transcription_start_site	-	145786	145786	-4093	18164	AN4212
1271	CONTIG68	149706	150052	1.71	1.84E-02	transcription_start_site	-	145398	145398	-4481	18165	AN4212
1271	CONTIG68	149706	150052	1.71	1.84E-02	transcription_start_site	-	145256	145256	-4623	18166	AN4212
715	CONTIG68	142207	142486	2.18	2.22E-03	transcription_start_site	+	147268	147268	-4921	18167	AN4213
1271	CONTIG68	149706	150052	1.71	1.84E-02	transcription_start_site	+	150378	150378	-499	18171	AN4214
1271	CONTIG68	149706	150052	1.71	1.84E-02	transcription_start_site	+	150558	150558	-679	18172	AN4214
2374	CONTIG68	151431	151865	1.24	9.26E-02	transcription_start_site	+	150558	150558	1090	18172	AN4214
1271	CONTIG68	149706	150052	1.71	1.84E-02	transcription_start_site	+	152754	152754	-2875	18173	AN4215
1271	CONTIG68	149706	150052	1.71	1.84E-02	transcription_start_site	+	152870	152870	-2991	18174	AN4215
1271	CONTIG68	149706	150052	1.71	1.84E-02	transcription_start_site	+	153051	153051	-3172	18175	AN4215
2374	CONTIG68	151431	151865	1.24	9.26E-02	transcription_start_site	+	152754	152754	-1106	18173	AN4215
2374	CONTIG68	151431	151865	1.24	9.26E-02	transcription_start_site	+	152870	152870	-1222	18174	AN4215
2374	CONTIG68	151431	151865	1.24	9.26E-02	transcription_start_site	+	153051	153051	-1403	18175	AN4215
2374	CONTIG68	151431	151865	1.24	9.26E-02	transcription_start_site	+	155158	155158	-3510	18177	AN4217
2374	CONTIG68	151431	151865	1.24	9.26E-02	transcription_start_site	+	155286	155286	-3638	18178	AN4217
2374	CONTIG68	151431	151865	1.24	9.26E-02	transcription_start_site	+	155391	155391	-3743	18179	AN4217
400	CONTIG68	161942	162207	2.6	2.39E-04	transcription_start_site	-	161800	161800	-274	18180	AN4218
400	CONTIG68	161942	162207	2.6	2.39E-04	transcription_start_site	-	161734	161734	-340	18181	AN4218
400	CONTIG68	161942	162207	2.6	2.39E-04	transcription_start_site	-	161611	161611	-463	18182	AN4218
400	CONTIG68	161942	162207	2.6	2.39E-04	transcription_start_site	-	161361	161361	-713	18183	AN4218
2526	CONTIG68	160427	161076	1.19	1.07E-01	transcription_start_site	-	161361	161361	609	18183	AN4218
2526	CONTIG68	160427	161076	1.19	1.07E-01	transcription_start_site	-	161611	161611	859	18182	AN4218
2526	CONTIG68	160427	161076	1.19	1.07E-01	transcription_start_site	-	161734	161734	982	18181	AN4218
2526	CONTIG68	160427	161076	1.19	1.07E-01	transcription_start_site	-	161800	161800	1048	18180	AN4218
400	CONTIG68	161942	162207	2.6	2.39E-04	transcription_start_site	-	162735	162735	660	18187	AN4219
400	CONTIG68	161942	162207	2.6	2.39E-04	transcription_start_site	-	163118	163118	1043	18186	AN4219
400	CONTIG68	161942	162207	2.6	2.39E-04	transcription_start_site	+	163801	163801	-1726	18188	AN4220
400	CONTIG68	161942	162207	2.6	2.39E-04	transcription_start_site	+	163992	163992	-1917	18189	AN4220
400	CONTIG68	161942	162207	2.6	2.39E-04	transcription_start_site	+	164071	164071	-1996	18190	AN4220
400	CONTIG68	161942	162207	2.6	2.39E-04	transcription_start_site	+	164449	164449	-2374	18191	AN4220

2526	CONTIG68	160427	161076	1.19	1.07E-01	transcription_start_site	+	163801	163801	-3049	18188 AN4220
2526	CONTIG68	160427	161076	1.19	1.07E-01	transcription_start_site	+	163992	163992	-3240	18189 AN4220
2526	CONTIG68	160427	161076	1.19	1.07E-01	transcription_start_site	+	164071	164071	-3319	18190 AN4220
2526	CONTIG68	160427	161076	1.19	1.07E-01	transcription_start_site	+	164449	164449	-3697	18191 AN4220
2375	CONTIG68	221115	221474	1.24	9.26E-02	transcription_start_site	-	216341	216341	-4953	18233 AN4237
401	CONTIG68	222980	223329	2.6	2.39E-04	transcription_start_site	-	222390	222390	-764	18235 AN4238
401	CONTIG68	222980	223329	2.6	2.39E-04	transcription_start_site	-	221934	221934	-1220	18236 AN4238
401	CONTIG68	222980	223329	2.6	2.39E-04	transcription_start_site	-	220589	220589	-2565	18237 AN4238
1801	CONTIG68	223965	224902	1.45	5.26E-03	transcription_start_site	-	222390	222390	-2043	18235 AN4238
1801	CONTIG68	223965	224902	1.45	5.26E-03	transcription_start_site	-	221934	221934	-2499	18236 AN4238
1801	CONTIG68	223965	224902	1.45	5.26E-03	transcription_start_site	-	220589	220589	-3844	18237 AN4238
2375	CONTIG68	221115	221474	1.24	9.26E-02	transcription_start_site	-	221934	221934	639	18236 AN4238
2375	CONTIG68	221115	221474	1.24	9.26E-02	transcription_start_site	-	220589	220589	-705	18237 AN4238
2375	CONTIG68	221115	221474	1.24	9.26E-02	transcription_start_site	-	222390	222390	1095	18235 AN4238
401	CONTIG68	222980	223329	2.6	2.39E-04	transcription_start_site	+	225186	225186	-2031	18238 AN4239
401	CONTIG68	222980	223329	2.6	2.39E-04	transcription_start_site	+	225345	225345	-2190	18239 AN4239
401	CONTIG68	222980	223329	2.6	2.39E-04	transcription_start_site	+	226542	226542	-3387	18240 AN4239
1801	CONTIG68	223965	224902	1.45	5.26E-03	transcription_start_site	+	225186	225186	-752	18238 AN4239
1801	CONTIG68	223965	224902	1.45	5.26E-03	transcription_start_site	+	225345	225345	-911	18239 AN4239
1801	CONTIG68	223965	224902	1.45	5.26E-03	transcription_start_site	+	226542	226542	-2108	18240 AN4239
2375	CONTIG68	221115	221474	1.24	9.26E-02	transcription_start_site	+	225186	225186	-3891	18238 AN4239
2375	CONTIG68	221115	221474	1.24	9.26E-02	transcription_start_site	+	225345	225345	-4050	18239 AN4239
1425	CONTIG68	232878	233302	1.62	2.62E-02	transcription_start_site	-	228658	228658	-4432	18241 AN4240
1425	CONTIG68	232878	233302	1.62	2.62E-02	transcription_start_site	-	228425	228425	-4665	18242 AN4240
1425	CONTIG68	232878	233302	1.62	2.62E-02	transcription_start_site	-	228168	228168	-4922	18243 AN4240
1425	CONTIG68	232878	233302	1.62	2.62E-02	transcription_start_site	-	231245	231245	-1845	18244 AN4241
1425	CONTIG68	232878	233302	1.62	2.62E-02	transcription_start_site	-	230936	230936	-2154	18245 AN4241
1425	CONTIG68	232878	233302	1.62	2.62E-02	transcription_start_site	-	230604	230604	-2486	18246 AN4241
2796	CONTIG68	234694	235108	1.07	1.79E-01	transcription_start_site	-	231245	231245	-3656	18244 AN4241
2796	CONTIG68	234694	235108	1.07	1.79E-01	transcription_start_site	-	230936	230936	-3965	18245 AN4241
2796	CONTIG68	234694	235108	1.07	1.79E-01	transcription_start_site	-	230604	230604	-4297	18246 AN4241
1425	CONTIG68	232878	233302	1.62	2.62E-02	transcription_start_site	+	232197	232197	893	18248 AN4242
1425	CONTIG68	232878	233302	1.62	2.62E-02	transcription_start_site	+	234177	234177	-1087	18249 AN4243
1425	CONTIG68	232878	233302	1.62	2.62E-02	transcription_start_site	+	234756	234756	-1666	18250 AN4243
1425	CONTIG68	232878	233302	1.62	2.62E-02	transcription_start_site	+	235426	235426	-2336	18251 AN4243
1425	CONTIG68	232878	233302	1.62	2.62E-02	transcription_start_site	+	236036	236036	-2946	18252 AN4243
2796	CONTIG68	234694	235108	1.07	1.79E-01	transcription_start_site	+	234756	234756	145	18250 AN4243
2796	CONTIG68	234694	235108	1.07	1.79E-01	transcription_start_site	+	235426	235426	-525	18251 AN4243
2796	CONTIG68	234694	235108	1.07	1.79E-01	transcription_start_site	+	234177	234177	724	18249 AN4243
2796	CONTIG68	234694	235108	1.07	1.79E-01	transcription_start_site	+	236036	236036	-1135	18252 AN4243
1425	CONTIG68	232878	233302	1.62	2.62E-02	transcription_start_site	+	238166	238166	-5076	18255 AN4245
2796	CONTIG68	234694	235108	1.07	1.79E-01	transcription_start_site	+	238166	238166	-3265	18255 AN4245
2796	CONTIG68	234694	235108	1.07	1.79E-01	transcription_start_site	+	238418	238418	-3517	18256 AN4245
2796	CONTIG68	234694	235108	1.07	1.79E-01	transcription_start_site	+	238550	238550	-3649	18257 AN4245
2796	CONTIG68	234694	235108	1.07	1.79E-01	transcription_start_site	+	238802	238802	-3901	18258 AN4245
1353	CONTIG68	246095	246504	1.66	2.12E-02	transcription_start_site	-	246194	246194	-105	18260 AN4247
1353	CONTIG68	246095	246504	1.66	2.12E-02	transcription_start_site	-	246057	246057	-242	18261 AN4247
1353	CONTIG68	246095	246504	1.66	2.12E-02	transcription_start_site	-	244478	244478	-1821	18262 AN4247
2636	CONTIG68	249535	249814	1.15	1.44E-01	transcription_start_site	-	246194	246194	-3480	18260 AN4247
2636	CONTIG68	249535	249814	1.15	1.44E-01	transcription_start_site	-	246057	246057	-3617	18261 AN4247
2636	CONTIG68	249535	249814	1.15	1.44E-01	transcription_start_site	-	248599	248599	-1075	18263 AN4248
2636	CONTIG68	249535	249814	1.15	1.44E-01	transcription_start_site	-	248374	248374	-1300	18264 AN4248
2636	CONTIG68	249535	249814	1.15	1.44E-01	transcription_start_site	-	247870	247870	-1804	18265 AN4248
1353	CONTIG68	246095	246504	1.66	2.12E-02	transcription_start_site	+	249283	249283	-2983	18266 AN4249
1353	CONTIG68	246095	246504	1.66	2.12E-02	transcription_start_site	+	249586	249586	-3286	18267 AN4249
1353	CONTIG68	246095	246504	1.66	2.12E-02	transcription_start_site	+	250261	250261	-3961	18268 AN4249
1353	CONTIG68	246095	246504	1.66	2.12E-02	transcription_start_site	+	250317	250317	-4017	18269 AN4249

2636	CONTIG68	249535	249814	1.15	1.44E-01	transcription_start_site	+	249586	249586	88	18267 AN4249
2636	CONTIG68	249535	249814	1.15	1.44E-01	transcription_start_site	+	249283	249283	391	18266 AN4249
2636	CONTIG68	249535	249814	1.15	1.44E-01	transcription_start_site	+	250261	250261	-586	18268 AN4249
2636	CONTIG68	249535	249814	1.15	1.44E-01	transcription_start_site	+	250317	250317	-642	18269 AN4249
877	CONTIG69	9310	9804	2.02	3.61E-03	transcription_start_site	-	6168	6168	-3389	18275 AN4251
877	CONTIG69	9310	9804	2.02	3.61E-03	transcription_start_site	-	6069	6069	-3488	18276 AN4251
877	CONTIG69	9310	9804	2.02	3.61E-03	transcription_start_site	-	5824	5824	-3733	18277 AN4251
1617	CONTIG69	9995	10494	1.53	3.08E-02	transcription_start_site	-	6168	6168	-4076	18275 AN4251
1617	CONTIG69	9995	10494	1.53	3.08E-02	transcription_start_site	-	6069	6069	-4175	18276 AN4251
1617	CONTIG69	9995	10494	1.53	3.08E-02	transcription_start_site	-	5824	5824	-4420	18277 AN4251
877	CONTIG69	9310	9804	2.02	3.61E-03	transcription_start_site	-	9256	9256	-301	18278 AN4252
877	CONTIG69	9310	9804	2.02	3.61E-03	transcription_start_site	-	8926	8926	-631	18279 AN4252
877	CONTIG69	9310	9804	2.02	3.61E-03	transcription_start_site	-	8784	8784	-773	18280 AN4252
1617	CONTIG69	9995	10494	1.53	3.08E-02	transcription_start_site	-	9256	9256	-988	18278 AN4252
1617	CONTIG69	9995	10494	1.53	3.08E-02	transcription_start_site	-	8926	8926	-1318	18279 AN4252
1617	CONTIG69	9995	10494	1.53	3.08E-02	transcription_start_site	-	8784	8784	-1460	18280 AN4252
2726	CONTIG69	11345	11852	1.11	1.44E-01	transcription_start_site	-	9256	9256	-2342	18278 AN4252
2726	CONTIG69	11345	11852	1.11	1.44E-01	transcription_start_site	-	8926	8926	-2672	18279 AN4252
2726	CONTIG69	11345	11852	1.11	1.44E-01	transcription_start_site	-	8784	8784	-2814	18280 AN4252
597	CONTIG69	15845	16196	2.31	9.51E-04	transcription_start_site	-	11387	11387	-4633	18281 AN4253
597	CONTIG69	15845	16196	2.31	9.51E-04	transcription_start_site	-	10981	10981	-5039	18282 AN4253
1617	CONTIG69	9995	10494	1.53	3.08E-02	transcription_start_site	-	10981	10981	736	18282 AN4253
1617	CONTIG69	9995	10494	1.53	3.08E-02	transcription_start_site	-	11387	11387	1142	18281 AN4253
2726	CONTIG69	11345	11852	1.11	1.44E-01	transcription_start_site	-	11387	11387	-211	18281 AN4253
2726	CONTIG69	11345	11852	1.11	1.44E-01	transcription_start_site	-	10981	10981	-617	18282 AN4253
877	CONTIG69	9310	9804	2.02	3.61E-03	transcription_start_site	+	12009	12009	-2452	18283 AN4254
877	CONTIG69	9310	9804	2.02	3.61E-03	transcription_start_site	+	12267	12267	-2710	18284 AN4254
877	CONTIG69	9310	9804	2.02	3.61E-03	transcription_start_site	+	12365	12365	-2808	18285 AN4254
1617	CONTIG69	9995	10494	1.53	3.08E-02	transcription_start_site	+	12009	12009	-1764	18283 AN4254
1617	CONTIG69	9995	10494	1.53	3.08E-02	transcription_start_site	+	12267	12267	-2022	18284 AN4254
1617	CONTIG69	9995	10494	1.53	3.08E-02	transcription_start_site	+	12365	12365	-2120	18285 AN4254
2726	CONTIG69	11345	11852	1.11	1.44E-01	transcription_start_site	+	12009	12009	-410	18283 AN4254
2726	CONTIG69	11345	11852	1.11	1.44E-01	transcription_start_site	+	12267	12267	-668	18284 AN4254
2726	CONTIG69	11345	11852	1.11	1.44E-01	transcription_start_site	+	12365	12365	-766	18285 AN4254
597	CONTIG69	15845	16196	2.31	9.51E-04	transcription_start_site	-	14686	14686	-1334	18286 AN4255
597	CONTIG69	15845	16196	2.31	9.51E-04	transcription_start_site	+	16953	16953	-932	18287 AN4256
597	CONTIG69	15845	16196	2.31	9.51E-04	transcription_start_site	+	17217	17217	-1196	18288 AN4256
597	CONTIG69	15845	16196	2.31	9.51E-04	transcription_start_site	+	17338	17338	-1317	18289 AN4256
597	CONTIG69	15845	16196	2.31	9.51E-04	transcription_start_site	+	20693	20693	-4672	18290 AN4257
597	CONTIG69	15845	16196	2.31	9.51E-04	transcription_start_site	+	20830	20830	-4809	18291 AN4257
597	CONTIG69	15845	16196	2.31	9.51E-04	transcription_start_site	+	21040	21040	-5019	18292 AN4257
2129	CONTIG69	23645	24144	1.32	5.05E-02	transcription_start_site	+	24068	24068	-173	18293 AN4258
2510	CONTIG69	25295	26169	1.2	9.26E-02	transcription_start_site	-	26044	26044	312	18295 AN4259
2510	CONTIG69	25295	26169	1.2	9.26E-02	transcription_start_site	-	26352	26352	620	18294 AN4259
2510	CONTIG69	25295	26169	1.2	9.26E-02	transcription_start_site	-	27022	27022	1290	18298 AN4260
2510	CONTIG69	25295	26169	1.2	9.26E-02	transcription_start_site	-	27154	27154	1422	18297 AN4260
2129	CONTIG69	23645	24144	1.32	5.05E-02	transcription_start_site	+	27857	27857	-3962	18299 AN4261
2510	CONTIG69	25295	26169	1.2	9.26E-02	transcription_start_site	+	27857	27857	-2125	18299 AN4261
2133	CONTIG69	45089	45358	1.32	6.84E-02	transcription_start_site	-	44910	44910	-313	18304 AN4265
2133	CONTIG69	45089	45358	1.32	6.84E-02	transcription_start_site	-	44810	44810	-413	18305 AN4265
2133	CONTIG69	45089	45358	1.32	6.84E-02	transcription_start_site	-	44137	44137	-1086	18306 AN4265
2133	CONTIG69	45089	45358	1.32	6.84E-02	transcription_start_site	+	45350	45350	-126	18307 AN4266
2133	CONTIG69	45089	45358	1.32	6.84E-02	transcription_start_site	+	45489	45489	-265	18308 AN4266
2133	CONTIG69	45089	45358	1.32	6.84E-02	transcription_start_site	+	45816	45816	-592	18309 AN4266
2632	CONTIG69	61511	61795	1.15	1.25E-01	transcription_start_site	+	61221	61221	432	18322 AN4270
2632	CONTIG69	61511	61795	1.15	1.25E-01	transcription_start_site	+	60617	60617	1036	18321 AN4270
1517	CONTIG69	68937	69295	1.57	2.62E-02	transcription_start_site	-	64832	64832	-4284	18323 AN4271

1517	CONTIG69	68937	69295	1.57	2.62E-02	transcription_start_site	-	64712	64712	-4404	18324	AN4271
1517	CONTIG69	68937	69295	1.57	2.62E-02	transcription_start_site	-	64128	64128	-4988	18325	AN4271
1517	CONTIG69	68937	69295	1.57	2.62E-02	transcription_start_site	-	64016	64016	-5100	18326	AN4271
1517	CONTIG69	68937	69295	1.57	2.62E-02	transcription_start_site	+	68531	68531	585	18337	AN4272
2632	CONTIG69	61511	61795	1.15	1.25E-01	transcription_start_site	+	65310	65310	-3657	18328	AN4272
2632	CONTIG69	61511	61795	1.15	1.25E-01	transcription_start_site	+	65445	65445	-3792	18329	AN4272
2632	CONTIG69	61511	61795	1.15	1.25E-01	transcription_start_site	+	66395	66395	-4742	18330	AN4272
2632	CONTIG69	61511	61795	1.15	1.25E-01	transcription_start_site	+	66776	66776	-5123	18331	AN4272
1517	CONTIG69	68937	69295	1.57	2.62E-02	transcription_start_site	+	69418	69418	-302	18338	AN4273
1517	CONTIG69	68937	69295	1.57	2.62E-02	transcription_start_site	+	70267	70267	-1151	18339	AN4273
1517	CONTIG69	68937	69295	1.57	2.62E-02	transcription_start_site	+	72442	72442	-3326	18340	AN4274
1517	CONTIG69	68937	69295	1.57	2.62E-02	transcription_start_site	+	72562	72562	-3446	18341	AN4274
1517	CONTIG69	68937	69295	1.57	2.62E-02	transcription_start_site	+	72757	72757	-3641	18342	AN4274
1208	CONTIG70	1126	2305	1.75	0.00E+00	transcription_start_site	+	1879	1879	-163	18351	AN4276
1208	CONTIG70	1126	2305	1.75	0.00E+00	transcription_start_site	+	1362	1362	353	18350	AN4276
1208	CONTIG70	1126	2305	1.75	0.00E+00	transcription_start_site	+	1077	1077	638	18349	AN4276
1208	CONTIG70	1126	2305	1.75	0.00E+00	transcription_start_site	+	1018	1018	697	18348	AN4276
1208	CONTIG70	1126	2305	1.75	0.00E+00	transcription_start_site	+	2466	2466	-750	18352	AN4276
1803	CONTIG70	5871	6154	1.45	1.11E-02	transcription_start_site	-	5735	5735	-277	18353	AN4277
1803	CONTIG70	5871	6154	1.45	1.11E-02	transcription_start_site	-	5357	5357	-655	18354	AN4277
1803	CONTIG70	5871	6154	1.45	1.11E-02	transcription_start_site	-	5107	5107	-905	18355	AN4277
1803	CONTIG70	5871	6154	1.45	1.11E-02	transcription_start_site	-	4981	4981	-1031	18356	AN4277
1803	CONTIG70	5871	6154	1.45	1.11E-02	transcription_start_site	-	4401	4401	-1611	18357	AN4277
1803	CONTIG70	5871	6154	1.45	1.11E-02	transcription_start_site	-	4060	4060	-1952	18358	AN4277
1803	CONTIG70	5871	6154	1.45	1.11E-02	transcription_start_site	-	3892	3892	-2120	18359	AN4277
2629	CONTIG70	16881	17156	1.15	5.22E-02	transcription_start_site	-	12678	12678	-4340	18360	AN4278
2629	CONTIG70	16881	17156	1.15	5.22E-02	transcription_start_site	-	12289	12289	-4729	18361	AN4278
2629	CONTIG70	16881	17156	1.15	5.22E-02	transcription_start_site	-	12051	12051	-4967	18362	AN4278
2629	CONTIG70	16881	17156	1.15	5.22E-02	transcription_start_site	-	16887	16887	-131	18377	AN4280
2629	CONTIG70	16881	17156	1.15	5.22E-02	transcription_start_site	-	16701	16701	-317	18378	AN4280
2629	CONTIG70	16881	17156	1.15	5.22E-02	transcription_start_site	-	15921	15921	-1097	18379	AN4280
3021	CONTIG70	36537	36807	0.88	1.66E-01	transcription_start_site	-	33624	33624	-3048	18410	AN4286
3021	CONTIG70	36537	36807	0.88	1.66E-01	transcription_start_site	-	33354	33354	-3318	18411	AN4286
3021	CONTIG70	36537	36807	0.88	1.66E-01	transcription_start_site	-	32989	32989	-3683	18412	AN4286
3021	CONTIG70	36537	36807	0.88	1.66E-01	transcription_start_site	-	32649	32649	-4023	18413	AN4286
3021	CONTIG70	36537	36807	0.88	1.66E-01	transcription_start_site	-	32464	32464	-4208	18414	AN4286
3021	CONTIG70	36537	36807	0.88	1.66E-01	transcription_start_site	-	32121	32121	-4551	18415	AN4286
3021	CONTIG70	36537	36807	0.88	1.66E-01	transcription_start_site	-	31945	31945	-4727	18416	AN4286
3021	CONTIG70	36537	36807	0.88	1.66E-01	transcription_start_site	-	31792	31792	-4880	18417	AN4286
3021	CONTIG70	36537	36807	0.88	1.66E-01	transcription_start_site	+	36958	36958	-286	18419	AN4288
3021	CONTIG70	36537	36807	0.88	1.66E-01	transcription_start_site	+	37133	37133	-461	18420	AN4288
3021	CONTIG70	36537	36807	0.88	1.66E-01	transcription_start_site	+	37213	37213	-541	18421	AN4288
3021	CONTIG70	36537	36807	0.88	1.66E-01	transcription_start_site	+	37325	37325	-653	18422	AN4288
3021	CONTIG70	36537	36807	0.88	1.66E-01	transcription_start_site	+	37406	37406	-734	18423	AN4288
3021	CONTIG70	36537	36807	0.88	1.66E-01	transcription_start_site	+	37488	37488	-816	18424	AN4288
3021	CONTIG70	36537	36807	0.88	1.66E-01	transcription_start_site	+	39421	39421	-2749	18425	AN4288
347	CONTIG71	2102	2455	2.69	1.57E-02	transcription_start_site	+	3865	3865	-1586	18426	AN4289
347	CONTIG71	2102	2455	2.69	1.57E-02	transcription_start_site	+	6306	6306	-4027	18427	AN4289
347	CONTIG71	2102	2455	2.69	1.57E-02	transcription_start_site	+	6976	6976	-4697	18428	AN4289
348	CONTIG72	9607	9892	2.68	0.00E+00	transcription_start_site	-	9347	9347	-402	18432	AN4291
348	CONTIG72	9607	9892	2.68	0.00E+00	transcription_start_site	-	8815	8815	-934	18433	AN4291
348	CONTIG72	9607	9892	2.68	0.00E+00	transcription_start_site	+	10351	10351	-601	18434	AN4292
348	CONTIG72	9607	9892	2.68	0.00E+00	transcription_start_site	+	10445	10445	-695	18435	AN4292
348	CONTIG72	9607	9892	2.68	0.00E+00	transcription_start_site	+	10665	10665	-915	18436	AN4292
2524	CONTIG73	2182	2461	1.19	5.88E-02	transcription_start_site	+	2672	2672	-350	18445	AN4295
2524	CONTIG73	2182	2461	1.19	5.88E-02	transcription_start_site	+	2877	2877	-555	18446	AN4295
2915	CONTIG74	1806	2076	0.99	9.24E-02	transcription_start_site	-	1206	1206	-735	18447	AN4296

2915	CONTIG74	1806	2076	0.99	9.24E-02	transcription_start_site	-	1116	1116	-825	18448 AN4296
2915	CONTIG74	1806	2076	0.99	9.24E-02	transcription_start_site	-	774	774	-1167	18449 AN4296
2915	CONTIG74	1806	2076	0.99	9.24E-02	transcription_start_site	+	2144	2144	-203	18450 AN4297
2915	CONTIG74	1806	2076	0.99	9.24E-02	transcription_start_site	+	2239	2239	-298	18451 AN4297
2915	CONTIG74	1806	2076	0.99	9.24E-02	transcription_start_site	+	2476	2476	-535	18452 AN4297
2915	CONTIG74	1806	2076	0.99	9.24E-02	transcription_start_site	+	2887	2887	-946	18453 AN4297
2915	CONTIG74	1806	2076	0.99	9.24E-02	transcription_start_site	+	3575	3575	-1634	18454 AN4298
2429	CONTIG74	6610	7329	1.22	3.08E-02	transcription_start_site	+	7256	7256	-286	18455 AN4299
2429	CONTIG74	6610	7329	1.22	3.08E-02	transcription_start_site	+	7321	7321	-351	18456 AN4299
2429	CONTIG74	6610	7329	1.22	3.08E-02	transcription_start_site	+	7676	7676	-706	18457 AN4299
2860	CONTIG74	8570	8836	1.03	8.01E-02	transcription_start_site	+	7676	7676	1027	18457 AN4299
2429	CONTIG74	6610	7329	1.22	3.08E-02	transcription_start_site	-	8252	8252	1282	18460 AN4300
2860	CONTIG74	8570	8836	1.03	8.01E-02	transcription_start_site	-	8802	8802	99	18459 AN4300
2860	CONTIG74	8570	8836	1.03	8.01E-02	transcription_start_site	-	8911	8911	208	18458 AN4300
2860	CONTIG74	8570	8836	1.03	8.01E-02	transcription_start_site	-	8252	8252	-451	18460 AN4300
2860	CONTIG74	8570	8836	1.03	8.01E-02	transcription_start_site	-	9823	9823	1120	18462 AN4301
80	CONTIG75	22738	23077	3.3	0.00E+00	transcription_start_site	-	18043	18043	-4864	18483 AN4310
80	CONTIG75	22738	23077	3.3	0.00E+00	transcription_start_site	-	17885	17885	-5022	18484 AN4310
80	CONTIG75	22738	23077	3.3	0.00E+00	transcription_start_site	-	21521	21521	-1386	18489 AN4311
937	CONTIG75	23942	24366	1.97	9.21E-03	transcription_start_site	-	21521	21521	-2633	18489 AN4311
80	CONTIG75	22738	23077	3.3	0.00E+00	transcription_start_site	-	23774	23774	866	18492 AN4312
937	CONTIG75	23942	24366	1.97	9.21E-03	transcription_start_site	-	23774	23774	-380	18492 AN4312
80	CONTIG75	22738	23077	3.3	0.00E+00	transcription_start_site	+	28036	28036	-5128	18493 AN4313
937	CONTIG75	23942	24366	1.97	9.21E-03	transcription_start_site	+	28036	28036	-3882	18493 AN4313
937	CONTIG75	23942	24366	1.97	9.21E-03	transcription_start_site	+	28540	28540	-4386	18494 AN4313
106	CONTIG75	35703	35993	3.2	0.00E+00	transcription_start_site	-	34069	34069	-1779	18497 AN4314
106	CONTIG75	35703	35993	3.2	0.00E+00	transcription_start_site	-	33880	33880	-1968	18498 AN4314
2274	CONTIG75	33318	33652	1.28	1.66E-01	transcription_start_site	-	33880	33880	395	18498 AN4314
2274	CONTIG75	33318	33652	1.28	1.66E-01	transcription_start_site	-	34069	34069	584	18497 AN4314
106	CONTIG75	35703	35993	3.2	0.00E+00	transcription_start_site	+	37528	37528	-1680	18499 AN4315
106	CONTIG75	35703	35993	3.2	0.00E+00	transcription_start_site	+	37645	37645	-1797	18500 AN4315
106	CONTIG75	35703	35993	3.2	0.00E+00	transcription_start_site	+	38416	38416	-2568	18501 AN4315
106	CONTIG75	35703	35993	3.2	0.00E+00	transcription_start_site	+	38544	38544	-2696	18502 AN4315
106	CONTIG75	35703	35993	3.2	0.00E+00	transcription_start_site	+	38894	38894	-3046	18503 AN4315
106	CONTIG75	35703	35993	3.2	0.00E+00	transcription_start_site	+	39119	39119	-3271	18504 AN4315
106	CONTIG75	35703	35993	3.2	0.00E+00	transcription_start_site	+	39258	39258	-3410	18505 AN4315
106	CONTIG75	35703	35993	3.2	0.00E+00	transcription_start_site	+	39772	39772	-3924	18506 AN4315
2274	CONTIG75	33318	33652	1.28	1.66E-01	transcription_start_site	+	37528	37528	-4043	18499 AN4315
2274	CONTIG75	33318	33652	1.28	1.66E-01	transcription_start_site	+	37645	37645	-4160	18500 AN4315
2274	CONTIG75	33318	33652	1.28	1.66E-01	transcription_start_site	+	38416	38416	-4931	18501 AN4315
2274	CONTIG75	33318	33652	1.28	1.66E-01	transcription_start_site	+	38544	38544	-5059	18502 AN4315
367	CONTIG75	68183	68615	2.66	1.22E-03	transcription_start_site	+	69057	69057	-658	18533 AN4324
367	CONTIG75	68183	68615	2.66	1.22E-03	transcription_start_site	+	69264	69264	-865	18534 AN4324
681	CONTIG75	69471	69740	2.22	7.85E-03	transcription_start_site	+	69264	69264	341	18534 AN4324
681	CONTIG75	69471	69740	2.22	7.85E-03	transcription_start_site	+	69057	69057	548	18533 AN4324
595	CONTIG75	70811	71375	2.32	5.20E-03	transcription_start_site	-	72219	72219	1126	18539 AN4325
595	CONTIG75	70811	71375	2.32	5.20E-03	transcription_start_site	-	72328	72328	1235	18538 AN4325
1408	CONTIG75	85744	86022	1.63	5.88E-02	transcription_start_site	-	84276	84276	-1607	18540 AN4328
1408	CONTIG75	85744	86022	1.63	5.88E-02	transcription_start_site	-	83535	83535	-2348	18541 AN4328
1408	CONTIG75	85744	86022	1.63	5.88E-02	transcription_start_site	-	83320	83320	-2563	18542 AN4328
1408	CONTIG75	85744	86022	1.63	5.88E-02	transcription_start_site	-	82410	82410	-3473	18543 AN4328
1408	CONTIG75	85744	86022	1.63	5.88E-02	transcription_start_site	-	81985	81985	-3898	18544 AN4328
1408	CONTIG75	85744	86022	1.63	5.88E-02	transcription_start_site	+	86086	86086	-203	18545 AN4329
1408	CONTIG75	85744	86022	1.63	5.88E-02	transcription_start_site	+	86298	86298	-415	18546 AN4329
1408	CONTIG75	85744	86022	1.63	5.88E-02	transcription_start_site	+	86479	86479	-596	18547 AN4329
941	CONTIG75	93680	93969	1.97	1.84E-02	transcription_start_site	-	89160	89160	-4664	18548 AN4330
1408	CONTIG75	85744	86022	1.63	5.88E-02	transcription_start_site	+	89332	89332	-3449	18549 AN4331

941	CONTIG75	93680	93969	1.97	1.84E-02	transcription_start_site	-	92937	92937	-887	18550	AN4332
941	CONTIG75	93680	93969	1.97	1.84E-02	transcription_start_site	-	92718	92718	-1106	18551	AN4332
941	CONTIG75	93680	93969	1.97	1.84E-02	transcription_start_site	-	92358	92358	-1466	18552	AN4332
941	CONTIG75	93680	93969	1.97	1.84E-02	transcription_start_site	-	92020	92020	-1804	18553	AN4332
941	CONTIG75	93680	93969	1.97	1.84E-02	transcription_start_site	-	94819	94819	994	18555	AN4333
941	CONTIG75	93680	93969	1.97	1.84E-02	transcription_start_site	-	94879	94879	1054	18554	AN4333
1974	CONTIG75	98405	100939	1.38	3.64E-02	transcription_start_site	-	94879	94879	-4793	18554	AN4333
1974	CONTIG75	98405	100939	1.38	3.64E-02	transcription_start_site	-	94819	94819	-4853	18555	AN4333
1974	CONTIG75	98405	100939	1.38	3.64E-02	transcription_start_site	-	101493	101493	1821	18557	AN4335
1974	CONTIG75	98405	100939	1.38	3.64E-02	transcription_start_site	+	104205	104205	-4533	18558	AN4336
1974	CONTIG75	98405	100939	1.38	3.64E-02	transcription_start_site	+	104606	104606	-4934	18559	AN4336
1974	CONTIG75	98405	100939	1.38	3.64E-02	transcription_start_site	+	105132	105132	-5460	18560	AN4336
1974	CONTIG75	98405	100939	1.38	3.64E-02	transcription_start_site	+	105461	105461	-5789	18561	AN4336
1974	CONTIG75	98405	100939	1.38	3.64E-02	transcription_start_site	+	105617	105617	-5945	18562	AN4336
1409	CONTIG75	114526	114815	1.63	5.88E-02	transcription_start_site	-	109578	109578	-5092	18569	AN4338
1409	CONTIG75	114526	114815	1.63	5.88E-02	transcription_start_site	+	114880	114880	-209	18582	AN4340
1409	CONTIG75	114526	114815	1.63	5.88E-02	transcription_start_site	+	115022	115022	-351	18583	AN4340
1409	CONTIG75	114526	114815	1.63	5.88E-02	transcription_start_site	+	115265	115265	-594	18584	AN4340
1409	CONTIG75	114526	114815	1.63	5.88E-02	transcription_start_site	+	117228	117228	-2557	18586	AN4342
1409	CONTIG75	114526	114815	1.63	5.88E-02	transcription_start_site	+	117369	117369	-2698	18587	AN4342
1409	CONTIG75	114526	114815	1.63	5.88E-02	transcription_start_site	+	117634	117634	-2963	18588	AN4342
1409	CONTIG75	114526	114815	1.63	5.88E-02	transcription_start_site	+	119673	119673	-5002	18589	AN4343
824	CONTIG75	133439	133793	2.07	5.41E-03	transcription_start_site	-	129290	129290	-4326	18607	AN4346
824	CONTIG75	133439	133793	2.07	5.41E-03	transcription_start_site	-	129208	129208	-4408	18608	AN4346
824	CONTIG75	133439	133793	2.07	5.41E-03	transcription_start_site	-	128548	128548	-5068	18609	AN4346
1410	CONTIG75	134044	134918	1.63	5.88E-02	transcription_start_site	-	129290	129290	-5191	18607	AN4346
1410	CONTIG75	134044	134918	1.63	5.88E-02	transcription_start_site	-	129208	129208	-5273	18608	AN4346
824	CONTIG75	133439	133793	2.07	5.41E-03	transcription_start_site	-	132487	132487	-1129	18616	AN4348
1410	CONTIG75	134044	134918	1.63	5.88E-02	transcription_start_site	-	132487	132487	-1994	18616	AN4348
824	CONTIG75	133439	133793	2.07	5.41E-03	transcription_start_site	+	134902	134902	-1286	18617	AN4349
824	CONTIG75	133439	133793	2.07	5.41E-03	transcription_start_site	+	135625	135625	-2009	18618	AN4349
824	CONTIG75	133439	133793	2.07	5.41E-03	transcription_start_site	+	137988	137988	-4372	18619	AN4349
824	CONTIG75	133439	133793	2.07	5.41E-03	transcription_start_site	+	138292	138292	-4676	18620	AN4349
1410	CONTIG75	134044	134918	1.63	5.88E-02	transcription_start_site	+	134902	134902	-421	18617	AN4349
1410	CONTIG75	134044	134918	1.63	5.88E-02	transcription_start_site	+	135625	135625	-1144	18618	AN4349
1410	CONTIG75	134044	134918	1.63	5.88E-02	transcription_start_site	+	137988	137988	-3507	18619	AN4349
1410	CONTIG75	134044	134918	1.63	5.88E-02	transcription_start_site	+	138292	138292	-3811	18620	AN4349
727	CONTIG75	152115	153209	2.17	0.00E+00	transcription_start_site	-	150145	150145	-2517	18621	AN4351
727	CONTIG75	152115	153209	2.17	0.00E+00	transcription_start_site	-	150088	150088	-2574	18622	AN4351
727	CONTIG75	152115	153209	2.17	0.00E+00	transcription_start_site	-	149800	149800	-2862	18623	AN4351
727	CONTIG75	152115	153209	2.17	0.00E+00	transcription_start_site	-	149693	149693	-2969	18624	AN4351
727	CONTIG75	152115	153209	2.17	0.00E+00	transcription_start_site	-	148753	148753	-3909	18625	AN4351
727	CONTIG75	152115	153209	2.17	0.00E+00	transcription_start_site	-	147725	147725	-4937	18626	AN4351
2275	CONTIG75	148430	148934	1.28	1.66E-01	transcription_start_site	-	148753	148753	71	18625	AN4351
2275	CONTIG75	148430	148934	1.28	1.66E-01	transcription_start_site	-	147725	147725	-957	18626	AN4351
2275	CONTIG75	148430	148934	1.28	1.66E-01	transcription_start_site	-	149693	149693	1011	18624	AN4351
2275	CONTIG75	148430	148934	1.28	1.66E-01	transcription_start_site	-	149800	149800	1118	18623	AN4351
727	CONTIG75	152115	153209	2.17	0.00E+00	transcription_start_site	-	151680	151680	-982	18627	AN4352
727	CONTIG75	152115	153209	2.17	0.00E+00	transcription_start_site	-	151443	151443	-1219	18628	AN4352
727	CONTIG75	152115	153209	2.17	0.00E+00	transcription_start_site	-	151338	151338	-1324	18629	AN4352
727	CONTIG75	152115	153209	2.17	0.00E+00	transcription_start_site	-	150991	150991	-1671	18630	AN4352
727	CONTIG75	152115	153209	2.17	0.00E+00	transcription_start_site	-	150592	150592	-2070	18631	AN4352
727	CONTIG75	152115	153209	2.17	0.00E+00	transcription_start_site	+	153479	153479	-817	18632	AN4353
727	CONTIG75	152115	153209	2.17	0.00E+00	transcription_start_site	+	154022	154022	-1360	18633	AN4353
2275	CONTIG75	148430	148934	1.28	1.66E-01	transcription_start_site	+	153479	153479	-4797	18632	AN4353
727	CONTIG75	152115	153209	2.17	0.00E+00	transcription_start_site	+	156103	156103	-3441	18634	AN4354
727	CONTIG75	152115	153209	2.17	0.00E+00	transcription_start_site	+	156290	156290	-3628	18635	AN4354

727	CONTIG75	152115	153209	2.17	0.00E+00	transcription_start_site	+	156814	156814	-4152	18636	AN4354
780	CONTIG75	165320	165894	2.12	1.11E-02	transcription_start_site	-	165344	165344	-263	18644	AN4357
780	CONTIG75	165320	165894	2.12	1.11E-02	transcription_start_site	-	165140	165140	-467	18645	AN4357
780	CONTIG75	165320	165894	2.12	1.11E-02	transcription_start_site	-	164401	164401	-1206	18646	AN4357
780	CONTIG75	165320	165894	2.12	1.11E-02	transcription_start_site	-	164246	164246	-1361	18647	AN4357
44	CONTIG75	173930	174354	3.45	0.00E+00	transcription_start_site	-	169295	169295	-4847	18648	AN4358
44	CONTIG75	173930	174354	3.45	0.00E+00	transcription_start_site	-	169189	169189	-4953	18649	AN4358
780	CONTIG75	165320	165894	2.12	1.11E-02	transcription_start_site	+	169738	169738	-4131	18652	AN4359
780	CONTIG75	165320	165894	2.12	1.11E-02	transcription_start_site	+	170094	170094	-4487	18653	AN4359
780	CONTIG75	165320	165894	2.12	1.11E-02	transcription_start_site	+	170366	170366	-4759	18654	AN4359
780	CONTIG75	165320	165894	2.12	1.11E-02	transcription_start_site	+	170476	170476	-4869	18655	AN4359
780	CONTIG75	165320	165894	2.12	1.11E-02	transcription_start_site	+	170770	170770	-5163	18656	AN4359
44	CONTIG75	173930	174354	3.45	0.00E+00	transcription_start_site	-	173101	173101	-1041	18658	AN4360
831	CONTIG75	176405	176681	2.07	1.30E-02	transcription_start_site	-	173101	173101	-3442	18658	AN4360
2421	CONTIG75	175440	176314	1.23	1.79E-01	transcription_start_site	-	173101	173101	-2776	18658	AN4360
44	CONTIG75	173930	174354	3.45	0.00E+00	transcription_start_site	+	174673	174673	-531	18659	AN4361
44	CONTIG75	173930	174354	3.45	0.00E+00	transcription_start_site	+	175427	175427	-1285	18660	AN4361
831	CONTIG75	176405	176681	2.07	1.30E-02	transcription_start_site	+	175427	175427	1116	18660	AN4361
2421	CONTIG75	175440	176314	1.23	1.79E-01	transcription_start_site	+	175427	175427	450	18660	AN4361
2421	CONTIG75	175440	176314	1.23	1.79E-01	transcription_start_site	+	174673	174673	1204	18659	AN4361
831	CONTIG75	176405	176681	2.07	1.30E-02	transcription_start_site	-	176334	176334	-209	18663	AN4362
831	CONTIG75	176405	176681	2.07	1.30E-02	transcription_start_site	-	177424	177424	881	18662	AN4362
831	CONTIG75	176405	176681	2.07	1.30E-02	transcription_start_site	-	177606	177606	1063	18661	AN4362
2421	CONTIG75	175440	176314	1.23	1.79E-01	transcription_start_site	-	176334	176334	457	18663	AN4362
1320	CONTIG75	183232	183726	1.68	5.22E-02	transcription_start_site	-	181272	181272	-2207	18664	AN4363
1320	CONTIG75	183232	183726	1.68	5.22E-02	transcription_start_site	-	180935	180935	-2544	18665	AN4363
1320	CONTIG75	183232	183726	1.68	5.22E-02	transcription_start_site	-	183954	183954	475	18667	AN4364
1320	CONTIG75	183232	183726	1.68	5.22E-02	transcription_start_site	-	184039	184039	560	18666	AN4364
1320	CONTIG75	183232	183726	1.68	5.22E-02	transcription_start_site	+	184964	184964	-1485	18668	AN4365
1320	CONTIG75	183232	183726	1.68	5.22E-02	transcription_start_site	+	185259	185259	-1780	18669	AN4365
1629	CONTIG75	210238	210502	1.53	8.01E-02	transcription_start_site	-	206634	206634	-3736	18681	AN4368
1629	CONTIG75	210238	210502	1.53	8.01E-02	transcription_start_site	-	206527	206527	-3843	18682	AN4368
2126	CONTIG75	206783	207060	1.33	1.44E-01	transcription_start_site	-	206634	206634	-287	18681	AN4368
2126	CONTIG75	206783	207060	1.33	1.44E-01	transcription_start_site	-	206527	206527	-394	18682	AN4368
2123	CONTIG75	204168	204503	1.33	1.28E-01	transcription_start_site	+	208698	208698	-4362	18683	AN4369
2126	CONTIG75	206783	207060	1.33	1.44E-01	transcription_start_site	+	208698	208698	-1776	18683	AN4369
2367	CONTIG76	15528	16028	1.25	1.44E-01	transcription_start_site	-	10550	10550	-5228	18690	AN4373
562	CONTIG76	18469	19343	2.36	0.00E+00	transcription_start_site	-	14013	14013	-4893	18692	AN4374
562	CONTIG76	18469	19343	2.36	0.00E+00	transcription_start_site	-	13903	13903	-5003	18693	AN4374
562	CONTIG76	18469	19343	2.36	0.00E+00	transcription_start_site	-	13471	13471	-5435	18694	AN4374
2367	CONTIG76	15528	16028	1.25	1.44E-01	transcription_start_site	-	14013	14013	-1765	18692	AN4374
2367	CONTIG76	15528	16028	1.25	1.44E-01	transcription_start_site	-	13903	13903	-1875	18693	AN4374
2367	CONTIG76	15528	16028	1.25	1.44E-01	transcription_start_site	-	13471	13471	-2307	18694	AN4374
2367	CONTIG76	15528	16028	1.25	1.44E-01	transcription_start_site	-	12953	12953	-2825	18695	AN4374
562	CONTIG76	18469	19343	2.36	0.00E+00	transcription_start_site	-	17469	17469	-1437	18696	AN4375
562	CONTIG76	18469	19343	2.36	0.00E+00	transcription_start_site	-	17320	17320	-1586	18697	AN4375
674	CONTIG76	20569	20913	2.22	4.61E-03	transcription_start_site	-	17469	17469	-3272	18696	AN4375
674	CONTIG76	20569	20913	2.22	4.61E-03	transcription_start_site	-	17320	17320	-3421	18697	AN4375
1757	CONTIG76	19589	20233	1.48	6.84E-02	transcription_start_site	-	17469	17469	-2442	18696	AN4375
1757	CONTIG76	19589	20233	1.48	6.84E-02	transcription_start_site	-	17320	17320	-2591	18697	AN4375
82	CONTIG76	22894	23153	3.29	0.00E+00	transcription_start_site	+	23350	23350	-326	18698	AN4376
82	CONTIG76	22894	23153	3.29	0.00E+00	transcription_start_site	+	23449	23449	-425	18699	AN4376
82	CONTIG76	22894	23153	3.29	0.00E+00	transcription_start_site	+	23778	23778	-754	18700	AN4376
562	CONTIG76	18469	19343	2.36	0.00E+00	transcription_start_site	+	23350	23350	-4444	18698	AN4376
562	CONTIG76	18469	19343	2.36	0.00E+00	transcription_start_site	+	23449	23449	-4543	18699	AN4376
562	CONTIG76	18469	19343	2.36	0.00E+00	transcription_start_site	+	23778	23778	-4872	18700	AN4376
674	CONTIG76	20569	20913	2.22	4.61E-03	transcription_start_site	+	23350	23350	-2609	18698	AN4376

674	CONTIG76	20569	20913	2.22	4.61E-03	transcription_start_site	+	23449	23449	-2708	18699 AN4376
674	CONTIG76	20569	20913	2.22	4.61E-03	transcription_start_site	+	23778	23778	-3037	18700 AN4376
1510	CONTIG76	24019	24588	1.58	5.22E-02	transcription_start_site	+	23778	23778	525	18700 AN4376
1510	CONTIG76	24019	24588	1.58	5.22E-02	transcription_start_site	+	23449	23449	854	18699 AN4376
1510	CONTIG76	24019	24588	1.58	5.22E-02	transcription_start_site	+	23350	23350	953	18698 AN4376
1757	CONTIG76	19589	20233	1.48	6.84E-02	transcription_start_site	+	23350	23350	-3439	18698 AN4376
1757	CONTIG76	19589	20233	1.48	6.84E-02	transcription_start_site	+	23449	23449	-3538	18699 AN4376
1757	CONTIG76	19589	20233	1.48	6.84E-02	transcription_start_site	+	23778	23778	-3867	18700 AN4376
82	CONTIG76	22894	23153	3.29	0.00E+00	transcription_start_site	+	25586	25586	-2562	18701 AN4377
82	CONTIG76	22894	23153	3.29	0.00E+00	transcription_start_site	+	26045	26045	-3021	18702 AN4377
82	CONTIG76	22894	23153	3.29	0.00E+00	transcription_start_site	+	26336	26336	-3312	18703 AN4377
82	CONTIG76	22894	23153	3.29	0.00E+00	transcription_start_site	+	26445	26445	-3421	18704 AN4377
82	CONTIG76	22894	23153	3.29	0.00E+00	transcription_start_site	+	26996	26996	-3972	18705 AN4377
82	CONTIG76	22894	23153	3.29	0.00E+00	transcription_start_site	+	27577	27577	-4553	18706 AN4377
674	CONTIG76	20569	20913	2.22	4.61E-03	transcription_start_site	+	25586	25586	-4845	18701 AN4377
1510	CONTIG76	24019	24588	1.58	5.22E-02	transcription_start_site	+	25586	25586	-1282	18701 AN4377
1510	CONTIG76	24019	24588	1.58	5.22E-02	transcription_start_site	+	26045	26045	-1741	18702 AN4377
1510	CONTIG76	24019	24588	1.58	5.22E-02	transcription_start_site	+	26336	26336	-2032	18703 AN4377
1510	CONTIG76	24019	24588	1.58	5.22E-02	transcription_start_site	+	26445	26445	-2141	18704 AN4377
1510	CONTIG76	24019	24588	1.58	5.22E-02	transcription_start_site	+	26996	26996	-2692	18705 AN4377
1510	CONTIG76	24019	24588	1.58	5.22E-02	transcription_start_site	+	27577	27577	-3273	18706 AN4377
860	CONTIG76	33389	35548	2.04	0.00E+00	transcription_start_site	-	33154	33154	-1314	18707 AN4378
860	CONTIG76	33389	35548	2.04	0.00E+00	transcription_start_site	-	32949	32949	-1519	18708 AN4378
860	CONTIG76	33389	35548	2.04	0.00E+00	transcription_start_site	-	32730	32730	-1738	18709 AN4378
860	CONTIG76	33389	35548	2.04	0.00E+00	transcription_start_site	-	32234	32234	-2234	18710 AN4378
860	CONTIG76	33389	35548	2.04	0.00E+00	transcription_start_site	-	35035	35035	566	18711 AN4379
718	CONTIG76	63613	63952	2.18	5.20E-03	transcription_start_site	-	61734	61734	-2048	18743 AN4389
718	CONTIG76	63613	63952	2.18	5.20E-03	transcription_start_site	-	60602	60602	-3180	18744 AN4389
718	CONTIG76	63613	63952	2.18	5.20E-03	transcription_start_site	-	60541	60541	-3241	18745 AN4389
718	CONTIG76	63613	63952	2.18	5.20E-03	transcription_start_site	-	60422	60422	-3360	18746 AN4389
718	CONTIG76	63613	63952	2.18	5.20E-03	transcription_start_site	+	65116	65116	-1333	18747 AN4390
718	CONTIG76	63613	63952	2.18	5.20E-03	transcription_start_site	+	65239	65239	-1456	18748 AN4390
718	CONTIG76	63613	63952	2.18	5.20E-03	transcription_start_site	+	67144	67144	-3361	18749 AN4391
2083	CONTIG76	80565	80839	1.34	1.08E-01	transcription_start_site	+	79772	79772	930	18764 AN4395
2083	CONTIG76	80565	80839	1.34	1.08E-01	transcription_start_site	+	80916	80916	-214	18765 AN4396
2083	CONTIG76	80565	80839	1.34	1.08E-01	transcription_start_site	+	81052	81052	-350	18766 AN4396
2083	CONTIG76	80565	80839	1.34	1.08E-01	transcription_start_site	+	81227	81227	-525	18767 AN4396
2083	CONTIG76	80565	80839	1.34	1.08E-01	transcription_start_site	+	81487	81487	-785	18768 AN4396
2083	CONTIG76	80565	80839	1.34	1.08E-01	transcription_start_site	-	81718	81718	1016	18772 AN4397
2083	CONTIG76	80565	80839	1.34	1.08E-01	transcription_start_site	+	83692	83692	-2990	18773 AN4398
2083	CONTIG76	80565	80839	1.34	1.08E-01	transcription_start_site	+	83940	83940	-3238	18774 AN4398
2083	CONTIG76	80565	80839	1.34	1.08E-01	transcription_start_site	+	84404	84404	-3702	18775 AN4398
2083	CONTIG76	80565	80839	1.34	1.08E-01	transcription_start_site	+	85242	85242	-4540	18776 AN4398
2083	CONTIG76	80565	80839	1.34	1.08E-01	transcription_start_site	+	85406	85406	-4704	18777 AN4398
1951	CONTIG76	91517	91801	1.39	9.24E-02	transcription_start_site	-	88510	88510	-3149	18782 AN4400
1951	CONTIG76	91517	91801	1.39	9.24E-02	transcription_start_site	-	88295	88295	-3364	18783 AN4400
1951	CONTIG76	91517	91801	1.39	9.24E-02	transcription_start_site	-	88165	88165	-3494	18784 AN4400
1951	CONTIG76	91517	91801	1.39	9.24E-02	transcription_start_site	+	91065	91065	594	18793 AN4401
1951	CONTIG76	91517	91801	1.39	9.24E-02	transcription_start_site	+	90901	90901	758	18792 AN4401
1951	CONTIG76	91517	91801	1.39	9.24E-02	transcription_start_site	+	91820	91820	-161	18794 AN4402
1951	CONTIG76	91517	91801	1.39	9.24E-02	transcription_start_site	+	91971	91971	-312	18795 AN4402
1951	CONTIG76	91517	91801	1.39	9.24E-02	transcription_start_site	+	92080	92080	-421	18796 AN4402
1951	CONTIG76	91517	91801	1.39	9.24E-02	transcription_start_site	+	92231	92231	-572	18797 AN4402
1951	CONTIG76	91517	91801	1.39	9.24E-02	transcription_start_site	+	92424	92424	-765	18798 AN4402
1951	CONTIG76	91517	91801	1.39	9.24E-02	transcription_start_site	+	92990	92990	-1331	18799 AN4402
1951	CONTIG76	91517	91801	1.39	9.24E-02	transcription_start_site	+	95152	95152	-3493	18803 AN4404
1951	CONTIG76	91517	91801	1.39	9.24E-02	transcription_start_site	+	95248	95248	-3589	18804 AN4404

1951	CONTIG76	91517	91801	1.39	9.24E-02	transcription_start_site	+	95457	95457	-3798	18805	AN4404
1951	CONTIG76	91517	91801	1.39	9.24E-02	transcription_start_site	+	96095	96095	-4436	18806	AN4404
1951	CONTIG76	91517	91801	1.39	9.24E-02	transcription_start_site	+	96417	96417	-4758	18807	AN4404
1951	CONTIG76	91517	91801	1.39	9.24E-02	transcription_start_site	+	96628	96628	-4969	18808	AN4404
1951	CONTIG76	91517	91801	1.39	9.24E-02	transcription_start_site	+	96765	96765	-5106	18809	AN4404
2200	CONTIG76	100803	101101	1.3	1.25E-01	transcription_start_site	-	100615	100615	-337	18813	AN4406
2200	CONTIG76	100803	101101	1.3	1.25E-01	transcription_start_site	-	100514	100514	-438	18814	AN4406
2200	CONTIG76	100803	101101	1.3	1.25E-01	transcription_start_site	-	100381	100381	-571	18815	AN4406
2200	CONTIG76	100803	101101	1.3	1.25E-01	transcription_start_site	-	100304	100304	-648	18816	AN4406
2200	CONTIG76	100803	101101	1.3	1.25E-01	transcription_start_site	-	100188	100188	-764	18817	AN4406
2200	CONTIG76	100803	101101	1.3	1.25E-01	transcription_start_site	-	100024	100024	-928	18818	AN4406
2200	CONTIG76	100803	101101	1.3	1.25E-01	transcription_start_site	-	99853	99853	-1099	18819	AN4406
2200	CONTIG76	100803	101101	1.3	1.25E-01	transcription_start_site	-	98991	98991	-1961	18820	AN4406
2200	CONTIG76	100803	101101	1.3	1.25E-01	transcription_start_site	+	103434	103434	-2482	18821	AN4407
2200	CONTIG76	100803	101101	1.3	1.25E-01	transcription_start_site	+	103779	103779	-2827	18822	AN4407
2200	CONTIG76	100803	101101	1.3	1.25E-01	transcription_start_site	+	103930	103930	-2978	18823	AN4407
2200	CONTIG76	100803	101101	1.3	1.25E-01	transcription_start_site	+	104161	104161	-3209	18824	AN4407
2200	CONTIG76	100803	101101	1.3	1.25E-01	transcription_start_site	+	104386	104386	-3434	18825	AN4407
959	CONTIG76	109054	109853	1.95	1.30E-02	transcription_start_site	-	108902	108902	-551	18826	AN4408
959	CONTIG76	109054	109853	1.95	1.30E-02	transcription_start_site	-	108055	108055	-1398	18827	AN4408
959	CONTIG76	109054	109853	1.95	1.30E-02	transcription_start_site	-	107051	107051	-2402	18828	AN4408
959	CONTIG76	109054	109853	1.95	1.30E-02	transcription_start_site	-	106318	106318	-3135	18829	AN4408
959	CONTIG76	109054	109853	1.95	1.30E-02	transcription_start_site	+	109243	109243	210	18830	AN4409
959	CONTIG76	109054	109853	1.95	1.30E-02	transcription_start_site	+	111323	111323	-1869	18831	AN4410
1758	CONTIG76	129392	129736	1.48	6.84E-02	transcription_start_site	-	125276	125276	-4288	18839	AN4414
1758	CONTIG76	129392	129736	1.48	6.84E-02	transcription_start_site	-	125152	125152	-4412	18840	AN4414
1758	CONTIG76	129392	129736	1.48	6.84E-02	transcription_start_site	-	127622	127622	-1942	18842	AN4416
1758	CONTIG76	129392	129736	1.48	6.84E-02	transcription_start_site	-	127259	127259	-2305	18843	AN4416
1758	CONTIG76	129392	129736	1.48	6.84E-02	transcription_start_site	+	128535	128535	1029	18846	AN4417
1011	CONTIG76	136208	136643	1.9	1.57E-02	transcription_start_site	-	133098	133098	-3327	18847	AN4418
1011	CONTIG76	136208	136643	1.9	1.57E-02	transcription_start_site	-	132659	132659	-3766	18848	AN4418
1011	CONTIG76	136208	136643	1.9	1.57E-02	transcription_start_site	-	131453	131453	-4972	18849	AN4418
1072	CONTIG76	134185	135442	1.85	1.00E-02	transcription_start_site	-	133098	133098	-1715	18847	AN4418
1072	CONTIG76	134185	135442	1.85	1.00E-02	transcription_start_site	-	132659	132659	-2154	18848	AN4418
1072	CONTIG76	134185	135442	1.85	1.00E-02	transcription_start_site	-	131453	131453	-3360	18849	AN4418
1952	CONTIG76	135463	136117	1.39	9.24E-02	transcription_start_site	-	133098	133098	-2692	18847	AN4418
1952	CONTIG76	135463	136117	1.39	9.24E-02	transcription_start_site	-	132659	132659	-3131	18848	AN4418
1952	CONTIG76	135463	136117	1.39	9.24E-02	transcription_start_site	-	131453	131453	-4337	18849	AN4418
2084	CONTIG76	137859	138138	1.34	1.08E-01	transcription_start_site	-	133098	133098	-4900	18847	AN4418
960	CONTIG76	140404	140678	1.95	1.30E-02	transcription_start_site	+	139584	139584	957	18854	AN4419
1011	CONTIG76	136208	136643	1.9	1.57E-02	transcription_start_site	+	138291	138291	-1865	18850	AN4419
1011	CONTIG76	136208	136643	1.9	1.57E-02	transcription_start_site	+	138396	138396	-1970	18851	AN4419
1011	CONTIG76	136208	136643	1.9	1.57E-02	transcription_start_site	+	138696	138696	-2270	18852	AN4419
1011	CONTIG76	136208	136643	1.9	1.57E-02	transcription_start_site	+	139113	139113	-2687	18853	AN4419
1011	CONTIG76	136208	136643	1.9	1.57E-02	transcription_start_site	+	139584	139584	-3158	18854	AN4419
1072	CONTIG76	134185	135442	1.85	1.00E-02	transcription_start_site	+	138291	138291	-3477	18850	AN4419
1072	CONTIG76	134185	135442	1.85	1.00E-02	transcription_start_site	+	138396	138396	-3582	18851	AN4419
1072	CONTIG76	134185	135442	1.85	1.00E-02	transcription_start_site	+	138696	138696	-3882	18852	AN4419
1072	CONTIG76	134185	135442	1.85	1.00E-02	transcription_start_site	+	139113	139113	-4299	18853	AN4419
1072	CONTIG76	134185	135442	1.85	1.00E-02	transcription_start_site	+	139584	139584	-4770	18854	AN4419
1952	CONTIG76	135463	136117	1.39	9.24E-02	transcription_start_site	+	138291	138291	-2501	18850	AN4419
1952	CONTIG76	135463	136117	1.39	9.24E-02	transcription_start_site	+	138396	138396	-2606	18851	AN4419
1952	CONTIG76	135463	136117	1.39	9.24E-02	transcription_start_site	+	138696	138696	-2906	18852	AN4419
1952	CONTIG76	135463	136117	1.39	9.24E-02	transcription_start_site	+	139113	139113	-3323	18853	AN4419
1952	CONTIG76	135463	136117	1.39	9.24E-02	transcription_start_site	+	139584	139584	-3794	18854	AN4419
2084	CONTIG76	137859	138138	1.34	1.08E-01	transcription_start_site	+	138291	138291	-292	18850	AN4419
2084	CONTIG76	137859	138138	1.34	1.08E-01	transcription_start_site	+	138396	138396	-397	18851	AN4419

2084	CONTIG76	137859	138138	1.34	1.08E-01	transcription_start_site	+	138696	138696	-697	18852	AN4419
2084	CONTIG76	137859	138138	1.34	1.08E-01	transcription_start_site	+	139113	139113	-1114	18853	AN4419
2084	CONTIG76	137859	138138	1.34	1.08E-01	transcription_start_site	+	139584	139584	-1585	18854	AN4419
1943	CONTIG76	147019	147653	1.39	7.51E-02	transcription_start_site	+	147272	147272	64	18856	AN4421
1943	CONTIG76	147019	147653	1.39	7.51E-02	transcription_start_site	+	147474	147474	-138	18857	AN4421
1943	CONTIG76	147019	147653	1.39	7.51E-02	transcription_start_site	+	147077	147077	259	18855	AN4421
1943	CONTIG76	147019	147653	1.39	7.51E-02	transcription_start_site	+	148631	148631	-1295	18858	AN4421
1746	CONTIG76	150839	151253	1.48	4.42E-02	transcription_start_site	-	150357	150357	-689	18859	AN4422
1379	CONTIG77	11	280	1.64	1.84E-02	transcription_start_site	+	4451	4451	-4305	18867	AN4425
1379	CONTIG77	11	280	1.64	1.84E-02	transcription_start_site	+	4532	4532	-4386	18868	AN4425
1379	CONTIG77	11	280	1.64	1.84E-02	transcription_start_site	+	4803	4803	-4657	18869	AN4425
2633	CONTIG77	17260	17609	1.15	1.25E-01	transcription_start_site	-	17701	17701	266	18891	AN4430
2633	CONTIG77	17260	17609	1.15	1.25E-01	transcription_start_site	-	16839	16839	-595	18892	AN4430
2633	CONTIG77	17260	17609	1.15	1.25E-01	transcription_start_site	+	18240	18240	-805	18893	AN4431
2633	CONTIG77	17260	17609	1.15	1.25E-01	transcription_start_site	+	18504	18504	-1069	18894	AN4431
2633	CONTIG77	17260	17609	1.15	1.25E-01	transcription_start_site	+	18651	18651	-1216	18895	AN4431
2633	CONTIG77	17260	17609	1.15	1.25E-01	transcription_start_site	+	18957	18957	-1522	18896	AN4431
2633	CONTIG77	17260	17609	1.15	1.25E-01	transcription_start_site	+	19054	19054	-1619	18897	AN4431
2633	CONTIG77	17260	17609	1.15	1.25E-01	transcription_start_site	+	19166	19166	-1731	18898	AN4431
2633	CONTIG77	17260	17609	1.15	1.25E-01	transcription_start_site	+	19381	19381	-1946	18899	AN4431
2633	CONTIG77	17260	17609	1.15	1.25E-01	transcription_start_site	+	19637	19637	-2202	18900	AN4431
2633	CONTIG77	17260	17609	1.15	1.25E-01	transcription_start_site	+	20196	20196	-2761	18901	AN4431
1305	CONTIG77	44041	44402	1.69	1.57E-02	transcription_start_site	-	43049	43049	-1172	18919	AN4438
1305	CONTIG77	44041	44402	1.69	1.57E-02	transcription_start_site	-	41861	41861	-2360	18920	AN4438
1305	CONTIG77	44041	44402	1.69	1.57E-02	transcription_start_site	-	41592	41592	-2629	18921	AN4438
2281	CONTIG77	41326	41924	1.27	6.16E-02	transcription_start_site	-	41592	41592	-33	18921	AN4438
2281	CONTIG77	41326	41924	1.27	6.16E-02	transcription_start_site	-	41861	41861	236	18920	AN4438
2727	CONTIG77	46881	47245	1.11	1.44E-01	transcription_start_site	-	43049	43049	-4014	18919	AN4438
1305	CONTIG77	44041	44402	1.69	1.57E-02	transcription_start_site	+	44534	44534	-312	18922	AN4439
2281	CONTIG77	41326	41924	1.27	6.16E-02	transcription_start_site	+	44534	44534	-2909	18922	AN4439
2727	CONTIG77	46881	47245	1.11	1.44E-01	transcription_start_site	-	46072	46072	-991	18923	AN4440
2727	CONTIG77	46881	47245	1.11	1.44E-01	transcription_start_site	-	45896	45896	-1167	18924	AN4440
1305	CONTIG77	44041	44402	1.69	1.57E-02	transcription_start_site	+	46473	46473	-2251	18925	AN4441
1305	CONTIG77	44041	44402	1.69	1.57E-02	transcription_start_site	+	46808	46808	-2586	18926	AN4441
1305	CONTIG77	44041	44402	1.69	1.57E-02	transcription_start_site	+	47106	47106	-2884	18927	AN4441
2281	CONTIG77	41326	41924	1.27	6.16E-02	transcription_start_site	+	46473	46473	-4848	18925	AN4441
2281	CONTIG77	41326	41924	1.27	6.16E-02	transcription_start_site	+	46808	46808	-5183	18926	AN4441
2727	CONTIG77	46881	47245	1.11	1.44E-01	transcription_start_site	+	47106	47106	-43	18927	AN4441
2727	CONTIG77	46881	47245	1.11	1.44E-01	transcription_start_site	+	46808	46808	255	18926	AN4441
2727	CONTIG77	46881	47245	1.11	1.44E-01	transcription_start_site	+	46473	46473	590	18925	AN4441
797	CONTIG77	56565	56919	2.1	0.00E+00	transcription_start_site	-	56353	56353	-389	18928	AN4443
797	CONTIG77	56565	56919	2.1	0.00E+00	transcription_start_site	-	56277	56277	-465	18929	AN4443
1243	CONTIG77	60840	61114	1.73	1.30E-02	transcription_start_site	-	56353	56353	-4624	18928	AN4443
1243	CONTIG77	60840	61114	1.73	1.30E-02	transcription_start_site	-	56277	56277	-4700	18929	AN4443
797	CONTIG77	56565	56919	2.1	0.00E+00	transcription_start_site	+	58394	58394	-1652	18930	AN4444
797	CONTIG77	56565	56919	2.1	0.00E+00	transcription_start_site	+	58458	58458	-1716	18931	AN4444
1243	CONTIG77	60840	61114	1.73	1.30E-02	transcription_start_site	-	61763	61763	786	18934	AN4445
1243	CONTIG77	60840	61114	1.73	1.30E-02	transcription_start_site	-	61957	61957	980	18933	AN4445
1243	CONTIG77	60840	61114	1.73	1.30E-02	transcription_start_site	-	62098	62098	1121	18932	AN4445
2134	CONTIG77	65410	65694	1.32	6.84E-02	transcription_start_site	-	62098	62098	-3454	18932	AN4445
2134	CONTIG77	65410	65694	1.32	6.84E-02	transcription_start_site	-	61957	61957	-3595	18933	AN4445
2134	CONTIG77	65410	65694	1.32	6.84E-02	transcription_start_site	-	61763	61763	-3789	18934	AN4445
1243	CONTIG77	60840	61114	1.73	1.30E-02	transcription_start_site	+	62366	62366	-1389	18935	AN4446
1243	CONTIG77	60840	61114	1.73	1.30E-02	transcription_start_site	+	62583	62583	-1606	18936	AN4446
1243	CONTIG77	60840	61114	1.73	1.30E-02	transcription_start_site	+	63061	63061	-2084	18937	AN4446
406	CONTIG77	68260	68529	2.59	0.00E+00	transcription_start_site	-	67554	67554	-840	18938	AN4447
406	CONTIG77	68260	68529	2.59	0.00E+00	transcription_start_site	-	66556	66556	-1838	18939	AN4447

2134	CONTIG77	65410	65694	1.32	6.84E-02	transcription_start_site	-	66556	66556	1004	18939	AN4447
406	CONTIG77	68260	68529	2.59	0.00E+00	transcription_start_site	-	68297	68297	-97	18942	AN4448
406	CONTIG77	68260	68529	2.59	0.00E+00	transcription_start_site	-	68663	68663	268	18941	AN4448
406	CONTIG77	68260	68529	2.59	0.00E+00	transcription_start_site	-	68054	68054	-340	18943	AN4448
406	CONTIG77	68260	68529	2.59	0.00E+00	transcription_start_site	-	68983	68983	588	18940	AN4448
406	CONTIG77	68260	68529	2.59	0.00E+00	transcription_start_site	-	69416	69416	1021	18945	AN4449
406	CONTIG77	68260	68529	2.59	0.00E+00	transcription_start_site	+	70452	70452	-2057	18946	AN4450
406	CONTIG77	68260	68529	2.59	0.00E+00	transcription_start_site	+	70647	70647	-2252	18947	AN4450
2134	CONTIG77	65410	65694	1.32	6.84E-02	transcription_start_site	+	70452	70452	-4900	18946	AN4450
2134	CONTIG77	65410	65694	1.32	6.84E-02	transcription_start_site	+	70647	70647	-5095	18947	AN4450
714	CONTIG77	93462	93741	2.18	1.54E-03	transcription_start_site	-	90715	90715	-2886	18970	AN4456
714	CONTIG77	93462	93741	2.18	1.54E-03	transcription_start_site	-	90292	90292	-3309	18971	AN4456
714	CONTIG77	93462	93741	2.18	1.54E-03	transcription_start_site	+	92958	92958	643	18977	AN4458
2407	CONTIG77	99752	100041	1.23	9.24E-02	transcription_start_site	-	96623	96623	-3273	18978	AN4459
2407	CONTIG77	99752	100041	1.23	9.24E-02	transcription_start_site	-	96351	96351	-3545	18979	AN4459
2407	CONTIG77	99752	100041	1.23	9.24E-02	transcription_start_site	-	96116	96116	-3780	18980	AN4459
714	CONTIG77	93462	93741	2.18	1.54E-03	transcription_start_site	+	97476	97476	-3874	18981	AN4460
714	CONTIG77	93462	93741	2.18	1.54E-03	transcription_start_site	+	97703	97703	-4101	18982	AN4460
714	CONTIG77	93462	93741	2.18	1.54E-03	transcription_start_site	+	97787	97787	-4185	18983	AN4460
2729	CONTIG77	106746	107013	1.11	1.44E-01	transcription_start_site	-	102438	102438	-4441	18984	AN4461
2794	CONTIG77	101717	101991	1.07	1.66E-01	transcription_start_site	-	102438	102438	584	18984	AN4461
1917	CONTIG77	107857	108131	1.4	5.22E-02	transcription_start_site	-	106846	106846	-1148	18985	AN4462
1917	CONTIG77	107857	108131	1.4	5.22E-02	transcription_start_site	-	103309	103309	-4685	18986	AN4462
2729	CONTIG77	106746	107013	1.11	1.44E-01	transcription_start_site	-	106846	106846	-33	18985	AN4462
2729	CONTIG77	106746	107013	1.11	1.44E-01	transcription_start_site	-	103309	103309	-3570	18986	AN4462
1917	CONTIG77	107857	108131	1.4	5.22E-02	transcription_start_site	-	108813	108813	819	18992	AN4463
2795	CONTIG77	118953	119302	1.07	1.66E-01	transcription_start_site	-	117244	117244	-1883	18993	AN4464
2795	CONTIG77	118953	119302	1.07	1.66E-01	transcription_start_site	-	117042	117042	-2085	18994	AN4464
2795	CONTIG77	118953	119302	1.07	1.66E-01	transcription_start_site	-	116803	116803	-2324	18995	AN4464
2795	CONTIG77	118953	119302	1.07	1.66E-01	transcription_start_site	-	115264	115264	-3863	18996	AN4464
2795	CONTIG77	118953	119302	1.07	1.66E-01	transcription_start_site	-	119318	119318	190	18997	AN4465
2795	CONTIG77	118953	119302	1.07	1.66E-01	transcription_start_site	-	118555	118555	-572	18998	AN4465
2725	CONTIG77	127430	128100	1.11	1.33E-01	transcription_start_site	-	128527	128527	762	19004	AN4467
2725	CONTIG77	127430	128100	1.11	1.33E-01	transcription_start_site	-	128726	128726	961	19003	AN4467
2725	CONTIG77	127430	128100	1.11	1.33E-01	transcription_start_site	-	128892	128892	1127	19002	AN4467
2725	CONTIG77	127430	128100	1.11	1.33E-01	transcription_start_site	-	128971	128971	1206	19001	AN4467
757	CONTIG77	126096	126590	2.14	1.80E-03	transcription_start_site	+	129607	129607	-3264	19005	AN4468
757	CONTIG77	126096	126590	2.14	1.80E-03	transcription_start_site	+	129662	129662	-3319	19006	AN4468
757	CONTIG77	126096	126590	2.14	1.80E-03	transcription_start_site	+	129788	129788	-3445	19007	AN4468
757	CONTIG77	126096	126590	2.14	1.80E-03	transcription_start_site	+	130118	130118	-3775	19008	AN4468
2725	CONTIG77	127430	128100	1.11	1.33E-01	transcription_start_site	+	129607	129607	-1842	19005	AN4468
2725	CONTIG77	127430	128100	1.11	1.33E-01	transcription_start_site	+	129662	129662	-1897	19006	AN4468
2725	CONTIG77	127430	128100	1.11	1.33E-01	transcription_start_site	+	129788	129788	-2023	19007	AN4468
2725	CONTIG77	127430	128100	1.11	1.33E-01	transcription_start_site	+	130118	130118	-2353	19008	AN4468
1949	CONTIG78	4071	4420	1.39	8.01E-02	transcription_start_site	-	1825	1825	-2420	19011	AN4470
1949	CONTIG78	4071	4420	1.39	8.01E-02	transcription_start_site	-	1235	1235	-3010	19012	AN4470
1949	CONTIG78	4071	4420	1.39	8.01E-02	transcription_start_site	-	171	171	-4074	19013	AN4470
2360	CONTIG78	4955	5529	1.25	1.07E-01	transcription_start_site	-	1825	1825	-3417	19011	AN4470
2360	CONTIG78	4955	5529	1.25	1.07E-01	transcription_start_site	-	1235	1235	-4007	19012	AN4470
2360	CONTIG78	4955	5529	1.25	1.07E-01	transcription_start_site	-	171	171	-5071	19013	AN4470
1949	CONTIG78	4071	4420	1.39	8.01E-02	transcription_start_site	-	3924	3924	-321	19014	AN4471
1949	CONTIG78	4071	4420	1.39	8.01E-02	transcription_start_site	-	2939	2939	-1306	19015	AN4471
2360	CONTIG78	4955	5529	1.25	1.07E-01	transcription_start_site	-	3924	3924	-1318	19014	AN4471
2360	CONTIG78	4955	5529	1.25	1.07E-01	transcription_start_site	-	2939	2939	-2303	19015	AN4471
1949	CONTIG78	4071	4420	1.39	8.01E-02	transcription_start_site	+	4590	4590	-344	19016	AN4472
1949	CONTIG78	4071	4420	1.39	8.01E-02	transcription_start_site	+	4734	4734	-488	19017	AN4472
1949	CONTIG78	4071	4420	1.39	8.01E-02	transcription_start_site	+	4960	4960	-714	19018	AN4472

1949	CONTIG78	4071	4420	1.39	8.01E-02	transcription_start_site	+	7902	7902	-3656	19019	AN4472
1949	CONTIG78	4071	4420	1.39	8.01E-02	transcription_start_site	+	8113	8113	-3867	19020	AN4472
2360	CONTIG78	4955	5529	1.25	1.07E-01	transcription_start_site	+	4960	4960	282	19018	AN4472
2360	CONTIG78	4955	5529	1.25	1.07E-01	transcription_start_site	+	4734	4734	508	19017	AN4472
2360	CONTIG78	4955	5529	1.25	1.07E-01	transcription_start_site	+	4590	4590	652	19016	AN4472
2360	CONTIG78	4955	5529	1.25	1.07E-01	transcription_start_site	+	7902	7902	-2660	19019	AN4472
2360	CONTIG78	4955	5529	1.25	1.07E-01	transcription_start_site	+	8113	8113	-2871	19020	AN4472
73	CONTIG78	11568	12502	3.31	0.00E+00	transcription_start_site	-	11773	11773	-262	19021	AN4473
73	CONTIG78	11568	12502	3.31	0.00E+00	transcription_start_site	-	11381	11381	-654	19022	AN4473
73	CONTIG78	11568	12502	3.31	0.00E+00	transcription_start_site	-	11298	11298	-737	19023	AN4473
885	CONTIG78	10593	10927	2.01	7.85E-03	transcription_start_site	-	11298	11298	538	19023	AN4473
885	CONTIG78	10593	10927	2.01	7.85E-03	transcription_start_site	-	11381	11381	621	19022	AN4473
885	CONTIG78	10593	10927	2.01	7.85E-03	transcription_start_site	-	11773	11773	1013	19021	AN4473
1459	CONTIG78	14935	15371	1.61	3.70E-02	transcription_start_site	-	11773	11773	-3380	19021	AN4473
1459	CONTIG78	14935	15371	1.61	3.70E-02	transcription_start_site	-	11381	11381	-3772	19022	AN4473
1459	CONTIG78	14935	15371	1.61	3.70E-02	transcription_start_site	-	11298	11298	-3855	19023	AN4473
73	CONTIG78	11568	12502	3.31	0.00E+00	transcription_start_site	+	12951	12951	-916	19024	AN4474
73	CONTIG78	11568	12502	3.31	0.00E+00	transcription_start_site	+	13185	13185	-1150	19025	AN4474
885	CONTIG78	10593	10927	2.01	7.85E-03	transcription_start_site	+	12951	12951	-2191	19024	AN4474
885	CONTIG78	10593	10927	2.01	7.85E-03	transcription_start_site	+	13185	13185	-2425	19025	AN4474
1459	CONTIG78	14935	15371	1.61	3.70E-02	transcription_start_site	-	15007	15007	-146	19026	AN4475
1459	CONTIG78	14935	15371	1.61	3.70E-02	transcription_start_site	-	14925	14925	-228	19027	AN4475
1459	CONTIG78	14935	15371	1.61	3.70E-02	transcription_start_site	-	14663	14663	-490	19028	AN4475
1459	CONTIG78	14935	15371	1.61	3.70E-02	transcription_start_site	-	14342	14342	-811	19029	AN4475
989	CONTIG78	25952	26320	1.92	1.11E-02	transcription_start_site	+	27350	27350	-1214	19036	AN4479
989	CONTIG78	25952	26320	1.92	1.11E-02	transcription_start_site	+	27645	27645	-1509	19037	AN4479
138	CONTIG78	38859	39428	3.08	0.00E+00	transcription_start_site	-	34838	34838	-4305	19038	AN4480
138	CONTIG78	38859	39428	3.08	0.00E+00	transcription_start_site	-	37171	37171	-1972	19039	AN4481
138	CONTIG78	38859	39428	3.08	0.00E+00	transcription_start_site	-	35802	35802	-3341	19040	AN4481
138	CONTIG78	38859	39428	3.08	0.00E+00	transcription_start_site	-	35738	35738	-3405	19041	AN4481
138	CONTIG78	38859	39428	3.08	0.00E+00	transcription_start_site	-	35558	35558	-3585	19042	AN4481
2717	CONTIG78	42007	42597	1.12	1.93E-01	transcription_start_site	-	37171	37171	-5131	19039	AN4481
138	CONTIG78	38859	39428	3.08	0.00E+00	transcription_start_site	+	40298	40298	-1154	19043	AN4482
138	CONTIG78	38859	39428	3.08	0.00E+00	transcription_start_site	+	40700	40700	-1556	19044	AN4482
138	CONTIG78	38859	39428	3.08	0.00E+00	transcription_start_site	+	40847	40847	-1703	19045	AN4482
138	CONTIG78	38859	39428	3.08	0.00E+00	transcription_start_site	+	40909	40909	-1765	19046	AN4482
138	CONTIG78	38859	39428	3.08	0.00E+00	transcription_start_site	+	41176	41176	-2032	19047	AN4482
138	CONTIG78	38859	39428	3.08	0.00E+00	transcription_start_site	+	41777	41777	-2633	19048	AN4482
2717	CONTIG78	42007	42597	1.12	1.93E-01	transcription_start_site	+	41777	41777	525	19048	AN4482
2717	CONTIG78	42007	42597	1.12	1.93E-01	transcription_start_site	+	41176	41176	1126	19047	AN4482
936	CONTIG78	46502	46786	1.97	8.81E-03	transcription_start_site	-	45899	45899	-745	19049	AN4483
936	CONTIG78	46502	46786	1.97	8.81E-03	transcription_start_site	-	45340	45340	-1304	19050	AN4483
936	CONTIG78	46502	46786	1.97	8.81E-03	transcription_start_site	-	45185	45185	-1459	19051	AN4483
936	CONTIG78	46502	46786	1.97	8.81E-03	transcription_start_site	-	44893	44893	-1751	19052	AN4483
2624	CONTIG78	43517	44241	1.16	1.66E-01	transcription_start_site	-	44893	44893	1014	19052	AN4483
2624	CONTIG78	43517	44241	1.16	1.66E-01	transcription_start_site	-	45185	45185	1306	19051	AN4483
838	CONTIG78	42917	43256	2.06	6.61E-03	transcription_start_site	+	47872	47872	-4785	19053	AN4484
936	CONTIG78	46502	46786	1.97	8.81E-03	transcription_start_site	+	47872	47872	-1228	19053	AN4484
2624	CONTIG78	43517	44241	1.16	1.66E-01	transcription_start_site	+	47872	47872	-3993	19053	AN4484
2082	CONTIG78	77480	77769	1.34	9.24E-02	transcription_start_site	+	76662	76662	962	19089	AN4494
2082	CONTIG78	77480	77769	1.34	9.24E-02	transcription_start_site	+	76522	76522	1102	19088	AN4494
1640	CONTIG78	84991	85410	1.52	5.22E-02	transcription_start_site	-	82578	82578	-2622	19091	AN4496
1640	CONTIG78	84991	85410	1.52	5.22E-02	transcription_start_site	-	82390	82390	-2810	19092	AN4496
1640	CONTIG78	84991	85410	1.52	5.22E-02	transcription_start_site	-	82178	82178	-3022	19093	AN4496
1640	CONTIG78	84991	85410	1.52	5.22E-02	transcription_start_site	+	84937	84937	263	19094	AN4497
1460	CONTIG78	95483	95757	1.61	3.70E-02	transcription_start_site	-	91218	91218	-4402	19095	AN4498
1460	CONTIG78	95483	95757	1.61	3.70E-02	transcription_start_site	-	91056	91056	-4564	19096	AN4498

2718	CONTIG78	91953	92237	1.12	1.93E-01	transcription_start_site	-	91218	91218	-877	19095	AN4498
2718	CONTIG78	91953	92237	1.12	1.93E-01	transcription_start_site	-	91056	91056	-1039	19096	AN4498
2718	CONTIG78	91953	92237	1.12	1.93E-01	transcription_start_site	-	90404	90404	-1691	19097	AN4498
2718	CONTIG78	91953	92237	1.12	1.93E-01	transcription_start_site	-	90115	90115	-1980	19098	AN4498
2718	CONTIG78	91953	92237	1.12	1.93E-01	transcription_start_site	+	91733	91733	362	19099	AN4499
2718	CONTIG78	91953	92237	1.12	1.93E-01	transcription_start_site	+	93346	93346	-1251	19100	AN4499
2718	CONTIG78	91953	92237	1.12	1.93E-01	transcription_start_site	+	93538	93538	-1443	19101	AN4499
1460	CONTIG78	95483	95757	1.61	3.70E-02	transcription_start_site	-	94819	94819	-801	19102	AN4500
1460	CONTIG78	95483	95757	1.61	3.70E-02	transcription_start_site	-	94499	94499	-1121	19103	AN4500
1460	CONTIG78	95483	95757	1.61	3.70E-02	transcription_start_site	-	94351	94351	-1269	19104	AN4500
1460	CONTIG78	95483	95757	1.61	3.70E-02	transcription_start_site	-	94200	94200	-1420	19105	AN4500
1460	CONTIG78	95483	95757	1.61	3.70E-02	transcription_start_site	+	96592	96592	-972	19106	AN4501
1460	CONTIG78	95483	95757	1.61	3.70E-02	transcription_start_site	+	96721	96721	-1101	19107	AN4501
1460	CONTIG78	95483	95757	1.61	3.70E-02	transcription_start_site	+	97087	97087	-1467	19108	AN4501
1460	CONTIG78	95483	95757	1.61	3.70E-02	transcription_start_site	+	97381	97381	-1761	19109	AN4501
1460	CONTIG78	95483	95757	1.61	3.70E-02	transcription_start_site	+	97502	97502	-1882	19110	AN4501
2718	CONTIG78	91953	92237	1.12	1.93E-01	transcription_start_site	+	96592	96592	-4497	19106	AN4501
2718	CONTIG78	91953	92237	1.12	1.93E-01	transcription_start_site	+	96721	96721	-4626	19107	AN4501
2718	CONTIG78	91953	92237	1.12	1.93E-01	transcription_start_site	+	97087	97087	-4992	19108	AN4501
262	CONTIG78	101121	102287	2.82	0.00E+00	transcription_start_site	-	101111	101111	-593	19111	AN4502
262	CONTIG78	101121	102287	2.82	0.00E+00	transcription_start_site	-	100731	100731	-973	19112	AN4502
801	CONTIG78	103143	103639	2.1	5.20E-03	transcription_start_site	-	101111	101111	-2280	19111	AN4502
801	CONTIG78	103143	103639	2.1	5.20E-03	transcription_start_site	-	100731	100731	-2660	19112	AN4502
836	CONTIG78	104330	104776	2.06	2.49E-03	transcription_start_site	-	101111	101111	-3442	19111	AN4502
836	CONTIG78	104330	104776	2.06	2.49E-03	transcription_start_site	-	100731	100731	-3822	19112	AN4502
1950	CONTIG78	118502	118781	1.39	8.01E-02	transcription_start_site	-	117172	117172	-1469	19125	AN4508
1950	CONTIG78	118502	118781	1.39	8.01E-02	transcription_start_site	-	116591	116591	-2050	19126	AN4508
1950	CONTIG78	118502	118781	1.39	8.01E-02	transcription_start_site	+	118824	118824	-182	19127	AN4509
1950	CONTIG78	118502	118781	1.39	8.01E-02	transcription_start_site	+	119659	119659	-1017	19128	AN4509
2620	CONTIG78	126529	126968	1.16	1.50E-01	transcription_start_site	-	126481	126481	-267	19129	AN4510
2620	CONTIG78	126529	126968	1.16	1.50E-01	transcription_start_site	-	124001	124001	-2747	19130	AN4510
2620	CONTIG78	126529	126968	1.16	1.50E-01	transcription_start_site	+	129091	129091	-2342	19133	AN4512
2620	CONTIG78	126529	126968	1.16	1.50E-01	transcription_start_site	+	129180	129180	-2431	19134	AN4512
2620	CONTIG78	126529	126968	1.16	1.50E-01	transcription_start_site	+	130396	130396	-3647	19135	AN4513
2620	CONTIG78	126529	126968	1.16	1.50E-01	transcription_start_site	+	130652	130652	-3903	19136	AN4513
2625	CONTIG78	138996	139353	1.16	1.66E-01	transcription_start_site	+	140026	140026	-851	19137	AN4515
2625	CONTIG78	138996	139353	1.16	1.66E-01	transcription_start_site	+	140202	140202	-1027	19138	AN4515
2625	CONTIG78	138996	139353	1.16	1.66E-01	transcription_start_site	+	140695	140695	-1520	19139	AN4515
1860	CONTIG78	214132	214931	1.43	5.05E-02	transcription_start_site	-	213479	213479	-1052	19202	AN4535
1860	CONTIG78	214132	214931	1.43	5.05E-02	transcription_start_site	-	215749	215749	1217	19206	AN4536
2365	CONTIG78	220212	220711	1.25	1.25E-01	transcription_start_site	-	219312	219312	-1149	19203	AN4536
2365	CONTIG78	220212	220711	1.25	1.25E-01	transcription_start_site	-	218978	218978	-1483	19204	AN4536
2365	CONTIG78	220212	220711	1.25	1.25E-01	transcription_start_site	-	218721	218721	-1740	19205	AN4536
2365	CONTIG78	220212	220711	1.25	1.25E-01	transcription_start_site	-	215749	215749	-4712	19206	AN4536
2365	CONTIG78	220212	220711	1.25	1.25E-01	transcription_start_site	-	220364	220364	-97	19213	AN4537
2365	CONTIG78	220212	220711	1.25	1.25E-01	transcription_start_site	+	223440	223440	-2978	19214	AN4538
707	CONTIG78	230937	231276	2.19	3.61E-03	transcription_start_site	-	229728	229728	-1378	19223	AN4541
707	CONTIG78	230937	231276	2.19	3.61E-03	transcription_start_site	-	229161	229161	-1945	19224	AN4541
2626	CONTIG78	232067	232631	1.16	1.66E-01	transcription_start_site	-	229728	229728	-2621	19223	AN4541
2626	CONTIG78	232067	232631	1.16	1.66E-01	transcription_start_site	-	229161	229161	-3188	19224	AN4541
707	CONTIG78	230937	231276	2.19	3.61E-03	transcription_start_site	+	232183	232183	-1076	19225	AN4542
707	CONTIG78	230937	231276	2.19	3.61E-03	transcription_start_site	+	232301	232301	-1194	19226	AN4542
707	CONTIG78	230937	231276	2.19	3.61E-03	transcription_start_site	+	233015	233015	-1908	19227	AN4542
2626	CONTIG78	232067	232631	1.16	1.66E-01	transcription_start_site	+	232301	232301	48	19226	AN4542
2626	CONTIG78	232067	232631	1.16	1.66E-01	transcription_start_site	+	232183	232183	166	19225	AN4542
2626	CONTIG78	232067	232631	1.16	1.66E-01	transcription_start_site	+	233015	233015	-666	19227	AN4542
753	CONTIG78	239854	240203	2.15	4.61E-03	transcription_start_site	-	236226	236226	-3802	19228	AN4543

753	CONTIG78	239854	240203	2.15	4.61E-03	transcription_start_site	-	236007	236007	-4021	19229 AN4543
241	CONTIG78	242929	243218	2.86	0.00E+00	transcription_start_site	-	238917	238917	-4156	19230 AN4544
526	CONTIG78	243244	243893	2.41	1.22E-03	transcription_start_site	-	238917	238917	-4651	19230 AN4544
753	CONTIG78	239854	240203	2.15	4.61E-03	transcription_start_site	-	238917	238917	-1111	19230 AN4544
241	CONTIG78	242929	243218	2.86	0.00E+00	transcription_start_site	+	243691	243691	-617	19231 AN4545
241	CONTIG78	242929	243218	2.86	0.00E+00	transcription_start_site	+	244863	244863	-1789	19232 AN4545
351	CONTIG78	243989	244263	2.68	2.16E-04	transcription_start_site	+	243691	243691	435	19231 AN4545
351	CONTIG78	243989	244263	2.68	2.16E-04	transcription_start_site	+	244863	244863	-737	19232 AN4545
526	CONTIG78	243244	243893	2.41	1.22E-03	transcription_start_site	+	243691	243691	-122	19231 AN4545
526	CONTIG78	243244	243893	2.41	1.22E-03	transcription_start_site	+	244863	244863	-1294	19232 AN4545
753	CONTIG78	239854	240203	2.15	4.61E-03	transcription_start_site	+	243691	243691	-3662	19231 AN4545
753	CONTIG78	239854	240203	2.15	4.61E-03	transcription_start_site	+	244863	244863	-4834	19232 AN4545
2621	CONTIG78	250069	250484	1.16	1.50E-01	transcription_start_site	-	250375	250375	98	19234 AN4546
2621	CONTIG78	250069	250484	1.16	1.50E-01	transcription_start_site	-	250445	250445	168	19233 AN4546
2621	CONTIG78	250069	250484	1.16	1.50E-01	transcription_start_site	-	249760	249760	-516	19235 AN4546
2621	CONTIG78	250069	250484	1.16	1.50E-01	transcription_start_site	-	249250	249250	-1026	19236 AN4546
2621	CONTIG78	250069	250484	1.16	1.50E-01	transcription_start_site	+	251217	251217	-940	19237 AN4547
2621	CONTIG78	250069	250484	1.16	1.50E-01	transcription_start_site	+	251370	251370	-1093	19238 AN4547
2621	CONTIG78	250069	250484	1.16	1.50E-01	transcription_start_site	+	251728	251728	-1451	19239 AN4547
2621	CONTIG78	250069	250484	1.16	1.50E-01	transcription_start_site	+	252748	252748	-2471	19240 AN4547
2621	CONTIG78	250069	250484	1.16	1.50E-01	transcription_start_site	+	253146	253146	-2869	19241 AN4547
2621	CONTIG78	250069	250484	1.16	1.50E-01	transcription_start_site	+	254014	254014	-3737	19242 AN4547
1553	CONTIG78	266035	266304	1.56	4.36E-02	transcription_start_site	-	265866	265866	-303	19254 AN4552
1553	CONTIG78	266035	266304	1.56	4.36E-02	transcription_start_site	-	265692	265692	-477	19255 AN4552
1553	CONTIG78	266035	266304	1.56	4.36E-02	transcription_start_site	-	265587	265587	-582	19256 AN4552
1553	CONTIG78	266035	266304	1.56	4.36E-02	transcription_start_site	-	265468	265468	-701	19257 AN4552
1553	CONTIG78	266035	266304	1.56	4.36E-02	transcription_start_site	-	265355	265355	-814	19258 AN4552
1553	CONTIG78	266035	266304	1.56	4.36E-02	transcription_start_site	-	265157	265157	-1012	19259 AN4552
1553	CONTIG78	266035	266304	1.56	4.36E-02	transcription_start_site	-	265032	265032	-1137	19260 AN4552
1553	CONTIG78	266035	266304	1.56	4.36E-02	transcription_start_site	-	264454	264454	-1715	19261 AN4552
1553	CONTIG78	266035	266304	1.56	4.36E-02	transcription_start_site	-	264019	264019	-2150	19262 AN4552
1553	CONTIG78	266035	266304	1.56	4.36E-02	transcription_start_site	-	263745	263745	-2424	19263 AN4552
1553	CONTIG78	266035	266304	1.56	4.36E-02	transcription_start_site	+	266265	266265	-95	19264 AN4553
1553	CONTIG78	266035	266304	1.56	4.36E-02	transcription_start_site	+	266352	266352	-182	19265 AN4553
1553	CONTIG78	266035	266304	1.56	4.36E-02	transcription_start_site	+	266441	266441	-271	19266 AN4553
1553	CONTIG78	266035	266304	1.56	4.36E-02	transcription_start_site	+	266773	266773	-603	19267 AN4553
1553	CONTIG78	266035	266304	1.56	4.36E-02	transcription_start_site	+	267422	267422	-1252	19268 AN4554
1158	CONTIG78	280446	280872	1.79	1.84E-02	transcription_start_site	-	276253	276253	-4406	19278 AN4557
1158	CONTIG78	280446	280872	1.79	1.84E-02	transcription_start_site	-	280067	280067	-592	19280 AN4558
1158	CONTIG78	280446	280872	1.79	1.84E-02	transcription_start_site	-	279793	279793	-866	19281 AN4558
1158	CONTIG78	280446	280872	1.79	1.84E-02	transcription_start_site	-	279607	279607	-1052	19282 AN4558
1158	CONTIG78	280446	280872	1.79	1.84E-02	transcription_start_site	-	279081	279081	-1578	19283 AN4558
1158	CONTIG78	280446	280872	1.79	1.84E-02	transcription_start_site	-	278625	278625	-2034	19284 AN4558
1158	CONTIG78	280446	280872	1.79	1.84E-02	transcription_start_site	-	278400	278400	-2259	19285 AN4558
1158	CONTIG78	280446	280872	1.79	1.84E-02	transcription_start_site	-	277780	277780	-2879	19286 AN4558
1158	CONTIG78	280446	280872	1.79	1.84E-02	transcription_start_site	-	281443	281443	784	19290 AN4559
1158	CONTIG78	280446	280872	1.79	1.84E-02	transcription_start_site	-	281552	281552	893	19289 AN4559
1158	CONTIG78	280446	280872	1.79	1.84E-02	transcription_start_site	-	281675	281675	1016	19288 AN4559
1158	CONTIG78	280446	280872	1.79	1.84E-02	transcription_start_site	-	281781	281781	1122	19287 AN4559
1158	CONTIG78	280446	280872	1.79	1.84E-02	transcription_start_site	+	282052	282052	-1393	19291 AN4560
1158	CONTIG78	280446	280872	1.79	1.84E-02	transcription_start_site	+	282126	282126	-1467	19292 AN4560
1158	CONTIG78	280446	280872	1.79	1.84E-02	transcription_start_site	+	283244	283244	-2585	19293 AN4561
1158	CONTIG78	280446	280872	1.79	1.84E-02	transcription_start_site	+	283367	283367	-2708	19294 AN4561
1158	CONTIG78	280446	280872	1.79	1.84E-02	transcription_start_site	+	283638	283638	-2979	19295 AN4561
1158	CONTIG78	280446	280872	1.79	1.84E-02	transcription_start_site	+	284061	284061	-3402	19296 AN4561
2720	CONTIG78	289501	289935	1.12	1.99E-01	transcription_start_site	-	285905	285905	-3813	19297 AN4562
2720	CONTIG78	289501	289935	1.12	1.99E-01	transcription_start_site	-	285708	285708	-4010	19298 AN4562

2720	CONTIG78	289501	289935	1.12	1.99E-01	transcription_start_site	-	285372	285372	-4346	19299 AN4562
2720	CONTIG78	289501	289935	1.12	1.99E-01	transcription_start_site	-	288873	288873	-845	19300 AN4563
2720	CONTIG78	289501	289935	1.12	1.99E-01	transcription_start_site	-	288800	288800	-918	19301 AN4563
2720	CONTIG78	289501	289935	1.12	1.99E-01	transcription_start_site	-	288674	288674	-1044	19302 AN4563
2720	CONTIG78	289501	289935	1.12	1.99E-01	transcription_start_site	-	287528	287528	-2190	19303 AN4563
2493	CONTIG78	296718	296977	1.21	1.44E-01	transcription_start_site	-	295712	295712	-1135	19304 AN4564
2493	CONTIG78	296718	296977	1.21	1.44E-01	transcription_start_site	-	295375	295375	-1472	19305 AN4564
2493	CONTIG78	296718	296977	1.21	1.44E-01	transcription_start_site	-	294926	294926	-1921	19306 AN4564
2493	CONTIG78	296718	296977	1.21	1.44E-01	transcription_start_site	-	294583	294583	-2264	19307 AN4564
2493	CONTIG78	296718	296977	1.21	1.44E-01	transcription_start_site	+	297869	297869	-1021	19308 AN4565
2493	CONTIG78	296718	296977	1.21	1.44E-01	transcription_start_site	+	298387	298387	-1539	19309 AN4565
123	CONTIG78	303093	303362	3.13	0.00E+00	transcription_start_site	-	302824	302824	-403	19310 AN4566
123	CONTIG78	303093	303362	3.13	0.00E+00	transcription_start_site	-	302483	302483	-744	19311 AN4566
123	CONTIG78	303093	303362	3.13	0.00E+00	transcription_start_site	-	302248	302248	-979	19312 AN4566
123	CONTIG78	303093	303362	3.13	0.00E+00	transcription_start_site	-	300568	300568	-2659	19313 AN4566
123	CONTIG78	303093	303362	3.13	0.00E+00	transcription_start_site	-	300118	300118	-3109	19314 AN4566
123	CONTIG78	303093	303362	3.13	0.00E+00	transcription_start_site	-	299552	299552	-3675	19315 AN4566
1641	CONTIG78	302258	302907	1.52	5.22E-02	transcription_start_site	-	302483	302483	-99	19311 AN4566
1641	CONTIG78	302258	302907	1.52	5.22E-02	transcription_start_site	-	302824	302824	241	19310 AN4566
1641	CONTIG78	302258	302907	1.52	5.22E-02	transcription_start_site	-	302248	302248	-334	19312 AN4566
1641	CONTIG78	302258	302907	1.52	5.22E-02	transcription_start_site	-	300568	300568	-2014	19313 AN4566
1641	CONTIG78	302258	302907	1.52	5.22E-02	transcription_start_site	-	300118	300118	-2464	19314 AN4566
1641	CONTIG78	302258	302907	1.52	5.22E-02	transcription_start_site	-	299552	299552	-3030	19315 AN4566
2494	CONTIG78	308780	309229	1.21	1.44E-01	transcription_start_site	-	305629	305629	-3375	19316 AN4567
2494	CONTIG78	308780	309229	1.21	1.44E-01	transcription_start_site	-	305410	305410	-3594	19317 AN4567
2494	CONTIG78	308780	309229	1.21	1.44E-01	transcription_start_site	-	304616	304616	-4388	19318 AN4567
2494	CONTIG78	308780	309229	1.21	1.44E-01	transcription_start_site	-	304451	304451	-4553	19319 AN4567
2494	CONTIG78	308780	309229	1.21	1.44E-01	transcription_start_site	-	308910	308910	-94	19320 AN4569
2494	CONTIG78	308780	309229	1.21	1.44E-01	transcription_start_site	+	309444	309444	-439	19321 AN4570
2494	CONTIG78	308780	309229	1.21	1.44E-01	transcription_start_site	+	309754	309754	-749	19322 AN4570
2494	CONTIG78	308780	309229	1.21	1.44E-01	transcription_start_site	+	310537	310537	-1532	19323 AN4570
2494	CONTIG78	308780	309229	1.21	1.44E-01	transcription_start_site	+	310726	310726	-1721	19324 AN4570
2494	CONTIG78	308780	309229	1.21	1.44E-01	transcription_start_site	+	311971	311971	-2966	19325 AN4571
2361	CONTIG78	322295	322864	1.25	1.07E-01	transcription_start_site	+	322211	322211	368	19335 AN4575
2361	CONTIG78	322295	322864	1.25	1.07E-01	transcription_start_site	+	323784	323784	-1204	19336 AN4576
2361	CONTIG78	322295	322864	1.25	1.07E-01	transcription_start_site	+	323891	323891	-1311	19337 AN4576
2361	CONTIG78	322295	322864	1.25	1.07E-01	transcription_start_site	+	324169	324169	-1589	19338 AN4576
1755	CONTIG78	335176	335470	1.48	5.88E-02	transcription_start_site	-	331587	331587	-3736	19350 AN4580
1755	CONTIG78	335176	335470	1.48	5.88E-02	transcription_start_site	-	331467	331467	-3856	19351 AN4580
1755	CONTIG78	335176	335470	1.48	5.88E-02	transcription_start_site	-	335995	335995	672	19355 AN4582
1755	CONTIG78	335176	335470	1.48	5.88E-02	transcription_start_site	-	336057	336057	734	19354 AN4582
2366	CONTIG78	340746	341000	1.25	1.25E-01	transcription_start_site	-	336057	336057	-4816	19354 AN4582
2366	CONTIG78	340746	341000	1.25	1.25E-01	transcription_start_site	-	335995	335995	-4878	19355 AN4582
2366	CONTIG78	340746	341000	1.25	1.25E-01	transcription_start_site	-	337946	337946	-2927	19356 AN4583
2366	CONTIG78	340746	341000	1.25	1.25E-01	transcription_start_site	-	337861	337861	-3012	19357 AN4583
2366	CONTIG78	340746	341000	1.25	1.25E-01	transcription_start_site	-	337739	337739	-3134	19358 AN4583
2366	CONTIG78	340746	341000	1.25	1.25E-01	transcription_start_site	-	337676	337676	-3197	19359 AN4583
2366	CONTIG78	340746	341000	1.25	1.25E-01	transcription_start_site	-	337531	337531	-3342	19360 AN4583
1756	CONTIG78	343893	344302	1.48	5.88E-02	transcription_start_site	-	339053	339053	-5044	19361 AN4584
1756	CONTIG78	343893	344302	1.48	5.88E-02	transcription_start_site	-	338931	338931	-5166	19362 AN4584
2366	CONTIG78	340746	341000	1.25	1.25E-01	transcription_start_site	-	339053	339053	-1820	19361 AN4584
2366	CONTIG78	340746	341000	1.25	1.25E-01	transcription_start_site	-	338931	338931	-1942	19362 AN4584
2366	CONTIG78	340746	341000	1.25	1.25E-01	transcription_start_site	-	338860	338860	-2013	19363 AN4584
2366	CONTIG78	340746	341000	1.25	1.25E-01	transcription_start_site	-	338564	338564	-2309	19364 AN4584
1755	CONTIG78	335176	335470	1.48	5.88E-02	transcription_start_site	+	339770	339770	-4447	19365 AN4585
1755	CONTIG78	335176	335470	1.48	5.88E-02	transcription_start_site	+	340071	340071	-4748	19366 AN4585
2366	CONTIG78	340746	341000	1.25	1.25E-01	transcription_start_site	+	341656	341656	-783	19367 AN4585

2366	CONTIG78	340746	341000	1.25	1.25E-01	transcription_start_site	+	340071	340071	802	19366	AN4585
2366	CONTIG78	340746	341000	1.25	1.25E-01	transcription_start_site	+	339770	339770	1103	19365	AN4585
1756	CONTIG78	343893	344302	1.48	5.88E-02	transcription_start_site	+	344129	344129	-31	19370	AN4586
1756	CONTIG78	343893	344302	1.48	5.88E-02	transcription_start_site	+	343652	343652	445	19369	AN4586
1756	CONTIG78	343893	344302	1.48	5.88E-02	transcription_start_site	+	343575	343575	522	19368	AN4586
2366	CONTIG78	340746	341000	1.25	1.25E-01	transcription_start_site	+	343575	343575	-2702	19368	AN4586
2366	CONTIG78	340746	341000	1.25	1.25E-01	transcription_start_site	+	343652	343652	-2779	19369	AN4586
2366	CONTIG78	340746	341000	1.25	1.25E-01	transcription_start_site	+	344129	344129	-3256	19370	AN4586
1756	CONTIG78	343893	344302	1.48	5.88E-02	transcription_start_site	-	345148	345148	1050	19373	AN4587
1756	CONTIG78	343893	344302	1.48	5.88E-02	transcription_start_site	+	348360	348360	-4262	19374	AN4588
1756	CONTIG78	343893	344302	1.48	5.88E-02	transcription_start_site	+	348581	348581	-4483	19375	AN4588
1050	CONTIG79	7658	7927	1.87	7.85E-03	transcription_start_site	-	4467	4467	-3325	19385	AN4591
1050	CONTIG79	7658	7927	1.87	7.85E-03	transcription_start_site	-	4400	4400	-3392	19386	AN4591
1050	CONTIG79	7658	7927	1.87	7.85E-03	transcription_start_site	-	4317	4317	-3475	19387	AN4591
1050	CONTIG79	7658	7927	1.87	7.85E-03	transcription_start_site	-	4172	4172	-3620	19388	AN4591
1050	CONTIG79	7658	7927	1.87	7.85E-03	transcription_start_site	-	3736	3736	-4056	19389	AN4591
1050	CONTIG79	7658	7927	1.87	7.85E-03	transcription_start_site	-	3452	3452	-4340	19390	AN4591
1050	CONTIG79	7658	7927	1.87	7.85E-03	transcription_start_site	-	3112	3112	-4680	19391	AN4591
2358	CONTIG79	246	500	1.25	9.24E-02	transcription_start_site	+	4821	4821	-4448	19393	AN4592
2358	CONTIG79	246	500	1.25	9.24E-02	transcription_start_site	+	5064	5064	-4691	19394	AN4592
1806	CONTIG79	11038	11387	1.45	4.36E-02	transcription_start_site	-	9042	9042	-2170	19395	AN4593
1050	CONTIG79	7658	7927	1.87	7.85E-03	transcription_start_site	+	9706	9706	-1913	19396	AN4594
1050	CONTIG79	7658	7927	1.87	7.85E-03	transcription_start_site	+	9831	9831	-2038	19397	AN4594
1050	CONTIG79	7658	7927	1.87	7.85E-03	transcription_start_site	+	10139	10139	-2346	19398	AN4594
1806	CONTIG79	11038	11387	1.45	4.36E-02	transcription_start_site	+	10139	10139	1073	19398	AN4594
1806	CONTIG79	11038	11387	1.45	4.36E-02	transcription_start_site	-	11931	11931	718	19402	AN4595
1806	CONTIG79	11038	11387	1.45	4.36E-02	transcription_start_site	-	12032	12032	819	19401	AN4595
1806	CONTIG79	11038	11387	1.45	4.36E-02	transcription_start_site	-	12316	12316	1103	19400	AN4595
1806	CONTIG79	11038	11387	1.45	4.36E-02	transcription_start_site	+	14303	14303	-3090	19406	AN4597
1503	CONTIG79	23853	24127	1.58	2.62E-02	transcription_start_site	-	21942	21942	-2048	19412	AN4598
1503	CONTIG79	23853	24127	1.58	2.62E-02	transcription_start_site	-	21591	21591	-2399	19413	AN4598
1590	CONTIG79	22593	23696	1.54	1.81E-02	transcription_start_site	-	21942	21942	-1202	19412	AN4598
1590	CONTIG79	22593	23696	1.54	1.81E-02	transcription_start_site	-	21591	21591	-1553	19413	AN4598
2002	CONTIG79	25959	26322	1.37	5.88E-02	transcription_start_site	-	21942	21942	-4198	19412	AN4598
2002	CONTIG79	25959	26322	1.37	5.88E-02	transcription_start_site	-	21591	21591	-4549	19413	AN4598
2855	CONTIG79	20638	21202	1.04	1.79E-01	transcription_start_site	-	21591	21591	671	19413	AN4598
2855	CONTIG79	20638	21202	1.04	1.79E-01	transcription_start_site	-	21942	21942	1022	19412	AN4598
744	CONTIG79	30321	30575	2.16	1.80E-03	transcription_start_site	-	28467	28467	-1981	19414	AN4599
744	CONTIG79	30321	30575	2.16	1.80E-03	transcription_start_site	-	28367	28367	-2081	19415	AN4599
744	CONTIG79	30321	30575	2.16	1.80E-03	transcription_start_site	-	27912	27912	-2536	19416	AN4599
744	CONTIG79	30321	30575	2.16	1.80E-03	transcription_start_site	-	27739	27739	-2709	19417	AN4599
744	CONTIG79	30321	30575	2.16	1.80E-03	transcription_start_site	-	27377	27377	-3071	19418	AN4599
1503	CONTIG79	23853	24127	1.58	2.62E-02	transcription_start_site	+	28811	28811	-4821	19419	AN4600
1503	CONTIG79	23853	24127	1.58	2.62E-02	transcription_start_site	+	29072	29072	-5082	19420	AN4600
2002	CONTIG79	25959	26322	1.37	5.88E-02	transcription_start_site	+	28811	28811	-2670	19419	AN4600
2002	CONTIG79	25959	26322	1.37	5.88E-02	transcription_start_site	+	29072	29072	-2931	19420	AN4600
744	CONTIG79	30321	30575	2.16	1.80E-03	transcription_start_site	+	31188	31188	-740	19421	AN4601
744	CONTIG79	30321	30575	2.16	1.80E-03	transcription_start_site	+	32546	32546	-2098	19422	AN4601
744	CONTIG79	30321	30575	2.16	1.80E-03	transcription_start_site	+	35178	35178	-4730	19423	AN4601
2002	CONTIG79	25959	26322	1.37	5.88E-02	transcription_start_site	+	31188	31188	-5047	19421	AN4601
650	CONTIG79	40652	41003	2.24	1.22E-03	transcription_start_site	-	36814	36814	-4013	19424	AN4602
650	CONTIG79	40652	41003	2.24	1.22E-03	transcription_start_site	-	36723	36723	-4104	19425	AN4602
650	CONTIG79	40652	41003	2.24	1.22E-03	transcription_start_site	-	36186	36186	-4641	19426	AN4602
1417	CONTIG79	39153	40042	1.62	7.64E-03	transcription_start_site	-	36814	36814	-2783	19424	AN4602
1417	CONTIG79	39153	40042	1.62	7.64E-03	transcription_start_site	-	36723	36723	-2874	19425	AN4602
1417	CONTIG79	39153	40042	1.62	7.64E-03	transcription_start_site	-	36186	36186	-3411	19426	AN4602
650	CONTIG79	40652	41003	2.24	1.22E-03	transcription_start_site	-	40222	40222	-605	19427	AN4603

650	CONTIG79	40652	41003	2.24	1.22E-03	transcription_start_site	-	39671	39671	-1156	19428 AN4603
1417	CONTIG79	39153	40042	1.62	7.64E-03	transcription_start_site	-	39671	39671	73	19428 AN4603
1417	CONTIG79	39153	40042	1.62	7.64E-03	transcription_start_site	-	40222	40222	624	19427 AN4603
650	CONTIG79	40652	41003	2.24	1.22E-03	transcription_start_site	+	44830	44830	-4002	19434 AN4605
650	CONTIG79	40652	41003	2.24	1.22E-03	transcription_start_site	+	45419	45419	-4591	19435 AN4605
1417	CONTIG79	39153	40042	1.62	7.64E-03	transcription_start_site	+	44830	44830	-5232	19434 AN4605
1351	CONTIG79	66768	67267	1.66	1.84E-02	transcription_start_site	-	65348	65348	-1669	19456 AN4613
2512	CONTIG79	70278	70632	1.2	1.08E-01	transcription_start_site	-	65348	65348	-5107	19456 AN4613
2788	CONTIG79	69393	69657	1.08	1.66E-01	transcription_start_site	-	65348	65348	-4177	19456 AN4613
1351	CONTIG79	66768	67267	1.66	1.84E-02	transcription_start_site	-	66758	66758	-259	19457 AN4614
1351	CONTIG79	66768	67267	1.66	1.84E-02	transcription_start_site	-	66625	66625	-392	19458 AN4614
1351	CONTIG79	66768	67267	1.66	1.84E-02	transcription_start_site	-	66376	66376	-641	19459 AN4614
1351	CONTIG79	66768	67267	1.66	1.84E-02	transcription_start_site	-	66023	66023	-994	19460 AN4614
2512	CONTIG79	70278	70632	1.2	1.08E-01	transcription_start_site	-	66758	66758	-3697	19457 AN4614
2512	CONTIG79	70278	70632	1.2	1.08E-01	transcription_start_site	-	66625	66625	-3830	19458 AN4614
2512	CONTIG79	70278	70632	1.2	1.08E-01	transcription_start_site	-	66376	66376	-4079	19459 AN4614
2512	CONTIG79	70278	70632	1.2	1.08E-01	transcription_start_site	-	66023	66023	-4432	19460 AN4614
2788	CONTIG79	69393	69657	1.08	1.66E-01	transcription_start_site	-	66758	66758	-2767	19457 AN4614
2788	CONTIG79	69393	69657	1.08	1.66E-01	transcription_start_site	-	66625	66625	-2900	19458 AN4614
2788	CONTIG79	69393	69657	1.08	1.66E-01	transcription_start_site	-	66376	66376	-3149	19459 AN4614
2788	CONTIG79	69393	69657	1.08	1.66E-01	transcription_start_site	-	66023	66023	-3502	19460 AN4614
1351	CONTIG79	66768	67267	1.66	1.84E-02	transcription_start_site	+	67480	67480	-462	19461 AN4615
1351	CONTIG79	66768	67267	1.66	1.84E-02	transcription_start_site	+	67551	67551	-533	19462 AN4615
1351	CONTIG79	66768	67267	1.66	1.84E-02	transcription_start_site	+	67905	67905	-887	19463 AN4615
1351	CONTIG79	66768	67267	1.66	1.84E-02	transcription_start_site	+	68141	68141	-1123	19464 AN4615
2512	CONTIG79	70278	70632	1.2	1.08E-01	transcription_start_site	-	70584	70584	129	19468 AN4616
2512	CONTIG79	70278	70632	1.2	1.08E-01	transcription_start_site	-	70773	70773	318	19467 AN4616
2512	CONTIG79	70278	70632	1.2	1.08E-01	transcription_start_site	-	70889	70889	434	19466 AN4616
2512	CONTIG79	70278	70632	1.2	1.08E-01	transcription_start_site	-	71055	71055	600	19465 AN4616
2788	CONTIG79	69393	69657	1.08	1.66E-01	transcription_start_site	-	70584	70584	1059	19468 AN4616
1351	CONTIG79	66768	67267	1.66	1.84E-02	transcription_start_site	+	71505	71505	-4487	19469 AN4617
2512	CONTIG79	70278	70632	1.2	1.08E-01	transcription_start_site	+	71505	71505	-1050	19469 AN4617
2788	CONTIG79	69393	69657	1.08	1.66E-01	transcription_start_site	+	71505	71505	-1980	19469 AN4617
2512	CONTIG79	70278	70632	1.2	1.08E-01	transcription_start_site	+	72525	72525	-2070	19470 AN4618
2512	CONTIG79	70278	70632	1.2	1.08E-01	transcription_start_site	+	72637	72637	-2182	19471 AN4618
2788	CONTIG79	69393	69657	1.08	1.66E-01	transcription_start_site	+	72525	72525	-3000	19470 AN4618
2788	CONTIG79	69393	69657	1.08	1.66E-01	transcription_start_site	+	72637	72637	-3112	19471 AN4618
560	CONTIG79	84826	85250	2.37	8.69E-04	transcription_start_site	+	84301	84301	737	19483 AN4621
560	CONTIG79	84826	85250	2.37	8.69E-04	transcription_start_site	+	83864	83864	1174	19482 AN4621
523	CONTIG79	86626	86920	2.41	5.64E-04	transcription_start_site	+	88024	88024	-1251	19484 AN4622
523	CONTIG79	86626	86920	2.41	5.64E-04	transcription_start_site	+	88647	88647	-1874	19485 AN4622
560	CONTIG79	84826	85250	2.37	8.69E-04	transcription_start_site	+	88024	88024	-2986	19484 AN4622
560	CONTIG79	84826	85250	2.37	8.69E-04	transcription_start_site	+	88647	88647	-3609	19485 AN4622
1105	CONTIG79	87156	87735	1.83	8.81E-03	transcription_start_site	+	88024	88024	-578	19484 AN4622
1105	CONTIG79	87156	87735	1.83	8.81E-03	transcription_start_site	+	88647	88647	-1201	19485 AN4622
523	CONTIG79	86626	86920	2.41	5.64E-04	transcription_start_site	+	90167	90167	-3394	19486 AN4623
523	CONTIG79	86626	86920	2.41	5.64E-04	transcription_start_site	+	90376	90376	-3603	19487 AN4623
523	CONTIG79	86626	86920	2.41	5.64E-04	transcription_start_site	+	90785	90785	-4012	19488 AN4623
523	CONTIG79	86626	86920	2.41	5.64E-04	transcription_start_site	+	91651	91651	-4878	19489 AN4623
560	CONTIG79	84826	85250	2.37	8.69E-04	transcription_start_site	+	90167	90167	-5129	19486 AN4623
1105	CONTIG79	87156	87735	1.83	8.81E-03	transcription_start_site	+	90167	90167	-2721	19486 AN4623
1105	CONTIG79	87156	87735	1.83	8.81E-03	transcription_start_site	+	90376	90376	-2930	19487 AN4623
1105	CONTIG79	87156	87735	1.83	8.81E-03	transcription_start_site	+	90785	90785	-3339	19488 AN4623
1105	CONTIG79	87156	87735	1.83	8.81E-03	transcription_start_site	+	91651	91651	-4205	19489 AN4623
1105	CONTIG79	87156	87735	1.83	8.81E-03	transcription_start_site	+	91961	91961	-4515	19490 AN4623
2359	CONTIG79	106446	106945	1.25	9.24E-02	transcription_start_site	-	102213	102213	-4482	19496 AN4626
2359	CONTIG79	106446	106945	1.25	9.24E-02	transcription_start_site	-	106558	106558	-137	19499 AN4628

2359	CONTIG79	106446	106945	1.25	9.24E-02	transcription_start_site	-	106398	106398	-297	19500 AN4628
2359	CONTIG79	106446	106945	1.25	9.24E-02	transcription_start_site	-	106059	106059	-636	19501 AN4628
2359	CONTIG79	106446	106945	1.25	9.24E-02	transcription_start_site	-	105691	105691	-1004	19502 AN4628
2359	CONTIG79	106446	106945	1.25	9.24E-02	transcription_start_site	-	105520	105520	-1175	19503 AN4628
2359	CONTIG79	106446	106945	1.25	9.24E-02	transcription_start_site	-	104929	104929	-1766	19504 AN4628
2359	CONTIG79	106446	106945	1.25	9.24E-02	transcription_start_site	-	104758	104758	-1937	19505 AN4628
2359	CONTIG79	106446	106945	1.25	9.24E-02	transcription_start_site	+	106683	106683	12	19506 AN4629
2359	CONTIG79	106446	106945	1.25	9.24E-02	transcription_start_site	+	107052	107052	-356	19507 AN4629
2359	CONTIG79	106446	106945	1.25	9.24E-02	transcription_start_site	+	109844	109844	-3148	19508 AN4630
2359	CONTIG79	106446	106945	1.25	9.24E-02	transcription_start_site	+	110428	110428	-3732	19509 AN4630
2359	CONTIG79	106446	106945	1.25	9.24E-02	transcription_start_site	+	110816	110816	-4120	19510 AN4630
1807	CONTIG79	123826	124100	1.45	4.36E-02	transcription_start_site	-	121335	121335	-2628	19521 AN4634
1807	CONTIG79	123826	124100	1.45	4.36E-02	transcription_start_site	-	121028	121028	-2935	19522 AN4634
1807	CONTIG79	123826	124100	1.45	4.36E-02	transcription_start_site	-	120811	120811	-3152	19523 AN4634
1807	CONTIG79	123826	124100	1.45	4.36E-02	transcription_start_site	-	119920	119920	-4043	19524 AN4634
1423	CONTIG79	128417	128761	1.62	2.12E-02	transcription_start_site	-	128609	128609	20	19525 AN4636
1423	CONTIG79	128417	128761	1.62	2.12E-02	transcription_start_site	-	128423	128423	-166	19526 AN4636
1423	CONTIG79	128417	128761	1.62	2.12E-02	transcription_start_site	-	127759	127759	-830	19527 AN4636
2789	CONTIG79	132082	132360	1.08	1.66E-01	transcription_start_site	-	128609	128609	-3612	19525 AN4636
2789	CONTIG79	132082	132360	1.08	1.66E-01	transcription_start_site	-	128423	128423	-3798	19526 AN4636
2789	CONTIG79	132082	132360	1.08	1.66E-01	transcription_start_site	-	127759	127759	-4462	19527 AN4636
1423	CONTIG79	128417	128761	1.62	2.12E-02	transcription_start_site	-	129325	129325	736	19529 AN4637
2789	CONTIG79	132082	132360	1.08	1.66E-01	transcription_start_site	-	131487	131487	-734	19528 AN4637
2789	CONTIG79	132082	132360	1.08	1.66E-01	transcription_start_site	-	129325	129325	-2896	19529 AN4637
1423	CONTIG79	128417	128761	1.62	2.12E-02	transcription_start_site	+	133141	133141	-4552	19530 AN4638
1423	CONTIG79	128417	128761	1.62	2.12E-02	transcription_start_site	+	133472	133472	-4883	19531 AN4638
2789	CONTIG79	132082	132360	1.08	1.66E-01	transcription_start_site	+	133141	133141	-920	19530 AN4638
2789	CONTIG79	132082	132360	1.08	1.66E-01	transcription_start_site	+	133472	133472	-1251	19531 AN4638
1591	CONTIG79	159696	160410	1.54	1.81E-02	transcription_start_site	-	159281	159281	-772	19547 AN4642
1591	CONTIG79	159696	160410	1.54	1.81E-02	transcription_start_site	-	159088	159088	-965	19548 AN4642
1591	CONTIG79	159696	160410	1.54	1.81E-02	transcription_start_site	-	158757	158757	-1296	19549 AN4642
1591	CONTIG79	159696	160410	1.54	1.81E-02	transcription_start_site	-	158555	158555	-1498	19550 AN4642
1591	CONTIG79	159696	160410	1.54	1.81E-02	transcription_start_site	-	158236	158236	-1817	19551 AN4642
1686	CONTIG79	176337	176691	1.5	2.18E-02	transcription_start_site	-	172809	172809	-3705	19566 AN4648
1690	CONTIG79	177457	177726	1.5	3.70E-02	transcription_start_site	-	172809	172809	-4782	19566 AN4648
1686	CONTIG79	176337	176691	1.5	2.18E-02	transcription_start_site	+	176922	176922	-408	19570 AN4650
1686	CONTIG79	176337	176691	1.5	2.18E-02	transcription_start_site	+	177189	177189	-675	19571 AN4650
1687	CONTIG79	177832	179076	1.5	2.18E-02	transcription_start_site	+	177189	177189	1265	19571 AN4650
1687	CONTIG79	177832	179076	1.5	2.18E-02	transcription_start_site	+	176922	176922	1532	19570 AN4650
1690	CONTIG79	177457	177726	1.5	3.70E-02	transcription_start_site	+	177189	177189	402	19571 AN4650
1690	CONTIG79	177457	177726	1.5	3.70E-02	transcription_start_site	+	176922	176922	669	19570 AN4650
2614	CONTIG79	188937	189201	1.16	1.25E-01	transcription_start_site	+	190170	190170	-1101	19572 AN4652
2614	CONTIG79	188937	189201	1.16	1.25E-01	transcription_start_site	+	190292	190292	-1223	19573 AN4652
2513	CONTIG79	192617	193113	1.2	1.08E-01	transcription_start_site	-	192781	192781	-84	19574 AN4653
2513	CONTIG79	192617	193113	1.2	1.08E-01	transcription_start_site	-	192592	192592	-273	19575 AN4653
2513	CONTIG79	192617	193113	1.2	1.08E-01	transcription_start_site	-	192287	192287	-578	19576 AN4653
2513	CONTIG79	192617	193113	1.2	1.08E-01	transcription_start_site	-	191950	191950	-915	19577 AN4653
2513	CONTIG79	192617	193113	1.2	1.08E-01	transcription_start_site	-	194112	194112	1247	19579 AN4654
2215	CONTIG79	201838	202640	1.29	5.52E-02	transcription_start_site	-	201793	201793	-446	19583 AN4657
2215	CONTIG79	201838	202640	1.29	5.52E-02	transcription_start_site	-	201285	201285	-954	19584 AN4657
2215	CONTIG79	201838	202640	1.29	5.52E-02	transcription_start_site	-	200784	200784	-1455	19585 AN4657
2215	CONTIG79	201838	202640	1.29	5.52E-02	transcription_start_site	+	203932	203932	-1693	19586 AN4658
2215	CONTIG79	201838	202640	1.29	5.52E-02	transcription_start_site	+	205750	205750	-3511	19587 AN4659
2215	CONTIG79	201838	202640	1.29	5.52E-02	transcription_start_site	+	205947	205947	-3708	19588 AN4659
2215	CONTIG79	201838	202640	1.29	5.52E-02	transcription_start_site	+	206091	206091	-3852	19589 AN4659
2215	CONTIG79	201838	202640	1.29	5.52E-02	transcription_start_site	+	206341	206341	-4102	19590 AN4659
2215	CONTIG79	201838	202640	1.29	5.52E-02	transcription_start_site	+	207461	207461	-5222	19591 AN4659

1511	CONTIG80	16954	17244	1.58	5.22E-02	transcription_start_site	-	16873	16873	-226	19623	AN4668
1511	CONTIG80	16954	17244	1.58	5.22E-02	transcription_start_site	-	16760	16760	-339	19624	AN4668
1511	CONTIG80	16954	17244	1.58	5.22E-02	transcription_start_site	-	16656	16656	-443	19625	AN4668
1511	CONTIG80	16954	17244	1.58	5.22E-02	transcription_start_site	-	16448	16448	-651	19626	AN4668
1511	CONTIG80	16954	17244	1.58	5.22E-02	transcription_start_site	-	16341	16341	-758	19627	AN4668
1511	CONTIG80	16954	17244	1.58	5.22E-02	transcription_start_site	-	16133	16133	-966	19628	AN4668
1511	CONTIG80	16954	17244	1.58	5.22E-02	transcription_start_site	-	15566	15566	-1533	19629	AN4668
226	CONTIG80	32939	33509	2.89	0.00E+00	transcription_start_site	-	29515	29515	-3709	19644	AN4673
226	CONTIG80	32939	33509	2.89	0.00E+00	transcription_start_site	-	28929	28929	-4295	19645	AN4673
226	CONTIG80	32939	33509	2.89	0.00E+00	transcription_start_site	-	28397	28397	-4827	19646	AN4673
226	CONTIG80	32939	33509	2.89	0.00E+00	transcription_start_site	-	31664	31664	-1560	19647	AN4674
226	CONTIG80	32939	33509	2.89	0.00E+00	transcription_start_site	-	31495	31495	-1729	19648	AN4674
226	CONTIG80	32939	33509	2.89	0.00E+00	transcription_start_site	-	31156	31156	-2068	19649	AN4674
226	CONTIG80	32939	33509	2.89	0.00E+00	transcription_start_site	-	30614	30614	-2610	19650	AN4674
1126	CONTIG80	35402	35826	1.81	2.12E-02	transcription_start_site	-	31664	31664	-3950	19647	AN4674
1126	CONTIG80	35402	35826	1.81	2.12E-02	transcription_start_site	-	31495	31495	-4119	19648	AN4674
1126	CONTIG80	35402	35826	1.81	2.12E-02	transcription_start_site	-	31156	31156	-4458	19649	AN4674
1126	CONTIG80	35402	35826	1.81	2.12E-02	transcription_start_site	-	30614	30614	-5000	19650	AN4674
226	CONTIG80	32939	33509	2.89	0.00E+00	transcription_start_site	+	32486	32486	738	19652	AN4675
226	CONTIG80	32939	33509	2.89	0.00E+00	transcription_start_site	+	32122	32122	1102	19651	AN4675
1126	CONTIG80	35402	35826	1.81	2.12E-02	transcription_start_site	-	36432	36432	818	19656	AN4676
2180	CONTIG80	44042	45216	1.3	6.32E-02	transcription_start_site	-	41021	41021	-3608	19657	AN4677
2180	CONTIG80	44042	45216	1.3	6.32E-02	transcription_start_site	-	40823	40823	-3806	19658	AN4677
47	CONTIG80	46142	46551	3.44	0.00E+00	transcription_start_site	-	44387	44387	-1959	19659	AN4678
657	CONTIG80	47107	47766	2.23	1.22E-03	transcription_start_site	-	44387	44387	-3049	19659	AN4678
2180	CONTIG80	44042	45216	1.3	6.32E-02	transcription_start_site	-	44387	44387	-242	19659	AN4678
47	CONTIG80	46142	46551	3.44	0.00E+00	transcription_start_site	+	48283	48283	-1936	19660	AN4680
47	CONTIG80	46142	46551	3.44	0.00E+00	transcription_start_site	+	48504	48504	-2157	19661	AN4680
47	CONTIG80	46142	46551	3.44	0.00E+00	transcription_start_site	+	48607	48607	-2260	19662	AN4680
657	CONTIG80	47107	47766	2.23	1.22E-03	transcription_start_site	+	48283	48283	-846	19660	AN4680
657	CONTIG80	47107	47766	2.23	1.22E-03	transcription_start_site	+	48504	48504	-1067	19661	AN4680
657	CONTIG80	47107	47766	2.23	1.22E-03	transcription_start_site	+	48607	48607	-1170	19662	AN4680
2180	CONTIG80	44042	45216	1.3	6.32E-02	transcription_start_site	+	48283	48283	-3654	19660	AN4680
2180	CONTIG80	44042	45216	1.3	6.32E-02	transcription_start_site	+	48504	48504	-3875	19661	AN4680
2180	CONTIG80	44042	45216	1.3	6.32E-02	transcription_start_site	+	48607	48607	-3978	19662	AN4680
47	CONTIG80	46142	46551	3.44	0.00E+00	transcription_start_site	+	50646	50646	-4299	19663	AN4681
47	CONTIG80	46142	46551	3.44	0.00E+00	transcription_start_site	+	50760	50760	-4413	19664	AN4681
657	CONTIG80	47107	47766	2.23	1.22E-03	transcription_start_site	+	50646	50646	-3209	19663	AN4681
657	CONTIG80	47107	47766	2.23	1.22E-03	transcription_start_site	+	50760	50760	-3323	19664	AN4681
2063	CONTIG80	53114	53388	1.35	1.08E-01	transcription_start_site	-	53134	53134	-117	19665	AN4682
2063	CONTIG80	53114	53388	1.35	1.08E-01	transcription_start_site	-	52859	52859	-392	19666	AN4682
2063	CONTIG80	53114	53388	1.35	1.08E-01	transcription_start_site	+	53558	53558	-307	19667	AN4683
2063	CONTIG80	53114	53388	1.35	1.08E-01	transcription_start_site	+	53761	53761	-510	19668	AN4683
2063	CONTIG80	53114	53388	1.35	1.08E-01	transcription_start_site	+	53925	53925	-674	19669	AN4683
2063	CONTIG80	53114	53388	1.35	1.08E-01	transcription_start_site	+	54671	54671	-1420	19670	AN4683
2063	CONTIG80	53114	53388	1.35	1.08E-01	transcription_start_site	+	56283	56283	-3032	19671	AN4684
2063	CONTIG80	53114	53388	1.35	1.08E-01	transcription_start_site	+	56453	56453	-3202	19672	AN4684
1728	CONTIG80	58964	59305	1.49	6.84E-02	transcription_start_site	+	59718	59718	-583	19673	AN4685
1728	CONTIG80	58964	59305	1.49	6.84E-02	transcription_start_site	+	59809	59809	-674	19674	AN4685
1728	CONTIG80	58964	59305	1.49	6.84E-02	transcription_start_site	+	59912	59912	-777	19675	AN4685
1728	CONTIG80	58964	59305	1.49	6.84E-02	transcription_start_site	+	60019	60019	-884	19676	AN4685
1728	CONTIG80	58964	59305	1.49	6.84E-02	transcription_start_site	+	60150	60150	-1015	19677	AN4685
1728	CONTIG80	58964	59305	1.49	6.84E-02	transcription_start_site	+	60238	60238	-1103	19678	AN4685
1728	CONTIG80	58964	59305	1.49	6.84E-02	transcription_start_site	+	60424	60424	-1289	19679	AN4685
1728	CONTIG80	58964	59305	1.49	6.84E-02	transcription_start_site	+	62199	62199	-3064	19680	AN4686
1927	CONTIG80	73669	73943	1.4	9.24E-02	transcription_start_site	-	69027	69027	-4779	19689	AN4689
1927	CONTIG80	73669	73943	1.4	9.24E-02	transcription_start_site	-	68875	68875	-4931	19690	AN4689

1927	CONTIG80	73669	73943	1.4	9.24E-02	transcription_start_site	+	74482	74482	-676	19696	AN4691
1927	CONTIG80	73669	73943	1.4	9.24E-02	transcription_start_site	+	74932	74932	-1126	19697	AN4691
1927	CONTIG80	73669	73943	1.4	9.24E-02	transcription_start_site	+	75097	75097	-1291	19698	AN4691
1927	CONTIG80	73669	73943	1.4	9.24E-02	transcription_start_site	+	75587	75587	-1781	19699	AN4691
1927	CONTIG80	73669	73943	1.4	9.24E-02	transcription_start_site	+	78760	78760	-4954	19700	AN4692
1927	CONTIG80	73669	73943	1.4	9.24E-02	transcription_start_site	+	78937	78937	-5131	19701	AN4692
2064	CONTIG80	77719	78203	1.35	1.08E-01	transcription_start_site	+	78760	78760	-799	19700	AN4692
2064	CONTIG80	77719	78203	1.35	1.08E-01	transcription_start_site	+	78937	78937	-976	19701	AN4692
2064	CONTIG80	77719	78203	1.35	1.08E-01	transcription_start_site	+	79864	79864	-1903	19702	AN4692
658	CONTIG80	93153	93526	2.23	1.22E-03	transcription_start_site	-	90854	90854	-2485	19706	AN4695
658	CONTIG80	93153	93526	2.23	1.22E-03	transcription_start_site	-	90700	90700	-2639	19707	AN4695
1313	CONTIG80	101628	102067	1.68	2.18E-02	transcription_start_site	-	98542	98542	-3305	19708	AN4696
1313	CONTIG80	101628	102067	1.68	2.18E-02	transcription_start_site	-	98417	98417	-3430	19709	AN4696
2065	CONTIG80	97363	97627	1.35	1.08E-01	transcription_start_site	-	98417	98417	922	19709	AN4696
2065	CONTIG80	97363	97627	1.35	1.08E-01	transcription_start_site	-	98542	98542	1047	19708	AN4696
2201	CONTIG80	98118	98462	1.3	1.25E-01	transcription_start_site	-	98417	98417	127	19709	AN4696
2201	CONTIG80	98118	98462	1.3	1.25E-01	transcription_start_site	-	98542	98542	252	19708	AN4696
1189	CONTIG80	105612	106041	1.77	2.62E-02	transcription_start_site	-	101522	101522	-4304	19710	AN4697
1189	CONTIG80	105612	106041	1.77	2.62E-02	transcription_start_site	-	101361	101361	-4465	19711	AN4697
1189	CONTIG80	105612	106041	1.77	2.62E-02	transcription_start_site	-	100813	100813	-5013	19712	AN4697
1313	CONTIG80	101628	102067	1.68	2.18E-02	transcription_start_site	-	101522	101522	-325	19710	AN4697
1313	CONTIG80	101628	102067	1.68	2.18E-02	transcription_start_site	-	101361	101361	-486	19711	AN4697
1313	CONTIG80	101628	102067	1.68	2.18E-02	transcription_start_site	-	100813	100813	-1034	19712	AN4697
1313	CONTIG80	101628	102067	1.68	2.18E-02	transcription_start_site	-	100511	100511	-1336	19713	AN4697
76	CONTIG80	109733	110017	3.3	0.00E+00	transcription_start_site	+	110463	110463	-588	19714	AN4698
76	CONTIG80	109733	110017	3.3	0.00E+00	transcription_start_site	+	112214	112214	-2339	19715	AN4698
1189	CONTIG80	105612	106041	1.77	2.62E-02	transcription_start_site	+	110463	110463	-4636	19714	AN4698
483	CONTIG80	119930	120279	2.47	1.54E-03	transcription_start_site	+	123054	123054	-2949	19716	AN4699
483	CONTIG80	119930	120279	2.47	1.54E-03	transcription_start_site	+	124439	124439	-4334	19717	AN4699
483	CONTIG80	119930	120279	2.47	1.54E-03	transcription_start_site	+	125253	125253	-5148	19718	AN4699
763	CONTIG80	122483	123432	2.14	6.61E-03	transcription_start_site	+	123054	123054	-96	19716	AN4699
763	CONTIG80	122483	123432	2.14	6.61E-03	transcription_start_site	+	124439	124439	-1481	19717	AN4699
763	CONTIG80	122483	123432	2.14	6.61E-03	transcription_start_site	+	125253	125253	-2295	19718	AN4699
164	CONTIG80	126688	127102	3.02	0.00E+00	transcription_start_site	+	127282	127282	-387	19719	AN4700
164	CONTIG80	126688	127102	3.02	0.00E+00	transcription_start_site	+	129159	129159	-2264	19720	AN4700
763	CONTIG80	122483	123432	2.14	6.61E-03	transcription_start_site	+	127282	127282	-4324	19719	AN4700
164	CONTIG80	126688	127102	3.02	0.00E+00	transcription_start_site	+	132013	132013	-5118	19723	AN4702
665	CONTIG80	172584	173608	2.23	4.61E-03	transcription_start_site	-	173402	173402	306	19754	AN4713
665	CONTIG80	172584	173608	2.23	4.61E-03	transcription_start_site	-	172762	172762	-334	19755	AN4713
1596	CONTIG80	177014	177503	1.54	3.99E-02	transcription_start_site	-	173402	173402	-3856	19754	AN4713
1596	CONTIG80	177014	177503	1.54	3.99E-02	transcription_start_site	-	172762	172762	-4496	19755	AN4713
1596	CONTIG80	177014	177503	1.54	3.99E-02	transcription_start_site	-	175467	175467	-1791	19756	AN4714
1596	CONTIG80	177014	177503	1.54	3.99E-02	transcription_start_site	-	175115	175115	-2143	19757	AN4714
665	CONTIG80	172584	173608	2.23	4.61E-03	transcription_start_site	+	175617	175617	-2521	19758	AN4715
665	CONTIG80	172584	173608	2.23	4.61E-03	transcription_start_site	+	175918	175918	-2822	19759	AN4715
665	CONTIG80	172584	173608	2.23	4.61E-03	transcription_start_site	+	178020	178020	-4924	19760	AN4716
1596	CONTIG80	177014	177503	1.54	3.99E-02	transcription_start_site	+	178020	178020	-761	19760	AN4716
1596	CONTIG80	177014	177503	1.54	3.99E-02	transcription_start_site	+	179133	179133	-1874	19761	AN4716
2202	CONTIG80	184504	185009	1.3	1.25E-01	transcription_start_site	-	182441	182441	-2315	19762	AN4717
2202	CONTIG80	184504	185009	1.3	1.25E-01	transcription_start_site	-	182126	182126	-2630	19763	AN4717
2202	CONTIG80	184504	185009	1.3	1.25E-01	transcription_start_site	-	181969	181969	-2787	19764	AN4717
2202	CONTIG80	184504	185009	1.3	1.25E-01	transcription_start_site	-	181790	181790	-2966	19765	AN4717
2202	CONTIG80	184504	185009	1.3	1.25E-01	transcription_start_site	-	184406	184406	-350	19766	AN4718
2202	CONTIG80	184504	185009	1.3	1.25E-01	transcription_start_site	-	184069	184069	-687	19767	AN4718
2202	CONTIG80	184504	185009	1.3	1.25E-01	transcription_start_site	-	183871	183871	-885	19768	AN4718
2202	CONTIG80	184504	185009	1.3	1.25E-01	transcription_start_site	-	183762	183762	-994	19769	AN4718
2202	CONTIG80	184504	185009	1.3	1.25E-01	transcription_start_site	-	183578	183578	-1178	19770	AN4718

2202	CONTIG80	184504	185009	1.3	1.25E-01	transcription_start_site	-	183410	183410	-1346	19771	AN4718
2202	CONTIG80	184504	185009	1.3	1.25E-01	transcription_start_site	-	183272	183272	-1484	19772	AN4718
2202	CONTIG80	184504	185009	1.3	1.25E-01	transcription_start_site	-	183133	183133	-1623	19773	AN4718
2202	CONTIG80	184504	185009	1.3	1.25E-01	transcription_start_site	+	184883	184883	-126	19774	AN4719
2202	CONTIG80	184504	185009	1.3	1.25E-01	transcription_start_site	+	187583	187583	-2826	19775	AN4719
2202	CONTIG80	184504	185009	1.3	1.25E-01	transcription_start_site	+	188065	188065	-3308	19776	AN4719
2202	CONTIG80	184504	185009	1.3	1.25E-01	transcription_start_site	+	188545	188545	-3788	19777	AN4719
56	CONTIG80	191860	192134	3.4	0.00E+00	transcription_start_site	-	191056	191056	-941	19778	AN4720
56	CONTIG80	191860	192134	3.4	0.00E+00	transcription_start_site	-	190843	190843	-1154	19779	AN4720
56	CONTIG80	191860	192134	3.4	0.00E+00	transcription_start_site	-	190352	190352	-1645	19780	AN4720
1403	CONTIG80	213093	213502	1.63	4.36E-02	transcription_start_site	-	209998	209998	-3299	19793	AN4725
1729	CONTIG80	214435	214929	1.49	6.84E-02	transcription_start_site	-	209998	209998	-4684	19793	AN4725
1403	CONTIG80	213093	213502	1.63	4.36E-02	transcription_start_site	-	213105	213105	-192	19796	AN4727
1403	CONTIG80	213093	213502	1.63	4.36E-02	transcription_start_site	-	213026	213026	-271	19797	AN4727
1403	CONTIG80	213093	213502	1.63	4.36E-02	transcription_start_site	-	212729	212729	-568	19798	AN4727
1403	CONTIG80	213093	213502	1.63	4.36E-02	transcription_start_site	-	212217	212217	-1080	19799	AN4727
1729	CONTIG80	214435	214929	1.49	6.84E-02	transcription_start_site	-	213105	213105	-1577	19796	AN4727
1729	CONTIG80	214435	214929	1.49	6.84E-02	transcription_start_site	-	213026	213026	-1656	19797	AN4727
1729	CONTIG80	214435	214929	1.49	6.84E-02	transcription_start_site	-	212729	212729	-1953	19798	AN4727
1729	CONTIG80	214435	214929	1.49	6.84E-02	transcription_start_site	-	212217	212217	-2465	19799	AN4727
1403	CONTIG80	213093	213502	1.63	4.36E-02	transcription_start_site	-	214035	214035	737	19803	AN4728
1729	CONTIG80	214435	214929	1.49	6.84E-02	transcription_start_site	-	214773	214773	91	19802	AN4728
1729	CONTIG80	214435	214929	1.49	6.84E-02	transcription_start_site	-	214035	214035	-647	19803	AN4728
2339	CONTIG80	220729	221543	1.26	1.28E-01	transcription_start_site	-	216877	216877	-4259	19800	AN4728
2339	CONTIG80	220729	221543	1.26	1.28E-01	transcription_start_site	-	216272	216272	-4864	19801	AN4728
2339	CONTIG80	220729	221543	1.26	1.28E-01	transcription_start_site	-	218579	218579	-2557	19804	AN4729
2339	CONTIG80	220729	221543	1.26	1.28E-01	transcription_start_site	-	218266	218266	-2870	19805	AN4729
2339	CONTIG80	220729	221543	1.26	1.28E-01	transcription_start_site	-	220642	220642	-494	19806	AN4730
2339	CONTIG80	220729	221543	1.26	1.28E-01	transcription_start_site	-	220413	220413	-723	19807	AN4730
2339	CONTIG80	220729	221543	1.26	1.28E-01	transcription_start_site	-	220238	220238	-898	19808	AN4730
2339	CONTIG80	220729	221543	1.26	1.28E-01	transcription_start_site	+	221268	221268	-132	19809	AN4731
2339	CONTIG80	220729	221543	1.26	1.28E-01	transcription_start_site	+	221341	221341	-205	19810	AN4731
2339	CONTIG80	220729	221543	1.26	1.28E-01	transcription_start_site	+	221851	221851	-715	19811	AN4731
1316	CONTIG80	225769	226105	1.68	3.70E-02	transcription_start_site	-	223794	223794	-2143	19812	AN4732
1316	CONTIG80	225769	226105	1.68	3.70E-02	transcription_start_site	-	223220	223220	-2717	19813	AN4732
1316	CONTIG80	225769	226105	1.68	3.70E-02	transcription_start_site	-	223079	223079	-2858	19814	AN4732
1316	CONTIG80	225769	226105	1.68	3.70E-02	transcription_start_site	+	226461	226461	-524	19815	AN4733
1316	CONTIG80	225769	226105	1.68	3.70E-02	transcription_start_site	+	226542	226542	-605	19816	AN4733
1316	CONTIG80	225769	226105	1.68	3.70E-02	transcription_start_site	+	226664	226664	-727	19817	AN4733
1316	CONTIG80	225769	226105	1.68	3.70E-02	transcription_start_site	+	228215	228215	-2278	19818	AN4733
1316	CONTIG80	225769	226105	1.68	3.70E-02	transcription_start_site	+	228628	228628	-2691	19819	AN4733
1316	CONTIG80	225769	226105	1.68	3.70E-02	transcription_start_site	+	228802	228802	-2865	19820	AN4733
2339	CONTIG80	220729	221543	1.26	1.28E-01	transcription_start_site	+	226461	226461	-5325	19815	AN4733
2339	CONTIG80	220729	221543	1.26	1.28E-01	transcription_start_site	+	226542	226542	-5406	19816	AN4733
1316	CONTIG80	225769	226105	1.68	3.70E-02	transcription_start_site	+	230913	230913	-4976	19821	AN4734
1184	CONTIG80	263932	264426	1.77	1.44E-02	transcription_start_site	-	260156	260156	-4023	19862	AN4743
1184	CONTIG80	263932	264426	1.77	1.44E-02	transcription_start_site	-	260056	260056	-4123	19863	AN4743
1184	CONTIG80	263932	264426	1.77	1.44E-02	transcription_start_site	-	259961	259961	-4218	19864	AN4743
1184	CONTIG80	263932	264426	1.77	1.44E-02	transcription_start_site	-	259839	259839	-4340	19865	AN4743
1184	CONTIG80	263932	264426	1.77	1.44E-02	transcription_start_site	-	259584	259584	-4595	19866	AN4743
1184	CONTIG80	263932	264426	1.77	1.44E-02	transcription_start_site	-	259292	259292	-4887	19867	AN4743
2340	CONTIG80	260795	261144	1.26	1.28E-01	transcription_start_site	-	260156	260156	-813	19862	AN4743
2340	CONTIG80	260795	261144	1.26	1.28E-01	transcription_start_site	-	260056	260056	-913	19863	AN4743
2340	CONTIG80	260795	261144	1.26	1.28E-01	transcription_start_site	-	259961	259961	-1008	19864	AN4743
2340	CONTIG80	260795	261144	1.26	1.28E-01	transcription_start_site	-	259839	259839	-1130	19865	AN4743
2340	CONTIG80	260795	261144	1.26	1.28E-01	transcription_start_site	-	259584	259584	-1385	19866	AN4743
2340	CONTIG80	260795	261144	1.26	1.28E-01	transcription_start_site	-	259292	259292	-1677	19867	AN4743

1184	CONTIG80	263932	264426	1.77	1.44E-02	transcription_start_site	-	263799	263799	-380	19868 AN4744
1184	CONTIG80	263932	264426	1.77	1.44E-02	transcription_start_site	-	263397	263397	-782	19869 AN4744
1184	CONTIG80	263932	264426	1.77	1.44E-02	transcription_start_site	-	263231	263231	-948	19870 AN4744
1184	CONTIG80	263932	264426	1.77	1.44E-02	transcription_start_site	-	263041	263041	-1138	19871 AN4744
1184	CONTIG80	263932	264426	1.77	1.44E-02	transcription_start_site	-	262671	262671	-1508	19872 AN4744
1184	CONTIG80	263932	264426	1.77	1.44E-02	transcription_start_site	-	262377	262377	-1802	19873 AN4744
1184	CONTIG80	263932	264426	1.77	1.44E-02	transcription_start_site	-	261994	261994	-2185	19874 AN4744
2340	CONTIG80	260795	261144	1.26	1.28E-01	transcription_start_site	-	261994	261994	1024	19874 AN4744
1184	CONTIG80	263932	264426	1.77	1.44E-02	transcription_start_site	+	264399	264399	-220	19875 AN4745
1184	CONTIG80	263932	264426	1.77	1.44E-02	transcription_start_site	+	264648	264648	-469	19876 AN4745
1184	CONTIG80	263932	264426	1.77	1.44E-02	transcription_start_site	+	264775	264775	-596	19877 AN4745
1184	CONTIG80	263932	264426	1.77	1.44E-02	transcription_start_site	+	265433	265433	-1254	19878 AN4745
1184	CONTIG80	263932	264426	1.77	1.44E-02	transcription_start_site	+	265688	265688	-1509	19879 AN4745
1184	CONTIG80	263932	264426	1.77	1.44E-02	transcription_start_site	+	266266	266266	-2087	19880 AN4745
1184	CONTIG80	263932	264426	1.77	1.44E-02	transcription_start_site	+	266465	266465	-2286	19881 AN4745
1184	CONTIG80	263932	264426	1.77	1.44E-02	transcription_start_site	+	266614	266614	-2435	19882 AN4745
1184	CONTIG80	263932	264426	1.77	1.44E-02	transcription_start_site	+	266734	266734	-2555	19883 AN4745
2340	CONTIG80	260795	261144	1.26	1.28E-01	transcription_start_site	+	264399	264399	-3429	19875 AN4745
2340	CONTIG80	260795	261144	1.26	1.28E-01	transcription_start_site	+	264648	264648	-3678	19876 AN4745
2340	CONTIG80	260795	261144	1.26	1.28E-01	transcription_start_site	+	264775	264775	-3805	19877 AN4745
2340	CONTIG80	260795	261144	1.26	1.28E-01	transcription_start_site	+	265433	265433	-4463	19878 AN4745
2340	CONTIG80	260795	261144	1.26	1.28E-01	transcription_start_site	+	265688	265688	-4718	19879 AN4745
1184	CONTIG80	263932	264426	1.77	1.44E-02	transcription_start_site	+	269262	269262	-5083	19887 AN4747
2496	CONTIG80	287927	288292	1.21	1.66E-01	transcription_start_site	-	285359	285359	-2750	19910 AN4753
2496	CONTIG80	287927	288292	1.21	1.66E-01	transcription_start_site	-	285045	285045	-3064	19911 AN4753
2496	CONTIG80	287927	288292	1.21	1.66E-01	transcription_start_site	-	284477	284477	-3632	19912 AN4753
2496	CONTIG80	287927	288292	1.21	1.66E-01	transcription_start_site	-	284306	284306	-3803	19913 AN4753
2496	CONTIG80	287927	288292	1.21	1.66E-01	transcription_start_site	+	287177	287177	932	19918 AN4754
2496	CONTIG80	287927	288292	1.21	1.66E-01	transcription_start_site	-	288182	288182	72	19919 AN4755
2496	CONTIG80	287927	288292	1.21	1.66E-01	transcription_start_site	-	287831	287831	-278	19920 AN4755
2496	CONTIG80	287927	288292	1.21	1.66E-01	transcription_start_site	-	287503	287503	-606	19921 AN4755
2496	CONTIG80	287927	288292	1.21	1.66E-01	transcription_start_site	+	293154	293154	-5044	19922 AN4757
2057	CONTIG80	295363	296007	1.35	8.36E-02	transcription_start_site	-	295029	295029	-656	19924 AN4758
2057	CONTIG80	295363	296007	1.35	8.36E-02	transcription_start_site	-	294922	294922	-763	19925 AN4758
2057	CONTIG80	295363	296007	1.35	8.36E-02	transcription_start_site	-	294769	294769	-916	19926 AN4758
2057	CONTIG80	295363	296007	1.35	8.36E-02	transcription_start_site	-	294724	294724	-961	19927 AN4758
2497	CONTIG80	299626	299910	1.21	1.66E-01	transcription_start_site	-	295029	295029	-4739	19924 AN4758
2497	CONTIG80	299626	299910	1.21	1.66E-01	transcription_start_site	-	294922	294922	-4846	19925 AN4758
2497	CONTIG80	299626	299910	1.21	1.66E-01	transcription_start_site	-	294769	294769	-4999	19926 AN4758
2497	CONTIG80	299626	299910	1.21	1.66E-01	transcription_start_site	-	294724	294724	-5044	19927 AN4758
2057	CONTIG80	295363	296007	1.35	8.36E-02	transcription_start_site	+	296518	296518	-833	19928 AN4759
2057	CONTIG80	295363	296007	1.35	8.36E-02	transcription_start_site	+	296587	296587	-902	19929 AN4759
2057	CONTIG80	295363	296007	1.35	8.36E-02	transcription_start_site	+	297259	297259	-1574	19930 AN4759
2497	CONTIG80	299626	299910	1.21	1.66E-01	transcription_start_site	-	300815	300815	1047	19933 AN4760
2497	CONTIG80	299626	299910	1.21	1.66E-01	transcription_start_site	+	301901	301901	-2133	19934 AN4761
2497	CONTIG80	299626	299910	1.21	1.66E-01	transcription_start_site	+	302217	302217	-2449	19935 AN4761
2497	CONTIG80	299626	299910	1.21	1.66E-01	transcription_start_site	+	302559	302559	-2791	19936 AN4761
2497	CONTIG80	299626	299910	1.21	1.66E-01	transcription_start_site	+	302901	302901	-3133	19937 AN4761
2497	CONTIG80	299626	299910	1.21	1.66E-01	transcription_start_site	+	304237	304237	-4469	19938 AN4761
2497	CONTIG80	299626	299910	1.21	1.66E-01	transcription_start_site	+	304429	304429	-4661	19939 AN4761
1404	CONTIG80	325896	326170	1.63	4.36E-02	transcription_start_site	-	326094	326094	61	19956 AN4768
764	CONTIG80	334276	334850	2.14	6.61E-03	transcription_start_site	-	329454	329454	-5109	19957 AN4769
764	CONTIG80	334276	334850	2.14	6.61E-03	transcription_start_site	-	333487	333487	-1076	19963 AN4770
2723	CONTIG81	6376	6725	1.11	1.07E-01	transcription_start_site	-	6116	6116	-434	19966 AN4772
2723	CONTIG81	6376	6725	1.11	1.07E-01	transcription_start_site	-	5746	5746	-804	19967 AN4772
2723	CONTIG81	6376	6725	1.11	1.07E-01	transcription_start_site	-	5343	5343	-1207	19968 AN4772
2723	CONTIG81	6376	6725	1.11	1.07E-01	transcription_start_site	-	4990	4990	-1560	19969 AN4772

2723	CONTIG81	6376	6725	1.11	1.07E-01	transcription_start_site	-	4091	4091	-2459	19970 AN4772
2723	CONTIG81	6376	6725	1.11	1.07E-01	transcription_start_site	-	7699	7699	1148	19978 AN4773
2525	CONTIG81	15996	16490	1.19	9.24E-02	transcription_start_site	-	13030	13030	-3213	19979 AN4774
2525	CONTIG81	15996	16490	1.19	9.24E-02	transcription_start_site	-	12655	12655	-3588	19980 AN4774
2525	CONTIG81	15996	16490	1.19	9.24E-02	transcription_start_site	+	15140	15140	1103	19985 AN4775
1572	CONTIG81	21005	21359	1.55	1.06E-02	transcription_start_site	-	16821	16821	-4361	19986 AN4776
1572	CONTIG81	21005	21359	1.55	1.06E-02	transcription_start_site	-	16243	16243	-4939	19987 AN4776
2400	CONTIG81	18095	18828	1.23	5.52E-02	transcription_start_site	-	16821	16821	-1640	19986 AN4776
2400	CONTIG81	18095	18828	1.23	5.52E-02	transcription_start_site	-	16243	16243	-2218	19987 AN4776
2525	CONTIG81	15996	16490	1.19	9.24E-02	transcription_start_site	-	16243	16243	0	19987 AN4776
2525	CONTIG81	15996	16490	1.19	9.24E-02	transcription_start_site	-	16821	16821	578	19986 AN4776
1572	CONTIG81	21005	21359	1.55	1.06E-02	transcription_start_site	-	18532	18532	-2650	19988 AN4777
1572	CONTIG81	21005	21359	1.55	1.06E-02	transcription_start_site	-	18343	18343	-2839	19989 AN4777
1572	CONTIG81	21005	21359	1.55	1.06E-02	transcription_start_site	-	18214	18214	-2968	19990 AN4777
2400	CONTIG81	18095	18828	1.23	5.52E-02	transcription_start_site	-	18532	18532	70	19988 AN4777
2400	CONTIG81	18095	18828	1.23	5.52E-02	transcription_start_site	-	18343	18343	-118	19989 AN4777
2400	CONTIG81	18095	18828	1.23	5.52E-02	transcription_start_site	-	18214	18214	-247	19990 AN4777
1572	CONTIG81	21005	21359	1.55	1.06E-02	transcription_start_site	-	20673	20673	-509	19991 AN4778
1572	CONTIG81	21005	21359	1.55	1.06E-02	transcription_start_site	-	20468	20468	-714	19992 AN4778
1572	CONTIG81	21005	21359	1.55	1.06E-02	transcription_start_site	-	20319	20319	-863	19993 AN4778
1572	CONTIG81	21005	21359	1.55	1.06E-02	transcription_start_site	-	20111	20111	-1071	19994 AN4778
1572	CONTIG81	21005	21359	1.55	1.06E-02	transcription_start_site	-	20009	20009	-1173	19995 AN4778
1572	CONTIG81	21005	21359	1.55	1.06E-02	transcription_start_site	-	19860	19860	-1322	19996 AN4778
1572	CONTIG81	21005	21359	1.55	1.06E-02	transcription_start_site	-	19146	19146	-2036	19997 AN4778
1765	CONTIG81	24005	24294	1.47	3.08E-02	transcription_start_site	-	20673	20673	-3476	19991 AN4778
1765	CONTIG81	24005	24294	1.47	3.08E-02	transcription_start_site	-	20468	20468	-3681	19992 AN4778
1765	CONTIG81	24005	24294	1.47	3.08E-02	transcription_start_site	-	20319	20319	-3830	19993 AN4778
1765	CONTIG81	24005	24294	1.47	3.08E-02	transcription_start_site	-	20111	20111	-4038	19994 AN4778
1765	CONTIG81	24005	24294	1.47	3.08E-02	transcription_start_site	-	20009	20009	-4140	19995 AN4778
1765	CONTIG81	24005	24294	1.47	3.08E-02	transcription_start_site	-	19860	19860	-4289	19996 AN4778
1765	CONTIG81	24005	24294	1.47	3.08E-02	transcription_start_site	-	19146	19146	-5003	19997 AN4778
1935	CONTIG81	25430	25709	1.39	4.36E-02	transcription_start_site	-	20673	20673	-4896	19991 AN4778
1935	CONTIG81	25430	25709	1.39	4.36E-02	transcription_start_site	-	20468	20468	-5101	19992 AN4778
2400	CONTIG81	18095	18828	1.23	5.52E-02	transcription_start_site	-	19146	19146	684	19997 AN4778
1572	CONTIG81	21005	21359	1.55	1.06E-02	transcription_start_site	-	22259	22259	1077	19999 AN4779
1765	CONTIG81	24005	24294	1.47	3.08E-02	transcription_start_site	-	23012	23012	-1137	19998 AN4779
1765	CONTIG81	24005	24294	1.47	3.08E-02	transcription_start_site	-	22259	22259	-1890	19999 AN4779
1935	CONTIG81	25430	25709	1.39	4.36E-02	transcription_start_site	-	23012	23012	-2557	19998 AN4779
1935	CONTIG81	25430	25709	1.39	4.36E-02	transcription_start_site	-	22259	22259	-3310	19999 AN4779
1572	CONTIG81	21005	21359	1.55	1.06E-02	transcription_start_site	+	24172	24172	-2990	20000 AN4780
1572	CONTIG81	21005	21359	1.55	1.06E-02	transcription_start_site	+	24402	24402	-3220	20001 AN4780
1765	CONTIG81	24005	24294	1.47	3.08E-02	transcription_start_site	+	24172	24172	-22	20000 AN4780
1765	CONTIG81	24005	24294	1.47	3.08E-02	transcription_start_site	+	24402	24402	-252	20001 AN4780
1572	CONTIG81	21005	21359	1.55	1.06E-02	transcription_start_site	+	25521	25521	-4339	20002 AN4781
1765	CONTIG81	24005	24294	1.47	3.08E-02	transcription_start_site	+	25521	25521	-1371	20002 AN4781
1765	CONTIG81	24005	24294	1.47	3.08E-02	transcription_start_site	+	26434	26434	-2284	20003 AN4781
1935	CONTIG81	25430	25709	1.39	4.36E-02	transcription_start_site	+	25521	25521	48	20002 AN4781
1935	CONTIG81	25430	25709	1.39	4.36E-02	transcription_start_site	+	26434	26434	-864	20003 AN4781
1765	CONTIG81	24005	24294	1.47	3.08E-02	transcription_start_site	+	28950	28950	-4800	20009 AN4783
1765	CONTIG81	24005	24294	1.47	3.08E-02	transcription_start_site	+	29055	29055	-4905	20010 AN4783
1765	CONTIG81	24005	24294	1.47	3.08E-02	transcription_start_site	+	29266	29266	-5116	20011 AN4783
1935	CONTIG81	25430	25709	1.39	4.36E-02	transcription_start_site	+	28950	28950	-3380	20009 AN4783
1935	CONTIG81	25430	25709	1.39	4.36E-02	transcription_start_site	+	29055	29055	-3485	20010 AN4783
1935	CONTIG81	25430	25709	1.39	4.36E-02	transcription_start_site	+	29266	29266	-3696	20011 AN4783
1935	CONTIG81	25430	25709	1.39	4.36E-02	transcription_start_site	+	29376	29376	-3806	20012 AN4783
2934	CONTIG81	43207	44011	0.99	1.99E-01	transcription_start_site	-	40030	40030	-3579	20020 AN4785
2934	CONTIG81	43207	44011	0.99	1.99E-01	transcription_start_site	-	39921	39921	-3688	20021 AN4785

2934	CONTIG81	43207	44011	0.99	1.99E-01	transcription_start_site	-	39837	39837	-3772	20022	AN4785
2934	CONTIG81	43207	44011	0.99	1.99E-01	transcription_start_site	-	39671	39671	-3938	20023	AN4785
2934	CONTIG81	43207	44011	0.99	1.99E-01	transcription_start_site	-	39297	39297	-4312	20024	AN4785
2934	CONTIG81	43207	44011	0.99	1.99E-01	transcription_start_site	+	43709	43709	-100	20027	AN4786
2934	CONTIG81	43207	44011	0.99	1.99E-01	transcription_start_site	+	43404	43404	205	20026	AN4786
2934	CONTIG81	43207	44011	0.99	1.99E-01	transcription_start_site	+	44264	44264	-655	20028	AN4786
2934	CONTIG81	43207	44011	0.99	1.99E-01	transcription_start_site	+	47933	47933	-4324	20034	AN4789
2934	CONTIG81	43207	44011	0.99	1.99E-01	transcription_start_site	+	48222	48222	-4613	20035	AN4789
2925	CONTIG81	62419	62838	0.99	1.79E-01	transcription_start_site	-	57750	57750	-4878	20036	AN4790
2925	CONTIG81	62419	62838	0.99	1.79E-01	transcription_start_site	-	57440	57440	-5188	20037	AN4790
2925	CONTIG81	62419	62838	0.99	1.79E-01	transcription_start_site	-	61992	61992	-636	20047	AN4791
2925	CONTIG81	62419	62838	0.99	1.79E-01	transcription_start_site	-	61853	61853	-775	20048	AN4791
2925	CONTIG81	62419	62838	0.99	1.79E-01	transcription_start_site	-	61647	61647	-981	20049	AN4791
2925	CONTIG81	62419	62838	0.99	1.79E-01	transcription_start_site	-	61486	61486	-1142	20050	AN4791
2925	CONTIG81	62419	62838	0.99	1.79E-01	transcription_start_site	-	60717	60717	-1911	20051	AN4791
2925	CONTIG81	62419	62838	0.99	1.79E-01	transcription_start_site	-	60622	60622	-2006	20052	AN4791
2925	CONTIG81	62419	62838	0.99	1.79E-01	transcription_start_site	-	59374	59374	-3254	20053	AN4791
2925	CONTIG81	62419	62838	0.99	1.79E-01	transcription_start_site	-	59199	59199	-3429	20054	AN4791
2925	CONTIG81	62419	62838	0.99	1.79E-01	transcription_start_site	-	59035	59035	-3593	20055	AN4791
2925	CONTIG81	62419	62838	0.99	1.79E-01	transcription_start_site	+	62940	62940	-311	20056	AN4792
2925	CONTIG81	62419	62838	0.99	1.79E-01	transcription_start_site	+	63330	63330	-701	20057	AN4792
2925	CONTIG81	62419	62838	0.99	1.79E-01	transcription_start_site	+	63907	63907	-1278	20058	AN4792
2925	CONTIG81	62419	62838	0.99	1.79E-01	transcription_start_site	+	64421	64421	-1792	20059	AN4792
2925	CONTIG81	62419	62838	0.99	1.79E-01	transcription_start_site	+	65323	65323	-2694	20060	AN4793
2925	CONTIG81	62419	62838	0.99	1.79E-01	transcription_start_site	+	65404	65404	-2775	20061	AN4793
2925	CONTIG81	62419	62838	0.99	1.79E-01	transcription_start_site	+	65724	65724	-3095	20062	AN4793
384	CONTIG81	72689	73178	2.62	0.00E+00	transcription_start_site	+	75419	75419	-2485	20068	AN4795
384	CONTIG81	72689	73178	2.62	0.00E+00	transcription_start_site	+	76131	76131	-3197	20069	AN4795
384	CONTIG81	72689	73178	2.62	0.00E+00	transcription_start_site	+	76655	76655	-3721	20070	AN4795
799	CONTIG81	94668	95084	2.1	1.54E-03	transcription_start_site	-	90633	90633	-4243	20079	AN4800
799	CONTIG81	94668	95084	2.1	1.54E-03	transcription_start_site	-	90358	90358	-4518	20080	AN4800
799	CONTIG81	94668	95084	2.1	1.54E-03	transcription_start_site	-	90196	90196	-4680	20081	AN4800
799	CONTIG81	94668	95084	2.1	1.54E-03	transcription_start_site	-	89783	89783	-5093	20082	AN4800
799	CONTIG81	94668	95084	2.1	1.54E-03	transcription_start_site	-	93223	93223	-1653	20085	AN4801
799	CONTIG81	94668	95084	2.1	1.54E-03	transcription_start_site	-	92620	92620	-2256	20086	AN4801
799	CONTIG81	94668	95084	2.1	1.54E-03	transcription_start_site	-	91323	91323	-3553	20087	AN4801
799	CONTIG81	94668	95084	2.1	1.54E-03	transcription_start_site	+	97601	97601	-2725	20092	AN4803
799	CONTIG81	94668	95084	2.1	1.54E-03	transcription_start_site	+	97685	97685	-2809	20093	AN4803
799	CONTIG81	94668	95084	2.1	1.54E-03	transcription_start_site	+	97791	97791	-2915	20094	AN4803
799	CONTIG81	94668	95084	2.1	1.54E-03	transcription_start_site	+	97890	97890	-3014	20095	AN4803
799	CONTIG81	94668	95084	2.1	1.54E-03	transcription_start_site	+	98050	98050	-3174	20096	AN4803
799	CONTIG81	94668	95084	2.1	1.54E-03	transcription_start_site	+	100068	100068	-5192	20100	AN4805
1113	CONTIG81	104938	105301	1.82	6.61E-03	transcription_start_site	+	104664	104664	455	20110	AN4807
1113	CONTIG81	104938	105301	1.82	6.61E-03	transcription_start_site	+	104072	104072	1047	20109	AN4807
1225	CONTIG81	109593	110017	1.74	1.17E-03	transcription_start_site	+	114650	114650	-4845	20111	AN4809
1225	CONTIG81	109593	110017	1.74	1.17E-03	transcription_start_site	+	114904	114904	-5099	20112	AN4809
1397	CONTIG81	110413	110912	1.63	1.57E-02	transcription_start_site	+	114650	114650	-3987	20111	AN4809
1397	CONTIG81	110413	110912	1.63	1.57E-02	transcription_start_site	+	114904	114904	-4241	20112	AN4809
1397	CONTIG81	110413	110912	1.63	1.57E-02	transcription_start_site	+	115255	115255	-4592	20113	AN4809
1397	CONTIG81	110413	110912	1.63	1.57E-02	transcription_start_site	+	115758	115758	-5095	20114	AN4809
1056	CONTIG81	123153	123432	1.86	5.20E-03	transcription_start_site	-	119339	119339	-3953	20119	AN4810
1056	CONTIG81	123153	123432	1.86	5.20E-03	transcription_start_site	-	118902	118902	-4390	20120	AN4810
1056	CONTIG81	123153	123432	1.86	5.20E-03	transcription_start_site	+	122890	122890	402	20131	AN4812
1056	CONTIG81	123153	123432	1.86	5.20E-03	transcription_start_site	-	124030	124030	737	20135	AN4813
490	CONTIG81	140851	141125	2.46	2.67E-04	transcription_start_site	-	136619	136619	-4369	20142	AN4816
490	CONTIG81	140851	141125	2.46	2.67E-04	transcription_start_site	-	136386	136386	-4602	20143	AN4816
2406	CONTIG81	135976	136330	1.23	6.16E-02	transcription_start_site	-	136386	136386	233	20143	AN4816

2406	CONTIG81	135976	136330	1.23	6.16E-02	transcription_start_site	-	136619	136619	466	20142	AN4816
2869	CONTIG81	137796	138207	1.03	1.50E-01	transcription_start_site	-	136619	136619	-1382	20142	AN4816
2869	CONTIG81	137796	138207	1.03	1.50E-01	transcription_start_site	-	136386	136386	-1615	20143	AN4816
490	CONTIG81	140851	141125	2.46	2.67E-04	transcription_start_site	+	140399	140399	589	20146	AN4817
2406	CONTIG81	135976	136330	1.23	6.16E-02	transcription_start_site	+	138282	138282	-2129	20144	AN4817
2406	CONTIG81	135976	136330	1.23	6.16E-02	transcription_start_site	+	138522	138522	-2369	20145	AN4817
2406	CONTIG81	135976	136330	1.23	6.16E-02	transcription_start_site	+	140399	140399	-4246	20146	AN4817
2869	CONTIG81	137796	138207	1.03	1.50E-01	transcription_start_site	+	138282	138282	-280	20144	AN4817
2869	CONTIG81	137796	138207	1.03	1.50E-01	transcription_start_site	+	138522	138522	-520	20145	AN4817
2869	CONTIG81	137796	138207	1.03	1.50E-01	transcription_start_site	+	140399	140399	-2397	20146	AN4817
490	CONTIG81	140851	141125	2.46	2.67E-04	transcription_start_site	+	141550	141550	-562	20147	AN4818
490	CONTIG81	140851	141125	2.46	2.67E-04	transcription_start_site	+	141932	141932	-944	20148	AN4818
490	CONTIG81	140851	141125	2.46	2.67E-04	transcription_start_site	+	142148	142148	-1160	20149	AN4818
490	CONTIG81	140851	141125	2.46	2.67E-04	transcription_start_site	+	143508	143508	-2520	20150	AN4818
490	CONTIG81	140851	141125	2.46	2.67E-04	transcription_start_site	+	145287	145287	-4299	20151	AN4818
2869	CONTIG81	137796	138207	1.03	1.50E-01	transcription_start_site	+	141550	141550	-3548	20147	AN4818
2869	CONTIG81	137796	138207	1.03	1.50E-01	transcription_start_site	+	141932	141932	-3930	20148	AN4818
2869	CONTIG81	137796	138207	1.03	1.50E-01	transcription_start_site	+	142148	142148	-4146	20149	AN4818
1932	CONTIG81	150318	150957	1.39	2.64E-02	transcription_start_site	-	150623	150623	-14	20157	AN4820
1932	CONTIG81	150318	150957	1.39	2.64E-02	transcription_start_site	-	150764	150764	126	20156	AN4820
1932	CONTIG81	150318	150957	1.39	2.64E-02	transcription_start_site	-	150844	150844	206	20155	AN4820
1932	CONTIG81	150318	150957	1.39	2.64E-02	transcription_start_site	+	152026	152026	-1388	20158	AN4821
1932	CONTIG81	150318	150957	1.39	2.64E-02	transcription_start_site	+	152979	152979	-2341	20159	AN4821
1932	CONTIG81	150318	150957	1.39	2.64E-02	transcription_start_site	+	153579	153579	-2941	20160	AN4821
1932	CONTIG81	150318	150957	1.39	2.64E-02	transcription_start_site	+	154337	154337	-3699	20161	AN4822
1932	CONTIG81	150318	150957	1.39	2.64E-02	transcription_start_site	+	154651	154651	-4013	20162	AN4822
1932	CONTIG81	150318	150957	1.39	2.64E-02	transcription_start_site	+	155203	155203	-4565	20163	AN4822
3030	CONTIG82	30153	30662	0.85	1.08E-01	transcription_start_site	-	25314	25314	-5093	20194	AN4830
3030	CONTIG82	30153	30662	0.85	1.08E-01	transcription_start_site	-	27101	27101	-3306	20196	AN4831
3030	CONTIG82	30153	30662	0.85	1.08E-01	transcription_start_site	-	26923	26923	-3484	20197	AN4831
3030	CONTIG82	30153	30662	0.85	1.08E-01	transcription_start_site	-	26777	26777	-3630	20198	AN4831
3030	CONTIG82	30153	30662	0.85	1.08E-01	transcription_start_site	-	26640	26640	-3767	20199	AN4831
3030	CONTIG82	30153	30662	0.85	1.08E-01	transcription_start_site	-	26023	26023	-4384	20200	AN4831
3030	CONTIG82	30153	30662	0.85	1.08E-01	transcription_start_site	-	29216	29216	-1191	20201	AN4832
3030	CONTIG82	30153	30662	0.85	1.08E-01	transcription_start_site	-	29077	29077	-1330	20202	AN4832
3030	CONTIG82	30153	30662	0.85	1.08E-01	transcription_start_site	-	28946	28946	-1461	20203	AN4832
3030	CONTIG82	30153	30662	0.85	1.08E-01	transcription_start_site	-	28889	28889	-1518	20204	AN4832
3030	CONTIG82	30153	30662	0.85	1.08E-01	transcription_start_site	-	28641	28641	-1766	20205	AN4832
3030	CONTIG82	30153	30662	0.85	1.08E-01	transcription_start_site	-	28535	28535	-1872	20206	AN4832
3030	CONTIG82	30153	30662	0.85	1.08E-01	transcription_start_site	+	29848	29848	559	20207	AN4833
3030	CONTIG82	30153	30662	0.85	1.08E-01	transcription_start_site	+	31968	31968	-1560	20208	AN4834
3030	CONTIG82	30153	30662	0.85	1.08E-01	transcription_start_site	+	32922	32922	-2514	20209	AN4834
2203	CONTIG82	42686	43490	1.29	4.17E-03	transcription_start_site	-	40376	40376	-2712	20214	AN4837
2203	CONTIG82	42686	43490	1.29	4.17E-03	transcription_start_site	-	40229	40229	-2859	20215	AN4837
2203	CONTIG82	42686	43490	1.29	4.17E-03	transcription_start_site	-	39910	39910	-3178	20216	AN4837
2203	CONTIG82	42686	43490	1.29	4.17E-03	transcription_start_site	+	42816	42816	272	20217	AN4838
2203	CONTIG82	42686	43490	1.29	4.17E-03	transcription_start_site	+	43473	43473	-385	20218	AN4838
1554	CONTIG83	1746	2005	1.56	4.36E-02	transcription_start_site	-	1735	1735	-140	20219	AN4839
1554	CONTIG83	1746	2005	1.56	4.36E-02	transcription_start_site	+	4364	4364	-2488	20220	AN4840
1554	CONTIG83	1746	2005	1.56	4.36E-02	transcription_start_site	+	4761	4761	-2885	20221	AN4840
1554	CONTIG83	1746	2005	1.56	4.36E-02	transcription_start_site	+	4896	4896	-3020	20222	AN4840
1554	CONTIG83	1746	2005	1.56	4.36E-02	transcription_start_site	+	5047	5047	-3171	20223	AN4840
1554	CONTIG83	1746	2005	1.56	4.36E-02	transcription_start_site	+	5878	5878	-4002	20224	AN4840
1294	CONTIG83	8701	8995	1.7	2.62E-02	transcription_start_site	+	8500	8500	348	20225	AN4841
1110	CONTIG83	12691	12955	1.83	1.57E-02	transcription_start_site	-	10151	10151	-2672	20226	AN4842
1110	CONTIG83	12691	12955	1.83	1.57E-02	transcription_start_site	-	9679	9679	-3144	20227	AN4842
1110	CONTIG83	12691	12955	1.83	1.57E-02	transcription_start_site	-	9290	9290	-3533	20228	AN4842

1294	CONTIG83	8701	8995	1.7	2.62E-02	transcription_start_site	-	9290	9290	442	20228	AN4842
1294	CONTIG83	8701	8995	1.7	2.62E-02	transcription_start_site	-	9679	9679	831	20227	AN4842
1295	CONTIG83	13066	14300	1.7	2.62E-02	transcription_start_site	-	10151	10151	-3532	20226	AN4842
1295	CONTIG83	13066	14300	1.7	2.62E-02	transcription_start_site	-	9679	9679	-4004	20227	AN4842
1295	CONTIG83	13066	14300	1.7	2.62E-02	transcription_start_site	-	9290	9290	-4393	20228	AN4842
802	CONTIG83	16052	16413	2.1	5.20E-03	transcription_start_site	-	12300	12300	-3932	20229	AN4843
802	CONTIG83	16052	16413	2.1	5.20E-03	transcription_start_site	-	11766	11766	-4466	20230	AN4843
1110	CONTIG83	12691	12955	1.83	1.57E-02	transcription_start_site	-	12300	12300	-523	20229	AN4843
1110	CONTIG83	12691	12955	1.83	1.57E-02	transcription_start_site	-	11766	11766	-1057	20230	AN4843
1110	CONTIG83	12691	12955	1.83	1.57E-02	transcription_start_site	-	10957	10957	-1866	20231	AN4843
1295	CONTIG83	13066	14300	1.7	2.62E-02	transcription_start_site	-	12300	12300	-1383	20229	AN4843
1295	CONTIG83	13066	14300	1.7	2.62E-02	transcription_start_site	-	11766	11766	-1917	20230	AN4843
1295	CONTIG83	13066	14300	1.7	2.62E-02	transcription_start_site	-	10957	10957	-2726	20231	AN4843
802	CONTIG83	16052	16413	2.1	5.20E-03	transcription_start_site	+	16807	16807	-574	20232	AN4845
802	CONTIG83	16052	16413	2.1	5.20E-03	transcription_start_site	+	17114	17114	-881	20233	AN4845
802	CONTIG83	16052	16413	2.1	5.20E-03	transcription_start_site	+	18163	18163	-1930	20234	AN4845
802	CONTIG83	16052	16413	2.1	5.20E-03	transcription_start_site	+	18255	18255	-2022	20235	AN4845
1110	CONTIG83	12691	12955	1.83	1.57E-02	transcription_start_site	+	16807	16807	-3984	20232	AN4845
1110	CONTIG83	12691	12955	1.83	1.57E-02	transcription_start_site	+	17114	17114	-4291	20233	AN4845
1295	CONTIG83	13066	14300	1.7	2.62E-02	transcription_start_site	+	16807	16807	-3124	20232	AN4845
1295	CONTIG83	13066	14300	1.7	2.62E-02	transcription_start_site	+	17114	17114	-3431	20233	AN4845
1295	CONTIG83	13066	14300	1.7	2.62E-02	transcription_start_site	+	18163	18163	-4480	20234	AN4845
1295	CONTIG83	13066	14300	1.7	2.62E-02	transcription_start_site	+	18255	18255	-4572	20235	AN4845
802	CONTIG83	16052	16413	2.1	5.20E-03	transcription_start_site	+	19893	19893	-3660	20236	AN4846
802	CONTIG83	16052	16413	2.1	5.20E-03	transcription_start_site	+	20254	20254	-4021	20237	AN4846
761	CONTIG83	52065	52414	2.14	4.61E-03	transcription_start_site	-	49403	49403	-2836	20258	AN4854
761	CONTIG83	52065	52414	2.14	4.61E-03	transcription_start_site	-	49158	49158	-3081	20259	AN4854
761	CONTIG83	52065	52414	2.14	4.61E-03	transcription_start_site	-	48723	48723	-3516	20260	AN4854
761	CONTIG83	52065	52414	2.14	4.61E-03	transcription_start_site	-	48619	48619	-3620	20261	AN4854
761	CONTIG83	52065	52414	2.14	4.61E-03	transcription_start_site	+	53765	53765	-1525	20262	AN4855
761	CONTIG83	52065	52414	2.14	4.61E-03	transcription_start_site	+	54193	54193	-1953	20263	AN4855
517	CONTIG84	15994	17603	2.42	0.00E+00	transcription_start_site	+	21823	21823	-5024	20266	AN4859
517	CONTIG84	15994	17603	2.42	0.00E+00	transcription_start_site	+	22286	22286	-5487	20267	AN4859
517	CONTIG84	15994	17603	2.42	0.00E+00	transcription_start_site	+	22576	22576	-5777	20268	AN4859
845	CONTIG84	21159	21662	2.06	1.84E-02	transcription_start_site	+	21823	21823	-412	20266	AN4859
845	CONTIG84	21159	21662	2.06	1.84E-02	transcription_start_site	+	22286	22286	-875	20267	AN4859
845	CONTIG84	21159	21662	2.06	1.84E-02	transcription_start_site	+	22576	22576	-1165	20268	AN4859
845	CONTIG84	21159	21662	2.06	1.84E-02	transcription_start_site	+	26591	26591	-5180	20269	AN4860
675	CONTIG84	47565	47989	2.22	4.81E-03	transcription_start_site	+	46608	46608	1169	20289	AN4869
675	CONTIG84	47565	47989	2.22	4.81E-03	transcription_start_site	+	48488	48488	-711	20290	AN4870
675	CONTIG84	47565	47989	2.22	4.81E-03	transcription_start_site	+	48560	48560	-783	20291	AN4870
675	CONTIG84	47565	47989	2.22	4.81E-03	transcription_start_site	+	48854	48854	-1077	20292	AN4870
675	CONTIG84	47565	47989	2.22	4.81E-03	transcription_start_site	+	49076	49076	-1299	20293	AN4870
675	CONTIG84	47565	47989	2.22	4.81E-03	transcription_start_site	+	51428	51428	-3651	20294	AN4871
675	CONTIG84	47565	47989	2.22	4.81E-03	transcription_start_site	+	51521	51521	-3744	20295	AN4871
675	CONTIG84	47565	47989	2.22	4.81E-03	transcription_start_site	+	51681	51681	-3904	20296	AN4871
675	CONTIG84	47565	47989	2.22	4.81E-03	transcription_start_site	+	51996	51996	-4219	20297	AN4871
675	CONTIG84	47565	47989	2.22	4.81E-03	transcription_start_site	+	52073	52073	-4296	20298	AN4871
675	CONTIG84	47565	47989	2.22	4.81E-03	transcription_start_site	+	52172	52172	-4395	20299	AN4871
675	CONTIG84	47565	47989	2.22	4.81E-03	transcription_start_site	+	52276	52276	-4499	20300	AN4871
1482	CONTIG84	54160	54659	1.6	8.01E-02	transcription_start_site	-	53784	53784	-625	20301	AN4872
1482	CONTIG84	54160	54659	1.6	8.01E-02	transcription_start_site	-	53633	53633	-776	20302	AN4872
1482	CONTIG84	54160	54659	1.6	8.01E-02	transcription_start_site	-	53302	53302	-1107	20303	AN4872
1482	CONTIG84	54160	54659	1.6	8.01E-02	transcription_start_site	+	55154	55154	-744	20304	AN4873
1482	CONTIG84	54160	54659	1.6	8.01E-02	transcription_start_site	+	55861	55861	-1451	20305	AN4873
676	CONTIG84	65857	66667	2.22	4.81E-03	transcription_start_site	-	61719	61719	-4543	20312	AN4875
676	CONTIG84	65857	66667	2.22	4.81E-03	transcription_start_site	-	61311	61311	-4951	20313	AN4875

676	CONTIG84	65857	66667	2.22	4.81E-03	transcription_start_site	+	65775	65775	487	20318	AN4876
846	CONTIG84	72242	72507	2.06	1.84E-02	transcription_start_site	-	71575	71575	-799	20319	AN4877
846	CONTIG84	72242	72507	2.06	1.84E-02	transcription_start_site	-	70965	70965	-1409	20320	AN4877
381	CONTIG84	67592	69361	2.63	0.00E+00	transcription_start_site	+	73365	73365	-4888	20321	AN4878
381	CONTIG84	67592	69361	2.63	0.00E+00	transcription_start_site	+	73452	73452	-4975	20322	AN4878
381	CONTIG84	67592	69361	2.63	0.00E+00	transcription_start_site	+	73707	73707	-5230	20323	AN4878
381	CONTIG84	67592	69361	2.63	0.00E+00	transcription_start_site	+	74279	74279	-5802	20324	AN4878
846	CONTIG84	72242	72507	2.06	1.84E-02	transcription_start_site	+	73365	73365	-990	20321	AN4878
846	CONTIG84	72242	72507	2.06	1.84E-02	transcription_start_site	+	73452	73452	-1077	20322	AN4878
846	CONTIG84	72242	72507	2.06	1.84E-02	transcription_start_site	+	73707	73707	-1332	20323	AN4878
846	CONTIG84	72242	72507	2.06	1.84E-02	transcription_start_site	+	74279	74279	-1904	20324	AN4878
846	CONTIG84	72242	72507	2.06	1.84E-02	transcription_start_site	+	75583	75583	-3208	20325	AN4878
473	CONTIG84	77037	77311	2.48	4.61E-03	transcription_start_site	+	78668	78668	-1494	20326	AN4879
473	CONTIG84	77037	77311	2.48	4.61E-03	transcription_start_site	+	78790	78790	-1616	20327	AN4879
1144	CONTIG84	83942	84441	1.8	4.36E-02	transcription_start_site	-	82800	82800	-1391	20328	AN4880
1144	CONTIG84	83942	84441	1.8	4.36E-02	transcription_start_site	-	82427	82427	-1764	20329	AN4880
1144	CONTIG84	83942	84441	1.8	4.36E-02	transcription_start_site	-	82275	82275	-1916	20330	AN4880
1144	CONTIG84	83942	84441	1.8	4.36E-02	transcription_start_site	-	80485	80485	-3706	20331	AN4880
1144	CONTIG84	83942	84441	1.8	4.36E-02	transcription_start_site	-	80303	80303	-3888	20332	AN4880
1145	CONTIG84	85502	85936	1.8	4.36E-02	transcription_start_site	-	82800	82800	-2919	20328	AN4880
1145	CONTIG84	85502	85936	1.8	4.36E-02	transcription_start_site	-	82427	82427	-3292	20329	AN4880
1145	CONTIG84	85502	85936	1.8	4.36E-02	transcription_start_site	-	82275	82275	-3444	20330	AN4880
1144	CONTIG84	83942	84441	1.8	4.36E-02	transcription_start_site	+	84984	84984	-792	20333	AN4881
1144	CONTIG84	83942	84441	1.8	4.36E-02	transcription_start_site	+	85105	85105	-913	20334	AN4881
1145	CONTIG84	85502	85936	1.8	4.36E-02	transcription_start_site	+	85105	85105	614	20334	AN4881
1145	CONTIG84	85502	85936	1.8	4.36E-02	transcription_start_site	+	84984	84984	735	20333	AN4881
1145	CONTIG84	85502	85936	1.8	4.36E-02	transcription_start_site	-	86734	86734	1015	20338	AN4882
1580	CONTIG84	112505	112940	1.55	9.24E-02	transcription_start_site	-	109397	109397	-3325	20350	AN4887
1580	CONTIG84	112505	112940	1.55	9.24E-02	transcription_start_site	-	112409	112409	-313	20353	AN4888
1580	CONTIG84	112505	112940	1.55	9.24E-02	transcription_start_site	-	112242	112242	-480	20354	AN4888
1580	CONTIG84	112505	112940	1.55	9.24E-02	transcription_start_site	-	112101	112101	-621	20355	AN4888
1580	CONTIG84	112505	112940	1.55	9.24E-02	transcription_start_site	-	110821	110821	-1901	20356	AN4888
1580	CONTIG84	112505	112940	1.55	9.24E-02	transcription_start_site	-	110562	110562	-2160	20357	AN4888
1580	CONTIG84	112505	112940	1.55	9.24E-02	transcription_start_site	+	112977	112977	-254	20358	AN4889
1580	CONTIG84	112505	112940	1.55	9.24E-02	transcription_start_site	+	113104	113104	-381	20359	AN4889
1580	CONTIG84	112505	112940	1.55	9.24E-02	transcription_start_site	+	113295	113295	-572	20360	AN4889
1580	CONTIG84	112505	112940	1.55	9.24E-02	transcription_start_site	+	113505	113505	-782	20361	AN4889
1580	CONTIG84	112505	112940	1.55	9.24E-02	transcription_start_site	+	113779	113779	-1056	20362	AN4889
1580	CONTIG84	112505	112940	1.55	9.24E-02	transcription_start_site	+	113969	113969	-1246	20363	AN4889
1580	CONTIG84	112505	112940	1.55	9.24E-02	transcription_start_site	+	116528	116528	-3805	20364	AN4890
1580	CONTIG84	112505	112940	1.55	9.24E-02	transcription_start_site	+	116678	116678	-3955	20365	AN4890
1580	CONTIG84	112505	112940	1.55	9.24E-02	transcription_start_site	+	117063	117063	-4340	20366	AN4890
1581	CONTIG84	134412	134837	1.55	9.24E-02	transcription_start_site	-	134770	134770	145	20383	AN4894
1581	CONTIG84	134412	134837	1.55	9.24E-02	transcription_start_site	-	133649	133649	-975	20384	AN4894
1581	CONTIG84	134412	134837	1.55	9.24E-02	transcription_start_site	-	132474	132474	-2150	20385	AN4894
1581	CONTIG84	134412	134837	1.55	9.24E-02	transcription_start_site	-	131735	131735	-2889	20386	AN4894
1581	CONTIG84	134412	134837	1.55	9.24E-02	transcription_start_site	-	131510	131510	-3114	20387	AN4894
1581	CONTIG84	134412	134837	1.55	9.24E-02	transcription_start_site	+	135389	135389	-764	20388	AN4895
1581	CONTIG84	134412	134837	1.55	9.24E-02	transcription_start_site	+	135525	135525	-900	20389	AN4895
118	CONTIG84	141618	141962	3.15	2.39E-04	transcription_start_site	-	138755	138755	-3035	20390	AN4896
118	CONTIG84	141618	141962	3.15	2.39E-04	transcription_start_site	-	138519	138519	-3271	20391	AN4896
118	CONTIG84	141618	141962	3.15	2.39E-04	transcription_start_site	-	138202	138202	-3588	20392	AN4896
2243	CONTIG84	142593	143546	1.29	1.79E-01	transcription_start_site	-	138755	138755	-4314	20390	AN4896
2243	CONTIG84	142593	143546	1.29	1.79E-01	transcription_start_site	-	138519	138519	-4550	20391	AN4896
2243	CONTIG84	142593	143546	1.29	1.79E-01	transcription_start_site	-	138202	138202	-4867	20392	AN4896
118	CONTIG84	141618	141962	3.15	2.39E-04	transcription_start_site	+	142558	142558	-768	20393	AN4897
2243	CONTIG84	142593	143546	1.29	1.79E-01	transcription_start_site	+	142558	142558	511	20393	AN4897

118	CONTIG84	141618	141962	3.15	2.39E-04	transcription_start_site	+	144006	144006	-2216	20394	AN4898
118	CONTIG84	141618	141962	3.15	2.39E-04	transcription_start_site	+	144091	144091	-2301	20395	AN4898
118	CONTIG84	141618	141962	3.15	2.39E-04	transcription_start_site	+	144489	144489	-2699	20396	AN4898
118	CONTIG84	141618	141962	3.15	2.39E-04	transcription_start_site	+	144847	144847	-3057	20397	AN4898
118	CONTIG84	141618	141962	3.15	2.39E-04	transcription_start_site	+	144979	144979	-3189	20398	AN4898
118	CONTIG84	141618	141962	3.15	2.39E-04	transcription_start_site	+	145585	145585	-3795	20399	AN4898
118	CONTIG84	141618	141962	3.15	2.39E-04	transcription_start_site	+	146563	146563	-4773	20400	AN4898
2243	CONTIG84	142593	143546	1.29	1.79E-01	transcription_start_site	+	144006	144006	-936	20394	AN4898
2243	CONTIG84	142593	143546	1.29	1.79E-01	transcription_start_site	+	144091	144091	-1021	20395	AN4898
2243	CONTIG84	142593	143546	1.29	1.79E-01	transcription_start_site	+	144489	144489	-1419	20396	AN4898
2243	CONTIG84	142593	143546	1.29	1.79E-01	transcription_start_site	+	144847	144847	-1777	20397	AN4898
2243	CONTIG84	142593	143546	1.29	1.79E-01	transcription_start_site	+	144979	144979	-1909	20398	AN4898
2243	CONTIG84	142593	143546	1.29	1.79E-01	transcription_start_site	+	145585	145585	-2515	20399	AN4898
2243	CONTIG84	142593	143546	1.29	1.79E-01	transcription_start_site	+	146563	146563	-3493	20400	AN4898
322	CONTIG84	149259	149619	2.73	1.54E-03	transcription_start_site	-	148476	148476	-963	20401	AN4899
322	CONTIG84	149259	149619	2.73	1.54E-03	transcription_start_site	-	148184	148184	-1255	20402	AN4899
322	CONTIG84	149259	149619	2.73	1.54E-03	transcription_start_site	-	147322	147322	-2117	20403	AN4899
1483	CONTIG84	151671	151940	1.6	8.01E-02	transcription_start_site	-	148476	148476	-3329	20401	AN4899
1483	CONTIG84	151671	151940	1.6	8.01E-02	transcription_start_site	-	148184	148184	-3621	20402	AN4899
1483	CONTIG84	151671	151940	1.6	8.01E-02	transcription_start_site	-	147322	147322	-4483	20403	AN4899
1484	CONTIG84	152781	153275	1.6	8.01E-02	transcription_start_site	-	148476	148476	-4552	20401	AN4899
1484	CONTIG84	152781	153275	1.6	8.01E-02	transcription_start_site	-	148184	148184	-4844	20402	AN4899
1702	CONTIG84	148144	148718	1.5	9.26E-02	transcription_start_site	-	148476	148476	45	20401	AN4899
1702	CONTIG84	148144	148718	1.5	9.26E-02	transcription_start_site	-	148184	148184	-247	20402	AN4899
1702	CONTIG84	148144	148718	1.5	9.26E-02	transcription_start_site	-	147322	147322	-1109	20403	AN4899
322	CONTIG84	149259	149619	2.73	1.54E-03	transcription_start_site	+	150842	150842	-1403	20404	AN4900
322	CONTIG84	149259	149619	2.73	1.54E-03	transcription_start_site	+	151360	151360	-1921	20405	AN4900
322	CONTIG84	149259	149619	2.73	1.54E-03	transcription_start_site	+	151630	151630	-2191	20406	AN4900
322	CONTIG84	149259	149619	2.73	1.54E-03	transcription_start_site	+	152180	152180	-2741	20407	AN4900
1483	CONTIG84	151671	151940	1.6	8.01E-02	transcription_start_site	+	151630	151630	175	20406	AN4900
1483	CONTIG84	151671	151940	1.6	8.01E-02	transcription_start_site	+	152180	152180	-374	20407	AN4900
1483	CONTIG84	151671	151940	1.6	8.01E-02	transcription_start_site	+	151360	151360	445	20405	AN4900
1483	CONTIG84	151671	151940	1.6	8.01E-02	transcription_start_site	+	150842	150842	963	20404	AN4900
1484	CONTIG84	152781	153275	1.6	8.01E-02	transcription_start_site	+	152180	152180	848	20407	AN4900
1702	CONTIG84	148144	148718	1.5	9.26E-02	transcription_start_site	+	150842	150842	-2411	20404	AN4900
1702	CONTIG84	148144	148718	1.5	9.26E-02	transcription_start_site	+	151360	151360	-2929	20405	AN4900
1702	CONTIG84	148144	148718	1.5	9.26E-02	transcription_start_site	+	151630	151630	-3199	20406	AN4900
1702	CONTIG84	148144	148718	1.5	9.26E-02	transcription_start_site	+	152180	152180	-3749	20407	AN4900
1706	CONTIG84	161116	161450	1.5	1.08E-01	transcription_start_site	-	158042	158042	-3241	20408	AN4901
1706	CONTIG84	161116	161450	1.5	1.08E-01	transcription_start_site	-	157732	157732	-3551	20409	AN4901
1706	CONTIG84	161116	161450	1.5	1.08E-01	transcription_start_site	-	157276	157276	-4007	20410	AN4901
1706	CONTIG84	161116	161450	1.5	1.08E-01	transcription_start_site	-	156220	156220	-5063	20411	AN4901
2245	CONTIG84	158251	158535	1.29	1.93E-01	transcription_start_site	-	158042	158042	-351	20408	AN4901
2245	CONTIG84	158251	158535	1.29	1.93E-01	transcription_start_site	-	157732	157732	-661	20409	AN4901
2245	CONTIG84	158251	158535	1.29	1.93E-01	transcription_start_site	-	157276	157276	-1117	20410	AN4901
2245	CONTIG84	158251	158535	1.29	1.93E-01	transcription_start_site	-	156220	156220	-2173	20411	AN4901
2245	CONTIG84	158251	158535	1.29	1.93E-01	transcription_start_site	-	156041	156041	-2352	20412	AN4901
2097	CONTIG84	153996	154485	1.34	1.66E-01	transcription_start_site	+	159242	159242	-5001	20413	AN4902
2245	CONTIG84	158251	158535	1.29	1.93E-01	transcription_start_site	+	159242	159242	-849	20413	AN4902
2245	CONTIG84	158251	158535	1.29	1.93E-01	transcription_start_site	+	159689	159689	-1296	20414	AN4902
1706	CONTIG84	161116	161450	1.5	1.08E-01	transcription_start_site	-	161583	161583	300	20416	AN4903
1706	CONTIG84	161116	161450	1.5	1.08E-01	transcription_start_site	-	162149	162149	866	20415	AN4903
1961	CONTIG84	172135	172479	1.39	1.44E-01	transcription_start_site	-	168821	168821	-3486	20422	AN4905
1961	CONTIG84	172135	172479	1.39	1.44E-01	transcription_start_site	-	168537	168537	-3770	20423	AN4905
1961	CONTIG84	172135	172479	1.39	1.44E-01	transcription_start_site	-	168433	168433	-3874	20424	AN4905
1961	CONTIG84	172135	172479	1.39	1.44E-01	transcription_start_site	-	171357	171357	-950	20425	AN4906
1961	CONTIG84	172135	172479	1.39	1.44E-01	transcription_start_site	-	170646	170646	-1661	20426	AN4906

1961	CONTIG84	172135	172479	1.39	1.44E-01	transcription_start_site	-	170538	170538	-1769	20427	AN4906
2098	CONTIG84	181064	181496	1.34	1.66E-01	transcription_start_site	-	176085	176085	-5195	20428	AN4907
1302	CONTIG84	185329	186432	1.7	5.88E-02	transcription_start_site	-	180837	180837	-5043	20429	AN4908
1302	CONTIG84	185329	186432	1.7	5.88E-02	transcription_start_site	-	180751	180751	-5129	20430	AN4908
1302	CONTIG84	185329	186432	1.7	5.88E-02	transcription_start_site	-	180619	180619	-5261	20431	AN4908
1302	CONTIG84	185329	186432	1.7	5.88E-02	transcription_start_site	-	180505	180505	-5375	20432	AN4908
2098	CONTIG84	181064	181496	1.34	1.66E-01	transcription_start_site	-	180837	180837	-443	20429	AN4908
2098	CONTIG84	181064	181496	1.34	1.66E-01	transcription_start_site	-	180751	180751	-529	20430	AN4908
2098	CONTIG84	181064	181496	1.34	1.66E-01	transcription_start_site	-	180619	180619	-661	20431	AN4908
2098	CONTIG84	181064	181496	1.34	1.66E-01	transcription_start_site	-	180505	180505	-775	20432	AN4908
2098	CONTIG84	181064	181496	1.34	1.66E-01	transcription_start_site	-	179254	179254	-2026	20433	AN4908
1302	CONTIG84	185329	186432	1.7	5.88E-02	transcription_start_site	-	182141	182141	-3739	20434	AN4909
1302	CONTIG84	185329	186432	1.7	5.88E-02	transcription_start_site	-	182053	182053	-3827	20435	AN4909
1302	CONTIG84	185329	186432	1.7	5.88E-02	transcription_start_site	-	181919	181919	-3961	20436	AN4909
1302	CONTIG84	185329	186432	1.7	5.88E-02	transcription_start_site	-	181588	181588	-4292	20437	AN4909
1302	CONTIG84	185329	186432	1.7	5.88E-02	transcription_start_site	-	181367	181367	-4513	20438	AN4909
2098	CONTIG84	181064	181496	1.34	1.66E-01	transcription_start_site	-	181367	181367	87	20438	AN4909
2098	CONTIG84	181064	181496	1.34	1.66E-01	transcription_start_site	-	181588	181588	308	20437	AN4909
2098	CONTIG84	181064	181496	1.34	1.66E-01	transcription_start_site	-	181919	181919	639	20436	AN4909
2098	CONTIG84	181064	181496	1.34	1.66E-01	transcription_start_site	-	182053	182053	773	20435	AN4909
2098	CONTIG84	181064	181496	1.34	1.66E-01	transcription_start_site	-	182141	182141	861	20434	AN4909
1302	CONTIG84	185329	186432	1.7	5.88E-02	transcription_start_site	+	184558	184558	1322	20443	AN4910
2098	CONTIG84	181064	181496	1.34	1.66E-01	transcription_start_site	+	183124	183124	-1844	20439	AN4910
2098	CONTIG84	181064	181496	1.34	1.66E-01	transcription_start_site	+	183446	183446	-2166	20440	AN4910
2098	CONTIG84	181064	181496	1.34	1.66E-01	transcription_start_site	+	183864	183864	-2584	20441	AN4910
2098	CONTIG84	181064	181496	1.34	1.66E-01	transcription_start_site	+	184167	184167	-2887	20442	AN4910
2098	CONTIG84	181064	181496	1.34	1.66E-01	transcription_start_site	+	184558	184558	-3278	20443	AN4910
1302	CONTIG84	185329	186432	1.7	5.88E-02	transcription_start_site	+	186048	186048	-167	20445	AN4911
1302	CONTIG84	185329	186432	1.7	5.88E-02	transcription_start_site	+	185690	185690	190	20444	AN4911
2098	CONTIG84	181064	181496	1.34	1.66E-01	transcription_start_site	+	185690	185690	-4410	20444	AN4911
2098	CONTIG84	181064	181496	1.34	1.66E-01	transcription_start_site	+	186048	186048	-4768	20445	AN4911
795	CONTIG84	208586	208925	2.11	1.57E-02	transcription_start_site	-	208991	208991	235	20470	AN4917
795	CONTIG84	208586	208925	2.11	1.57E-02	transcription_start_site	-	209570	209570	814	20469	AN4917
795	CONTIG84	208586	208925	2.11	1.57E-02	transcription_start_site	+	210243	210243	-1487	20471	AN4918
795	CONTIG84	208586	208925	2.11	1.57E-02	transcription_start_site	+	210552	210552	-1796	20472	AN4918
1222	CONTIG84	216996	217415	1.75	3.33E-02	transcription_start_site	-	212185	212185	-5020	20473	AN4919
1222	CONTIG84	216996	217415	1.75	3.33E-02	transcription_start_site	-	216620	216620	-585	20475	AN4920
1222	CONTIG84	216996	217415	1.75	3.33E-02	transcription_start_site	-	215670	215670	-1535	20476	AN4920
1222	CONTIG84	216996	217415	1.75	3.33E-02	transcription_start_site	-	215186	215186	-2019	20477	AN4920
1222	CONTIG84	216996	217415	1.75	3.33E-02	transcription_start_site	-	214994	214994	-2211	20478	AN4920
1222	CONTIG84	216996	217415	1.75	3.33E-02	transcription_start_site	+	220432	220432	-3226	20479	AN4921
1222	CONTIG84	216996	217415	1.75	3.33E-02	transcription_start_site	+	221610	221610	-4404	20480	AN4921
2246	CONTIG84	227487	227981	1.29	1.93E-01	transcription_start_site	-	224496	224496	-3238	20481	AN4922
2246	CONTIG84	227487	227981	1.29	1.93E-01	transcription_start_site	-	223551	223551	-4183	20482	AN4922
2246	CONTIG84	227487	227981	1.29	1.93E-01	transcription_start_site	-	227439	227439	-295	20483	AN4923
2246	CONTIG84	227487	227981	1.29	1.93E-01	transcription_start_site	-	227221	227221	-513	20484	AN4923
2246	CONTIG84	227487	227981	1.29	1.93E-01	transcription_start_site	+	231186	231186	-3452	20487	AN4925
1962	CONTIG84	238217	238626	1.39	1.44E-01	transcription_start_site	-	238637	238637	215	20489	AN4926
1962	CONTIG84	238217	238626	1.39	1.44E-01	transcription_start_site	-	237726	237726	-695	20490	AN4926
1962	CONTIG84	238217	238626	1.39	1.44E-01	transcription_start_site	-	236108	236108	-2313	20491	AN4926
1962	CONTIG84	238217	238626	1.39	1.44E-01	transcription_start_site	+	242652	242652	-4230	20495	AN4928
729	CONTIG84	249231	249880	2.17	4.13E-03	transcription_start_site	-	247347	247347	-2208	20496	AN4929
729	CONTIG84	249231	249880	2.17	4.13E-03	transcription_start_site	-	246358	246358	-3197	20497	AN4929
1223	CONTIG84	248029	248391	1.75	5.22E-02	transcription_start_site	-	247347	247347	-863	20496	AN4929
1223	CONTIG84	248029	248391	1.75	5.22E-02	transcription_start_site	-	246358	246358	-1852	20497	AN4929
729	CONTIG84	249231	249880	2.17	4.13E-03	transcription_start_site	+	253493	253493	-3937	20498	AN4930
729	CONTIG84	249231	249880	2.17	4.13E-03	transcription_start_site	+	254637	254637	-5081	20499	AN4930

136	CONTIG84	264979	265628	3.09	2.16E-04	transcription_start_site	-	263173	263173	-2130	20509	AN4935
136	CONTIG84	264979	265628	3.09	2.16E-04	transcription_start_site	-	262460	262460	-2843	20510	AN4935
446	CONTIG84	263869	264138	2.53	3.61E-03	transcription_start_site	-	263173	263173	-830	20509	AN4935
446	CONTIG84	263869	264138	2.53	3.61E-03	transcription_start_site	-	262460	262460	-1543	20510	AN4935
955	CONTIG84	263034	263384	1.96	2.62E-02	transcription_start_site	-	263173	263173	-36	20509	AN4935
955	CONTIG84	263034	263384	1.96	2.62E-02	transcription_start_site	-	262460	262460	-749	20510	AN4935
383	CONTIG84	270766	271030	2.63	2.22E-03	transcription_start_site	-	268912	268912	-1986	20511	AN4936
383	CONTIG84	270766	271030	2.63	2.22E-03	transcription_start_site	-	268165	268165	-2733	20512	AN4936
383	CONTIG84	270766	271030	2.63	2.22E-03	transcription_start_site	-	267905	267905	-2993	20513	AN4936
136	CONTIG84	264979	265628	3.09	2.16E-04	transcription_start_site	+	269374	269374	-4070	20514	AN4937
136	CONTIG84	264979	265628	3.09	2.16E-04	transcription_start_site	+	269462	269462	-4158	20515	AN4937
136	CONTIG84	264979	265628	3.09	2.16E-04	transcription_start_site	+	269616	269616	-4312	20516	AN4937
383	CONTIG84	270766	271030	2.63	2.22E-03	transcription_start_site	-	271664	271664	766	20518	AN4938
383	CONTIG84	270766	271030	2.63	2.22E-03	transcription_start_site	-	271860	271860	962	20517	AN4938
383	CONTIG84	270766	271030	2.63	2.22E-03	transcription_start_site	+	274571	274571	-3673	20519	AN4939
383	CONTIG84	270766	271030	2.63	2.22E-03	transcription_start_site	+	274638	274638	-3740	20520	AN4939
383	CONTIG84	270766	271030	2.63	2.22E-03	transcription_start_site	+	274731	274731	-3833	20521	AN4939
383	CONTIG84	270766	271030	2.63	2.22E-03	transcription_start_site	+	275043	275043	-4145	20522	AN4939
474	CONTIG84	277746	278155	2.48	4.61E-03	transcription_start_site	-	277490	277490	-460	20523	AN4940
474	CONTIG84	277746	278155	2.48	4.61E-03	transcription_start_site	-	277350	277350	-600	20524	AN4940
474	CONTIG84	277746	278155	2.48	4.61E-03	transcription_start_site	-	277248	277248	-702	20525	AN4940
474	CONTIG84	277746	278155	2.48	4.61E-03	transcription_start_site	-	277054	277054	-896	20526	AN4940
474	CONTIG84	277746	278155	2.48	4.61E-03	transcription_start_site	-	276214	276214	-1736	20527	AN4940
474	CONTIG84	277746	278155	2.48	4.61E-03	transcription_start_site	+	278273	278273	-322	20528	AN4941
474	CONTIG84	277746	278155	2.48	4.61E-03	transcription_start_site	+	278388	278388	-437	20529	AN4941
474	CONTIG84	277746	278155	2.48	4.61E-03	transcription_start_site	+	278697	278697	-746	20530	AN4941
956	CONTIG84	285836	286342	1.96	2.62E-02	transcription_start_site	-	283294	283294	-2795	20537	AN4943
956	CONTIG84	285836	286342	1.96	2.62E-02	transcription_start_site	+	286718	286718	-629	20540	AN4945
956	CONTIG84	285836	286342	1.96	2.62E-02	transcription_start_site	+	289608	289608	-3519	20541	AN4946
956	CONTIG84	285836	286342	1.96	2.62E-02	transcription_start_site	+	289824	289824	-3735	20542	AN4946
956	CONTIG84	285836	286342	1.96	2.62E-02	transcription_start_site	+	290476	290476	-4387	20543	AN4947
956	CONTIG84	285836	286342	1.96	2.62E-02	transcription_start_site	+	290670	290670	-4581	20544	AN4947
1582	CONTIG84	328956	329380	1.55	9.24E-02	transcription_start_site	-	324080	324080	-5088	20579	AN4960
1582	CONTIG84	328956	329380	1.55	9.24E-02	transcription_start_site	-	328418	328418	-750	20592	AN4963
2247	CONTIG84	334666	335080	1.29	1.93E-01	transcription_start_site	-	331784	331784	-3089	20586	AN4963
2247	CONTIG84	334666	335080	1.29	1.93E-01	transcription_start_site	-	331687	331687	-3186	20587	AN4963
2247	CONTIG84	334666	335080	1.29	1.93E-01	transcription_start_site	-	331526	331526	-3347	20588	AN4963
2247	CONTIG84	334666	335080	1.29	1.93E-01	transcription_start_site	-	331170	331170	-3703	20589	AN4963
2247	CONTIG84	334666	335080	1.29	1.93E-01	transcription_start_site	-	330926	330926	-3947	20590	AN4963
2247	CONTIG84	334666	335080	1.29	1.93E-01	transcription_start_site	-	330768	330768	-4105	20591	AN4963
1582	CONTIG84	328956	329380	1.55	9.24E-02	transcription_start_site	+	332733	332733	-3565	20593	AN4964
1582	CONTIG84	328956	329380	1.55	9.24E-02	transcription_start_site	+	332825	332825	-3657	20594	AN4964
2247	CONTIG84	334666	335080	1.29	1.93E-01	transcription_start_site	+	334734	334734	139	20595	AN4965
2247	CONTIG84	334666	335080	1.29	1.93E-01	transcription_start_site	+	335499	335499	-626	20596	AN4965
2247	CONTIG84	334666	335080	1.29	1.93E-01	transcription_start_site	+	335880	335880	-1007	20597	AN4965
28	CONTIG84	365719	366213	3.56	0.00E+00	transcription_start_site	-	362115	362115	-3851	20612	AN4972
28	CONTIG84	365719	366213	3.56	0.00E+00	transcription_start_site	-	361680	361680	-4286	20613	AN4972
28	CONTIG84	365719	366213	3.56	0.00E+00	transcription_start_site	-	361514	361514	-4452	20614	AN4972
28	CONTIG84	365719	366213	3.56	0.00E+00	transcription_start_site	-	360729	360729	-5237	20615	AN4972
249	CONTIG84	365038	365688	2.84	1.01E-03	transcription_start_site	-	362115	362115	-3248	20612	AN4972
249	CONTIG84	365038	365688	2.84	1.01E-03	transcription_start_site	-	361680	361680	-3683	20613	AN4972
249	CONTIG84	365038	365688	2.84	1.01E-03	transcription_start_site	-	361514	361514	-3849	20614	AN4972
249	CONTIG84	365038	365688	2.84	1.01E-03	transcription_start_site	-	360729	360729	-4634	20615	AN4972
28	CONTIG84	365719	366213	3.56	0.00E+00	transcription_start_site	-	364628	364628	-1338	20616	AN4973
28	CONTIG84	365719	366213	3.56	0.00E+00	transcription_start_site	-	364140	364140	-1826	20617	AN4973
249	CONTIG84	365038	365688	2.84	1.01E-03	transcription_start_site	-	364628	364628	-735	20616	AN4973
249	CONTIG84	365038	365688	2.84	1.01E-03	transcription_start_site	-	364140	364140	-1223	20617	AN4973

1583	CONTIG84	369609	370118	1.55	9.24E-02	transcription_start_site	-	364628	364628	-5235	20616	AN4973
1701	CONTIG84	367739	368613	1.5	8.36E-02	transcription_start_site	-	364628	364628	-3548	20616	AN4973
1701	CONTIG84	367739	368613	1.5	8.36E-02	transcription_start_site	-	364140	364140	-4036	20617	AN4973
288	CONTIG84	372908	373192	2.78	1.22E-03	transcription_start_site	-	368654	368654	-4396	20618	AN4974
288	CONTIG84	372908	373192	2.78	1.22E-03	transcription_start_site	-	368489	368489	-4561	20619	AN4974
1583	CONTIG84	369609	370118	1.55	9.24E-02	transcription_start_site	-	368654	368654	-1209	20618	AN4974
1583	CONTIG84	369609	370118	1.55	9.24E-02	transcription_start_site	-	368489	368489	-1374	20619	AN4974
1701	CONTIG84	367739	368613	1.5	8.36E-02	transcription_start_site	-	368489	368489	313	20619	AN4974
1701	CONTIG84	367739	368613	1.5	8.36E-02	transcription_start_site	-	368654	368654	478	20618	AN4974
28	CONTIG84	365719	366213	3.56	0.00E+00	transcription_start_site	+	368981	368981	-3015	20620	AN4975
28	CONTIG84	365719	366213	3.56	0.00E+00	transcription_start_site	+	369125	369125	-3159	20621	AN4975
28	CONTIG84	365719	366213	3.56	0.00E+00	transcription_start_site	+	370173	370173	-4207	20622	AN4975
249	CONTIG84	365038	365688	2.84	1.01E-03	transcription_start_site	+	368981	368981	-3618	20620	AN4975
249	CONTIG84	365038	365688	2.84	1.01E-03	transcription_start_site	+	369125	369125	-3762	20621	AN4975
249	CONTIG84	365038	365688	2.84	1.01E-03	transcription_start_site	+	370173	370173	-4810	20622	AN4975
1583	CONTIG84	369609	370118	1.55	9.24E-02	transcription_start_site	+	370173	370173	-309	20622	AN4975
1583	CONTIG84	369609	370118	1.55	9.24E-02	transcription_start_site	+	369125	369125	738	20621	AN4975
1583	CONTIG84	369609	370118	1.55	9.24E-02	transcription_start_site	+	368981	368981	882	20620	AN4975
1701	CONTIG84	367739	368613	1.5	8.36E-02	transcription_start_site	+	368981	368981	-805	20620	AN4975
1701	CONTIG84	367739	368613	1.5	8.36E-02	transcription_start_site	+	369125	369125	-949	20621	AN4975
1701	CONTIG84	367739	368613	1.5	8.36E-02	transcription_start_site	+	370173	370173	-1997	20622	AN4975
288	CONTIG84	372908	373192	2.78	1.22E-03	transcription_start_site	-	372220	372220	-830	20623	AN4976
288	CONTIG84	372908	373192	2.78	1.22E-03	transcription_start_site	-	371869	371869	-1181	20624	AN4976
288	CONTIG84	372908	373192	2.78	1.22E-03	transcription_start_site	-	371711	371711	-1339	20625	AN4976
475	CONTIG84	376655	376941	2.48	4.61E-03	transcription_start_site	-	372220	372220	-4578	20623	AN4976
475	CONTIG84	376655	376941	2.48	4.61E-03	transcription_start_site	-	371869	371869	-4929	20624	AN4976
475	CONTIG84	376655	376941	2.48	4.61E-03	transcription_start_site	-	371711	371711	-5087	20625	AN4976
1374	CONTIG84	373963	374452	1.65	6.84E-02	transcription_start_site	-	372220	372220	-1987	20623	AN4976
1374	CONTIG84	373963	374452	1.65	6.84E-02	transcription_start_site	-	371869	371869	-2338	20624	AN4976
1374	CONTIG84	373963	374452	1.65	6.84E-02	transcription_start_site	-	371711	371711	-2496	20625	AN4976
288	CONTIG84	372908	373192	2.78	1.22E-03	transcription_start_site	+	374527	374527	-1477	20626	AN4977
288	CONTIG84	372908	373192	2.78	1.22E-03	transcription_start_site	+	374893	374893	-1843	20627	AN4977
288	CONTIG84	372908	373192	2.78	1.22E-03	transcription_start_site	+	376468	376468	-3418	20628	AN4977
475	CONTIG84	376655	376941	2.48	4.61E-03	transcription_start_site	+	376468	376468	330	20628	AN4977
1374	CONTIG84	373963	374452	1.65	6.84E-02	transcription_start_site	+	374527	374527	-319	20626	AN4977
1374	CONTIG84	373963	374452	1.65	6.84E-02	transcription_start_site	+	374893	374893	-685	20627	AN4977
1374	CONTIG84	373963	374452	1.65	6.84E-02	transcription_start_site	+	376468	376468	-2260	20628	AN4977
1583	CONTIG84	369609	370118	1.55	9.24E-02	transcription_start_site	+	374527	374527	-4663	20626	AN4977
1583	CONTIG84	369609	370118	1.55	9.24E-02	transcription_start_site	+	374893	374893	-5029	20627	AN4977
288	CONTIG84	372908	373192	2.78	1.22E-03	transcription_start_site	+	377474	377474	-4424	20629	AN4978
288	CONTIG84	372908	373192	2.78	1.22E-03	transcription_start_site	+	377877	377877	-4827	20630	AN4978
288	CONTIG84	372908	373192	2.78	1.22E-03	transcription_start_site	+	378056	378056	-5006	20631	AN4978
288	CONTIG84	372908	373192	2.78	1.22E-03	transcription_start_site	+	378116	378116	-5066	20632	AN4978
475	CONTIG84	376655	376941	2.48	4.61E-03	transcription_start_site	+	377474	377474	-676	20629	AN4978
475	CONTIG84	376655	376941	2.48	4.61E-03	transcription_start_site	+	377877	377877	-1079	20630	AN4978
475	CONTIG84	376655	376941	2.48	4.61E-03	transcription_start_site	+	378056	378056	-1258	20631	AN4978
475	CONTIG84	376655	376941	2.48	4.61E-03	transcription_start_site	+	378116	378116	-1318	20632	AN4978
475	CONTIG84	376655	376941	2.48	4.61E-03	transcription_start_site	+	378659	378659	-1861	20633	AN4978
475	CONTIG84	376655	376941	2.48	4.61E-03	transcription_start_site	+	378787	378787	-1989	20634	AN4978
475	CONTIG84	376655	376941	2.48	4.61E-03	transcription_start_site	+	378943	378943	-2145	20635	AN4978
1374	CONTIG84	373963	374452	1.65	6.84E-02	transcription_start_site	+	377474	377474	-3266	20629	AN4978
1374	CONTIG84	373963	374452	1.65	6.84E-02	transcription_start_site	+	377877	377877	-3669	20630	AN4978
1374	CONTIG84	373963	374452	1.65	6.84E-02	transcription_start_site	+	378056	378056	-3848	20631	AN4978
1374	CONTIG84	373963	374452	1.65	6.84E-02	transcription_start_site	+	378116	378116	-3908	20632	AN4978
1374	CONTIG84	373963	374452	1.65	6.84E-02	transcription_start_site	+	378659	378659	-4451	20633	AN4978
1374	CONTIG84	373963	374452	1.65	6.84E-02	transcription_start_site	+	378787	378787	-4579	20634	AN4978
1374	CONTIG84	373963	374452	1.65	6.84E-02	transcription_start_site	+	378943	378943	-4735	20635	AN4978

475	CONTIG84	376655	376941	2.48	4.61E-03	transcription_start_site	+	380005	380005	-3207	20636	AN4979
475	CONTIG84	376655	376941	2.48	4.61E-03	transcription_start_site	+	380939	380939	-4141	20637	AN4979
321	CONTIG84	396230	396819	2.73	0.00E+00	transcription_start_site	-	395471	395471	-1053	20644	AN4984
796	CONTIG84	395114	395544	2.11	1.57E-02	transcription_start_site	-	395471	395471	142	20644	AN4984
66	CONTIG84	407495	407979	3.35	0.00E+00	transcription_start_site	-	402654	402654	-5083	20645	AN4985
250	CONTIG84	406595	407169	2.84	1.01E-03	transcription_start_site	-	402654	402654	-4228	20645	AN4985
1301	CONTIG84	402695	403194	1.7	3.99E-02	transcription_start_site	-	402654	402654	-290	20645	AN4985
1301	CONTIG84	402695	403194	1.7	3.99E-02	transcription_start_site	+	402976	402976	-31	20646	AN4986
1301	CONTIG84	402695	403194	1.7	3.99E-02	transcription_start_site	+	403582	403582	-637	20647	AN4986
66	CONTIG84	407495	407979	3.35	0.00E+00	transcription_start_site	-	405592	405592	-2145	20648	AN4987
250	CONTIG84	406595	407169	2.84	1.01E-03	transcription_start_site	-	405592	405592	-1290	20648	AN4987
1146	CONTIG84	408910	409334	1.8	4.36E-02	transcription_start_site	-	405592	405592	-3530	20648	AN4987
66	CONTIG84	407495	407979	3.35	0.00E+00	transcription_start_site	+	407908	407908	-171	20649	AN4988
66	CONTIG84	407495	407979	3.35	0.00E+00	transcription_start_site	+	408015	408015	-278	20650	AN4988
66	CONTIG84	407495	407979	3.35	0.00E+00	transcription_start_site	+	408430	408430	-693	20651	AN4988
250	CONTIG84	406595	407169	2.84	1.01E-03	transcription_start_site	+	407908	407908	-1026	20649	AN4988
250	CONTIG84	406595	407169	2.84	1.01E-03	transcription_start_site	+	408015	408015	-1133	20650	AN4988
250	CONTIG84	406595	407169	2.84	1.01E-03	transcription_start_site	+	408430	408430	-1548	20651	AN4988
1146	CONTIG84	408910	409334	1.8	4.36E-02	transcription_start_site	+	408430	408430	692	20651	AN4988
1146	CONTIG84	408910	409334	1.8	4.36E-02	transcription_start_site	+	408015	408015	1107	20650	AN4988
1301	CONTIG84	402695	403194	1.7	3.99E-02	transcription_start_site	+	407908	407908	-4963	20649	AN4988
1301	CONTIG84	402695	403194	1.7	3.99E-02	transcription_start_site	+	408015	408015	-5070	20650	AN4988
66	CONTIG84	407495	407979	3.35	0.00E+00	transcription_start_site	+	411117	411117	-3380	20652	AN4989
66	CONTIG84	407495	407979	3.35	0.00E+00	transcription_start_site	+	411406	411406	-3669	20653	AN4989
66	CONTIG84	407495	407979	3.35	0.00E+00	transcription_start_site	+	411604	411604	-3867	20654	AN4989
250	CONTIG84	406595	407169	2.84	1.01E-03	transcription_start_site	+	411117	411117	-4235	20652	AN4989
250	CONTIG84	406595	407169	2.84	1.01E-03	transcription_start_site	+	411406	411406	-4524	20653	AN4989
250	CONTIG84	406595	407169	2.84	1.01E-03	transcription_start_site	+	411604	411604	-4722	20654	AN4989
1146	CONTIG84	408910	409334	1.8	4.36E-02	transcription_start_site	+	411117	411117	-1995	20652	AN4989
1146	CONTIG84	408910	409334	1.8	4.36E-02	transcription_start_site	+	411406	411406	-2284	20653	AN4989
1146	CONTIG84	408910	409334	1.8	4.36E-02	transcription_start_site	+	411604	411604	-2482	20654	AN4989
1146	CONTIG84	408910	409334	1.8	4.36E-02	transcription_start_site	+	413152	413152	-4030	20655	AN4990
1146	CONTIG84	408910	409334	1.8	4.36E-02	transcription_start_site	+	413547	413547	-4425	20656	AN4990
1147	CONTIG84	413645	413994	1.8	4.36E-02	transcription_start_site	+	413547	413547	272	20656	AN4990
1147	CONTIG84	413645	413994	1.8	4.36E-02	transcription_start_site	+	413152	413152	667	20655	AN4990
417	CONTIG84	417242	418101	2.58	3.03E-03	transcription_start_site	+	418807	418807	-1135	20657	AN4991
417	CONTIG84	417242	418101	2.58	3.03E-03	transcription_start_site	+	419285	419285	-1613	20658	AN4991
1147	CONTIG84	413645	413994	1.8	4.36E-02	transcription_start_site	+	418807	418807	-4987	20657	AN4991
2248	CONTIG84	415890	416233	1.29	1.93E-01	transcription_start_site	+	418807	418807	-2745	20657	AN4991
2248	CONTIG84	415890	416233	1.29	1.93E-01	transcription_start_site	+	419285	419285	-3223	20658	AN4991
1963	CONTIG84	422344	422603	1.39	1.44E-01	transcription_start_site	-	421786	421786	-687	20661	AN4992
1963	CONTIG84	422344	422603	1.39	1.44E-01	transcription_start_site	-	423253	423253	779	20660	AN4992
1963	CONTIG84	422344	422603	1.39	1.44E-01	transcription_start_site	+	423944	423944	-1470	20662	AN4993
1963	CONTIG84	422344	422603	1.39	1.44E-01	transcription_start_site	+	424425	424425	-1951	20663	AN4993
629	CONTIG84	439657	439926	2.27	8.81E-03	transcription_start_site	-	437543	437543	-2248	20672	AN4998
629	CONTIG84	439657	439926	2.27	8.81E-03	transcription_start_site	-	437372	437372	-2419	20673	AN4998
629	CONTIG84	439657	439926	2.27	8.81E-03	transcription_start_site	-	437184	437184	-2607	20674	AN4998
629	CONTIG84	439657	439926	2.27	8.81E-03	transcription_start_site	-	440442	440442	650	20678	AN4999
629	CONTIG84	439657	439926	2.27	8.81E-03	transcription_start_site	-	440636	440636	844	20677	AN4999
629	CONTIG84	439657	439926	2.27	8.81E-03	transcription_start_site	+	441951	441951	-2159	20679	AN5000
629	CONTIG84	439657	439926	2.27	8.81E-03	transcription_start_site	+	442243	442243	-2451	20680	AN5000
629	CONTIG84	439657	439926	2.27	8.81E-03	transcription_start_site	+	442521	442521	-2729	20681	AN5000
629	CONTIG84	439657	439926	2.27	8.81E-03	transcription_start_site	+	442694	442694	-2902	20682	AN5000
629	CONTIG84	439657	439926	2.27	8.81E-03	transcription_start_site	+	443934	443934	-4142	20683	AN5001
1707	CONTIG84	447762	448101	1.5	1.08E-01	transcription_start_site	+	446799	446799	1132	20684	AN5001
1707	CONTIG84	447762	448101	1.5	1.08E-01	transcription_start_site	+	449695	449695	-1763	20685	AN5002
1707	CONTIG84	447762	448101	1.5	1.08E-01	transcription_start_site	+	450002	450002	-2070	20686	AN5002

1707	CONTIG84	447762	448101	1.5	1.08E-01	transcription_start_site	+	450261	450261	-2329	20687	AN5002
57	CONTIG84	451592	452156	3.4	0.00E+00	transcription_start_site	+	454782	454782	-2908	20688	AN5003
58	CONTIG84	452177	453651	3.4	0.00E+00	transcription_start_site	+	454782	454782	-1868	20688	AN5003
1708	CONTIG84	455251	456808	1.5	1.08E-01	transcription_start_site	+	454782	454782	1247	20688	AN5003
58	CONTIG84	452177	453651	3.4	0.00E+00	transcription_start_site	+	457755	457755	-4841	20689	AN5004
58	CONTIG84	452177	453651	3.4	0.00E+00	transcription_start_site	+	457945	457945	-5031	20690	AN5004
58	CONTIG84	452177	453651	3.4	0.00E+00	transcription_start_site	+	458105	458105	-5191	20691	AN5004
1708	CONTIG84	455251	456808	1.5	1.08E-01	transcription_start_site	+	457755	457755	-1725	20689	AN5004
1708	CONTIG84	455251	456808	1.5	1.08E-01	transcription_start_site	+	457945	457945	-1915	20690	AN5004
1708	CONTIG84	455251	456808	1.5	1.08E-01	transcription_start_site	+	458105	458105	-2075	20691	AN5004
1708	CONTIG84	455251	456808	1.5	1.08E-01	transcription_start_site	+	458956	458956	-2926	20692	AN5005
2244	CONTIG84	459826	460255	1.29	1.79E-01	transcription_start_site	+	458956	458956	1084	20692	AN5005
1480	CONTIG84	474905	475271	1.6	6.16E-02	transcription_start_site	-	474723	474723	-365	20700	AN5009
1480	CONTIG84	474905	475271	1.6	6.16E-02	transcription_start_site	-	473416	473416	-1672	20701	AN5009
1480	CONTIG84	474905	475271	1.6	6.16E-02	transcription_start_site	-	473179	473179	-1909	20702	AN5009
1480	CONTIG84	474905	475271	1.6	6.16E-02	transcription_start_site	-	472643	472643	-2445	20703	AN5009
1480	CONTIG84	474905	475271	1.6	6.16E-02	transcription_start_site	-	472465	472465	-2623	20704	AN5009
1480	CONTIG84	474905	475271	1.6	6.16E-02	transcription_start_site	-	472373	472373	-2715	20705	AN5009
1480	CONTIG84	474905	475271	1.6	6.16E-02	transcription_start_site	+	478130	478130	-3042	20707	AN5011
1480	CONTIG84	474905	475271	1.6	6.16E-02	transcription_start_site	+	478222	478222	-3134	20708	AN5011
1480	CONTIG84	474905	475271	1.6	6.16E-02	transcription_start_site	+	478823	478823	-3735	20709	AN5011
1480	CONTIG84	474905	475271	1.6	6.16E-02	transcription_start_site	+	479053	479053	-3965	20710	AN5011
1480	CONTIG84	474905	475271	1.6	6.16E-02	transcription_start_site	+	479146	479146	-4058	20711	AN5011
1480	CONTIG84	474905	475271	1.6	6.16E-02	transcription_start_site	+	479283	479283	-4195	20712	AN5011
1964	CONTIG84	485261	485766	1.39	1.44E-01	transcription_start_site	+	485423	485423	90	20724	AN5012
1964	CONTIG84	485261	485766	1.39	1.44E-01	transcription_start_site	+	485283	485283	230	20723	AN5012
1964	CONTIG84	485261	485766	1.39	1.44E-01	transcription_start_site	+	485745	485745	-231	20725	AN5012
1964	CONTIG84	485261	485766	1.39	1.44E-01	transcription_start_site	+	485213	485213	300	20722	AN5012
1964	CONTIG84	485261	485766	1.39	1.44E-01	transcription_start_site	+	485083	485083	430	20721	AN5012
1964	CONTIG84	485261	485766	1.39	1.44E-01	transcription_start_site	+	484807	484807	706	20720	AN5012
1964	CONTIG84	485261	485766	1.39	1.44E-01	transcription_start_site	+	486269	486269	-755	20726	AN5012
1964	CONTIG84	485261	485766	1.39	1.44E-01	transcription_start_site	+	484638	484638	875	20719	AN5012
1964	CONTIG84	485261	485766	1.39	1.44E-01	transcription_start_site	+	489071	489071	-3557	20727	AN5013
203	CONTIG84	494346	494836	2.94	8.69E-04	transcription_start_site	-	492012	492012	-2579	20728	AN5014
203	CONTIG84	494346	494836	2.94	8.69E-04	transcription_start_site	-	491805	491805	-2786	20729	AN5014
203	CONTIG84	494346	494836	2.94	8.69E-04	transcription_start_site	-	491503	491503	-3088	20730	AN5014
203	CONTIG84	494346	494836	2.94	8.69E-04	transcription_start_site	+	494859	494859	-268	20731	AN5015
203	CONTIG84	494346	494836	2.94	8.69E-04	transcription_start_site	+	495065	495065	-474	20732	AN5015
1148	CONTIG84	499727	500016	1.8	4.36E-02	transcription_start_site	-	497230	497230	-2641	20733	AN5016
1148	CONTIG84	499727	500016	1.8	4.36E-02	transcription_start_site	-	496910	496910	-2961	20734	AN5016
1148	CONTIG84	499727	500016	1.8	4.36E-02	transcription_start_site	-	496446	496446	-3425	20735	AN5016
1148	CONTIG84	499727	500016	1.8	4.36E-02	transcription_start_site	-	499396	499396	-475	20736	AN5017
1148	CONTIG84	499727	500016	1.8	4.36E-02	transcription_start_site	-	499045	499045	-826	20737	AN5017
1709	CONTIG84	511726	512005	1.5	1.08E-01	transcription_start_site	-	508272	508272	-3593	20745	AN5020
1709	CONTIG84	511726	512005	1.5	1.08E-01	transcription_start_site	-	508120	508120	-3745	20746	AN5020
1709	CONTIG84	511726	512005	1.5	1.08E-01	transcription_start_site	-	507909	507909	-3956	20747	AN5020
1709	CONTIG84	511726	512005	1.5	1.08E-01	transcription_start_site	-	507711	507711	-4154	20748	AN5020
1709	CONTIG84	511726	512005	1.5	1.08E-01	transcription_start_site	-	507547	507547	-4318	20749	AN5020
1709	CONTIG84	511726	512005	1.5	1.08E-01	transcription_start_site	+	510861	510861	1004	20755	AN5021
1709	CONTIG84	511726	512005	1.5	1.08E-01	transcription_start_site	-	512306	512306	440	20757	AN5022
1709	CONTIG84	511726	512005	1.5	1.08E-01	transcription_start_site	-	512553	512553	687	20756	AN5022
1709	CONTIG84	511726	512005	1.5	1.08E-01	transcription_start_site	+	515823	515823	-3957	20763	AN5024
1709	CONTIG84	511726	512005	1.5	1.08E-01	transcription_start_site	+	515978	515978	-4112	20764	AN5024
1709	CONTIG84	511726	512005	1.5	1.08E-01	transcription_start_site	+	516159	516159	-4293	20765	AN5024
1709	CONTIG84	511726	512005	1.5	1.08E-01	transcription_start_site	+	516570	516570	-4704	20766	AN5024
1709	CONTIG84	511726	512005	1.5	1.08E-01	transcription_start_site	+	516728	516728	-4862	20767	AN5024
476	CONTIG84	522228	522507	2.48	4.61E-03	transcription_start_site	-	522413	522413	45	20777	AN5026

1149	CONTIG84	526951	527300	1.8	4.36E-02	transcription_start_site	-	522413	522413	-4712	20777 AN5026
1149	CONTIG84	526951	527300	1.8	4.36E-02	transcription_start_site	-	525540	525540	-1585	20778 AN5027
1149	CONTIG84	526951	527300	1.8	4.36E-02	transcription_start_site	-	525362	525362	-1763	20779 AN5027
1149	CONTIG84	526951	527300	1.8	4.36E-02	transcription_start_site	+	529075	529075	-1949	20780 AN5028
1149	CONTIG84	526951	527300	1.8	4.36E-02	transcription_start_site	+	529567	529567	-2441	20781 AN5028
1149	CONTIG84	526951	527300	1.8	4.36E-02	transcription_start_site	+	529718	529718	-2592	20782 AN5028
1149	CONTIG84	526951	527300	1.8	4.36E-02	transcription_start_site	+	530011	530011	-2885	20783 AN5028
1149	CONTIG84	526951	527300	1.8	4.36E-02	transcription_start_site	+	530270	530270	-3144	20784 AN5028
1149	CONTIG84	526951	527300	1.8	4.36E-02	transcription_start_site	+	530459	530459	-3333	20785 AN5028
1149	CONTIG84	526951	527300	1.8	4.36E-02	transcription_start_site	+	530597	530597	-3471	20786 AN5028
1149	CONTIG84	526951	527300	1.8	4.36E-02	transcription_start_site	+	530978	530978	-3852	20787 AN5028
1149	CONTIG84	526951	527300	1.8	4.36E-02	transcription_start_site	+	531370	531370	-4244	20788 AN5028
1149	CONTIG84	526951	527300	1.8	4.36E-02	transcription_start_site	+	531906	531906	-4780	20789 AN5028
1844	CONTIG84	544137	544476	1.44	1.25E-01	transcription_start_site	-	539480	539480	-4826	20797 AN5031
1844	CONTIG84	544137	544476	1.44	1.25E-01	transcription_start_site	-	539348	539348	-4958	20798 AN5031
1844	CONTIG84	544137	544476	1.44	1.25E-01	transcription_start_site	+	544673	544673	-366	20805 AN5033
1844	CONTIG84	544137	544476	1.44	1.25E-01	transcription_start_site	+	544958	544958	-651	20806 AN5033
1844	CONTIG84	544137	544476	1.44	1.25E-01	transcription_start_site	+	545053	545053	-746	20807 AN5033
1844	CONTIG84	544137	544476	1.44	1.25E-01	transcription_start_site	+	545275	545275	-968	20808 AN5033
1844	CONTIG84	544137	544476	1.44	1.25E-01	transcription_start_site	+	545396	545396	-1089	20809 AN5033
1844	CONTIG84	544137	544476	1.44	1.25E-01	transcription_start_site	+	545802	545802	-1495	20810 AN5033
1844	CONTIG84	544137	544476	1.44	1.25E-01	transcription_start_site	+	545894	545894	-1587	20811 AN5033
1844	CONTIG84	544137	544476	1.44	1.25E-01	transcription_start_site	+	546398	546398	-2091	20812 AN5033
1844	CONTIG84	544137	544476	1.44	1.25E-01	transcription_start_site	+	546641	546641	-2334	20813 AN5033
1844	CONTIG84	544137	544476	1.44	1.25E-01	transcription_start_site	+	548987	548987	-4680	20814 AN5034
1844	CONTIG84	544137	544476	1.44	1.25E-01	transcription_start_site	+	549072	549072	-4765	20815 AN5034
293	CONTIG85	5946	6425	2.77	2.16E-04	transcription_start_site	-	2231	2231	-3954	20877 AN5053
293	CONTIG85	5946	6425	2.77	2.16E-04	transcription_start_site	-	5022	5022	-1163	20878 AN5054
293	CONTIG85	5946	6425	2.77	2.16E-04	transcription_start_site	-	4345	4345	-1840	20879 AN5054
293	CONTIG85	5946	6425	2.77	2.16E-04	transcription_start_site	-	4236	4236	-1949	20880 AN5054
293	CONTIG85	5946	6425	2.77	2.16E-04	transcription_start_site	+	7136	7136	-950	20881 AN5055
293	CONTIG85	5946	6425	2.77	2.16E-04	transcription_start_site	+	7368	7368	-1182	20882 AN5055
293	CONTIG85	5946	6425	2.77	2.16E-04	transcription_start_site	+	7740	7740	-1554	20883 AN5055
293	CONTIG85	5946	6425	2.77	2.16E-04	transcription_start_site	+	8367	8367	-2181	20884 AN5055
293	CONTIG85	5946	6425	2.77	2.16E-04	transcription_start_site	+	11265	11265	-5079	20885 AN5056
293	CONTIG85	5946	6425	2.77	2.16E-04	transcription_start_site	+	11345	11345	-5159	20886 AN5056
1076	CONTIG85	10956	11243	1.85	1.84E-02	transcription_start_site	+	11265	11265	-165	20885 AN5056
1076	CONTIG85	10956	11243	1.85	1.84E-02	transcription_start_site	+	11345	11345	-245	20886 AN5056
1076	CONTIG85	10956	11243	1.85	1.84E-02	transcription_start_site	+	11610	11610	-510	20887 AN5056
1863	CONTIG85	29629	29918	1.43	8.01E-02	transcription_start_site	+	29075	29075	698	20905 AN5061
1863	CONTIG85	29629	29918	1.43	8.01E-02	transcription_start_site	+	28971	28971	802	20904 AN5061
1863	CONTIG85	29629	29918	1.43	8.01E-02	transcription_start_site	+	28677	28677	1096	20903 AN5061
1863	CONTIG85	29629	29918	1.43	8.01E-02	transcription_start_site	+	31013	31013	-1239	20906 AN5061
210	CONTIG86	21089	21658	2.92	0.00E+00	transcription_start_site	-	17444	17444	-3929	20922 AN5067
210	CONTIG86	21089	21658	2.92	0.00E+00	transcription_start_site	-	17300	17300	-4073	20923 AN5067
210	CONTIG86	21089	21658	2.92	0.00E+00	transcription_start_site	-	16965	16965	-4408	20924 AN5067
210	CONTIG86	21089	21658	2.92	0.00E+00	transcription_start_site	-	16363	16363	-5010	20925 AN5067
210	CONTIG86	21089	21658	2.92	0.00E+00	transcription_start_site	-	16223	16223	-5150	20926 AN5067
210	CONTIG86	21089	21658	2.92	0.00E+00	transcription_start_site	+	20945	20945	428	20935 AN5068
210	CONTIG86	21089	21658	2.92	0.00E+00	transcription_start_site	+	20097	20097	1276	20934 AN5068
210	CONTIG86	21089	21658	2.92	0.00E+00	transcription_start_site	+	22106	22106	-732	20936 AN5069
210	CONTIG86	21089	21658	2.92	0.00E+00	transcription_start_site	+	22345	22345	-971	20937 AN5069
210	CONTIG86	21089	21658	2.92	0.00E+00	transcription_start_site	+	22429	22429	-1055	20938 AN5069
210	CONTIG86	21089	21658	2.92	0.00E+00	transcription_start_site	+	22530	22530	-1156	20939 AN5069
210	CONTIG86	21089	21658	2.92	0.00E+00	transcription_start_site	+	22665	22665	-1291	20940 AN5069
210	CONTIG86	21089	21658	2.92	0.00E+00	transcription_start_site	+	23212	23212	-1838	20941 AN5069
980	CONTIG86	28141	28420	1.93	4.36E-02	transcription_start_site	+	28527	28527	-246	20942 AN5070

980	CONTIG86	28141	28420	1.93	4.36E-02	transcription_start_site	+	28636	28636	-355	20943 AN5070
980	CONTIG86	28141	28420	1.93	4.36E-02	transcription_start_site	+	29675	29675	-1394	20944 AN5070
980	CONTIG86	28141	28420	1.93	4.36E-02	transcription_start_site	+	29969	29969	-1688	20945 AN5070
980	CONTIG86	28141	28420	1.93	4.36E-02	transcription_start_site	+	30719	30719	-2438	20946 AN5070
1610	CONTIG86	24454	24803	1.54	1.25E-01	transcription_start_site	+	28527	28527	-3898	20942 AN5070
1610	CONTIG86	24454	24803	1.54	1.25E-01	transcription_start_site	+	28636	28636	-4007	20943 AN5070
1610	CONTIG86	24454	24803	1.54	1.25E-01	transcription_start_site	+	29675	29675	-5046	20944 AN5070
980	CONTIG86	28141	28420	1.93	4.36E-02	transcription_start_site	+	31965	31965	-3684	20947 AN5071
980	CONTIG86	28141	28420	1.93	4.36E-02	transcription_start_site	+	32081	32081	-3800	20948 AN5071
980	CONTIG86	28141	28420	1.93	4.36E-02	transcription_start_site	+	32708	32708	-4427	20949 AN5071
980	CONTIG86	28141	28420	1.93	4.36E-02	transcription_start_site	+	33154	33154	-4873	20950 AN5071
1486	CONTIG86	38478	38755	1.6	1.08E-01	transcription_start_site	-	34748	34748	-3868	20951 AN5072
1486	CONTIG86	38478	38755	1.6	1.08E-01	transcription_start_site	-	34446	34446	-4170	20952 AN5072
1486	CONTIG86	38478	38755	1.6	1.08E-01	transcription_start_site	-	34278	34278	-4338	20953 AN5072
1486	CONTIG86	38478	38755	1.6	1.08E-01	transcription_start_site	-	34189	34189	-4427	20954 AN5072
1486	CONTIG86	38478	38755	1.6	1.08E-01	transcription_start_site	+	40696	40696	-2079	20960 AN5075
1486	CONTIG86	38478	38755	1.6	1.08E-01	transcription_start_site	+	41085	41085	-2468	20961 AN5075
1486	CONTIG86	38478	38755	1.6	1.08E-01	transcription_start_site	+	41293	41293	-2676	20962 AN5075
1486	CONTIG86	38478	38755	1.6	1.08E-01	transcription_start_site	+	41381	41381	-2764	20963 AN5075
1486	CONTIG86	38478	38755	1.6	1.08E-01	transcription_start_site	+	41760	41760	-3143	20964 AN5075
1486	CONTIG86	38478	38755	1.6	1.08E-01	transcription_start_site	+	42128	42128	-3511	20965 AN5075
1486	CONTIG86	38478	38755	1.6	1.08E-01	transcription_start_site	+	42444	42444	-3827	20966 AN5075
1486	CONTIG86	38478	38755	1.6	1.08E-01	transcription_start_site	+	42648	42648	-4031	20967 AN5075
1486	CONTIG86	38478	38755	1.6	1.08E-01	transcription_start_site	+	43163	43163	-4546	20968 AN5075
378	CONTIG86	47420	47684	2.64	4.61E-03	transcription_start_site	-	47055	47055	-497	20969 AN5076
378	CONTIG86	47420	47684	2.64	4.61E-03	transcription_start_site	-	46826	46826	-726	20970 AN5076
378	CONTIG86	47420	47684	2.64	4.61E-03	transcription_start_site	-	46671	46671	-881	20971 AN5076
378	CONTIG86	47420	47684	2.64	4.61E-03	transcription_start_site	-	46506	46506	-1046	20972 AN5076
378	CONTIG86	47420	47684	2.64	4.61E-03	transcription_start_site	-	46223	46223	-1329	20973 AN5076
378	CONTIG86	47420	47684	2.64	4.61E-03	transcription_start_site	-	46088	46088	-1464	20974 AN5076
931	CONTIG86	51835	52189	1.98	3.70E-02	transcription_start_site	-	47055	47055	-4957	20969 AN5076
1735	CONTIG86	45920	46179	1.49	1.44E-01	transcription_start_site	-	46088	46088	38	20974 AN5076
1735	CONTIG86	45920	46179	1.49	1.44E-01	transcription_start_site	-	46223	46223	173	20973 AN5076
1735	CONTIG86	45920	46179	1.49	1.44E-01	transcription_start_site	-	46506	46506	456	20972 AN5076
1735	CONTIG86	45920	46179	1.49	1.44E-01	transcription_start_site	-	46671	46671	621	20971 AN5076
1735	CONTIG86	45920	46179	1.49	1.44E-01	transcription_start_site	-	46826	46826	776	20970 AN5076
1735	CONTIG86	45920	46179	1.49	1.44E-01	transcription_start_site	-	47055	47055	1005	20969 AN5076
378	CONTIG86	47420	47684	2.64	4.61E-03	transcription_start_site	+	47748	47748	-196	20975 AN5077
378	CONTIG86	47420	47684	2.64	4.61E-03	transcription_start_site	+	50158	50158	-2606	20976 AN5077
378	CONTIG86	47420	47684	2.64	4.61E-03	transcription_start_site	+	50730	50730	-3178	20977 AN5077
378	CONTIG86	47420	47684	2.64	4.61E-03	transcription_start_site	+	50847	50847	-3295	20978 AN5077
378	CONTIG86	47420	47684	2.64	4.61E-03	transcription_start_site	+	51008	51008	-3456	20979 AN5077
378	CONTIG86	47420	47684	2.64	4.61E-03	transcription_start_site	+	51848	51848	-4296	20980 AN5077
378	CONTIG86	47420	47684	2.64	4.61E-03	transcription_start_site	+	52575	52575	-5023	20981 AN5077
931	CONTIG86	51835	52189	1.98	3.70E-02	transcription_start_site	+	51848	51848	164	20980 AN5077
931	CONTIG86	51835	52189	1.98	3.70E-02	transcription_start_site	+	52575	52575	-563	20981 AN5077
931	CONTIG86	51835	52189	1.98	3.70E-02	transcription_start_site	+	51008	51008	1004	20979 AN5077
931	CONTIG86	51835	52189	1.98	3.70E-02	transcription_start_site	+	50847	50847	1165	20978 AN5077
931	CONTIG86	51835	52189	1.98	3.70E-02	transcription_start_site	+	53182	53182	-1170	20982 AN5077
1487	CONTIG86	44326	44694	1.6	1.08E-01	transcription_start_site	+	47748	47748	-3238	20975 AN5077
1735	CONTIG86	45920	46179	1.49	1.44E-01	transcription_start_site	+	47748	47748	-1698	20975 AN5077
1735	CONTIG86	45920	46179	1.49	1.44E-01	transcription_start_site	+	50158	50158	-4108	20976 AN5077
1735	CONTIG86	45920	46179	1.49	1.44E-01	transcription_start_site	+	50730	50730	-4680	20977 AN5077
1735	CONTIG86	45920	46179	1.49	1.44E-01	transcription_start_site	+	50847	50847	-4797	20978 AN5077
1735	CONTIG86	45920	46179	1.49	1.44E-01	transcription_start_site	+	51008	51008	-4958	20979 AN5077
931	CONTIG86	51835	52189	1.98	3.70E-02	transcription_start_site	+	55476	55476	-3464	20983 AN5078
931	CONTIG86	51835	52189	1.98	3.70E-02	transcription_start_site	+	55821	55821	-3809	20984 AN5078

931	CONTIG86	51835	52189	1.98	3.70E-02	transcription_start_site	+	55925	55925	-3913	20985	AN5078
267	CONTIG86	66230	66890	2.81	2.22E-03	transcription_start_site	+	67694	67694	-1134	20991	AN5082
267	CONTIG86	66230	66890	2.81	2.22E-03	transcription_start_site	+	68163	68163	-1603	20992	AN5082
267	CONTIG86	66230	66890	2.81	2.22E-03	transcription_start_site	+	68748	68748	-2188	20993	AN5082
313	CONTIG86	74490	74851	2.75	3.03E-03	transcription_start_site	-	73173	73173	-1497	20994	AN5083
313	CONTIG86	74490	74851	2.75	3.03E-03	transcription_start_site	-	72973	72973	-1697	20995	AN5083
313	CONTIG86	74490	74851	2.75	3.03E-03	transcription_start_site	-	72824	72824	-1846	20996	AN5083
313	CONTIG86	74490	74851	2.75	3.03E-03	transcription_start_site	-	71315	71315	-3355	20997	AN5083
313	CONTIG86	74490	74851	2.75	3.03E-03	transcription_start_site	-	70380	70380	-4290	20998	AN5083
1282	CONTIG86	82587	83006	1.71	8.01E-02	transcription_start_site	+	83100	83100	-303	20999	AN5086
1609	CONTIG86	79067	79558	1.54	1.07E-01	transcription_start_site	+	83100	83100	-3787	20999	AN5086
161	CONTIG86	89252	89766	3.03	1.01E-03	transcription_start_site	-	85868	85868	-3641	21000	AN5087
161	CONTIG86	89252	89766	3.03	1.01E-03	transcription_start_site	-	85767	85767	-3742	21001	AN5087
161	CONTIG86	89252	89766	3.03	1.01E-03	transcription_start_site	-	85358	85358	-4151	21002	AN5087
161	CONTIG86	89252	89766	3.03	1.01E-03	transcription_start_site	+	90401	90401	-892	21003	AN5088
161	CONTIG86	89252	89766	3.03	1.01E-03	transcription_start_site	+	93082	93082	-3573	21004	AN5088
161	CONTIG86	89252	89766	3.03	1.01E-03	transcription_start_site	+	93314	93314	-3805	21005	AN5088
161	CONTIG86	89252	89766	3.03	1.01E-03	transcription_start_site	+	93599	93599	-4090	21006	AN5088
161	CONTIG86	89252	89766	3.03	1.01E-03	transcription_start_site	+	93842	93842	-4333	21007	AN5088
756	CONTIG86	96396	96808	2.15	1.06E-02	transcription_start_site	+	96899	96899	-297	21008	AN5089
1283	CONTIG86	103745	104019	1.71	8.01E-02	transcription_start_site	-	99164	99164	-4718	21009	AN5090
756	CONTIG86	96396	96808	2.15	1.06E-02	transcription_start_site	+	100480	100480	-3878	21010	AN5091
756	CONTIG86	96396	96808	2.15	1.06E-02	transcription_start_site	+	100805	100805	-4203	21011	AN5091
756	CONTIG86	96396	96808	2.15	1.06E-02	transcription_start_site	+	101128	101128	-4526	21012	AN5091
756	CONTIG86	96396	96808	2.15	1.06E-02	transcription_start_site	+	101309	101309	-4707	21013	AN5091
756	CONTIG86	96396	96808	2.15	1.06E-02	transcription_start_site	+	101395	101395	-4793	21014	AN5091
1283	CONTIG86	103745	104019	1.71	8.01E-02	transcription_start_site	+	105324	105324	-1442	21016	AN5092
1283	CONTIG86	103745	104019	1.71	8.01E-02	transcription_start_site	+	105726	105726	-1844	21017	AN5092
1283	CONTIG86	103745	104019	1.71	8.01E-02	transcription_start_site	+	107242	107242	-3360	21018	AN5092
2033	CONTIG88	10501	10935	1.36	3.33E-02	transcription_start_site	+	11647	11647	-929	21034	AN5099
2033	CONTIG88	10501	10935	1.36	3.33E-02	transcription_start_site	+	13986	13986	-3268	21035	AN5100
2033	CONTIG88	10501	10935	1.36	3.33E-02	transcription_start_site	+	14138	14138	-3420	21036	AN5100
2033	CONTIG88	10501	10935	1.36	3.33E-02	transcription_start_site	+	14229	14229	-3511	21037	AN5100
2033	CONTIG88	10501	10935	1.36	3.33E-02	transcription_start_site	+	14385	14385	-3667	21038	AN5100
2033	CONTIG88	10501	10935	1.36	3.33E-02	transcription_start_site	+	15149	15149	-4431	21039	AN5100
2033	CONTIG88	10501	10935	1.36	3.33E-02	transcription_start_site	+	15404	15404	-4686	21040	AN5100
646	CONTIG88	23476	23830	2.24	9.51E-04	transcription_start_site	-	20864	20864	-2789	21043	AN5102
646	CONTIG88	23476	23830	2.24	9.51E-04	transcription_start_site	-	20634	20634	-3019	21044	AN5102
646	CONTIG88	23476	23830	2.24	9.51E-04	transcription_start_site	-	20489	20489	-3164	21045	AN5102
646	CONTIG88	23476	23830	2.24	9.51E-04	transcription_start_site	-	20284	20284	-3369	21046	AN5102
646	CONTIG88	23476	23830	2.24	9.51E-04	transcription_start_site	-	20078	20078	-3575	21047	AN5102
646	CONTIG88	23476	23830	2.24	9.51E-04	transcription_start_site	-	18597	18597	-5056	21048	AN5102
646	CONTIG88	23476	23830	2.24	9.51E-04	transcription_start_site	+	24966	24966	-1313	21054	AN5104
646	CONTIG88	23476	23830	2.24	9.51E-04	transcription_start_site	+	25079	25079	-1426	21055	AN5104
646	CONTIG88	23476	23830	2.24	9.51E-04	transcription_start_site	+	25220	25220	-1567	21056	AN5104
646	CONTIG88	23476	23830	2.24	9.51E-04	transcription_start_site	+	25575	25575	-1922	21057	AN5104
646	CONTIG88	23476	23830	2.24	9.51E-04	transcription_start_site	+	25839	25839	-2186	21058	AN5104
646	CONTIG88	23476	23830	2.24	9.51E-04	transcription_start_site	+	26599	26599	-2946	21059	AN5104
646	CONTIG88	23476	23830	2.24	9.51E-04	transcription_start_site	+	27951	27951	-4298	21060	AN5105
646	CONTIG88	23476	23830	2.24	9.51E-04	transcription_start_site	+	28162	28162	-4509	21061	AN5105
646	CONTIG88	23476	23830	2.24	9.51E-04	transcription_start_site	+	28349	28349	-4696	21062	AN5105
647	CONTIG88	65046	65310	2.24	9.51E-04	transcription_start_site	+	65264	65264	-86	21104	AN5117
647	CONTIG88	65046	65310	2.24	9.51E-04	transcription_start_site	+	65082	65082	96	21103	AN5117
647	CONTIG88	65046	65310	2.24	9.51E-04	transcription_start_site	+	64761	64761	417	21102	AN5117
647	CONTIG88	65046	65310	2.24	9.51E-04	transcription_start_site	+	64485	64485	693	21101	AN5117
647	CONTIG88	65046	65310	2.24	9.51E-04	transcription_start_site	+	66073	66073	-895	21105	AN5118
647	CONTIG88	65046	65310	2.24	9.51E-04	transcription_start_site	+	66707	66707	-1529	21106	AN5118

647	CONTIG88	65046	65310	2.24	9.51E-04	transcription_start_site	+	66959	66959	-1781	21107	AN5118
2781	CONTIG88	66377	67478	1.08	1.44E-01	transcription_start_site	+	66959	66959	-31	21107	AN5118
2781	CONTIG88	66377	67478	1.08	1.44E-01	transcription_start_site	+	66707	66707	220	21106	AN5118
2781	CONTIG88	66377	67478	1.08	1.44E-01	transcription_start_site	+	66073	66073	854	21105	AN5118
2781	CONTIG88	66377	67478	1.08	1.44E-01	transcription_start_site	-	68228	68228	1300	21111	AN5119
647	CONTIG88	65046	65310	2.24	9.51E-04	transcription_start_site	+	69593	69593	-4415	21112	AN5120
647	CONTIG88	65046	65310	2.24	9.51E-04	transcription_start_site	+	70118	70118	-4940	21113	AN5120
2781	CONTIG88	66377	67478	1.08	1.44E-01	transcription_start_site	+	69593	69593	-2665	21112	AN5120
2781	CONTIG88	66377	67478	1.08	1.44E-01	transcription_start_site	+	70118	70118	-3190	21113	AN5120
2706	CONTIG88	78010	78284	1.12	1.25E-01	transcription_start_site	-	73338	73338	-4809	21114	AN5121
2706	CONTIG88	78010	78284	1.12	1.25E-01	transcription_start_site	-	73105	73105	-5042	21115	AN5121
2706	CONTIG88	78010	78284	1.12	1.25E-01	transcription_start_site	-	76421	76421	-1726	21121	AN5123
2706	CONTIG88	78010	78284	1.12	1.25E-01	transcription_start_site	-	76325	76325	-1822	21122	AN5123
2706	CONTIG88	78010	78284	1.12	1.25E-01	transcription_start_site	-	76252	76252	-1895	21123	AN5123
2706	CONTIG88	78010	78284	1.12	1.25E-01	transcription_start_site	-	75663	75663	-2484	21124	AN5123
2706	CONTIG88	78010	78284	1.12	1.25E-01	transcription_start_site	-	78401	78401	254	21127	AN5124
2706	CONTIG88	78010	78284	1.12	1.25E-01	transcription_start_site	-	77646	77646	-501	21128	AN5124
2706	CONTIG88	78010	78284	1.12	1.25E-01	transcription_start_site	-	78890	78890	743	21126	AN5124
2706	CONTIG88	78010	78284	1.12	1.25E-01	transcription_start_site	-	77403	77403	-744	21129	AN5124
2706	CONTIG88	78010	78284	1.12	1.25E-01	transcription_start_site	-	79142	79142	995	21125	AN5124
2706	CONTIG88	78010	78284	1.12	1.25E-01	transcription_start_site	-	76988	76988	-1159	21130	AN5124
2706	CONTIG88	78010	78284	1.12	1.25E-01	transcription_start_site	+	80016	80016	-1869	21131	AN5125
2706	CONTIG88	78010	78284	1.12	1.25E-01	transcription_start_site	+	80199	80199	-2052	21132	AN5125
2706	CONTIG88	78010	78284	1.12	1.25E-01	transcription_start_site	+	80278	80278	-2131	21133	AN5125
2706	CONTIG88	78010	78284	1.12	1.25E-01	transcription_start_site	+	80353	80353	-2206	21134	AN5125
2706	CONTIG88	78010	78284	1.12	1.25E-01	transcription_start_site	+	81044	81044	-2897	21135	AN5126
2706	CONTIG88	78010	78284	1.12	1.25E-01	transcription_start_site	+	82980	82980	-4833	21136	AN5126
2706	CONTIG88	78010	78284	1.12	1.25E-01	transcription_start_site	+	83214	83214	-5067	21137	AN5126
1914	CONTIG88	87539	87968	1.4	4.36E-02	transcription_start_site	-	85842	85842	-1911	21138	AN5127
1914	CONTIG88	87539	87968	1.4	4.36E-02	transcription_start_site	-	85778	85778	-1975	21139	AN5127
1914	CONTIG88	87539	87968	1.4	4.36E-02	transcription_start_site	-	85502	85502	-2251	21140	AN5127
1914	CONTIG88	87539	87968	1.4	4.36E-02	transcription_start_site	-	85330	85330	-2423	21141	AN5127
2508	CONTIG88	88884	89158	1.2	9.24E-02	transcription_start_site	-	85842	85842	-3179	21138	AN5127
2508	CONTIG88	88884	89158	1.2	9.24E-02	transcription_start_site	-	85778	85778	-3243	21139	AN5127
2508	CONTIG88	88884	89158	1.2	9.24E-02	transcription_start_site	-	85502	85502	-3519	21140	AN5127
2508	CONTIG88	88884	89158	1.2	9.24E-02	transcription_start_site	-	85330	85330	-3691	21141	AN5127
1914	CONTIG88	87539	87968	1.4	4.36E-02	transcription_start_site	-	86796	86796	-957	21142	AN5128
1914	CONTIG88	87539	87968	1.4	4.36E-02	transcription_start_site	-	86576	86576	-1177	21143	AN5128
1914	CONTIG88	87539	87968	1.4	4.36E-02	transcription_start_site	-	86509	86509	-1244	21144	AN5128
2508	CONTIG88	88884	89158	1.2	9.24E-02	transcription_start_site	-	86796	86796	-2225	21142	AN5128
2508	CONTIG88	88884	89158	1.2	9.24E-02	transcription_start_site	-	86576	86576	-2445	21143	AN5128
2508	CONTIG88	88884	89158	1.2	9.24E-02	transcription_start_site	-	86509	86509	-2512	21144	AN5128
1914	CONTIG88	87539	87968	1.4	4.36E-02	transcription_start_site	+	88164	88164	-410	21145	AN5129
2508	CONTIG88	88884	89158	1.2	9.24E-02	transcription_start_site	+	88164	88164	857	21145	AN5129
2508	CONTIG88	88884	89158	1.2	9.24E-02	transcription_start_site	+	93965	93965	-4944	21148	AN5131
376	CONTIG88	103351	103700	2.64	0.00E+00	transcription_start_site	-	98456	98456	-5069	21157	AN5133
376	CONTIG88	103351	103700	2.64	0.00E+00	transcription_start_site	-	98373	98373	-5152	21158	AN5133
898	CONTIG88	101176	101465	2	3.03E-03	transcription_start_site	-	98456	98456	-2864	21157	AN5133
898	CONTIG88	101176	101465	2	3.03E-03	transcription_start_site	-	98373	98373	-2947	21158	AN5133
1915	CONTIG88	99830	100119	1.4	4.36E-02	transcription_start_site	-	98456	98456	-1518	21157	AN5133
1915	CONTIG88	99830	100119	1.4	4.36E-02	transcription_start_site	-	98373	98373	-1601	21158	AN5133
376	CONTIG88	103351	103700	2.64	0.00E+00	transcription_start_site	+	104045	104045	-519	21159	AN5134
376	CONTIG88	103351	103700	2.64	0.00E+00	transcription_start_site	+	104285	104285	-759	21160	AN5134
376	CONTIG88	103351	103700	2.64	0.00E+00	transcription_start_site	+	104384	104384	-858	21161	AN5134
376	CONTIG88	103351	103700	2.64	0.00E+00	transcription_start_site	+	107783	107783	-4257	21162	AN5134
376	CONTIG88	103351	103700	2.64	0.00E+00	transcription_start_site	+	107870	107870	-4344	21163	AN5134
898	CONTIG88	101176	101465	2	3.03E-03	transcription_start_site	+	104045	104045	-2724	21159	AN5134

898	CONTIG88	101176	101465	2	3.03E-03	transcription_start_site	+	104285	104285	-2964	21160	AN5134
898	CONTIG88	101176	101465	2	3.03E-03	transcription_start_site	+	104384	104384	-3063	21161	AN5134
1915	CONTIG88	99830	100119	1.4	4.36E-02	transcription_start_site	+	104045	104045	-4070	21159	AN5134
1915	CONTIG88	99830	100119	1.4	4.36E-02	transcription_start_site	+	104285	104285	-4310	21160	AN5134
1915	CONTIG88	99830	100119	1.4	4.36E-02	transcription_start_site	+	104384	104384	-4409	21161	AN5134
2370	CONTIG88	113566	113991	1.24	8.01E-02	transcription_start_site	-	112272	112272	-1506	21164	AN5135
2370	CONTIG88	113566	113991	1.24	8.01E-02	transcription_start_site	-	113464	113464	-314	21165	AN5136
2370	CONTIG88	113566	113991	1.24	8.01E-02	transcription_start_site	+	118738	118738	-4959	21166	AN5138
2843	CONTIG88	126231	126735	1.04	1.50E-01	transcription_start_site	-	126761	126761	278	21170	AN5140
2843	CONTIG88	126231	126735	1.04	1.50E-01	transcription_start_site	-	126884	126884	401	21169	AN5140
1035	CONTIG88	139581	139935	1.88	2.32E-03	transcription_start_site	-	139114	139114	-644	21175	AN5144
1035	CONTIG88	139581	139935	1.88	2.32E-03	transcription_start_site	-	138555	138555	-1203	21176	AN5144
2608	CONTIG88	138161	138505	1.16	1.08E-01	transcription_start_site	-	138555	138555	222	21176	AN5144
2608	CONTIG88	138161	138505	1.16	1.08E-01	transcription_start_site	-	139114	139114	781	21175	AN5144
2850	CONTIG88	141236	141520	1.04	1.66E-01	transcription_start_site	-	139114	139114	-2264	21175	AN5144
2850	CONTIG88	141236	141520	1.04	1.66E-01	transcription_start_site	-	138555	138555	-2823	21176	AN5144
1035	CONTIG88	139581	139935	1.88	2.32E-03	transcription_start_site	-	140833	140833	1075	21178	AN5145
2850	CONTIG88	141236	141520	1.04	1.66E-01	transcription_start_site	-	140945	140945	-433	21177	AN5145
2850	CONTIG88	141236	141520	1.04	1.66E-01	transcription_start_site	-	140833	140833	-545	21178	AN5145
1035	CONTIG88	139581	139935	1.88	2.32E-03	transcription_start_site	+	141707	141707	-1949	21179	AN5146
1035	CONTIG88	139581	139935	1.88	2.32E-03	transcription_start_site	+	141788	141788	-2030	21180	AN5146
1035	CONTIG88	139581	139935	1.88	2.32E-03	transcription_start_site	+	141904	141904	-2146	21181	AN5146
1035	CONTIG88	139581	139935	1.88	2.32E-03	transcription_start_site	+	143145	143145	-3387	21182	AN5146
1035	CONTIG88	139581	139935	1.88	2.32E-03	transcription_start_site	+	143270	143270	-3512	21183	AN5146
2608	CONTIG88	138161	138505	1.16	1.08E-01	transcription_start_site	+	141707	141707	-3374	21179	AN5146
2608	CONTIG88	138161	138505	1.16	1.08E-01	transcription_start_site	+	141788	141788	-3455	21180	AN5146
2608	CONTIG88	138161	138505	1.16	1.08E-01	transcription_start_site	+	141904	141904	-3571	21181	AN5146
2608	CONTIG88	138161	138505	1.16	1.08E-01	transcription_start_site	+	143145	143145	-4812	21182	AN5146
2608	CONTIG88	138161	138505	1.16	1.08E-01	transcription_start_site	+	143270	143270	-4937	21183	AN5146
2850	CONTIG88	141236	141520	1.04	1.66E-01	transcription_start_site	+	141707	141707	-329	21179	AN5146
2850	CONTIG88	141236	141520	1.04	1.66E-01	transcription_start_site	+	141788	141788	-410	21180	AN5146
2850	CONTIG88	141236	141520	1.04	1.66E-01	transcription_start_site	+	141904	141904	-526	21181	AN5146
2850	CONTIG88	141236	141520	1.04	1.66E-01	transcription_start_site	+	143145	143145	-1767	21182	AN5146
2850	CONTIG88	141236	141520	1.04	1.66E-01	transcription_start_site	+	143270	143270	-1892	21183	AN5146
1312	CONTIG88	151818	152092	1.68	1.30E-02	transcription_start_site	-	149034	149034	-2921	21184	AN5148
1312	CONTIG88	151818	152092	1.68	1.30E-02	transcription_start_site	-	148909	148909	-3046	21185	AN5148
1312	CONTIG88	151818	152092	1.68	1.30E-02	transcription_start_site	-	148638	148638	-3317	21186	AN5148
1312	CONTIG88	151818	152092	1.68	1.30E-02	transcription_start_site	-	148455	148455	-3500	21187	AN5148
1312	CONTIG88	151818	152092	1.68	1.30E-02	transcription_start_site	+	154813	154813	-2858	21189	AN5151
1312	CONTIG88	151818	152092	1.68	1.30E-02	transcription_start_site	+	155038	155038	-3083	21190	AN5151
1136	CONTIG88	159019	159293	1.8	7.85E-03	transcription_start_site	+	160062	160062	-906	21191	AN5152
1136	CONTIG88	159019	159293	1.8	7.85E-03	transcription_start_site	+	160165	160165	-1009	21192	AN5152
1136	CONTIG88	159019	159293	1.8	7.85E-03	transcription_start_site	+	160273	160273	-1117	21193	AN5152
1136	CONTIG88	159019	159293	1.8	7.85E-03	transcription_start_site	+	160417	160417	-1261	21194	AN5152
1136	CONTIG88	159019	159293	1.8	7.85E-03	transcription_start_site	+	160558	160558	-1402	21195	AN5152
1136	CONTIG88	159019	159293	1.8	7.85E-03	transcription_start_site	+	160702	160702	-1546	21196	AN5152
1136	CONTIG88	159019	159293	1.8	7.85E-03	transcription_start_site	+	161089	161089	-1933	21197	AN5152
2131	CONTIG88	163589	163853	1.32	5.88E-02	transcription_start_site	-	162930	162930	-791	21198	AN5153
2131	CONTIG88	163589	163853	1.32	5.88E-02	transcription_start_site	-	162833	162833	-888	21199	AN5153
1136	CONTIG88	159019	159293	1.8	7.85E-03	transcription_start_site	+	163524	163524	-4368	21200	AN5154
2131	CONTIG88	163589	163853	1.32	5.88E-02	transcription_start_site	+	163524	163524	197	21200	AN5154
2131	CONTIG88	163589	163853	1.32	5.88E-02	transcription_start_site	+	167543	167543	-3822	21201	AN5154
2131	CONTIG88	163589	163853	1.32	5.88E-02	transcription_start_site	+	167781	167781	-4060	21202	AN5154
449	CONTIG88	172070	172559	2.52	0.00E+00	transcription_start_site	-	170208	170208	-2106	21203	AN5155
449	CONTIG88	172070	172559	2.52	0.00E+00	transcription_start_site	-	170059	170059	-2255	21204	AN5155
449	CONTIG88	172070	172559	2.52	0.00E+00	transcription_start_site	-	169952	169952	-2362	21205	AN5155
2910	CONTIG88	168979	169488	1	1.93E-01	transcription_start_site	-	169952	169952	718	21205	AN5155

2910	CONTIG88	168979	169488	1	1.93E-01	transcription_start_site	-	170059	170059	825	21204	AN5155
2910	CONTIG88	168979	169488	1	1.93E-01	transcription_start_site	-	170208	170208	974	21203	AN5155
449	CONTIG88	172070	172559	2.52	0.00E+00	transcription_start_site	-	172010	172010	-304	21206	AN5156
449	CONTIG88	172070	172559	2.52	0.00E+00	transcription_start_site	+	173224	173224	-909	21207	AN5157
449	CONTIG88	172070	172559	2.52	0.00E+00	transcription_start_site	+	173573	173573	-1258	21208	AN5157
449	CONTIG88	172070	172559	2.52	0.00E+00	transcription_start_site	+	175319	175319	-3004	21209	AN5157
2910	CONTIG88	168979	169488	1	1.93E-01	transcription_start_site	+	173224	173224	-3990	21207	AN5157
2910	CONTIG88	168979	169488	1	1.93E-01	transcription_start_site	+	173573	173573	-4339	21208	AN5157
1505	CONTIG89	18540	19569	1.58	2.64E-02	transcription_start_site	-	19646	19646	591	21229	AN5163
598	CONTIG89	32262	32686	2.31	2.22E-03	transcription_start_site	+	33967	33967	-1493	21239	AN5167
598	CONTIG89	32262	32686	2.31	2.22E-03	transcription_start_site	+	34437	34437	-1963	21240	AN5167
598	CONTIG89	32262	32686	2.31	2.22E-03	transcription_start_site	+	35752	35752	-3278	21241	AN5167
598	CONTIG89	32262	32686	2.31	2.22E-03	transcription_start_site	+	36770	36770	-4296	21242	AN5167
1921	CONTIG89	33677	34854	1.4	6.16E-02	transcription_start_site	+	34437	34437	-171	21240	AN5167
1921	CONTIG89	33677	34854	1.4	6.16E-02	transcription_start_site	+	33967	33967	298	21239	AN5167
1921	CONTIG89	33677	34854	1.4	6.16E-02	transcription_start_site	+	35752	35752	-1486	21241	AN5167
1921	CONTIG89	33677	34854	1.4	6.16E-02	transcription_start_site	+	36770	36770	-2504	21242	AN5167
1399	CONTIG89	45760	46029	1.63	3.70E-02	transcription_start_site	-	43680	43680	-2214	21243	AN5168
1399	CONTIG89	45760	46029	1.63	3.70E-02	transcription_start_site	-	43214	43214	-2680	21244	AN5168
1399	CONTIG89	45760	46029	1.63	3.70E-02	transcription_start_site	-	43090	43090	-2804	21245	AN5168
1399	CONTIG89	45760	46029	1.63	3.70E-02	transcription_start_site	-	42840	42840	-3054	21246	AN5168
1399	CONTIG89	45760	46029	1.63	3.70E-02	transcription_start_site	-	42761	42761	-3133	21247	AN5168
2169	CONTIG89	48395	48812	1.31	1.08E-01	transcription_start_site	-	43680	43680	-4923	21243	AN5168
1257	CONTIG89	50630	51066	1.72	2.62E-02	transcription_start_site	-	47954	47954	-2894	21249	AN5169
1257	CONTIG89	50630	51066	1.72	2.62E-02	transcription_start_site	-	47903	47903	-2945	21250	AN5169
1257	CONTIG89	50630	51066	1.72	2.62E-02	transcription_start_site	-	47648	47648	-3200	21251	AN5169
2169	CONTIG89	48395	48812	1.31	1.08E-01	transcription_start_site	-	47954	47954	-649	21249	AN5169
2169	CONTIG89	48395	48812	1.31	1.08E-01	transcription_start_site	-	47903	47903	-700	21250	AN5169
2169	CONTIG89	48395	48812	1.31	1.08E-01	transcription_start_site	-	47648	47648	-955	21251	AN5169
1257	CONTIG89	50630	51066	1.72	2.62E-02	transcription_start_site	+	51651	51651	-803	21252	AN5170
1257	CONTIG89	50630	51066	1.72	2.62E-02	transcription_start_site	+	51878	51878	-1030	21253	AN5170
1257	CONTIG89	50630	51066	1.72	2.62E-02	transcription_start_site	+	52124	52124	-1276	21254	AN5170
2169	CONTIG89	48395	48812	1.31	1.08E-01	transcription_start_site	+	51651	51651	-3047	21252	AN5170
2169	CONTIG89	48395	48812	1.31	1.08E-01	transcription_start_site	+	51878	51878	-3274	21253	AN5170
2169	CONTIG89	48395	48812	1.31	1.08E-01	transcription_start_site	+	52124	52124	-3520	21254	AN5170
1257	CONTIG89	50630	51066	1.72	2.62E-02	transcription_start_site	+	55454	55454	-4606	21255	AN5171
1257	CONTIG89	50630	51066	1.72	2.62E-02	transcription_start_site	+	55637	55637	-4789	21256	AN5171
2170	CONTIG89	77719	77993	1.31	1.08E-01	transcription_start_site	+	77326	77326	530	21276	AN5177
2170	CONTIG89	77719	77993	1.31	1.08E-01	transcription_start_site	+	76886	76886	970	21275	AN5177
2170	CONTIG89	77719	77993	1.31	1.08E-01	transcription_start_site	+	78704	78704	-848	21277	AN5178
2170	CONTIG89	77719	77993	1.31	1.08E-01	transcription_start_site	+	79289	79289	-1433	21278	AN5178
2170	CONTIG89	77719	77993	1.31	1.08E-01	transcription_start_site	+	80060	80060	-2204	21279	AN5179
2170	CONTIG89	77719	77993	1.31	1.08E-01	transcription_start_site	+	82389	82389	-4533	21280	AN5180
463	CONTIG89	99088	99960	2.49	1.01E-03	transcription_start_site	-	98214	98214	-1310	21292	AN5185
463	CONTIG89	99088	99960	2.49	1.01E-03	transcription_start_site	-	97917	97917	-1607	21293	AN5185
463	CONTIG89	99088	99960	2.49	1.01E-03	transcription_start_site	-	97520	97520	-2004	21294	AN5185
463	CONTIG89	99088	99960	2.49	1.01E-03	transcription_start_site	+	99504	99504	20	21296	AN5186
463	CONTIG89	99088	99960	2.49	1.01E-03	transcription_start_site	+	98958	98958	566	21295	AN5186
1400	CONTIG89	105316	106700	1.63	3.70E-02	transcription_start_site	-	104539	104539	-1469	21297	AN5187
1400	CONTIG89	105316	106700	1.63	3.70E-02	transcription_start_site	-	104373	104373	-1635	21298	AN5187
1400	CONTIG89	105316	106700	1.63	3.70E-02	transcription_start_site	-	103651	103651	-2357	21299	AN5187
1400	CONTIG89	105316	106700	1.63	3.70E-02	transcription_start_site	-	103567	103567	-2441	21300	AN5187
1400	CONTIG89	105316	106700	1.63	3.70E-02	transcription_start_site	-	102589	102589	-3419	21301	AN5187
1400	CONTIG89	105316	106700	1.63	3.70E-02	transcription_start_site	-	102443	102443	-3565	21302	AN5187
1726	CONTIG89	104721	104975	1.49	5.88E-02	transcription_start_site	-	104539	104539	-309	21297	AN5187
1726	CONTIG89	104721	104975	1.49	5.88E-02	transcription_start_site	-	104373	104373	-475	21298	AN5187
1726	CONTIG89	104721	104975	1.49	5.88E-02	transcription_start_site	-	103651	103651	-1197	21299	AN5187

1726	CONTIG89	104721	104975	1.49	5.88E-02	transcription_start_site	-	103567	103567	-1281	21300	AN5187
1726	CONTIG89	104721	104975	1.49	5.88E-02	transcription_start_site	-	102589	102589	-2259	21301	AN5187
1726	CONTIG89	104721	104975	1.49	5.88E-02	transcription_start_site	-	102443	102443	-2405	21302	AN5187
1400	CONTIG89	105316	106700	1.63	3.70E-02	transcription_start_site	-	106106	106106	98	21304	AN5188
1400	CONTIG89	105316	106700	1.63	3.70E-02	transcription_start_site	-	106533	106533	525	21303	AN5188
1400	CONTIG89	105316	106700	1.63	3.70E-02	transcription_start_site	-	105474	105474	-534	21305	AN5188
1400	CONTIG89	105316	106700	1.63	3.70E-02	transcription_start_site	-	104996	104996	-1012	21306	AN5188
1726	CONTIG89	104721	104975	1.49	5.88E-02	transcription_start_site	-	104996	104996	148	21306	AN5188
1726	CONTIG89	104721	104975	1.49	5.88E-02	transcription_start_site	-	105474	105474	626	21305	AN5188
1400	CONTIG89	105316	106700	1.63	3.70E-02	transcription_start_site	+	107983	107983	-1975	21307	AN5189
1400	CONTIG89	105316	106700	1.63	3.70E-02	transcription_start_site	+	108649	108649	-2641	21308	AN5189
1400	CONTIG89	105316	106700	1.63	3.70E-02	transcription_start_site	+	108947	108947	-2939	21309	AN5189
1726	CONTIG89	104721	104975	1.49	5.88E-02	transcription_start_site	+	107983	107983	-3135	21307	AN5189
1726	CONTIG89	104721	104975	1.49	5.88E-02	transcription_start_site	+	108649	108649	-3801	21308	AN5189
1726	CONTIG89	104721	104975	1.49	5.88E-02	transcription_start_site	+	108947	108947	-4099	21309	AN5189
1400	CONTIG89	105316	106700	1.63	3.70E-02	transcription_start_site	+	110462	110462	-4454	21310	AN5190
1400	CONTIG89	105316	106700	1.63	3.70E-02	transcription_start_site	+	111661	111661	-5653	21311	AN5190
464	CONTIG89	119328	119619	2.49	1.01E-03	transcription_start_site	-	114482	114482	-4991	21313	AN5191
2567	CONTIG89	113701	114050	1.18	1.50E-01	transcription_start_site	-	113813	113813	-62	21314	AN5191
2567	CONTIG89	113701	114050	1.18	1.50E-01	transcription_start_site	-	114482	114482	606	21313	AN5191
464	CONTIG89	119328	119619	2.49	1.01E-03	transcription_start_site	-	117344	117344	-2129	21315	AN5192
464	CONTIG89	119328	119619	2.49	1.01E-03	transcription_start_site	-	116849	116849	-2624	21316	AN5192
464	CONTIG89	119328	119619	2.49	1.01E-03	transcription_start_site	-	116772	116772	-2701	21317	AN5192
464	CONTIG89	119328	119619	2.49	1.01E-03	transcription_start_site	-	115940	115940	-3533	21318	AN5192
464	CONTIG89	119328	119619	2.49	1.01E-03	transcription_start_site	-	119065	119065	-408	21319	AN5193
464	CONTIG89	119328	119619	2.49	1.01E-03	transcription_start_site	-	118972	118972	-501	21320	AN5193
464	CONTIG89	119328	119619	2.49	1.01E-03	transcription_start_site	-	118456	118456	-1017	21321	AN5193
464	CONTIG89	119328	119619	2.49	1.01E-03	transcription_start_site	+	120088	120088	-614	21322	AN5194
464	CONTIG89	119328	119619	2.49	1.01E-03	transcription_start_site	+	121400	121400	-1926	21323	AN5194
464	CONTIG89	119328	119619	2.49	1.01E-03	transcription_start_site	+	122847	122847	-3373	21324	AN5194
464	CONTIG89	119328	119619	2.49	1.01E-03	transcription_start_site	+	123662	123662	-4188	21325	AN5195
464	CONTIG89	119328	119619	2.49	1.01E-03	transcription_start_site	+	123869	123869	-4395	21326	AN5195
464	CONTIG89	119328	119619	2.49	1.01E-03	transcription_start_site	+	124101	124101	-4627	21327	AN5195
464	CONTIG89	119328	119619	2.49	1.01E-03	transcription_start_site	+	124229	124229	-4755	21328	AN5195
224	CONTIG89	127218	127552	2.89	0.00E+00	transcription_start_site	+	126846	126846	539	21331	AN5196
2043	CONTIG89	135765	136189	1.36	9.24E-02	transcription_start_site	-	132614	132614	-3363	21333	AN5198
2043	CONTIG89	135765	136189	1.36	9.24E-02	transcription_start_site	-	132371	132371	-3606	21334	AN5198
2043	CONTIG89	135765	136189	1.36	9.24E-02	transcription_start_site	-	132057	132057	-3920	21335	AN5198
2043	CONTIG89	135765	136189	1.36	9.24E-02	transcription_start_site	-	131858	131858	-4119	21336	AN5198
2043	CONTIG89	135765	136189	1.36	9.24E-02	transcription_start_site	-	131778	131778	-4199	21337	AN5198
2043	CONTIG89	135765	136189	1.36	9.24E-02	transcription_start_site	-	131041	131041	-4936	21338	AN5198
2043	CONTIG89	135765	136189	1.36	9.24E-02	transcription_start_site	-	134381	134381	-1596	21339	AN5199
2043	CONTIG89	135765	136189	1.36	9.24E-02	transcription_start_site	-	134174	134174	-1803	21340	AN5199
2043	CONTIG89	135765	136189	1.36	9.24E-02	transcription_start_site	-	133711	133711	-2266	21341	AN5199
2043	CONTIG89	135765	136189	1.36	9.24E-02	transcription_start_site	+	135354	135354	623	21344	AN5200
2043	CONTIG89	135765	136189	1.36	9.24E-02	transcription_start_site	+	135084	135084	893	21343	AN5200
2043	CONTIG89	135765	136189	1.36	9.24E-02	transcription_start_site	+	134802	134802	1175	21342	AN5200
817	CONTIG89	146855	147279	2.08	6.61E-03	transcription_start_site	-	142174	142174	-4893	21348	AN5202
817	CONTIG89	146855	147279	2.08	6.61E-03	transcription_start_site	-	145540	145540	-1527	21351	AN5203
817	CONTIG89	146855	147279	2.08	6.61E-03	transcription_start_site	+	146377	146377	690	21354	AN5204
817	CONTIG89	146855	147279	2.08	6.61E-03	transcription_start_site	+	146160	146160	907	21353	AN5204
817	CONTIG89	146855	147279	2.08	6.61E-03	transcription_start_site	+	146070	146070	997	21352	AN5204
2568	CONTIG89	153755	154044	1.18	1.66E-01	transcription_start_site	-	150562	150562	-3337	21355	AN5205
2568	CONTIG89	153755	154044	1.18	1.66E-01	transcription_start_site	+	153777	153777	122	21359	AN5206
2568	CONTIG89	153755	154044	1.18	1.66E-01	transcription_start_site	+	153551	153551	348	21358	AN5206
2568	CONTIG89	153755	154044	1.18	1.66E-01	transcription_start_site	+	153447	153447	452	21357	AN5206
2568	CONTIG89	153755	154044	1.18	1.66E-01	transcription_start_site	+	153353	153353	546	21356	AN5206

1401	CONTIG89	155780	156069	1.63	3.70E-02	transcription_start_site	-	155884	155884	-40	21360	AN5207
1401	CONTIG89	155780	156069	1.63	3.70E-02	transcription_start_site	-	155654	155654	-270	21361	AN5207
1401	CONTIG89	155780	156069	1.63	3.70E-02	transcription_start_site	-	155056	155056	-868	21362	AN5207
2171	CONTIG89	162995	163264	1.31	1.08E-01	transcription_start_site	-	159140	159140	-3989	21363	AN5208
2171	CONTIG89	162995	163264	1.31	1.08E-01	transcription_start_site	-	158817	158817	-4312	21364	AN5208
1401	CONTIG89	155780	156069	1.63	3.70E-02	transcription_start_site	+	159729	159729	-3804	21365	AN5209
1401	CONTIG89	155780	156069	1.63	3.70E-02	transcription_start_site	+	161028	161028	-5103	21366	AN5209
2171	CONTIG89	162995	163264	1.31	1.08E-01	transcription_start_site	+	163364	163364	-234	21367	AN5210
2171	CONTIG89	162995	163264	1.31	1.08E-01	transcription_start_site	+	163560	163560	-430	21368	AN5210
2171	CONTIG89	162995	163264	1.31	1.08E-01	transcription_start_site	+	163656	163656	-526	21369	AN5210
2171	CONTIG89	162995	163264	1.31	1.08E-01	transcription_start_site	+	163761	163761	-631	21370	AN5210
2171	CONTIG89	162995	163264	1.31	1.08E-01	transcription_start_site	+	163894	163894	-764	21371	AN5210
2171	CONTIG89	162995	163264	1.31	1.08E-01	transcription_start_site	+	164071	164071	-941	21372	AN5210
2171	CONTIG89	162995	163264	1.31	1.08E-01	transcription_start_site	+	164955	164955	-1825	21373	AN5210
2171	CONTIG89	162995	163264	1.31	1.08E-01	transcription_start_site	+	165086	165086	-1956	21374	AN5210
731	CONTIG89	181438	181780	2.17	4.61E-03	transcription_start_site	-	181089	181089	-520	21381	AN5213
731	CONTIG89	181438	181780	2.17	4.61E-03	transcription_start_site	-	180895	180895	-714	21382	AN5213
966	CONTIG89	192618	193267	1.94	1.11E-02	transcription_start_site	-	189010	189010	-3932	21389	AN5215
966	CONTIG89	192618	193267	1.94	1.11E-02	transcription_start_site	-	188947	188947	-3995	21390	AN5215
966	CONTIG89	192618	193267	1.94	1.11E-02	transcription_start_site	+	193766	193766	-823	21392	AN5217
966	CONTIG89	192618	193267	1.94	1.11E-02	transcription_start_site	+	193937	193937	-994	21393	AN5217
966	CONTIG89	192618	193267	1.94	1.11E-02	transcription_start_site	+	194119	194119	-1176	21394	AN5217
966	CONTIG89	192618	193267	1.94	1.11E-02	transcription_start_site	+	194350	194350	-1407	21395	AN5217
1598	CONTIG89	200256	200530	1.54	5.22E-02	transcription_start_site	-	200058	200058	-335	21396	AN5218
1598	CONTIG89	200256	200530	1.54	5.22E-02	transcription_start_site	-	199217	199217	-1176	21397	AN5218
1598	CONTIG89	200256	200530	1.54	5.22E-02	transcription_start_site	-	201433	201433	1040	21401	AN5219
1598	CONTIG89	200256	200530	1.54	5.22E-02	transcription_start_site	+	202631	202631	-2238	21402	AN5220
1598	CONTIG89	200256	200530	1.54	5.22E-02	transcription_start_site	+	202799	202799	-2406	21403	AN5220
1598	CONTIG89	200256	200530	1.54	5.22E-02	transcription_start_site	+	202971	202971	-2578	21404	AN5220
1598	CONTIG89	200256	200530	1.54	5.22E-02	transcription_start_site	+	203362	203362	-2969	21405	AN5220
1598	CONTIG89	200256	200530	1.54	5.22E-02	transcription_start_site	+	204081	204081	-3688	21406	AN5220
1598	CONTIG89	200256	200530	1.54	5.22E-02	transcription_start_site	+	204677	204677	-4284	21407	AN5220
414	CONTIG89	218043	218767	2.58	0.00E+00	transcription_start_site	-	214665	214665	-3740	21416	AN5223
414	CONTIG89	218043	218767	2.58	0.00E+00	transcription_start_site	-	214196	214196	-4209	21417	AN5223
414	CONTIG89	218043	218767	2.58	0.00E+00	transcription_start_site	-	214088	214088	-4317	21418	AN5223
596	CONTIG89	217218	218012	2.31	0.00E+00	transcription_start_site	-	214665	214665	-2950	21416	AN5223
596	CONTIG89	217218	218012	2.31	0.00E+00	transcription_start_site	-	214196	214196	-3419	21417	AN5223
596	CONTIG89	217218	218012	2.31	0.00E+00	transcription_start_site	-	214088	214088	-3527	21418	AN5223
596	CONTIG89	217218	218012	2.31	0.00E+00	transcription_start_site	+	216553	216553	1062	21423	AN5224
596	CONTIG89	217218	218012	2.31	0.00E+00	transcription_start_site	+	216386	216386	1229	21422	AN5224
414	CONTIG89	218043	218767	2.58	0.00E+00	transcription_start_site	+	218647	218647	-242	21424	AN5225
414	CONTIG89	218043	218767	2.58	0.00E+00	transcription_start_site	+	219609	219609	-1204	21425	AN5225
414	CONTIG89	218043	218767	2.58	0.00E+00	transcription_start_site	+	219970	219970	-1565	21426	AN5225
414	CONTIG89	218043	218767	2.58	0.00E+00	transcription_start_site	+	220131	220131	-1726	21427	AN5225
596	CONTIG89	217218	218012	2.31	0.00E+00	transcription_start_site	+	218647	218647	-1032	21424	AN5225
596	CONTIG89	217218	218012	2.31	0.00E+00	transcription_start_site	+	219609	219609	-1994	21425	AN5225
596	CONTIG89	217218	218012	2.31	0.00E+00	transcription_start_site	+	219970	219970	-2355	21426	AN5225
596	CONTIG89	217218	218012	2.31	0.00E+00	transcription_start_site	+	220131	220131	-2516	21427	AN5225
24	CONTIG89	224567	225366	3.57	0.00E+00	transcription_start_site	-	223332	223332	-1634	21428	AN5226
24	CONTIG89	224567	225366	3.57	0.00E+00	transcription_start_site	-	222866	222866	-2100	21429	AN5226
24	CONTIG89	224567	225366	3.57	0.00E+00	transcription_start_site	-	222641	222641	-2325	21430	AN5226
765	CONTIG89	227552	227901	2.13	2.32E-03	transcription_start_site	-	223332	223332	-4394	21428	AN5226
765	CONTIG89	227552	227901	2.13	2.32E-03	transcription_start_site	-	222866	222866	-4860	21429	AN5226
765	CONTIG89	227552	227901	2.13	2.32E-03	transcription_start_site	-	222641	222641	-5085	21430	AN5226
765	CONTIG89	227552	227901	2.13	2.32E-03	transcription_start_site	+	232393	232393	-4666	21431	AN5227
765	CONTIG89	227552	227901	2.13	2.32E-03	transcription_start_site	+	232572	232572	-4845	21432	AN5227
2449	CONTIG89	238051	239020	1.22	1.44E-01	transcription_start_site	-	237276	237276	-1259	21434	AN5228

2569	CONTIG89	236570	237204	1.18	1.66E-01	transcription_start_site	-	237276	237276	389	21434 AN5228
1926	CONTIG89	242852	243216	1.4	8.01E-02	transcription_start_site	-	240909	240909	-2125	21435 AN5229
1926	CONTIG89	242852	243216	1.4	8.01E-02	transcription_start_site	-	240838	240838	-2196	21436 AN5229
1926	CONTIG89	242852	243216	1.4	8.01E-02	transcription_start_site	-	240741	240741	-2293	21437 AN5229
1926	CONTIG89	242852	243216	1.4	8.01E-02	transcription_start_site	-	240326	240326	-2708	21438 AN5229
1926	CONTIG89	242852	243216	1.4	8.01E-02	transcription_start_site	-	238244	238244	-4790	21439 AN5229
2449	CONTIG89	238051	239020	1.22	1.44E-01	transcription_start_site	-	238244	238244	-291	21439 AN5229
2449	CONTIG89	238051	239020	1.22	1.44E-01	transcription_start_site	+	241183	241183	-2647	21440 AN5230
2449	CONTIG89	238051	239020	1.22	1.44E-01	transcription_start_site	+	241705	241705	-3169	21441 AN5230
2569	CONTIG89	236570	237204	1.18	1.66E-01	transcription_start_site	+	241183	241183	-4296	21440 AN5230
2569	CONTIG89	236570	237204	1.18	1.66E-01	transcription_start_site	+	241705	241705	-4818	21441 AN5230
1926	CONTIG89	242852	243216	1.4	8.01E-02	transcription_start_site	+	242821	242821	213	21442 AN5231
2449	CONTIG89	238051	239020	1.22	1.44E-01	transcription_start_site	+	242821	242821	-4285	21442 AN5231
2163	CONTIG89	252383	253402	1.31	8.36E-02	transcription_start_site	-	249094	249094	-3798	21444 AN5233
2163	CONTIG89	252383	253402	1.31	8.36E-02	transcription_start_site	-	251095	251095	-1797	21448 AN5234
2163	CONTIG89	252383	253402	1.31	8.36E-02	transcription_start_site	-	250702	250702	-2190	21449 AN5234
2163	CONTIG89	252383	253402	1.31	8.36E-02	transcription_start_site	+	251737	251737	1155	21451 AN5235
2163	CONTIG89	252383	253402	1.31	8.36E-02	transcription_start_site	+	251637	251637	1255	21450 AN5235
2163	CONTIG89	252383	253402	1.31	8.36E-02	transcription_start_site	+	253706	253706	-813	21452 AN5236
2163	CONTIG89	252383	253402	1.31	8.36E-02	transcription_start_site	+	253963	253963	-1070	21453 AN5236
2163	CONTIG89	252383	253402	1.31	8.36E-02	transcription_start_site	+	254223	254223	-1330	21454 AN5236
2252	CONTIG90	2486	3205	1.28	5.88E-02	transcription_start_site	-	2710	2710	-135	21474 AN5245
2252	CONTIG90	2486	3205	1.28	5.88E-02	transcription_start_site	-	3126	3126	280	21473 AN5245
3018	CONTIG92	50483	50912	0.88	5.05E-02	transcription_start_site	-	45899	45899	-4798	21542 AN5259
3018	CONTIG92	50483	50912	0.88	5.05E-02	transcription_start_site	-	45699	45699	-4998	21543 AN5259
3018	CONTIG92	50483	50912	0.88	5.05E-02	transcription_start_site	-	45581	45581	-5116	21544 AN5259
3018	CONTIG92	50483	50912	0.88	5.05E-02	transcription_start_site	-	49243	49243	-1454	21556 AN5261
3018	CONTIG92	50483	50912	0.88	5.05E-02	transcription_start_site	-	49172	49172	-1525	21557 AN5261
3018	CONTIG92	50483	50912	0.88	5.05E-02	transcription_start_site	-	48967	48967	-1730	21558 AN5261
3018	CONTIG92	50483	50912	0.88	5.05E-02	transcription_start_site	-	48593	48593	-2104	21559 AN5261
3018	CONTIG92	50483	50912	0.88	5.05E-02	transcription_start_site	-	48362	48362	-2335	21560 AN5261
3018	CONTIG92	50483	50912	0.88	5.05E-02	transcription_start_site	+	50285	50285	412	21562 AN5262
3018	CONTIG92	50483	50912	0.88	5.05E-02	transcription_start_site	+	51204	51204	-506	21563 AN5262
3018	CONTIG92	50483	50912	0.88	5.05E-02	transcription_start_site	+	49997	49997	700	21561 AN5262
829	CONTIG93	25428	25867	2.07	8.81E-03	transcription_start_site	+	26242	26242	-594	21609 AN5274
829	CONTIG93	25428	25867	2.07	8.81E-03	transcription_start_site	+	26362	26362	-714	21610 AN5274
829	CONTIG93	25428	25867	2.07	8.81E-03	transcription_start_site	+	26486	26486	-838	21611 AN5274
829	CONTIG93	25428	25867	2.07	8.81E-03	transcription_start_site	+	26882	26882	-1234	21612 AN5274
829	CONTIG93	25428	25867	2.07	8.81E-03	transcription_start_site	+	27848	27848	-2200	21613 AN5274
829	CONTIG93	25428	25867	2.07	8.81E-03	transcription_start_site	+	29070	29070	-3422	21614 AN5275
829	CONTIG93	25428	25867	2.07	8.81E-03	transcription_start_site	+	29161	29161	-3513	21615 AN5275
829	CONTIG93	25428	25867	2.07	8.81E-03	transcription_start_site	+	29542	29542	-3894	21616 AN5275
829	CONTIG93	25428	25867	2.07	8.81E-03	transcription_start_site	+	29823	29823	-4175	21617 AN5275
829	CONTIG93	25428	25867	2.07	8.81E-03	transcription_start_site	+	30228	30228	-4580	21618 AN5275
829	CONTIG93	25428	25867	2.07	8.81E-03	transcription_start_site	+	30791	30791	-5143	21619 AN5275
377	CONTIG93	55819	56090	2.64	9.51E-04	transcription_start_site	+	56062	56062	-107	21660 AN5283
377	CONTIG93	55819	56090	2.64	9.51E-04	transcription_start_site	+	56881	56881	-926	21661 AN5283
409	CONTIG93	97727	98226	2.59	1.01E-03	transcription_start_site	-	97067	97067	-909	21709 AN5295
409	CONTIG93	97727	98226	2.59	1.01E-03	transcription_start_site	+	98122	98122	-145	21710 AN5296
409	CONTIG93	97727	98226	2.59	1.01E-03	transcription_start_site	+	101728	101728	-3751	21712 AN5298
409	CONTIG93	97727	98226	2.59	1.01E-03	transcription_start_site	+	101978	101978	-4001	21713 AN5298
409	CONTIG93	97727	98226	2.59	1.01E-03	transcription_start_site	+	102346	102346	-4369	21714 AN5298
1291	CONTIG93	140859	141358	1.7	2.18E-02	transcription_start_site	-	137064	137064	-4044	21742 AN5309
1291	CONTIG93	140859	141358	1.7	2.18E-02	transcription_start_site	-	136924	136924	-4184	21743 AN5309
1291	CONTIG93	140859	141358	1.7	2.18E-02	transcription_start_site	-	136782	136782	-4326	21744 AN5309
1291	CONTIG93	140859	141358	1.7	2.18E-02	transcription_start_site	-	141152	141152	43	21752 AN5311
1291	CONTIG93	140859	141358	1.7	2.18E-02	transcription_start_site	-	141479	141479	370	21751 AN5311

1291	CONTIG93	140859	141358	1.7	2.18E-02	transcription_start_site	-	141714	141714	605	21750	AN5311
1291	CONTIG93	140859	141358	1.7	2.18E-02	transcription_start_site	-	141963	141963	854	21749	AN5311
1551	CONTIG93	142964	144063	1.56	3.99E-02	transcription_start_site	-	141963	141963	-1550	21749	AN5311
1551	CONTIG93	142964	144063	1.56	3.99E-02	transcription_start_site	-	141714	141714	-1799	21750	AN5311
1551	CONTIG93	142964	144063	1.56	3.99E-02	transcription_start_site	-	141479	141479	-2034	21751	AN5311
1551	CONTIG93	142964	144063	1.56	3.99E-02	transcription_start_site	-	141152	141152	-2361	21752	AN5311
1291	CONTIG93	140859	141358	1.7	2.18E-02	transcription_start_site	+	144794	144794	-3685	21753	AN5312
1291	CONTIG93	140859	141358	1.7	2.18E-02	transcription_start_site	+	145038	145038	-3929	21754	AN5312
1291	CONTIG93	140859	141358	1.7	2.18E-02	transcription_start_site	+	145322	145322	-4213	21755	AN5312
1291	CONTIG93	140859	141358	1.7	2.18E-02	transcription_start_site	+	145520	145520	-4411	21756	AN5312
1291	CONTIG93	140859	141358	1.7	2.18E-02	transcription_start_site	+	145710	145710	-4601	21757	AN5312
1551	CONTIG93	142964	144063	1.56	3.99E-02	transcription_start_site	+	144794	144794	-1280	21753	AN5312
1551	CONTIG93	142964	144063	1.56	3.99E-02	transcription_start_site	+	145038	145038	-1524	21754	AN5312
1551	CONTIG93	142964	144063	1.56	3.99E-02	transcription_start_site	+	145322	145322	-1808	21755	AN5312
1551	CONTIG93	142964	144063	1.56	3.99E-02	transcription_start_site	+	145520	145520	-2006	21756	AN5312
1551	CONTIG93	142964	144063	1.56	3.99E-02	transcription_start_site	+	145710	145710	-2196	21757	AN5312
532	CONTIG93	163960	164244	2.4	2.22E-03	transcription_start_site	-	161650	161650	-2452	21767	AN5317
532	CONTIG93	163960	164244	2.4	2.22E-03	transcription_start_site	-	161143	161143	-2959	21768	AN5317
2010	CONTIG93	164420	165070	1.37	9.26E-02	transcription_start_site	-	161650	161650	-3095	21767	AN5317
2010	CONTIG93	164420	165070	1.37	9.26E-02	transcription_start_site	-	161143	161143	-3602	21768	AN5317
532	CONTIG93	163960	164244	2.4	2.22E-03	transcription_start_site	+	164592	164592	-490	21769	AN5318
2010	CONTIG93	164420	165070	1.37	9.26E-02	transcription_start_site	+	164592	164592	153	21769	AN5318
165	CONTIG93	181576	181870	3.02	0.00E+00	transcription_start_site	-	177062	177062	-4661	21778	AN5321
165	CONTIG93	181576	181870	3.02	0.00E+00	transcription_start_site	-	176713	176713	-5010	21779	AN5321
165	CONTIG93	181576	181870	3.02	0.00E+00	transcription_start_site	-	180921	180921	-802	21784	AN5323
165	CONTIG93	181576	181870	3.02	0.00E+00	transcription_start_site	-	180508	180508	-1215	21785	AN5323
165	CONTIG93	181576	181870	3.02	0.00E+00	transcription_start_site	-	180379	180379	-1344	21786	AN5323
165	CONTIG93	181576	181870	3.02	0.00E+00	transcription_start_site	-	179992	179992	-1731	21787	AN5323
345	CONTIG93	183241	184571	2.69	0.00E+00	transcription_start_site	-	180921	180921	-2985	21784	AN5323
345	CONTIG93	183241	184571	2.69	0.00E+00	transcription_start_site	-	180508	180508	-3398	21785	AN5323
345	CONTIG93	183241	184571	2.69	0.00E+00	transcription_start_site	-	180379	180379	-3527	21786	AN5323
345	CONTIG93	183241	184571	2.69	0.00E+00	transcription_start_site	-	179992	179992	-3914	21787	AN5323
1025	CONTIG93	185105	185459	1.89	1.84E-02	transcription_start_site	-	180921	180921	-4361	21784	AN5323
1025	CONTIG93	185105	185459	1.89	1.84E-02	transcription_start_site	-	180508	180508	-4774	21785	AN5323
1025	CONTIG93	185105	185459	1.89	1.84E-02	transcription_start_site	-	180379	180379	-4903	21786	AN5323
2141	CONTIG93	182336	182975	1.32	1.25E-01	transcription_start_site	-	180921	180921	-1734	21784	AN5323
2141	CONTIG93	182336	182975	1.32	1.25E-01	transcription_start_site	-	180508	180508	-2147	21785	AN5323
2141	CONTIG93	182336	182975	1.32	1.25E-01	transcription_start_site	-	180379	180379	-2276	21786	AN5323
2141	CONTIG93	182336	182975	1.32	1.25E-01	transcription_start_site	-	179992	179992	-2663	21787	AN5323
345	CONTIG93	183241	184571	2.69	0.00E+00	transcription_start_site	-	184515	184515	609	21788	AN5324
345	CONTIG93	183241	184571	2.69	0.00E+00	transcription_start_site	-	183047	183047	-859	21789	AN5324
1025	CONTIG93	185105	185459	1.89	1.84E-02	transcription_start_site	-	184515	184515	-767	21788	AN5324
1025	CONTIG93	185105	185459	1.89	1.84E-02	transcription_start_site	-	183047	183047	-2235	21789	AN5324
2141	CONTIG93	182336	182975	1.32	1.25E-01	transcription_start_site	-	183047	183047	391	21789	AN5324
345	CONTIG93	183241	184571	2.69	0.00E+00	transcription_start_site	+	188097	188097	-4191	21790	AN5325
345	CONTIG93	183241	184571	2.69	0.00E+00	transcription_start_site	+	188419	188419	-4513	21791	AN5325
1025	CONTIG93	185105	185459	1.89	1.84E-02	transcription_start_site	+	188097	188097	-2815	21790	AN5325
1025	CONTIG93	185105	185459	1.89	1.84E-02	transcription_start_site	+	188419	188419	-3137	21791	AN5325
345	CONTIG93	183241	184571	2.69	0.00E+00	transcription_start_site	+	189394	189394	-5488	21792	AN5326
1025	CONTIG93	185105	185459	1.89	1.84E-02	transcription_start_site	+	189394	189394	-4112	21792	AN5326
1025	CONTIG93	185105	185459	1.89	1.84E-02	transcription_start_site	+	189769	189769	-4487	21793	AN5326
1025	CONTIG93	185105	185459	1.89	1.84E-02	transcription_start_site	+	190053	190053	-4771	21794	AN5326
1025	CONTIG93	185105	185459	1.89	1.84E-02	transcription_start_site	+	190337	190337	-5055	21795	AN5326
1559	CONTIG93	231089	231443	1.56	5.88E-02	transcription_start_site	-	227053	227053	-4213	21825	AN5339
1559	CONTIG93	231089	231443	1.56	5.88E-02	transcription_start_site	-	226890	226890	-4376	21826	AN5339
1559	CONTIG93	231089	231443	1.56	5.88E-02	transcription_start_site	-	226605	226605	-4661	21827	AN5339
145	CONTIG93	233264	233618	3.06	0.00E+00	transcription_start_site	-	228558	228558	-4883	21828	AN5340

1559	CONTIG93	231089	231443	1.56	5.88E-02	transcription_start_site	-	228558	228558	-2708	21828 AN5340
145	CONTIG93	233264	233618	3.06	0.00E+00	transcription_start_site	+	232580	232580	861	21830 AN5341
145	CONTIG93	233264	233618	3.06	0.00E+00	transcription_start_site	+	232371	232371	1070	21829 AN5341
1559	CONTIG93	231089	231443	1.56	5.88E-02	transcription_start_site	+	232371	232371	-1105	21829 AN5341
1559	CONTIG93	231089	231443	1.56	5.88E-02	transcription_start_site	+	232580	232580	-1314	21830 AN5341
145	CONTIG93	233264	233618	3.06	0.00E+00	transcription_start_site	+	233821	233821	-380	21831 AN5342
145	CONTIG93	233264	233618	3.06	0.00E+00	transcription_start_site	+	234206	234206	-765	21832 AN5342
1559	CONTIG93	231089	231443	1.56	5.88E-02	transcription_start_site	+	233821	233821	-2555	21831 AN5342
1559	CONTIG93	231089	231443	1.56	5.88E-02	transcription_start_site	+	234206	234206	-2940	21832 AN5342
2142	CONTIG93	256285	256564	1.32	1.25E-01	transcription_start_site	-	253021	253021	-3403	21854 AN5349
2142	CONTIG93	256285	256564	1.32	1.25E-01	transcription_start_site	-	252936	252936	-3488	21855 AN5349
2142	CONTIG93	256285	256564	1.32	1.25E-01	transcription_start_site	-	252566	252566	-3858	21856 AN5349
872	CONTIG93	258170	258504	2.03	1.11E-02	transcription_start_site	-	256217	256217	-2120	21860 AN5351
872	CONTIG93	258170	258504	2.03	1.11E-02	transcription_start_site	-	255965	255965	-2372	21861 AN5351
872	CONTIG93	258170	258504	2.03	1.11E-02	transcription_start_site	-	255802	255802	-2535	21862 AN5351
2142	CONTIG93	256285	256564	1.32	1.25E-01	transcription_start_site	-	256217	256217	-207	21860 AN5351
2142	CONTIG93	256285	256564	1.32	1.25E-01	transcription_start_site	-	255965	255965	-459	21861 AN5351
2142	CONTIG93	256285	256564	1.32	1.25E-01	transcription_start_site	-	255802	255802	-622	21862 AN5351
346	CONTIG93	264980	265344	2.69	8.69E-04	transcription_start_site	-	262385	262385	-2777	21863 AN5353
346	CONTIG93	264980	265344	2.69	8.69E-04	transcription_start_site	-	262246	262246	-2916	21864 AN5353
636	CONTIG93	267320	267579	2.26	4.61E-03	transcription_start_site	-	262385	262385	-5064	21863 AN5353
1161	CONTIG93	266030	266314	1.79	2.62E-02	transcription_start_site	-	262385	262385	-3787	21863 AN5353
1161	CONTIG93	266030	266314	1.79	2.62E-02	transcription_start_site	-	262246	262246	-3926	21864 AN5353
346	CONTIG93	264980	265344	2.69	8.69E-04	transcription_start_site	+	265652	265652	-490	21865 AN5354
346	CONTIG93	264980	265344	2.69	8.69E-04	transcription_start_site	+	265778	265778	-616	21866 AN5354
346	CONTIG93	264980	265344	2.69	8.69E-04	transcription_start_site	+	266126	266126	-964	21867 AN5354
1161	CONTIG93	266030	266314	1.79	2.62E-02	transcription_start_site	+	266126	266126	46	21867 AN5354
1161	CONTIG93	266030	266314	1.79	2.62E-02	transcription_start_site	+	265778	265778	394	21866 AN5354
1161	CONTIG93	266030	266314	1.79	2.62E-02	transcription_start_site	+	265652	265652	520	21865 AN5354
346	CONTIG93	264980	265344	2.69	8.69E-04	transcription_start_site	+	267703	267703	-2541	21868 AN5355
346	CONTIG93	264980	265344	2.69	8.69E-04	transcription_start_site	+	267948	267948	-2786	21869 AN5355
636	CONTIG93	267320	267579	2.26	4.61E-03	transcription_start_site	+	267703	267703	-253	21868 AN5355
636	CONTIG93	267320	267579	2.26	4.61E-03	transcription_start_site	+	267948	267948	-498	21869 AN5355
1161	CONTIG93	266030	266314	1.79	2.62E-02	transcription_start_site	+	267703	267703	-1531	21868 AN5355
1161	CONTIG93	266030	266314	1.79	2.62E-02	transcription_start_site	+	267948	267948	-1776	21869 AN5355
407	CONTIG93	272560	273214	2.59	0.00E+00	transcription_start_site	-	272549	272549	-338	21870 AN5356
407	CONTIG93	272560	273214	2.59	0.00E+00	transcription_start_site	-	272249	272249	-638	21871 AN5356
407	CONTIG93	272560	273214	2.59	0.00E+00	transcription_start_site	-	272147	272147	-740	21872 AN5356
407	CONTIG93	272560	273214	2.59	0.00E+00	transcription_start_site	-	271468	271468	-1419	21873 AN5356
115	CONTIG93	279615	279954	3.16	0.00E+00	transcription_start_site	-	279054	279054	-730	21874 AN5357
115	CONTIG93	279615	279954	3.16	0.00E+00	transcription_start_site	-	278922	278922	-862	21875 AN5357
115	CONTIG93	279615	279954	3.16	0.00E+00	transcription_start_site	-	278694	278694	-1090	21876 AN5357
115	CONTIG93	279615	279954	3.16	0.00E+00	transcription_start_site	-	278229	278229	-1555	21877 AN5357
2419	CONTIG93	280888	281527	1.23	1.50E-01	transcription_start_site	-	279054	279054	-2153	21874 AN5357
2419	CONTIG93	280888	281527	1.23	1.50E-01	transcription_start_site	-	278922	278922	-2285	21875 AN5357
2419	CONTIG93	280888	281527	1.23	1.50E-01	transcription_start_site	-	278694	278694	-2513	21876 AN5357
2419	CONTIG93	280888	281527	1.23	1.50E-01	transcription_start_site	-	278229	278229	-2978	21877 AN5357
2572	CONTIG93	278410	278981	1.18	1.93E-01	transcription_start_site	-	278694	278694	-1	21876 AN5357
2572	CONTIG93	278410	278981	1.18	1.93E-01	transcription_start_site	-	278922	278922	226	21875 AN5357
2572	CONTIG93	278410	278981	1.18	1.93E-01	transcription_start_site	-	279054	279054	358	21874 AN5357
2572	CONTIG93	278410	278981	1.18	1.93E-01	transcription_start_site	-	278229	278229	-466	21877 AN5357
115	CONTIG93	279615	279954	3.16	0.00E+00	transcription_start_site	-	280005	280005	220	21880 AN5358
115	CONTIG93	279615	279954	3.16	0.00E+00	transcription_start_site	-	280424	280424	639	21879 AN5358
115	CONTIG93	279615	279954	3.16	0.00E+00	transcription_start_site	-	280582	280582	797	21878 AN5358
2419	CONTIG93	280888	281527	1.23	1.50E-01	transcription_start_site	-	280582	280582	-625	21878 AN5358
2419	CONTIG93	280888	281527	1.23	1.50E-01	transcription_start_site	-	280424	280424	-783	21879 AN5358
2419	CONTIG93	280888	281527	1.23	1.50E-01	transcription_start_site	-	280005	280005	-1202	21880 AN5358

2419	CONTIG93	280888	281527	1.23	1.50E-01	transcription_start_site	-	281511	281511	303	21881 AN5359
115	CONTIG93	279615	279954	3.16	0.00E+00	transcription_start_site	+	282134	282134	-2349	21882 AN5360
115	CONTIG93	279615	279954	3.16	0.00E+00	transcription_start_site	+	283301	283301	-3516	21883 AN5360
115	CONTIG93	279615	279954	3.16	0.00E+00	transcription_start_site	+	283464	283464	-3679	21884 AN5360
115	CONTIG93	279615	279954	3.16	0.00E+00	transcription_start_site	+	283717	283717	-3932	21885 AN5360
115	CONTIG93	279615	279954	3.16	0.00E+00	transcription_start_site	+	283909	283909	-4124	21886 AN5360
115	CONTIG93	279615	279954	3.16	0.00E+00	transcription_start_site	+	284016	284016	-4231	21887 AN5360
2419	CONTIG93	280888	281527	1.23	1.50E-01	transcription_start_site	+	282134	282134	-926	21882 AN5360
2419	CONTIG93	280888	281527	1.23	1.50E-01	transcription_start_site	+	283301	283301	-2093	21883 AN5360
2419	CONTIG93	280888	281527	1.23	1.50E-01	transcription_start_site	+	283464	283464	-2256	21884 AN5360
2419	CONTIG93	280888	281527	1.23	1.50E-01	transcription_start_site	+	283717	283717	-2509	21885 AN5360
2419	CONTIG93	280888	281527	1.23	1.50E-01	transcription_start_site	+	283909	283909	-2701	21886 AN5360
2419	CONTIG93	280888	281527	1.23	1.50E-01	transcription_start_site	+	284016	284016	-2808	21887 AN5360
2572	CONTIG93	278410	278981	1.18	1.93E-01	transcription_start_site	+	282134	282134	-3438	21882 AN5360
2572	CONTIG93	278410	278981	1.18	1.93E-01	transcription_start_site	+	283301	283301	-4605	21883 AN5360
2572	CONTIG93	278410	278981	1.18	1.93E-01	transcription_start_site	+	283464	283464	-4768	21884 AN5360
2572	CONTIG93	278410	278981	1.18	1.93E-01	transcription_start_site	+	283717	283717	-5021	21885 AN5360
2572	CONTIG93	278410	278981	1.18	1.93E-01	transcription_start_site	+	283909	283909	-5213	21886 AN5360
2419	CONTIG93	280888	281527	1.23	1.50E-01	transcription_start_site	+	285862	285862	-4654	21888 AN5361
2015	CONTIG93	286958	287302	1.37	1.08E-01	transcription_start_site	-	288299	288299	1169	21890 AN5362
2015	CONTIG93	286958	287302	1.37	1.08E-01	transcription_start_site	+	292231	292231	-5101	21896 AN5364
1560	CONTIG93	297393	297654	1.56	5.88E-02	transcription_start_site	-	297475	297475	-48	21898 AN5365
1560	CONTIG93	297393	297654	1.56	5.88E-02	transcription_start_site	-	296807	296807	-716	21899 AN5365
1231	CONTIG93	303841	304190	1.74	1.81E-02	transcription_start_site	-	298867	298867	-5148	21900 AN5366
1560	CONTIG93	297393	297654	1.56	5.88E-02	transcription_start_site	-	298226	298226	702	21904 AN5366
1560	CONTIG93	297393	297654	1.56	5.88E-02	transcription_start_site	-	298338	298338	814	21903 AN5366
1560	CONTIG93	297393	297654	1.56	5.88E-02	transcription_start_site	-	298452	298452	928	21902 AN5366
1560	CONTIG93	297393	297654	1.56	5.88E-02	transcription_start_site	+	299345	299345	-1821	21905 AN5367
1560	CONTIG93	297393	297654	1.56	5.88E-02	transcription_start_site	+	299735	299735	-2211	21906 AN5367
1560	CONTIG93	297393	297654	1.56	5.88E-02	transcription_start_site	+	299848	299848	-2324	21907 AN5367
1560	CONTIG93	297393	297654	1.56	5.88E-02	transcription_start_site	+	301164	301164	-3640	21908 AN5367
1560	CONTIG93	297393	297654	1.56	5.88E-02	transcription_start_site	+	302581	302581	-5057	21909 AN5368
1231	CONTIG93	303841	304190	1.74	1.81E-02	transcription_start_site	+	303962	303962	53	21910 AN5369
1231	CONTIG93	303841	304190	1.74	1.81E-02	transcription_start_site	+	304430	304430	-414	21911 AN5369
1231	CONTIG93	303841	304190	1.74	1.81E-02	transcription_start_site	+	305652	305652	-1636	21912 AN5369
2573	CONTIG93	304596	305010	1.18	1.93E-01	transcription_start_site	+	304430	304430	373	21911 AN5369
2573	CONTIG93	304596	305010	1.18	1.93E-01	transcription_start_site	+	303962	303962	841	21910 AN5369
2573	CONTIG93	304596	305010	1.18	1.93E-01	transcription_start_site	+	305652	305652	-849	21912 AN5369
1231	CONTIG93	303841	304190	1.74	1.81E-02	transcription_start_site	+	307531	307531	-3515	21913 AN5370
1231	CONTIG93	303841	304190	1.74	1.81E-02	transcription_start_site	+	307894	307894	-3878	21914 AN5370
1231	CONTIG93	303841	304190	1.74	1.81E-02	transcription_start_site	+	308154	308154	-4138	21915 AN5370
1231	CONTIG93	303841	304190	1.74	1.81E-02	transcription_start_site	+	308352	308352	-4336	21916 AN5370
1231	CONTIG93	303841	304190	1.74	1.81E-02	transcription_start_site	+	308562	308562	-4546	21917 AN5370
1231	CONTIG93	303841	304190	1.74	1.81E-02	transcription_start_site	+	309137	309137	-5121	21918 AN5370
2573	CONTIG93	304596	305010	1.18	1.93E-01	transcription_start_site	+	307531	307531	-2728	21913 AN5370
2573	CONTIG93	304596	305010	1.18	1.93E-01	transcription_start_site	+	307894	307894	-3091	21914 AN5370
2573	CONTIG93	304596	305010	1.18	1.93E-01	transcription_start_site	+	308154	308154	-3351	21915 AN5370
2573	CONTIG93	304596	305010	1.18	1.93E-01	transcription_start_site	+	308352	308352	-3549	21916 AN5370
2573	CONTIG93	304596	305010	1.18	1.93E-01	transcription_start_site	+	308562	308562	-3759	21917 AN5370
2573	CONTIG93	304596	305010	1.18	1.93E-01	transcription_start_site	+	309137	309137	-4334	21918 AN5370
2573	CONTIG93	304596	305010	1.18	1.93E-01	transcription_start_site	+	309390	309390	-4587	21919 AN5370
8	CONTIG93	312463	312730	3.77	0.00E+00	transcription_start_site	-	312434	312434	-162	21920 AN5371
8	CONTIG93	312463	312730	3.77	0.00E+00	transcription_start_site	-	311124	311124	-1472	21921 AN5371
8	CONTIG93	312463	312730	3.77	0.00E+00	transcription_start_site	-	311052	311052	-1544	21922 AN5371
1906	CONTIG93	319139	319418	1.41	9.24E-02	transcription_start_site	-	314620	314620	-4658	21923 AN5372
1906	CONTIG93	319139	319418	1.41	9.24E-02	transcription_start_site	-	314554	314554	-4724	21924 AN5372
1906	CONTIG93	319139	319418	1.41	9.24E-02	transcription_start_site	-	316670	316670	-2608	21925 AN5373

1906	CONTIG93	319139	319418	1.41	9.24E-02	transcription_start_site	-	316540	316540	-2738	21926	AN5373
8	CONTIG93	312463	312730	3.77	0.00E+00	transcription_start_site	+	317583	317583	-4986	21927	AN5374
1906	CONTIG93	319139	319418	1.41	9.24E-02	transcription_start_site	-	319919	319919	640	21928	AN5375
1906	CONTIG93	319139	319418	1.41	9.24E-02	transcription_start_site	+	320318	320318	-1039	21929	AN5376
1906	CONTIG93	319139	319418	1.41	9.24E-02	transcription_start_site	+	320699	320699	-1420	21930	AN5376
1906	CONTIG93	319139	319418	1.41	9.24E-02	transcription_start_site	+	321170	321170	-1891	21931	AN5376
1906	CONTIG93	319139	319418	1.41	9.24E-02	transcription_start_site	+	321392	321392	-2113	21932	AN5376
1447	CONTIG94	3546	3885	1.61	1.84E-02	transcription_start_site	-	1102	1102	-2613	21958	AN5382
1447	CONTIG94	3546	3885	1.61	1.84E-02	transcription_start_site	+	5741	5741	-2025	21960	AN5384
1447	CONTIG94	3546	3885	1.61	1.84E-02	transcription_start_site	+	5838	5838	-2122	21961	AN5384
1447	CONTIG94	3546	3885	1.61	1.84E-02	transcription_start_site	+	8600	8600	-4884	21962	AN5385
2851	CONTIG94	30301	30810	1.04	1.66E-01	transcription_start_site	-	25394	25394	-5161	21968	AN5390
2851	CONTIG94	30301	30810	1.04	1.66E-01	transcription_start_site	-	31453	31453	897	21978	AN5393
2851	CONTIG94	30301	30810	1.04	1.66E-01	transcription_start_site	+	32289	32289	-1733	21979	AN5394
2851	CONTIG94	30301	30810	1.04	1.66E-01	transcription_start_site	+	32511	32511	-1955	21980	AN5394
2851	CONTIG94	30301	30810	1.04	1.66E-01	transcription_start_site	+	32755	32755	-2199	21981	AN5394
2851	CONTIG94	30301	30810	1.04	1.66E-01	transcription_start_site	+	33173	33173	-2617	21982	AN5394
2851	CONTIG94	30301	30810	1.04	1.66E-01	transcription_start_site	+	33510	33510	-2954	21983	AN5394
2851	CONTIG94	30301	30810	1.04	1.66E-01	transcription_start_site	+	33678	33678	-3122	21984	AN5394
2851	CONTIG94	30301	30810	1.04	1.66E-01	transcription_start_site	+	34616	34616	-4060	21985	AN5395
2851	CONTIG94	30301	30810	1.04	1.66E-01	transcription_start_site	+	34724	34724	-4168	21986	AN5395
2851	CONTIG94	30301	30810	1.04	1.66E-01	transcription_start_site	+	34785	34785	-4229	21987	AN5395
2851	CONTIG94	30301	30810	1.04	1.66E-01	transcription_start_site	+	34960	34960	-4404	21988	AN5395
2851	CONTIG94	30301	30810	1.04	1.66E-01	transcription_start_site	+	35414	35414	-4858	21989	AN5395
1819	CONTIG94	51845	52644	1.44	2.18E-02	transcription_start_site	-	48132	48132	-4112	21995	AN5398
1819	CONTIG94	51845	52644	1.44	2.18E-02	transcription_start_site	-	52313	52313	68	21996	AN5399
1819	CONTIG94	51845	52644	1.44	2.18E-02	transcription_start_site	-	51300	51300	-944	21997	AN5399
2852	CONTIG94	65412	65914	1.04	1.66E-01	transcription_start_site	-	61152	61152	-4511	22004	AN5401
2852	CONTIG94	65412	65914	1.04	1.66E-01	transcription_start_site	-	61047	61047	-4616	22005	AN5401
2852	CONTIG94	65412	65914	1.04	1.66E-01	transcription_start_site	-	60839	60839	-4824	22006	AN5401
2249	CONTIG94	67445	68679	1.28	4.42E-02	transcription_start_site	-	65170	65170	-2892	22007	AN5402
2249	CONTIG94	67445	68679	1.28	4.42E-02	transcription_start_site	-	64955	64955	-3107	22008	AN5402
2249	CONTIG94	67445	68679	1.28	4.42E-02	transcription_start_site	-	64633	64633	-3429	22009	AN5402
2852	CONTIG94	65412	65914	1.04	1.66E-01	transcription_start_site	-	65170	65170	-493	22007	AN5402
2852	CONTIG94	65412	65914	1.04	1.66E-01	transcription_start_site	-	64955	64955	-708	22008	AN5402
2852	CONTIG94	65412	65914	1.04	1.66E-01	transcription_start_site	-	64633	64633	-1030	22009	AN5402
2904	CONTIG94	69980	70700	1	1.79E-01	transcription_start_site	-	65170	65170	-5170	22007	AN5402
2249	CONTIG94	67445	68679	1.28	4.42E-02	transcription_start_site	-	67785	67785	-277	22013	AN5403
2249	CONTIG94	67445	68679	1.28	4.42E-02	transcription_start_site	-	67438	67438	-624	22014	AN5403
2904	CONTIG94	69980	70700	1	1.79E-01	transcription_start_site	-	70380	70380	40	22012	AN5403
2904	CONTIG94	69980	70700	1	1.79E-01	transcription_start_site	-	70517	70517	177	22011	AN5403
2904	CONTIG94	69980	70700	1	1.79E-01	transcription_start_site	-	70750	70750	410	22010	AN5403
2904	CONTIG94	69980	70700	1	1.79E-01	transcription_start_site	-	67785	67785	-2555	22013	AN5403
2904	CONTIG94	69980	70700	1	1.79E-01	transcription_start_site	-	67438	67438	-2902	22014	AN5403
2911	CONTIG94	71706	72650	1	1.93E-01	transcription_start_site	-	70750	70750	-1428	22010	AN5403
2911	CONTIG94	71706	72650	1	1.93E-01	transcription_start_site	-	70517	70517	-1661	22011	AN5403
2911	CONTIG94	71706	72650	1	1.93E-01	transcription_start_site	-	70380	70380	-1798	22012	AN5403
2911	CONTIG94	71706	72650	1	1.93E-01	transcription_start_site	-	67785	67785	-4393	22013	AN5403
2911	CONTIG94	71706	72650	1	1.93E-01	transcription_start_site	-	67438	67438	-4740	22014	AN5403
2249	CONTIG94	67445	68679	1.28	4.42E-02	transcription_start_site	+	72128	72128	-4066	22015	AN5404
2249	CONTIG94	67445	68679	1.28	4.42E-02	transcription_start_site	+	72322	72322	-4260	22016	AN5404
2249	CONTIG94	67445	68679	1.28	4.42E-02	transcription_start_site	+	72397	72397	-4335	22017	AN5404
2249	CONTIG94	67445	68679	1.28	4.42E-02	transcription_start_site	+	72567	72567	-4505	22018	AN5404
2249	CONTIG94	67445	68679	1.28	4.42E-02	transcription_start_site	+	72658	72658	-4596	22019	AN5404
2249	CONTIG94	67445	68679	1.28	4.42E-02	transcription_start_site	+	72826	72826	-4764	22020	AN5404
2249	CONTIG94	67445	68679	1.28	4.42E-02	transcription_start_site	+	73085	73085	-5023	22021	AN5404
2904	CONTIG94	69980	70700	1	1.79E-01	transcription_start_site	+	72128	72128	-1788	22015	AN5404

2904	CONTIG94	69980	70700	1	1.79E-01	transcription_start_site	+	72322	72322	-1982	22016 AN5404
2904	CONTIG94	69980	70700	1	1.79E-01	transcription_start_site	+	72397	72397	-2057	22017 AN5404
2904	CONTIG94	69980	70700	1	1.79E-01	transcription_start_site	+	72567	72567	-2227	22018 AN5404
2904	CONTIG94	69980	70700	1	1.79E-01	transcription_start_site	+	72658	72658	-2318	22019 AN5404
2904	CONTIG94	69980	70700	1	1.79E-01	transcription_start_site	+	72826	72826	-2486	22020 AN5404
2904	CONTIG94	69980	70700	1	1.79E-01	transcription_start_site	+	73085	73085	-2745	22021 AN5404
2911	CONTIG94	71706	72650	1	1.93E-01	transcription_start_site	+	72128	72128	50	22015 AN5404
2911	CONTIG94	71706	72650	1	1.93E-01	transcription_start_site	+	72322	72322	-144	22016 AN5404
2911	CONTIG94	71706	72650	1	1.93E-01	transcription_start_site	+	72397	72397	-219	22017 AN5404
2911	CONTIG94	71706	72650	1	1.93E-01	transcription_start_site	+	72567	72567	-389	22018 AN5404
2911	CONTIG94	71706	72650	1	1.93E-01	transcription_start_site	+	72658	72658	-480	22019 AN5404
2911	CONTIG94	71706	72650	1	1.93E-01	transcription_start_site	+	72826	72826	-648	22020 AN5404
2911	CONTIG94	71706	72650	1	1.93E-01	transcription_start_site	+	73085	73085	-907	22021 AN5404
2911	CONTIG94	71706	72650	1	1.93E-01	transcription_start_site	+	76974	76974	-4796	22022 AN5405
2911	CONTIG94	71706	72650	1	1.93E-01	transcription_start_site	+	77377	77377	-5199	22023 AN5405
2911	CONTIG94	71706	72650	1	1.93E-01	transcription_start_site	+	77539	77539	-5361	22024 AN5405
1822	CONTIG94	86108	86682	1.44	3.70E-02	transcription_start_site	-	85127	85127	-1268	22026 AN5406
1822	CONTIG94	86108	86682	1.44	3.70E-02	transcription_start_site	-	84989	84989	-1406	22027 AN5406
1822	CONTIG94	86108	86682	1.44	3.70E-02	transcription_start_site	-	84647	84647	-1748	22028 AN5406
1822	CONTIG94	86108	86682	1.44	3.70E-02	transcription_start_site	-	81614	81614	-4781	22029 AN5406
1822	CONTIG94	86108	86682	1.44	3.70E-02	transcription_start_site	+	86989	86989	-594	22030 AN5407
1822	CONTIG94	86108	86682	1.44	3.70E-02	transcription_start_site	+	87392	87392	-997	22031 AN5407
1822	CONTIG94	86108	86682	1.44	3.70E-02	transcription_start_site	+	88048	88048	-1653	22032 AN5407
1822	CONTIG94	86108	86682	1.44	3.70E-02	transcription_start_site	+	88449	88449	-2054	22033 AN5407
1822	CONTIG94	86108	86682	1.44	3.70E-02	transcription_start_site	+	91318	91318	-4923	22034 AN5408
1822	CONTIG94	86108	86682	1.44	3.70E-02	transcription_start_site	+	91588	91588	-5193	22035 AN5408
2609	CONTIG94	139052	139491	1.16	1.08E-01	transcription_start_site	-	135486	135486	-3785	22080 AN5425
2609	CONTIG94	139052	139491	1.16	1.08E-01	transcription_start_site	-	135390	135390	-3881	22081 AN5425
2609	CONTIG94	139052	139491	1.16	1.08E-01	transcription_start_site	-	135018	135018	-4253	22082 AN5425
2912	CONTIG94	136367	136641	1	1.93E-01	transcription_start_site	-	135486	135486	-1018	22080 AN5425
2912	CONTIG94	136367	136641	1	1.93E-01	transcription_start_site	-	135390	135390	-1114	22081 AN5425
2912	CONTIG94	136367	136641	1	1.93E-01	transcription_start_site	-	135018	135018	-1486	22082 AN5425
2609	CONTIG94	139052	139491	1.16	1.08E-01	transcription_start_site	-	137059	137059	-2212	22083 AN5426
2912	CONTIG94	136367	136641	1	1.93E-01	transcription_start_site	-	137059	137059	555	22083 AN5426
2609	CONTIG94	139052	139491	1.16	1.08E-01	transcription_start_site	-	138951	138951	-320	22088 AN5427
2609	CONTIG94	139052	139491	1.16	1.08E-01	transcription_start_site	-	139855	139855	583	22087 AN5427
1823	CONTIG94	149331	149750	1.44	3.70E-02	transcription_start_site	-	144880	144880	-4660	22089 AN5428
1823	CONTIG94	149331	149750	1.44	3.70E-02	transcription_start_site	-	144590	144590	-4950	22090 AN5428
1823	CONTIG94	149331	149750	1.44	3.70E-02	transcription_start_site	-	144367	144367	-5173	22091 AN5428
1823	CONTIG94	149331	149750	1.44	3.70E-02	transcription_start_site	-	150516	150516	975	22094 AN5430
1823	CONTIG94	149331	149750	1.44	3.70E-02	transcription_start_site	-	150658	150658	1117	22093 AN5430
1823	CONTIG94	149331	149750	1.44	3.70E-02	transcription_start_site	+	152944	152944	-3403	22095 AN5431
1823	CONTIG94	149331	149750	1.44	3.70E-02	transcription_start_site	+	153193	153193	-3652	22096 AN5431
1823	CONTIG94	149331	149750	1.44	3.70E-02	transcription_start_site	+	154344	154344	-4803	22097 AN5431
1823	CONTIG94	149331	149750	1.44	3.70E-02	transcription_start_site	+	154503	154503	-4962	22098 AN5431
2610	CONTIG94	171314	171750	1.16	1.08E-01	transcription_start_site	+	174432	174432	-2900	22119 AN5437
2610	CONTIG94	171314	171750	1.16	1.08E-01	transcription_start_site	+	174488	174488	-2956	22120 AN5437
2610	CONTIG94	171314	171750	1.16	1.08E-01	transcription_start_site	+	174736	174736	-3204	22121 AN5437
2610	CONTIG94	171314	171750	1.16	1.08E-01	transcription_start_site	+	174873	174873	-3341	22122 AN5437
2610	CONTIG94	171314	171750	1.16	1.08E-01	transcription_start_site	+	175268	175268	-3736	22123 AN5437
2610	CONTIG94	171314	171750	1.16	1.08E-01	transcription_start_site	+	175505	175505	-3973	22124 AN5437
1368	CONTIG94	180004	180580	1.65	1.57E-02	transcription_start_site	-	179279	179279	-1013	22125 AN5438
1368	CONTIG94	180004	180580	1.65	1.57E-02	transcription_start_site	-	178919	178919	-1373	22126 AN5438
1368	CONTIG94	180004	180580	1.65	1.57E-02	transcription_start_site	-	178576	178576	-1716	22127 AN5438
1368	CONTIG94	180004	180580	1.65	1.57E-02	transcription_start_site	+	179715	179715	577	22128 AN5439
1368	CONTIG94	180004	180580	1.65	1.57E-02	transcription_start_site	+	180621	180621	-329	22129 AN5440
1368	CONTIG94	180004	180580	1.65	1.57E-02	transcription_start_site	+	180794	180794	-502	22130 AN5440

1368	CONTIG94	180004	180580	1.65	1.57E-02	transcription_start_site	+	183708	183708	-3416	22131 AN5441
1368	CONTIG94	180004	180580	1.65	1.57E-02	transcription_start_site	+	183944	183944	-3652	22132 AN5441
1368	CONTIG94	180004	180580	1.65	1.57E-02	transcription_start_site	+	184228	184228	-3936	22133 AN5441
559	CONTIG94	194568	194977	2.37	6.10E-04	transcription_start_site	-	190371	190371	-4401	22137 AN5444
2372	CONTIG94	193438	193792	1.24	8.01E-02	transcription_start_site	-	190371	190371	-3244	22137 AN5444
559	CONTIG94	194568	194977	2.37	6.10E-04	transcription_start_site	-	193856	193856	-916	22138 AN5445
559	CONTIG94	194568	194977	2.37	6.10E-04	transcription_start_site	-	193453	193453	-1319	22139 AN5445
2372	CONTIG94	193438	193792	1.24	8.01E-02	transcription_start_site	-	193453	193453	-162	22139 AN5445
2372	CONTIG94	193438	193792	1.24	8.01E-02	transcription_start_site	-	193856	193856	241	22138 AN5445
2707	CONTIG94	197038	197392	1.12	1.25E-01	transcription_start_site	-	193856	193856	-3359	22138 AN5445
2707	CONTIG94	197038	197392	1.12	1.25E-01	transcription_start_site	-	193453	193453	-3762	22139 AN5445
559	CONTIG94	194568	194977	2.37	6.10E-04	transcription_start_site	+	196941	196941	-2168	22140 AN5446
559	CONTIG94	194568	194977	2.37	6.10E-04	transcription_start_site	+	197168	197168	-2395	22141 AN5446
559	CONTIG94	194568	194977	2.37	6.10E-04	transcription_start_site	+	197517	197517	-2744	22142 AN5446
2372	CONTIG94	193438	193792	1.24	8.01E-02	transcription_start_site	+	196941	196941	-3326	22140 AN5446
2372	CONTIG94	193438	193792	1.24	8.01E-02	transcription_start_site	+	197168	197168	-3553	22141 AN5446
2372	CONTIG94	193438	193792	1.24	8.01E-02	transcription_start_site	+	197517	197517	-3902	22142 AN5446
2707	CONTIG94	197038	197392	1.12	1.25E-01	transcription_start_site	+	197168	197168	47	22141 AN5446
2707	CONTIG94	197038	197392	1.12	1.25E-01	transcription_start_site	+	196941	196941	274	22140 AN5446
2707	CONTIG94	197038	197392	1.12	1.25E-01	transcription_start_site	+	197517	197517	-302	22142 AN5446
559	CONTIG94	194568	194977	2.37	6.10E-04	transcription_start_site	+	199831	199831	-5058	22143 AN5447
1070	CONTIG94	202353	202852	1.85	6.61E-03	transcription_start_site	+	201413	201413	1189	22146 AN5447
2707	CONTIG94	197038	197392	1.12	1.25E-01	transcription_start_site	+	199831	199831	-2616	22143 AN5447
2707	CONTIG94	197038	197392	1.12	1.25E-01	transcription_start_site	+	200116	200116	-2901	22144 AN5447
2707	CONTIG94	197038	197392	1.12	1.25E-01	transcription_start_site	+	200192	200192	-2977	22145 AN5447
2707	CONTIG94	197038	197392	1.12	1.25E-01	transcription_start_site	+	201413	201413	-4198	22146 AN5447
1070	CONTIG94	202353	202852	1.85	6.61E-03	transcription_start_site	+	201971	201971	631	22147 AN5448
1070	CONTIG94	202353	202852	1.85	6.61E-03	transcription_start_site	+	203272	203272	-669	22148 AN5448
2509	CONTIG94	203943	204217	1.2	9.24E-02	transcription_start_site	+	203272	203272	808	22148 AN5448
2707	CONTIG94	197038	197392	1.12	1.25E-01	transcription_start_site	+	201971	201971	-4756	22147 AN5448
342	CONTIG94	209265	209529	2.69	0.00E+00	transcription_start_site	-	208052	208052	-1345	22149 AN5449
342	CONTIG94	209265	209529	2.69	0.00E+00	transcription_start_site	-	207586	207586	-1811	22150 AN5449
1020	CONTIG94	206415	206979	1.89	1.46E-03	transcription_start_site	-	207586	207586	889	22150 AN5449
1448	CONTIG94	207830	208414	1.61	1.84E-02	transcription_start_site	-	208052	208052	-70	22149 AN5449
1448	CONTIG94	207830	208414	1.61	1.84E-02	transcription_start_site	-	207586	207586	-536	22150 AN5449
2782	CONTIG94	207080	207659	1.08	1.44E-01	transcription_start_site	-	207586	207586	216	22150 AN5449
2782	CONTIG94	207080	207659	1.08	1.44E-01	transcription_start_site	-	208052	208052	682	22149 AN5449
1121	CONTIG94	215420	215694	1.81	7.85E-03	transcription_start_site	-	212841	212841	-2716	22151 AN5450
1121	CONTIG94	215420	215694	1.81	7.85E-03	transcription_start_site	-	212698	212698	-2859	22152 AN5450
1121	CONTIG94	215420	215694	1.81	7.85E-03	transcription_start_site	-	211326	211326	-4231	22153 AN5450
1121	CONTIG94	215420	215694	1.81	7.85E-03	transcription_start_site	-	210818	210818	-4739	22154 AN5450
1121	CONTIG94	215420	215694	1.81	7.85E-03	transcription_start_site	-	210541	210541	-5016	22155 AN5450
1121	CONTIG94	215420	215694	1.81	7.85E-03	transcription_start_site	-	215347	215347	-210	22156 AN5451
1121	CONTIG94	215420	215694	1.81	7.85E-03	transcription_start_site	-	214821	214821	-736	22157 AN5451
1121	CONTIG94	215420	215694	1.81	7.85E-03	transcription_start_site	+	216008	216008	-451	22158 AN5452
1121	CONTIG94	215420	215694	1.81	7.85E-03	transcription_start_site	+	216425	216425	-868	22159 AN5452
1121	CONTIG94	215420	215694	1.81	7.85E-03	transcription_start_site	+	218693	218693	-3136	22160 AN5452
1121	CONTIG94	215420	215694	1.81	7.85E-03	transcription_start_site	+	220038	220038	-4481	22161 AN5452
855	CONTIG94	227630	227909	2.05	2.22E-03	transcription_start_site	+	227965	227965	-195	22169 AN5454
855	CONTIG94	227630	227909	2.05	2.22E-03	transcription_start_site	+	227321	227321	448	22168 AN5454
855	CONTIG94	227630	227909	2.05	2.22E-03	transcription_start_site	+	227170	227170	599	22167 AN5454
855	CONTIG94	227630	227909	2.05	2.22E-03	transcription_start_site	+	227043	227043	726	22166 AN5454
1071	CONTIG94	252692	253031	1.85	6.61E-03	transcription_start_site	-	249539	249539	-3322	22187 AN5460
1071	CONTIG94	252692	253031	1.85	6.61E-03	transcription_start_site	-	249394	249394	-3467	22188 AN5460
1071	CONTIG94	252692	253031	1.85	6.61E-03	transcription_start_site	-	249174	249174	-3687	22189 AN5460
1071	CONTIG94	252692	253031	1.85	6.61E-03	transcription_start_site	-	248835	248835	-4026	22190 AN5460
1071	CONTIG94	252692	253031	1.85	6.61E-03	transcription_start_site	-	248707	248707	-4154	22191 AN5460

1071	CONTIG94	252692	253031	1.85	6.61E-03	transcription_start_site	-	248613	248613	-4248	22192	AN5460
1071	CONTIG94	252692	253031	1.85	6.61E-03	transcription_start_site	-	248433	248433	-4428	22193	AN5460
1071	CONTIG94	252692	253031	1.85	6.61E-03	transcription_start_site	-	247998	247998	-4863	22194	AN5460
1071	CONTIG94	252692	253031	1.85	6.61E-03	transcription_start_site	-	247784	247784	-5077	22195	AN5460
1071	CONTIG94	252692	253031	1.85	6.61E-03	transcription_start_site	-	252715	252715	-146	22197	AN5461
1071	CONTIG94	252692	253031	1.85	6.61E-03	transcription_start_site	+	254216	254216	-1354	22198	AN5462
1071	CONTIG94	252692	253031	1.85	6.61E-03	transcription_start_site	+	254486	254486	-1624	22199	AN5462
1071	CONTIG94	252692	253031	1.85	6.61E-03	transcription_start_site	+	256660	256660	-3798	22200	AN5463
1071	CONTIG94	252692	253031	1.85	6.61E-03	transcription_start_site	+	256821	256821	-3959	22201	AN5463
1824	CONTIG94	314029	314693	1.44	3.70E-02	transcription_start_site	+	313488	313488	873	22255	AN5479
1824	CONTIG94	314029	314693	1.44	3.70E-02	transcription_start_site	-	315026	315026	665	22258	AN5480
1824	CONTIG94	314029	314693	1.44	3.70E-02	transcription_start_site	-	315215	315215	854	22257	AN5480
1824	CONTIG94	314029	314693	1.44	3.70E-02	transcription_start_site	-	315459	315459	1098	22256	AN5480
1916	CONTIG94	315604	315878	1.4	4.36E-02	transcription_start_site	-	315459	315459	-282	22256	AN5480
1916	CONTIG94	315604	315878	1.4	4.36E-02	transcription_start_site	-	315215	315215	-526	22257	AN5480
1916	CONTIG94	315604	315878	1.4	4.36E-02	transcription_start_site	-	315026	315026	-715	22258	AN5480
368	CONTIG94	354921	355870	2.65	0.00E+00	transcription_start_site	-	350844	350844	-4551	22282	AN5488
726	CONTIG94	351246	352410	2.17	0.00E+00	transcription_start_site	-	350844	350844	-984	22282	AN5488
726	CONTIG94	351246	352410	2.17	0.00E+00	transcription_start_site	-	348869	348869	-2959	22283	AN5488
726	CONTIG94	351246	352410	2.17	0.00E+00	transcription_start_site	-	348224	348224	-3604	22284	AN5488
726	CONTIG94	351246	352410	2.17	0.00E+00	transcription_start_site	-	347953	347953	-3875	22285	AN5488
726	CONTIG94	351246	352410	2.17	0.00E+00	transcription_start_site	-	347867	347867	-3961	22286	AN5488
726	CONTIG94	351246	352410	2.17	0.00E+00	transcription_start_site	-	346939	346939	-4889	22287	AN5488
726	CONTIG94	351246	352410	2.17	0.00E+00	transcription_start_site	-	346635	346635	-5193	22288	AN5488
368	CONTIG94	354921	355870	2.65	0.00E+00	transcription_start_site	+	355655	355655	-259	22295	AN5489
726	CONTIG94	351246	352410	2.17	0.00E+00	transcription_start_site	+	355655	355655	-3827	22295	AN5489
2708	CONTIG94	360244	360518	1.12	1.25E-01	transcription_start_site	-	359641	359641	-740	22296	AN5490
2708	CONTIG94	360244	360518	1.12	1.25E-01	transcription_start_site	-	359086	359086	-1295	22297	AN5490
2708	CONTIG94	360244	360518	1.12	1.25E-01	transcription_start_site	-	358952	358952	-1429	22298	AN5490
495	CONTIG94	369844	370553	2.45	0.00E+00	transcription_start_site	-	367310	367310	-2888	22299	AN5491
495	CONTIG94	369844	370553	2.45	0.00E+00	transcription_start_site	-	367065	367065	-3133	22300	AN5491
495	CONTIG94	369844	370553	2.45	0.00E+00	transcription_start_site	-	369698	369698	-500	22302	AN5492
495	CONTIG94	369844	370553	2.45	0.00E+00	transcription_start_site	-	369517	369517	-681	22303	AN5492
495	CONTIG94	369844	370553	2.45	0.00E+00	transcription_start_site	+	371333	371333	-1134	22304	AN5493
495	CONTIG94	369844	370553	2.45	0.00E+00	transcription_start_site	+	371719	371719	-1520	22305	AN5493
495	CONTIG94	369844	370553	2.45	0.00E+00	transcription_start_site	+	373031	373031	-2832	22306	AN5493
1615	CONTIG94	391430	391709	1.53	2.62E-02	transcription_start_site	+	390892	390892	677	22324	AN5500
1615	CONTIG94	391430	391709	1.53	2.62E-02	transcription_start_site	+	390797	390797	772	22323	AN5500
2913	CONTIG94	389270	389604	1	1.93E-01	transcription_start_site	+	390797	390797	-1360	22323	AN5500
2913	CONTIG94	389270	389604	1	1.93E-01	transcription_start_site	+	390892	390892	-1455	22324	AN5500
1615	CONTIG94	391430	391709	1.53	2.62E-02	transcription_start_site	+	392816	392816	-1246	22325	AN5501
1615	CONTIG94	391430	391709	1.53	2.62E-02	transcription_start_site	+	393892	393892	-2322	22326	AN5501
1615	CONTIG94	391430	391709	1.53	2.62E-02	transcription_start_site	+	394063	394063	-2493	22327	AN5501
1615	CONTIG94	391430	391709	1.53	2.62E-02	transcription_start_site	+	394139	394139	-2569	22328	AN5501
2606	CONTIG94	392570	393204	1.16	8.36E-02	transcription_start_site	+	392816	392816	71	22325	AN5501
2606	CONTIG94	392570	393204	1.16	8.36E-02	transcription_start_site	+	393892	393892	-1005	22326	AN5501
2606	CONTIG94	392570	393204	1.16	8.36E-02	transcription_start_site	+	394063	394063	-1176	22327	AN5501
2606	CONTIG94	392570	393204	1.16	8.36E-02	transcription_start_site	+	394139	394139	-1252	22328	AN5501
2913	CONTIG94	389270	389604	1	1.93E-01	transcription_start_site	+	392816	392816	-3379	22325	AN5501
2913	CONTIG94	389270	389604	1	1.93E-01	transcription_start_site	+	393892	393892	-4455	22326	AN5501
2913	CONTIG94	389270	389604	1	1.93E-01	transcription_start_site	+	394063	394063	-4626	22327	AN5501
2913	CONTIG94	389270	389604	1	1.93E-01	transcription_start_site	+	394139	394139	-4702	22328	AN5501
1615	CONTIG94	391430	391709	1.53	2.62E-02	transcription_start_site	+	395820	395820	-4250	22329	AN5502
1615	CONTIG94	391430	391709	1.53	2.62E-02	transcription_start_site	+	396540	396540	-4970	22330	AN5502
2606	CONTIG94	392570	393204	1.16	8.36E-02	transcription_start_site	+	395820	395820	-2933	22329	AN5502
2606	CONTIG94	392570	393204	1.16	8.36E-02	transcription_start_site	+	396540	396540	-3653	22330	AN5502
2606	CONTIG94	392570	393204	1.16	8.36E-02	transcription_start_site	+	396936	396936	-4049	22331	AN5502

2606	CONTIG94	392570	393204	1.16	8.36E-02	transcription_start_site	+	397245	397245	-4358	22332 AN5502
2606	CONTIG94	392570	393204	1.16	8.36E-02	transcription_start_site	+	397932	397932	-5045	22333 AN5503
2606	CONTIG94	392570	393204	1.16	8.36E-02	transcription_start_site	+	397973	397973	-5086	22334 AN5503
2606	CONTIG94	392570	393204	1.16	8.36E-02	transcription_start_site	+	398183	398183	-5296	22335 AN5503
2373	CONTIG94	422102	422766	1.24	8.01E-02	transcription_start_site	+	423794	423794	-1360	22361 AN5510
933	CONTIG94	442968	443467	1.97	3.61E-03	transcription_start_site	-	438576	438576	-4641	22371 AN5514
933	CONTIG94	442968	443467	1.97	3.61E-03	transcription_start_site	-	438216	438216	-5001	22372 AN5514
933	CONTIG94	442968	443467	1.97	3.61E-03	transcription_start_site	-	437998	437998	-5219	22373 AN5514
1516	CONTIG94	441678	441958	1.57	2.12E-02	transcription_start_site	-	438576	438576	-3242	22371 AN5514
1516	CONTIG94	441678	441958	1.57	2.12E-02	transcription_start_site	-	438216	438216	-3602	22372 AN5514
1516	CONTIG94	441678	441958	1.57	2.12E-02	transcription_start_site	-	437998	437998	-3820	22373 AN5514
1516	CONTIG94	441678	441958	1.57	2.12E-02	transcription_start_site	-	436944	436944	-4874	22374 AN5514
1516	CONTIG94	441678	441958	1.57	2.12E-02	transcription_start_site	-	436713	436713	-5105	22375 AN5514
2250	CONTIG94	440788	441657	1.28	5.05E-02	transcription_start_site	-	438576	438576	-2646	22371 AN5514
2250	CONTIG94	440788	441657	1.28	5.05E-02	transcription_start_site	-	438216	438216	-3006	22372 AN5514
2250	CONTIG94	440788	441657	1.28	5.05E-02	transcription_start_site	-	437998	437998	-3224	22373 AN5514
2250	CONTIG94	440788	441657	1.28	5.05E-02	transcription_start_site	-	436944	436944	-4278	22374 AN5514
2250	CONTIG94	440788	441657	1.28	5.05E-02	transcription_start_site	-	436713	436713	-4509	22375 AN5514
2250	CONTIG94	440788	441657	1.28	5.05E-02	transcription_start_site	-	436533	436533	-4689	22376 AN5514
2250	CONTIG94	440788	441657	1.28	5.05E-02	transcription_start_site	-	435977	435977	-5245	22377 AN5514
2783	CONTIG94	441993	442637	1.08	1.44E-01	transcription_start_site	-	438576	438576	-3739	22371 AN5514
2783	CONTIG94	441993	442637	1.08	1.44E-01	transcription_start_site	-	438216	438216	-4099	22372 AN5514
2783	CONTIG94	441993	442637	1.08	1.44E-01	transcription_start_site	-	437998	437998	-4317	22373 AN5514
1182	CONTIG94	451808	452082	1.77	8.81E-03	transcription_start_site	-	450434	450434	-1511	22383 AN5516
1182	CONTIG94	451808	452082	1.77	8.81E-03	transcription_start_site	-	450163	450163	-1782	22384 AN5516
1182	CONTIG94	451808	452082	1.77	8.81E-03	transcription_start_site	-	450084	450084	-1861	22385 AN5516
1182	CONTIG94	451808	452082	1.77	8.81E-03	transcription_start_site	-	449822	449822	-2123	22386 AN5516
1182	CONTIG94	451808	452082	1.77	8.81E-03	transcription_start_site	-	449494	449494	-2451	22387 AN5516
1182	CONTIG94	451808	452082	1.77	8.81E-03	transcription_start_site	-	449287	449287	-2658	22388 AN5516
1182	CONTIG94	451808	452082	1.77	8.81E-03	transcription_start_site	-	449054	449054	-2891	22389 AN5516
1182	CONTIG94	451808	452082	1.77	8.81E-03	transcription_start_site	-	448931	448931	-3014	22390 AN5516
1182	CONTIG94	451808	452082	1.77	8.81E-03	transcription_start_site	-	448654	448654	-3291	22391 AN5516
1182	CONTIG94	451808	452082	1.77	8.81E-03	transcription_start_site	-	448375	448375	-3570	22392 AN5516
1182	CONTIG94	451808	452082	1.77	8.81E-03	transcription_start_site	-	448134	448134	-3811	22393 AN5516
1182	CONTIG94	451808	452082	1.77	8.81E-03	transcription_start_site	+	452495	452495	-550	22394 AN5517
1182	CONTIG94	451808	452082	1.77	8.81E-03	transcription_start_site	+	452842	452842	-897	22395 AN5517
1182	CONTIG94	451808	452082	1.77	8.81E-03	transcription_start_site	+	455605	455605	-3660	22396 AN5517
2905	CONTIG94	459088	459504	1	1.79E-01	transcription_start_site	-	457537	457537	-1759	22397 AN5518
2905	CONTIG94	459088	459504	1	1.79E-01	transcription_start_site	-	456921	456921	-2375	22398 AN5518
2905	CONTIG94	459088	459504	1	1.79E-01	transcription_start_site	+	459357	459357	-61	22403 AN5520
2905	CONTIG94	459088	459504	1	1.79E-01	transcription_start_site	+	459219	459219	77	22402 AN5520
2905	CONTIG94	459088	459504	1	1.79E-01	transcription_start_site	+	458948	458948	348	22401 AN5520
2905	CONTIG94	459088	459504	1	1.79E-01	transcription_start_site	+	459923	459923	-627	22404 AN5520
1183	CONTIG94	468827	469116	1.77	8.81E-03	transcription_start_site	-	463929	463929	-5042	22405 AN5521
1183	CONTIG94	468827	469116	1.77	8.81E-03	transcription_start_site	-	465470	465470	-3501	22409 AN5522
1183	CONTIG94	468827	469116	1.77	8.81E-03	transcription_start_site	-	468355	468355	-616	22410 AN5523
1183	CONTIG94	468827	469116	1.77	8.81E-03	transcription_start_site	-	467988	467988	-983	22411 AN5523
1183	CONTIG94	468827	469116	1.77	8.81E-03	transcription_start_site	-	467560	467560	-1411	22412 AN5523
1183	CONTIG94	468827	469116	1.77	8.81E-03	transcription_start_site	-	467141	467141	-1830	22413 AN5523
1183	CONTIG94	468827	469116	1.77	8.81E-03	transcription_start_site	-	466746	466746	-2225	22414 AN5523
2038	CONTIG94	471077	471366	1.36	5.22E-02	transcription_start_site	-	468355	468355	-2866	22410 AN5523
2038	CONTIG94	471077	471366	1.36	5.22E-02	transcription_start_site	-	467988	467988	-3233	22411 AN5523
2038	CONTIG94	471077	471366	1.36	5.22E-02	transcription_start_site	-	467560	467560	-3661	22412 AN5523
2038	CONTIG94	471077	471366	1.36	5.22E-02	transcription_start_site	-	467141	467141	-4080	22413 AN5523
2038	CONTIG94	471077	471366	1.36	5.22E-02	transcription_start_site	-	466746	466746	-4475	22414 AN5523
972	CONTIG94	474392	474726	1.93	4.61E-03	transcription_start_site	-	470334	470334	-4225	22415 AN5524
972	CONTIG94	474392	474726	1.93	4.61E-03	transcription_start_site	-	470135	470135	-4424	22416 AN5524

972	CONTIG94	474392	474726	1.93	4.61E-03	transcription_start_site	-	470066	470066	-4493	22417	AN5524
972	CONTIG94	474392	474726	1.93	4.61E-03	transcription_start_site	-	469643	469643	-4916	22418	AN5524
1183	CONTIG94	468827	469116	1.77	8.81E-03	transcription_start_site	-	469643	469643	671	22418	AN5524
1183	CONTIG94	468827	469116	1.77	8.81E-03	transcription_start_site	-	470066	470066	1094	22417	AN5524
2038	CONTIG94	471077	471366	1.36	5.22E-02	transcription_start_site	-	470334	470334	-887	22415	AN5524
2038	CONTIG94	471077	471366	1.36	5.22E-02	transcription_start_site	-	470135	470135	-1086	22416	AN5524
2038	CONTIG94	471077	471366	1.36	5.22E-02	transcription_start_site	-	470066	470066	-1155	22417	AN5524
2038	CONTIG94	471077	471366	1.36	5.22E-02	transcription_start_site	-	469643	469643	-1578	22418	AN5524
972	CONTIG94	474392	474726	1.93	4.61E-03	transcription_start_site	-	473810	473810	-749	22419	AN5525
972	CONTIG94	474392	474726	1.93	4.61E-03	transcription_start_site	-	473646	473646	-913	22420	AN5525
972	CONTIG94	474392	474726	1.93	4.61E-03	transcription_start_site	-	473554	473554	-1005	22421	AN5525
972	CONTIG94	474392	474726	1.93	4.61E-03	transcription_start_site	-	473385	473385	-1174	22422	AN5525
972	CONTIG94	474392	474726	1.93	4.61E-03	transcription_start_site	-	473275	473275	-1284	22423	AN5525
972	CONTIG94	474392	474726	1.93	4.61E-03	transcription_start_site	-	472612	472612	-1947	22424	AN5525
972	CONTIG94	474392	474726	1.93	4.61E-03	transcription_start_site	-	471207	471207	-3352	22425	AN5525
2038	CONTIG94	471077	471366	1.36	5.22E-02	transcription_start_site	-	471207	471207	-14	22425	AN5525
972	CONTIG94	474392	474726	1.93	4.61E-03	transcription_start_site	+	475023	475023	-464	22426	AN5526
972	CONTIG94	474392	474726	1.93	4.61E-03	transcription_start_site	+	475129	475129	-570	22427	AN5526
972	CONTIG94	474392	474726	1.93	4.61E-03	transcription_start_site	+	475377	475377	-818	22428	AN5526
2038	CONTIG94	471077	471366	1.36	5.22E-02	transcription_start_site	+	475023	475023	-3801	22426	AN5526
2038	CONTIG94	471077	471366	1.36	5.22E-02	transcription_start_site	+	475129	475129	-3907	22427	AN5526
2038	CONTIG94	471077	471366	1.36	5.22E-02	transcription_start_site	+	475377	475377	-4155	22428	AN5526
810	CONTIG94	491253	491542	2.09	1.80E-03	transcription_start_site	-	488710	488710	-2687	22429	AN5528
810	CONTIG94	491253	491542	2.09	1.80E-03	transcription_start_site	-	488288	488288	-3109	22430	AN5528
810	CONTIG94	491253	491542	2.09	1.80E-03	transcription_start_site	-	488171	488171	-3226	22431	AN5528
810	CONTIG94	491253	491542	2.09	1.80E-03	transcription_start_site	-	488003	488003	-3394	22432	AN5528
810	CONTIG94	491253	491542	2.09	1.80E-03	transcription_start_site	-	487682	487682	-3715	22433	AN5528
810	CONTIG94	491253	491542	2.09	1.80E-03	transcription_start_site	-	486564	486564	-4833	22434	AN5528
810	CONTIG94	491253	491542	2.09	1.80E-03	transcription_start_site	-	486284	486284	-5113	22435	AN5528
810	CONTIG94	491253	491542	2.09	1.80E-03	transcription_start_site	+	491499	491499	-101	22436	AN5529
810	CONTIG94	491253	491542	2.09	1.80E-03	transcription_start_site	+	492042	492042	-644	22437	AN5529
810	CONTIG94	491253	491542	2.09	1.80E-03	transcription_start_site	+	492608	492608	-1210	22438	AN5529
810	CONTIG94	491253	491542	2.09	1.80E-03	transcription_start_site	+	493135	493135	-1737	22439	AN5529
810	CONTIG94	491253	491542	2.09	1.80E-03	transcription_start_site	+	495001	495001	-3603	22442	AN5531
810	CONTIG94	491253	491542	2.09	1.80E-03	transcription_start_site	+	495274	495274	-3876	22443	AN5531
810	CONTIG94	491253	491542	2.09	1.80E-03	transcription_start_site	+	495771	495771	-4373	22444	AN5531
810	CONTIG94	491253	491542	2.09	1.80E-03	transcription_start_site	+	495908	495908	-4510	22445	AN5531
1808	CONTIG95	12901	14525	1.45	5.02E-02	transcription_start_site	-	8433	8433	-5280	22468	AN5542
1808	CONTIG95	12901	14525	1.45	5.02E-02	transcription_start_site	-	7989	7989	-5724	22469	AN5542
1808	CONTIG95	12901	14525	1.45	5.02E-02	transcription_start_site	-	10696	10696	-3017	22475	AN5544
456	CONTIG95	18686	19325	2.51	1.80E-03	transcription_start_site	-	16373	16373	-2632	22477	AN5546
456	CONTIG95	18686	19325	2.51	1.80E-03	transcription_start_site	-	15005	15005	-4000	22478	AN5546
456	CONTIG95	18686	19325	2.51	1.80E-03	transcription_start_site	-	14724	14724	-4281	22479	AN5546
1808	CONTIG95	12901	14525	1.45	5.02E-02	transcription_start_site	-	14724	14724	1011	22479	AN5546
1808	CONTIG95	12901	14525	1.45	5.02E-02	transcription_start_site	-	15005	15005	1292	22478	AN5546
456	CONTIG95	18686	19325	2.51	1.80E-03	transcription_start_site	-	18630	18630	-375	22480	AN5547
456	CONTIG95	18686	19325	2.51	1.80E-03	transcription_start_site	-	18349	18349	-656	22481	AN5547
456	CONTIG95	18686	19325	2.51	1.80E-03	transcription_start_site	-	18190	18190	-815	22482	AN5547
456	CONTIG95	18686	19325	2.51	1.80E-03	transcription_start_site	-	18011	18011	-994	22483	AN5547
456	CONTIG95	18686	19325	2.51	1.80E-03	transcription_start_site	-	17377	17377	-1628	22484	AN5547
2173	CONTIG95	40818	41152	1.31	1.44E-01	transcription_start_site	-	40907	40907	-78	22496	AN5553
2173	CONTIG95	40818	41152	1.31	1.44E-01	transcription_start_site	-	40270	40270	-715	22497	AN5553
2173	CONTIG95	40818	41152	1.31	1.44E-01	transcription_start_site	-	39612	39612	-1373	22498	AN5553
83	CONTIG95	72246	72505	3.29	0.00E+00	transcription_start_site	-	71963	71963	-412	22516	AN5563
83	CONTIG95	72246	72505	3.29	0.00E+00	transcription_start_site	-	71497	71497	-878	22517	AN5563
335	CONTIG95	74866	75290	2.71	9.51E-04	transcription_start_site	-	71963	71963	-3115	22516	AN5563
335	CONTIG95	74866	75290	2.71	9.51E-04	transcription_start_site	-	71497	71497	-3581	22517	AN5563

1380	CONTIG95	70066	71600	1.64	3.08E-02	transcription_start_site	-	71497	71497	664	22517 AN5563
1380	CONTIG95	70066	71600	1.64	3.08E-02	transcription_start_site	-	71963	71963	1130	22516 AN5563
1928	CONTIG95	73956	74680	1.4	9.26E-02	transcription_start_site	-	71963	71963	-2355	22516 AN5563
1928	CONTIG95	73956	74680	1.4	9.26E-02	transcription_start_site	-	71497	71497	-2821	22517 AN5563
83	CONTIG95	72246	72505	3.29	0.00E+00	transcription_start_site	+	73248	73248	-872	22518 AN5564
1380	CONTIG95	70066	71600	1.64	3.08E-02	transcription_start_site	+	73248	73248	-2415	22518 AN5564
1928	CONTIG95	73956	74680	1.4	9.26E-02	transcription_start_site	+	73248	73248	1070	22518 AN5564
83	CONTIG95	72246	72505	3.29	0.00E+00	transcription_start_site	+	76227	76227	-3851	22519 AN5565
83	CONTIG95	72246	72505	3.29	0.00E+00	transcription_start_site	+	76329	76329	-3953	22520 AN5565
335	CONTIG95	74866	75290	2.71	9.51E-04	transcription_start_site	+	76227	76227	-1149	22519 AN5565
335	CONTIG95	74866	75290	2.71	9.51E-04	transcription_start_site	+	76329	76329	-1251	22520 AN5565
1380	CONTIG95	70066	71600	1.64	3.08E-02	transcription_start_site	+	76227	76227	-5394	22519 AN5565
1380	CONTIG95	70066	71600	1.64	3.08E-02	transcription_start_site	+	76329	76329	-5496	22520 AN5565
1928	CONTIG95	73956	74680	1.4	9.26E-02	transcription_start_site	+	76227	76227	-1909	22519 AN5565
1928	CONTIG95	73956	74680	1.4	9.26E-02	transcription_start_site	+	76329	76329	-2011	22520 AN5565
335	CONTIG95	74866	75290	2.71	9.51E-04	transcription_start_site	+	79641	79641	-4563	22521 AN5566
335	CONTIG95	74866	75290	2.71	9.51E-04	transcription_start_site	+	79758	79758	-4680	22522 AN5566
1928	CONTIG95	73956	74680	1.4	9.26E-02	transcription_start_site	+	79641	79641	-5323	22521 AN5566
299	CONTIG95	85518	86109	2.76	0.00E+00	transcription_start_site	+	87440	87440	-1626	22527 AN5568
299	CONTIG95	85518	86109	2.76	0.00E+00	transcription_start_site	+	87739	87739	-1925	22528 AN5568
299	CONTIG95	85518	86109	2.76	0.00E+00	transcription_start_site	+	88115	88115	-2301	22529 AN5568
299	CONTIG95	85518	86109	2.76	0.00E+00	transcription_start_site	+	90143	90143	-4329	22530 AN5569
299	CONTIG95	85518	86109	2.76	0.00E+00	transcription_start_site	+	90337	90337	-4523	22531 AN5569
819	CONTIG96	7971	8320	2.08	2.12E-02	transcription_start_site	-	4471	4471	-3674	22545 AN5573
942	CONTIG96	4731	5155	1.97	3.08E-02	transcription_start_site	-	4471	4471	-472	22545 AN5573
819	CONTIG96	7971	8320	2.08	2.12E-02	transcription_start_site	+	7329	7329	816	22549 AN5574
819	CONTIG96	7971	8320	2.08	2.12E-02	transcription_start_site	+	7004	7004	1141	22548 AN5574
942	CONTIG96	4731	5155	1.97	3.08E-02	transcription_start_site	+	6566	6566	-1623	22546 AN5574
942	CONTIG96	4731	5155	1.97	3.08E-02	transcription_start_site	+	6674	6674	-1731	22547 AN5574
942	CONTIG96	4731	5155	1.97	3.08E-02	transcription_start_site	+	7004	7004	-2061	22548 AN5574
942	CONTIG96	4731	5155	1.97	3.08E-02	transcription_start_site	+	7329	7329	-2386	22549 AN5574
819	CONTIG96	7971	8320	2.08	2.12E-02	transcription_start_site	+	9581	9581	-1435	22550 AN5575
819	CONTIG96	7971	8320	2.08	2.12E-02	transcription_start_site	+	9667	9667	-1521	22551 AN5575
819	CONTIG96	7971	8320	2.08	2.12E-02	transcription_start_site	+	9820	9820	-1674	22552 AN5575
942	CONTIG96	4731	5155	1.97	3.08E-02	transcription_start_site	+	9581	9581	-4638	22550 AN5575
942	CONTIG96	4731	5155	1.97	3.08E-02	transcription_start_site	+	9667	9667	-4724	22551 AN5575
942	CONTIG96	4731	5155	1.97	3.08E-02	transcription_start_site	+	9820	9820	-4877	22552 AN5575
713	CONTIG96	14420	14684	2.19	1.57E-02	transcription_start_site	-	14903	14903	351	22554 AN5576
713	CONTIG96	14420	14684	2.19	1.57E-02	transcription_start_site	-	15257	15257	705	22553 AN5576
938	CONTIG96	15245	15804	1.97	1.20E-02	transcription_start_site	-	15257	15257	-267	22553 AN5576
938	CONTIG96	15245	15804	1.97	1.20E-02	transcription_start_site	-	14903	14903	-621	22554 AN5576
938	CONTIG96	15245	15804	1.97	1.20E-02	transcription_start_site	-	15857	15857	332	22558 AN5577
938	CONTIG96	15245	15804	1.97	1.20E-02	transcription_start_site	-	15976	15976	451	22557 AN5577
938	CONTIG96	15245	15804	1.97	1.20E-02	transcription_start_site	-	16447	16447	922	22556 AN5577
938	CONTIG96	15245	15804	1.97	1.20E-02	transcription_start_site	-	16620	16620	1095	22555 AN5577
713	CONTIG96	14420	14684	2.19	1.57E-02	transcription_start_site	+	16871	16871	-2319	22559 AN5578
938	CONTIG96	15245	15804	1.97	1.20E-02	transcription_start_site	+	16871	16871	-1346	22559 AN5578
943	CONTIG96	11710	11979	1.97	3.08E-02	transcription_start_site	+	16871	16871	-5026	22559 AN5578
768	CONTIG96	32255	33439	2.13	1.84E-02	transcription_start_site	-	29618	29618	-3229	22560 AN5579
874	CONTIG96	30315	30729	2.03	2.62E-02	transcription_start_site	-	29618	29618	-904	22560 AN5579
768	CONTIG96	32255	33439	2.13	1.84E-02	transcription_start_site	+	31338	31338	1509	22567 AN5580
874	CONTIG96	30315	30729	2.03	2.62E-02	transcription_start_site	+	30598	30598	-76	22564 AN5580
874	CONTIG96	30315	30729	2.03	2.62E-02	transcription_start_site	+	30896	30896	-374	22565 AN5580
874	CONTIG96	30315	30729	2.03	2.62E-02	transcription_start_site	+	31044	31044	-522	22566 AN5580
874	CONTIG96	30315	30729	2.03	2.62E-02	transcription_start_site	+	31338	31338	-816	22567 AN5580
768	CONTIG96	32255	33439	2.13	1.84E-02	transcription_start_site	+	32961	32961	-114	22569 AN5581
768	CONTIG96	32255	33439	2.13	1.84E-02	transcription_start_site	+	32473	32473	374	22568 AN5581

874	CONTIG96	30315	30729	2.03	2.62E-02	transcription_start_site	+	32473	32473	-1951	22568	AN5581
874	CONTIG96	30315	30729	2.03	2.62E-02	transcription_start_site	+	32961	32961	-2439	22569	AN5581
330	CONTIG96	36621	37030	2.72	2.22E-03	transcription_start_site	+	36974	36974	-148	22574	AN5582
330	CONTIG96	36621	37030	2.72	2.22E-03	transcription_start_site	+	36561	36561	264	22573	AN5582
330	CONTIG96	36621	37030	2.72	2.22E-03	transcription_start_site	+	36481	36481	344	22572	AN5582
330	CONTIG96	36621	37030	2.72	2.22E-03	transcription_start_site	+	35951	35951	874	22571	AN5582
330	CONTIG96	36621	37030	2.72	2.22E-03	transcription_start_site	+	35624	35624	1201	22570	AN5582
768	CONTIG96	32255	33439	2.13	1.84E-02	transcription_start_site	+	35624	35624	-2777	22570	AN5582
768	CONTIG96	32255	33439	2.13	1.84E-02	transcription_start_site	+	35951	35951	-3104	22571	AN5582
768	CONTIG96	32255	33439	2.13	1.84E-02	transcription_start_site	+	36481	36481	-3634	22572	AN5582
768	CONTIG96	32255	33439	2.13	1.84E-02	transcription_start_site	+	36561	36561	-3714	22573	AN5582
768	CONTIG96	32255	33439	2.13	1.84E-02	transcription_start_site	+	36974	36974	-4127	22574	AN5582
874	CONTIG96	30315	30729	2.03	2.62E-02	transcription_start_site	+	35624	35624	-5102	22570	AN5582
358	CONTIG96	42313	42657	2.67	3.03E-03	transcription_start_site	-	41205	41205	-1280	22575	AN5583
358	CONTIG96	42313	42657	2.67	3.03E-03	transcription_start_site	-	41097	41097	-1388	22576	AN5583
358	CONTIG96	42313	42657	2.67	3.03E-03	transcription_start_site	-	40850	40850	-1635	22577	AN5583
358	CONTIG96	42313	42657	2.67	3.03E-03	transcription_start_site	-	40719	40719	-1766	22578	AN5583
820	CONTIG96	39906	40250	2.08	2.12E-02	transcription_start_site	-	40719	40719	641	22578	AN5583
820	CONTIG96	39906	40250	2.08	2.12E-02	transcription_start_site	-	40850	40850	772	22577	AN5583
820	CONTIG96	39906	40250	2.08	2.12E-02	transcription_start_site	-	41097	41097	1019	22576	AN5583
820	CONTIG96	39906	40250	2.08	2.12E-02	transcription_start_site	-	41205	41205	1127	22575	AN5583
1277	CONTIG96	41271	42284	1.71	3.41E-02	transcription_start_site	-	41205	41205	-572	22575	AN5583
1277	CONTIG96	41271	42284	1.71	3.41E-02	transcription_start_site	-	41097	41097	-680	22576	AN5583
1277	CONTIG96	41271	42284	1.71	3.41E-02	transcription_start_site	-	40850	40850	-927	22577	AN5583
1277	CONTIG96	41271	42284	1.71	3.41E-02	transcription_start_site	-	40719	40719	-1058	22578	AN5583
1485	CONTIG96	43221	43475	1.6	9.24E-02	transcription_start_site	-	41205	41205	-2143	22575	AN5583
1485	CONTIG96	43221	43475	1.6	9.24E-02	transcription_start_site	-	41097	41097	-2251	22576	AN5583
1485	CONTIG96	43221	43475	1.6	9.24E-02	transcription_start_site	-	40850	40850	-2498	22577	AN5583
1485	CONTIG96	43221	43475	1.6	9.24E-02	transcription_start_site	-	40719	40719	-2629	22578	AN5583
426	CONTIG96	59345	59619	2.56	4.61E-03	transcription_start_site	-	54698	54698	-4784	22590	AN5587
426	CONTIG96	59345	59619	2.56	4.61E-03	transcription_start_site	+	59878	59878	-396	22596	AN5589
426	CONTIG96	59345	59619	2.56	4.61E-03	transcription_start_site	+	60218	60218	-736	22597	AN5589
426	CONTIG96	59345	59619	2.56	4.61E-03	transcription_start_site	+	61261	61261	-1779	22598	AN5589
426	CONTIG96	59345	59619	2.56	4.61E-03	transcription_start_site	+	61490	61490	-2008	22599	AN5589
1965	CONTIG96	63320	63669	1.39	1.50E-01	transcription_start_site	-	64024	64024	529	22600	AN5590
1845	CONTIG96	74195	74459	1.44	1.44E-01	transcription_start_site	-	70070	70070	-4257	22604	AN5592
527	CONTIG97	9691	10495	2.41	1.22E-03	transcription_start_site	-	6427	6427	-3666	22639	AN5603
527	CONTIG97	9691	10495	2.41	1.22E-03	transcription_start_site	-	6334	6334	-3759	22640	AN5603
527	CONTIG97	9691	10495	2.41	1.22E-03	transcription_start_site	-	6281	6281	-3812	22641	AN5603
527	CONTIG97	9691	10495	2.41	1.22E-03	transcription_start_site	-	9467	9467	-626	22642	AN5604
527	CONTIG97	9691	10495	2.41	1.22E-03	transcription_start_site	-	9282	9282	-811	22643	AN5604
2450	CONTIG98	10060	10944	1.22	1.53E-01	transcription_start_site	+	10826	10826	-324	22657	AN5610
2450	CONTIG98	10060	10944	1.22	1.53E-01	transcription_start_site	+	11218	11218	-716	22658	AN5610
2450	CONTIG98	10060	10944	1.22	1.53E-01	transcription_start_site	+	12869	12869	-2367	22659	AN5611
2450	CONTIG98	10060	10944	1.22	1.53E-01	transcription_start_site	+	13235	13235	-2733	22660	AN5611
2455	CONTIG98	22580	22854	1.22	1.93E-01	transcription_start_site	+	26209	26209	-3492	22667	AN5615
2455	CONTIG98	22580	22854	1.22	1.93E-01	transcription_start_site	+	26750	26750	-4033	22668	AN5615
2455	CONTIG98	22580	22854	1.22	1.93E-01	transcription_start_site	+	27638	27638	-4921	22669	AN5615
2455	CONTIG98	22580	22854	1.22	1.93E-01	transcription_start_site	+	27834	27834	-5117	22670	AN5615
1082	CONTIG98	46221	46475	1.85	2.62E-02	transcription_start_site	-	44977	44977	-1371	22685	AN5621
2456	CONTIG98	50701	51210	1.22	1.93E-01	transcription_start_site	-	47646	47646	-3309	22686	AN5622
1082	CONTIG98	46221	46475	1.85	2.62E-02	transcription_start_site	+	48275	48275	-1927	22687	AN5623
5	CONTIG98	59417	59837	3.85	0.00E+00	transcription_start_site	-	54423	54423	-5204	22688	AN5624
1467	CONTIG98	56183	56462	1.61	5.88E-02	transcription_start_site	-	54423	54423	-1899	22688	AN5624
1467	CONTIG98	56183	56462	1.61	5.88E-02	transcription_start_site	-	54061	54061	-2261	22689	AN5624
1467	CONTIG98	56183	56462	1.61	5.88E-02	transcription_start_site	-	53971	53971	-2351	22690	AN5624
1467	CONTIG98	56183	56462	1.61	5.88E-02	transcription_start_site	-	53545	53545	-2777	22691	AN5624

1467	CONTIG98	56183	56462	1.61	5.88E-02	transcription_start_site	-	52011	52011	-4311	22692	AN5624
1677	CONTIG98	54688	55117	1.51	8.01E-02	transcription_start_site	-	54423	54423	-479	22688	AN5624
1677	CONTIG98	54688	55117	1.51	8.01E-02	transcription_start_site	-	54061	54061	-841	22689	AN5624
1677	CONTIG98	54688	55117	1.51	8.01E-02	transcription_start_site	-	53971	53971	-931	22690	AN5624
1677	CONTIG98	54688	55117	1.51	8.01E-02	transcription_start_site	-	53545	53545	-1357	22691	AN5624
1677	CONTIG98	54688	55117	1.51	8.01E-02	transcription_start_site	-	52011	52011	-2891	22692	AN5624
2456	CONTIG98	50701	51210	1.22	1.93E-01	transcription_start_site	-	52011	52011	1055	22692	AN5624
1467	CONTIG98	56183	56462	1.61	5.88E-02	transcription_start_site	+	56288	56288	34	22694	AN5625
1467	CONTIG98	56183	56462	1.61	5.88E-02	transcription_start_site	+	56052	56052	270	22693	AN5625
1677	CONTIG98	54688	55117	1.51	8.01E-02	transcription_start_site	+	56052	56052	-1149	22693	AN5625
1677	CONTIG98	54688	55117	1.51	8.01E-02	transcription_start_site	+	56288	56288	-1385	22694	AN5625
2456	CONTIG98	50701	51210	1.22	1.93E-01	transcription_start_site	+	56052	56052	-5096	22693	AN5625
5	CONTIG98	59417	59837	3.85	0.00E+00	transcription_start_site	+	60217	60217	-590	22695	AN5626
5	CONTIG98	59417	59837	3.85	0.00E+00	transcription_start_site	+	60320	60320	-693	22696	AN5626
5	CONTIG98	59417	59837	3.85	0.00E+00	transcription_start_site	+	61627	61627	-2000	22697	AN5626
5	CONTIG98	59417	59837	3.85	0.00E+00	transcription_start_site	+	61944	61944	-2317	22698	AN5626
5	CONTIG98	59417	59837	3.85	0.00E+00	transcription_start_site	+	62123	62123	-2496	22699	AN5626
5	CONTIG98	59417	59837	3.85	0.00E+00	transcription_start_site	+	62452	62452	-2825	22700	AN5626
1137	CONTIG98	62327	63291	1.8	1.81E-02	transcription_start_site	+	62452	62452	357	22700	AN5626
1137	CONTIG98	62327	63291	1.8	1.81E-02	transcription_start_site	+	62123	62123	686	22699	AN5626
1137	CONTIG98	62327	63291	1.8	1.81E-02	transcription_start_site	+	61944	61944	865	22698	AN5626
1137	CONTIG98	62327	63291	1.8	1.81E-02	transcription_start_site	+	61627	61627	1182	22697	AN5626
1467	CONTIG98	56183	56462	1.61	5.88E-02	transcription_start_site	+	60217	60217	-3894	22695	AN5626
1467	CONTIG98	56183	56462	1.61	5.88E-02	transcription_start_site	+	60320	60320	-3997	22696	AN5626
5	CONTIG98	59417	59837	3.85	0.00E+00	transcription_start_site	+	63735	63735	-4108	22701	AN5627
5	CONTIG98	59417	59837	3.85	0.00E+00	transcription_start_site	+	64101	64101	-4474	22702	AN5627
5	CONTIG98	59417	59837	3.85	0.00E+00	transcription_start_site	+	64490	64490	-4863	22703	AN5627
1137	CONTIG98	62327	63291	1.8	1.81E-02	transcription_start_site	+	63735	63735	-926	22701	AN5627
1137	CONTIG98	62327	63291	1.8	1.81E-02	transcription_start_site	+	64101	64101	-1292	22702	AN5627
1137	CONTIG98	62327	63291	1.8	1.81E-02	transcription_start_site	+	64490	64490	-1681	22703	AN5627
1137	CONTIG98	62327	63291	1.8	1.81E-02	transcription_start_site	+	68233	68233	-5424	22704	AN5627
2295	CONTIG98	73652	74606	1.27	1.50E-01	transcription_start_site	-	74211	74211	82	22710	AN5629
2295	CONTIG98	73652	74606	1.27	1.50E-01	transcription_start_site	-	74345	74345	216	22709	AN5629
2295	CONTIG98	73652	74606	1.27	1.50E-01	transcription_start_site	-	74670	74670	541	22708	AN5629
2295	CONTIG98	73652	74606	1.27	1.50E-01	transcription_start_site	+	74938	74938	-809	22711	AN5630
2295	CONTIG98	73652	74606	1.27	1.50E-01	transcription_start_site	+	75009	75009	-880	22712	AN5630
2295	CONTIG98	73652	74606	1.27	1.50E-01	transcription_start_site	+	75605	75605	-1476	22713	AN5630
2295	CONTIG98	73652	74606	1.27	1.50E-01	transcription_start_site	+	75915	75915	-1786	22714	AN5630
2457	CONTIG98	86252	86531	1.22	1.93E-01	transcription_start_site	-	81337	81337	-5054	22715	AN5631
2457	CONTIG98	86252	86531	1.22	1.93E-01	transcription_start_site	+	86321	86321	70	22722	AN5633
2457	CONTIG98	86252	86531	1.22	1.93E-01	transcription_start_site	+	87017	87017	-625	22723	AN5633
2457	CONTIG98	86252	86531	1.22	1.93E-01	transcription_start_site	+	87335	87335	-943	22724	AN5633
233	CONTIG98	90757	91267	2.88	6.10E-04	transcription_start_site	-	90541	90541	-471	22725	AN5634
233	CONTIG98	90757	91267	2.88	6.10E-04	transcription_start_site	-	90274	90274	-738	22726	AN5634
233	CONTIG98	90757	91267	2.88	6.10E-04	transcription_start_site	-	90056	90056	-956	22727	AN5634
858	CONTIG98	89267	89831	2.05	1.30E-02	transcription_start_site	-	90056	90056	507	22727	AN5634
858	CONTIG98	89267	89831	2.05	1.30E-02	transcription_start_site	-	90274	90274	725	22726	AN5634
858	CONTIG98	89267	89831	2.05	1.30E-02	transcription_start_site	-	90541	90541	992	22725	AN5634
2151	CONTIG98	104552	104977	1.32	1.44E-01	transcription_start_site	-	100700	100700	-4064	22742	AN5637
2151	CONTIG98	104552	104977	1.32	1.44E-01	transcription_start_site	-	100595	100595	-4169	22743	AN5637
2151	CONTIG98	104552	104977	1.32	1.44E-01	transcription_start_site	-	100311	100311	-4453	22744	AN5637
2151	CONTIG98	104552	104977	1.32	1.44E-01	transcription_start_site	-	99941	99941	-4823	22745	AN5637
2151	CONTIG98	104552	104977	1.32	1.44E-01	transcription_start_site	-	104279	104279	-485	22747	AN5639
2151	CONTIG98	104552	104977	1.32	1.44E-01	transcription_start_site	-	104190	104190	-574	22748	AN5639
2151	CONTIG98	104552	104977	1.32	1.44E-01	transcription_start_site	-	103998	103998	-766	22749	AN5639
2151	CONTIG98	104552	104977	1.32	1.44E-01	transcription_start_site	-	103770	103770	-994	22750	AN5639
2151	CONTIG98	104552	104977	1.32	1.44E-01	transcription_start_site	-	103686	103686	-1078	22751	AN5639

2151	CONTIG98	104552	104977	1.32	1.44E-01	transcription_start_site	-	103566	103566	-1198	22752 AN5639
2151	CONTIG98	104552	104977	1.32	1.44E-01	transcription_start_site	-	103127	103127	-1637	22753 AN5639
2151	CONTIG98	104552	104977	1.32	1.44E-01	transcription_start_site	-	103003	103003	-1761	22754 AN5639
1564	CONTIG98	113851	114145	1.56	6.84E-02	transcription_start_site	-	108858	108858	-5140	22755 AN5640
1564	CONTIG98	113851	114145	1.56	6.84E-02	transcription_start_site	-	112716	112716	-1282	22759 AN5641
1564	CONTIG98	113851	114145	1.56	6.84E-02	transcription_start_site	-	112618	112618	-1380	22760 AN5641
1564	CONTIG98	113851	114145	1.56	6.84E-02	transcription_start_site	-	112136	112136	-1862	22761 AN5641
1564	CONTIG98	113851	114145	1.56	6.84E-02	transcription_start_site	-	114384	114384	386	22762 AN5642
1564	CONTIG98	113851	114145	1.56	6.84E-02	transcription_start_site	+	115107	115107	-1109	22763 AN5643
1564	CONTIG98	113851	114145	1.56	6.84E-02	transcription_start_site	+	115325	115325	-1327	22764 AN5643
1564	CONTIG98	113851	114145	1.56	6.84E-02	transcription_start_site	+	115406	115406	-1408	22765 AN5643
1564	CONTIG98	113851	114145	1.56	6.84E-02	transcription_start_site	+	115610	115610	-1612	22766 AN5643
1564	CONTIG98	113851	114145	1.56	6.84E-02	transcription_start_site	+	118620	118620	-4622	22767 AN5643
2296	CONTIG98	135977	137016	1.27	1.50E-01	transcription_start_site	-	137216	137216	719	22784 AN5649
2296	CONTIG98	135977	137016	1.27	1.50E-01	transcription_start_site	-	137375	137375	878	22783 AN5649
2296	CONTIG98	135977	137016	1.27	1.50E-01	transcription_start_site	-	137658	137658	1161	22782 AN5649
2296	CONTIG98	135977	137016	1.27	1.50E-01	transcription_start_site	-	134423	134423	-2073	22785 AN5649
1276	CONTIG98	167709	168888	1.71	2.64E-02	transcription_start_site	-	166525	166525	-1773	22817 AN5659
1276	CONTIG98	167709	168888	1.71	2.64E-02	transcription_start_site	-	166323	166323	-1975	22818 AN5659
1276	CONTIG98	167709	168888	1.71	2.64E-02	transcription_start_site	-	166166	166166	-2132	22819 AN5659
1276	CONTIG98	167709	168888	1.71	2.64E-02	transcription_start_site	-	165567	165567	-2731	22820 AN5659
234	CONTIG98	178876	179311	2.88	6.10E-04	transcription_start_site	-	177655	177655	-1438	22821 AN5660
234	CONTIG98	178876	179311	2.88	6.10E-04	transcription_start_site	-	177296	177296	-1797	22822 AN5660
234	CONTIG98	178876	179311	2.88	6.10E-04	transcription_start_site	+	182988	182988	-3894	22823 AN5662
234	CONTIG98	178876	179311	2.88	6.10E-04	transcription_start_site	+	183112	183112	-4018	22824 AN5662
234	CONTIG98	178876	179311	2.88	6.10E-04	transcription_start_site	+	183602	183602	-4508	22825 AN5662
234	CONTIG98	178876	179311	2.88	6.10E-04	transcription_start_site	+	183919	183919	-4825	22826 AN5662
1083	CONTIG98	187882	188166	1.85	2.62E-02	transcription_start_site	+	190863	190863	-2839	22834 AN5665
1083	CONTIG98	187882	188166	1.85	2.62E-02	transcription_start_site	+	191240	191240	-3216	22835 AN5665
1083	CONTIG98	187882	188166	1.85	2.62E-02	transcription_start_site	+	191530	191530	-3506	22836 AN5665
1083	CONTIG98	187882	188166	1.85	2.62E-02	transcription_start_site	+	192472	192472	-4448	22837 AN5665
1468	CONTIG98	196512	196866	1.61	5.88E-02	transcription_start_site	-	193961	193961	-2728	22838 AN5666
1468	CONTIG98	196512	196866	1.61	5.88E-02	transcription_start_site	-	193847	193847	-2842	22839 AN5666
1468	CONTIG98	196512	196866	1.61	5.88E-02	transcription_start_site	-	193482	193482	-3207	22840 AN5666
2021	CONTIG98	197027	197606	1.37	1.25E-01	transcription_start_site	-	193961	193961	-3355	22838 AN5666
2021	CONTIG98	197027	197606	1.37	1.25E-01	transcription_start_site	-	193847	193847	-3469	22839 AN5666
2021	CONTIG98	197027	197606	1.37	1.25E-01	transcription_start_site	-	193482	193482	-3834	22840 AN5666
1468	CONTIG98	196512	196866	1.61	5.88E-02	transcription_start_site	-	197315	197315	626	22842 AN5667
1468	CONTIG98	196512	196866	1.61	5.88E-02	transcription_start_site	-	197632	197632	943	22841 AN5667
1907	CONTIG98	199292	199865	1.41	9.26E-02	transcription_start_site	-	197632	197632	-1946	22841 AN5667
1907	CONTIG98	199292	199865	1.41	9.26E-02	transcription_start_site	-	197315	197315	-2263	22842 AN5667
2021	CONTIG98	197027	197606	1.37	1.25E-01	transcription_start_site	-	197315	197315	-1	22842 AN5667
2021	CONTIG98	197027	197606	1.37	1.25E-01	transcription_start_site	-	197632	197632	315	22841 AN5667
1907	CONTIG98	199292	199865	1.41	9.26E-02	transcription_start_site	-	199509	199509	-69	22843 AN5668
1907	CONTIG98	199292	199865	1.41	9.26E-02	transcription_start_site	-	199384	199384	-194	22844 AN5668
1907	CONTIG98	199292	199865	1.41	9.26E-02	transcription_start_site	-	198809	198809	-769	22845 AN5668
903	CONTIG98	210002	210593	2	1.57E-02	transcription_start_site	+	211676	211676	-1378	22853 AN5671
903	CONTIG98	210002	210593	2	1.57E-02	transcription_start_site	+	212260	212260	-1962	22854 AN5671
903	CONTIG98	210002	210593	2	1.57E-02	transcription_start_site	+	212796	212796	-2498	22855 AN5671
903	CONTIG98	210002	210593	2	1.57E-02	transcription_start_site	+	213183	213183	-2885	22856 AN5671
1084	CONTIG98	210757	211331	1.85	2.62E-02	transcription_start_site	+	211676	211676	-632	22853 AN5671
1084	CONTIG98	210757	211331	1.85	2.62E-02	transcription_start_site	+	212260	212260	-1216	22854 AN5671
1084	CONTIG98	210757	211331	1.85	2.62E-02	transcription_start_site	+	212796	212796	-1752	22855 AN5671
1084	CONTIG98	210757	211331	1.85	2.62E-02	transcription_start_site	+	213183	213183	-2139	22856 AN5671
1678	CONTIG98	216839	217195	1.51	8.01E-02	transcription_start_site	-	216711	216711	-306	22857 AN5672
1678	CONTIG98	216839	217195	1.51	8.01E-02	transcription_start_site	-	216570	216570	-447	22858 AN5672
1678	CONTIG98	216839	217195	1.51	8.01E-02	transcription_start_site	-	216297	216297	-720	22859 AN5672

1678	CONTIG98	216839	217195	1.51	8.01E-02	transcription_start_site	+	217454	217454	-437	22860 AN5673
1678	CONTIG98	216839	217195	1.51	8.01E-02	transcription_start_site	+	217737	217737	-720	22861 AN5673
1678	CONTIG98	216839	217195	1.51	8.01E-02	transcription_start_site	+	217875	217875	-858	22862 AN5673
1678	CONTIG98	216839	217195	1.51	8.01E-02	transcription_start_site	+	218066	218066	-1049	22863 AN5673
1678	CONTIG98	216839	217195	1.51	8.01E-02	transcription_start_site	+	219470	219470	-2453	22864 AN5673
1678	CONTIG98	216839	217195	1.51	8.01E-02	transcription_start_site	+	220391	220391	-3374	22865 AN5673
1678	CONTIG98	216839	217195	1.51	8.01E-02	transcription_start_site	+	220765	220765	-3748	22866 AN5673
1794	CONTIG98	230855	231135	1.46	9.24E-02	transcription_start_site	-	227591	227591	-3404	22870 AN5675
1794	CONTIG98	230855	231135	1.46	9.24E-02	transcription_start_site	-	227062	227062	-3933	22871 AN5675
1794	CONTIG98	230855	231135	1.46	9.24E-02	transcription_start_site	-	231708	231708	713	22880 AN5677
1794	CONTIG98	230855	231135	1.46	9.24E-02	transcription_start_site	-	231964	231964	969	22879 AN5677
1794	CONTIG98	230855	231135	1.46	9.24E-02	transcription_start_site	-	232109	232109	1114	22878 AN5677
2022	CONTIG98	244127	244411	1.37	1.25E-01	transcription_start_site	-	241131	241131	-3138	22892 AN5681
2022	CONTIG98	244127	244411	1.37	1.25E-01	transcription_start_site	-	243818	243818	-451	22899 AN5683
2022	CONTIG98	244127	244411	1.37	1.25E-01	transcription_start_site	-	244900	244900	631	22898 AN5683
2022	CONTIG98	244127	244411	1.37	1.25E-01	transcription_start_site	+	246922	246922	-2653	22905 AN5685
2022	CONTIG98	244127	244411	1.37	1.25E-01	transcription_start_site	+	247178	247178	-2909	22906 AN5685
2022	CONTIG98	244127	244411	1.37	1.25E-01	transcription_start_site	+	249101	249101	-4832	22910 AN5687
1142	CONTIG98	258237	258516	1.8	3.08E-02	transcription_start_site	-	256697	256697	-1679	22917 AN5689
1142	CONTIG98	258237	258516	1.8	3.08E-02	transcription_start_site	-	255925	255925	-2451	22918 AN5689
1142	CONTIG98	258237	258516	1.8	3.08E-02	transcription_start_site	-	259101	259101	724	22922 AN5690
1142	CONTIG98	258237	258516	1.8	3.08E-02	transcription_start_site	+	262015	262015	-3638	22923 AN5691
1142	CONTIG98	258237	258516	1.8	3.08E-02	transcription_start_site	+	262218	262218	-3841	22924 AN5691
1142	CONTIG98	258237	258516	1.8	3.08E-02	transcription_start_site	+	263062	263062	-4685	22925 AN5691
1142	CONTIG98	258237	258516	1.8	3.08E-02	transcription_start_site	+	263344	263344	-4967	22926 AN5691
2458	CONTIG98	283140	283419	1.22	1.93E-01	transcription_start_site	-	281317	281317	-1962	22939 AN5698
2458	CONTIG98	283140	283419	1.22	1.93E-01	transcription_start_site	-	282978	282978	-301	22940 AN5699
2458	CONTIG98	283140	283419	1.22	1.93E-01	transcription_start_site	-	282841	282841	-438	22941 AN5699
2458	CONTIG98	283140	283419	1.22	1.93E-01	transcription_start_site	+	283605	283605	-325	22942 AN5700
2458	CONTIG98	283140	283419	1.22	1.93E-01	transcription_start_site	+	287571	287571	-4291	22943 AN5701
2458	CONTIG98	283140	283419	1.22	1.93E-01	transcription_start_site	+	287649	287649	-4369	22944 AN5701
2458	CONTIG98	283140	283419	1.22	1.93E-01	transcription_start_site	+	287787	287787	-4507	22945 AN5701
2013	CONTIG98	297227	298123	1.37	1.07E-01	transcription_start_site	-	292658	292658	-5017	22949 AN5703
2013	CONTIG98	297227	298123	1.37	1.07E-01	transcription_start_site	-	292259	292259	-5416	22950 AN5703
2013	CONTIG98	297227	298123	1.37	1.07E-01	transcription_start_site	+	300815	300815	-3140	22962 AN5707
2013	CONTIG98	297227	298123	1.37	1.07E-01	transcription_start_site	+	300885	300885	-3210	22963 AN5707
2013	CONTIG98	297227	298123	1.37	1.07E-01	transcription_start_site	+	302197	302197	-4522	22964 AN5708
2013	CONTIG98	297227	298123	1.37	1.07E-01	transcription_start_site	+	302623	302623	-4948	22965 AN5708
27	CONTIG98	308778	309137	3.56	0.00E+00	transcription_start_site	-	308403	308403	-554	22968 AN5709
27	CONTIG98	308778	309137	3.56	0.00E+00	transcription_start_site	-	308254	308254	-703	22969 AN5709
27	CONTIG98	308778	309137	3.56	0.00E+00	transcription_start_site	-	306578	306578	-2379	22970 AN5709
102	CONTIG98	305938	306282	3.22	0.00E+00	transcription_start_site	-	306578	306578	468	22970 AN5709
706	CONTIG98	307218	307852	2.19	3.31E-03	transcription_start_site	-	308254	308254	719	22969 AN5709
706	CONTIG98	307218	307852	2.19	3.31E-03	transcription_start_site	-	308403	308403	868	22968 AN5709
706	CONTIG98	307218	307852	2.19	3.31E-03	transcription_start_site	-	306578	306578	-957	22970 AN5709
617	CONTIG98	316426	316720	2.29	5.20E-03	transcription_start_site	-	312966	312966	-3607	22971 AN5710
617	CONTIG98	316426	316720	2.29	5.20E-03	transcription_start_site	-	312834	312834	-3739	22972 AN5710
617	CONTIG98	316426	316720	2.29	5.20E-03	transcription_start_site	-	312660	312660	-3913	22973 AN5710
617	CONTIG98	316426	316720	2.29	5.20E-03	transcription_start_site	-	312522	312522	-4051	22974 AN5710
617	CONTIG98	316426	316720	2.29	5.20E-03	transcription_start_site	-	312178	312178	-4395	22975 AN5710
617	CONTIG98	316426	316720	2.29	5.20E-03	transcription_start_site	-	311855	311855	-4718	22976 AN5710
617	CONTIG98	316426	316720	2.29	5.20E-03	transcription_start_site	-	311437	311437	-5136	22977 AN5710
27	CONTIG98	308778	309137	3.56	0.00E+00	transcription_start_site	+	313279	313279	-4321	22979 AN5711
27	CONTIG98	308778	309137	3.56	0.00E+00	transcription_start_site	+	313569	313569	-4611	22980 AN5711
27	CONTIG98	308778	309137	3.56	0.00E+00	transcription_start_site	+	313761	313761	-4803	22981 AN5711
617	CONTIG98	316426	316720	2.29	5.20E-03	transcription_start_site	-	316393	316393	-180	22982 AN5712
617	CONTIG98	316426	316720	2.29	5.20E-03	transcription_start_site	-	316147	316147	-426	22983 AN5712

617	CONTIG98	316426	316720	2.29	5.20E-03	transcription_start_site	-	315952	315952	-621	22984 AN5712
617	CONTIG98	316426	316720	2.29	5.20E-03	transcription_start_site	-	315832	315832	-741	22985 AN5712
617	CONTIG98	316426	316720	2.29	5.20E-03	transcription_start_site	-	314779	314779	-1794	22986 AN5712
617	CONTIG98	316426	316720	2.29	5.20E-03	transcription_start_site	-	317524	317524	951	22992 AN5713
617	CONTIG98	316426	316720	2.29	5.20E-03	transcription_start_site	+	321504	321504	-4931	22993 AN5714
1202	CONTIG98	325295	325649	1.76	2.18E-02	transcription_start_site	-	325172	325172	-300	22995 AN5715
1202	CONTIG98	325295	325649	1.76	2.18E-02	transcription_start_site	-	324940	324940	-532	22996 AN5715
1202	CONTIG98	325295	325649	1.76	2.18E-02	transcription_start_site	-	324630	324630	-842	22997 AN5715
1202	CONTIG98	325295	325649	1.76	2.18E-02	transcription_start_site	-	324459	324459	-1013	22998 AN5715
1202	CONTIG98	325295	325649	1.76	2.18E-02	transcription_start_site	+	325945	325945	-473	22999 AN5716
1202	CONTIG98	325295	325649	1.76	2.18E-02	transcription_start_site	+	326257	326257	-785	23000 AN5716
1202	CONTIG98	325295	325649	1.76	2.18E-02	transcription_start_site	+	327181	327181	-1709	23001 AN5716
1202	CONTIG98	325295	325649	1.76	2.18E-02	transcription_start_site	+	327332	327332	-1860	23002 AN5716
1202	CONTIG98	325295	325649	1.76	2.18E-02	transcription_start_site	+	328877	328877	-3405	23003 AN5717
1202	CONTIG98	325295	325649	1.76	2.18E-02	transcription_start_site	+	329165	329165	-3693	23004 AN5717
1202	CONTIG98	325295	325649	1.76	2.18E-02	transcription_start_site	+	329464	329464	-3992	23005 AN5717
1202	CONTIG98	325295	325649	1.76	2.18E-02	transcription_start_site	+	329708	329708	-4236	23006 AN5717
1202	CONTIG98	325295	325649	1.76	2.18E-02	transcription_start_site	+	330191	330191	-4719	23007 AN5717
904	CONTIG98	340504	340778	2	1.57E-02	transcription_start_site	-	337630	337630	-3011	23020 AN5720
904	CONTIG98	340504	340778	2	1.57E-02	transcription_start_site	-	337453	337453	-3188	23021 AN5720
904	CONTIG98	340504	340778	2	1.57E-02	transcription_start_site	-	336770	336770	-3871	23022 AN5720
904	CONTIG98	340504	340778	2	1.57E-02	transcription_start_site	-	336669	336669	-3972	23023 AN5720
904	CONTIG98	340504	340778	2	1.57E-02	transcription_start_site	-	336558	336558	-4083	23024 AN5720
904	CONTIG98	340504	340778	2	1.57E-02	transcription_start_site	-	340314	340314	-327	23025 AN5721
904	CONTIG98	340504	340778	2	1.57E-02	transcription_start_site	+	340709	340709	-68	23026 AN5722
904	CONTIG98	340504	340778	2	1.57E-02	transcription_start_site	+	341424	341424	-783	23027 AN5722
904	CONTIG98	340504	340778	2	1.57E-02	transcription_start_site	+	342504	342504	-1863	23028 AN5722
904	CONTIG98	340504	340778	2	1.57E-02	transcription_start_site	+	343263	343263	-2622	23029 AN5723
904	CONTIG98	340504	340778	2	1.57E-02	transcription_start_site	+	343457	343457	-2816	23030 AN5723
904	CONTIG98	340504	340778	2	1.57E-02	transcription_start_site	+	343550	343550	-2909	23031 AN5723
2297	CONTIG98	350409	351654	1.27	1.59E-01	transcription_start_site	-	345672	345672	-5359	23032 AN5724
1206	CONTIG98	351754	352193	1.76	3.70E-02	transcription_start_site	-	350632	350632	-1341	23036 AN5726
1206	CONTIG98	351754	352193	1.76	3.70E-02	transcription_start_site	-	349408	349408	-2565	23037 AN5726
2297	CONTIG98	350409	351654	1.27	1.59E-01	transcription_start_site	-	350632	350632	-399	23036 AN5726
2297	CONTIG98	350409	351654	1.27	1.59E-01	transcription_start_site	-	349408	349408	-1623	23037 AN5726
1206	CONTIG98	351754	352193	1.76	3.70E-02	transcription_start_site	-	352315	352315	341	23040 AN5727
1206	CONTIG98	351754	352193	1.76	3.70E-02	transcription_start_site	-	353089	353089	1115	23039 AN5727
2297	CONTIG98	350409	351654	1.27	1.59E-01	transcription_start_site	-	352315	352315	1283	23040 AN5727
166	CONTIG98	359034	359538	3.02	0.00E+00	transcription_start_site	-	357976	357976	-1310	23041 AN5728
166	CONTIG98	359034	359538	3.02	0.00E+00	transcription_start_site	-	357204	357204	-2082	23042 AN5728
207	CONTIG98	360086	360580	2.93	2.16E-04	transcription_start_site	-	357976	357976	-2357	23041 AN5728
207	CONTIG98	360086	360580	2.93	2.16E-04	transcription_start_site	-	357204	357204	-3129	23042 AN5728
1013	CONTIG98	373353	373632	1.9	2.12E-02	transcription_start_site	-	373531	373531	38	23050 AN5731
1013	CONTIG98	373353	373632	1.9	2.12E-02	transcription_start_site	-	373949	373949	456	23049 AN5731
1013	CONTIG98	373353	373632	1.9	2.12E-02	transcription_start_site	-	374041	374041	548	23048 AN5731
1013	CONTIG98	373353	373632	1.9	2.12E-02	transcription_start_site	+	374382	374382	-889	23051 AN5732
1013	CONTIG98	373353	373632	1.9	2.12E-02	transcription_start_site	+	375397	375397	-1904	23052 AN5732
1013	CONTIG98	373353	373632	1.9	2.12E-02	transcription_start_site	+	375555	375555	-2062	23053 AN5732
1013	CONTIG98	373353	373632	1.9	2.12E-02	transcription_start_site	+	376614	376614	-3121	23054 AN5733
1013	CONTIG98	373353	373632	1.9	2.12E-02	transcription_start_site	+	377157	377157	-3664	23055 AN5733
1013	CONTIG98	373353	373632	1.9	2.12E-02	transcription_start_site	+	377872	377872	-4379	23056 AN5733
1013	CONTIG98	373353	373632	1.9	2.12E-02	transcription_start_site	+	378489	378489	-4996	23057 AN5733
1795	CONTIG98	412143	412402	1.46	9.24E-02	transcription_start_site	-	409790	409790	-2482	23116 AN5745
1795	CONTIG98	412143	412402	1.46	9.24E-02	transcription_start_site	-	409717	409717	-2555	23117 AN5745
1795	CONTIG98	412143	412402	1.46	9.24E-02	transcription_start_site	-	409582	409582	-2690	23118 AN5745
1795	CONTIG98	412143	412402	1.46	9.24E-02	transcription_start_site	-	411863	411863	-409	23119 AN5746
1795	CONTIG98	412143	412402	1.46	9.24E-02	transcription_start_site	-	411676	411676	-596	23120 AN5746

1795	CONTIG98	412143	412402	1.46	9.24E-02	transcription_start_site	-	411552	411552	-720	23121 AN5746
1795	CONTIG98	412143	412402	1.46	9.24E-02	transcription_start_site	-	411158	411158	-1114	23122 AN5746
1795	CONTIG98	412143	412402	1.46	9.24E-02	transcription_start_site	-	410666	410666	-1606	23123 AN5746
1795	CONTIG98	412143	412402	1.46	9.24E-02	transcription_start_site	+	413073	413073	-800	23124 AN5747
1795	CONTIG98	412143	412402	1.46	9.24E-02	transcription_start_site	+	413356	413356	-1083	23125 AN5747
1795	CONTIG98	412143	412402	1.46	9.24E-02	transcription_start_site	+	413792	413792	-1519	23126 AN5747
1795	CONTIG98	412143	412402	1.46	9.24E-02	transcription_start_site	+	414298	414298	-2025	23127 AN5747
1795	CONTIG98	412143	412402	1.46	9.24E-02	transcription_start_site	+	415019	415019	-2746	23128 AN5748
1795	CONTIG98	412143	412402	1.46	9.24E-02	transcription_start_site	+	415576	415576	-3303	23129 AN5748
1795	CONTIG98	412143	412402	1.46	9.24E-02	transcription_start_site	+	416269	416269	-3996	23130 AN5748
257	CONTIG98	420229	420527	2.83	5.64E-04	transcription_start_site	-	418762	418762	-1616	23131 AN5749
411	CONTIG98	419409	420128	2.59	1.54E-03	transcription_start_site	-	418762	418762	-1006	23131 AN5749
257	CONTIG98	420229	420527	2.83	5.64E-04	transcription_start_site	+	421082	421082	-704	23132 AN5750
257	CONTIG98	420229	420527	2.83	5.64E-04	transcription_start_site	+	421488	421488	-1110	23133 AN5750
411	CONTIG98	419409	420128	2.59	1.54E-03	transcription_start_site	+	421082	421082	-1313	23132 AN5750
411	CONTIG98	419409	420128	2.59	1.54E-03	transcription_start_site	+	421488	421488	-1719	23133 AN5750
539	CONTIG98	427893	428465	2.39	4.46E-04	transcription_start_site	-	426324	426324	-1855	23134 AN5751
539	CONTIG98	427893	428465	2.39	4.46E-04	transcription_start_site	-	426181	426181	-1998	23135 AN5751
539	CONTIG98	427893	428465	2.39	4.46E-04	transcription_start_site	-	425955	425955	-2224	23136 AN5751
539	CONTIG98	427893	428465	2.39	4.46E-04	transcription_start_site	-	425794	425794	-2385	23137 AN5751
539	CONTIG98	427893	428465	2.39	4.46E-04	transcription_start_site	-	425440	425440	-2739	23138 AN5751
539	CONTIG98	427893	428465	2.39	4.46E-04	transcription_start_site	-	425252	425252	-2927	23139 AN5751
539	CONTIG98	427893	428465	2.39	4.46E-04	transcription_start_site	-	425061	425061	-3118	23140 AN5751
539	CONTIG98	427893	428465	2.39	4.46E-04	transcription_start_site	-	424728	424728	-3451	23141 AN5751
539	CONTIG98	427893	428465	2.39	4.46E-04	transcription_start_site	-	424349	424349	-3830	23142 AN5751
539	CONTIG98	427893	428465	2.39	4.46E-04	transcription_start_site	-	423761	423761	-4418	23143 AN5751
539	CONTIG98	427893	428465	2.39	4.46E-04	transcription_start_site	-	423656	423656	-4523	23144 AN5751
539	CONTIG98	427893	428465	2.39	4.46E-04	transcription_start_site	-	423306	423306	-4873	23145 AN5751
539	CONTIG98	427893	428465	2.39	4.46E-04	transcription_start_site	-	427671	427671	-508	23146 AN5752
539	CONTIG98	427893	428465	2.39	4.46E-04	transcription_start_site	-	427127	427127	-1052	23147 AN5752
2310	CONTIG98	432467	432741	1.27	1.66E-01	transcription_start_site	-	427671	427671	-4933	23146 AN5752
539	CONTIG98	427893	428465	2.39	4.46E-04	transcription_start_site	+	428704	428704	-525	23148 AN5753
539	CONTIG98	427893	428465	2.39	4.46E-04	transcription_start_site	+	428833	428833	-654	23149 AN5753
539	CONTIG98	427893	428465	2.39	4.46E-04	transcription_start_site	+	429806	429806	-1627	23150 AN5754
539	CONTIG98	427893	428465	2.39	4.46E-04	transcription_start_site	+	430015	430015	-1836	23151 AN5754
539	CONTIG98	427893	428465	2.39	4.46E-04	transcription_start_site	+	430175	430175	-1996	23152 AN5754
539	CONTIG98	427893	428465	2.39	4.46E-04	transcription_start_site	+	430235	430235	-2056	23153 AN5754
539	CONTIG98	427893	428465	2.39	4.46E-04	transcription_start_site	+	430370	430370	-2191	23154 AN5754
539	CONTIG98	427893	428465	2.39	4.46E-04	transcription_start_site	+	430802	430802	-2623	23155 AN5754
63	CONTIG98	436515	437082	3.37	0.00E+00	transcription_start_site	-	433532	433532	-3266	23156 AN5755
412	CONTIG98	433953	434251	2.59	1.54E-03	transcription_start_site	-	433532	433532	-570	23156 AN5755
701	CONTIG98	434343	435442	2.19	1.28E-03	transcription_start_site	-	433532	433532	-1360	23156 AN5755
2310	CONTIG98	432467	432741	1.27	1.66E-01	transcription_start_site	-	433532	433532	928	23156 AN5755
63	CONTIG98	436515	437082	3.37	0.00E+00	transcription_start_site	+	436227	436227	571	23158 AN5756
63	CONTIG98	436515	437082	3.37	0.00E+00	transcription_start_site	+	436065	436065	733	23157 AN5756
412	CONTIG98	433953	434251	2.59	1.54E-03	transcription_start_site	+	436065	436065	-1963	23157 AN5756
412	CONTIG98	433953	434251	2.59	1.54E-03	transcription_start_site	+	436227	436227	-2125	23158 AN5756
701	CONTIG98	434343	435442	2.19	1.28E-03	transcription_start_site	+	436065	436065	-1172	23157 AN5756
701	CONTIG98	434343	435442	2.19	1.28E-03	transcription_start_site	+	436227	436227	-1334	23158 AN5756
2310	CONTIG98	432467	432741	1.27	1.66E-01	transcription_start_site	+	436065	436065	-3461	23157 AN5756
2310	CONTIG98	432467	432741	1.27	1.66E-01	transcription_start_site	+	436227	436227	-3623	23158 AN5756
1679	CONTIG98	443705	443986	1.51	8.01E-02	transcription_start_site	-	443055	443055	-790	23159 AN5757
1679	CONTIG98	443705	443986	1.51	8.01E-02	transcription_start_site	-	442873	442873	-972	23160 AN5757
1679	CONTIG98	443705	443986	1.51	8.01E-02	transcription_start_site	-	442757	442757	-1088	23161 AN5757
1679	CONTIG98	443705	443986	1.51	8.01E-02	transcription_start_site	-	442554	442554	-1291	23162 AN5757
1679	CONTIG98	443705	443986	1.51	8.01E-02	transcription_start_site	-	442102	442102	-1743	23163 AN5757
1679	CONTIG98	443705	443986	1.51	8.01E-02	transcription_start_site	-	441471	441471	-2374	23164 AN5757

1014	CONTIG98	448802	449086	1.9	2.12E-02	transcription_start_site	-	446674	446674	-2270	23165	AN5758
1014	CONTIG98	448802	449086	1.9	2.12E-02	transcription_start_site	-	446479	446479	-2465	23166	AN5758
1014	CONTIG98	448802	449086	1.9	2.12E-02	transcription_start_site	-	446074	446074	-2870	23167	AN5758
1014	CONTIG98	448802	449086	1.9	2.12E-02	transcription_start_site	-	445587	445587	-3357	23168	AN5758
1014	CONTIG98	448802	449086	1.9	2.12E-02	transcription_start_site	-	449997	449997	1053	23172	AN5759
1014	CONTIG98	448802	449086	1.9	2.12E-02	transcription_start_site	+	453767	453767	-4823	23177	AN5761
1014	CONTIG98	448802	449086	1.9	2.12E-02	transcription_start_site	+	453972	453972	-5028	23178	AN5761
230	CONTIG98	459308	459732	2.88	0.00E+00	transcription_start_site	-	455565	455565	-3955	23181	AN5762
230	CONTIG98	459308	459732	2.88	0.00E+00	transcription_start_site	-	455399	455399	-4121	23182	AN5762
230	CONTIG98	459308	459732	2.88	0.00E+00	transcription_start_site	-	455184	455184	-4336	23183	AN5762
230	CONTIG98	459308	459732	2.88	0.00E+00	transcription_start_site	+	459095	459095	425	23190	AN5763
230	CONTIG98	459308	459732	2.88	0.00E+00	transcription_start_site	+	458879	458879	641	23189	AN5763
230	CONTIG98	459308	459732	2.88	0.00E+00	transcription_start_site	+	458423	458423	1097	23188	AN5763
230	CONTIG98	459308	459732	2.88	0.00E+00	transcription_start_site	+	459902	459902	-382	23191	AN5764
230	CONTIG98	459308	459732	2.88	0.00E+00	transcription_start_site	+	460036	460036	-516	23192	AN5764
230	CONTIG98	459308	459732	2.88	0.00E+00	transcription_start_site	+	460179	460179	-659	23193	AN5764
230	CONTIG98	459308	459732	2.88	0.00E+00	transcription_start_site	+	461496	461496	-1976	23194	AN5764
230	CONTIG98	459308	459732	2.88	0.00E+00	transcription_start_site	+	463086	463086	-3566	23195	AN5765
230	CONTIG98	459308	459732	2.88	0.00E+00	transcription_start_site	+	463274	463274	-3754	23196	AN5765
230	CONTIG98	459308	459732	2.88	0.00E+00	transcription_start_site	+	463495	463495	-3975	23197	AN5765
2459	CONTIG98	463518	463785	1.22	1.93E-01	transcription_start_site	+	463495	463495	156	23197	AN5765
2459	CONTIG98	463518	463785	1.22	1.93E-01	transcription_start_site	+	463274	463274	377	23196	AN5765
2459	CONTIG98	463518	463785	1.22	1.93E-01	transcription_start_site	+	463086	463086	565	23195	AN5765
2459	CONTIG98	463518	463785	1.22	1.93E-01	transcription_start_site	+	468477	468477	-4825	23198	AN5767
462	CONTIG98	470562	471051	2.49	0.00E+00	transcription_start_site	-	470596	470596	-210	23200	AN5768
462	CONTIG98	470562	471051	2.49	0.00E+00	transcription_start_site	-	470486	470486	-320	23201	AN5768
462	CONTIG98	470562	471051	2.49	0.00E+00	transcription_start_site	-	470351	470351	-455	23202	AN5768
462	CONTIG98	470562	471051	2.49	0.00E+00	transcription_start_site	-	470012	470012	-794	23203	AN5768
462	CONTIG98	470562	471051	2.49	0.00E+00	transcription_start_site	-	469754	469754	-1052	23204	AN5768
1469	CONTIG98	471527	471886	1.61	5.88E-02	transcription_start_site	-	470596	470596	-1110	23200	AN5768
1469	CONTIG98	471527	471886	1.61	5.88E-02	transcription_start_site	-	470486	470486	-1220	23201	AN5768
1469	CONTIG98	471527	471886	1.61	5.88E-02	transcription_start_site	-	470351	470351	-1355	23202	AN5768
1469	CONTIG98	471527	471886	1.61	5.88E-02	transcription_start_site	-	470012	470012	-1694	23203	AN5768
1469	CONTIG98	471527	471886	1.61	5.88E-02	transcription_start_site	-	469754	469754	-1952	23204	AN5768
462	CONTIG98	470562	471051	2.49	0.00E+00	transcription_start_site	+	474607	474607	-3800	23205	AN5770
462	CONTIG98	470562	471051	2.49	0.00E+00	transcription_start_site	+	474736	474736	-3929	23206	AN5770
462	CONTIG98	470562	471051	2.49	0.00E+00	transcription_start_site	+	474855	474855	-4048	23207	AN5770
1469	CONTIG98	471527	471886	1.61	5.88E-02	transcription_start_site	+	474607	474607	-2900	23205	AN5770
1469	CONTIG98	471527	471886	1.61	5.88E-02	transcription_start_site	+	474736	474736	-3029	23206	AN5770
1469	CONTIG98	471527	471886	1.61	5.88E-02	transcription_start_site	+	474855	474855	-3148	23207	AN5770
2023	CONTIG98	477313	477592	1.37	1.25E-01	transcription_start_site	-	476734	476734	-718	23208	AN5771
255	CONTIG98	500037	500526	2.83	0.00E+00	transcription_start_site	+	499778	499778	503	23219	AN5775
255	CONTIG98	500037	500526	2.83	0.00E+00	transcription_start_site	+	499202	499202	1079	23218	AN5775
613	CONTIG98	500567	501516	2.29	0.00E+00	transcription_start_site	+	499778	499778	1263	23219	AN5775
255	CONTIG98	500037	500526	2.83	0.00E+00	transcription_start_site	+	500928	500928	-646	23220	AN5776
255	CONTIG98	500037	500526	2.83	0.00E+00	transcription_start_site	+	503388	503388	-3106	23221	AN5776
255	CONTIG98	500037	500526	2.83	0.00E+00	transcription_start_site	+	504078	504078	-3796	23222	AN5776
613	CONTIG98	500567	501516	2.29	0.00E+00	transcription_start_site	+	500928	500928	113	23220	AN5776
613	CONTIG98	500567	501516	2.29	0.00E+00	transcription_start_site	+	503388	503388	-2346	23221	AN5776
613	CONTIG98	500567	501516	2.29	0.00E+00	transcription_start_site	+	504078	504078	-3036	23222	AN5776
1680	CONTIG98	501827	502331	1.51	8.01E-02	transcription_start_site	+	500928	500928	1151	23220	AN5776
1680	CONTIG98	501827	502331	1.51	8.01E-02	transcription_start_site	+	503388	503388	-1309	23221	AN5776
1680	CONTIG98	501827	502331	1.51	8.01E-02	transcription_start_site	+	504078	504078	-1999	23222	AN5776
2311	CONTIG98	525983	526253	1.27	1.66E-01	transcription_start_site	-	522800	522800	-3318	23239	AN5785
2311	CONTIG98	525983	526253	1.27	1.66E-01	transcription_start_site	-	522672	522672	-3446	23240	AN5785
2311	CONTIG98	525983	526253	1.27	1.66E-01	transcription_start_site	-	521978	521978	-4140	23241	AN5785
2311	CONTIG98	525983	526253	1.27	1.66E-01	transcription_start_site	+	525277	525277	841	23245	AN5786

2311	CONTIG98	525983	526253	1.27	1.66E-01	transcription_start_site	+	526094	526094	24	23246	AN5787
2311	CONTIG98	525983	526253	1.27	1.66E-01	transcription_start_site	+	526236	526236	-118	23247	AN5787
2311	CONTIG98	525983	526253	1.27	1.66E-01	transcription_start_site	+	529768	529768	-3650	23248	AN5787
1280	CONTIG98	544202	544491	1.71	4.36E-02	transcription_start_site	+	547450	547450	-3103	23266	AN5795
1015	CONTIG98	553371	553630	1.9	2.12E-02	transcription_start_site	-	553691	553691	190	23268	AN5796
1015	CONTIG98	553371	553630	1.9	2.12E-02	transcription_start_site	+	555825	555825	-2324	23269	AN5797
1015	CONTIG98	553371	553630	1.9	2.12E-02	transcription_start_site	+	557171	557171	-3670	23270	AN5797
1015	CONTIG98	553371	553630	1.9	2.12E-02	transcription_start_site	+	558299	558299	-4798	23271	AN5797
555	CONTIG100	15167	15516	2.38	4.61E-03	transcription_start_site	-	14743	14743	-598	23296	AN5803
555	CONTIG100	15167	15516	2.38	4.61E-03	transcription_start_site	-	14630	14630	-711	23297	AN5803
555	CONTIG100	15167	15516	2.38	4.61E-03	transcription_start_site	-	14361	14361	-980	23298	AN5803
555	CONTIG100	15167	15516	2.38	4.61E-03	transcription_start_site	-	13585	13585	-1756	23299	AN5803
555	CONTIG100	15167	15516	2.38	4.61E-03	transcription_start_site	-	12541	12541	-2800	23300	AN5803
555	CONTIG100	15167	15516	2.38	4.61E-03	transcription_start_site	+	15872	15872	-530	23301	AN5804
555	CONTIG100	15167	15516	2.38	4.61E-03	transcription_start_site	+	16696	16696	-1354	23302	AN5804
555	CONTIG100	15167	15516	2.38	4.61E-03	transcription_start_site	+	17359	17359	-2017	23303	AN5804
555	CONTIG100	15167	15516	2.38	4.61E-03	transcription_start_site	+	17922	17922	-2580	23304	AN5804
555	CONTIG100	15167	15516	2.38	4.61E-03	transcription_start_site	+	18062	18062	-2720	23305	AN5804
555	CONTIG100	15167	15516	2.38	4.61E-03	transcription_start_site	+	18589	18589	-3247	23306	AN5804
1175	CONTIG100	50935	51296	1.78	3.70E-02	transcription_start_site	-	48613	48613	-2502	23340	AN5816
1175	CONTIG100	50935	51296	1.78	3.70E-02	transcription_start_site	-	48310	48310	-2805	23341	AN5816
1175	CONTIG100	50935	51296	1.78	3.70E-02	transcription_start_site	-	47985	47985	-3130	23342	AN5816
1176	CONTIG100	53120	53469	1.78	3.70E-02	transcription_start_site	-	48613	48613	-4681	23340	AN5816
1176	CONTIG100	53120	53469	1.78	3.70E-02	transcription_start_site	-	48310	48310	-4984	23341	AN5816
1175	CONTIG100	50935	51296	1.78	3.70E-02	transcription_start_site	-	51266	51266	150	23348	AN5818
1175	CONTIG100	50935	51296	1.78	3.70E-02	transcription_start_site	-	50748	50748	-367	23349	AN5818
1176	CONTIG100	53120	53469	1.78	3.70E-02	transcription_start_site	-	51266	51266	-2028	23348	AN5818
1176	CONTIG100	53120	53469	1.78	3.70E-02	transcription_start_site	-	50748	50748	-2546	23349	AN5818
1175	CONTIG100	50935	51296	1.78	3.70E-02	transcription_start_site	+	51768	51768	-652	23350	AN5819
1175	CONTIG100	50935	51296	1.78	3.70E-02	transcription_start_site	+	52037	52037	-921	23351	AN5819
1176	CONTIG100	53120	53469	1.78	3.70E-02	transcription_start_site	-	53741	53741	446	23355	AN5820
1176	CONTIG100	53120	53469	1.78	3.70E-02	transcription_start_site	-	54464	54464	1169	23354	AN5820
1176	CONTIG100	53120	53469	1.78	3.70E-02	transcription_start_site	+	56969	56969	-3674	23356	AN5821
1176	CONTIG100	53120	53469	1.78	3.70E-02	transcription_start_site	+	57842	57842	-4547	23357	AN5821
1176	CONTIG100	53120	53469	1.78	3.70E-02	transcription_start_site	+	58201	58201	-4906	23358	AN5821
2392	CONTIG100	63831	64120	1.24	1.93E-01	transcription_start_site	-	62773	62773	-1202	23359	AN5822
2392	CONTIG100	63831	64120	1.24	1.93E-01	transcription_start_site	-	60843	60843	-3132	23360	AN5822
2392	CONTIG100	63831	64120	1.24	1.93E-01	transcription_start_site	-	60243	60243	-3732	23361	AN5822
2392	CONTIG100	63831	64120	1.24	1.93E-01	transcription_start_site	+	68439	68439	-4463	23362	AN5823
1601	CONTIG100	92795	93062	1.54	8.01E-02	transcription_start_site	-	91060	91060	-1868	23378	AN5829
1601	CONTIG100	92795	93062	1.54	8.01E-02	transcription_start_site	-	88024	88024	-4904	23379	AN5829
1601	CONTIG100	92795	93062	1.54	8.01E-02	transcription_start_site	+	94174	94174	-1245	23380	AN5830
1601	CONTIG100	92795	93062	1.54	8.01E-02	transcription_start_site	+	95256	95256	-2327	23381	AN5831
1601	CONTIG100	92795	93062	1.54	8.01E-02	transcription_start_site	+	95310	95310	-2381	23382	AN5831
1601	CONTIG100	92795	93062	1.54	8.01E-02	transcription_start_site	+	96261	96261	-3332	23383	AN5831
2390	CONTIG100	99765	100104	1.24	1.79E-01	transcription_start_site	+	100831	100831	-896	23384	AN5832
2390	CONTIG100	99765	100104	1.24	1.79E-01	transcription_start_site	+	100972	100972	-1037	23385	AN5832
2390	CONTIG100	99765	100104	1.24	1.79E-01	transcription_start_site	+	101476	101476	-1541	23386	AN5832
2390	CONTIG100	99765	100104	1.24	1.79E-01	transcription_start_site	+	103177	103177	-3242	23387	AN5833
2390	CONTIG100	99765	100104	1.24	1.79E-01	transcription_start_site	+	103614	103614	-3679	23388	AN5833
2390	CONTIG100	99765	100104	1.24	1.79E-01	transcription_start_site	+	104059	104059	-4124	23389	AN5833
2390	CONTIG100	99765	100104	1.24	1.79E-01	transcription_start_site	+	104987	104987	-5052	23390	AN5833
1107	CONTIG100	106366	106870	1.83	1.20E-02	transcription_start_site	-	106502	106502	-116	23391	AN5834
1107	CONTIG100	106366	106870	1.83	1.20E-02	transcription_start_site	-	106263	106263	-355	23392	AN5834
1107	CONTIG100	106366	106870	1.83	1.20E-02	transcription_start_site	-	106075	106075	-543	23393	AN5834
1177	CONTIG100	111466	111725	1.78	3.70E-02	transcription_start_site	-	106502	106502	-5093	23391	AN5834
2087	CONTIG100	108921	109405	1.34	1.44E-01	transcription_start_site	-	106502	106502	-2661	23391	AN5834

2087	CONTIG100	108921	109405	1.34	1.44E-01	transcription_start_site	-	106263	106263	-2900	23392 AN5834
2087	CONTIG100	108921	109405	1.34	1.44E-01	transcription_start_site	-	106075	106075	-3088	23393 AN5834
2393	CONTIG100	108076	108425	1.24	1.93E-01	transcription_start_site	-	106502	106502	-1748	23391 AN5834
2393	CONTIG100	108076	108425	1.24	1.93E-01	transcription_start_site	-	106263	106263	-1987	23392 AN5834
2393	CONTIG100	108076	108425	1.24	1.93E-01	transcription_start_site	-	106075	106075	-2175	23393 AN5834
439	CONTIG100	119718	120140	2.53	0.00E+00	transcription_start_site	+	122060	122060	-2131	23394 AN5836
439	CONTIG100	119718	120140	2.53	0.00E+00	transcription_start_site	+	122379	122379	-2450	23395 AN5836
439	CONTIG100	119718	120140	2.53	0.00E+00	transcription_start_site	+	122621	122621	-2692	23396 AN5836
439	CONTIG100	119718	120140	2.53	0.00E+00	transcription_start_site	+	122800	122800	-2871	23397 AN5836
929	CONTIG100	120168	120802	1.98	1.84E-02	transcription_start_site	+	122060	122060	-1575	23394 AN5836
929	CONTIG100	120168	120802	1.98	1.84E-02	transcription_start_site	+	122379	122379	-1894	23395 AN5836
929	CONTIG100	120168	120802	1.98	1.84E-02	transcription_start_site	+	122621	122621	-2136	23396 AN5836
929	CONTIG100	120168	120802	1.98	1.84E-02	transcription_start_site	+	122800	122800	-2315	23397 AN5836
2088	CONTIG100	123243	123577	1.34	1.44E-01	transcription_start_site	+	122800	122800	610	23397 AN5836
2088	CONTIG100	123243	123577	1.34	1.44E-01	transcription_start_site	+	122621	122621	789	23396 AN5836
2088	CONTIG100	123243	123577	1.34	1.44E-01	transcription_start_site	+	122379	122379	1031	23395 AN5836
439	CONTIG100	119718	120140	2.53	0.00E+00	transcription_start_site	+	124906	124906	-4977	23398 AN5837
439	CONTIG100	119718	120140	2.53	0.00E+00	transcription_start_site	+	125047	125047	-5118	23399 AN5837
929	CONTIG100	120168	120802	1.98	1.84E-02	transcription_start_site	+	124906	124906	-4421	23398 AN5837
929	CONTIG100	120168	120802	1.98	1.84E-02	transcription_start_site	+	125047	125047	-4562	23399 AN5837
2088	CONTIG100	123243	123577	1.34	1.44E-01	transcription_start_site	+	124906	124906	-1496	23398 AN5837
2088	CONTIG100	123243	123577	1.34	1.44E-01	transcription_start_site	+	125047	125047	-1637	23399 AN5837
2089	CONTIG100	125493	125977	1.34	1.44E-01	transcription_start_site	+	125047	125047	688	23399 AN5837
2089	CONTIG100	125493	125977	1.34	1.44E-01	transcription_start_site	+	124906	124906	829	23398 AN5837
2088	CONTIG100	123243	123577	1.34	1.44E-01	transcription_start_site	+	127313	127313	-3903	23400 AN5838
2089	CONTIG100	125493	125977	1.34	1.44E-01	transcription_start_site	+	127313	127313	-1578	23400 AN5838
1317	CONTIG100	154887	155170	1.68	5.22E-02	transcription_start_site	-	153888	153888	-1140	23418 AN5843
1317	CONTIG100	154887	155170	1.68	5.22E-02	transcription_start_site	-	153586	153586	-1442	23419 AN5843
1953	CONTIG100	153832	154401	1.39	1.07E-01	transcription_start_site	-	153888	153888	-228	23418 AN5843
1953	CONTIG100	153832	154401	1.39	1.07E-01	transcription_start_site	-	153586	153586	-530	23419 AN5843
1317	CONTIG100	154887	155170	1.68	5.22E-02	transcription_start_site	+	154683	154683	345	23422 AN5844
1317	CONTIG100	154887	155170	1.68	5.22E-02	transcription_start_site	+	154465	154465	563	23421 AN5844
1317	CONTIG100	154887	155170	1.68	5.22E-02	transcription_start_site	+	154138	154138	890	23420 AN5844
1953	CONTIG100	153832	154401	1.39	1.07E-01	transcription_start_site	+	154138	154138	-21	23420 AN5844
1953	CONTIG100	153832	154401	1.39	1.07E-01	transcription_start_site	+	154465	154465	-348	23421 AN5844
1953	CONTIG100	153832	154401	1.39	1.07E-01	transcription_start_site	+	154683	154683	-566	23422 AN5844
1317	CONTIG100	154887	155170	1.68	5.22E-02	transcription_start_site	+	155460	155460	-431	23423 AN5845
1317	CONTIG100	154887	155170	1.68	5.22E-02	transcription_start_site	+	155675	155675	-646	23424 AN5845
1953	CONTIG100	153832	154401	1.39	1.07E-01	transcription_start_site	+	155460	155460	-1343	23423 AN5845
1953	CONTIG100	153832	154401	1.39	1.07E-01	transcription_start_site	+	155675	155675	-1558	23424 AN5845
1317	CONTIG100	154887	155170	1.68	5.22E-02	transcription_start_site	+	156726	156726	-1697	23425 AN5846
1317	CONTIG100	154887	155170	1.68	5.22E-02	transcription_start_site	+	156975	156975	-1946	23426 AN5846
1317	CONTIG100	154887	155170	1.68	5.22E-02	transcription_start_site	+	158217	158217	-3188	23427 AN5846
1953	CONTIG100	153832	154401	1.39	1.07E-01	transcription_start_site	+	156726	156726	-2609	23425 AN5846
1953	CONTIG100	153832	154401	1.39	1.07E-01	transcription_start_site	+	156975	156975	-2858	23426 AN5846
1953	CONTIG100	153832	154401	1.39	1.07E-01	transcription_start_site	+	158217	158217	-4100	23427 AN5846
661	CONTIG100	160292	162282	2.23	3.31E-03	transcription_start_site	-	160265	160265	-1022	23428 AN5847
661	CONTIG100	160292	162282	2.23	3.31E-03	transcription_start_site	-	159892	159892	-1395	23429 AN5847
661	CONTIG100	160292	162282	2.23	3.31E-03	transcription_start_site	+	161237	161237	50	23430 AN5848
661	CONTIG100	160292	162282	2.23	3.31E-03	transcription_start_site	+	161354	161354	-67	23431 AN5848
661	CONTIG100	160292	162282	2.23	3.31E-03	transcription_start_site	+	162792	162792	-1505	23432 AN5848
443	CONTIG100	170779	171128	2.53	2.22E-03	transcription_start_site	+	173304	173304	-2350	23433 AN5849
443	CONTIG100	170779	171128	2.53	2.22E-03	transcription_start_site	+	174066	174066	-3112	23434 AN5849
443	CONTIG100	170779	171128	2.53	2.22E-03	transcription_start_site	+	174235	174235	-3281	23435 AN5849
443	CONTIG100	170779	171128	2.53	2.22E-03	transcription_start_site	+	175712	175712	-4758	23436 AN5849
443	CONTIG100	170779	171128	2.53	2.22E-03	transcription_start_site	+	175842	175842	-4888	23437 AN5849
443	CONTIG100	170779	171128	2.53	2.22E-03	transcription_start_site	+	176097	176097	-5143	23438 AN5849

1602	CONTIG100	170119	170678	1.54	8.01E-02	transcription_start_site	+	173304	173304	-2905	23433	AN5849
1602	CONTIG100	170119	170678	1.54	8.01E-02	transcription_start_site	+	174066	174066	-3667	23434	AN5849
1602	CONTIG100	170119	170678	1.54	8.01E-02	transcription_start_site	+	174235	174235	-3836	23435	AN5849
1730	CONTIG100	173187	173616	1.49	9.24E-02	transcription_start_site	+	173304	173304	97	23433	AN5849
1730	CONTIG100	173187	173616	1.49	9.24E-02	transcription_start_site	+	174066	174066	-664	23434	AN5849
1730	CONTIG100	173187	173616	1.49	9.24E-02	transcription_start_site	+	174235	174235	-833	23435	AN5849
1730	CONTIG100	173187	173616	1.49	9.24E-02	transcription_start_site	+	175712	175712	-2310	23436	AN5849
1730	CONTIG100	173187	173616	1.49	9.24E-02	transcription_start_site	+	175842	175842	-2440	23437	AN5849
1730	CONTIG100	173187	173616	1.49	9.24E-02	transcription_start_site	+	176097	176097	-2695	23438	AN5849
1730	CONTIG100	173187	173616	1.49	9.24E-02	transcription_start_site	+	176649	176649	-3247	23439	AN5849
1955	CONTIG100	171837	172709	1.39	1.25E-01	transcription_start_site	+	173304	173304	-1031	23433	AN5849
1955	CONTIG100	171837	172709	1.39	1.25E-01	transcription_start_site	+	174066	174066	-1793	23434	AN5849
1955	CONTIG100	171837	172709	1.39	1.25E-01	transcription_start_site	+	174235	174235	-1962	23435	AN5849
1955	CONTIG100	171837	172709	1.39	1.25E-01	transcription_start_site	+	175712	175712	-3439	23436	AN5849
1955	CONTIG100	171837	172709	1.39	1.25E-01	transcription_start_site	+	175842	175842	-3569	23437	AN5849
1955	CONTIG100	171837	172709	1.39	1.25E-01	transcription_start_site	+	176097	176097	-3824	23438	AN5849
1955	CONTIG100	171837	172709	1.39	1.25E-01	transcription_start_site	+	176649	176649	-4376	23439	AN5849
1730	CONTIG100	173187	173616	1.49	9.24E-02	transcription_start_site	+	177740	177740	-4338	23440	AN5850
1730	CONTIG100	173187	173616	1.49	9.24E-02	transcription_start_site	+	177820	177820	-4418	23441	AN5850
354	CONTIG100	194927	195366	2.67	0.00E+00	transcription_start_site	+	198112	198112	-2965	23442	AN5852
354	CONTIG100	194927	195366	2.67	0.00E+00	transcription_start_site	+	198598	198598	-3451	23443	AN5852
53	CONTIG100	204917	205191	3.42	0.00E+00	transcription_start_site	-	201180	201180	-3874	23444	AN5853
53	CONTIG100	204917	205191	3.42	0.00E+00	transcription_start_site	-	200674	200674	-4380	23445	AN5853
53	CONTIG100	204917	205191	3.42	0.00E+00	transcription_start_site	-	200614	200614	-4440	23446	AN5853
53	CONTIG100	204917	205191	3.42	0.00E+00	transcription_start_site	-	200313	200313	-4741	23447	AN5853
53	CONTIG100	204917	205191	3.42	0.00E+00	transcription_start_site	-	200117	200117	-4937	23448	AN5853
53	CONTIG100	204917	205191	3.42	0.00E+00	transcription_start_site	-	199952	199952	-5102	23449	AN5853
2234	CONTIG100	203552	204202	1.29	1.50E-01	transcription_start_site	-	201180	201180	-2697	23444	AN5853
2234	CONTIG100	203552	204202	1.29	1.50E-01	transcription_start_site	-	200674	200674	-3203	23445	AN5853
2234	CONTIG100	203552	204202	1.29	1.50E-01	transcription_start_site	-	200614	200614	-3263	23446	AN5853
2234	CONTIG100	203552	204202	1.29	1.50E-01	transcription_start_site	-	200313	200313	-3564	23447	AN5853
2234	CONTIG100	203552	204202	1.29	1.50E-01	transcription_start_site	-	200117	200117	-3760	23448	AN5853
2234	CONTIG100	203552	204202	1.29	1.50E-01	transcription_start_site	-	199952	199952	-3925	23449	AN5853
2234	CONTIG100	203552	204202	1.29	1.50E-01	transcription_start_site	-	199756	199756	-4121	23450	AN5853
2234	CONTIG100	203552	204202	1.29	1.50E-01	transcription_start_site	-	199450	199450	-4427	23451	AN5853
53	CONTIG100	204917	205191	3.42	0.00E+00	transcription_start_site	-	204755	204755	-299	23455	AN5855
53	CONTIG100	204917	205191	3.42	0.00E+00	transcription_start_site	-	204591	204591	-463	23456	AN5855
2234	CONTIG100	203552	204202	1.29	1.50E-01	transcription_start_site	-	204591	204591	714	23456	AN5855
2234	CONTIG100	203552	204202	1.29	1.50E-01	transcription_start_site	-	204755	204755	878	23455	AN5855
53	CONTIG100	204917	205191	3.42	0.00E+00	transcription_start_site	+	206946	206946	-1892	23457	AN5856
53	CONTIG100	204917	205191	3.42	0.00E+00	transcription_start_site	+	207388	207388	-2334	23458	AN5856
53	CONTIG100	204917	205191	3.42	0.00E+00	transcription_start_site	+	209397	209397	-4343	23459	AN5856
53	CONTIG100	204917	205191	3.42	0.00E+00	transcription_start_site	+	209656	209656	-4602	23460	AN5856
1833	CONTIG100	210002	210741	1.44	9.26E-02	transcription_start_site	+	209656	209656	715	23460	AN5856
1833	CONTIG100	210002	210741	1.44	9.26E-02	transcription_start_site	+	209397	209397	974	23459	AN5856
2234	CONTIG100	203552	204202	1.29	1.50E-01	transcription_start_site	+	206946	206946	-3069	23457	AN5856
2234	CONTIG100	203552	204202	1.29	1.50E-01	transcription_start_site	+	207388	207388	-3511	23458	AN5856
1833	CONTIG100	210002	210741	1.44	9.26E-02	transcription_start_site	+	211293	211293	-921	23461	AN5857
142	CONTIG100	218628	218987	3.07	2.67E-04	transcription_start_site	+	220655	220655	-1847	23462	AN5859
142	CONTIG100	218628	218987	3.07	2.67E-04	transcription_start_site	+	221131	221131	-2323	23463	AN5859
142	CONTIG100	218628	218987	3.07	2.67E-04	transcription_start_site	+	221429	221429	-2621	23464	AN5859
975	CONTIG100	219528	220192	1.93	1.06E-02	transcription_start_site	+	220655	220655	-795	23462	AN5859
975	CONTIG100	219528	220192	1.93	1.06E-02	transcription_start_site	+	221131	221131	-1271	23463	AN5859
975	CONTIG100	219528	220192	1.93	1.06E-02	transcription_start_site	+	221429	221429	-1569	23464	AN5859
2238	CONTIG100	215868	216362	1.29	1.66E-01	transcription_start_site	+	220655	220655	-4540	23462	AN5859
2238	CONTIG100	215868	216362	1.29	1.66E-01	transcription_start_site	+	221131	221131	-5016	23463	AN5859
2391	CONTIG100	216463	217112	1.24	1.79E-01	transcription_start_site	+	220655	220655	-3867	23462	AN5859

2391	CONTIG100	216463	217112	1.24	1.79E-01	transcription_start_site	+	221131	221131	-4343	23463 AN5859
2391	CONTIG100	216463	217112	1.24	1.79E-01	transcription_start_site	+	221429	221429	-4641	23464 AN5859
263	CONTIG100	250746	251231	2.82	0.00E+00	transcription_start_site	+	255700	255700	-4711	23504 AN5871
263	CONTIG100	250746	251231	2.82	0.00E+00	transcription_start_site	+	256208	256208	-5219	23505 AN5871
1245	CONTIG100	262221	262705	1.73	2.64E-02	transcription_start_site	-	258584	258584	-3879	23507 AN5872
1245	CONTIG100	262221	262705	1.73	2.64E-02	transcription_start_site	-	258506	258506	-3957	23508 AN5872
1245	CONTIG100	262221	262705	1.73	2.64E-02	transcription_start_site	-	258427	258427	-4036	23509 AN5872
1245	CONTIG100	262221	262705	1.73	2.64E-02	transcription_start_site	-	258229	258229	-4234	23510 AN5872
1245	CONTIG100	262221	262705	1.73	2.64E-02	transcription_start_site	-	257775	257775	-4688	23511 AN5872
1245	CONTIG100	262221	262705	1.73	2.64E-02	transcription_start_site	+	261647	261647	816	23515 AN5873
1245	CONTIG100	262221	262705	1.73	2.64E-02	transcription_start_site	+	261363	261363	1100	23514 AN5873
1245	CONTIG100	262221	262705	1.73	2.64E-02	transcription_start_site	+	264477	264477	-2014	23516 AN5874
1245	CONTIG100	262221	262705	1.73	2.64E-02	transcription_start_site	+	264799	264799	-2336	23517 AN5874
1245	CONTIG100	262221	262705	1.73	2.64E-02	transcription_start_site	+	265006	265006	-2543	23518 AN5874
1245	CONTIG100	262221	262705	1.73	2.64E-02	transcription_start_site	+	265233	265233	-2770	23519 AN5874
1245	CONTIG100	262221	262705	1.73	2.64E-02	transcription_start_site	+	265477	265477	-3014	23520 AN5874
1245	CONTIG100	262221	262705	1.73	2.64E-02	transcription_start_site	+	265672	265672	-3209	23521 AN5874
1318	CONTIG100	291014	291301	1.68	5.22E-02	transcription_start_site	-	287164	287164	-3993	23551 AN5883
1318	CONTIG100	291014	291301	1.68	5.22E-02	transcription_start_site	-	286289	286289	-4868	23552 AN5883
1111	CONTIG100	301966	302240	1.83	3.08E-02	transcription_start_site	+	300993	300993	1110	23561 AN5885
1318	CONTIG100	291014	291301	1.68	5.22E-02	transcription_start_site	+	293102	293102	-1944	23555 AN5885
1318	CONTIG100	291014	291301	1.68	5.22E-02	transcription_start_site	+	293511	293511	-2353	23556 AN5885
1318	CONTIG100	291014	291301	1.68	5.22E-02	transcription_start_site	+	293739	293739	-2581	23557 AN5885
1318	CONTIG100	291014	291301	1.68	5.22E-02	transcription_start_site	+	294236	294236	-3078	23558 AN5885
512	CONTIG100	319514	319793	2.43	3.61E-03	transcription_start_site	+	319392	319392	261	23577 AN5892
294	CONTIG100	325515	326019	2.77	9.51E-04	transcription_start_site	+	329870	329870	-4103	23578 AN5893
294	CONTIG100	325515	326019	2.77	9.51E-04	transcription_start_site	+	330638	330638	-4871	23579 AN5893
356	CONTIG100	328817	329076	2.67	1.22E-03	transcription_start_site	+	329870	329870	-923	23578 AN5893
356	CONTIG100	328817	329076	2.67	1.22E-03	transcription_start_site	+	330638	330638	-1691	23579 AN5893
356	CONTIG100	328817	329076	2.67	1.22E-03	transcription_start_site	+	331827	331827	-2880	23580 AN5893
356	CONTIG100	328817	329076	2.67	1.22E-03	transcription_start_site	+	332066	332066	-3119	23581 AN5893
2086	CONTIG100	329795	330439	1.34	1.28E-01	transcription_start_site	+	329870	329870	247	23578 AN5893
2086	CONTIG100	329795	330439	1.34	1.28E-01	transcription_start_site	+	330638	330638	-521	23579 AN5893
2086	CONTIG100	329795	330439	1.34	1.28E-01	transcription_start_site	+	331827	331827	-1710	23580 AN5893
2086	CONTIG100	329795	330439	1.34	1.28E-01	transcription_start_site	+	332066	332066	-1949	23581 AN5893
1246	CONTIG100	333760	334104	1.73	2.64E-02	transcription_start_site	-	334167	334167	235	23583 AN5894
1246	CONTIG100	333760	334104	1.73	2.64E-02	transcription_start_site	-	334994	334994	1062	23582 AN5894
2640	CONTIG101	4807	5151	1.15	1.93E-01	transcription_start_site	-	2496	2496	-2483	23584 AN5895
2640	CONTIG101	4807	5151	1.15	1.93E-01	transcription_start_site	-	2392	2392	-2587	23585 AN5895
2640	CONTIG101	4807	5151	1.15	1.93E-01	transcription_start_site	-	2304	2304	-2675	23586 AN5895
2640	CONTIG101	4807	5151	1.15	1.93E-01	transcription_start_site	-	2189	2189	-2790	23587 AN5895
2640	CONTIG101	4807	5151	1.15	1.93E-01	transcription_start_site	-	2105	2105	-2874	23588 AN5895
2640	CONTIG101	4807	5151	1.15	1.93E-01	transcription_start_site	-	1714	1714	-3265	23589 AN5895
2640	CONTIG101	4807	5151	1.15	1.93E-01	transcription_start_site	-	843	843	-4136	23590 AN5895
2640	CONTIG101	4807	5151	1.15	1.93E-01	transcription_start_site	-	4902	4902	-77	23592 AN5897
2640	CONTIG101	4807	5151	1.15	1.93E-01	transcription_start_site	-	4479	4479	-500	23593 AN5897
2640	CONTIG101	4807	5151	1.15	1.93E-01	transcription_start_site	+	5575	5575	-596	23594 AN5898
2640	CONTIG101	4807	5151	1.15	1.93E-01	transcription_start_site	+	5919	5919	-940	23595 AN5898
2640	CONTIG101	4807	5151	1.15	1.93E-01	transcription_start_site	+	6100	6100	-1121	23596 AN5898
2640	CONTIG101	4807	5151	1.15	1.93E-01	transcription_start_site	+	6318	6318	-1339	23597 AN5898
2640	CONTIG101	4807	5151	1.15	1.93E-01	transcription_start_site	+	6894	6894	-1915	23598 AN5898
2640	CONTIG101	4807	5151	1.15	1.93E-01	transcription_start_site	+	7414	7414	-2435	23599 AN5898
2640	CONTIG101	4807	5151	1.15	1.93E-01	transcription_start_site	+	7660	7660	-2681	23600 AN5898
2640	CONTIG101	4807	5151	1.15	1.93E-01	transcription_start_site	+	9507	9507	-4528	23601 AN5899
2640	CONTIG101	4807	5151	1.15	1.93E-01	transcription_start_site	+	9599	9599	-4620	23602 AN5899
2640	CONTIG101	4807	5151	1.15	1.93E-01	transcription_start_site	+	9721	9721	-4742	23603 AN5899
2223	CONTIG101	14043	14392	1.29	1.25E-01	transcription_start_site	+	14293	14293	-75	23607 AN5900

2223	CONTIG101	14043	14392	1.29	1.25E-01	transcription_start_site	+	13909	13909	308	23606	AN5900
928	CONTIG101	15318	15801	1.98	1.11E-02	transcription_start_site	-	15858	15858	298	23609	AN5901
928	CONTIG101	15318	15801	1.98	1.11E-02	transcription_start_site	-	15211	15211	-348	23610	AN5901
928	CONTIG101	15318	15801	1.98	1.11E-02	transcription_start_site	-	15964	15964	404	23608	AN5901
2223	CONTIG101	14043	14392	1.29	1.25E-01	transcription_start_site	-	15211	15211	993	23610	AN5901
928	CONTIG101	15318	15801	1.98	1.11E-02	transcription_start_site	+	16282	16282	-722	23611	AN5902
928	CONTIG101	15318	15801	1.98	1.11E-02	transcription_start_site	+	17083	17083	-1523	23612	AN5902
2223	CONTIG101	14043	14392	1.29	1.25E-01	transcription_start_site	+	16282	16282	-2064	23611	AN5902
2223	CONTIG101	14043	14392	1.29	1.25E-01	transcription_start_site	+	17083	17083	-2865	23612	AN5902
928	CONTIG101	15318	15801	1.98	1.11E-02	transcription_start_site	+	18508	18508	-2948	23613	AN5903
928	CONTIG101	15318	15801	1.98	1.11E-02	transcription_start_site	+	19062	19062	-3502	23614	AN5903
928	CONTIG101	15318	15801	1.98	1.11E-02	transcription_start_site	+	19223	19223	-3663	23615	AN5903
928	CONTIG101	15318	15801	1.98	1.11E-02	transcription_start_site	+	19688	19688	-4128	23616	AN5903
928	CONTIG101	15318	15801	1.98	1.11E-02	transcription_start_site	+	20440	20440	-4880	23617	AN5903
2223	CONTIG101	14043	14392	1.29	1.25E-01	transcription_start_site	+	18508	18508	-4290	23613	AN5903
2223	CONTIG101	14043	14392	1.29	1.25E-01	transcription_start_site	+	19062	19062	-4844	23614	AN5903
2223	CONTIG101	14043	14392	1.29	1.25E-01	transcription_start_site	+	19223	19223	-5005	23615	AN5903
469	CONTIG101	29405	29689	2.48	1.22E-03	transcription_start_site	-	27205	27205	-2342	23626	AN5906
469	CONTIG101	29405	29689	2.48	1.22E-03	transcription_start_site	-	27050	27050	-2497	23627	AN5906
469	CONTIG101	29405	29689	2.48	1.22E-03	transcription_start_site	-	26436	26436	-3111	23628	AN5906
469	CONTIG101	29405	29689	2.48	1.22E-03	transcription_start_site	-	28619	28619	-928	23629	AN5907
469	CONTIG101	29405	29689	2.48	1.22E-03	transcription_start_site	+	30450	30450	-903	23630	AN5908
1771	CONTIG101	55809	56238	1.47	5.05E-02	transcription_start_site	-	52385	52385	-3638	23671	AN5917
1771	CONTIG101	55809	56238	1.47	5.05E-02	transcription_start_site	-	52009	52009	-4014	23672	AN5917
1771	CONTIG101	55809	56238	1.47	5.05E-02	transcription_start_site	+	55191	55191	832	23675	AN5918
1771	CONTIG101	55809	56238	1.47	5.05E-02	transcription_start_site	+	55091	55091	932	23674	AN5918
1771	CONTIG101	55809	56238	1.47	5.05E-02	transcription_start_site	+	55016	55016	1007	23673	AN5918
1771	CONTIG101	55809	56238	1.47	5.05E-02	transcription_start_site	+	59301	59301	-3277	23678	AN5920
1771	CONTIG101	55809	56238	1.47	5.05E-02	transcription_start_site	+	59672	59672	-3648	23679	AN5920
2224	CONTIG101	65928	66277	1.29	1.25E-01	transcription_start_site	-	65545	65545	-557	23680	AN5921
2224	CONTIG101	65928	66277	1.29	1.25E-01	transcription_start_site	-	65193	65193	-909	23681	AN5921
2224	CONTIG101	65928	66277	1.29	1.25E-01	transcription_start_site	-	64033	64033	-2069	23682	AN5921
2224	CONTIG101	65928	66277	1.29	1.25E-01	transcription_start_site	-	63752	63752	-2350	23683	AN5921
1462	CONTIG101	88804	89078	1.61	4.36E-02	transcription_start_site	-	85207	85207	-3734	23689	AN5926
1462	CONTIG101	88804	89078	1.61	4.36E-02	transcription_start_site	-	85051	85051	-3890	23690	AN5926
1462	CONTIG101	88804	89078	1.61	4.36E-02	transcription_start_site	-	84576	84576	-4365	23691	AN5926
1462	CONTIG101	88804	89078	1.61	4.36E-02	transcription_start_site	-	84362	84362	-4579	23692	AN5926
219	CONTIG101	92644	93063	2.9	0.00E+00	transcription_start_site	-	88250	88250	-4603	23693	AN5927
1462	CONTIG101	88804	89078	1.61	4.36E-02	transcription_start_site	-	88250	88250	-691	23693	AN5927
1462	CONTIG101	88804	89078	1.61	4.36E-02	transcription_start_site	-	87561	87561	-1380	23694	AN5927
1547	CONTIG101	91284	91928	1.56	3.33E-02	transcription_start_site	-	88250	88250	-3356	23693	AN5927
1547	CONTIG101	91284	91928	1.56	3.33E-02	transcription_start_site	-	87561	87561	-4045	23694	AN5927
2641	CONTIG101	90384	90668	1.15	1.93E-01	transcription_start_site	-	88250	88250	-2276	23693	AN5927
2641	CONTIG101	90384	90668	1.15	1.93E-01	transcription_start_site	-	87561	87561	-2965	23694	AN5927
1462	CONTIG101	88804	89078	1.61	4.36E-02	transcription_start_site	+	88843	88843	98	23696	AN5928
1462	CONTIG101	88804	89078	1.61	4.36E-02	transcription_start_site	+	88763	88763	178	23695	AN5928
1462	CONTIG101	88804	89078	1.61	4.36E-02	transcription_start_site	+	89183	89183	-242	23697	AN5928
1462	CONTIG101	88804	89078	1.61	4.36E-02	transcription_start_site	+	89269	89269	-328	23698	AN5928
219	CONTIG101	92644	93063	2.9	0.00E+00	transcription_start_site	-	91881	91881	-972	23699	AN5929
219	CONTIG101	92644	93063	2.9	0.00E+00	transcription_start_site	-	91448	91448	-1405	23700	AN5929
219	CONTIG101	92644	93063	2.9	0.00E+00	transcription_start_site	-	91018	91018	-1835	23701	AN5929
219	CONTIG101	92644	93063	2.9	0.00E+00	transcription_start_site	-	90658	90658	-2195	23702	AN5929
219	CONTIG101	92644	93063	2.9	0.00E+00	transcription_start_site	-	90280	90280	-2573	23703	AN5929
219	CONTIG101	92644	93063	2.9	0.00E+00	transcription_start_site	-	90163	90163	-2690	23704	AN5929
977	CONTIG101	96241	96651	1.93	1.30E-02	transcription_start_site	-	91881	91881	-4565	23699	AN5929
977	CONTIG101	96241	96651	1.93	1.30E-02	transcription_start_site	-	91448	91448	-4998	23700	AN5929
1547	CONTIG101	91284	91928	1.56	3.33E-02	transcription_start_site	-	91448	91448	-158	23700	AN5929

1547	CONTIG101	91284	91928	1.56	3.33E-02	transcription_start_site	-	91881	91881	275	23699 AN5929
1547	CONTIG101	91284	91928	1.56	3.33E-02	transcription_start_site	-	91018	91018	-588	23701 AN5929
1547	CONTIG101	91284	91928	1.56	3.33E-02	transcription_start_site	-	90658	90658	-948	23702 AN5929
1547	CONTIG101	91284	91928	1.56	3.33E-02	transcription_start_site	-	90280	90280	-1326	23703 AN5929
1547	CONTIG101	91284	91928	1.56	3.33E-02	transcription_start_site	-	90163	90163	-1443	23704 AN5929
2641	CONTIG101	90384	90668	1.15	1.93E-01	transcription_start_site	-	90658	90658	132	23702 AN5929
2641	CONTIG101	90384	90668	1.15	1.93E-01	transcription_start_site	-	90280	90280	-246	23703 AN5929
2641	CONTIG101	90384	90668	1.15	1.93E-01	transcription_start_site	-	90163	90163	-363	23704 AN5929
2641	CONTIG101	90384	90668	1.15	1.93E-01	transcription_start_site	-	91018	91018	492	23701 AN5929
2641	CONTIG101	90384	90668	1.15	1.93E-01	transcription_start_site	-	91448	91448	922	23700 AN5929
219	CONTIG101	92644	93063	2.9	0.00E+00	transcription_start_site	+	97469	97469	-4615	23705 AN5930
219	CONTIG101	92644	93063	2.9	0.00E+00	transcription_start_site	+	97681	97681	-4827	23706 AN5930
219	CONTIG101	92644	93063	2.9	0.00E+00	transcription_start_site	+	97872	97872	-5018	23707 AN5930
977	CONTIG101	96241	96651	1.93	1.30E-02	transcription_start_site	+	97469	97469	-1023	23705 AN5930
977	CONTIG101	96241	96651	1.93	1.30E-02	transcription_start_site	+	97681	97681	-1235	23706 AN5930
977	CONTIG101	96241	96651	1.93	1.30E-02	transcription_start_site	+	97872	97872	-1426	23707 AN5930
977	CONTIG101	96241	96651	1.93	1.30E-02	transcription_start_site	+	98104	98104	-1658	23708 AN5930
2516	CONTIG101	102154	102441	1.2	1.66E-01	transcription_start_site	-	101955	101955	-342	23709 AN5931
2516	CONTIG101	102154	102441	1.2	1.66E-01	transcription_start_site	-	101664	101664	-633	23710 AN5931
2516	CONTIG101	102154	102441	1.2	1.66E-01	transcription_start_site	-	101385	101385	-912	23711 AN5931
2516	CONTIG101	102154	102441	1.2	1.66E-01	transcription_start_site	-	100365	100365	-1932	23712 AN5931
2516	CONTIG101	102154	102441	1.2	1.66E-01	transcription_start_site	-	99735	99735	-2562	23713 AN5931
2516	CONTIG101	102154	102441	1.2	1.66E-01	transcription_start_site	-	99554	99554	-2743	23714 AN5931
271	CONTIG101	118894	119166	2.8	2.39E-04	transcription_start_site	-	115857	115857	-3173	23722 AN5934
271	CONTIG101	118894	119166	2.8	2.39E-04	transcription_start_site	-	114503	114503	-4527	23723 AN5934
271	CONTIG101	118894	119166	2.8	2.39E-04	transcription_start_site	-	114005	114005	-5025	23724 AN5934
271	CONTIG101	118894	119166	2.8	2.39E-04	transcription_start_site	-	113904	113904	-5126	23725 AN5934
271	CONTIG101	118894	119166	2.8	2.39E-04	transcription_start_site	-	118673	118673	-357	23728 AN5935
271	CONTIG101	118894	119166	2.8	2.39E-04	transcription_start_site	-	118304	118304	-726	23729 AN5935
271	CONTIG101	118894	119166	2.8	2.39E-04	transcription_start_site	-	118072	118072	-958	23730 AN5935
271	CONTIG101	118894	119166	2.8	2.39E-04	transcription_start_site	-	117879	117879	-1151	23731 AN5935
334	CONTIG101	130579	130863	2.71	6.10E-04	transcription_start_site	-	127445	127445	-3276	23732 AN5936
334	CONTIG101	130579	130863	2.71	6.10E-04	transcription_start_site	-	127288	127288	-3433	23733 AN5936
334	CONTIG101	130579	130863	2.71	6.10E-04	transcription_start_site	-	127111	127111	-3610	23734 AN5936
334	CONTIG101	130579	130863	2.71	6.10E-04	transcription_start_site	-	129661	129661	-1060	23735 AN5937
334	CONTIG101	130579	130863	2.71	6.10E-04	transcription_start_site	+	131544	131544	-823	23736 AN5938
334	CONTIG101	130579	130863	2.71	6.10E-04	transcription_start_site	+	131704	131704	-983	23737 AN5938
334	CONTIG101	130579	130863	2.71	6.10E-04	transcription_start_site	+	132237	132237	-1516	23738 AN5938
334	CONTIG101	130579	130863	2.71	6.10E-04	transcription_start_site	+	132433	132433	-1712	23739 AN5938
334	CONTIG101	130579	130863	2.71	6.10E-04	transcription_start_site	+	132680	132680	-1959	23740 AN5938
2225	CONTIG101	134794	135218	1.29	1.25E-01	transcription_start_site	-	134997	134997	-9	23742 AN5939
2225	CONTIG101	134794	135218	1.29	1.25E-01	transcription_start_site	-	135216	135216	210	23741 AN5939
821	CONTIG101	149106	149757	2.07	1.28E-03	transcription_start_site	-	145929	145929	-3502	23750 AN5942
821	CONTIG101	149106	149757	2.07	1.28E-03	transcription_start_site	-	145640	145640	-3791	23751 AN5942
300	CONTIG101	153846	154120	2.76	2.16E-04	transcription_start_site	-	148998	148998	-4985	23752 AN5943
300	CONTIG101	153846	154120	2.76	2.16E-04	transcription_start_site	-	148919	148919	-5064	23753 AN5943
821	CONTIG101	149106	149757	2.07	1.28E-03	transcription_start_site	-	148998	148998	-433	23752 AN5943
821	CONTIG101	149106	149757	2.07	1.28E-03	transcription_start_site	-	148919	148919	-512	23753 AN5943
821	CONTIG101	149106	149757	2.07	1.28E-03	transcription_start_site	-	148696	148696	-735	23754 AN5943
821	CONTIG101	149106	149757	2.07	1.28E-03	transcription_start_site	-	148547	148547	-884	23755 AN5943
821	CONTIG101	149106	149757	2.07	1.28E-03	transcription_start_site	-	148274	148274	-1157	23756 AN5943
821	CONTIG101	149106	149757	2.07	1.28E-03	transcription_start_site	-	148164	148164	-1267	23757 AN5943
821	CONTIG101	149106	149757	2.07	1.28E-03	transcription_start_site	-	147929	147929	-1502	23758 AN5943
2220	CONTIG101	152326	152900	1.29	1.07E-01	transcription_start_site	-	148998	148998	-3615	23752 AN5943
2220	CONTIG101	152326	152900	1.29	1.07E-01	transcription_start_site	-	148919	148919	-3694	23753 AN5943
2220	CONTIG101	152326	152900	1.29	1.07E-01	transcription_start_site	-	148696	148696	-3917	23754 AN5943
2220	CONTIG101	152326	152900	1.29	1.07E-01	transcription_start_site	-	148547	148547	-4066	23755 AN5943

2220	CONTIG101	152326	152900	1.29	1.07E-01	transcription_start_site	-	148274	148274	-4339	23756	AN5943
2220	CONTIG101	152326	152900	1.29	1.07E-01	transcription_start_site	-	148164	148164	-4449	23757	AN5943
2220	CONTIG101	152326	152900	1.29	1.07E-01	transcription_start_site	-	147929	147929	-4684	23758	AN5943
2642	CONTIG101	153096	153725	1.15	1.93E-01	transcription_start_site	-	148998	148998	-4412	23752	AN5943
2642	CONTIG101	153096	153725	1.15	1.93E-01	transcription_start_site	-	148919	148919	-4491	23753	AN5943
2642	CONTIG101	153096	153725	1.15	1.93E-01	transcription_start_site	-	148696	148696	-4714	23754	AN5943
2642	CONTIG101	153096	153725	1.15	1.93E-01	transcription_start_site	-	148547	148547	-4863	23755	AN5943
2642	CONTIG101	153096	153725	1.15	1.93E-01	transcription_start_site	-	148274	148274	-5136	23756	AN5943
2642	CONTIG101	153096	153725	1.15	1.93E-01	transcription_start_site	-	148164	148164	-5246	23757	AN5943
300	CONTIG101	153846	154120	2.76	2.16E-04	transcription_start_site	-	153810	153810	-173	23759	AN5944
300	CONTIG101	153846	154120	2.76	2.16E-04	transcription_start_site	-	153160	153160	-823	23760	AN5944
300	CONTIG101	153846	154120	2.76	2.16E-04	transcription_start_site	-	152017	152017	-1966	23761	AN5944
2220	CONTIG101	152326	152900	1.29	1.07E-01	transcription_start_site	-	153160	153160	547	23760	AN5944
2220	CONTIG101	152326	152900	1.29	1.07E-01	transcription_start_site	-	152017	152017	-596	23761	AN5944
2220	CONTIG101	152326	152900	1.29	1.07E-01	transcription_start_site	-	153810	153810	1197	23759	AN5944
2642	CONTIG101	153096	153725	1.15	1.93E-01	transcription_start_site	-	153160	153160	-250	23760	AN5944
2642	CONTIG101	153096	153725	1.15	1.93E-01	transcription_start_site	-	153810	153810	399	23759	AN5944
2642	CONTIG101	153096	153725	1.15	1.93E-01	transcription_start_site	-	152017	152017	-1393	23761	AN5944
685	CONTIG101	166503	167242	2.21	4.61E-03	transcription_start_site	-	165669	165669	-1203	23766	AN5947
685	CONTIG101	166503	167242	2.21	4.61E-03	transcription_start_site	-	165428	165428	-1444	23767	AN5947
685	CONTIG101	166503	167242	2.21	4.61E-03	transcription_start_site	-	164494	164494	-2378	23768	AN5947
685	CONTIG101	166503	167242	2.21	4.61E-03	transcription_start_site	-	164059	164059	-2813	23769	AN5947
685	CONTIG101	166503	167242	2.21	4.61E-03	transcription_start_site	-	163222	163222	-3650	23770	AN5947
685	CONTIG101	166503	167242	2.21	4.61E-03	transcription_start_site	-	161796	161796	-5076	23771	AN5947
685	CONTIG101	166503	167242	2.21	4.61E-03	transcription_start_site	-	161645	161645	-5227	23772	AN5947
1770	CONTIG101	167263	168353	1.47	4.42E-02	transcription_start_site	-	165669	165669	-2139	23766	AN5947
1770	CONTIG101	167263	168353	1.47	4.42E-02	transcription_start_site	-	165428	165428	-2380	23767	AN5947
1770	CONTIG101	167263	168353	1.47	4.42E-02	transcription_start_site	-	164494	164494	-3314	23768	AN5947
1770	CONTIG101	167263	168353	1.47	4.42E-02	transcription_start_site	-	164059	164059	-3749	23769	AN5947
1770	CONTIG101	167263	168353	1.47	4.42E-02	transcription_start_site	-	163222	163222	-4586	23770	AN5947
2643	CONTIG101	170345	170694	1.15	1.93E-01	transcription_start_site	-	165669	165669	-4850	23766	AN5947
2643	CONTIG101	170345	170694	1.15	1.93E-01	transcription_start_site	-	165428	165428	-5091	23767	AN5947
685	CONTIG101	166503	167242	2.21	4.61E-03	transcription_start_site	-	168208	168208	1335	23776	AN5948
1770	CONTIG101	167263	168353	1.47	4.42E-02	transcription_start_site	-	168208	168208	400	23776	AN5948
1770	CONTIG101	167263	168353	1.47	4.42E-02	transcription_start_site	-	168522	168522	714	23775	AN5948
1770	CONTIG101	167263	168353	1.47	4.42E-02	transcription_start_site	-	168616	168616	808	23774	AN5948
1770	CONTIG101	167263	168353	1.47	4.42E-02	transcription_start_site	-	168936	168936	1128	23773	AN5948
2643	CONTIG101	170345	170694	1.15	1.93E-01	transcription_start_site	-	168936	168936	-1583	23773	AN5948
2643	CONTIG101	170345	170694	1.15	1.93E-01	transcription_start_site	-	168616	168616	-1903	23774	AN5948
2643	CONTIG101	170345	170694	1.15	1.93E-01	transcription_start_site	-	168522	168522	-1997	23775	AN5948
2643	CONTIG101	170345	170694	1.15	1.93E-01	transcription_start_site	-	168208	168208	-2311	23776	AN5948
685	CONTIG101	166503	167242	2.21	4.61E-03	transcription_start_site	+	169790	169790	-2917	23777	AN5949
685	CONTIG101	166503	167242	2.21	4.61E-03	transcription_start_site	+	169872	169872	-2999	23778	AN5949
1770	CONTIG101	167263	168353	1.47	4.42E-02	transcription_start_site	+	169790	169790	-1982	23777	AN5949
1770	CONTIG101	167263	168353	1.47	4.42E-02	transcription_start_site	+	169872	169872	-2064	23778	AN5949
2643	CONTIG101	170345	170694	1.15	1.93E-01	transcription_start_site	+	169872	169872	647	23778	AN5949
2643	CONTIG101	170345	170694	1.15	1.93E-01	transcription_start_site	+	169790	169790	729	23777	AN5949
2643	CONTIG101	170345	170694	1.15	1.93E-01	transcription_start_site	+	174272	174272	-3752	23785	AN5951
2643	CONTIG101	170345	170694	1.15	1.93E-01	transcription_start_site	+	174751	174751	-4231	23786	AN5951
2643	CONTIG101	170345	170694	1.15	1.93E-01	transcription_start_site	+	175048	175048	-4528	23787	AN5951
2643	CONTIG101	170345	170694	1.15	1.93E-01	transcription_start_site	+	175134	175134	-4614	23788	AN5951
2226	CONTIG101	179565	179909	1.29	1.25E-01	transcription_start_site	-	177000	177000	-2737	23789	AN5952
2226	CONTIG101	179565	179909	1.29	1.25E-01	transcription_start_site	-	176742	176742	-2995	23790	AN5952
2226	CONTIG101	179565	179909	1.29	1.25E-01	transcription_start_site	-	176363	176363	-3374	23791	AN5952
2226	CONTIG101	179565	179909	1.29	1.25E-01	transcription_start_site	-	175829	175829	-3908	23792	AN5952
421	CONTIG101	185405	185679	2.57	9.51E-04	transcription_start_site	-	180869	180869	-4673	23795	AN5954
421	CONTIG101	185405	185679	2.57	9.51E-04	transcription_start_site	-	180419	180419	-5123	23796	AN5954

2226	CONTIG101	179565	179909	1.29	1.25E-01	transcription_start_site	-	180419	180419	682	23796	AN5954
2226	CONTIG101	179565	179909	1.29	1.25E-01	transcription_start_site	-	180869	180869	1132	23795	AN5954
200	CONTIG101	187820	188089	2.94	0.00E+00	transcription_start_site	-	184649	184649	-3305	23797	AN5955
200	CONTIG101	187820	188089	2.94	0.00E+00	transcription_start_site	-	184425	184425	-3529	23798	AN5955
200	CONTIG101	187820	188089	2.94	0.00E+00	transcription_start_site	-	183804	183804	-4150	23799	AN5955
200	CONTIG101	187820	188089	2.94	0.00E+00	transcription_start_site	-	183573	183573	-4381	23800	AN5955
200	CONTIG101	187820	188089	2.94	0.00E+00	transcription_start_site	-	183343	183343	-4611	23801	AN5955
200	CONTIG101	187820	188089	2.94	0.00E+00	transcription_start_site	-	182935	182935	-5019	23802	AN5955
421	CONTIG101	185405	185679	2.57	9.51E-04	transcription_start_site	-	184649	184649	-893	23797	AN5955
421	CONTIG101	185405	185679	2.57	9.51E-04	transcription_start_site	-	184425	184425	-1117	23798	AN5955
421	CONTIG101	185405	185679	2.57	9.51E-04	transcription_start_site	-	183804	183804	-1738	23799	AN5955
421	CONTIG101	185405	185679	2.57	9.51E-04	transcription_start_site	-	183573	183573	-1969	23800	AN5955
421	CONTIG101	185405	185679	2.57	9.51E-04	transcription_start_site	-	183343	183343	-2199	23801	AN5955
421	CONTIG101	185405	185679	2.57	9.51E-04	transcription_start_site	-	182935	182935	-2607	23802	AN5955
200	CONTIG101	187820	188089	2.94	0.00E+00	transcription_start_site	-	187883	187883	-71	23804	AN5956
200	CONTIG101	187820	188089	2.94	0.00E+00	transcription_start_site	-	188173	188173	218	23803	AN5956
200	CONTIG101	187820	188089	2.94	0.00E+00	transcription_start_site	-	187725	187725	-229	23805	AN5956
200	CONTIG101	187820	188089	2.94	0.00E+00	transcription_start_site	-	187362	187362	-592	23806	AN5956
1210	CONTIG101	192615	192959	1.75	1.44E-02	transcription_start_site	-	188173	188173	-4614	23803	AN5956
1210	CONTIG101	192615	192959	1.75	1.44E-02	transcription_start_site	-	187883	187883	-4904	23804	AN5956
1210	CONTIG101	192615	192959	1.75	1.44E-02	transcription_start_site	-	187725	187725	-5062	23805	AN5956
200	CONTIG101	187820	188089	2.94	0.00E+00	transcription_start_site	+	190469	190469	-2514	23807	AN5957
200	CONTIG101	187820	188089	2.94	0.00E+00	transcription_start_site	+	190937	190937	-2982	23808	AN5957
200	CONTIG101	187820	188089	2.94	0.00E+00	transcription_start_site	+	191598	191598	-3643	23809	AN5957
421	CONTIG101	185405	185679	2.57	9.51E-04	transcription_start_site	+	190469	190469	-4927	23807	AN5957
1470	CONTIG102	1446	2005	1.61	6.84E-02	transcription_start_site	+	628	628	1097	23830	AN5964
492	CONTIG102	5790	6289	2.46	3.61E-03	transcription_start_site	-	1622	1622	-4417	23831	AN5965
1360	CONTIG102	4358	4787	1.66	5.88E-02	transcription_start_site	-	1622	1622	-2950	23831	AN5965
1470	CONTIG102	1446	2005	1.61	6.84E-02	transcription_start_site	-	1622	1622	-103	23831	AN5965
26	CONTIG102	7955	9281	3.57	0.00E+00	transcription_start_site	-	4871	4871	-3747	23832	AN5966
26	CONTIG102	7955	9281	3.57	0.00E+00	transcription_start_site	-	4031	4031	-4587	23833	AN5966
26	CONTIG102	7955	9281	3.57	0.00E+00	transcription_start_site	-	3223	3223	-5395	23834	AN5966
492	CONTIG102	5790	6289	2.46	3.61E-03	transcription_start_site	-	4871	4871	-1168	23832	AN5966
492	CONTIG102	5790	6289	2.46	3.61E-03	transcription_start_site	-	4031	4031	-2008	23833	AN5966
492	CONTIG102	5790	6289	2.46	3.61E-03	transcription_start_site	-	3223	3223	-2816	23834	AN5966
1360	CONTIG102	4358	4787	1.66	5.88E-02	transcription_start_site	-	4871	4871	298	23832	AN5966
1360	CONTIG102	4358	4787	1.66	5.88E-02	transcription_start_site	-	4031	4031	-541	23833	AN5966
1360	CONTIG102	4358	4787	1.66	5.88E-02	transcription_start_site	-	3223	3223	-1349	23834	AN5966
26	CONTIG102	7955	9281	3.57	0.00E+00	transcription_start_site	+	12859	12859	-4241	23835	AN5967
26	CONTIG102	7955	9281	3.57	0.00E+00	transcription_start_site	+	13014	13014	-4396	23836	AN5967
26	CONTIG102	7955	9281	3.57	0.00E+00	transcription_start_site	+	13807	13807	-5189	23837	AN5968
26	CONTIG102	7955	9281	3.57	0.00E+00	transcription_start_site	+	14115	14115	-5497	23838	AN5968
26	CONTIG102	7955	9281	3.57	0.00E+00	transcription_start_site	+	14279	14279	-5661	23839	AN5968
599	CONTIG102	29181	29537	2.31	6.61E-03	transcription_start_site	-	28746	28746	-613	23857	AN5973
599	CONTIG102	29181	29537	2.31	6.61E-03	transcription_start_site	-	28640	28640	-719	23858	AN5973
599	CONTIG102	29181	29537	2.31	6.61E-03	transcription_start_site	-	28445	28445	-914	23859	AN5973
599	CONTIG102	29181	29537	2.31	6.61E-03	transcription_start_site	-	27623	27623	-1736	23860	AN5973
599	CONTIG102	29181	29537	2.31	6.61E-03	transcription_start_site	-	27157	27157	-2202	23861	AN5973
599	CONTIG102	29181	29537	2.31	6.61E-03	transcription_start_site	-	26733	26733	-2626	23862	AN5973
888	CONTIG102	34726	35242	2.01	9.21E-03	transcription_start_site	-	34330	34330	-654	23863	AN5975
1908	CONTIG102	53871	54215	1.41	1.25E-01	transcription_start_site	-	50498	50498	-3545	23876	AN5980
1908	CONTIG102	53871	54215	1.41	1.25E-01	transcription_start_site	+	53308	53308	735	23879	AN5981
1908	CONTIG102	53871	54215	1.41	1.25E-01	transcription_start_site	+	53149	53149	894	23878	AN5981
1908	CONTIG102	53871	54215	1.41	1.25E-01	transcription_start_site	+	56958	56958	-2915	23880	AN5982
1908	CONTIG102	53871	54215	1.41	1.25E-01	transcription_start_site	+	57149	57149	-3106	23881	AN5982
1908	CONTIG102	53871	54215	1.41	1.25E-01	transcription_start_site	+	57673	57673	-3630	23882	AN5982
1908	CONTIG102	53871	54215	1.41	1.25E-01	transcription_start_site	+	58186	58186	-4143	23883	AN5982

2045	CONTIG102	76584	76943	1.36	1.28E-01	transcription_start_site	-	73982	73982	-2781	23894	AN5987
600	CONTIG102	79444	79793	2.31	6.61E-03	transcription_start_site	-	78551	78551	-1067	23896	AN5988
2046	CONTIG102	81394	81811	1.36	1.44E-01	transcription_start_site	-	78551	78551	-3051	23896	AN5988
600	CONTIG102	79444	79793	2.31	6.61E-03	transcription_start_site	+	81481	81481	-1862	23897	AN5989
600	CONTIG102	79444	79793	2.31	6.61E-03	transcription_start_site	+	81809	81809	-2190	23898	AN5989
2045	CONTIG102	76584	76943	1.36	1.28E-01	transcription_start_site	+	81481	81481	-4717	23897	AN5989
2045	CONTIG102	76584	76943	1.36	1.28E-01	transcription_start_site	+	81809	81809	-5045	23898	AN5989
2046	CONTIG102	81394	81811	1.36	1.44E-01	transcription_start_site	+	81481	81481	121	23897	AN5989
2046	CONTIG102	81394	81811	1.36	1.44E-01	transcription_start_site	+	81809	81809	-206	23898	AN5989
1007	CONTIG102	120092	120366	1.91	2.62E-02	transcription_start_site	-	115789	115789	-4440	23935	AN6002
1007	CONTIG102	120092	120366	1.91	2.62E-02	transcription_start_site	-	118398	118398	-1831	23936	AN6003
1007	CONTIG102	120092	120366	1.91	2.62E-02	transcription_start_site	-	118224	118224	-2005	23937	AN6003
1681	CONTIG102	122033	122922	1.51	9.24E-02	transcription_start_site	-	118398	118398	-4079	23936	AN6003
1681	CONTIG102	122033	122922	1.51	9.24E-02	transcription_start_site	-	118224	118224	-4253	23937	AN6003
1007	CONTIG102	120092	120366	1.91	2.62E-02	transcription_start_site	+	120404	120404	-175	23938	AN6004
1007	CONTIG102	120092	120366	1.91	2.62E-02	transcription_start_site	+	120537	120537	-308	23939	AN6004
1007	CONTIG102	120092	120366	1.91	2.62E-02	transcription_start_site	+	120687	120687	-458	23940	AN6004
1681	CONTIG102	122033	122922	1.51	9.24E-02	transcription_start_site	-	122099	122099	-378	23941	AN6005
1681	CONTIG102	122033	122922	1.51	9.24E-02	transcription_start_site	-	123263	123263	785	23949	AN6006
1681	CONTIG102	122033	122922	1.51	9.24E-02	transcription_start_site	-	123433	123433	955	23948	AN6006
1681	CONTIG102	122033	122922	1.51	9.24E-02	transcription_start_site	-	123656	123656	1178	23947	AN6006
1672	CONTIG103	5101	5390	1.51	5.22E-02	transcription_start_site	-	3905	3905	-1340	23958	AN6009
1672	CONTIG103	5101	5390	1.51	5.22E-02	transcription_start_site	-	3810	3810	-1435	23959	AN6009
1672	CONTIG103	5101	5390	1.51	5.22E-02	transcription_start_site	-	3684	3684	-1561	23960	AN6009
1672	CONTIG103	5101	5390	1.51	5.22E-02	transcription_start_site	-	3157	3157	-2088	23961	AN6009
1672	CONTIG103	5101	5390	1.51	5.22E-02	transcription_start_site	-	1517	1517	-3728	23962	AN6009
1672	CONTIG103	5101	5390	1.51	5.22E-02	transcription_start_site	+	5484	5484	-238	23963	AN6010
1672	CONTIG103	5101	5390	1.51	5.22E-02	transcription_start_site	+	5569	5569	-323	23964	AN6010
1672	CONTIG103	5101	5390	1.51	5.22E-02	transcription_start_site	+	5815	5815	-569	23965	AN6010
1372	CONTIG103	11557	11831	1.65	3.08E-02	transcription_start_site	-	9287	9287	-2407	23966	AN6011
1372	CONTIG103	11557	11831	1.65	3.08E-02	transcription_start_site	-	9141	9141	-2553	23967	AN6011
2737	CONTIG103	9001	9434	1.11	1.93E-01	transcription_start_site	-	9287	9287	69	23966	AN6011
2737	CONTIG103	9001	9434	1.11	1.93E-01	transcription_start_site	-	9141	9141	-76	23967	AN6011
1372	CONTIG103	11557	11831	1.65	3.08E-02	transcription_start_site	+	11310	11310	384	23969	AN6012
1372	CONTIG103	11557	11831	1.65	3.08E-02	transcription_start_site	+	10852	10852	842	23968	AN6012
2737	CONTIG103	9001	9434	1.11	1.93E-01	transcription_start_site	+	10852	10852	-1634	23968	AN6012
2737	CONTIG103	9001	9434	1.11	1.93E-01	transcription_start_site	+	11310	11310	-2092	23969	AN6012
759	CONTIG103	14417	14691	2.14	4.61E-03	transcription_start_site	-	13763	13763	-791	23970	AN6013
759	CONTIG103	14417	14691	2.14	4.61E-03	transcription_start_site	-	13629	13629	-925	23971	AN6013
759	CONTIG103	14417	14691	2.14	4.61E-03	transcription_start_site	-	13405	13405	-1149	23972	AN6013
759	CONTIG103	14417	14691	2.14	4.61E-03	transcription_start_site	+	14994	14994	-440	23973	AN6014
759	CONTIG103	14417	14691	2.14	4.61E-03	transcription_start_site	+	15487	15487	-933	23974	AN6014
759	CONTIG103	14417	14691	2.14	4.61E-03	transcription_start_site	+	15585	15585	-1031	23975	AN6014
759	CONTIG103	14417	14691	2.14	4.61E-03	transcription_start_site	+	16029	16029	-1475	23976	AN6014
759	CONTIG103	14417	14691	2.14	4.61E-03	transcription_start_site	+	17226	17226	-2672	23977	AN6014
1372	CONTIG103	11557	11831	1.65	3.08E-02	transcription_start_site	+	14994	14994	-3300	23973	AN6014
1372	CONTIG103	11557	11831	1.65	3.08E-02	transcription_start_site	+	15487	15487	-3793	23974	AN6014
1372	CONTIG103	11557	11831	1.65	3.08E-02	transcription_start_site	+	15585	15585	-3891	23975	AN6014
1372	CONTIG103	11557	11831	1.65	3.08E-02	transcription_start_site	+	16029	16029	-4335	23976	AN6014
140	CONTIG103	27531	27875	3.07	0.00E+00	transcription_start_site	-	23366	23366	-4337	23978	AN6015
140	CONTIG103	27531	27875	3.07	0.00E+00	transcription_start_site	-	23259	23259	-4444	23979	AN6015
140	CONTIG103	27531	27875	3.07	0.00E+00	transcription_start_site	-	23170	23170	-4533	23980	AN6015
140	CONTIG103	27531	27875	3.07	0.00E+00	transcription_start_site	+	29776	29776	-2073	23981	AN6016
140	CONTIG103	27531	27875	3.07	0.00E+00	transcription_start_site	+	29931	29931	-2228	23982	AN6016
140	CONTIG103	27531	27875	3.07	0.00E+00	transcription_start_site	+	30019	30019	-2316	23983	AN6016
140	CONTIG103	27531	27875	3.07	0.00E+00	transcription_start_site	+	30142	30142	-2439	23984	AN6016
140	CONTIG103	27531	27875	3.07	0.00E+00	transcription_start_site	+	30251	30251	-2548	23985	AN6016

140	CONTIG103	27531	27875	3.07	0.00E+00	transcription_start_site	+	30537	30537	-2834	23986	AN6016
1859	CONTIG103	49596	50162	1.43	5.05E-02	transcription_start_site	-	49788	49788	-91	24002	AN6022
1859	CONTIG103	49596	50162	1.43	5.05E-02	transcription_start_site	-	50109	50109	230	24001	AN6022
1859	CONTIG103	49596	50162	1.43	5.05E-02	transcription_start_site	-	50447	50447	568	24000	AN6022
1859	CONTIG103	49596	50162	1.43	5.05E-02	transcription_start_site	-	50649	50649	770	23999	AN6022
1859	CONTIG103	49596	50162	1.43	5.05E-02	transcription_start_site	-	50969	50969	1090	23998	AN6022
1774	CONTIG103	61143	61402	1.47	5.88E-02	transcription_start_site	-	59389	59389	-1883	24010	AN6025
1774	CONTIG103	61143	61402	1.47	5.88E-02	transcription_start_site	-	59293	59293	-1979	24011	AN6025
1774	CONTIG103	61143	61402	1.47	5.88E-02	transcription_start_site	-	61401	61401	128	24013	AN6026
1774	CONTIG103	61143	61402	1.47	5.88E-02	transcription_start_site	-	61713	61713	440	24012	AN6026
1774	CONTIG103	61143	61402	1.47	5.88E-02	transcription_start_site	-	60582	60582	-690	24014	AN6026
1774	CONTIG103	61143	61402	1.47	5.88E-02	transcription_start_site	-	59824	59824	-1448	24015	AN6026
1774	CONTIG103	61143	61402	1.47	5.88E-02	transcription_start_site	+	65494	65494	-4221	24025	AN6029
1774	CONTIG103	61143	61402	1.47	5.88E-02	transcription_start_site	+	65958	65958	-4685	24026	AN6029
386	CONTIG104	39989	40628	2.62	0.00E+00	transcription_start_site	-	36338	36338	-3970	24093	AN6048
386	CONTIG104	39989	40628	2.62	0.00E+00	transcription_start_site	-	35917	35917	-4391	24094	AN6048
2538	CONTIG104	35326	36361	1.19	1.66E-01	transcription_start_site	-	35917	35917	73	24094	AN6048
2538	CONTIG104	35326	36361	1.19	1.66E-01	transcription_start_site	-	36338	36338	494	24093	AN6048
2538	CONTIG104	35326	36361	1.19	1.66E-01	transcription_start_site	-	34881	34881	-962	24095	AN6048
386	CONTIG104	39989	40628	2.62	0.00E+00	transcription_start_site	-	39898	39898	-410	24096	AN6049
386	CONTIG104	39989	40628	2.62	0.00E+00	transcription_start_site	-	39504	39504	-804	24097	AN6049
386	CONTIG104	39989	40628	2.62	0.00E+00	transcription_start_site	-	39025	39025	-1283	24098	AN6049
386	CONTIG104	39989	40628	2.62	0.00E+00	transcription_start_site	-	38436	38436	-1872	24099	AN6049
2379	CONTIG104	43969	44396	1.24	1.28E-01	transcription_start_site	-	39898	39898	-4284	24096	AN6049
2379	CONTIG104	43969	44396	1.24	1.28E-01	transcription_start_site	-	39504	39504	-4678	24097	AN6049
2379	CONTIG104	43969	44396	1.24	1.28E-01	transcription_start_site	-	39025	39025	-5157	24098	AN6049
386	CONTIG104	39989	40628	2.62	0.00E+00	transcription_start_site	+	44840	44840	-4531	24100	AN6050
386	CONTIG104	39989	40628	2.62	0.00E+00	transcription_start_site	+	45067	45067	-4758	24101	AN6050
386	CONTIG104	39989	40628	2.62	0.00E+00	transcription_start_site	+	45452	45452	-5143	24102	AN6050
2379	CONTIG104	43969	44396	1.24	1.28E-01	transcription_start_site	+	44840	44840	-657	24100	AN6050
2379	CONTIG104	43969	44396	1.24	1.28E-01	transcription_start_site	+	45067	45067	-884	24101	AN6050
2379	CONTIG104	43969	44396	1.24	1.28E-01	transcription_start_site	+	45452	45452	-1269	24102	AN6050
2379	CONTIG104	43969	44396	1.24	1.28E-01	transcription_start_site	+	46121	46121	-1938	24103	AN6050
2379	CONTIG104	43969	44396	1.24	1.28E-01	transcription_start_site	+	47688	47688	-3505	24104	AN6051
2379	CONTIG104	43969	44396	1.24	1.28E-01	transcription_start_site	+	49187	49187	-5004	24105	AN6051
1216	CONTIG104	52359	52933	1.75	2.62E-02	transcription_start_site	+	51665	51665	981	24110	AN6052
1216	CONTIG104	52359	52933	1.75	2.62E-02	transcription_start_site	+	51585	51585	1061	24109	AN6052
1216	CONTIG104	52359	52933	1.75	2.62E-02	transcription_start_site	+	53484	53484	-838	24111	AN6053
1556	CONTIG104	57379	57669	1.56	5.22E-02	transcription_start_site	-	57599	57599	75	24112	AN6054
1556	CONTIG104	57379	57669	1.56	5.22E-02	transcription_start_site	-	56961	56961	-563	24113	AN6054
2539	CONTIG104	55734	56003	1.19	1.66E-01	transcription_start_site	-	56961	56961	1092	24113	AN6054
1556	CONTIG104	57379	57669	1.56	5.22E-02	transcription_start_site	+	58122	58122	-598	24114	AN6055
1556	CONTIG104	57379	57669	1.56	5.22E-02	transcription_start_site	+	58395	58395	-871	24115	AN6055
1556	CONTIG104	57379	57669	1.56	5.22E-02	transcription_start_site	+	58505	58505	-981	24116	AN6055
1556	CONTIG104	57379	57669	1.56	5.22E-02	transcription_start_site	+	59195	59195	-1671	24117	AN6055
1556	CONTIG104	57379	57669	1.56	5.22E-02	transcription_start_site	+	59815	59815	-2291	24118	AN6055
2539	CONTIG104	55734	56003	1.19	1.66E-01	transcription_start_site	+	58122	58122	-2253	24114	AN6055
2539	CONTIG104	55734	56003	1.19	1.66E-01	transcription_start_site	+	58395	58395	-2526	24115	AN6055
2539	CONTIG104	55734	56003	1.19	1.66E-01	transcription_start_site	+	58505	58505	-2636	24116	AN6055
2539	CONTIG104	55734	56003	1.19	1.66E-01	transcription_start_site	+	59195	59195	-3326	24117	AN6055
2539	CONTIG104	55734	56003	1.19	1.66E-01	transcription_start_site	+	59815	59815	-3946	24118	AN6055
438	CONTIG104	68269	68768	2.53	0.00E+00	transcription_start_site	-	66406	66406	-2112	24127	AN6058
438	CONTIG104	68269	68768	2.53	0.00E+00	transcription_start_site	-	66246	66246	-2272	24128	AN6058
470	CONTIG104	71271	71681	2.48	1.22E-03	transcription_start_site	-	66406	66406	-5070	24127	AN6058
1642	CONTIG104	67669	68233	1.52	5.88E-02	transcription_start_site	-	66406	66406	-1545	24127	AN6058
1642	CONTIG104	67669	68233	1.52	5.88E-02	transcription_start_site	-	66246	66246	-1705	24128	AN6058
1772	CONTIG104	70291	70925	1.47	5.05E-02	transcription_start_site	-	66406	66406	-4202	24127	AN6058

1772	CONTIG104	70291	70925	1.47	5.05E-02	transcription_start_site	-	66246	66246	-4362	24128	AN6058
438	CONTIG104	68269	68768	2.53	0.00E+00	transcription_start_site	+	67375	67375	1143	24130	AN6059
438	CONTIG104	68269	68768	2.53	0.00E+00	transcription_start_site	+	67284	67284	1234	24129	AN6059
1642	CONTIG104	67669	68233	1.52	5.88E-02	transcription_start_site	+	67375	67375	576	24130	AN6059
1642	CONTIG104	67669	68233	1.52	5.88E-02	transcription_start_site	+	67284	67284	667	24129	AN6059
438	CONTIG104	68269	68768	2.53	0.00E+00	transcription_start_site	+	70038	70038	-1519	24131	AN6060
438	CONTIG104	68269	68768	2.53	0.00E+00	transcription_start_site	+	70809	70809	-2290	24132	AN6060
470	CONTIG104	71271	71681	2.48	1.22E-03	transcription_start_site	+	70809	70809	667	24132	AN6060
470	CONTIG104	71271	71681	2.48	1.22E-03	transcription_start_site	+	74156	74156	-2680	24133	AN6060
470	CONTIG104	71271	71681	2.48	1.22E-03	transcription_start_site	+	74290	74290	-2814	24134	AN6060
470	CONTIG104	71271	71681	2.48	1.22E-03	transcription_start_site	+	74731	74731	-3255	24135	AN6060
1642	CONTIG104	67669	68233	1.52	5.88E-02	transcription_start_site	+	70038	70038	-2087	24131	AN6060
1642	CONTIG104	67669	68233	1.52	5.88E-02	transcription_start_site	+	70809	70809	-2858	24132	AN6060
1772	CONTIG104	70291	70925	1.47	5.05E-02	transcription_start_site	+	70809	70809	-201	24132	AN6060
1772	CONTIG104	70291	70925	1.47	5.05E-02	transcription_start_site	+	70038	70038	570	24131	AN6060
1772	CONTIG104	70291	70925	1.47	5.05E-02	transcription_start_site	+	74156	74156	-3548	24133	AN6060
1772	CONTIG104	70291	70925	1.47	5.05E-02	transcription_start_site	+	74290	74290	-3682	24134	AN6060
1772	CONTIG104	70291	70925	1.47	5.05E-02	transcription_start_site	+	74731	74731	-4123	24135	AN6060
2644	CONTIG104	74332	74611	1.15	1.93E-01	transcription_start_site	+	74290	74290	181	24134	AN6060
2644	CONTIG104	74332	74611	1.15	1.93E-01	transcription_start_site	+	74731	74731	-259	24135	AN6060
2644	CONTIG104	74332	74611	1.15	1.93E-01	transcription_start_site	+	74156	74156	315	24133	AN6060
1088	CONTIG104	81002	81281	1.84	1.84E-02	transcription_start_site	-	77798	77798	-3343	24136	AN6061
1088	CONTIG104	81002	81281	1.84	1.84E-02	transcription_start_site	-	77472	77472	-3669	24137	AN6061
1088	CONTIG104	81002	81281	1.84	1.84E-02	transcription_start_site	-	77029	77029	-4112	24138	AN6061
1088	CONTIG104	81002	81281	1.84	1.84E-02	transcription_start_site	-	81107	81107	-34	24139	AN6062
1088	CONTIG104	81002	81281	1.84	1.84E-02	transcription_start_site	-	80936	80936	-205	24140	AN6062
1088	CONTIG104	81002	81281	1.84	1.84E-02	transcription_start_site	-	80641	80641	-500	24141	AN6062
1088	CONTIG104	81002	81281	1.84	1.84E-02	transcription_start_site	-	80285	80285	-856	24142	AN6062
1088	CONTIG104	81002	81281	1.84	1.84E-02	transcription_start_site	-	79998	79998	-1143	24143	AN6062
1088	CONTIG104	81002	81281	1.84	1.84E-02	transcription_start_site	-	79802	79802	-1339	24144	AN6062
1088	CONTIG104	81002	81281	1.84	1.84E-02	transcription_start_site	-	79572	79572	-1569	24145	AN6062
1088	CONTIG104	81002	81281	1.84	1.84E-02	transcription_start_site	-	79142	79142	-1999	24146	AN6062
1088	CONTIG104	81002	81281	1.84	1.84E-02	transcription_start_site	+	83589	83589	-2447	24147	AN6063
1088	CONTIG104	81002	81281	1.84	1.84E-02	transcription_start_site	+	83793	83793	-2651	24148	AN6063
1088	CONTIG104	81002	81281	1.84	1.84E-02	transcription_start_site	+	84274	84274	-3132	24149	AN6063
1088	CONTIG104	81002	81281	1.84	1.84E-02	transcription_start_site	+	85077	85077	-3935	24150	AN6063
1088	CONTIG104	81002	81281	1.84	1.84E-02	transcription_start_site	+	85559	85559	-4417	24151	AN6064
1088	CONTIG104	81002	81281	1.84	1.84E-02	transcription_start_site	+	85735	85735	-4593	24152	AN6064
1088	CONTIG104	81002	81281	1.84	1.84E-02	transcription_start_site	+	85834	85834	-4692	24153	AN6064
1088	CONTIG104	81002	81281	1.84	1.84E-02	transcription_start_site	+	85967	85967	-4825	24154	AN6064
1775	CONTIG104	107927	108276	1.47	6.84E-02	transcription_start_site	-	106120	106120	-1981	24168	AN6070
1775	CONTIG104	107927	108276	1.47	6.84E-02	transcription_start_site	-	106059	106059	-2042	24169	AN6070
1775	CONTIG104	107927	108276	1.47	6.84E-02	transcription_start_site	-	105310	105310	-2791	24170	AN6070
1775	CONTIG104	107927	108276	1.47	6.84E-02	transcription_start_site	-	105089	105089	-3012	24171	AN6070
1775	CONTIG104	107927	108276	1.47	6.84E-02	transcription_start_site	-	104808	104808	-3293	24172	AN6070
1775	CONTIG104	107927	108276	1.47	6.84E-02	transcription_start_site	+	108979	108979	-877	24173	AN6071
1775	CONTIG104	107927	108276	1.47	6.84E-02	transcription_start_site	+	109207	109207	-1105	24174	AN6071
2645	CONTIG104	116927	117201	1.15	1.93E-01	transcription_start_site	-	114509	114509	-2555	24175	AN6072
2645	CONTIG104	116927	117201	1.15	1.93E-01	transcription_start_site	-	116211	116211	-853	24176	AN6073
2645	CONTIG104	116927	117201	1.15	1.93E-01	transcription_start_site	-	115969	115969	-1095	24177	AN6073
2645	CONTIG104	116927	117201	1.15	1.93E-01	transcription_start_site	-	115841	115841	-1223	24178	AN6073
2645	CONTIG104	116927	117201	1.15	1.93E-01	transcription_start_site	-	115415	115415	-1649	24179	AN6073
2645	CONTIG104	116927	117201	1.15	1.93E-01	transcription_start_site	+	116664	116664	400	24181	AN6074
2645	CONTIG104	116927	117201	1.15	1.93E-01	transcription_start_site	+	116550	116550	514	24180	AN6074
2645	CONTIG104	116927	117201	1.15	1.93E-01	transcription_start_site	+	118816	118816	-1752	24182	AN6075
2645	CONTIG104	116927	117201	1.15	1.93E-01	transcription_start_site	+	119024	119024	-1960	24183	AN6075
2645	CONTIG104	116927	117201	1.15	1.93E-01	transcription_start_site	+	119178	119178	-2114	24184	AN6075

2645	CONTIG104	116927	117201	1.15	1.93E-01	transcription_start_site	+	121538	121538	-4474	24185	AN6076
1156	CONTIG104	149414	149753	1.79	1.06E-02	transcription_start_site	-	148413	148413	-1170	24205	AN6084
1156	CONTIG104	149414	149753	1.79	1.06E-02	transcription_start_site	-	149945	149945	361	24207	AN6085
1156	CONTIG104	149414	149753	1.79	1.06E-02	transcription_start_site	+	154722	154722	-5138	24210	AN6087
2540	CONTIG104	161794	162203	1.19	1.66E-01	transcription_start_site	-	163155	163155	1156	24224	AN6089
2540	CONTIG104	161794	162203	1.19	1.66E-01	transcription_start_site	+	166734	166734	-4735	24225	AN6090
2540	CONTIG104	161794	162203	1.19	1.66E-01	transcription_start_site	+	166875	166875	-4876	24226	AN6090
2540	CONTIG104	161794	162203	1.19	1.66E-01	transcription_start_site	+	167025	167025	-5026	24227	AN6090
2541	CONTIG104	169521	169787	1.19	1.66E-01	transcription_start_site	+	170432	170432	-778	24234	AN6091
2541	CONTIG104	169521	169787	1.19	1.66E-01	transcription_start_site	+	171234	171234	-1580	24235	AN6091
2541	CONTIG104	169521	169787	1.19	1.66E-01	transcription_start_site	+	171549	171549	-1895	24236	AN6091
2541	CONTIG104	169521	169787	1.19	1.66E-01	transcription_start_site	+	171737	171737	-2083	24237	AN6091
2541	CONTIG104	169521	169787	1.19	1.66E-01	transcription_start_site	+	171893	171893	-2239	24238	AN6091
1047	CONTIG104	182481	182762	1.88	1.57E-02	transcription_start_site	-	182443	182443	-178	24248	AN6095
1047	CONTIG104	182481	182762	1.88	1.57E-02	transcription_start_site	-	181846	181846	-775	24249	AN6095
1047	CONTIG104	182481	182762	1.88	1.57E-02	transcription_start_site	-	181650	181650	-971	24250	AN6095
1047	CONTIG104	182481	182762	1.88	1.57E-02	transcription_start_site	-	181414	181414	-1207	24251	AN6095
1047	CONTIG104	182481	182762	1.88	1.57E-02	transcription_start_site	-	181014	181014	-1607	24252	AN6095
2536	CONTIG104	182928	183819	1.19	1.59E-01	transcription_start_site	-	182443	182443	-930	24248	AN6095
2536	CONTIG104	182928	183819	1.19	1.59E-01	transcription_start_site	-	181846	181846	-1527	24249	AN6095
2536	CONTIG104	182928	183819	1.19	1.59E-01	transcription_start_site	-	181650	181650	-1723	24250	AN6095
2536	CONTIG104	182928	183819	1.19	1.59E-01	transcription_start_site	-	181414	181414	-1959	24251	AN6095
2536	CONTIG104	182928	183819	1.19	1.59E-01	transcription_start_site	-	181014	181014	-2359	24252	AN6095
1047	CONTIG104	182481	182762	1.88	1.57E-02	transcription_start_site	+	186260	186260	-3638	24257	AN6097
1047	CONTIG104	182481	182762	1.88	1.57E-02	transcription_start_site	+	186548	186548	-3926	24258	AN6097
1047	CONTIG104	182481	182762	1.88	1.57E-02	transcription_start_site	+	186726	186726	-4104	24259	AN6097
1047	CONTIG104	182481	182762	1.88	1.57E-02	transcription_start_site	+	187069	187069	-4447	24260	AN6097
2536	CONTIG104	182928	183819	1.19	1.59E-01	transcription_start_site	+	186260	186260	-2886	24257	AN6097
2536	CONTIG104	182928	183819	1.19	1.59E-01	transcription_start_site	+	186548	186548	-3174	24258	AN6097
2536	CONTIG104	182928	183819	1.19	1.59E-01	transcription_start_site	+	186726	186726	-3352	24259	AN6097
2536	CONTIG104	182928	183819	1.19	1.59E-01	transcription_start_site	+	187069	187069	-3695	24260	AN6097
2536	CONTIG104	182928	183819	1.19	1.59E-01	transcription_start_site	+	187976	187976	-4602	24261	AN6098
2536	CONTIG104	182928	183819	1.19	1.59E-01	transcription_start_site	+	188470	188470	-5096	24262	AN6098
55	CONTIG104	208882	209462	3.4	0.00E+00	transcription_start_site	-	204490	204490	-4682	24280	AN6103
55	CONTIG104	208882	209462	3.4	0.00E+00	transcription_start_site	-	204312	204312	-4860	24281	AN6103
364	CONTIG104	207167	207437	2.66	5.64E-04	transcription_start_site	-	204490	204490	-2812	24280	AN6103
364	CONTIG104	207167	207437	2.66	5.64E-04	transcription_start_site	-	204312	204312	-2990	24281	AN6103
364	CONTIG104	207167	207437	2.66	5.64E-04	transcription_start_site	-	203569	203569	-3733	24282	AN6103
364	CONTIG104	207167	207437	2.66	5.64E-04	transcription_start_site	-	203094	203094	-4208	24283	AN6103
364	CONTIG104	207167	207437	2.66	5.64E-04	transcription_start_site	-	202943	202943	-4359	24284	AN6103
364	CONTIG104	207167	207437	2.66	5.64E-04	transcription_start_site	-	202880	202880	-4422	24285	AN6103
55	CONTIG104	208882	209462	3.4	0.00E+00	transcription_start_site	+	209866	209866	-694	24286	AN6104
364	CONTIG104	207167	207437	2.66	5.64E-04	transcription_start_site	+	209866	209866	-2564	24286	AN6104
1874	CONTIG104	210303	211192	1.42	5.52E-02	transcription_start_site	+	209866	209866	881	24286	AN6104
55	CONTIG104	208882	209462	3.4	0.00E+00	transcription_start_site	+	214001	214001	-4829	24287	AN6105
55	CONTIG104	208882	209462	3.4	0.00E+00	transcription_start_site	+	214418	214418	-5246	24288	AN6105
978	CONTIG104	213828	214112	1.93	1.30E-02	transcription_start_site	+	214001	214001	-31	24287	AN6105
978	CONTIG104	213828	214112	1.93	1.30E-02	transcription_start_site	+	214418	214418	-448	24288	AN6105
1874	CONTIG104	210303	211192	1.42	5.52E-02	transcription_start_site	+	214001	214001	-3253	24287	AN6105
1874	CONTIG104	210303	211192	1.42	5.52E-02	transcription_start_site	+	214418	214418	-3670	24288	AN6105
389	CONTIG104	221869	222288	2.62	8.69E-04	transcription_start_site	+	222253	222253	-174	24293	AN6107
389	CONTIG104	221869	222288	2.62	8.69E-04	transcription_start_site	+	224884	224884	-2805	24294	AN6107
441	CONTIG104	248416	248765	2.53	1.01E-03	transcription_start_site	+	248999	248999	-408	24327	AN6118
441	CONTIG104	248416	248765	2.53	1.01E-03	transcription_start_site	+	249194	249194	-603	24328	AN6118
441	CONTIG104	248416	248765	2.53	1.01E-03	transcription_start_site	+	249415	249415	-824	24329	AN6118
441	CONTIG104	248416	248765	2.53	1.01E-03	transcription_start_site	+	249638	249638	-1047	24330	AN6118
1643	CONTIG104	249545	250404	1.52	5.88E-02	transcription_start_site	+	249638	249638	336	24330	AN6118

1643	CONTIG104	249545	250404	1.52	5.88E-02	transcription_start_site	+	249415	249415	559	24329	AN6118
1643	CONTIG104	249545	250404	1.52	5.88E-02	transcription_start_site	+	249194	249194	780	24328	AN6118
1643	CONTIG104	249545	250404	1.52	5.88E-02	transcription_start_site	+	248999	248999	975	24327	AN6118
2638	CONTIG104	250520	251154	1.15	1.79E-01	transcription_start_site	+	249638	249638	1199	24330	AN6118
788	CONTIG104	254120	254534	2.11	2.49E-03	transcription_start_site	-	253419	253419	-908	24331	AN6119
788	CONTIG104	254120	254534	2.11	2.49E-03	transcription_start_site	-	252973	252973	-1354	24332	AN6119
788	CONTIG104	254120	254534	2.11	2.49E-03	transcription_start_site	+	255057	255057	-730	24333	AN6120
788	CONTIG104	254120	254534	2.11	2.49E-03	transcription_start_site	+	255524	255524	-1197	24334	AN6120
788	CONTIG104	254120	254534	2.11	2.49E-03	transcription_start_site	+	256821	256821	-2494	24335	AN6120
1643	CONTIG104	249545	250404	1.52	5.88E-02	transcription_start_site	+	255057	255057	-5082	24333	AN6120
2638	CONTIG104	250520	251154	1.15	1.79E-01	transcription_start_site	+	255057	255057	-4220	24333	AN6120
2638	CONTIG104	250520	251154	1.15	1.79E-01	transcription_start_site	+	255524	255524	-4687	24334	AN6120
225	CONTIG104	270845	271119	2.89	0.00E+00	transcription_start_site	-	267342	267342	-3640	24341	AN6123
225	CONTIG104	270845	271119	2.89	0.00E+00	transcription_start_site	-	269789	269789	-1193	24342	AN6124
225	CONTIG104	270845	271119	2.89	0.00E+00	transcription_start_site	-	269613	269613	-1369	24343	AN6124
225	CONTIG104	270845	271119	2.89	0.00E+00	transcription_start_site	-	269136	269136	-1846	24344	AN6124
225	CONTIG104	270845	271119	2.89	0.00E+00	transcription_start_site	-	268957	268957	-2025	24345	AN6124
225	CONTIG104	270845	271119	2.89	0.00E+00	transcription_start_site	+	271321	271321	-339	24346	AN6125
225	CONTIG104	270845	271119	2.89	0.00E+00	transcription_start_site	+	271619	271619	-637	24347	AN6125
1089	CONTIG104	274876	275766	1.84	1.84E-02	transcription_start_site	+	276453	276453	-1132	24348	AN6126
1089	CONTIG104	274876	275766	1.84	1.84E-02	transcription_start_site	+	276693	276693	-1372	24349	AN6126
1089	CONTIG104	274876	275766	1.84	1.84E-02	transcription_start_site	+	276934	276934	-1613	24350	AN6126
1607	CONTIG105	16068	16412	1.54	9.24E-02	transcription_start_site	-	16162	16162	-78	24370	AN6132
1607	CONTIG105	16068	16412	1.54	9.24E-02	transcription_start_site	-	12682	12682	-3558	24371	AN6132
1841	CONTIG105	91734	92243	1.44	1.25E-01	transcription_start_site	-	87385	87385	-4603	24438	AN6155
1841	CONTIG105	91734	92243	1.44	1.25E-01	transcription_start_site	-	86745	86745	-5243	24439	AN6155
1841	CONTIG105	91734	92243	1.44	1.25E-01	transcription_start_site	-	90268	90268	-1720	24440	AN6156
1841	CONTIG105	91734	92243	1.44	1.25E-01	transcription_start_site	-	90025	90025	-1963	24441	AN6156
1842	CONTIG105	92554	93068	1.44	1.25E-01	transcription_start_site	-	90268	90268	-2543	24440	AN6156
1842	CONTIG105	92554	93068	1.44	1.25E-01	transcription_start_site	-	90025	90025	-2786	24441	AN6156
1016	CONTIG105	95789	96058	1.9	3.08E-02	transcription_start_site	-	92549	92549	-3374	24442	AN6157
1016	CONTIG105	95789	96058	1.9	3.08E-02	transcription_start_site	-	92333	92333	-3590	24443	AN6157
1841	CONTIG105	91734	92243	1.44	1.25E-01	transcription_start_site	-	92333	92333	344	24443	AN6157
1841	CONTIG105	91734	92243	1.44	1.25E-01	transcription_start_site	-	92549	92549	560	24442	AN6157
1842	CONTIG105	92554	93068	1.44	1.25E-01	transcription_start_site	-	92549	92549	-262	24442	AN6157
1842	CONTIG105	92554	93068	1.44	1.25E-01	transcription_start_site	-	92333	92333	-478	24443	AN6157
1016	CONTIG105	95789	96058	1.9	3.08E-02	transcription_start_site	-	93418	93418	-2505	24444	AN6158
1016	CONTIG105	95789	96058	1.9	3.08E-02	transcription_start_site	-	93283	93283	-2640	24445	AN6158
1016	CONTIG105	95789	96058	1.9	3.08E-02	transcription_start_site	-	93107	93107	-2816	24446	AN6158
1841	CONTIG105	91734	92243	1.44	1.25E-01	transcription_start_site	-	93107	93107	1118	24446	AN6158
1842	CONTIG105	92554	93068	1.44	1.25E-01	transcription_start_site	-	93107	93107	296	24446	AN6158
1842	CONTIG105	92554	93068	1.44	1.25E-01	transcription_start_site	-	93283	93283	472	24445	AN6158
1842	CONTIG105	92554	93068	1.44	1.25E-01	transcription_start_site	-	93418	93418	607	24444	AN6158
1016	CONTIG105	95789	96058	1.9	3.08E-02	transcription_start_site	-	95307	95307	-616	24447	AN6159
1016	CONTIG105	95789	96058	1.9	3.08E-02	transcription_start_site	-	95120	95120	-803	24448	AN6159
1016	CONTIG105	95789	96058	1.9	3.08E-02	transcription_start_site	-	95028	95028	-895	24449	AN6159
1016	CONTIG105	95789	96058	1.9	3.08E-02	transcription_start_site	-	93987	93987	-1936	24450	AN6159
1842	CONTIG105	92554	93068	1.44	1.25E-01	transcription_start_site	-	93987	93987	1176	24450	AN6159
1016	CONTIG105	95789	96058	1.9	3.08E-02	transcription_start_site	+	96169	96169	-245	24451	AN6160
1016	CONTIG105	95789	96058	1.9	3.08E-02	transcription_start_site	+	96298	96298	-374	24452	AN6160
1016	CONTIG105	95789	96058	1.9	3.08E-02	transcription_start_site	+	96471	96471	-547	24453	AN6160
1016	CONTIG105	95789	96058	1.9	3.08E-02	transcription_start_site	+	96798	96798	-874	24454	AN6160
1841	CONTIG105	91734	92243	1.44	1.25E-01	transcription_start_site	+	96169	96169	-4180	24451	AN6160
1841	CONTIG105	91734	92243	1.44	1.25E-01	transcription_start_site	+	96298	96298	-4309	24452	AN6160
1841	CONTIG105	91734	92243	1.44	1.25E-01	transcription_start_site	+	96471	96471	-4482	24453	AN6160
1841	CONTIG105	91734	92243	1.44	1.25E-01	transcription_start_site	+	96798	96798	-4809	24454	AN6160
1842	CONTIG105	92554	93068	1.44	1.25E-01	transcription_start_site	+	96169	96169	-3358	24451	AN6160

1842	CONTIG105	92554	93068	1.44	1.25E-01	transcription_start_site	+	96298	96298	-3487	24452 AN6160
1842	CONTIG105	92554	93068	1.44	1.25E-01	transcription_start_site	+	96471	96471	-3660	24453 AN6160
1842	CONTIG105	92554	93068	1.44	1.25E-01	transcription_start_site	+	96798	96798	-3987	24454 AN6160
1016	CONTIG105	95789	96058	1.9	3.08E-02	transcription_start_site	+	99106	99106	-3182	24455 AN6161
1016	CONTIG105	95789	96058	1.9	3.08E-02	transcription_start_site	+	99250	99250	-3326	24456 AN6161
1016	CONTIG105	95789	96058	1.9	3.08E-02	transcription_start_site	+	100303	100303	-4379	24457 AN6161
1016	CONTIG105	95789	96058	1.9	3.08E-02	transcription_start_site	+	100804	100804	-4880	24458 AN6161
418	CONTIG105	103426	103860	2.57	0.00E+00	transcription_start_site	-	102504	102504	-1139	24459 AN6162
418	CONTIG105	103426	103860	2.57	0.00E+00	transcription_start_site	-	101624	101624	-2019	24460 AN6162
418	CONTIG105	103426	103860	2.57	0.00E+00	transcription_start_site	-	103673	103673	30	24463 AN6163
418	CONTIG105	103426	103860	2.57	0.00E+00	transcription_start_site	-	103850	103850	207	24462 AN6163
418	CONTIG105	103426	103860	2.57	0.00E+00	transcription_start_site	-	103930	103930	287	24461 AN6163
418	CONTIG105	103426	103860	2.57	0.00E+00	transcription_start_site	+	104758	104758	-1115	24464 AN6164
418	CONTIG105	103426	103860	2.57	0.00E+00	transcription_start_site	+	105442	105442	-1799	24465 AN6164
418	CONTIG105	103426	103860	2.57	0.00E+00	transcription_start_site	+	105650	105650	-2007	24466 AN6164
418	CONTIG105	103426	103860	2.57	0.00E+00	transcription_start_site	+	108430	108430	-4787	24467 AN6164
418	CONTIG105	103426	103860	2.57	0.00E+00	transcription_start_site	+	108647	108647	-5004	24468 AN6164
418	CONTIG105	103426	103860	2.57	0.00E+00	transcription_start_site	+	108856	108856	-5213	24469 AN6164
206	CONTIG105	123920	124404	2.93	0.00E+00	transcription_start_site	-	120204	120204	-3958	24482 AN6167
206	CONTIG105	123920	124404	2.93	0.00E+00	transcription_start_site	-	120033	120033	-4129	24483 AN6167
206	CONTIG105	123920	124404	2.93	0.00E+00	transcription_start_site	-	119463	119463	-4699	24484 AN6167
206	CONTIG105	123920	124404	2.93	0.00E+00	transcription_start_site	-	119155	119155	-5007	24485 AN6167
601	CONTIG105	125035	125479	2.31	7.85E-03	transcription_start_site	-	120204	120204	-5053	24482 AN6167
961	CONTIG105	120610	120884	1.95	2.62E-02	transcription_start_site	-	120204	120204	-543	24482 AN6167
961	CONTIG105	120610	120884	1.95	2.62E-02	transcription_start_site	-	120033	120033	-714	24483 AN6167
961	CONTIG105	120610	120884	1.95	2.62E-02	transcription_start_site	-	119463	119463	-1284	24484 AN6167
961	CONTIG105	120610	120884	1.95	2.62E-02	transcription_start_site	-	119155	119155	-1592	24485 AN6167
961	CONTIG105	120610	120884	1.95	2.62E-02	transcription_start_site	-	118734	118734	-2013	24486 AN6167
206	CONTIG105	123920	124404	2.93	0.00E+00	transcription_start_site	-	123800	123800	-362	24487 AN6168
206	CONTIG105	123920	124404	2.93	0.00E+00	transcription_start_site	-	123491	123491	-671	24488 AN6168
206	CONTIG105	123920	124404	2.93	0.00E+00	transcription_start_site	-	123335	123335	-827	24489 AN6168
206	CONTIG105	123920	124404	2.93	0.00E+00	transcription_start_site	-	123164	123164	-998	24490 AN6168
206	CONTIG105	123920	124404	2.93	0.00E+00	transcription_start_site	-	123024	123024	-1138	24491 AN6168
206	CONTIG105	123920	124404	2.93	0.00E+00	transcription_start_site	-	122613	122613	-1549	24492 AN6168
206	CONTIG105	123920	124404	2.93	0.00E+00	transcription_start_site	-	121445	121445	-2717	24493 AN6168
601	CONTIG105	125035	125479	2.31	7.85E-03	transcription_start_site	-	123800	123800	-1457	24487 AN6168
601	CONTIG105	125035	125479	2.31	7.85E-03	transcription_start_site	-	123491	123491	-1766	24488 AN6168
601	CONTIG105	125035	125479	2.31	7.85E-03	transcription_start_site	-	123335	123335	-1922	24489 AN6168
601	CONTIG105	125035	125479	2.31	7.85E-03	transcription_start_site	-	123164	123164	-2093	24490 AN6168
601	CONTIG105	125035	125479	2.31	7.85E-03	transcription_start_site	-	123024	123024	-2233	24491 AN6168
601	CONTIG105	125035	125479	2.31	7.85E-03	transcription_start_site	-	122613	122613	-2644	24492 AN6168
601	CONTIG105	125035	125479	2.31	7.85E-03	transcription_start_site	-	121445	121445	-3812	24493 AN6168
961	CONTIG105	120610	120884	1.95	2.62E-02	transcription_start_site	-	121445	121445	698	24493 AN6168
1079	CONTIG105	125930	126284	1.85	2.18E-02	transcription_start_site	-	123800	123800	-2307	24487 AN6168
1079	CONTIG105	125930	126284	1.85	2.18E-02	transcription_start_site	-	123491	123491	-2616	24488 AN6168
1079	CONTIG105	125930	126284	1.85	2.18E-02	transcription_start_site	-	123335	123335	-2772	24489 AN6168
1079	CONTIG105	125930	126284	1.85	2.18E-02	transcription_start_site	-	123164	123164	-2943	24490 AN6168
1079	CONTIG105	125930	126284	1.85	2.18E-02	transcription_start_site	-	123024	123024	-3083	24491 AN6168
1079	CONTIG105	125930	126284	1.85	2.18E-02	transcription_start_site	-	122613	122613	-3494	24492 AN6168
1079	CONTIG105	125930	126284	1.85	2.18E-02	transcription_start_site	-	121445	121445	-4662	24493 AN6168
206	CONTIG105	123920	124404	2.93	0.00E+00	transcription_start_site	+	126296	126296	-2134	24494 AN6169
206	CONTIG105	123920	124404	2.93	0.00E+00	transcription_start_site	+	126744	126744	-2582	24495 AN6169
601	CONTIG105	125035	125479	2.31	7.85E-03	transcription_start_site	+	126296	126296	-1039	24494 AN6169
601	CONTIG105	125035	125479	2.31	7.85E-03	transcription_start_site	+	126744	126744	-1487	24495 AN6169
1079	CONTIG105	125930	126284	1.85	2.18E-02	transcription_start_site	+	126296	126296	-189	24494 AN6169
1079	CONTIG105	125930	126284	1.85	2.18E-02	transcription_start_site	+	126744	126744	-637	24495 AN6169
206	CONTIG105	123920	124404	2.93	0.00E+00	transcription_start_site	+	129108	129108	-4946	24496 AN6170

206	CONTIG105	123920	124404	2.93	0.00E+00	transcription_start_site	+	129339	129339	-5177	24497 AN6170
601	CONTIG105	125035	125479	2.31	7.85E-03	transcription_start_site	+	129108	129108	-3851	24496 AN6170
601	CONTIG105	125035	125479	2.31	7.85E-03	transcription_start_site	+	129339	129339	-4082	24497 AN6170
1079	CONTIG105	125930	126284	1.85	2.18E-02	transcription_start_site	+	129108	129108	-3001	24496 AN6170
1079	CONTIG105	125930	126284	1.85	2.18E-02	transcription_start_site	+	129339	129339	-3232	24497 AN6170
1079	CONTIG105	125930	126284	1.85	2.18E-02	transcription_start_site	+	130659	130659	-4552	24498 AN6171
1079	CONTIG105	125930	126284	1.85	2.18E-02	transcription_start_site	+	130755	130755	-4648	24499 AN6171
1079	CONTIG105	125930	126284	1.85	2.18E-02	transcription_start_site	+	131193	131193	-5086	24500 AN6171
793	CONTIG105	133955	134379	2.11	1.57E-02	transcription_start_site	-	132900	132900	-1267	24501 AN6172
793	CONTIG105	133955	134379	2.11	1.57E-02	transcription_start_site	+	139062	139062	-4895	24507 AN6175
793	CONTIG105	133955	134379	2.11	1.57E-02	transcription_start_site	+	139151	139151	-4984	24508 AN6175
60	CONTIG105	173720	174204	3.39	0.00E+00	transcription_start_site	-	174405	174405	443	24561 AN6192
95	CONTIG105	189979	190483	3.24	0.00E+00	transcription_start_site	-	188429	188429	-1802	24565 AN6195
794	CONTIG105	187971	188230	2.11	1.57E-02	transcription_start_site	-	188429	188429	328	24565 AN6195
2085	CONTIG105	191784	192655	1.34	1.11E-01	transcription_start_site	-	188429	188429	-3790	24565 AN6195
357	CONTIG105	196894	197228	2.67	1.80E-03	transcription_start_site	+	198753	198753	-1692	24566 AN6196
357	CONTIG105	196894	197228	2.67	1.80E-03	transcription_start_site	+	199090	199090	-2029	24567 AN6196
391	CONTIG105	195094	195510	2.62	2.22E-03	transcription_start_site	+	198753	198753	-3451	24566 AN6196
391	CONTIG105	195094	195510	2.62	2.22E-03	transcription_start_site	+	199090	199090	-3788	24567 AN6196
2276	CONTIG105	193894	194388	1.28	1.93E-01	transcription_start_site	+	198753	198753	-4612	24566 AN6196
2276	CONTIG105	193894	194388	1.28	1.93E-01	transcription_start_site	+	199090	199090	-4949	24567 AN6196
856	CONTIG105	213085	214794	2.05	3.45E-03	transcription_start_site	-	213169	213169	-770	24579 AN6201
856	CONTIG105	213085	214794	2.05	3.45E-03	transcription_start_site	-	212557	212557	-1382	24580 AN6201
109	CONTIG105	220504	220929	3.18	2.67E-04	transcription_start_site	+	225737	225737	-5020	24581 AN6202
109	CONTIG105	220504	220929	3.18	2.67E-04	transcription_start_site	+	225812	225812	-5095	24582 AN6202
109	CONTIG105	220504	220929	3.18	2.67E-04	transcription_start_site	+	225925	225925	-5208	24583 AN6202
1608	CONTIG105	224121	224615	1.54	9.24E-02	transcription_start_site	+	225737	225737	-1369	24581 AN6202
1608	CONTIG105	224121	224615	1.54	9.24E-02	transcription_start_site	+	225812	225812	-1444	24582 AN6202
1608	CONTIG105	224121	224615	1.54	9.24E-02	transcription_start_site	+	225925	225925	-1557	24583 AN6202
1608	CONTIG105	224121	224615	1.54	9.24E-02	transcription_start_site	+	226050	226050	-1682	24584 AN6202
1608	CONTIG105	224121	224615	1.54	9.24E-02	transcription_start_site	+	226262	226262	-1894	24585 AN6202
1608	CONTIG105	224121	224615	1.54	9.24E-02	transcription_start_site	+	226986	226986	-2618	24586 AN6202
1608	CONTIG105	224121	224615	1.54	9.24E-02	transcription_start_site	+	227833	227833	-3465	24587 AN6203
1608	CONTIG105	224121	224615	1.54	9.24E-02	transcription_start_site	+	228168	228168	-3800	24588 AN6203
1384	CONTIG105	241728	242842	1.64	4.42E-02	transcription_start_site	-	237916	237916	-4369	24589 AN6205
1384	CONTIG105	241728	242842	1.64	4.42E-02	transcription_start_site	-	237801	237801	-4484	24590 AN6205
1384	CONTIG105	241728	242842	1.64	4.42E-02	transcription_start_site	-	237648	237648	-4637	24591 AN6205
2096	CONTIG105	238578	239317	1.34	1.66E-01	transcription_start_site	-	237916	237916	-1031	24589 AN6205
2096	CONTIG105	238578	239317	1.34	1.66E-01	transcription_start_site	-	237801	237801	-1146	24590 AN6205
2096	CONTIG105	238578	239317	1.34	1.66E-01	transcription_start_site	-	237648	237648	-1299	24591 AN6205
2096	CONTIG105	238578	239317	1.34	1.66E-01	transcription_start_site	-	236509	236509	-2438	24592 AN6205
2096	CONTIG105	238578	239317	1.34	1.66E-01	transcription_start_site	-	236286	236286	-2661	24593 AN6205
2096	CONTIG105	238578	239317	1.34	1.66E-01	transcription_start_site	+	238860	238860	87	24597 AN6206
2096	CONTIG105	238578	239317	1.34	1.66E-01	transcription_start_site	+	238575	238575	372	24596 AN6206
2096	CONTIG105	238578	239317	1.34	1.66E-01	transcription_start_site	+	238500	238500	447	24595 AN6206
2096	CONTIG105	238578	239317	1.34	1.66E-01	transcription_start_site	+	238275	238275	672	24594 AN6206
482	CONTIG105	243243	243666	2.47	1.22E-03	transcription_start_site	-	241591	241591	-1863	24598 AN6207
482	CONTIG105	243243	243666	2.47	1.22E-03	transcription_start_site	-	241394	241394	-2060	24599 AN6207
482	CONTIG105	243243	243666	2.47	1.22E-03	transcription_start_site	-	241102	241102	-2352	24600 AN6207
482	CONTIG105	243243	243666	2.47	1.22E-03	transcription_start_site	-	240158	240158	-3296	24601 AN6207
1384	CONTIG105	241728	242842	1.64	4.42E-02	transcription_start_site	-	241591	241591	-694	24598 AN6207
1384	CONTIG105	241728	242842	1.64	4.42E-02	transcription_start_site	-	241394	241394	-891	24599 AN6207
1384	CONTIG105	241728	242842	1.64	4.42E-02	transcription_start_site	-	241102	241102	-1183	24600 AN6207
1384	CONTIG105	241728	242842	1.64	4.42E-02	transcription_start_site	-	240158	240158	-2127	24601 AN6207
2096	CONTIG105	238578	239317	1.34	1.66E-01	transcription_start_site	-	240158	240158	1210	24601 AN6207
482	CONTIG105	243243	243666	2.47	1.22E-03	transcription_start_site	+	244603	244603	-1148	24602 AN6208
482	CONTIG105	243243	243666	2.47	1.22E-03	transcription_start_site	+	244977	244977	-1522	24603 AN6208

482	CONTIG105	243243	243666	2.47	1.22E-03	transcription_start_site	+	245199	245199	-1744	24604 AN6208
482	CONTIG105	243243	243666	2.47	1.22E-03	transcription_start_site	+	245554	245554	-2099	24605 AN6208
482	CONTIG105	243243	243666	2.47	1.22E-03	transcription_start_site	+	246079	246079	-2624	24606 AN6208
1384	CONTIG105	241728	242842	1.64	4.42E-02	transcription_start_site	+	244603	244603	-2318	24602 AN6208
1384	CONTIG105	241728	242842	1.64	4.42E-02	transcription_start_site	+	244977	244977	-2692	24603 AN6208
1384	CONTIG105	241728	242842	1.64	4.42E-02	transcription_start_site	+	245199	245199	-2914	24604 AN6208
1384	CONTIG105	241728	242842	1.64	4.42E-02	transcription_start_site	+	245554	245554	-3269	24605 AN6208
1384	CONTIG105	241728	242842	1.64	4.42E-02	transcription_start_site	+	246079	246079	-3794	24606 AN6208
2515	CONTIG106	4446	5390	1.2	1.33E-01	transcription_start_site	-	3414	3414	-1504	24634 AN6220
2515	CONTIG106	4446	5390	1.2	1.33E-01	transcription_start_site	-	2822	2822	-2096	24635 AN6220
2515	CONTIG106	4446	5390	1.2	1.33E-01	transcription_start_site	-	2759	2759	-2159	24636 AN6220
2515	CONTIG106	4446	5390	1.2	1.33E-01	transcription_start_site	-	2606	2606	-2312	24637 AN6220
1381	CONTIG106	22884	23153	1.64	3.08E-02	transcription_start_site	-	19167	19167	-3851	24640 AN6223
1381	CONTIG106	22884	23153	1.64	3.08E-02	transcription_start_site	-	19059	19059	-3959	24641 AN6223
1791	CONTIG106	18319	20168	1.46	3.99E-02	transcription_start_site	-	19167	19167	-76	24640 AN6223
1791	CONTIG106	18319	20168	1.46	3.99E-02	transcription_start_site	-	19059	19059	-184	24641 AN6223
1381	CONTIG106	22884	23153	1.64	3.08E-02	transcription_start_site	-	21148	21148	-1870	24642 AN6224
1381	CONTIG106	22884	23153	1.64	3.08E-02	transcription_start_site	-	21074	21074	-1944	24643 AN6224
1381	CONTIG106	22884	23153	1.64	3.08E-02	transcription_start_site	-	20629	20629	-2389	24644 AN6224
1791	CONTIG106	18319	20168	1.46	3.99E-02	transcription_start_site	-	20629	20629	1385	24644 AN6224
1791	CONTIG106	18319	20168	1.46	3.99E-02	transcription_start_site	-	21074	21074	1830	24643 AN6224
1791	CONTIG106	18319	20168	1.46	3.99E-02	transcription_start_site	-	21148	21148	1904	24642 AN6224
1791	CONTIG106	18319	20168	1.46	3.99E-02	transcription_start_site	+	21389	21389	-2145	24645 AN6225
1791	CONTIG106	18319	20168	1.46	3.99E-02	transcription_start_site	+	21463	21463	-2219	24646 AN6225
1791	CONTIG106	18319	20168	1.46	3.99E-02	transcription_start_site	+	21538	21538	-2294	24647 AN6225
1791	CONTIG106	18319	20168	1.46	3.99E-02	transcription_start_site	+	21701	21701	-2457	24648 AN6225
2738	CONTIG106	29789	30208	1.11	1.93E-01	transcription_start_site	-	28884	28884	-1114	24649 AN6227
2738	CONTIG106	29789	30208	1.11	1.93E-01	transcription_start_site	-	28756	28756	-1242	24650 AN6227
2738	CONTIG106	29789	30208	1.11	1.93E-01	transcription_start_site	-	28094	28094	-1904	24651 AN6227
2738	CONTIG106	29789	30208	1.11	1.93E-01	transcription_start_site	-	29336	29336	-662	24655 AN6228
633	CONTIG106	45242	45651	2.26	2.22E-03	transcription_start_site	+	47883	47883	-2436	24656 AN6230
633	CONTIG106	45242	45651	2.26	2.22E-03	transcription_start_site	+	47975	47975	-2528	24657 AN6230
633	CONTIG106	45242	45651	2.26	2.22E-03	transcription_start_site	+	49529	49529	-4082	24658 AN6230
1382	CONTIG106	58203	58557	1.64	3.08E-02	transcription_start_site	-	55789	55789	-2591	24662 AN6232
1382	CONTIG106	58203	58557	1.64	3.08E-02	transcription_start_site	-	55599	55599	-2781	24663 AN6232
1382	CONTIG106	58203	58557	1.64	3.08E-02	transcription_start_site	-	55437	55437	-2943	24664 AN6232
1382	CONTIG106	58203	58557	1.64	3.08E-02	transcription_start_site	-	55337	55337	-3043	24665 AN6232
1382	CONTIG106	58203	58557	1.64	3.08E-02	transcription_start_site	-	55227	55227	-3153	24666 AN6232
1382	CONTIG106	58203	58557	1.64	3.08E-02	transcription_start_site	-	55007	55007	-3373	24667 AN6232
1382	CONTIG106	58203	58557	1.64	3.08E-02	transcription_start_site	-	54633	54633	-3747	24668 AN6232
[truncated: 3,122,583 more chars]
