# Supplementary material for: The Velvet Family of Fungal Regulators Contains a DNA-Binding Domain Structurally Similar to NF-κB
Source: PLoS Biol. 2013 Dec 31;11(12):e1001750. doi: 10.1371/journal.pbio.1001750 (PMC3876986; doi:10.1371/journal.pbio.1001750)
Supplement: Table S2 — Proposed VosA-binding motifs from VosA-ChIP-chip data. (PDF) [file pbio.1001750.s017.pdf]

Table S2. Proposed VosA-binding motifs from VosA-ChIP-chip data

Motif	p-value	% of Targets	% of Background
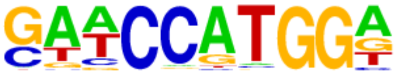	1e-48	51.33%	14.72%
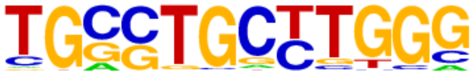	1e-15	10.67%	1.68%
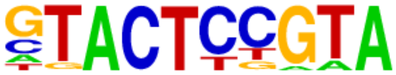	1e-15	12.67%	2.48%
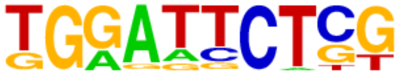	1e-14	12.67%	2.61%
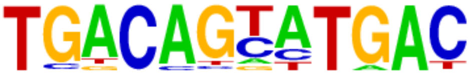	1e-14	5.00%	0.24%
