# Supplementary material for: The Velvet Family of Fungal Regulators Contains a DNA-Binding Domain Structurally Similar to NF-κB
Source: PLoS Biol. 2013 Dec 31;11(12):e1001750. doi: 10.1371/journal.pbio.1001750 (PMC3876986; doi:10.1371/journal.pbio.1001750)
Supplement: Table S3 — Selected VosA target genes used for MEME analysis. (PDF) [file pbio.1001750.s018.pdf]

Table S3. Selected VosA target genes used for MEME analysis

Contig	Locus	maxLevel[a]	Score[b]	Annotation
CONTIG160	AN8694	3.78	182.65	Fungal Zn(2)-Cys(6) binuclear cluster domain
CONTIG51	AN3152	3.71	215.62	sexual development transcription factor , NsdD
CONTIG93	AN5371	3.47	72.32	Hypothetical protein
CONTIG51	AN3079	3.48	112.28	Secreted thaumatin-like protein, CteA
CONTIG5	AN0279	3.48	338.19	myb-like DNA-binding transcription factor, FlbD
CONTIG29	AN1959	3.50	109.89	The *velvet* protein, VosA
CONTIG161	AN8741	3.45	297.17	C_2H_2 finger domain transcription factor
CONTIG109	AN6508	3.31	138.97	Putative protein kinase, Gsk3
CONTIG80	AN4680	3.33	82.71	Putative transcription factor, RfeG
CONTIG98	AN5709	3.29	85.44	Hypothetical protein
CONTIG29	AN1936	2.98	65.75	Regulatory protein involved in conidiation, WetA
CONTIG14	AN0973	1.53	13.28	C_2H_2 zinc finger transcription factor, BrlA
CONTIG94	AN5523	1.34	14.68	Putative α,α-trehalose-phosphate synthase, TpsA
CONTIG57	AN3441	1.60	14.69	Trehalose 6-phosphate phosphatase, OrlA
CONTIG172	AN9340	2.56	32.99	α,α-trehalose with a role in trehalose hydrolysis, TreA

a, The highest smoothed probe level in the enriched region.

b, The sum of smoothed probe levels minus the threshold.
