# Supplementary material for: The Velvet Family of Fungal Regulators Contains a DNA-Binding Domain Structurally Similar to NF-κB
Source: PLoS Biol. 2013 Dec 31;11(12):e1001750. doi: 10.1371/journal.pbio.1001750 (PMC3876986; doi:10.1371/journal.pbio.1001750)
Supplement: Table S4 — DNA oligos used in this study. (PDF) [file pbio.1001750.s019.pdf]

Table S4: DNA oligos used in this study

Oligoname	Sequence (5'-3')
JG45	CCCCATGGCGATGTACGCTGTTGAGGATAGG
JG46	CCCTCGAGGTATTCGTTATCCAGACCATC
JG337	TTTCCCATGGCGATGCAGCCGGAACCAGAGTCGCTT
JG338	TTCATGTGTACCGTCCGCGTTC
JG339	ACGCGGACGGTACACATGAAGCATCGATCCCGCCGTCGACGGCAGGTTG CTTGAGTGCCAGTGCAT
JG342	AAACCATGGTTAGTGGTGGTGGTGGTGGTGAGCGCTGGGGATCTTGATT CCTTGGTTCG
JG365	CGGCGGGTCAACCGGTTTGCGCTCTTTCTCCGCCCC
JG366	CCGGTTGACCCGCCGCCGAT
JG367	TTTCCATGGTTATTTTTCGAACTGCGGGTGGCT
JG368	CGGCGGGTCAACCGGTTTGCGCTCTGCCTC
JG369	CGGCGGGTCAACCGGTTTGGCCTCTTTCTC
JG370	CGGCGGGTCAACCGGTGCGCGCTCTTTCTC
JG371	CGGCGGGTCAACCGGTGCGGCCTCTGCCTCCGCCCC
JG372	AAACGGTGAACCGCTCCGAGACGAT
JG489	ATCGATAAGCTTGATGTTTAAACAAGGGCTCCTGTCGGATC
JG490	GCGCGCCCGATTGGGGTTTTG
JG491	CCCAATCGGGCGCGCGTGGCCGGTGGGGCGGAGGCAGGT
JG492	ATGGGATCCCGTAATTCACCGAGGAGTTCCGTTCGCT
JG493	TACAAACAAAGATGCGGATTCTCGTTTGTGGAACACCT
JG494	CTGCAGGAATTCGATGTTTAAACTTGTTAACTGTAAAGTCTCTGA
JG553	ATGCCCGACTTTCTCTCTGGAT
JG554	CTGCCTGGTGACGGTAGTTGTT
JG557	CTTCCATTCCACCGTCTACTGC
JG558	CGTCCGTCTTTCGCATTTCA
JG636	ATTCGGCGACTAGGATCTTACCCGCGGCCGAG
JG637	TAAGCCGCTGATCCTAGAATGGGCGCCGGCTC
JG638	ATTCGGCGACTAGGATCTTACTGGCCGAG
JG639	TAAGCCGCTGATCCTAGAATGACCGGCTC
JG640	ATTCGGCGACTAGGATCTTACCCGAG
JG641	TAAGCCGCTGATCCTAGAATGGGCTC
JG642	CGGCGGGTCAACCGGTTTGCGCTCTGCCTCCGCCCC

OHS301	ATTCGGCGACTAGGATCTTACTGGCCGCGGCCGAG
OHS302	TAAGCCGCTGATCCTAGAATGACCGGCGCCGGCTC
OHS419	GATTGAGTGCCGTCCTAGCGTGC
OHS420	AGGTAGTCTGAGGACGTGGACG
OHS421	CGTTAGTCCGCATTCCTCTCCC
OHS422	ATGGAAGAGGCTGCCAGAAGACC
OHS425	CCAAACGCTCTCTTGTTCTGG
OHS426	CAGTTGGAGAAGTAGCTAGCAGG
OHS427	CTTGGCATAATCATGCAAATCCC
OHS428	CTGAGCTTGGGATTGGGAACG
OHS431	GGTGTTTTGTCCCATTCCCGAC
OHS432	CATCACATACTGACTCACTTTCCCG
OHS512	ATGCGAAATCAGTCCAGCCTGTC
OHS513	AAGCAGAACCAGGAGTTCGTTCG
OHS616	CGTTTCTTCCACCCGAGATGG
OHS617	CAGGAGTCTCTTTGGTGGGAAG
OHS620	ACAATGTCGAAGCACATGCGCC
OHS621	TCTACATCGCCGCCTGCTTTCC
OHS723	ATATGTCGACTCATGGCTACACTTGCAGCACCACC
OHS724	ATATGCGGCCGCACGCATGGTGGCAGGCTTTG
OHS739	ATGTCGAACAATCCGAACCCGAG
OHS740	GGTTGACCATGAGCACCGTAAAG
OHS741	ATGATGTTCACCAAGGCTCTCG
OHS742	TTACGCGCCGAGAGTCAGG
OHS743	ATGGATCTCGCCAACCTCATCTC
OHS744	TTACACCATCGCGACAGCCCTC
OHS745	ATGTCACAGAATCGGTCTGGGG
OHS746	TCCCGTGTAGGAGTACCGAGAAC
OHS747	ATGACTAGCCGTCAGAATGAATACT
OHS748	AGCCTCTCTTTCTCTTGCTGGC
OHS753	ATGTCTCCCAGACCACCAAGTATC
OHS754	GGCTTGAGCTTGGATATGAACCG
ONK14	ATATGAATTCATGAGTGCGGCGAACTATCCAG
ONK322	ATGGCCAACACACCGTCGTG
ONK323	TCAGTTCAAGAGGTTGTCGAGG
OMN178	CGA GTA CGC CAA TCA ATT CCG

OMN179	AGT AGA GGC ACA GGC TGG ATC
OMN184	CGGGATCCATGAGTGCGGCGAACTATCCAG
OMN185	CGGGATCCATGCAAATCCCTGGAAGTAGC
OMN303	ACGCGTCGACCCCGAGGAGTTCCGTTCGCTGAG
OMN340	ATGGGATCACTAGAGGCTGGA
OMN341	TTAATGACTCCTCGGTGACACC
OJA142	CTGGCAGGTGAACAAGTC
OJA143	AGAAGTTAACACCGTAGA
OZG479	TTTCCCATGGGTGCGGCGAACTATCCAGATC
OZG480	TTTCCATGGTTATTTTTCGAACTGCGGGTGGCTCCAAGCGCTTGTGCGCG GCTCCTTTCGAATCCTTAG
RT H2A 5'(+)	TGCGGTCGTGTTAAGCGTTT
RT H2A 3'(-)	CGGATGGCAAGCTGTAGGTG
