# Supplementary material for: The Velvet Family of Fungal Regulators Contains a DNA-Binding Domain Structurally Similar to NF-κB
Source: PLoS Biol. 2013 Dec 31;11(12):e1001750. doi: 10.1371/journal.pbio.1001750 (PMC3876986; doi:10.1371/journal.pbio.1001750)
Supplement: Text S1 — Supplementary material and methods. (PDF) [file pbio.1001750.s020.pdf]

Supporting Information

Material and Methods

Due to the fact that VelB in the VosA$_{1-190}$-VelB structure did not reveal electron density for the 99 residues inserted into the VelB *velvet* domain, a version of VelB lacking this insertion was generated (VelB_ΔIL, IL = inserted loop). It was cloned into a modified pET-24d-vector (pETM13), encompassing amino acid residues 44-126 and 240-343 connected by a short linker of eight amino acids with the sequence ASIPPSTA (the corresponding loop in VosA).

The resulting C-terminally His-tagged protein VelB_ΔIL was used for size exclusion chromatography and multi angle light scattering to verify stable hetero-dimer formation.

VelB_ΔIL was expressed in Rosetta 2 (DE3) cells (Merck) at 16°C for 72h using autoinduction medium [1]. After harvesting, the cells were lysed in lysis buffer (35 mM Imidazol, 400 mM NaCl und 35 mM HEPES pH 7.5.) using the Fluidizer (Microfluidics) and a cleared supernatant obtained by centrifugation for 20 min at 4°C and 30,000 ×g. VelB_ΔIL was purified from the supernatant using a two-step purification scheme. Affinity chromatography was applied first using HisTrap columns (GE-Healthcare). The bound protein was washed in 2 M LiCl in Lysis buffer to remove putative DNA bound to VelB, and subsequently eluted with a gradient of Imidazol (35 – 400mM) in lysis buffer. The final purification step was performed using an S75 (16/600; GE-Healthcare) equilibrated in 10 mM HEPES pH 7.5 und 150 mM NaCl.

The identical buffer was used for the SEC and MALS experiments. MALS has been performed using a Mini Dawn Treos (Wyatt) and ASTRA version 5.4.1 in line with an Äkta purifier (GE Healthcare). MM (molecular mass) and extinction coefficient have been determined using ProtParam: http://web.expasy.org/protparam/.

For the CD-measurements, the individual proteins, stored at -80°C in the buffer (10 mM HEPES pH 7.5 und 150 mM NaCl) and a concentration of 5 mg/ml, were diluted 1:10 in 10 mM sodium-phosphate buffer pH 7.5 before measurement. Secondary structure deduction has been performed using CDNN (200- 260 nm) and normalized to 100 [2].

Aspergillus nidulans strains and growth conditions

Glasgow wild type strain A4 (Fungal Genetics Stock Center) and *vosA* deletion strain RNI14.1 (Ni et al 2007) were used. Strains were grown on or in minimal medium (0.52 g/l KCl, 0.52 g/l MgSO4, 1.52 g/l KH_2PO_4, 10 mM sodium nitrate, 1% D-glucose, 0.1% trace element solution, pH 6.5) (Barratt et al., 1965) supplemented with 5 µg/l biotin. For induction of asexual development, plates were incubated in light. For induction of sexual development, plates were sealed with parafilm and incubated in darkness. For growth tests (if not stated otherwise), 1,000 spores were spotted on an agar plate.

Construction of *Aspergillus nidulans* mutant *vosA*K37/39A

For construction of strain *vosA*K37/39A (AGB800), the *vosA* gene was mutated by PCR with the mutated primer JG491 and with JG492. The 5'- and 3'-UTR of *vosA* were amplified from genomic DNA with oligos JG489/490 and JG493/494. The *ptrA* resistance cassette was amplified from plasmid pPTRII (Takara) with primer pair JG21/22. The fragments together with *Eco*RI digested pBluescript II SK+ (Stratagene) were assembled with the GeneArt® Seamless Cloning & Assembly Kit (Invitrogen GmbH) resulting in plasmid pME4163 and cloned in *Escherichia coli* DH5α. For transformation in *A. nidulans* RNI14.1, the transformation cassette

was digested from the plasmid pME4163 with *Pme*I. Transformation in *A. nidulans* was performed by polyethylene glycol mediated fusion of protoplasts [3,4].

Genomic DNA extraction and Southern hybridization

Genomic DNA was extracted from vegetative grown cultures by standard protocols as described previously [5]. For Southern analysis, 25 µg genomic DNA were digested with *Sca*I overnight. The hybridization was performed according to standard protocols [6] with a non-radioactively labelled probe. The DNA for probe design was amplified from genomic DNA with primers JG493/494.

Viability test

Fresh spores were spread on an agar plate and cultivated for 10 days at 37°C. The spores were harvested and 250 of these old spores were grown on new agar plates for two days at 37°C. The amount of surviving colonies has been compared between all strains.

Extraction of RNA

RNA was extracted from vegetative and asexual grown cultures. For vegetative cultures, the strain was grown 20 h in liquid medium at 37°C. For asexual cultures, the strain was grown 20 h in liquid medium and the obtained mycelium was transferred to solid medium and grown 48 h in light at 37°C. Mycelia were ground in liquid nitrogen and total RNA was extracted with the RNEasy Plant Kit (Qiagen).

Quantitative real-time PCR

DNase digestion and cDNA synthesis were carried out with the QuantiTect Reverse Transcription Kit (Qiagen) with 0.8 mg of RNA for each sample. Amplification was performed in a LightCycler 2.0 (Roche) with the RealMaster SYBR Rox Kit (5Prime) using 1 µl of the cDNA and *A. nidulans* oligos JG553/554, JG557/558 and RT H2A 5'(+)/3'(-). For normalization, the constitutively expressed H2A encoding gene was used and a sample without cDNA served as additional control. Amplification conditions were as follows: 36 cycles of 15 s at 95°C, 22 s at 64°C, 22 s at 70°C, with an adjacent melting step (42–95°C). The amount of *brlA* and *vosA* relative to histone H2A RNA was quantified using the DCT method with efficiency [7]. All real-time PCR experiments were performed in triplicate.

Statistical analysis

Statistical differences between WT and mutant strains were evaluated with student's unpaired *t*-test (2-tailed). Mean ±SD are shown. P values < 0.05 were considered significant.

Figures

Structure representations have been made using PYMOL (The PyMOL Molecular Graphics System, Version 1.5.0.4 Schrödinger, LLC) and CHIMERA [8]. The alignments in Fig S7 have been extracted from the respective PDB-file using CHIMERA, missing residues have been manually inserted into the alignment file and the final figure has been made using ESPRIPT [9]

References

1. Studier FW (2005) Protein production by auto-induction in high density shaking cultures. Protein Expr Purif 41: 207-234.
2. Bohm G, Muhr R, Jaenicke R (1992) Quantitative analysis of protein far UV circular dichroism spectra by neural networks. Protein Eng 5: 191-195.
3. Eckert SE, Kubler E, Hoffmann B, Braus GH (2000) The tryptophan synthase-encoding trpB gene of Aspergillus nidulans is regulated by the cross-pathway control system. Mol Gen Genet 263: 867-876.
4. Punt PJ, van den Hondel CA (1992) Transformation of filamentous fungi based on hygromycin B and phleomycin resistance markers. Methods Enzymol 216: 447-457.
5. Gerke J, Bayram O, Feussner K, Landesfeind M, Shelest E, et al. (2012) Breaking the silence: protein stabilization uncovers silenced biosynthetic gene clusters in the fungus Aspergillus nidulans. Appl Environ Microbiol 78: 8234-8244.
6. Busch S, Eckert SE, Krappmann S, Braus GH (2003) The COP9 signalosome is an essential regulator of development in the filamentous fungus Aspergillus nidulans. Mol Microbiol 49: 717-730.
7. Pfaffl MW, Horgan GW, Dempfle L (2002) Relative expression software tool (REST) for group-wise comparison and statistical analysis of relative expression results in real-time PCR. Nucleic Acids Res 30: e36.
8. Pettersen EF, Goddard TD, Huang CC, Couch GS, Greenblatt DM, et al. (2004) UCSF Chimera--a visualization system for exploratory research and analysis. J Comput Chem 25: 1605-1612.
9. Gouet P, Courcelle E, Stuart DI, Métoz F (1999) ESPript: analysis of multiple sequence alignments in PostScript. Bioinformatics 15: 305-308.
